# Phyto-Mediated Synthesized Gold Nanoparticles (AuNPs) from *Ozoroa paniculosa* Leaf Extracts: Chemical Characterization and Antidiabetic Evaluation

**DOI:** 10.3390/ph19101635

**Published:** 2026-10-15

**Authors:** Nkosinathi D. Cele, Katleho Ramile, Sphamandla C. Masikane, Micheal Ojo Chukwuka, Sandile H. Khoza, Neerish Revaprasadu, Andrew R. Opoku

**Affiliations:** 1 Department of Biochemistry and Microbiology, Faculty of Science, Agriculture, and Engineering, University of Zululand, Private Bag X1001, KwaDlangezwa 3886, South Africa; ramilekatleho3@gmail.com (K.R.); mikekonyegwachie2015@gmail.com (M.O.C.); opokua@unizulu.ac.za (A.R.O.); 2 Department of Chemistry, Faculty of Science, Agriculture and Engineering, University of Zululand, Private Bag X1001, KwaDlangezwa 3886, South Africakhozas@unizulu.ac.za (S.H.K.); revaprasadun@unizulu.ac.za (N.R.)

**Keywords:** *Ozoroa paniculosa*, gold nanoparticles, diabetes mellitus, physicochemical characterization, antioxidant

## Abstract

**Background:** Type 2 diabetes mellitus is a serious health concern, as it remains the leading cause of mortality worldwide. Despite the available antidiabetic drugs used in the management of diabetes, the continuous increase in hyperglycemia has prompted growing interest in developing effective, green alternative therapeutic strategies for diabetes mellitus. Hence, this study aimed to synthesize *O. paniculosa* extract-passivated AuNPs and evaluate their antidiabetic properties. **Methods:** The plant material was sequentially extracted with n-hexane, dichloromethane (DCM) and 70% ethanol (70% EtOH), after which the resulting extracts were used to synthesize AuNPs that were characterized through various analytical techniques. The antioxidant activity of AuNPs was screened against DPPH, ABTS, metal iron chelating and reducing power. The enzyme inhibitory activity of AuNPs on α-amylase, as well as the ability of AuNPs to glycate protein, was investigated on a BSA-fructose model over a period of 28 days. **Results:** The FT-IR, pXRD, HR-TEM, STEM-EDS and UV-Vis spectroscopy analyses confirmed the synthesis of *O. paniculosa* extract-passivated AuNPs bearing faceted NPs and triangular 2D nanoplates morphologies. Despite the poor scavenging activity of AuNPs on DPPH and ABTS, a significant metal-ion chelating potential with IC_50_ values ranging from 76.7 ± 0.02 μg/mL to 83.3 ± 0.03 μg/mL and a good reduction potential was observed. Furthermore, an inhibition in concentration-dependent α-amylase activity was observed, with IC_50_ values ranging from 76.7 ± 0.02 μg/mL to 380 ± 0.12 μg/mL, and the formation of AGEs was suppressed, with an inhibitory effect observed over a period of 28 days. **Conclusions:** The results obtained suggest that *O. paniculosa* extract-passivated AuNPs possess some potential antioxidant and antidiabetic properties.

## 1. Introduction

Diabetes mellitus, a complex metabolic disorder of carbohydrates, lipids and proteins, is characterized by hyperglycemia due to defective insulin function, disrupting normal glucose homeostasis [1,2]. Even though diabetes mellitus is classified into different types, type 2 diabetes accounts for between 90% and 95% of all diabetes cases worldwide [3,4]. Hence, it has become a serious health concern, with 589 cases reported in the year 2024 and an anticipated increase to 783 million by 2045 [5].

Although the increase in diabetes prevalence is occurring globally, Africa has the lowest prevalence compared to other regions. However, due to growing shifts towards more urbanization associated with low physical activity and unhealthy diet, there is a rapidly increasing trend, with 24 million cases in 2021 envisaged to be doubled by 2045 [5,6,7]. Various risk factors attributed to an unhealthy lifestyle have been shown to strongly contribute to the onset and progression of diabetes [8], identifying the combination of oxidative stress and increased formation of advanced glycation end-products (AGEs) as a major contributor [9]. 

AGEs are a group of highly reactive compounds formed through the Maillard reaction pathway; the reaction forms Schiff bases, which subsequently undergo rearrangement to form Amadori products, notoriously known for being intermediate precursors for AGEs [10]. Upon formation, AGEs interact specifically with receptors for advanced glycation end-products (RAGE), activating a series of intracellular signalling cascades leading to excessive production of reactive oxygen species (ROS), disruption of the endogenous antioxidant defence system, and ultimately induced oxidative stress. Persistent oxidative stress contributes to cellular dysfunction, tissue damage, and the development of various diabetes-associated pathologies [11,12]. 

Several pharmaceutical agents such as Metformin and Thiazolidinediones are currently used in the therapeutic management of diabetes mellitus. However, these drugs are associated with side effects, which limit their use [13]. Therefore, the search for alternative remedies from natural resources such as medicinal plants, which exhibit mild-to-no side effects, is increasingly gaining interest among researchers [14]. Scientific advances have also shown the incorporation of nanoparticles into phytochemical remedies earmarked for diabetes mellitus. The unique physicochemical properties of nanoparticles exhibiting good biocompatibility [e.g., gold nanoparticles (AuNPs)] have been demonstrated to enhance drug delivery, bioavailability, and therapeutic efficacy in the management of various diseases including diabetes mellitus [15,16,17,18]. Recent trends identify a one-pot green synthesis of phytochemical-encapsulated nanoparticles as an ideal approach suitable for this application; the choice of phytochemicals has a major influence on the physicochemical properties of the nanoparticles and subsequently their performance efficacy. 

*Ozoroa paniculosa* plant belongs to the Anacardiaceae family and is traditionally used to treat fever, headaches, as well as stomach pains [19]. Phytochemical composition studies have reported that this plant extract possesses antioxidant, anti-cancer properties and ameliorative effects on testicular dysfunction [19,20]. Furthermore, plant-derived bioactive compounds, particularly polysaccharides, polyphenols, and flavonoids, have attracted considerable attention for the management of metabolic disorders due to their antioxidant, anti-inflammatory, and metabolism-modulating properties, which may help regulate glucose and lipid homeostasis and mitigate metabolic dysfunction [21,22]. These phytochemicals have potential for the green synthesis of nanoparticles and subsequent integration of their therapeutic potential into the nanocomposite systems. Hence, for the first time, this study aimed to develop a green synthesis of phyto-mediated and encapsulated AuNPs using *Ozoroa paniculosa* leaf extract. Furthermore, the study investigated the *in vitro* antidiabetic efficacy of the as-synthesized nanoparticles. 

## 2. Results

### 2.1. Percentage Yield

Powdered leaves of *O. paniculosa* were sequentially extracted with n-hexane, DCM and 70% EtOH. The obtained yield from each extract was 8.28%, 2.89% and 4.78%, respectively.

### 2.2. Phytochemical Evaluation

#### 2.2.1. The Total Phenolic (TPC) and Flavonoid Contents (TFC) of *O. paniculosa* Extracts 

**Figure 1:**
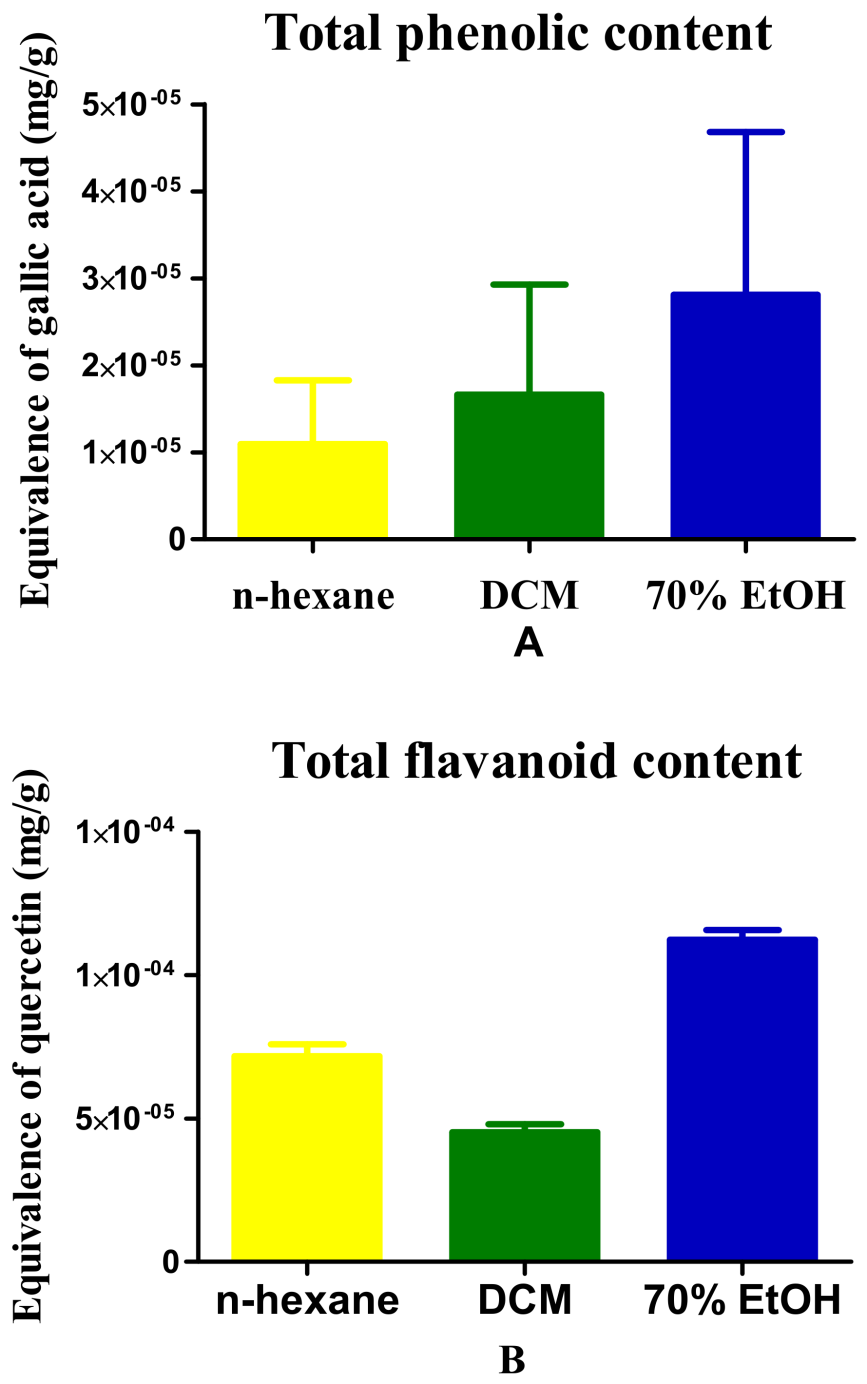
The graphs for (**A**) TPC and (**B**) TFC depict data expressed as mean ± standard deviation (SD) (*n* = 3). The results were analyzed with one-way analysis of variance (ANOVA), and the differences were considered statistically significant at *p* ≤ 0.05.

The results obtained in this study showed a significant level of phenolic and flavonoid bioactive compounds in n- hexane, DCM and 70% EtOH crude extracts, as shown in Figure 1. Then, the 70% EtOH extract presented a higher content than the other extracts in phenolic and flavonoid compounds. 

#### 2.2.2. Chromatographic Chemical Characterization of *O. paniculosa* Crude Extracts

The GC-MS analysis identified 135, 122 and 93 compounds detected in *O. paniculosa* crude extracts obtained from n-hexane, DCM and 70% EtOH, respectively (Table 1). The only common compounds detected among the three extracts are 4-methyl-octane, 4-(1-methylethyl)-Heptane, o-xylene, Nonane, 3,3-dimethyl-2-Hexanone, 1-(3-ethyloxiranyl)-ethanone, and 2-methyl-Nonane. 

### 2.3. Characterization of O. paniculosa Crude Extract-Passivated AuNPs

#### 2.3.1. FT-IR Analysis of *O. paniculosa* Crude Extracts and Green Synthesized AuNPs

**Figure 2:**
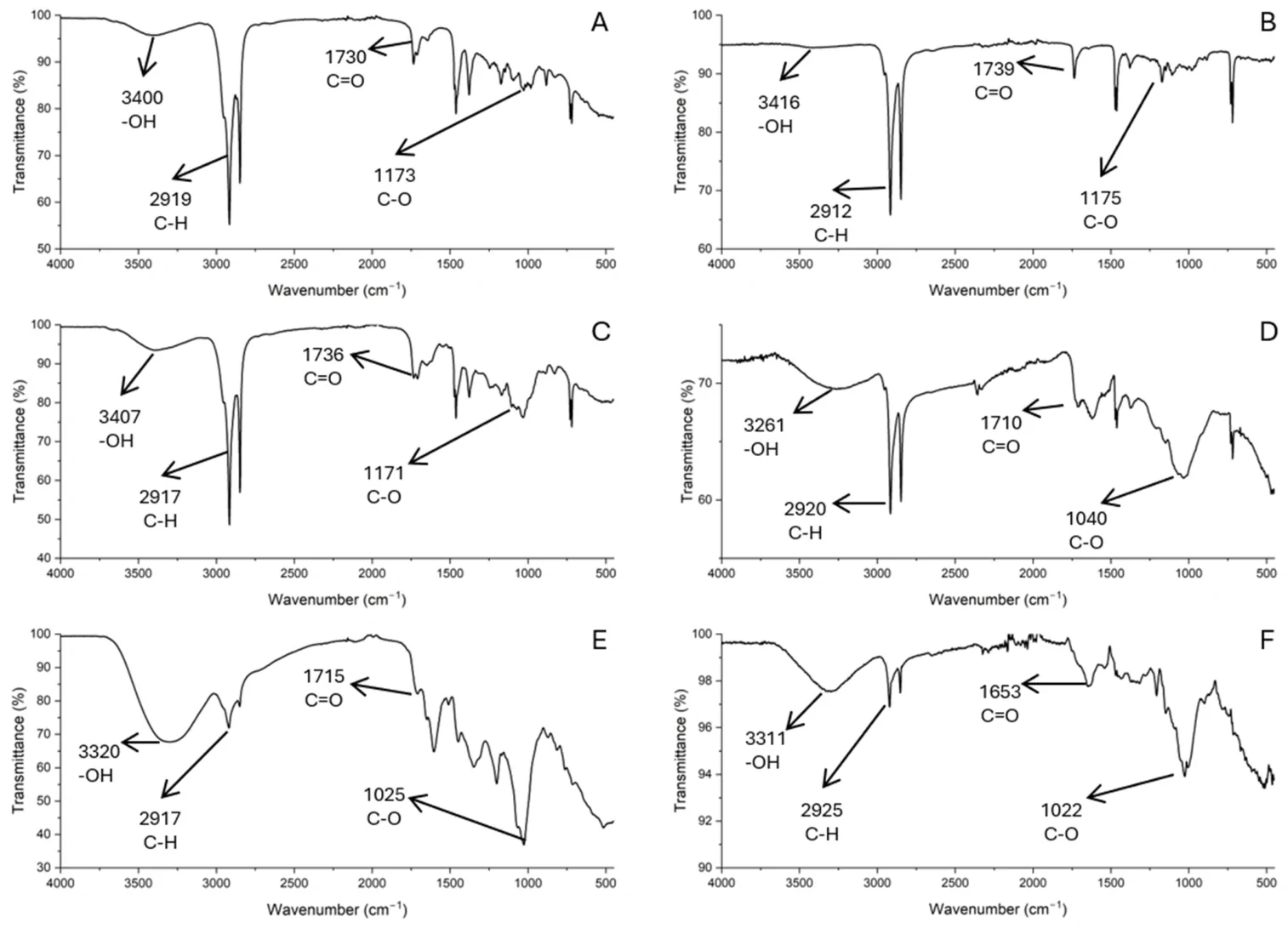
The FT-IR spectra of (**A**) n-hexane, (**C**) DCM and (**E**) 70% EtOH extracts and their corresponding FT-IR spectra of (**B**,**D**,**F**) green-synthesized AuNPs, respectively.

The FT-IR spectra of the *O. paniculosa* crude extract, which was sequentially extracted using n-hexane, DCM and 70% ethanol, are shown in Figure 2A, C and E, respectively. Complementary to the GC-MS analysis, the FT-IR spectra showed the transmittance peaks and bands in different regions, indicating the presence of phytochemical constituents mostly bearing hydroxyl, hydrocarbon, as well as ketone, aldehyde and ester moieties. The as-synthesized AuNPs, which were obtained as brown-to-purple solids, produced FT-IR spectra (Figure 2B,D,F) bearing similar characteristics to the extracts. Furthermore, the presence of functional groups with their vibrational frequencies appearing to be shifted from the parent extracts is generally an indication of extract-decorated and/or capped metal NPs.

#### 2.3.2. Powder X-Ray Diffraction (pXRD) Analysis of *O. paniculosa* Crude Extract-Passivated AuNPs

**Figure 3:**
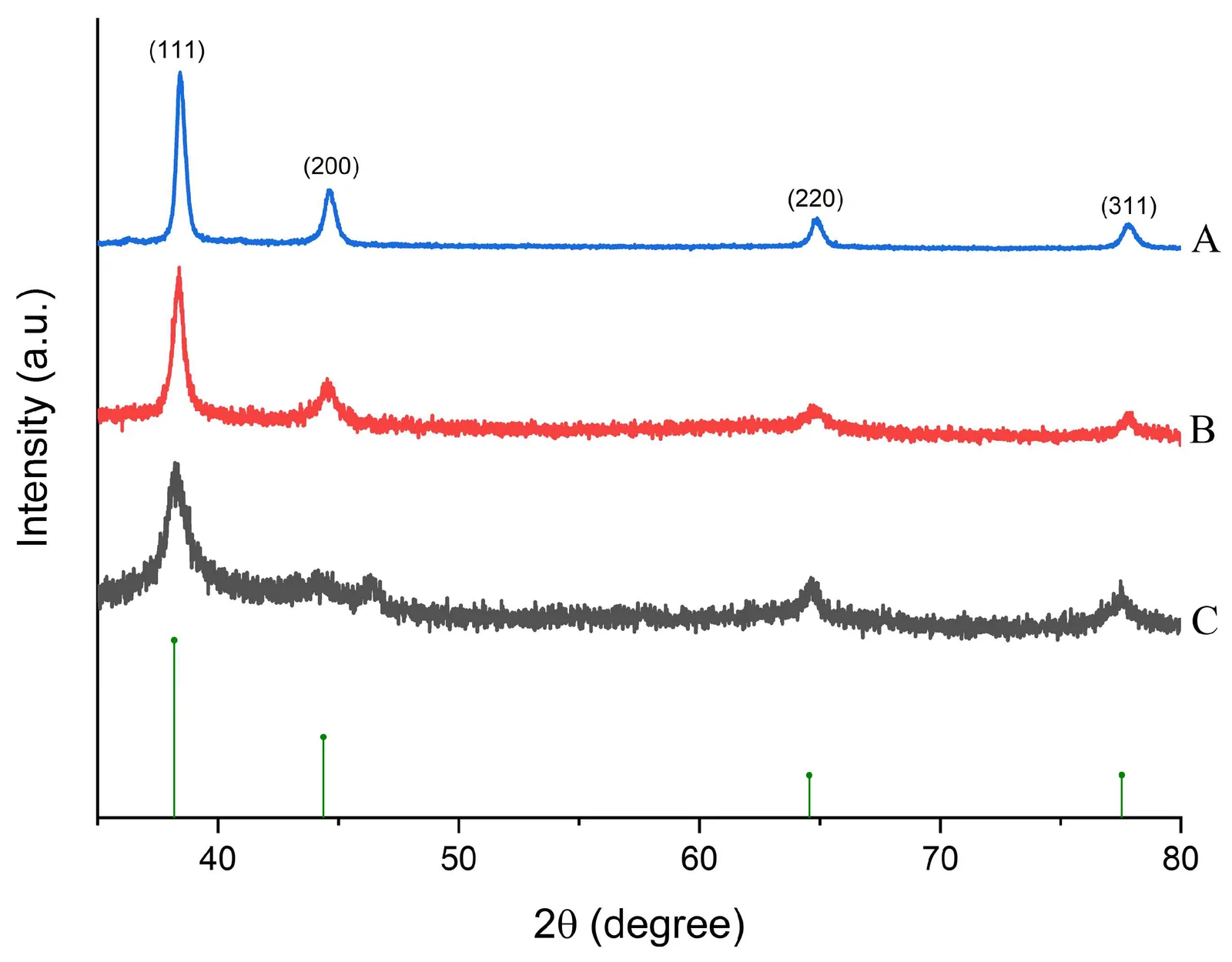
The pXRD analysis of (**A**) n-hexane, (**B**) DCM and (**C**) 70% EtOH-AuNPs. Green stick pattern: Au face-centred cubic structure, ICDD # 01-089-3697.

The formation of the AuNPs envisaged to be decorated by extracts bearing diverse bioactive functional groups was confirmed by the pXRD analysis. The pXRD patterns depicted in Figure 3 display the presence of diffraction peaks at 2θ = 38.44°, 44.38°, 64.56° and 77.55°, which were indexed to (111), (200), (220) and (311) planes for the Au face-centred cubic structure, ICDD # 01-089-3697. The broadness of the diffraction peaks indicates that the crystallite sizes range below 100 nm, a phenomenon technically detailed by the Scherrer equation. Evidently, the crystallite sizes calculated using the same equation were recorded to be 19.2 ± 2.76, 9.67 ± 3.24 and 7.79 ± 2.20 nm using all four diffraction peaks from the patterns for n-hexane, DCM and 70% EtOH-AuNPs, respectively. This clearly indicates that the various phytochemical compositions in extracts have an influence on controlling the crystallite sizes during synthesis and passivating the nanoparticles from overgrowth and severe agglomeration.

#### 2.3.3. High Resolution Transmission Electron Microscopy (HR-TEM) and Energy Dispersive X-Ray Spectroscopy (EDS) Analysis of *O. paniculosa* Crude Extract-Passivated AuNPs

**Figure 4:**
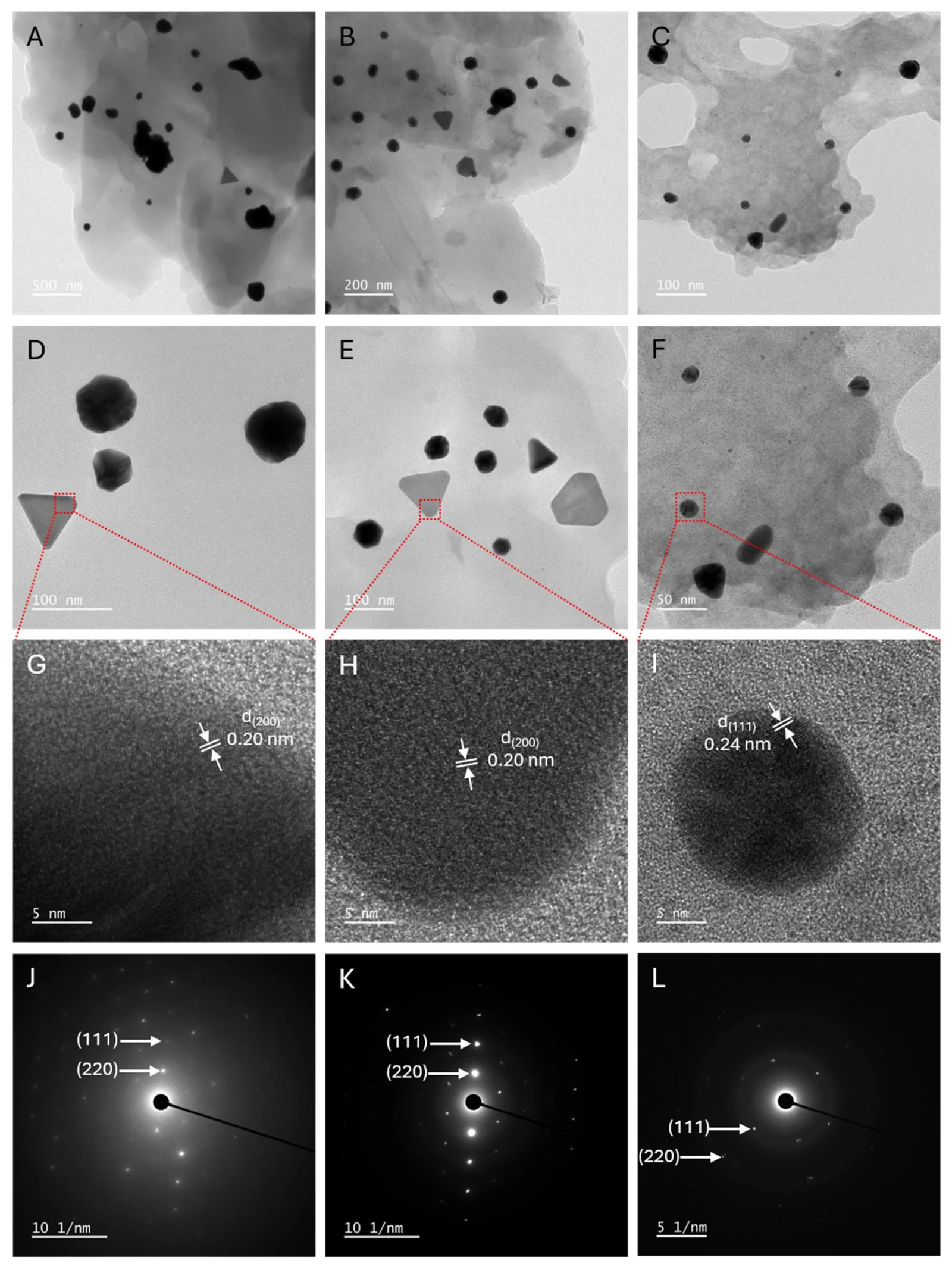
The (**A**–**C**) low-magnification HR-TEM, (**D**–**F**) high-magnification HR-TEM, (**G**–**I**) HR-TEM and (**J**–**L**) SAED micrographs for n-hexane, DCM and 70% EtOH AuNPs, respectively.

**Figure 5:**
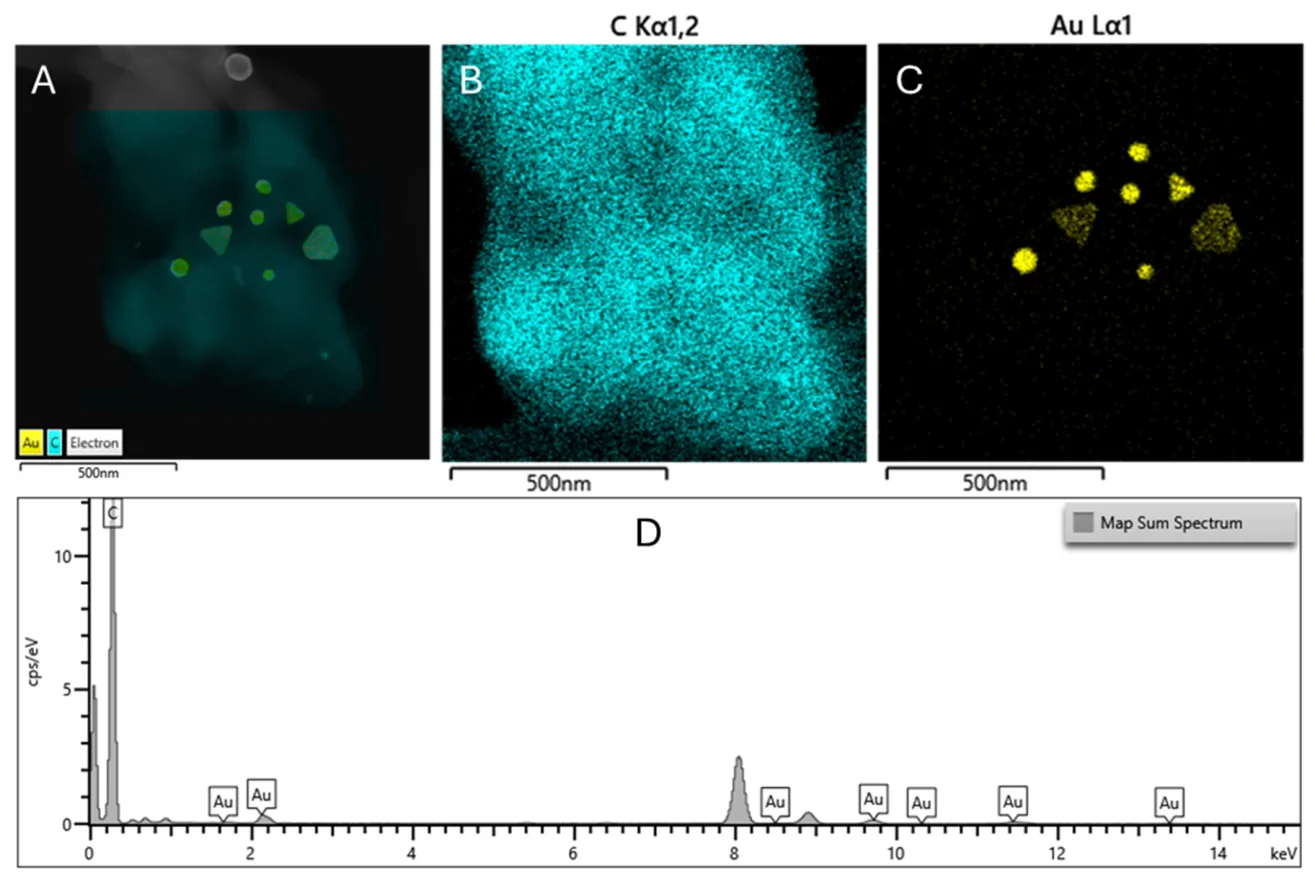
The (**A**–**C**) STEM-EDS elemental mapping micrographs and (**D**) EDX spectrum of DCM-AuNP. Unmarked peaks are attributed to copper TEM grid material and residual oxygen (from the atmosphere) on the surface of the specimen.

The TEM micrographs in Figure 4A–F show AuNPs bearing different morphologies, which include faceted NPs and triangular 2D nanoplates, with varying lateral sizes between 10 and 120 nm. The mixed morphology can also be attributed to the various phytochemical compositions in extracts; different constituents exhibit different passivating mechanisms that mainly determine the particle shape. Ref. [22] also reported the same mixed morphology observation on AuNPs synthesized from *Albizia lebbeck* leaf crude extracts. The HR-TEM (Figure 4G–I) and SAED (Figure 4J–L) micrographs confirmed both morphologies to be AuNPs due to the matching of d-spacing values with the corresponding diffraction planes of the Au face-centred cubic structure, ICDD # 01-089-3697. The thick material engulfing AuNPs is presumed to be the organic passivating components from the extracts; the STEM-EDS elemental mapping micrographs (Figure 5A–C) and EDX spectrum (Figure 5D) of the representative DCM-AuNPs indicate that the thick material, appearing to be of organic nature, has formed a nanocomposite with AuNPs, complementary to the FT-IR results.

#### 2.3.4. Ultraviolet–Visible (UV-Vis) Analysis of *O. paniculosa* Crude Extract-Passivated AuNPs

**Figure 6:**
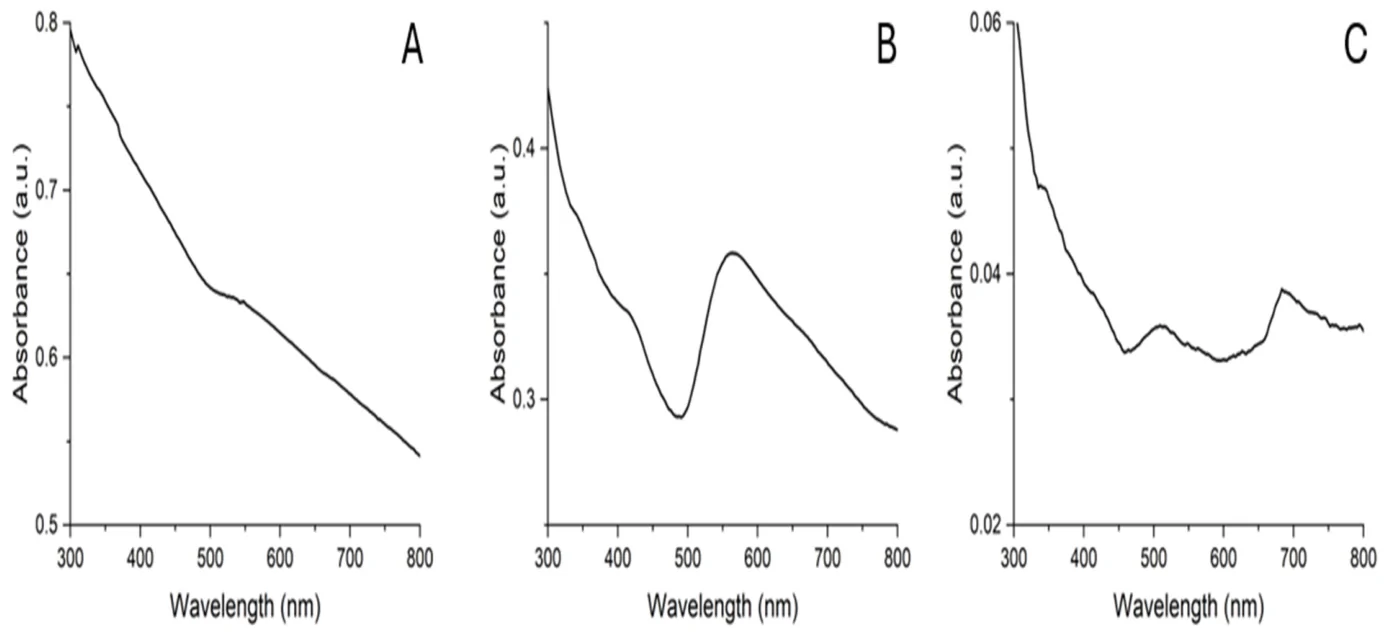
UV–visible analysis of (**A**) n-hexane-AuNP, (**B**) DCM-AuNP and (**C**) 70% EtOH-AuNP.

The UV-Vis spectra were used to confirm the formation of extract–AuNP composites, as shown in Figure 6. The shape and size of AuNPs determine the spectral position of the surface plasmon resonance (SPR) bands and their width, which generally appear between 500 and 560 nm. It should be noted that conjugated compounds also absorb strongly in the UV-Vis spectral range; the GC-MS data have identified various conjugated compounds. Thus, the multiple absorption bands observed in all UV-Vis absorption spectra may be attributed to the presence of both extracts and AuNPs, complementary to the data obtained through other analytical techniques in this paper.

### 2.4. Antioxidant Assays

#### Free Radicals Scavenging Activity

**Figure 7:**
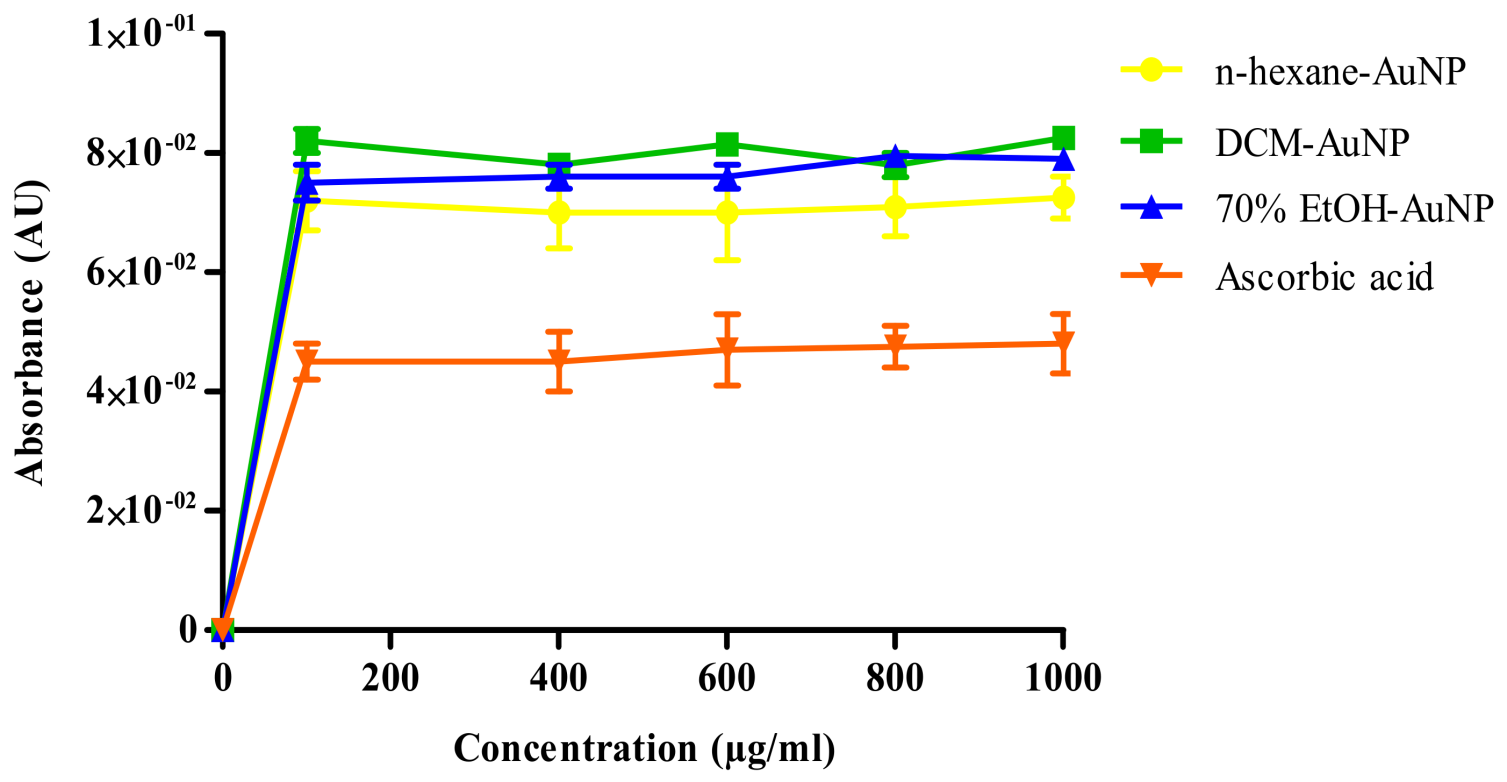
Reducing power graph. Data were expressed as mean ± standard deviation (SD) (*n* = 3). The results were analyzed with one-way analysis of variance (ANOVA), and the differences were considered statistically significant at *p* ≤ 0.05.

Table 2 presents the antioxidant activity of AuNPs on DPPH, ABTS and metal iron chelating, whereby AuNPs exhibited poor efficacy on DPPH and ABTS radicals when compared to ascorbic acid. Moreover, a noticeable, moderate metal Fe^2+^ chelating activity of AuNPs with IC_50_ values ranging from 76.7 to 83.3 µg/mL was also observed, which was higher than citric acid 63.3 µg/mL. Furthermore, the reduction potential of AuNP was concentration-dependent, with a double effect compared to ascorbic acid, and the results are presented in Figure 7.

### 2.5. Enzyme Inhibition Assay

#### Inhibitory Effect of AuNP on α-Amylase Activity

**Figure 8:**
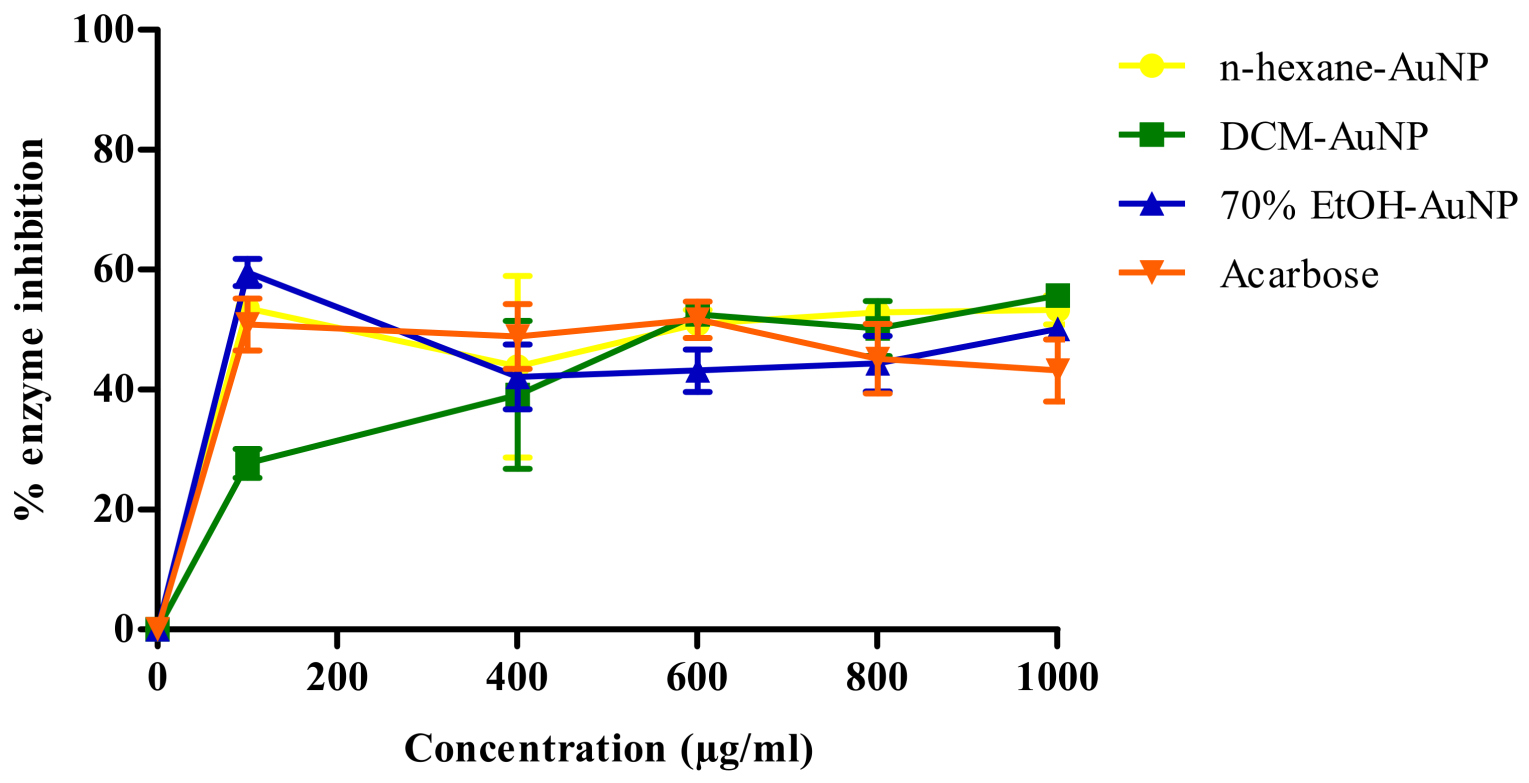
Effect of AuNPs on α-amylase activity. Data were expressed as mean ± standard deviation (SD) (*n* = 3). The results were analyzed with one-way analysis of variance (ANOVA), and the differences were considered statistically significant at *p* ≤ 0.05.

Figure 8 presents the results of AuNP on α-amylase activity. The 70% EtOH-AuNPs showed an IC_50_ value of 76.7 ± 0.02 µg/mL, followed by n-hexane-AuNPs with an IC_50_ of 84.6 ± 0.01 µg/mL and DCM-AuNPs with an IC_50_ of 380 ± 0.12 µg/mL. The reference inhibitor, acarbose, exhibited an IC_50_ value of 86.7 ± 0.02 µg/mL under the same experimental conditions. Thus, the 70% EtOH-AuNPs and n-hexane-AuNPs exhibited lower IC_50_ values than acarbose, whereas the DCM-AuNPs exhibited a higher IC_50_ value.

### 2.6. BSA-Fructose Glycation Assay

**Figure 9:**
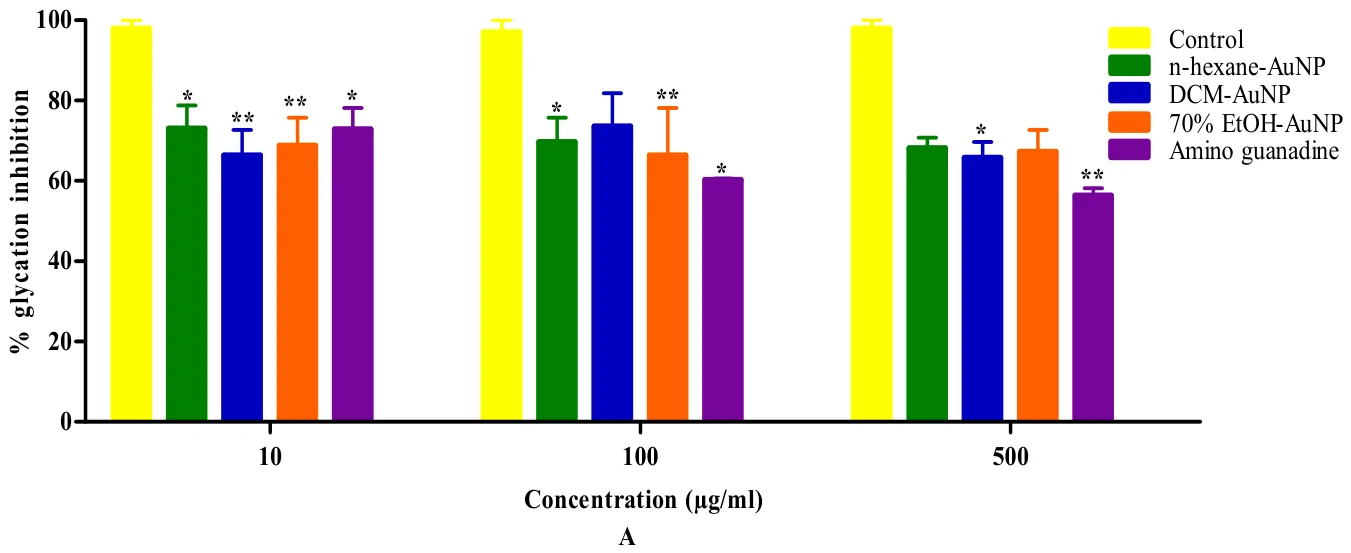

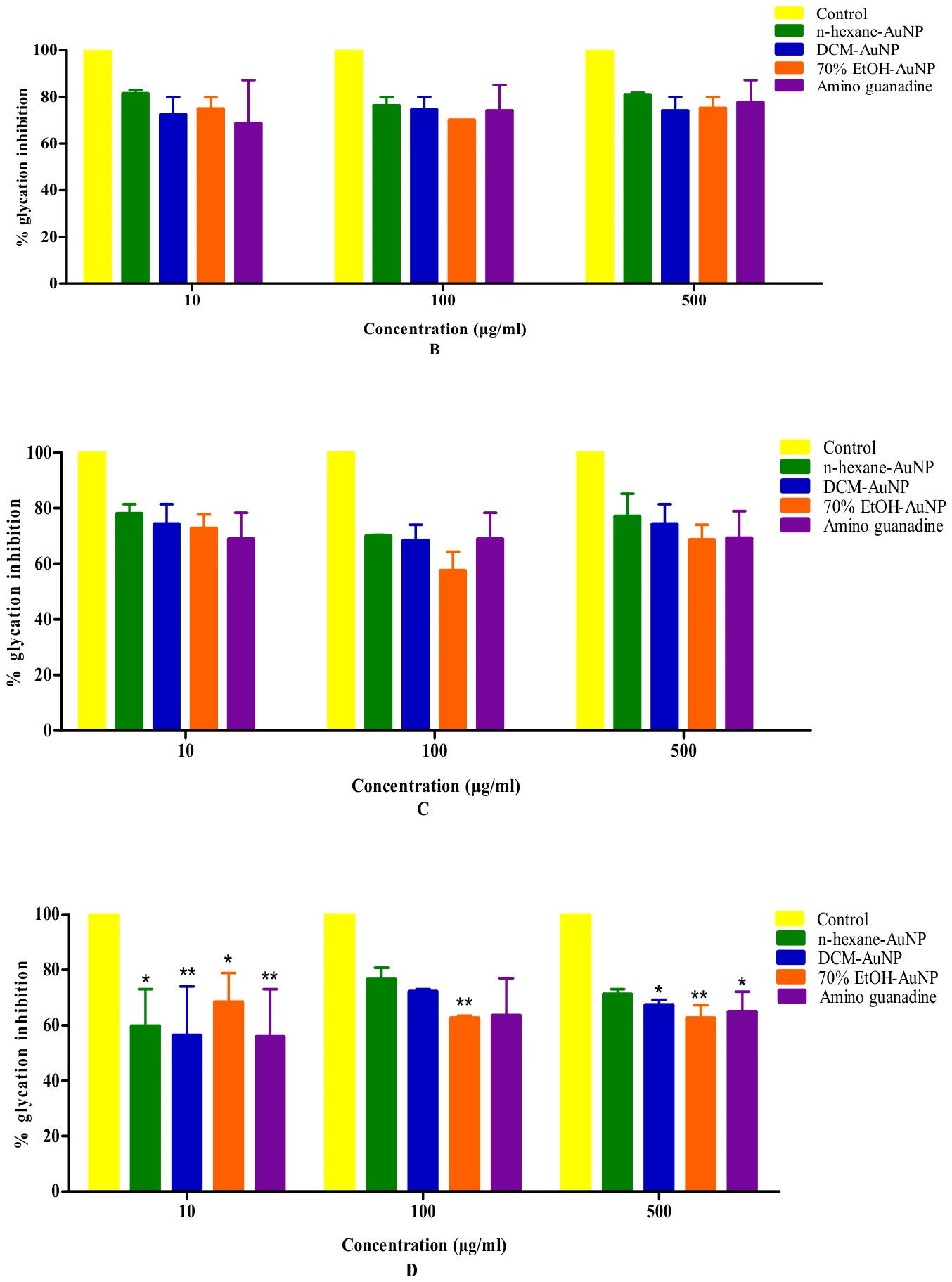
The effect of AuNP on BSA-fructose glycation: (**A**) day 7, (**B**) day 14, (**C**) day 21 and (**D**) day 28. Data were expressed as mean ± standard deviation (SD) (*n* = 3). The results were analyzed with one-way analysis of variance (ANOVA), * *p* ˂ 0.05, ** *p* ˂ 0.001, when compared to the normal control.

The antiglycation of AuNPs was evaluated on BSA over a period of 28 days at 7-day intervals. The results obtained are presented in Figure 9. The antiglycation activity of the AuNPs was evaluated using the BSA glycation model over a 28-day incubation period, with measurements taken at 7-day intervals. The results are presented in Figure 9. All AuNPs exhibited concentration-dependent inhibition of protein glycation throughout the incubation period. Notably, DCM-AuNPs demonstrated significant inhibition of AGE formation (*p* < 0.001) at the lowest tested concentration on both days 7 and 28, with an inhibitory effect comparable to that of the positive control, aminoguanidine. Furthermore, EtOH-AuNPs exhibited the greatest antiglycation activity at the highest tested concentration on day 28, significantly surpassing the inhibitory effect of aminoguanidine.

### 2.7. Molecular Docking Evaluation

**Figure 10:**
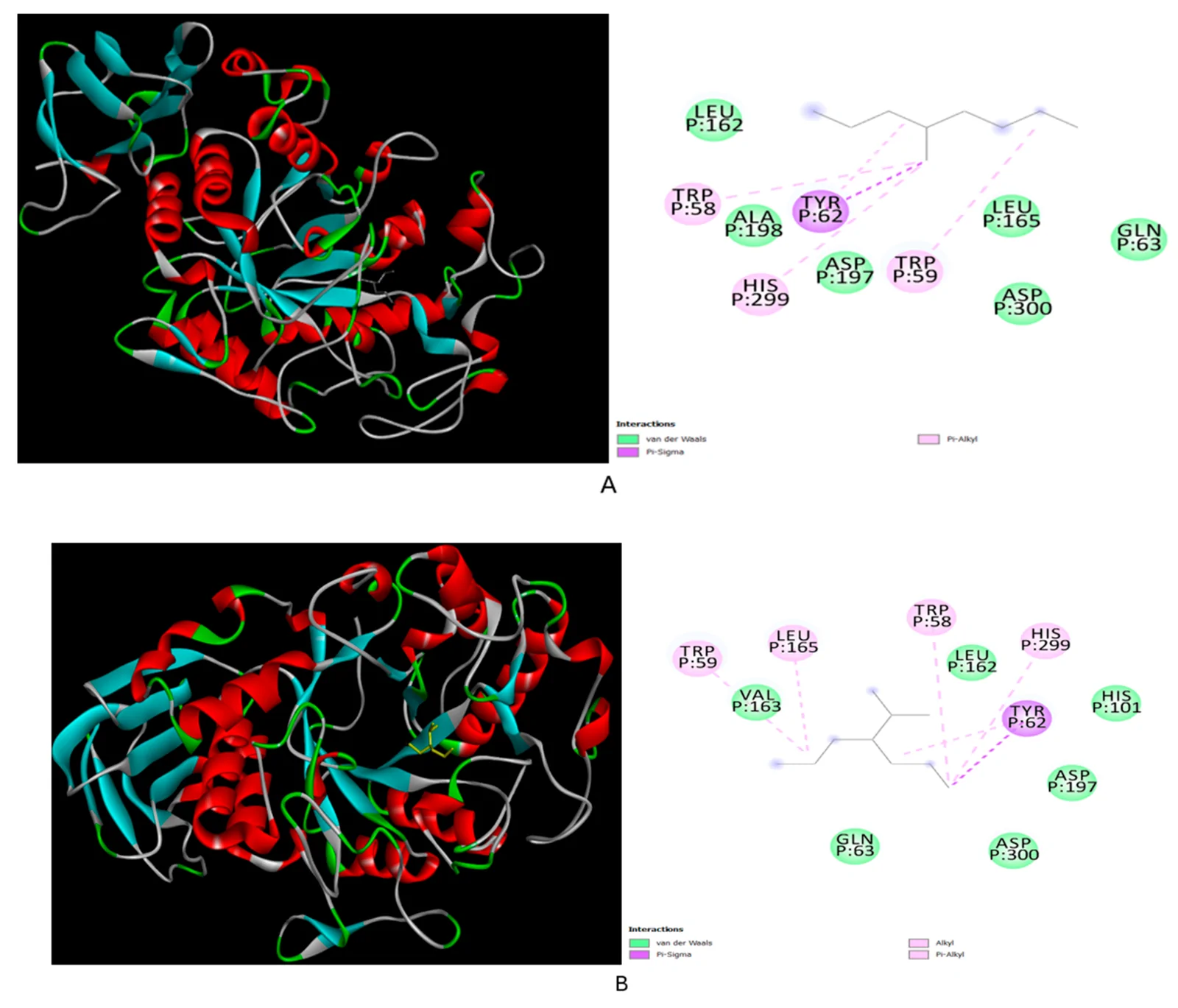

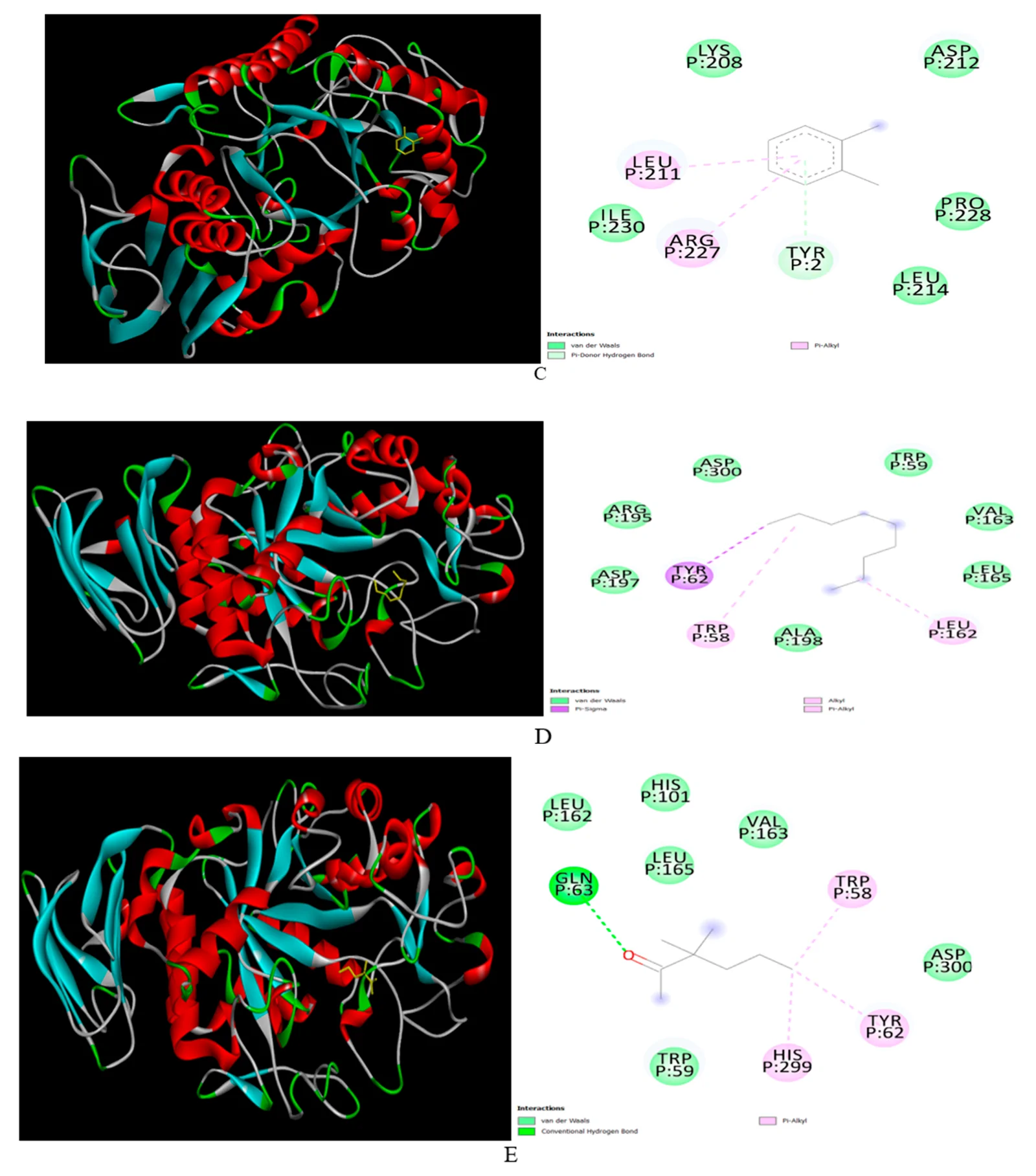

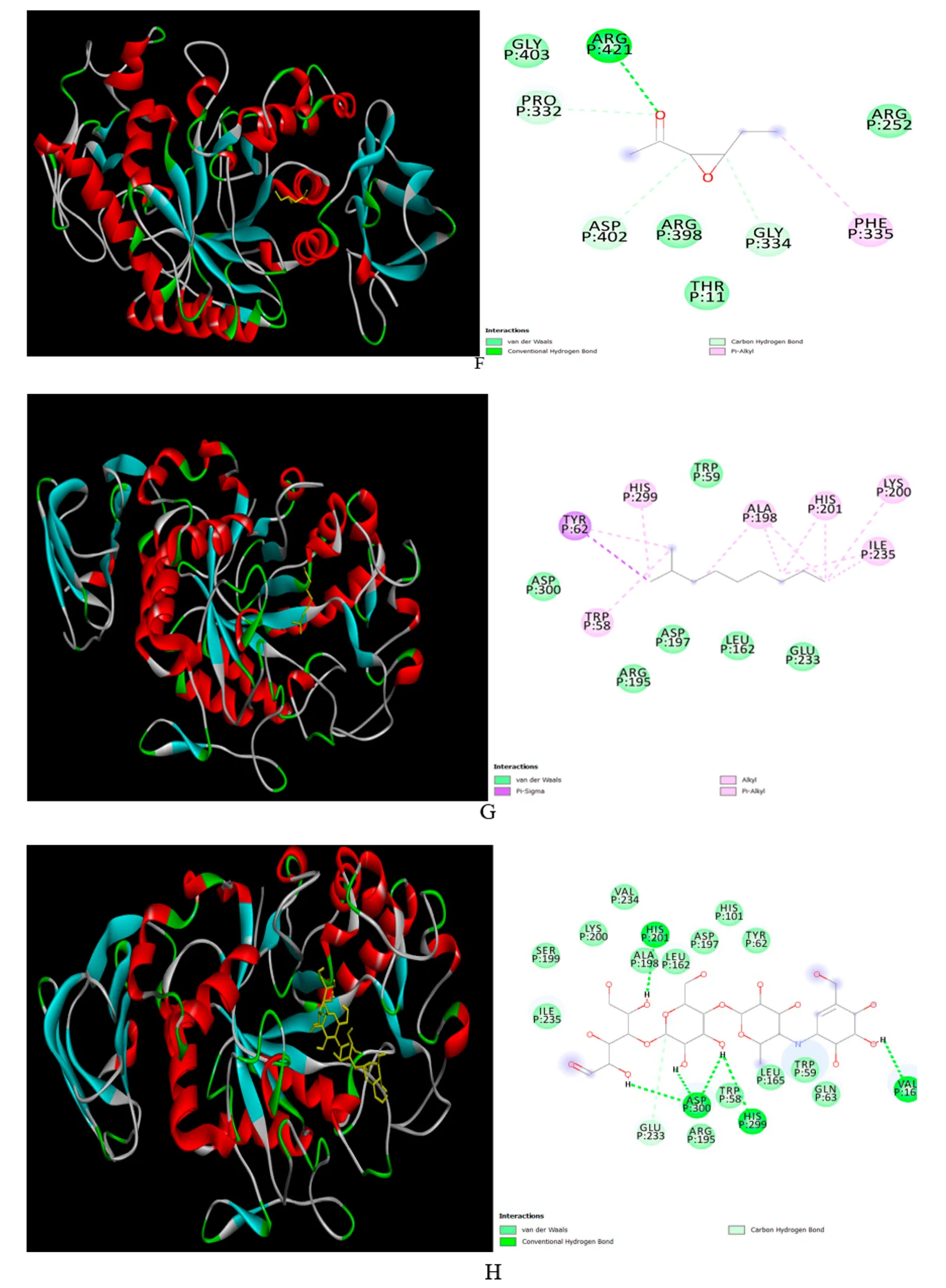

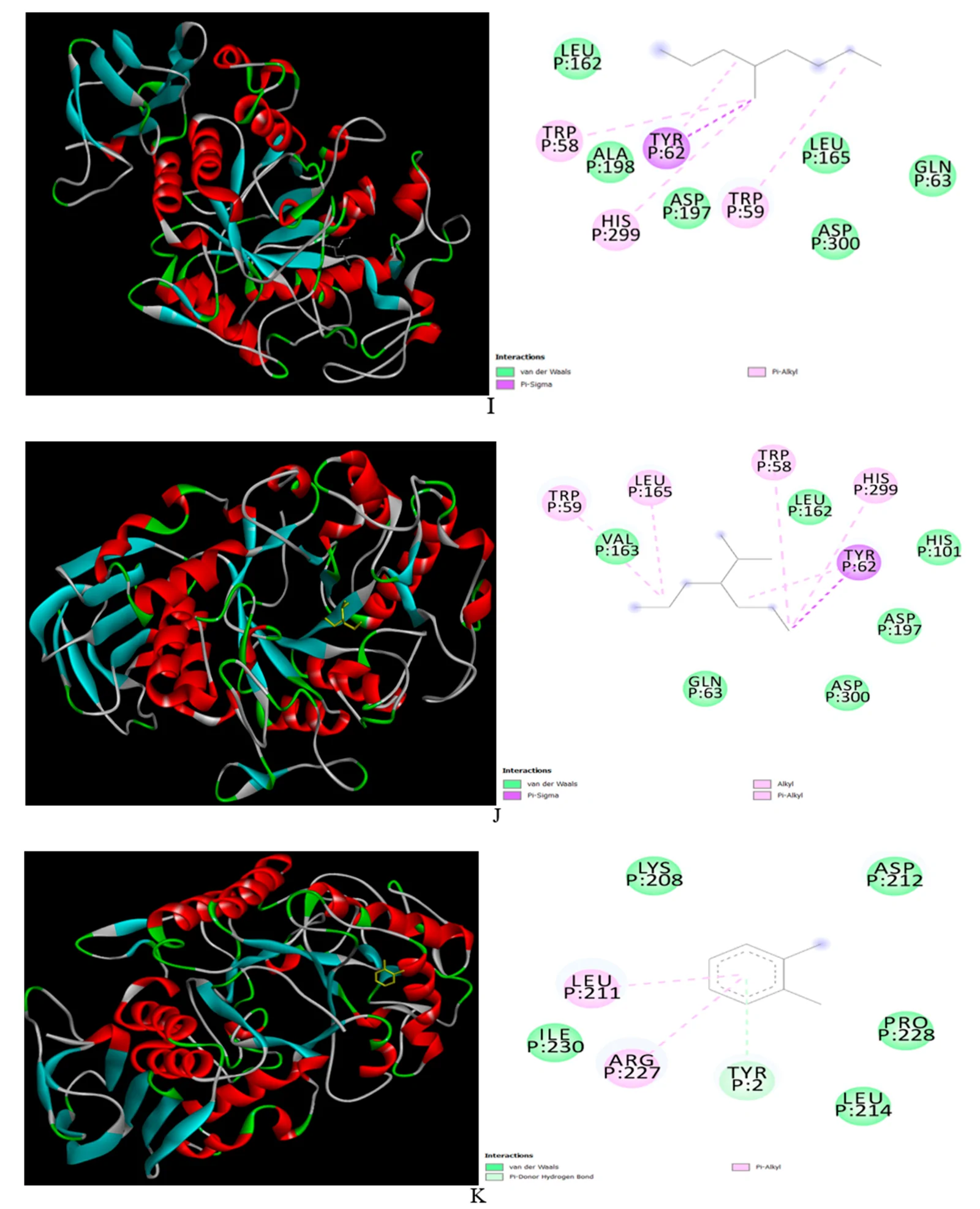

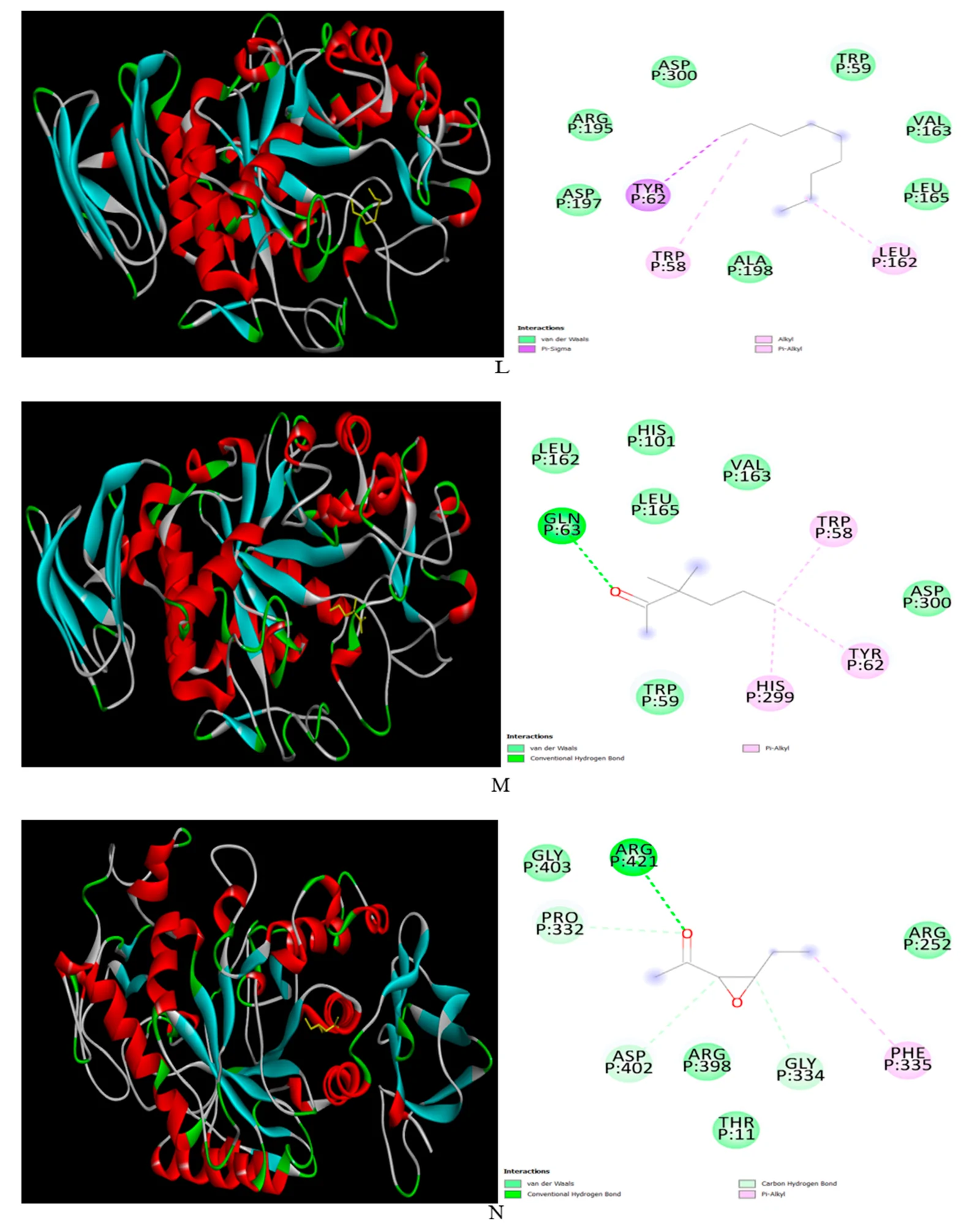

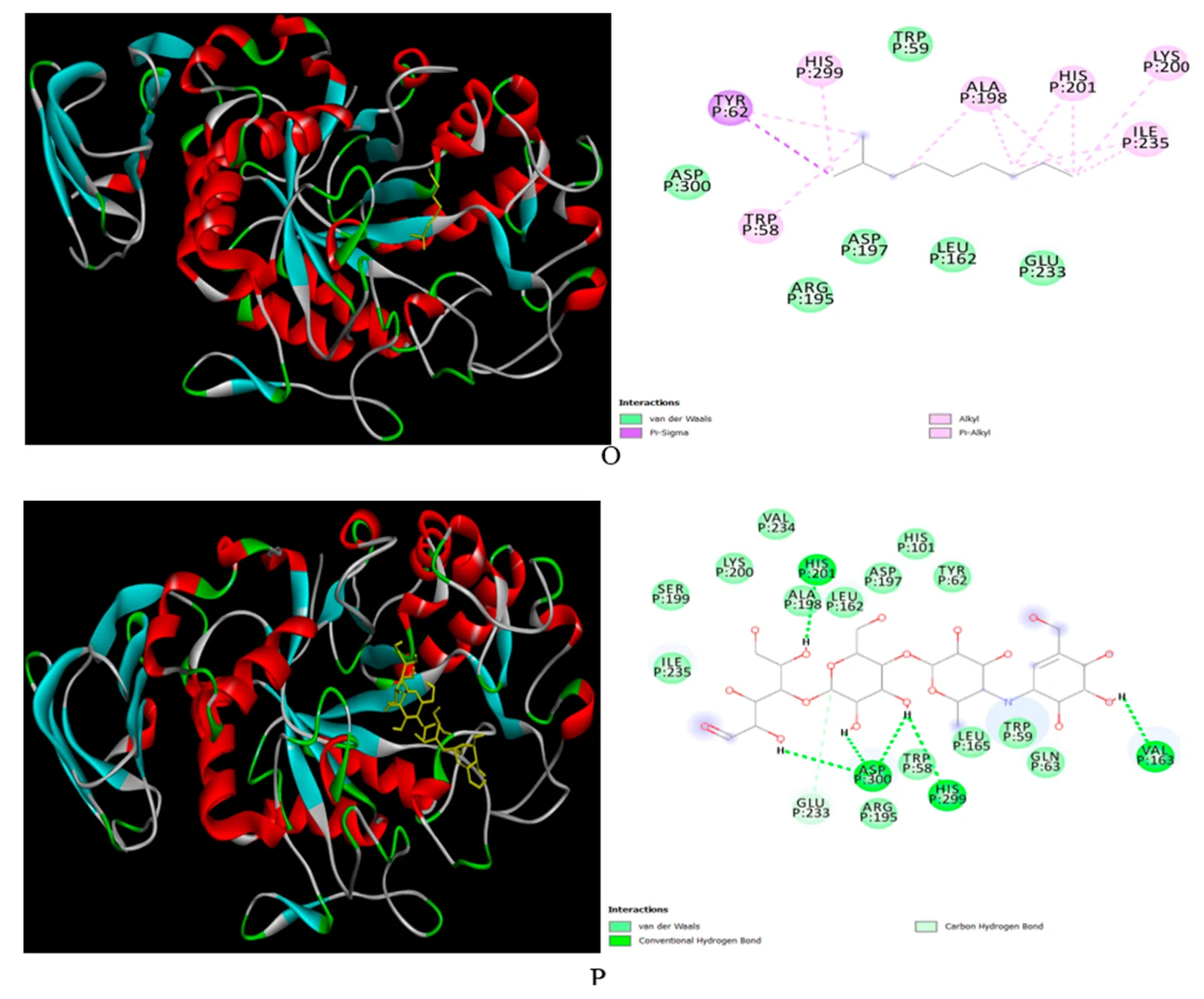
Molecular docking interactions of selected compounds with the target enzyme. A representation of the 2D and 3D structures of ligand–enzyme interactions, demonstrating the binding between specific amino acids and complexes. Whereby octane, 4-methyl- with (**A**)-α-amylase and (**I**)-α-glucosidase; Heptane, 4-(1-methylethyl)– with (**B**)-α-amylase: (**J**)-α-glucosidase; o-xylene–with (**C**)-α-amylase and (**K**)-α-glucosidase; Nonane with (**D**)-α-amylase and (**L**)-α-glucosidase; 2-hexanone, 3,3-dimethyl- with (**E**)-α-amylase and (**M**)-α-glucosidase; ethanone, 1-(3-ethyloxyranyl) with (**F**)-α-amylase and (**N**)-α-glucosidase, Nonane, 2-methyl- with (**G**)-α-amylase and (**O**)-α-glucosidase and acarbose with (**H**)-α-amylase and (**P**)-α glucosidase.

The docking scores presented in Table 3 represent the binding affinities of primary compounds to target proteins, accompanied by enzyme interaction binding between specific amino acids and complexes as shown in Figure 10, as well as standards, expressed in kilocalories per mole (kcal/mol). Interestingly, octane, 4-methyl-, Heptane, 4-(1-methylethyl), o-xylene, Nonane, 2-hexanone, 3,3-dimethyl-, ethanone, 1-(3-ethyloxyranyl)-, Nonane, 2-methyl-, and acarbose demonstrated significant binding affinity to α-amylase and α-glucosidase, respectively. These scores in Table 3 indicate a high binding affinity of these compounds to the diabetic enzymes. In addition, the docking score of o-xylene −5.6 kcal/mol for α-glucosidase is the highest binding affinity among the evaluated proteins.

## 3. Discussion

The increasing global reliance on traditional herbal medicines has generated significant interest in their potential as alternative and complementary therapeutic agents for various ailments, including diabetes mellitus. Medicinal plants are rich sources of secondary metabolites and bioactive compounds, including flavonoids, phenolics, alkaloids, and terpenoids, which exhibit diverse pharmacological activities. These phytochemicals have been reported to possess antioxidant, anti-inflammatory, antihyperglycemic, and antihyperlipidemic properties, making them promising candidates for the management of diabetes mellitus and its associated complications [23].

Their ability to modulate multiple biological pathways simultaneously offers a distinct advantage in the treatment of complex metabolic disorders. Recent advances in nanotechnology have further expanded the therapeutic potential of plant-derived compounds. Nanoparticles possess unique physicochemical characteristics, including a high surface area to volume ratio, tenable size, and enhanced interaction with biological systems, making them effective platforms for drug delivery and therapeutic applications. Among various nanomaterials, AuNPs have attracted considerable attention due to their excellent biocompatibility, chemical stability, low toxicity, and unique optical properties [24].

Moreover, AuNPs facilitate targeted and controlled drug delivery, improve the bioavailability of therapeutic agents, and protect bioactive compounds from degradation, thereby enhancing their efficacy [17,22]. The use of AuNPs with medicinal plant extracts combines the therapeutic benefits of phytochemicals with nanotechnology, offering a sustainable and promising approach for developing safe and effective treatments for several ailments, including diabetes mellitus [23]. Hence, this study aimed to synthesize and characterize AuNPs from *O. paniculosa* crude extracts and investigate some of their antidiabetic properties. 

The results obtained provide strong evidence for the successful biosynthesis of AuNPs using *O. paniculosa* crude extracts. While the extracts of *O. paniculosa* were employed as both reducing and stabilizing agents in the synthesis of AuNPs, the successful formation of AuNPs was confirmed through comprehensive physicochemical characterization. HR-TEM is an advanced imaging technique that uses a beam of high-energy electrons to visualize materials at the nanometre and even atomic scale to determine the size, shape, morphology, crystallinity, and internal structure of nanoparticles and other nanomaterials [24]. AuNPs are known to exhibit diverse morphologies, including spherical, cubic, rod-shaped, star-shaped, polyhedral, and triangular structures, typically ranging in size from 10 to 100 nm [23,25]. In this study, HR-TEM images (Figure 5) further validated the successful synthesis of AuNPs by revealing their size distribution and morphology. The observed nanoscale dimensions and structural characteristics are consistent with those reported for biologically synthesized AuNPs [26,27].

XRD analysis, a non-destructive analytical technique used to determine the crystalline structure, phase composition, crystallinity, and average crystallite size of materials, is one of the most important characterization tools for nanoparticles and other crystalline substances [28]. Our findings on XRD analysis (Figure 4) confirmed the crystalline nature of the synthesized AuNPs. UV-Vis spectroscopy, a rapid, non-destructive analytical technique that measures the absorption of ultraviolet and visible light by molecules and nanoparticles, is very specific in wavelengths, which induces electronic transitions between energy levels and generates an absorption spectrum that provides information on the sample’s composition, concentration, and optical properties [29]. 

The observed absorption maximum at 550–560 nm (Figure 6B) suggests the formation of predominantly spherical AuNPs with relatively larger particle sizes or slight aggregation compared with smaller AuNPs, which typically exhibit SPR bands near 520–530 nm. Furthermore, the absorption bands observed outside the characteristic SPR region (Figure 6A,C) are likely attributable to phytochemicals present in the leaf extract, which adsorb onto the nanoparticle surface and function as capping agents during biosynthesis. Similar findings were reported by [30,31] on UV-Vis observations. These findings demonstrate the dual role of the plant extract in mediating the reduction of gold ions and enhancing nanoparticle stability through phytochemical capping [32].

FT-IR spectroscopy, an analytical technique used to identify and characterize chemical compounds based on their interaction with infrared radiation, measures the absorption of infrared light by molecules at different wavelengths, producing a spectrum that serves as a unique molecular fingerprint of the sample [33]. In this study, FT-IR spectroscopy (Figure 3) revealed the involvement of phytochemical functional groups in the reduction and stabilization processes. Collectively, these findings provide strong evidence for the successful biosynthesis of AuNPs using *O. paniculosa* crude extracts, suggesting that the green synthesis approach from *O. paniculosa* may enhance the biological activity and therapeutic potential of the plant extract, particularly in the management of diabetes mellitus. 

Type 2 diabetes mellitus, which constitutes almost 90% of diabetic cases, remains one of the leading causes of death globally [3]. The observed inhibitory effect of AuNP on enzyme α-amylase activity (Figure 8) and prevention of advanced glycation end-products (AGEs) (Figure 9) could be one possible mechanism through which AuNP exhibits its antidiabetic potential. The salivary α-amylase catalyzes the hydrolysis of the linear α-(1.4)-glycosidic bonds of large insoluble carbohydrates into oligosaccharides and disaccharides. Persistent hyperglycemia is a principal etiology of diabetic complications, since excessive glucose in the bloodstream enters a toxic and non-enzymatic pathway leading to the formation of Schiff bases, which leads to the production of AGEs [34,35]. AGEs are known as key players in the progression of diabetes mellitus. Several studies have reported that green-synthesized nanoparticles from plant extracts exhibit partial antidiabetic properties through inhibitory effects on α-amylase and suppression of non-enzymatic protein glycation, thereby reducing postprandial hyperglycemia and preventing the formation of AGEs [36,37]. The antidiabetic properties of *O. paniculosa* crude extracts were further supported by the observed strong binding affinities with docking scores of −5.3 and −5.6 on α-amylase and α-glucosidase, demonstrated by o-xylene (Table 3) with the catalytic and regulatory sites of these enzymes, thereby interfering with substrate binding and enzymatic activity [38]. The docking analysis indicated that o-xylene interacted with amino acid residues including Arg, Asp, Trp, Ala, and Val through predicted carbon–hydrogen bonding interactions. These interactions suggest that o-xylene may be accommodated within functionally important regions of the enzyme and could potentially influence substrate recognition in the active-site environment. The favourable docking scores and predicted interactions provide a possible molecular explanation for the experimentally observed inhibitory effects of the AuNP preparations against α-amylase and α-glucosidase. In particular, the predicted interactions with residues such as Arg and Asp, together with hydrophobic residues such as Trp, Ala, and Val, may contribute to the stabilization of the ligand within the enzyme-binding pocket. In addition, the observed inhibitory effects of the *O. paniculosa* crude extracts on α-amylase activity and antiglycation could be attributed to the presence of phenolic and flavonoid contents (Figure 1). Studies have shown that antiglycation and enzyme activity inhibition effects are due to the presence of phenolic and flavonoid contents [39]. Furthermore, chronic hyperglycemia triggers cellular cascade reactions, leading to an increase in the production of reactive oxygen species (ROS), which causes oxidative stress. However, under physiological conditions, the body protects itself from oxidative stress through its antioxidant defence mechanism. Thus, the antioxidant properties demonstrated by AuNPs (Table 2) partially support their antioxidant property in the management of oxidative stress-induced diabetes mellitus. The observed antioxidant potential may be attributed to the presence of bioactive compounds such as octane, 4-methyl-, Heptane, 4-(1-, methylethyl)-, o-xylene, Nonane, 2-Hexanone, 3,3-, dimethyl-, ethanone, 1-(3-, thyloxiranyl)- and Nonane, 2-methyl (Table 1). The antioxidant properties of o-xylene, 2-Hexanone, 3,3-, dimethyl-have been reported [40]. Moreover, in vitro antioxidant properties of AuNPs have been reported [28,40]. The green synthesis of nanoparticles could be a significant approach in pharmaceutical applications due to their potential effect in oxidative stress inhibition, which is considered a key player in the progression of diabetes [31,32]. 

## 4. Materials and Methods

### 4.1. Chemical Reagents

Unless stated otherwise, all chemicals and reagents used in the study were purchased from Sigma-Aldrich Co. Ltd., St. Louis, MO, USA.

### 4.2. Methodology

#### Collection, Preparation and Solvent Extraction of *O. paniculosa*

The fresh leaves of *O. paniculosa* were collected at Ndumo, KwaZulu-Natal, South Africa (26°53′ S 32°16′ E), identified and catalogued in the Department of Botany, University of Zululand. The plant material was air-dried at room temperature and ground into fine powder (grinding machine: IKA-Werke, Polychem suppliers) (IKA-Werke GmbH & Co. KG, Staufen, Germany). The powdered material was sequentially extracted with an increasing polarity of different solvents (ratio of 1:5 *w*/*v*), namely, n-hexane, dichloromethane (DCM), and 70% ethanol (70% EtOH) (incubated for 72 h on a shaker at 180 rpm, 30 °C) and each extract was rinsed twice with the same solvent of extraction for another 48 h, respectively. Thereafter, the extracts were subjected to filtration via Whatman No.1 filter paper (Whatman, Maidstone, Kent, UK) and subsequently concentrated under reduced pressure using a rotary evaporator at 40 °C. The obtained extracts were preserved in sterile brown bottles at 4 °C to prevent light-induced degradation until used for characterization and the synthesis of extract-passivated AuNPs.

### 4.3. Phytochemical Profiling

#### 4.3.1. Determination of the Total Phenolic Content (TPC)

The method described by [41] was used to determine the TPC of *O. paniculosa* extracts. In brief, 1.5 mL of diluted (1:10 *v*/*v*) Folin−Ciocalteu reagent and 1.2 mL of 7.5% sodium carbonate solution were mixed with each extract (0.5 mg/mL) and then kept in the dark for 30 min at 21 °C. The absorbance of the coloured mixture was read at 765 nm. The TPC of *O. paniculosa* was calculated as gallic acid equivalent to a calibration curve of gallic acid and expressed as mg/g dry crude extract. 

#### 4.3.2. Determination of the Total Flavonoid Content (TFC)

The method previously described by [42] was used to determine the TFC of *O. paniculosa* extracts. In brief, 0.5 mg/mL of each extract, added to 0.5 mL of 2% alcoholic aluminum chloride solution, was incubated at standard room temperature for 1 h. The absorbance of the colour change was read at 420 nm against the blank containing alcoholic aluminum chloride. The TFC of the extract was calculated from a standard curve of quercetin and expressed as mg/g dry crude extract.

#### 4.3.3. The FT-IR Spectral and GC-MS Analysis of *O. paniculosa* Extracts

The method by [43] was used to determine the functional groups of *O. paniculosa* extracts. FT-IR spectrophotometer (PerkinElmer SA (Pty) Ltd., Midrand, South Africa) at room temperature was used to scan the 370–4000 cm^−1^ spectral range, and the functional groups were determined by comparing the peak frequencies to the IR spectroscopy correlation table. 

*O. paniculosa* extracts were phytochemically profiled using GC-MS following a modified method of [44]. The extracts were analyzed using an Agilent 7890A GC(Agilent Technologies, Santa Clara, CA, USA) coupled to a 5975C mass spectrometer (Agilent Technologies, Santa Clara, CA, USA) under optimized chromatographic conditions. Samples were injected into the GC column, separated according to their chemical properties, and detected by MS using electron ionization at 70 eV. The compounds were identified by comparing their mass spectra and retention times with compounds in the NIST 2011 library. The relative abundance of each identified compound was calculated as its percentage peak area relative to the total chromatographic peak area.

### 4.4. Synthesis of Extract-Passivated AuNPs

Following the modified method reported elsewhere [22], 10 mg of gold chloride trihydrate (HAuCl_4_·3H_2_O; batch no. MKCH8321; molecular weight 393.83 g/mol) was dissolved in 40 mL of 70% ethanol to obtain a 0.64 mM gold precursor solution. Separately, 15 mg of each crude extract was dispersed in 25 mL of 70% ethanol. The precursor and extract solutions were then combined to obtain a final reaction volume of 65 mL. Thus, the final concentrations in the reaction mixture were approximately 0.391 mM gold precursor and 0.231 mg/mL crude extract, corresponding to a mass ratio of 10:15 (1:1.5) of gold precursor to crude extract. The reaction mixture was stirred at 37 °C until a visible colour change and precipitate formation were observed. Thereafter, the mixture was centrifuged at 6000 rpm at room temperature for 10 min. The supernatant was decanted, and the resulting AuNP precipitate was washed thoroughly with 70% ethanol. The purified AuNPs were subsequently air-dried at room temperature for 24 h.

#### Characterization

*O. paniculosa* extracts were phytochemically profiled using an Agilent 7890A GC coupled (Agilent Technologies, Santa Clara, CA, USA) to a 5975C mass spectrometer (Agilent Technologies, Santa Clara, CA, USA) under optimized chromatographic conditions adapted from a method reported elsewhere [45,46,47]. The compounds were identified by matching their spectral data to the NIST 2011 library database. 

The functional group identification of the *O. paniculosa* extracts and the corresponding AuNP composites was conducted on a PerkinElmer Spectrum 2 Fourier Transform Infrared (FT-IR) spectrometer (PerkinElmer SA (Pty) Ltd., Midrand, South Africa) equipped with a uATR. The spectra were recorded at room temperature, in the 500 to 4000 cm^−1^ range. 

The structural and crystallographic phase patterns of the extracts–AuNPs composites were recorded at room temperature, on a Bruker AXS D8 diffractometer (Bruker AXS, Karlsruhe, Germany), which uses nickel-filtered Cu Kα radiation (λ = 1.5418 Å) at 40 kV and 40 mA. Scans were recorded in the high-angle 2θ range of 30–80° at a scan speed of 0.5 s/step. 

Morphological, crystallographic and elemental composition features of the extracts–AuNPs composites were recorded on a JEOL F200 high-resolution electron transmission microscope (HR-TEM) (JEOL Ltd., Tokyo, Japan) equipped with a Gatan Rio camera (Gatan, Inc., Pleasanton, CA, USA) and Oxford X-Max^n^ 80 T Energy Dispersive X-ray Spectroscopy (EDS) detector (Oxford Instruments, Abingdon, UK). The microscope was operated at an accelerating voltage of 200 kV. EDS data were captured and processed using the AZtec software suite (version 6.3). The optical absorbance measurements of the extracts–AuNPs composites were recorded on a PerkinElmer Lambda 1050 UV-Vis-NIR spectrophotometer, using a PerkinElmer UV/NIR spectroscopy cell quartz cuvette (B0631107) (PerkinElmer, Waltham, MA, USA) and ethanol as a dispersing solvent.

### 4.5. In Vitro Antioxidant Assays

The antioxidant potential of the synthesized AuNPs was investigated using four complementary assays, namely DPPH and ABTS radical-scavenging assays, metal-ion chelation, and reducing power determination. Unless stated otherwise, methanol was used as the negative/vehicle control because it was the solvent used for dissolving and/or diluting the test samples and was therefore included to account for any response attributable to the solvent rather than the tested AuNPs. Ascorbic acid was used as the positive control/reference antioxidant because of its established antioxidant activity. The responses obtained for the AuNP samples were therefore compared with the solvent control and the ascorbic acid standard to assess the antioxidant activity of the synthesized nanoparticles under the experimental conditions. All measurements were performed in three independent replicates (*n* = 3). The percentage of free-radical scavenging was determined according to the following equation.

Scavenging activity (%) = ((A_c_ − A_t_)/A_c_ × 100)

where A_c_ is the absorbance of the control sample, and A_t_ is the absorbance of the AuNP sample.

#### 4.5.1. DPPH Radical-Scavenging Activity

The free-radical-scavenging capacity of the synthesized AuNPs was evaluated using the DPPH assay according to the procedure described by [48], with minor modifications. In brief, equal volumes (1:1, *v*/*v*) of DPPH solution (500 μg/mL) and AuNP suspensions at varying concentrations (0–1000 μg/mL) were mixed and incubated in the dark for 60 min at room temperature. Following incubation, the absorbance of the reaction mixtures was measured at 517 nm. Methanol was used as the negative control, whereas ascorbic acid served as the positive control. The DPPH radical-scavenging activity was subsequently expressed as a percentage and calculated using the appropriate equation.

#### 4.5.2. ABTS Radical-Scavenging Activity

The ABTS radical-scavenging capacity of the *O. paniculosa*-mediated AuNPs was determined according to the method reported by [49], with appropriate modifications. The ABTS radical cation was generated by mixing 7 mM ABTS with 2.45 mM potassium persulfate, followed by incubation in the dark for 16 h. The resulting ABTS radical solution was subsequently diluted with methanol at a ratio of 1:60 (1 mL ABTS solution to 60 mL methanol) to obtain the working solution. For the assay, 1 mL of the diluted ABTS working solution was combined with 1 mL of AuNPs at concentrations ranging from 0 to 1000 μg/mL. The reaction mixtures were incubated at room temperature for 6 min, after which absorbance was measured at 734 nm. Ascorbic acid and methanol were used as the positive and negative controls, respectively. The radical-scavenging activity was expressed as a percentage and calculated using the corresponding equation. 

#### 4.5.3. Reducing Power

The reducing capacity of the AuNPs was assessed using the method previously reported by [50], with modifications where applicable. In brief, 2.5 mL of AuNP suspensions at concentrations ranging from 0 to 1000 μg/mL were mixed with 2.5 mL of 1% (*w*/*v*) potassium ferricyanide and 2.5 mL of 200 mM phosphate buffer (pH 6.6). The reaction mixtures were incubated at 50 °C for 20 min. After incubation, the samples were centrifuged at 1000 rpm for 10 min, and the resulting supernatants were collected. Subsequently, 0.05% ferric chloride (FeCl_3_) was added to the supernatants, and the absorbance was measured at 700 nm. The reducing power of the AuNPs was determined based on the absorbance values obtained, with higher absorbance indicating greater reducing capacity.

#### 4.5.4. Metal-Ion (Fe^2+^) Chelating Activity Assay

The Fe^2+^-chelating activity of the AuNPs was evaluated following the procedure described by [51], with suitable modifications. In brief, AuNP suspensions at concentrations ranging from 0 to 1000 μg/mL were reacted with 2.5 mM ferrozine and 1 mM FeCl_2_ in deionized water. The reaction mixtures were incubated at 25 °C for 10 min to allow the formation of the ferrozine Fe^2+^ complex. Subsequently, the absorbance was measured at 562 nm using a spectrophotometer. Citric acid was employed as the reference standard, and the Fe^2+^-chelating activity was expressed as a percentage based on the corresponding absorbance values.

### 4.6. Enzyme Inhibition Assay

#### Effect of AuNPs on α-Amylase Activity

The inhibitory activity of the AuNPs against α-amylase was assessed according to the procedure reported by [52], with modifications. In brief, α-amylase (2000 μg/mL) and 0.5% (*w*/*v*) starch were prepared in 15 mM phosphate buffer (pH 7.0). The enzyme-substrate mixture was subsequently treated with different concentrations of AuNPs (0–1000 μg/mL) and incubated at 37 °C for 15 min. Following incubation, 120 μL of 3,5-dinitrosalicylic acid (DNS) reagent was added to terminate the enzymatic reaction. The reaction mixtures were then heated at 95 °C for 5 min, after which the absorbance was measured at 540 nm. Acarbose was included as the reference inhibitor. The inhibitory activity of the AuNPs was expressed as percentage inhibition and calculated using the following equation:

Inhibitory activity (%) = ((A_c_ − A_t_)/A_c_ × 100)

where A_c_ is the absorbance of the control, and A_t_ is the absorbance in the presence of the AuNP sample.

### 4.7. The Effect of AuNP on Bovine Serum Albumin (BSA)-Fructose Glycation

The inhibitory effect of AuNPs on BSA glycation was evaluated following the procedure reported by [53,54], with appropriate modifications. The assay mixture consisted of BSA (0.5 mg/mL) and fructose (25 M) prepared in 0.1 M phosphate-buffered saline (PBS, pH 7.4). Different concentrations of AuNPs ranging from 0 to 1000 µg/mL were incorporated into the reaction mixtures. Sodium azide (0.01%) was subsequently added to minimize microbial growth. The prepared samples were incubated in the dark at 37 °C for 28 days, with measurements performed at 7-day intervals. Aminoguanidine was included as the reference antiglycation agent. AGE formation was quantified fluorometrically using excitation and emission wavelengths of 355 and 460 nm, respectively. 

### 4.8. Molecular Docking Evaluation

As described by [55], the in silico antihyperglycemic activity of the selected phytochemical compounds was evaluated using molecular docking techniques. Molecular docking was performed using PyRx 0.8 and BIOVIA Discovery Studio 2021 to assess the binding potential of selected ligands against target proteins involved in glucose metabolism. The 2D structures of the selected compounds (CID 822, 985, 5280435, 5363097, and 5367327), together with the respective positive-control compounds, were retrieved from the PubChem database. The corresponding 3D structures of the target proteins, including α-amylase (PDB ID: 1BVN) and glucosidase (PDB ID: 1UP4), were obtained from the Protein Data Bank (PDB).

The retrieved ligands were prepared and energy-minimized using PyRx, while the protein structures were prepared using BIOVIA Discovery Studio. Molecular docking simulations were subsequently performed using PyRx to predict the binding affinities and interactions between the selected ligands and target proteins. Ligand–protein complexes exhibiting favourable binding affinities (>5.4 kcal/mol) were further analyzed and visualized in both 2D and 3D representations using BIOVIA Discovery Studio 2021. The resulting interactions were used to assess the potential of the identified compounds to modulate proteins associated with glucose metabolism and, consequently, their possible contribution to the antihyperglycemic activity of *O. paniculosa* crude extracts.

## 5. Conclusions

This study successfully synthesized AuNPs using *Ozoroa paniculosa* leaf extract via a green, phyto-mediated approach and comprehensively characterized them using UV–Vis spectroscopy, FT-IR spectroscopy, XRD, and HR-TEM, confirming the formation of predominantly crystalline, spherical and triangular AuNPs. The biosynthesized AuNPs exhibited in vitro antidiabetic activity through the inhibition of α-amylase and AGE formation, alongside potent antioxidant properties. These findings highlight the potential of *O. paniculosa*-mediated AuNPs as multifunctional nanomaterials for diabetes management. Nevertheless, further studies are required to elucidate the underlying molecular mechanisms, evaluate their biocompatibility and toxicity, and validate their therapeutic efficacy through in vivo and clinical investigations before potential biomedical application.

## Figures and Tables

**Table 1:** GC-MS analysis of *O. paniculosa* leaf extracts.

Compound Name	RT (min)	n-Hexane	DCM	70% EtOH
4-methyl-octane	4.134	0.41	0.32	0.85
4-(1-methylethyl)-Heptane	4.225	0.24	0.14	0.36
o-xylene	4.308	0.49	0.19	0.39
Nonane	4.917	0.80	0.75	1.96
3,3-dimethyl-2-Hexanone	4.736	1.92	0.94	2.35
1-(3-ethyloxiranyl)-ethanone	4.934	2.24	0.91	1.92
2-methyl-Nonane	5.371	0.18	0.03	0.24

**Table 2:** AuNP IC_50_ values of DPPH, ABTS and metal iron chelating.

NP IC_50_ (µg/mL)	DPPH	ABTS	Metal Fe^2+^ Chelating
n-hexane-AuNP	-	-	76.7 ± 0.02
DCM-AuNP	-	-	76.7 ± 0.11
70% EtOH-AuNP	-	-	83.3 ± 0.03
Ascorbic Acid	96.7 ± 0.00	580 ± 0.03	-
Citric acid	-	-	63.3 ± 0.04

Data were expressed as mean ± standard deviation (SD) (*n* = 3). The results were analyzed with one-way analysis of variance (ANOVA), and the differences were considered statistically significant at *p* ≤ 0.05 (whereby ‘-’ means IC_50_ values not reached within the tested concentration range).

**Table 3:** Docking scores of tested compounds against selected target proteins.

Compounds	Binding Affinity (kcal/mol) 1BVN Amylase	Binding Affinity (kcal/mol) 1UP4Glucosidase
Octane, 4-methyl-	−4	−4.6
Heptane, 4-(1-methylethyl)	−4.5	−4.9
O-xylene	−5.3	−5.6
Nonane	−3.9	−4.5
2-hexanone, 3,3-dimethyl-	−4.4	−5.4
Ethanone, 1-(3-ethyloxyranyl)-	−4.2	−5.4
Nonane, 2-methyl-	−4.1	−5
Acarbose	−7.0	−8.7

## Data Availability

The original contributions presented in this study are included in the article and Supplementary Materials. Further inquiries can be directed to the corresponding author.

## Supplementary Materials

The following supporting information can be downloaded at: https://www.mdpi.com/article/10.3390/ph19101635/s1, File S1—GC-MS analysis.
