# Supplementary material for: Phyto-Mediated Synthesized Gold Nanoparticles (AuNPs) from Ozoroa paniculosa Leaf Extracts: Chemical Characterization and Antidiabetic Evaluation
Source: Pharmaceuticals (Basel). 2026 Oct 15;19(10):1635. doi: 10.3390/ph19101635 (PMC13708053; doi:10.3390/ph19101635)

Qualitative Analysis Report

Sample Information

Sample Type	: Unknown
Sample Name	: C:\GCMSData\Raw Data\Ext - UniZulu\16-E-C.qgd
Sample ID	: Prince
Vial #	: 98
Method File	: C:\GCMSData\general - split - extended.qgm
Tuning File	: C:\GCMSsolution\System\Tune1\Normal Conc - 2025 04.qgt

Chromatogram OPE hex

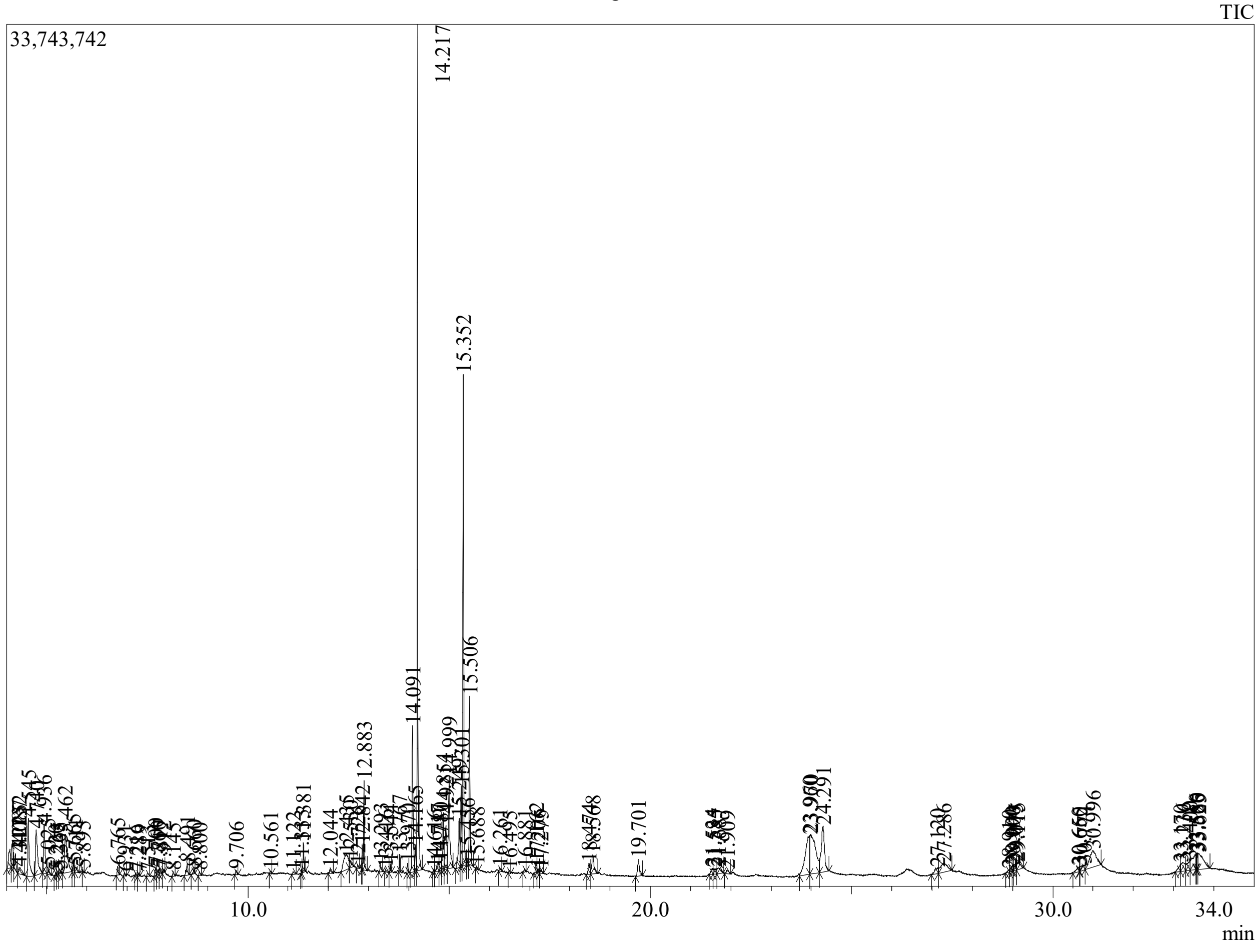

Peak Report TIC

Peak#	R.Time	Area	Area%	Height	Height%	A/H	Name
1	4.087	2565709	0.93	748303	0.63	3.43	1-Heptanol, 6-methyl-
2	4.132	2343272	0.85	924584	0.78	2.53	Octane, 4-methyl-
3	4.225	998223	0.36	643879	0.55	1.55	Heptane, 4-(1-methylethyl)-
4	4.317	1063211	0.39	259023	0.22	4.10	o-Xylene
5	4.545	5398886	1.96	2285544	1.94	2.36	Nonane
6	4.740	6471554	2.35	1792286	1.52	3.61	2-Hexanone, 3,3-dimethyl-
7	4.936	5274422	1.92	1930980	1.64	2.73	Ethanone, 1-(3-ethyloxiranyl)-
8	5.025	700544	0.25	288181	0.24	2.43	1,3,2-Dioxaborolane-4-methanol, 2-ethyl-, acet
9	5.220	169907	0.06	101770	0.09	1.67	Oxalic acid, dodecyl hexyl ester
10	5.265	215924	0.08	134583	0.11	1.60	Oxalic acid, dodecyl hexyl ester
11	5.335	252214	0.09	103363	0.09	2.44	Isobutyl isopropyl carbonate
12	5.462	6747939	2.46	1513940	1.28	4.46	3-Hexen-2-one
13	5.665	649239	0.24	369767	0.31	1.76	Nonane, 2-methyl-
14	5.711	759272	0.28	219127	0.19	3.46	Benzene, 1,2,4-trimethyl-
15	5.895	132156	0.05	106922	0.09	1.24	Pentane, 2,3,3-trimethyl-
16	6.765	698252	0.25	353588	0.30	1.97	Nonane, 2-methyl-
17	6.931	216521	0.08	126897	0.11	1.71	1-Pentanol, 2,2-dimethyl-
18	7.216	209733	0.08	127596	0.11	1.64	3-Methylpentan-3-yl propyl carbonate
19	7.289	146236	0.05	101690	0.09	1.44	3-Methylpentan-3-yl propyl carbonate
20	7.513	317484	0.12	124901	0.11	2.54	1,1-Hexylenedioxybutane
21	7.700	439411	0.16	326391	0.28	1.35	3-Heptanol, 2,6-dimethyl-
22	7.760	559816	0.20	194109	0.16	2.88	6,6,7-Trimethyl-octane-2,5-dione
23	7.817	543430	0.20	219736	0.19	2.47	Undecane, 3,7-dimethyl-
24	7.912	203180	0.07	107154	0.09	1.90	6,6,7-Trimethyl-octane-2,5-dione
25	8.145	350172	0.13	173588	0.15	2.02	n-Caproic acid vinyl ester
26	8.491	1066543	0.39	443090	0.38	2.41	n-Caproic acid vinyl ester

Qualitative Analysis Report

Peak#	R.Time	Area	Area%	Height	Height%	A/H	Name
27	8.660	399287	0.15	218521	0.19	1.83	2-Pentenoic acid, 4-hydroxy-
28	8.800	355576	0.13	156384	0.13	2.27	Octane, 2,3,3-trimethyl-
29	9.706	382107	0.14	160521	0.14	2.38	Nonadecane, 2-methyl-
30	10.561	238433	0.09	109297	0.09	2.18	.beta.-copaene
31	11.122	330986	0.12	112686	0.10	2.94	Cyclohexanemethanol, 4-ethenyl-.alpha.,.alpha
32	11.333	234983	0.09	169641	0.14	1.39	1-Dodecanesulfonyl chloride
33	11.381	1885052	0.69	1175547	1.00	1.60	Tridecane, 6-propyl-
34	12.044	540238	0.20	191293	0.16	2.82	2-Naphthalenemethanol, decahydro-.alpha.,.alp
35	12.435	5056485	1.84	618997	0.52	8.17	.alpha.-d-Mannofuranoside, methyl
36	12.530	879463	0.32	321542	0.27	2.74	1-Methoxy- 5-trimethylsilyloxy-3-phenylpenta
37	12.726	248133	0.09	131302	0.11	1.89	Tridecanoic acid
38	12.842	1320872	0.48	972900	0.82	1.36	1-Heptadecene
39	12.883	4562665	1.66	3498844	2.96	1.30	Tridecane, 6-propyl-
40	13.293	650219	0.24	357222	0.30	1.82	3-Buten-2-ol, 2-methyl-4-(1,3,3-trimethyl-7-ox
41	13.416	248047	0.09	121031	0.10	2.05	Pentadecanoic acid
42	13.544	347212	0.13	321020	0.27	1.08	Pentadecanoic acid, ethyl ester
43	13.767	829283	0.30	684811	0.58	1.21	Pentadecanoic acid, 14-methyl-, methyl ester
44	13.970	486024	0.18	391084	0.33	1.24	1,6,10-Dodecatriene, 7,11-dimethyl-3-methylen
45	14.091	10349983	3.77	5732202	4.86	1.81	n-Hexadecanoic acid
46	14.165	1616487	0.59	1035904	0.88	1.56	(E)-9-Octadecenoic acid ethyl ester
47	14.217	44342700	16.14	33124557	28.06	1.34	Hexadecanoic acid, ethyl ester
48	14.616	127043	0.05	112603	0.10	1.13	2-Bromononane
49	14.717	1277369	0.46	320045	0.27	3.99	(E)-9-Octadecenoic acid ethyl ester
50	14.780	976793	0.36	347517	0.29	2.81	Oxalic acid, 6-ethyloct-3-yl isobutyl ester
51	14.854	3964544	1.44	2247468	1.90	1.76	Heptadecanoic acid, ethyl ester
52	14.921	6007107	2.19	1732039	1.47	3.47	8,11,14-Eicosatrienoic acid, methyl ester
53	14.999	10002028	3.64	3622608	3.07	2.76	Phytol
54	15.249	5237070	1.91	1929746	1.63	2.71	9,12,15-Octadecatrienoic acid, (Z,Z,Z)-
55	15.301	4502720	1.64	3154705	2.67	1.43	9,12-Octadecadienoic acid, ethyl ester
56	15.352	31617065	11.50	19235764	16.30	1.64	9,12,15-Octadecatrienoic acid, (Z,Z,Z)-
57	15.456	301789	0.11	219526	0.19	1.37	Hexadecanoic acid, butyl ester
58	15.506	9845246	3.58	6576506	5.57	1.50	Eicosanoic acid, ethyl ester
59	15.688	152164	0.06	111142	0.09	1.37	Benzoic acid, tetradecyl ester
60	16.261	396994	0.14	147187	0.12	2.70	Nonadecanoic acid, ethyl ester
61	16.495	189439	0.07	114818	0.10	1.65	Benzoic acid, pentadecyl ester
62	16.881	320734	0.12	135633	0.11	2.36	9,12,15-Octadecatrienoic acid, (Z,Z,Z)-
63	17.162	707821	0.26	394742	0.33	1.79	Docosanoic acid, ethyl ester
64	17.206	207041	0.08	138287	0.12	1.50	Tridecane, 7-propyl-
65	17.275	447643	0.16	153414	0.13	2.92	1,3-Bis-(2-cyclopropyl,2-methylcyclopropyl)-b
66	18.474	1414039	0.51	438967	0.37	3.22	Benzene, 1-methyl-4-(2-pentenyloxy)-
67	18.568	2922953	1.06	746724	0.63	3.91	Phenol, 3-pentadecyl-
68	19.701	2091469	0.76	660131	0.56	3.17	Docosanoic acid, ethyl ester
69	21.534	876555	0.32	234203	0.20	3.74	Docosanoic acid, ethyl ester
70	21.585	1078108	0.39	192993	0.16	5.59	3-Octen-2-one, 4-(methylamino)-
71	21.687	1249949	0.45	235327	0.20	5.31	1,2-Octadecanediol
72	21.909	511870	0.19	119833	0.10	4.27	rotundene
73	23.960	12064807	4.39	1522865	1.29	7.92	Murolan-3,9(11)-diene-10-peroxy
74	23.970	14768447	5.37	1554428	1.32	9.50	Cyclopropa[5,6]-33-norgorgostan-3-ol, 3',6-dih
75	24.291	10107795	3.68	1798968	1.52	5.62	Squalene
76	27.120	1385851	0.50	212933	0.18	6.51	Tetradecane, 5-methyl-
77	27.286	4148224	1.51	352168	0.30	11.78	1-Hexadecanol
78	28.910	343148	0.12	106478	0.09	3.22	Cholestan-3-ol, 2-methylene-, (3.beta.,5.alpha.)
79	28.990	645727	0.23	200698	0.17	3.22	1-Acetyl-2-methyl-6-(prop-2-enyl)piperidine (N
80	29.000	257309	0.09	239042	0.20	1.08	Lupeol
81	29.075	1222491	0.44	265698	0.23	4.60	4,4,6a,6b,8a,11,11,14b-Octamethyl-1,4,4a,5,6,6
82	29.115	1620109	0.59	280885	0.24	5.77	A-Norcholestane-3-carboxylic acid, 2-oxo-, me
83	30.650	1067447	0.39	188660	0.16	5.66	C(14a)-Homo-27-norgammacer-13-en-21-one, 3
84	30.665	163768	0.06	199931	0.17	0.82	C(14a)-Homo-27-norgammacer-13-en-21-one, 3
85	30.774	1335710	0.49	190468	0.16	7.01	Dimethylmalonic acid, 4-chlorophenyl octadecy
86	30.996	8066209	2.94	670814	0.57	12.02	1-Dodecanol, 2-octyl-
87	33.170	1141019	0.42	286368	0.24	3.98	Adenosine, 2',3'-O-di-tert-butyldimethylsilyl-5',
88	33.236	2098862	0.76	341422	0.29	6.15	Tridecanenitrile
89	33.400	2107426	0.77	383075	0.32	5.50	6a,14a-Methanopicene, perhydro-1,2,4a,6b,9,9,
90	33.550	4271085	1.55	653803	0.55	6.53	2-Bicyclo[3.3.0]octene-4-carbanilide
91	33.566	769165	0.28	648661	0.55	1.19	4,7,7-Trimethylbicyclo[2.2.1]heptan-2-one O-a
92	33.580	1330922	0.48	660047	0.56	2.02	2H-2,4a-Ethanonaphthalene, 1,3,4,5,6,7-hexah
93	33.625	5681581	2.07	630738	0.53	9.01	17,18-Dinor-8.beta.H,9.beta.H-labda-13(16),14
		274820140	100.00	118036376	100.00		

Qualitative Analysis Report

Library

<< Target >>
Line#:1 R.Time:4.085(Scan#:18) MassPeaks:368
RawMode:Averaged 4.080-4.090(17-19) BasePeak:41.05(68676)
BG Mode:Calc. from Peak Group 1 - Event 1 Scan

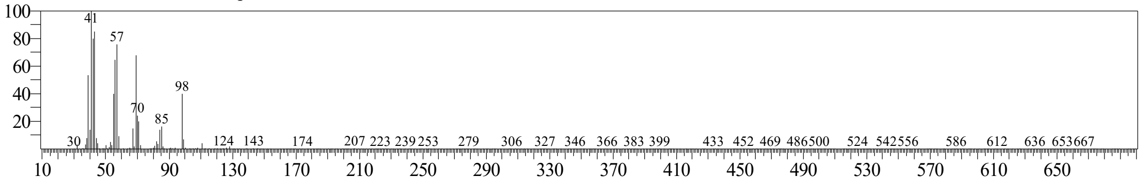

Hit#:1 Entry:8012 Library:NIST11.lib
SI:86 Formula:C8H18O CAS:1653-40-3 MolWeight:130 RetIndex:995
CompName:1-Heptanol, 6-methyl- $$ 6-Methyl-1-heptanol $$

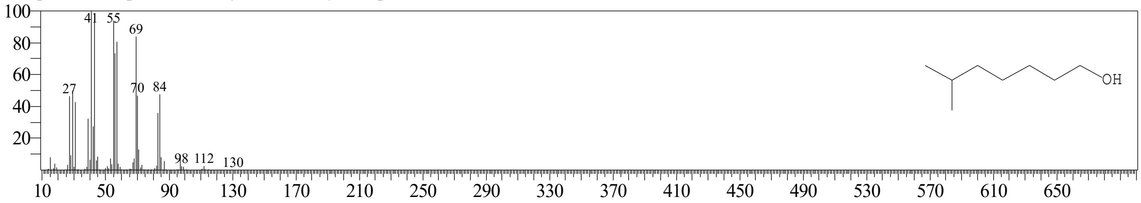

Hit#:2 Entry:24882 Library:NIST11.lib
SI:85 Formula:C12H24 CAS:74630-49-2 MolWeight:168 RetIndex:1158
CompName:3-Undecene, 7-methyl-, (Z)- $$ (3Z)-7-Methyl-3-undecene # $$

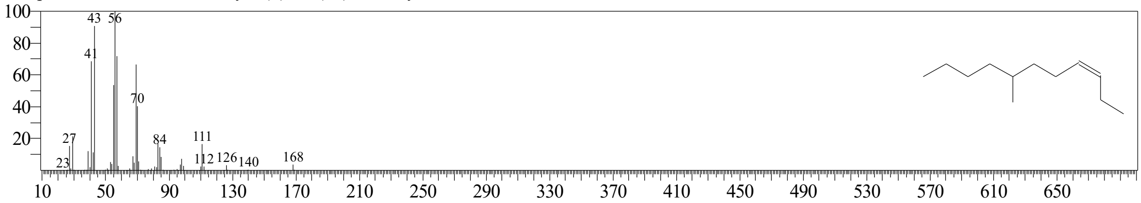

Hit#:3 Entry:17712 Library:NIST11.lib
SI:84 Formula:C11H22 CAS:61142-78-7 MolWeight:154 RetIndex:1041
CompName:1-Decene, 9-methyl- $$ 9-Methyl-1-decene # $$

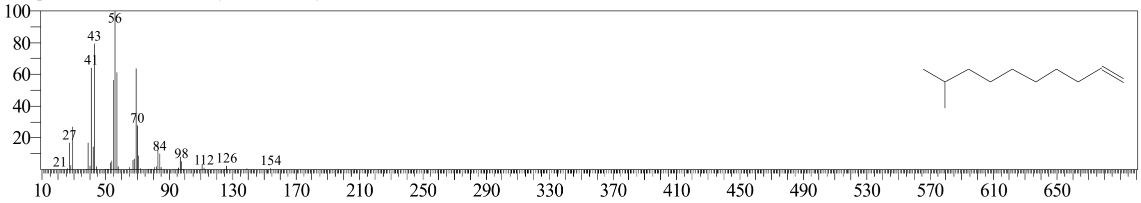

Hit#:4 Entry:17685 Library:NIST11.lib
SI:84 Formula:C11H22 CAS:821-96-5 MolWeight:154 RetIndex:1123
CompName:2-Undecene, (Z)- $$ 2-Undecene, cis- $$ (Z)-2-Undecene $$ cis-2-Undecene $$ (2Z)-2-Undecene # $$

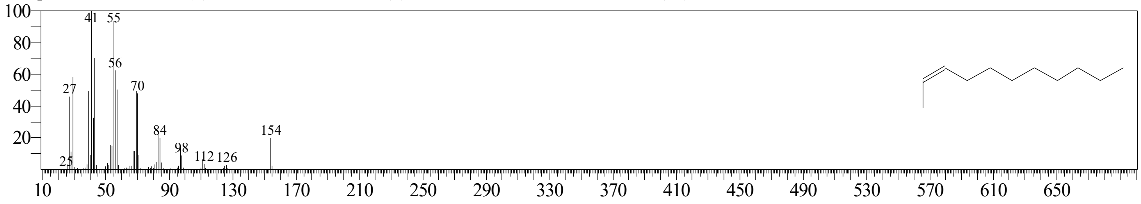

Hit#:5 Entry:4119 Library:NIST11.lib
SI:84 Formula:C7H14O CAS:7045-79-6 MolWeight:114 RetIndex:790
CompName:Oxetane, 2-methyl-4-propyl- $$ 2-Methyl-4-propyloxetane # $$

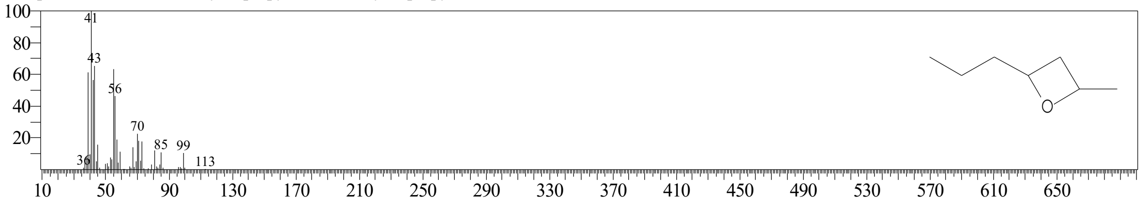

<< Target >>
Line#:2 R.Time:4.130(Scan#:27) MassPeaks:346
RawMode:Averaged 4.125-4.135(26-28) BasePeak:43.05(203915)
BG Mode:Calc. from Peak Group 1 - Event 1 Scan

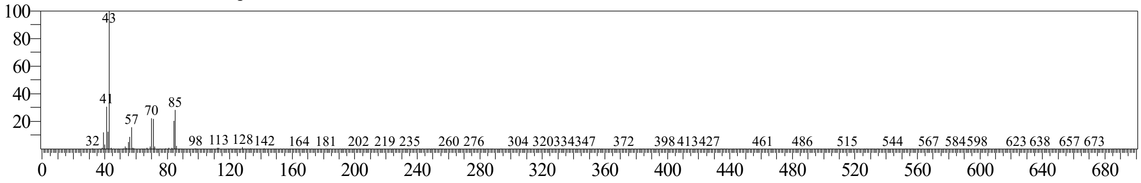

Hit#:1 Entry:7426 Library:NIST11.lib
SI:95 Formula:C9H20 CAS:2216-34-4 MolWeight:128 RetIndex:852
CompName:Octane, 4-methyl- $$ 4-Methyloctane $$ 1,3-Dimethylheptane $$

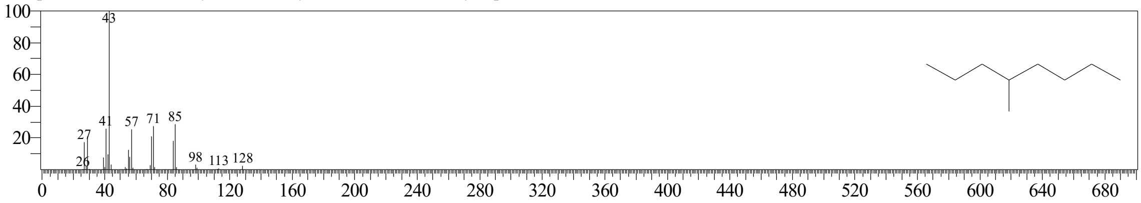

Hit#:2 Entry:7413 Library:NIST11.lib
SI:94 Formula:C9H20 CAS:921-47-1 MolWeight:128 RetIndex:724
CompName:Hexane, 2,3,4-trimethyl- $$ 2,3,4-Trimethylhexane $$

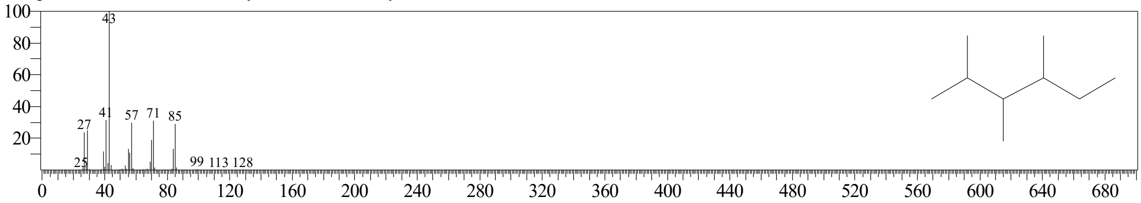

Hit#:3 Entry:4245 Library:NIST11.lib
SI:93 Formula:C8H18 CAS:619-99-8 MolWeight:114 RetIndex:752
CompName:Hexane, 3-ethyl- $$ 3-Ethylhexane $$

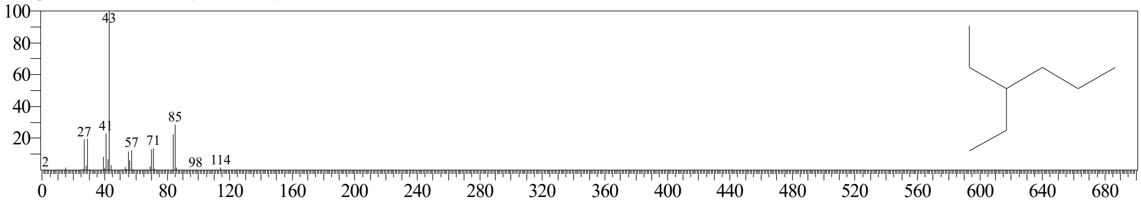

Hit#:4 Entry:7422 Library:NIST11.lib
SI:92 Formula:C9H20 CAS:1069-53-0 MolWeight:128 RetIndex:724
CompName:Hexane, 2,3,5-trimethyl- $$ 2,3,5-Trimethylhexane $$

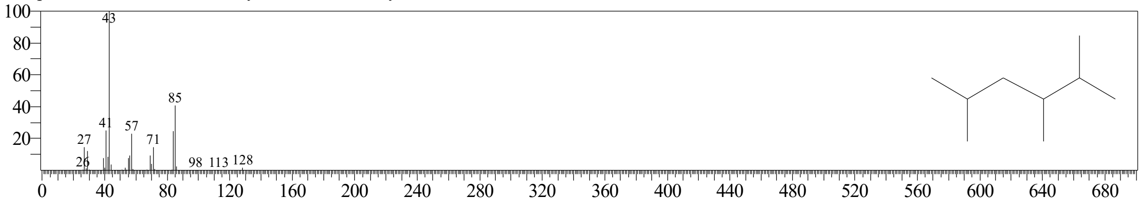

Hit#:5 Entry:7424 Library:NIST11.lib
SI:92 Formula:C9H20 CAS:2213-23-2 MolWeight:128 RetIndex:788
CompName:Heptane, 2,4-dimethyl- $$ 2,4-Dimethylheptane $$

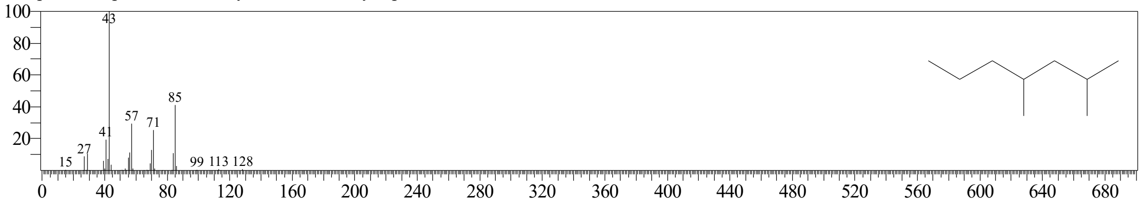

<< Target >>
Line#:3 R.Time:4.225(Scan#:46) MassPeaks:341
RawMode:Averaged 4.220-4.230(45-47) BasePeak:57.10(178393)
BG Mode:Calc. from Peak Group 1 - Event 1 Scan

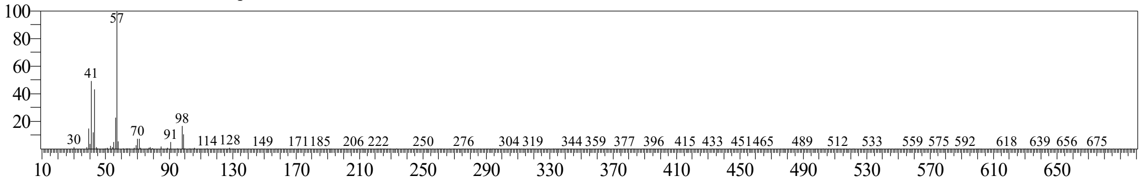

Hit#:1 Entry:12322 Library:NIST11.lib
SI:91 Formula:C10H22 CAS:52896-87-4 MolWeight:142 RetIndex:887
CompName:Heptane, 4-(1-methylethyl)- $$ 4-Isopropylheptane $$

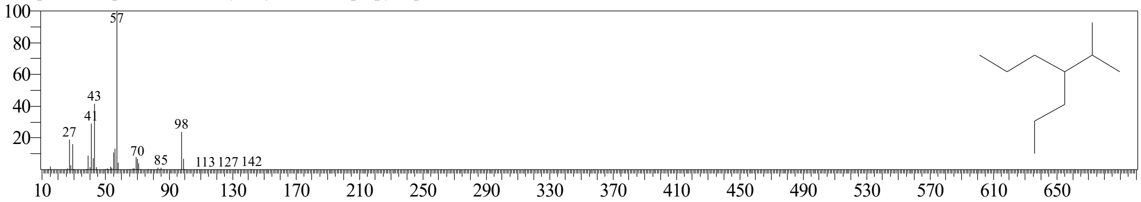

Hit#:2 Entry:29894 Library:NIST11.lib
SI:91 Formula:C7H15Br CAS:1974-05-6 MolWeight:178 RetIndex:949
CompName:Heptane, 3-bromo- $$ 3-Bromoheptane $$

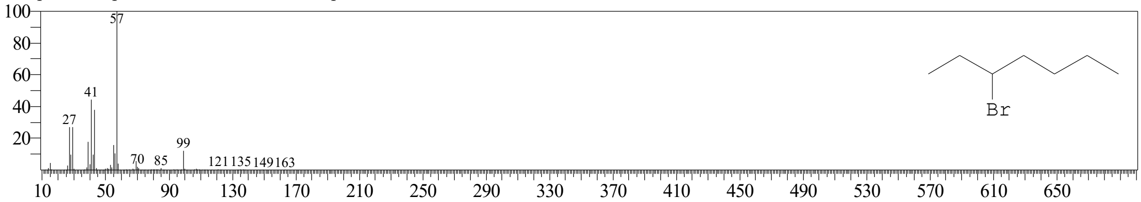

Hit#:3 Entry:7433 Library:NIST11.lib
SI:90 Formula:C9H20 CAS:2216-30-0 MolWeight:128 RetIndex:788
CompName:Heptane, 2,5-dimethyl- $$ 2,5-Dimethylheptane $$

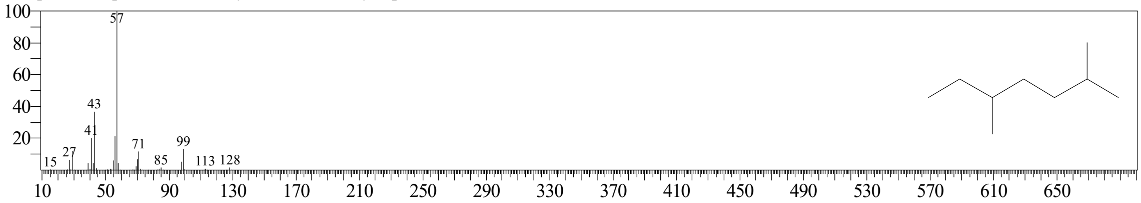

Hit#:4 Entry:7434 Library:NIST11.lib
SI:90 Formula:C9H20 CAS:2216-33-3 MolWeight:128 RetIndex:852
CompName:Octane, 3-methyl- $$ 3-Methyloctane $$ Isononane $$

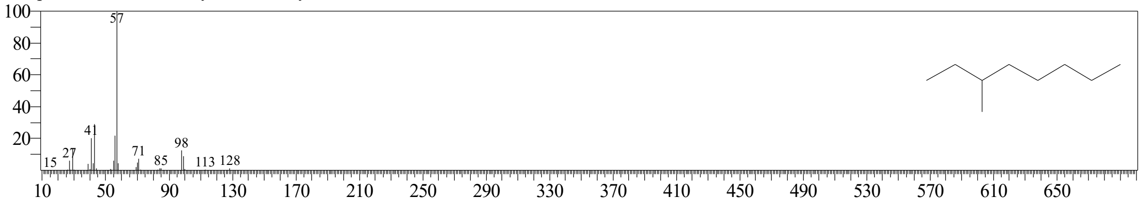

Hit#:5 Entry:7431 Library:NIST11.lib
SI:90 Formula:C9H20 CAS:3074-75-7 MolWeight:128 RetIndex:788
CompName:Hexane, 4-ethyl-2-methyl- $$ 4-Ethyl-2-methylhexane $$ 2-Methyl-4-ethylhexane $$ Hexane, 3-ethyl-5-methyl $$

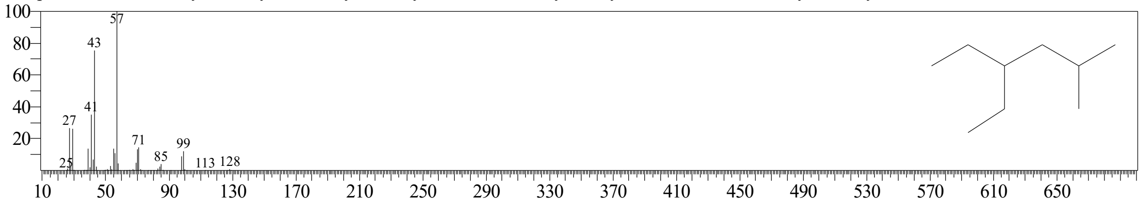

<< Target >>
Line#:4 R.Time:4.315(Scan#:64) MassPeaks:351
RawMode:Averaged 4.310-4.320(63-65) BasePeak:91.05(106067)
BG Mode:Calc. from Peak Group 1 - Event 1 Scan

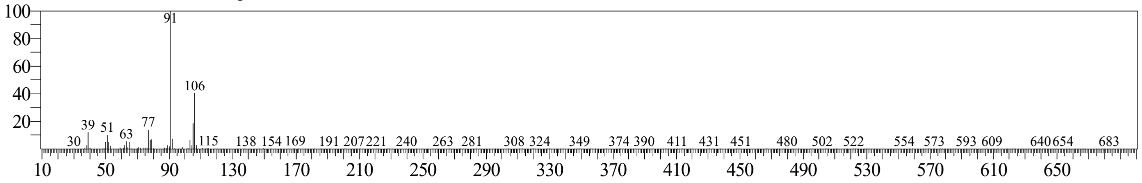

Hit#:1 Entry:2687 Library:NIST11.lib
SI:95 Formula:C8H10 CAS:95-47-6 MolWeight:106 RetIndex:907
CompName:o-Xylene $$ Benzene, 1,2-dimethyl- $$ o-Dimethylbenzene $$ o-Methyltoluene $$ o-Xylol $$ 1,2-Dimethylbenzene $$ 1,2-Xylene $$ 3,4-Xylene $

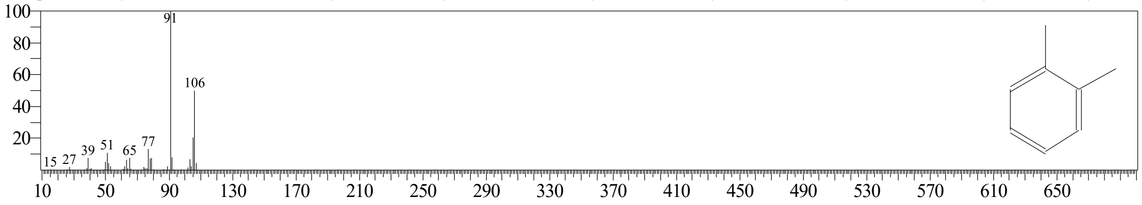

Hit#:2 Entry:2685 Library:NIST11.lib
SI:95 Formula:C8H10 CAS:108-38-3 MolWeight:106 RetIndex:907
CompName:Benzene, 1,3-dimethyl- $$ m-Xylene $$ m-Dimethylbenzene $$ m-Xylol $$ 1,3-Dimethylbenzene $$ 1,3-Xylene $$ 2,4-Xylene $$ m-Methyltoluene

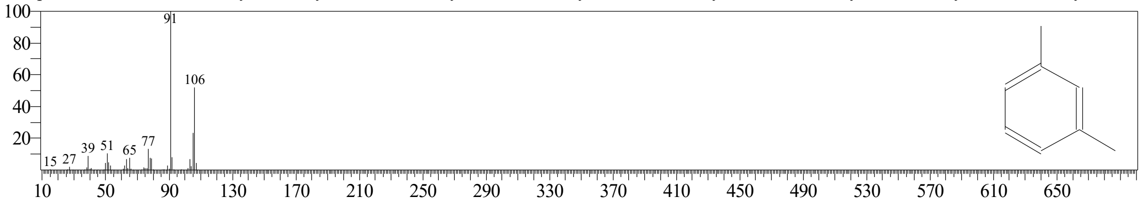

Hit#:3 Entry:2686 Library:NIST11.lib
SI:93 Formula:C8H10 CAS:106-42-3 MolWeight:106 RetIndex:907
CompName:p-Xylene $$ Benzene, 1,4-dimethyl- $$ p-Dimethylbenzene $$ p-Xylol $$ 1,4-Dimethylbenzene $$ 1,4-Xylene $$ p-Methyltoluene $$ para-Xylene

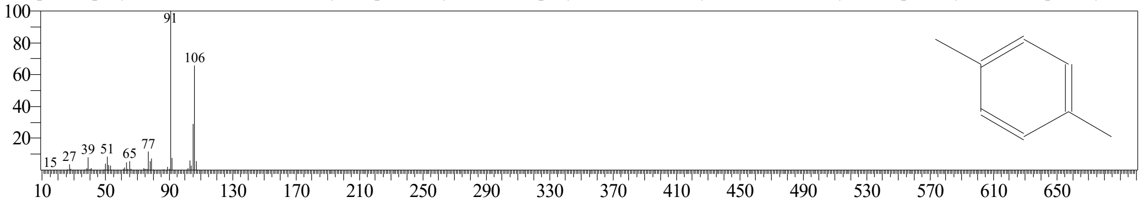

Hit#:4 Entry:2683 Library:NIST11.lib
SI:92 Formula:C8H10 CAS:100-41-4 MolWeight:106 RetIndex:893
CompName:Ethylbenzene $$ Benzene, ethyl- $$ Ethylbenzol $$ EB $$ Phenylethane $$ Aethylbenzol $$ Ethylbenzeen $$ Etilbenzene $$ Etylobenzen $$ NCI-C

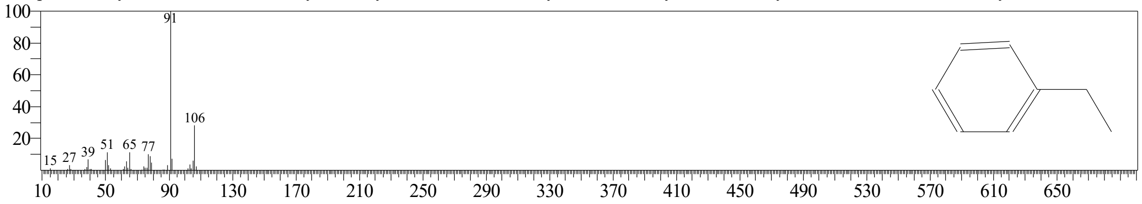

Hit#:5 Entry:9917 Library:NIST11.lib
SI:88 Formula:C7H7NO2 CAS:622-42-4 MolWeight:137 RetIndex:1175
CompName:Benzene, (nitromethyl)- $$ Toluene, .alpha.-nitro- $$ .alpha.-Nitrotoluene $$ (Nitromethyl)benzene $$ Nitrophenylmethane $$ Phenylnitromethane

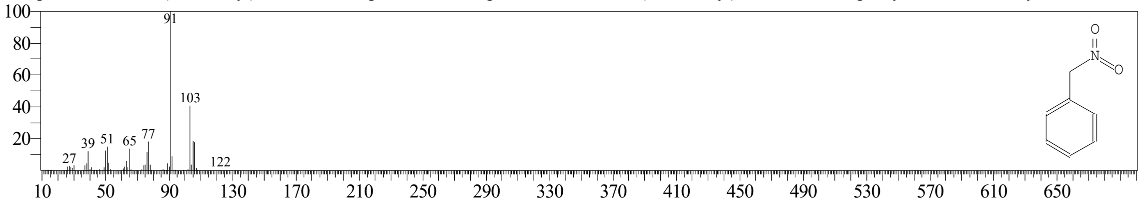

<< Target >>
Line#:5 R.Time:4.545(Scan#:110) MassPeaks:407
RawMode:Averaged 4.540-4.550(109-111) BasePeak:43.10(511521)
BG Mode:Calc. from Peak Group 1 - Event 1 Scan

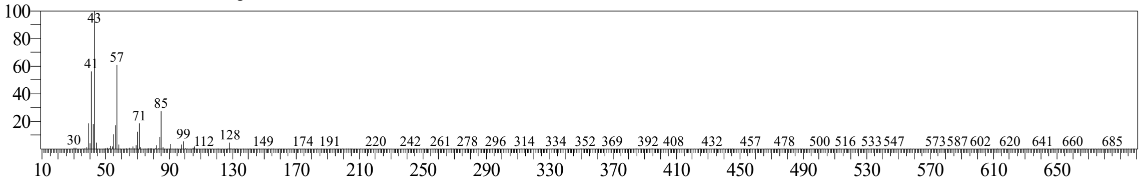

Hit#:1 Entry:7416 Library:NIST11.lib
SI:93 Formula:C9H20 CAS:111-84-2 MolWeight:128 RetIndex:916
CompName:Nonane $$ n-Nonane $$ Shellsol 140 $$ n-C9H20 $$ UN 1920 $$

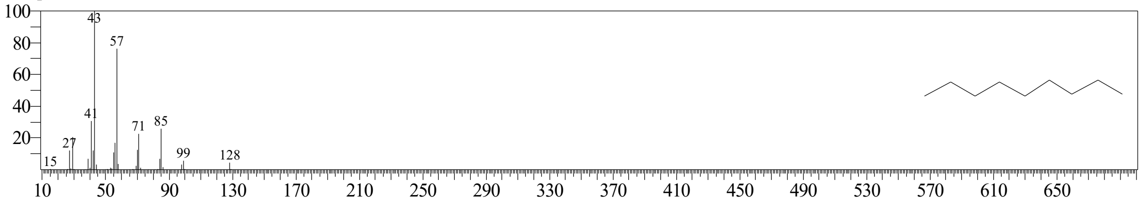

Hit#:2 Entry:4236 Library:NIST11.lib
SI:92 Formula:C8H18 CAS:589-43-5 MolWeight:114 RetIndex:688
CompName:Hexane, 2,4-dimethyl- $$ 2,4-Dimethylhexane $$

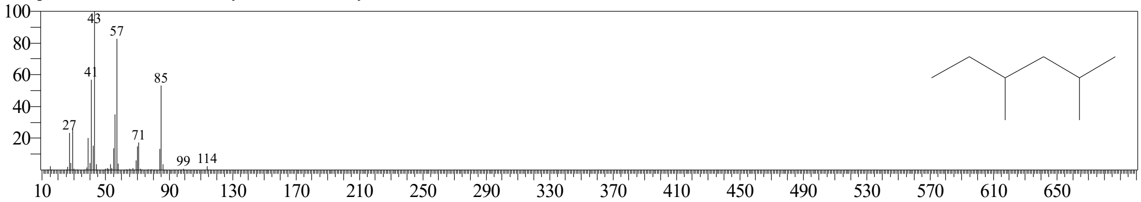

Hit#:3 Entry:4235 Library:NIST11.lib
SI:91 Formula:C8H18 CAS:111-65-9 MolWeight:114 RetIndex:816
CompName:Octane $$ n-Octane $$ n-C8H18 $$ Oktan $$ Oktanen $$ Ottani $$ UN 1262 $$

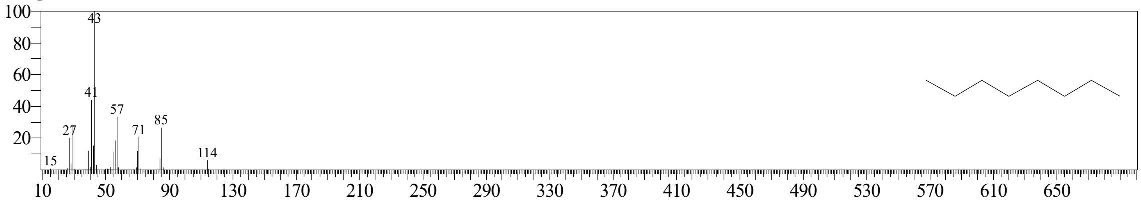

Hit#:4 Entry:12306 Library:NIST11.lib
SI:90 Formula:C10H22 CAS:871-83-0 MolWeight:142 RetIndex:951
CompName:Nonane, 2-methyl- $$ 2-Methylnonane $$

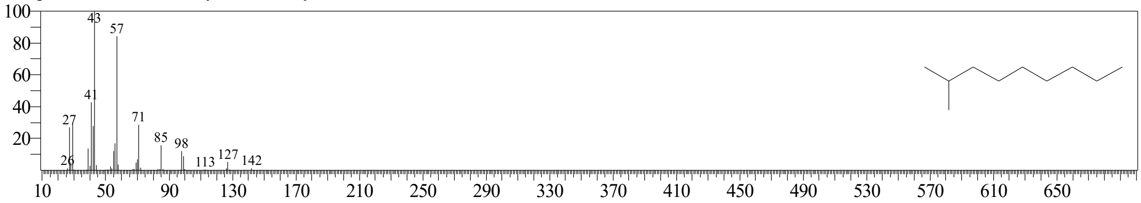

Hit#:5 Entry:12315 Library:NIST11.lib
SI:90 Formula:C10H22 CAS:2613-61-8 MolWeight:142 RetIndex:823
CompName:Heptane, 2,4,6-trimethyl- $$ 2,4,6-Trimethylheptane $$

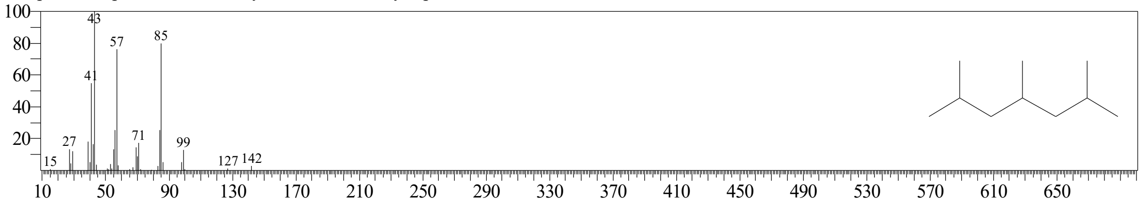

<< Target >>
Line#:6 R.Time:4.740(Scan#:149) MassPeaks:356
RawMode:Averaged 4.735-4.745(148-150) BasePeak:43.05(678694)
BG Mode:Calc. from Peak Group 1 - Event 1 Scan

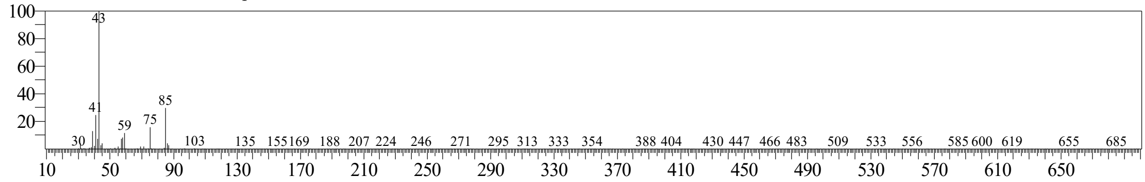

Hit#:1 Entry:7299 Library:NIST11.lib
SI:87 Formula:C8H16O CAS:26118-38-7 MolWeight:128 RetIndex:868
CompName:2-Hexanone, 3,3-dimethyl- $$ 3,3-Dimethyl-2-hexanone # $$

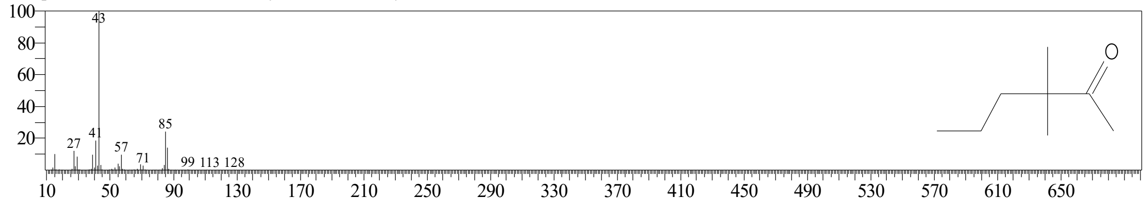

Hit#:2 Entry:8164 Library:NIST11.lib
SI:87 Formula:C6H13NO2 CAS:14255-44-8 MolWeight:131 RetIndex:935
CompName:Hexane, 2-nitro- $$ 2-Nitrohexane # $$

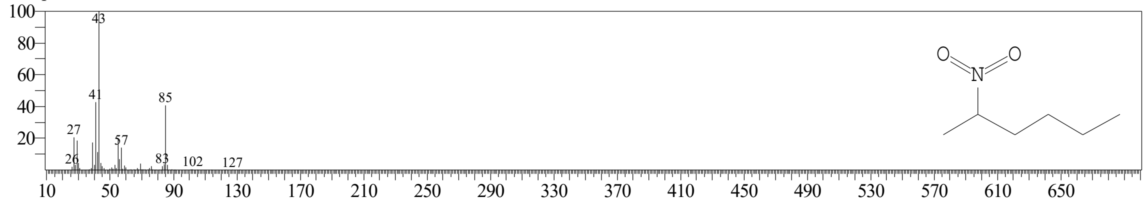

Hit#:3 Entry:51590 Library:NIST11.lib
SI:86 Formula:C5H9IO CAS:3695-29-2 MolWeight:212 RetIndex:1069
CompName:5-Iodopentan-2-one $$ 5-Iodo-2-pentanone # $$

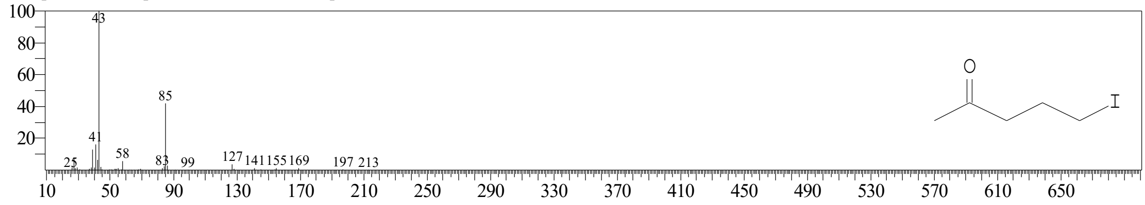

Hit#:4 Entry:4540 Library:NIST11.lib
SI:86 Formula:C6H12O2 CAS:25910-96-7 MolWeight:116 RetIndex:815
CompName:3-Oxetanol, 2,2,3-trimethyl- $$ 2,2,3-Trimethyl-3-oxetanol # $$

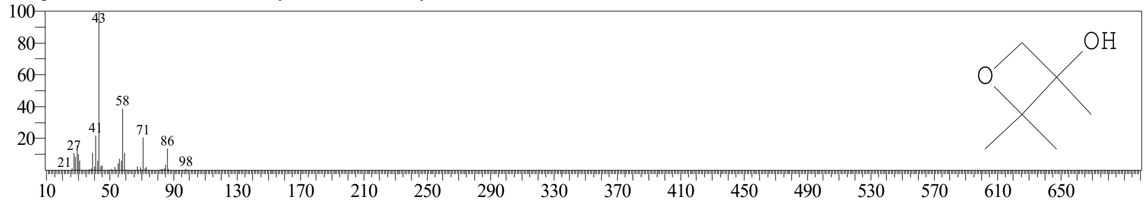

Hit#:5 Entry:11925 Library:NIST11.lib
SI:86 Formula:C7H10O3 CAS:1118-84-9 MolWeight:142 RetIndex:1010
CompName:Butanoic acid, 3-oxo-, 2-propenyl ester $$ Acetoacetic acid, allyl ester $$ Allyl acetoacetate $$ Allyl acetylacetate $$ Allyl 3-oxobutanoate # $$

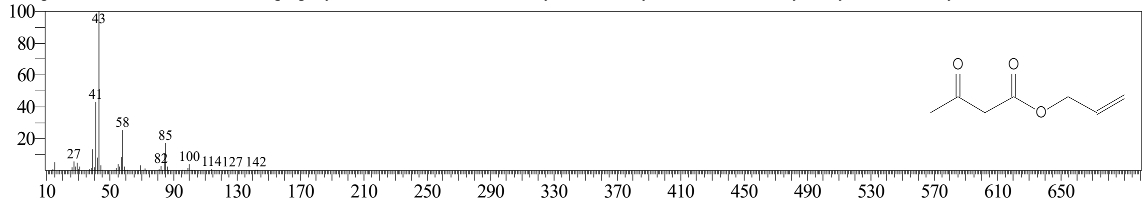

<< Target >>
Line#:7 R.Time:4.935(Scan#:188) MassPeaks:342
RawMode:Averaged 4.930-4.940(187-189) BasePeak:43.05(692781)
BG Mode:Calc. from Peak Group 1 - Event 1 Scan

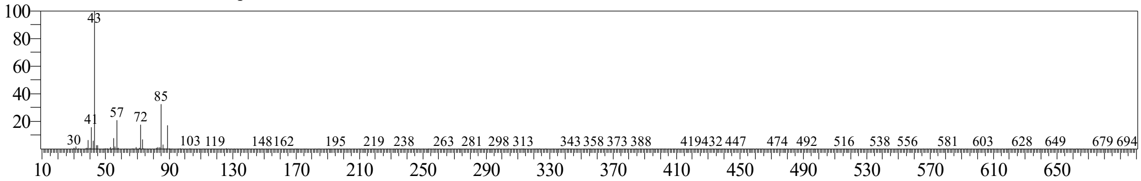

Hit#:1 Entry:3988 Library:NIST11.lib
SI:87 Formula:C6H10O2 CAS:17257-81-7 MolWeight:114 RetIndex:806
CompName:Ethanone, 1-(3-ethyloxiranyl)- $$ 2-Hexanone, 3,4-epoxy- $$ 1-(3-Ethyl-2-oxiranyl)ethanone # $$

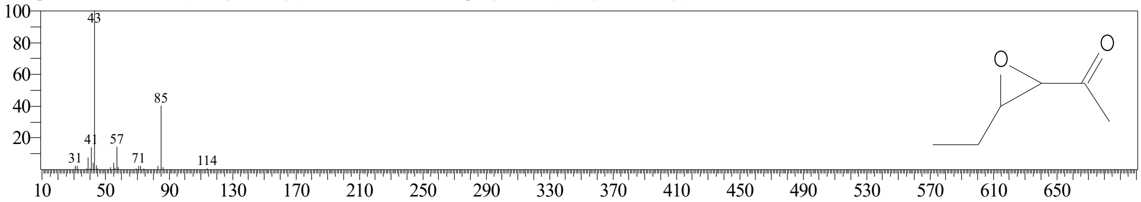

Hit#:2 Entry:7295 Library:NIST11.lib
SI:86 Formula:C8H16O CAS:2371-19-9 MolWeight:128 RetIndex:888
CompName:2-Heptanone, 3-methyl- $$ 3-Methyl-2-heptanone $$ 3-Methylheptan-2-one $$

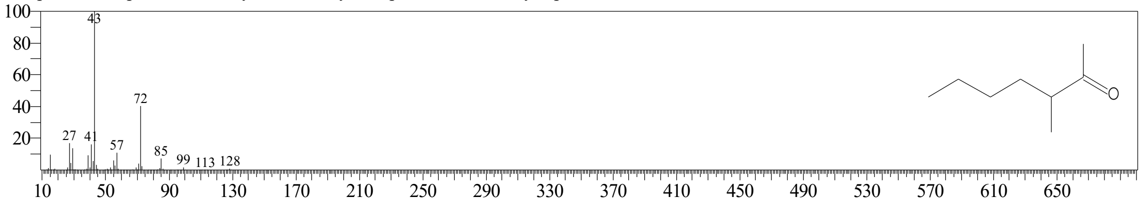

Hit#:3 Entry:18593 Library:NIST11.lib
SI:86 Formula:C10H20O CAS:107995-55-1 MolWeight:156 RetIndex:1081
CompName:Hexane, 1-(3-butenyloxy)- $$ 3-Butenyl hexyl ether # $$

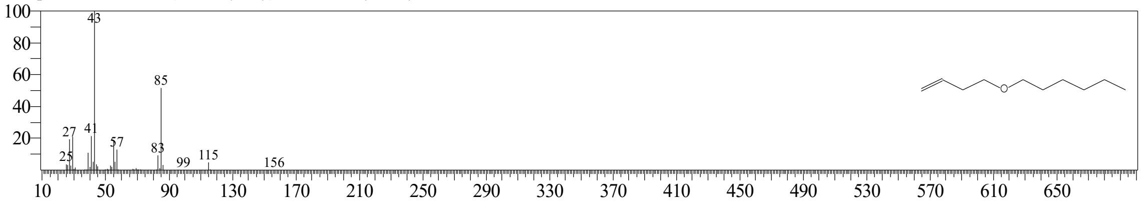

Hit#:4 Entry:12929 Library:NIST11.lib
SI:86 Formula:C8H16O2 CAS:30536-44-8 MolWeight:144 RetIndex:1028
CompName:4-Butoxy-2-butanone

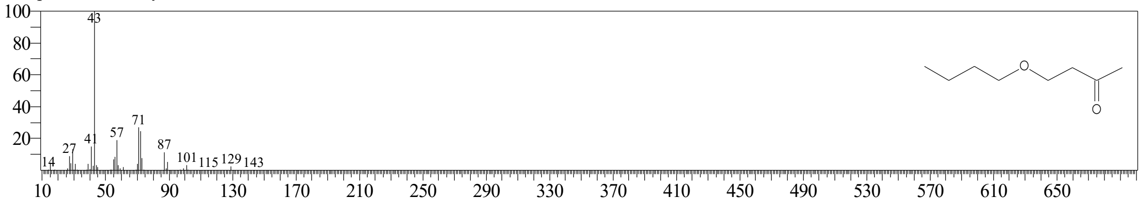

Hit#:5 Entry:7299 Library:NIST11.lib
SI:86 Formula:C8H16O CAS:26118-38-7 MolWeight:128 RetIndex:868
CompName:2-Hexanone, 3,3-dimethyl- $$ 3,3-Dimethyl-2-hexanone # $$

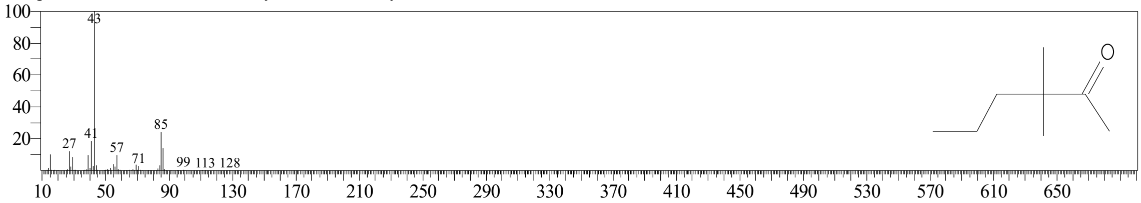

<< Target >>
Line#:8 R.Time:5.025(Scan#:206) MassPeaks:361
RawMode:Averaged 5.020-5.030(205-207) BasePeak:43.10(19738)
BG Mode:Calc. from Peak Group 1 - Event 1 Scan

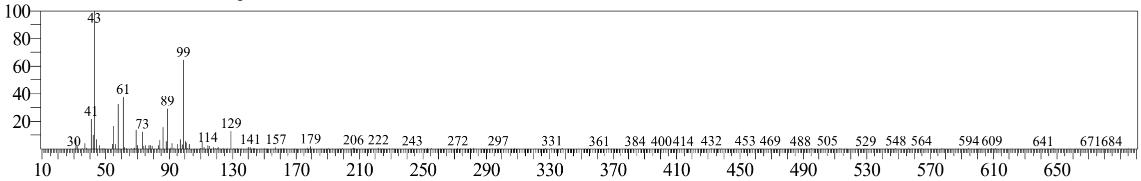

Hit#:1 Entry:26658 Library:NIST11.lib
SI:72 Formula:C7H13BO4 CAS:58163-59-0 MolWeight:172 RetIndex:0
CompName:1,3,2-Dioxaborolane-4-methanol, 2-ethyl-, acetate $$ (2-Ethyl-1,3,2-dioxaborolan-4-yl)methyl acetate # $$

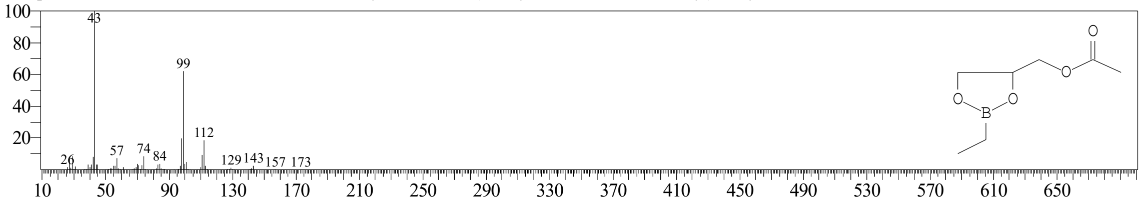

Hit#:2 Entry:12916 Library:NIST11.lib
SI:70 Formula:C8H16O2 CAS:142-92-7 MolWeight:144 RetIndex:984
CompName:Acetic acid, hexyl ester $$ n-Hexyl acetate $$ Hexyl acetate $$ Hexyl ethanoate $$ 1-Hexyl acetate $$ Acetic acid n-hexyl ester $$ n-Hexyl ethanoat

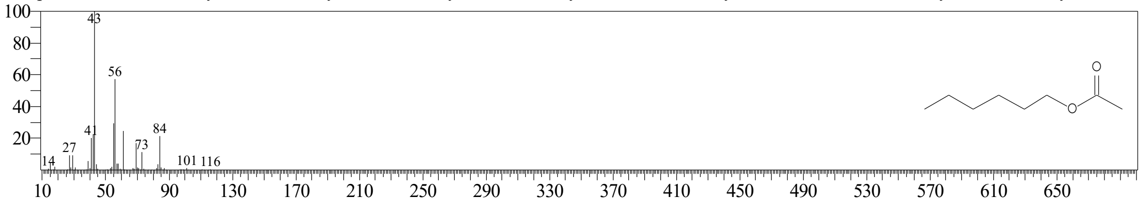

Hit#:3 Entry:12830 Library:NIST11.lib
SI:70 Formula:C7H12O3 CAS:539-88-8 MolWeight:144 RetIndex:1020
CompName:Pentanoic acid, 4-oxo-, ethyl ester $$ Levulinic acid, ethyl ester $$ Ethyl laevulinate $$ Ethyl levulate $$ Ethyl levulinate $$ Ethyl 3-acetylpropiona

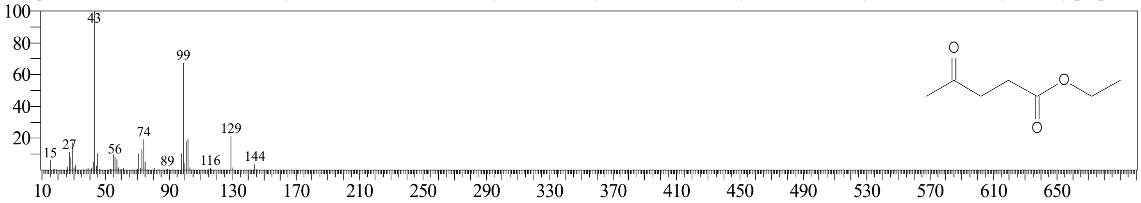

Hit#:4 Entry:12920 Library:NIST11.lib
SI:70 Formula:C8H16O2 CAS:25368-54-1 MolWeight:144 RetIndex:1195
CompName:8-Hydroxy-2-octanone

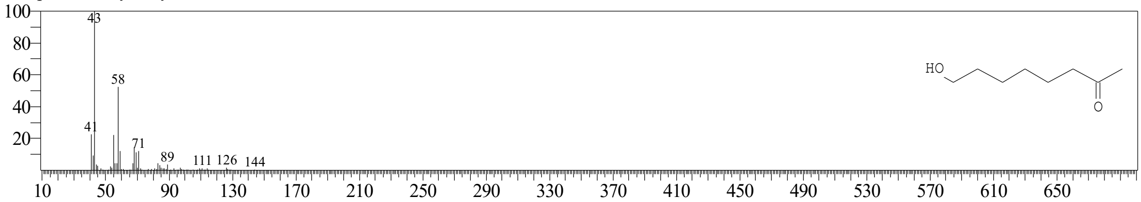

Hit#:5 Entry:12816 Library:NIST11.lib
SI:69 Formula:C7H12O3 CAS:5185-97-7 MolWeight:144 RetIndex:1020
CompName:2-Pentanone, 5-(acetyloxy)- $$ 2-Pentanone, 5-hydroxy-, acetate $$ .gamma.-Acetylpropyl acetate $$ Acetopropyl acetate $$ 3-Acetylpropyl acetate

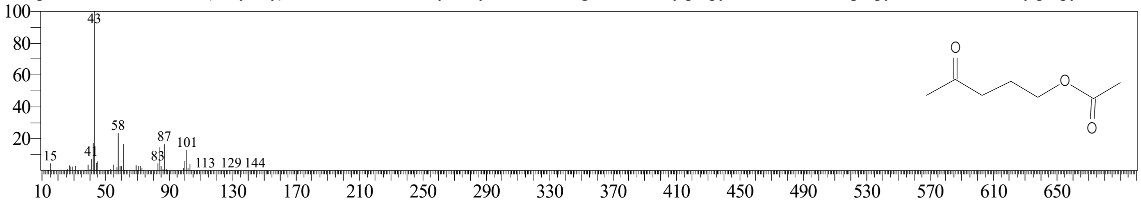

<< Target >>
Line#:9 R.Time:5.220(Scan#:245) MassPeaks:385
RawMode:Averaged 5.215-5.225(244-246) BasePeak:43.05(13923)
BG Mode:Calc. from Peak Group 1 - Event 1 Scan

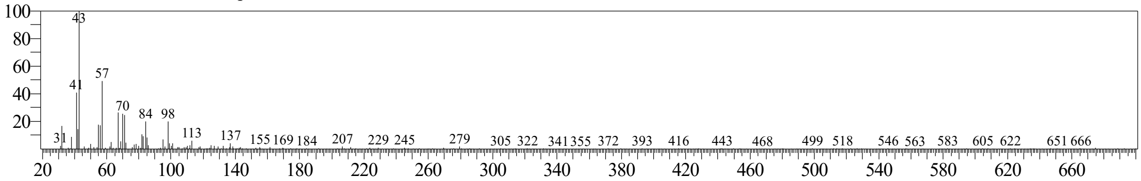

Hit#:1 Entry:150228 Library:NIST11.lib
SI:78 Formula:C20H38O4 CAS:0-00-0 MolWeight:342 RetIndex:2344
CompName:Oxalic acid, dodecyl hexyl ester

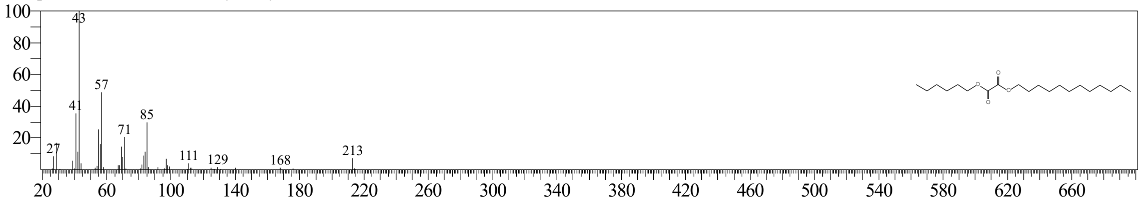

Hit#:2 Entry:167834 Library:NIST11.lib
SI:78 Formula:C22H42O4 CAS:0-00-0 MolWeight:370 RetIndex:2543
CompName:Oxalic acid, hexyl tetradecyl ester

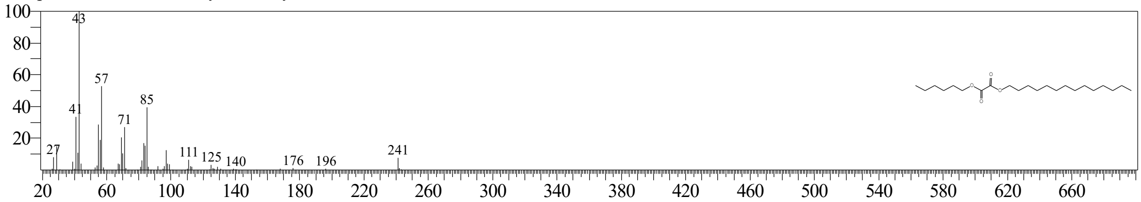

Hit#:3 Entry:26100 Library:NIST11.lib
SI:78 Formula:C12H26 CAS:1636-43-7 MolWeight:170 RetIndex:1086
CompName:Decane, 5,6-dimethyl- $$ 5,6-Dimethyldecane $$

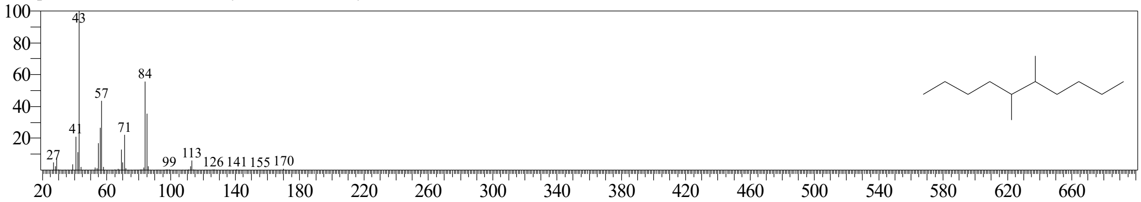

Hit#:4 Entry:34135 Library:NIST11.lib
SI:78 Formula:C13H28 CAS:17312-83-3 MolWeight:184 RetIndex:1185
CompName:Undecane, 5,7-dimethyl- $$ 5,7-Dimethylundecane # $$

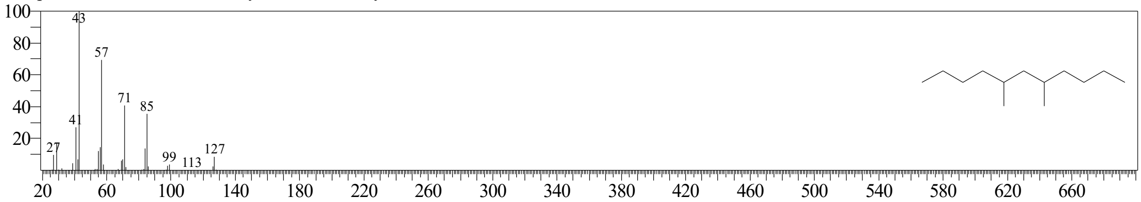

Hit#:5 Entry:34131 Library:NIST11.lib
SI:78 Formula:C13H28 CAS:17301-25-6 MolWeight:184 RetIndex:1185
CompName:Undecane, 2,8-dimethyl- $$ 2,8-Dimethylundecane # $$

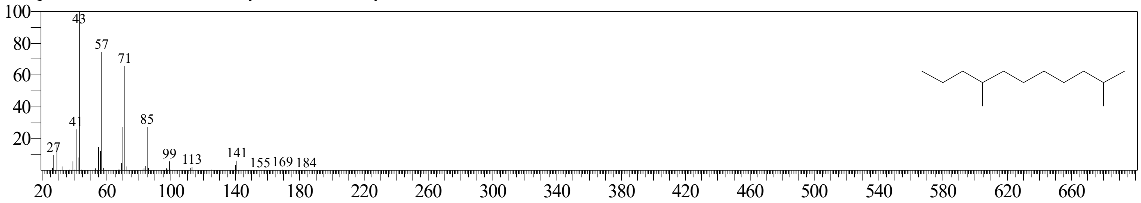

<< Target >>
Line#:10 R.Time:5.265(Scan#:254) MassPeaks:374
RawMode:Averaged 5.260-5.270(253-255) BasePeak:43.05(24325)
BG Mode:Calc. from Peak Group 1 - Event 1 Scan

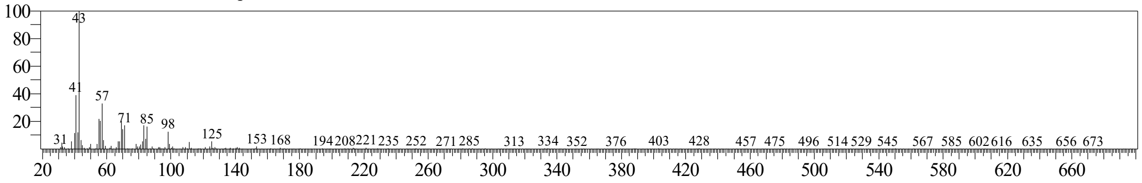

Hit#:1 Entry:150228 Library:NIST11.lib
SI:84 Formula:C20H38O4 CAS:0-00-0 MolWeight:342 RetIndex:2344
CompName:Oxalic acid, dodecyl hexyl ester

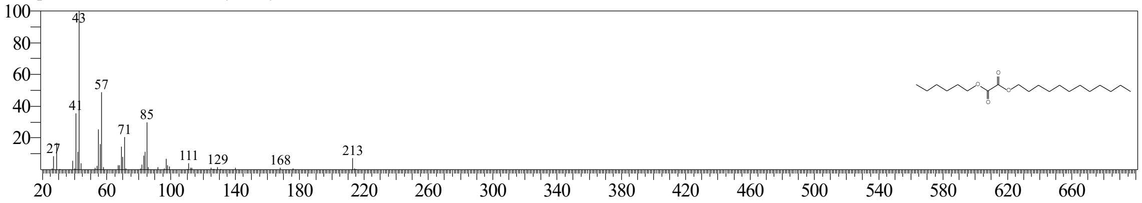

Hit#:2 Entry:181537 Library:NIST11.lib
SI:83 Formula:C24H46O4 CAS:0-00-0 MolWeight:398 RetIndex:2741
CompName:Oxalic acid, hexadecyl hexyl ester

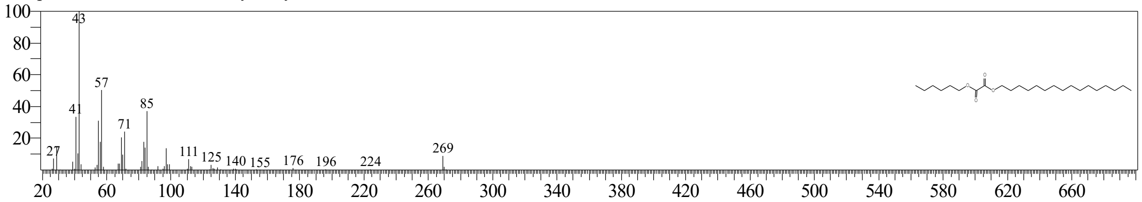

Hit#:3 Entry:96490 Library:NIST11.lib
SI:83 Formula:C15H28O4 CAS:0-00-0 MolWeight:272 RetIndex:1847
CompName:Oxalic acid, decyl propyl ester

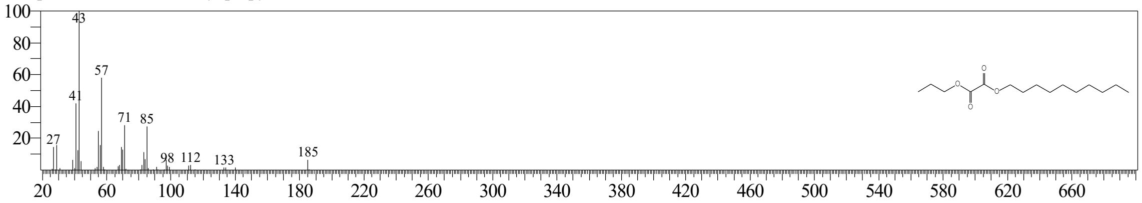

Hit#:4 Entry:107435 Library:NIST11.lib
SI:83 Formula:C16H30O4 CAS:0-00-0 MolWeight:286 RetIndex:1946
CompName:Oxalic acid, propyl undecyl ester

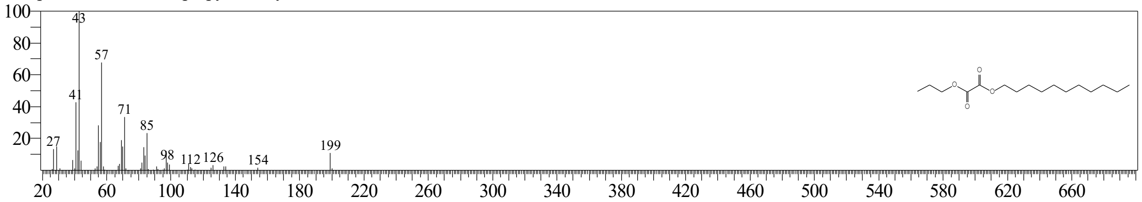

Hit#:5 Entry:167834 Library:NIST11.lib
SI:83 Formula:C22H42O4 CAS:0-00-0 MolWeight:370 RetIndex:2543
CompName:Oxalic acid, hexyl tetradecyl ester

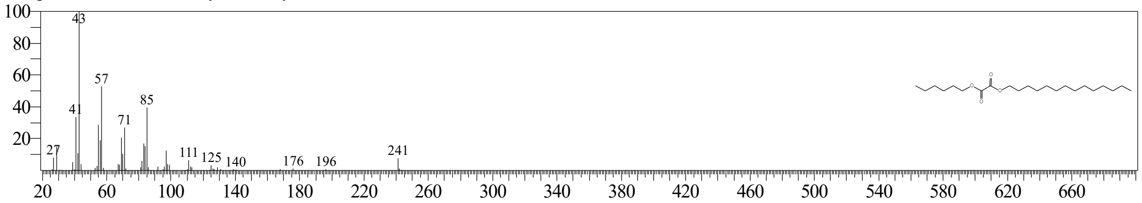

Qualitative Analysis Report

<< Target >>
Line#:11 R.Time:5.335(Scan#:268) MassPeaks:371
RawMode:Averaged 5.330-5.340(267-269) BasePeak:43.05(13893)
BG Mode:Calc. from Peak Group 1 - Event 1 Scan

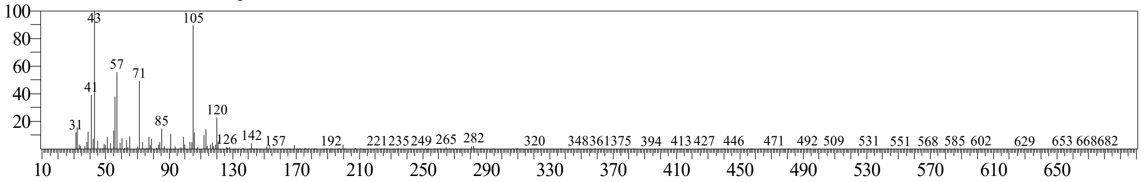

Hit#:1 Entry:20162 Library:NIST11.lib
SI:71 Formula:C8H16O3 CAS:0-00-0 MolWeight:160 RetIndex:931
CompName:Isobutyl isopropyl carbonate

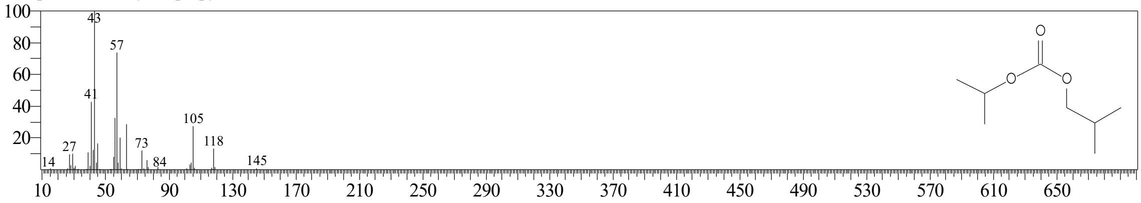

Hit#:2 Entry:12310 Library:NIST11.lib
SI:71 Formula:C10H22 CAS:13475-78-0 MolWeight:142 RetIndex:887
CompName:Heptane, 5-ethyl-2-methyl- $$ 5-Ethyl-2-methylheptane $$ Heptane, 3-ethyl-6-methyl $$

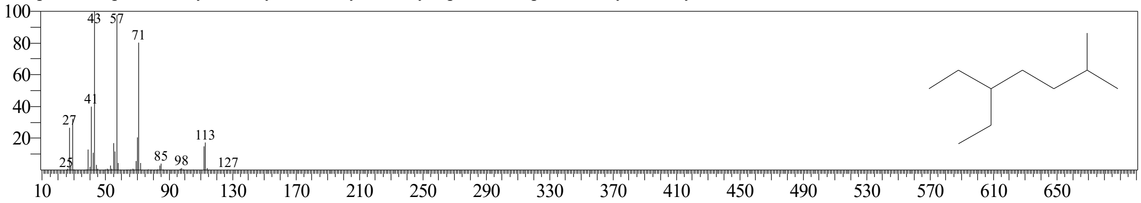

Hit#:3 Entry:18707 Library:NIST11.lib
SI:69 Formula:C11H24 CAS:62016-30-2 MolWeight:156 RetIndex:966
CompName:Octane, 2,3,3-trimethyl- $$ 2,3,3-Trimethyloctane # $$

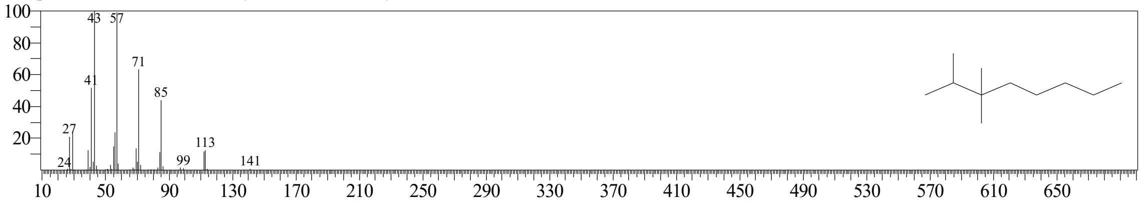

Hit#:4 Entry:54744 Library:NIST11.lib
SI:69 Formula:C12H24O3 CAS:0-00-0 MolWeight:216 RetIndex:1457
CompName:Carbonic acid, octyl propyl ester

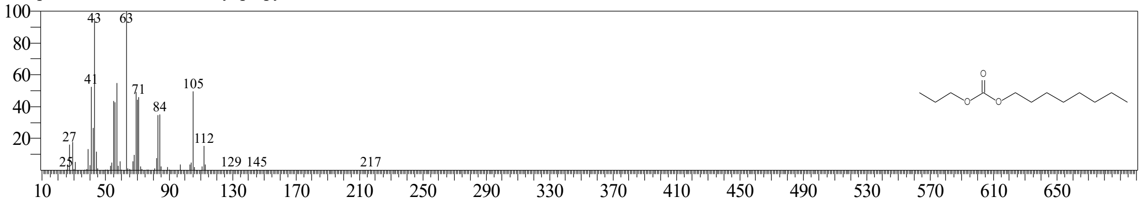

Hit#:5 Entry:92779 Library:NIST11.lib
SI:69 Formula:C10H21I CAS:0-00-0 MolWeight:268 RetIndex:1365
CompName:1-Iodo-2-methylnonane

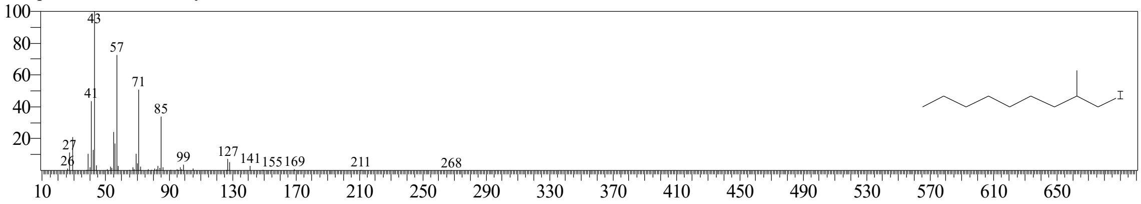

<< Target >>
Line#:12 R.Time:5.460(Scan#:293) MassPeaks:379
RawMode:Averaged 5.455-5.465(292-294) BasePeak:43.00(402025)
BG Mode:Calc. from Peak Group 1 - Event 1 Scan

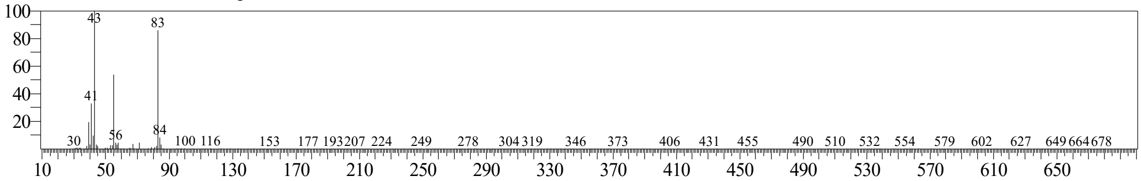

Hit#:1 Entry:1702 Library:NIST11.lib
SI:86 Formula:C6H10O CAS:763-93-9 MolWeight:98 RetIndex:762
CompName:3-Hexen-2-one $$ 1-Butenyl methyl ketone $$ Methyl 1-butenyl ketone $$ Hex-3-en-2-one $$

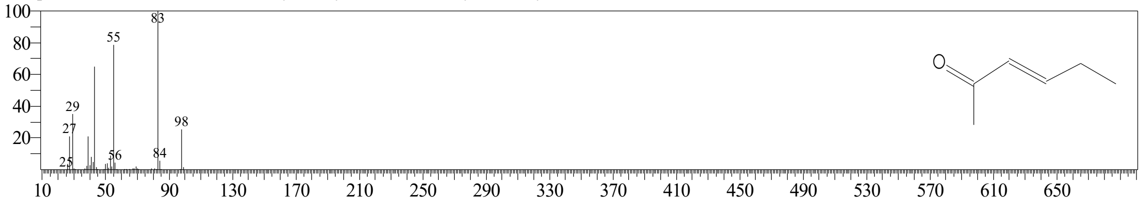

Hit#:2 Entry:6418 Library:NIST11.lib
SI:86 Formula:C7H10O2 CAS:15121-02-5 MolWeight:126 RetIndex:920
CompName:Cyclopentane, 1-acetyl-1,2-epoxy- $$ 1-(6-Oxabicyclo[3.1.0]hex-1-yl)ethanone # $$

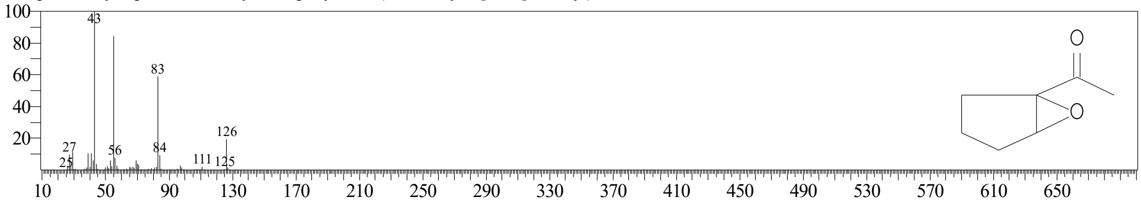

Hit#:3 Entry:1648 Library:NIST11.lib
SI:86 Formula:C6H10O CAS:4359-77-7 MolWeight:98 RetIndex:721
CompName:2-Pentanone, 3-methylene- $$ 3-Buten-2-one, 3-ethyl- $$ 3-Ethyl-3-buten-2-one # $$ 3-Methylene-2-pentanone $$

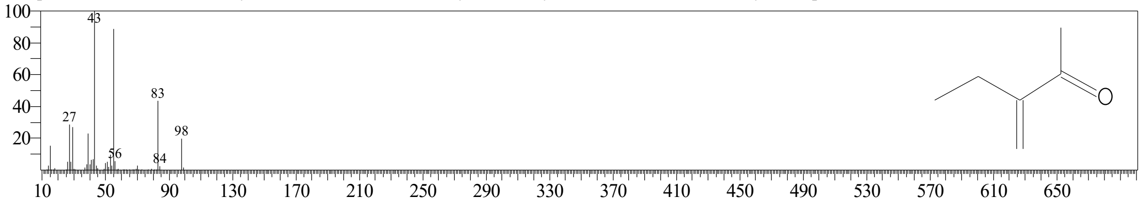

Hit#:4 Entry:63462 Library:NIST11.lib
SI:85 Formula:C12H20O4 CAS:0-00-0 MolWeight:228 RetIndex:1548
CompName:Oxalic acid, cyclohexyl isobutyl ester

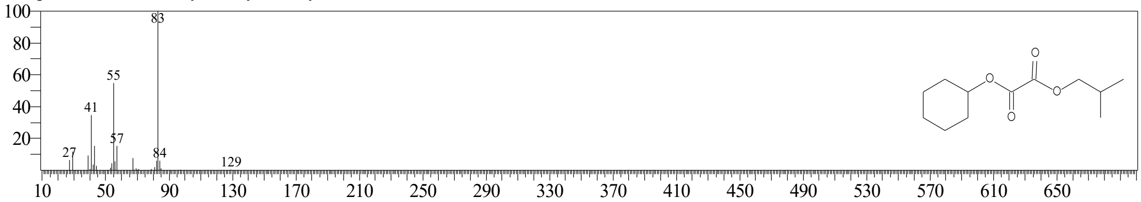

Hit#:5 Entry:6711 Library:NIST11.lib
SI:85 Formula:C9H18 CAS:55702-61-9 MolWeight:126 RetIndex:775
CompName:2-Hexene, 4,4,5-trimethyl- $$ (2E)-4,4,5-Trimethyl-2-hexene # $$

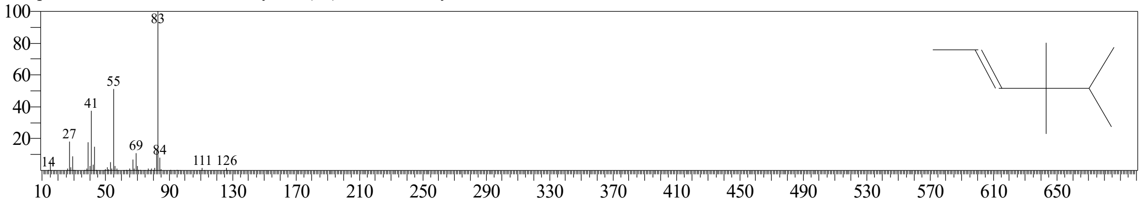

<< Target >>
Line#:13   R.Time:5.665(Scan#:334) MassPeaks:299
RawMode:Averaged 5.660-5.670(333-335) BasePeak:43.05(62603)
BG Mode:Calc. from Peak Group 1 - Event 1 Scan

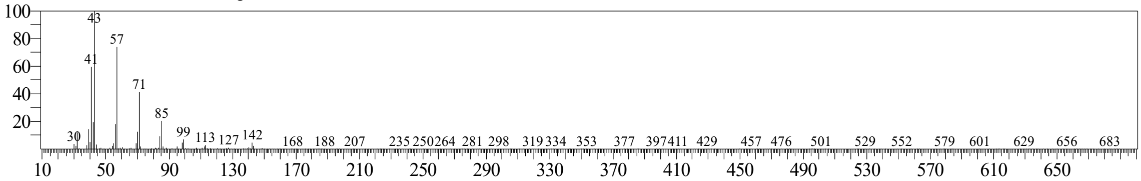

Hit#:1 Entry:12306 Library:NIST11.lib
SI:90 Formula:C10H22 CAS:871-83-0 MolWeight:142 RetIndex:951
CompName:Nonane, 2-methyl- $$ 2-Methylnonane $$

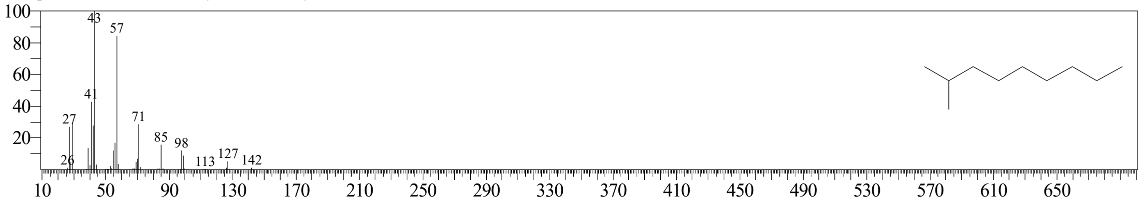

Hit#:2 Entry:26094 Library:NIST11.lib
SI:89 Formula:C12H26 CAS:1002-17-1 MolWeight:170 RetIndex:1086
CompName:Decane, 2,9-dimethyl- $$ 2,9-Dimethyldecane $$

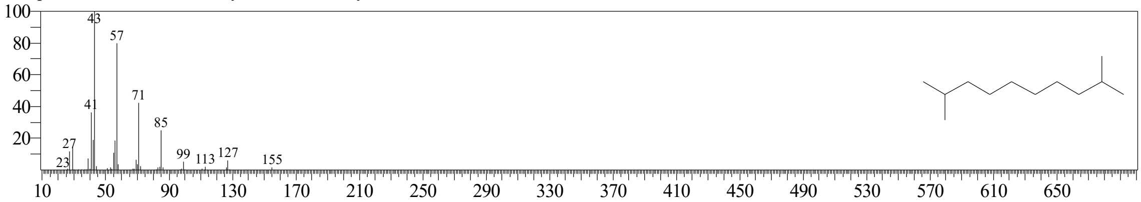

Hit#:3 Entry:34138 Library:NIST11.lib
SI:89 Formula:C13H28 CAS:1560-97-0 MolWeight:184 RetIndex:1249
CompName:Dodecane, 2-methyl- $$ 11-Methyldodecane $$ 2-Methyldodecane $$

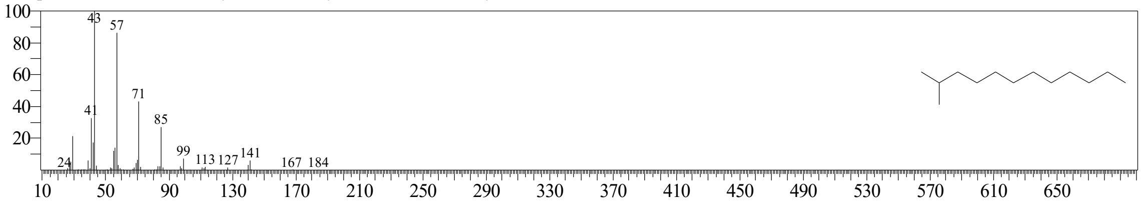

Hit#:4 Entry:12320 Library:NIST11.lib
SI:89 Formula:C10H22 CAS:124-18-5 MolWeight:142 RetIndex:1015
CompName:Decane $$ n-Decane $$ n-C10H22 $$ UN 2247 $$

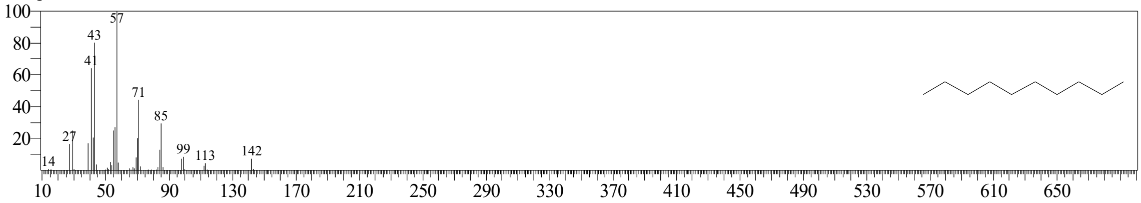

Hit#:5 Entry:18715 Library:NIST11.lib
SI:88 Formula:C11H24 CAS:1120-21-4 MolWeight:156 RetIndex:1115
CompName:Undecane $$ n-Undecane $$ Hendecane $$ n-C11H24 $$ UN 2330 $$

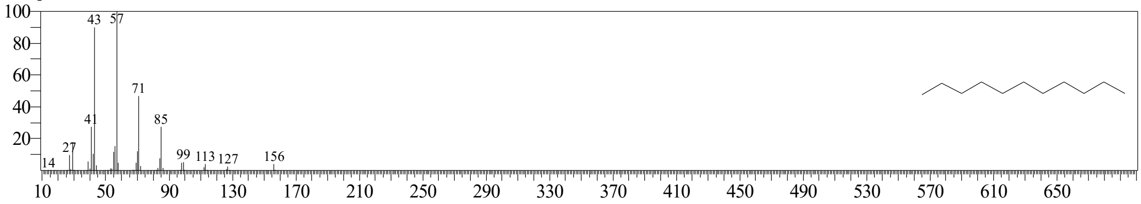

<< Target >>
Line#:14 R.Time:5.710(Scan#:343) MassPeaks:345
RawMode:Averaged 5.705-5.715(342-344) BasePeak:105.05(37917)
BG Mode:Calc. from Peak Group 1 - Event 1 Scan

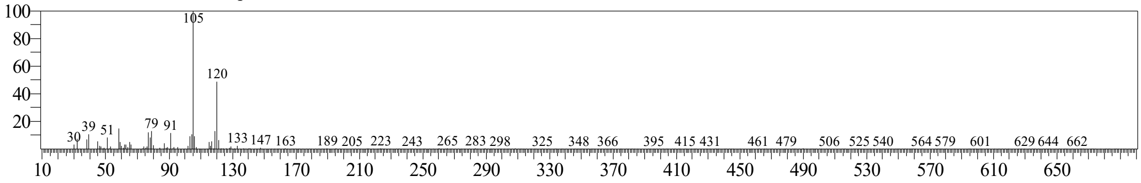

Hit#:1 Entry:5337 Library:NIST11.lib
SI:89 Formula:C9H12 CAS:95-63-6 MolWeight:120 RetIndex:1020
CompName:Benzene, 1,2,4-trimethyl- $$ .psi.-Cumene $$ aS-Trimethylbenzene $$ Pseudocumene $$ Pseudocumol $$ 1,2,4-Trimethylbenzene $$ 1,2,5-Trimeth

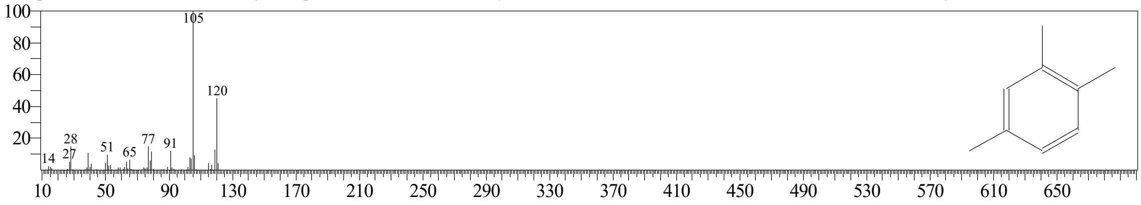

Hit#:2 Entry:5339 Library:NIST11.lib
SI:87 Formula:C9H12 CAS:526-73-8 MolWeight:120 RetIndex:1020
CompName:Benzene, 1,2,3-trimethyl- $$ Hemimellitene $$ 1,2,3-Trimethylbenzene $$ Hemellitol $$ 1,2,3-Trimethyl benzene $$ 1,2,3-Trimethylbenzene $$

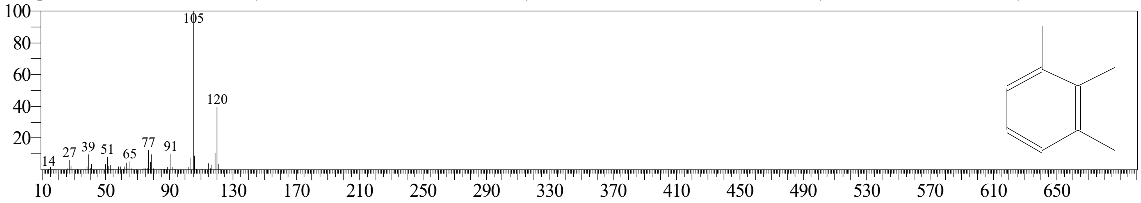

Hit#:3 Entry:5347 Library:NIST11.lib
SI:85 Formula:C9H12 CAS:108-67-8 MolWeight:120 RetIndex:1020
CompName:Mesitylene $$ Benzene, 1,3,5-trimethyl- $$ s-Trimethylbenzene $$ 1,3,5-Trimethylbenzene $$ sym-Trimethylbenzene $$ Fleet-X $$ TMB $$ UN 23

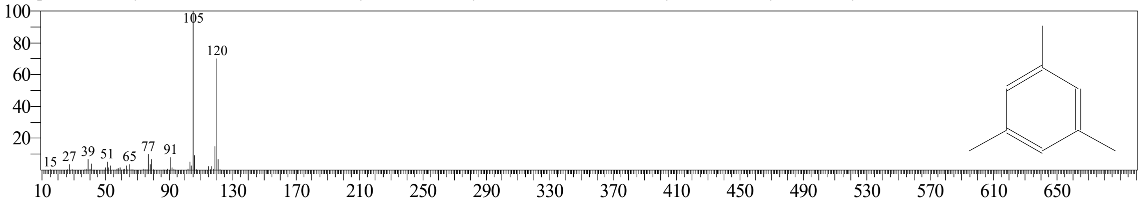

Hit#:4 Entry:5343 Library:NIST11.lib
SI:84 Formula:C9H12 CAS:620-14-4 MolWeight:120 RetIndex:1006
CompName:Benzene, 1-ethyl-3-methyl- $$ Toluene, m-ethyl- $$ m-Ethylmethylbenzene $$ m-Ethyltoluene $$ m-Methylethylbenzene $$ 1-Ethyl-3-methylbenze

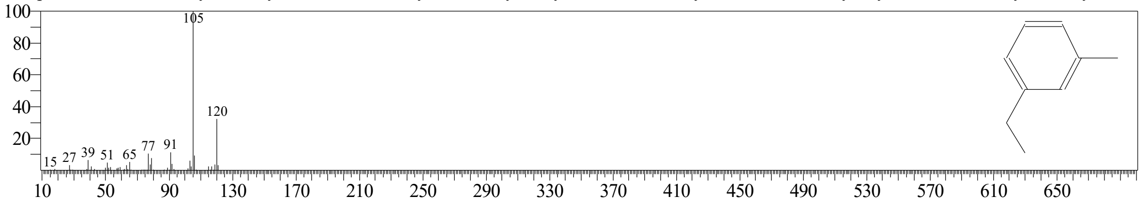

Hit#:5 Entry:5341 Library:NIST11.lib
SI:83 Formula:C9H12 CAS:611-14-3 MolWeight:120 RetIndex:1006
CompName:Benzene, 1-ethyl-2-methyl- $$ Toluene, o-ethyl- $$ o-Ethylmethylbenzene $$ o-Ethyltoluene $$ o-Methylethylbenzene $$ 1-Ethyl-2-methylbenzene

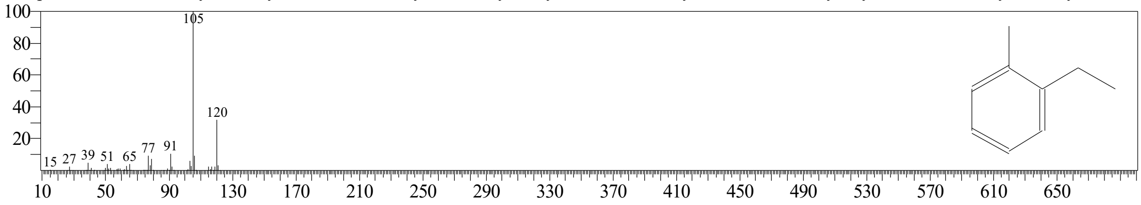

<< Target >>
Line#:15 R.Time:5.850(Scan#:371) MassPeaks:376
RawMode:Averaged 5.845-5.855(370-372) BasePeak:73.05(5355)
BG Mode:Calc. from Peak Group 1 - Event 1 Scan

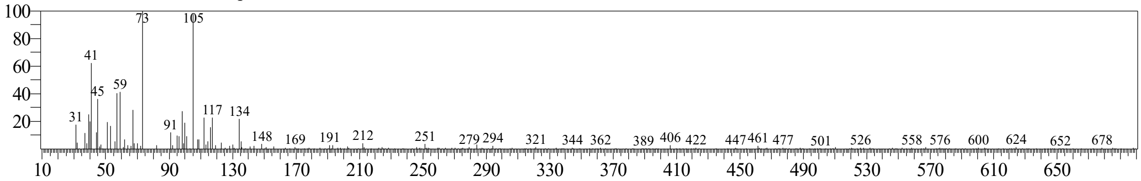

Hit#:1 Entry:14405 Library:NIST11.lib
SI:57 Formula:C7H16O3 CAS:55956-21-3 MolWeight:148 RetIndex:983
CompName:1-Propanol, 2-(2-methoxy-1-methylethoxy)- $$ 2-(2-Methoxy-1-methylethoxy)-1-propanol $$ Dipropylene glycol monomethyl ether (Salt/Mix) $$ I

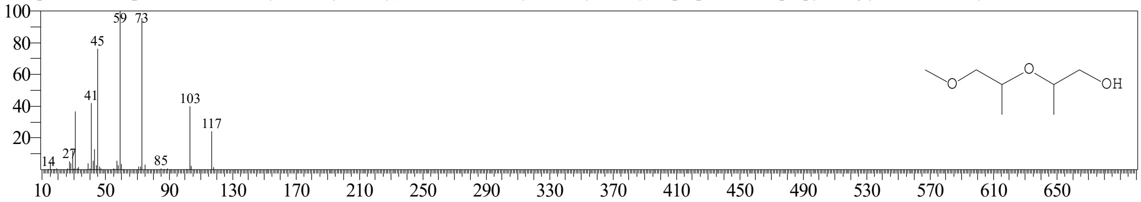

Hit#:2 Entry:29789 Library:NIST11.lib
SI:56 Formula:C6H10S3 CAS:2050-87-5 MolWeight:178 RetIndex:1350
CompName:Trisulfide, di-2-propenyl $$ Allyl trisulfide $$ Diallyl trisulfide $$ 1,3-Diallyltrisulfane # $$

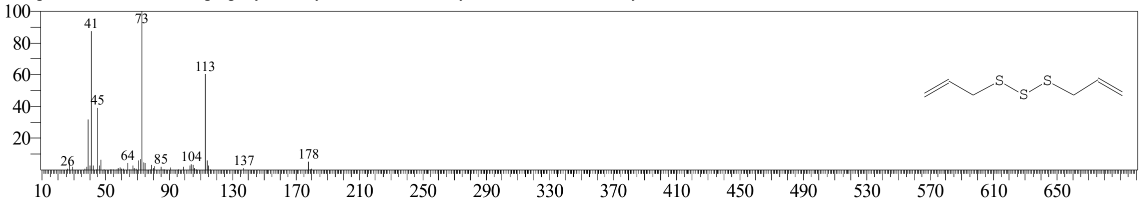

Hit#:3 Entry:7617 Library:NIST11.lib
SI:55 Formula:C7H15NO CAS:1188-63-2 MolWeight:129 RetIndex:1050
CompName:4-Heptanone, oxime $$ Dipropyl ketone oxime $$ Dipropyl ketoxime $$

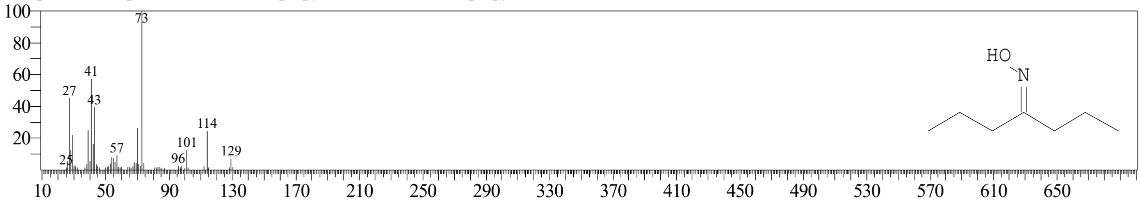

Hit#:4 Entry:18962 Library:NIST11.lib
SI:55 Formula:C9H19NO CAS:14475-42-4 MolWeight:157 RetIndex:1248
CompName:5-Nonanone, oxime

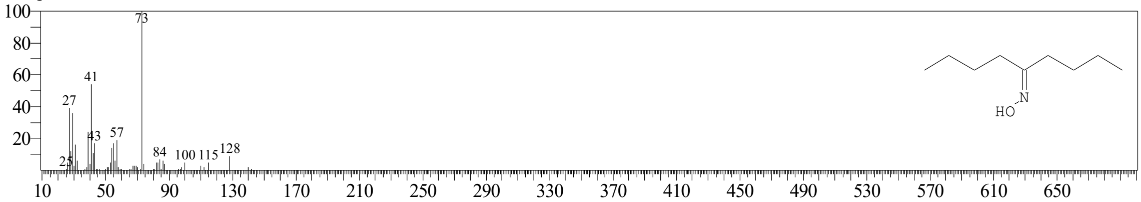

Hit#:5 Entry:49514 Library:NIST11.lib
SI:54 Formula:C13H24Si CAS:97778-16-0 MolWeight:208 RetIndex:1157
CompName:Silane, [1-(5-hexenyl)-2-methylenecyclopropyl]trimethyl- $$ [1-(5-Hexenyl)-2-methylenecyclopropyl](trimethyl)silane # $$

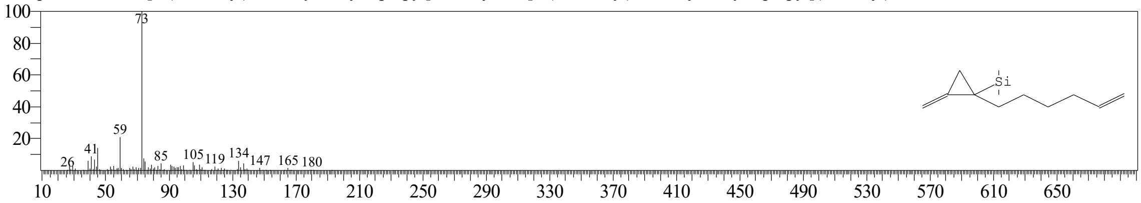

Qualitative Analysis Report

<< Target >>
Line#:16 R.Time:5.895(Scan#:380) MassPeaks:350
RawMode:Averaged 5.890-5.900(379-381) BasePeak:43.05(30852)
BG Mode:Calc. from Peak Group 1 - Event 1 Scan

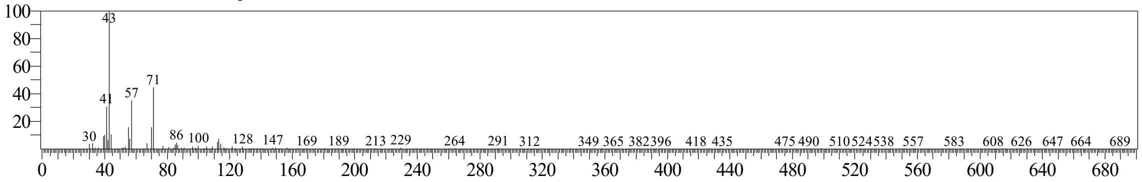

Hit#:1 Entry:4243 Library:NIST11.lib
SI:85 Formula:C8H18 CAS:560-21-4 MolWeight:114 RetIndex:668
CompName:Pentane, 2,3,3-trimethyl- $$ 2,3,3-Trimethylpentane $$ C2H5C(CH3)2CH(CH3)2 $$

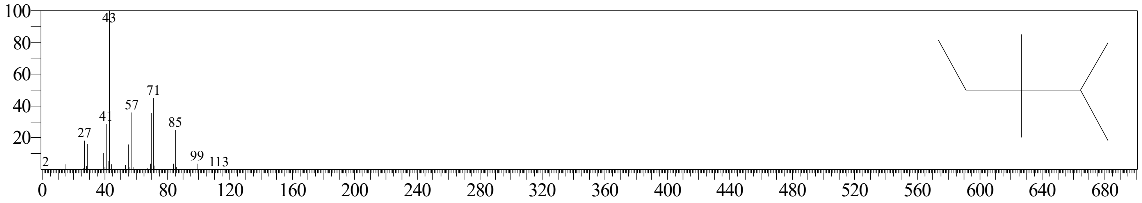

Hit#:2 Entry:7413 Library:NIST11.lib
SI:84 Formula:C9H20 CAS:921-47-1 MolWeight:128 RetIndex:724
CompName:Hexane, 2,3,4-trimethyl- $$ 2,3,4-Trimethylhexane $$

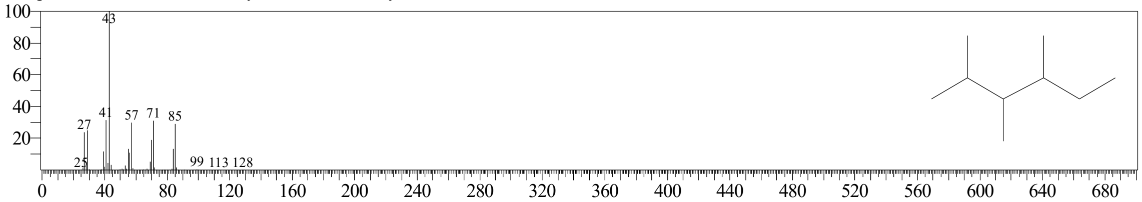

Hit#:3 Entry:12309 Library:NIST11.lib
SI:84 Formula:C10H22 CAS:5881-17-4 MolWeight:142 RetIndex:951
CompName:Octane, 3-ethyl- $$ 3-Ethyloctane $$

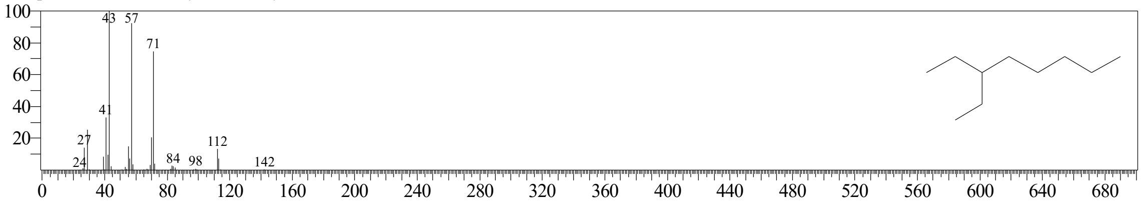

Hit#:4 Entry:7426 Library:NIST11.lib
SI:84 Formula:C9H20 CAS:2216-34-4 MolWeight:128 RetIndex:852
CompName:Octane, 4-methyl- $$ 4-Methyloctane $$ 1,3-Dimethylheptane $$

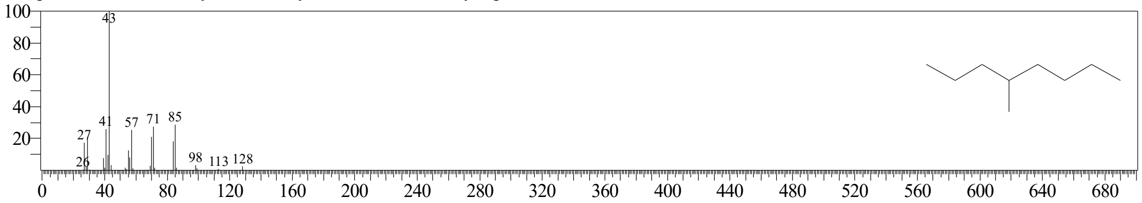

Hit#:5 Entry:7415 Library:NIST11.lib
SI:84 Formula:C9H20 CAS:1072-05-5 MolWeight:128 RetIndex:788
CompName:Heptane, 2,6-dimethyl- $$ 2,6-Dimethylheptane $$

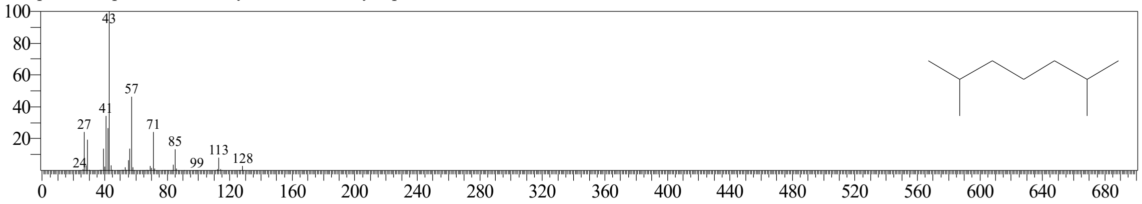

<< Target >>
Line#:17 R.Time:5.950(Scan#:391) MassPeaks:367
RawMode:Averaged 5.945-5.955(390-392) BasePeak:55.05(4827)
BG Mode:Calc. from Peak Group 1 - Event 1 Scan

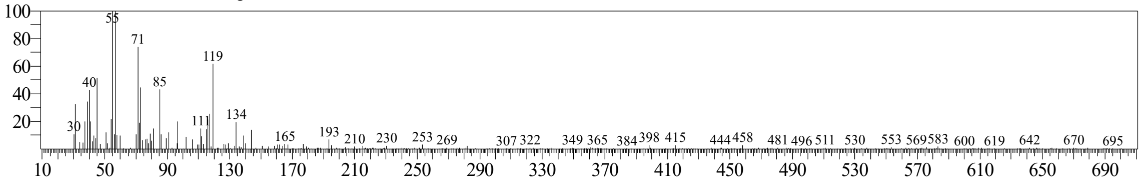

Hit#:1 Entry:4152 Library:NIST11.lib
SI:65 Formula:C7H14O CAS:14925-96-3 MolWeight:114 RetIndex:769
CompName:Heptane, 2,3-epoxy- $$ 2,3-Epoxyheptane $$ Oxirane, 2-butyl-3-methyl- $$ 2-Butyl-3-methyloxirane # $$

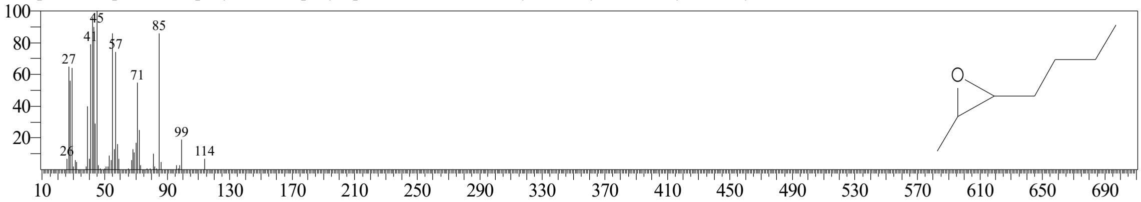

Hit#:2 Entry:12834 Library:NIST11.lib
SI:65 Formula:C7H12O3 CAS:0-00-0 MolWeight:144 RetIndex:968
CompName:(E)-But-2-enyl ethyl carbonate

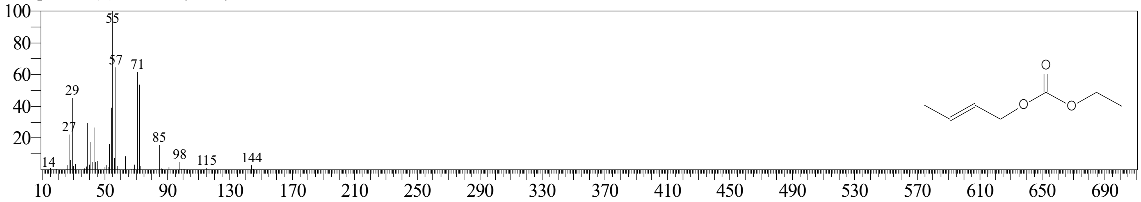

Hit#:3 Entry:13761 Library:NIST11.lib
SI:65 Formula:C8H18O2 CAS:4161-24-4 MolWeight:146 RetIndex:1135
CompName:1-Butanol, 4-butoxy- $$ 1,4-Tetrabutyleneglycol monobutyl ether $$ 4-Butoxybutanol $$ Butylene glycol monobutyl ether $$ 4-Butoxy-1-butanol #

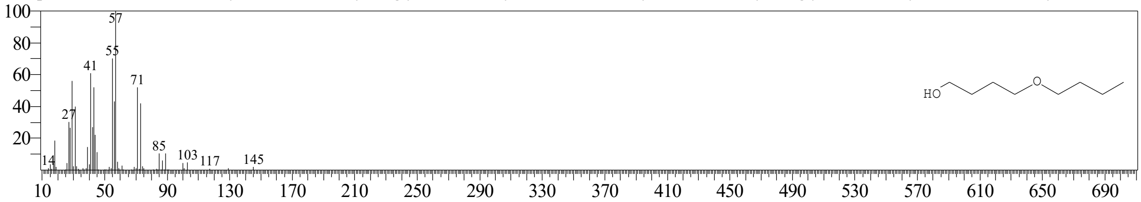

Hit#:4 Entry:26875 Library:NIST11.lib
SI:64 Formula:C9H16O3 CAS:0-00-0 MolWeight:172 RetIndex:1103
CompName:(E)-But-2-enyl isobutyl carbonate

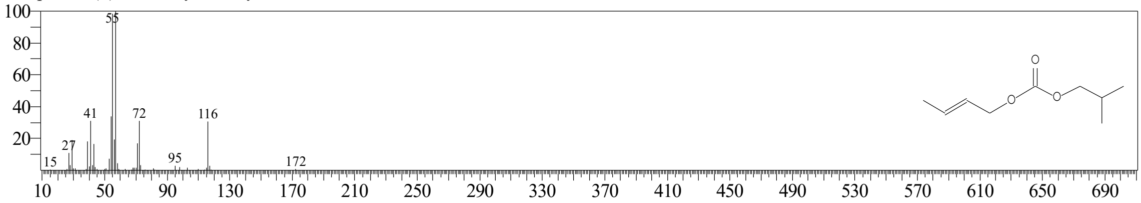

Hit#:5 Entry:35006 Library:NIST11.lib
SI:64 Formula:C10H18O3 CAS:157733-17-0 MolWeight:186 RetIndex:1349
CompName:2-t-Butyl-5-propyl-[1,3]dioxolan-4-one $$ 2-tert-Butyl-5-propyl-1,3-dioxolan-4-one # $$

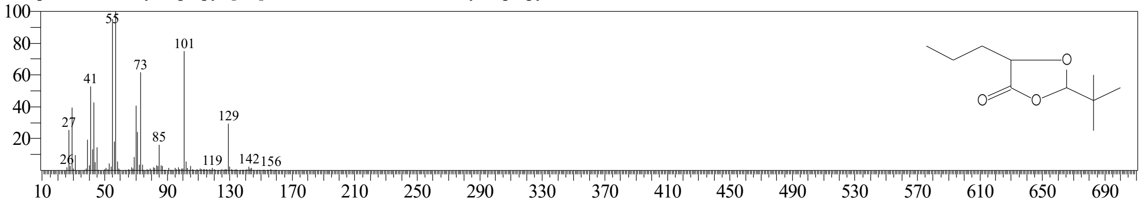

<< Target >>
Line#:18 R.Time:6.015(Scan#:404) MassPeaks:325
RawMode:Averaged 6.010-6.020(403-405) BasePeak:105.10(12135)
BG Mode:Calc. from Peak Group 1 - Event 1 Scan

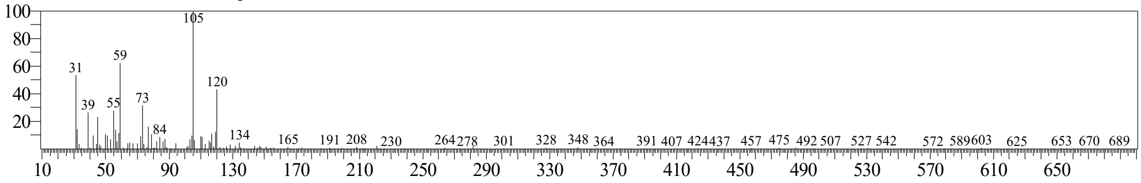

Hit#:1 Entry:5214 Library:NIST11.lib
SI:62 Formula:C4H12SSi CAS:3908-55-2 MolWeight:120 RetIndex:580
CompName:Trimethylsilyl methyl sulfide $$ (CH3)3SiSCH3 $$ Methylthiotrimethylsilane $$ Silane, trimethyl(methylthio)- $$ Methyl trimethylsilyl sulfide # $

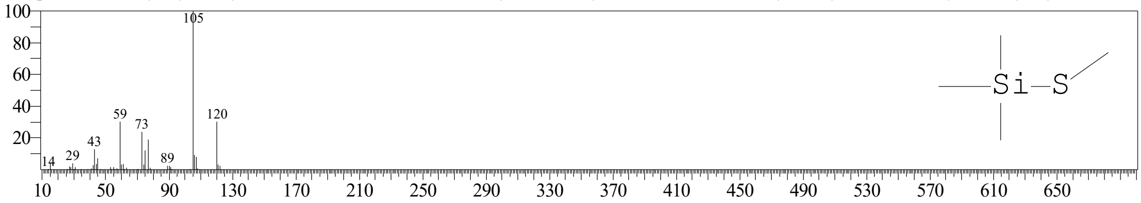

Hit#:2 Entry:56724 Library:NIST11.lib
SI:61 Formula:C12H17N3O CAS:0-00-0 MolWeight:219 RetIndex:1961
CompName:1-(1-Phenylethylcarbamidoyl)-3,3-dimethyldiaziridine $$ 3,3-Dimethyl-N-(1-phenylethyl)-1-diaziridinecarboxamide # $$

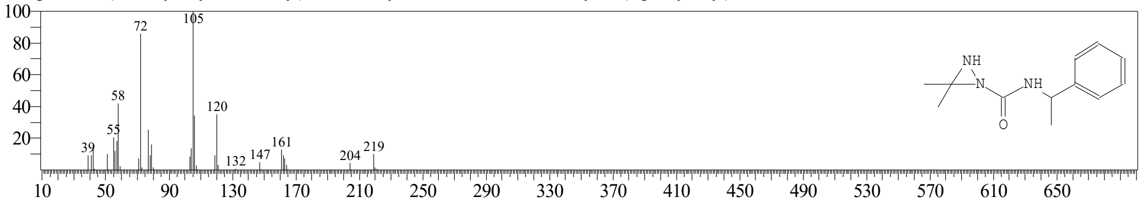

Hit#:3 Entry:157747 Library:NIST11.lib
SI:60 Formula:C16H22N2O7 CAS:0-00-0 MolWeight:354 RetIndex:3193
CompName:Methyl N-hippuroyl-beta-d-glucosaminide $$ Methyl 2-deoxy-2-(([(2-oxo-2-phenylethyl)amino]carbonyl)amino)hexopyranoside # $$

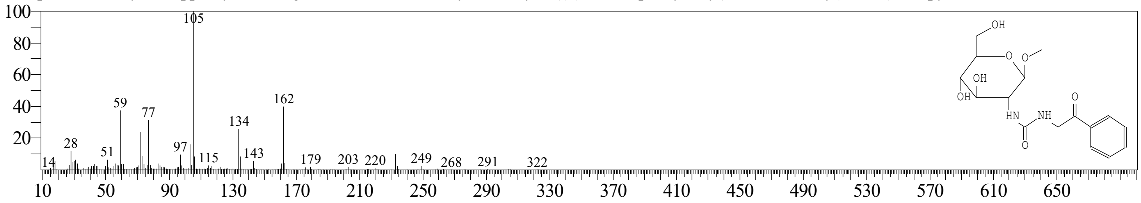

Hit#:4 Entry:5339 Library:NIST11.lib
SI:60 Formula:C9H12 CAS:526-73-8 MolWeight:120 RetIndex:1020
CompName:Benzene, 1,2,3-trimethyl- $$ Hemimellitene $$ 1,2,3-Trimethylbenzene $$ Hemellitol $$ 1,2,3-Trimethyl benzene $$ 1,2,3-Trimethylbenzene $$

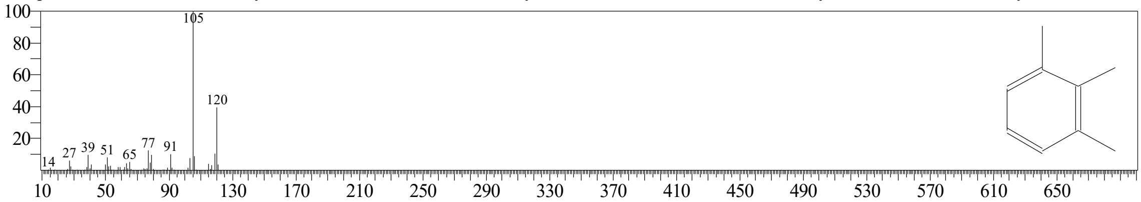

Hit#:5 Entry:70498 Library:NIST11.lib
SI:60 Formula:C13H18O4 CAS:0-00-0 MolWeight:238 RetIndex:1709
CompName:2-(2-Ethoxyethoxy)ethyl benzoate

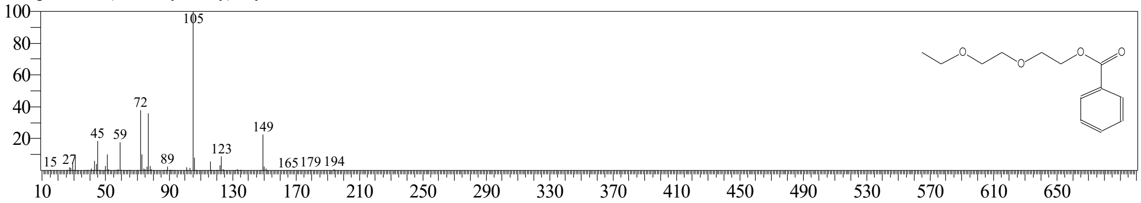

<< Target >>
Line#:19 R.Time:6.080(Scan#:417) MassPeaks:323
RawMode:Averaged 6.075-6.085(416-418) BasePeak:55.10(7941)
BG Mode:Calc. from Peak Group 1 - Event 1 Scan

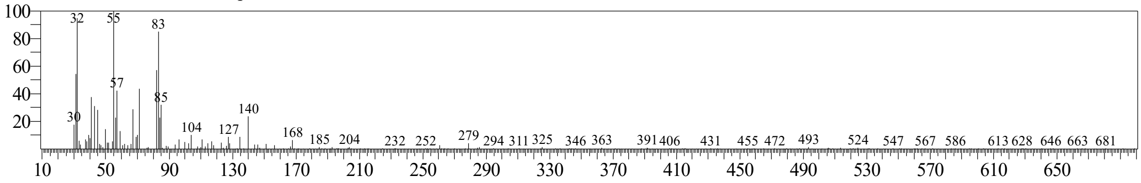

Hit#:1 Entry:8030 Library:NIST11.lib
SI:70 Formula:C8H18O CAS:57803-73-3 MolWeight:130 RetIndex:995
CompName:(S)-(+)-5-Methyl-1-heptanol $$ 5-Methyl-1-heptanol # $$

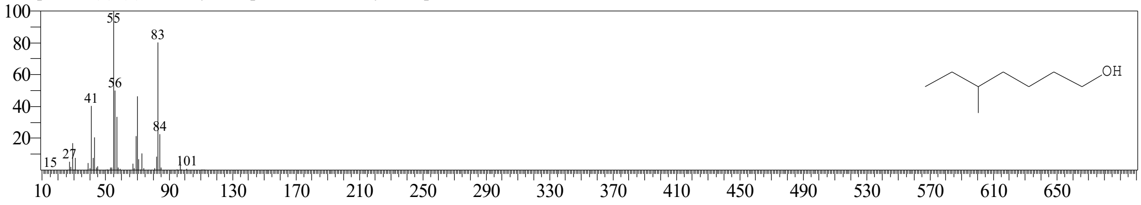

Hit#:2 Entry:27227 Library:NIST11.lib
SI:69 Formula:C11H24O CAS:213547-15-0 MolWeight:172 RetIndex:1213
CompName:4-Methyl-5-decanol

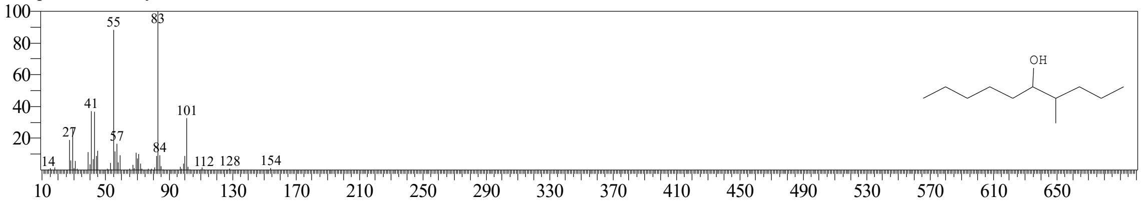

Hit#:3 Entry:13115 Library:NIST11.lib
SI:69 Formula:C9H20O CAS:18450-74-3 MolWeight:144 RetIndex:1030
CompName:1-Heptanol, 2,4-dimethyl-, (2S,4R)-(-)- $$ 2,4-Dimethyl-1-heptanol # $$

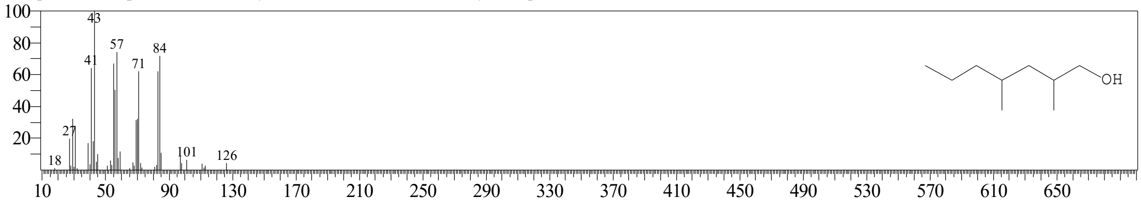

Hit#:4 Entry:13292 Library:NIST11.lib
SI:68 Formula:C7H15NO2 CAS:693-39-0 MolWeight:145 RetIndex:1099
CompName:Heptane, 1-nitro- $$ 1-Nitroheptane $$

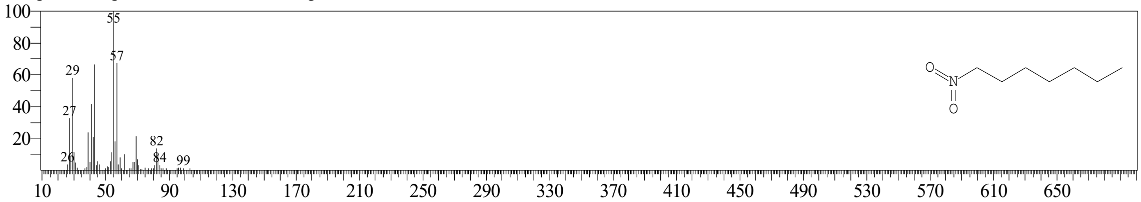

Hit#:5 Entry:8511 Library:NIST11.lib
SI:68 Formula:C7H16O2 CAS:78-26-2 MolWeight:132 RetIndex:1118
CompName:1,3-Propanediol, 2-methyl-2-propyl- $$ 2-Methyl-2-propyl-1,3-propanediol $$ 2-Methyl-2-n-propyl-1,3-propanediol $$

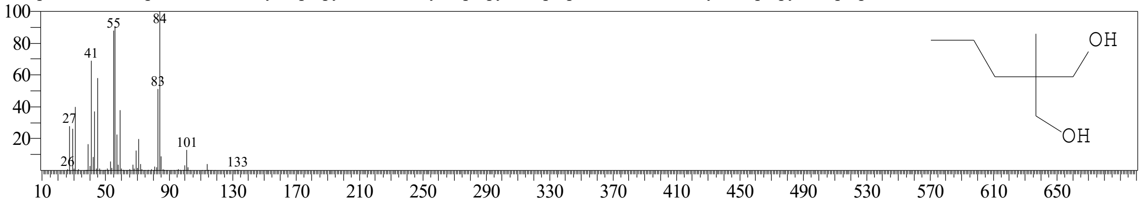

<< Target >>
Line#:20 R.Time:6.365(Scan#:474) MassPeaks:378
RawMode:Averaged 6.360-6.370(473-475) BasePeak:41.10(10863)
BG Mode:Calc. from Peak Group 1 - Event 1 Scan

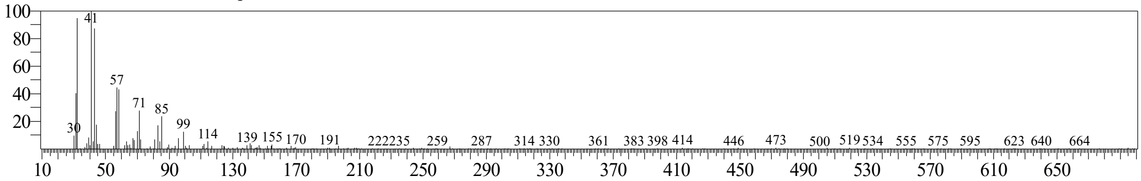

Hit#:1 Entry:7279 Library:NIST11.lib
SI:74 Formula:C8H16O CAS:23186-70-1 MolWeight:128 RetIndex:882
CompName:Pentane, 1-(2-propenyloxy)- $$ Ether, allyl pentyl $$ Allyl pentyl ether $$ Pentyl allyl ether $$ 3-(Pentyloxy)-1-propene # $$

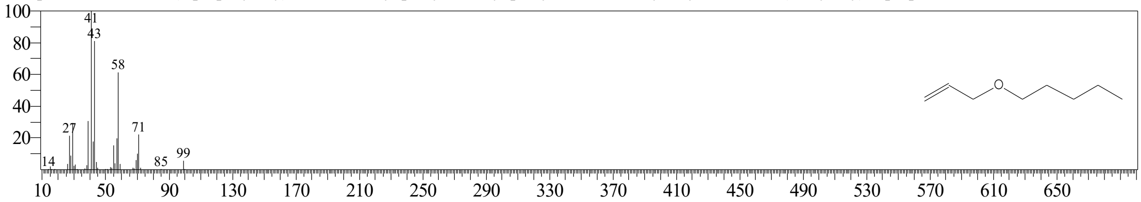

Hit#:2 Entry:26031 Library:NIST11.lib
SI:73 Formula:C11H22O CAS:3295-97-4 MolWeight:170 RetIndex:1181
CompName:Allyl n-octyl ether $$ Allyl octyl ether $$ 3-(Octyloxy)-1-propene # $$

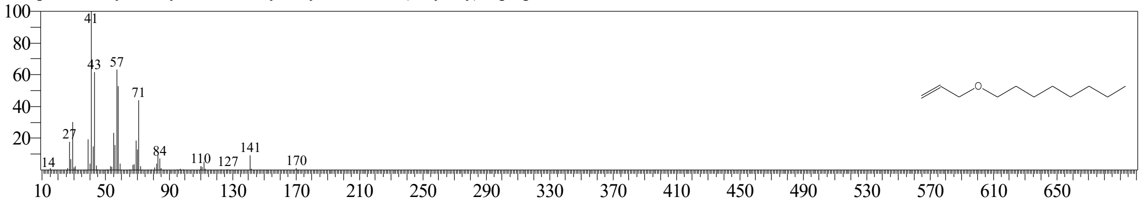

Hit#:3 Entry:18620 Library:NIST11.lib
SI:73 Formula:C10H20O CAS:103-44-6 MolWeight:156 RetIndex:1017
CompName:Heptane, 3-[(ethenyloxy)methyl]- $$ Ether, 2-ethylhexyl vinyl $$ Vinyl 2-ethylhexyl ether $$ 1-Ethenoxy-2-ethylhexane $$ 2-Ethylhexyl vinyl ether

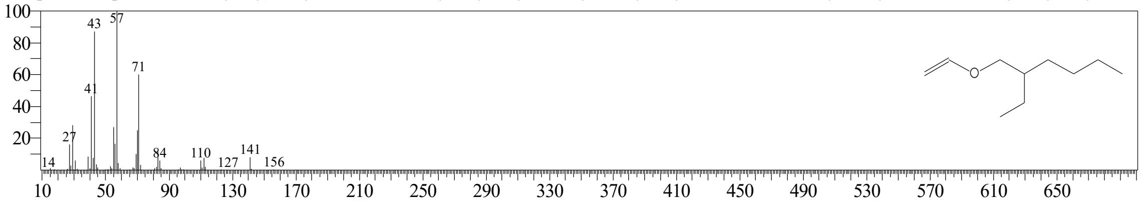

Hit#:4 Entry:19558 Library:NIST11.lib
SI:72 Formula:C10H22O CAS:68680-98-8 MolWeight:158 RetIndex:1130
CompName:1-Octanol, 3,7-dimethyl-, (S)- $$ (-)-3,7-Dimethyl-1-octanol $$ (S)-3,7-Dimethyl-1-octanol $$ (S)-Dihydrocitronellol $$

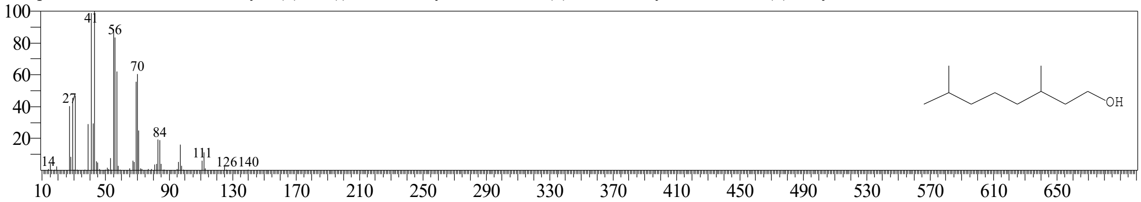

Hit#:5 Entry:94828 Library:NIST11.lib
SI:72 Formula:C15H26O4 CAS:0-00-0 MolWeight:270 RetIndex:1837
CompName:Oxalic acid, allyl decyl ester

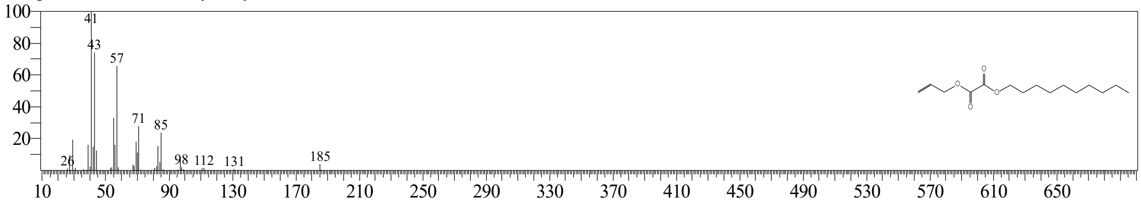

<< Target >>
Line#:21 R.Time:6.430(Scan#:487) MassPeaks:378
RawMode:Averaged 6.425-6.435(486-488) BasePeak:41.10(5064)
BG Mode:Calc. from Peak Group 1 - Event 1 Scan

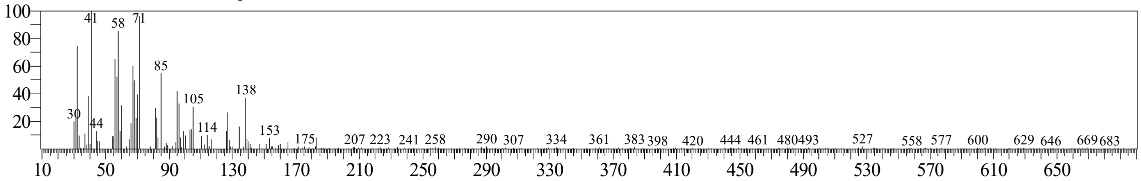

Hit#:1 Entry:18667 Library:NIST11.lib
SI:69 Formula:C10H20O CAS:2404-44-6 MolWeight:156 RetIndex:1106
CompName:Oxirane, octyl- $$ 1,2-Epoxydecane $$ 2-Octyloxirane # $$ Decane, 1,2-epoxy $$

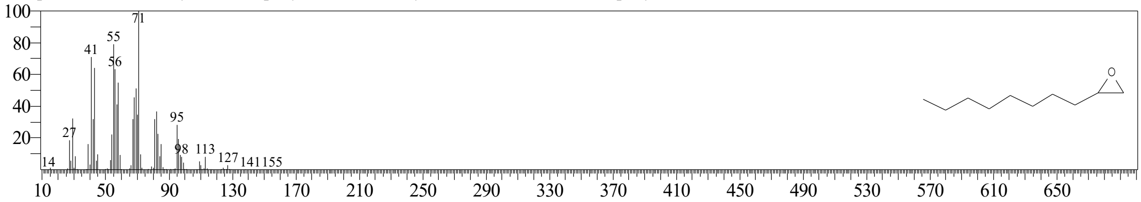

Hit#:2 Entry:12266 Library:NIST11.lib
SI:69 Formula:C9H18O CAS:28114-20-7 MolWeight:142 RetIndex:1006
CompName:1,2-Epoxynonane $$ 2-Heptyloxirane # $$

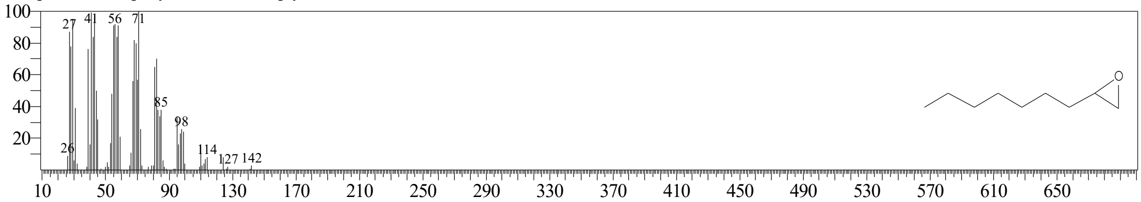

Hit#:3 Entry:34098 Library:NIST11.lib
SI:69 Formula:C12H24O CAS:2855-19-8 MolWeight:184 RetIndex:1304
CompName:Oxirane, decyl- $$ Dodecane, 1,2-epoxy- $$ Nedox 1200 $$ 1-Dodecene oxide $$ 1,2-Dodecane oxide $$ 1,2-Dodecene oxide $$ 1,2-Epoxy-n-dod

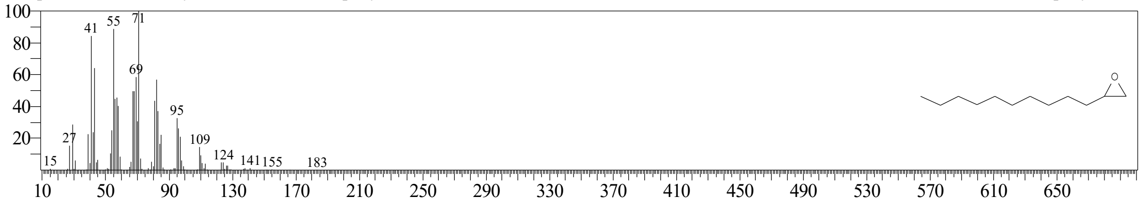

Hit#:4 Entry:72291 Library:NIST11.lib
SI:68 Formula:C15H28O2 CAS:0-00-0 MolWeight:240 RetIndex:1670
CompName:Pentanoic acid, 9-decenyl ester $$ 9-Decenyl pentanoate # $$

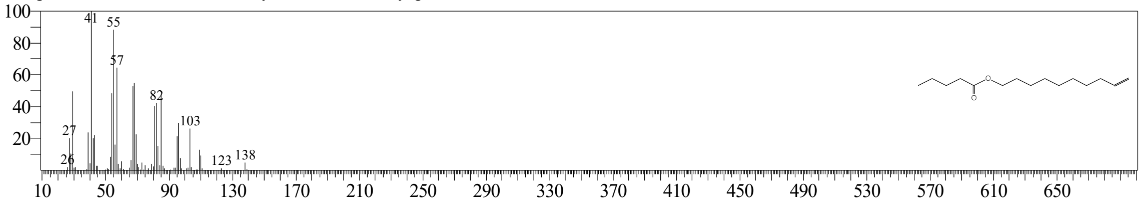

Hit#:5 Entry:7367 Library:NIST11.lib
SI:67 Formula:C8H16O CAS:2984-50-1 MolWeight:128 RetIndex:907
CompName:Oxirane, hexyl- $$ Octane, 1,2-epoxy- $$ .alpha.-Epoxyoctane $$ n-Octene-1,2-oxide $$ Hexyloxirane $$ Octane 1,2-oxide $$ Octene-1,2-oxide $$

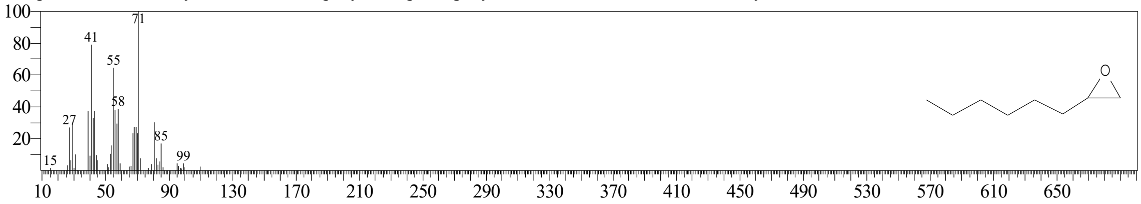

<< Target >>
Line#:22 R.Time:6.765(Scan#:554) MassPeaks:379
RawMode:Averaged 6.760-6.770(553-555) BasePeak:43.10(72257)
BG Mode:Calc. from Peak Group 1 - Event 1 Scan

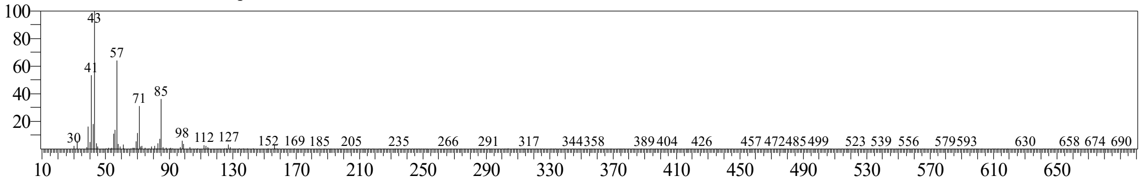

Hit#:1 Entry:12306 Library:NIST11.lib
SI:89 Formula:C10H22 CAS:871-83-0 MolWeight:142 RetIndex:951
CompName:Nonane, 2-methyl- $$ 2-Methylnonane $$

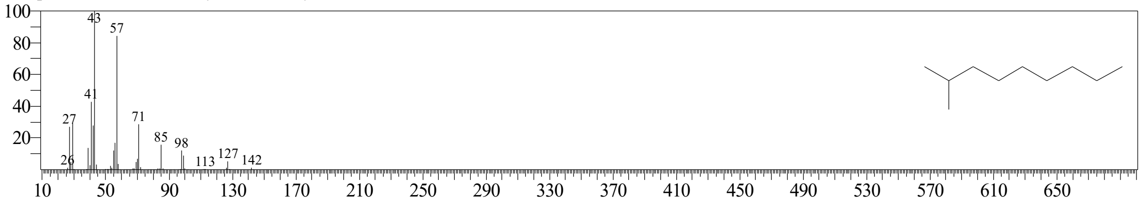

Hit#:2 Entry:4236 Library:NIST11.lib
SI:89 Formula:C8H18 CAS:589-43-5 MolWeight:114 RetIndex:688
CompName:Hexane, 2,4-dimethyl- $$ 2,4-Dimethylhexane $$

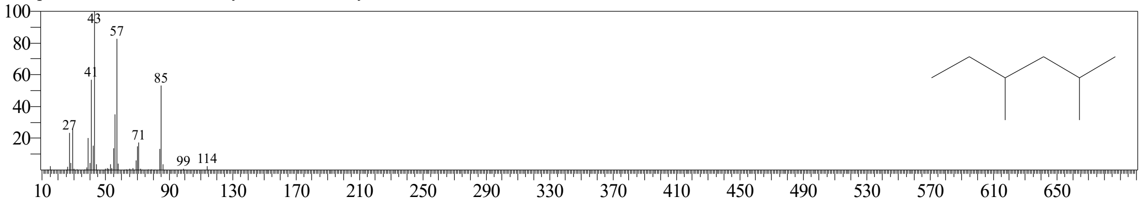

Hit#:3 Entry:26094 Library:NIST11.lib
SI:89 Formula:C12H26 CAS:1002-17-1 MolWeight:170 RetIndex:1086
CompName:Decane, 2,9-dimethyl- $$ 2,9-Dimethyldecane $$

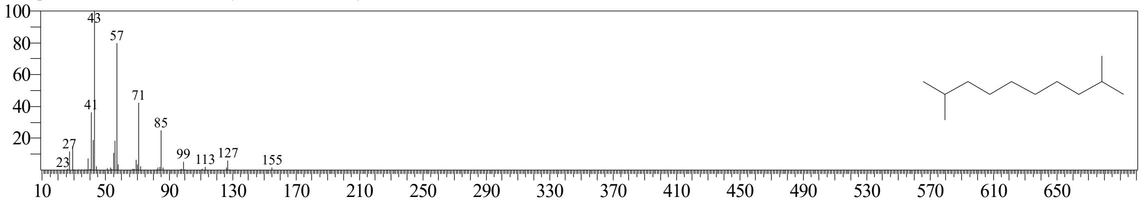

Hit#:4 Entry:34137 Library:NIST11.lib
SI:89 Formula:C13H28 CAS:62108-27-4 MolWeight:184 RetIndex:1121
CompName:Decane, 2,4,6-trimethyl- $$ 2,4,6-Trimethyldecane # $$

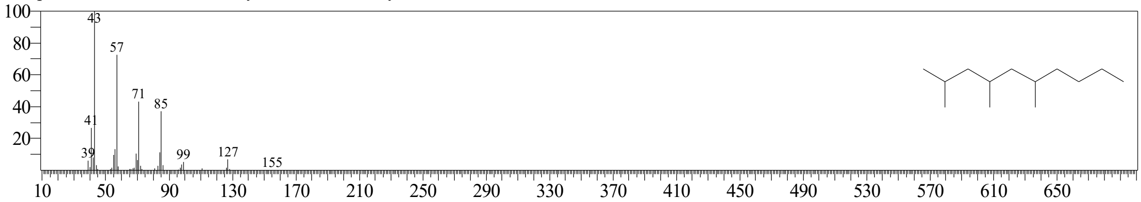

Hit#:5 Entry:34147 Library:NIST11.lib
SI:89 Formula:C13H28 CAS:17312-80-0 MolWeight:184 RetIndex:1185
CompName:Undecane, 2,4-dimethyl- $$ 2,4-Dimethylundecane $$

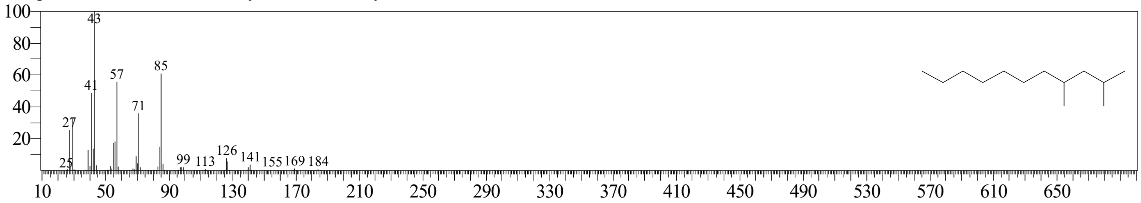

<< Target >>
Line#:23 R.Time:6.930(Scan#:587) MassPeaks:368
RawMode:Averaged 6.925-6.935(586-588) BasePeak:85.10(22766)
BG Mode:Calc. from Peak Group 1 - Event 1 Scan

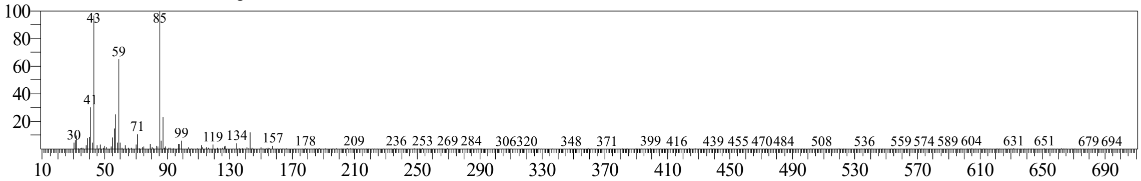

Hit#:1 Entry:4701 Library:NIST11.lib
SI:78 Formula:C7H16O CAS:2370-12-9 MolWeight:116 RetIndex:875
CompName:1-Pentanol, 2,2-dimethyl- $$ Neoheptanol $$ 2,2-Dimethyl-1-pentanol $$ 2,2-Dimethylpentanol $$

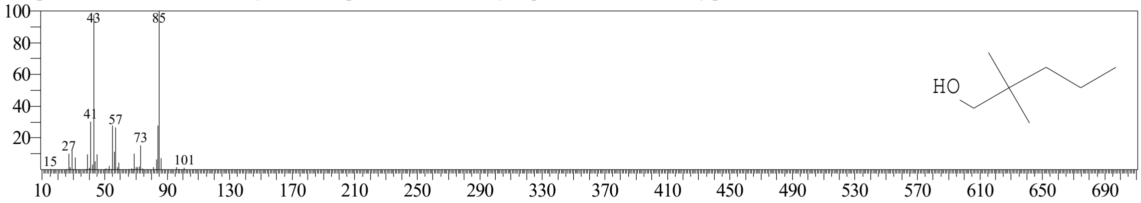

Hit#:2 Entry:18403 Library:NIST11.lib
SI:78 Formula:C9H16O2 CAS:44970-05-0 MolWeight:156 RetIndex:989
CompName:Pentanoic acid, 2,2-dimethyl-, ethenyl ester $$ Vinyl 2,2-dimethylpentanoate # $$

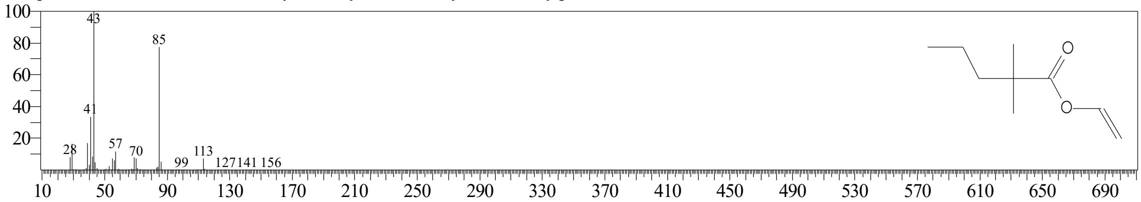

Hit#:3 Entry:2082 Library:NIST11.lib
SI:78 Formula:C6H12O CAS:10473-13-9 MolWeight:100 RetIndex:677
CompName:3-Buten-2-ol, 2,3-dimethyl- $$ CH2=C(CH3)C(CH3)2OH $$ 2,3-Dimethyl-3-buten-2-ol $$ 2,3-Dimethyl-1-buten-3-ol $$ 3-Hydroxy-2,3-dimethyl-

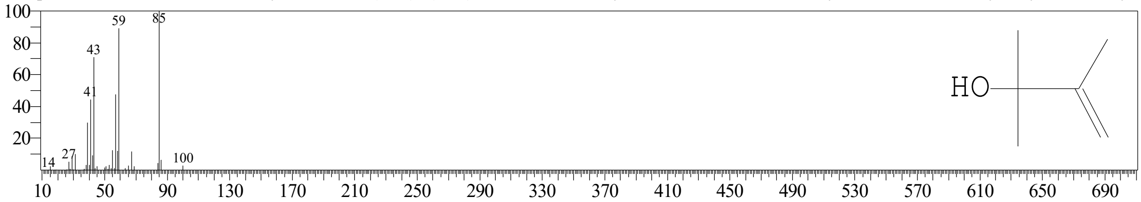

Hit#:4 Entry:4139 Library:NIST11.lib
SI:77 Formula:C7H14O CAS:17612-35-0 MolWeight:114 RetIndex:743
CompName:Oxirane, 2,2-dimethyl-3-propyl- $$ Hexane, 2,3-epoxy-2-methyl- $$ 2,2-Dimethyl-3-propyloxirane # $$

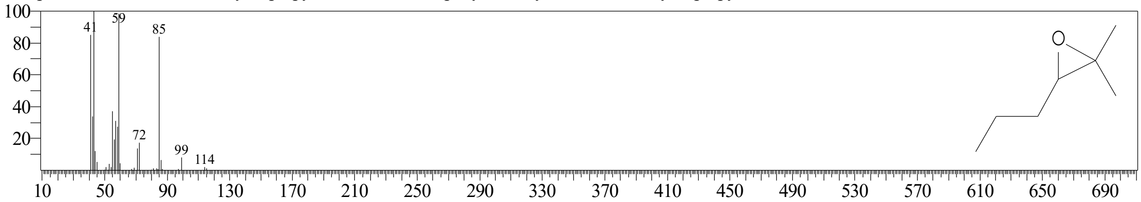

Hit#:5 Entry:19377 Library:NIST11.lib
SI:77 Formula:C9H18O2 CAS:102840-52-8 MolWeight:158 RetIndex:1009
CompName:Pentan-2-ol, 4-allyloxy-2-methyl- $$ 4-(Allyloxy)-2-methyl-2-pentanol # $$

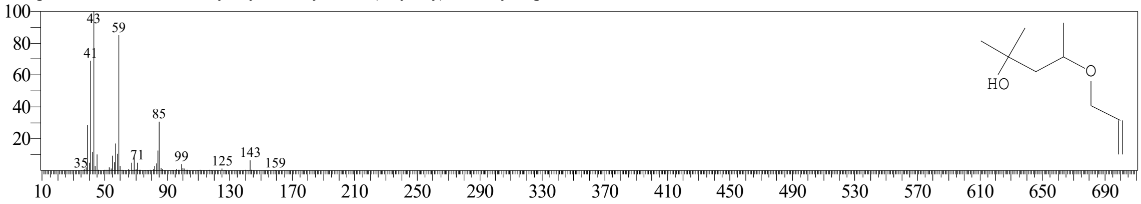

Qualitative Analysis Report

<< Target >>
Line#:24 R.Time:7.215(Scan#:644) MassPeaks:368
RawMode:Averaged 7.210-7.220(643-645) BasePeak:73.05(32611)
BG Mode:Calc. from Peak Group 1 - Event 1 Scan

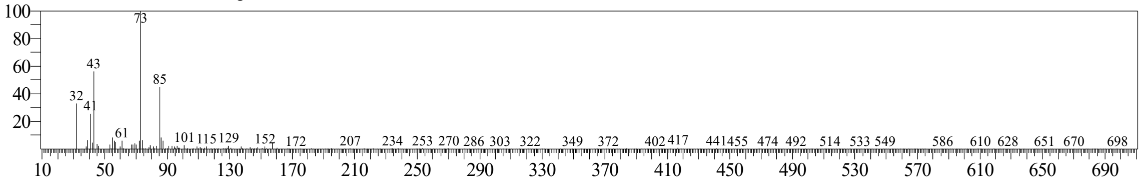

Hit#:1 Entry:36153 Library:NIST11.lib
SI:77 Formula:C10H20O3 CAS:0-00-0 MolWeight:188 RetIndex:1174
CompName:3-Methylpentan-3-yl propyl carbonate

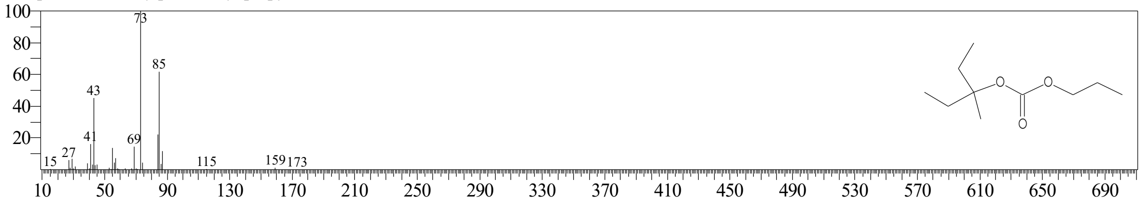

Hit#:2 Entry:12734 Library:NIST11.lib
SI:76 Formula:C6H12N2O2 CAS:871-78-3 MolWeight:144 RetIndex:1418
CompName:N,N'-Diacetylethylenediamine $$ Acetamide, N,N'-1,2-ethanediylbis- $$ Acetamide,N,N'-ethylenebis- $$ Ethane-1,2-diamine, N,N'-diacetyl- $$ N1-

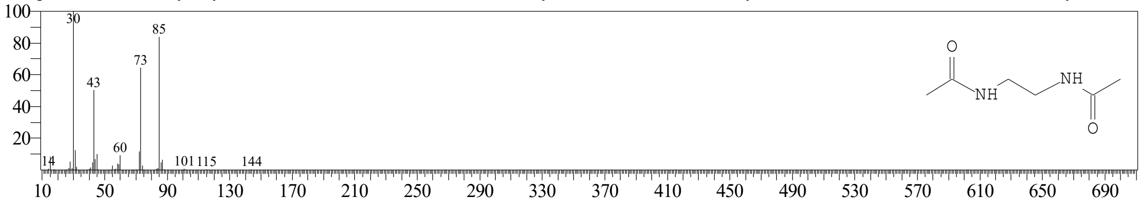

Hit#:3 Entry:8507 Library:NIST11.lib
SI:75 Formula:C7H16O2 CAS:141-73-1 MolWeight:132 RetIndex:871
CompName:4-Methoxy-4-methyl-2-pentanol $$ 2-Pentanol, 4-methoxy-4-methyl- $$

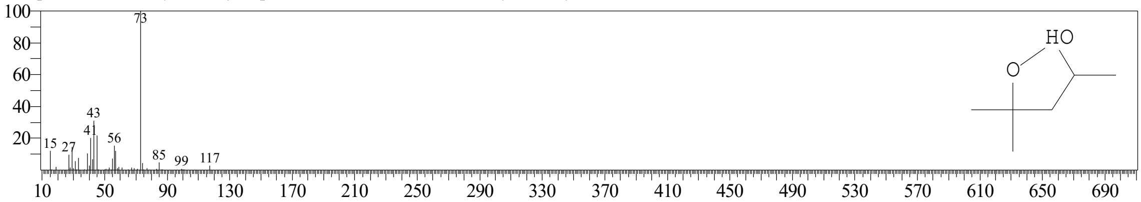

Hit#:4 Entry:72975 Library:NIST11.lib
SI:74 Formula:C15H31NO CAS:36475-50-0 MolWeight:241 RetIndex:1804
CompName:Tetradecanal, O-methyloxime $$ (1E)-Tetradecanal o-methyloxime # $$

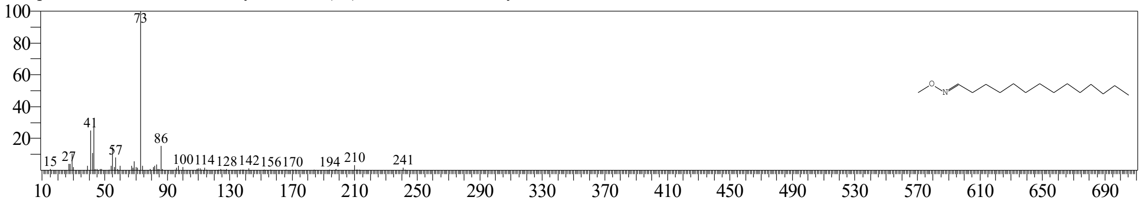

Hit#:5 Entry:34486 Library:NIST11.lib
SI:74 Formula:C11H23NO CAS:35473-94-0 MolWeight:185 RetIndex:1406
CompName:Decanal, O-methyloxime $$ (1E)-Decanal o-methyloxime # $$

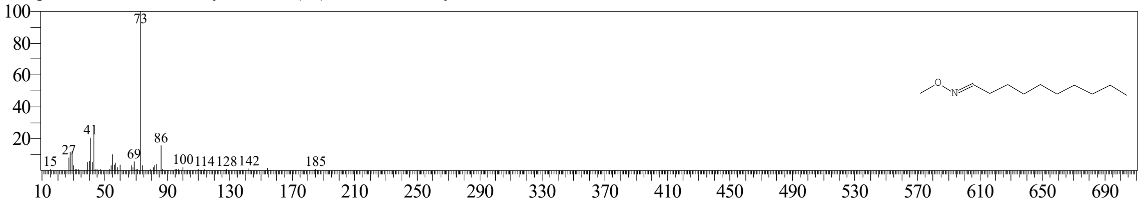

<< Target >>
Line#:25 R.Time:7.290(Scan#:659) MassPeaks:362
RawMode:Averaged 7.285-7.295(658-660) BasePeak:73.05(23735)
BG Mode:Calc. from Peak Group 1 - Event 1 Scan

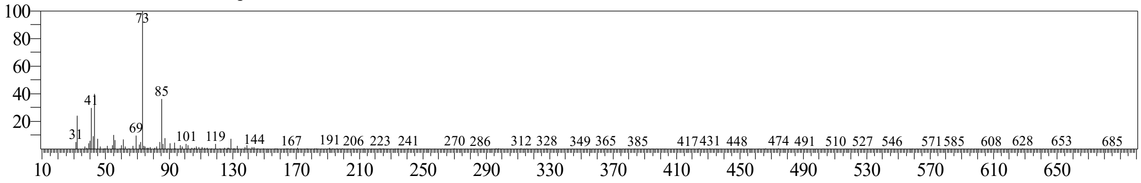

Hit#:1 Entry:36153 Library:NIST11.lib
SI:77 Formula:C10H20O3 CAS:0-00-0 MolWeight:188 RetIndex:1174
CompName:3-Methylpentan-3-yl propyl carbonate

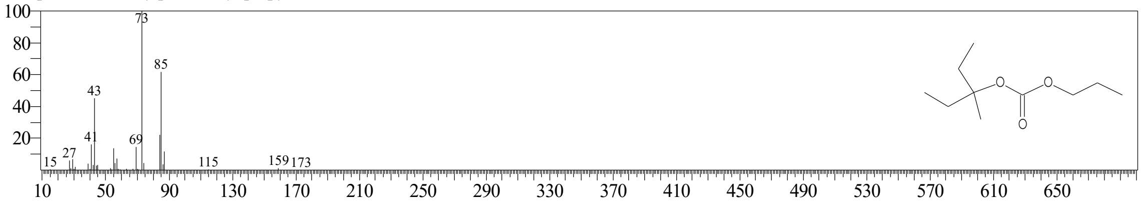

Hit#:2 Entry:13303 Library:NIST11.lib
SI:76 Formula:C7H15NO2 CAS:78-28-4 MolWeight:145 RetIndex:1043
CompName:Emylcamate $$ 3-Pentanol, 3-methyl-, carbamate $$ tert-Hexanol carbamate $$ Diethyl methyl carbinol urethan $$ Emylcamat $$ KABI-925 $$ Met

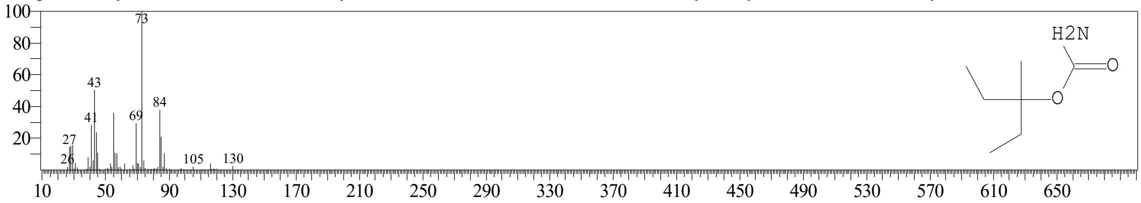

Hit#:3 Entry:14985 Library:NIST11.lib
SI:74 Formula:C5H10O5 CAS:1949-78-6 MolWeight:150 RetIndex:1436
CompName:L-Lyxose $$ Pentose # $$

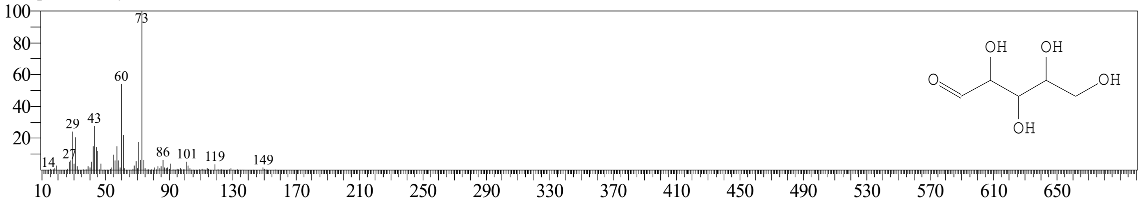

Hit#:4 Entry:28105 Library:NIST11.lib
SI:74 Formula:C10H22O2 CAS:54484-73-0 MolWeight:174 RetIndex:1103
CompName:Hexane, 1-(1-ethoxyethoxy)- $$ Acetaldehyde ethyl hexyl acetal $$ 1-(1-Ethoxyethoxy)hexane $$ 1-Ethoxy-1-hexyloxyethane $$ Ethyl hexyl acetal

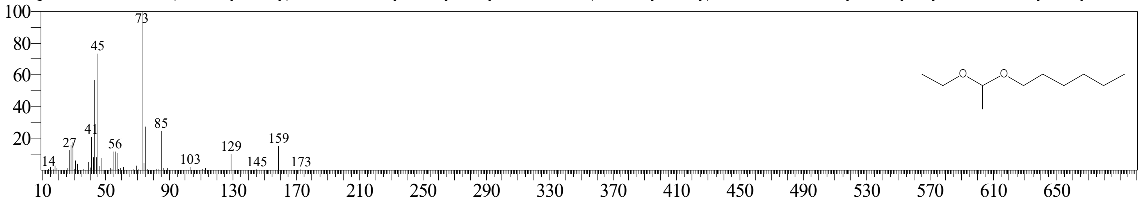

Hit#:5 Entry:12985 Library:NIST11.lib
SI:74 Formula:C8H16O2 CAS:815-77-0 MolWeight:144 RetIndex:987
CompName:2,5-Dimethyl-4-hydroxy-3-hexanone $$ 4-Hydroxy-2,5-dimethyl-3-hexanone # $$

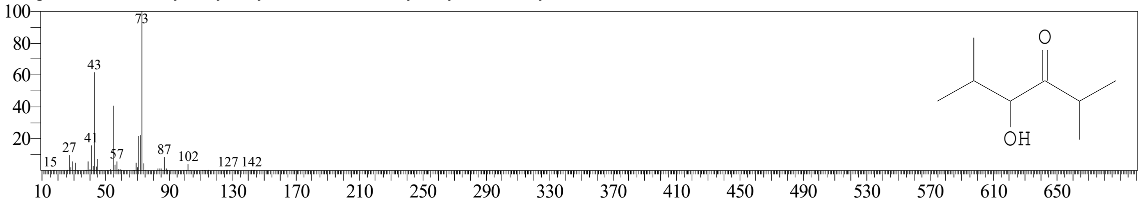

<< Target >>
Line#:26 R.Time:7.515(Scan#:704) MassPeaks:353
RawMode:Averaged 7.510-7.520(703-705) BasePeak:43.00(24987)
BG Mode:Calc. from Peak Group 1 - Event 1 Scan

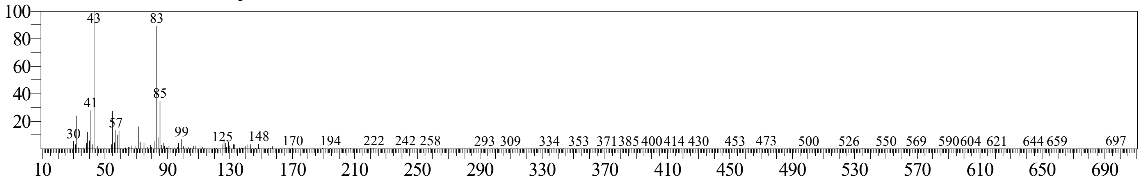

Hit#:1 Entry:27105 Library:NIST11.lib
SI:76 Formula:C10H20O2 CAS:0-00-0 MolWeight:172 RetIndex:1317
CompName:1,1-Hexylenedioxybutane $$ 2-Propyl-1,3-dioxonane # $$

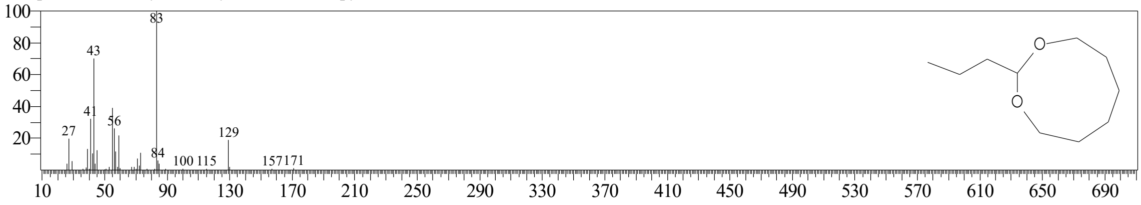

Hit#:2 Entry:89993 Library:NIST11.lib
SI:75 Formula:C13H28O3S CAS:0-00-0 MolWeight:264 RetIndex:1873
CompName:Sulfurous acid, decyl 2-propyl ester

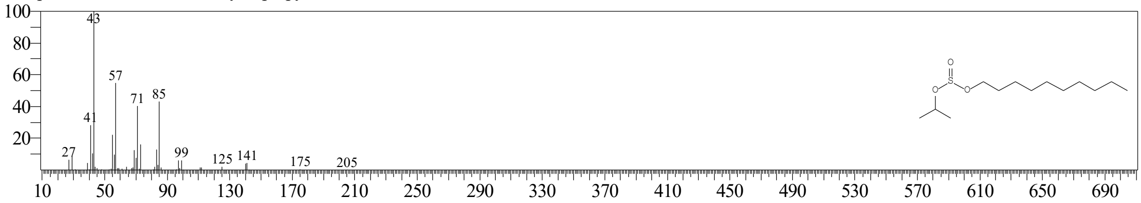

Hit#:3 Entry:4656 Library:NIST11.lib
SI:74 Formula:C7H16O CAS:111768-02-6 MolWeight:116 RetIndex:832
CompName:1-Pentanol, 2,4-dimethyl-, (.+/-.)- $$ 2,4-Dimethyl-1-pentanol $$

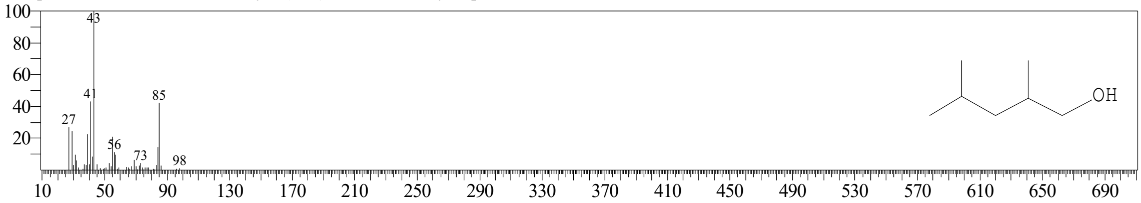

Hit#:4 Entry:43829 Library:NIST11.lib
SI:74 Formula:C11H20O3 CAS:0-00-0 MolWeight:200 RetIndex:1199
CompName:Acetic acid, (3-allyloxy-1,1-dimethylbutyl) ester $$ 3-(Allyloxy)-1,1-dimethylbutyl acetate # $$

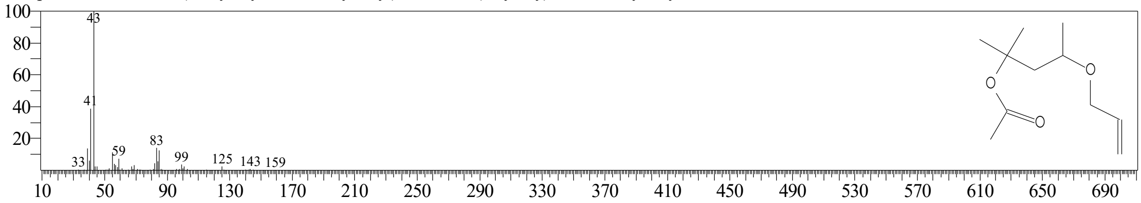

Hit#:5 Entry:4701 Library:NIST11.lib
SI:74 Formula:C7H16O CAS:2370-12-9 MolWeight:116 RetIndex:875
CompName:1-Pentanol, 2,2-dimethyl- $$ Neoheptanol $$ 2,2-Dimethyl-1-pentanol $$ 2,2-Dimethylpentanol $$

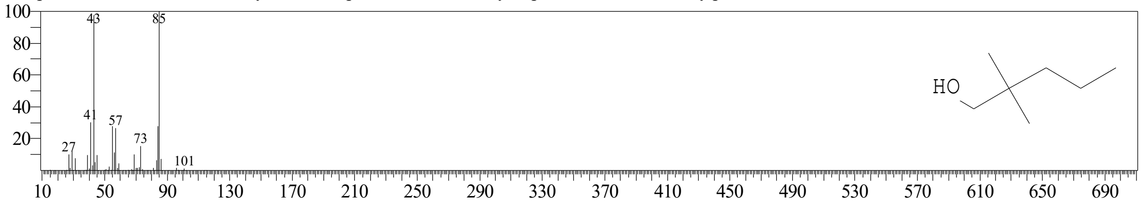

<< Target >>
Line#:27 R.Time:7.700(Scan#:741) MassPeaks:357
RawMode:Averaged 7.695-7.705(740-742) BasePeak:83.10(53858)
BG Mode:Calc. from Peak Group 1 - Event 1 Scan

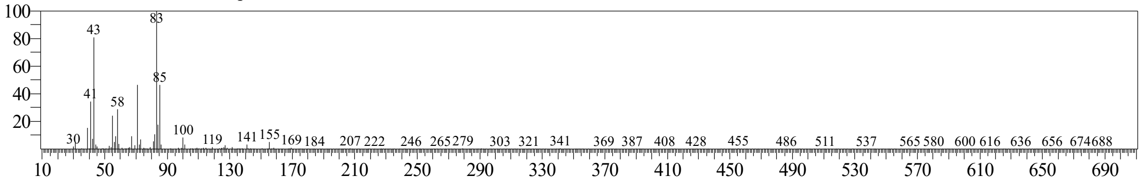

Hit#:1 Entry:13161 Library:NIST11.lib
SI:79 Formula:C9H20O CAS:19549-73-6 MolWeight:144 RetIndex:950
CompName:3-Heptanol, 2,6-dimethyl- $$ 2,6-Dimethyl-3-heptanol $$

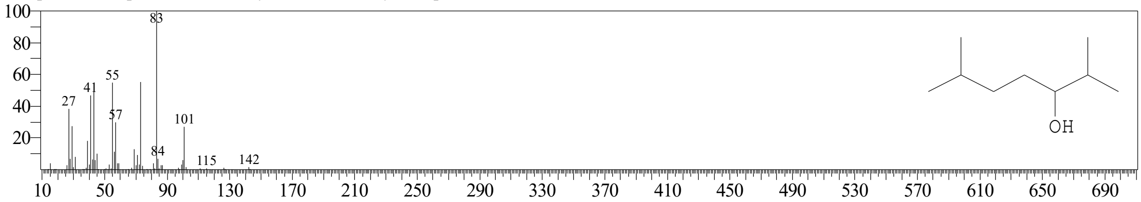

Hit#:2 Entry:27105 Library:NIST11.lib
SI:79 Formula:C10H20O2 CAS:0-00-0 MolWeight:172 RetIndex:1317
CompName:1,1-Hexylenedioxybutane $$ 2-Propyl-1,3-dioxonane # $$

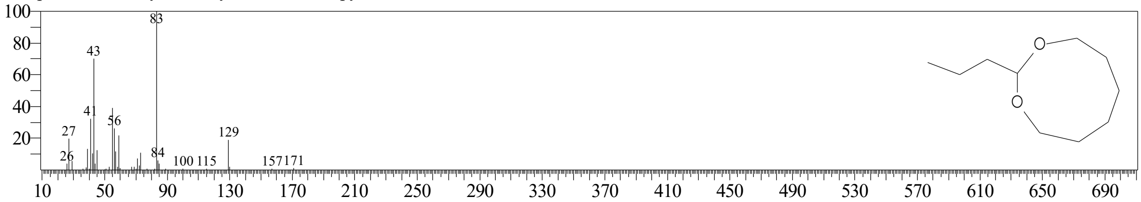

Hit#:3 Entry:26096 Library:NIST11.lib
SI:79 Formula:C12H26 CAS:1636-44-8 MolWeight:170 RetIndex:1150
CompName:Decane, 4-ethyl- $$ 4-Ethyldecane # $$

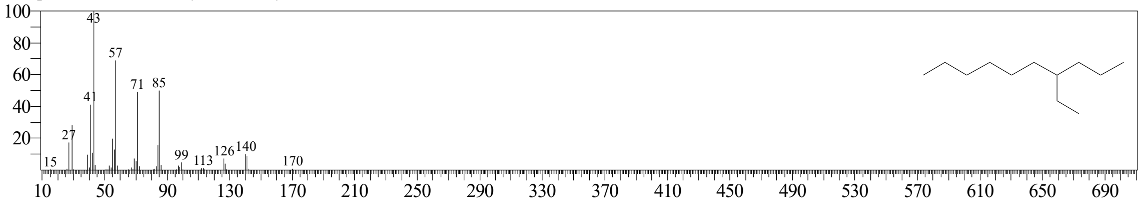

Hit#:4 Entry:27227 Library:NIST11.lib
SI:79 Formula:C11H24O CAS:213547-15-0 MolWeight:172 RetIndex:1213
CompName:4-Methyl-5-decanol

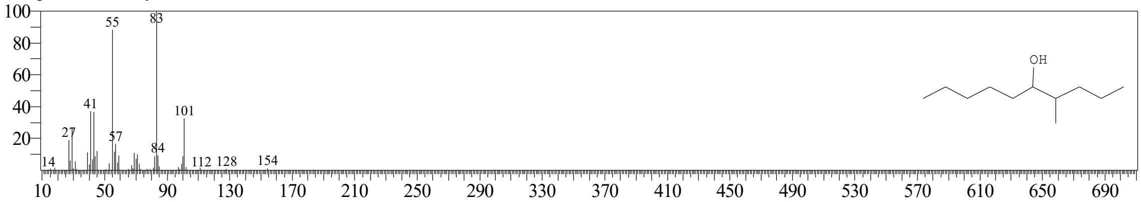

Hit#:5 Entry:89993 Library:NIST11.lib
SI:78 Formula:C13H28O3S CAS:0-00-0 MolWeight:264 RetIndex:1873
CompName:Sulfurous acid, decyl 2-propyl ester

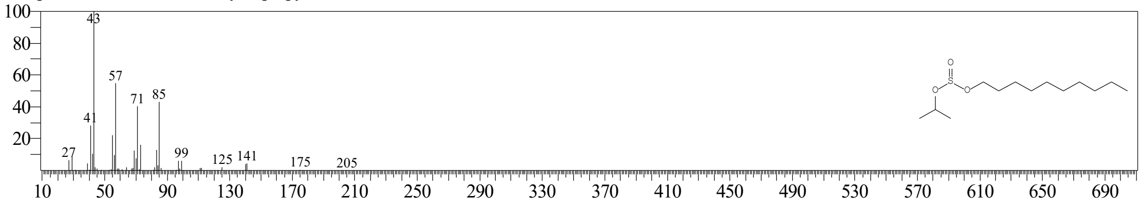

<< Target >>
Line#:28 R.Time:7.760(Scan#:753) MassPeaks:354
RawMode:Averaged 7.755-7.765(752-754) BasePeak:43.05(29065)
BG Mode:Calc. from Peak Group 1 - Event 1 Scan

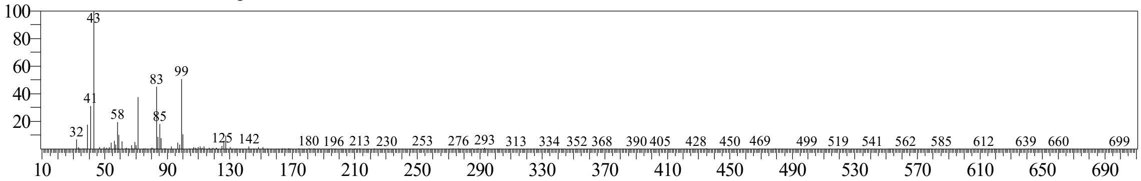

Hit#:1 Entry:33872 Library:NIST11.lib
SI:77 Formula:C11H20O2 CAS:0-00-0 MolWeight:184 RetIndex:1238
CompName:6,6,7-Trimethyl-octane-2,5-dione $$ 6,6,7-Trimethyl-2,5-octanedione # $$

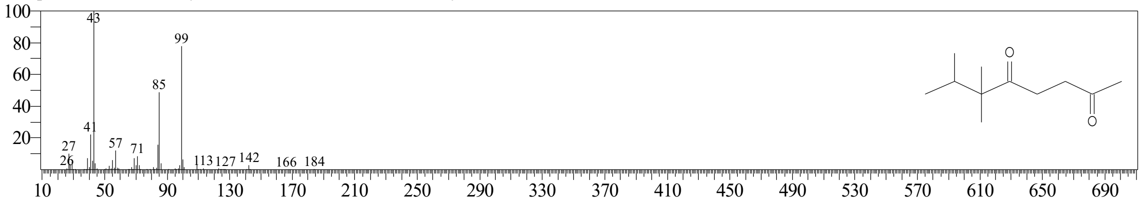

Hit#:2 Entry:12227 Library:NIST11.lib
SI:76 Formula:C9H18O CAS:4485-09-0 MolWeight:142 RetIndex:1052
CompName:4-Nonanone $$ Propyl amyl ketone $$ Nonan-4-one $$

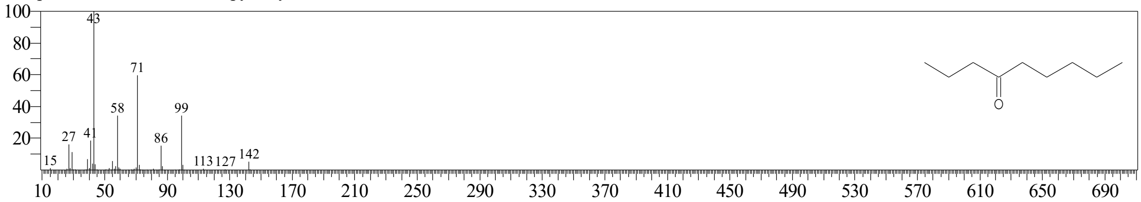

Hit#:3 Entry:12033 Library:NIST11.lib
SI:75 Formula:C8H14O2 CAS:3050-69-9 MolWeight:142 RetIndex:974
CompName:n-Caproic acid vinyl ester $$ Hexanoic acid, ethenyl ester $$ Caproic acid, vinyl ester $$ Hexanoic acid, vinyl ester $$ Vinyl caproate $$ Vinyl hexa

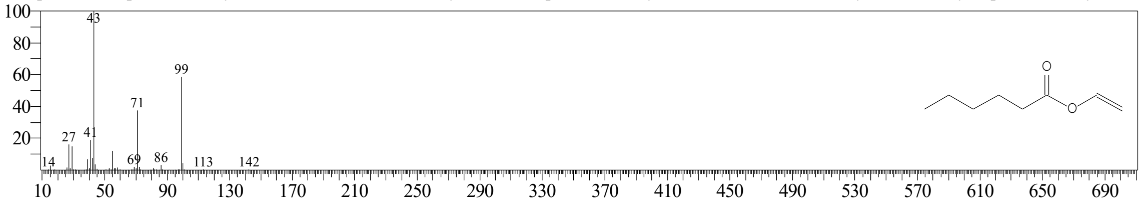

Hit#:4 Entry:19369 Library:NIST11.lib
SI:75 Formula:C9H18O2 CAS:51149-70-3 MolWeight:158 RetIndex:1128
CompName:2-Heptanone, 1-ethoxy- $$ 1-Ethoxy-2-heptanone # $$ 1-Ethoxy-heptan-2-one $$

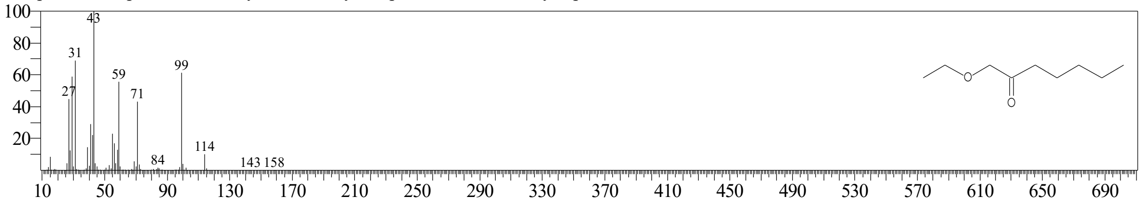

Hit#:5 Entry:26036 Library:NIST11.lib
SI:75 Formula:C11H22O CAS:927-49-1 MolWeight:170 RetIndex:1251
CompName:6-Undecanone $$ Amyl ketone $$ Diamyl ketone $$ Dipentyl ketone $$ Pentyl ketone $$ Undecan-6-one $$ Undecanone-(6) $$ 6-Oxoundecane $$

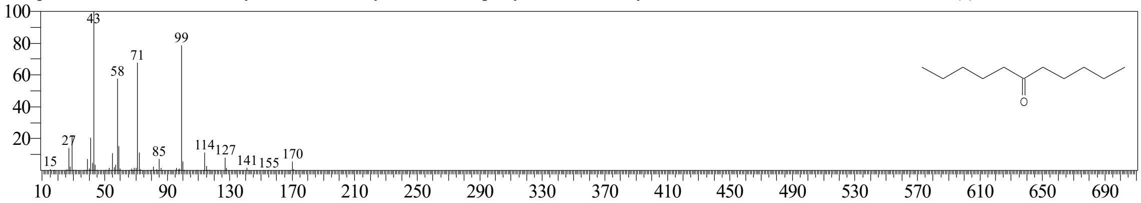

<< Target >>
Line#:29 R.Time:7.815(Scan#:764) MassPeaks:378
RawMode:Averaged 7.810-7.820(763-765) BasePeak:43.05(27197)
BG Mode:Calc. from Peak Group 1 - Event 1 Scan

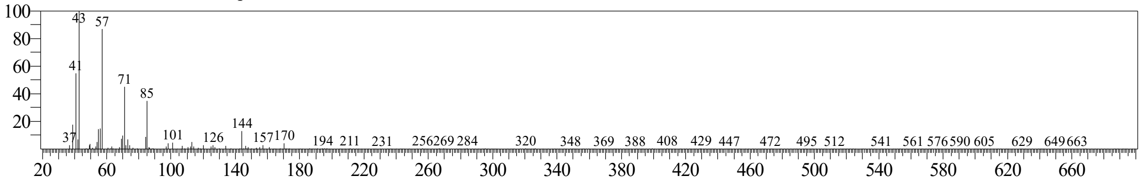

Hit#:1 Entry:34134 Library:NIST11.lib
SI:87 Formula:C13H28 CAS:17301-29-0 MolWeight:184 RetIndex:1185
CompName:Undecane, 3,7-dimethyl- $$ 3,7-Dimethylundecane # $$

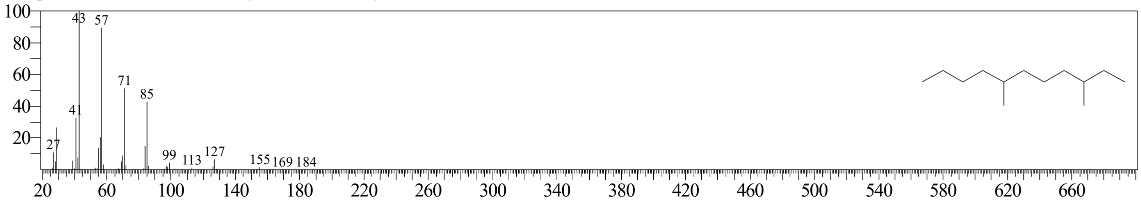

Hit#:2 Entry:18707 Library:NIST11.lib
SI:87 Formula:C11H24 CAS:62016-30-2 MolWeight:156 RetIndex:966
CompName:Octane, 2,3,3-trimethyl- $$ 2,3,3-Trimethyloctane # $$

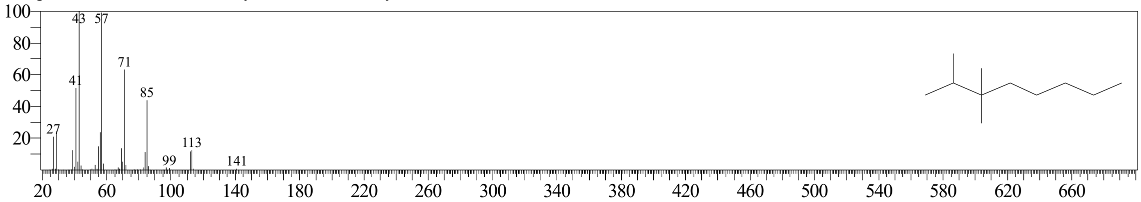

Hit#:3 Entry:18708 Library:NIST11.lib
SI:86 Formula:C11H24 CAS:62016-18-6 MolWeight:156 RetIndex:986
CompName:Octane, 5-ethyl-2-methyl- $$ 5-Ethyl-2-methyloctane # $$

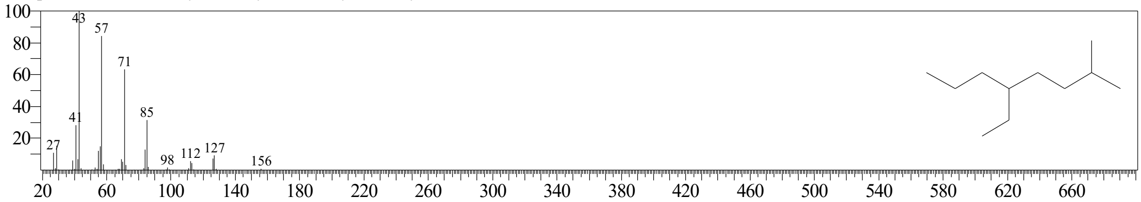

Hit#:4 Entry:26091 Library:NIST11.lib
SI:86 Formula:C12H26 CAS:13150-81-7 MolWeight:170 RetIndex:1086
CompName:2,6-Dimethyldecane

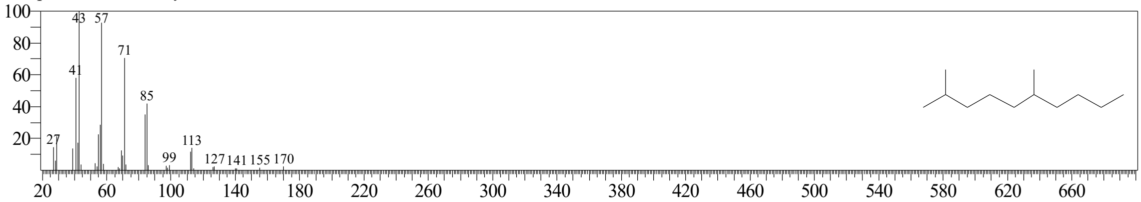

Hit#:5 Entry:26110 Library:NIST11.lib
SI:86 Formula:C12H26 CAS:62185-21-1 MolWeight:170 RetIndex:958
CompName:Octane, 3,4,5,6-tetramethyl- $$ 3,4,5,6-Tetramethyloctane # $$

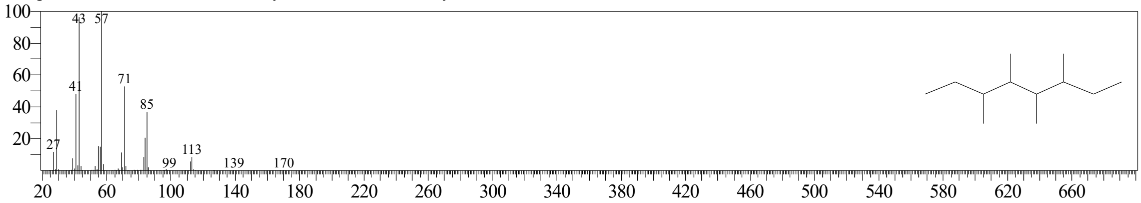

<< Target >>
Line#:30 R.Time:7.910(Scan#:783) MassPeaks:378
RawMode:Averaged 7.905-7.915(782-784) BasePeak:43.05(32748)
BG Mode:Calc. from Peak Group 1 - Event 1 Scan

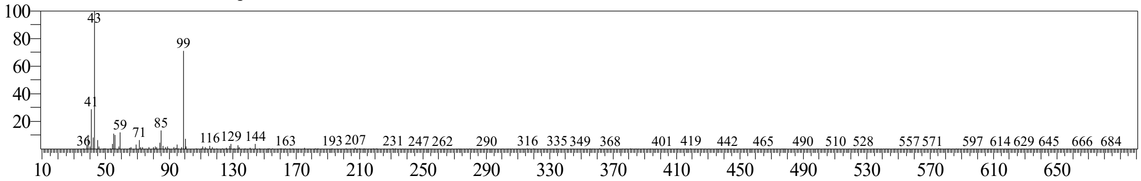

Hit#:1 Entry:33872 Library:NIST11.lib
SI:82 Formula:C11H20O2 CAS:0-00-0 MolWeight:184 RetIndex:1238
CompName:6,6,7-Trimethyl-octane-2,5-dione $$ 6,6,7-Trimethyl-2,5-octanedione # $$

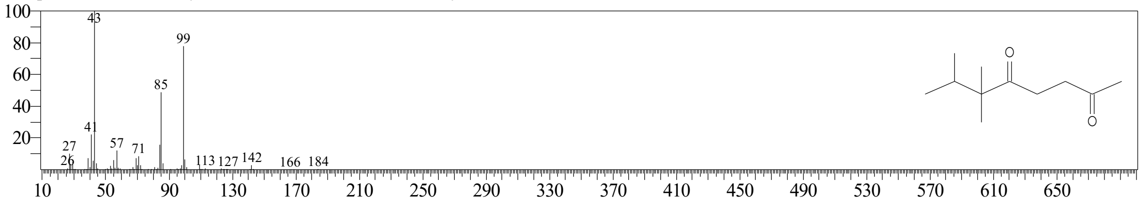

Hit#:2 Entry:12033 Library:NIST11.lib
SI:81 Formula:C8H14O2 CAS:3050-69-9 MolWeight:142 RetIndex:974
CompName:n-Caproic acid vinyl ester $$ Hexanoic acid, ethenyl ester $$ Caproic acid, vinyl ester $$ Hexanoic acid, vinyl ester $$ Vinyl caproate $$ Vinyl hexa

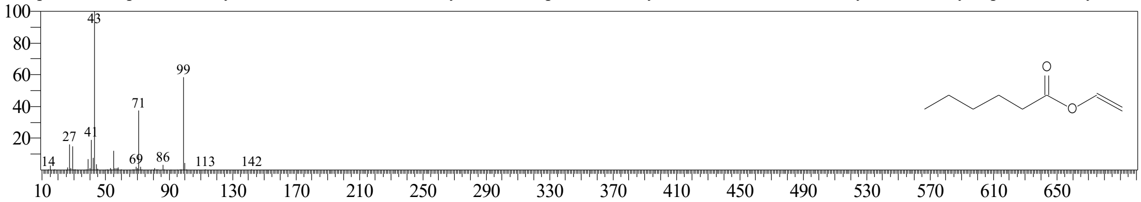

Hit#:3 Entry:4482 Library:NIST11.lib
SI:80 Formula:C5H8O3 CAS:28525-83-9 MolWeight:116 RetIndex:1045
CompName:2-Pentenoic acid, 4-hydroxy- $$ (2E)-4-Hydroxy-2-pentenoic acid # $$

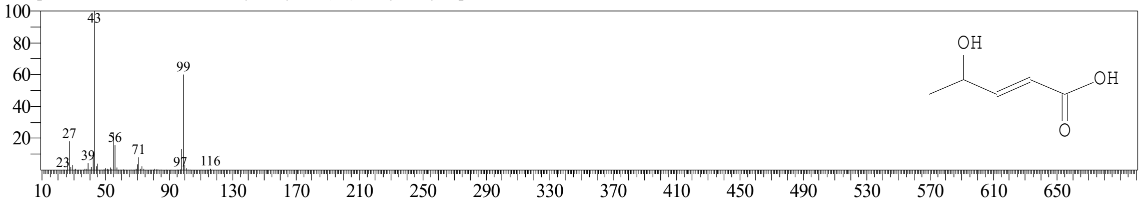

Hit#:4 Entry:4720 Library:NIST11.lib
SI:80 Formula:C3H7N3O2 CAS:352-97-6 MolWeight:117 RetIndex:1076
CompName:Guanidineacetic acid $$ Glycocyamine $$ Guanidinoacetic acid $$ Glucocyamine $$ N-Amidinoglycine $$ Glycine, N-(aminoiminomethyl)- $$ .alp

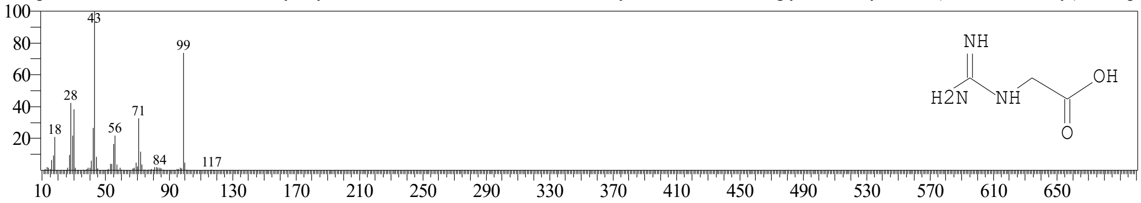

Hit#:5 Entry:19369 Library:NIST11.lib
SI:80 Formula:C9H18O2 CAS:51149-70-3 MolWeight:158 RetIndex:1128
CompName:2-Heptanone, 1-ethoxy- $$ 1-Ethoxy-2-heptanone # $$ 1-Ethoxy-heptan-2-one $$

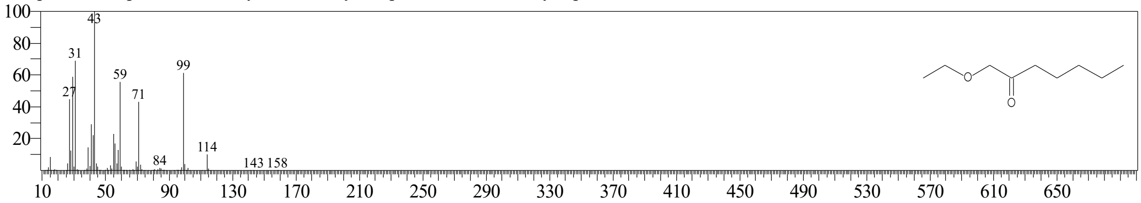

<< Target >>
Line#:31 R.Time:7.950(Scan#:791) MassPeaks:349
RawMode:Averaged 7.945-7.955(790-792) BasePeak:99.15(32189)
BG Mode:Calc. from Peak Group 1 - Event 1 Scan

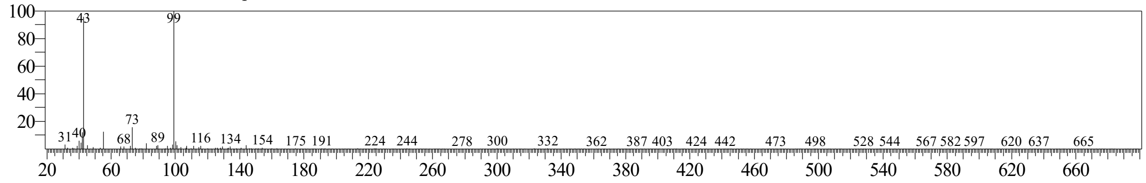

Hit#:1 Entry:19146 Library:NIST11.lib
SI:79 Formula:C7H10O4 CAS:57681-51-3 MolWeight:158 RetIndex:1226
CompName:2(3H)-Furanone, 5-(acetyloxy)dihydro-5-methyl- $$ 2-Methyl-5-oxotetrahydro-2-furanyl acetate # $$

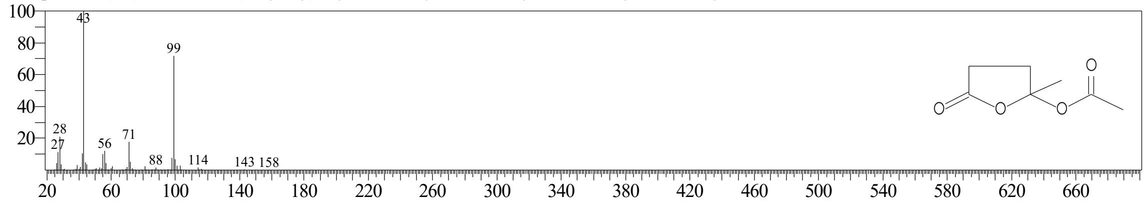

Hit#:2 Entry:11476 Library:NIST11.lib
SI:79 Formula:C4H7N5O CAS:0-00-0 MolWeight:141 RetIndex:1454
CompName:1H-1,2,4-Triazole-3,5-diamine, 1-acetyl-

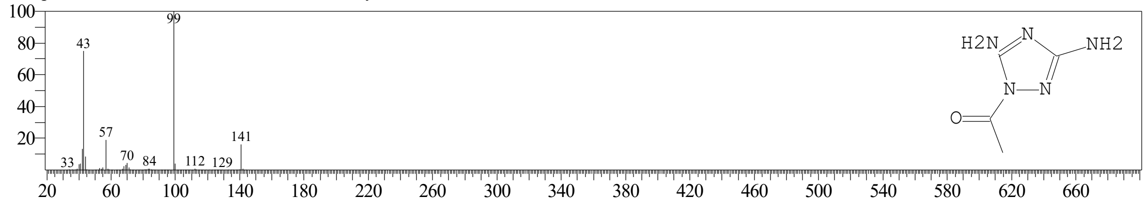

Hit#:3 Entry:4482 Library:NIST11.lib
SI:78 Formula:C5H8O3 CAS:28525-83-9 MolWeight:116 RetIndex:1045
CompName:2-Pentenoic acid, 4-hydroxy- $$ (2E)-4-Hydroxy-2-pentenoic acid # $$

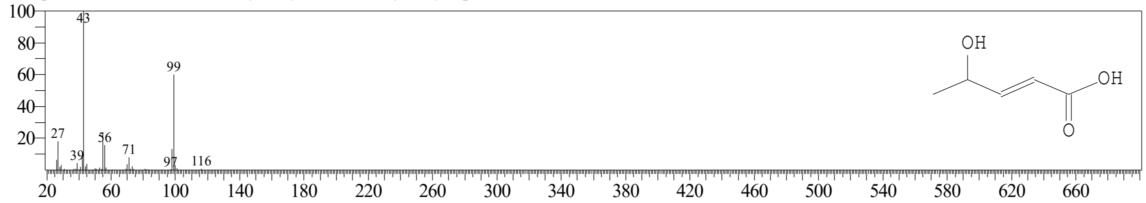

Hit#:4 Entry:1770 Library:NIST11.lib
SI:77 Formula:C2H5N5 CAS:38104-45-9 MolWeight:99 RetIndex:1130
CompName:3,4-Diamino-1,2,4(4H)-triazole $$ 3,4-Diamino-1,2,4-triazole $$ 4H-1,2,4-Triazole-3,4-diamine # $$

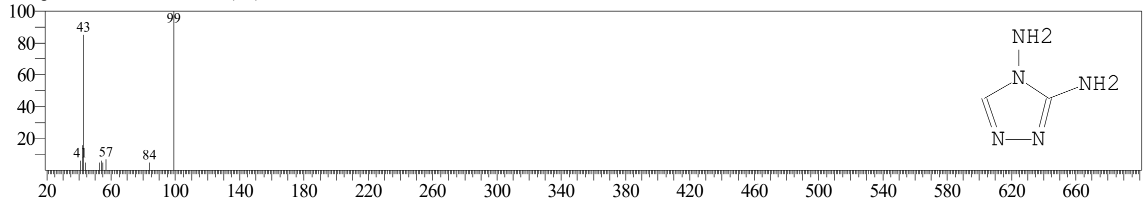

Hit#:5 Entry:25949 Library:NIST11.lib
SI:76 Formula:C10H18O2 CAS:0-00-0 MolWeight:170 RetIndex:1214
CompName:2,5-Dimethyl-2-(2-tetrahydrofuryl)tetrahydrofuran

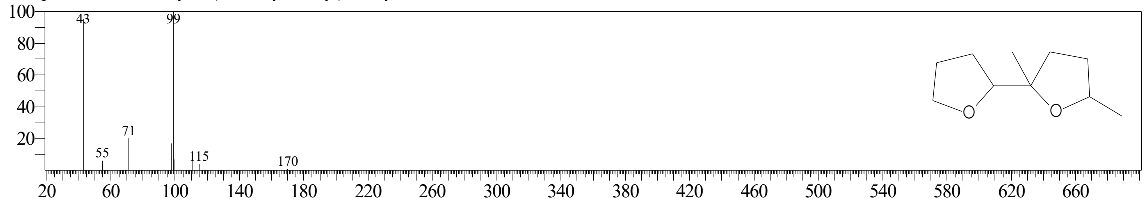

<< Target >>
Line#:32 R.Time:8.020(Scan#:805) MassPeaks:375
RawMode:Averaged 8.015-8.025(804-806) BasePeak:83.05(13428)
BG Mode:Calc. from Peak Group 1 - Event 1 Scan

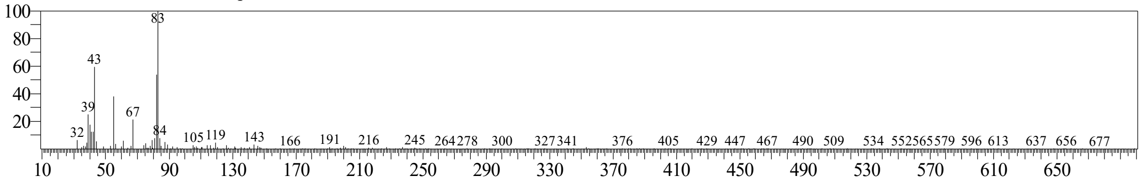

Hit#:1 Entry:6620 Library:NIST11.lib
SI:76 Formula:C8H14O CAS:13388-93-7 MolWeight:126 RetIndex:970
CompName:Ethanone, 1-(1-methylcyclopentyl)- $$ 1-(1-Methyl-cyclopentyl)-ethanone $$

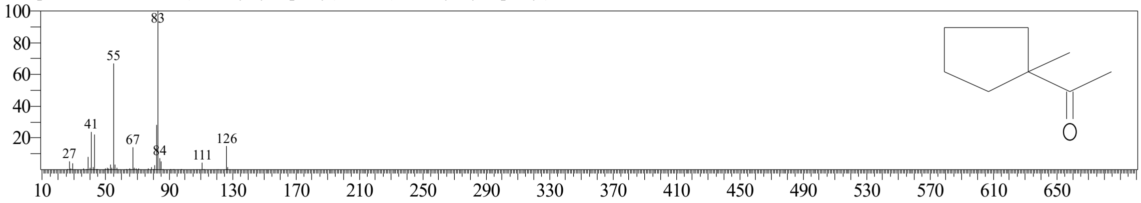

Hit#:2 Entry:6038 Library:NIST11.lib
SI:75 Formula:C9H16 CAS:2114-42-3 MolWeight:124 RetIndex:969
CompName:Cyclohexane, 2-propenyl- $$ Cyclohexane, allyl- $$ Allylcyclohexane $$ 1-Cyclohexyl-2-propene $$ 1-Propene, 3-cyclohexyl- $$ 3-Cyclohexyl-1-

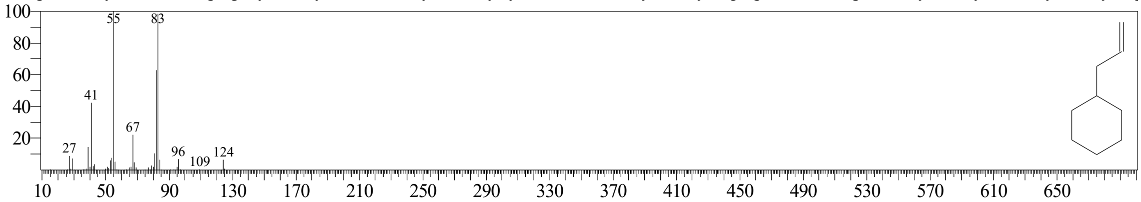

Hit#:3 Entry:6713 Library:NIST11.lib
SI:74 Formula:C9H18 CAS:696-29-7 MolWeight:126 RetIndex:915
CompName:Cyclohexane, (1-methylethyl)- $$ Cyclohexane, isopropyl- $$ Isopropylcyclohexane $$ Hexahydrocumene $$ Normanthane $$

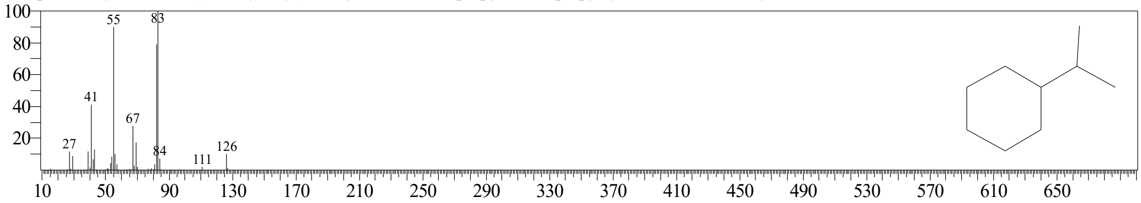

Hit#:4 Entry:24951 Library:NIST11.lib
SI:74 Formula:C12H24 CAS:4292-75-5 MolWeight:168 RetIndex:1278
CompName:Cyclohexane, hexyl- $$ Hexane, 1-cyclohexyl- $$ Cyclohexane, n-hexyl- $$ Hexylcyclohexane $$ 1-Cyclohexylhexane $$ 1-Hexylcyclohexane $$

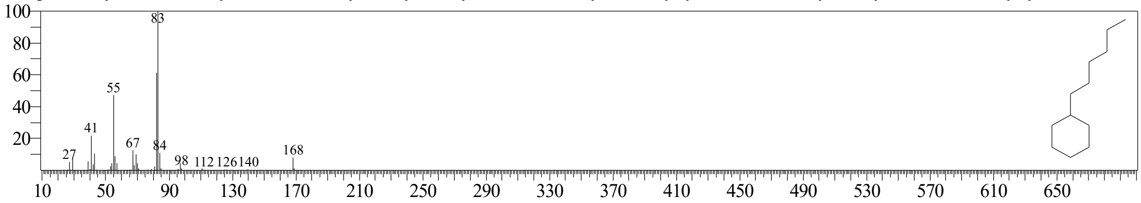

Hit#:5 Entry:11455 Library:NIST11.lib
SI:74 Formula:C10H20 CAS:1678-93-9 MolWeight:140 RetIndex:1079
CompName:Cyclohexane, butyl- $$ n-Butylcyclohexane $$ Butane, 1-cyclohexyl- $$ Butylcyclohexane $$ 1-Cyclohexylbutane $$ Cyclohexane, n-butyl- $$

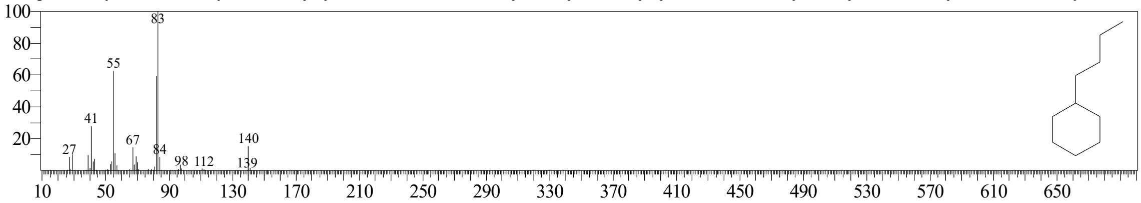

<< Target >>
Line#:33 R.Time:8.145(Scan#:830) MassPeaks:369
RawMode:Averaged 8.140-8.150(829-831) BasePeak:43.05(39985)
BG Mode:Calc. from Peak Group 1 - Event 1 Scan

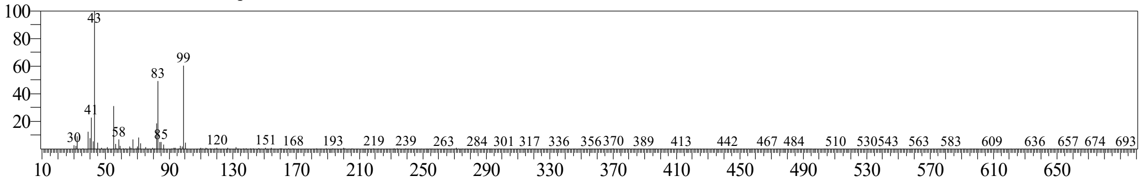

Hit#:1 Entry:12033 Library:NIST11.lib
SI:81 Formula:C8H14O2 CAS:3050-69-9 MolWeight:142 RetIndex:974
CompName:n-Caproic acid vinyl ester $$ Hexanoic acid, ethenyl ester $$ Caproic acid, vinyl ester $$ Hexanoic acid, vinyl ester $$ Vinyl caproate $$ Vinyl hexa

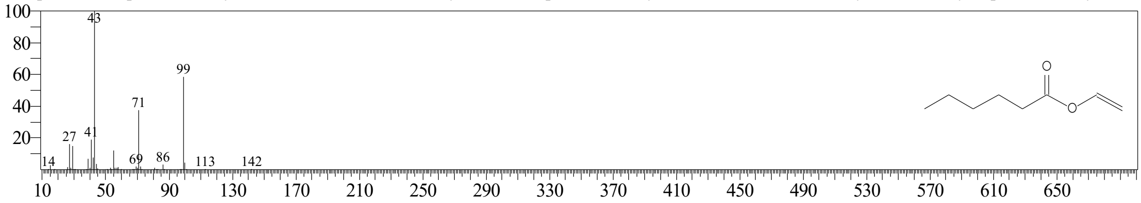

Hit#:2 Entry:32924 Library:NIST11.lib
SI:79 Formula:C12H22O CAS:0-00-0 MolWeight:182 RetIndex:1359
CompName:2-Cyclohexyl-hex-5-en-2-ol $$ 2-Cyclohexyl-5-hexen-2-ol # $$

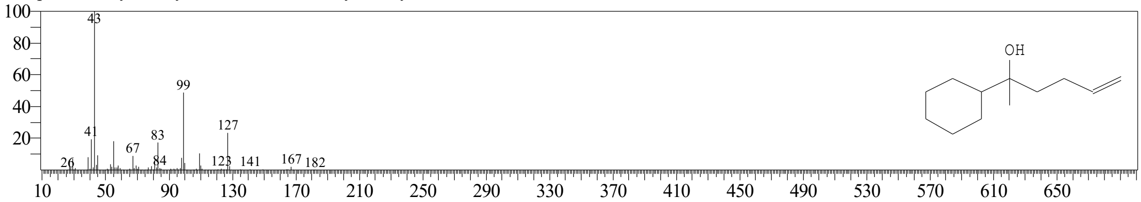

Hit#:3 Entry:33872 Library:NIST11.lib
SI:79 Formula:C11H20O2 CAS:0-00-0 MolWeight:184 RetIndex:1238
CompName:6,6,7-Trimethyl-octane-2,5-dione $$ 6,6,7-Trimethyl-2,5-octanedione # $$

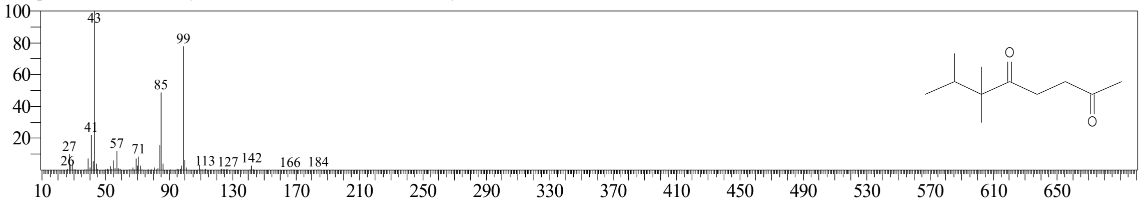

Hit#:4 Entry:4482 Library:NIST11.lib
SI:79 Formula:C5H8O3 CAS:28525-83-9 MolWeight:116 RetIndex:1045
CompName:2-Pentenoic acid, 4-hydroxy- $$ (2E)-4-Hydroxy-2-pentenoic acid # $$

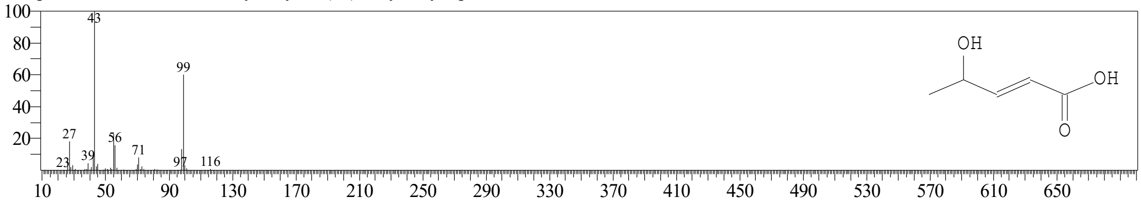

Hit#:5 Entry:4534 Library:NIST11.lib
SI:77 Formula:C6H12O2 CAS:7319-23-5 MolWeight:116 RetIndex:950
CompName:3-Hexene-2,5-diol $$ Hex-3-ene-2,5-diol $$ (3E)-3-Hexene-2,5-diol # $$

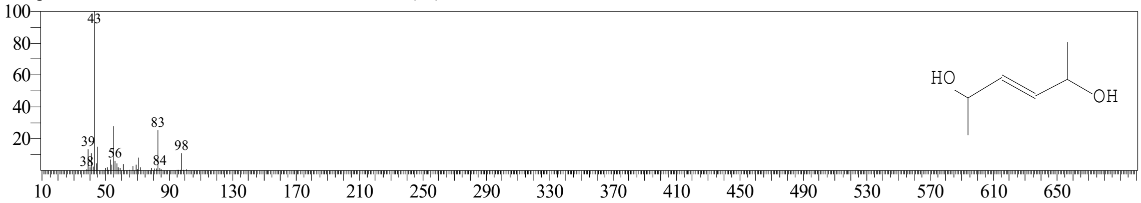

<< Target >>
Line#:34 R.Time:8.490(Scan#:899) MassPeaks:353
RawMode:Averaged 8.485-8.495(898-900) BasePeak:43.05(152618)
BG Mode:Calc. from Peak Group 1 - Event 1 Scan

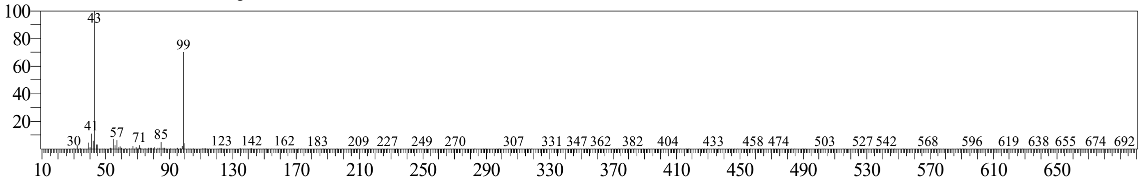

Hit#:1 Entry:12033 Library:NIST11.lib
SI:89 Formula:C8H14O2 CAS:3050-69-9 MolWeight:142 RetIndex:974
CompName:n-Caproic acid vinyl ester $$ Hexanoic acid, ethenyl ester $$ Caproic acid, vinyl ester $$ Hexanoic acid, vinyl ester $$ Vinyl caproate $$ Vinyl hexa

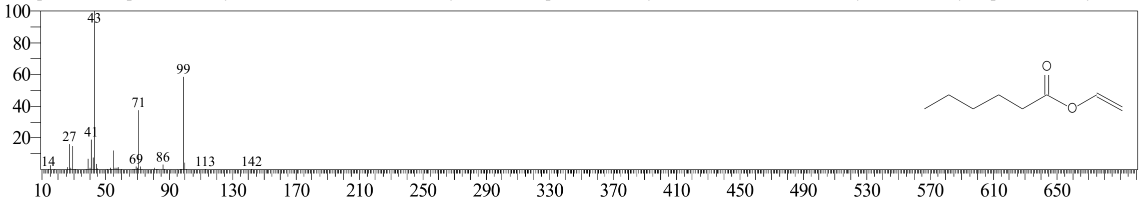

Hit#:2 Entry:33872 Library:NIST11.lib
SI:87 Formula:C11H20O2 CAS:0-00-0 MolWeight:184 RetIndex:1238
CompName:6,6,7-Trimethyl-octane-2,5-dione $$ 6,6,7-Trimethyl-2,5-octanedione # $$

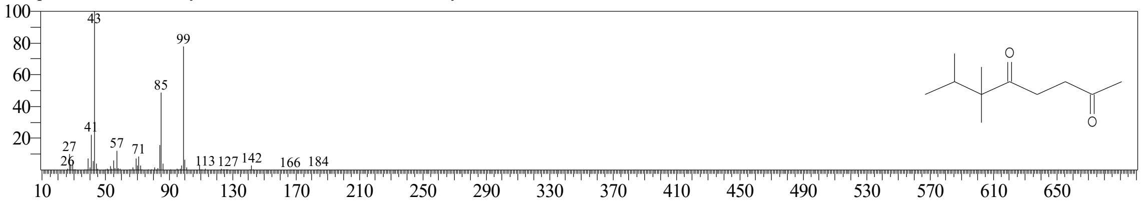

Hit#:3 Entry:11254 Library:NIST11.lib
SI:86 Formula:C9H16O CAS:80192-56-9 MolWeight:140 RetIndex:1070
CompName:2,6-Heptadien-1-ol, 2,4-dimethyl- $$ (2E)-2,4-Dimethyl-2,6-heptadien-1-ol # $$

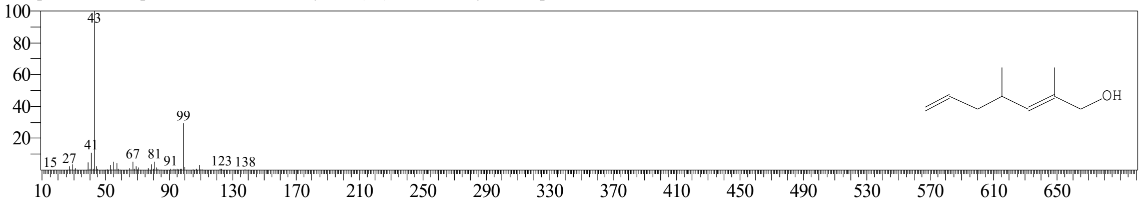

Hit#:4 Entry:4482 Library:NIST11.lib
SI:86 Formula:C5H8O3 CAS:28525-83-9 MolWeight:116 RetIndex:1045
CompName:2-Pentenoic acid, 4-hydroxy- $$ (2E)-4-Hydroxy-2-pentenoic acid # $$

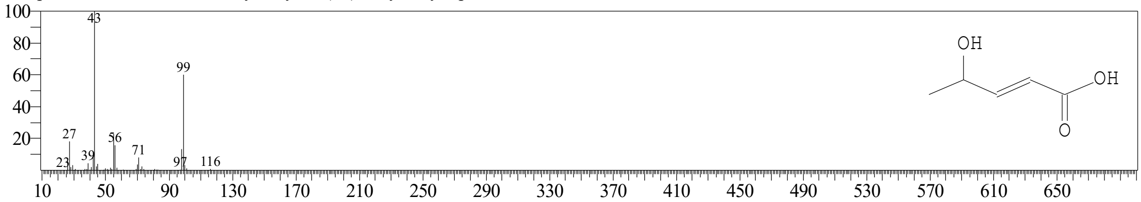

Hit#:5 Entry:19146 Library:NIST11.lib
SI:86 Formula:C7H10O4 CAS:57681-51-3 MolWeight:158 RetIndex:1226
CompName:2(3H)-Furanone, 5-(acetyloxy)dihydro-5-methyl- $$ 2-Methyl-5-oxotetrahydro-2-furanyl acetate # $$

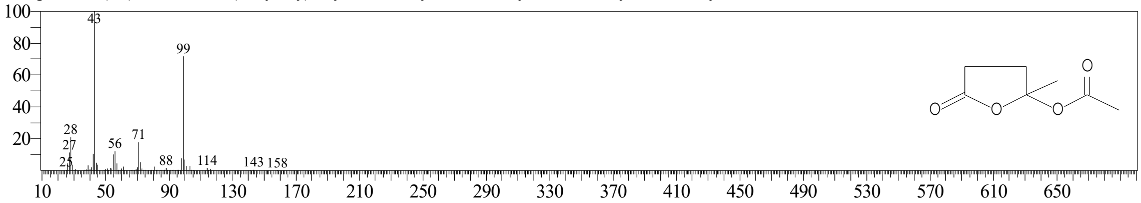

<< Target >>
Line#:35 R.Time:8.660(Scan#:933) MassPeaks:391
RawMode:Averaged 8.655-8.665(932-934) BasePeak:43.05(90400)
BG Mode:Calc. from Peak Group 1 - Event 1 Scan

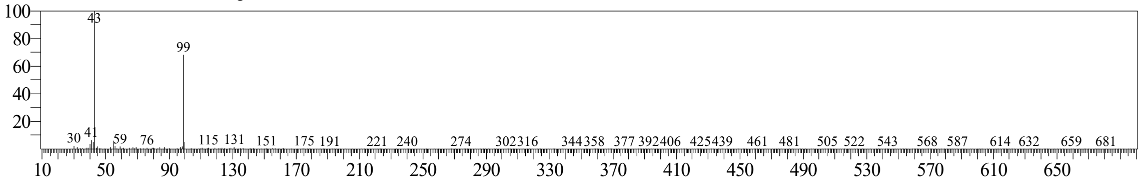

Hit#:1 Entry:4482 Library:NIST11.lib
SI:84 Formula:C5H8O3 CAS:28525-83-9 MolWeight:116 RetIndex:1045
CompName:2-Pentenoic acid, 4-hydroxy- $$ (2E)-4-Hydroxy-2-pentenoic acid # $$

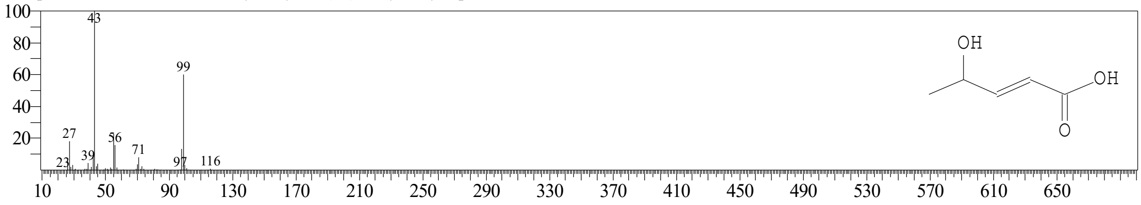

Hit#:2 Entry:19146 Library:NIST11.lib
SI:84 Formula:C7H10O4 CAS:57681-51-3 MolWeight:158 RetIndex:1226
CompName:2(3H)-Furanone, 5-(acetyloxy)dihydro-5-methyl- $$ 2-Methyl-5-oxotetrahydro-2-furanyl acetate # $$

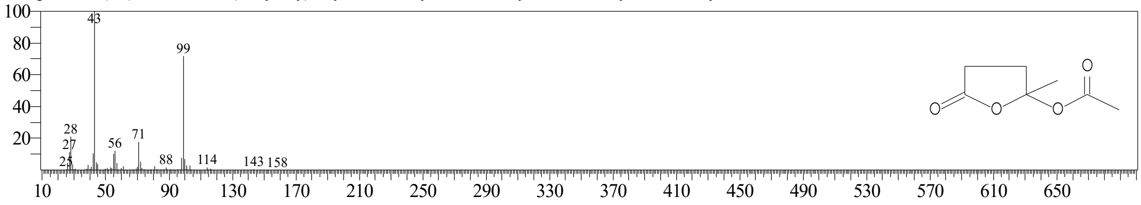

Hit#:3 Entry:29728 Library:NIST11.lib
SI:83 Formula:C5H7BrO2 CAS:63915-88-8 MolWeight:178 RetIndex:949
CompName:2-Propen-1-ol, 2-bromo-, acetate $$ 2-Bromo-2-propen-1-ol acetate $$ 2-Bromo-2-propenyl acetate # $$

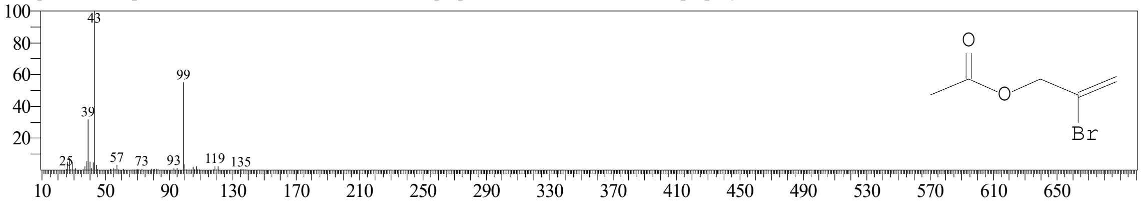

Hit#:4 Entry:11084 Library:NIST11.lib
SI:83 Formula:C8H12O2 CAS:0-00-0 MolWeight:140 RetIndex:900
CompName:1,5-Hexadien-3-ol, acetate

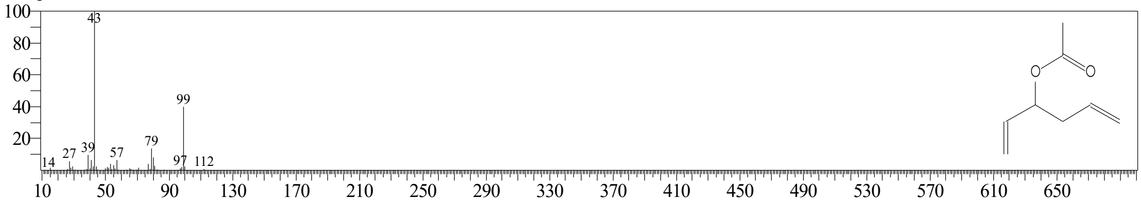

Hit#:5 Entry:12034 Library:NIST11.lib
SI:83 Formula:C8H14O2 CAS:90112-64-4 MolWeight:142 RetIndex:1064
CompName:Cyclobutene-3,4-diol, tetramethyl- $$ 1,2,3,4-Tetramethyl-3-cyclobutene-1,2-diol # $$

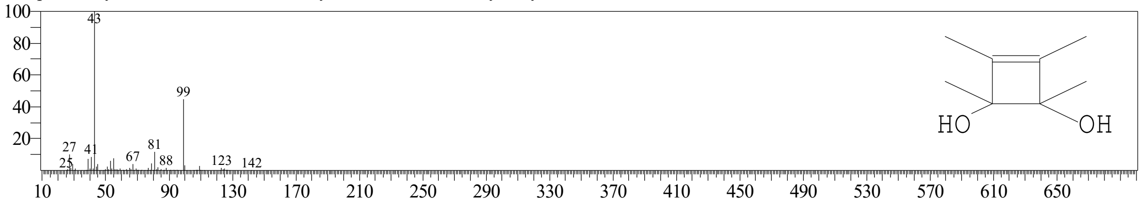

<< Target >>
Line#:36 R.Time:8.700(Scan#:941) MassPeaks:391
RawMode:Averaged 8.695-8.705(940-942) BasePeak:32.10(5488)
BG Mode:Calc. from Peak Group 1 - Event 1 Scan

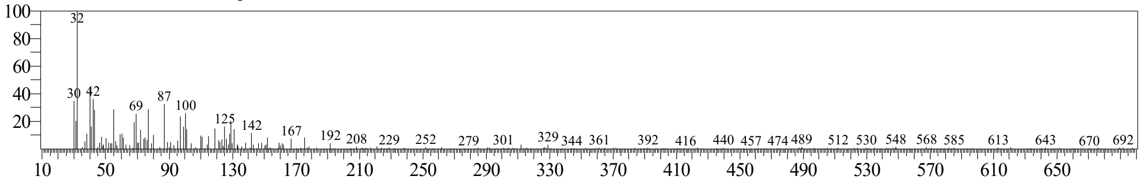

Hit#:1 Entry:4861 Library:NIST11.lib
SI:58 Formula:C4H10N2O2 CAS:3879-08-1 MolWeight:118 RetIndex:1305
CompName:4-Hydroxybutyric acid hydrazide $$ 4-Hydroxybutanoyl hydrazine $$ Butanoic acid, 4-hydroxy-, hydrazide $$

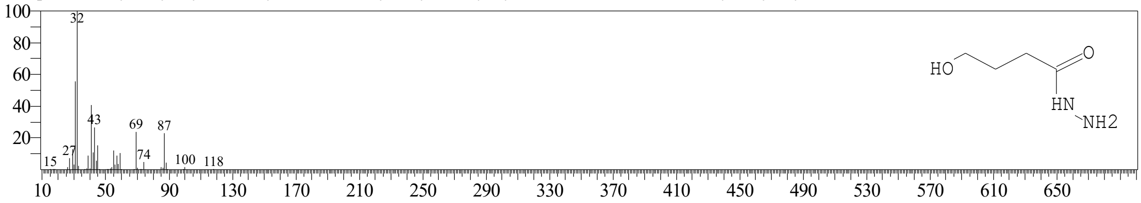

Hit#:2 Entry:1772 Library:NIST11.lib
SI:58 Formula:C3H5N3O CAS:140-87-4 MolWeight:99 RetIndex:1208
CompName:Cyacetacide $$ Cyanoacetohydrazide $$ 2-Cyanoacetylhydrazide $$ Cyanoacethydrazide $$ Cyanoacetic acid hydrazide $$ Acetic acid, cyano-, hyd

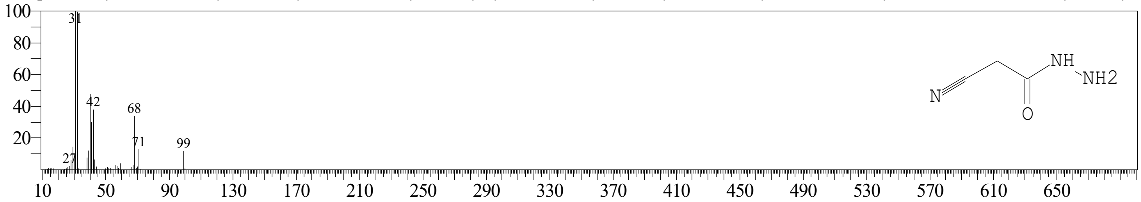

Hit#:3 Entry:7886 Library:NIST11.lib
SI:55 Formula:C7H14O2 CAS:0-00-0 MolWeight:130 RetIndex:834
CompName:iso-Butyl aldehyde propylene glycol acetal 2 $$ 2-Isopropyl-4-methyl-1,3-dioxolane # $$

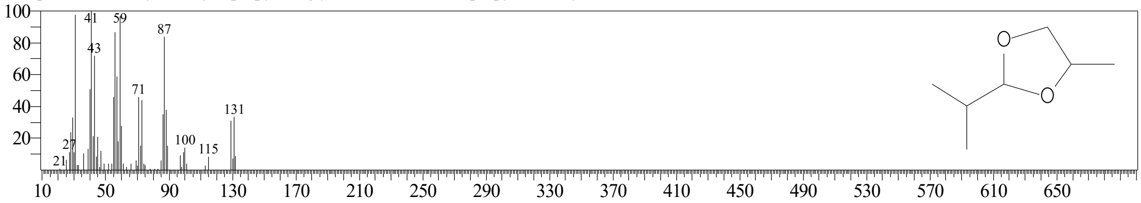

Hit#:4 Entry:43285 Library:NIST11.lib
SI:55 Formula:C12H25NO CAS:153953-62-9 MolWeight:199 RetIndex:1483
CompName:Undecanone, 2-methyl oxime $$ (2Z)-2-Undecanone o-methyloxime # $$

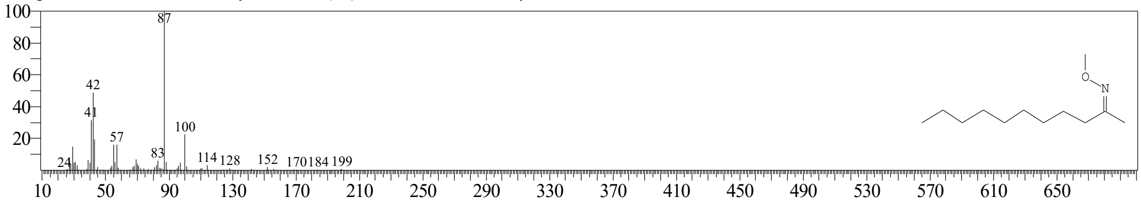

Hit#:5 Entry:20047 Library:NIST11.lib
SI:55 Formula:C7H12O4 CAS:1119-40-0 MolWeight:160 RetIndex:1052
CompName:Pentanedioic acid, dimethyl ester $$ Glutaric acid, dimethyl ester $$ Dimethyl glutarate $$ Dimethyl pentanedioate $$ Methyl glutarate $$

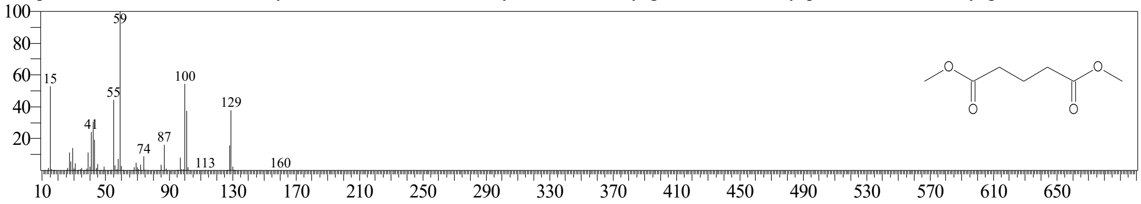

<< Target >>
Line#:37 R.Time:8.800(Scan#:961) MassPeaks:384
RawMode:Averaged 8.795-8.805(960-962) BasePeak:43.00(21194)
BG Mode:Calc. from Peak Group 1 - Event 1 Scan

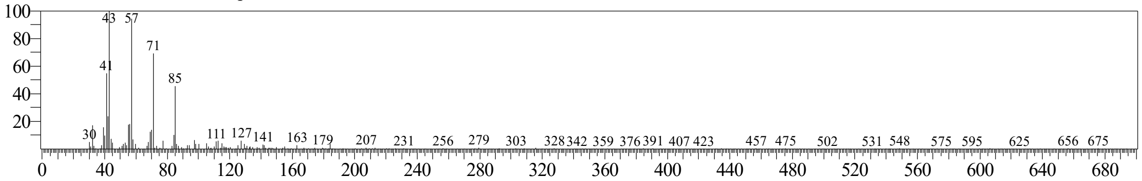

Hit#:1 Entry:18707 Library:NIST11.lib
SI:87 Formula:C11H24 CAS:62016-30-2 MolWeight:156 RetIndex:966
CompName:Octane, 2,3,3-trimethyl- $$ 2,3,3-Trimethyloctane # $$

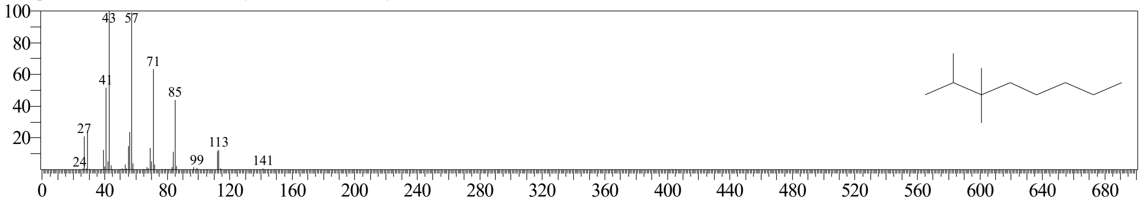

Hit#:2 Entry:26089 Library:NIST11.lib
SI:86 Formula:C12H26 CAS:17312-54-8 MolWeight:170 RetIndex:1086
CompName:Decane, 3,7-dimethyl- $$ 3,7-Dimethyldecane # $$

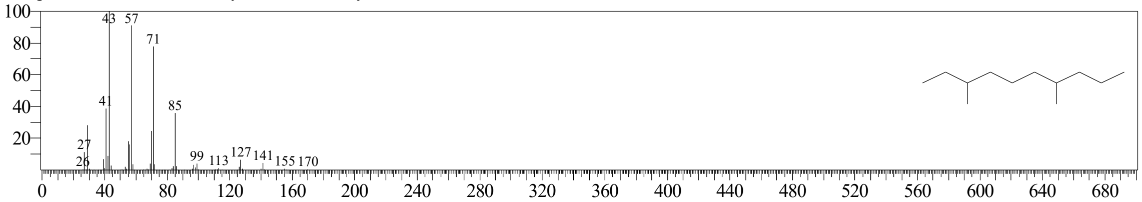

Hit#:3 Entry:18717 Library:NIST11.lib
SI:86 Formula:C11H24 CAS:6975-98-0 MolWeight:156 RetIndex:1051
CompName:Decane, 2-methyl- $$ 2-Methyldecane $$ n-C8H17CH(CH3)2 $$

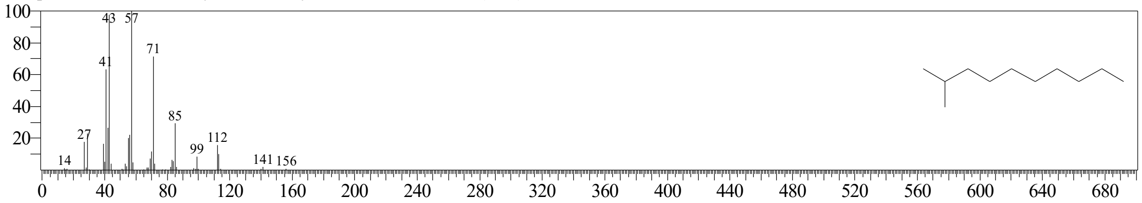

Hit#:4 Entry:47433 Library:NIST11.lib
SI:85 Formula:C9H19Br CAS:2216-35-5 MolWeight:206 RetIndex:1148
CompName:2-Bromononane $$ sec-Nonyl bromide $$ Nonane, 2-bromo- $$

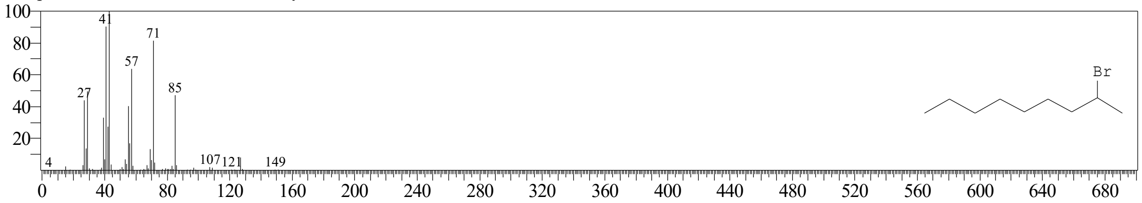

Hit#:5 Entry:26091 Library:NIST11.lib
SI:85 Formula:C12H26 CAS:13150-81-7 MolWeight:170 RetIndex:1086
CompName:2,6-Dimethyldecane

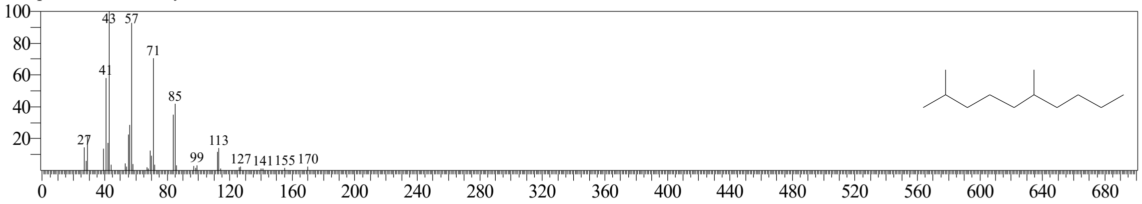

<< Target >>
Line#:38 R.Time:9.240(Scan#:1049) MassPeaks:375
RawMode:Averaged 9.235-9.245(1048-1050) BasePeak:99.10(8568)
BG Mode:Calc. from Peak Group 1 - Event 1 Scan

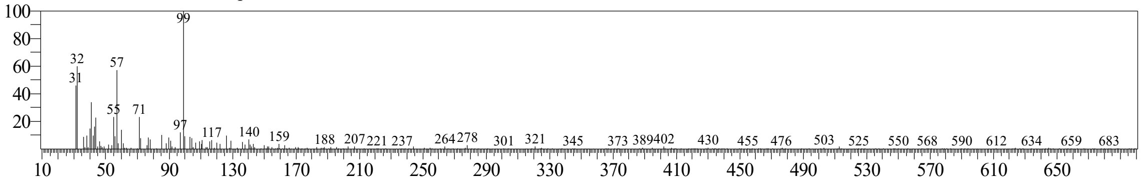

Hit#:1 Entry:27131 Library:NIST11.lib
SI:65 Formula:C10H20O2 CAS:105-79-3 MolWeight:172 RetIndex:1118
CompName:Hexanoic acid, 2-methylpropyl ester $$ Hexanoic acid, isobutyl ester $$ Isobutyl caproate $$ Isobutyl hexanoate $$ 2-Methylpropyl hexanoate $$ n·

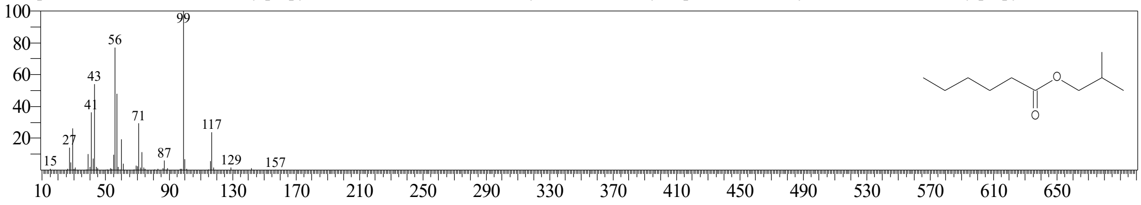

Hit#:2 Entry:7236 Library:NIST11.lib
SI:65 Formula:C7H16N2 CAS:6794-92-9 MolWeight:128 RetIndex:984
CompName:Diaziridine, 1,2-dipropyl- $$ 1,2-Dipropyldiaziridine # $$

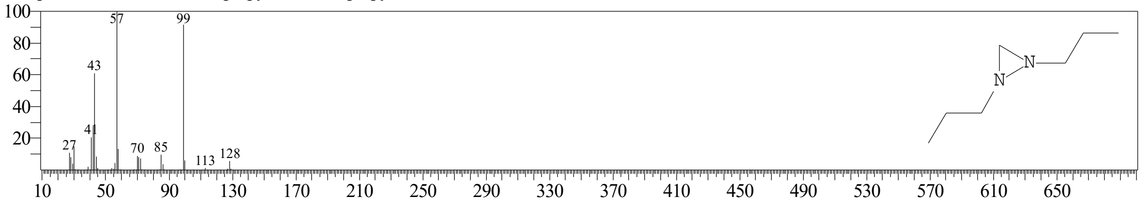

Hit#:3 Entry:26745 Library:NIST11.lib
SI:65 Formula:C8H12O4 CAS:925-21-3 MolWeight:172 RetIndex:1348
CompName:2-Butenedioic acid (Z)-, monobutyl ester $$ Maleic acid, monobutyl ester $$ Butyl hydrogen maleate $$ Hydrogen butyl maleate $$ Monobutyl mal

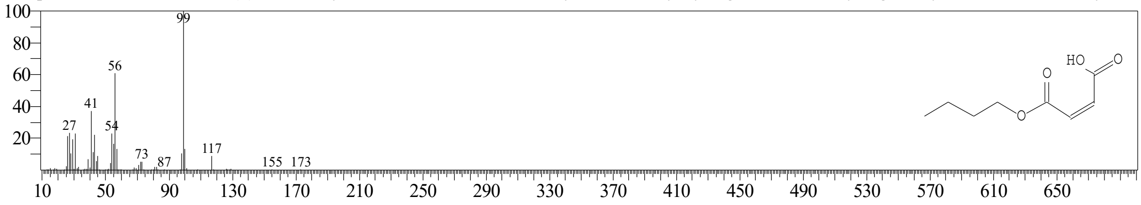

Hit#:4 Entry:18585 Library:NIST11.lib
SI:64 Formula:C10H20O CAS:35778-39-3 MolWeight:156 RetIndex:1087
CompName:3-Methyl-4-nonanone

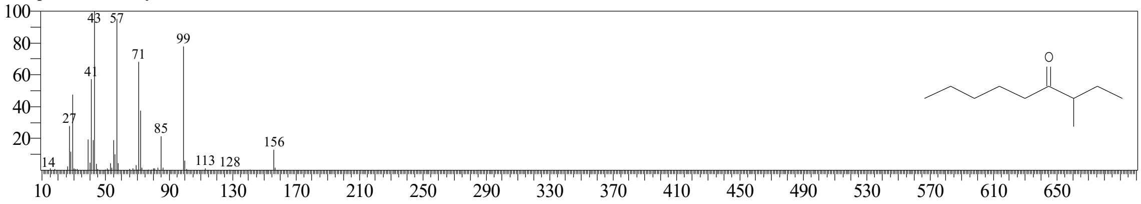

Hit#:5 Entry:149100 Library:NIST11.lib
SI:63 Formula:C22H44O2 CAS:0-00-0 MolWeight:340 RetIndex:2311
CompName:Hexanoic acid, 4-hexadecyl ester $$ 1-Propyltridecyl hexanoate # $$

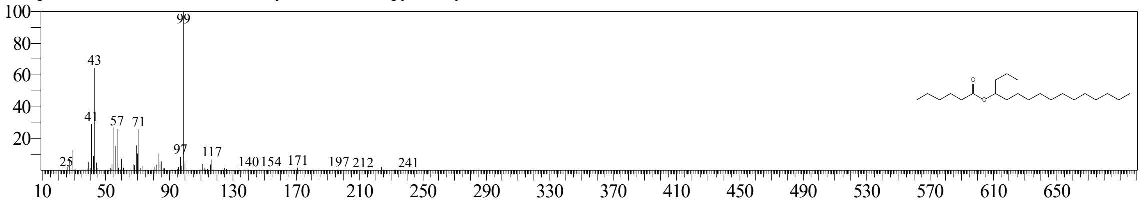

<< Target >>
Line#:39 R.Time:9.705(Scan#:1142) MassPeaks:374
RawMode:Averaged 9.700-9.710(1141-1143) BasePeak:43.10(23704)
BG Mode:Calc. from Peak Group 1 - Event 1 Scan

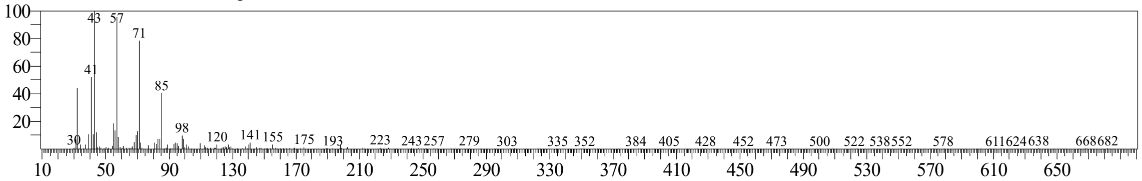

Hit#:1 Entry:104531 Library:NIST11.lib
SI:85 Formula:C20H42 CAS:1560-86-7 MolWeight:282 RetIndex:1945
CompName:Nonadecane, 2-methyl- $$ 2-Methylnonadecane $$

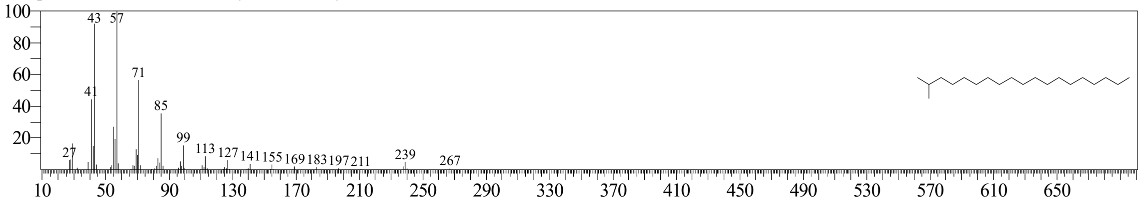

Hit#:2 Entry:26099 Library:NIST11.lib
SI:84 Formula:C12H26 CAS:17312-44-6 MolWeight:170 RetIndex:1086
CompName:2,3-Dimethyldecane $$ Decane, 2,3-dimethyl $$

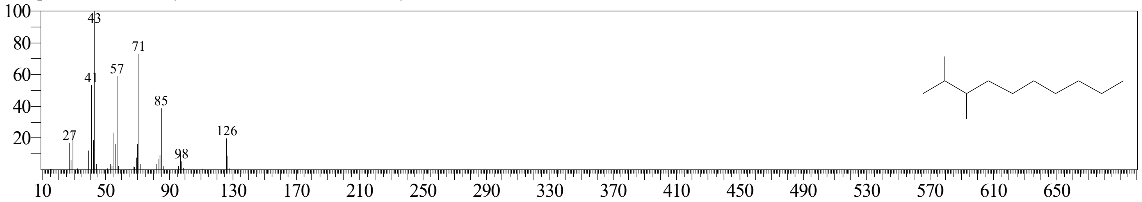

Hit#:3 Entry:26089 Library:NIST11.lib
SI:84 Formula:C12H26 CAS:17312-54-8 MolWeight:170 RetIndex:1086
CompName:Decane, 3,7-dimethyl- $$ 3,7-Dimethyldecane # $$

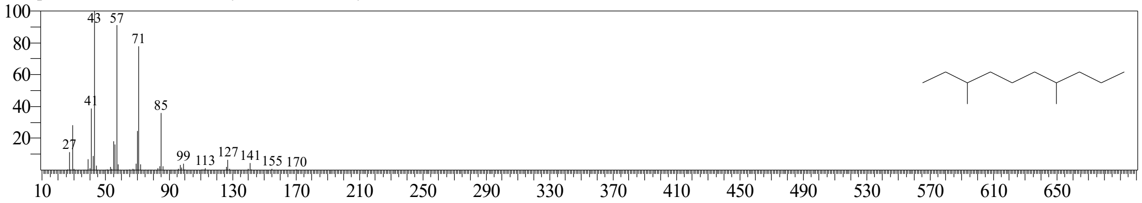

Hit#:4 Entry:93613 Library:NIST11.lib
SI:84 Formula:C19H40 CAS:1560-88-9 MolWeight:268 RetIndex:1846
CompName:Octadecane, 2-methyl- $$ 2-Methyloctadecane $$

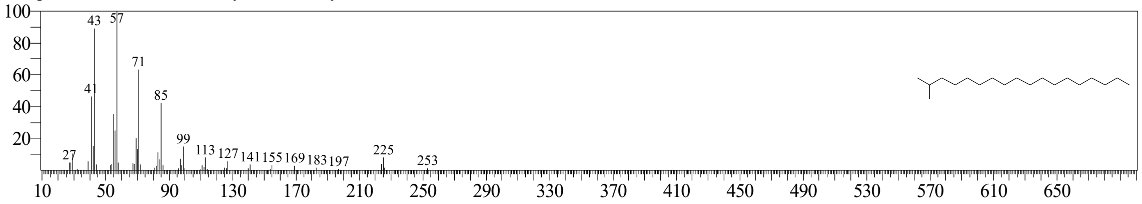

Hit#:5 Entry:115538 Library:NIST11.lib
SI:84 Formula:C21H44 CAS:1560-84-5 MolWeight:296 RetIndex:2045
CompName:Eicosane, 2-methyl- $$ 2-Methyleicosane $$ 2-Methylicosane # $$

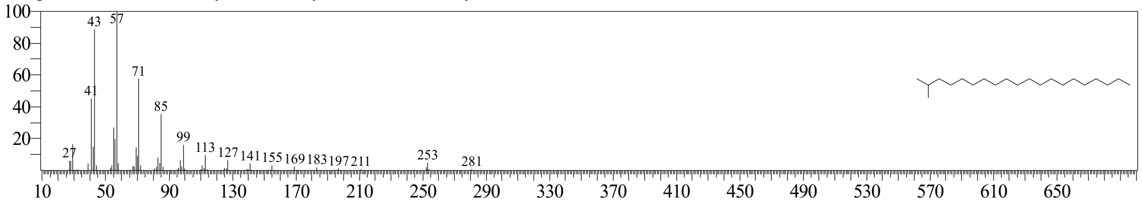

<< Target >>
Line#:40 R.Time:10.560(Scan#:1313) MassPeaks:339
RawMode:Averaged 10.555-10.565(1312-1314) BasePeak:43.10(11527)
BG Mode:Calc. from Peak Group 1 - Event 1 Scan

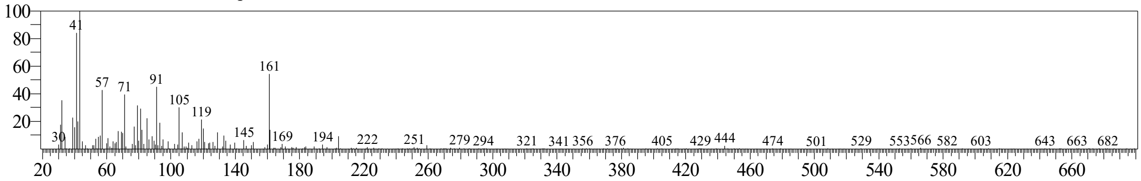

Hit#:1 Entry:46720 Library:NIST11.lib
SI:71 Formula:C15H24 CAS:0-00-0 MolWeight:204 RetIndex:1216
CompName:.beta.-copaene

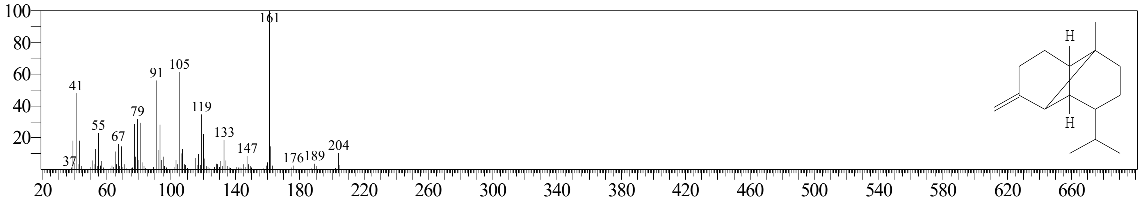

Hit#:2 Entry:134365 Library:NIST11.lib
SI:70 Formula:C21H36O2 CAS:21061-10-9 MolWeight:320 RetIndex:2300
CompName:8,11,14-Eicosatrienoic acid, methyl ester, (Z,Z,Z)- $$ Methyl dihomo-.gamma.-linolenate $$ Methyl (8Z,11Z,14Z)-8,11,14-icosatrienoate # $$ Dihc

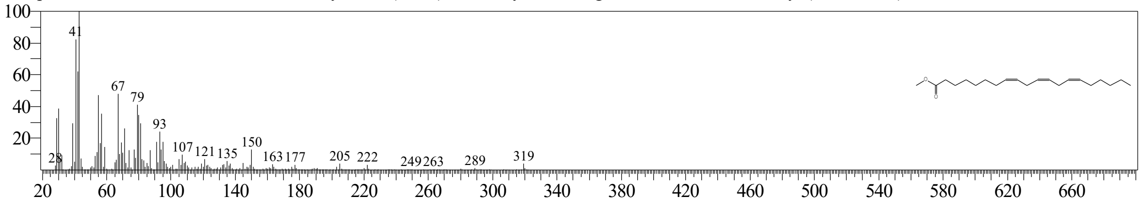

Hit#:3 Entry:181811 Library:NIST11.lib
SI:70 Formula:C28H46O CAS:7199-92-0 MolWeight:398 RetIndex:2735
CompName:Cholesta-8,24-dien-3-ol, 4-methyl-, (3.beta.,4.alpha.)- $$ 4-Methylcholesta-8,24-dien-3-ol # $$

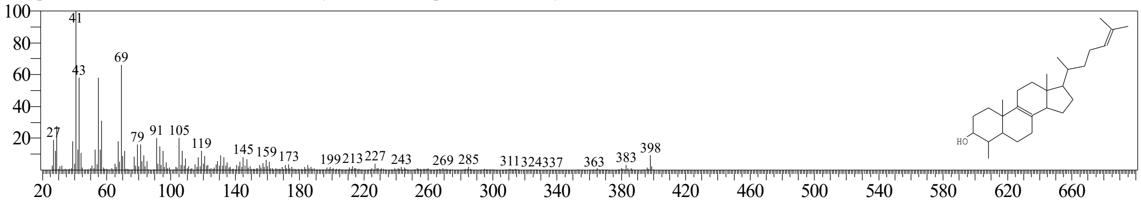

Hit#:4 Entry:59440 Library:NIST11.lib
SI:69 Formula:C15H26O CAS:19435-97-3 MolWeight:222 RetIndex:1580
CompName:1-Naphthalenol, 1,2,3,4,4a,7,8,8a-octahydro-1,6-dimethyl-4-(1-methylethyl)-, [1R-(1.alpha.,4.beta.,4a.beta.,8a.beta.)]- $$ 1.beta.-Cadin-4-en-10-ol

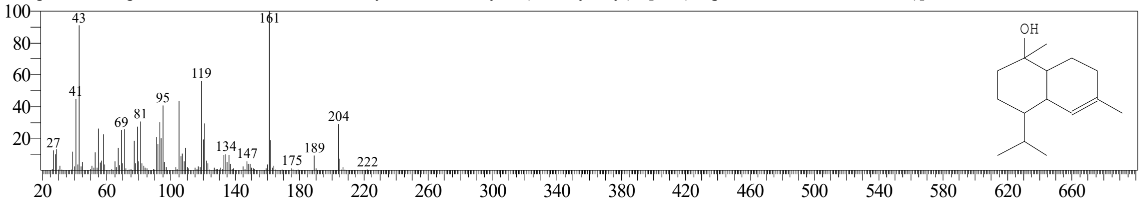

Hit#:5 Entry:59360 Library:NIST11.lib
SI:69 Formula:C15H26O CAS:515-69-5 MolWeight:222 RetIndex:1625
CompName:.alpha.-Bisabolol $$ Bisabolol $$ 3-Cyclohexene-1-methanol, .alpha.,4-dimethyl-.alpha.-(4-methyl-3-pentenyl)-, (R*,R*)- $$ 5-Hepten-2-ol, 6-metl

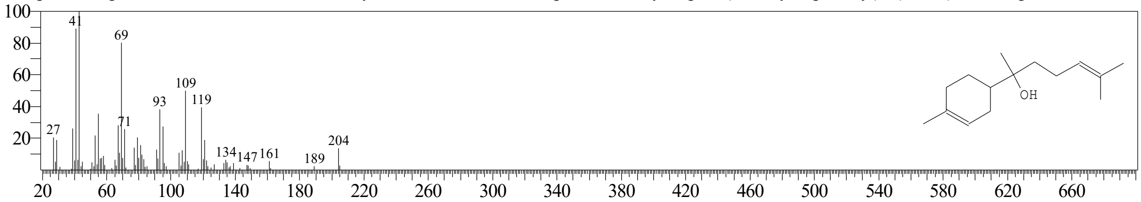

<< Target >>
Line#:41 R.Time:11.120(Scan#:1425) MassPeaks:345
RawMode:Averaged 11.115-11.125(1424-1426) BasePeak:41.10(6271)
BG Mode:Calc. from Peak Group 1 - Event 1 Scan

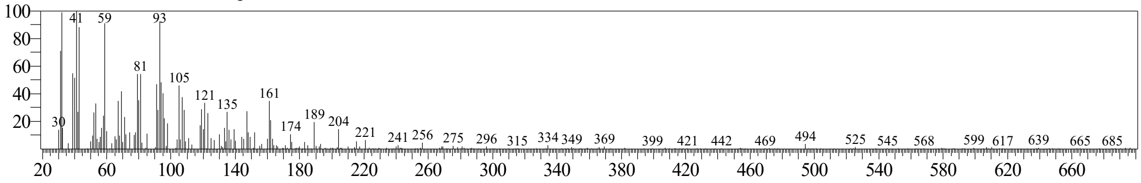

Hit#:1 Entry:59376 Library:NIST11.lib
SI:69 Formula:C15H26O CAS:639-99-6 MolWeight:222 RetIndex:1522
CompName:Cyclohexanemethanol, 4-ethenyl-.alpha.,.alpha.,4-trimethyl-3-(1-methylethenyl)-, [1R-(1.alpha.,3.alpha.,4.beta.)]- $$ o-Menth-8-ene-4-methanol, .a

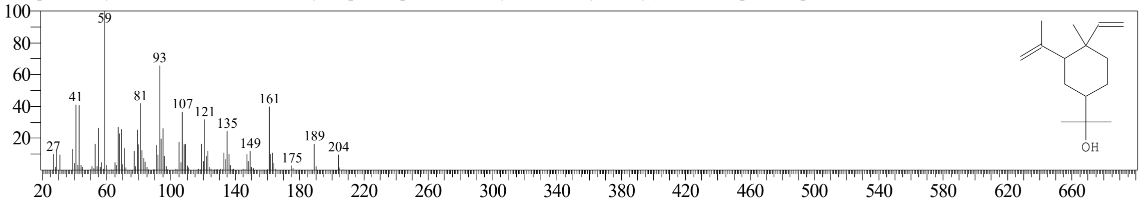

Hit#:2 Entry:46618 Library:NIST11.lib
SI:69 Formula:C15H24 CAS:13877-93-5 MolWeight:204 RetIndex:1494
CompName:Bicyclo[7.2.0]undec-4-ene, 4,11,11-trimethyl-8-methylene- $$ Bicyclo[7.2.0]undec-4-ene, 4,11,11-trimethyl-8-methylene-, (Z)- $$ cis-4,11,11-Trim

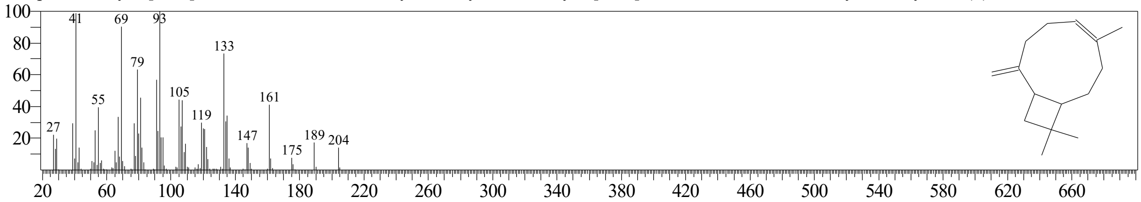

Hit#:3 Entry:46594 Library:NIST11.lib
SI:68 Formula:C15H24 CAS:72747-25-2 MolWeight:204 RetIndex:1386
CompName:1H-Cycloprop[e]azulene, decahydro-1,1,7-trimethyl-4-methylene- $$ 1H-Cycloprop[e]azulene, decahydro-1,1,7-trimethyl-4-methylene-, (1aR,4aR,7

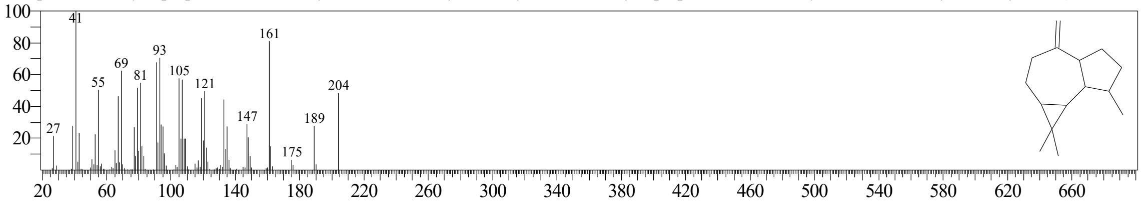

Hit#:4 Entry:59424 Library:NIST11.lib
SI:68 Formula:C15H26O CAS:21284-22-0 MolWeight:222 RetIndex:1580
CompName:Cubenol $$ 1-Isopropyl-4,7-dimethyl-1,3,4,5,6,8a-hexahydro-4a(2H)-naphthalenol-, [1S-(1.alpha.,4.beta.,4a.beta.,8a.alpha.)]- $$ (-)-Cubenol $$ 10

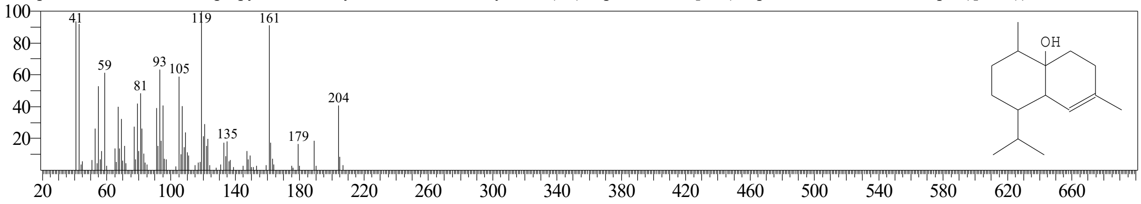

Hit#:5 Entry:46664 Library:NIST11.lib
SI:68 Formula:C15H24 CAS:0-00-0 MolWeight:204 RetIndex:1475
CompName:Cycloheptane, 4-methylene-1-methyl-2-(2-methyl-1-propen-1-yl)-1-vinyl- $$ 1-Methyl-4-methylene-2-(2-methyl-1-propenyl)-1-vinylcycloheptane #

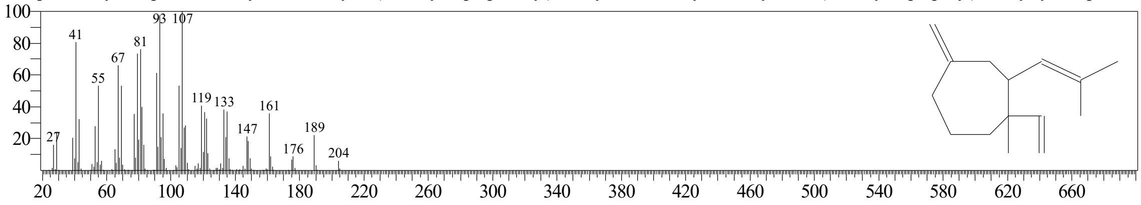

<< Target >>
Line#:42 R.Time:11.335(Scan#:1468) MassPeaks:340
RawMode:Averaged 11.330-11.340(1467-1469) BasePeak:41.00(16948)
BG Mode:Calc. from Peak Group 1 - Event 1 Scan

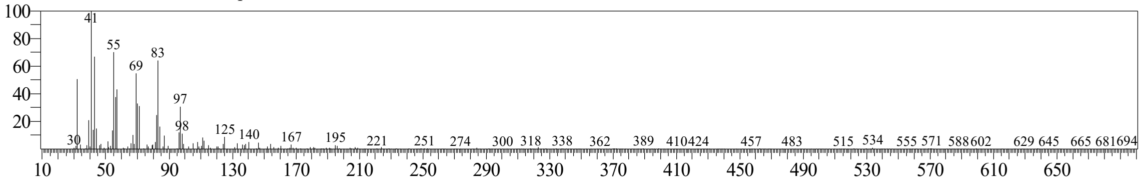

Hit#:1 Entry:92916 Library:NIST11.lib
SI:84 Formula:C12H25ClO2S CAS:10147-40-7 MolWeight:268 RetIndex:1897
CompName:1-Dodecanesulfonyl chloride $$ 1-Dodecanesulphonyl chloride $$

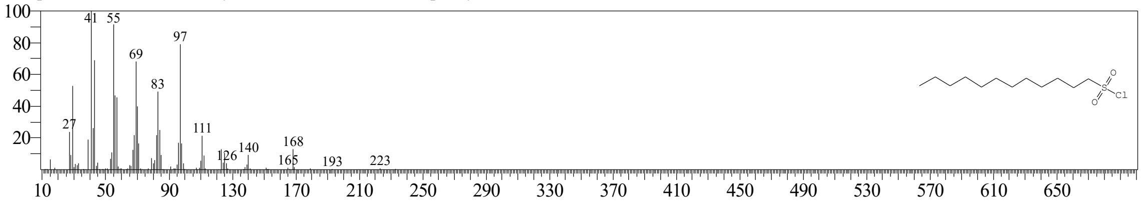

Hit#:2 Entry:32996 Library:NIST11.lib
SI:84 Formula:C13H26 CAS:295-02-3 MolWeight:182 RetIndex:1559
CompName:Cyclotridecane

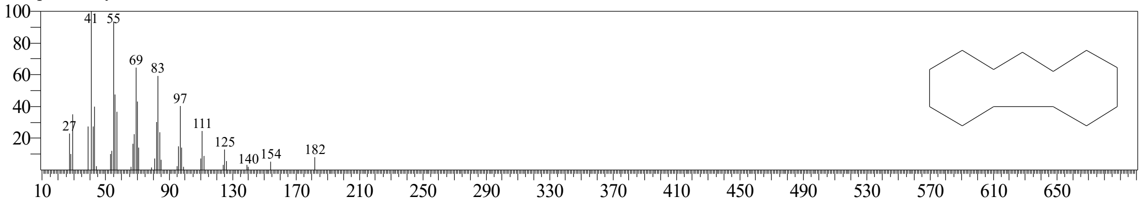

Hit#:3 Entry:41639 Library:NIST11.lib
SI:83 Formula:C14H28 CAS:41446-68-8 MolWeight:196 RetIndex:1421
CompName:3-Tetradecene, (E)- $$ (3E)-3-Tetradecene $$ (E)-3-Tetradecene $$ trans-3-Tetradecene $$

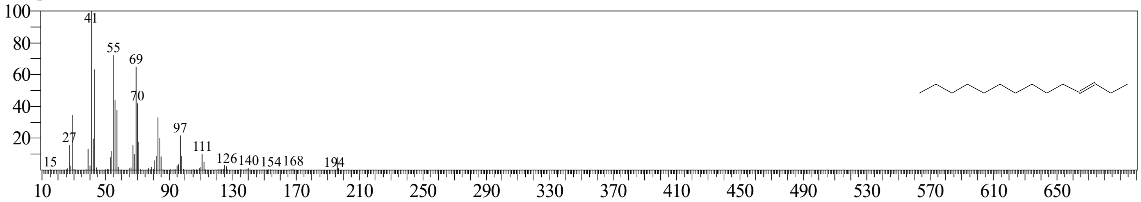

Hit#:4 Entry:24832 Library:NIST11.lib
SI:83 Formula:C12H24 CAS:112-41-4 MolWeight:168 RetIndex:1204
CompName:1-Dodecene $$ .alpha.-Dodecene $$ n-Dodec-1-ene $$ Adacene 12 $$ Dodec-1-ene $$ .alpha.-Dodecylene $$ Dodecylene .alpha.- $$ Dodecene-1 $

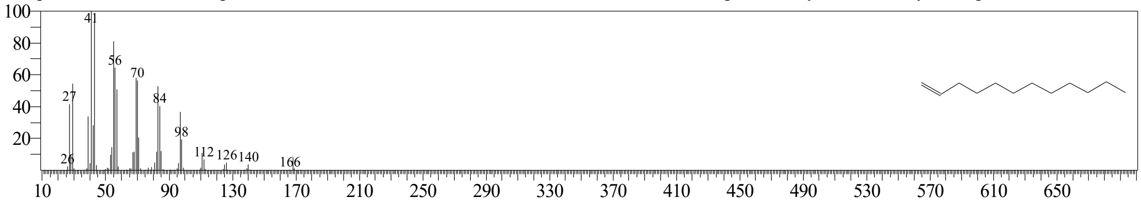

Hit#:5 Entry:24855 Library:NIST11.lib
SI:83 Formula:C12H24 CAS:74663-85-7 MolWeight:168 RetIndex:1216
CompName:Cyclopropane, nonyl- $$ Nonylcyclopropane # $$ n-Nonyl-cyclopropane $$

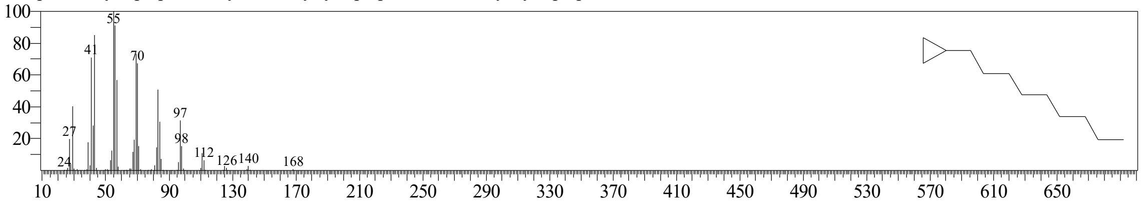

<< Target >>
Line#:43 R.Time:11.380(Scan#:1477) MassPeaks:358
RawMode:Averaged 11.375-11.385(1476-1478) BasePeak:43.10(199076)
BG Mode:Calc. from Peak Group 1 - Event 1 Scan

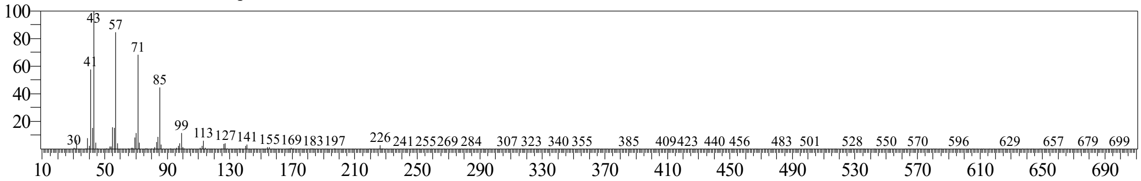

Hit#:1 Entry:62482 Library:NIST11.lib
SI:93 Formula:C16H34 CAS:55045-10-8 MolWeight:226 RetIndex:1548
CompName:Tridecane, 6-propyl- $$ 6-Propyltridecane # $$

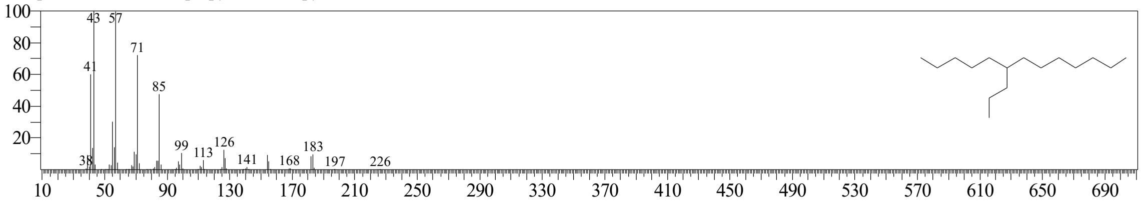

Hit#:2 Entry:62481 Library:NIST11.lib
SI:93 Formula:C16H34 CAS:55045-09-5 MolWeight:226 RetIndex:1548
CompName:Tridecane, 7-propyl- $$ 7-n-Propyltridecane $$ 7-Propyltridecane # $$

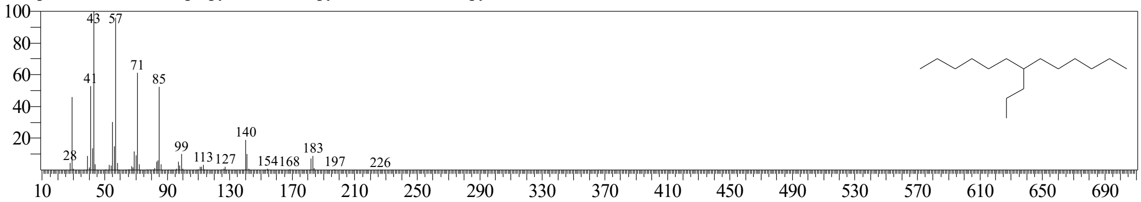

Hit#:3 Entry:62480 Library:NIST11.lib
SI:93 Formula:C16H34 CAS:55045-14-2 MolWeight:226 RetIndex:1548
CompName:Tetradecane, 4-ethyl- $$ 4-Ethyltetradecane $$

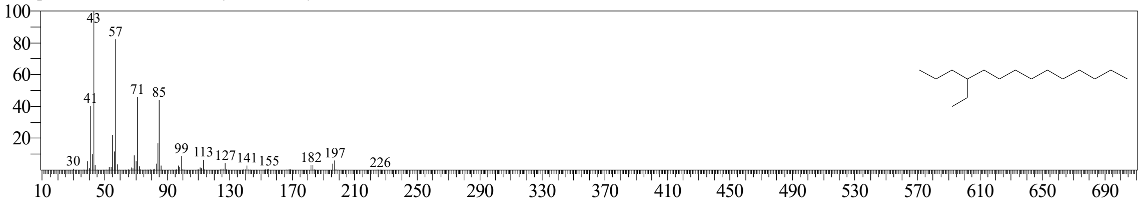

Hit#:4 Entry:26089 Library:NIST11.lib
SI:92 Formula:C12H26 CAS:17312-54-8 MolWeight:170 RetIndex:1086
CompName:Decane, 3,7-dimethyl- $$ 3,7-Dimethyldecane # $$

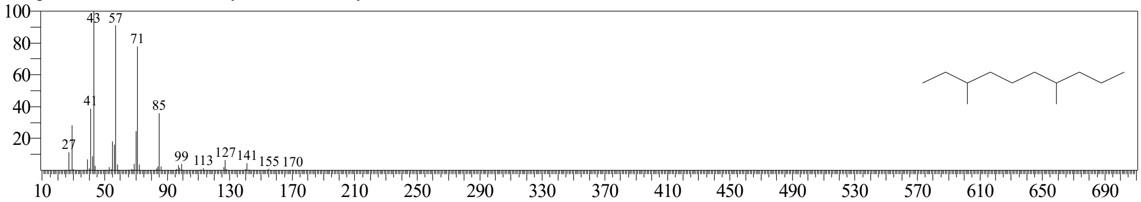

Hit#:5 Entry:42951 Library:NIST11.lib
SI:92 Formula:C14H30 CAS:6117-98-2 MolWeight:198 RetIndex:1285
CompName:2,3-Dimethyldodecane

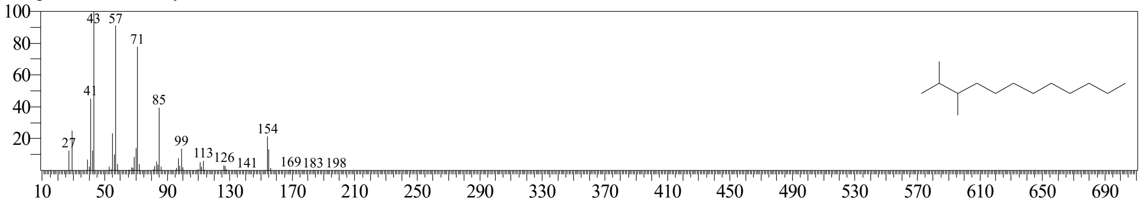

<< Target >>
Line#:44 R.Time:11.445(Scan#:1490) MassPeaks:356
RawMode:Averaged 11.440-11.450(1489-1491) BasePeak:32.10(9584)
BG Mode:Calc. from Peak Group 1 - Event 1 Scan

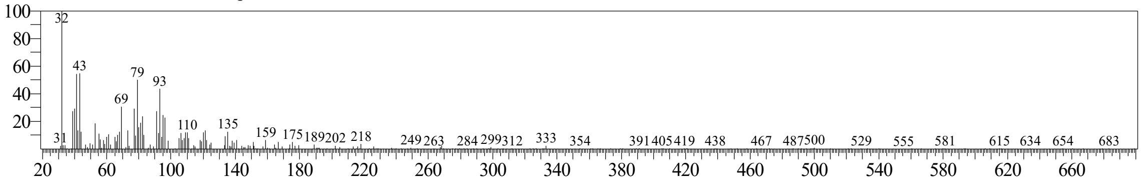

Hit#:1 Entry:22485 Library:NIST11.lib
SI:74 Formula:C12H20 CAS:0-00-0 MolWeight:164 RetIndex:1086
CompName:1-Pentene, 5-(2,2-dimethylcyclopropyl)-2-methyl-4-methylene- $$ 1,1-Dimethyl-2-(4-methyl-2-methylene-4-pentenyl)cyclopropane # $$

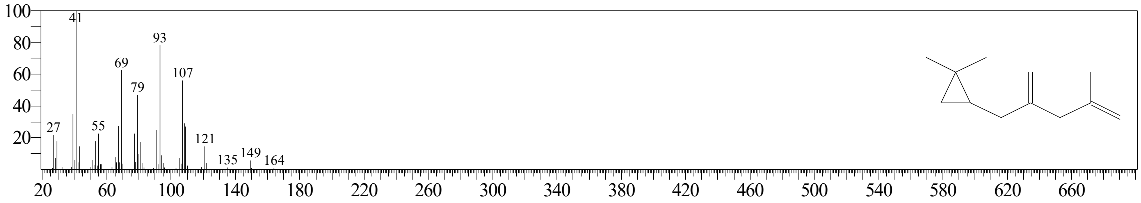

Hit#:2 Entry:57732 Library:NIST11.lib
SI:74 Formula:C15H24O CAS:1139-30-6 MolWeight:220 RetIndex:1507
CompName:Caryophyllene oxide $$ 5-Oxatricyclo[8.2.0.0(4,6)-]dodecane, 4,12,12-trimethyl-9-methylene-, [1R-(1R*,4R*,6R*,10S*)]- $$ 5-Oxatricyclo(8.2.0.

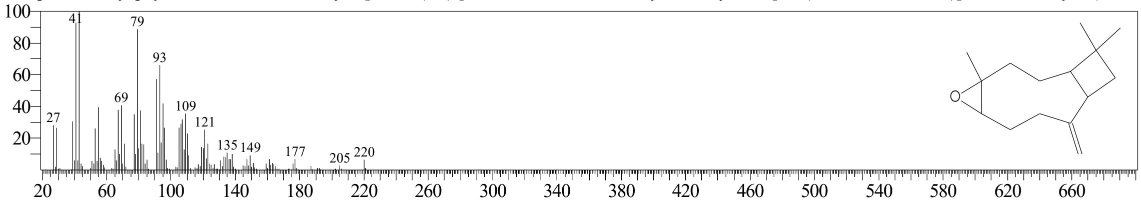

Hit#:3 Entry:37595 Library:NIST11.lib
SI:73 Formula:C14H22 CAS:62338-27-6 MolWeight:190 RetIndex:1404
CompName:1,5,9,11-Tridecatetraene, 12-methyl-, (E,E)- $$ (5E,9E)-12-Methyl-1,5,9,11-tridecatetraene # $$

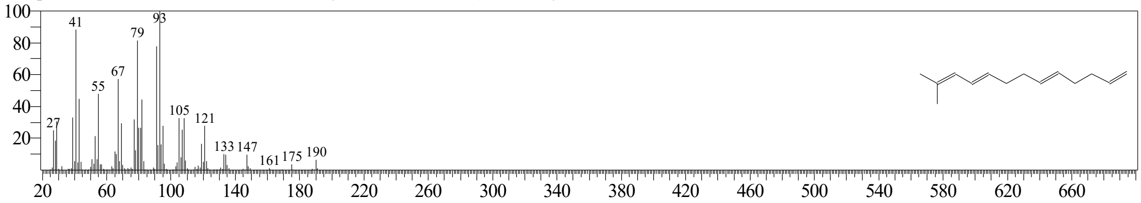

Hit#:4 Entry:37594 Library:NIST11.lib
SI:73 Formula:C14H22 CAS:62338-42-5 MolWeight:190 RetIndex:1380
CompName:Cyclobutene, 4,4-dimethyl-1-(2,7-octadienyl)- $$ 4,4-Dimethyl-1-[(2E)-2,7-octadienyl]-1-cyclobutene # $$

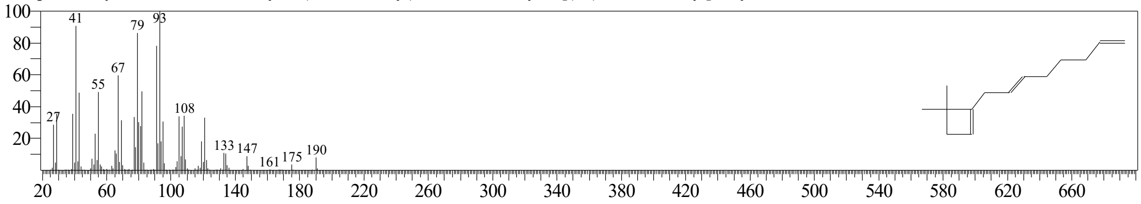

Hit#:5 Entry:38806 Library:NIST11.lib
SI:73 Formula:C14H24 CAS:74752-91-3 MolWeight:192 RetIndex:1420
CompName:1-Tetradecen-3-yne

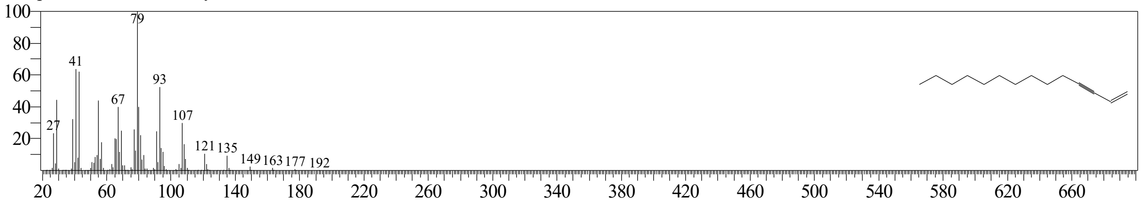

<< Target >>
Line#:45 R.Time:11.510(Scan#:1503) MassPeaks:346
RawMode:Averaged 11.505-11.515(1502-1504) BasePeak:43.00(35069)
BG Mode:Calc. from Peak Group 1 - Event 1 Scan

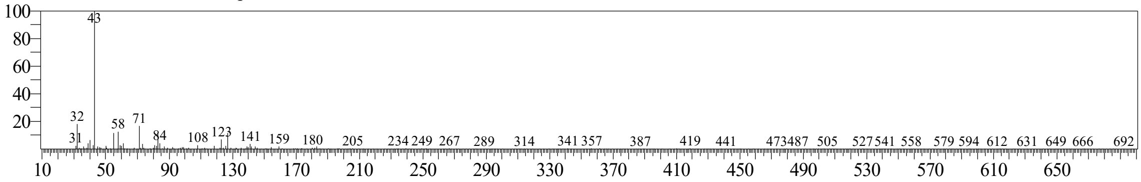

Hit#:1 Entry:42682 Library:NIST11.lib
SI:75 Formula:C12H22O2 CAS:7029-09-6 MolWeight:198 RetIndex:1486
CompName:2,11-Dodecanedione

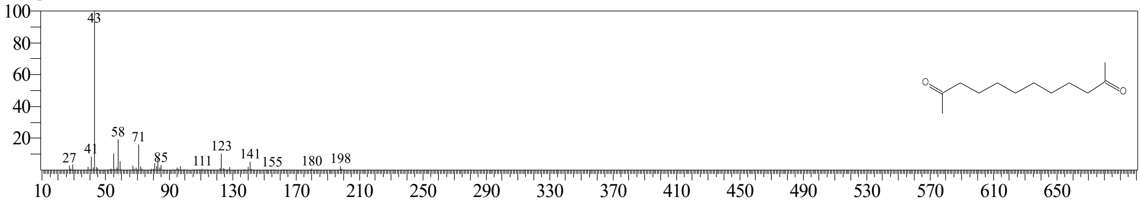

Hit#:2 Entry:12813 Library:NIST11.lib
SI:72 Formula:C7H12O3 CAS:3128-07-2 MolWeight:144 RetIndex:1209
CompName:Heptanoic acid, 6-oxo- $$ 6-Oxoheptanoic acid # $$

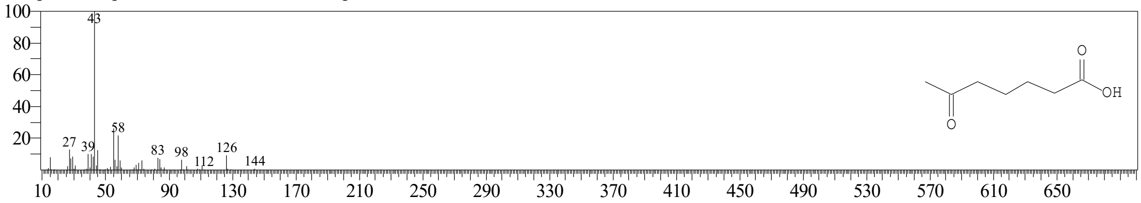

Hit#:3 Entry:7794 Library:NIST11.lib
SI:69 Formula:C6H10O3 CAS:0-00-0 MolWeight:130 RetIndex:811
CompName:2,3-Dimethyloxirane-2-carboxylic acid, methyl ester

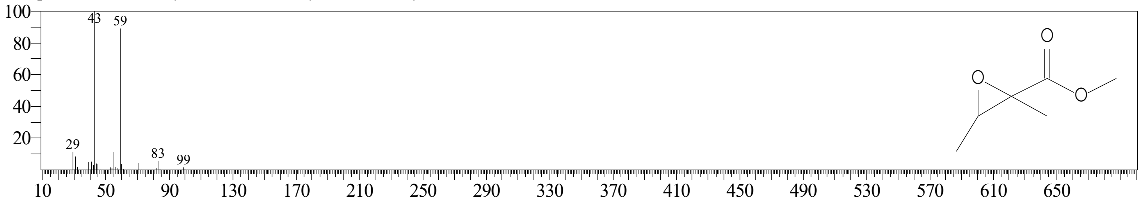

Hit#:4 Entry:8387 Library:NIST11.lib
SI:69 Formula:C6H12OS CAS:19872-52-7 MolWeight:132 RetIndex:973
CompName:2-Pentanone, 4-mercapto-4-methyl- $$ 4-Methyl-4-thiolpentan-2-one $$ 4-Methyl-4-sulfanyl-2-pentanone $$ 4-Mercapto-4-methyl-2-pentanone $$

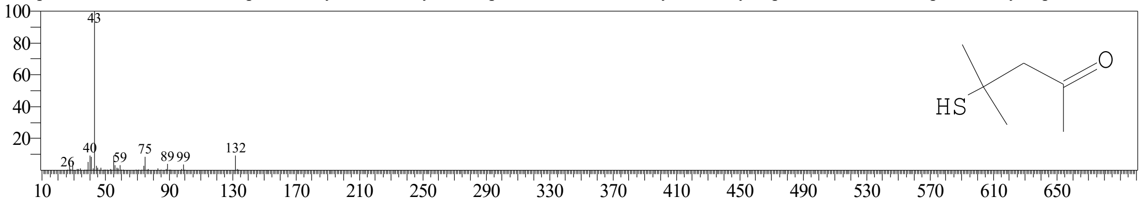

Hit#:5 Entry:4135 Library:NIST11.lib
SI:69 Formula:C7H14O CAS:110-12-3 MolWeight:114 RetIndex:789
CompName:2-Hexanone, 5-methyl- $$ Isoamyl methyl ketone $$ Isopentyl methyl ketone $$ Methyl isoamyl ketone $$ Methyl isopentyl ketone $$ MIAK $$ 2-I

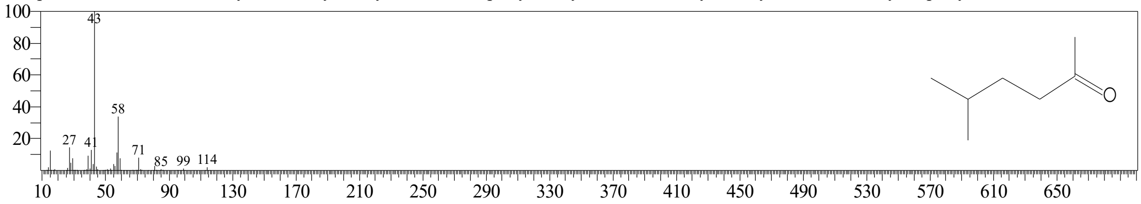

<< Target >>
Line#:46 R.Time:12.045(Scan#:1610) MassPeaks:416
RawMode:Averaged 12.040-12.050(1609-1611) BasePeak:59.05(15867)
BG Mode:Calc. from Peak Group 1 - Event 1 Scan

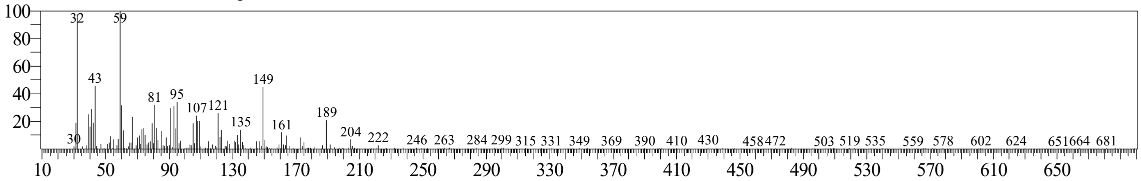

Hit#:1 Entry:59380 Library:NIST11.lib
SI:71 Formula:C15H26O CAS:473-15-4 MolWeight:222 RetIndex:1593
CompName:2-Naphthalenemethanol, decahydro-.alpha.,.alpha.,4a-trimethyl-8-methylene-, [2R-(2.alpha.,4a.alpha.,8a.beta.)]- $$ Eudesm-4(14)-en-11-ol $$ .beta

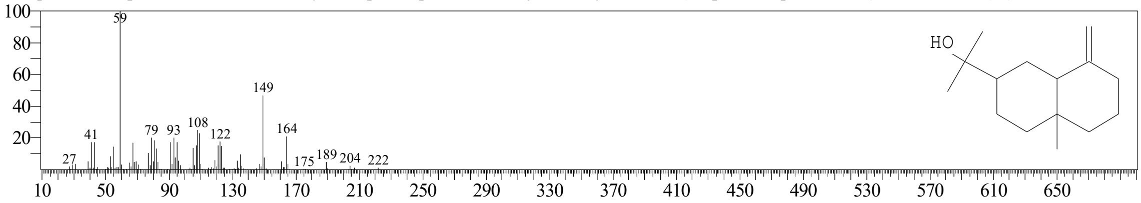

Hit#:2 Entry:59379 Library:NIST11.lib
SI:69 Formula:C15H26O CAS:63891-61-2 MolWeight:222 RetIndex:1598
CompName:2-Naphthalenemethanol, 2,3,4,4a,5,6,7,8-octahydro-.alpha.,.alpha.,4a,8-tetramethyl-, [2R-(2.alpha.,4a.beta.,8.beta.)]- $$ 2-(4a,8-Dimethyl-2,3,4,4a,

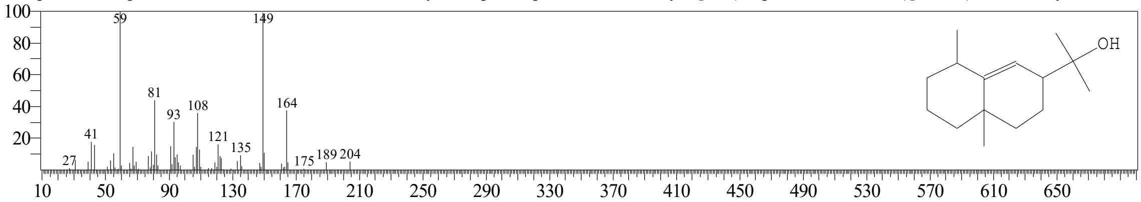

Hit#:3 Entry:59377 Library:NIST11.lib
SI:68 Formula:C15H26O CAS:21657-90-9 MolWeight:222 RetIndex:1694
CompName:3,7-Cyclodecadiene-1-methanol, .alpha.,.alpha.,4,8-tetramethyl-, [s-(Z,Z)] $$ Hedycaryol $$ 2-(4,8-Dimethyl-3,7-cyclodecadien-1-yl)-2-propanol #

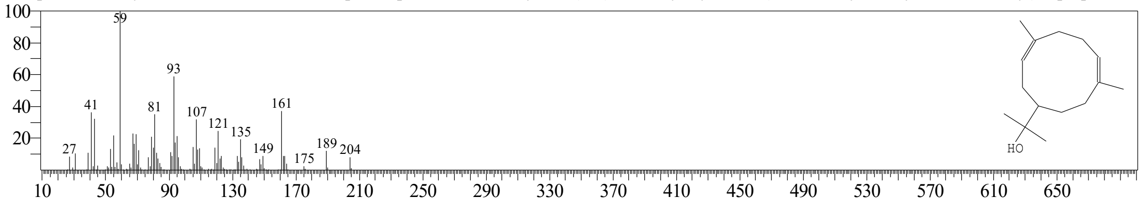

Hit#:4 Entry:59376 Library:NIST11.lib
SI:68 Formula:C15H26O CAS:639-99-6 MolWeight:222 RetIndex:1522
CompName:Cyclohexanemethanol, 4-ethenyl-.alpha.,.alpha.,4-trimethyl-3-(1-methylethenyl)-, [1R-(1.alpha.,3.alpha.,4.beta.)]- $$ o-Menth-8-ene-4-methanol, .a

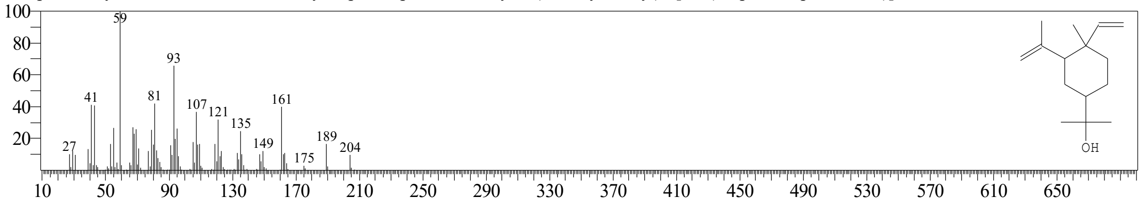

Hit#:5 Entry:59378 Library:NIST11.lib
SI:67 Formula:C15H26O CAS:79254-46-9 MolWeight:222 RetIndex:1598
CompName:2-Naphthalenemethanol, 1,2,3,4,4a,5,6,8a-octahydro-.alpha.,.alpha.,4a,8-tetramethyl-, (2.alpha.,4a.alpha.,8a.alpha.)- $$ 2-(4a,8-Dimethyl-1,2,3,4,4a

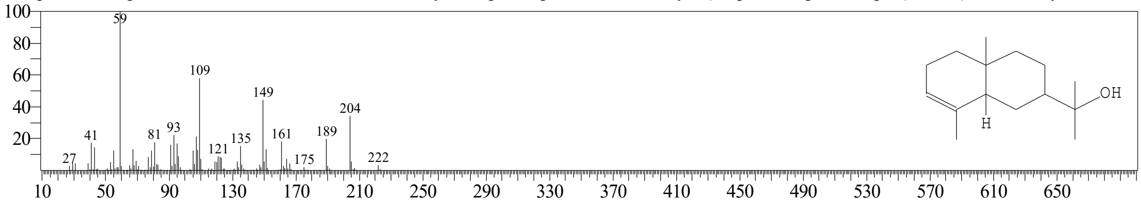

Qualitative Analysis Report

<< Target >>
Line#:47 R.Time:12.435(Scan#:1688) MassPeaks:363
RawMode:Averaged 12.430-12.440(1687-1689) BasePeak:87.10(73756)
BG Mode:Calc. from Peak Group 1 - Event 1 Scan

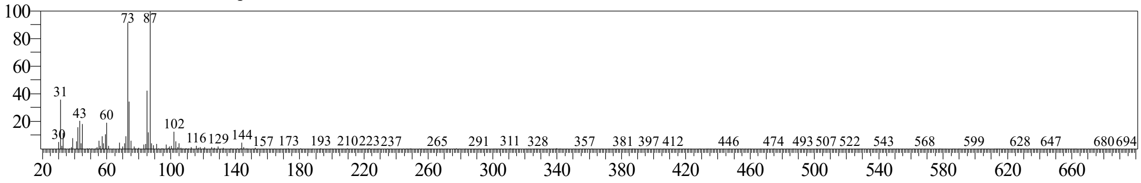

Hit#:1 Entry:39436 Library:NIST11.lib
SI:80 Formula:C7H14O6 CAS:4097-91-0 MolWeight:194 RetIndex:1667
CompName:.alpha.-d-Mannofuranoside, methyl $$ Methyl hexofuranoside # $$

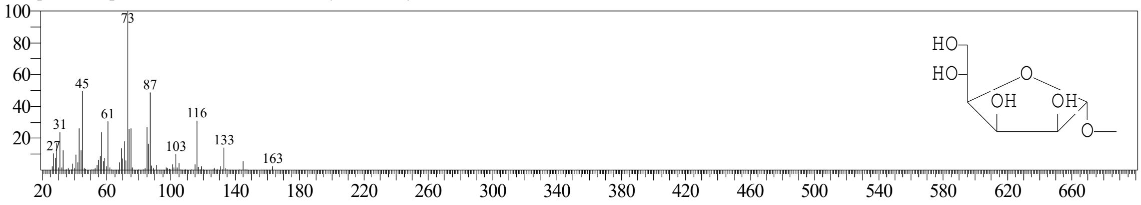

Hit#:2 Entry:39445 Library:NIST11.lib
SI:79 Formula:C7H14O6 CAS:36864-61-6 MolWeight:194 RetIndex:1647
CompName:2-O-Methyl-D-mannopyranosa $$ 2-O-Methylhexose # $$

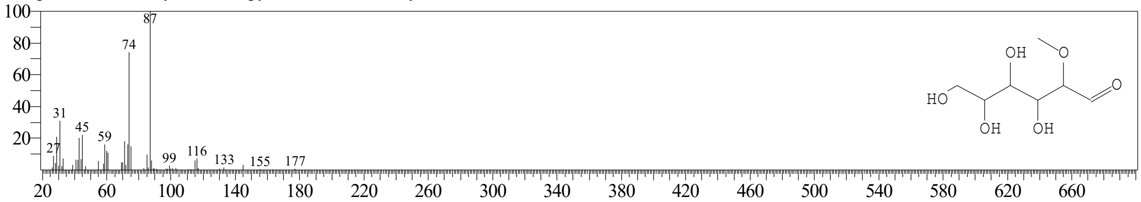

Hit#:3 Entry:39437 Library:NIST11.lib
SI:78 Formula:C7H14O6 CAS:26295-70-5 MolWeight:194 RetIndex:1667
CompName:.beta.-d-Mannofuranoside, methyl $$ Methyl hexofuranoside # $$

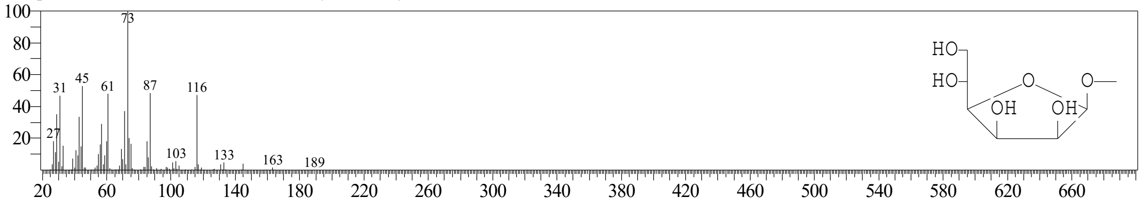

Hit#:4 Entry:39444 Library:NIST11.lib
SI:78 Formula:C7H14O6 CAS:36256-85-6 MolWeight:194 RetIndex:1675
CompName:D-Fructose, 3-O-methyl- $$ 3-O-Methyl-D-fructose $$ 3-O-Methylhex-2-ulose # $$

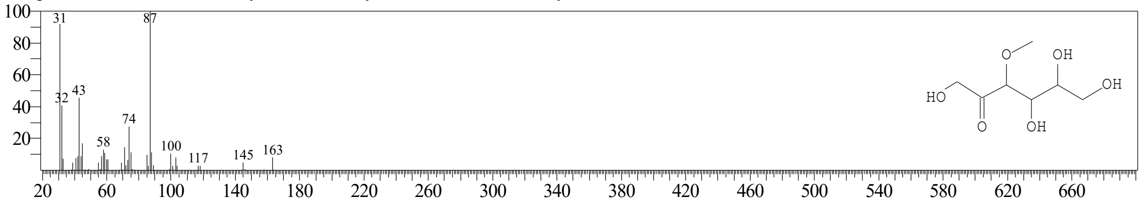

Hit#:5 Entry:38099 Library:NIST11.lib
SI:77 Formula:C7H12O6 CAS:0-00-0 MolWeight:192 RetIndex:1541
CompName:.alpha.-d-6,3-Furanose, methyl-.beta.-d-glucohexodialdo-1,4-furanoside

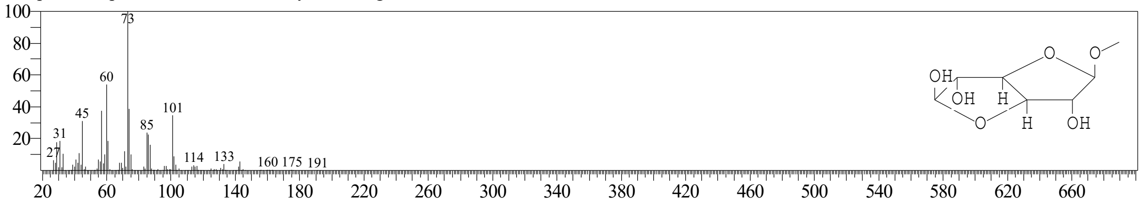

<< Target >>
Line#:48 R.Time:12.530(Scan#:1707) MassPeaks:350
RawMode:Averaged 12.525-12.535(1706-1708) BasePeak:31.10(4564)
BG Mode:Calc. from Peak Group 1 - Event 1 Scan

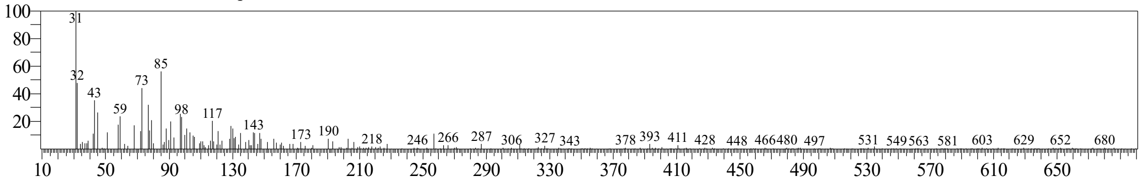

Hit#:1 Entry:91642 Library:NIST11.lib
SI:53 Formula:C15H26O2Si CAS:0-00-0 MolWeight:266 RetIndex:1587
CompName:1-Methoxy- 5-trimethylsilyloxy-3-phenylpentane $$ [(5-Methoxy-3-phenylpentyl)oxy](trimethyl)silane # $$

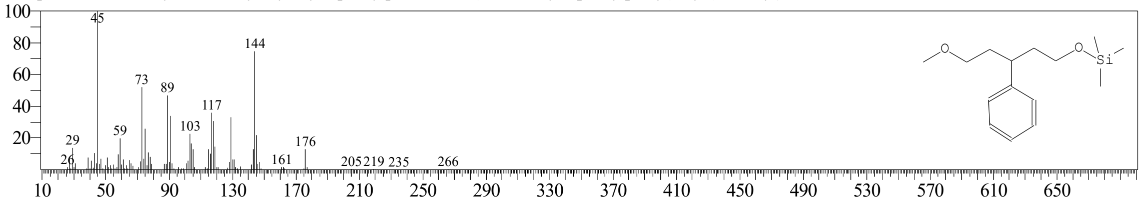

Hit#:2 Entry:76030 Library:NIST11.lib
SI:53 Formula:C8H13ClF2O4 CAS:0-00-0 MolWeight:246 RetIndex:1154
CompName:Diethylene glycol monoethyl ether, chlorodifluoroacetate

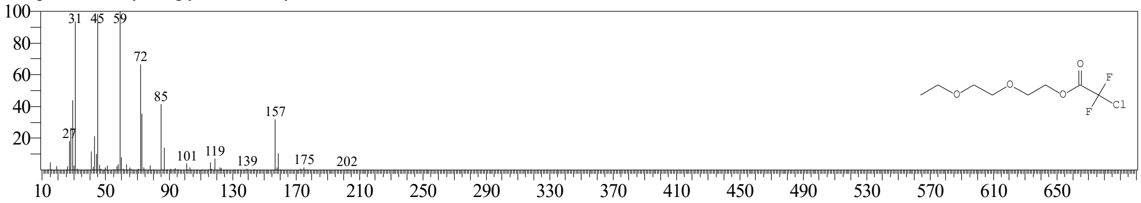

Hit#:3 Entry:40061 Library:NIST11.lib
SI:52 Formula:C12H18O2 CAS:70928-46-0 MolWeight:194 RetIndex:1545
CompName:1-Hydroxy-5-methoxy-3-phenylpentane $$ 5-Methoxy-3-phenyl-1-pentanol # $$

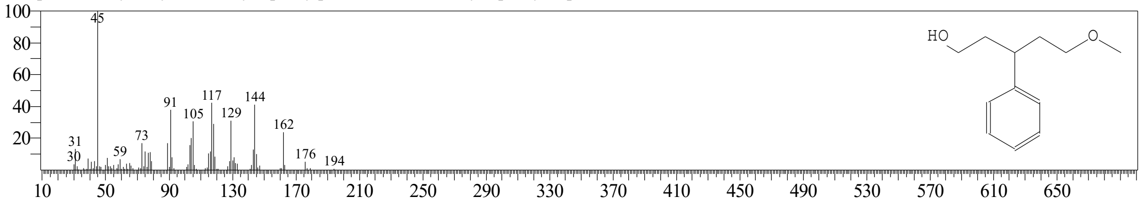

Hit#:4 Entry:4630 Library:NIST11.lib
SI:52 Formula:C6H16N2 CAS:3711-34-0 MolWeight:116 RetIndex:886
CompName:Hydrazine, 1,2-bis(1-methylethyl)- $$ Hydrazine, 1,2-diisopropyl- $$ 1,2-Diisopropylhydrazine $$ N,N'-Diisopropylhydrazine $$

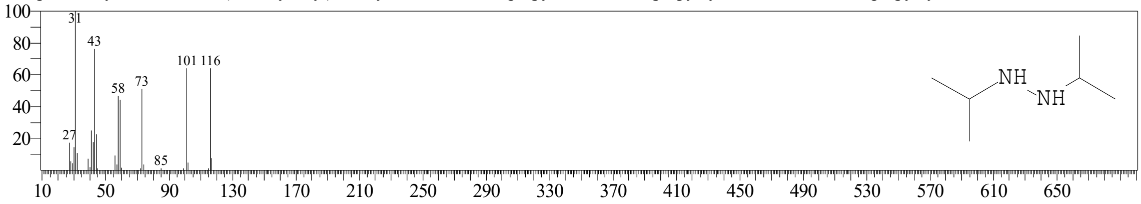

Hit#:5 Entry:13584 Library:NIST11.lib
SI:52 Formula:C6H14SSi CAS:82316-79-8 MolWeight:146 RetIndex:780
CompName:1,1,2-Trimethyl-1-sila-3-thiacyclopentane $$ 2,3,3-Trimethyl-1,3-thiasilolane # $$

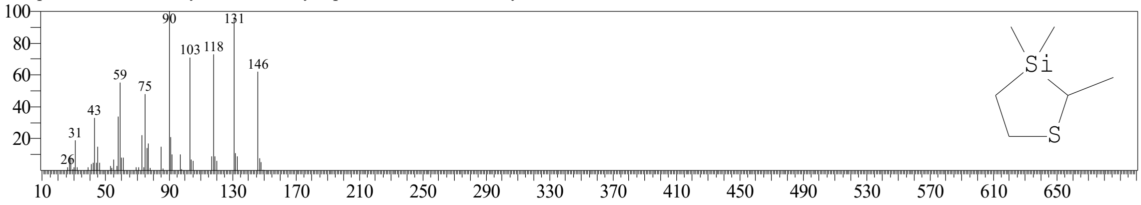

<< Target >>
Line#:49 R.Time:12.660(Scan#:1733) MassPeaks:362
RawMode:Averaged 12.655-12.665(1732-1734) BasePeak:32.10(15979)
BG Mode:Calc. from Peak Group 1 - Event 1 Scan

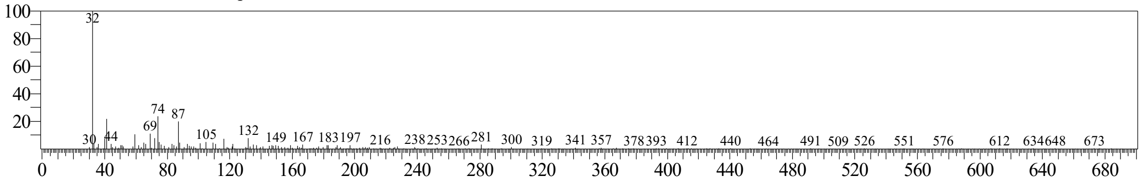

Hit#:1 Entry:4861 Library:NIST11.lib
SI:62 Formula:C4H10N2O2 CAS:3879-08-1 MolWeight:118 RetIndex:1305
CompName:4-Hydroxybutyric acid hydrazide $$ 4-Hydroxybutanoyl hydrazine $$ Butanoic acid, 4-hydroxy-, hydrazide $$

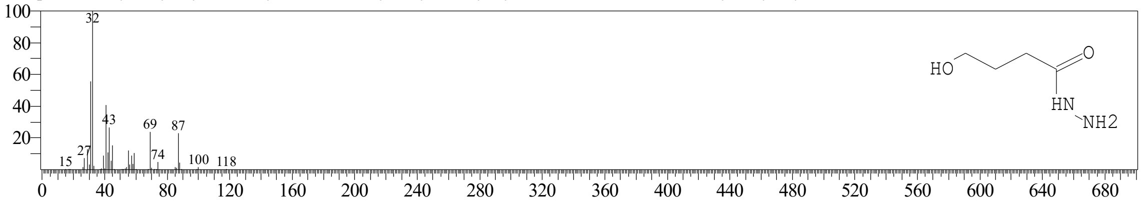

Hit#:2 Entry:4862 Library:NIST11.lib
SI:59 Formula:C4H10N2O2 CAS:0-00-0 MolWeight:118 RetIndex:1224
CompName:2-Hydroxybutanoic acid hydrazide $$ 2-Hydroxybutanohydrazide # $$

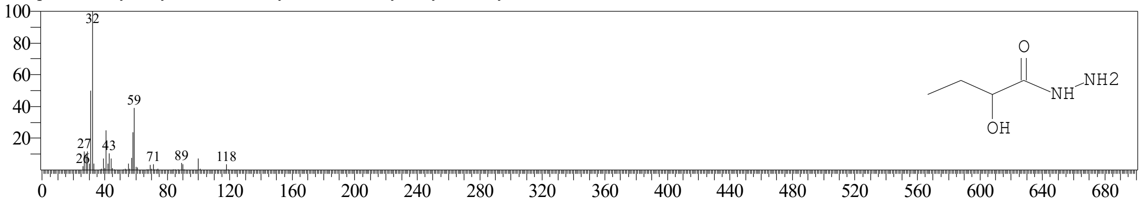

Hit#:3 Entry:8281 Library:NIST11.lib
SI:59 Formula:C3H8N4O2 CAS:3815-86-9 MolWeight:132 RetIndex:1606
CompName:Malonic acid, dihydrazide $$ Propanedioyl dihydrazide $$ Malonhydrazide $$ Malonic acid hydrazide $$ Malonic dihydrazide $$ Malonyl dihydra

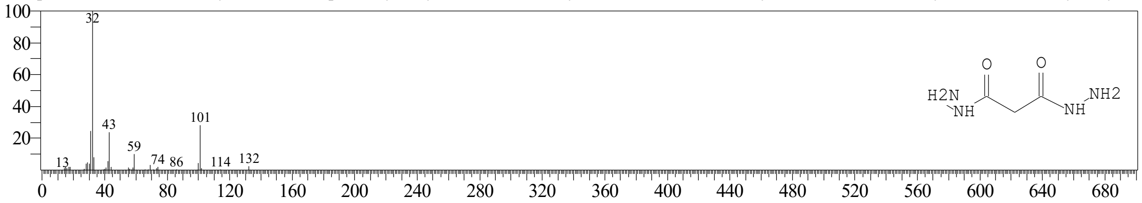

Hit#:4 Entry:95054 Library:NIST11.lib
SI:59 Formula:C16H34N2O CAS:2619-88-7 MolWeight:270 RetIndex:2255
CompName:16-Hexadecanoyl hydrazide $$ Hexadecanohydrazide # $$

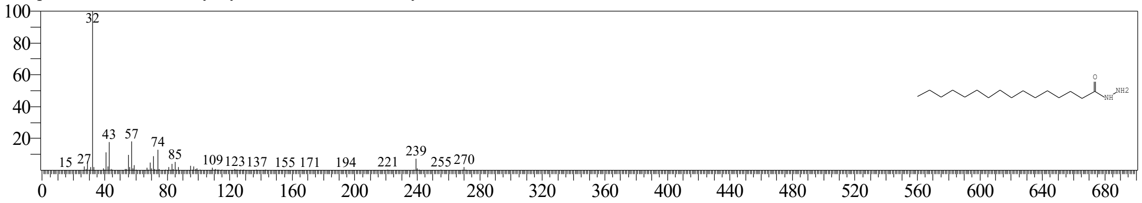

Hit#:5 Entry:117059 Library:NIST11.lib
SI:58 Formula:C18H38N2O CAS:4130-54-5 MolWeight:298 RetIndex:2454
CompName:Stearic acid hydrazide $$ Stearic hydrazide $$ Octadecanoic acid, hydrazide $$ Stearohydrazide $$ Stearoylhydrazine $$ Octadecanohydrazide # $$

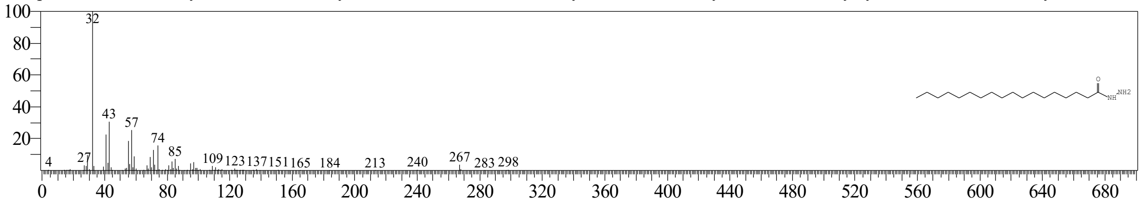

<< Target >>
Line#:50 R.Time:12.725(Scan#:1746) MassPeaks:386
RawMode:Averaged 12.720-12.730(1745-1747) BasePeak:43.10(13505)
BG Mode:Calc. from Peak Group 1 - Event 1 Scan

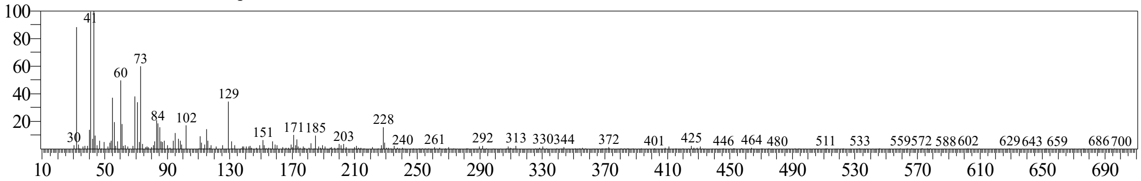

Hit#:1 Entry:53599 Library:NIST11.lib
SI:72 Formula:C13H26O2 CAS:638-53-9 MolWeight:214 RetIndex:1670
CompName:Tridecanoic acid $$ n-Tridecanoic acid $$ n-Tridecoic acid $$ Tridecylic acid $$

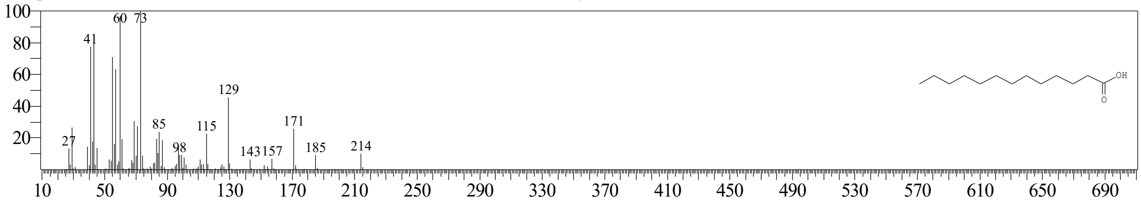

Hit#:2 Entry:128151 Library:NIST11.lib
SI:71 Formula:C20H40O2 CAS:506-30-9 MolWeight:312 RetIndex:2366
CompName:Eicosanoic acid $$ Arachic acid $$ Arachidic acid $$ Icosanoic acid $$ n-Eicosanoic acid $$ Arachidic acid (synthetic) $$

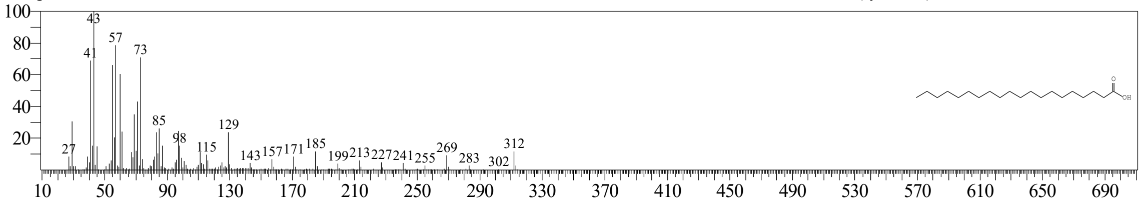

Hit#:3 Entry:84362 Library:NIST11.lib
SI:71 Formula:C16H32O2 CAS:57-10-3 MolWeight:256 RetIndex:1968
CompName:n-Hexadecanoic acid $$ Hexadecanoic acid $$ n-Hexadecoic acid $$ Palmitic acid $$ Pentadecanecarboxylic acid $$ 1-Pentadecanecarboxylic acid

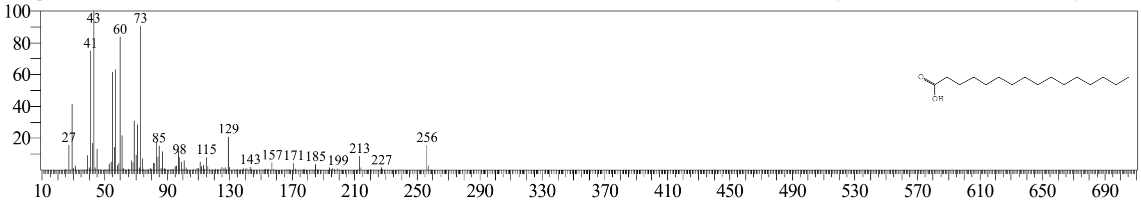

Hit#:4 Entry:169171 Library:NIST11.lib
SI:71 Formula:C22H44O4 CAS:106-11-6 MolWeight:372 RetIndex:2694
CompName:Octadecanoic acid, 2-(2-hydroxyethoxy)ethyl ester $$ Aqua Cera $$ Atlas G 2146 $$ Cerasynt $$ Cerasynt Special $$ Clindrol SDG $$ Diethylene

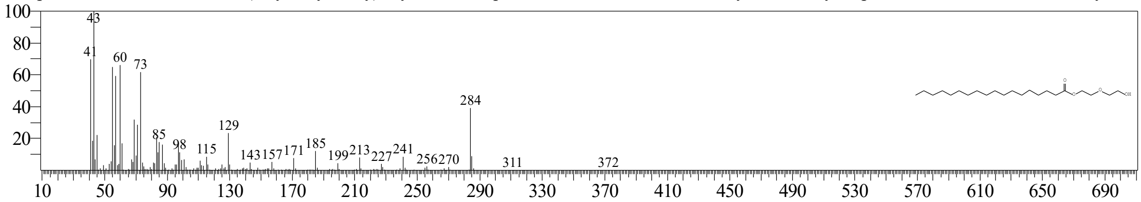

Hit#:5 Entry:63810 Library:NIST11.lib
SI:71 Formula:C14H28O2 CAS:544-63-8 MolWeight:228 RetIndex:1769
CompName:Tetradecanoic acid $$ Myristic acid $$ n-Tetradecanoic acid $$ n-Tetradecoic acid $$ Neo-Fat 14 $$ Univol U 316S $$ 1-Tridecanecarboxylic acid $

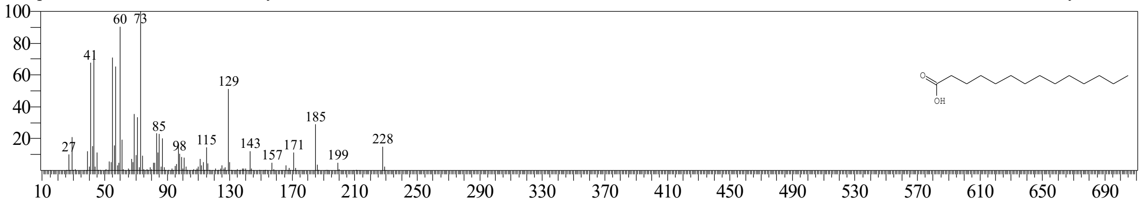

<< Target >>
Line#:51 R.Time:12.790(Scan#:1759) MassPeaks:366
RawMode:Averaged 12.785-12.795(1758-1760) BasePeak:70.10(10306)
BG Mode:Calc. from Peak Group 1 - Event 1 Scan

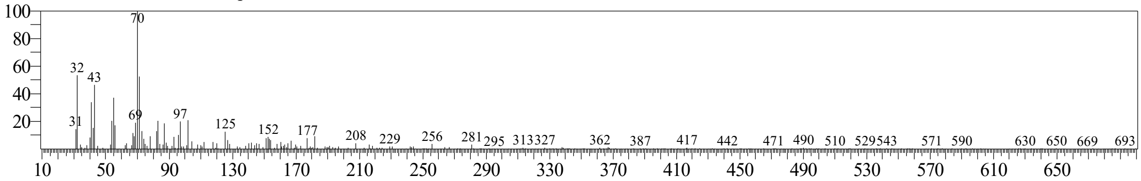

Hit#:1 Entry:17744 Library:NIST11.lib
SI:66 Formula:C11H22 CAS:31797-64-5 MolWeight:154 RetIndex:1094
CompName:Cyclohexane, (1,1-dimethylpropyl)- $$ Cyclohexane, tert-pentyl- $$ tert-Pentylcyclohexane $$ 2-Cyclohexyl-2-methylbutane $$ 2-Methyl-2-cycloh

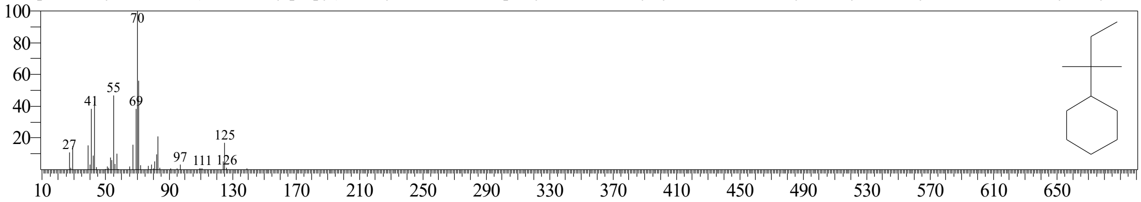

Hit#:2 Entry:24937 Library:NIST11.lib
SI:66 Formula:C12H24 CAS:74630-67-4 MolWeight:168 RetIndex:1158
CompName:5-Undecene, 3-methyl-, (E)- $$ (5E)-3-Methyl-5-undecene # $$

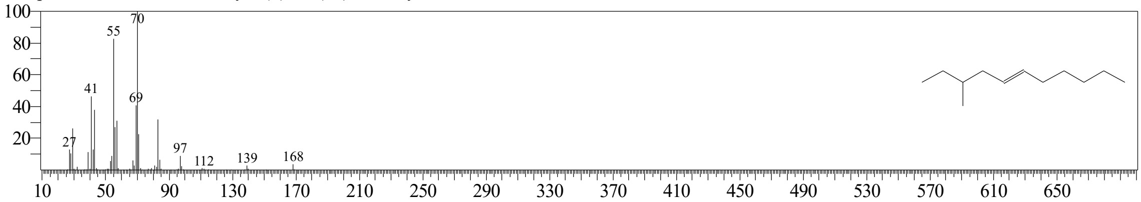

Hit#:3 Entry:44008 Library:NIST11.lib
SI:65 Formula:C12H24O2 CAS:25415-84-3 MolWeight:200 RetIndex:1317
CompName:n-Butyric acid 2-ethylhexyl ester $$ Butanoic acid, 2-ethylhexyl ester $$ 2-Ethylhexyl butyrate # $$

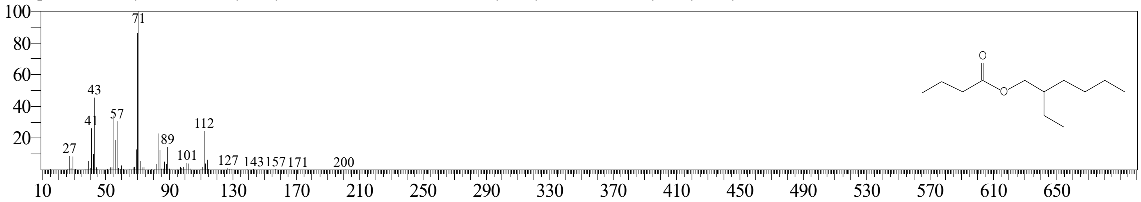

Hit#:4 Entry:11452 Library:NIST11.lib
SI:65 Formula:C10H20 CAS:74630-07-2 MolWeight:140 RetIndex:918
CompName:Octane, 3-methyl-6-methylene- $$ 2-Ethyl-5-methyl-1-heptene # $$

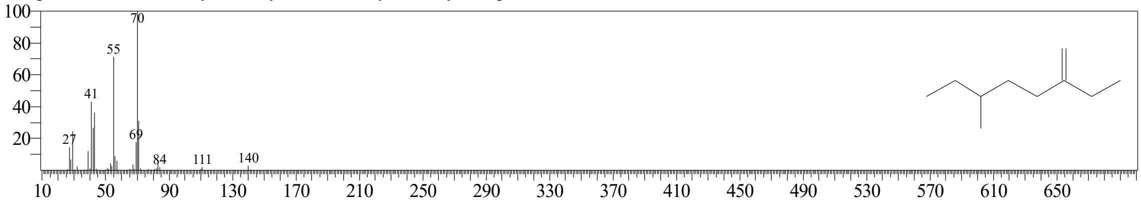

Hit#:5 Entry:3637 Library:NIST11.lib
SI:65 Formula:C8H16 CAS:16746-85-3 MolWeight:112 RetIndex:742
CompName:1-Hexene, 4-ethyl- $$ 4-Ethyl-1-hexene $$ 4-Ethylhex-1-ene $$

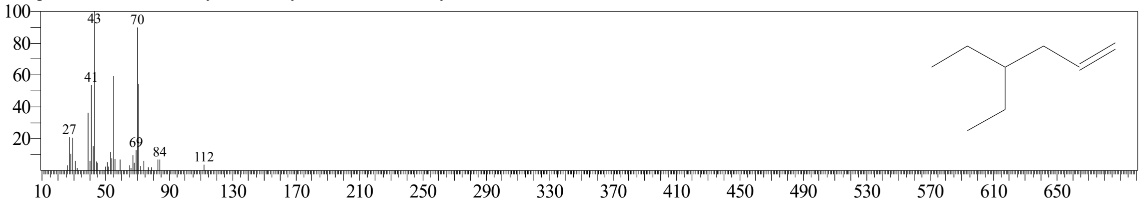

<< Target >>
Line#:52   R.Time:12.840(Scan#:1769) MassPeaks:389
RawMode:Averaged 12.835-12.845(1768-1770) BasePeak:41.05(77463)
BG Mode:Calc. from Peak Group 1 - Event 1 Scan

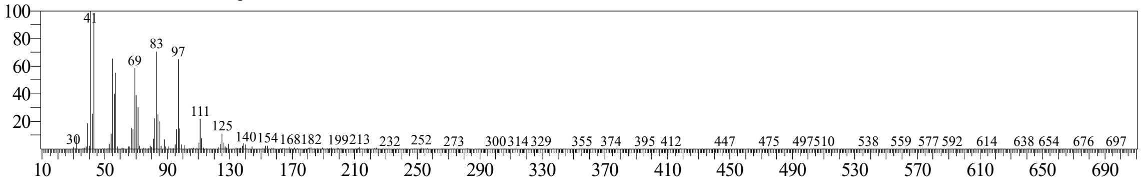

Hit#:1 Entry:70935 Library:NIST11.lib
SI:94 Formula:C17H34 CAS:6765-39-5 MolWeight:238 RetIndex:1701
CompName:1-Heptadecene $$ Hexahydroaplotaxene $$ Heptadec-1-ene $$ NSC 77132 $$ Heptadecene (Related) $$

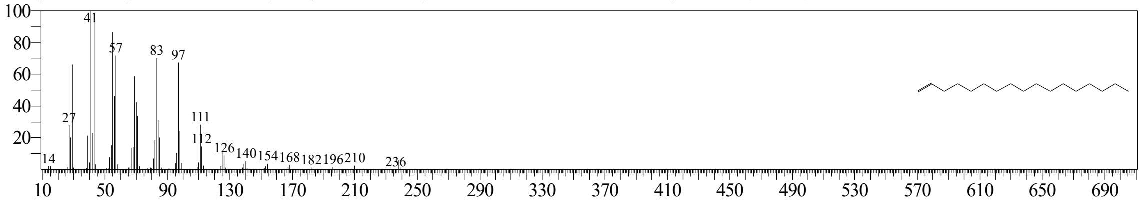

Hit#:2 Entry:81317 Library:NIST11.lib
SI:93 Formula:C17H32O CAS:0-00-0 MolWeight:252 RetIndex:1907
CompName:E-15-Heptadecenal $$ (15E)-15-Heptadecenal # $$

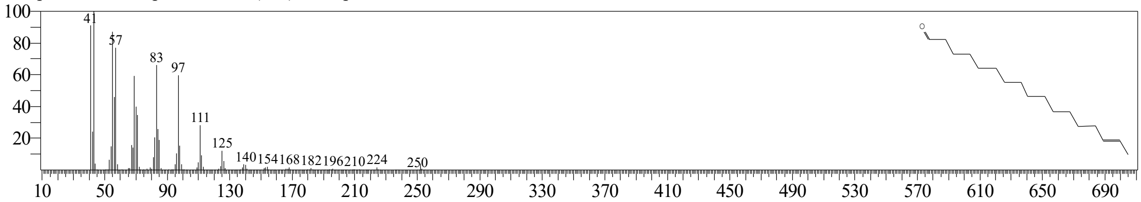

Hit#:3 Entry:70890 Library:NIST11.lib
SI:93 Formula:C16H30O CAS:330207-53-9 MolWeight:238 RetIndex:1808
CompName:E-14-Hexadecenal $$ (14E)-14-Hexadecenal $$ 14-Hexadecenal, (E)- $$

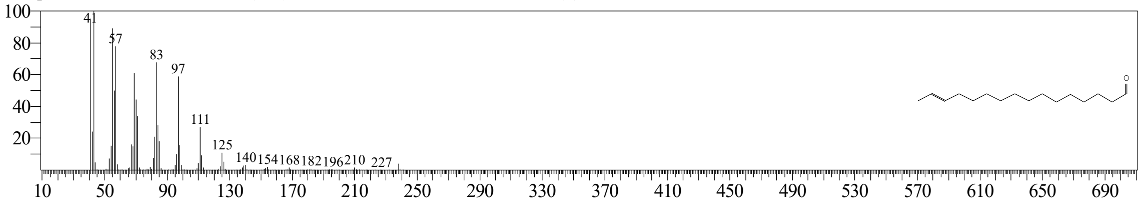

Hit#:4 Entry:60964 Library:NIST11.lib
SI:93 Formula:C16H32 CAS:629-73-2 MolWeight:224 RetIndex:1602
CompName:Cetene $$ 1-Hexadecene $$ .alpha.-Hexadecene $$ n-Hexadec-1-ene $$ 1-Cetene $$ Hexadecylene-1 $$ Hexadec-1-ene $$ Hexadecene-1 $$ Neode

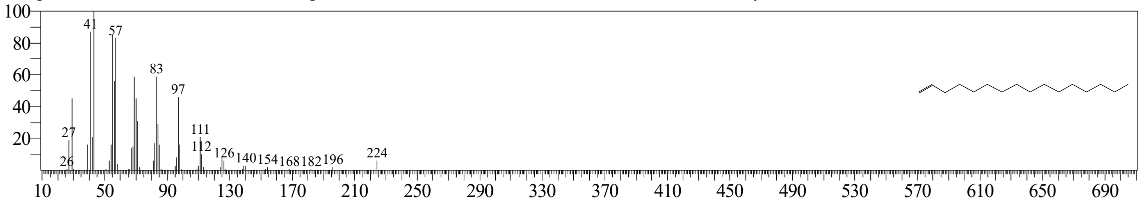

Hit#:5 Entry:51049 Library:NIST11.lib
SI:93 Formula:C15H30 CAS:13360-61-7 MolWeight:210 RetIndex:1502
CompName:1-Pentadecene $$ Pentadecene,1- $$ Pentadec-1-ene $$

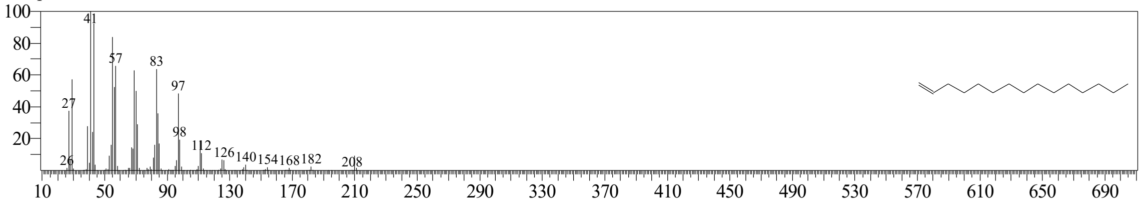

<< Target >>
Line#:53 R.Time:12.885(Scan#:1778) MassPeaks:396
RawMode:Averaged 12.880-12.890(1777-1779) BasePeak:43.10(524330)
BG Mode:Calc. from Peak Group 1 - Event 1 Scan

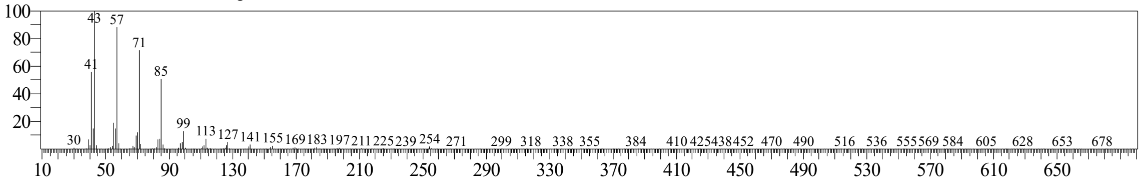

Hit#:1 Entry:62482 Library:NIST11.lib
SI:95 Formula:C16H34 CAS:55045-10-8 MolWeight:226 RetIndex:1548
CompName:Tridecane, 6-propyl- $$ 6-Propyltridecane # $$

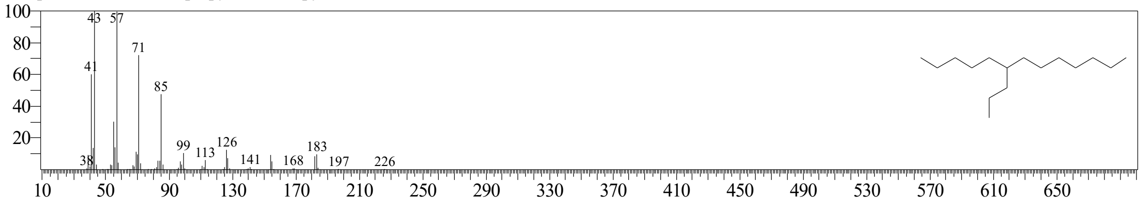

Hit#:2 Entry:62481 Library:NIST11.lib
SI:95 Formula:C16H34 CAS:55045-09-5 MolWeight:226 RetIndex:1548
CompName:Tridecane, 7-propyl- $$ 7-n-Propyltridecane $$ 7-Propyltridecane # $$

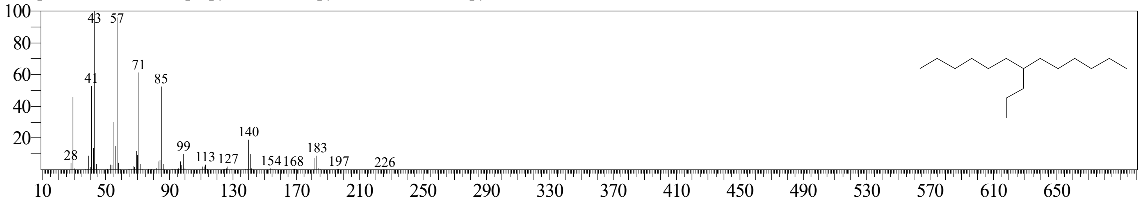

Hit#:3 Entry:82861 Library:NIST11.lib
SI:94 Formula:C18H38 CAS:593-45-3 MolWeight:254 RetIndex:1810
CompName:Octadecane $$ n-Octadecane $$ Octadecan $$

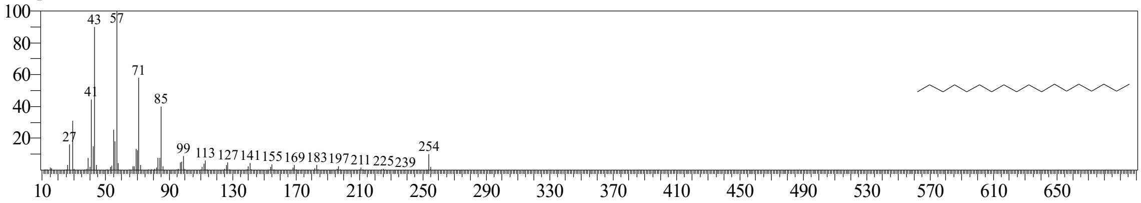

Hit#:4 Entry:42951 Library:NIST11.lib
SI:94 Formula:C14H30 CAS:6117-98-2 MolWeight:198 RetIndex:1285
CompName:2,3-Dimethyldodecane

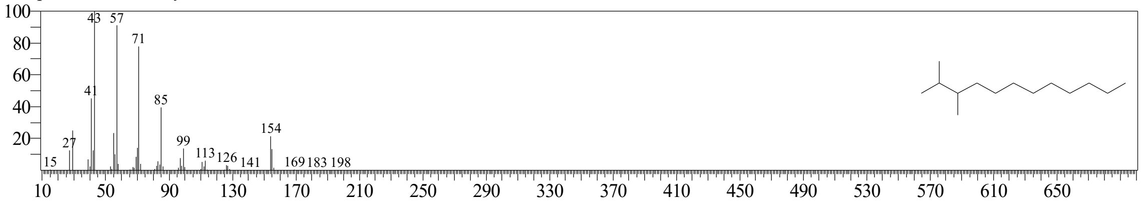

Hit#:5 Entry:93610 Library:NIST11.lib
SI:94 Formula:C19H40 CAS:7225-66-3 MolWeight:268 RetIndex:1846
CompName:Tridecane, 7-hexyl- $$ 7-Hexyltridecane $$

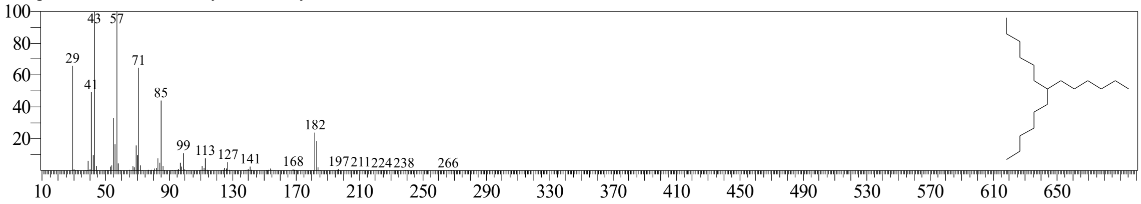

<< Target >>
Line#:54 R.Time:13.015(Scan#:1804) MassPeaks:379
RawMode:Averaged 13.010-13.020(1803-1805) BasePeak:32.10(4651)
BG Mode:Calc. from Peak Group 1 - Event 1 Scan

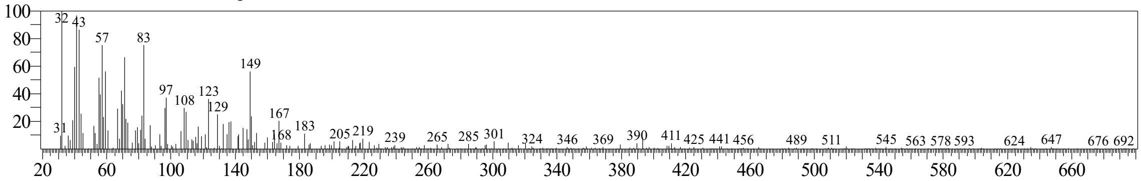

Hit#:1 Entry:27072 Library:NIST11.lib
SI:65 Formula:C10H20O2 CAS:0-00-0 MolWeight:172 RetIndex:1220
CompName:Citronellol epoxide (R or S) $$ 5-(3,3-Dimethyl-2-oxiranyl)-3-methyl-1-pentanol # $$

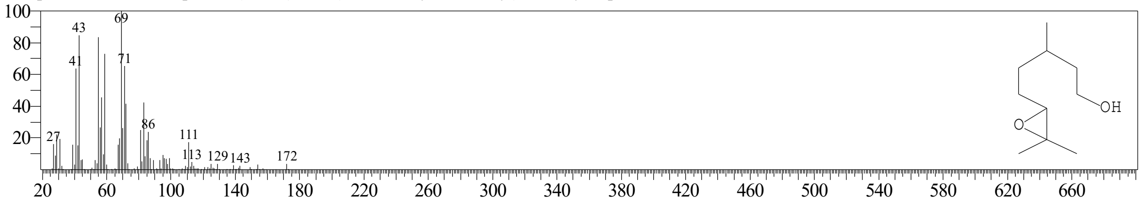

Hit#:2 Entry:137860 Library:NIST11.lib
SI:65 Formula:C12H24Br2 CAS:55334-42-4 MolWeight:326 RetIndex:1742
CompName:Dodecane, 1,2-dibromo- $$ 1,2-Dibromododecane $$

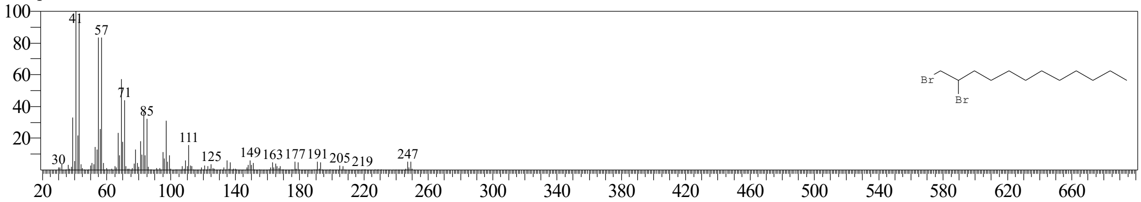

Hit#:3 Entry:172461 Library:NIST11.lib
SI:64 Formula:C26H50O CAS:56247-96-2 MolWeight:378 RetIndex:2785
CompName:4-Heneicosanone, 1-cyclopentyl- $$ 1-Cyclopentyl-4-henicosanone # $$

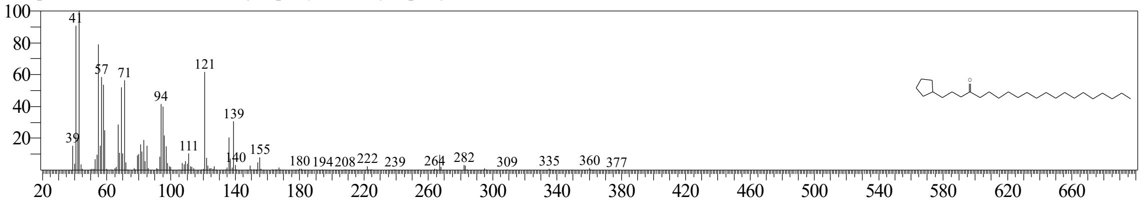

Hit#:4 Entry:41581 Library:NIST11.lib
SI:64 Formula:C13H24O CAS:7069-41-2 MolWeight:196 RetIndex:1510
CompName:2-Tridecenal, (E)- $$ (2E)-2-Tridecenal $$ (E)-2-Tridecenal $$ (E)-Tridec-2-enal $$

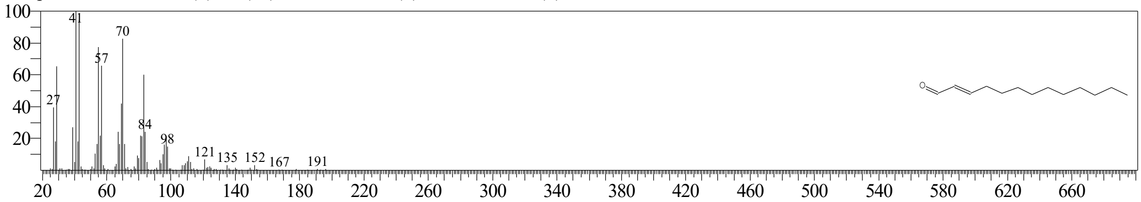

Hit#:5 Entry:35303 Library:NIST11.lib
SI:64 Formula:C12H26O CAS:91482-38-1 MolWeight:186 RetIndex:1329
CompName:1-Decanol, 5,9-dimethyl- $$ 5,9-Dimethyl-1-decanol # $$

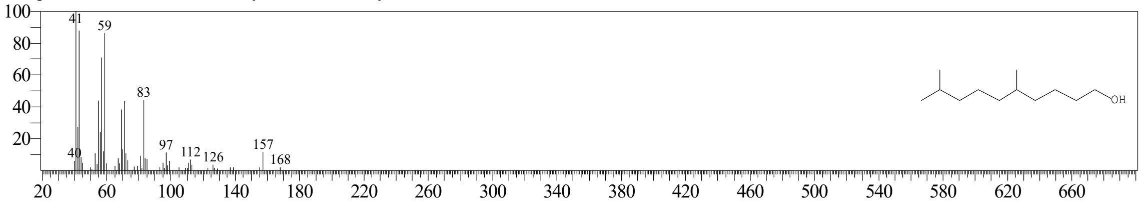

<< Target >>
Line#:55 R.Time:13.045(Scan#:1810) MassPeaks:326
RawMode:Averaged 13.040-13.050(1809-1811) BasePeak:32.10(6412)
BG Mode:Calc. from Peak Group 1 - Event 1 Scan

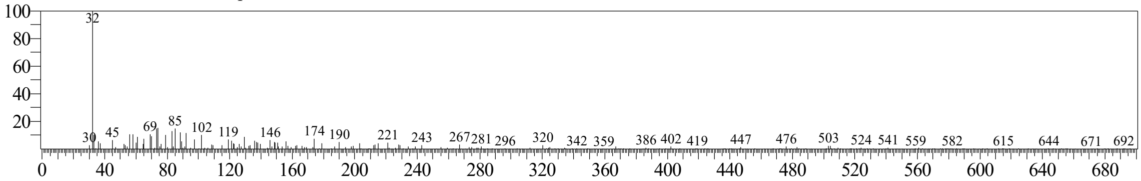

Hit#:1 Entry:5196 Library:NIST11.lib
SI:50 Formula:C4H8O4 CAS:23147-58-2 MolWeight:120 RetIndex:1074
CompName:Glycolaldehyde dimer $$ 1,4-Dioxane-2,5-diol # $$

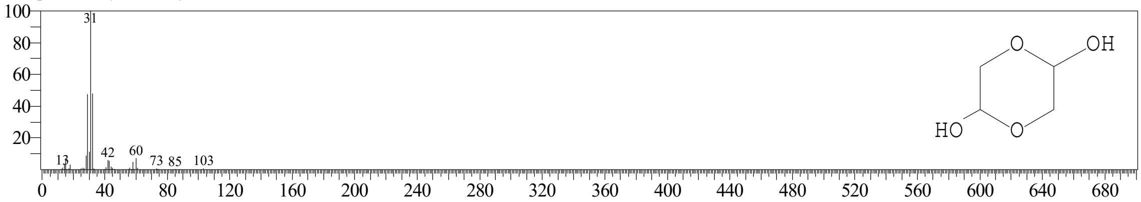

Hit#:2 Entry:1011 Library:NIST11.lib
SI:49 Formula:C4H12N2 CAS:32064-67-8 MolWeight:88 RetIndex:776
CompName:Hydrazine, (1,1-dimethylethyl)- $$ tert-Butylhydrazine $$ 1-tert-Butylhydrazine # $$

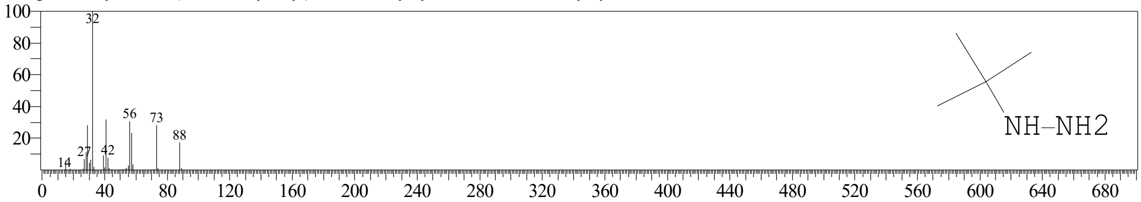

Hit#:3 Entry:956 Library:NIST11.lib
SI:47 Formula:C2H4N2O2 CAS:628-36-4 MolWeight:88 RetIndex:994
CompName:1,2-Hydrazinedicarboxaldehyde $$ Hydrazine, 1,2-diformyl- $$ s-Diformylhydrazine $$ Bicarbamaldehyde $$ Diformylhydrazine $$ N,N'-Diformyl

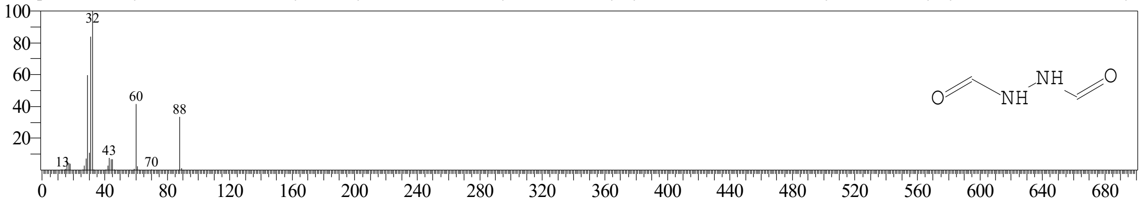

Hit#:4 Entry:117059 Library:NIST11.lib
SI:46 Formula:C18H38N2O CAS:4130-54-5 MolWeight:298 RetIndex:2454
CompName:Stearic acid hydrazide $$ Stearic hydrazide $$ Octadecanoic acid, hydrazide $$ Stearohydrazide $$ Stearoylhydrazine $$ Octadecanohydrazide # $$

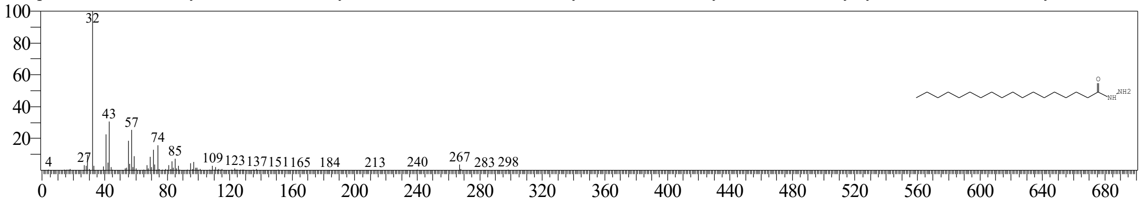

Hit#:5 Entry:2433 Library:NIST11.lib
SI:46 Formula:C3H8N2O2 CAS:24535-11-3 MolWeight:104 RetIndex:1205
CompName:Propanoic acid, 3-hydroxy-, hydrazide $$ 3-Hydroxypropanoic acid hydrazide $$ 3-Hydroxypropanohydrazide # $$

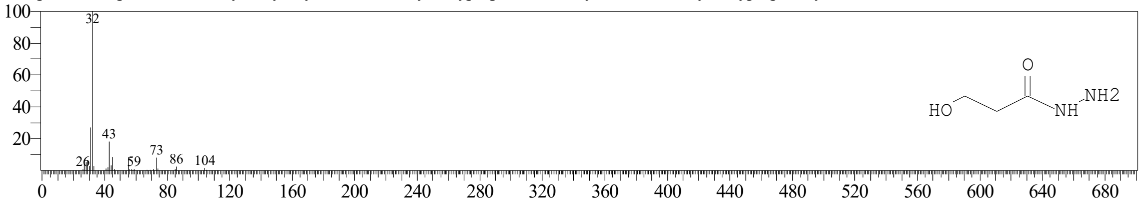

<< Target >>
Line#:56 R.Time:13.100(Scan#:1821) MassPeaks:327
RawMode:Averaged 13.095-13.105(1820-1822) BasePeak:69.00(2723)
BG Mode:Calc. from Peak Group 1 - Event 1 Scan

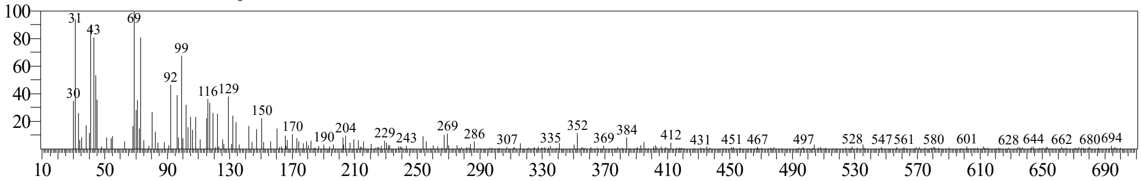

Hit#:1 Entry:4437 Library:NIST11.lib
SI:54 Formula:C4H8N2O2 CAS:3760-55-2 MolWeight:116 RetIndex:995
CompName:Pyrrolidine, 1-nitro- $$ 1-Nitropyrrolidine $$

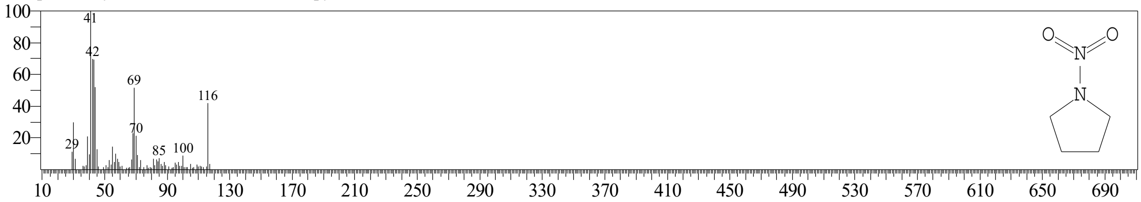

Hit#:2 Entry:111639 Library:NIST11.lib
SI:53 Formula:C11H14F2N2O5 CAS:102302-63-6 MolWeight:292 RetIndex:2101
CompName:1-(.beta.-d-2-Desoxyribofuranosyl)-4-O-difluormethyl-thymine

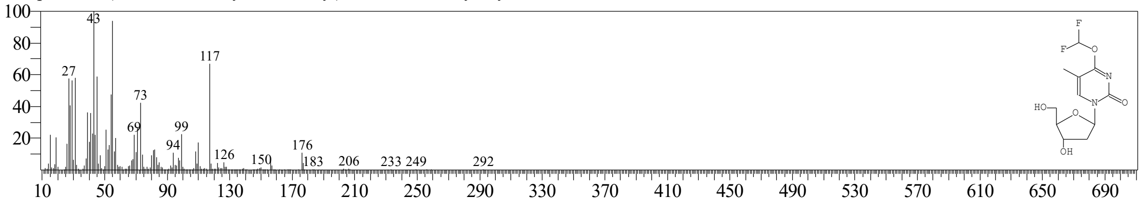

Hit#:3 Entry:34992 Library:NIST11.lib
SI:53 Formula:C10H18O3 CAS:36555-25-6 MolWeight:186 RetIndex:1592
CompName:2H-Pyran-2-one, tetrahydro-4-hydroxy-6-pentyl- $$ 4-Hydroxy-6-pentyltetrahydro-2H-pyran-2-one # $$

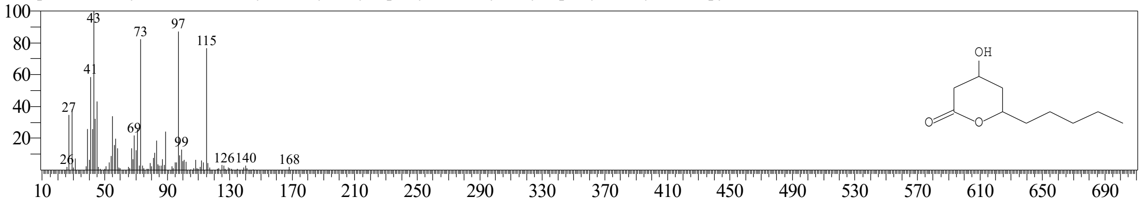

Hit#:4 Entry:7781 Library:NIST11.lib
SI:52 Formula:C6H10O3 CAS:124744-30-5 MolWeight:130 RetIndex:849
CompName:(2-Methyl-oxiran-2-yl)-acetic acid, methyl ester $$ Methyl (2-methyl-2-oxiranyl)acetate # $$

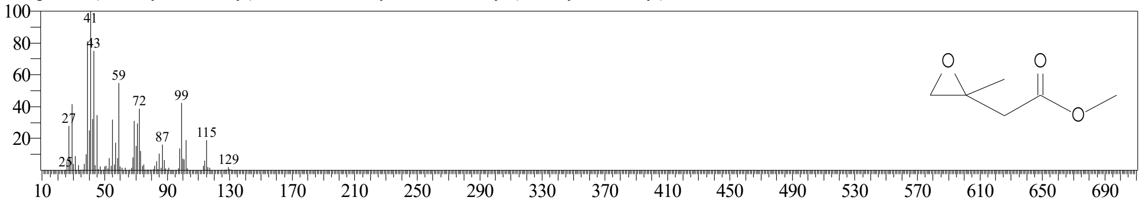

Hit#:5 Entry:11660 Library:NIST11.lib
SI:52 Formula:C8H15NO CAS:59346-56-4 MolWeight:141 RetIndex:1075
CompName:2,2,4-Trimethyl-3-hydroxy-n-valeronitrile $$ 3-Hydroxy-2,2,4-trimethylpentanenitrile # $$

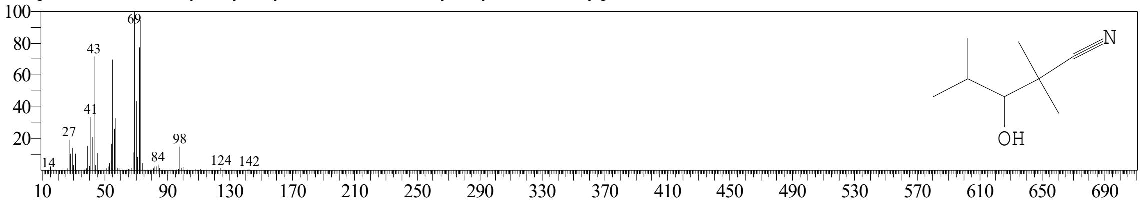

<< Target >>
Line#:57 R.Time:13.150(Scan#:1831) MassPeaks:382
RawMode:Averaged 13.145-13.155(1830-1832) BasePeak:43.05(9389)
BG Mode:Calc. from Peak Group 1 - Event 1 Scan

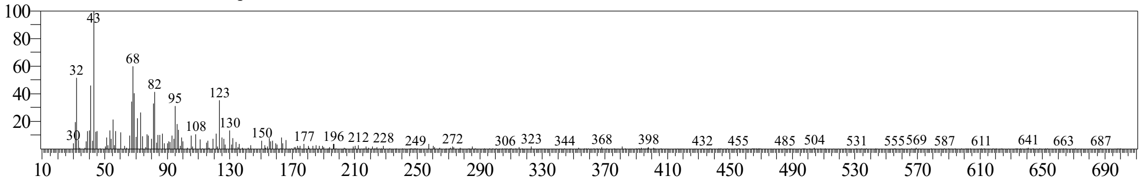

Hit#:1 Entry:62270 Library:NIST11.lib
SI:72 Formula:C14H26O2 CAS:38363-29-0 MolWeight:226 RetIndex:1588
CompName:8-Dodecen-1-ol, acetate, (E)- $$ (E)-8-Dodecen-1-ol acetate $$ E-8-Dodecen-1-yl acetate $$ E-8-Dodecenyl acetate $$ (8E)-8-Dodecenyl acetate #

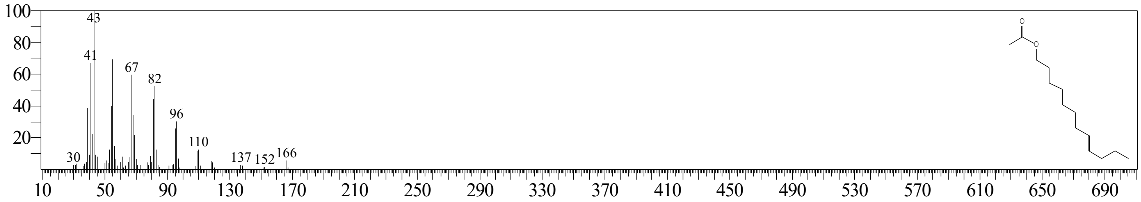

Hit#:2 Entry:25784 Library:NIST11.lib
SI:71 Formula:C10H18O2 CAS:26488-97-1 MolWeight:170 RetIndex:1471
CompName:trans,trans-2,6-Dimethyl-2,6-octadiene-1,8-diol $$ (2E,6E)-2,6-Dimethyl-2,6-octadiene-1,8-diol # $$

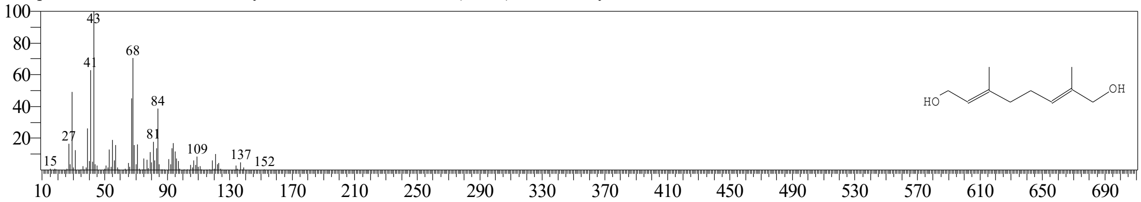

Hit#:3 Entry:33870 Library:NIST11.lib
SI:71 Formula:C11H20O2 CAS:76238-22-7 MolWeight:184 RetIndex:1290
CompName:6-Nonen-1-ol, acetate, (Z)- $$ (6Z)-6-Nonenyl acetate $$ (Z)-6 Nonen-1-yl acetate $$ (Z)-6-Nonenyl acetate $$ (Z)-Non-6-en-1-yl acetate $$

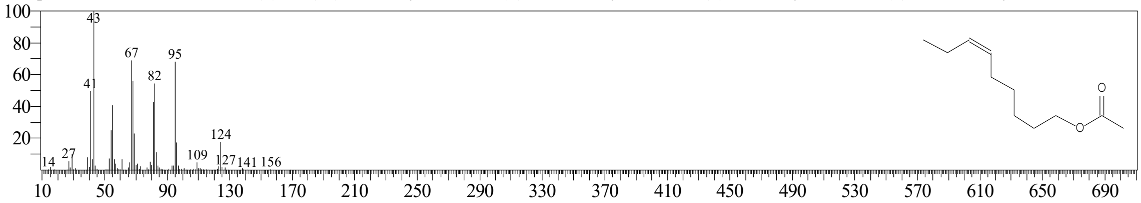

Hit#:4 Entry:31743 Library:NIST11.lib
SI:71 Formula:C13H24 CAS:28467-75-6 MolWeight:180 RetIndex:1331
CompName:2-Tridecyne $$ 2-C13H24 $$

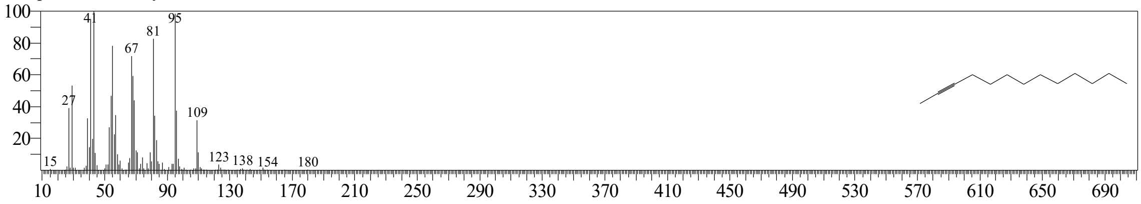

Hit#:5 Entry:82675 Library:NIST11.lib
SI:70 Formula:C16H30O2 CAS:23192-82-7 MolWeight:254 RetIndex:1787
CompName:9-Tetradecen-1-ol, acetate, (E)- $$ (E)-9-Tetradecen-1-ol acetate $$ E-9-Tetradecen-1-yl acetate $$ E-9-Tetradecenyl acetate $$ (9E)-9-Tetradecenyl

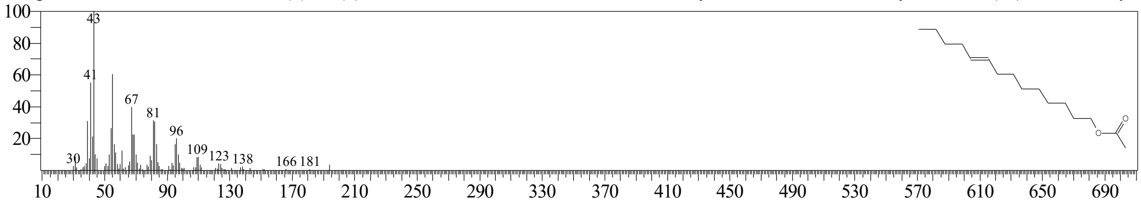

<< Target >>
Line#:58 R.Time:13.205(Scan#:1842) MassPeaks:353
RawMode:Averaged 13.200-13.210(1841-1843) BasePeak:43.00(6488)
BG Mode:Calc. from Peak Group 1 - Event 1 Scan

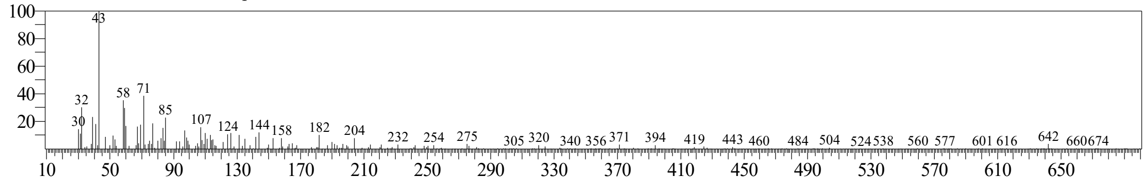

Hit#:1 Entry:6520 Library:NIST11.lib
SI:63 Formula:C8H14O CAS:3212-16-6 MolWeight:126 RetIndex:953
CompName:endo-2-Methyl-2-norbornanol $$ endo-2-Methyl-2-norborneol $$ 2-exo-Methyl-2-endo-norbornanol $$ 2-Methyl-endo-norbornanol $$ 2-Norborna

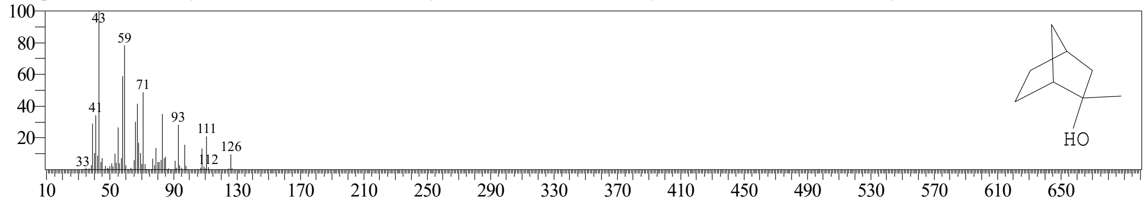

Hit#:2 Entry:6521 Library:NIST11.lib
SI:61 Formula:C8H14O CAS:1122-98-1 MolWeight:126 RetIndex:995
CompName:2-Propanone, 1-cyclopentyl- $$ Cyclopentylacetone $$ 3-Cyclopentylpropanone $$ Cyclopentyl-2-propanone $$ 1-Cyclopentyl-2-propanone $$ 2-I

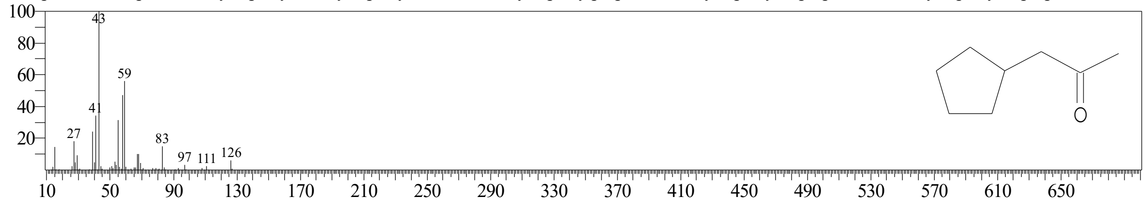

Hit#:3 Entry:6519 Library:NIST11.lib
SI:61 Formula:C8H14O CAS:3664-60-6 MolWeight:126 RetIndex:943
CompName:7-Octen-2-one

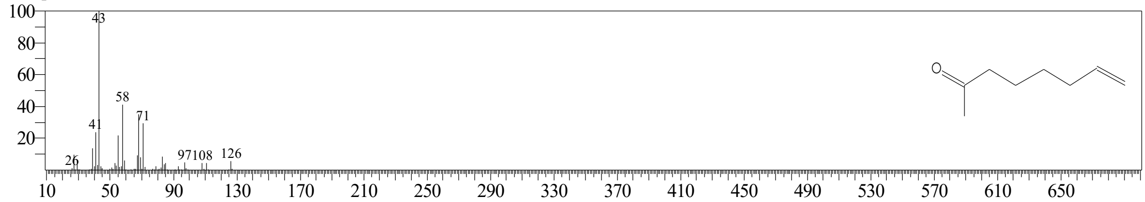

Hit#:4 Entry:34980 Library:NIST11.lib
SI:61 Formula:C10H18O3 CAS:0-00-0 MolWeight:186 RetIndex:1224
CompName:Epoxy-linalooloxide

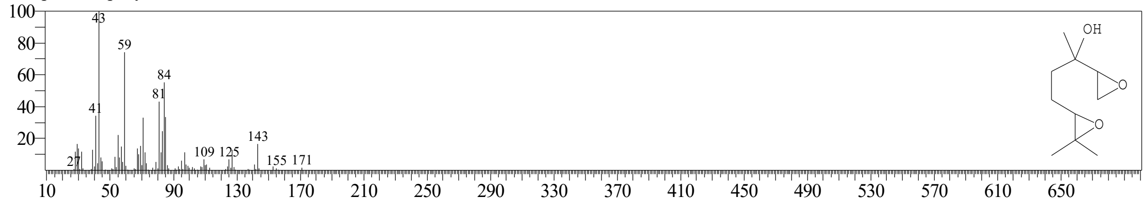

Hit#:5 Entry:12928 Library:NIST11.lib
SI:61 Formula:C8H16O2 CAS:0-00-0 MolWeight:144 RetIndex:1115
CompName:6-hydroxy-4-octanone

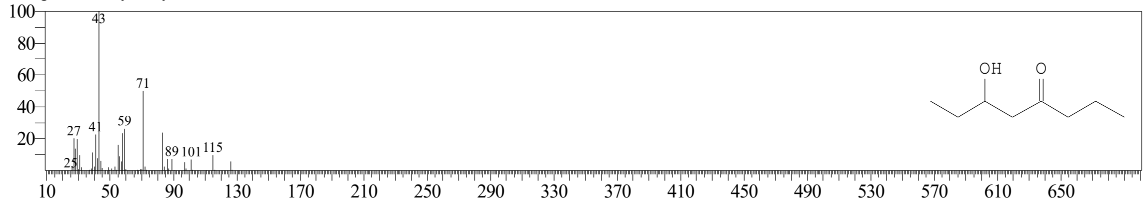

<< Target >>
Line#:59 R.Time:13.295(Scan#:1860) MassPeaks:390
RawMode:Averaged 13.290-13.300(1859-1861) BasePeak:43.05(37656)
BG Mode:Calc. from Peak Group 1 - Event 1 Scan

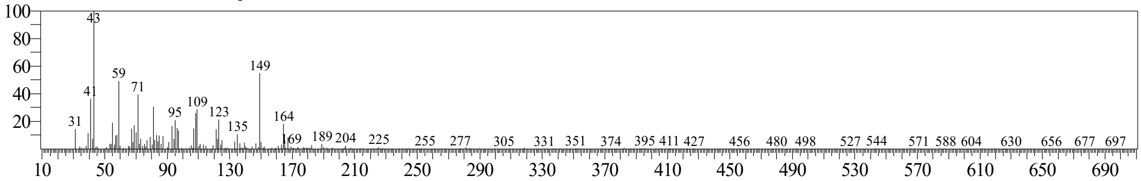

Hit#:1 Entry:60792 Library:NIST11.lib
SI:78 Formula:C14H24O2 CAS:72294-84-9 MolWeight:224 RetIndex:1497
CompName:3-Buten-2-ol, 2-methyl-4-(1,3,3-trimethyl-7-oxabicyclo[4.1.0]hept-2-yl)- $$ (3E)-2-Methyl-4-(1,3,3-trimethyl-7-oxabicyclo[4.1.0]hept-2-yl)-3-bute

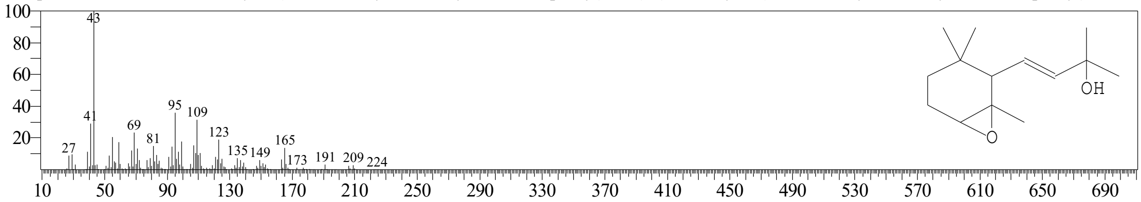

Hit#:2 Entry:59380 Library:NIST11.lib
SI:76 Formula:C15H26O CAS:473-15-4 MolWeight:222 RetIndex:1593
CompName:2-Naphthalenemethanol, decahydro-.alpha.,.alpha.,4a-trimethyl-8-methylene-, [2R-(2.alpha.,4a.alpha.,8a.beta.)]- $$ Eudesm-4(14)-en-11-ol $$ .beta

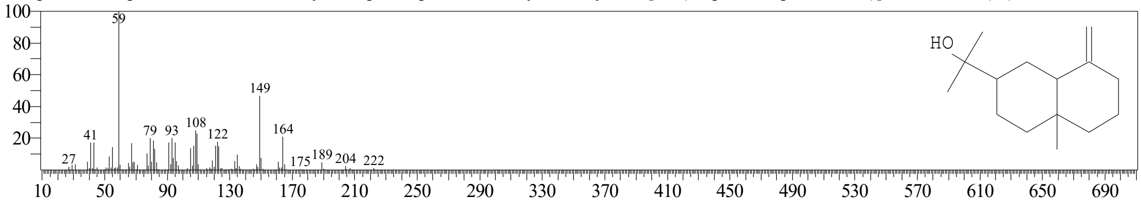

Hit#:3 Entry:72318 Library:NIST11.lib
SI:76 Formula:C15H28O2 CAS:60714-01-4 MolWeight:240 RetIndex:1766
CompName:2-Methyl-5-(2,6,6-trimethyl-cyclohex-1-enyl)-pentane-2,3-diol $$ 2-Methyl-5-(2,6,6-trimethyl-1-cyclohexen-1-yl)-2,3-pentanediol # $$

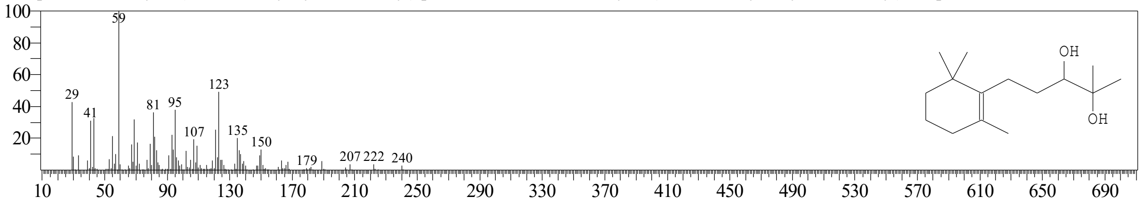

Hit#:4 Entry:70772 Library:NIST11.lib
SI:75 Formula:C15H26O2 CAS:0-00-0 MolWeight:238 RetIndex:1751
CompName:9-(3,3-Dimethyloxiran-2-yl)-2,7-dimethylnona-2,6-dien-1-ol $$ (2E,6E)-9-(3,3-Dimethyl-2-oxiranyl)-2,7-dimethyl-2,6-nonadien-1-ol # $$

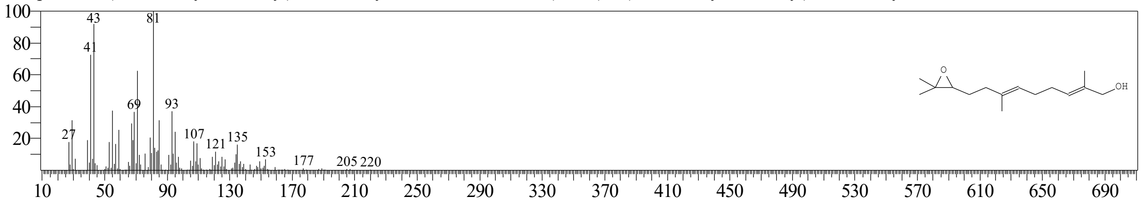

Hit#:5 Entry:17472 Library:NIST11.lib
SI:74 Formula:C10H18O CAS:4028-60-8 MolWeight:154 RetIndex:1099
CompName:Cyclopentanol, 1,2-dimethyl-3-(1-methylethenyl)-, [1R-(1.alpha.,2.alpha.,3.alpha.)]- $$ Cyclopentanol, 3-isopropenyl-1,2-dimethyl-, (1R,2S,3S)-(-

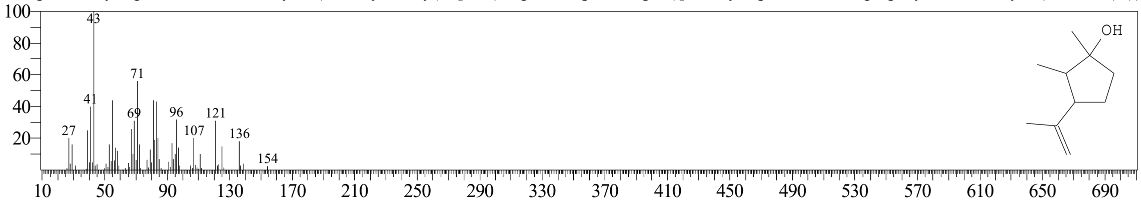

<< Target >>
Line#:60 R.Time:13.415(Scan#:1884) MassPeaks:385
RawMode:Averaged 13.410-13.420(1883-1885) BasePeak:41.00(8003)
BG Mode:Calc. from Peak Group 1 - Event 1 Scan

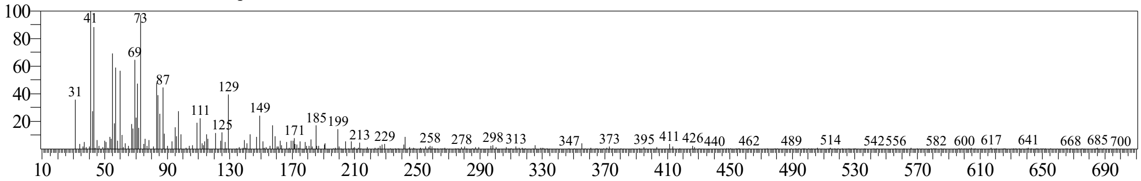

Hit#:1 Entry:73851 Library:NIST11.lib
SI:81 Formula:C15H30O2 CAS:1002-84-2 MolWeight:242 RetIndex:1869
CompName:Pentadecanoic acid $$ Pentadecylic acid $$ n-Pentadecanoic acid $$ n-Pentadecylic acid $$

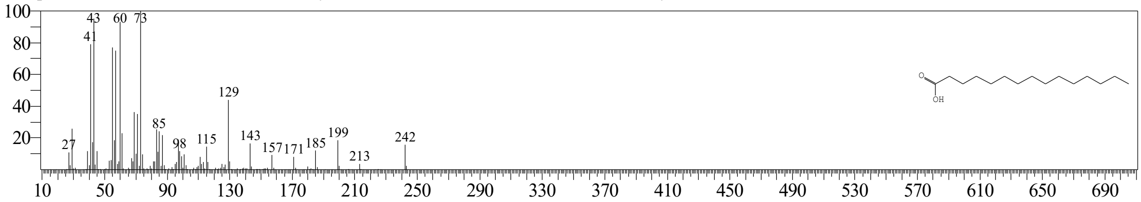

Hit#:2 Entry:128151 Library:NIST11.lib
SI:79 Formula:C20H40O2 CAS:506-30-9 MolWeight:312 RetIndex:2366
CompName:Eicosanoic acid $$ Arachic acid $$ Arachidic acid $$ Icosanoic acid $$ n-Eicosanoic acid $$ Arachidic acid (synthetic) $$

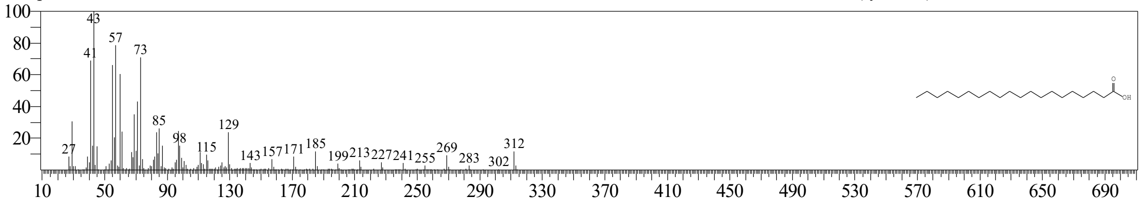

Hit#:3 Entry:106158 Library:NIST11.lib
SI:79 Formula:C18H36O2 CAS:57-11-4 MolWeight:284 RetIndex:2167
CompName:Octadecanoic acid $$ Stearic acid $$ n-Octadecanoic acid $$ Humko Industrene R $$ Hydrofol Acid 150 $$ Hystrene S-97 $$ Hystrene T-70 $$ Hys

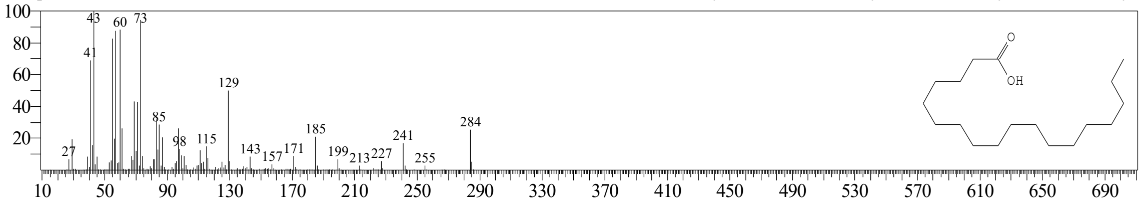

Hit#:4 Entry:63810 Library:NIST11.lib
SI:79 Formula:C14H28O2 CAS:544-63-8 MolWeight:228 RetIndex:1769
CompName:Tetradecanoic acid $$ Myristic acid $$ n-Tetradecanoic acid $$ n-Tetradecoic acid $$ Neo-Fat 14 $$ Univol U 316S $$ 1-Tridecanecarboxylic acid $

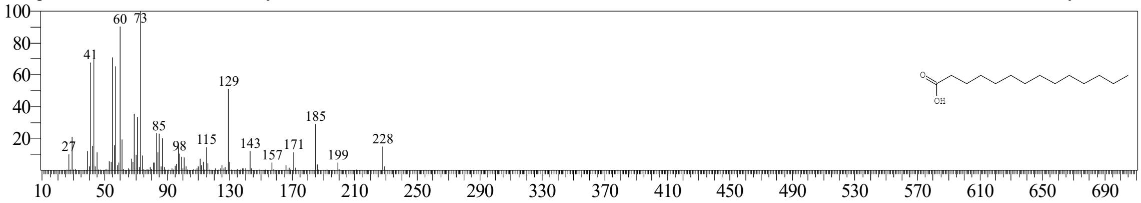

Hit#:5 Entry:53599 Library:NIST11.lib
SI:78 Formula:C13H26O2 CAS:638-53-9 MolWeight:214 RetIndex:1670
CompName:Tridecanoic acid $$ n-Tridecanoic acid $$ n-Tridecoic acid $$ Tridecylic acid $$

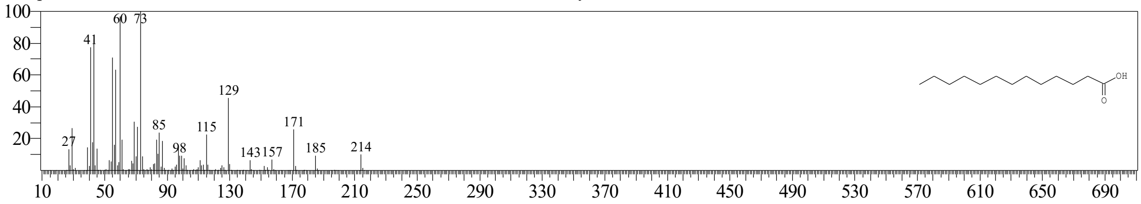

<< Target >>
Line#:61 R.Time:13.545(Scan#:1910) MassPeaks:403
RawMode:Averaged 13.540-13.550(1909-1911) BasePeak:88.10(44307)
BG Mode:Calc. from Peak Group 1 - Event 1 Scan

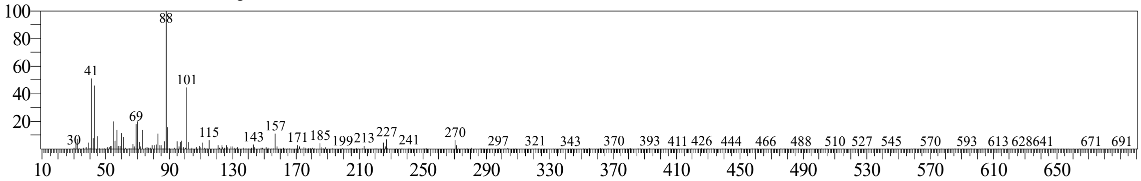

Hit#:1 Entry:95197 Library:NIST11.lib
SI:89 Formula:C17H34O2 CAS:41114-00-5 MolWeight:270 RetIndex:1878
CompName:Pentadecanoic acid, ethyl ester $$ Ethyl pentadecanoate $$ n-Pentadecanoic acid ethyl ester $$

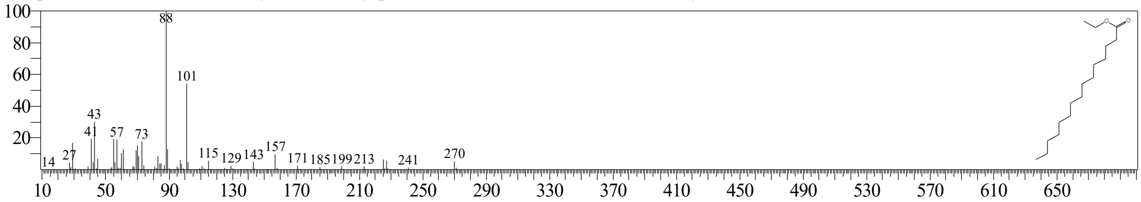

Hit#:2 Entry:149096 Library:NIST11.lib
SI:86 Formula:C22H44O2 CAS:18281-05-5 MolWeight:340 RetIndex:2375
CompName:Eicosanoic acid, ethyl ester $$ Ethyl icosanoate $$ Ethyl arachidate $$ Ethyl eicosanoate $$ Arachidic acid, ethyl ester $$

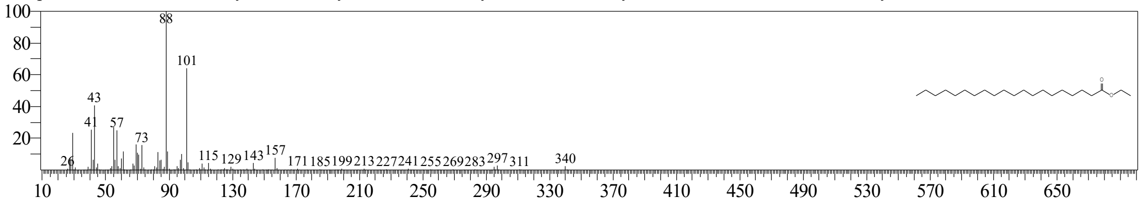

Hit#:3 Entry:44020 Library:NIST11.lib
SI:86 Formula:C12H24O2 CAS:116530-40-6 MolWeight:200 RetIndex:1317
CompName:Nonanoic acid, 5-methyl-, ethyl ester $$ Ethyl 5-methylnonanoate # $$

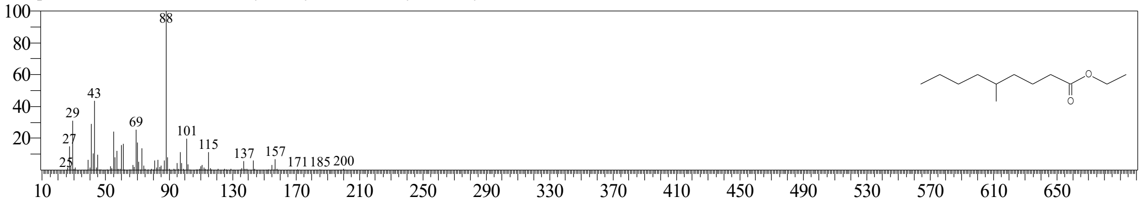

Hit#:4 Entry:53613 Library:NIST11.lib
SI:86 Formula:C13H26O2 CAS:627-90-7 MolWeight:214 RetIndex:1481
CompName:Undecanoic acid, ethyl ester $$ Ethyl undecanoate $$ Ethyl undecylate $$ n-Undecanoic acid ethyl ester $$ Ethyl n-undecanoate $$

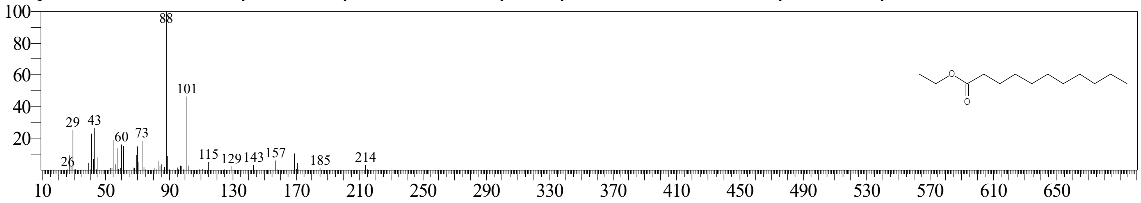

Hit#:5 Entry:117163 Library:NIST11.lib
SI:85 Formula:C19H38O2 CAS:14010-23-2 MolWeight:298 RetIndex:2077
CompName:Heptadecanoic acid, ethyl ester $$ Ethyl heptadecanoate $$ Ethyl n-heptadecanoate $$

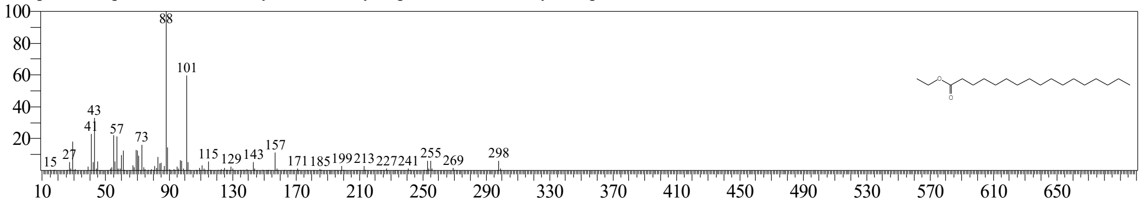

<< Target >>
Line#:62 R.Time:13.585(Scan#:1918) MassPeaks:334
RawMode:Averaged 13.580-13.590(1917-1919) BasePeak:57.10(10233)
BG Mode:Calc. from Peak Group 1 - Event 1 Scan

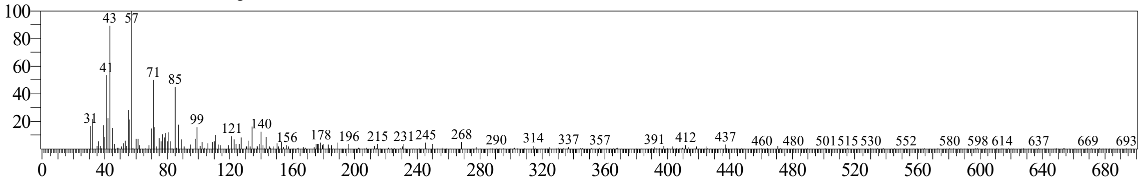

Hit#:1 Entry:19561 Library:NIST11.lib
SI:72 Formula:C10H22O CAS:10042-59-8 MolWeight:158 RetIndex:1194
CompName:1-Heptanol, 2-propyl- $$ 2-Propylheptanol $$ 2-Propyl-1-heptanol # $$

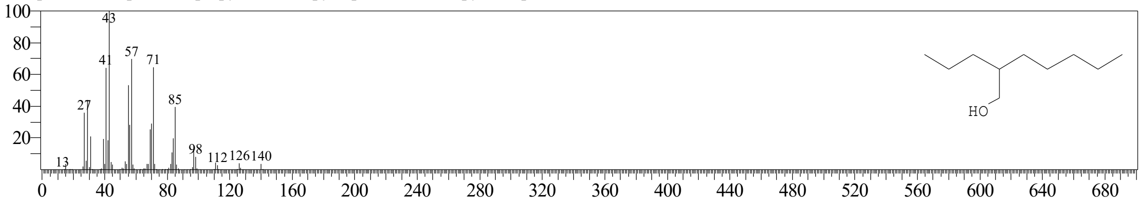

Hit#:2 Entry:27215 Library:NIST11.lib
SI:72 Formula:C11H24O CAS:54461-00-6 MolWeight:172 RetIndex:1273
CompName:1-Hexanol, 2-ethyl-2-propyl- $$ 2-Ethyl-2-propyl-1-hexanol $$

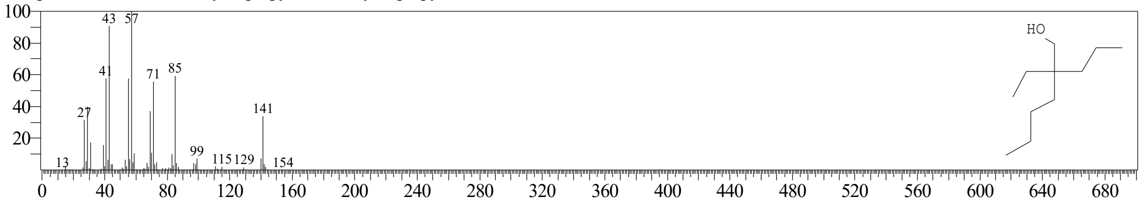

Hit#:3 Entry:47433 Library:NIST11.lib
SI:72 Formula:C9H19Br CAS:2216-35-5 MolWeight:206 RetIndex:1148
CompName:2-Bromononane $$ sec-Nonyl bromide $$ Nonane, 2-bromo- $$

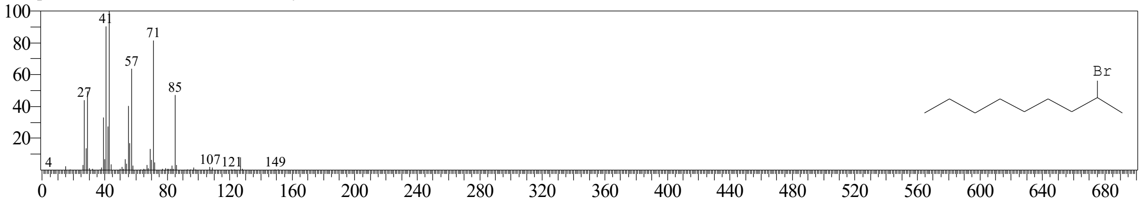

Hit#:4 Entry:18585 Library:NIST11.lib
SI:72 Formula:C10H20O CAS:35778-39-3 MolWeight:156 RetIndex:1087
CompName:3-Methyl-4-nonanone

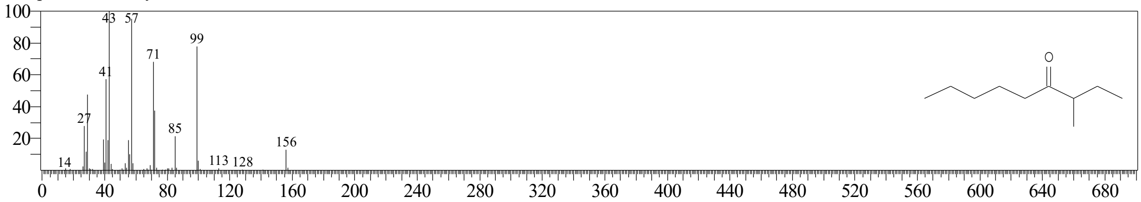

Hit#:5 Entry:19560 Library:NIST11.lib
SI:71 Formula:C10H22O CAS:2370-14-1 MolWeight:158 RetIndex:1173
CompName:1-Octanol, 2,2-dimethyl- $$ 2,2-Dimethyl-1-octanol $$ 2,2-Dimethyloctanol $$

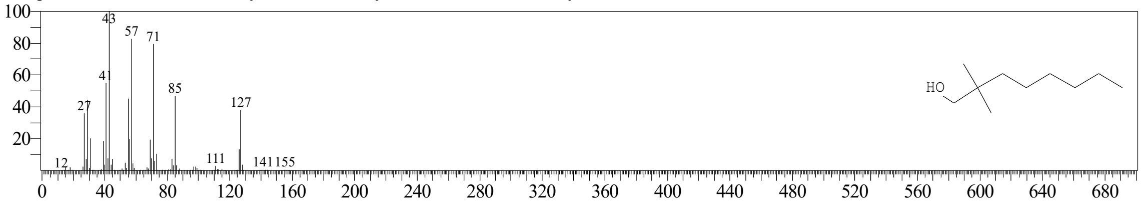

<< Target >>
Line#:63 R.Time:13.765(Scan#:1954) MassPeaks:405
RawMode:Averaged 13.760-13.770(1953-1955) BasePeak:74.05(117420)
BG Mode:Calc. from Peak Group 1 - Event 1 Scan

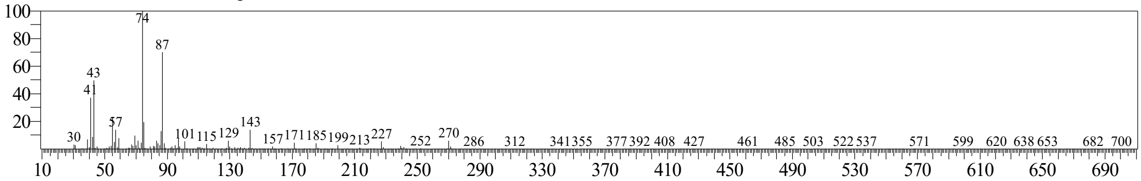

Hit#:1 Entry:95189 Library:NIST11.lib
SI:92 Formula:C17H34O2 CAS:5129-60-2 MolWeight:270 RetIndex:1814
CompName:Pentadecanoic acid, 14-methyl-, methyl ester $$ Methyl 14-methylpentadecanoate # $$ Methyl isohexadecanoate $$

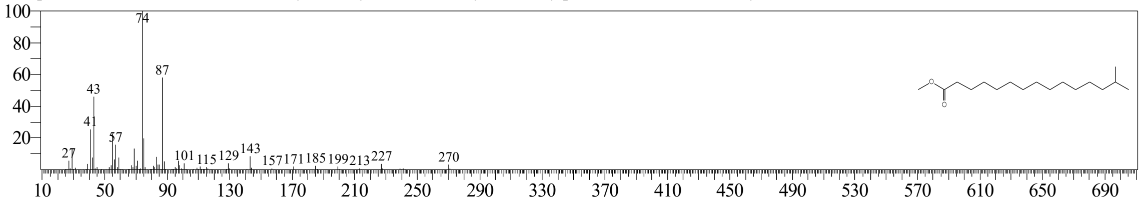

Hit#:2 Entry:95188 Library:NIST11.lib
SI:91 Formula:C17H34O2 CAS:112-39-0 MolWeight:270 RetIndex:1878
CompName:Hexadecanoic acid, methyl ester $$ Palmitic acid, methyl ester $$ n-Hexadecanoic acid methyl ester $$ Metholene 2216 $$ Methyl hexadecanoate $

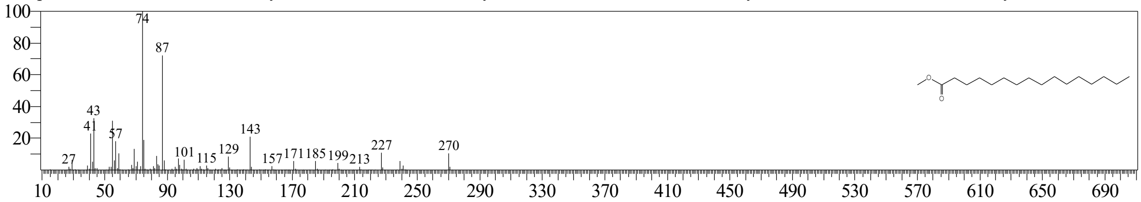

Hit#:3 Entry:106180 Library:NIST11.lib
SI:90 Formula:C18H36O2 CAS:6929-04-0 MolWeight:284 RetIndex:1914
CompName:Hexadecanoic acid, 15-methyl-, methyl ester $$ Methyl isoheptadecanoate $$ Methyl 15-methylhexadecanoate $$

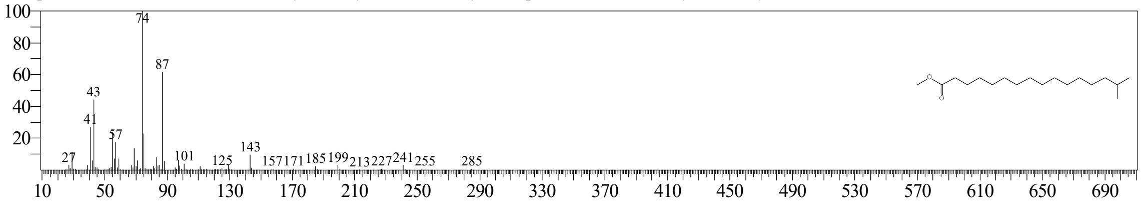

Hit#:4 Entry:73852 Library:NIST11.lib
SI:89 Formula:C15H30O2 CAS:5129-58-8 MolWeight:242 RetIndex:1615
CompName:Tridecanoic acid, 12-methyl-, methyl ester $$ Methyl isomyristate $$ Methyl 12-methyltridecanoate $$

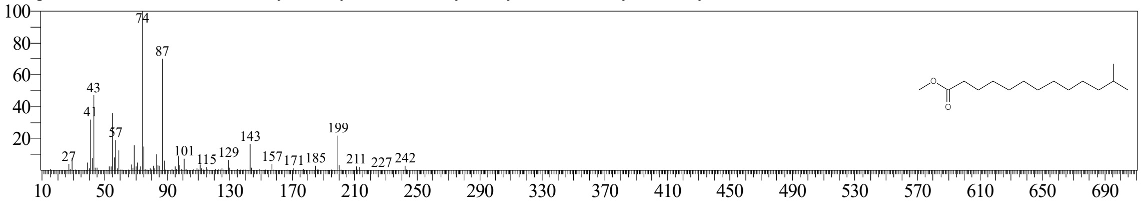

Hit#:5 Entry:84388 Library:NIST11.lib
SI:88 Formula:C16H32O2 CAS:7132-64-1 MolWeight:256 RetIndex:1779
CompName:Pentadecanoic acid, methyl ester $$ Methyl n-pentadecanoate $$ Methyl pentadecanoate $$ n-Pentadecanoic acid methyl ester $$

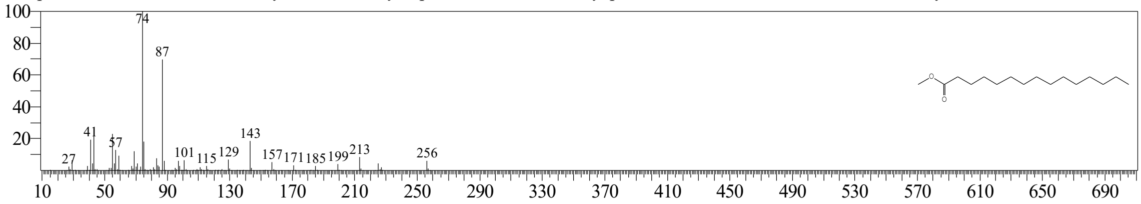

<< Target >>
Line#:64 R.Time:13.855(Scan#:1972) MassPeaks:375
RawMode:Averaged 13.850-13.860(1971-1973) BasePeak:277.20(7046)
BG Mode:Calc. from Peak Group 1 - Event 1 Scan

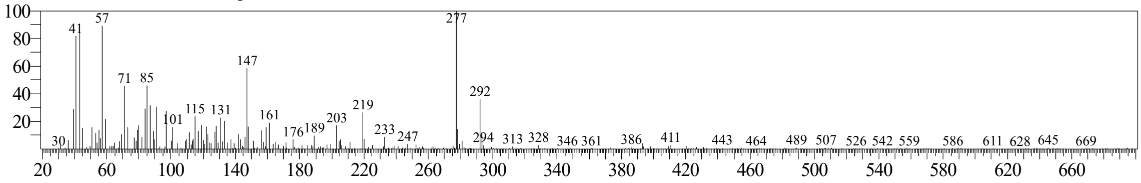

Hit#:1 Entry:112373 Library:NIST11.lib
SI:63 Formula:C18H28O3 CAS:6386-38-5 MolWeight:292 RetIndex:2134
CompName:Benzenepropanoic acid, 3,5-bis(1,1-dimethylethyl)-4-hydroxy-, methyl ester $$ Methyl 3-(3,5-di-tert-butyl-4-hydroxyphenyl)propionate $$ Methyl

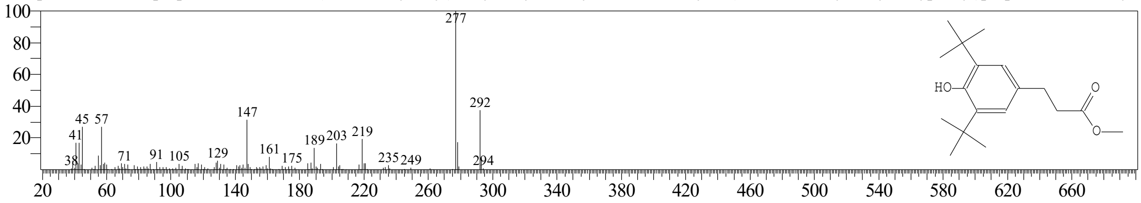

Hit#:2 Entry:35143 Library:NIST11.lib
SI:57 Formula:C11H22O2 CAS:54798-83-3 MolWeight:186 RetIndex:1197
CompName:Hexanoic acid, 2-methyl-2-propyl-, methyl ester $$ 2-Methyl-2-propylhexanoic acid methyl ester $$ Methyl 2-methyl-2-propylhexanoate # $$

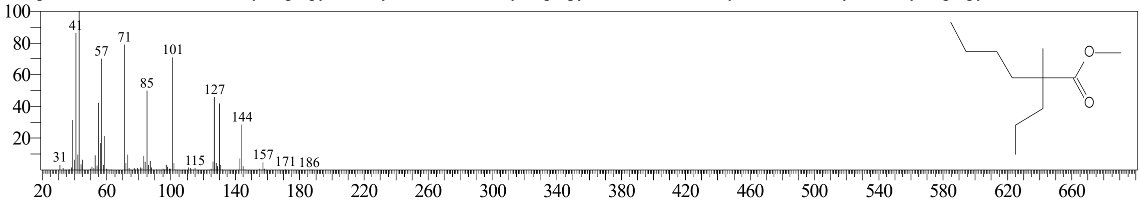

Hit#:3 Entry:103791 Library:NIST11.lib
SI:57 Formula:C12H26O5S CAS:0-00-0 MolWeight:282 RetIndex:2357
CompName:d-Galactitol, 1-thiohexyl-1-deoxy-- $$ 1-S-Hexyl-1-thio-d-galactitol # $$

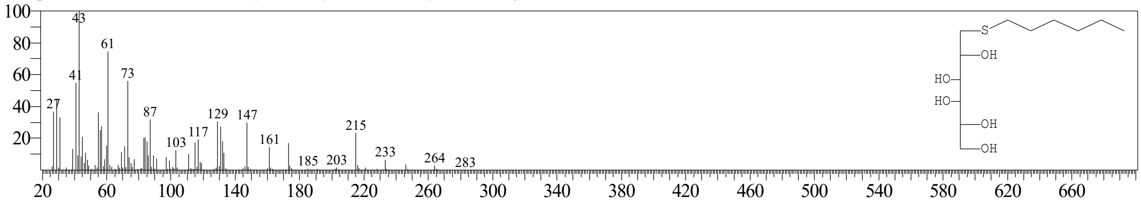

Hit#:4 Entry:125921 Library:NIST11.lib
SI:57 Formula:C14H30O5S CAS:0-00-0 MolWeight:310 RetIndex:2556
CompName:d-Mannitol, 1-thiooctyl-1-deoxy- $$ 1-S-Octyl-1-thio-d-mannitol # $$

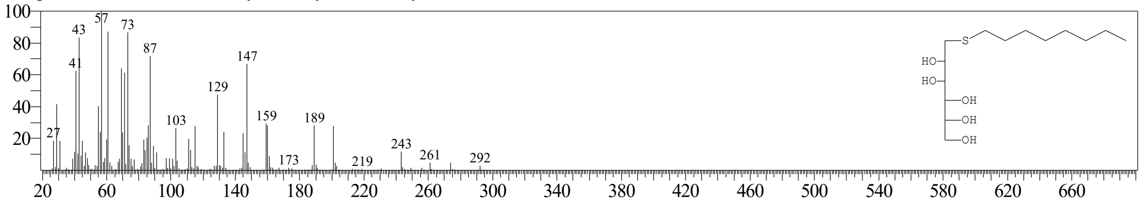

Hit#:5 Entry:187893 Library:NIST11.lib
SI:56 Formula:C22H31F3O4 CAS:0-00-0 MolWeight:416 RetIndex:2459
CompName:Dimethylmalonic acid, 2,3,4-trifluorophenyl undecyl ester

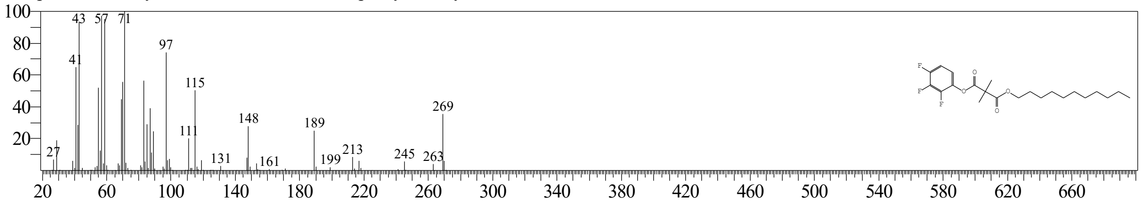

<< Target >>
Line#:65 R.Time:13.970(Scan#:1995) MassPeaks:410
RawMode:Averaged 13.965-13.975(1994-1996) BasePeak:41.05(53650)
BG Mode:Calc. from Peak Group 1 - Event 1 Scan

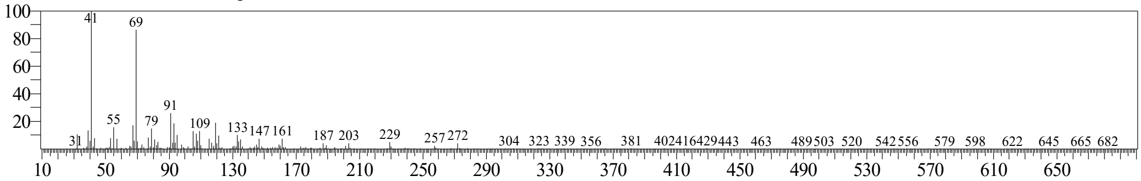

Hit#:1 Entry:46578 Library:NIST11.lib
SI:81 Formula:C15H24 CAS:77129-48-7 MolWeight:204 RetIndex:1440
CompName:1,6,10-Dodecatriene, 7,11-dimethyl-3-methylene- $$ 7,11-Dimethyl-3-methylene-1,6,10-dodecatriene $$

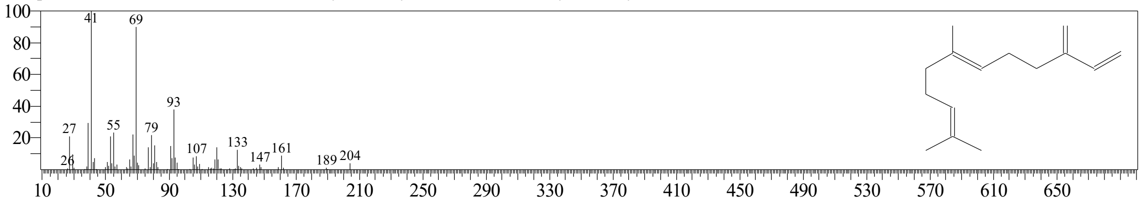

Hit#:2 Entry:110903 Library:NIST11.lib
SI:81 Formula:C20H34O CAS:1113-21-9 MolWeight:290 RetIndex:2046
CompName:1,6,10,14-Hexadecatetraen-3-ol, 3,7,11,15-tetramethyl-, (E,E)- $$ (6E,10E)-3,7,11,15-Tetramethyl-1,6,10,14-hexadecatetraen-3-ol # $$ Geranyl lina

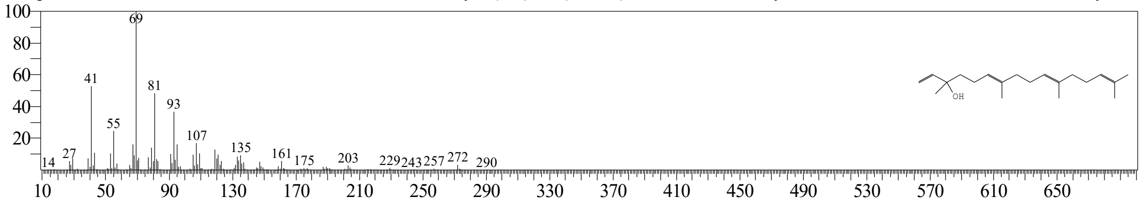

Hit#:3 Entry:57745 Library:NIST11.lib
SI:80 Formula:C15H24O CAS:2387-68-0 MolWeight:220 RetIndex:1572
CompName:6,10-Dodecadien-1-yn-3-ol, 3,7,11-trimethyl- $$ Deca-4,8-dienol, 1-ethynyl-1,5,9-trimethyl- $$ (6E)-3,7,11-Trimethyl-6,10-dodecadien-1-yn-3-ol #

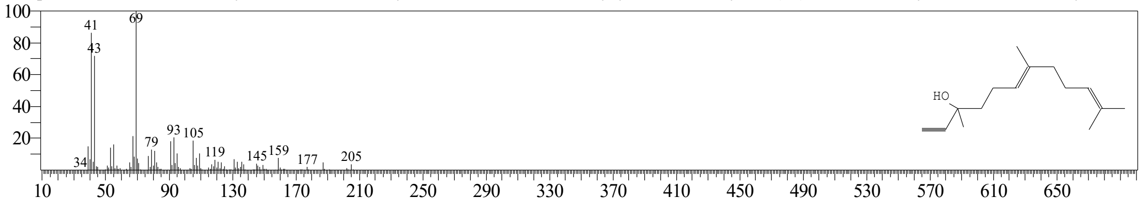

Hit#:4 Entry:96991 Library:NIST11.lib
SI:80 Formula:C20H32 CAS:38259-79-9 MolWeight:272 RetIndex:1958
CompName:2,6,11,15-Tetramethyl-hexadeca-2,6,8,10,14-pentaene $$ (6E,8E,10E)-2,6,11,15-Tetramethyl-2,6,8,10,14-hexadecapentaene # $$

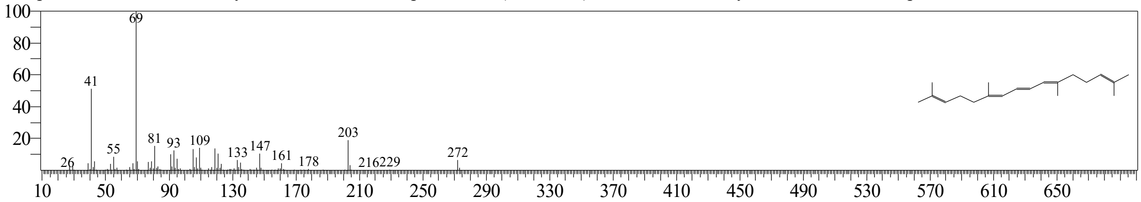

Hit#:5 Entry:96990 Library:NIST11.lib
SI:80 Formula:C20H32 CAS:70901-63-2 MolWeight:272 RetIndex:1922
CompName:(E,E)-7,11,15-Trimethyl-3-methylene-hexadeca-1,6,10,14-tetraene $$ (6E,10E)-7,11,15-Trimethyl-3-methylene-1,6,10,14-hexadecatetraene # $$

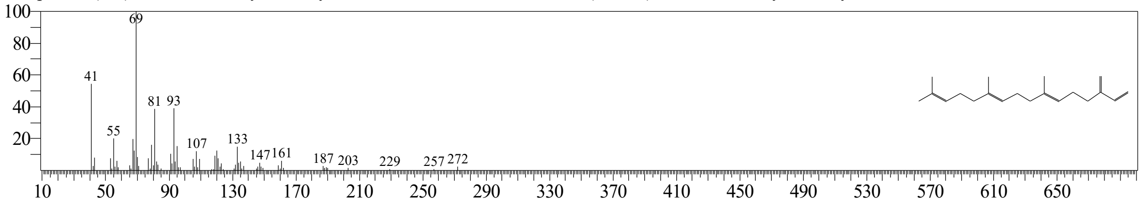

<< Target >>
Line#:66 R.Time:14.090(Scan#:2019) MassPeaks:491
RawMode:Averaged 14.085-14.095(2018-2020) BasePeak:43.10(537046)
BG Mode:Calc. from Peak Group 1 - Event 1 Scan

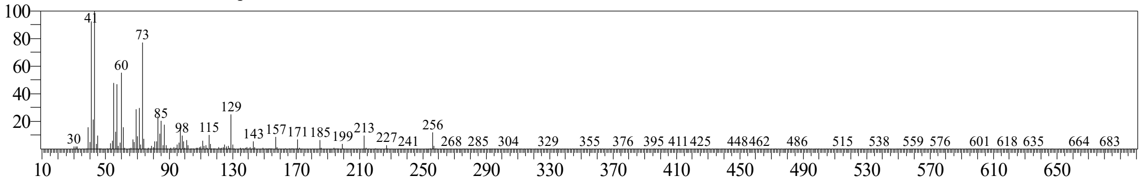

Hit#:1 Entry:84362 Library:NIST11.lib
SI:93 Formula:C16H32O2 CAS:57-10-3 MolWeight:256 RetIndex:1968
CompName:n-Hexadecanoic acid $$ Hexadecanoic acid $$ n-Hexadecoic acid $$ Palmitic acid $$ Pentadecanecarboxylic acid $$ 1-Pentadecanecarboxylic acid

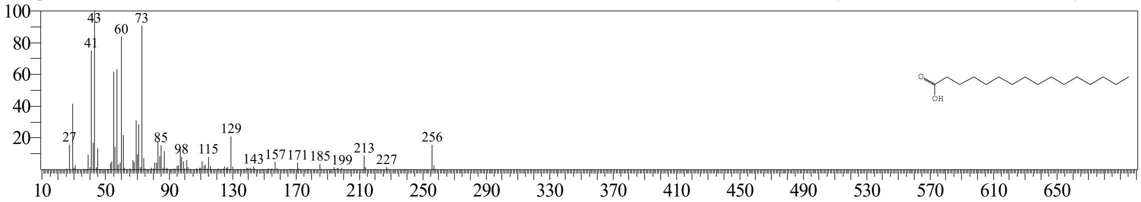

Hit#:2 Entry:128151 Library:NIST11.lib
SI:91 Formula:C20H40O2 CAS:506-30-9 MolWeight:312 RetIndex:2366
CompName:Eicosanoic acid $$ Arachic acid $$ Arachidic acid $$ Icosanoic acid $$ n-Eicosanoic acid $$ Arachidic acid (synthetic) $$

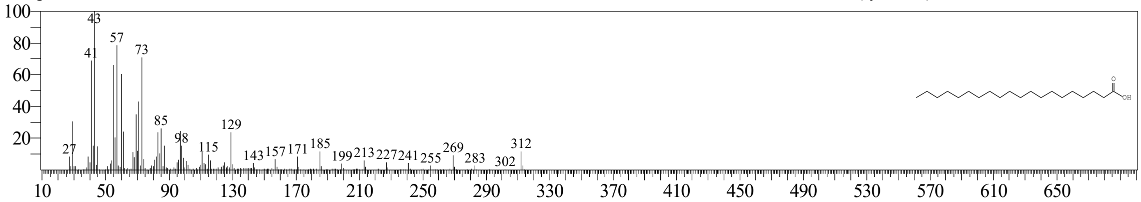

Hit#:3 Entry:73851 Library:NIST11.lib
SI:90 Formula:C15H30O2 CAS:1002-84-2 MolWeight:242 RetIndex:1869
CompName:Pentadecanoic acid $$ Pentadecylic acid $$ n-Pentadecanoic acid $$ n-Pentadecylic acid $$

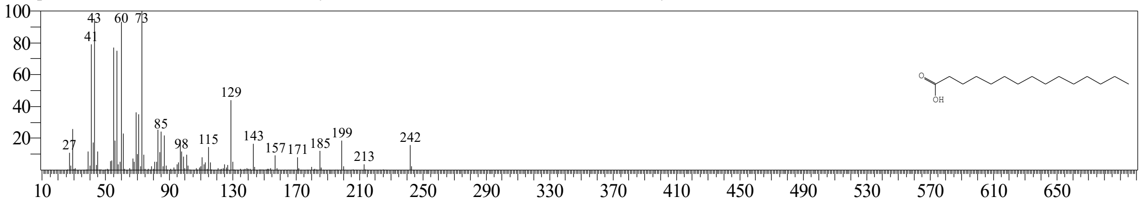

Hit#:4 Entry:53599 Library:NIST11.lib
SI:90 Formula:C13H26O2 CAS:638-53-9 MolWeight:214 RetIndex:1670
CompName:Tridecanoic acid $$ n-Tridecanoic acid $$ n-Tridecoic acid $$ Tridecylic acid $$

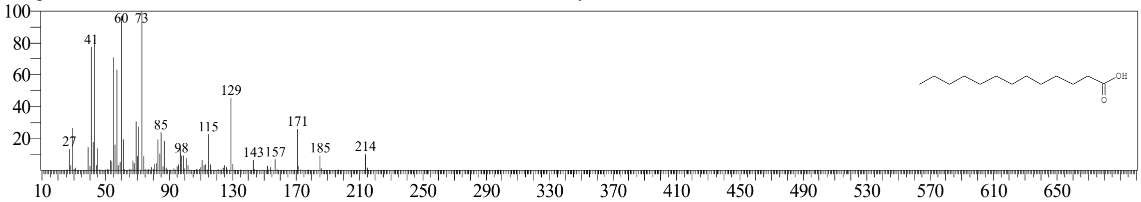

Hit#:5 Entry:169171 Library:NIST11.lib
SI:90 Formula:C22H44O4 CAS:106-11-6 MolWeight:372 RetIndex:2694
CompName:Octadecanoic acid, 2-(2-hydroxyethoxy)ethyl ester $$ Aqua Cera $$ Atlas G 2146 $$ Cerasynt $$ Cerasynt Special $$ Clindrol SDG $$ Diethylene

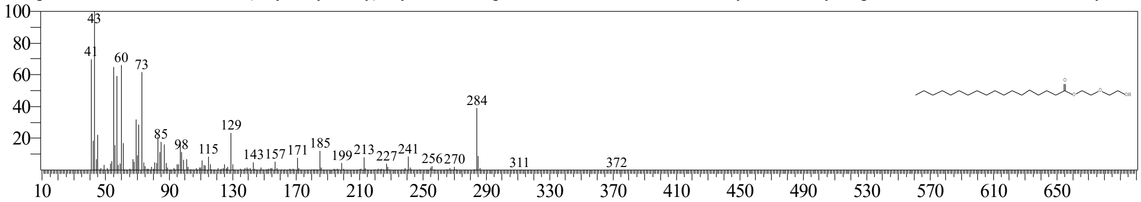

<< Target >>
Line#:67 R.Time:14.165(Scan#:2034) MassPeaks:386
RawMode:Averaged 14.160-14.170(2033-2035) BasePeak:41.05(51963)
BG Mode:Calc. from Peak Group 1 - Event 1 Scan

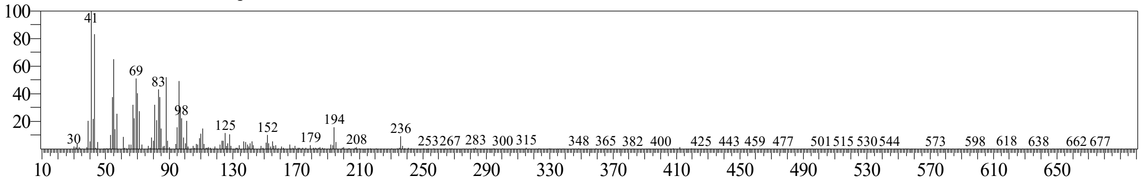

Hit#:1 Entry:126491 Library:NIST11.lib
SI:86 Formula:C20H38O2 CAS:6114-18-7 MolWeight:310 RetIndex:2185
CompName:(E)-9-Octadecenoic acid ethyl ester $$ 9-Octadecenoic acid, ethyl ester, (E)- $$ Ethyl 9-octadecenoate, (E)- $$ Ethyl (9E)-9-octadecenoate $$ Elaidi

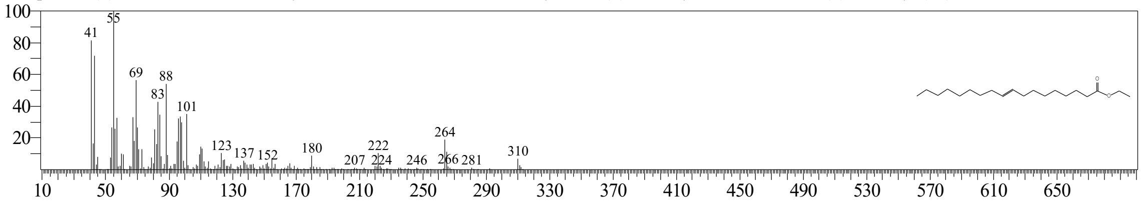

Hit#:2 Entry:104404 Library:NIST11.lib
SI:85 Formula:C18H34O2 CAS:0-00-0 MolWeight:282 RetIndex:1986
CompName:E-11-Hexadecenoic acid, ethyl ester $$ Ethyl (11E)-11-hexadecenoate # $$

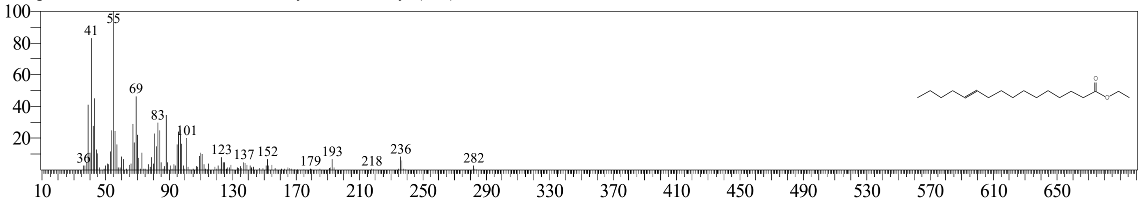

Hit#:3 Entry:126497 Library:NIST11.lib
SI:84 Formula:C20H38O2 CAS:6512-99-8 MolWeight:310 RetIndex:2185
CompName:9-Octadecenoic acid, ethyl ester $$ Ethyl 9-octadecenoate $$ Ethyl octadec-9-enoate $$

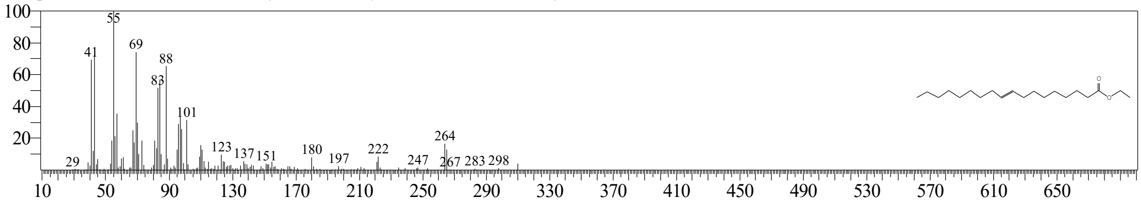

Hit#:4 Entry:165539 Library:NIST11.lib
SI:83 Formula:C24H46O2 CAS:37910-77-3 MolWeight:366 RetIndex:2582
CompName:Ethyl 13-docosenoate(ethyl erucate) $$ Erucic acid ethyl ester $$ 13-Docosenoic acid, ethyl ester, (Z)- $$ Ethyl (13Z)-13-docosenoate # $$

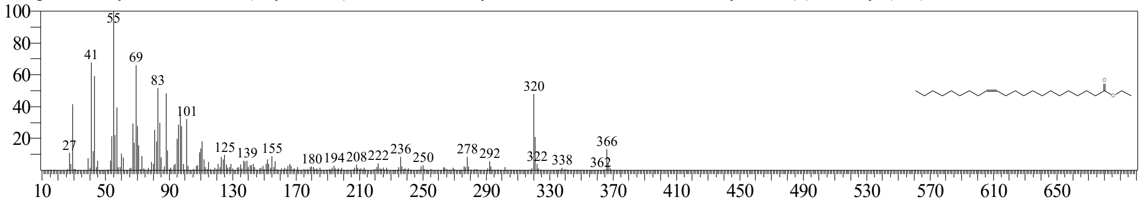

Hit#:5 Entry:82693 Library:NIST11.lib
SI:83 Formula:C16H30O2 CAS:2091-29-4 MolWeight:254 RetIndex:1976
CompName:9-Hexadecenoic acid $$ (9E)-9-Hexadecenoic acid # $$

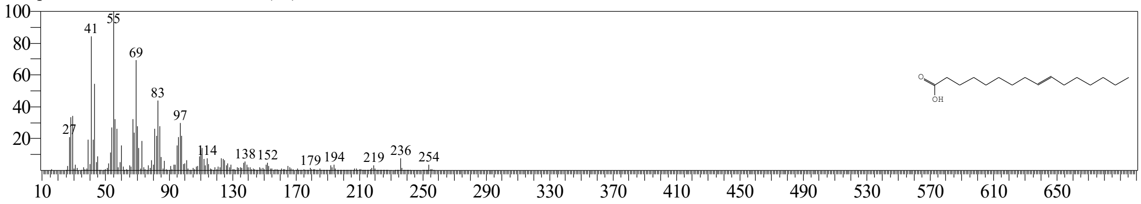

<< Target >>
Line#:68 R.Time:14.215(Scan#:2044) MassPeaks:450
RawMode:Averaged 14.210-14.220(2043-2045) BasePeak:88.05(5407039)
BG Mode:Calc. from Peak Group 1 - Event 1 Scan

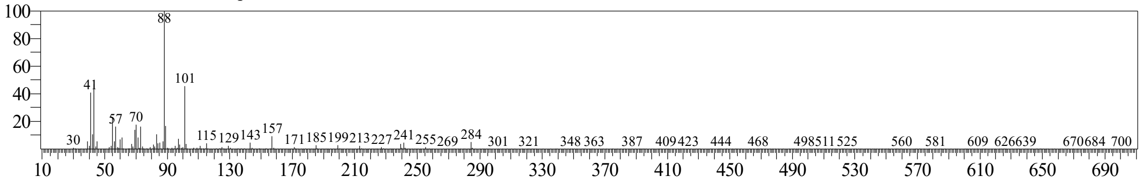

Hit#:1 Entry:106188 Library:NIST11.lib
SI:94 Formula:C18H36O2 CAS:628-97-7 MolWeight:284 RetIndex:1978
CompName:Hexadecanoic acid, ethyl ester $$ Palmitic acid, ethyl ester $$ Ethyl hexadecanoate $$ Ethyl palmitate $$ Ethyl n-hexadecanoate $$

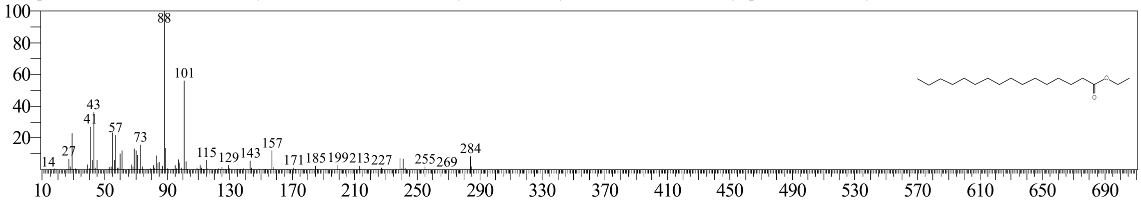

Hit#:2 Entry:95197 Library:NIST11.lib
SI:91 Formula:C17H34O2 CAS:41114-00-5 MolWeight:270 RetIndex:1878
CompName:Pentadecanoic acid, ethyl ester $$ Ethyl pentadecanoate $$ n-Pentadecanoic acid ethyl ester $$

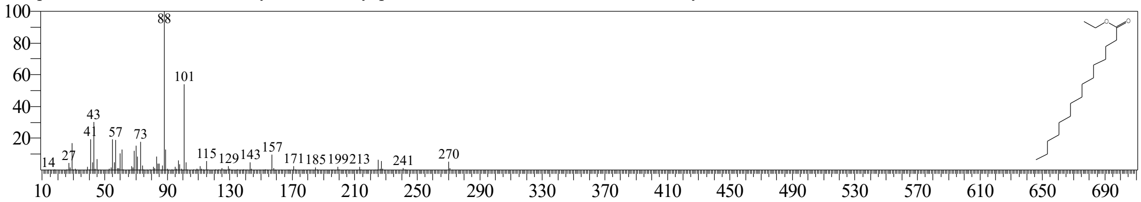

Hit#:3 Entry:117163 Library:NIST11.lib
SI:91 Formula:C19H38O2 CAS:14010-23-2 MolWeight:298 RetIndex:2077
CompName:Heptadecanoic acid, ethyl ester $$ Ethyl heptadecanoate $$ Ethyl n-heptadecanoate $$

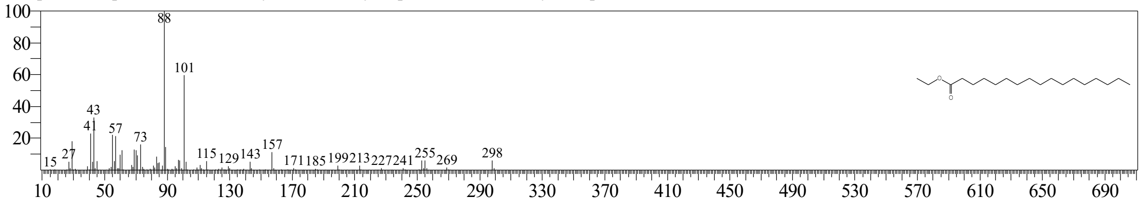

Hit#:4 Entry:149096 Library:NIST11.lib
SI:90 Formula:C22H44O2 CAS:18281-05-5 MolWeight:340 RetIndex:2375
CompName:Eicosanoic acid, ethyl ester $$ Ethyl icosanoate $$ Ethyl arachidate $$ Ethyl eicosanoate $$ Arachidic acid, ethyl ester $$

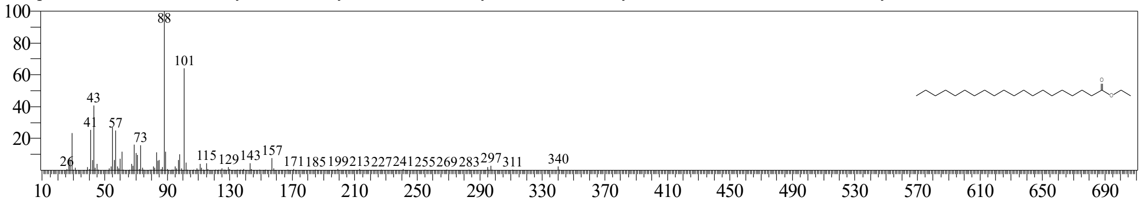

Hit#:5 Entry:138751 Library:NIST11.lib
SI:89 Formula:C21H42O2 CAS:18281-04-4 MolWeight:326 RetIndex:2276
CompName:Nonadecanoic acid, ethyl ester $$ Ethyl nonadecanoate # $$

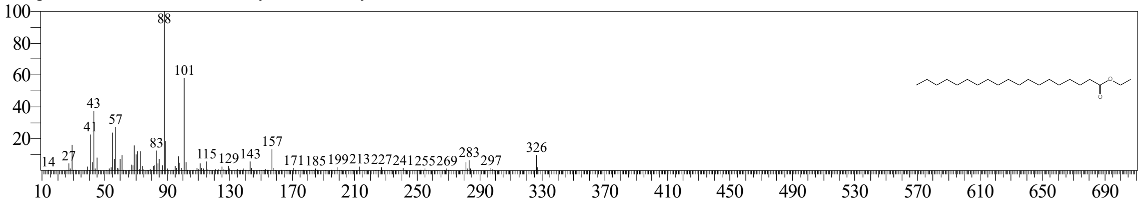

<< Target >>
Line#:69 R.Time:14.375(Scan#:2076) MassPeaks:377
RawMode:Averaged 14.370-14.380(2075-2077) BasePeak:32.10(3977)
BG Mode:Calc. from Peak Group 1 - Event 1 Scan

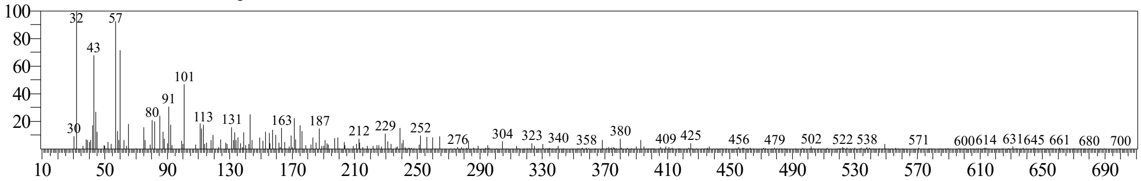

Hit#:1 Entry:12439 Library:NIST11.lib
SI:49 Formula:C6H13N3O CAS:0-00-0 MolWeight:143 RetIndex:1377
CompName:Isovaleraldehyde semicarbazone

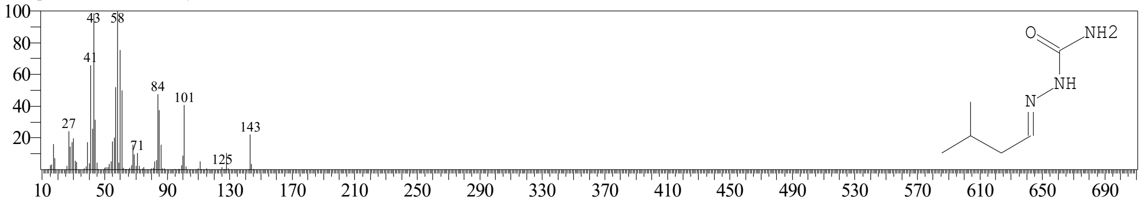

Hit#:2 Entry:20185 Library:NIST11.lib
SI:48 Formula:C8H16O3 CAS:0-00-0 MolWeight:160 RetIndex:996
CompName:2-Isopropoxyethyl propionate

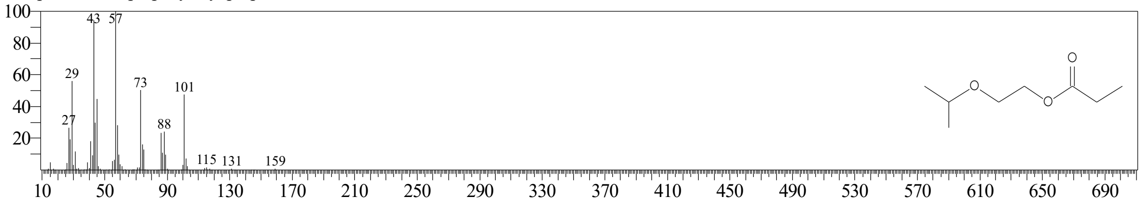

Hit#:3 Entry:5068 Library:NIST11.lib
SI:48 Formula:C3H9N3S CAS:31424-47-2 MolWeight:119 RetIndex:1249
CompName:Hydrazinecarboximidothioic acid, ethyl ester $$ Ethyl hydrazonothiocarbamate # $$

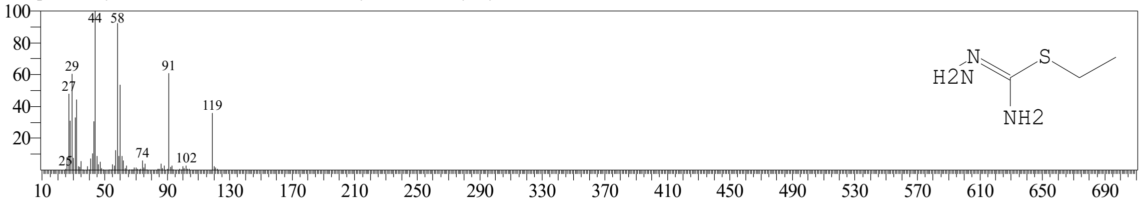

Hit#:4 Entry:28820 Library:NIST11.lib
SI:47 Formula:C7H16N2OS CAS:29146-57-4 MolWeight:176 RetIndex:1658
CompName:1-[t-Butyl]-3-[2-hydroxyethyl]-2-thiourea $$ N-(tert-Butyl)-N'-(2-hydroxyethyl)thiourea # $$

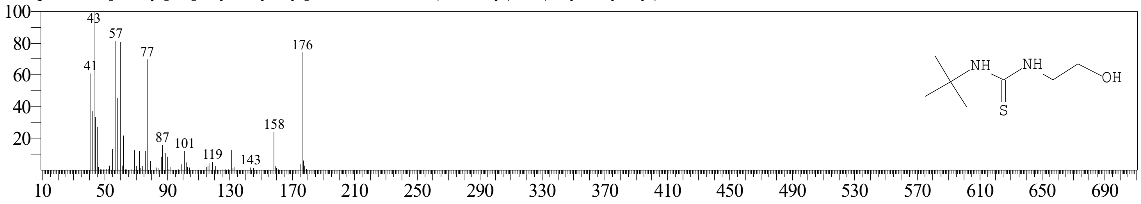

Hit#:5 Entry:14206 Library:NIST11.lib
SI:46 Formula:C3H8N4OS CAS:56742-24-6 MolWeight:148 RetIndex:1746
CompName:N-(N-Methylformamidyl)-semithiocarbazide $$ 2-(Aminocarbothioyl)-N-methylhydrazinecarboxamide # $$

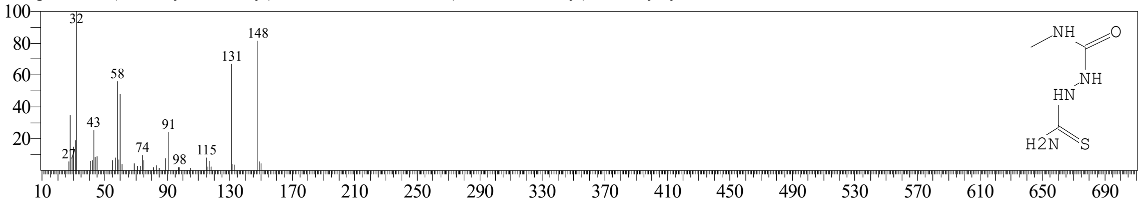

Qualitative Analysis Report

<< Target >>
Line#:70 R.Time:14.385(Scan#:2078) MassPeaks:345
RawMode:Averaged 14.380-14.390(2077-2079) BasePeak:32.10(4964)
BG Mode:Calc. from Peak Group 1 - Event 1 Scan

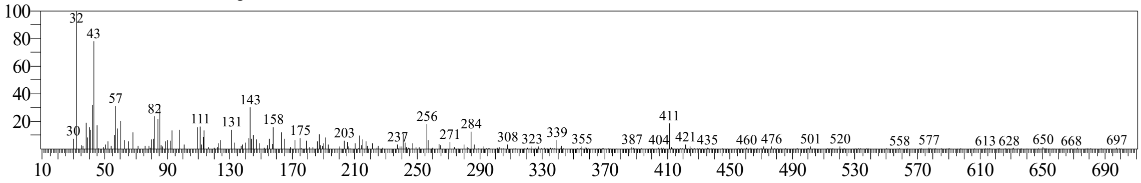

Hit#:1 Entry:12439 Library:NIST11.lib
SI:53 Formula:C6H13N3O CAS:0-00-0 MolWeight:143 RetIndex:1377
CompName:Isovaleraldehyde semicarbazone

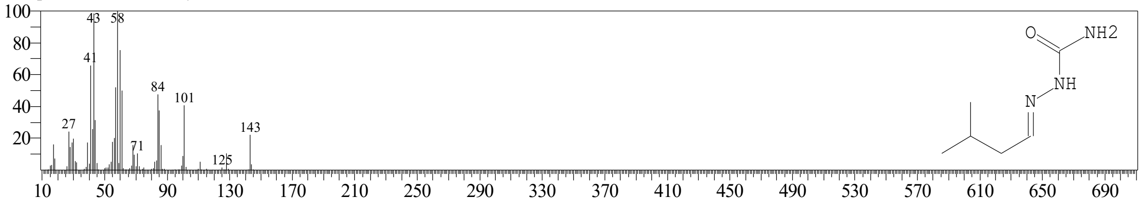

Hit#:2 Entry:19326 Library:NIST11.lib
SI:52 Formula:C8H18N2O CAS:997-95-5 MolWeight:158 RetIndex:1147
CompName:1-Propanamine, 2-methyl-N-(2-methylpropyl)-N-nitroso- $$ Dipropylamine, 2,2'-dimethyl-N-nitroso- $$ Diisobutylnitrosamine $$ N-Nitrosodiisob

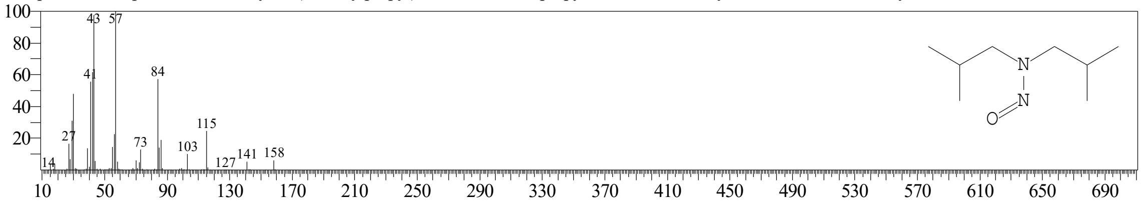

Hit#:3 Entry:74167 Library:NIST11.lib
SI:51 Formula:C11H21N3O3 CAS:57006-62-9 MolWeight:243 RetIndex:0
CompName:4-i-Propylcarbamoyl-1-hydroxy-2,2,5,5-tetramethyl-3-imidazoline-3-oxide $$ 4-Isopropylcarbamoyl-1-hydroxy-2,2,5,5-tetramethyl-3-imidazoline-3

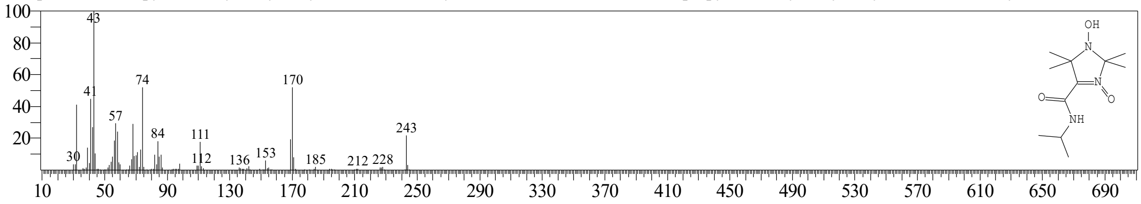

Hit#:4 Entry:188399 Library:NIST11.lib
SI:51 Formula:C18H24N4NiO4 CAS:0-00-0 MolWeight:418 RetIndex:0
CompName:Ni(ii)-2,7-bis[2-hydroxy-5,5-dimethyl-4,5-dihydro-1H-pyrrol-4-on-3-yl]-3,6-diazaocta-2,6-dien

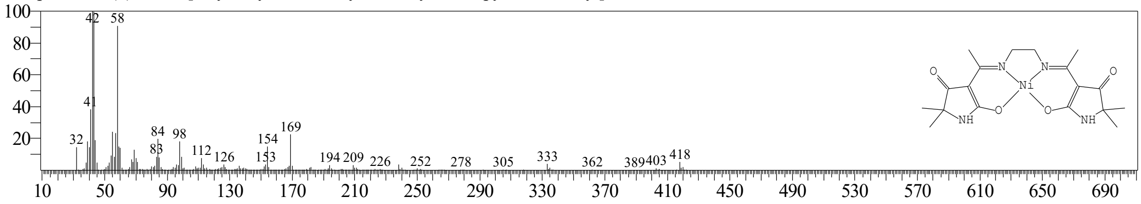

Hit#:5 Entry:35053 Library:NIST11.lib
SI:51 Formula:C10H22N2O CAS:20478-70-0 MolWeight:186 RetIndex:1658
CompName:Decanohydrazide

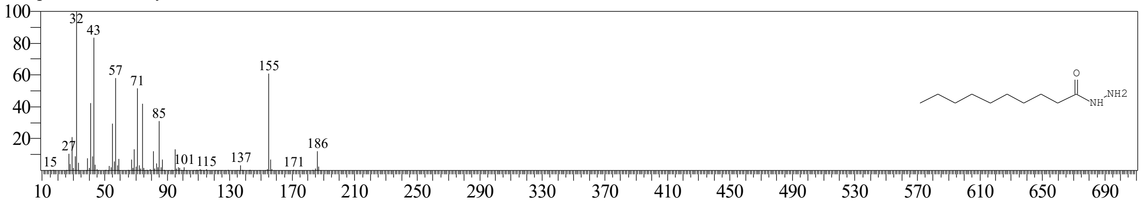

<< Target >>
Line#:71 R.Time:14.425(Scan#:2086) MassPeaks:333
RawMode:Averaged 14.420-14.430(2085-2087) BasePeak:43.10(3261)
BG Mode:Calc. from Peak Group 1 - Event 1 Scan

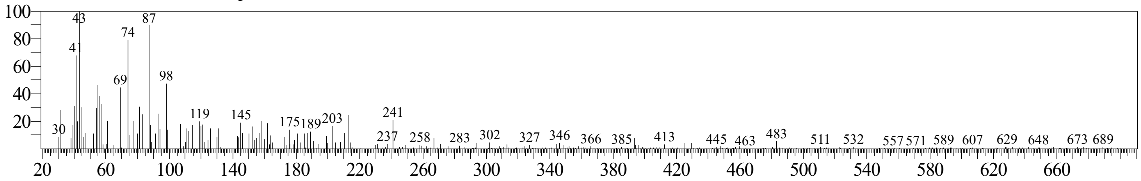

Hit#:1 Entry:27122 Library:NIST11.lib
SI:61 Formula:C10H20O2 CAS:15870-07-2 MolWeight:172 RetIndex:1118
CompName:Octanoic acid, 4-methyl-, methyl ester $$ Methyl 4-methyloctanoate $$ 4-Methyloctanoic acid methyl ester $$ 3-Methyl-heptanecarboxylic acid me

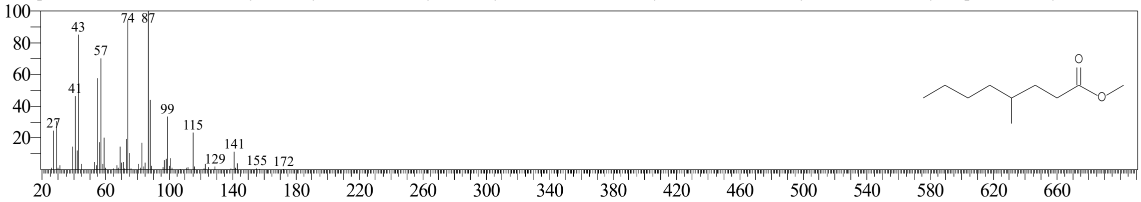

Hit#:2 Entry:159439 Library:NIST11.lib
SI:60 Formula:C21H40O4 CAS:25751-93-3 MolWeight:356 RetIndex:2379
CompName:Tridecanedioic acid, 2-hexyl-, dimethyl ester $$ Dimethyl 2-hexyltridecanedioate # $$

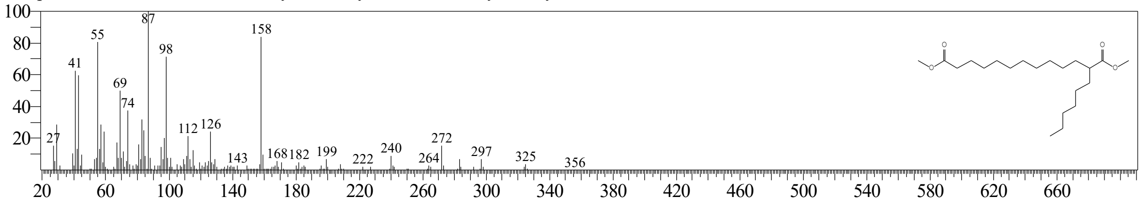

Hit#:3 Entry:138750 Library:NIST11.lib
SI:60 Formula:C21H42O2 CAS:124324-23-8 MolWeight:326 RetIndex:2212
CompName:Undecanoic acid, 2-nonyl-, methyl ester $$ Methyl 2-nonylundecanoate # $$

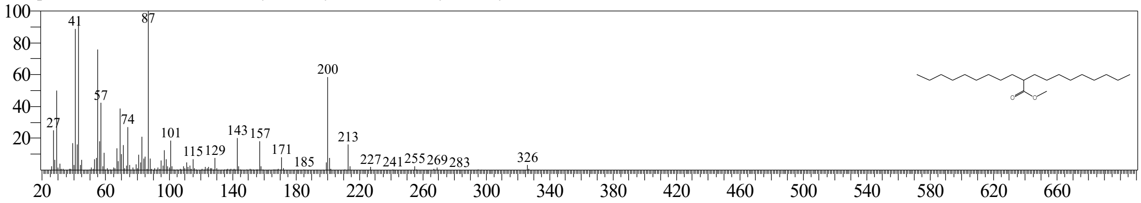

Hit#:4 Entry:84386 Library:NIST11.lib
SI:60 Formula:C16H32O2 CAS:5129-66-8 MolWeight:256 RetIndex:1715
CompName:Tetradecanoic acid, 12-methyl-, methyl ester $$ Methyl 12-methyltetradecanoate $$ Methyl tetradecanoate, 12-methyl $$

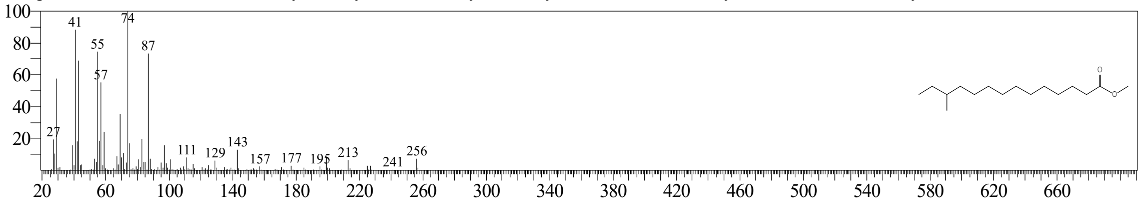

Hit#:5 Entry:64813 Library:NIST11.lib
SI:59 Formula:C12H22O4 CAS:3927-60-4 MolWeight:230 RetIndex:1738
CompName:Undecanedioic acid, monomethyl ester $$ 11-Methoxy-11-oxoundecanoic acid # $$

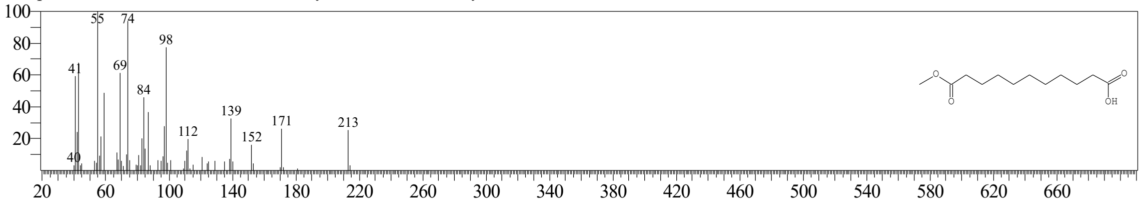

<< Target >>
Line#:72 R.Time:14.615(Scan#:2124) MassPeaks:378
RawMode:Averaged 14.610-14.620(2123-2125) BasePeak:71.15(10950)
BG Mode:Calc. from Peak Group 1 - Event 1 Scan

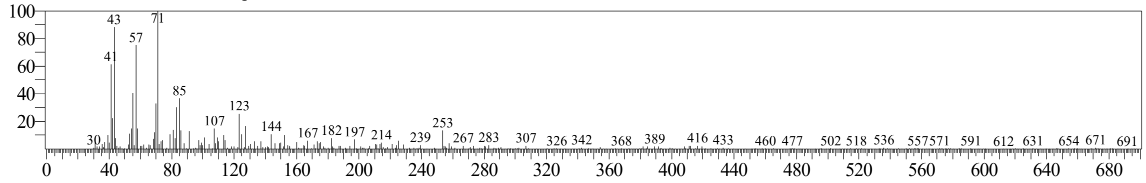

Hit#:1 Entry:47433 Library:NIST11.lib
SI:75 Formula:C9H19Br CAS:2216-35-5 MolWeight:206 RetIndex:1148
CompName:2-Bromononane $$ sec-Nonyl bromide $$ Nonane, 2-bromo- $$

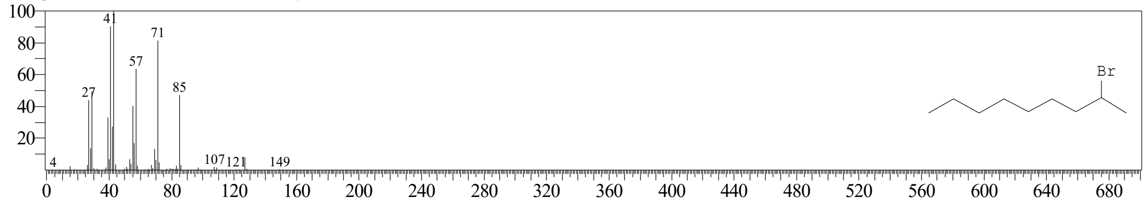

Hit#:2 Entry:137183 Library:NIST11.lib
SI:74 Formula:C23H48 CAS:25117-30-0 MolWeight:324 RetIndex:2243
CompName:4-Methyldocosane $$ Docosane, 4-methyl $$

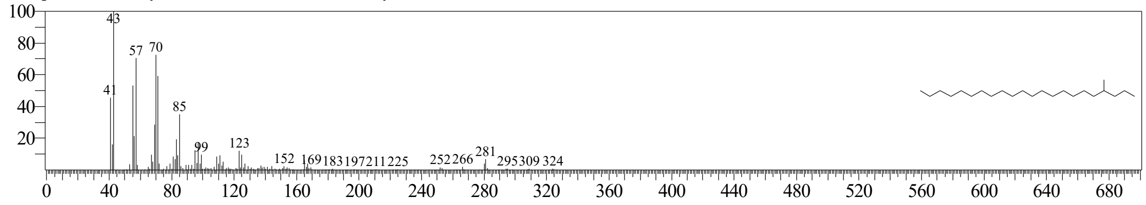

Hit#:3 Entry:115534 Library:NIST11.lib
SI:74 Formula:C21H44 CAS:75163-99-4 MolWeight:296 RetIndex:1980
CompName:Nonadecane, 2,3-dimethyl- $$ 2,3-Dimethylnonadecane # $$

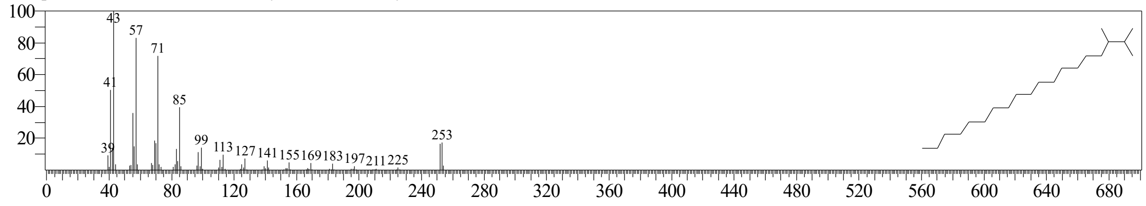

Hit#:4 Entry:72482 Library:NIST11.lib
SI:74 Formula:C17H36 CAS:25117-26-4 MolWeight:240 RetIndex:1647
CompName:Hexadecane, 4-methyl- $$ 4-Methylhexadecane # $$

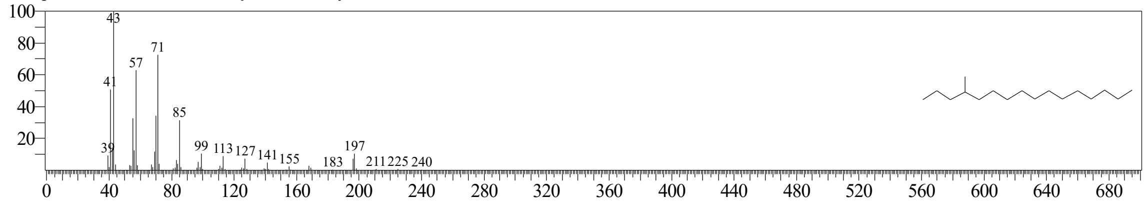

Hit#:5 Entry:42959 Library:NIST11.lib
SI:73 Formula:C14H30 CAS:192823-15-7 MolWeight:198 RetIndex:1156
CompName:Decane, 2,3,5,8-tetramethyl- $$ 2,3,5,8-Tetramethyldecane # $$

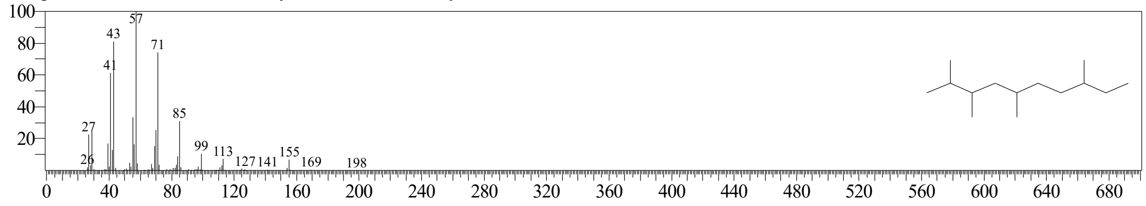

<< Target >>
Line#:73 R.Time:14.715(Scan#:2144) MassPeaks:365
RawMode:Averaged 14.710-14.720(2143-2145) BasePeak:41.05(12004)
BG Mode:Calc. from Peak Group 1 - Event 1 Scan

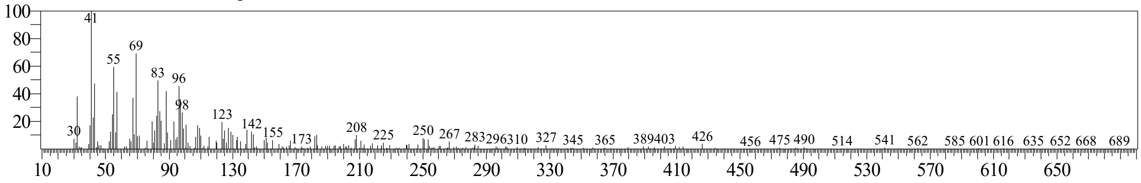

Hit#:1 Entry:126491 Library:NIST11.lib
SI:75 Formula:C20H38O2 CAS:6114-18-7 MolWeight:310 RetIndex:2185
CompName:(E)-9-Octadecenoic acid ethyl ester $$ 9-Octadecenoic acid, ethyl ester, (E)- $$ Ethyl 9-octadecenoate, (E)- $$ Ethyl (9E)-9-octadecenoate $$ Elaidi

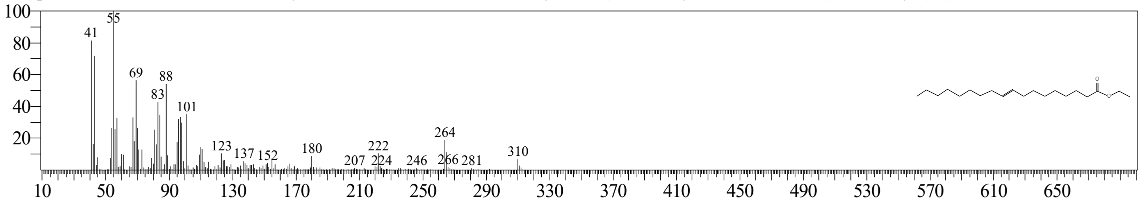

Hit#:2 Entry:126497 Library:NIST11.lib
SI:74 Formula:C20H38O2 CAS:6512-99-8 MolWeight:310 RetIndex:2185
CompName:9-Octadecenoic acid, ethyl ester $$ Ethyl 9-octadecenoate $$ Ethyl octadec-9-enoate $$

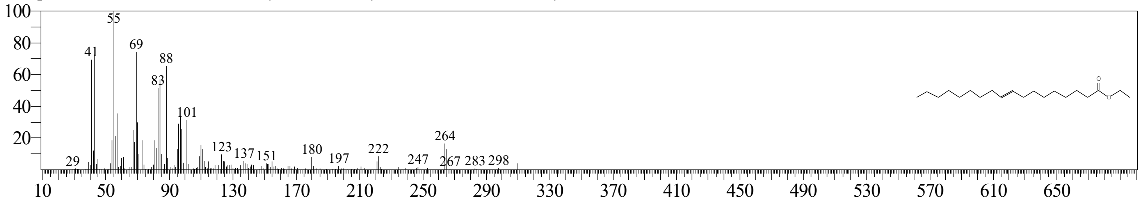

Hit#:3 Entry:72302 Library:NIST11.lib
SI:74 Formula:C15H28O2 CAS:4727-18-8 MolWeight:240 RetIndex:2158
CompName:Cyclopentadecanone, 2-hydroxy- $$ 2-Hydroxycyclopentadecanone # $$

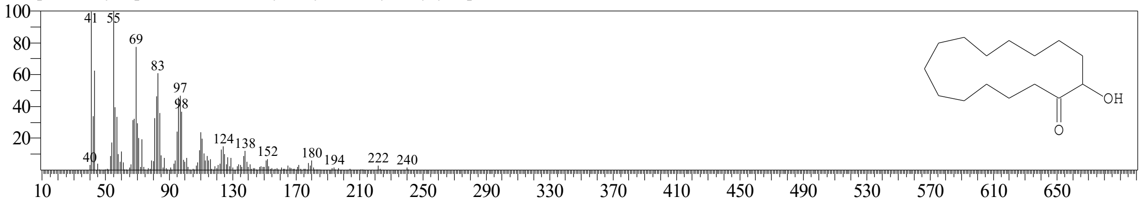

Hit#:4 Entry:164236 Library:NIST11.lib
SI:74 Formula:C20H35F3O2 CAS:0-00-0 MolWeight:364 RetIndex:2019
CompName:9-octadecenoic acid, 2,2,2-trifluoroethyl ester

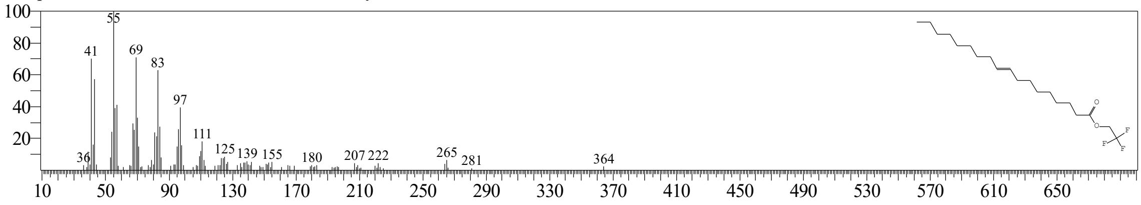

Hit#:5 Entry:115406 Library:NIST11.lib
SI:73 Formula:C19H36O2 CAS:73033-09-7 MolWeight:296 RetIndex:2274
CompName:cis-10-Nonadecenoic acid

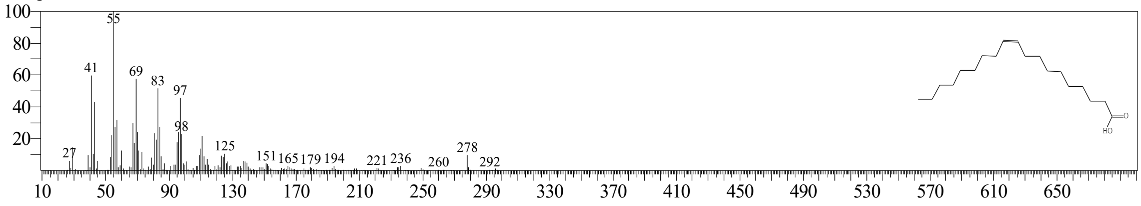

<< Target >>
Line#:74 R.Time:14.780(Scan#:2157) MassPeaks:346
RawMode:Averaged 14.775-14.785(2156-2158) BasePeak:57.05(14539)
BG Mode:Calc. from Peak Group 1 - Event 1 Scan

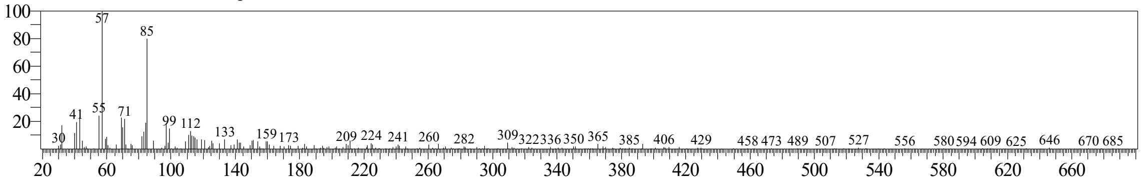

Hit#:1 Entry:107453 Library:NIST11.lib
SI:71 Formula:C16H30O4 CAS:0-00-0 MolWeight:286 RetIndex:1754
CompName:Oxalic acid, 6-ethyloct-3-yl isobutyl ester

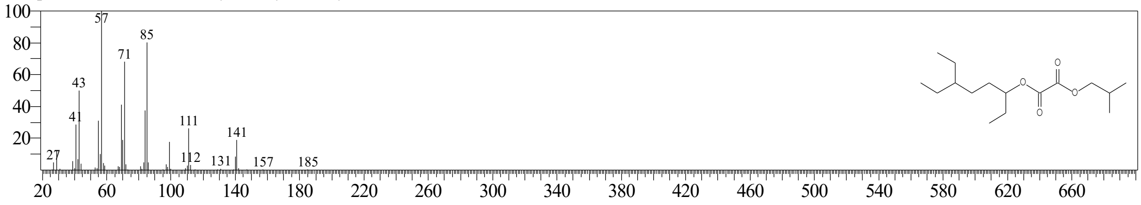

Hit#:2 Entry:185354 Library:NIST11.lib
SI:71 Formula:C29H60 CAS:0-00-0 MolWeight:408 RetIndex:2840
CompName:2-methyloctacosane

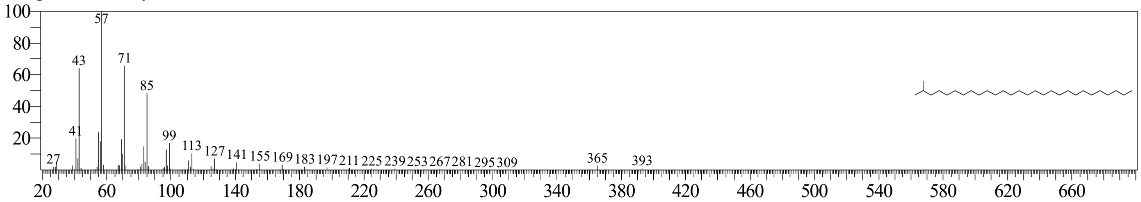

Hit#:3 Entry:126569 Library:NIST11.lib
SI:71 Formula:C22H46 CAS:0-00-0 MolWeight:310 RetIndex:2124
CompName:5-Ethyl-5-methylnonadecane

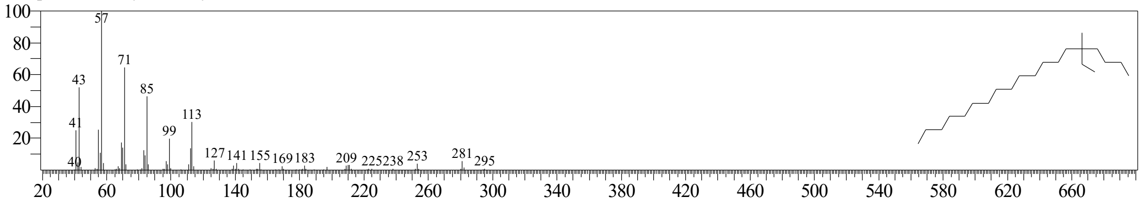

Hit#:4 Entry:104539 Library:NIST11.lib
SI:71 Formula:C20H42 CAS:56862-62-5 MolWeight:282 RetIndex:1945
CompName:10-Methylnonadecane $$ Nonadecane, 10-methyl $$

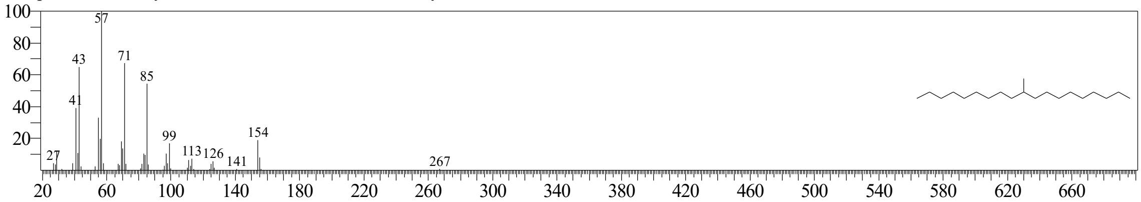

Hit#:5 Entry:104535 Library:NIST11.lib
SI:70 Formula:C20H42 CAS:112-95-8 MolWeight:282 RetIndex:2009
CompName:Eicosane $$ n-Eicosane $$ Icosane # $$ n-Icosane $$

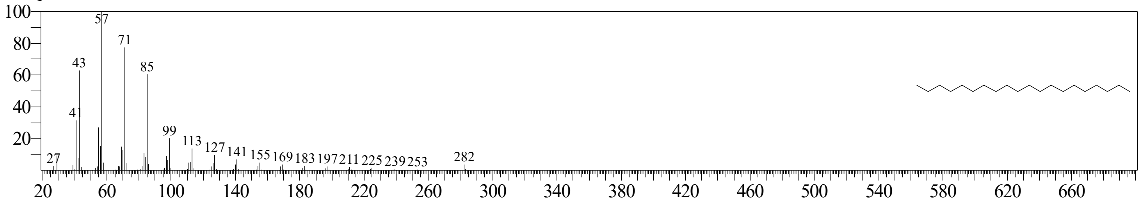

<< Target >>
Line#:75 R.Time:14.855(Scan#:2172) MassPeaks:383
RawMode:Averaged 14.850-14.860(2171-2173) BasePeak:88.05(229411)
BG Mode:Calc. from Peak Group 1 - Event 1 Scan

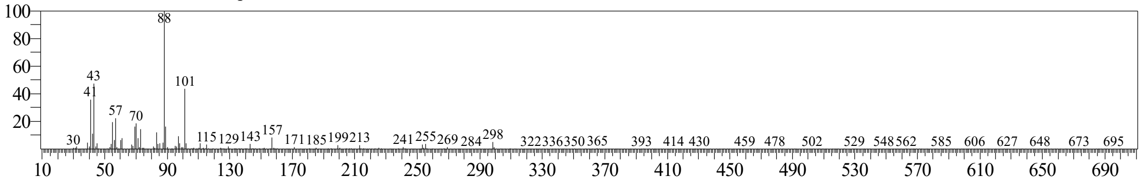

Hit#:1 Entry:117163 Library:NIST11.lib
SI:92 Formula:C19H38O2 CAS:14010-23-2 MolWeight:298 RetIndex:2077
CompName:Heptadecanoic acid, ethyl ester $$ Ethyl heptadecanoate $$ Ethyl n-heptadecanoate $$

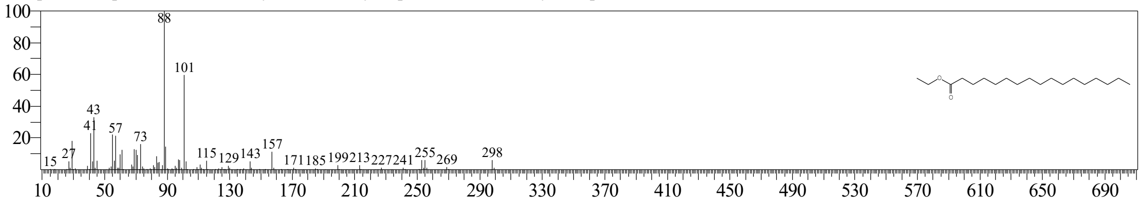

Hit#:2 Entry:149096 Library:NIST11.lib
SI:90 Formula:C22H44O2 CAS:18281-05-5 MolWeight:340 RetIndex:2375
CompName:Eicosanoic acid, ethyl ester $$ Ethyl icosanoate $$ Ethyl arachidate $$ Ethyl eicosanoate $$ Arachidic acid, ethyl ester $$

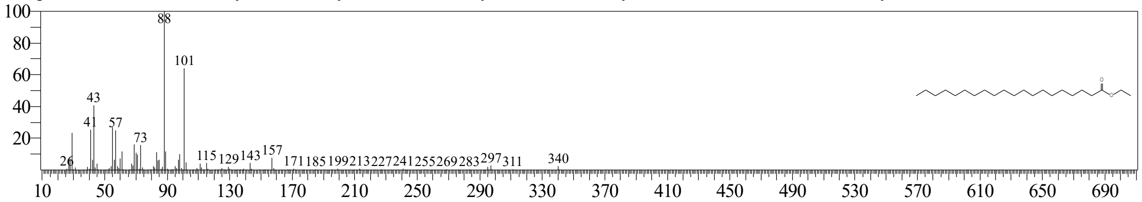

Hit#:3 Entry:106188 Library:NIST11.lib
SI:90 Formula:C18H36O2 CAS:628-97-7 MolWeight:284 RetIndex:1978
CompName:Hexadecanoic acid, ethyl ester $$ Palmitic acid, ethyl ester $$ Ethyl hexadecanoate $$ Ethyl palmitate $$ Ethyl n-hexadecanoate $$

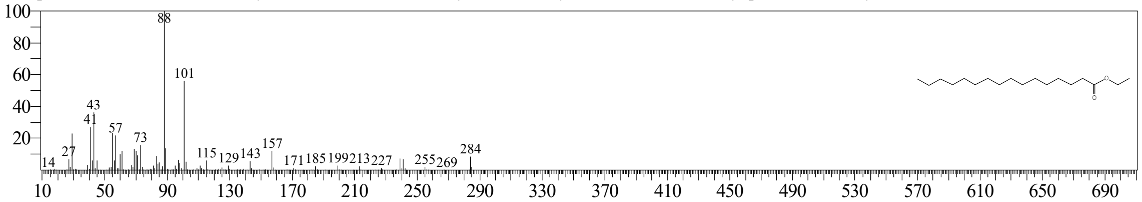

Hit#:4 Entry:95197 Library:NIST11.lib
SI:89 Formula:C17H34O2 CAS:41114-00-5 MolWeight:270 RetIndex:1878
CompName:Pentadecanoic acid, ethyl ester $$ Ethyl pentadecanoate $$ n-Pentadecanoic acid ethyl ester $$

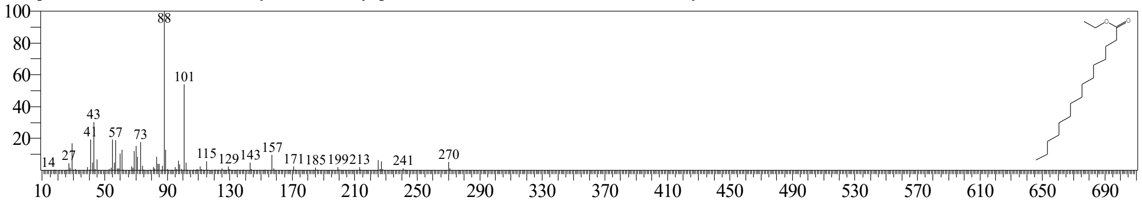

Hit#:5 Entry:138751 Library:NIST11.lib
SI:89 Formula:C21H42O2 CAS:18281-04-4 MolWeight:326 RetIndex:2276
CompName:Nonadecanoic acid, ethyl ester $$ Ethyl nonadecanoate # $$

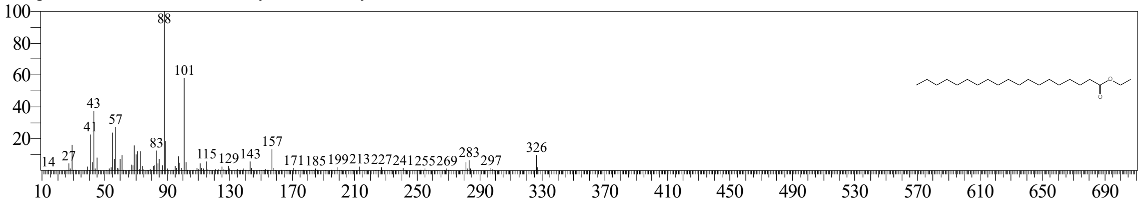

<< Target >>
Line#:76 R.Time:14.920(Scan#:2185) MassPeaks:416
RawMode:Averaged 14.915-14.925(2184-2186) BasePeak:79.10(45577)
BG Mode:Calc. from Peak Group 1 - Event 1 Scan

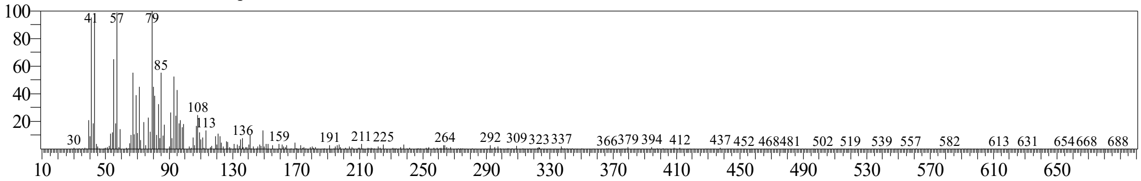

Hit#:1 Entry:134374 Library:NIST11.lib
SI:83 Formula:C21H36O2 CAS:17364-32-8 MolWeight:320 RetIndex:2300
CompName:8,11,14-Eicosatrienoic acid, methyl ester $$ Methyl (8E,11E,14E)-8,11,14-icosatrienoate # $$

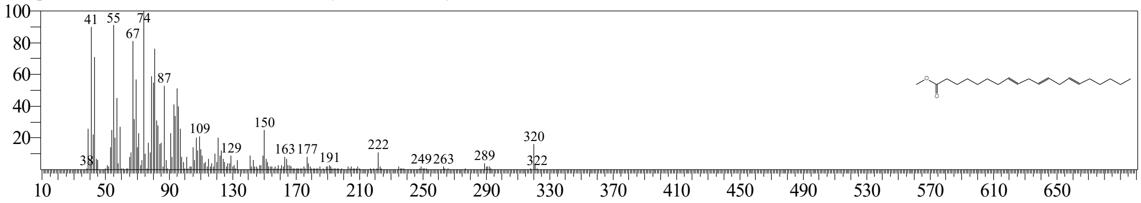

Hit#:2 Entry:156918 Library:NIST11.lib
SI:83 Formula:C21H36O4 CAS:18465-99-1 MolWeight:352 RetIndex:2705
CompName:9,12,15-Octadecatrienoic acid, 2,3-dihydroxypropyl ester, (Z,Z,Z)- $$ Linolenin, 1-mono- $$ .alpha.-Glyceryl linolenate $$ 2,3-Dihydroxypropyl (1

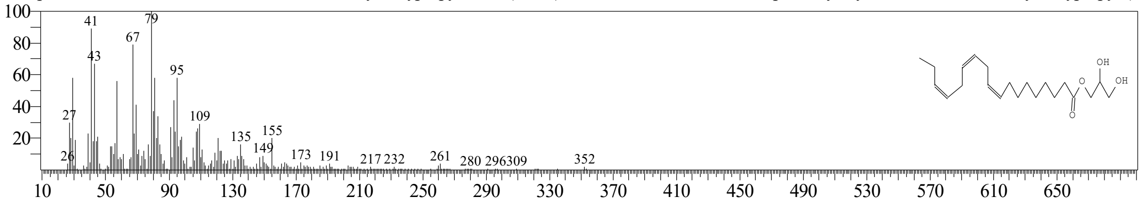

Hit#:3 Entry:113919 Library:NIST11.lib
SI:83 Formula:C19H34O2 CAS:56846-97-0 MolWeight:294 RetIndex:2093
CompName:2,5-Octadecadienoic acid, methyl ester $$ Methyl (2E,5E)-2,5-octadecadienoate # $$

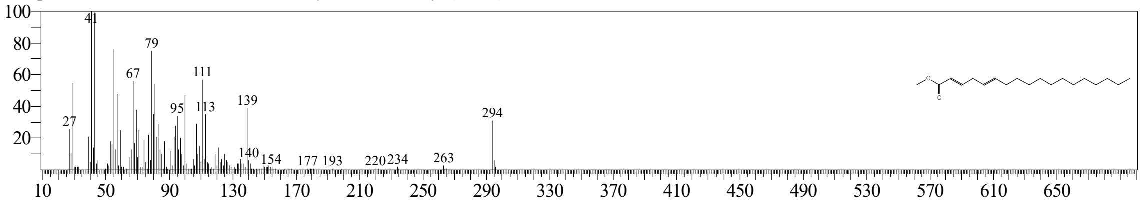

Hit#:4 Entry:101391 Library:NIST11.lib
SI:82 Formula:C18H30O2 CAS:16714-85-5 MolWeight:278 RetIndex:2003
CompName:10-Heptadecen-8-ynoic acid, methyl ester, (E)- $$ Methyl (10E)-10-heptadecen-8-ynoate # $$

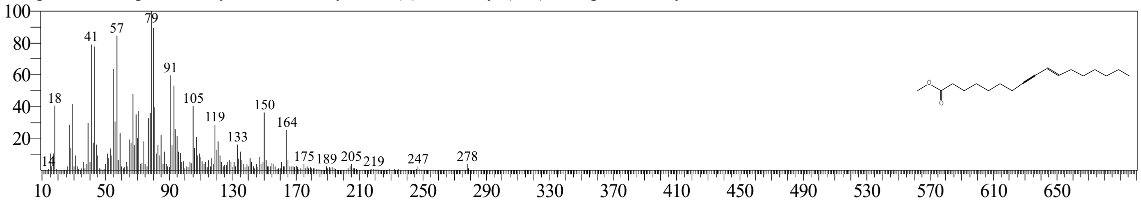

Hit#:5 Entry:154662 Library:NIST11.lib
SI:82 Formula:C23H40O2 CAS:56847-02-0 MolWeight:348 RetIndex:2499
CompName:8,11,14-Docosatrienoic acid, methyl ester $$ Methyl (8E,11E,14E)-8,11,14-docosatrienoate # $$

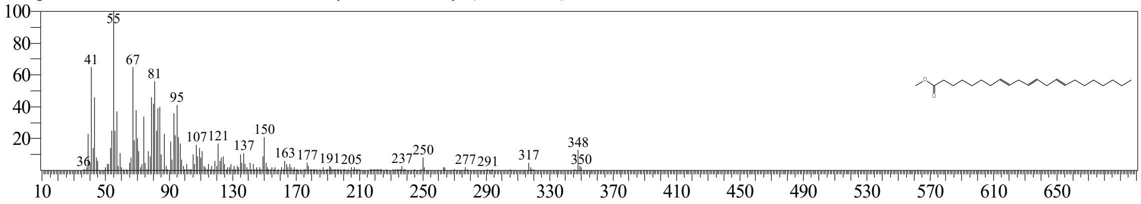

<< Target >>
Line#:77 R.Time:15.000(Scan#:2201) MassPeaks:417
RawMode:Averaged 14.995-15.005(2200-2202) BasePeak:71.05(462137)
BG Mode:Calc. from Peak Group 1 - Event 1 Scan

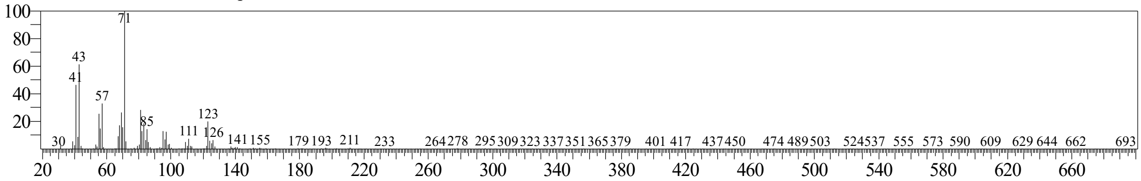

Hit#:1 Entry:115516 Library:NIST11.lib
SI:94 Formula:C20H40O CAS:150-86-7 MolWeight:296 RetIndex:2045
CompName:Phytol $$ 2-Hexadecen-1-ol, 3,7,11,15-tetramethyl-, [R-[R*,R*-(E)]]- $$ trans-Phytol $$ 3,7,11,15-Tetramethyl-2-hexadecen-1-ol-, (2E,7R,11R)- $

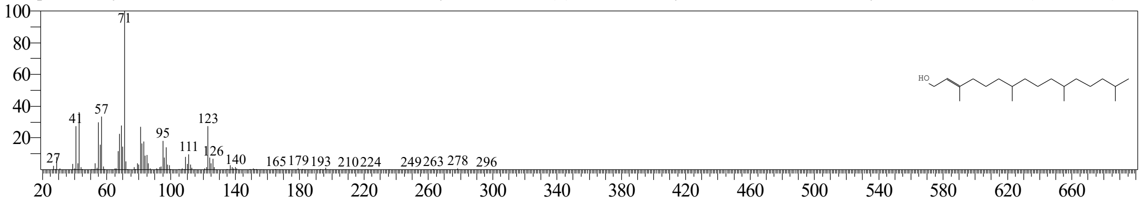

Hit#:2 Entry:183967 Library:NIST11.lib
SI:87 Formula:C23H48O3S CAS:0-00-0 MolWeight:404 RetIndex:2867
CompName:Sulfurous acid, octadecyl 2-pentyl ester

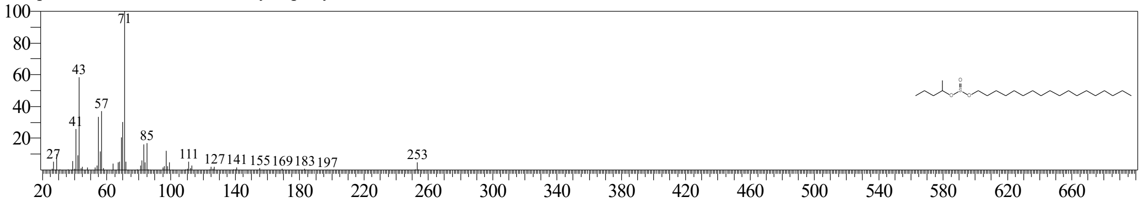

Hit#:3 Entry:84384 Library:NIST11.lib
SI:87 Formula:C16H32O2 CAS:0-00-0 MolWeight:256 RetIndex:1936
CompName:13-Methylpentadec-14-ene-1,13-diol $$ 13-Methyl-14-pentadecene-1,13-diol # $$

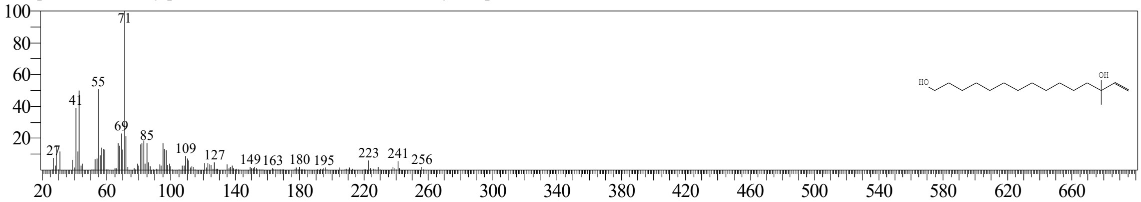

Hit#:4 Entry:183966 Library:NIST11.lib
SI:86 Formula:C23H48O3S CAS:0-00-0 MolWeight:404 RetIndex:2931
CompName:Sulfurous acid, octadecyl pentyl ester

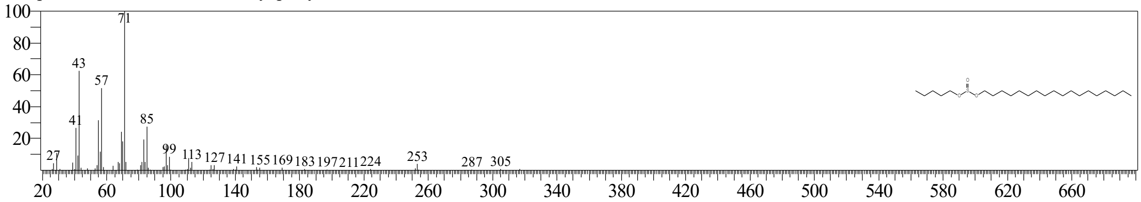

Hit#:5 Entry:171265 Library:NIST11.lib
SI:86 Formula:C21H44O3S CAS:0-00-0 MolWeight:376 RetIndex:2732
CompName:Sulfurous acid, hexadecyl pentyl ester

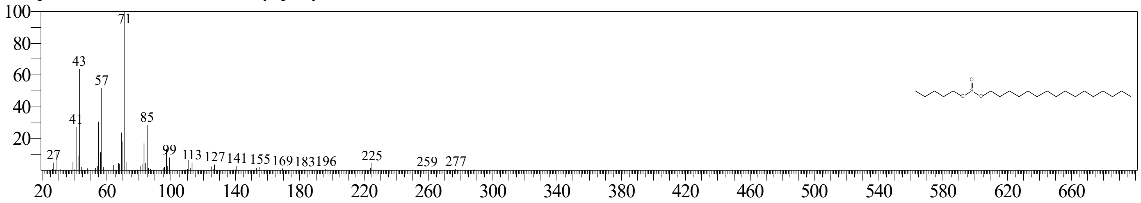

<< Target >>
Line#:78 R.Time:15.250(Scan#:2251) MassPeaks:411
RawMode:Averaged 15.245-15.255(2250-2252) BasePeak:41.05(110093)
BG Mode:Calc. from Peak Group 1 - Event 1 Scan

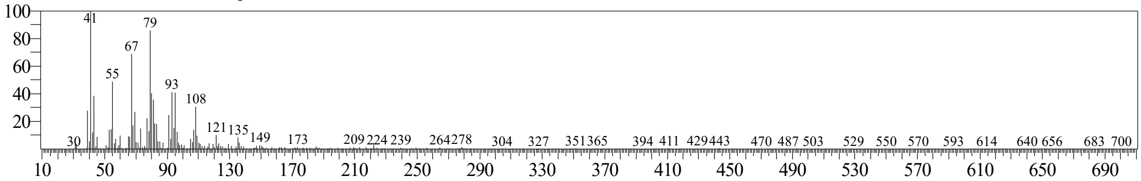

Hit#:1 Entry:101390 Library:NIST11.lib
SI:90 Formula:C18H30O2 CAS:463-40-1 MolWeight:278 RetIndex:2191
CompName:9,12,15-Octadecatrienoic acid, (Z,Z,Z)- $$ Linolenic acid $$ .alpha.-Linolenic acid $$ All-cis-9,12,15-Octadecatrienoic acid $$ cis,cis,cis-9,12,15-(

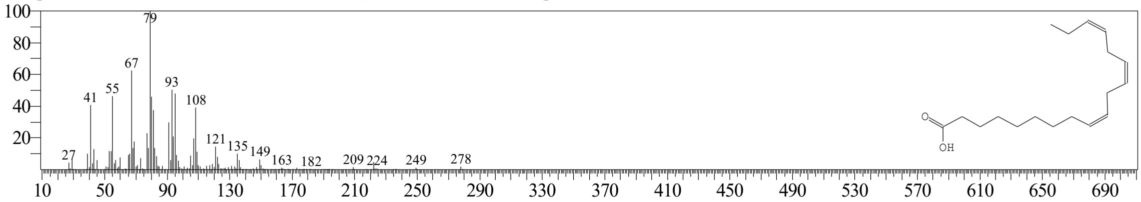

Hit#:2 Entry:90503 Library:NIST11.lib
SI:90 Formula:C18H32O CAS:506-44-5 MolWeight:264 RetIndex:2077
CompName:9,12,15-Octadecatrien-1-ol, (Z,Z,Z)- $$ (9E,12E,15E)-9,12,15-Octadecatrien-1-ol # $$ (Z,Z,Z)-9,12,15-Octadecatrien-1-ol $$

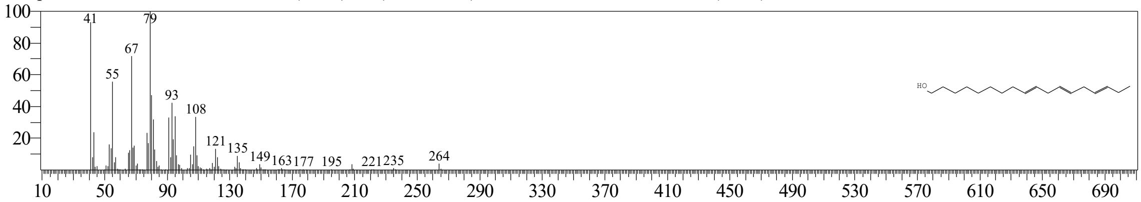

Hit#:3 Entry:67943 Library:NIST11.lib
SI:87 Formula:C16H26O CAS:56797-43-4 MolWeight:234 RetIndex:1824
CompName:cis,cis,cis-7,10,13-Hexadecatrienal $$ (7Z,10Z,13Z)-7,10,13-Hexadecatrienal # $$

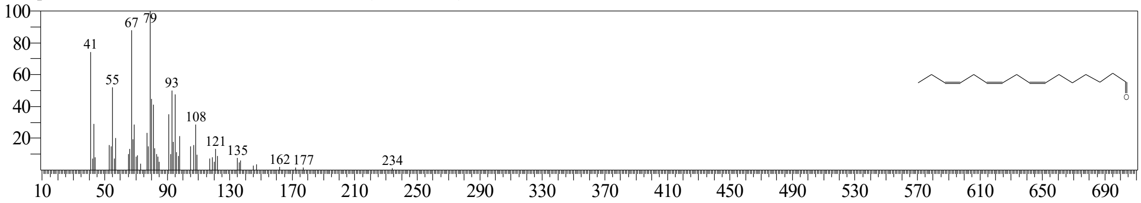

Hit#:4 Entry:101387 Library:NIST11.lib
SI:87 Formula:C18H30O2 CAS:506-26-3 MolWeight:278 RetIndex:2191
CompName:Gamolenic Acid $$ (Z,Z,Z)-6,9,12-Octadecatrienoic acid $$ .gamma.-Linolenic acid $$

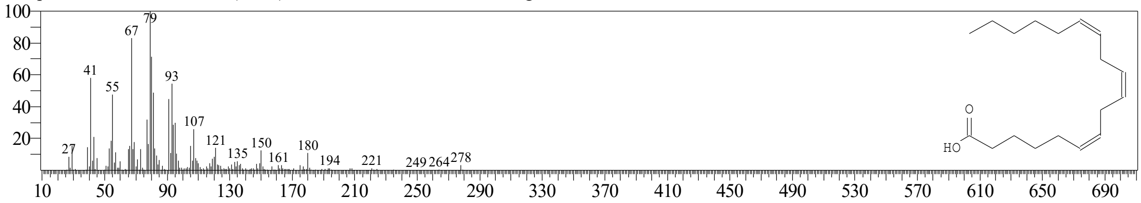

Hit#:5 Entry:89012 Library:NIST11.lib
SI:87 Formula:C18H30O CAS:26537-71-3 MolWeight:262 RetIndex:2023
CompName:9,12,15-Octadecatrienal $$ (9E,12E,15E)-9,12,15-Octadecatrienal # $$

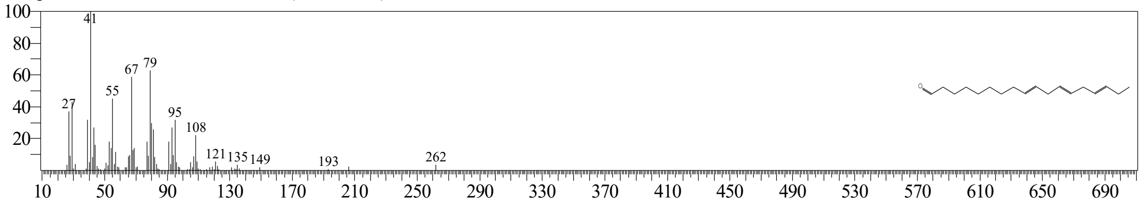

<< Target >>
Line#:79 R.Time:15.300(Scan#:2261) MassPeaks:430
RawMode:Averaged 15.295-15.305(2260-2262) BasePeak:67.10(178860)
BG Mode:Calc. from Peak Group 1 - Event 1 Scan

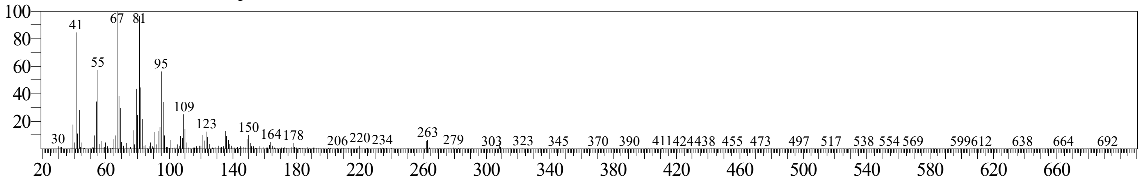

Hit#:1 Entry:124948 Library:NIST11.lib
SI:93 Formula:C20H36O2 CAS:7619-08-1 MolWeight:308 RetIndex:2193
CompName:9,12-Octadecadienoic acid, ethyl ester $$ Ethyl-9,12-octadecadienoate $$ Ethyl octadec-9,12-dienoate $$

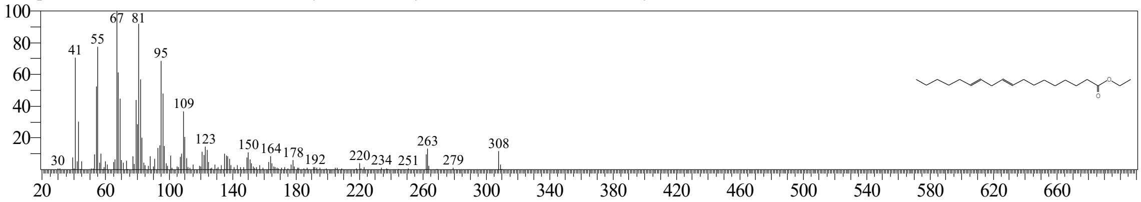

Hit#:2 Entry:113947 Library:NIST11.lib
SI:91 Formula:C19H34O2 CAS:2462-85-3 MolWeight:294 RetIndex:2093
CompName:9,12-Octadecadienoic acid, methyl ester $$ 9,12-Octadecenoic acid, methyl ester $$ Methyl octadeca-9,12-dienoate $$ Methyl 9,12-octadecadienoa

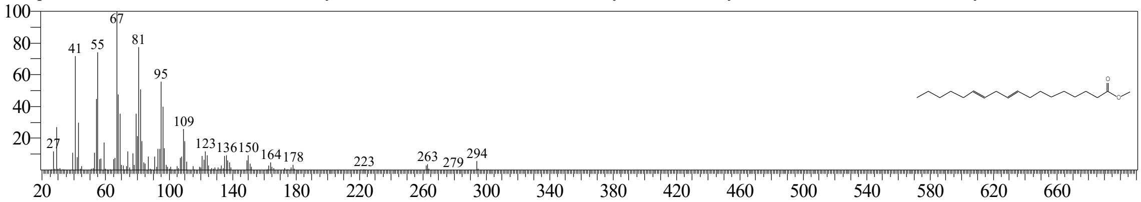

Hit#:3 Entry:158161 Library:NIST11.lib
SI:90 Formula:C21H38O4 CAS:2277-28-3 MolWeight:354 RetIndex:2697
CompName:9,12-Octadecadienoic acid (Z,Z)-, 2,3-dihydroxypropyl ester $$ Linolein, 1-mono- $$ .alpha.-Glyceryl linoleate $$ Glycerol 1-monolinolate $$ Olei

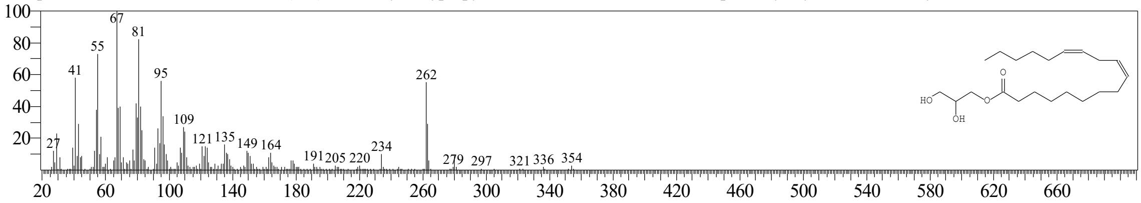

Hit#:4 Entry:113940 Library:NIST11.lib
SI:90 Formula:C19H34O2 CAS:56599-58-7 MolWeight:294 RetIndex:2093
CompName:8,11-Octadecadienoic acid, methyl ester $$ Methyl (8E,11E)-8,11-octadecadienoate # $$

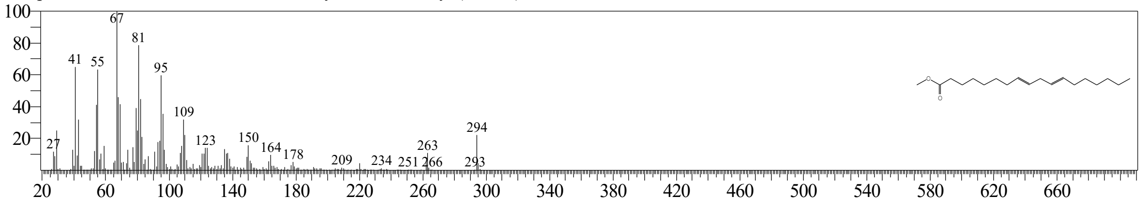

Hit#:5 Entry:113931 Library:NIST11.lib
SI:90 Formula:C19H34O2 CAS:2566-97-4 MolWeight:294 RetIndex:2093
CompName:9,12-Octadecadienoic acid, methyl ester, (E,E)- $$ Linolelaidic acid, methyl ester $$ Methyl linolelaidate $$ Methyl trans,trans-9,12-octadecadieno

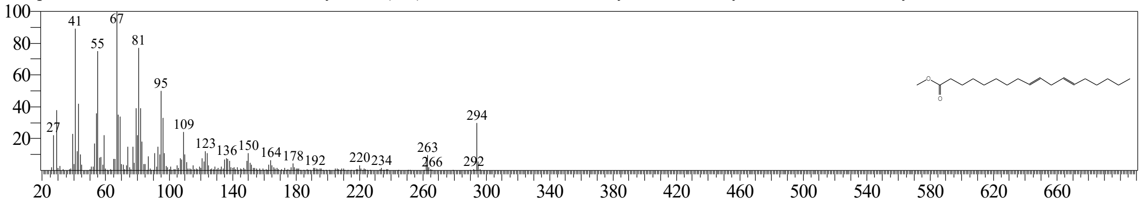

<< Target >>
Line#:80 R.Time:15.350(Scan#:2271) MassPeaks:485
RawMode:Averaged 15.345-15.355(2270-2272) BasePeak:79.10(1522534)
BG Mode:Calc. from Peak Group 1 - Event 1 Scan

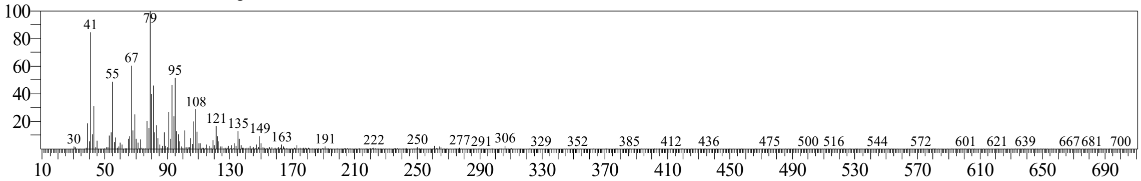

Hit#:1 Entry:101390 Library:NIST11.lib
SI:91 Formula:C18H30O2 CAS:463-40-1 MolWeight:278 RetIndex:2191
CompName:9,12,15-Octadecatrienoic acid, (Z,Z,Z)- $$ Linolenic acid $$ .alpha.-Linolenic acid $$ All-cis-9,12,15-Octadecatrienoic acid $$ cis,cis,cis-9,12,15-(

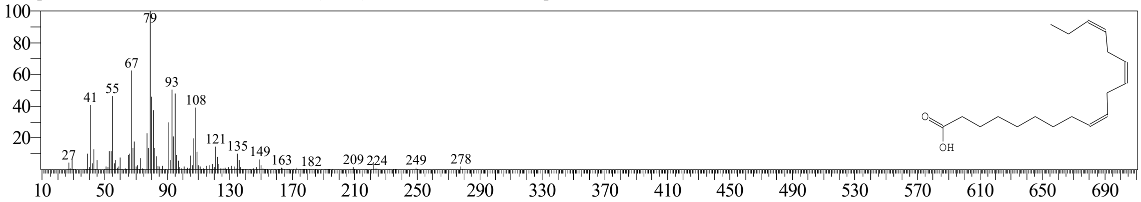

Hit#:2 Entry:90503 Library:NIST11.lib
SI:90 Formula:C18H32O CAS:506-44-5 MolWeight:264 RetIndex:2077
CompName:9,12,15-Octadecatrien-1-ol, (Z,Z,Z)- $$ (9E,12E,15E)-9,12,15-Octadecatrien-1-ol # $$ (Z,Z,Z)-9,12,15-Octadecatrien-1-ol $$

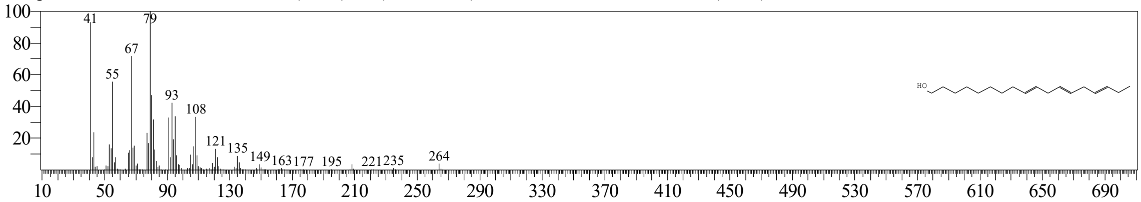

Hit#:3 Entry:67943 Library:NIST11.lib
SI:89 Formula:C16H26O CAS:56797-43-4 MolWeight:234 RetIndex:1824
CompName:cis,cis,cis-7,10,13-Hexadecatrienal $$ (7Z,10Z,13Z)-7,10,13-Hexadecatrienal # $$

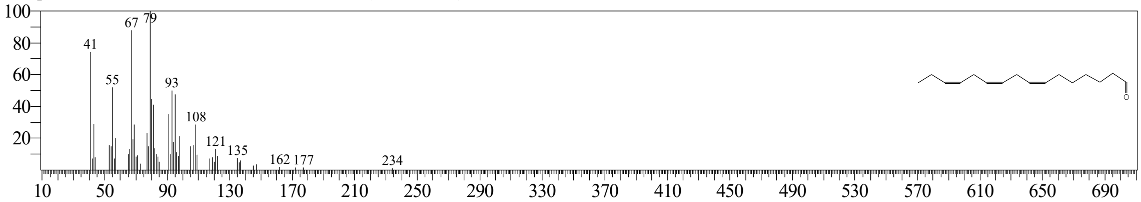

Hit#:4 Entry:67981 Library:NIST11.lib
SI:89 Formula:C17H30 CAS:74744-55-1 MolWeight:234 RetIndex:1736
CompName:3-Heptadecen-5-yne, (Z)- $$ (3Z)-3-Heptadecen-5-yne # $$

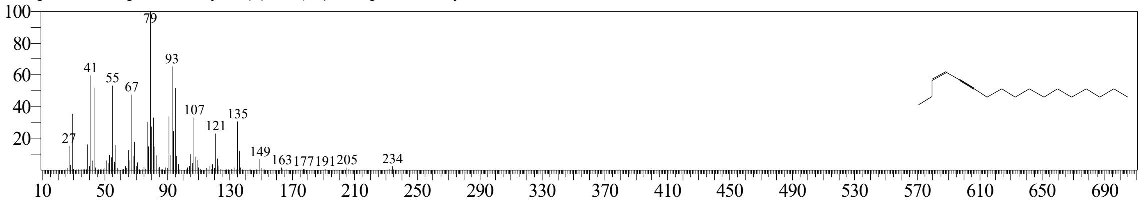

Hit#:5 Entry:132823 Library:NIST11.lib
SI:88 Formula:C21H34O2 CAS:59149-01-8 MolWeight:318 RetIndex:2308
CompName:Methyl (Z)-5,11,14,17-eicosatetraenoate $$ Methyl (5Z,11Z,14Z,17Z)-5,11,14,17-icosatetraenoate # $$

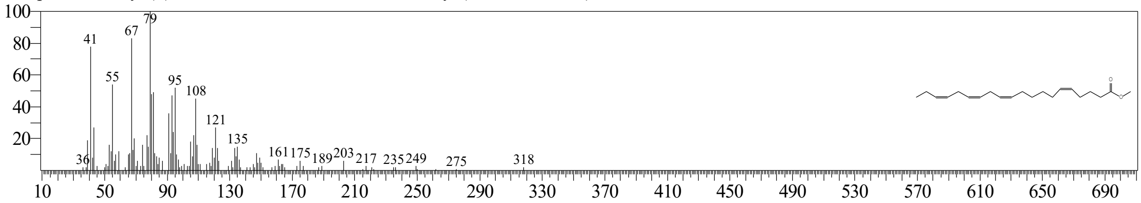

<< Target >>
Line#:81 R.Time:15.455(Scan#:2292) MassPeaks:339
RawMode:Averaged 15.450-15.460(2291-2293) BasePeak:56.05(21885)
BG Mode:Calc. from Peak Group 1 - Event 1 Scan

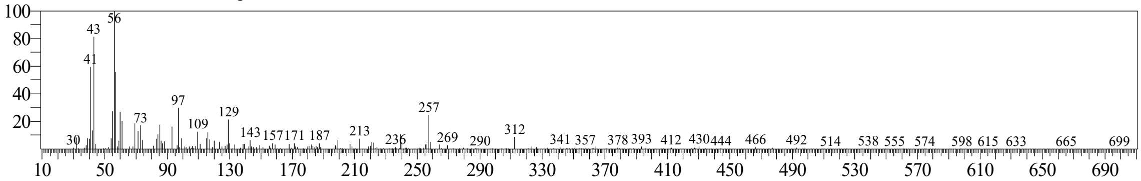

Hit#:1 Entry:128159 Library:NIST11.lib
SI:81 Formula:C20H40O2 CAS:111-06-8 MolWeight:312 RetIndex:2177
CompName:Hexadecanoic acid, butyl ester $$ Palmitic acid, butyl ester $$ n-Butyl hexadecanoate $$ n-Butyl palmitate $$ Butyl palmitate $$ Butyl ester of hex

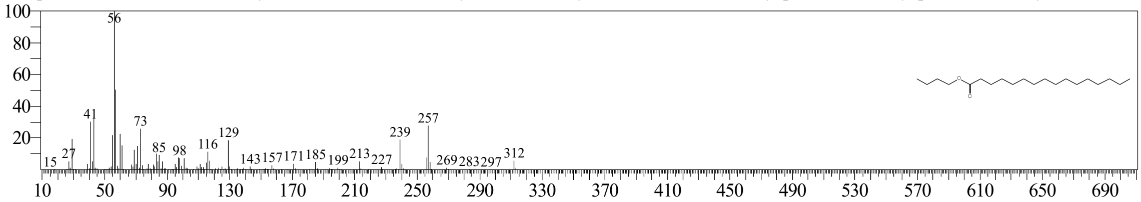

Hit#:2 Entry:128162 Library:NIST11.lib
SI:79 Formula:C20H40O2 CAS:110-34-9 MolWeight:312 RetIndex:2112
CompName:Hexadecanoic acid, 2-methylpropyl ester $$ 2-methylpropyl hexadecanoate $$ isobutyl hexadecanoate $$

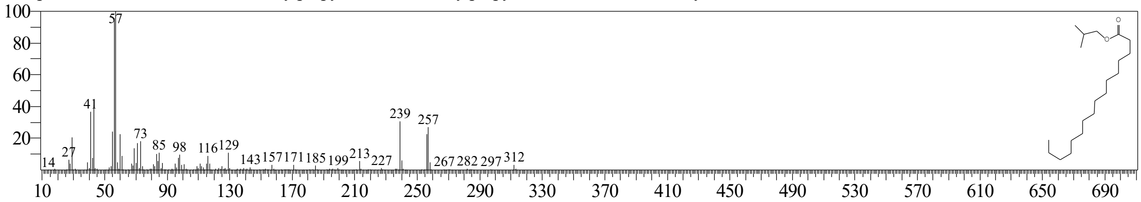

Hit#:3 Entry:128158 Library:NIST11.lib
SI:79 Formula:C20H40O2 CAS:31158-91-5 MolWeight:312 RetIndex:2092
CompName:Hexadecanoic acid, 1,1-dimethylethyl ester $$ tert-Butyl palmitate # $$

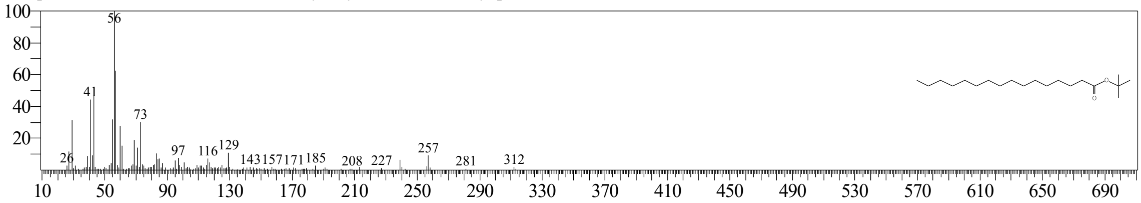

Hit#:4 Entry:106162 Library:NIST11.lib
SI:75 Formula:C18H36O2 CAS:110-36-1 MolWeight:284 RetIndex:1978
CompName:Butyl myristate $$ n-Butyl myristate $$ Myristic acid n-butyl ester $$ Tetradecanoic acid, butyl ester $$ Bumyr $$ Myristic acid, butyl ester $$ Buty

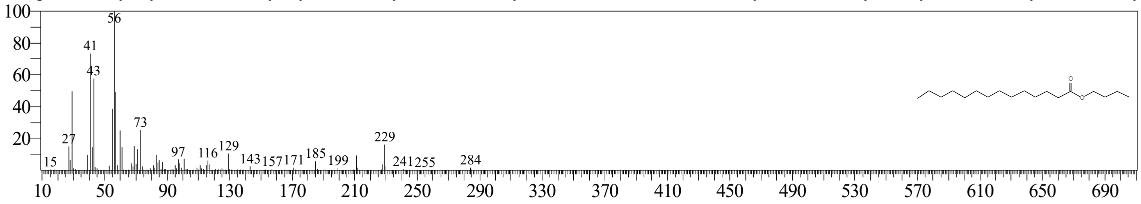

Hit#:5 Entry:84362 Library:NIST11.lib
SI:73 Formula:C16H32O2 CAS:57-10-3 MolWeight:256 RetIndex:1968
CompName:n-Hexadecanoic acid $$ Hexadecanoic acid $$ n-Hexadecoic acid $$ Palmitic acid $$ Pentadecanecarboxylic acid $$ 1-Pentadecanecarboxylic acid

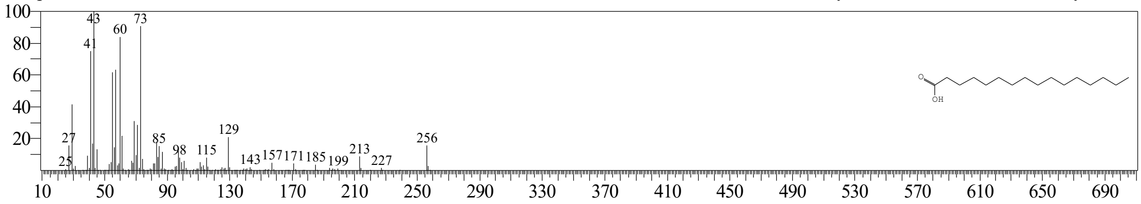

<< Target >>
Line#:82 R.Time:15.505(Scan#:2302) MassPeaks:456
RawMode:Averaged 15.500-15.510(2301-2303) BasePeak:88.05(1016942)
BG Mode:Calc. from Peak Group 1 - Event 1 Scan

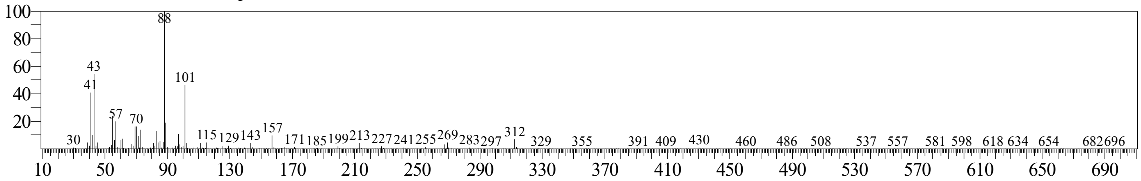

Hit#:1 Entry:149096 Library:NIST11.lib
SI:90 Formula:C22H44O2 CAS:18281-05-5 MolWeight:340 RetIndex:2375
CompName:Eicosanoic acid, ethyl ester $$ Ethyl icosanoate $$ Ethyl arachidate $$ Ethyl eicosanoate $$ Arachidic acid, ethyl ester $$

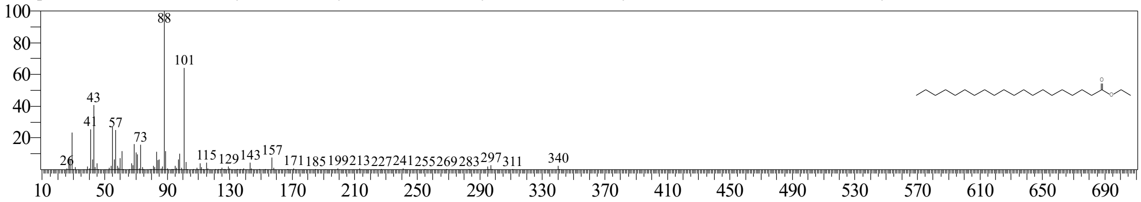

Hit#:2 Entry:138751 Library:NIST11.lib
SI:90 Formula:C21H42O2 CAS:18281-04-4 MolWeight:326 RetIndex:2276
CompName:Nonadecanoic acid, ethyl ester $$ Ethyl nonadecanoate # $$

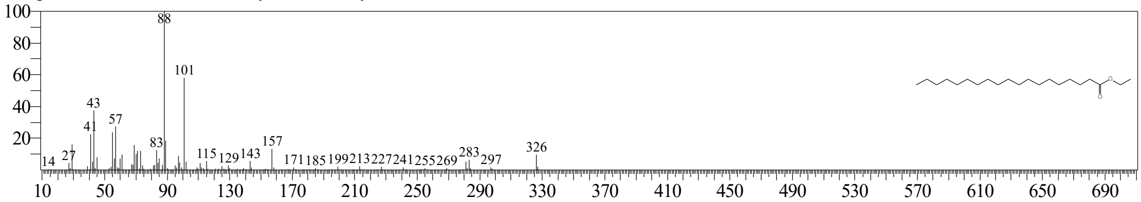

Hit#:3 Entry:117163 Library:NIST11.lib
SI:90 Formula:C19H38O2 CAS:14010-23-2 MolWeight:298 RetIndex:2077
CompName:Heptadecanoic acid, ethyl ester $$ Ethyl heptadecanoate $$ Ethyl n-heptadecanoate $$

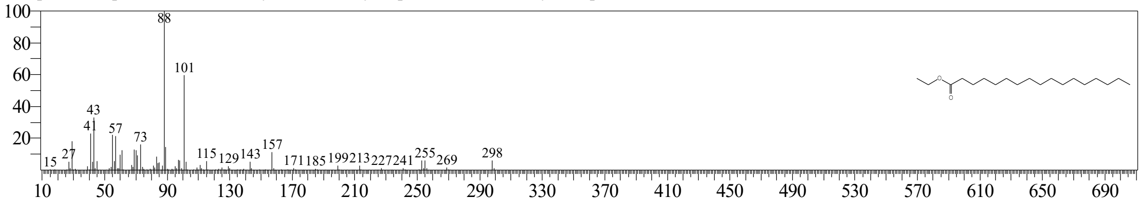

Hit#:4 Entry:95197 Library:NIST11.lib
SI:89 Formula:C17H34O2 CAS:41114-00-5 MolWeight:270 RetIndex:1878
CompName:Pentadecanoic acid, ethyl ester $$ Ethyl pentadecanoate $$ n-Pentadecanoic acid ethyl ester $$

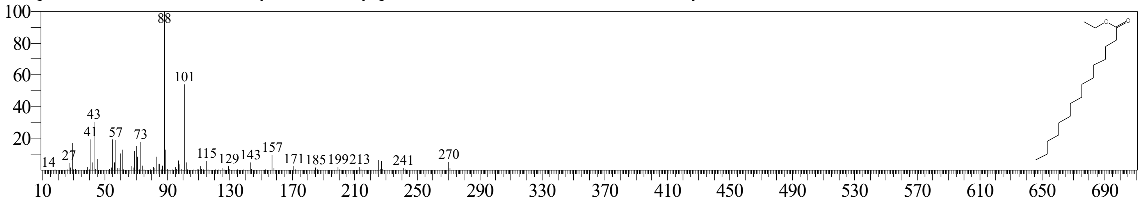

Hit#:5 Entry:106188 Library:NIST11.lib
SI:89 Formula:C18H36O2 CAS:628-97-7 MolWeight:284 RetIndex:1978
CompName:Hexadecanoic acid, ethyl ester $$ Palmitic acid, ethyl ester $$ Ethyl hexadecanoate $$ Ethyl palmitate $$ Ethyl n-hexadecanoate $$

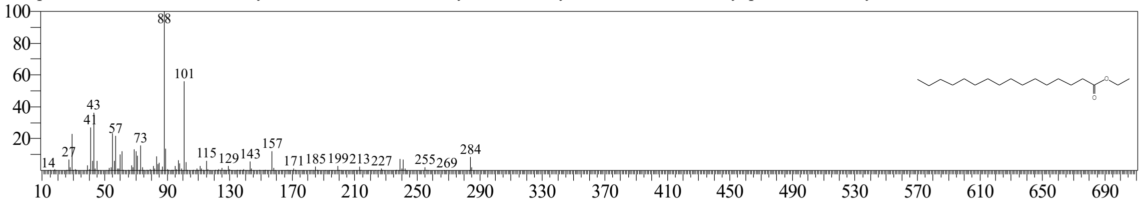

<< Target >>
Line#:83 R.Time:15.690(Scan#:2339) MassPeaks:349
RawMode:Averaged 15.685-15.695(2338-2340) BasePeak:123.10(14004)
BG Mode:Calc. from Peak Group 1 - Event 1 Scan

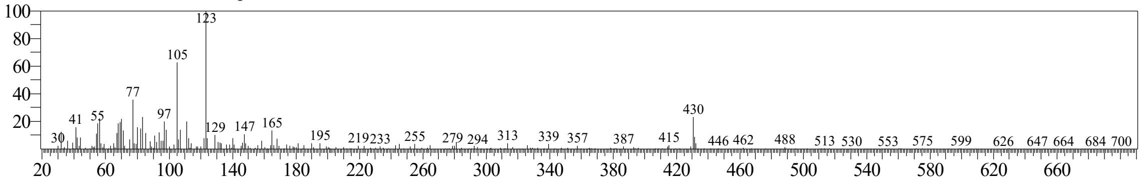

Hit#:1 Entry:132854 Library:NIST11.lib
SI:70 Formula:C21H34O2 CAS:0-00-0 MolWeight:318 RetIndex:2353
CompName:Benzoic acid, tetradecyl ester

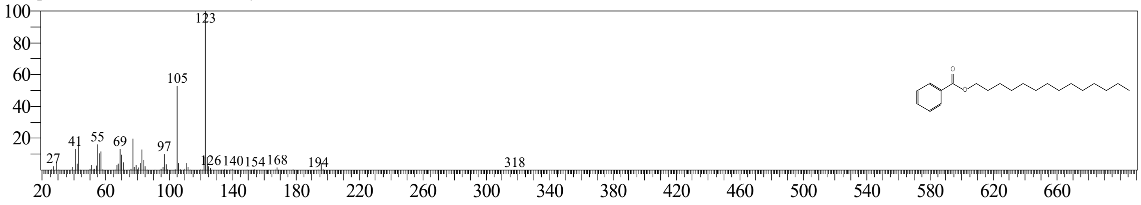

Hit#:2 Entry:121896 Library:NIST11.lib
SI:69 Formula:C20H32O2 CAS:0-00-0 MolWeight:304 RetIndex:2253
CompName:Benzoic acid, tridecyl ester

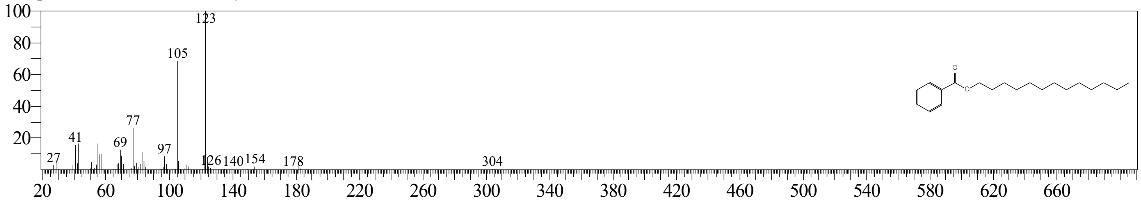

Hit#:3 Entry:183288 Library:NIST11.lib
SI:68 Formula:C24H47ClO2 CAS:0-00-0 MolWeight:402 RetIndex:2799
CompName:4-Chlorobutyric acid, eicosyl ester

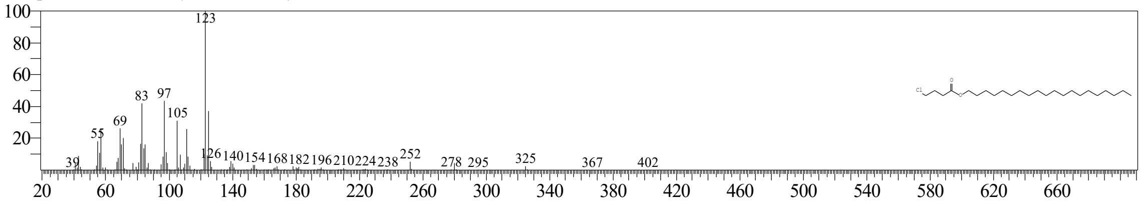

Hit#:4 Entry:143354 Library:NIST11.lib
SI:68 Formula:C22H36O2 CAS:0-00-0 MolWeight:332 RetIndex:2452
CompName:Benzoic acid, pentadecyl ester

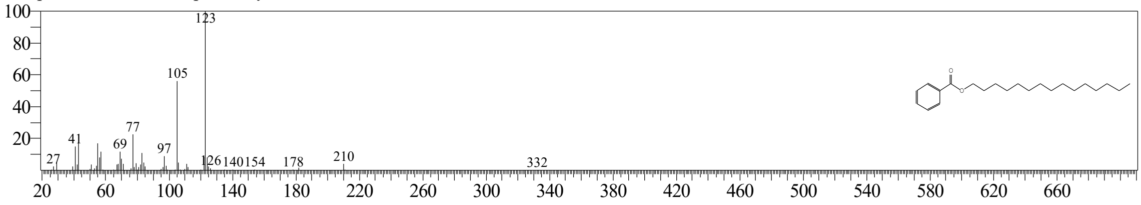

Hit#:5 Entry:153268 Library:NIST11.lib
SI:68 Formula:C23H38O2 CAS:0-00-0 MolWeight:346 RetIndex:2551
CompName:Benzoic acid, hexadecyl ester

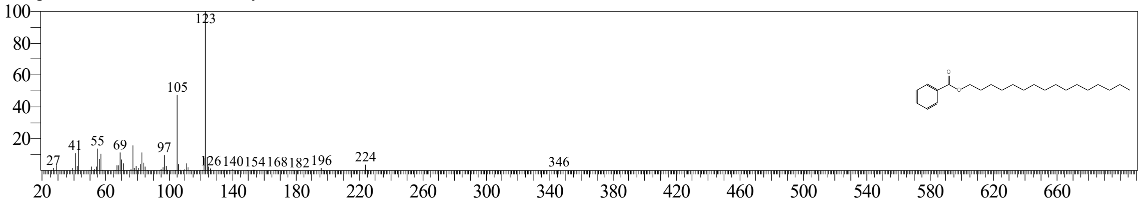

<< Target >>
Line#:84 R.Time:16.260(Scan#:2453) MassPeaks:372
RawMode:Averaged 16.255-16.265(2452-2454) BasePeak:88.10(19452)
BG Mode:Calc. from Peak Group 1 - Event 1 Scan

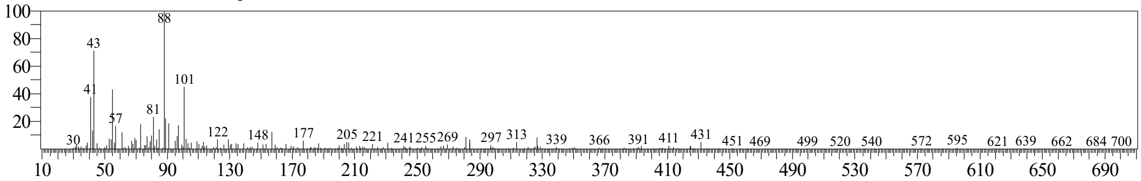

Hit#:1 Entry:138751 Library:NIST11.lib
SI:75 Formula:C21H42O2 CAS:18281-04-4 MolWeight:326 RetIndex:2276
CompName:Nonadecanoic acid, ethyl ester $$ Ethyl nonadecanoate # $$

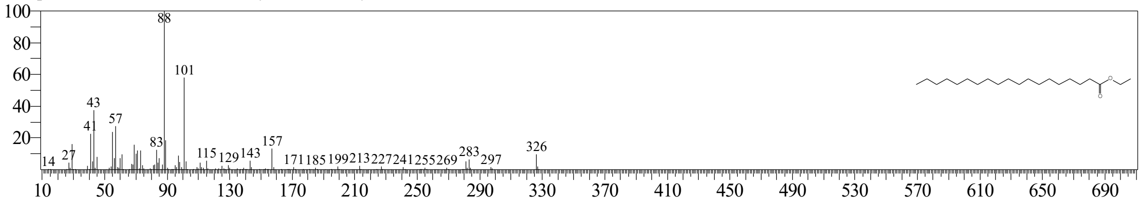

Hit#:2 Entry:166830 Library:NIST11.lib
SI:74 Formula:C24H48O2 CAS:5908-87-2 MolWeight:368 RetIndex:2574
CompName:Docosanoic acid, ethyl ester $$ Ethyl docosanoate # $$

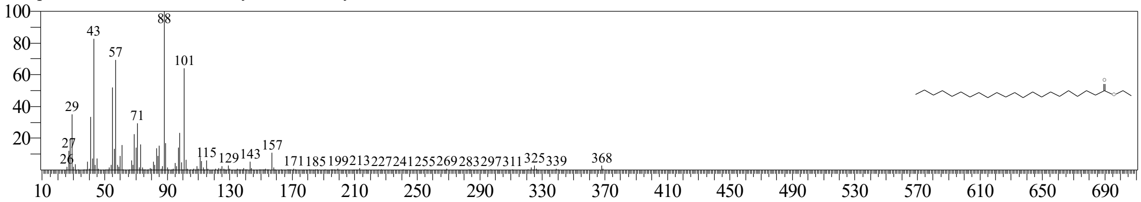

Hit#:3 Entry:73860 Library:NIST11.lib
SI:73 Formula:C15H30O2 CAS:28267-29-0 MolWeight:242 RetIndex:1680
CompName:Ethyl tridecanoate $$ Tridecanoic acid ethyl ester $$ n-Tridecanoic acid ethyl ester $$

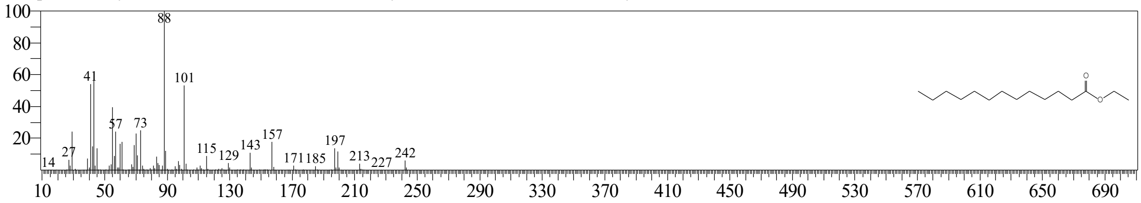

Hit#:4 Entry:95196 Library:NIST11.lib
SI:73 Formula:C17H34O2 CAS:0-00-0 MolWeight:270 RetIndex:1686
CompName:Methyl 2,6,10-trimethyltridecanoate

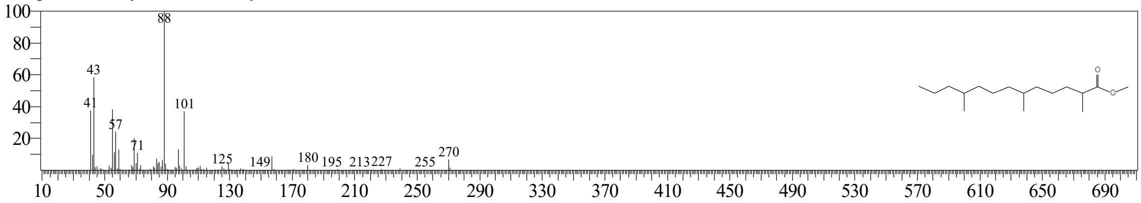

Hit#:5 Entry:149096 Library:NIST11.lib
SI:73 Formula:C22H44O2 CAS:18281-05-5 MolWeight:340 RetIndex:2375
CompName:Eicosanoic acid, ethyl ester $$ Ethyl icosanoate $$ Ethyl arachidate $$ Ethyl eicosanoate $$ Arachidic acid, ethyl ester $$

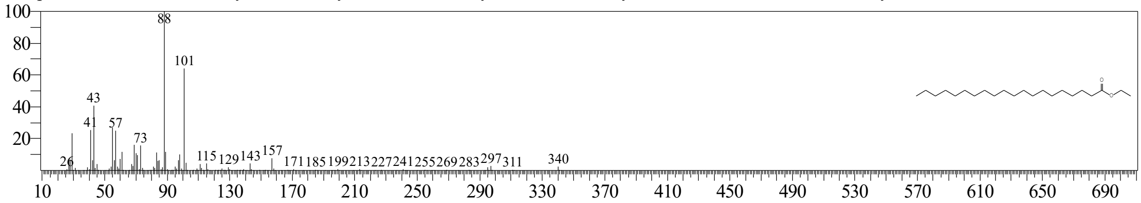

<< Target >>
Line#:85 R.Time:16.495(Scan#:2500) MassPeaks:349
RawMode:Averaged 16.490-16.500(2499-2501) BasePeak:122.95(16790)
BG Mode:Calc. from Peak Group 1 - Event 1 Scan

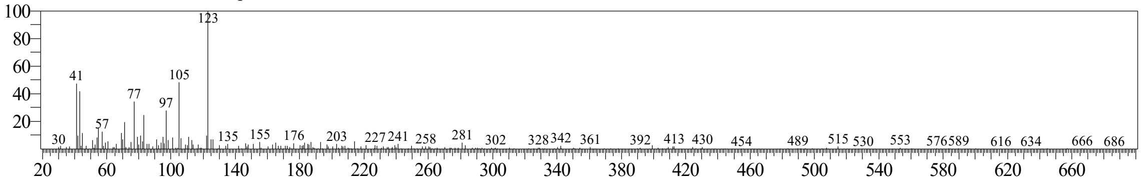

Hit#:1 Entry:143354 Library:NIST11.lib
SI:75 Formula:C22H36O2 CAS:0-00-0 MolWeight:332 RetIndex:2452
CompName:Benzoic acid, pentadecyl ester

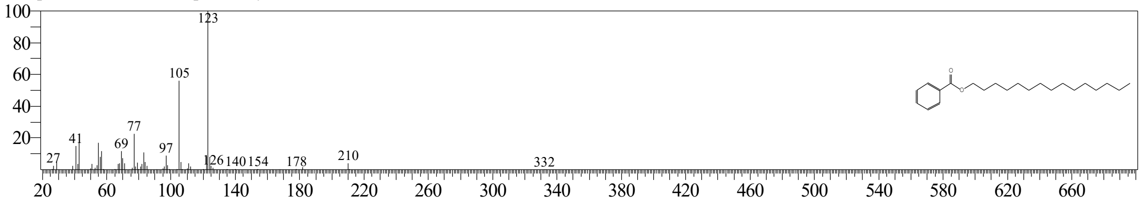

Hit#:2 Entry:121896 Library:NIST11.lib
SI:74 Formula:C20H32O2 CAS:0-00-0 MolWeight:304 RetIndex:2253
CompName:Benzoic acid, tridecyl ester

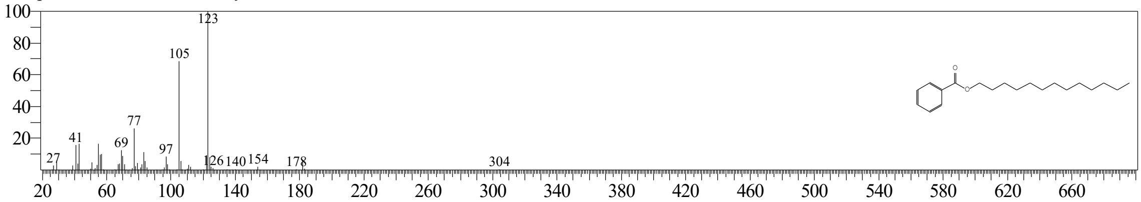

Hit#:3 Entry:132854 Library:NIST11.lib
SI:74 Formula:C21H34O2 CAS:0-00-0 MolWeight:318 RetIndex:2353
CompName:Benzoic acid, tetradecyl ester

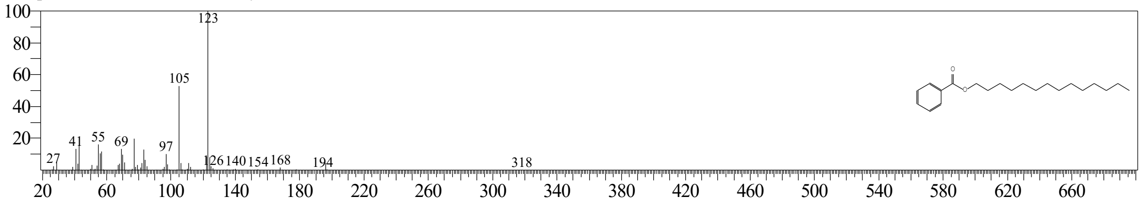

Hit#:4 Entry:153268 Library:NIST11.lib
SI:73 Formula:C23H38O2 CAS:0-00-0 MolWeight:346 RetIndex:2551
CompName:Benzoic acid, hexadecyl ester

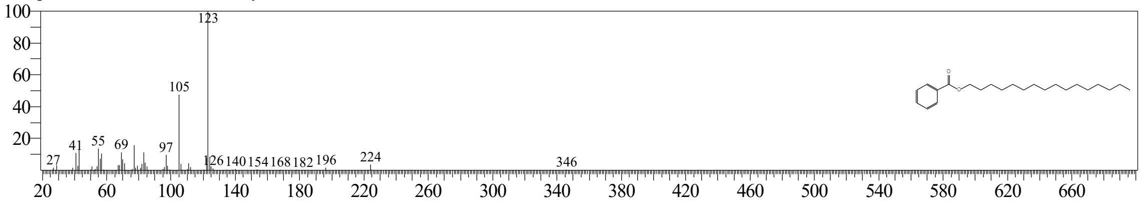

Hit#:5 Entry:170440 Library:NIST11.lib
SI:72 Formula:C25H42O2 CAS:0-00-0 MolWeight:374 RetIndex:2750
CompName:Benzoic acid, octadecyl ester

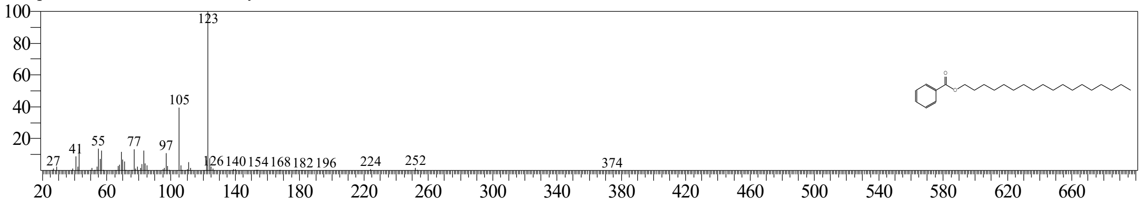

<< Target >>
Line#:86 R.Time:16.880(Scan#:2577) MassPeaks:415
RawMode:Averaged 16.875-16.885(2576-2578) BasePeak:79.05(13641)
BG Mode:Calc. from Peak Group 1 - Event 1 Scan

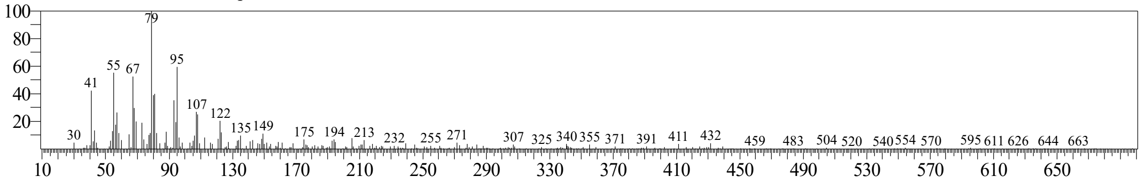

Hit#:1 Entry:101390 Library:NIST11.lib
SI:81 Formula:C18H30O2 CAS:463-40-1 MolWeight:278 RetIndex:2191
CompName:9,12,15-Octadecatrienoic acid, (Z,Z,Z)- $$ Linolenic acid $$ .alpha.-Linolenic acid $$ All-cis-9,12,15-Octadecatrienoic acid $$ cis,cis,cis-9,12,15-(

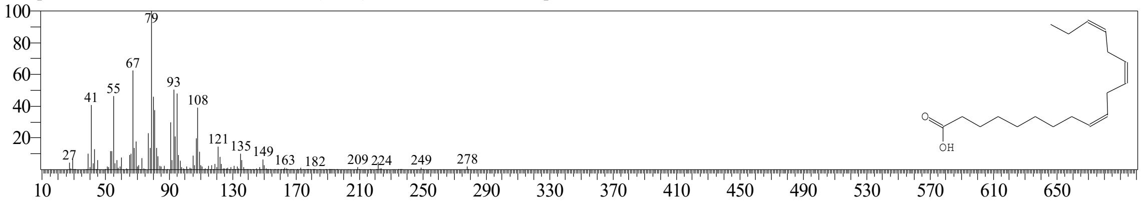

Hit#:2 Entry:90503 Library:NIST11.lib
SI:78 Formula:C18H32O CAS:506-44-5 MolWeight:264 RetIndex:2077
CompName:9,12,15-Octadecatrien-1-ol, (Z,Z,Z)- $$ (9E,12E,15E)-9,12,15-Octadecatrien-1-ol # $$ (Z,Z,Z)-9,12,15-Octadecatrien-1-ol $$

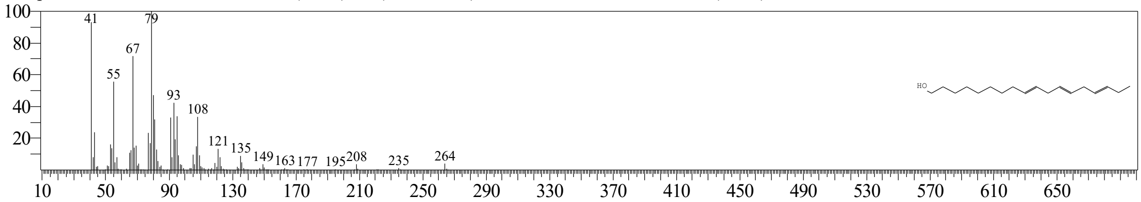

Hit#:3 Entry:132823 Library:NIST11.lib
SI:78 Formula:C21H34O2 CAS:59149-01-8 MolWeight:318 RetIndex:2308
CompName:Methyl (Z)-5,11,14,17-eicosatetraenoate $$ Methyl (5Z,11Z,14Z,17Z)-5,11,14,17-icosatetraenoate # $$

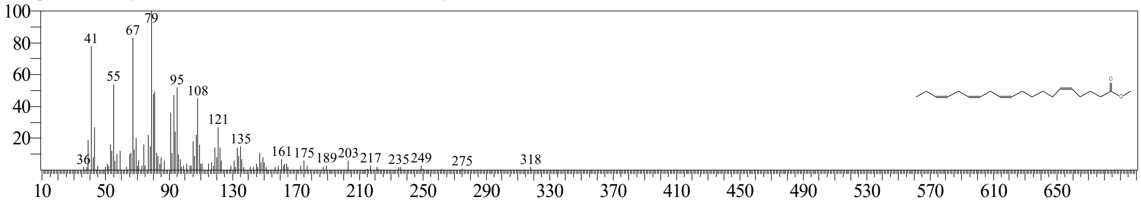

Hit#:4 Entry:67981 Library:NIST11.lib
SI:78 Formula:C17H30 CAS:74744-55-1 MolWeight:234 RetIndex:1736
CompName:3-Heptadecen-5-yne, (Z)- $$ (3Z)-3-Heptadecen-5-yne # $$

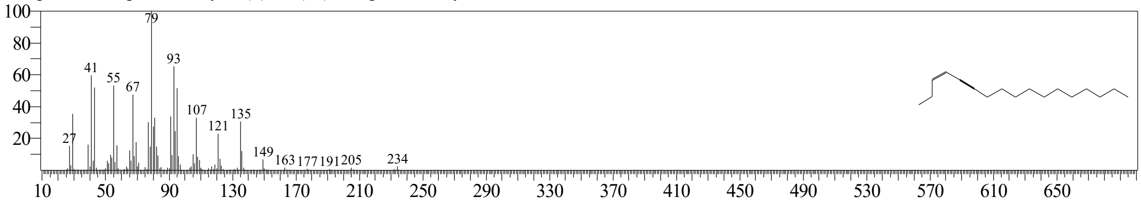

Hit#:5 Entry:112450 Library:NIST11.lib
SI:77 Formula:C19H32O2 CAS:301-00-8 MolWeight:292 RetIndex:2101
CompName:9,12,15-Octadecatrienoic acid, methyl ester, (Z,Z,Z)- $$ Linolenic acid, methyl ester $$ Methyl all-cis-9,12,15-octadecatrienoate $$ Methyl linolen

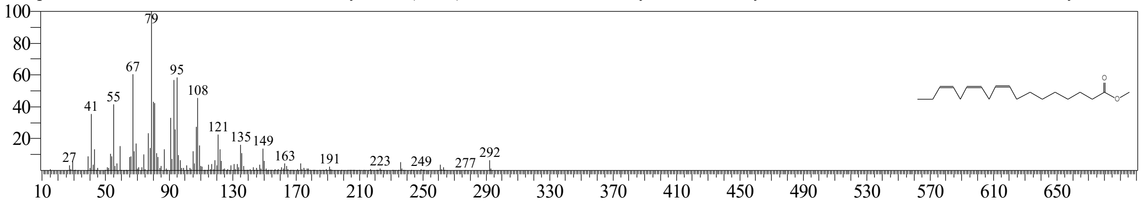

<< Target >>
Line#:87 R.Time:16.960(Scan#:2593) MassPeaks:411
RawMode:Averaged 16.955-16.965(2592-2594) BasePeak:79.10(9346)
BG Mode:Calc. from Peak Group 1 - Event 1 Scan

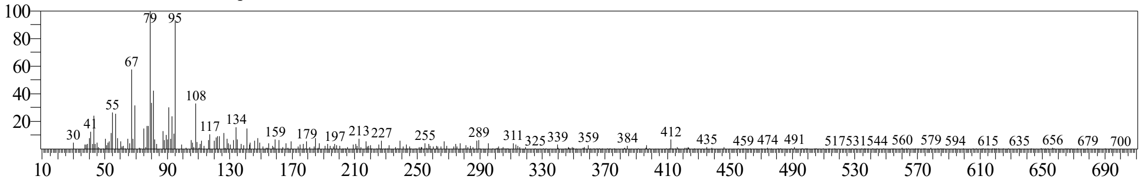

Hit#:1 Entry:101390 Library:NIST11.lib
SI:73 Formula:C18H30O2 CAS:463-40-1 MolWeight:278 RetIndex:2191
CompName:9,12,15-Octadecatrienoic acid, (Z,Z,Z)- $$ Linolenic acid $$ .alpha.-Linolenic acid $$ All-cis-9,12,15-Octadecatrienoic acid $$ cis,cis,cis-9,12,15-

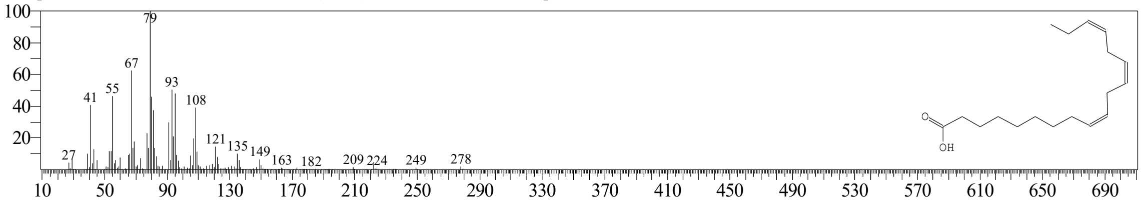

Hit#:2 Entry:112450 Library:NIST11.lib
SI:72 Formula:C19H32O2 CAS:301-00-8 MolWeight:292 RetIndex:2101
CompName:9,12,15-Octadecatrienoic acid, methyl ester, (Z,Z,Z)- $$ Linolenic acid, methyl ester $$ Methyl all-cis-9,12,15-octadecatrienoate $$ Methyl linolen

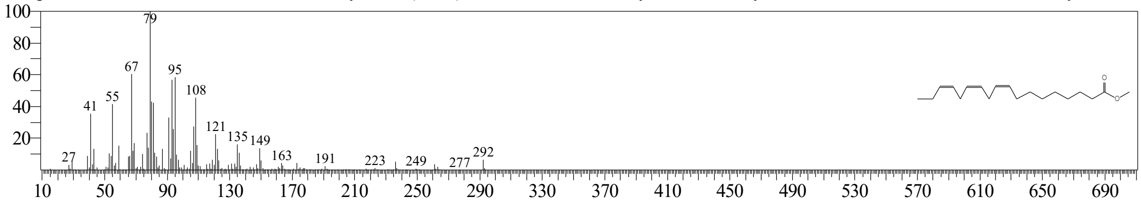

Hit#:3 Entry:101388 Library:NIST11.lib
SI:72 Formula:C18H30O2 CAS:0-00-0 MolWeight:278 RetIndex:2002
CompName:Methyl 8,11,14-heptadecatrienoate

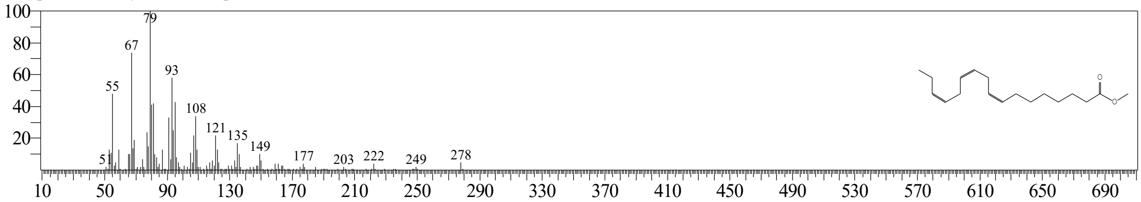

Hit#:4 Entry:124861 Library:NIST11.lib
SI:72 Formula:C19H32O3 CAS:0-00-0 MolWeight:308 RetIndex:2263
CompName:Methyl 2-hydroxy-octadeca-9,12,15-trienoate

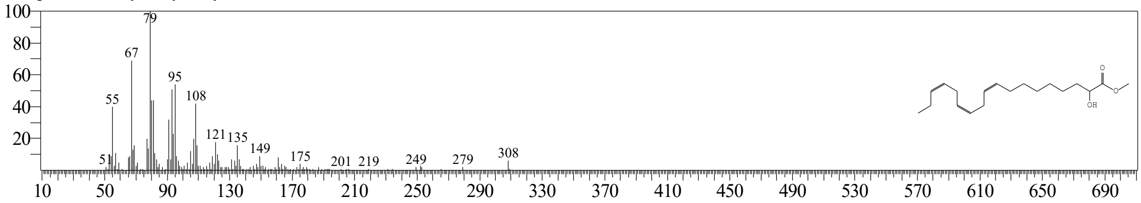

Hit#:5 Entry:90503 Library:NIST11.lib
SI:71 Formula:C18H32O CAS:506-44-5 MolWeight:264 RetIndex:2077
CompName:9,12,15-Octadecatrien-1-ol, (Z,Z,Z)- $$ (9E,12E,15E)-9,12,15-Octadecatrien-1-ol # $$ (Z,Z,Z)-9,12,15-Octadecatrien-1-ol $$

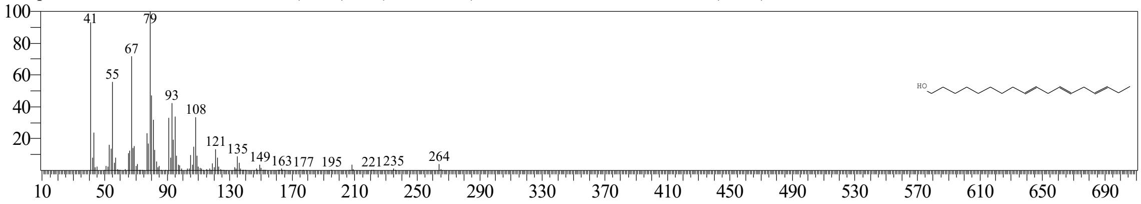

<< Target >>
Line#:88 R.Time:17.160(Scan#:2633) MassPeaks:401
RawMode:Averaged 17.155-17.165(2632-2634) BasePeak:88.05(47810)
BG Mode:Calc. from Peak Group 1 - Event 1 Scan

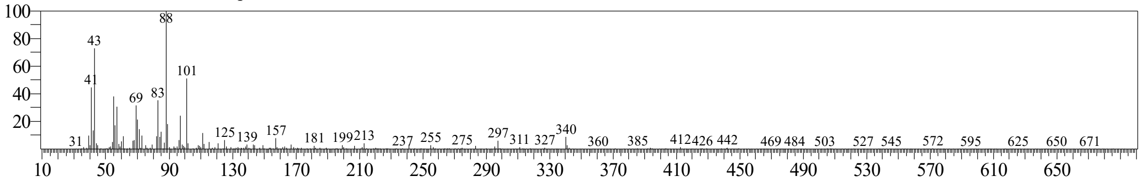

Hit#:1 Entry:166830 Library:NIST11.lib
SI:86 Formula:C24H48O2 CAS:5908-87-2 MolWeight:368 RetIndex:2574
CompName:Docosanoic acid, ethyl ester $$ Ethyl docosanoate # $$

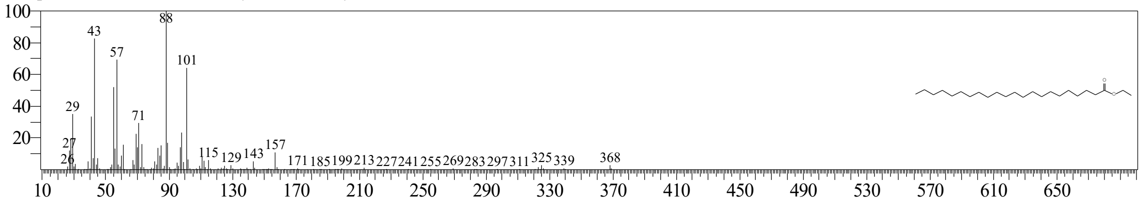

Hit#:2 Entry:149096 Library:NIST11.lib
SI:85 Formula:C22H44O2 CAS:18281-05-5 MolWeight:340 RetIndex:2375
CompName:Eicosanoic acid, ethyl ester $$ Ethyl icosanoate $$ Ethyl arachidate $$ Ethyl eicosanoate $$ Arachidic acid, ethyl ester $$

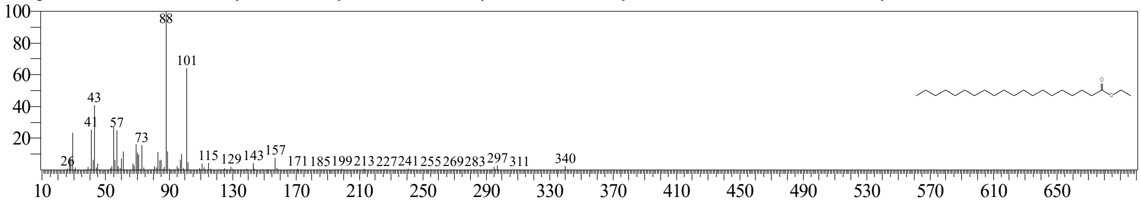

Hit#:3 Entry:138751 Library:NIST11.lib
SI:83 Formula:C21H42O2 CAS:18281-04-4 MolWeight:326 RetIndex:2276
CompName:Nonadecanoic acid, ethyl ester $$ Ethyl nonadecanoate # $$

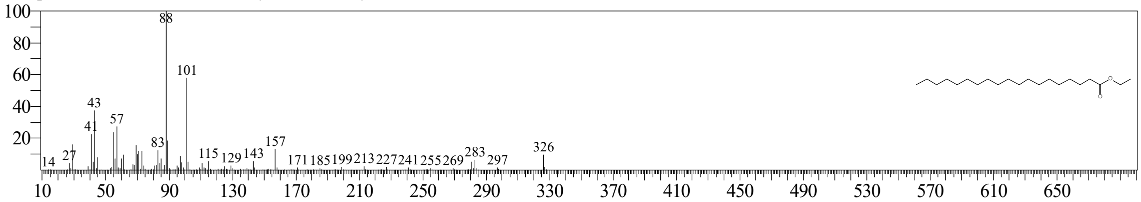

Hit#:4 Entry:73860 Library:NIST11.lib
SI:83 Formula:C15H30O2 CAS:28267-29-0 MolWeight:242 RetIndex:1680
CompName:Ethyl tridecanoate $$ Tridecanoic acid ethyl ester $$ n-Tridecanoic acid ethyl ester $$

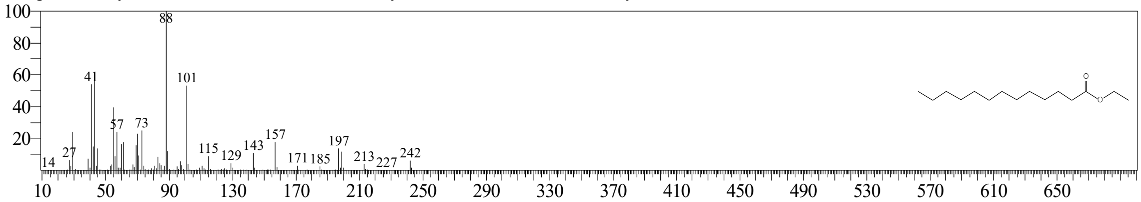

Hit#:5 Entry:190419 Library:NIST11.lib
SI:82 Formula:C28H56O2 CAS:55335-03-0 MolWeight:424 RetIndex:2908
CompName:Hexacosanoic acid, 2-methyl-, methyl ester $$ Methyl 2-methylhexacosanoate $$

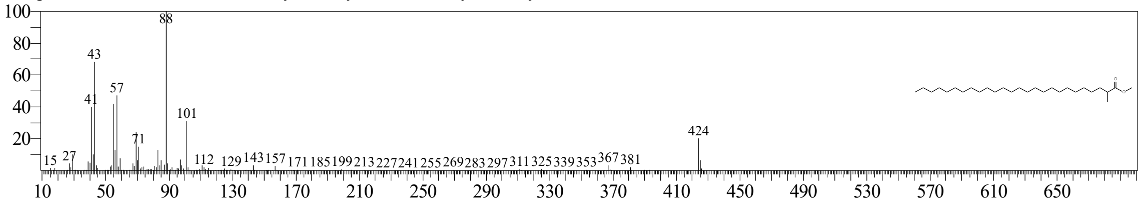

<< Target >>
Line#:89 R.Time:17.205(Scan#:2642) MassPeaks:348
RawMode:Averaged 17.200-17.210(2641-2643) BasePeak:43.05(21409)
BG Mode:Calc. from Peak Group 1 - Event 1 Scan

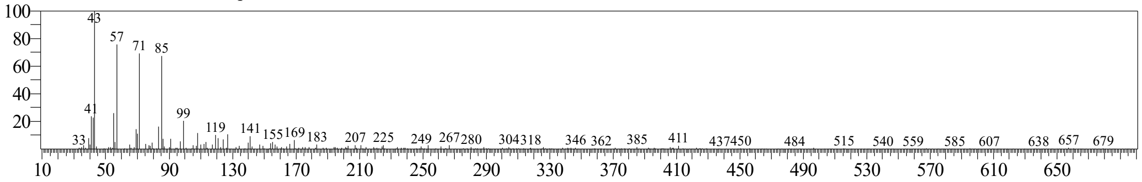

Hit#:1 Entry:62481 Library:NIST11.lib
SI:81 Formula:C16H34 CAS:55045-09-5 MolWeight:226 RetIndex:1548
CompName:Tridecane, 7-propyl- $$ 7-n-Propyltridecane $$ 7-Propyltridecane # $$

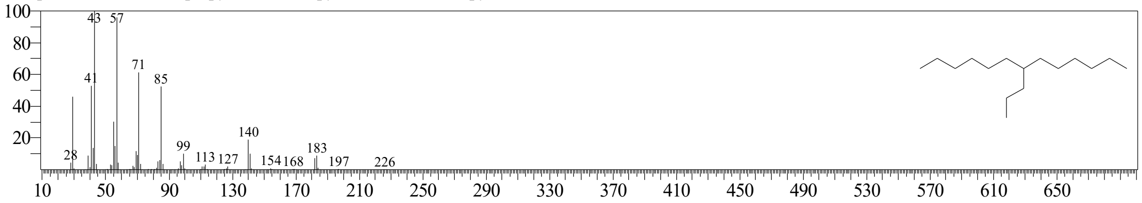

Hit#:2 Entry:93606 Library:NIST11.lib
SI:81 Formula:C19H40 CAS:61868-03-9 MolWeight:268 RetIndex:1782
CompName:Heptadecane, 2,3-dimethyl- $$ 2,3-Dimethylheptadecane # $$

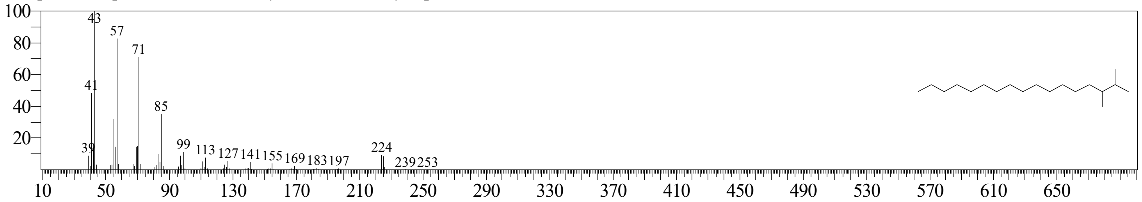

Hit#:3 Entry:34144 Library:NIST11.lib
SI:80 Formula:C13H28 CAS:6117-97-1 MolWeight:184 RetIndex:1249
CompName:Dodecane, 4-methyl- $$ 4-Methyldodecane $$

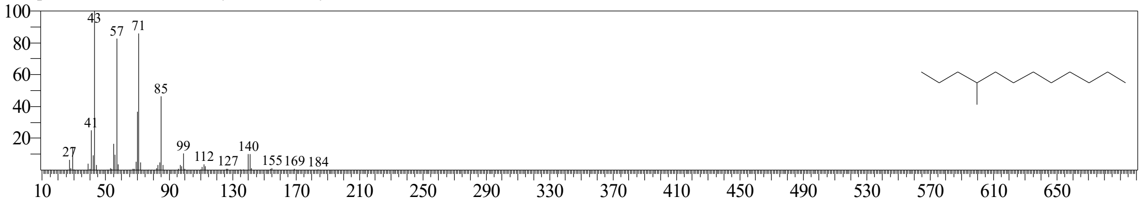

Hit#:4 Entry:104535 Library:NIST11.lib
SI:80 Formula:C20H42 CAS:112-95-8 MolWeight:282 RetIndex:2009
CompName:Eicosane $$ n-Eicosane $$ Icosane # $$ n-Icosane $$

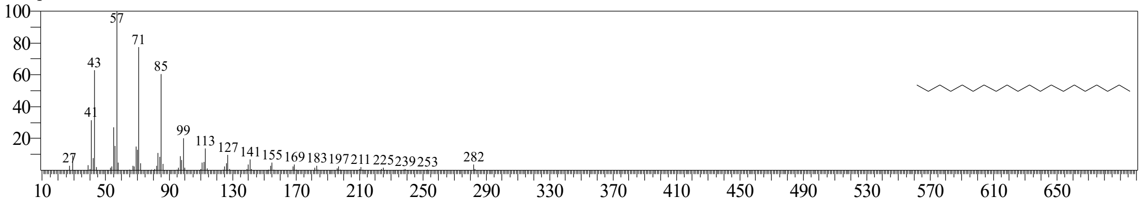

Hit#:5 Entry:173386 Library:NIST11.lib
SI:80 Formula:C27H56 CAS:593-49-7 MolWeight:380 RetIndex:2705
CompName:Heptacosane $$ n-Heptacosane $$

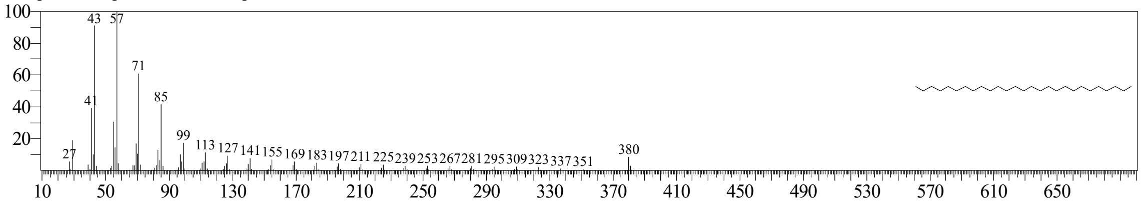

<< Target >>
Line#:90 R.Time:17.275(Scan#:2656) MassPeaks:364
RawMode:Averaged 17.270-17.280(2655-2657) BasePeak:41.10(10097)
BG Mode:Calc. from Peak Group 1 - Event 1 Scan

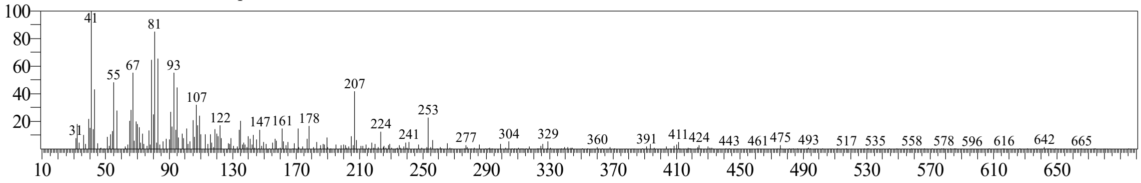

Hit#:1 Entry:86005 Library:NIST11.lib
SI:74 Formula:C18H26O CAS:0-00-0 MolWeight:258 RetIndex:1811
CompName:1,3-Bis-(2-cyclopropyl,2-methylcyclopropyl)-but-2-en-1-one

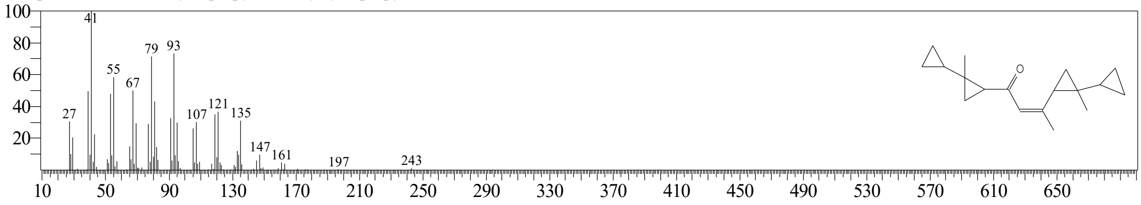

Hit#:2 Entry:56385 Library:NIST11.lib
SI:73 Formula:C16H26 CAS:71673-32-0 MolWeight:218 RetIndex:1627
CompName:1,11-Hexadecadiyne

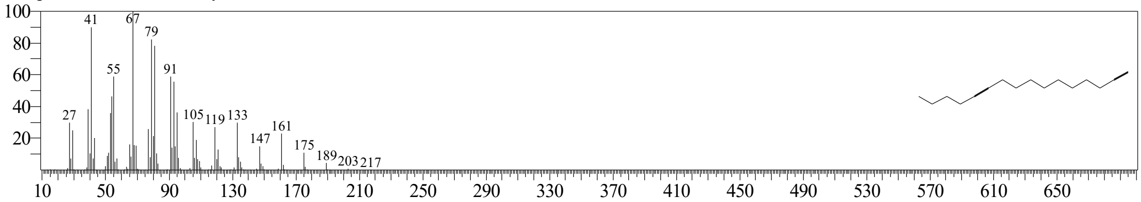

Hit#:3 Entry:132823 Library:NIST11.lib
SI:73 Formula:C21H34O2 CAS:59149-01-8 MolWeight:318 RetIndex:2308
CompName:Methyl (Z)-5,11,14,17-eicosatetraenoate $$ Methyl (5Z,11Z,14Z,17Z)-5,11,14,17-icosatetraenoate # $$

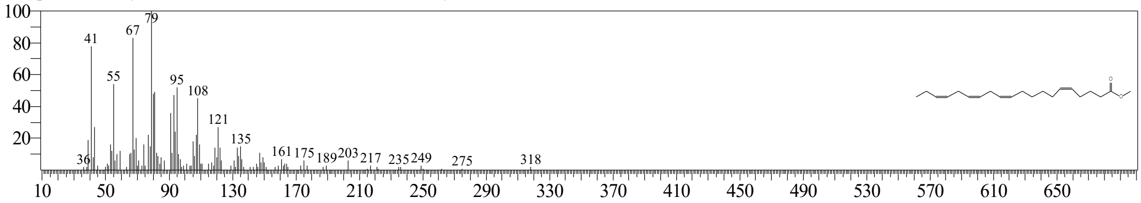

Hit#:4 Entry:30491 Library:NIST11.lib
SI:73 Formula:C13H22 CAS:50746-55-9 MolWeight:178 RetIndex:1187
CompName:Bicyclo[3.1.1]heptane, 2,6,6-trimethyl-3-(2-propenyl)-, (1.alpha.,2.beta.,3.alpha.,5.alpha.)- $$ 3-Allyl-2,6,6-trimethylbicyclo[3.1.1]heptane # $$

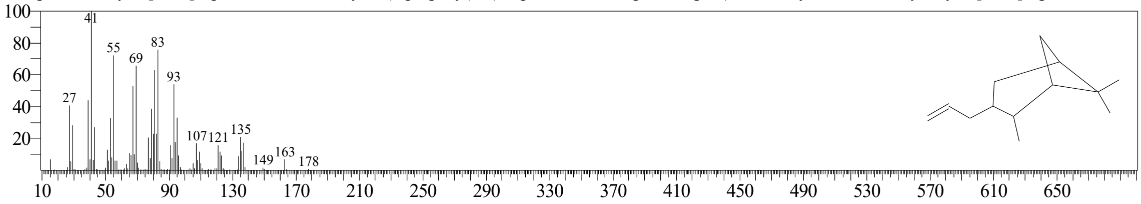

Hit#:5 Entry:67943 Library:NIST11.lib
SI:73 Formula:C16H26O CAS:56797-43-4 MolWeight:234 RetIndex:1824
CompName:cis,cis,cis-7,10,13-Hexadecatrienal $$ (7Z,10Z,13Z)-7,10,13-Hexadecatrienal # $$

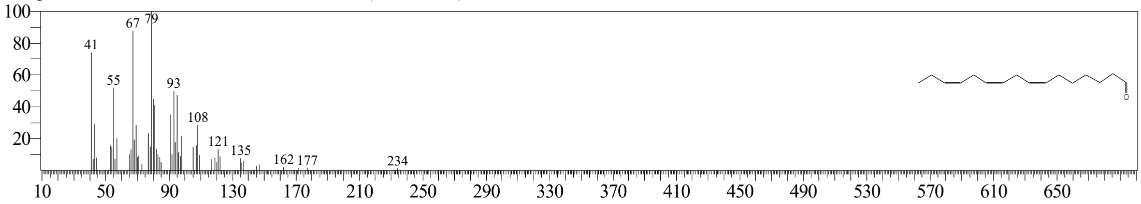

<< Target >>
Line#:91 R.Time:18.475(Scan#:2896) MassPeaks:358
RawMode:Averaged 18.470-18.480(2895-2897) BasePeak:108.05(78711)
BG Mode:Calc. from Peak Group 1 - Event 1 Scan

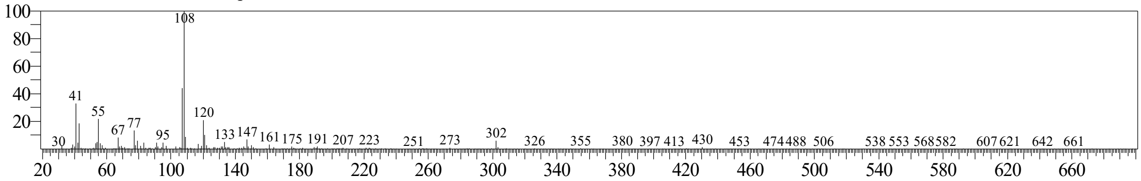

Hit#:1 Entry:29267 Library:NIST11.lib
SI:73 Formula:C12H16O CAS:156089-29-1 MolWeight:176 RetIndex:1388
CompName:Benzene, 1-methyl-4-(2-pentenyloxy)- $$ 1-Methyl-4-[(2E)-2-pentenyloxy]benzene # $$

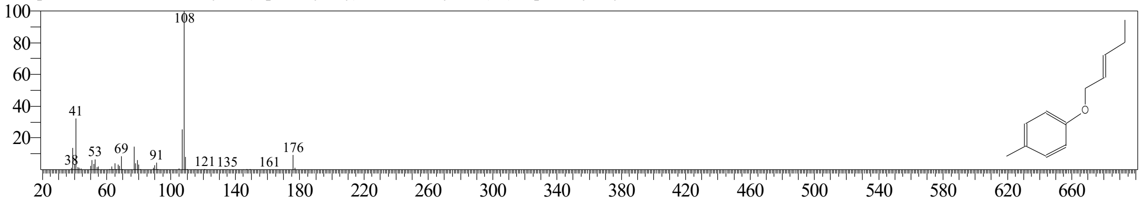

Hit#:2 Entry:101114 Library:NIST11.lib
SI:72 Formula:C16H22O4 CAS:0-00-0 MolWeight:278 RetIndex:2037
CompName:Oxalic acid, heptyl 2-methylphenyl ester

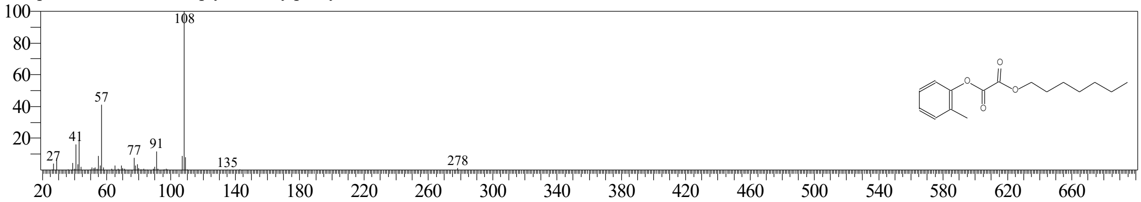

Hit#:3 Entry:154416 Library:NIST11.lib
SI:72 Formula:C21H32O4 CAS:0-00-0 MolWeight:348 RetIndex:2534
CompName:Oxalic acid, dodecyl 2-methylphenyl ester

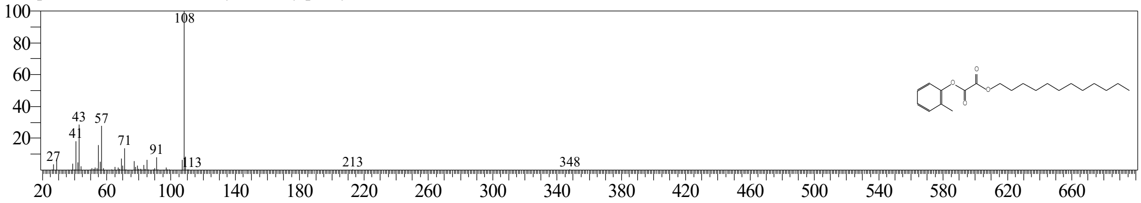

Hit#:4 Entry:46672 Library:NIST11.lib
SI:72 Formula:C15H24 CAS:0-00-0 MolWeight:204 RetIndex:1450
CompName:rotundene

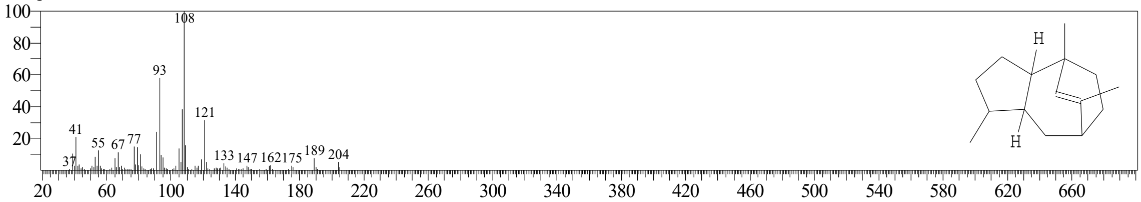

Hit#:5 Entry:144667 Library:NIST11.lib
SI:72 Formula:C20H30O4 CAS:0-00-0 MolWeight:334 RetIndex:2434
CompName:Oxalic acid, 2-methylphenyl undecyl ester

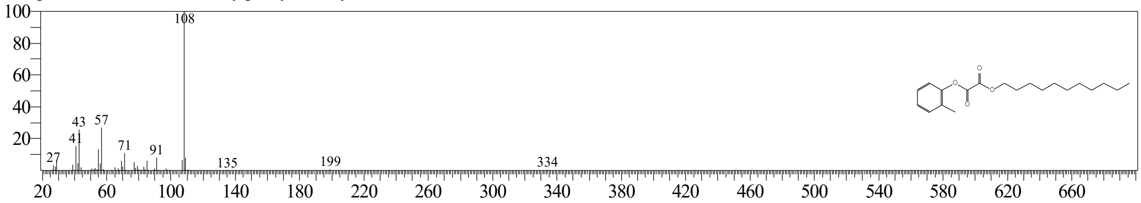

Qualitative Analysis Report

<< Target >>
Line#:92 R.Time:18.570(Scan#:2915) MassPeaks:369
RawMode:Averaged 18.565-18.575(2914-2916) BasePeak:108.05(214897)
BG Mode:Calc. from Peak Group 1 - Event 1 Scan

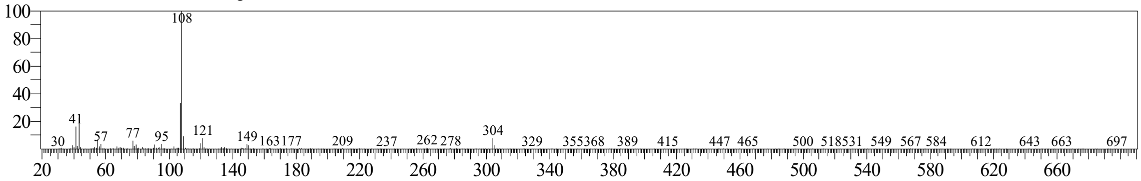

Hit#:1 Entry:121943 Library:NIST11.lib
SI:83 Formula:C21H36O CAS:501-24-6 MolWeight:304 RetIndex:2406
CompName:Phenol, 3-pentadecyl- $$ Phenol, m-pentadecyl- $$ m-Pentadecylphenol $$ Anacardol, tetrahydro- $$ Cyclogallipharaol $$ Hydrocardanol $$ Hydrc

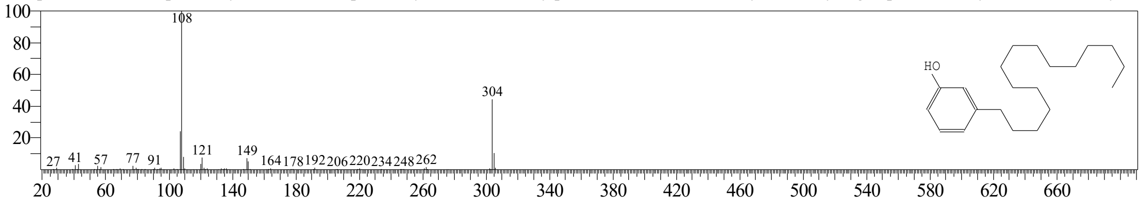

Hit#:2 Entry:30439 Library:NIST11.lib
SI:82 Formula:C12H18O CAS:85709-98-4 MolWeight:178 RetIndex:1296
CompName:Benzene, 1-(1,1-dimethylpropoxy)-4-methyl- $$ Toluene, 4-(1,1-dimethylpropoxy)- $$ 1-Methyl-4-(tert-pentyloxy)benzene # $$

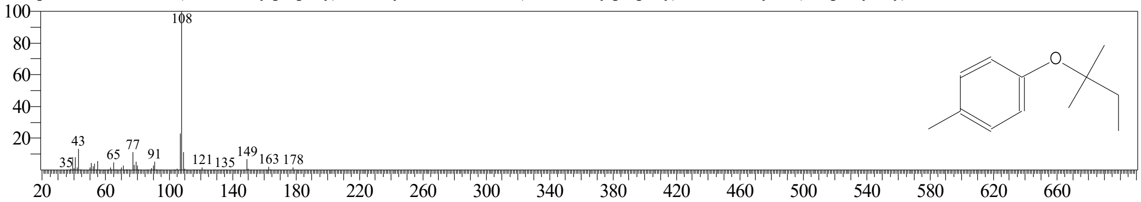

Hit#:3 Entry:79486 Library:NIST11.lib
SI:82 Formula:C14H18O4 CAS:0-00-0 MolWeight:250 RetIndex:1838
CompName:Oxalic acid, 2-methylphenyl pentyl ester

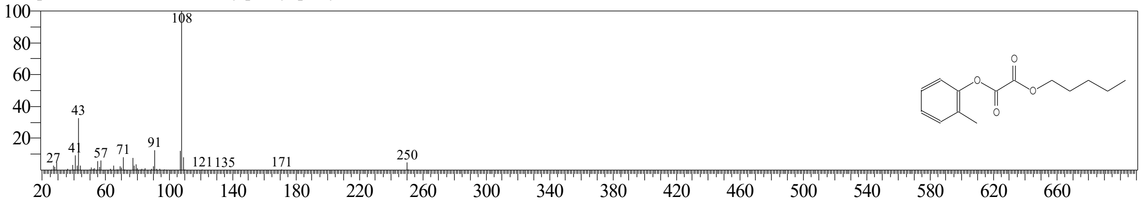

Hit#:4 Entry:59043 Library:NIST11.lib
SI:82 Formula:C13H18O3 CAS:0-00-0 MolWeight:222 RetIndex:1563
CompName:Carbonic acid, neopentyl 3-methylphenyl ester

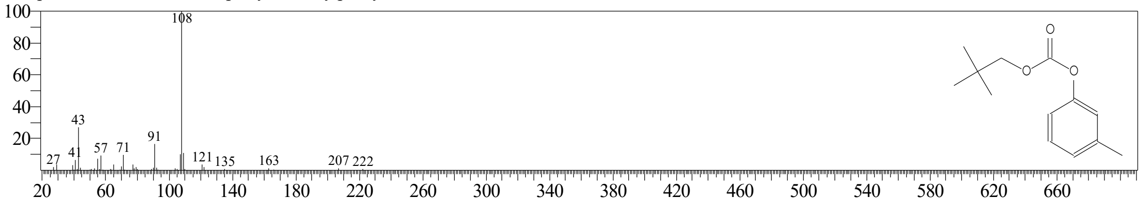

Hit#:5 Entry:112148 Library:NIST11.lib
SI:81 Formula:C17H24O4 CAS:0-00-0 MolWeight:292 RetIndex:2136
CompName:Oxalic acid, 2-methylphenyl octyl ester

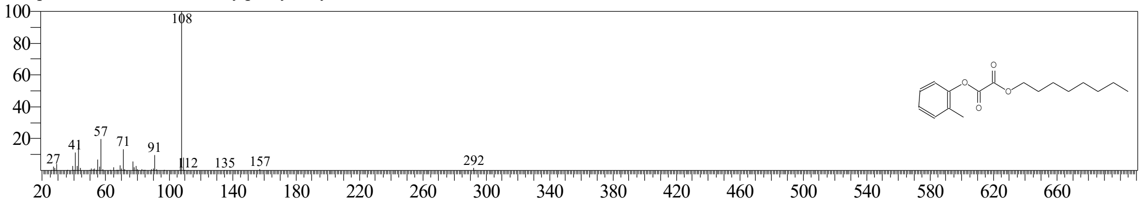

<< Target >>
Line#:93 R.Time:18.740(Scan#:2949) MassPeaks:366
RawMode:Averaged 18.735-18.745(2948-2950) BasePeak:123.10(9395)
BG Mode:Calc. from Peak Group 1 - Event 1 Scan

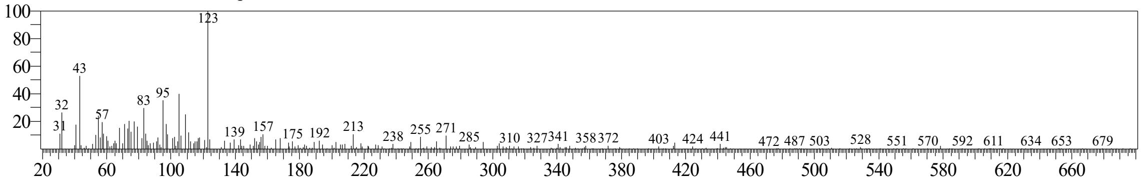

Hit#:1 Entry:132854 Library:NIST11.lib
SI:61 Formula:C21H34O2 CAS:0-00-0 MolWeight:318 RetIndex:2353
CompName:Benzoic acid, tetradecyl ester

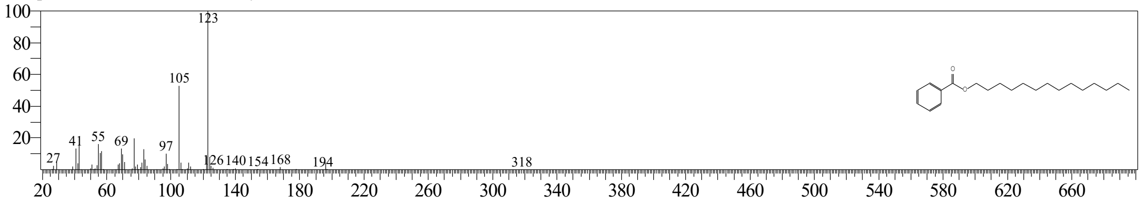

Hit#:2 Entry:143354 Library:NIST11.lib
SI:61 Formula:C22H36O2 CAS:0-00-0 MolWeight:332 RetIndex:2452
CompName:Benzoic acid, pentadecyl ester

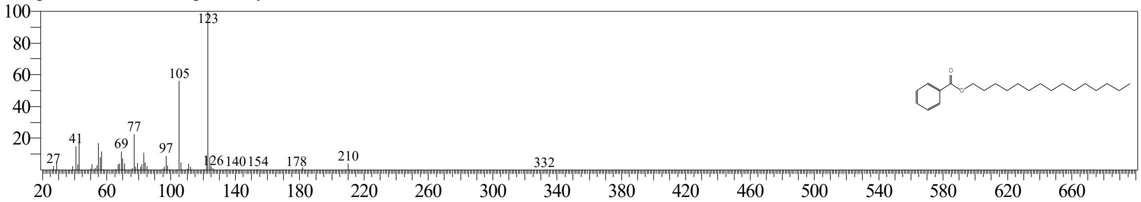

Hit#:3 Entry:170440 Library:NIST11.lib
SI:60 Formula:C25H42O2 CAS:0-00-0 MolWeight:374 RetIndex:2750
CompName:Benzoic acid, octadecyl ester

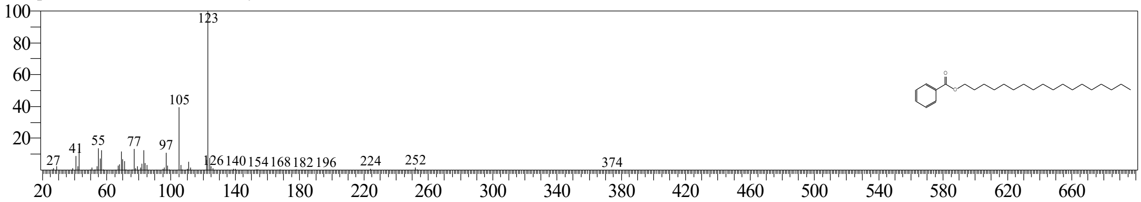

Hit#:4 Entry:31716 Library:NIST11.lib
SI:60 Formula:C12H20O CAS:211563-96-1 MolWeight:180 RetIndex:1322
CompName:Cyclohexene, 1,5,5-trimethyl-6-acetylmethyl- $$ 1-(2,6,6-Trimethyl-2-cyclohexen-1-yl)acetone # $$

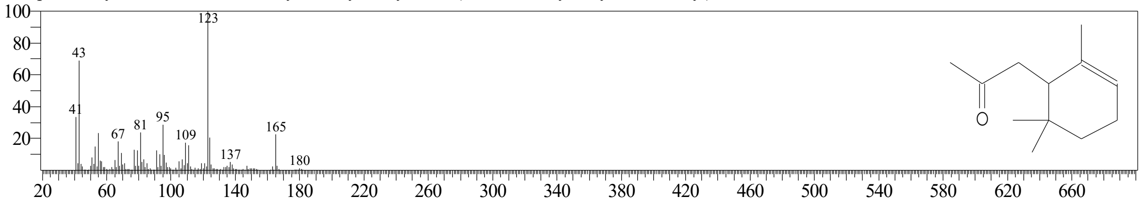

Hit#:5 Entry:153268 Library:NIST11.lib
SI:60 Formula:C23H38O2 CAS:0-00-0 MolWeight:346 RetIndex:2551
CompName:Benzoic acid, hexadecyl ester

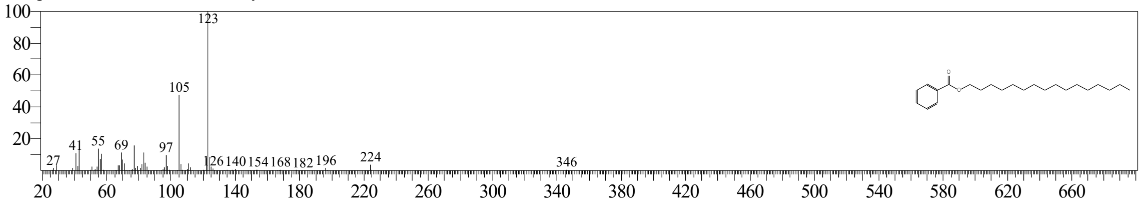

<< Target >>
Line#:94 R.Time:19.700(Scan#:3141) MassPeaks:439
RawMode:Averaged 19.695-19.705(3140-3142) BasePeak:88.10(79694)
BG Mode:Calc. from Peak Group 1 - Event 1 Scan

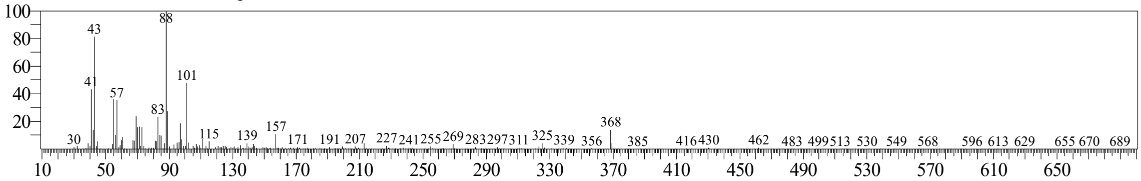

Hit#:1 Entry:166830 Library:NIST11.lib
SI:89 Formula:C24H48O2 CAS:5908-87-2 MolWeight:368 RetIndex:2574
CompName:Docosanoic acid, ethyl ester $$ Ethyl docosanoate # $$

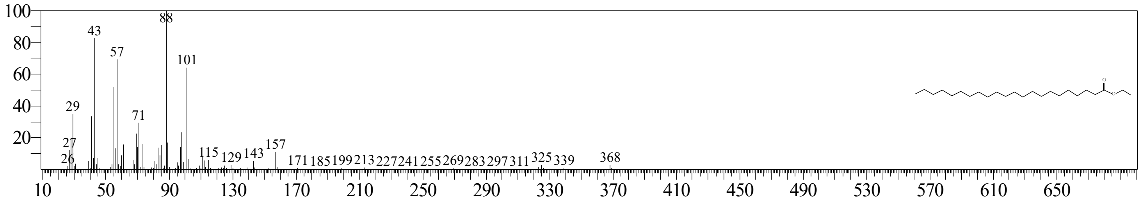

Hit#:2 Entry:149096 Library:NIST11.lib
SI:85 Formula:C22H44O2 CAS:18281-05-5 MolWeight:340 RetIndex:2375
CompName:Eicosanoic acid, ethyl ester $$ Ethyl icosanoate $$ Ethyl arachidate $$ Ethyl eicosanoate $$ Arachidic acid, ethyl ester $$

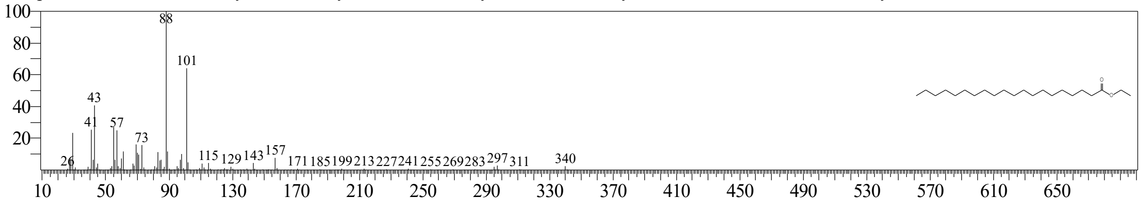

Hit#:3 Entry:138751 Library:NIST11.lib
SI:84 Formula:C21H42O2 CAS:18281-04-4 MolWeight:326 RetIndex:2276
CompName:Nonadecanoic acid, ethyl ester $$ Ethyl nonadecanoate # $$

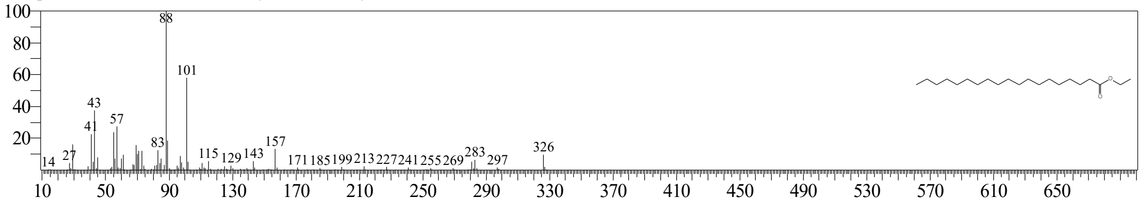

Hit#:4 Entry:73860 Library:NIST11.lib
SI:83 Formula:C15H30O2 CAS:28267-29-0 MolWeight:242 RetIndex:1680
CompName:Ethyl tridecanoate $$ Tridecanoic acid ethyl ester $$ n-Tridecanoic acid ethyl ester $$

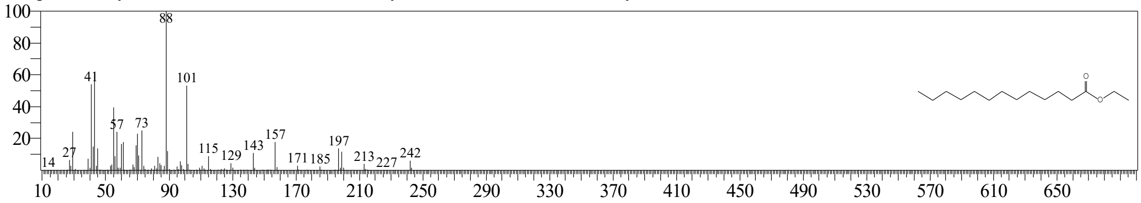

Hit#:5 Entry:117163 Library:NIST11.lib
SI:83 Formula:C19H38O2 CAS:14010-23-2 MolWeight:298 RetIndex:2077
CompName:Heptadecanoic acid, ethyl ester $$ Ethyl heptadecanoate $$ Ethyl n-heptadecanoate $$

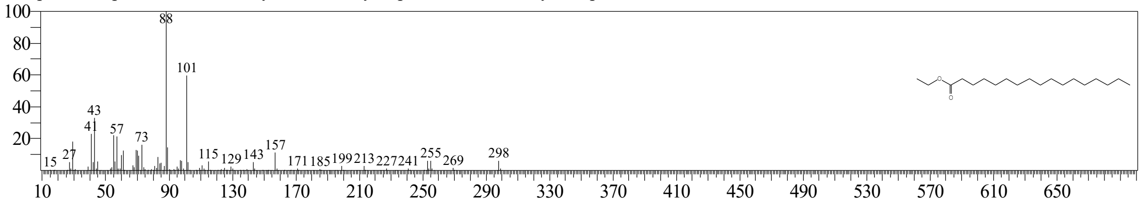

<< Target >>
Line#:95 R.Time:21.535(Scan#:3508) MassPeaks:383
RawMode:Averaged 21.530-21.540(3507-3509) BasePeak:43.05(10579)
BG Mode:Calc. from Peak Group 1 - Event 1 Scan

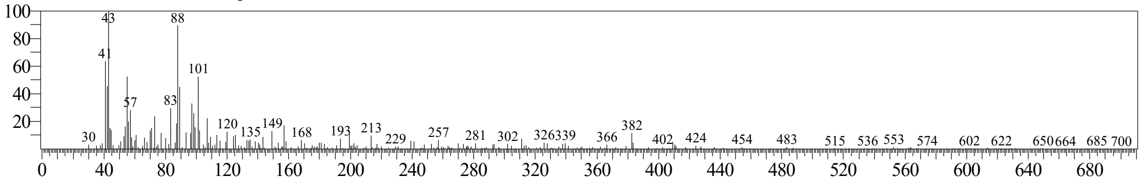

Hit#:1 Entry:166830 Library:NIST11.lib
SI:73 Formula:C24H48O2 CAS:5908-87-2 MolWeight:368 RetIndex:2574
CompName:Docosanoic acid, ethyl ester $$ Ethyl docosanoate # $$

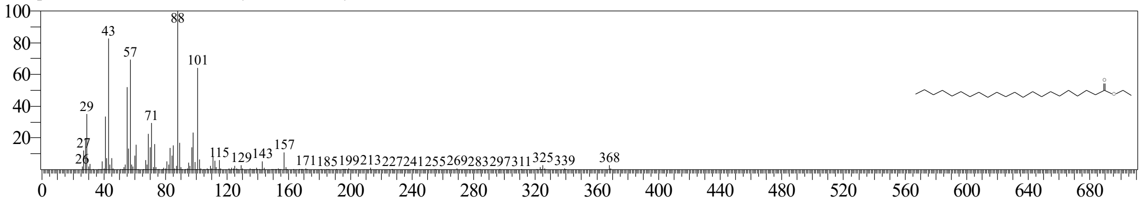

Hit#:2 Entry:73860 Library:NIST11.lib
SI:72 Formula:C15H30O2 CAS:28267-29-0 MolWeight:242 RetIndex:1680
CompName:Ethyl tridecanoate $$ Tridecanoic acid ethyl ester $$ n-Tridecanoic acid ethyl ester $$

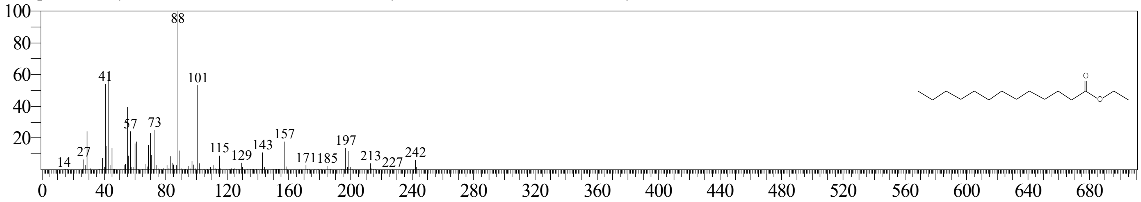

Hit#:3 Entry:111781 Library:NIST11.lib
SI:69 Formula:C13H25BrO2 CAS:0-00-0 MolWeight:292 RetIndex:1777
CompName:Undecanoic acid, 11-bromo-ethylester $$ Ethyl 11-bromoundecanoate # $$

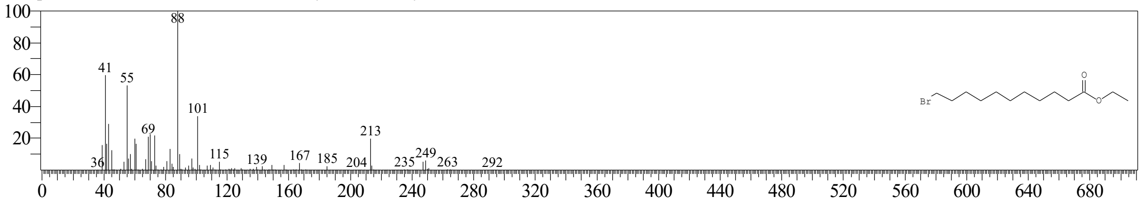

Hit#:4 Entry:13012 Library:NIST11.lib
SI:69 Formula:C8H16O2 CAS:123-66-0 MolWeight:144 RetIndex:984
CompName:Hexanoic acid, ethyl ester $$ Ethyl caproate $$ Ethyl hexanoate $$ Caproic acid ethyl ester $$ n-Caproic acid ethyl ester $$ Acetic acid, butyl-, ethy

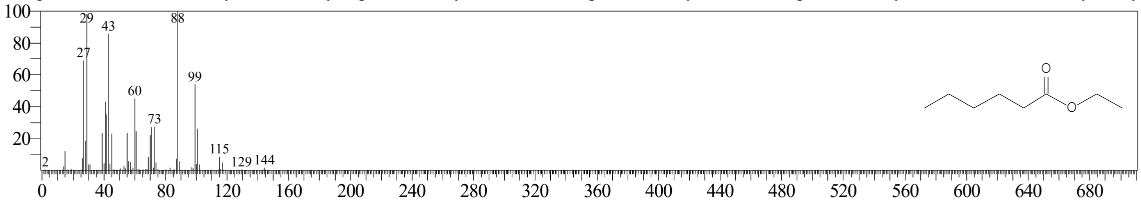

Hit#:5 Entry:128147 Library:NIST11.lib
SI:69 Formula:C20H40O2 CAS:1001-80-5 MolWeight:312 RetIndex:1920
CompName:Pentadecanoic acid, 2,6,10,14-tetramethyl-, methyl ester $$ Pentadecanoic acid, 2,6,10,14-tetramethyl-, methyl ester, (2RS,6R,10R)- $$ Methyl 2,6,

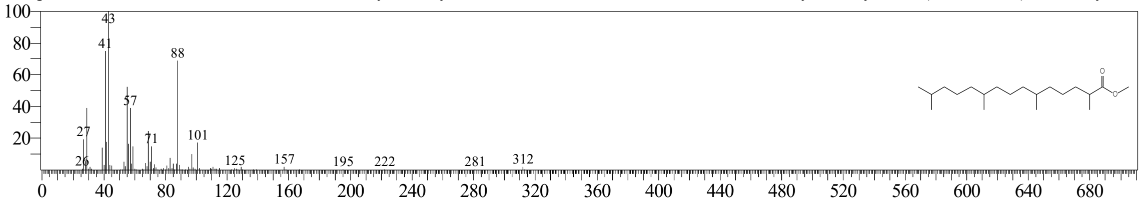

Qualitative Analysis Report

<< Target >>
Line#:96 R.Time:21.585(Scan#:3518) MassPeaks:357
RawMode:Averaged 21.580-21.590(3517-3519) BasePeak:71.05(4829)
BG Mode:Calc. from Peak Group 1 - Event 1 Scan

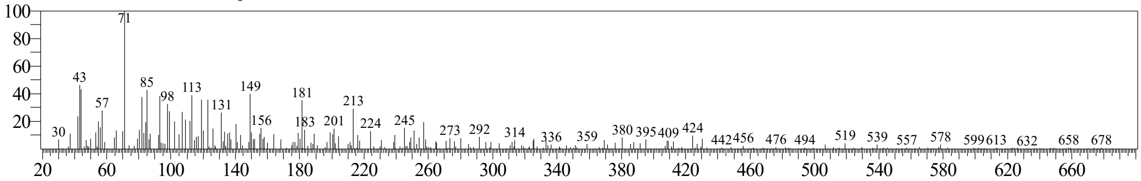

Hit#:1 Entry:17979 Library:NIST11.lib
SI:53 Formula:C9H17NO CAS:24985-58-8 MolWeight:155 RetIndex:1235
CompName:3-Octen-2-one, 4-(methylamino)- $$ (3Z)-4-(Methylamino)-3-octen-2-one # $$

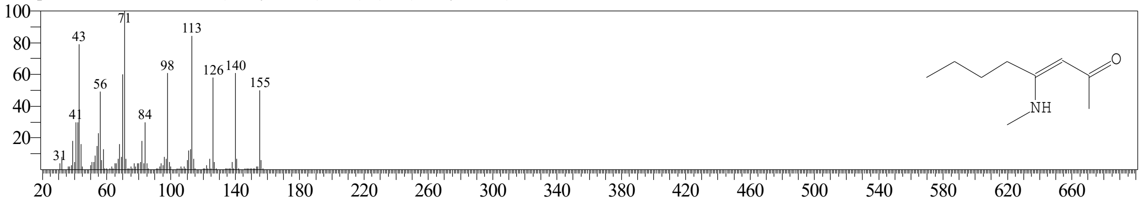

Hit#:2 Entry:133228 Library:NIST11.lib
SI:53 Formula:C17H25N3O3 CAS:0-00-0 MolWeight:319 RetIndex:2518
CompName:Cyano(5'-ethoxy-1,2,3,4,3',4'-hexahydro-2'H-[2,2']bipyrrolyl-5-ylidene)acetic acid, t-butyl ester $$ tert-Butyl (2E)-cyano[5-(5-ethoxy-3,4-dihydro-2

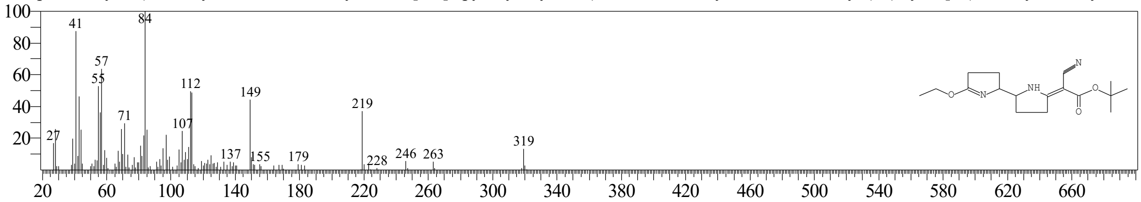

Hit#:3 Entry:6906 Library:NIST11.lib
SI:53 Formula:C8H17N CAS:22398-09-0 MolWeight:127 RetIndex:1125
CompName:Piperidine, 4-propyl- $$ 4-Propylpiperidine $$ 4-n-Propylpiperidine $$

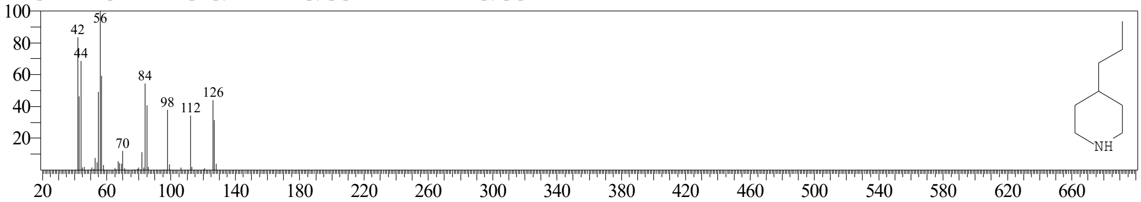

Hit#:4 Entry:72487 Library:NIST11.lib
SI:52 Formula:C17H36 CAS:14905-56-7 MolWeight:240 RetIndex:1519
CompName:Tetradecane, 2,6,10-trimethyl- $$ 2,6,10-Trimethyltetradecane $$

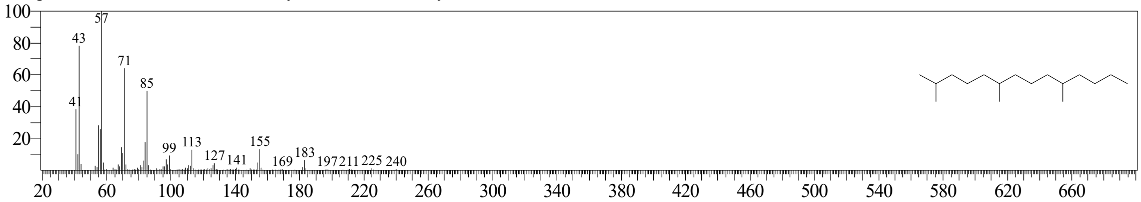

Hit#:5 Entry:33349 Library:NIST11.lib
SI:52 Formula:C12H25N CAS:46237-34-7 MolWeight:183 RetIndex:1367
CompName:1-(1-Azacyclopropyl)decane $$ 1-Decylaziridine # $$

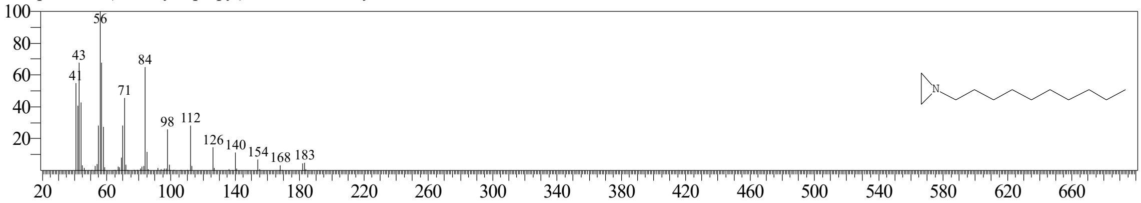

<< Target >>
Line#:97 R.Time:21.685(Scan#:3538) MassPeaks:392
RawMode:Averaged 21.680-21.690(3537-3539) BasePeak:57.10(6729)
BG Mode:Calc. from Peak Group 1 - Event 1 Scan

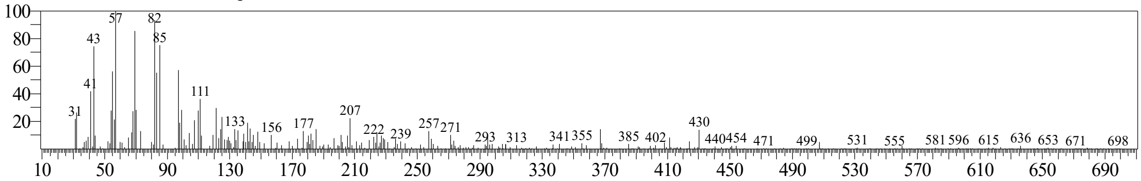

Hit#:1 Entry:107819 Library:NIST11.lib
SI:71 Formula:C18H38O2 CAS:20294-76-2 MolWeight:286 RetIndex:2215
CompName:1,2-Octadecanediol $$ 1,2-Dihydroxyoctadecane $$

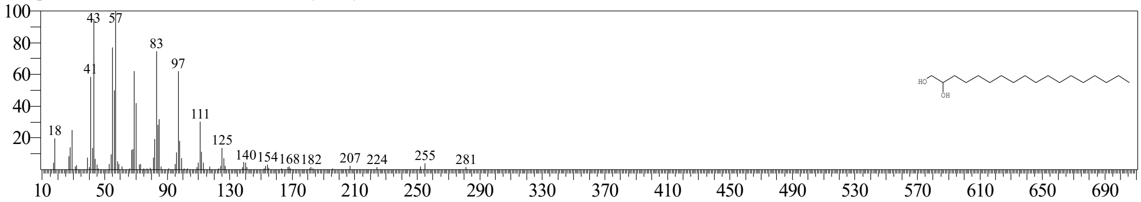

Hit#:2 Entry:158461 Library:NIST11.lib
SI:70 Formula:C24H50O CAS:506-51-4 MolWeight:354 RetIndex:2650
CompName:n-Tetracosanol-1 $$ Lignoceric alcohol $$ Lignoceryl alcohol $$ 1-Tetracosanol $$ Tetracosan-1-ol $$ Tetracosyl alcohol $$ Lignocerol $$ Tetracos

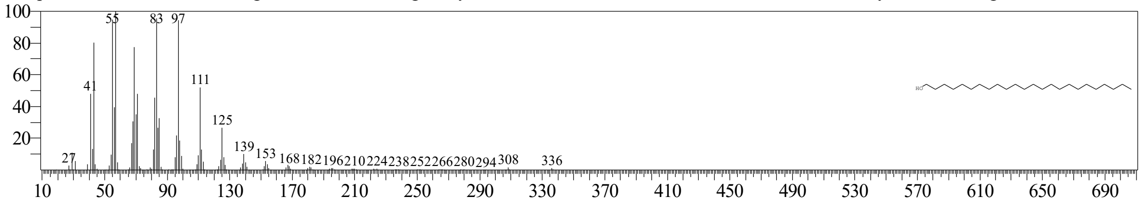

Hit#:3 Entry:180868 Library:NIST11.lib
SI:70 Formula:C27H56O CAS:2004-39-9 MolWeight:396 RetIndex:2948
CompName:1-Heptacosanol $$ Heptacosan-1-ol $$ Heptacosanol $$

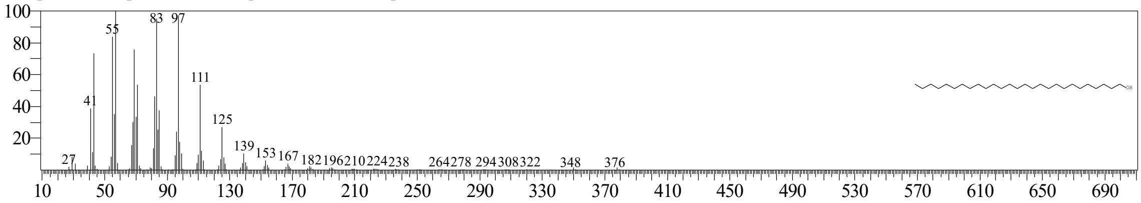

Hit#:4 Entry:187874 Library:NIST11.lib
SI:70 Formula:C21H37F5O2 CAS:959261-25-7 MolWeight:416 RetIndex:1971
CompName:Pentafluoropropionic acid, octadecyl ester

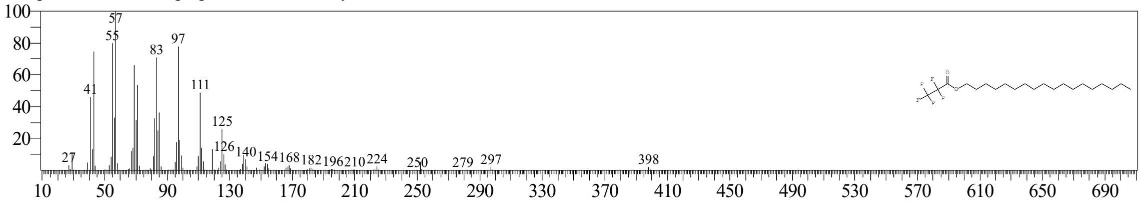

Hit#:5 Entry:84449 Library:NIST11.lib
SI:70 Formula:C17H36O CAS:1454-85-9 MolWeight:256 RetIndex:1954
CompName:n-Heptadecanol-1 $$ 1-Hydroxyheptadecane $$ 1-Heptadecanol $$ Heptadecan-1-ol $$ Heptadecyl alcohol $$

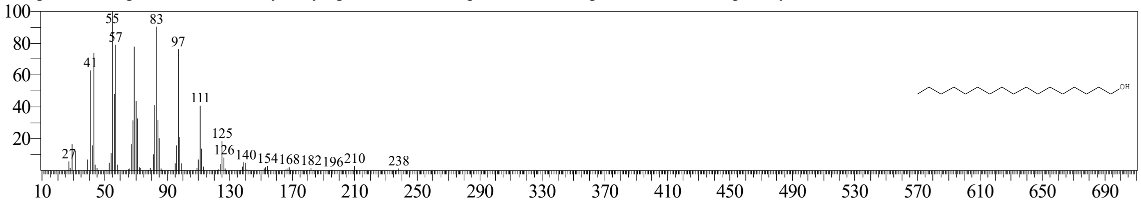

<< Target >>
Line#:98 R.Time:21.910(Scan#:3583) MassPeaks:355
RawMode:Averaged 21.905-21.915(3582-3584) BasePeak:108.05(28347)
BG Mode:Calc. from Peak Group 1 - Event 1 Scan

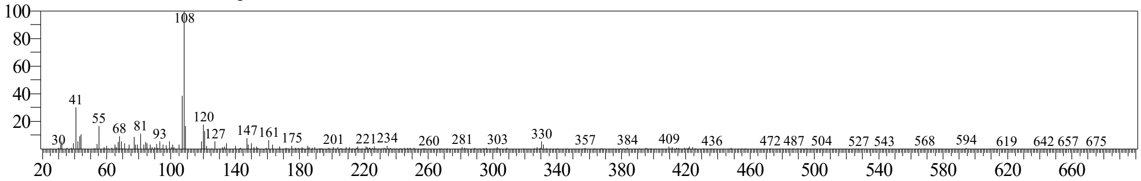

Hit#:1 Entry:46672 Library:NIST11.lib
SI:71 Formula:C15H24 CAS:0-00-0 MolWeight:204 RetIndex:1450
CompName:rotundene

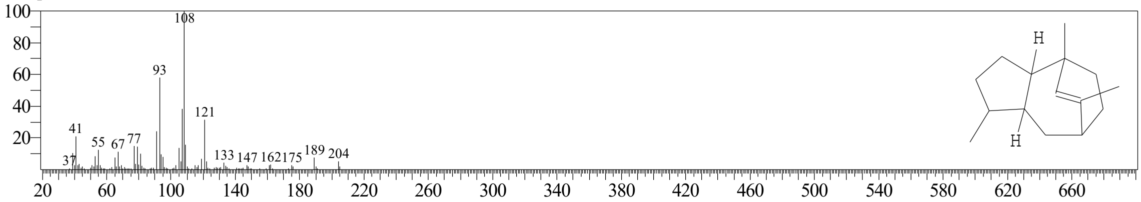

Hit#:2 Entry:29267 Library:NIST11.lib
SI:68 Formula:C12H16O CAS:156089-29-1 MolWeight:176 RetIndex:1388
CompName:Benzene, 1-methyl-4-(2-pentenyloxy)- $$ 1-Methyl-4-[(2E)-2-pentenyloxy]benzene # $$

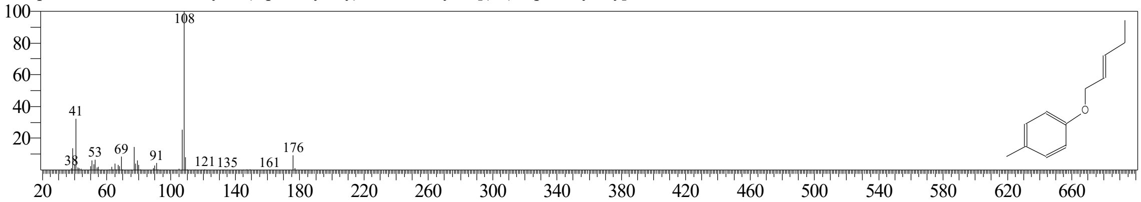

Hit#:3 Entry:88657 Library:NIST11.lib
SI:68 Formula:C14H18N2O3 CAS:0-00-0 MolWeight:262 RetIndex:2148
CompName:5-[2-(4,5,5-Trimethyl-cyclopent-1-enyl)ethylidene]pyrimidine-2,4,6(1H,3H,5H)-trione $$ 5-[2-(4,5,5-Trimethyl-1-cyclopenten-1-yl)ethylidene]-2,4,

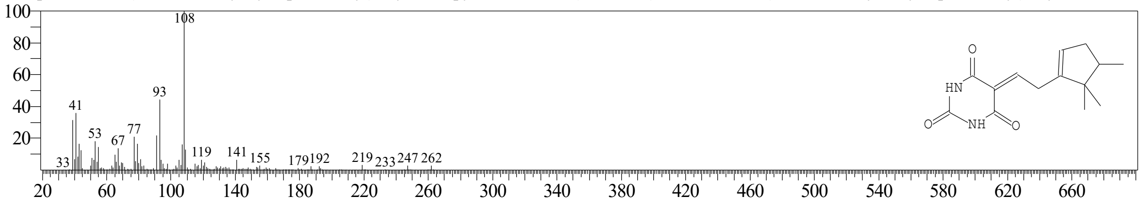

Hit#:4 Entry:16472 Library:NIST11.lib
SI:68 Formula:C10H16O CAS:4501-58-0 MolWeight:152 RetIndex:1155
CompName:.alpha.-Campholenal $$ (R)-.alpha.-Campholene aldehyde $$ 3-Cyclopentene-1-acetaldehyde, 2,2,3-trimethyl- $$ .alpha.-Compholene aldehyde $$

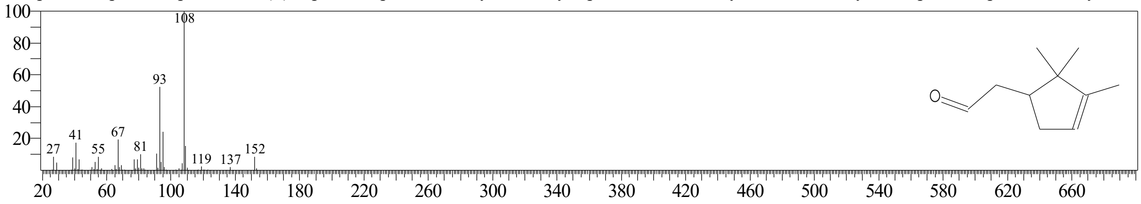

Hit#:5 Entry:30439 Library:NIST11.lib
SI:67 Formula:C12H18O CAS:85709-98-4 MolWeight:178 RetIndex:1296
CompName:Benzene, 1-(1,1-dimethylpropoxy)-4-methyl- $$ Toluene, 4-(1,1-dimethylpropoxy)- $$ 1-Methyl-4-(tert-pentyloxy)benzene # $$

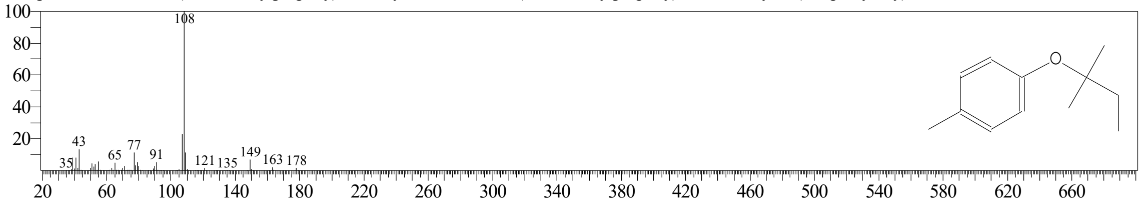

<< Target >>
Line#:99 R.Time:22.060(Scan#:3613) MassPeaks:386
RawMode:Averaged 22.055-22.065(3612-3614) BasePeak:108.15(28400)
BG Mode:Calc. from Peak Group 1 - Event 1 Scan

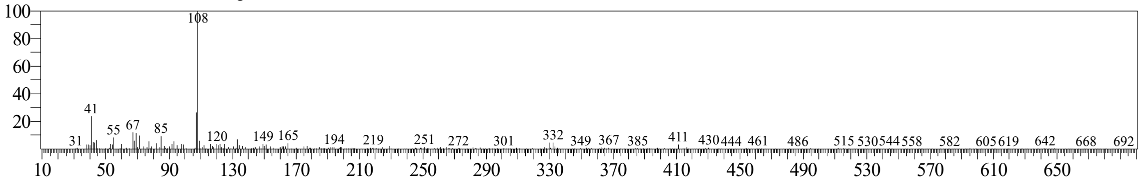

Hit#:1 Entry:29267 Library:NIST11.lib
SI:71 Formula:C12H16O CAS:156089-29-1 MolWeight:176 RetIndex:1388
CompName:Benzene, 1-methyl-4-(2-pentenyloxy)- $$ 1-Methyl-4-[(2E)-2-pentenyloxy]benzene # $$

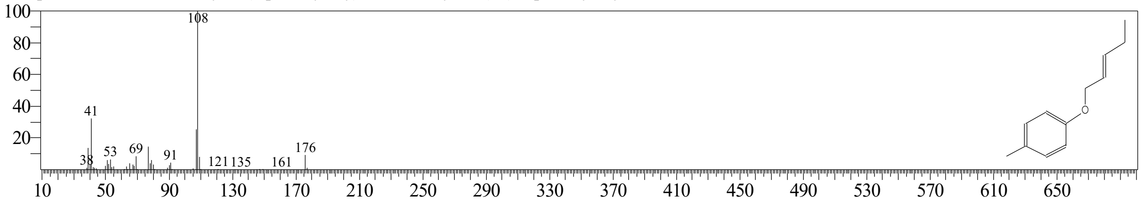

Hit#:2 Entry:15297 Library:NIST11.lib
SI:70 Formula:C9H14N2 CAS:13925-06-9 MolWeight:150 RetIndex:1129
CompName:2-Isobutyl-3-methylpyrazine $$ Pyrazine, 2-methyl-3-(2-methylpropyl)- $$ 2-Methyl-3-(2-methylpropyl)pyrazine $$ 3-Methyl-2-isobutyl pyrazine $

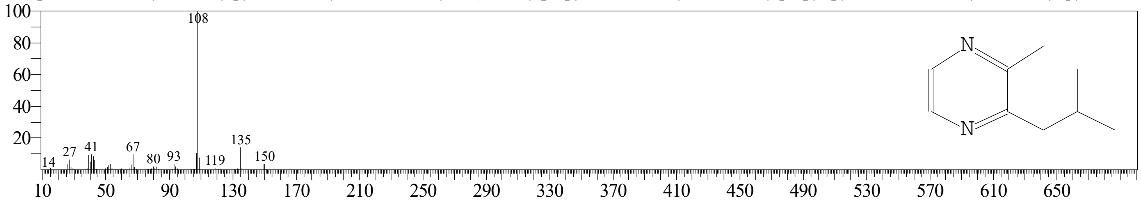

Hit#:3 Entry:15296 Library:NIST11.lib
SI:70 Formula:C9H14N2 CAS:15987-00-5 MolWeight:150 RetIndex:1193
CompName:2-Butyl-3-methylpyrazine $$ 2-n-Butyl-3-methylpyrazine $$ Pyrazine, 2-butyl-3-methyl- $$ 2-Methyl-3- butylpyrazine $$

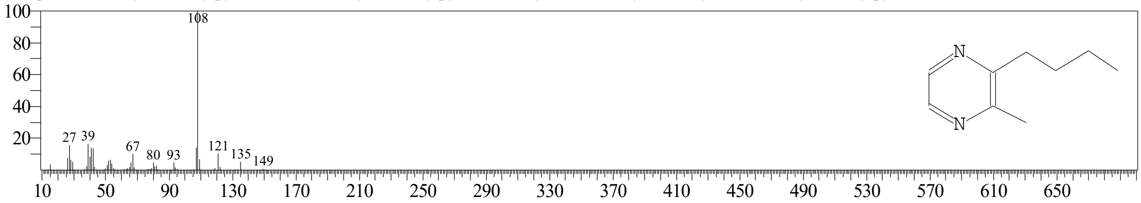

Hit#:4 Entry:30439 Library:NIST11.lib
SI:69 Formula:C12H18O CAS:85709-98-4 MolWeight:178 RetIndex:1296
CompName:Benzene, 1-(1,1-dimethylpropoxy)-4-methyl- $$ Toluene, 4-(1,1-dimethylpropoxy)- $$ 1-Methyl-4-(tert-pentyloxy)benzene # $$

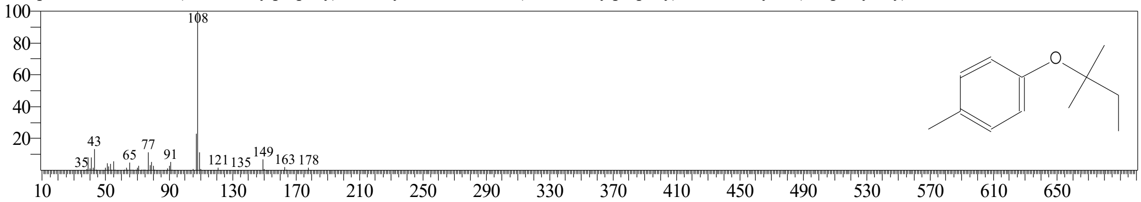

Hit#:5 Entry:123190 Library:NIST11.lib
SI:69 Formula:C18H26O4 CAS:0-00-0 MolWeight:306 RetIndex:2235
CompName:Oxalic acid, 2-methylphenyl nonyl ester

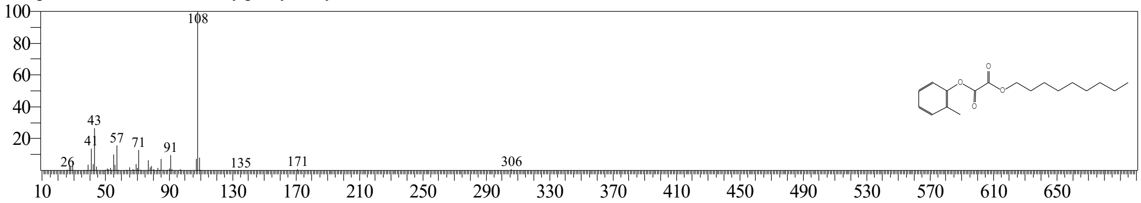

<< Target >>
Line#:100 R.Time:23.690(Scan#:3939) MassPeaks:349
RawMode:Averaged 23.685-23.695(3938-3940) BasePeak:43.10(4698)
BG Mode:Calc. from Peak Group 1 - Event 1 Scan

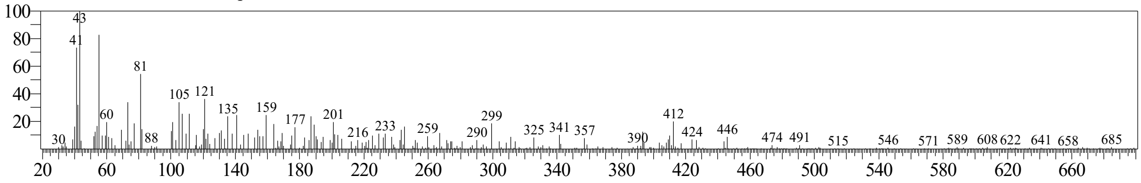

Hit#:1 Entry:57730 Library:NIST11.lib
SI:56 Formula:C15H24O CAS:0-00-0 MolWeight:220 RetIndex:1293
CompName:Ledene oxide-(II) $$ 1,4a,7,7-Tetramethyldecahydrocyclopropa[7,8]azuleno[3a,4-b]oxirene # $$

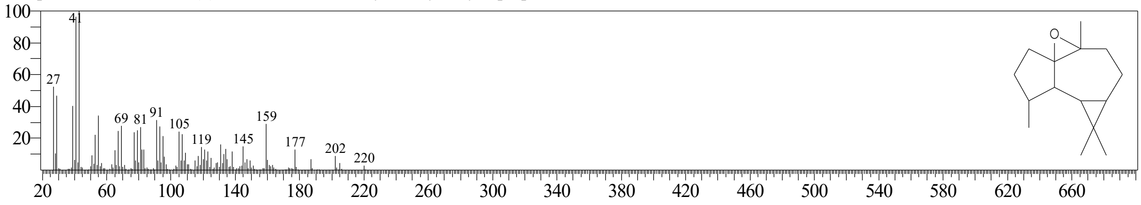

Hit#:2 Entry:57731 Library:NIST11.lib
SI:55 Formula:C15H24O CAS:0-00-0 MolWeight:220 RetIndex:1531
CompName:trans-Z-.alpha.-Bisabolene epoxide $$ 4-[(1Z)-1,5-Dimethyl-1,4-hexadienyl]-1-methyl-7-oxabicyclo[4.1.0]heptane # $$

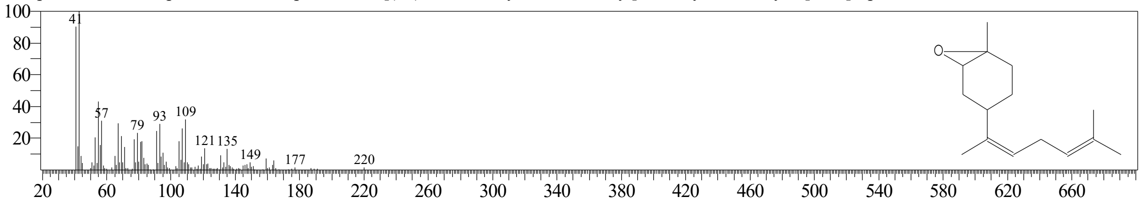

Hit#:3 Entry:57727 Library:NIST11.lib
SI:55 Formula:C15H24O CAS:0-00-0 MolWeight:220 RetIndex:1293
CompName:Aristolene epoxide $$ 1,7,7,7b-Tetramethyldecahydrocyclopropa[3,4]naphtho[1,8a-b]oxirene # $$

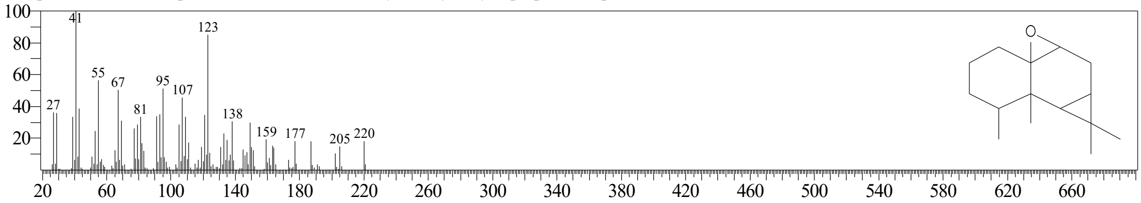

Hit#:4 Entry:57713 Library:NIST11.lib
SI:54 Formula:C15H24O CAS:0-00-0 MolWeight:220 RetIndex:1293
CompName:Ledene oxide-(I) $$ 1,4a,7,7-Tetramethyldecahydrocyclopropa[7,8]azuleno[3a,4-b]oxirene # $$

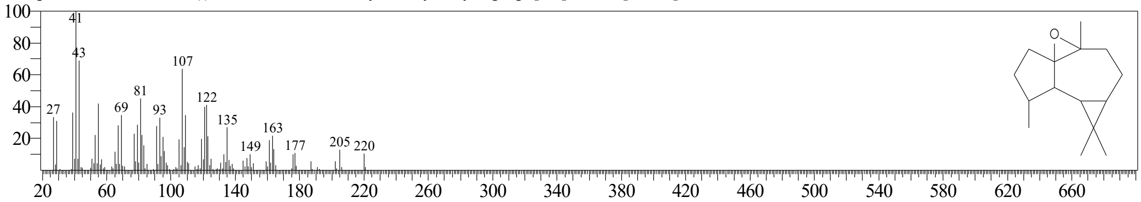

Hit#:5 Entry:144898 Library:NIST11.lib
SI:54 Formula:C21H34O3 CAS:0-00-0 MolWeight:334 RetIndex:2201
CompName:Dihydro-isosteviol methyl ester $$ Methyl 12-hydroxyatisan-18-oate # $$

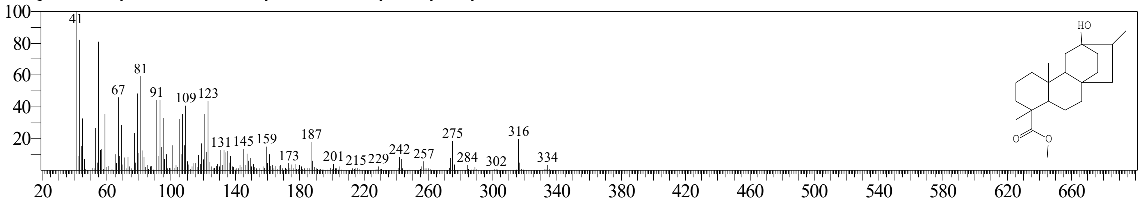

Qualitative Analysis Report

<< Target >>
Line#:101   R.Time:23.960(Scan#:3993) MassPeaks:340
RawMode:Averaged 23.955-23.965(3992-3994) BasePeak:79.10(6125)
BG Mode:Calc. from Peak Group 1 - Event 1 Scan

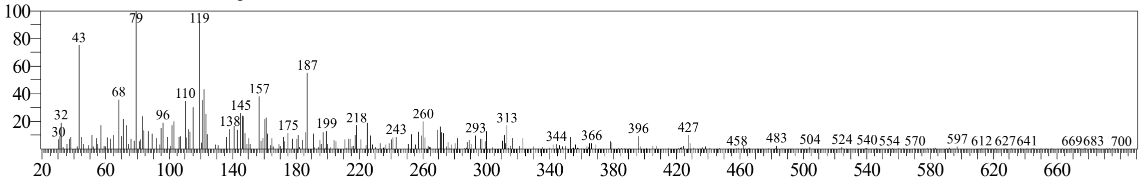

Hit#:1 Entry:69283 Library:NIST11.lib
SI:43 Formula:C15H24O2 CAS:0-00-0 MolWeight:236 RetIndex:1729
CompName:Murolan-3,9(11)-diene-10-peroxy $$ 8-Isopropyl-5-methyl-2-methylene-1,2,3,4,4a,5,8,8a-octahydro-1-naphthalenyl hydroperoxide # $$

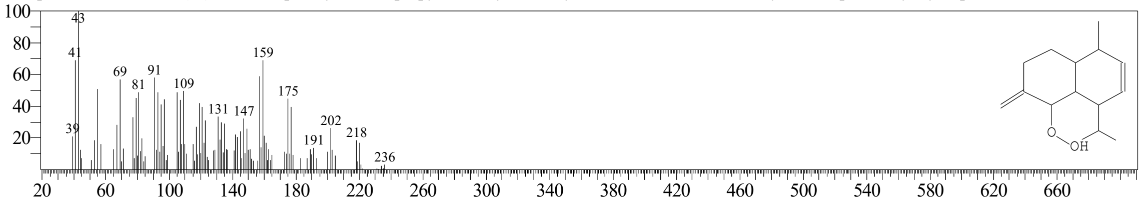

Hit#:2 Entry:129810 Library:NIST11.lib
SI:42 Formula:C21H30O2 CAS:65556-93-6 MolWeight:314 RetIndex:2219
CompName:1-Allopregnene-3,20-dione $$ Pregn-1-ene-3,20-dione # $$

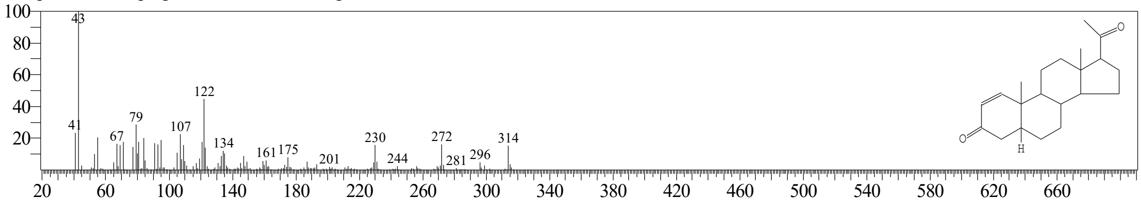

Hit#:3 Entry:201003 Library:NIST11.lib
SI:41 Formula:C31H52O3 CAS:0-00-0 MolWeight:472 RetIndex:3022
CompName:9,19-Cyclochloestene-3,7-diol, 4,14-dimethyl-, 3-acetate $$ 1-(1,5-Dimethylhexyl)-4-hydroxy-3a,6,12a-trimethyltetradecahydro-1H-cyclopenta[a]c

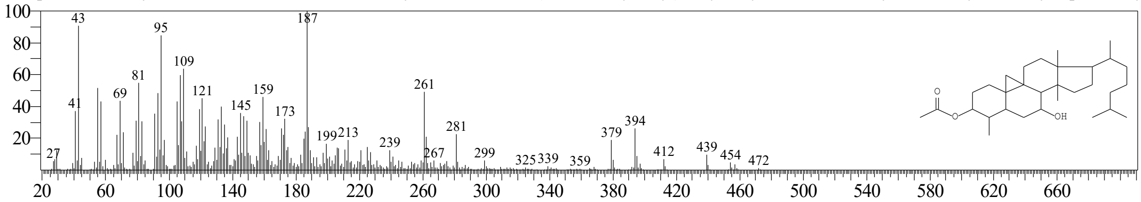

Hit#:4 Entry:211708 Library:NIST11.lib
SI:41 Formula:C35H48O13 CAS:0-00-0 MolWeight:676 RetIndex:4250
CompName:Testosterone pentacetylgluconate $$ 3-Oxoandrost-4-en-17-yl 2,3,4,5,6-penta-O-acetylhexonate # $$

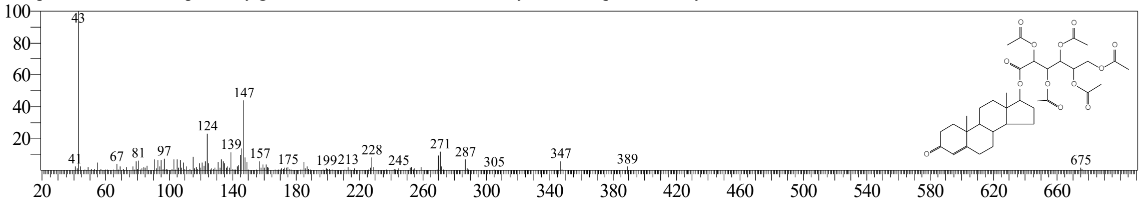

Hit#:5 Entry:118812 Library:NIST11.lib
SI:41 Formula:C19H24O3 CAS:968-93-4 MolWeight:300 RetIndex:2278
CompName:Testolactone $$ D-Homo-17a-oxaandrosta-1,4-diene-3,17-dione $$ .DELTA.1-Dehydrotestolactone $$ .DELTA.1-Dehydrotestololactone $$ .DELTA

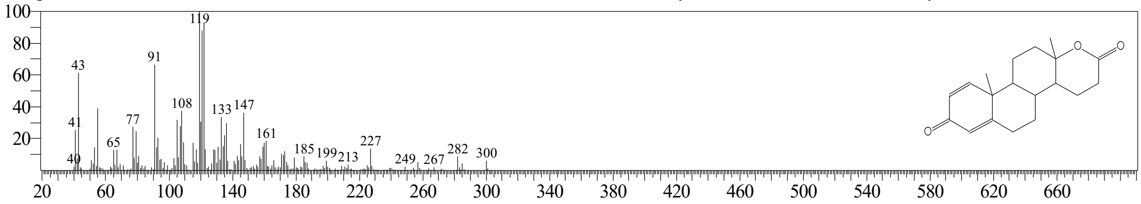

<< Target >>
Line#:102 R.Time:23.970(Scan#:3995) MassPeaks:336
RawMode:Averaged 23.965-23.975(3994-3996) BasePeak:69.10(5898)
BG Mode:Calc. from Peak Group 1 - Event 1 Scan

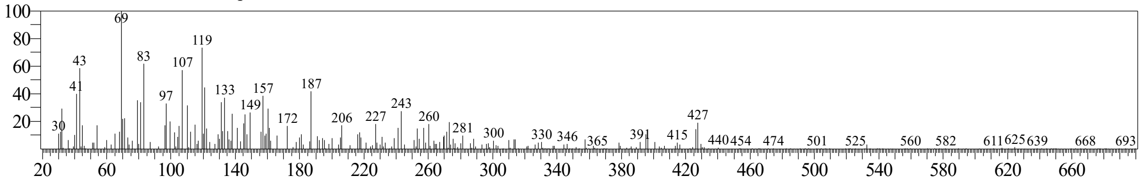

Hit#:1 Entry:191115 Library:NIST11.lib
SI:53 Formula:C30H50O CAS:53761-94-7 MolWeight:426 RetIndex:2719
CompName:Cyclopropa[5,6]-33-norgorgostan-3-ol, 3',6-dihydro-, (3.beta.,5.beta.,6.alpha.,22.xi.,23.xi.)- $$ 3-(1-[2-(1,2-Dimethylpropyl)cyclopropyl]ethyl)-3a,

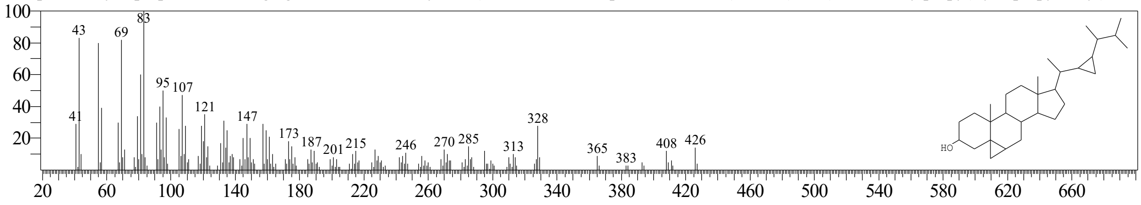

Hit#:2 Entry:191107 Library:NIST11.lib
SI:53 Formula:C30H50O CAS:79-63-0 MolWeight:426 RetIndex:2882
CompName:Lanosterol $$ Lanosta-8,24-dien-3-ol, (3.beta.)- $$ Lanosta-8,24-dien-3.beta.-ol $$ Botalan base 138 $$ Cholesta-8,24-dien-3-ol, 4,4,14-trimethyl-,

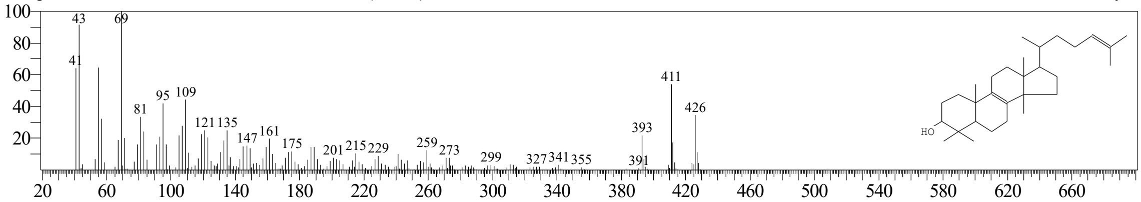

Hit#:3 Entry:69283 Library:NIST11.lib
SI:53 Formula:C15H24O2 CAS:0-00-0 MolWeight:236 RetIndex:1729
CompName:Murolan-3,9(11)-diene-10-peroxy $$ 8-Isopropyl-5-methyl-2-methylene-1,2,3,4,4a,5,8,8a-octahydro-1-naphthalenyl hydroperoxide # $$

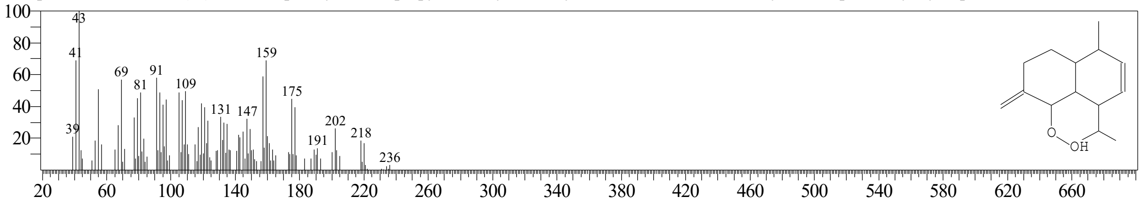

Hit#:4 Entry:182634 Library:NIST11.lib
SI:52 Formula:C27H44O2 CAS:0-00-0 MolWeight:400 RetIndex:2695
CompName:26-Dehydroxy-dihydropseudoprogenin-25-ene $$ Furost-25-en-3-ol # $$

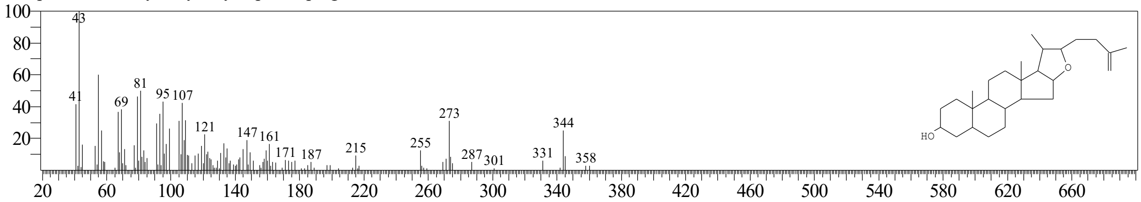

Hit#:5 Entry:135566 Library:NIST11.lib
SI:51 Formula:C19H30O4 CAS:0-00-0 MolWeight:322 RetIndex:2131
CompName:Acetate, [6-(acetyloxy)-5,5,8a-trimethyl-2-methyleneperhydro-1-naphthalenyl]methyl ester $$ [6-(Acetyloxy)-5,5,8a-trimethyl-2-methylenedecahyd

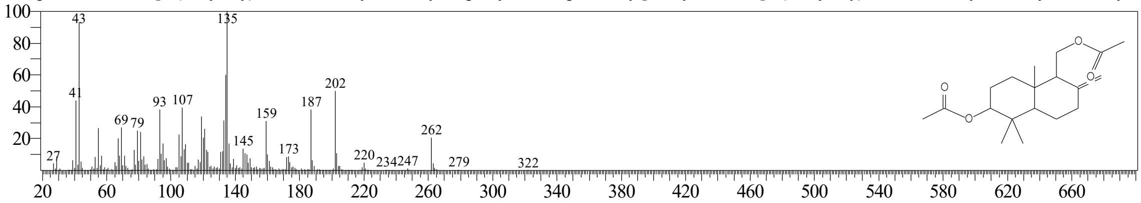

<< Target >>
Line#:103 R.Time:24.290(Scan#:4059) MassPeaks:415
RawMode:Averaged 24.285-24.295(4058-4060) BasePeak:69.05(361737)
BG Mode:Calc. from Peak Group 1 - Event 1 Scan

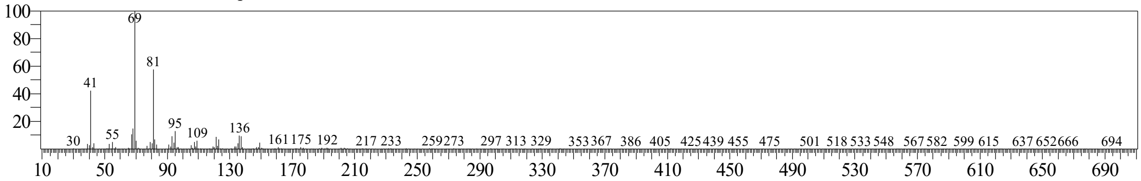

Hit#:1 Entry:186078 Library:NIST11.lib
SI:95 Formula:C30H50 CAS:111-02-4 MolWeight:410 RetIndex:2914
CompName:Squalene $$ 2,6,10,14,18,22-Tetracosahexaene, 2,6,10,15,19,23-hexamethyl-, (all-E)- $$ all-trans-Squalene $$ trans-Squalene $$ Spinacen $$ Spin

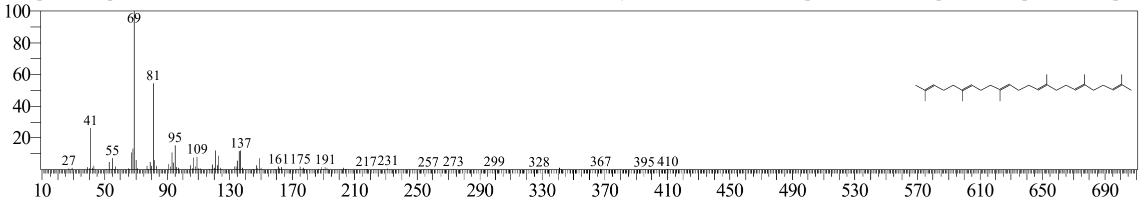

Hit#:2 Entry:110904 Library:NIST11.lib
SI:92 Formula:C20H34O CAS:7614-21-3 MolWeight:290 RetIndex:2192
CompName:Hexadeca-2,6,10,14-tetraen-1-ol, 3,7,11,16-tetramethyl- $$ 3,7,11,15-Tetramethyl-2,6,10,14-hexadecatetraen-1-ol $$ Tetraprenol $$

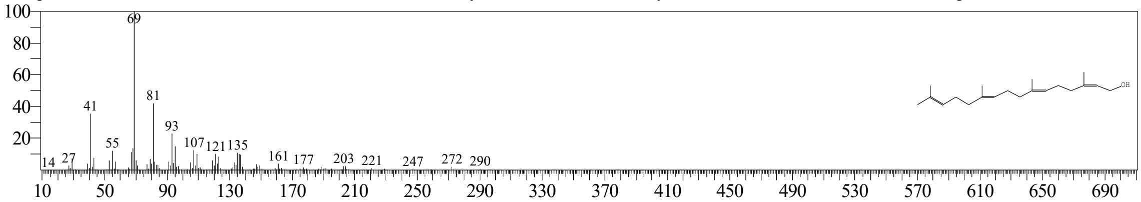

Hit#:3 Entry:59385 Library:NIST11.lib
SI:91 Formula:C15H26O CAS:0-00-0 MolWeight:222 RetIndex:1710
CompName:Farnesol isomer a $$ (2E,6E)-3,7,11-Trimethyl-2,6,10-dodecatrien-1-ol # $$

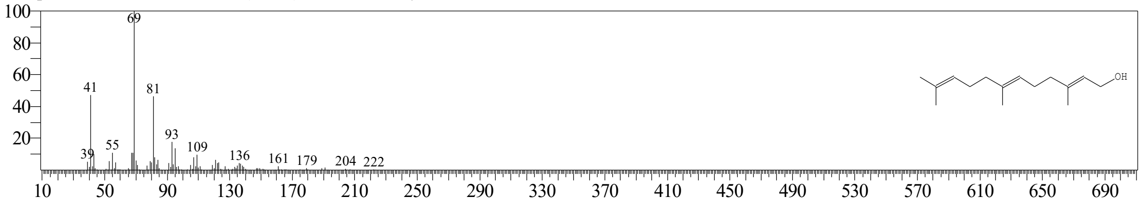

Hit#:4 Entry:150629 Library:NIST11.lib
SI:90 Formula:C25H42 CAS:75581-03-2 MolWeight:342 RetIndex:2432
CompName:2,6,10,14,18-Pentamethyl-2,6,10,14,18-eicosapentaene $$ (6E,10E,14E,18E)-2,6,10,14,18-Pentamethyl-2,6,10,14,18-icosapentaene # $$

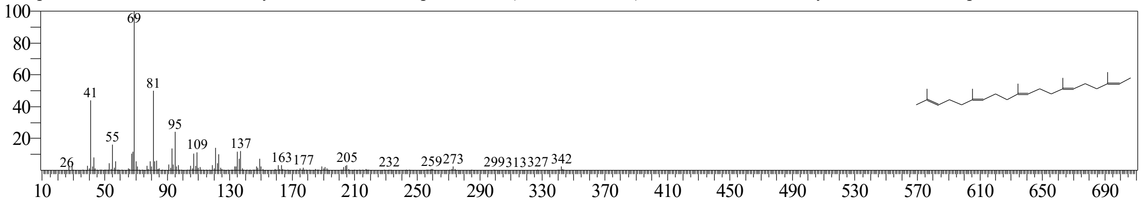

Hit#:5 Entry:175446 Library:NIST11.lib
SI:90 Formula:C27H44O CAS:0-00-0 MolWeight:384 RetIndex:2819
CompName:Docosa-2,6,10,14,18-pentaen-22-al, 2,6,10,15,18-pentamethyl-, all-trans $$ (4E,8E,12E,16E)-4,8,13,17,21-Pentamethyl-4,8,12,16,20-docosapentae

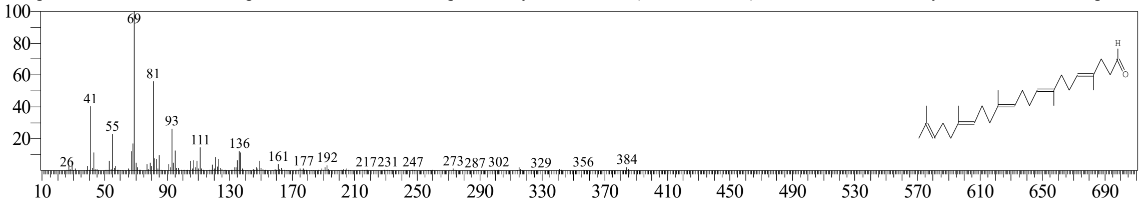

<< Target >>
Line#:104 R.Time:24.460(Scan#:4093) MassPeaks:380
RawMode:Averaged 24.455-24.465(4092-4094) BasePeak:69.05(2986)
BG Mode:Calc. from Peak Group 1 - Event 1 Scan

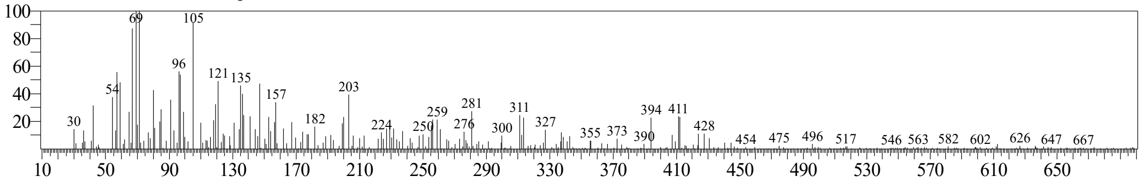

Hit#:1 Entry:182687 Library:NIST11.lib
SI:49 Formula:C28H48O CAS:22599-96-8 MolWeight:400 RetIndex:2652
CompName:Cholestan-3-ol, 2-methylene-, (3.beta.,5.alpha.)- $$ 5.alpha.-Cholestan-3.beta.-ol, 2-methylene- $$ 2-Methylenecholestan-3-ol # $$

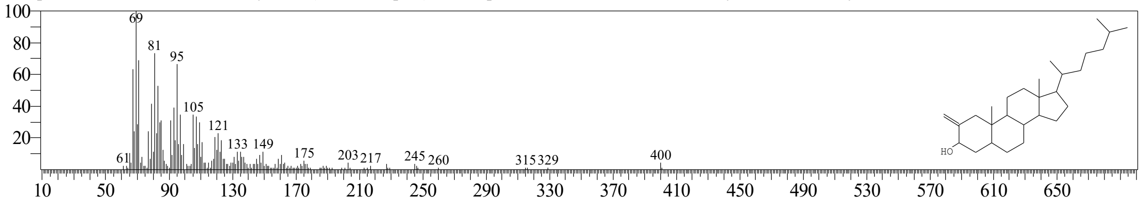

Hit#:2 Entry:159768 Library:NIST11.lib
SI:46 Formula:C24H36O2 CAS:40013-88-5 MolWeight:356 RetIndex:2760
CompName:9,10-Secochola-5,7,10(19)-trien-24-al, 3-hydroxy-, (3.beta.,5Z,7E)- $$ 25,26,27-Trinorcholecalcifer-24-al $$ 25,26,27-Trisnorcholecalcif-24-al $$ (

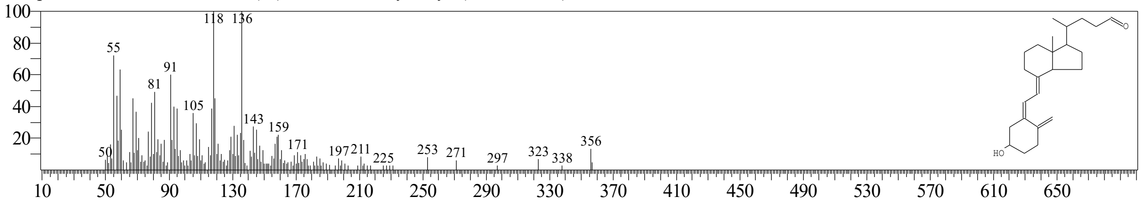

Hit#:3 Entry:112444 Library:NIST11.lib
SI:45 Formula:C19H32O2 CAS:0-00-0 MolWeight:292 RetIndex:2101
CompName:Methyl 3-cis,9-cis,12-cis-octadecatrienoate

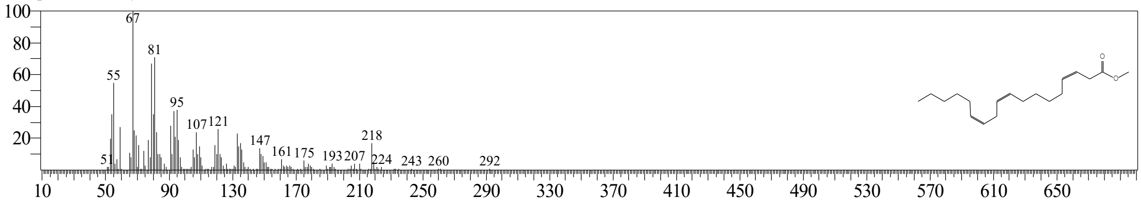

Hit#:4 Entry:170304 Library:NIST11.lib
SI:45 Formula:C23H34O4 CAS:0-00-0 MolWeight:374 RetIndex:2676
CompName:Fumaric acid, decyl 2-isopropylphenyl ester

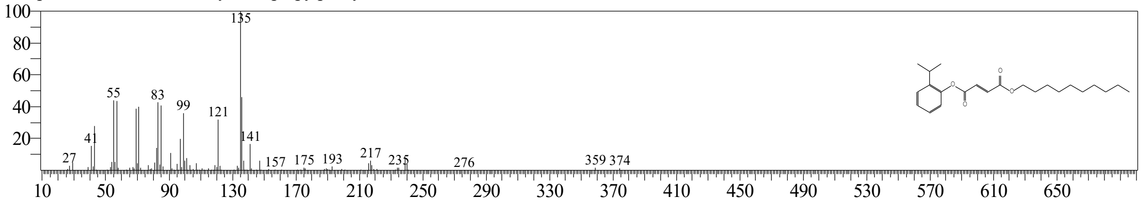

Hit#:5 Entry:123524 Library:NIST11.lib
SI:45 Formula:C20H34O2 CAS:1438-66-0 MolWeight:306 RetIndex:2204
CompName:1-Naphthalenepropanol, .alpha.-ethenyldecahydro-4-hydroxy-.alpha.,5,5,8a-tetramethyl-2-methylene-, [1S-[1.alpha.(R*),4.beta.,4a.beta.,8a.alpha.]]

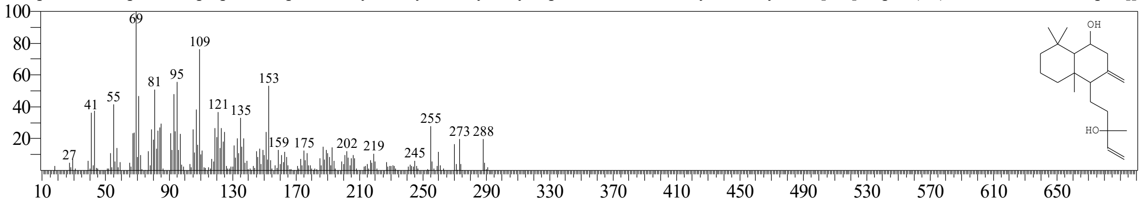

Qualitative Analysis Report

<< Target >>
Line#:105 R.Time:26.240(Scan#:4449) MassPeaks:352
RawMode:Averaged 26.235-26.245(4448-4450) BasePeak:55.05(2169)
BG Mode:Calc. from Peak Group 1 - Event 1 Scan

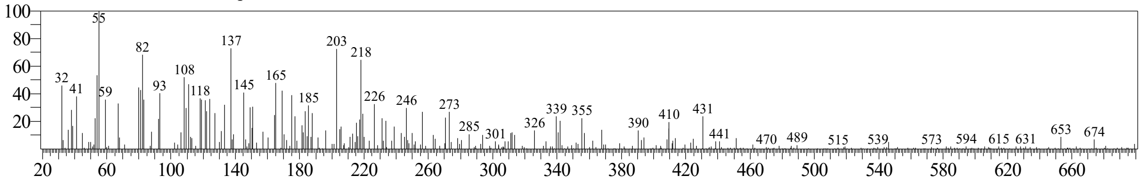

Hit#:1 Entry:140437 Library:NIST11.lib
SI:47 Formula:C24H40 CAS:54411-82-4 MolWeight:328 RetIndex:2154
CompName:Chol-23-ene, (5.beta.)- $$ Chol-23-ene # $$

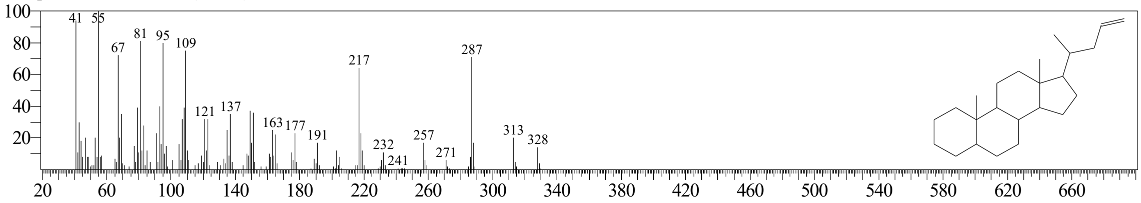

Hit#:2 Entry:168182 Library:NIST11.lib
SI:47 Formula:C27H46 CAS:30658-62-9 MolWeight:370 RetIndex:2406
CompName:Cholest-23-ene, (5.beta.)- $$ (23E)-Cholest-23-ene # $$

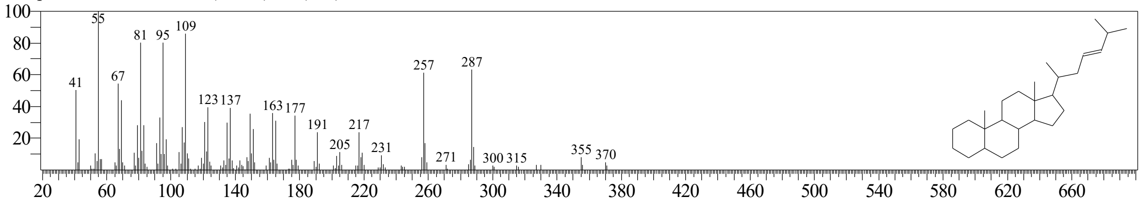

Hit#:3 Entry:101403 Library:NIST11.lib
SI:47 Formula:C18H30O2 CAS:1153-34-0 MolWeight:278 RetIndex:1774
CompName:5H-3,5a-Epoxynaphth[2,1-c]oxepin, dodecahydro-3,8,8,11a-tetramethyl-, [3S-(3.alpha.,5a.alpha.,7a.alpha.,11a.beta.,11b.alpha.)]- $$ 15,16-Dinorla

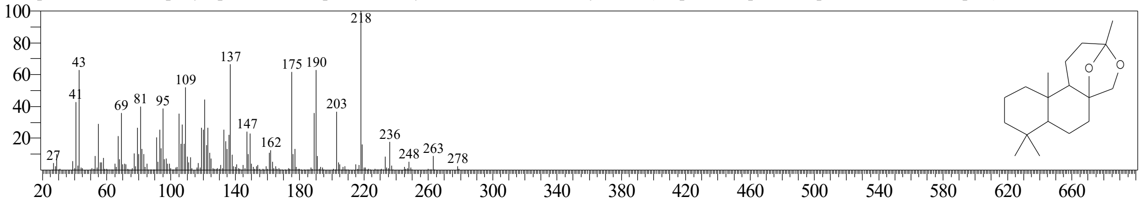

Hit#:4 Entry:98409 Library:NIST11.lib
SI:46 Formula:C17H26N2O CAS:0-00-0 MolWeight:274 RetIndex:2254
CompName:2-Cyclohexyl-4-ethylidene-octahydrobenzo[e][1,2]oxazine-3-carbonitrile $$ (4Z)-2-Cyclohexyl-4-ethylideneoctahydro-2H-1,2-benzoxazine-3-carb

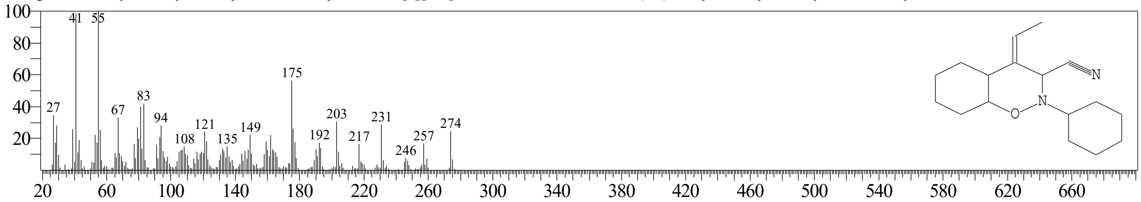

Hit#:5 Entry:109378 Library:NIST11.lib
SI:46 Formula:C21H36 CAS:481-26-5 MolWeight:288 RetIndex:1929
CompName:Pregnane $$ Pregnane, (5.beta.)- $$ 5.beta.-Pregnane $$ 17.beta.-Ethyletiocholane $$

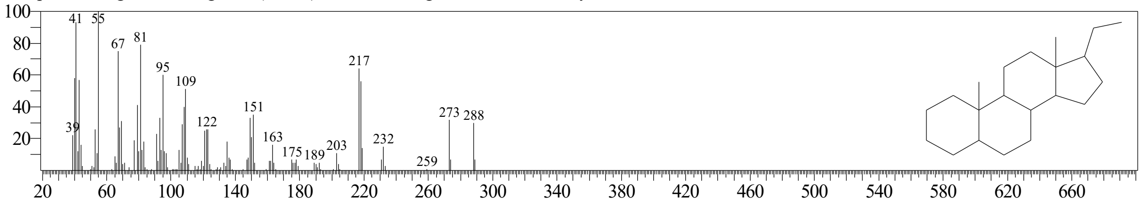

<< Target >>
Line#:106 R.Time:26.385(Scan#:4478) MassPeaks:399
RawMode:Averaged 26.380-26.390(4477-4479) BasePeak:218.15(5771)
BG Mode:Calc. from Peak Group 1 - Event 1 Scan

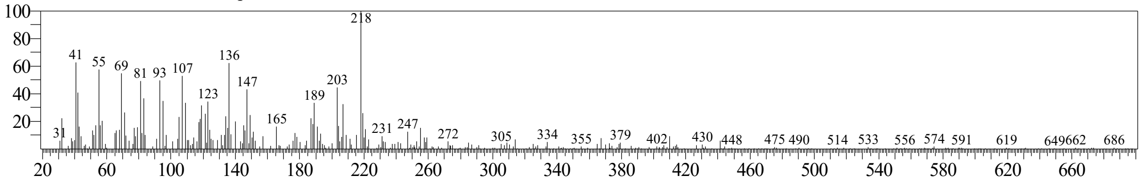

Hit#:1 Entry:200402 Library:NIST11.lib
SI:66 Formula:C32H52O2 CAS:0-00-0 MolWeight:468 RetIndex:3022
CompName:Acetic acid, 17-(1,5-dimethylhex-4-enyl)-4,4,8,10,14-pentamethyl-2,3,4,5,6,7,8,9,10,11,12,14,15,16-tetradecahydro-1H-cyclopenta[a]phenanthrene

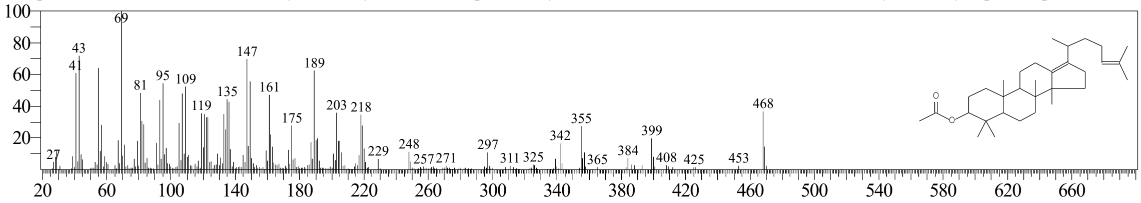

Hit#:2 Entry:191097 Library:NIST11.lib
SI:66 Formula:C30H50O CAS:545-47-1 MolWeight:426 RetIndex:2848
CompName:Lupeol $$ Lup-20(29)-en-3-ol, (3.beta.)- $$ Lup-20(29)-en-3.beta.-ol $$ .beta.-Viscol $$ Clerodol $$ Fagarasterol $$ Fagarsterol $$ Lupenol $$ Mo

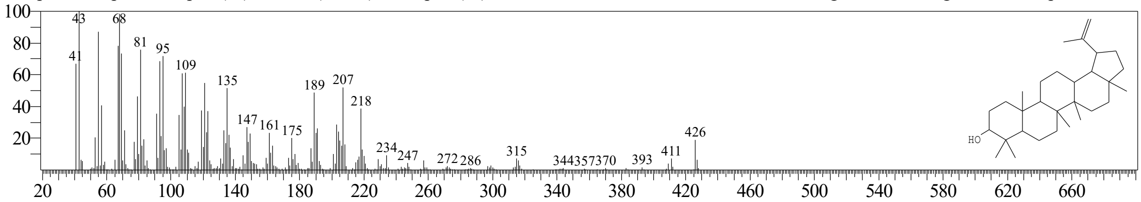

Hit#:3 Entry:200399 Library:NIST11.lib
SI:65 Formula:C32H52O2 CAS:1617-68-1 MolWeight:468 RetIndex:2987
CompName:Lup-20(29)-en-3-ol, acetate, (3.beta.)- $$ Lup-20(29)-en-3.beta.-ol, acetate $$ Lupenyl acetate $$ Lupeol acetate $$ Lupeyl acetate $$ 3-O-Acetyllu

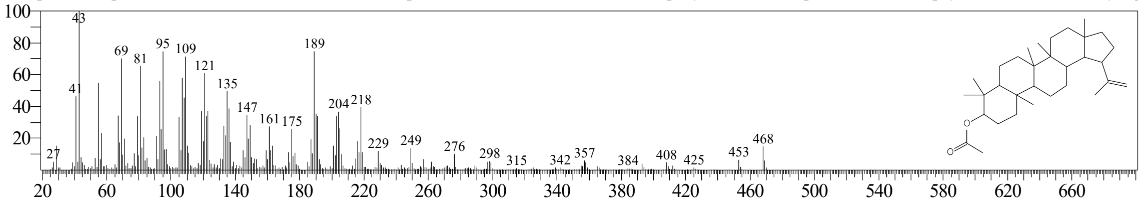

Hit#:4 Entry:186088 Library:NIST11.lib
SI:64 Formula:C30H50 CAS:464-97-1 MolWeight:410 RetIndex:2685
CompName:Urs-12-ene

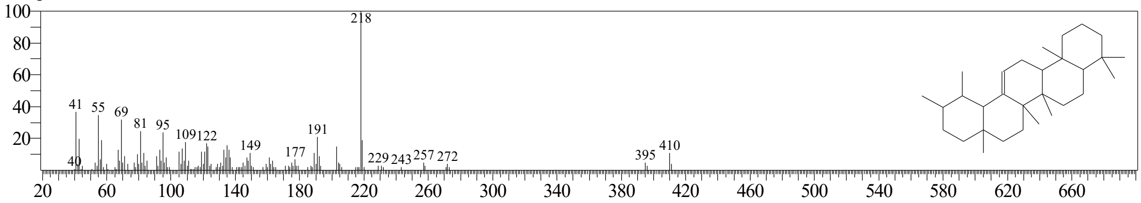

Hit#:5 Entry:56374 Library:NIST11.lib
SI:63 Formula:C15H22O CAS:0-00-0 MolWeight:218 RetIndex:1673
CompName:2(1H)Naphthalenone, 3,5,6,7,8,8a-hexahydro-4,8a-dimethyl-6-(1-methylethenyl)- $$ 6-Isopropenyl-4,8a-dimethyl-3,5,6,7,8,8a-hexahydro-2(1H)-na

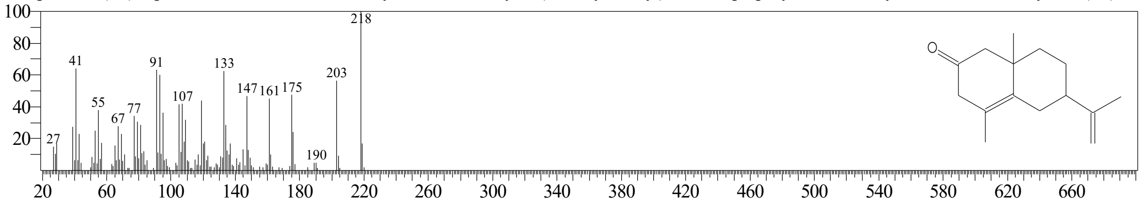

<< Target >>
Line#:107 R.Time:26.460(Scan#:4493) MassPeaks:349
RawMode:Averaged 26.455-26.465(4492-4494) BasePeak:218.15(1958)
BG Mode:Calc. from Peak Group 1 - Event 1 Scan

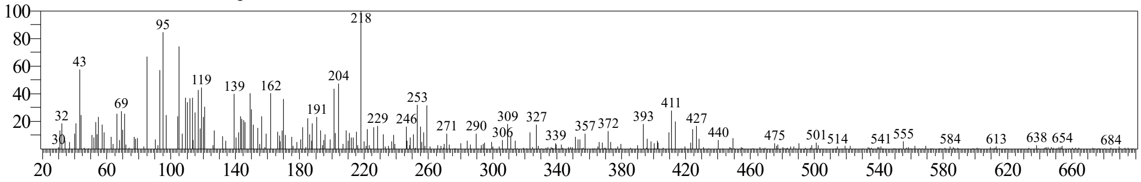

Hit#:1 Entry:69278 Library:NIST11.lib
SI:46 Formula:C15H24O2 CAS:0-00-0 MolWeight:236 RetIndex:1904
CompName:Bicyclo[4.4.0]dec-2-ene-4-ol, 2-methyl-9-(prop-1-en-3-ol-2-yl)- $$ 6-[1-(Hydroxymethyl)vinyl]-4,8a-dimethyl-1,2,4a,5,6,7,8,8a-octahydro-2-naphtl

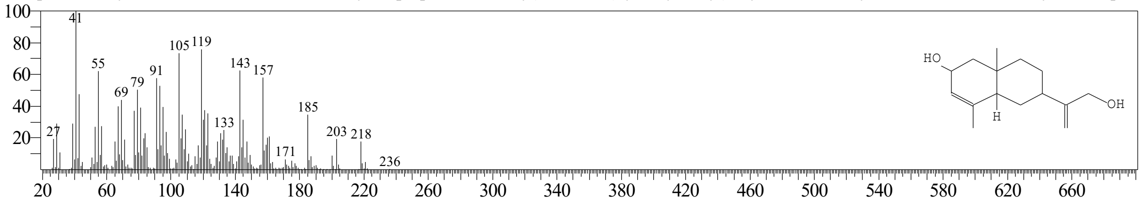

Hit#:2 Entry:88712 Library:NIST11.lib
SI:45 Formula:C15H18O4 CAS:139343-89-8 MolWeight:262 RetIndex:1714
CompName:2H-Bisoxireno[2,3:8,8a]azuleno[4,5-b]furan-7(3aH)-one, octahydro-3a,8c-dimethyl-6-methylene-, (1aR,2aR,3aS,5aS,8aS,8bS,8cS)- $$ Estafiatin, i

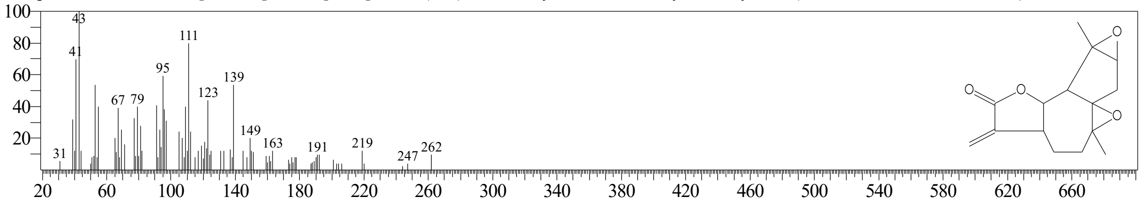

Hit#:3 Entry:163086 Library:NIST11.lib
SI:45 Formula:C21H30O5 CAS:10005-77-3 MolWeight:362 RetIndex:2774
CompName:Pregn-5-ene-12,20-dione, 3,14,15-trihydroxy-, (3.beta.,14.beta.,15.alpha.)- $$ 14.beta.-Pregn-5-ene-12,20-dione, 3.beta.,14,15.alpha.-trihydroxy- $

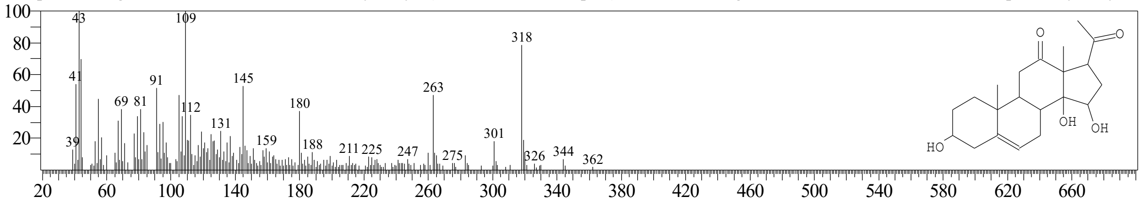

Hit#:4 Entry:155526 Library:NIST11.lib
SI:45 Formula:C19H26O6 CAS:33649-17-1 MolWeight:350 RetIndex:2612
CompName:Propanoic acid, 2-methyl-, (dodecahydro-6a-hydroxy-9a-methyl-3-methylene-2,9-dioxoazuleno[4,5-b]furan-6-yl)methyl ester, [3aS-(3a.alpha.,6.bet

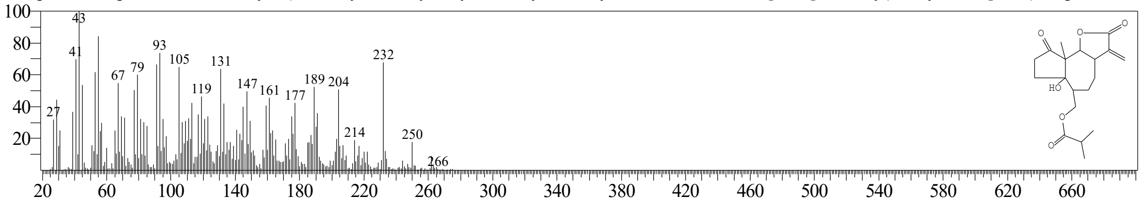

Hit#:5 Entry:143314 Library:NIST11.lib
SI:44 Formula:C22H20OS CAS:0-00-0 MolWeight:332 RetIndex:2788
CompName:1,1-Diphenyl-4-phenylthiobut-3-en-1-ol $$ (3E)-1,1-Diphenyl-4-(phenylsulfanyl)-3-buten-1-ol # $$

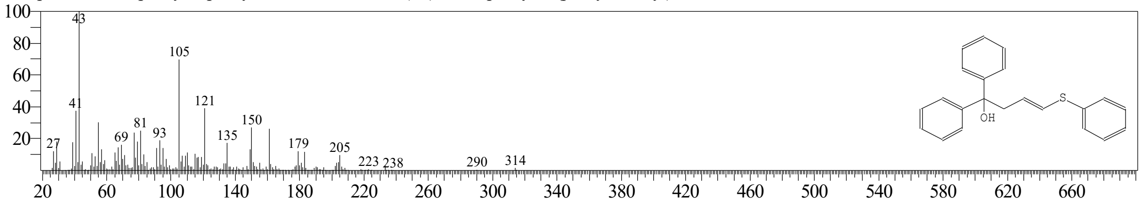

Qualitative Analysis Report

<< Target >>
Line#:108 R.Time:26.475(Scan#:4496) MassPeaks:361
RawMode:Averaged 26.470-26.480(4495-4497) BasePeak:95.00(3095)
BG Mode:Calc. from Peak Group 1 - Event 1 Scan

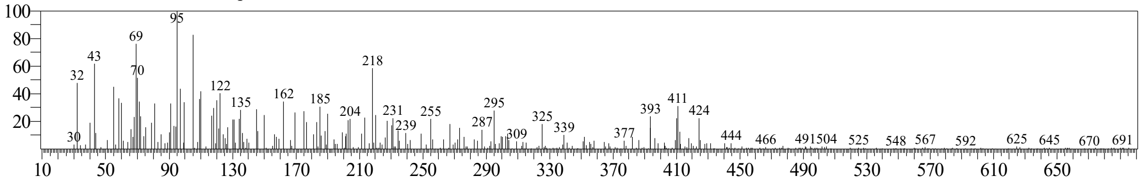

Hit#:1 Entry:176221 Library:NIST11.lib
SI:49 Formula:C22H30N2O4 CAS:55283-40-4 MolWeight:386 RetIndex:2967
CompName:3,9-Methano-10H-furo[3,2-d]azonine-10,11-dione, 9-[2-(dimethylamino)-3-methoxyphenyl]decahydro-2,6-dimethyl-, [2R-(2R*,3R*,3aS*,9R*,10a

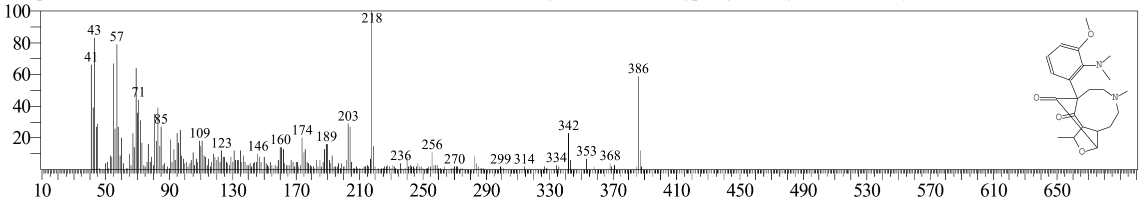

Hit#:2 Entry:186093 Library:NIST11.lib
SI:47 Formula:C30H50 CAS:0-00-0 MolWeight:410 RetIndex:2673
CompName:3-(1,5-Dimethyl-hexyl)-3a,10,10,12b-tetramethyl-1,2,3,3a,4,6,8,9,10,10a,11,12,12a,12b-tetradecahydro-benzo[4,5]cyclohepta[1,2-E]indene $$ 1(10

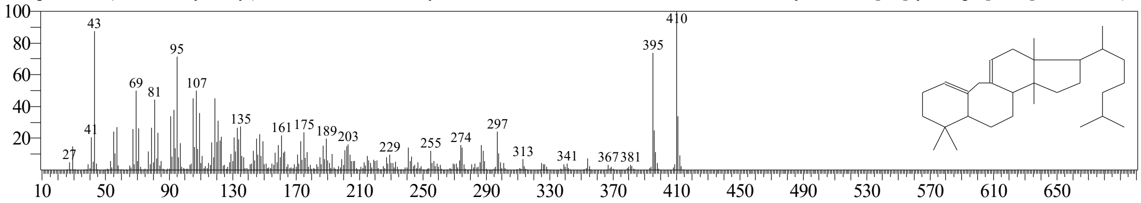

Hit#:3 Entry:191101 Library:NIST11.lib
SI:47 Formula:C30H50O CAS:469-39-6 MolWeight:426 RetIndex:2760
CompName:9,19-Cycloergost-24(28)-en-3-ol, 4,14-dimethyl-, (3.beta.,4.alpha.,5.alpha.)- $$ 9,19-Cyclo-5.alpha.,9.beta.-ergost-24(28)-en-3.beta.-ol, 4.alpha.,14

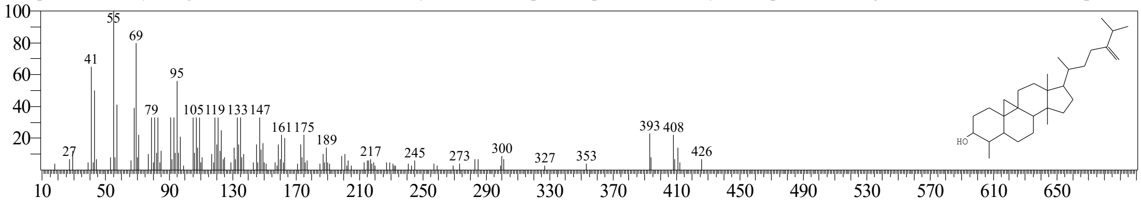

Hit#:4 Entry:99975 Library:NIST11.lib
SI:47 Formula:C19H32O CAS:54411-60-8 MolWeight:276 RetIndex:1953
CompName:D-Norandrostane-16-methanol, (5.alpha.,16.beta.)- $$ (8a,10a-Dimethylhexadecahydrocyclobuta[a]phenanthren-1-yl)methanol # $$

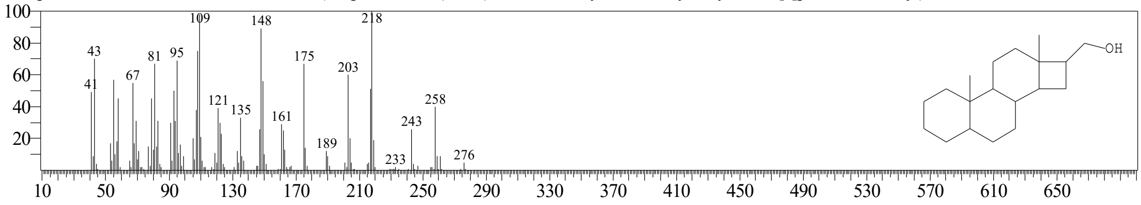

Hit#:5 Entry:205488 Library:NIST11.lib
SI:46 Formula:C30H52O6 CAS:56053-00-0 MolWeight:508 RetIndex:3493
CompName:Ergostane-3,5,6,12,25-pentol, 25-acetate, (3.beta.,5.alpha.,6.beta.,12.beta.)- $$ 3,5,6,12-Tetrahydroxyergostan-25-yl acetate # $$

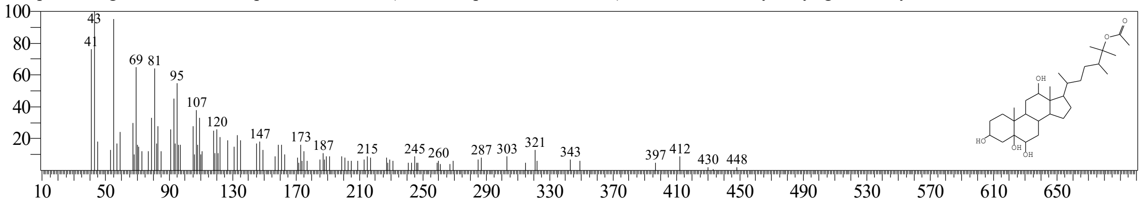

Qualitative Analysis Report

<< Target >>
Line#:109 R.Time:26.490(Scan#:4499) MassPeaks:308
RawMode:Averaged 26.485-26.495(4498-4500) BasePeak:95.10(1478)
BG Mode:Calc. from Peak Group 1 - Event 1 Scan

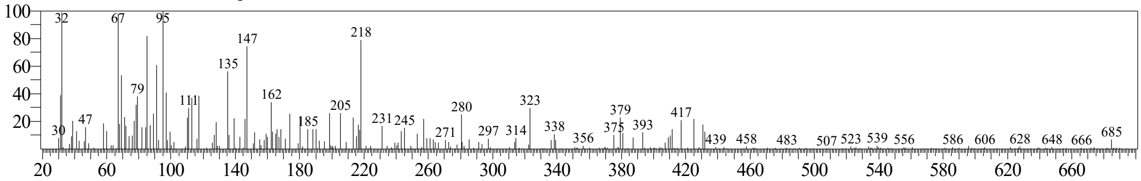

Hit#:1 Entry:7226 Library:NIST11.lib
SI:43 Formula:C7H12S CAS:54725-38-1 MolWeight:128 RetIndex:944
CompName:7-Thiabicyclo[4.1.0]heptane, 3-methyl- $$ 3-Methyl-7-thiabicyclo[4.1.0]heptane # $$

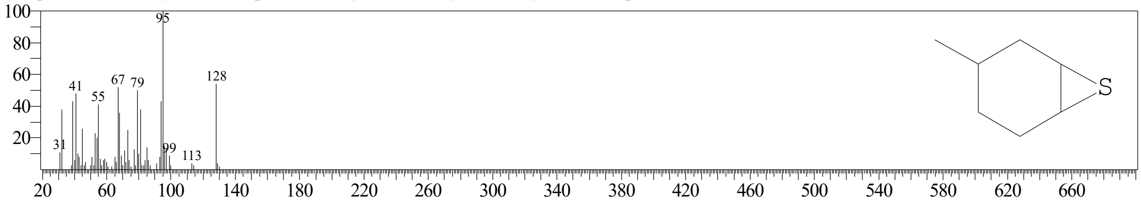

Hit#:2 Entry:7225 Library:NIST11.lib
SI:42 Formula:C7H12S CAS:54773-76-1 MolWeight:128 RetIndex:944
CompName:7-Thiabicyclo[4.1.0]heptane, 2-methyl- $$ 2-Methyl-7-thiabicyclo[4.1.0]heptane # $$

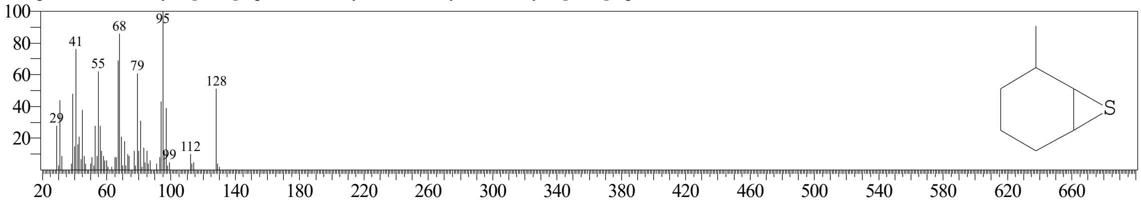

Hit#:3 Entry:65974 Library:NIST11.lib
SI:42 Formula:C10H17BrO CAS:82252-98-0 MolWeight:232 RetIndex:1434
CompName:Bicyclo[4.1.0]heptane-7-methanol, 7-bromo-.alpha.-ethyl-, (1.alpha.,6.alpha.,7.alpha.)- $$ 1-(7-Bromobicyclo[4.1.0]hept-7-yl)-1-propanol # $$

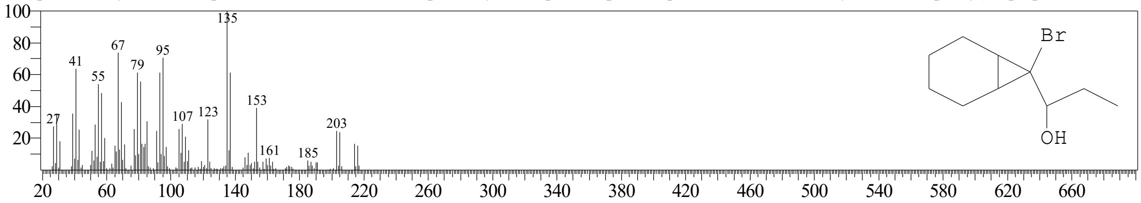

Hit#:4 Entry:182687 Library:NIST11.lib
SI:40 Formula:C28H48O CAS:22599-96-8 MolWeight:400 RetIndex:2652
CompName:Cholestan-3-ol, 2-methylene-, (3.beta.,5.alpha.)- $$ 5.alpha.-Cholestan-3.beta.-ol, 2-methylene- $$ 2-Methylenecholestan-3-ol # $$

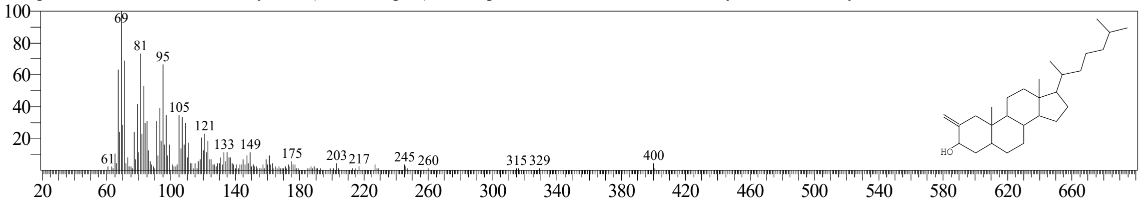

Hit#:5 Entry:135064 Library:NIST11.lib
SI:40 Formula:C12H12Cl2O4S CAS:0-00-0 MolWeight:322 RetIndex:2531
CompName:Cyclopentanecarboxylic acid, 1-(3,4-dichlorophenylsulfonyl)- $$ 1-[(3,4-Dichlorophenyl)sulfonyl]cyclopentanecarboxylic acid # $$

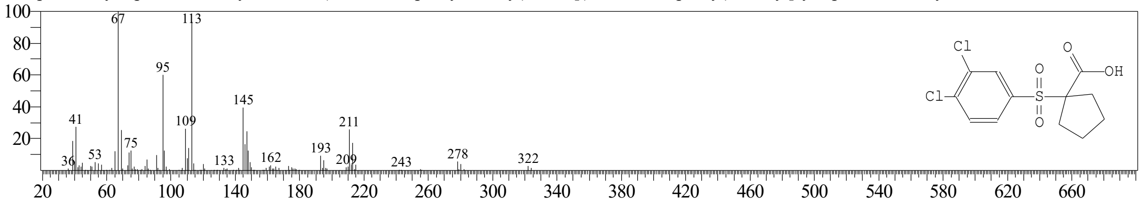

<< Target >>
Line#:110 R.Time:26.500(Scan#:4501) MassPeaks:345
RawMode:Averaged 26.495-26.505(4500-4502) BasePeak:69.10(3090)
BG Mode:Calc. from Peak Group 1 - Event 1 Scan

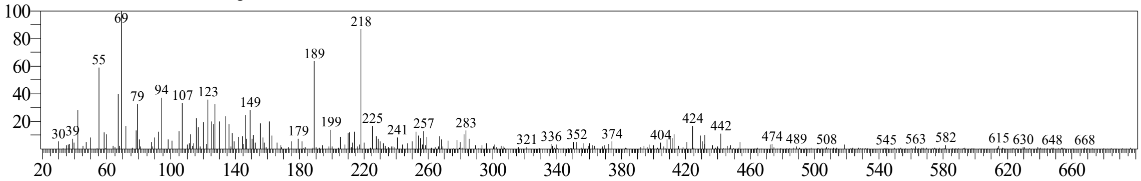

Hit#:1 Entry:190451 Library:NIST11.lib
SI:43 Formula:C30H48O CAS:0-00-0 MolWeight:424 RetIndex:2869
CompName:4,4,6a,6b,8a,11,11,14b-Octamethyl-1,4,4a,5,6,6a,6b,7,8,8a,9,10,11,12,12a,14,14a,14b-octadecahydro-2H-picen-3-one $$ Olean-12-en-3-one # $$

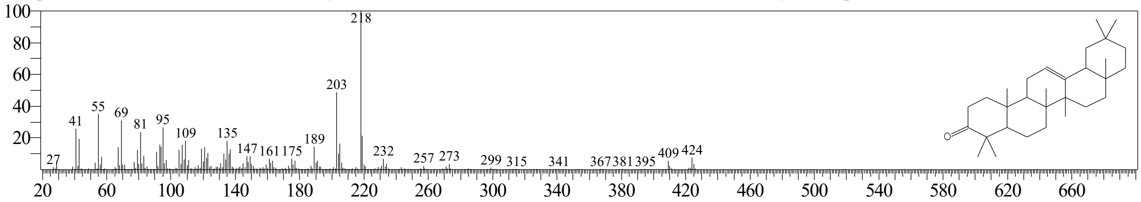

Hit#:2 Entry:56401 Library:NIST11.lib
SI:42 Formula:C16H26 CAS:2435-85-0 MolWeight:218 RetIndex:1502
CompName:Pyrene, hexadecahydro- $$ Hexadecahydropyrene $$ Perhydropyrene $$

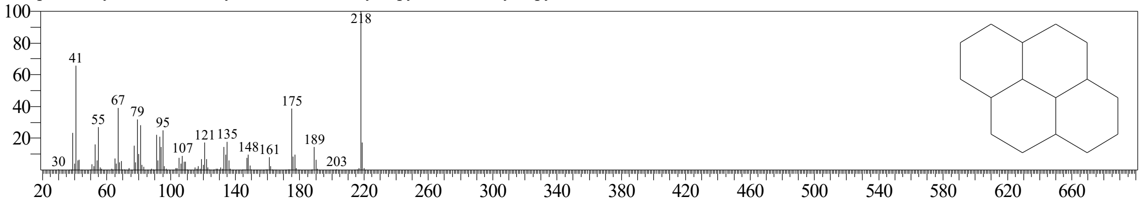

Hit#:3 Entry:190452 Library:NIST11.lib
SI:42 Formula:C30H48O CAS:0-00-0 MolWeight:424 RetIndex:2856
CompName:4,4,6a,6b,8a,11,12,14b-Octamethyl-1,4,4a,5,6,6a,6b,7,8,8a,9,10,11,12,12a,14,14a,14b-octadecahydro-2H-picen-3-one $$ Urs-12-en-3-one # $$

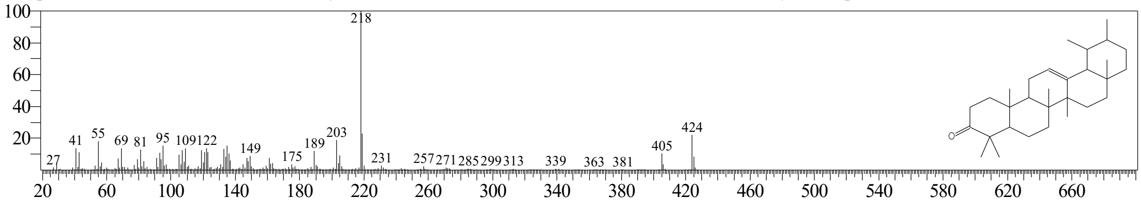

Hit#:4 Entry:104026 Library:NIST11.lib
SI:42 Formula:C15H19ClO3 CAS:0-00-0 MolWeight:282 RetIndex:1998
CompName:Diethylmalonic acid, monochloride, 2-ethylphenyl ester

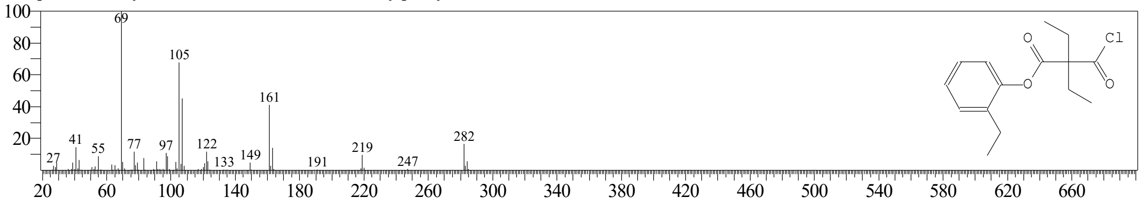

Hit#:5 Entry:56241 Library:NIST11.lib
SI:41 Formula:C14H18O2 CAS:0-00-0 MolWeight:218 RetIndex:1700
CompName:3-Cyclopentylpropionic acid, phenyl ester $$ Phenyl 3-cyclopentylpropanoate # $$

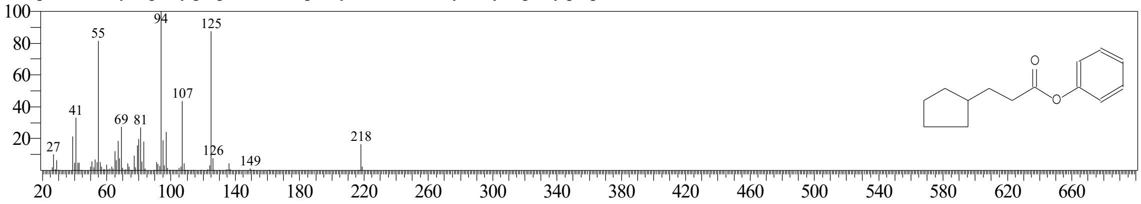

<< Target >>
Line#:111 R.Time:26.550(Scan#:4511) MassPeaks:370
RawMode:Averaged 26.545-26.555(4510-4512) BasePeak:55.00(3811)
BG Mode:Calc. from Peak Group 1 - Event 1 Scan

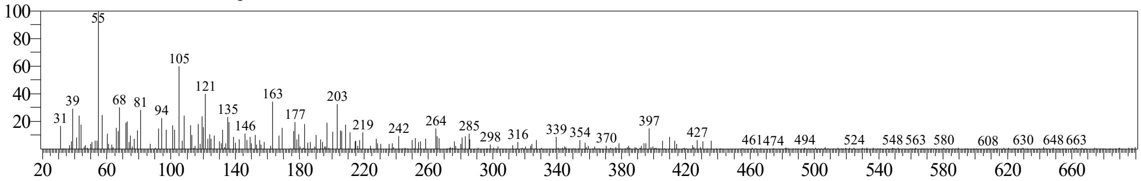

Hit#:1 Entry:30385 Library:NIST11.lib
SI:46 Formula:C12H18O CAS:0-00-0 MolWeight:178 RetIndex:1193
CompName:1-(3-Isopropylidene-5,5-dimethyl-bicyclo[2.1.0]pent-2-yl)-ethanone $$ 1-[5,5-Dimethyl-3-(1-methylethylidene)bicyclo[2.1.0]pent-2-yl]ethanone #

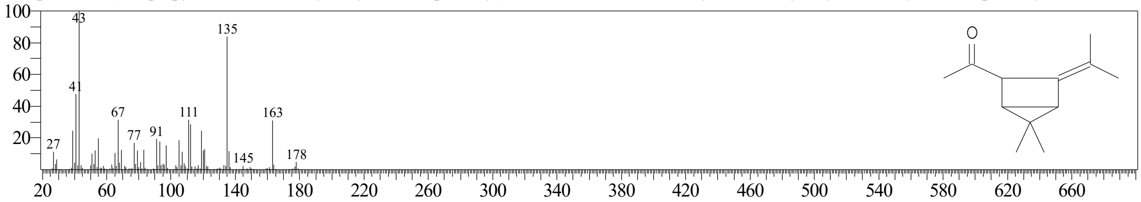

Hit#:2 Entry:40071 Library:NIST11.lib
SI:46 Formula:C12H18O2 CAS:0-00-0 MolWeight:194 RetIndex:1397
CompName:Cyclohexene, 4-(2-(ethoxycarbonyl)ethenyl)-5-methyl- $$ Ethyl (2E)-3-(6-methyl-3-cyclohexen-1-yl)-2-propenoate # $$

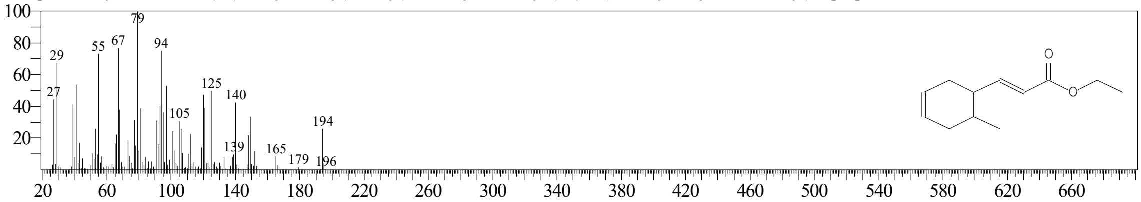

Hit#:3 Entry:72715 Library:NIST11.lib
SI:46 Formula:C12H19NO4 CAS:29085-46-9 MolWeight:241 RetIndex:1697
CompName:4,9-Decadienoic acid, 2-nitro-, ethyl ester $$ Acetic acid, 2-nitro-2-(2,7-octadien-1-yl)-, ethyl ester (isomer 2) $$ Ethyl (4E)-2-nitro-4,9-decadienoa

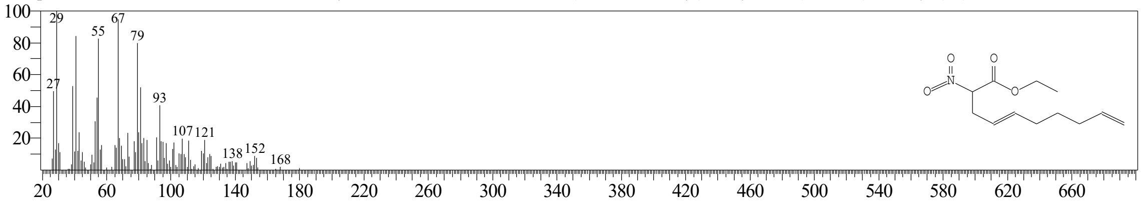

Hit#:4 Entry:56315 Library:NIST11.lib
SI:46 Formula:C15H22O CAS:70038-20-9 MolWeight:218 RetIndex:1452
CompName:7-Oxabicyclo[4.1.0]heptane, 2,2,6-trimethyl-1-(3-methyl-1,3-butadienyl)-5-methylene- $$ 2,2,6-Trimethyl-1-[(1E)-3-methyl-1,3-butadienyl]-5-meth

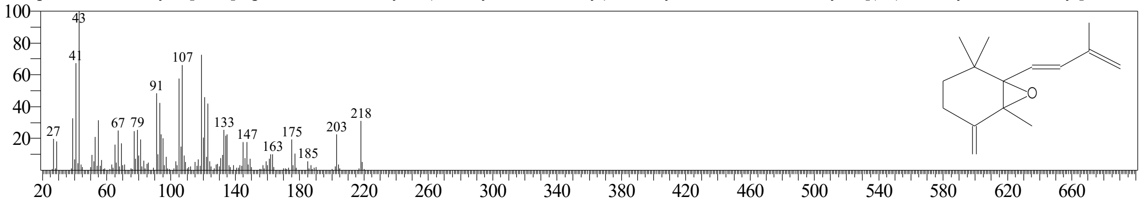

Hit#:5 Entry:123533 Library:NIST11.lib
SI:46 Formula:C20H34O2 CAS:0-00-0 MolWeight:306 RetIndex:2179
CompName:11-(3-Ethenylcyclopentyl)undec-10-enoic acid, ethyl ester $$ Ethyl (10Z)-11-(3-vinylcyclopentyl)-10-undecenoate # $$

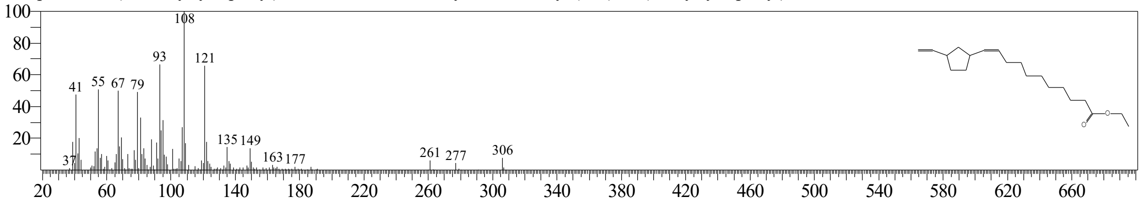

Qualitative Analysis Report

<< Target >>
Line#:112   R.Time:27.120(Scan#:4625) MassPeaks:367
RawMode:Averaged 27.115-27.125(4624-4626) BasePeak:43.10(12762)
BG Mode:Calc. from Peak Group 1 - Event 1 Scan

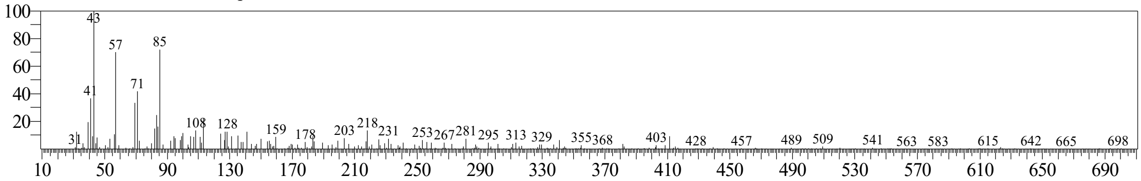

Hit#:1 Entry:52479 Library:NIST11.lib
SI:72 Formula:C15H32 CAS:25117-32-2 MolWeight:212 RetIndex:1448
CompName:Tetradecane, 5-methyl- $$ 5-Methyltetradecane # $$

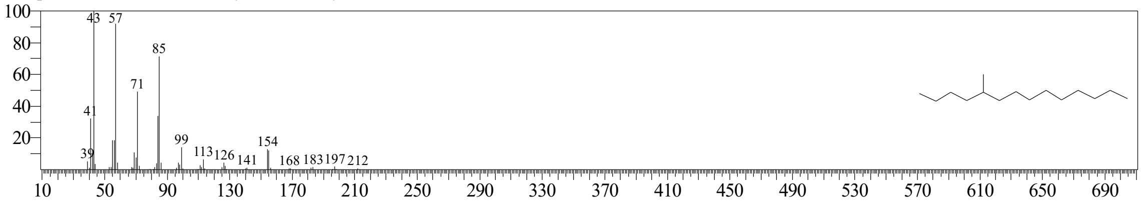

Hit#:2 Entry:126575 Library:NIST11.lib
SI:71 Formula:C22H46 CAS:75163-98-3 MolWeight:310 RetIndex:2080
CompName:Eicosane, 2,4-dimethyl- $$ 2,4-Dimethylicosane # $$

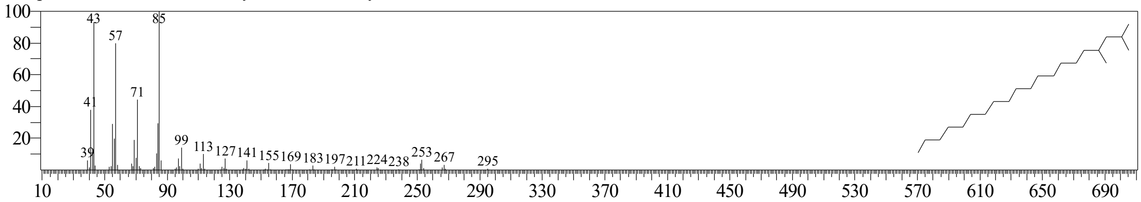

Hit#:3 Entry:42954 Library:NIST11.lib
SI:71 Formula:C14H30 CAS:6117-99-3 MolWeight:198 RetIndex:1285
CompName:2,4-Dimethyldodecane

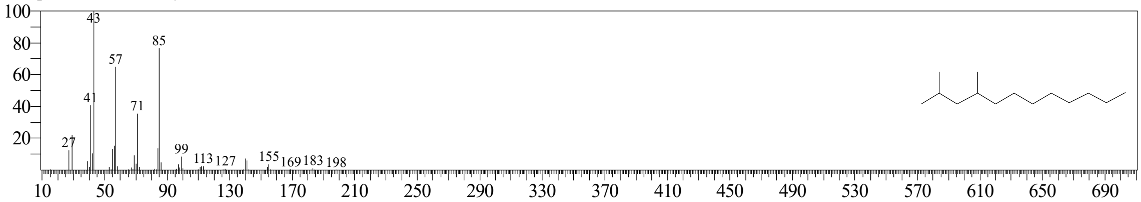

Hit#:4 Entry:154257 Library:NIST11.lib
SI:71 Formula:C19H40O3S CAS:0-00-0 MolWeight:348 RetIndex:2533
CompName:Sulfurous acid, hexyl tridecyl ester

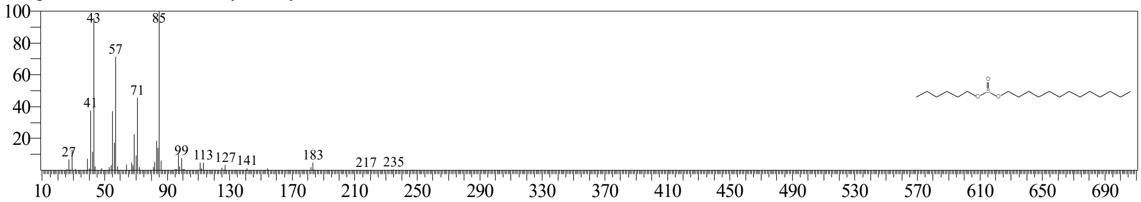

Hit#:5 Entry:147563 Library:NIST11.lib
SI:71 Formula:C24H50 CAS:77536-30-2 MolWeight:338 RetIndex:2279
CompName:Docosane, 2,4-dimethyl- $$ 2,4-Dimethyldocosane # $$

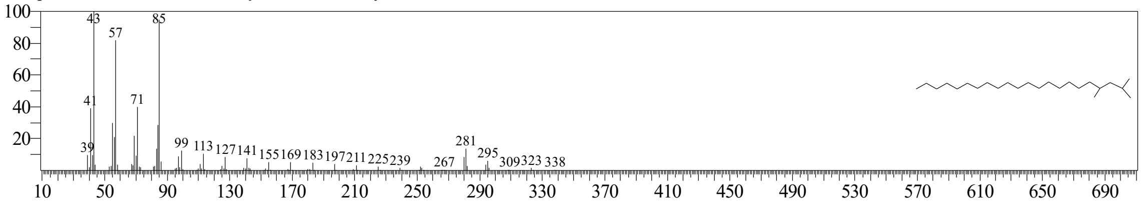

<< Target >>
Line#:113 R.Time:27.285(Scan#:4658) MassPeaks:430
RawMode:Averaged 27.280-27.290(4657-4659) BasePeak:69.10(16330)
BG Mode:Calc. from Peak Group 1 - Event 1 Scan

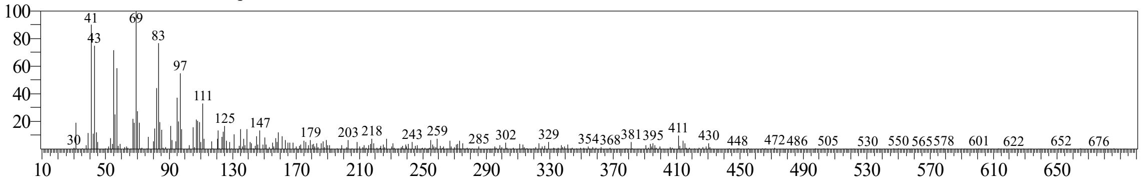

Hit#:1 Entry:73939 Library:NIST11.lib
SI:80 Formula:C16H34O CAS:36653-82-4 MolWeight:242 RetIndex:1854
CompName:1-Hexadecanol $$ n-Cetyl alcohol $$ n-Hexadecan-1-ol $$ n-Hexadecanol $$ n-1-Hexadecanol $$ Adol 52 $$ Adol 52 NF $$ Adol 54 $$ Aldol 54 $

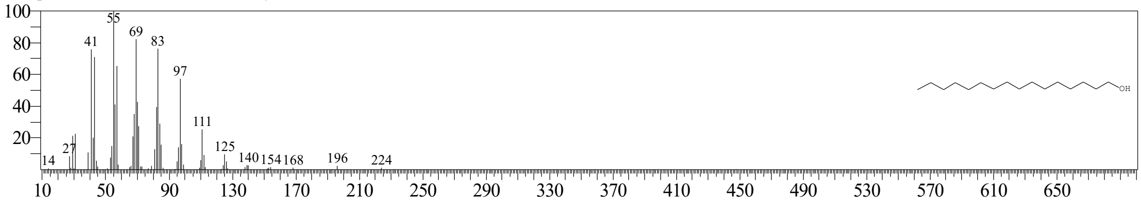

Hit#:2 Entry:91766 Library:NIST11.lib
SI:79 Formula:C16H26O3 CAS:19780-11-1 MolWeight:266 RetIndex:2159
CompName:2-Dodecen-1-yl(-)succinic anhydride $$ 2,5-Furandione, 3-dodecenyl- $$ n-Dodecenylsuccinic anhydride $$ Dodecenyl succinic anhydride $$ 2,5-I

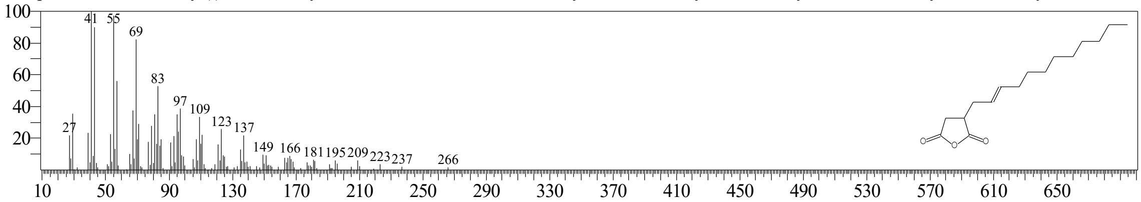

Hit#:3 Entry:177914 Library:NIST11.lib
SI:79 Formula:C20H39BrO2 CAS:18992-03-5 MolWeight:390 RetIndex:2473
CompName:Bromoacetic acid, octadecyl ester $$ Octadecyl bromoacetate # $$

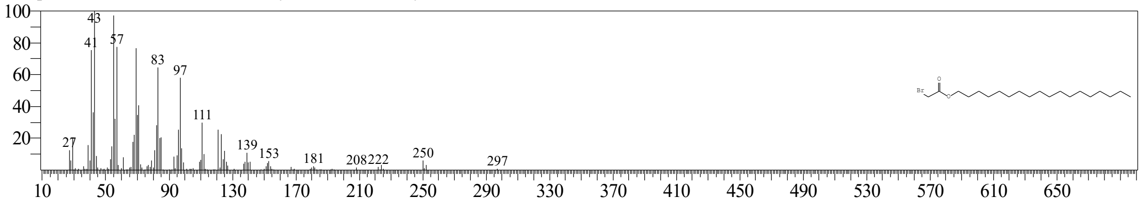

Hit#:4 Entry:162891 Library:NIST11.lib
SI:79 Formula:C18H35BrO2 CAS:5454-48-8 MolWeight:362 RetIndex:2274
CompName:Bromoacetic acid, hexadecyl ester $$ Hexadecyl bromoacetate # $$

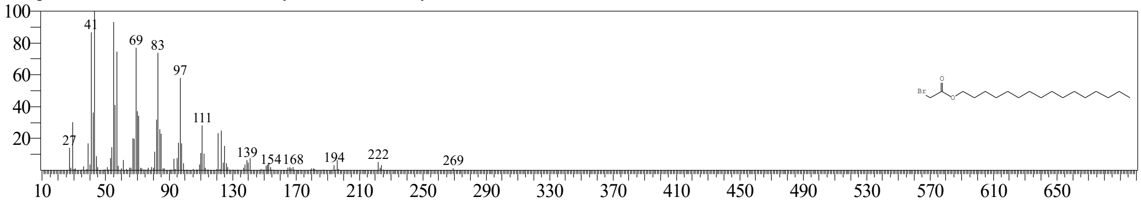

Hit#:5 Entry:154192 Library:NIST11.lib
SI:79 Formula:C18H37BrO CAS:59101-17-6 MolWeight:348 RetIndex:2349
CompName:1-Octadecanol, 18-bromo- $$ 18-Bromo-1-octadecanol # $$

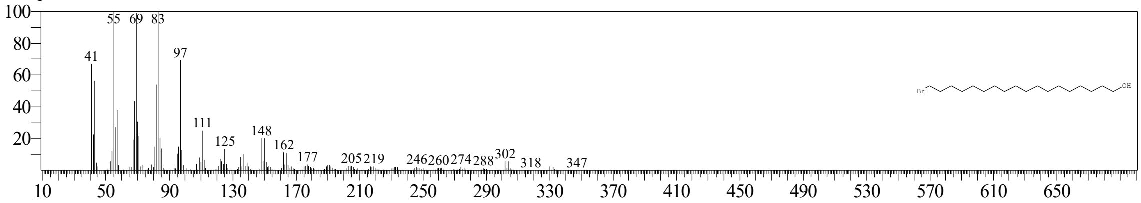

Qualitative Analysis Report

<< Target >>
Line#:114 R.Time:27.505(Scan#:4702) MassPeaks:363
RawMode:Averaged 27.500-27.510(4701-4703) BasePeak:83.10(2452)
BG Mode:Calc. from Peak Group 1 - Event 1 Scan

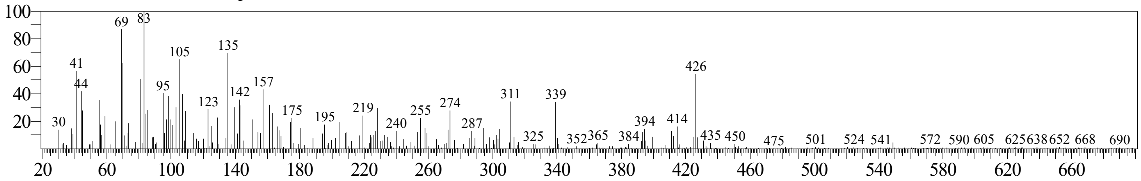

Hit#:1 Entry:60833 Library:NIST11.lib
SI:49 Formula:C14H24O2 CAS:0-00-0 MolWeight:224 RetIndex:1742
CompName:1-Hydroxymethyl-3,3-dimethyl-2-(3-methylbuta-1,3-dienyl)cyclohexanol $$ 1-(Hydroxymethyl)-3,3-dimethyl-2-[(1E)-3-methyl-1,3-butadienyl]cycl

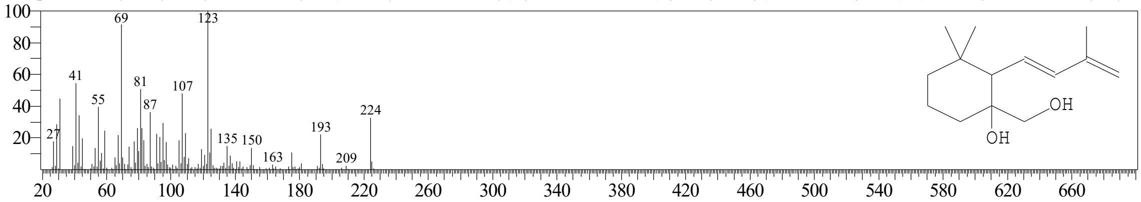

Hit#:2 Entry:31675 Library:NIST11.lib
SI:48 Formula:C12H20O CAS:762-26-5 MolWeight:180 RetIndex:1373
CompName:4,8-Decadienal, 5,9-dimethyl-

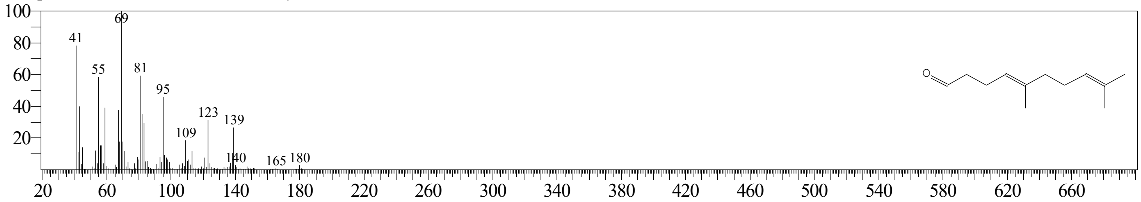

Hit#:3 Entry:151626 Library:NIST11.lib
SI:48 Formula:C19H36O5 CAS:55398-23-7 MolWeight:344 RetIndex:2347
CompName:1,2,4-Trioxolane-2-octanoic acid, 5-octyl-, methyl ester $$ Methyl oleate ozonide $$ Methyl 8-(5-octyl-1,2,4-trioxolan-3-yl)octanoate # $$

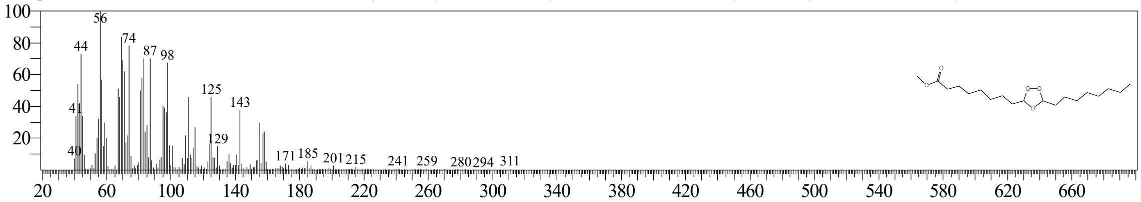

Hit#:4 Entry:42753 Library:NIST11.lib
SI:48 Formula:C12H22O2 CAS:7362-81-4 MolWeight:198 RetIndex:1419
CompName:Cyclohexanecarboxylic acid, 1-butyl-, methyl ester $$ 1-Butylcyclohexanecarboxylic acid methyl ester $$ Methyl 1-butylcyclohexanecarboxylate #

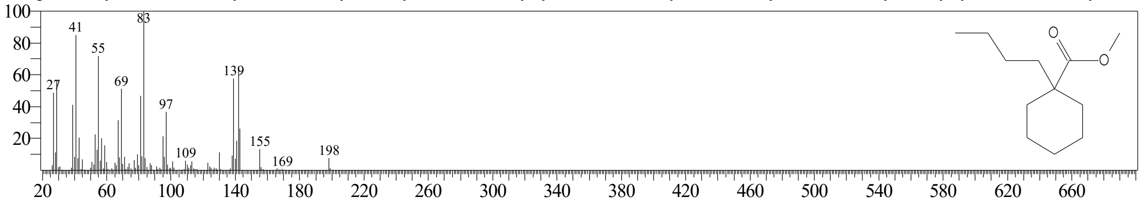

Hit#:5 Entry:210358 Library:NIST11.lib
SI:47 Formula:C33H46ClNO7 CAS:107024-98-6 MolWeight:603 RetIndex:4391
CompName:Milbemycin b, 13-chloro-5-demethoxy-28-deoxy-6,28-epoxy-5-(hydroxyimino)-25-(1-methylethyl)-, (6R,13R,25R)-

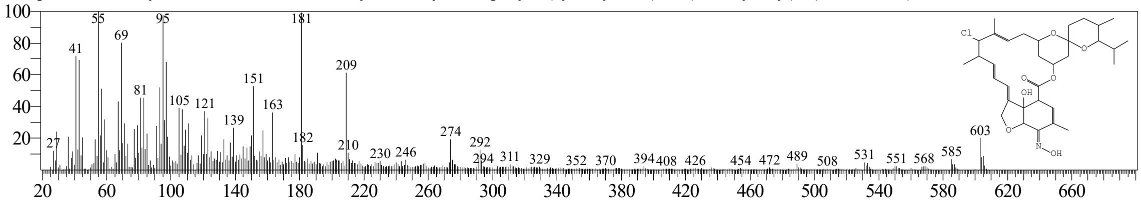

<< Target >>
Line#:115 R.Time:27.655(Scan#:4732) MassPeaks:336
RawMode:Averaged 27.650-27.660(4731-4733) BasePeak:81.00(3547)
BG Mode:Calc. from Peak Group 1 - Event 1 Scan

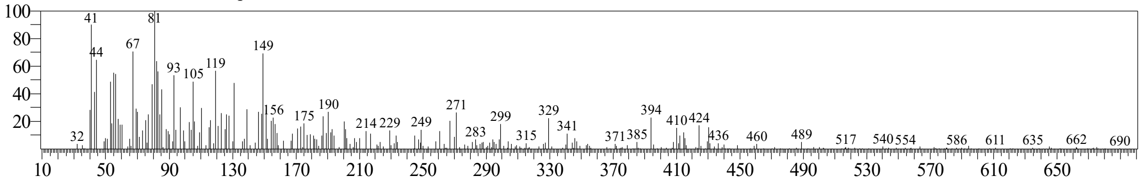

Hit#:1 Entry:141207 Library:NIST11.lib
SI:57 Formula:C15H23F5O2 CAS:0-00-0 MolWeight:330 RetIndex:1383
CompName:trans-2-Dodecen-1-ol, pentafluoropropionate

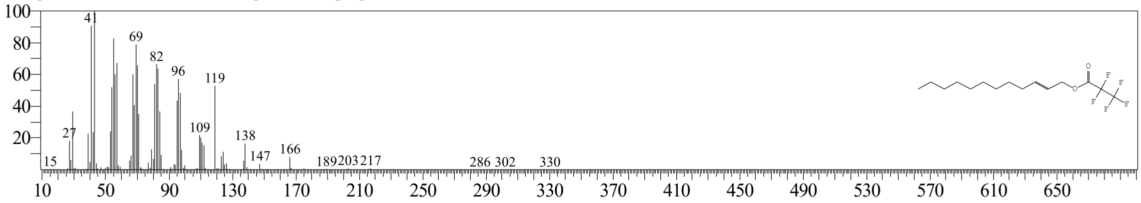

Hit#:2 Entry:119706 Library:NIST11.lib
SI:57 Formula:C13H19F5O2 CAS:0-00-0 MolWeight:302 RetIndex:1184
CompName:trans-2-Decen-1-ol, pentafluoropropionate

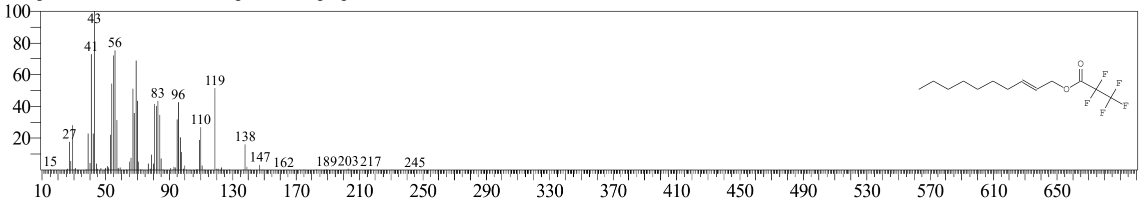

Hit#:3 Entry:119708 Library:NIST11.lib
SI:57 Formula:C13H19F5O2 CAS:0-00-0 MolWeight:302 RetIndex:1184
CompName:(Z)-4-Decen-1-ol, pentafluoropropionate

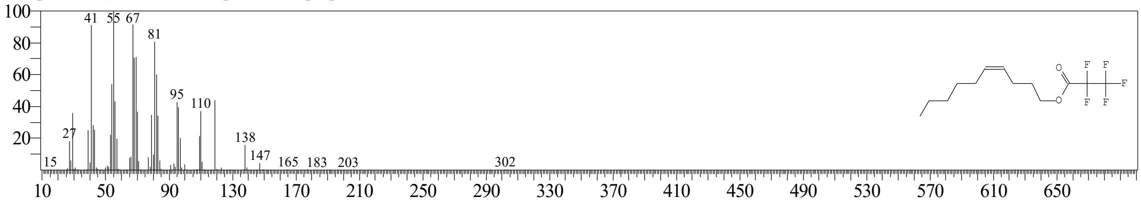

Hit#:4 Entry:66256 Library:NIST11.lib
SI:57 Formula:C12H21ClO2 CAS:15356-62-4 MolWeight:232 RetIndex:1525
CompName:(-)-Menthoxyacetyl chloride $$ L-Menthoxyacetyl chloride $$ Acetyl chloride, [[5-methyl-2-(1-methylethyl)cyclohexyl]oxy]-, [1R-(1.alpha.,2.beta.,

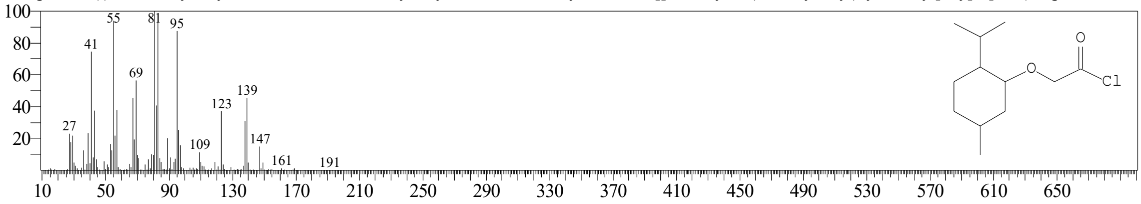

Hit#:5 Entry:57866 Library:NIST11.lib
SI:57 Formula:C16H28 CAS:0-00-0 MolWeight:220 RetIndex:1683
CompName:1,3-Di(cyclohexyl)but-1-ene $$ [(1Z)-3-Cyclohexyl-1-butenyl]cyclohexane # $$

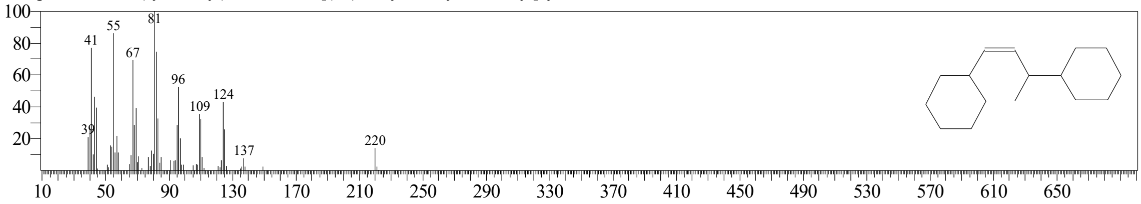

<< Target >>
Line#:116 R.Time:28.910(Scan#:4983) MassPeaks:373
RawMode:Averaged 28.905-28.915(4982-4984) BasePeak:69.00(4715)
BG Mode:Calc. from Peak Group 1 - Event 1 Scan

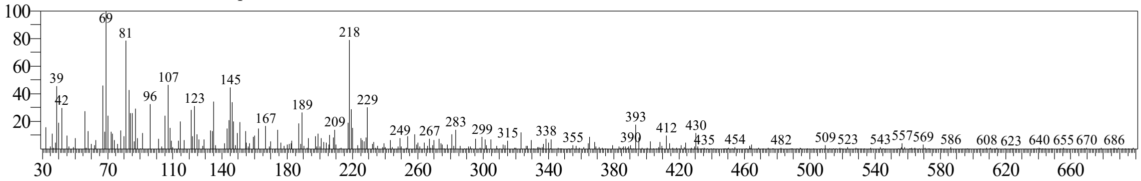

Hit#:1 Entry:182687 Library:NIST11.lib
SI:47 Formula:C28H48O CAS:22599-96-8 MolWeight:400 RetIndex:2652
CompName:Cholestan-3-ol, 2-methylene-, (3.beta.,5.alpha.)- $$ 5.alpha.-Cholestan-3.beta.-ol, 2-methylene- $$ 2-Methylenecholestan-3-ol # $$

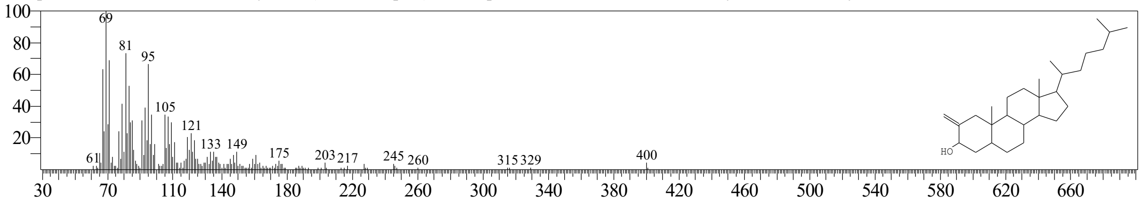

Hit#:2 Entry:55761 Library:NIST11.lib
SI:47 Formula:C10H10N4S CAS:0-00-0 MolWeight:218 RetIndex:1820
CompName:[1,2,4]Triazolo[1,5-a]pyrimidine, 5,7-dimethyl-2-(2-propynylthio)-

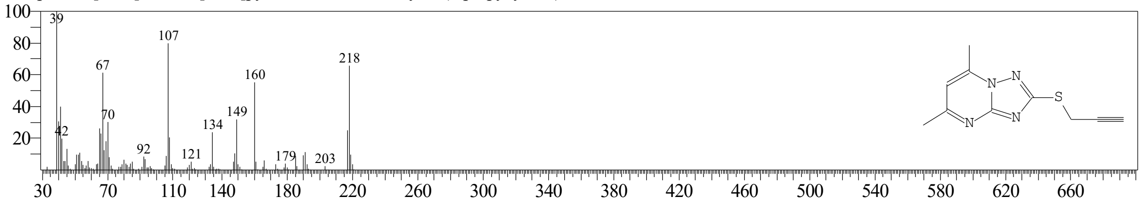

Hit#:3 Entry:182716 Library:NIST11.lib
SI:45 Formula:C29H52 CAS:601-58-1 MolWeight:400 RetIndex:2532
CompName:Stigmastane $$ 5.alpha.-Stigmastane $$ .alpha.-Stigmastane $$ (24R)-5.alpha.-Stigmastane $$ Cholestane, 24-ethyl-, (24R)- $$ Stigmastan $$ 1H-C

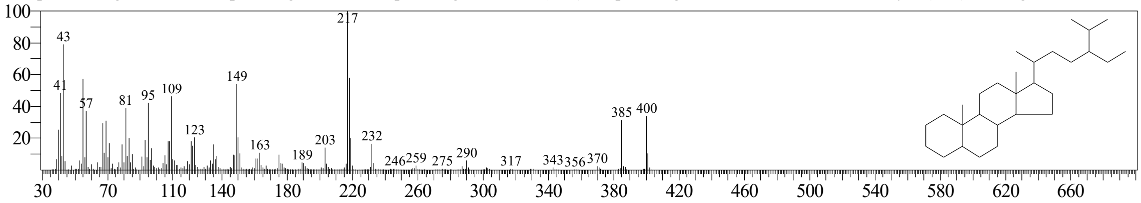

Hit#:4 Entry:191689 Library:NIST11.lib
SI:44 Formula:C30H52O CAS:24739-08-0 MolWeight:428 RetIndex:2921
CompName:C(14a)-Homo-27-nor-14.beta.-gammaceran-3.alpha.-ol

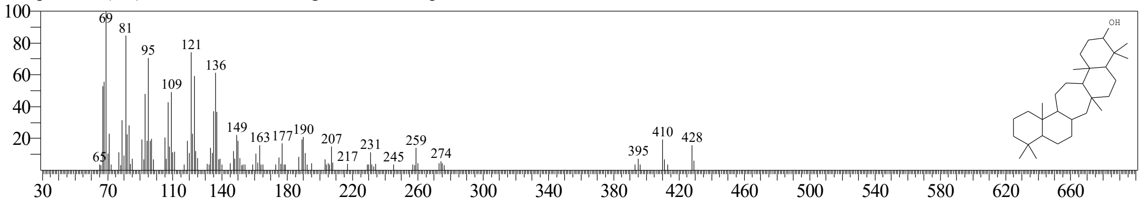

Hit#:5 Entry:186088 Library:NIST11.lib
SI:44 Formula:C30H50 CAS:464-97-1 MolWeight:410 RetIndex:2685
CompName:Urs-12-ene

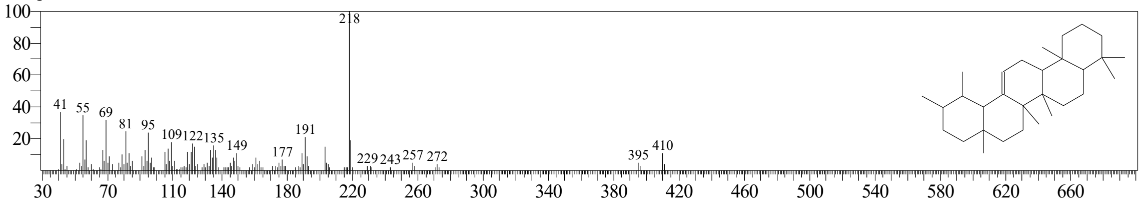

<< Target >>
Line#:117 R.Time:28.990(Scan#:4999) MassPeaks:332
RawMode:Averaged 28.985-28.995(4998-5000) BasePeak:43.10(3647)
BG Mode:Calc. from Peak Group 1 - Event 1 Scan

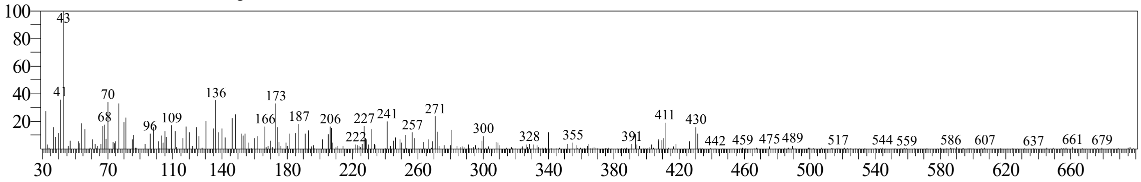

Hit#:1 Entry:32139 Library:NIST11.lib
SI:45 Formula:C11H19NO CAS:0-00-0 MolWeight:181 RetIndex:1379
CompName:1-Acetyl-2-methyl-6-(prop-2-enyl)piperidine (N-Acetylpinidine) $$ 1-Acetyl-2-allyl-6-methylpiperidine # $$

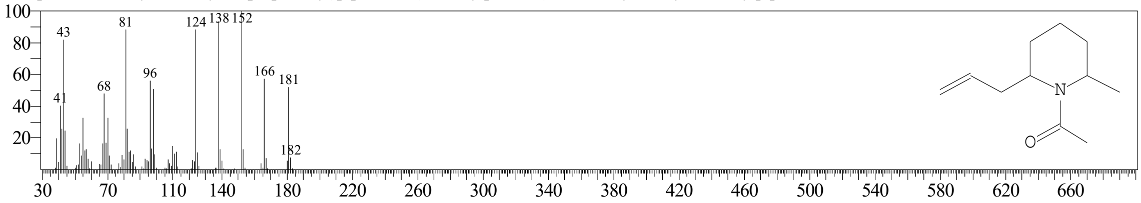

Hit#:2 Entry:39115 Library:NIST11.lib
SI:43 Formula:C11H15NO2 CAS:24417-32-1 MolWeight:193 RetIndex:1660
CompName:2-Cyclopentenone, 2-acethyl-3-(pyrrolidinyl)- $$ 2-Acetyl-3-(1-pyrrolidinyl)-2-cyclopenten-1-one # $$ 2-Cyclopentenone, 2-acetyl-3-(1-pyrrolidiny

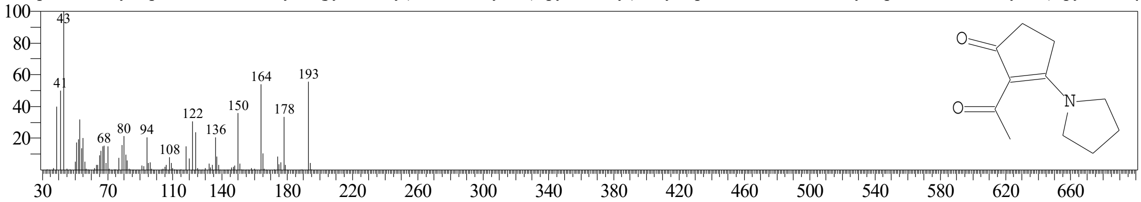

Hit#:3 Entry:101944 Library:NIST11.lib
SI:43 Formula:C17H29NO2 CAS:0-00-0 MolWeight:279 RetIndex:2068
CompName:Z-2-Acetoxy-12-tetradecenitrile $$ (11Z)-1-Cyano-11-tetradecenyl acetate # $$

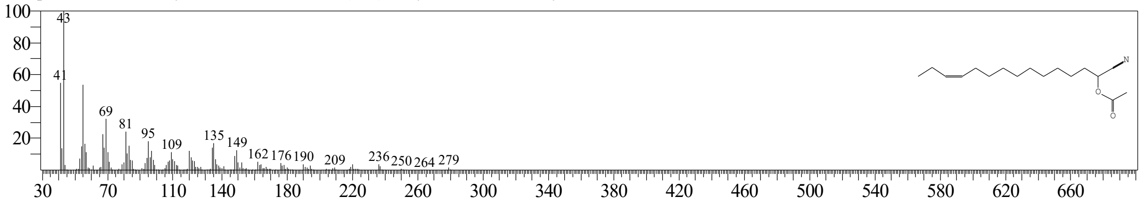

Hit#:4 Entry:23383 Library:NIST11.lib
SI:43 Formula:C10H14O2 CAS:0-00-0 MolWeight:166 RetIndex:1290
CompName:1-Hydroxybicyclo[2.2.2]oct-5-en-2-yl, methyl ketone $$ 1-(1-Hydroxybicyclo[2.2.2]oct-5-en-2-yl)ethanone # $$

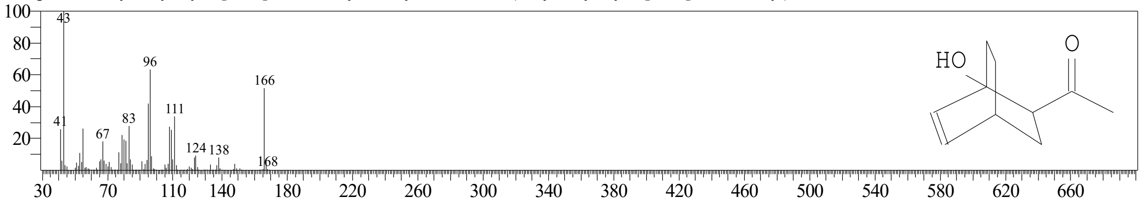

Hit#:5 Entry:168332 Library:NIST11.lib
SI:43 Formula:C18H25N7O2 CAS:0-00-0 MolWeight:371 RetIndex:0
CompName:1-[2-(5-Amino-tetrazol-1-yl)-acetylamino]-cyclohexanecarboxylic acid (2,6-dimethyl-phenyl)-amide $$ 1-([(5-Amino-1H-tetraazol-1-yl)acetyl]amin

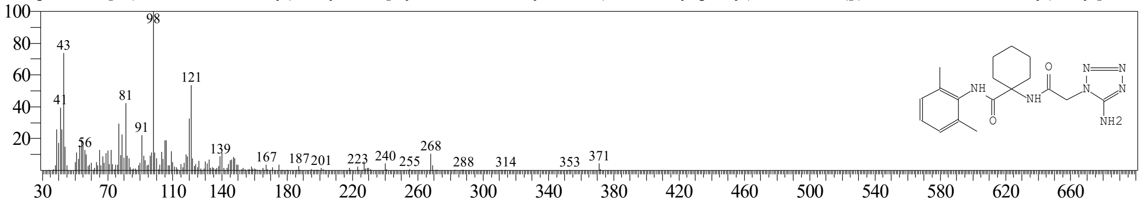

<< Target >>
Line#:118 R.Time:29.000(Scan#:5001) MassPeaks:337
RawMode:Averaged 28.995-29.005(5000-5002) BasePeak:43.05(2982)
BG Mode:Calc. from Peak Group 1 - Event 1 Scan

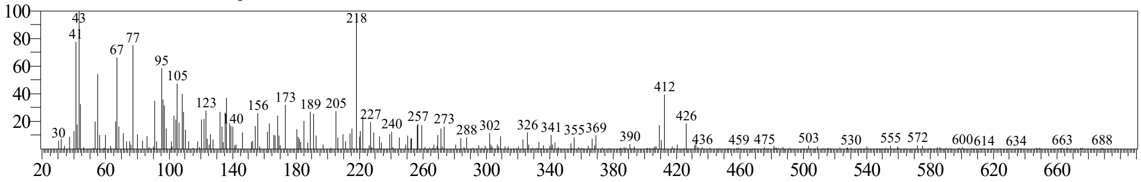

Hit#:1 Entry:191097 Library:NIST11.lib
SI:54 Formula:C30H50O CAS:545-47-1 MolWeight:426 RetIndex:2848
CompName:Lupeol $$ Lup-20(29)-en-3-ol, (3.beta.)- $$ Lup-20(29)-en-3.beta.-ol $$ .beta.-Viscol $$ Clerodol $$ Fagarasterol $$ Fagarsterol $$ Lupenol $$ Mo

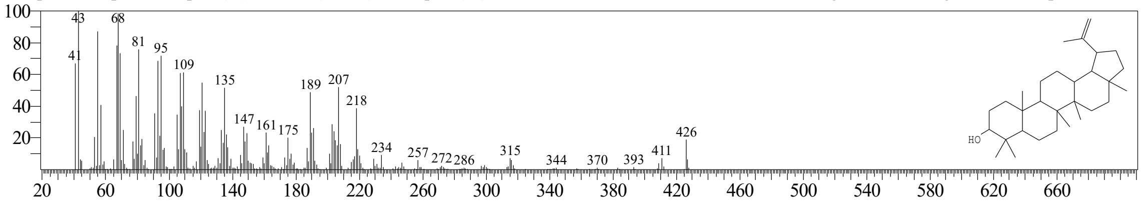

Hit#:2 Entry:109378 Library:NIST11.lib
SI:54 Formula:C21H36 CAS:481-26-5 MolWeight:288 RetIndex:1929
CompName:Pregnane $$ Pregnane, (5.beta.)- $$ 5.beta.-Pregnane $$ 17.beta.-Ethyletiocholane $$

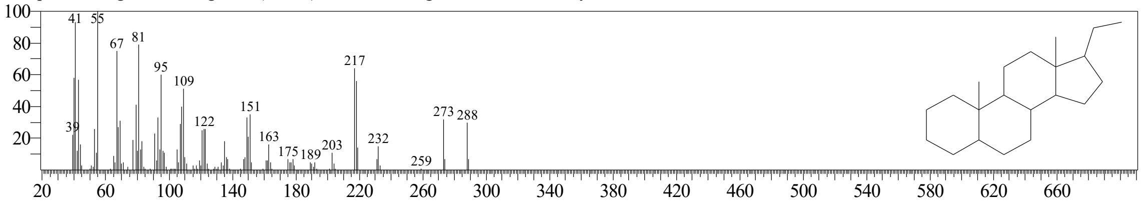

Hit#:3 Entry:56348 Library:NIST11.lib
SI:52 Formula:C15H22O CAS:471-05-6 MolWeight:218 RetIndex:1750
CompName:2,6,10-Cycloundecatrien-1-one, 2,6,9,9-tetramethyl-, (E,E,E)- $$ 2,6,9,9-Tetramethyl-2,6,10-cycloundecatrien-1-one # $$ Zerumbone $$

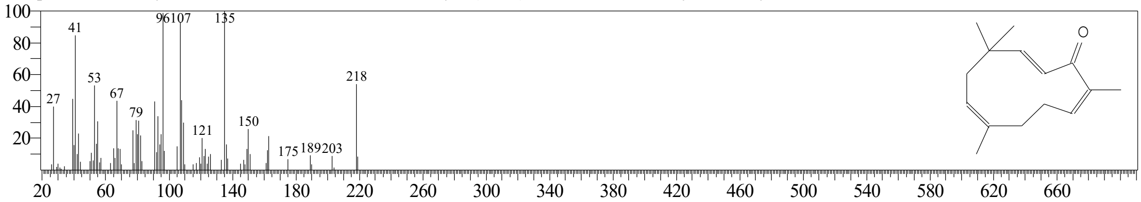

Hit#:4 Entry:204687 Library:NIST11.lib
SI:52 Formula:C28H36O8 CAS:0-00-0 MolWeight:500 RetIndex:3755
CompName:Isodihydrobisvertinol $$ (2E,4E)-1-(8-[(4E)-4-Hexenoyl]-2,4,5a,6,9-pentahydroxy-3,4a,6,9a-tetramethyl-4a,5a,6,7,9a,9b-hexahydrodibenzo[b,d]fur

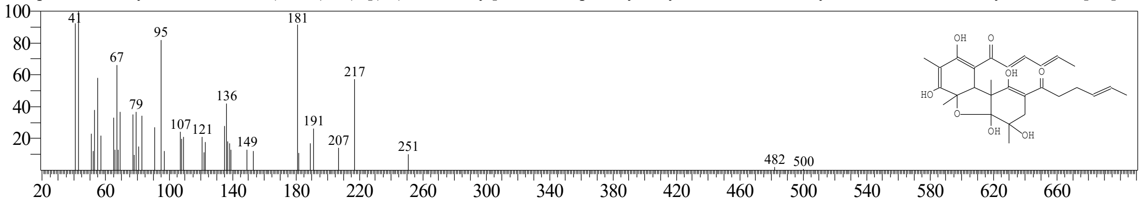

Hit#:5 Entry:101320 Library:NIST11.lib
SI:52 Formula:C17H26O3 CAS:0-00-0 MolWeight:278 RetIndex:2018
CompName:Acetic acid, 3-hydroxy-7-isopropenyl-1,4a-dimethyl-2,3,4,4a,5,6,7,8-octahydronaphthalen-2-yl ester $$ 3-Hydroxy-7-isopropenyl-1,4a-dimethyl-2,3

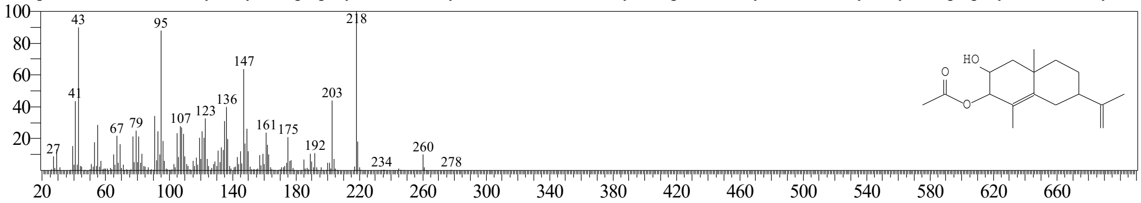

<< Target >>
Line#:119 R.Time:29.075(Scan#:5016) MassPeaks:356
RawMode:Averaged 29.070-29.080(5015-5017) BasePeak:218.20(5544)
BG Mode:Calc. from Peak Group 1 - Event 1 Scan

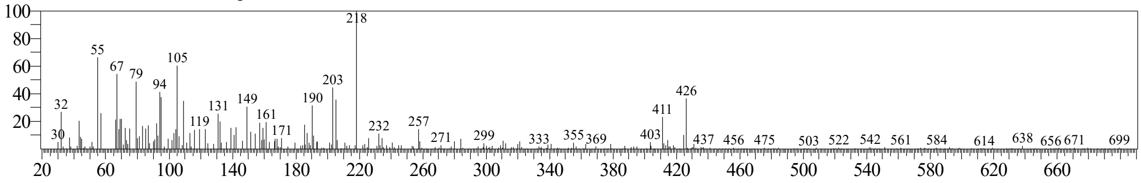

Hit#:1 Entry:190451 Library:NIST11.lib
SI:52 Formula:C30H48O CAS:0-00-0 MolWeight:424 RetIndex:2869
CompName:4,4,6a,6b,8a,11,11,14b-Octamethyl-1,4,4a,5,6,6a,6b,7,8,8a,9,10,11,12,12a,14,14a,14b-octadecahydro-2H-picen-3-one $$ Olean-12-en-3-one # $$

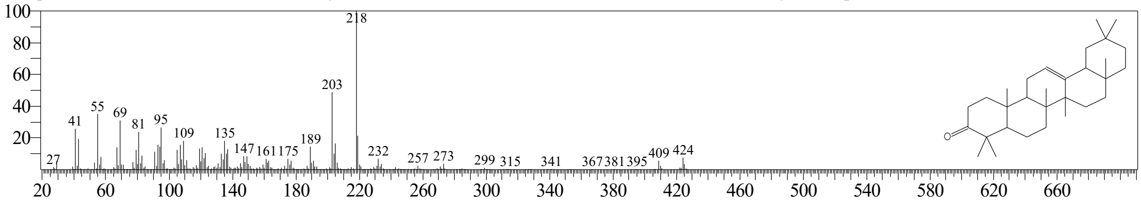

Hit#:2 Entry:191127 Library:NIST11.lib
SI:51 Formula:C30H50O CAS:559-70-6 MolWeight:426 RetIndex:2886
CompName:.beta.-Amyrin $$ Olean-12-en-3-ol, (3.beta.)- $$ Olean-12-en-3.beta.-ol $$ .beta.-Amyrenol $$ Olean-12-en-3-ol # $$

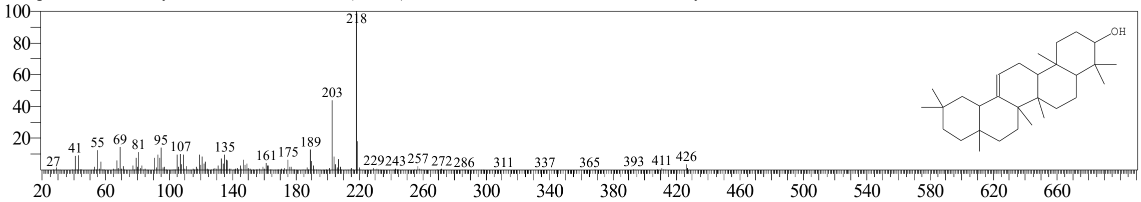

Hit#:3 Entry:186090 Library:NIST11.lib
SI:51 Formula:C30H50 CAS:471-68-1 MolWeight:410 RetIndex:2697
CompName:Olean-12-ene $$ .beta.-Amyrene $$

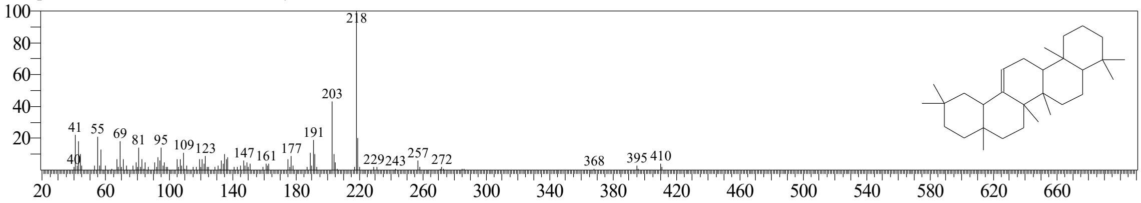

Hit#:4 Entry:191097 Library:NIST11.lib
SI:49 Formula:C30H50O CAS:545-47-1 MolWeight:426 RetIndex:2848
CompName:Lupeol $$ Lup-20(29)-en-3-ol, (3.beta.)- $$ Lup-20(29)-en-3.beta.-ol $$ .beta.-Viscol $$ Clerodol $$ Fagarasterol $$ Fagarsterol $$ Lupenol $$ Mo

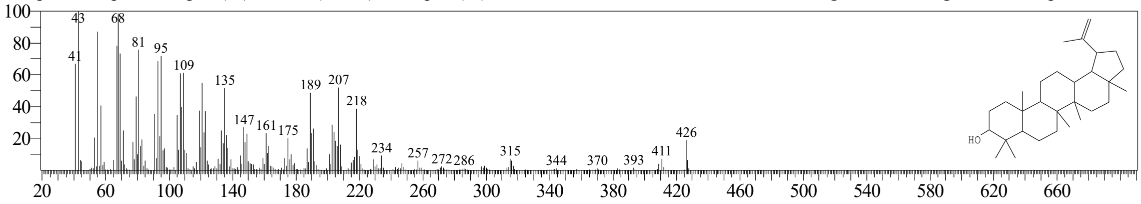

Hit#:5 Entry:186088 Library:NIST11.lib
SI:49 Formula:C30H50 CAS:464-97-1 MolWeight:410 RetIndex:2685
CompName:Urs-12-ene

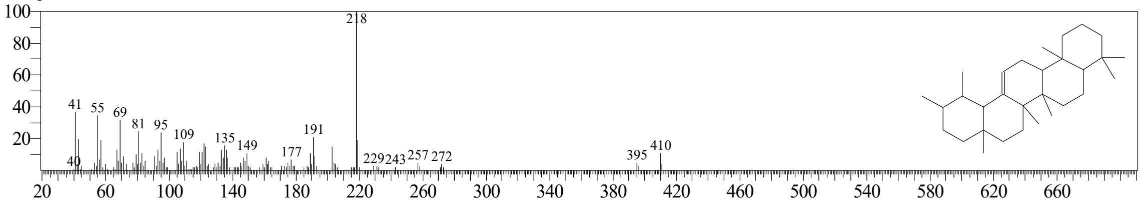

<< Target >>
Line#:120 R.Time:29.115(Scan#:5024) MassPeaks:366
RawMode:Averaged 29.110-29.120(5023-5025) BasePeak:55.05(4381)
BG Mode:Calc. from Peak Group 1 - Event 1 Scan

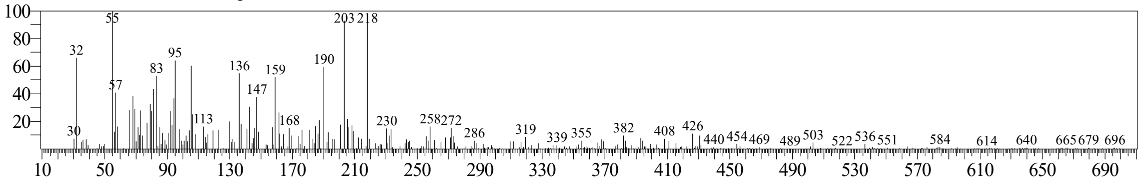

Hit#:1 Entry:192204 Library:NIST11.lib
SI:50 Formula:C28H46O3 CAS:75023-18-6 MolWeight:430 RetIndex:2777
CompName:A-Norcholestane-3-carboxylic acid, 2-oxo-, methyl ester, (3.alpha.)- $$ Methyl 6-(1,5-dimethylhexyl)-3a,5a-dimethyl-2-oxohexadecahydrodicyclop

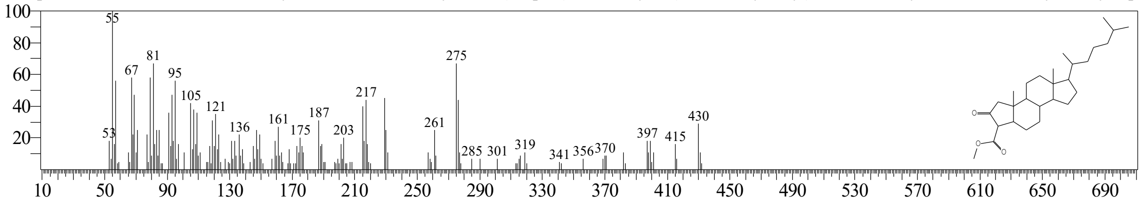

Hit#:2 Entry:191690 Library:NIST11.lib
SI:48 Formula:C30H52O CAS:0-00-0 MolWeight:428 RetIndex:3025
CompName:Ambrein $$ 1-[(3E)-6-(2,2-Dimethyl-6-methylenecyclohexyl)-4-methyl-3-hexenyl]-2,5,5,8a-tetramethyldecahydro-2-naphthalenol # $$

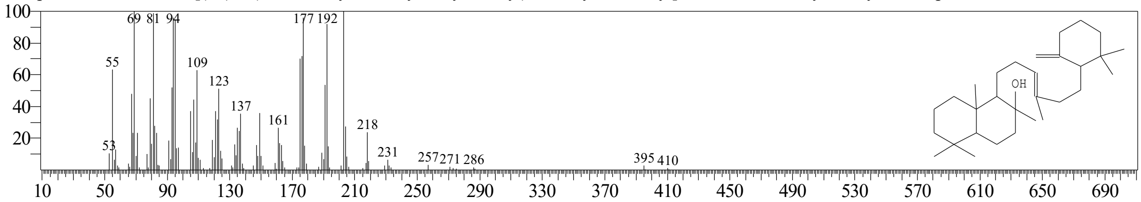

Hit#:3 Entry:17578 Library:NIST11.lib
SI:47 Formula:C10H18O CAS:5502-99-8 MolWeight:154 RetIndex:1250
CompName:Cyclohexaneethanol, 4-methyl-.beta.-methylene- $$ p-Menth-8(10)-en-9-ol $$ 2-(4-Methylcyclohexyl)-2-propen-1-ol # $$

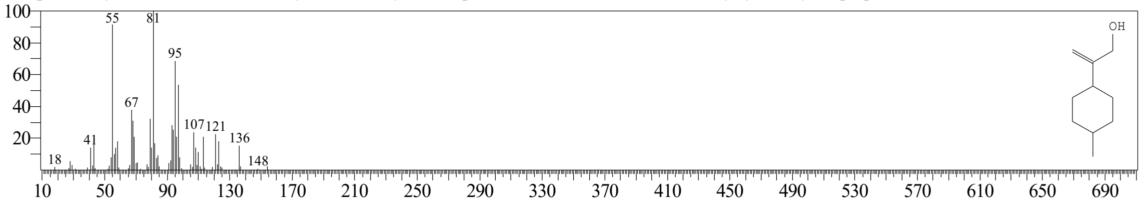

Hit#:4 Entry:56374 Library:NIST11.lib
SI:47 Formula:C15H22O CAS:0-00-0 MolWeight:218 RetIndex:1673
CompName:2(1H)Naphthalenone, 3,5,6,7,8,8a-hexahydro-4,8a-dimethyl-6-(1-methylethenyl)- $$ 6-Isopropenyl-4,8a-dimethyl-3,5,6,7,8,8a-hexahydro-2(1H)-na

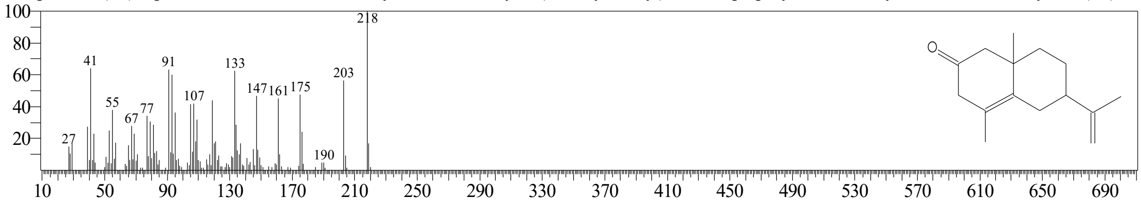

Hit#:5 Entry:86930 Library:NIST11.lib
SI:47 Formula:C12H21BrO CAS:90598-26-8 MolWeight:260 RetIndex:1652
CompName:Cyclohexane, (1-Bromo-3,3-dimethyl-2-hydroxybutylidene)- $$ 1-Bromo-1-cyclohexylidene-3,3-dimethyl-2-butanol # $$

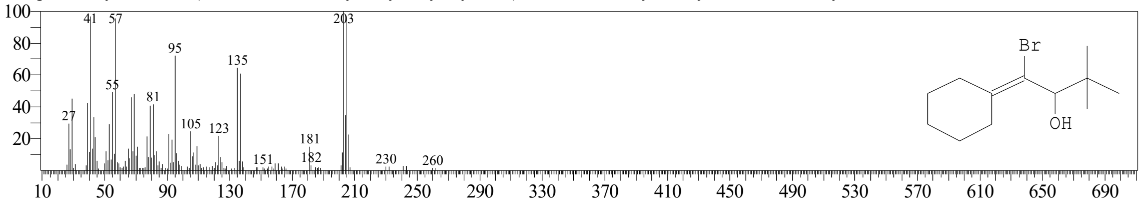

<< Target >>
Line#:121 R.Time:29.275(Scan#:5056) MassPeaks:342
RawMode:Averaged 29.270-29.280(5055-5057) BasePeak:55.10(3193)
BG Mode:Calc. from Peak Group 1 - Event 1 Scan

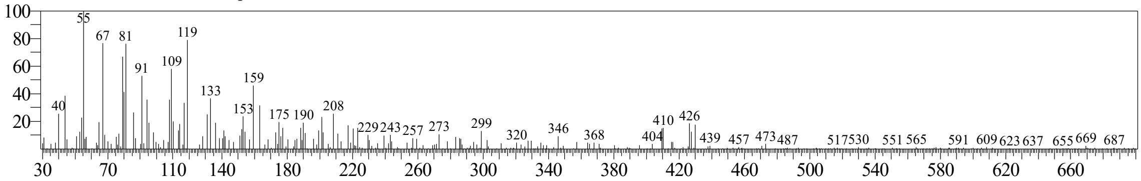

Hit#:1 Entry:169342 Library:NIST11.lib
SI:54 Formula:C25H40O2 CAS:0-00-0 MolWeight:372 RetIndex:2649
CompName:i-Propyl 7,10,13,16,19-docosapentaenoate

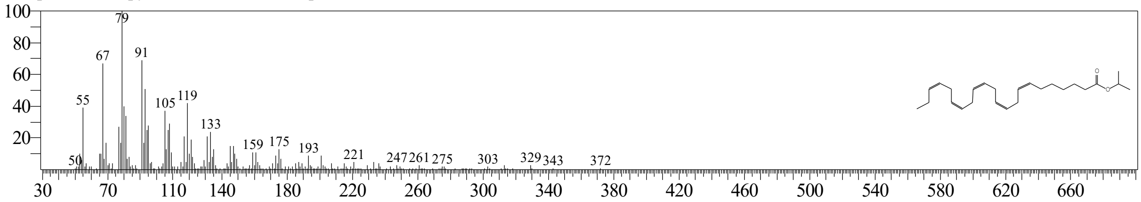

Hit#:2 Entry:151922 Library:NIST11.lib
SI:54 Formula:C23H36O2 CAS:0-00-0 MolWeight:344 RetIndex:2515
CompName:Methyl 7,10,13,16,19-docosapentaenoate

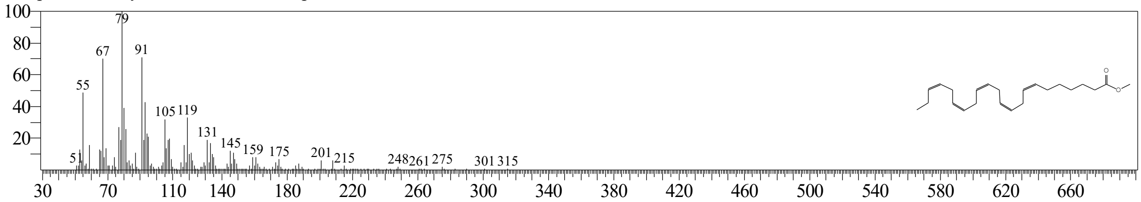

Hit#:3 Entry:47990 Library:NIST11.lib
SI:53 Formula:C14H22O CAS:2221-71-8 MolWeight:206 RetIndex:1622
CompName:2-Naphthalenemethanol, decahydro-5-methylene-8-vinyl- $$ Khusilol, dihydro- $$ (5-Methylene-8-vinyldecahydro-2-naphthalenyl)methanol # $$

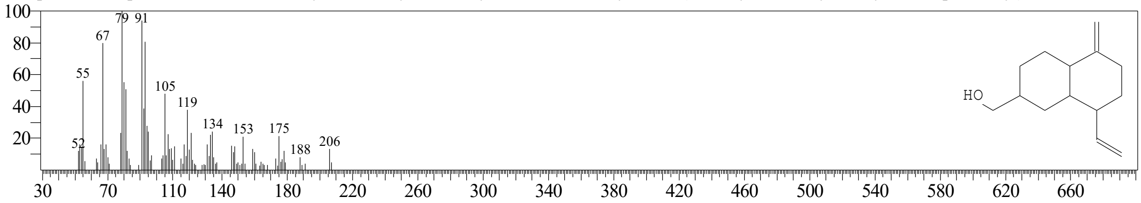

Hit#:4 Entry:112443 Library:NIST11.lib
SI:53 Formula:C19H32O2 CAS:0-00-0 MolWeight:292 RetIndex:2101
CompName:Methyl 5,9,12-octadecatrienoate

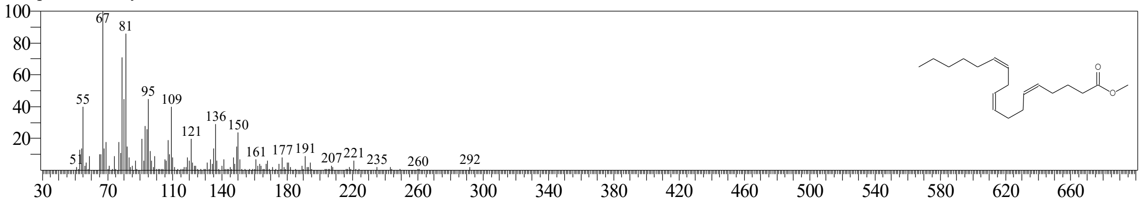

Hit#:5 Entry:159304 Library:NIST11.lib
SI:53 Formula:C20H24N2O4 CAS:321142-70-5 MolWeight:356 RetIndex:3176
CompName:Bicyclo[10.1.0]trideca-4,8-diene-13-carboxylic acid (2-hydroxy-4-nitrophenyl)amide $$ N-(2-Hydroxy-4-nitrophenyl)bicyclo[10.1.0]trideca-4,8-di

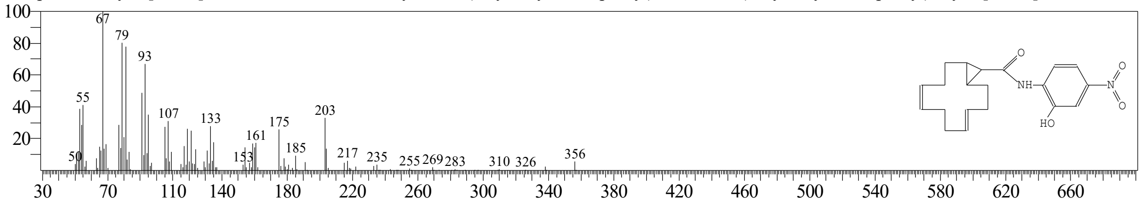

<< Target >>
Line#:122 R.Time:30.650(Scan#:5331) MassPeaks:374
RawMode:Averaged 30.645-30.655(5330-5332) BasePeak:203.25(3446)
BG Mode:Calc. from Peak Group 1 - Event 1 Scan

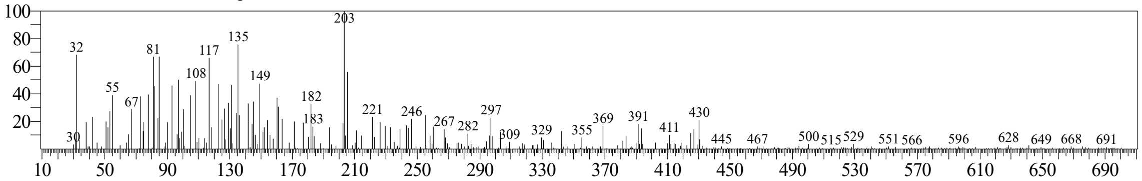

Hit#:1 Entry:197851 Library:NIST11.lib
SI:43 Formula:C31H50O2 CAS:24759-09-9 MolWeight:454 RetIndex:3081
CompName:C(14a)-Homo-27-norgammacer-13-en-21-one, 3-methoxy-, (3.alpha.)- $$ C(14a)-Homo-27-norgammacer-13-en-21-one, 3.alpha.-methoxy- $$

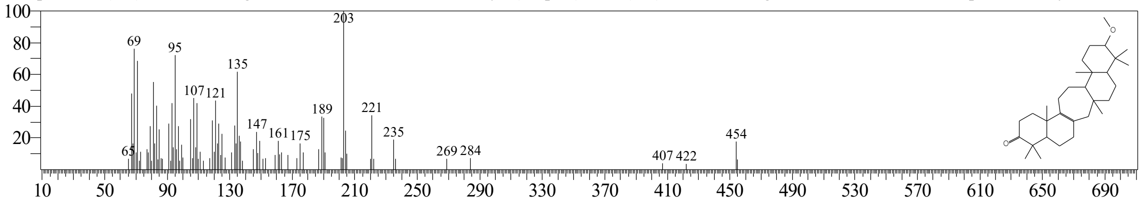

Hit#:2 Entry:30460 Library:NIST11.lib
SI:42 Formula:C12H18O CAS:174226-46-1 MolWeight:178 RetIndex:1159
CompName:9-Methyl-10-methylenetricyclo[4.2.1.1(2,5)]decan-9-ol

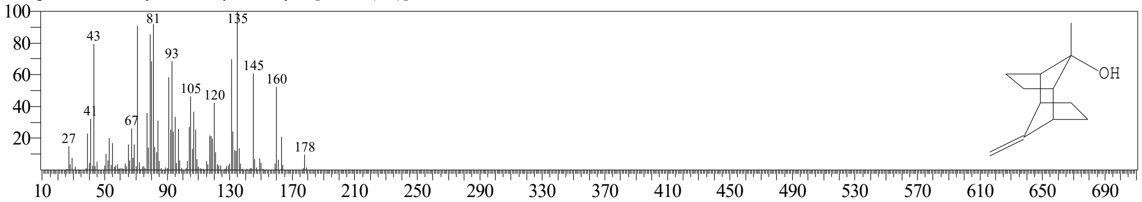

Hit#:3 Entry:69426 Library:NIST11.lib
SI:41 Formula:C16H28O CAS:137235-47-3 MolWeight:236 RetIndex:1721
CompName:2-Hydroxymethyl-2,6,8,8-tetramethyltricyclo[5.2.2.0(1,6)]undecane

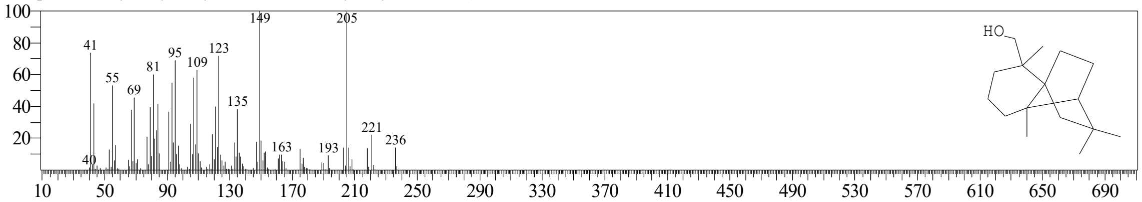

Hit#:4 Entry:172389 Library:NIST11.lib
SI:40 Formula:C23H42O2Si CAS:0-00-0 MolWeight:378 RetIndex:2409
CompName:cis-5,8,11-Eicosatrienoic acid, trimethylsilyl ester

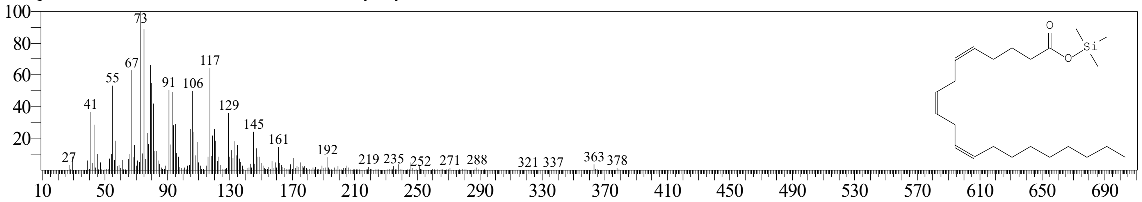

Hit#:5 Entry:184166 Library:NIST11.lib
SI:39 Formula:C25H44O2Si CAS:0-00-0 MolWeight:404 RetIndex:2616
CompName:cis-7,10,13,16-Docosatetraenoic acid, trimethylsilyl ester

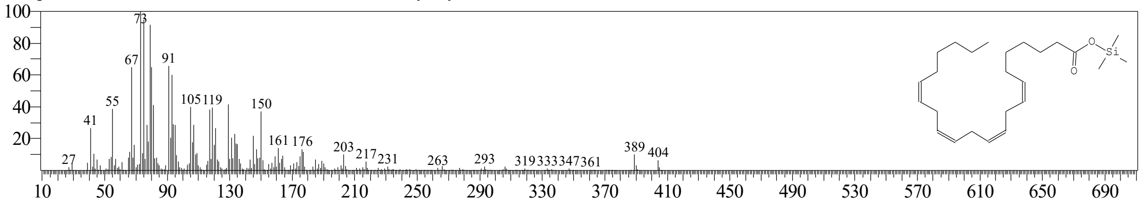

Qualitative Analysis Report

<< Target >>
Line#:123 R.Time:30.665(Scan#:5334) MassPeaks:332
RawMode:Averaged 30.660-30.670(5333-5335) BasePeak:93.00(2768)
BG Mode:Calc. from Peak Group 1 - Event 1 Scan

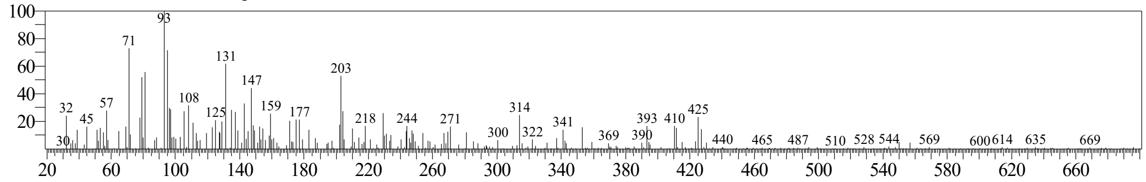

Hit#:1 Entry:197851 Library:NIST11.lib
SI:49 Formula:C31H50O2 CAS:24759-09-9 MolWeight:454 RetIndex:3081
CompName:C(14a)-Homo-27-norgammacer-13-en-21-one, 3-methoxy-, (3.alpha.)- $$ C(14a)-Homo-27-norgammacer-13-en-21-one, 3.alpha.-methoxy- $$

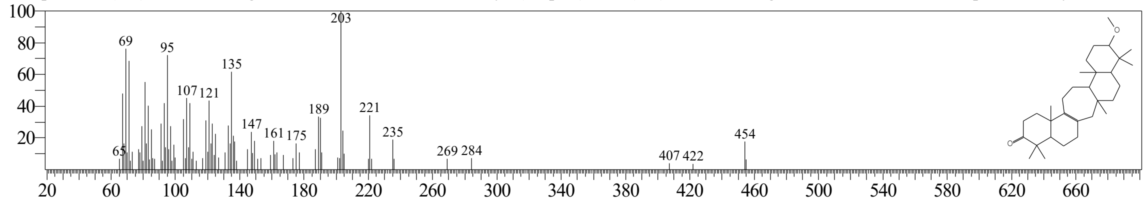

Hit#:2 Entry:198232 Library:NIST11.lib
SI:47 Formula:C31H52O2 CAS:24433-34-9 MolWeight:456 RetIndex:3097
CompName:C(14a)-Homo-27-norgammacer-13-en-21-ol, 3-methoxy-, (3.alpha.,21.beta.)- $$ C(14a)-Homo-27-norgammacer-13-en-21.beta.-ol, 3.alpha.-methox

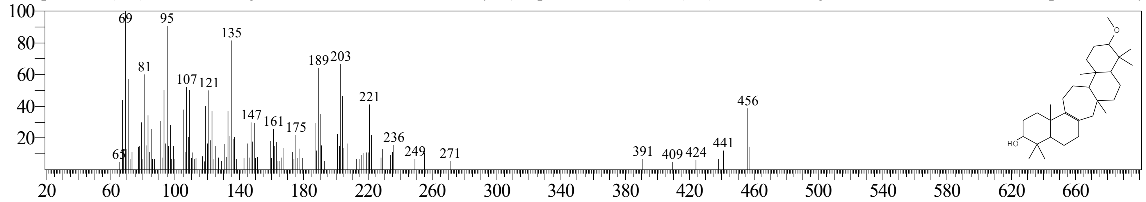

Hit#:3 Entry:197367 Library:NIST11.lib
SI:46 Formula:C33H56 CAS:0-00-0 MolWeight:452 RetIndex:2885
CompName:1,1,6-trimethyl-3-methylene-2-(3,6,9,13-tetramethyl-6-ethenye-10,14-dimethylene-pentadec-4-enyl)cyclohexane

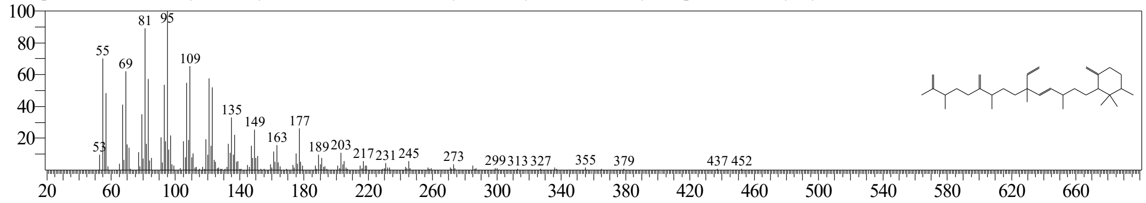

Hit#:4 Entry:65974 Library:NIST11.lib
SI:46 Formula:C10H17BrO CAS:82252-98-0 MolWeight:232 RetIndex:1434
CompName:Bicyclo[4.1.0]heptane-7-methanol, 7-bromo-.alpha.-ethyl-, (1.alpha.,6.alpha.,7.alpha.)- $$ 1-(7-Bromobicyclo[4.1.0]hept-7-yl)-1-propanol # $$

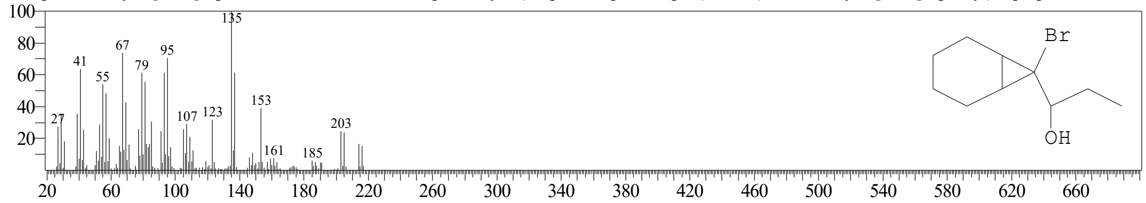

Hit#:5 Entry:195292 Library:NIST11.lib
SI:45 Formula:C32H58 CAS:0-00-0 MolWeight:442 RetIndex:2788
CompName:1,1,6-trimethyl-3-methylene-2-(3,6,10,13,14-pentamethyl-3-ethenyl-pentadec-4-enye)cyclohexane

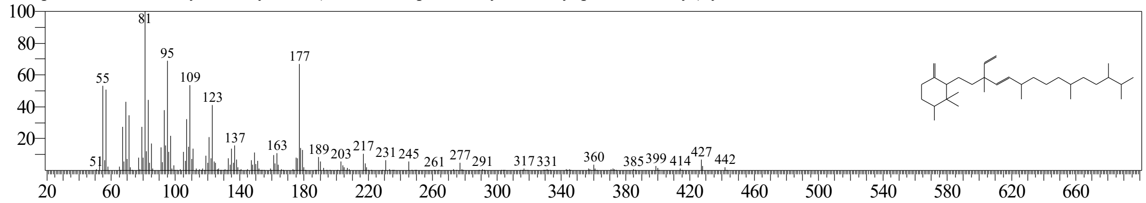

<< Target >>
Line#:124 R.Time:30.775(Scan#:5356) MassPeaks:378
RawMode:Averaged 30.770-30.780(5355-5357) BasePeak:71.05(5285)
BG Mode:Calc. from Peak Group 1 - Event 1 Scan

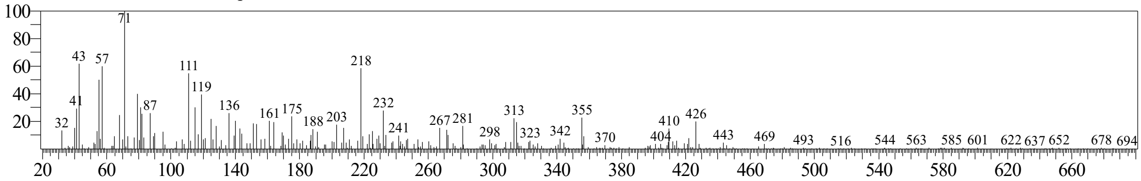

Hit#:1 Entry:203987 Library:NIST11.lib
SI:45 Formula:C29H47ClO4 CAS:0-00-0 MolWeight:494 RetIndex:3410
CompName:Dimethylmalonic acid, 4-chlorophenyl octadecyl ester

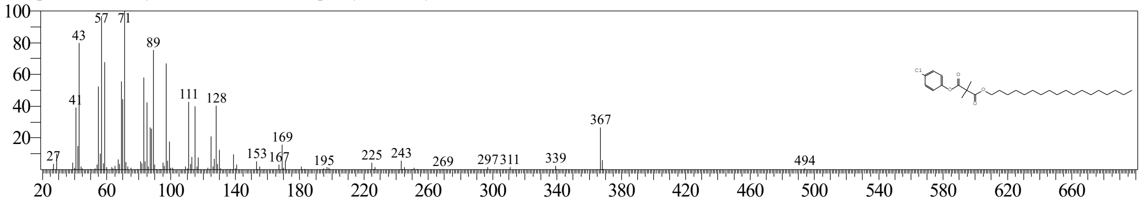

Hit#:2 Entry:199856 Library:NIST11.lib
SI:45 Formula:C27H43ClO4 CAS:0-00-0 MolWeight:466 RetIndex:3212
CompName:Dimethylmalonic acid, 4-chlorophenyl hexadecyl ester

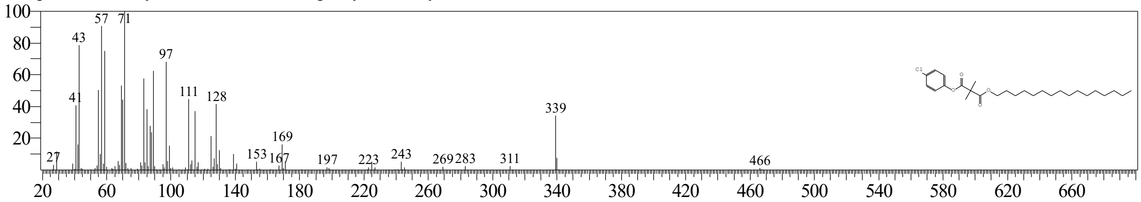

Hit#:3 Entry:202083 Library:NIST11.lib
SI:45 Formula:C28H45ClO4 CAS:0-00-0 MolWeight:480 RetIndex:3311
CompName:Dimethylmalonic acid, 4-chlorophenyl heptadecyl ester

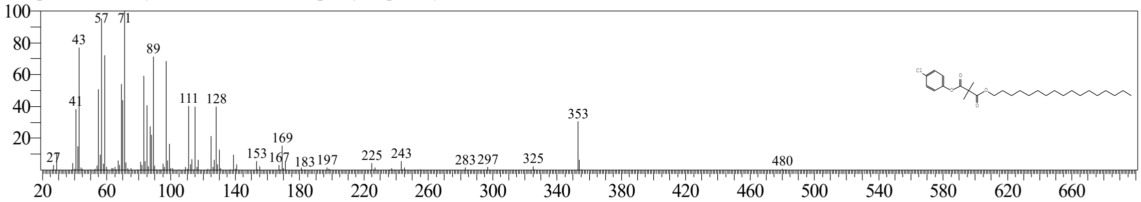

Hit#:4 Entry:42699 Library:NIST11.lib
SI:45 Formula:C12H22O2 CAS:16491-54-6 MolWeight:198 RetIndex:1307
CompName:Butanoic acid, 1-ethenylhexyl ester $$ Amylvinylcarbinyl butyrate $$ 1-Octen-3-yl butyrate $$ Butyric acid, 1-vinylhexyl ester $$ 1-Octen-3-ol buty

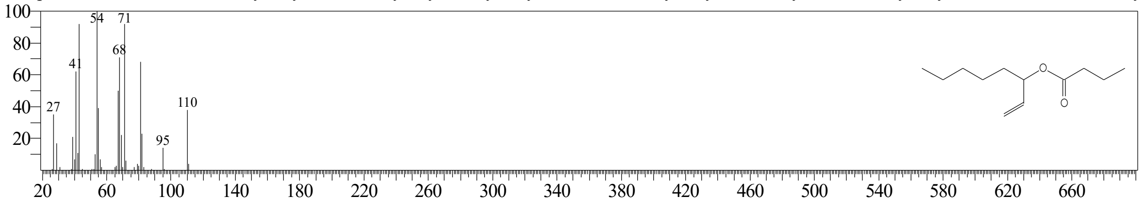

Hit#:5 Entry:96638 Library:NIST11.lib
SI:45 Formula:C15H28O4 CAS:0-00-0 MolWeight:272 RetIndex:1698
CompName:Dimethylmalonic acid, ethyl 2-octyl ester

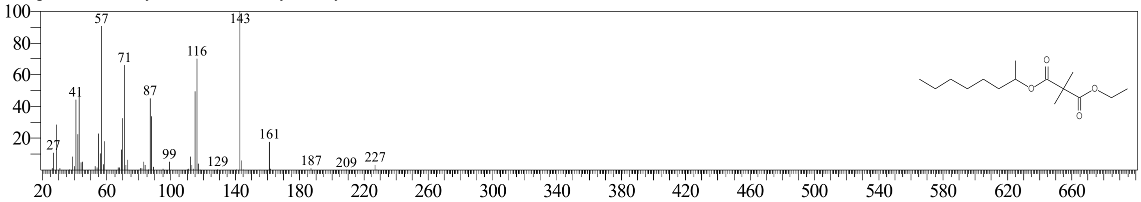

<< Target >>
Line#:125 R.Time:30.995(Scan#:5400) MassPeaks:387
RawMode:Averaged 30.990-31.000(5399-5401) BasePeak:43.05(78385)
BG Mode:Calc. from Peak Group 1 - Event 1 Scan

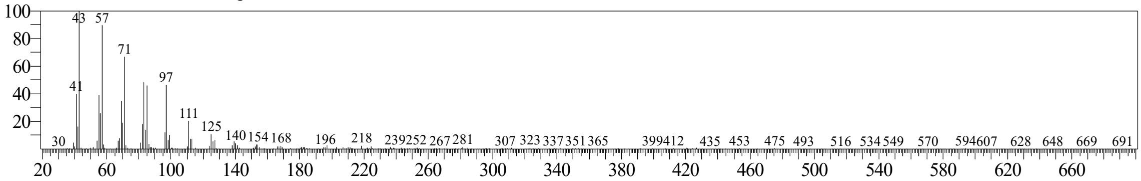

Hit#:1 Entry:117235 Library:NIST11.lib
SI:93 Formula:C20H42O CAS:5333-42-6 MolWeight:298 RetIndex:2188
CompName:1-Dodecanol, 2-octyl- $$ Eutanol G $$ Standamul G $$ 2-Octyl-1-dodecanol $$ 2-Octyldecanol $$ 2-Octyldodecanol $$ Octyldodecanol $$ 2-Octy

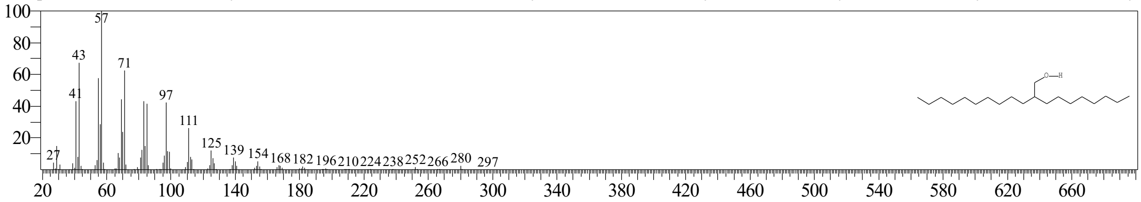

Hit#:2 Entry:95295 Library:NIST11.lib
SI:92 Formula:C18H38O CAS:110225-00-8 MolWeight:270 RetIndex:1989
CompName:1-Dodecanol, 2-hexyl- $$ 2-Hexyldodecan-1-ol $$ 2-Hexyldodecanol $$ 2-Hexyldodecyl alcohol $$ Eutanol G 16 $$

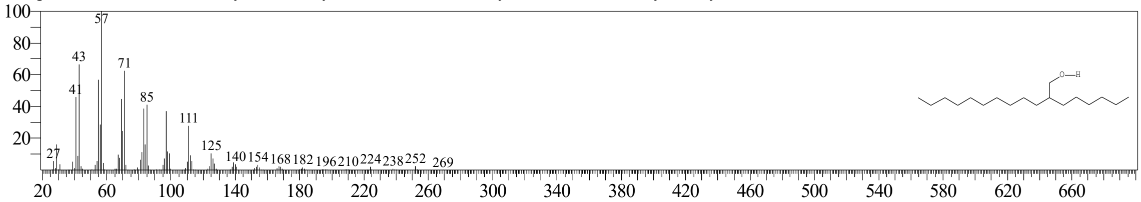

Hit#:3 Entry:95294 Library:NIST11.lib
SI:92 Formula:C18H38O CAS:45235-48-1 MolWeight:270 RetIndex:1989
CompName:1-Decanol, 2-octyl- $$ 2-Octyldecan-1-ol $$ Guerbet C18 Alcohol $$

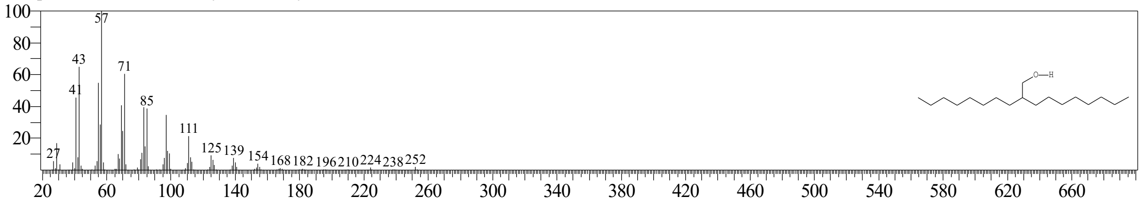

Hit#:4 Entry:211602 Library:NIST11.lib
SI:92 Formula:C39H73F5O2 CAS:0-00-0 MolWeight:668 RetIndex:3761
CompName:Hexatriacontyl pentafluoropropionate $$ Hexatriacontyl 2,2,3,3,3-pentafluoropropanoate $$ 1-Hexatriacontanol, pentafluoropropionate $$

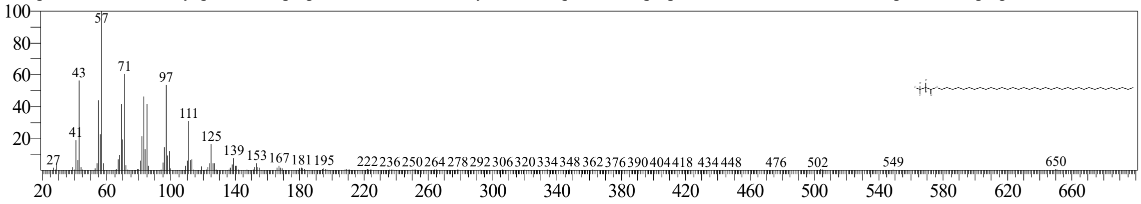

Hit#:5 Entry:211167 Library:NIST11.lib
SI:92 Formula:C37H69F5O2 CAS:0-00-0 MolWeight:640 RetIndex:3562
CompName:Tetratriacontyl pentafluoropropionate $$ Tetratriacontyl 2,2,3,3,3-pentafluoropropanoate $$ 1-Tetratriacontanol, pentafluoropropionate $$

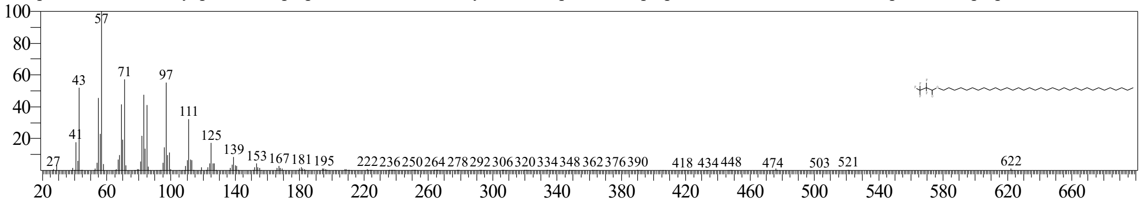

<< Target >>
Line#:126 R.Time:31.205(Scan#:5442) MassPeaks:332
RawMode:Averaged 31.200-31.210(5441-5443) BasePeak:69.10(2083)
BG Mode:Calc. from Peak Group 1 - Event 1 Scan

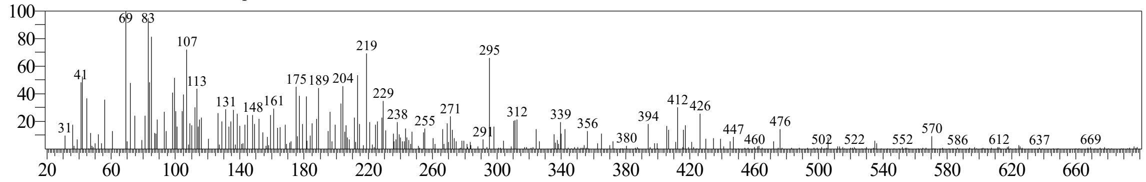

Hit#:1 Entry:167753 Library:NIST11.lib
SI:40 Formula:C21H38O5 CAS:0-00-0 MolWeight:370 RetIndex:2463
CompName:Fumaric acid, decyl 5-methoxy-3-methylpentyl ester

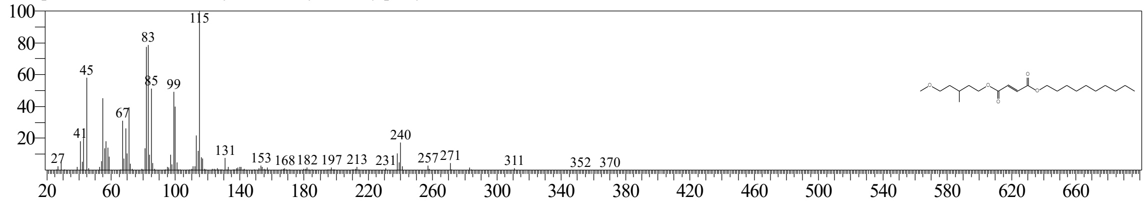

Hit#:2 Entry:111875 Library:NIST11.lib
SI:40 Formula:C14H25ClO4 CAS:0-00-0 MolWeight:292 RetIndex:1820
CompName:Diethylmalonic acid, monochloride, 5-methoxy-3-methylpentyl ester

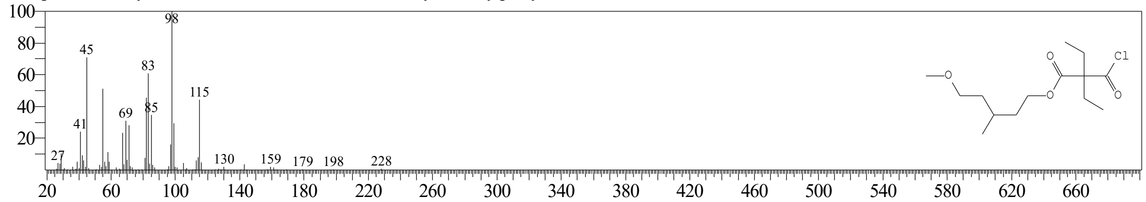

Hit#:3 Entry:114086 Library:NIST11.lib
SI:39 Formula:C12H10F5NO2 CAS:0-00-0 MolWeight:295 RetIndex:1565
CompName:2-Methyltetrahydrofuran-3-one oxime, o-[(pentafluorophenyl)methyl]- $$ (3E)-2-Methyldihydro-3(2H)-furanone o-(2,3,4,5,6-pentafluorobenzyl)ox

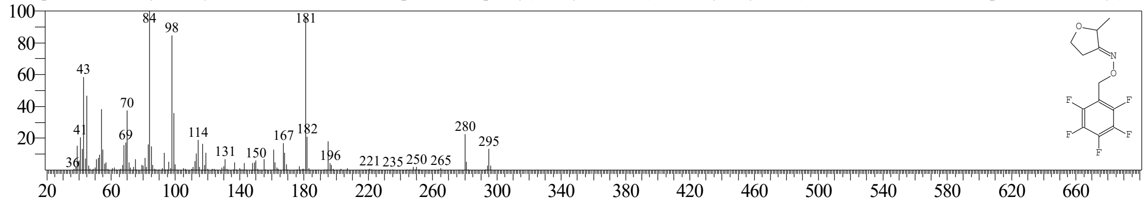

Hit#:4 Entry:134578 Library:NIST11.lib
SI:39 Formula:C15H16F5NO CAS:0-00-0 MolWeight:321 RetIndex:1717
CompName:6-Methyl-5-hepten-2-one oxime, o-[(pentafluorophenyl)methyl]- $$ (2E)-6-Methyl-5-hepten-2-one o-(2,3,4,5,6-pentafluorobenzyl)oxime # $$

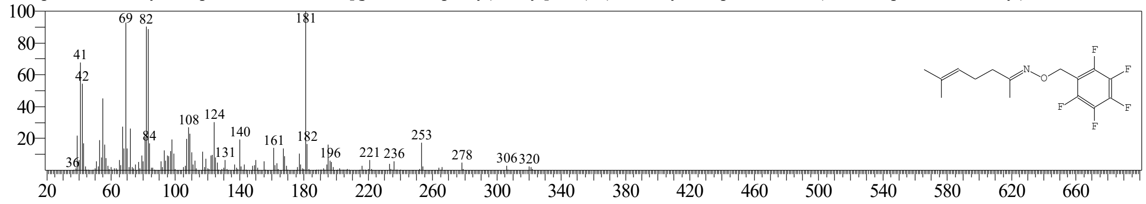

Hit#:5 Entry:121379 Library:NIST11.lib
SI:39 Formula:C15H25ClO4 CAS:0-00-0 MolWeight:304 RetIndex:1996
CompName:Fumaric acid, 2-chloropropyl octyl ester

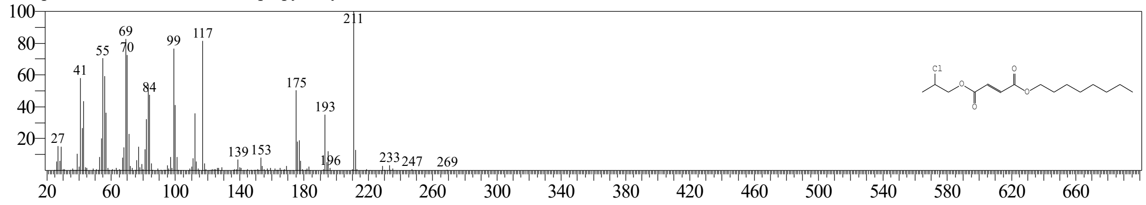

<< Target >>
Line#:127 R.Time:31.260(Scan#:5453) MassPeaks:361
RawMode:Averaged 31.255-31.265(5452-5454) BasePeak:43.05(5407)
BG Mode:Calc. from Peak Group 1 - Event 1 Scan

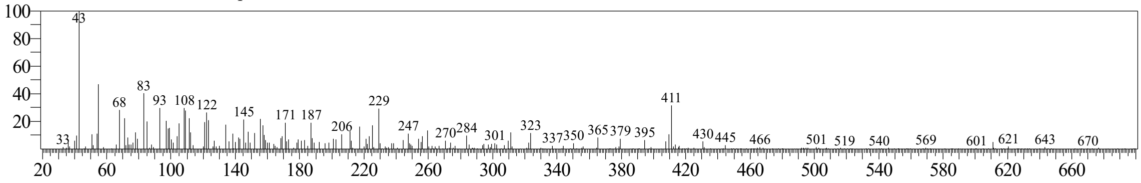

Hit#:1 Entry:23578 Library:NIST11.lib
SI:49 Formula:C11H18O CAS:0-00-0 MolWeight:166 RetIndex:1230
CompName:5-Isopropenyl-1,2-dimethylcyclohex-2-enol $$ 5-Isopropenyl-1,2-dimethyl-2-cyclohexen-1-ol # $$

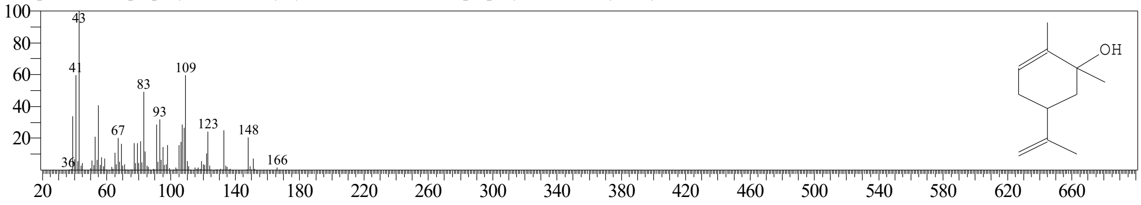

Hit#:2 Entry:57305 Library:NIST11.lib
SI:49 Formula:C11H21ClO2 CAS:67317-62-8 MolWeight:220 RetIndex:1507
CompName:Chloromethyl decanoate $$ Decanoic acid, chloromethyl ester $$

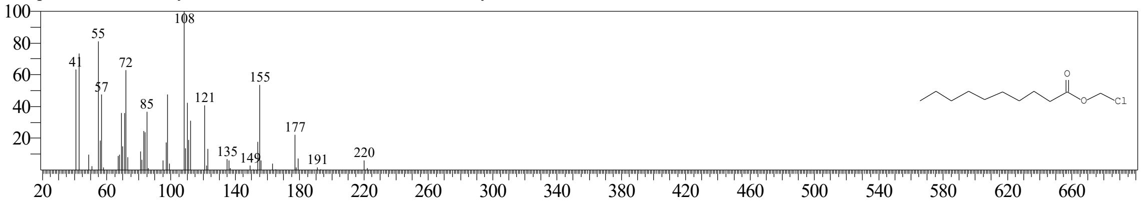

Hit#:3 Entry:17461 Library:NIST11.lib
SI:49 Formula:C10H18O CAS:64165-21-5 MolWeight:154 RetIndex:1072
CompName:5-Octen-2-one, 3,6-dimethyl- $$ (5E)-3,6-Dimethyl-5-octen-2-one # $$

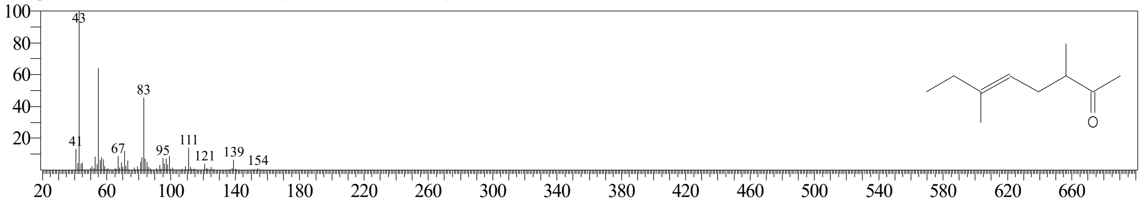

Hit#:4 Entry:16467 Library:NIST11.lib
SI:48 Formula:C10H16O CAS:54703-57-0 MolWeight:152 RetIndex:1238
CompName:Ethanone, 1-(4-cycloocten-1-yl)- $$ 1-(4-Cycloocten-1-yl)ethanone # $$

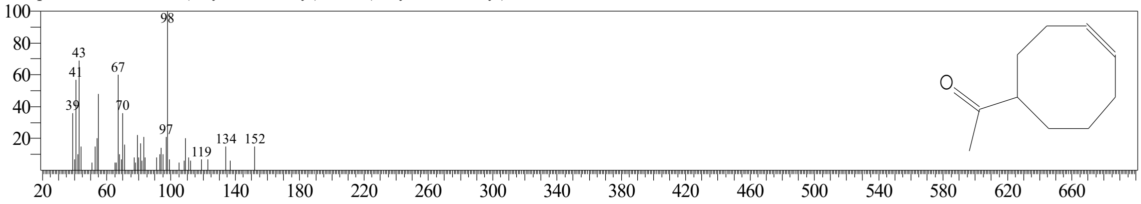

Hit#:5 Entry:150042 Library:NIST11.lib
SI:48 Formula:C18H30O6 CAS:102175-50-8 MolWeight:342 RetIndex:2422
CompName:Cyclododecane, 1,5,9-tris(acetoxy)- $$ 5,9-Bis(acetyloxy)cyclododecyl acetate # $$

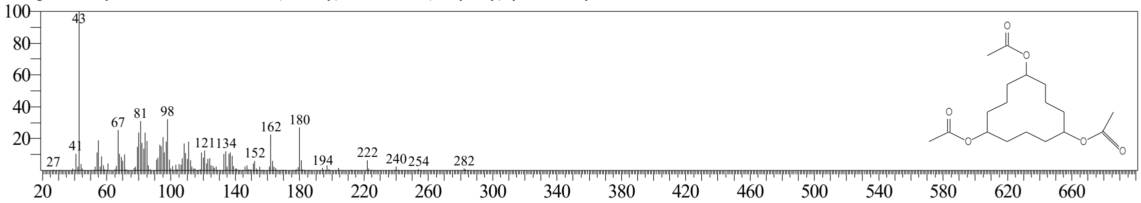

<< Target >>
Line#:128 R.Time:31.375(Scan#:5476) MassPeaks:364
RawMode:Averaged 31.370-31.380(5475-5477) BasePeak:67.00(3571)
BG Mode:Calc. from Peak Group 1 - Event 1 Scan

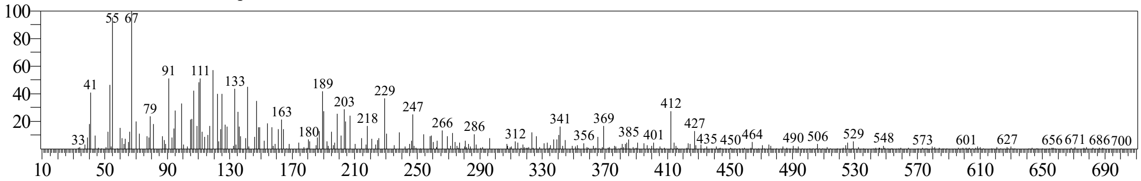

Hit#:1 Entry:46704 Library:NIST11.lib
SI:59 Formula:C15H24 CAS:489-83-8 MolWeight:204 RetIndex:1497
CompName:Guaia-3,9-diene $$ 5-Isopropyl-3,8-dimethyl-1,2,4,5,6,7-hexahydroazulene # $$

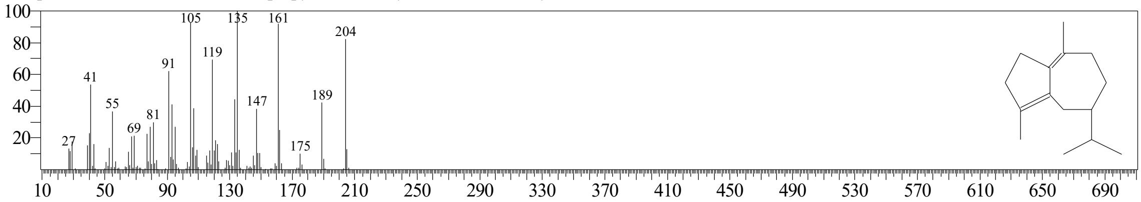

Hit#:2 Entry:46655 Library:NIST11.lib
SI:58 Formula:C15H24 CAS:0-00-0 MolWeight:204 RetIndex:1528
CompName:Caryophyllene-(I1) $$ 2,6,10,10-Tetramethylbicyclo[7.2.0]undeca-1,6-diene # $$

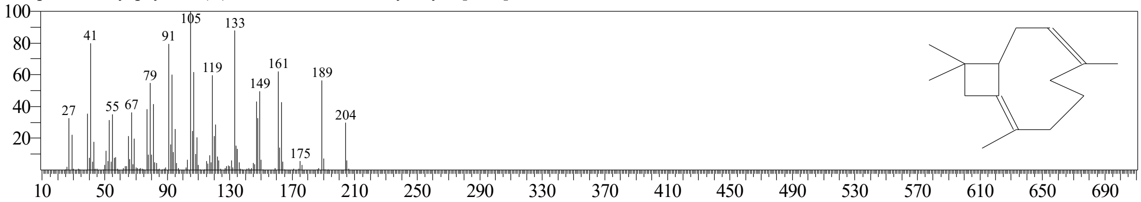

Hit#:3 Entry:46591 Library:NIST11.lib
SI:57 Formula:C15H24 CAS:54832-80-3 MolWeight:204 RetIndex:1270
CompName:Tricyclo[4.3.0.0(7,9)]non-3-ene, 2,2,5,5,8,8-hexamethyl-, (1.alpha.,6.beta.,7.alpha.,9.alpha.)-

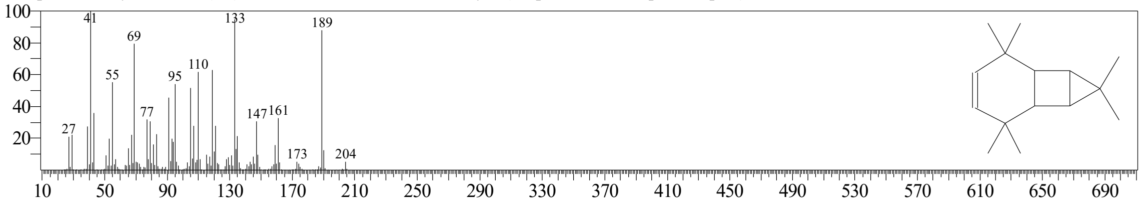

Hit#:4 Entry:46770 Library:NIST11.lib
SI:57 Formula:C15H24 CAS:0-00-0 MolWeight:204 RetIndex:1443
CompName:1,1,4a-Trimethyl-5,6-dimethylenedecahydronaphthalene

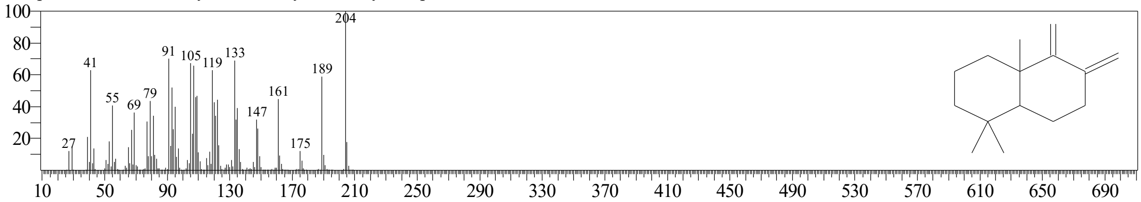

Hit#:5 Entry:46773 Library:NIST11.lib
SI:57 Formula:C15H24 CAS:489-40-7 MolWeight:204 RetIndex:1419
CompName:1H-Cycloprop[e]azulene, 1a,2,3,4,4a,5,6,7b-octahydro-1,1,4,7-tetramethyl-, [1aR-(1a.alpha.,4.alpha.,4a.beta.,7b.alpha.)]- $$ 1H-Cycloprop[e]azule

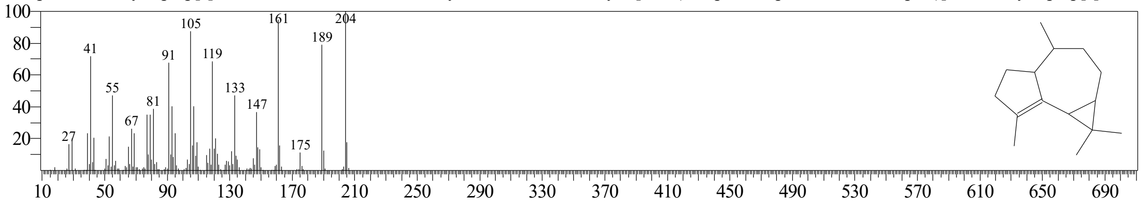

<< Target >>
Line#:129 R.Time:33.170(Scan#:5835) MassPeaks:378
RawMode:Averaged 33.165-33.175(5834-5836) BasePeak:71.10(4644)
BG Mode:Calc. from Peak Group 1 - Event 1 Scan

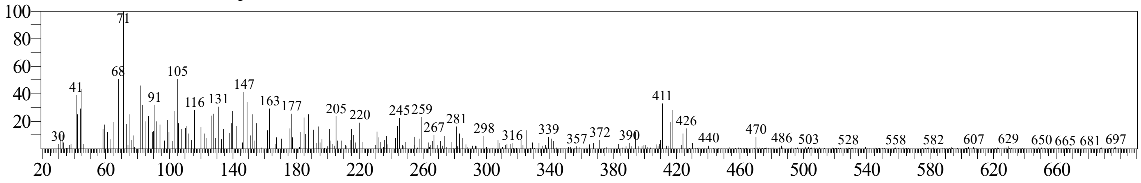

Hit#:1 Entry:206731 Library:NIST11.lib
SI:43 Formula:C24H45N5O4Si2 CAS:0-00-0 MolWeight:523 RetIndex:3179
CompName:Adenosine, 2',3'-O-di-tert-butyldimethylsilyl-5',5'-dimethyl-

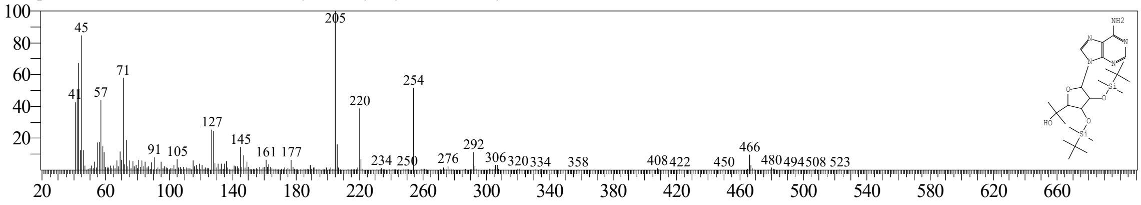

Hit#:2 Entry:115616 Library:NIST11.lib
SI:41 Formula:C11H15N5O5 CAS:2140-71-8 MolWeight:297 RetIndex:2758
CompName:Guanosine, 2'-O-methyl- $$ 2'-O-Methylguanosine $$

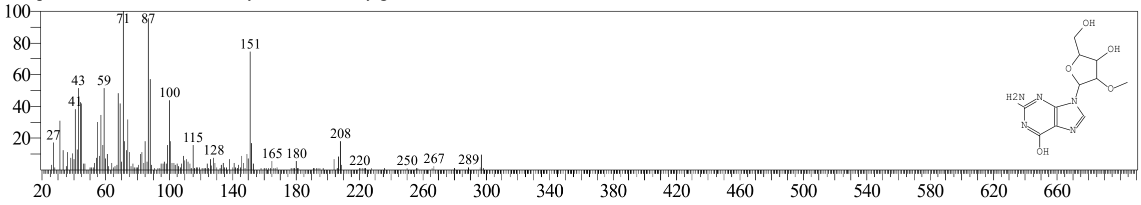

Hit#:3 Entry:39435 Library:NIST11.lib
SI:41 Formula:C7H14O6 CAS:0-00-0 MolWeight:194 RetIndex:1667
CompName:.alpha.-Methyl mannofuranoside $$ Methyl hexofuranoside # $$

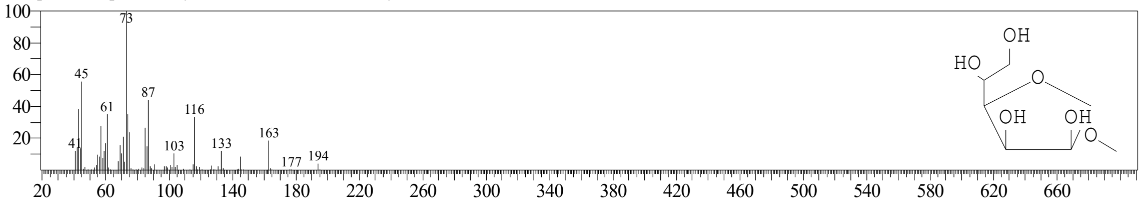

Hit#:4 Entry:12095 Library:NIST11.lib
SI:40 Formula:C8H14O2 CAS:17429-01-5 MolWeight:142 RetIndex:1148
CompName:Cycloheptanone, 4-methoxy- $$ 4-Methoxycycloheptanone # $$

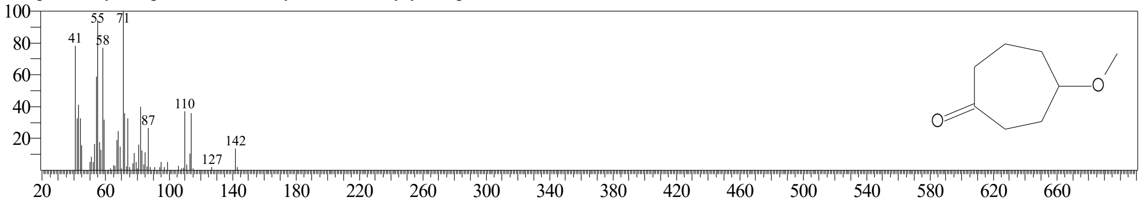

Hit#:5 Entry:65893 Library:NIST11.lib
SI:40 Formula:C9H17BO6 CAS:0-00-0 MolWeight:232 RetIndex:0
CompName:.beta.-d-Mannofuranoside, methyl-2,3-O-(ethylboranediyl)- $$ 1-(2-Ethyl-6-methoxytetrahydrofuro[3,4-d][1,3,2]dioxaborol-4-yl)-1,2-ethanediol #

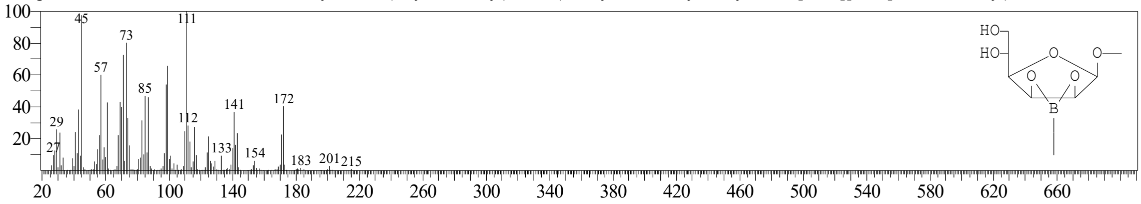

Qualitative Analysis Report

<< Target >>
Line#:130 R.Time:33.235(Scan#:5848) MassPeaks:379
RawMode:Averaged 33.230-33.240(5847-5849) BasePeak:82.05(5879)
BG Mode:Calc. from Peak Group 1 - Event 1 Scan

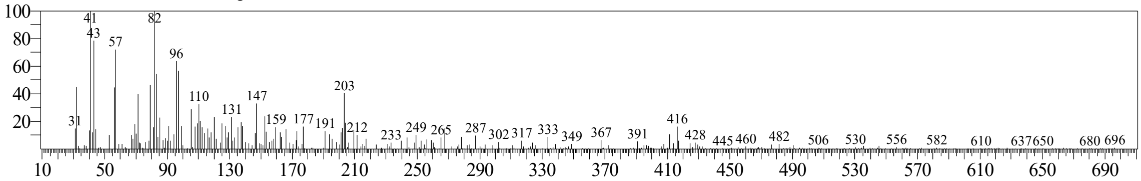

Hit#:1 Entry:40780 Library:NIST11.lib
SI:62 Formula:C13H25N CAS:629-60-7 MolWeight:195 RetIndex:1559
CompName:Tridecanenitrile $$ Dodecyl cyanide $$ n-Dodecyl cyanide $$ 1-Cyanododecane $$ Tridecanonitrile $$ Tridecane nitrile $$

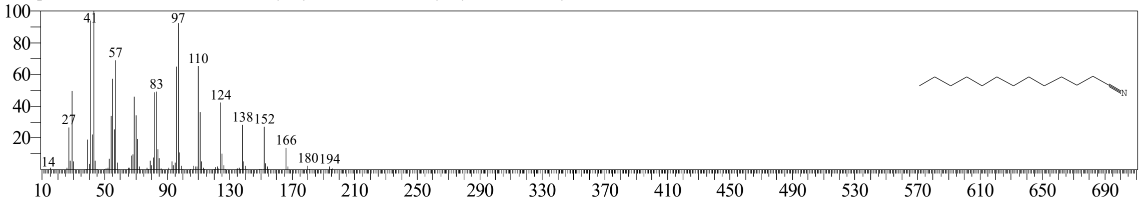

Hit#:2 Entry:50097 Library:NIST11.lib
SI:62 Formula:C14H27N CAS:629-63-0 MolWeight:209 RetIndex:1658
CompName:Tetradecanenitrile $$ Myristonitrile $$ 1-Cyanotridecane $$ Tetradecanonitrile $$ Myristyl nitrile $$

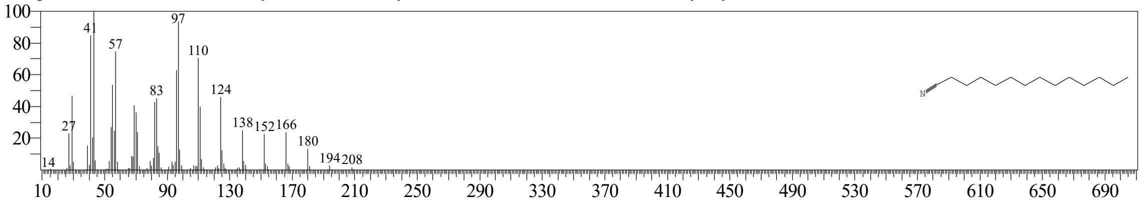

Hit#:3 Entry:32161 Library:NIST11.lib
SI:62 Formula:C12H23N CAS:2437-25-4 MolWeight:181 RetIndex:1459
CompName:Dodecanenitrile $$ Lauronitrile $$ Decylacetonitrile $$ Dodecanonitrile $$ Lauric nitrile $$ Undecyl cyanide $$ Lauric acid nitrile $$ 1-Cyanounde

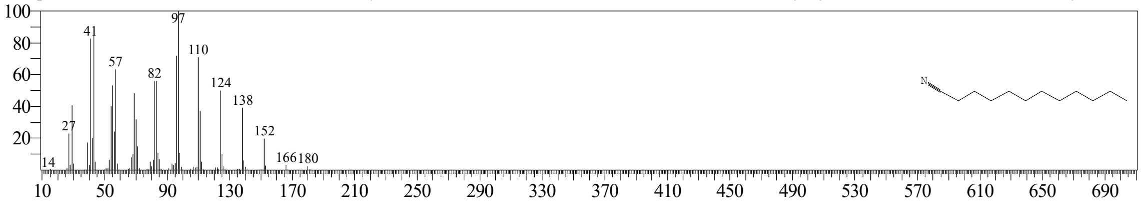

Hit#:4 Entry:104508 Library:NIST11.lib
SI:62 Formula:C19H38O CAS:67860-04-2 MolWeight:282 RetIndex:2000
CompName:Oxirane, heptadecyl- $$ 1,2-Epoxynonadecane $$ 2-Heptadecyloxirane # $$

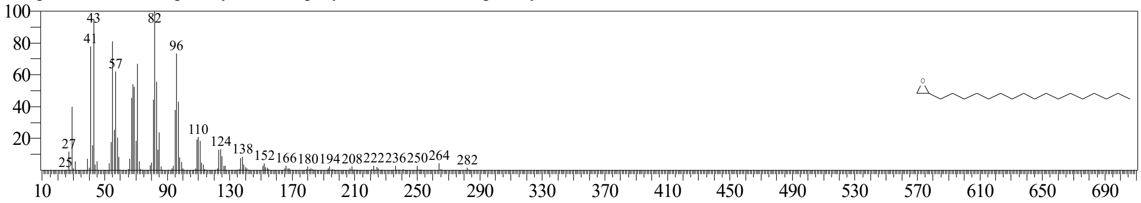

Hit#:5 Entry:166856 Library:NIST11.lib
SI:61 Formula:C25H52O CAS:26040-98-2 MolWeight:368 RetIndex:2749
CompName:1-Pentacosanol

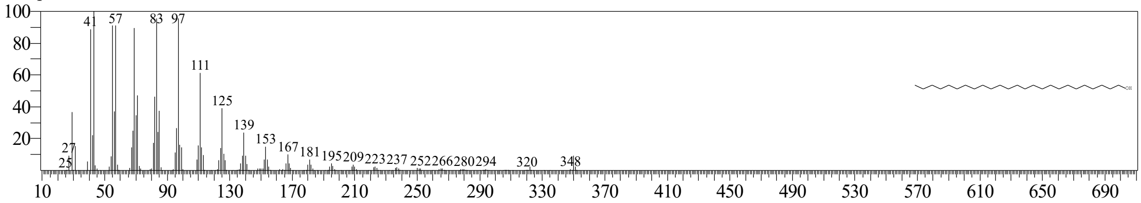

<< Target >>
Line#:131 R.Time:33.400(Scan#:5881) MassPeaks:391
RawMode:Averaged 33.395-33.405(5880-5882) BasePeak:69.05(5939)
BG Mode:Calc. from Peak Group 1 - Event 1 Scan

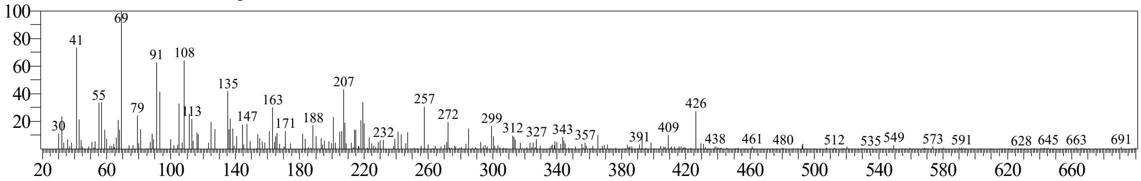

Hit#:1 Entry:191148 Library:NIST11.lib
SI:54 Formula:C30H50O CAS:0-00-0 MolWeight:426 RetIndex:2836
CompName:6a,14a-Methanopicene, perhydro-1,2,4a,6b,9,9,12a-heptamethyl-10-hydroxy-

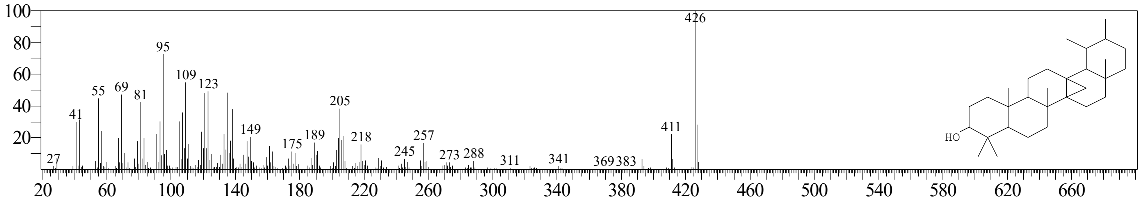

Hit#:2 Entry:191097 Library:NIST11.lib
SI:53 Formula:C30H50O CAS:545-47-1 MolWeight:426 RetIndex:2848
CompName:Lupeol $$ Lup-20(29)-en-3-ol, (3.beta.)- $$ Lup-20(29)-en-3.beta.-ol $$ .beta.-Viscol $$ Clerodol $$ Fagarasterol $$ Fagarsterol $$ Lupenol $$ Mo

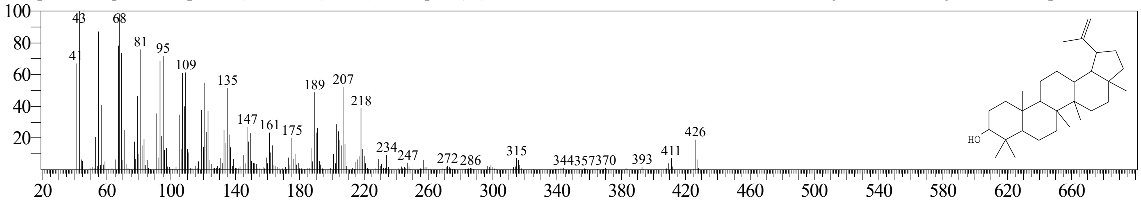

Hit#:3 Entry:191111 Library:NIST11.lib
SI:51 Formula:C30H50O CAS:54159-46-5 MolWeight:426 RetIndex:3058
CompName:1,6,10,14,18,22-Tetracosahexaen-3-ol, 2,6,10,15,19,23-hexamethyl-, (all-E)- $$ (6E,10E,14E,18E)-2,6,10,15,19,23-Hexamethyl-1,6,10,14,18,22-te

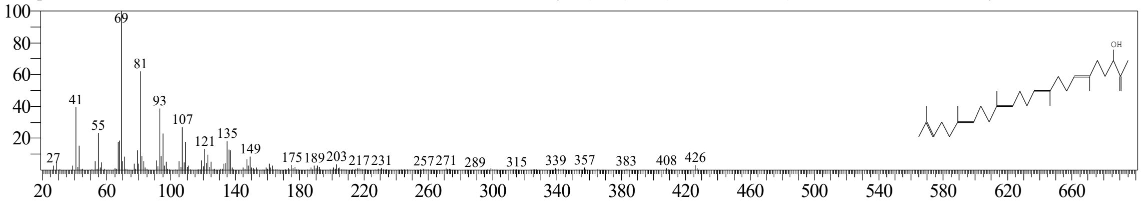

Hit#:4 Entry:101384 Library:NIST11.lib
SI:50 Formula:C18H30O2 CAS:0-00-0 MolWeight:278 RetIndex:1854
CompName:Nerolidyl propionate $$ (4E)-1,5,9-Trimethyl-1-vinyl-4,8-decadienyl propionate # $$

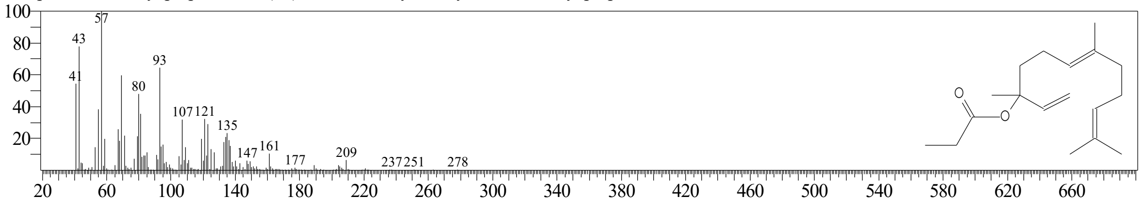

Hit#:5 Entry:124851 Library:NIST11.lib
SI:50 Formula:C19H29ClO CAS:4834-82-6 MolWeight:308 RetIndex:2107
CompName:3.alpha.-Chloro-5.alpha.-androstan-17-one

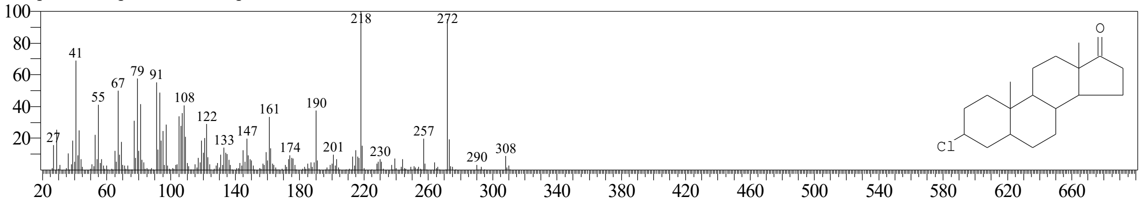

<< Target >>
Line#:132 R.Time:33.550(Scan#:5911) MassPeaks:355
RawMode:Averaged 33.545-33.555(5910-5912) BasePeak:79.05(4039)
BG Mode:Calc. from Peak Group 1 - Event 1 Scan

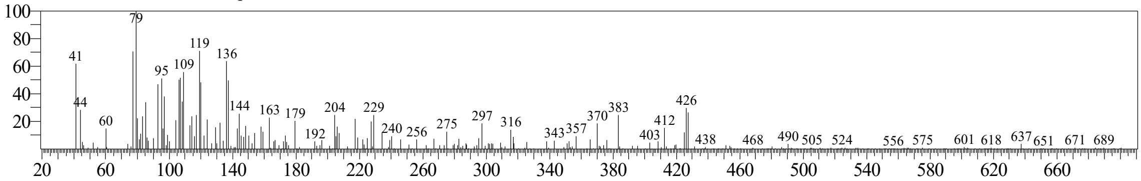

Hit#:1 Entry:62923 Library:NIST11.lib
SI:50 Formula:C15H17NO CAS:136309-85-8 MolWeight:227 RetIndex:1978
CompName:2-Bicyclo[3.3.0]octene-4-carbanilide $$ N-Phenyl-1,3a,4,5,6,6a-hexahydro-1-pentalenecarboxamide # $$

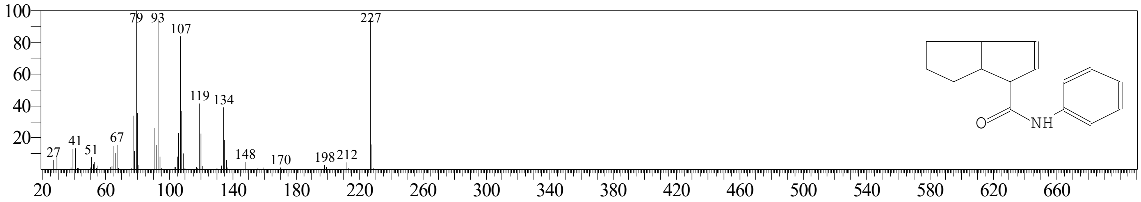

Hit#:2 Entry:47981 Library:NIST11.lib
SI:50 Formula:C14H22O CAS:0-00-0 MolWeight:206 RetIndex:1431
CompName:1-Oxaspiro[2.5]octane, 5,5-dimethyl-4-(3-methyl-1,3-butadienyl)- $$ 5,5-Dimethyl-4-[(1E)-3-methyl-1,3-butadienyl]-1-oxaspiro[2.5]octane # $$

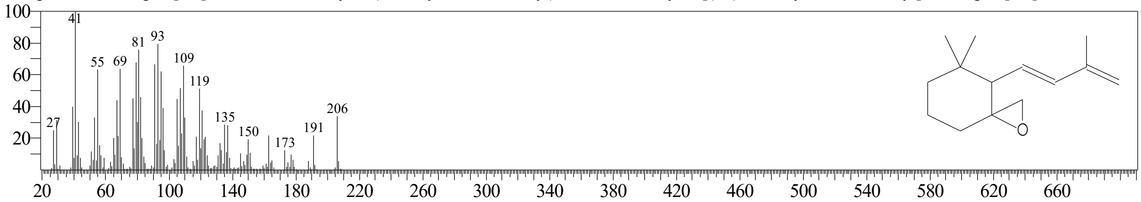

Hit#:3 Entry:88409 Library:NIST11.lib
SI:47 Formula:C12H16Cl2O2 CAS:0-00-0 MolWeight:262 RetIndex:1583
CompName:Dichloroacetic acid, 2,7-dimethyloct-7-en-5-yn-4-yl

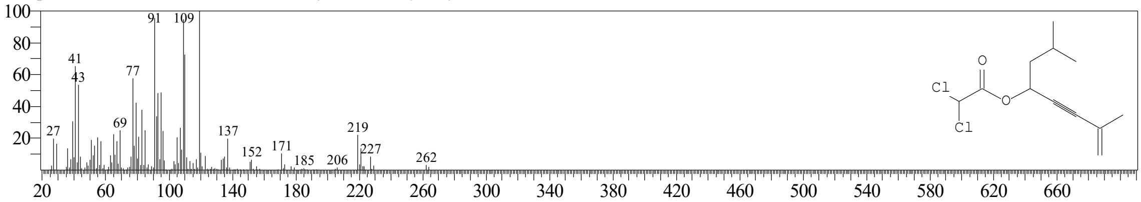

Hit#:4 Entry:59458 Library:NIST11.lib
SI:47 Formula:C15H26O CAS:465-24-7 MolWeight:222 RetIndex:1593
CompName:1,4-Methanoazulen-9-ol, decahydro-1,5,5,8a-tetramethyl-, [1R-(1.alpha.,3a.beta.,4.alpha.,8a.beta.,9S*)]- $$ Longiborneol $$ Juniperol $$

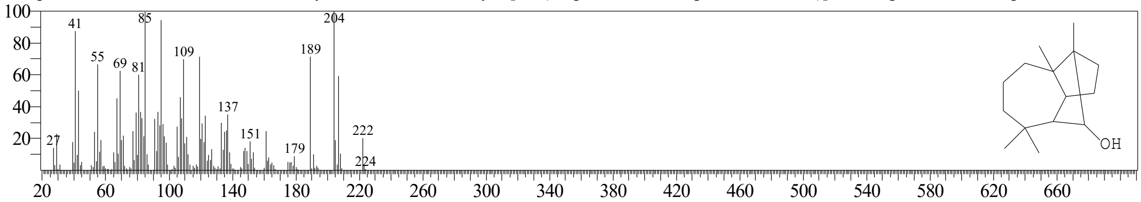

Hit#:5 Entry:46573 Library:NIST11.lib
SI:47 Formula:C14H20O CAS:452049-62-6 MolWeight:204 RetIndex:1581
CompName:Spiro[cyclopropane-1,8'(1H')[3a.6]methano[3ah]cyclopentacycloocten]-10'-one, octahydro-, (3'as,6'R,9'ar)-

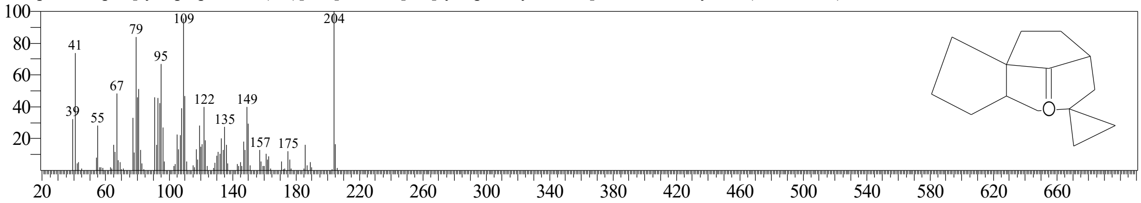

<< Target >>
Line#:133 R.Time:33.565(Scan#:5914) MassPeaks:344
RawMode:Averaged 33.560-33.570(5913-5915) BasePeak:55.05(4664)
BG Mode:Calc. from Peak Group 1 - Event 1 Scan

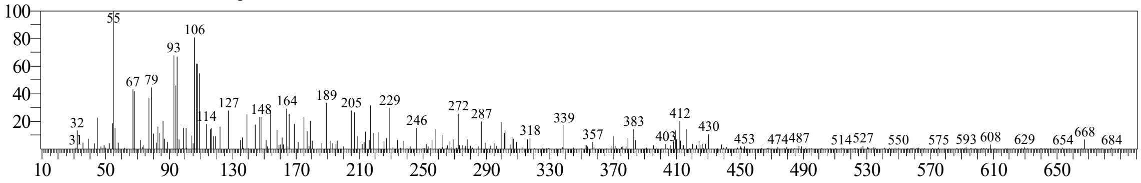

Hit#:1 Entry:48598 Library:NIST11.lib
SI:53 Formula:C13H21NO CAS:0-00-0 MolWeight:207 RetIndex:1507
CompName:4,7,7-Trimethylbicyclo[2.2.1]heptan-2-one O-allyloxime $$ (2E)-4,7,7-Trimethylbicyclo[2.2.1]heptan-2-one o-allyloxime # $$

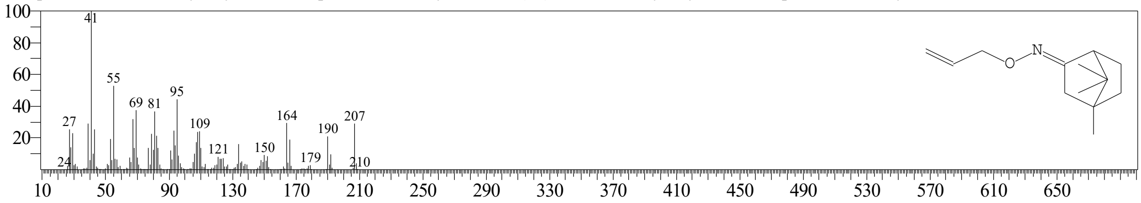

Hit#:2 Entry:161970 Library:NIST11.lib
SI:51 Formula:C22H32O4 CAS:10124-02-4 MolWeight:360 RetIndex:2477
CompName:5.beta.,14.beta.-Androstane-17.beta.-carboxylic acid, 3.beta.,14-dihydroxy-, .gamma.-lactone, acetate

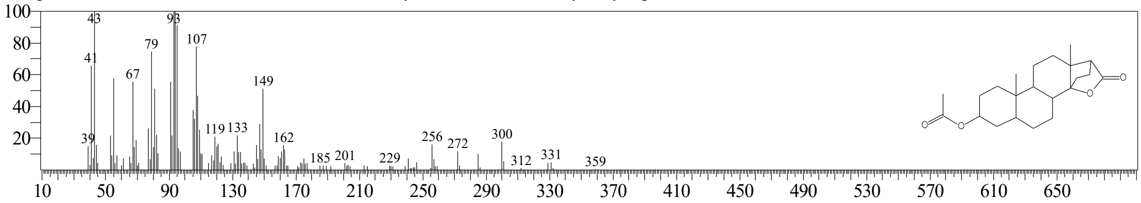

Hit#:3 Entry:60659 Library:NIST11.lib
SI:51 Formula:C13H20O3 CAS:22469-70-1 MolWeight:224 RetIndex:1548
CompName:Ethyl (+)-camphorcarboxylate $$ Ethyl 4,7,7-trimethyl-3-oxobicyclo[2.2.1]heptane-2-carboxylate # $$

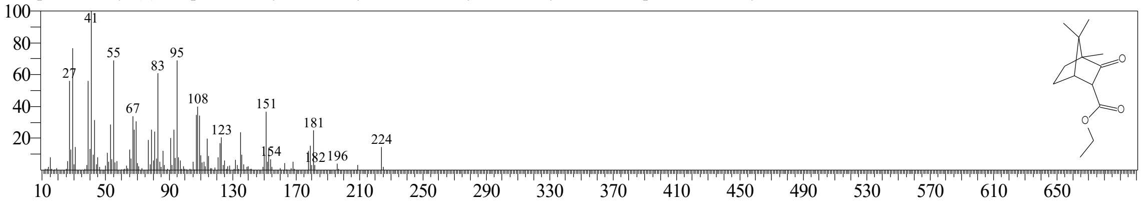

Hit#:4 Entry:10380 Library:NIST11.lib
SI:51 Formula:C9H14O CAS:6975-94-6 MolWeight:138 RetIndex:1073
CompName:3,5-Dimethylcyclohex-1-ene-4-carboxaldehyde $$ 2,6-Dimethyl-3-cyclohexene-1-carbaldehyde # $$

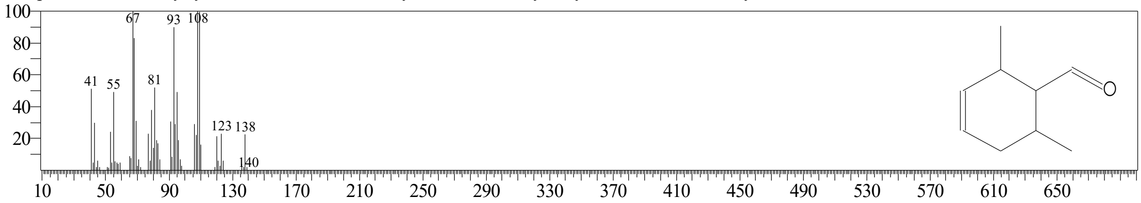

Hit#:5 Entry:109916 Library:NIST11.lib
SI:50 Formula:C19H31NO CAS:14475-43-5 MolWeight:289 RetIndex:2102
CompName:Androstan-11-one, oxime, (5.alpha.)- $$ 5.alpha.-Androstan-11-one, oxime $$ Androstan-11-one oxime # $$

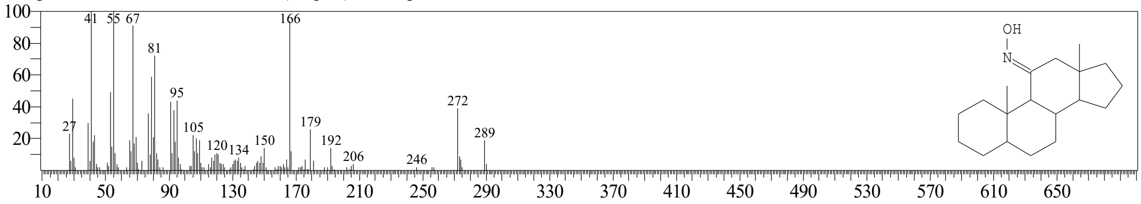

<< Target >>
Line#:134 R.Time:33.580(Scan#:5917) MassPeaks:349
RawMode:Averaged 33.575-33.585(5916-5918) BasePeak:79.05(5445)
BG Mode:Calc. from Peak Group 1 - Event 1 Scan

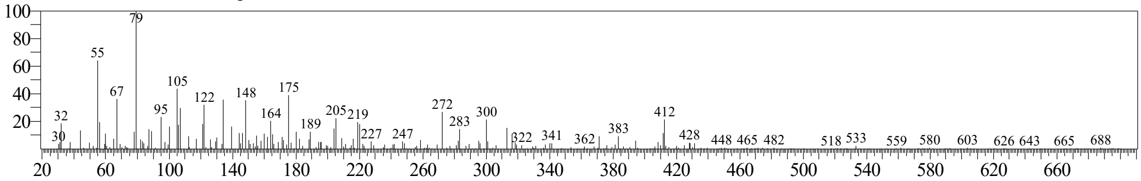

Hit#:1 Entry:46753 Library:NIST11.lib
SI:45 Formula:C15H24 CAS:32391-44-9 MolWeight:204 RetIndex:1351
CompName:2H-2,4a-Ethanonaphthalene, 1,3,4,5,6,7-hexahydro-2,5,5-trimethyl-

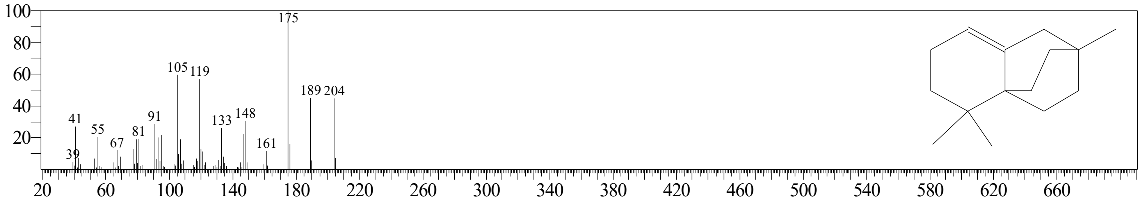

Hit#:2 Entry:143338 Library:NIST11.lib
SI:44 Formula:C22H36O2 CAS:0-00-0 MolWeight:332 RetIndex:2407
CompName:Butyl 6,9,12,15-octadecatetraenoate

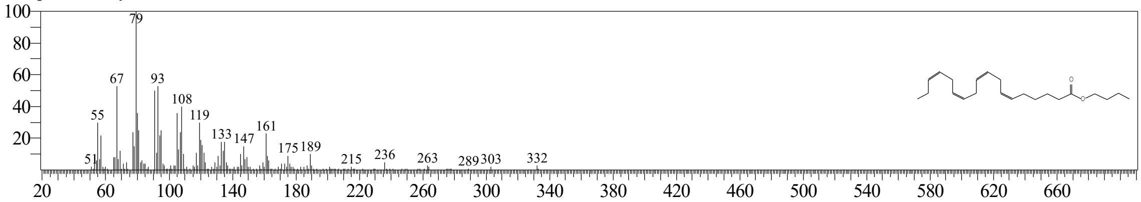

Hit#:3 Entry:46569 Library:NIST11.lib
SI:43 Formula:C14H20O CAS:0-00-0 MolWeight:204 RetIndex:1513
CompName:2,10,10-Trimethyltricyclo[7.1.1.0(2,7)]undec-6-en-8-one

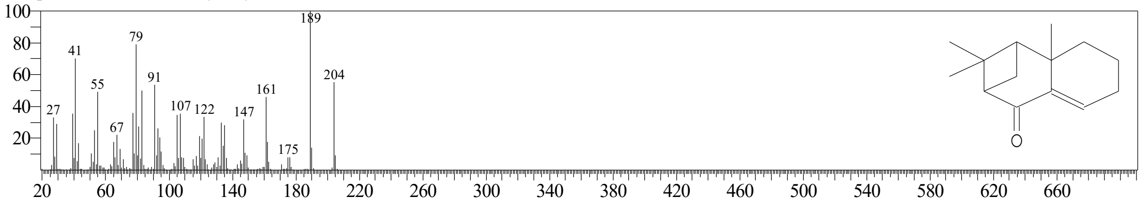

Hit#:4 Entry:46644 Library:NIST11.lib
SI:43 Formula:C15H24 CAS:87745-31-1 MolWeight:204 RetIndex:1490
CompName:Aciphyllene $$ 5-Isopropenyl-3,8-dimethyl-1,2,4,5,6,7,8,8a-octahydroazulene # $$ Guaia-4,11-diene $$

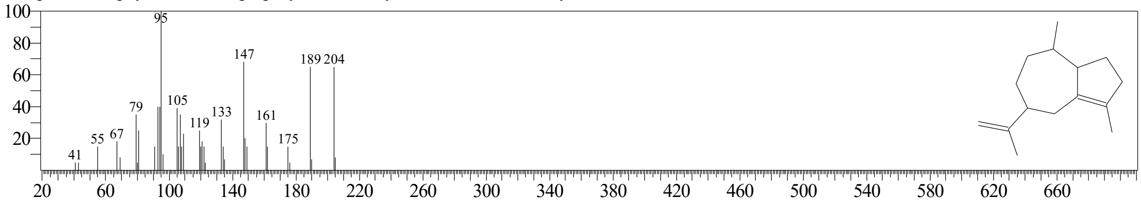

Hit#:5 Entry:121880 Library:NIST11.lib
SI:43 Formula:C20H32O2 CAS:0-00-0 MolWeight:304 RetIndex:2209
CompName:Ethyl 6,9,12,15-octadecatetraenoate

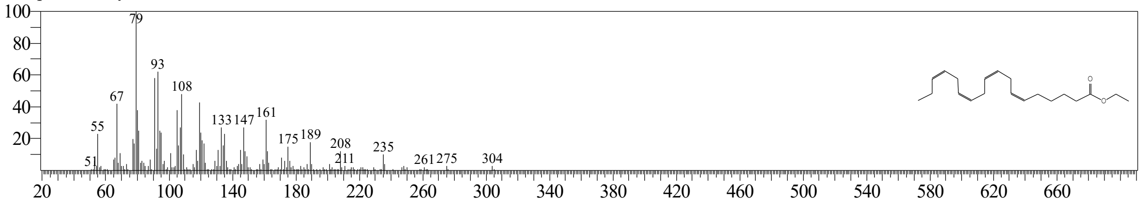

Qualitative Analysis Report

<< Target >>
Line#:135 R.Time:33.625(Scan#:5926) MassPeaks:384
RawMode:Averaged 33.620-33.630(5925-5927) BasePeak:71.05(4904)
BG Mode:Calc. from Peak Group 1 - Event 1 Scan

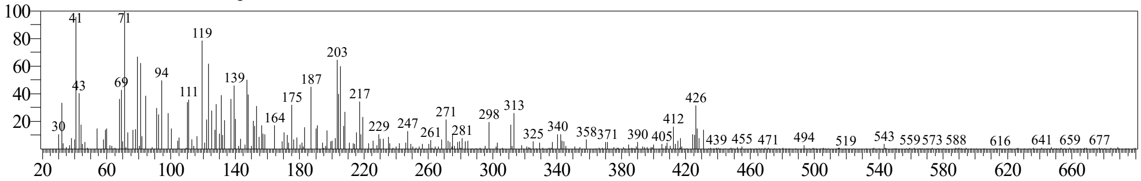

Hit#:1 Entry:161855 Library:NIST11.lib
SI:47 Formula:C20H24O6 CAS:10207-58-6 MolWeight:360 RetIndex:2734
CompName:17,18-Dinor-8.beta.H,9.beta.H-labda-13(16),14-diene-19,20-dioic acid, 15,16-epoxy-1.beta.,4,12-trihydroxy-, di-.delta.-lactone

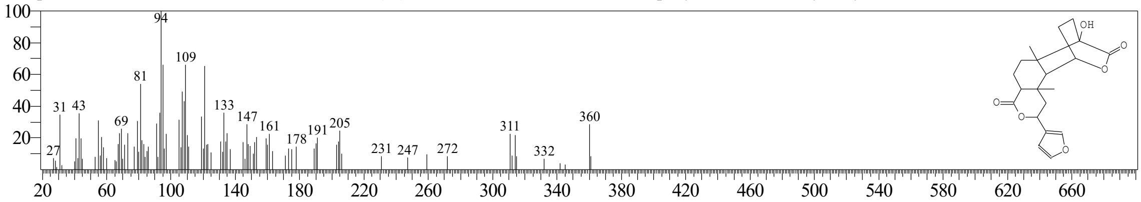

Hit#:2 Entry:191097 Library:NIST11.lib
SI:47 Formula:C30H50O CAS:545-47-1 MolWeight:426 RetIndex:2848
CompName:Lupeol $$ Lup-20(29)-en-3-ol, (3.beta.)- $$ Lup-20(29)-en-3.beta.-ol $$ .beta.-Viscol $$ Clerodol $$ Fagarasterol $$ Fagarsterol $$ Lupenol $$ Mo

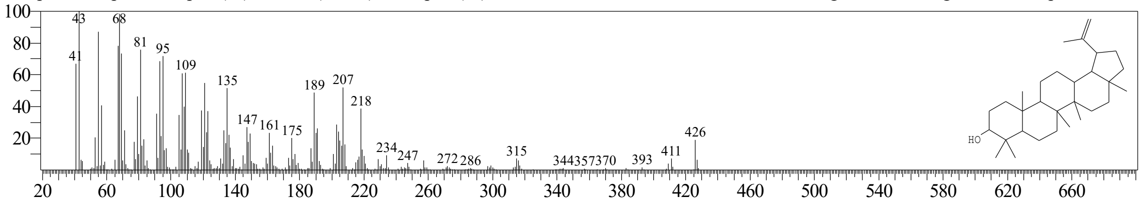

Hit#:3 Entry:190447 Library:NIST11.lib
SI:47 Formula:C30H48O CAS:10070-76-5 MolWeight:424 RetIndex:2886
CompName:Olean-12-en-28-al

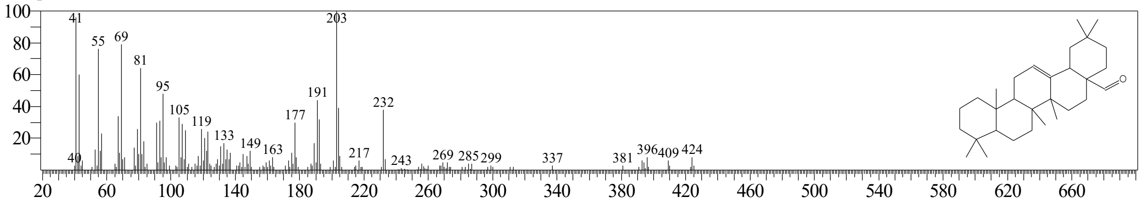

Hit#:4 Entry:200399 Library:NIST11.lib
SI:46 Formula:C32H52O2 CAS:1617-68-1 MolWeight:468 RetIndex:2987
CompName:Lup-20(29)-en-3-ol, acetate, (3.beta.)- $$ Lup-20(29)-en-3.beta.-ol, acetate $$ Lupenyl acetate $$ Lupeol acetate $$ Lupeyl acetate $$ 3-O-Acetyllu

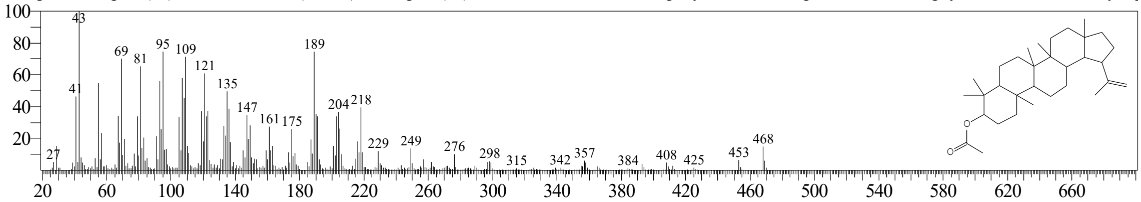

Hit#:5 Entry:183126 Library:NIST11.lib
SI:46 Formula:C20H35BrO3 CAS:115346-29-7 MolWeight:402 RetIndex:2709
CompName:1,2-Pentanediol, 5-(6-bromodecahydro-2-hydroxy-2,5,5a,8a-tetramethyl-1-naphthalenyl)-3-methylene- $$ 3-[2-(6-Bromo-2-hydroxy-2,5,5,8a-tetran

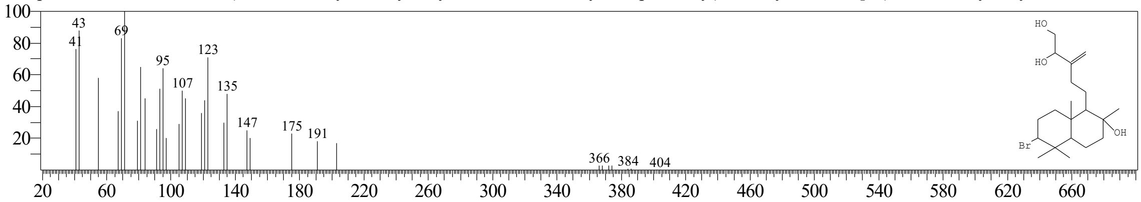

Supplement: Supplementary file 1 [file pharmaceuticals-19-01635-s001.zip › File S1—GC-MS analysis/Ozoroa paniculosa 70% Ethanol (Ramile).pdf]
