# Supplementary material for: Phyto-Mediated Synthesized Gold Nanoparticles (AuNPs) from Ozoroa paniculosa Leaf Extracts: Chemical Characterization and Antidiabetic Evaluation
Source: Pharmaceuticals (Basel). 2026 Oct 15;19(10):1635. doi: 10.3390/ph19101635 (PMC13708053; doi:10.3390/ph19101635)

Qualitative Analysis Report

Sample Information

Sample Type	: Unknown
Sample Name	: C:\GCMSData\Raw Data\Ext - UniZulu\16-E-C.qgd
Sample ID	: Prince
Vial #	: 98
Method File	: C:\GCMSData\general - split - extended.qgm
Tuning File	: C:\GCMSsolution\System\Tune1\Normal Conc - 2025 04.qgt

Chromatogram OPD hex

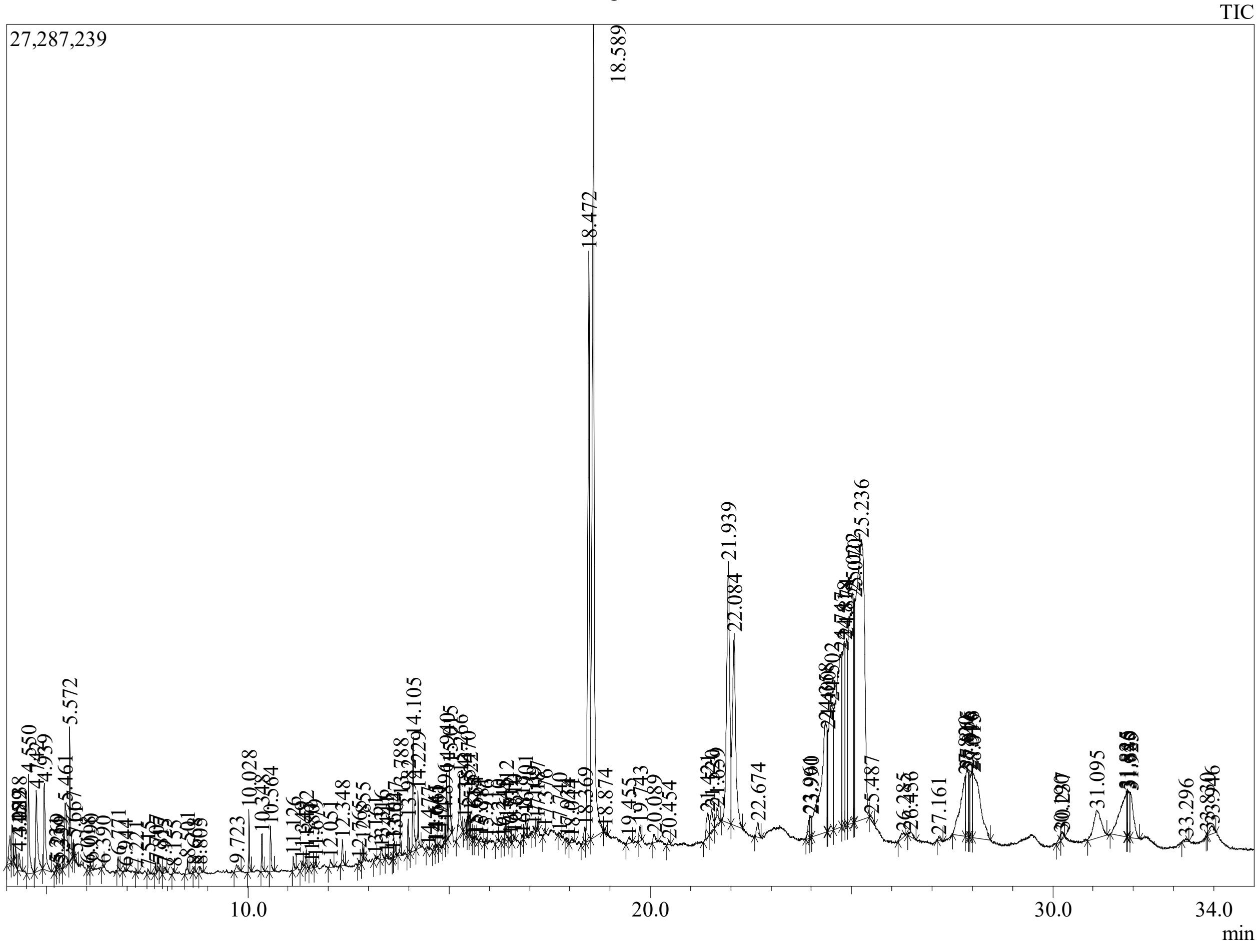

Peak Report TIC

Peak#	R.Time	Area	Area%	Height	Height%	A/H	Name
1	4.082	2926115	0.34	790963	0.42	3.70	Cycloheptanone, 3-methyl-, (R)-
2	4.138	2740156	0.32	1182223	0.63	2.32	Octane, 4-methyl-
3	4.229	1193686	0.14	849331	0.45	1.41	Heptane, 4-(1-methylethyl)-
4	4.315	1618651	0.19	550452	0.29	2.94	o-Xylene
5	4.550	6497079	0.75	3149834	1.67	2.06	Nonane
6	4.742	8121987	0.94	2583865	1.37	3.14	2-Hexanone, 3,3-dimethyl-
7	4.939	7851001	0.91	2729201	1.45	2.88	Ethanone, 1-(3-ethyloxiranyl)-
8	5.231	294822	0.03	184551	0.10	1.60	Nonane, 2-methyl-
9	5.269	273123	0.03	205176	0.11	1.33	Oxalic acid, decyl propyl ester
10	5.339	249790	0.03	119197	0.06	2.10	Octane, 4-bromo-
11	5.461	7907821	0.92	2006567	1.06	3.94	Cyclopentane, 1-acetyl-1,2-epoxy-
12	5.572	8284448	0.96	4260184	2.26	1.94	.beta.-Myrcene
13	5.667	640823	0.07	585957	0.31	1.09	Nonane
14	5.711	467191	0.05	203811	0.11	2.29	Benzene, 1,2,4-trimethyl-
15	6.018	557291	0.06	160708	0.09	3.47	Benzene, 1,2,4-trimethyl-
16	6.090	483072	0.06	255643	0.14	1.89	Bicyclo[3.1.0]hexane, 4-methylene-1-(1-methyl
17	6.390	204721	0.02	110330	0.06	1.86	(+)-3-Carene, 2-(acetylmethyl)-
18	6.771	815288	0.09	446068	0.24	1.83	Decane, 2,9-dimethyl-
19	6.944	222892	0.03	109765	0.06	2.03	3-Buten-2-ol, 2,3-dimethyl-
20	7.221	185625	0.02	114634	0.06	1.62	3-Methylpentan-3-yl propyl carbonate
21	7.515	350862	0.04	134989	0.07	2.60	Undecane, 2,4-dimethyl-
22	7.707	464537	0.05	325794	0.17	1.43	3-Methyl-2-butenoic acid, 2-tetrahydrofurylmet
23	7.817	386160	0.04	182673	0.10	2.11	Nonane, 2-methyl-
24	7.925	680892	0.08	205995	0.11	3.31	6,6,7-Trimethyl-octane-2,5-dione
25	8.155	271053	0.03	174698	0.09	1.55	n-Caproic acid vinyl ester
26	8.501	1058816	0.12	467767	0.25	2.26	n-Caproic acid vinyl ester

Qualitative Analysis Report

Peak#	R.Time	Area	Area%	Height	Height%	A/H	Name
27	8.668	273542	0.03	196955	0.10	1.39	2-Pentenoic acid, 4-hydroxy-
28	8.805	548472	0.06	208730	0.11	2.63	2,3-Dimethyldecane
29	9.723	967073	0.11	221082	0.12	4.37	1-Nonene, 4,6,8-trimethyl-
30	10.028	2801608	0.32	1993305	1.06	1.41	Bicyclo[7.2.0]undec-4-ene, 4,11,11-trimethyl-8
31	10.348	1929311	0.22	1209938	0.64	1.59	Humulene
32	10.564	2499761	0.29	1435157	0.76	1.74	1,6-Cyclodecadiene, 1-methyl-5-methylene-8-(
33	11.126	884245	0.10	471704	0.25	1.87	Cyclohexanemethanol, 4-ethenyl-.alpha.,.alpha
34	11.349	415820	0.05	237585	0.13	1.75	1-Pentadecene
35	11.452	886553	0.10	547278	0.29	1.62	Caryophyllene oxide
36	11.580	429986	0.05	128932	0.07	3.33	Octan-2-one, 3,6-dimethyl-
37	11.679	396014	0.05	285300	0.15	1.39	12-Oxabicyclo[9.1.0]dodeca-3,7-diene, 1,5,5,8-
38	12.051	743738	0.09	254995	0.14	2.92	2-Naphthalenemethanol, decahydro-.alpha.,.alp
39	12.348	1713102	0.20	811928	0.43	2.11	Octadecanal
40	12.765	303787	0.04	150581	0.08	2.02	Tetradecanoic acid
41	12.855	808730	0.09	648394	0.34	1.25	1-Heptadecene
42	13.161	385937	0.04	163465	0.09	2.36	1,1'-Bicyclopentyl, 2-hexadecyl-
43	13.316	924896	0.11	329989	0.17	2.80	2-Naphthalenemethanol, decahydro-.alpha.,.alp
44	13.441	316060	0.04	151547	0.08	2.09	Undecanoic acid, 10-bromo-
45	13.602	365158	0.04	221747	0.12	1.65	9,12,15-Octadecatrienoic acid, 2,3-dihydroxypr
46	13.647	1136809	0.13	521584	0.28	2.18	9,12,15-Octadecatrienal
47	13.788	2608514	0.30	1835207	0.97	1.42	Pentadecanoic acid, 14-methyl-, methyl ester
48	13.982	1554090	0.18	1114594	0.59	1.39	1,6,10-Dodecatriene, 7,11-dimethyl-3-methylen
49	14.105	7395630	0.86	3623288	1.92	2.04	n-Hexadecanoic acid
50	14.229	3158443	0.37	1824899	0.97	1.73	3-Tetradecene, (E)-
51	14.477	284489	0.03	102009	0.05	2.79	2,2-Dimethyl-3-vinyl-bicyclo[2.2.1]heptane
52	14.645	343420	0.04	150977	0.08	2.27	Oxazolidin-2-one, 3-tert-butyl-5-phenoxymethy
53	14.661	294779	0.03	181605	0.10	1.62	Ergostane-3,5,6,12,25-pentol, 25-acetate, (3.be
54	14.751	237348	0.03	108565	0.06	2.19	Pentadecanoic acid, 14-bromo-
55	14.896	987172	0.11	634609	0.34	1.56	9,12-Octadecadienoic acid, methyl ester
56	14.940	3014104	0.35	2140759	1.13	1.41	Methyl (Z)-5,11,14,17-eicosatetraenoate
57	15.015	4996262	0.58	2257086	1.20	2.21	Phytol
58	15.266	5535368	0.64	1947680	1.03	2.84	9,12,15-Octadecatrienoic acid, (Z,Z,Z)-
59	15.384	1168390	0.14	460224	0.24	2.54	Octadecanoic acid, 2-(2-hydroxyethoxy)ethyl e
60	15.470	1807574	0.21	1401869	0.74	1.29	Hexadecanoic acid, butyl ester
61	15.522	871673	0.10	772298	0.41	1.13	1-Heptadecene
62	15.615	154603	0.02	107023	0.06	1.44	Cyclopentanecarboxylic acid, 3-methylene-2,2-
63	15.654	294025	0.03	141891	0.08	2.07	Cycloheptane, 4-methylene-1-methyl-2-(2-meth
64	15.781	514226	0.06	140716	0.07	3.65	9,19-Cycloergost-24(28)-en-3-ol, 4,14-dimethy
65	15.916	309308	0.04	156147	0.08	1.98	1-Acetyl-4-amino-5-isobutyl-2,5-dihydro-1H-p
66	16.210	378268	0.04	101640	0.05	3.72	Rhodopin
67	16.345	289103	0.03	117625	0.06	2.46	4-Bromobutanoic acid, 2-methylphenyl ester
68	16.412	1481800	0.17	652921	0.35	2.27	Acetic acid, 4-methylphenyl ester
69	16.512	265486	0.03	142135	0.08	1.87	Cyclohexene, 1,5,5-trimethyl-6-acetylmethyl-
70	16.610	491295	0.06	244599	0.13	2.01	(E)-13-Docosenoic acid
71	16.817	494189	0.06	168101	0.09	2.94	6-Methyloctahydrocoumarin
72	16.901	1512541	0.18	755832	0.40	2.00	Methyl (Z)-5,11,14,17-eicosatetraenoate
73	17.109	450123	0.05	198914	0.11	2.26	Octadecanoic acid, 2-methylpropyl ester
74	17.187	546851	0.06	339964	0.18	1.61	10-Heneicosene (c,t)
75	17.376	401118	0.05	189739	0.10	2.11	Oxalic acid, hexyl 2-methylphenyl ester
76	17.740	344284	0.04	111703	0.06	3.08	Ethanone, 1-(octahydro-1H-inden-1-yl)-, (1.alp
77	17.934	376044	0.04	125140	0.07	3.00	Bicyclo[2.2.1]heptane-3-methylene-2,2-dimethy
78	18.044	443276	0.05	116411	0.06	3.81	5,8-Decadien-2-one, 5,9-dimethyl-, (E)-
79	18.369	1378206	0.16	516845	0.27	2.67	Docosane, 11-decyl-
80	18.472	57792697	6.69	18653094	9.88	3.10	rotundene
81	18.589	98327353	11.38	25733937	13.63	3.82	Phenol, 3-pentadecyl-
82	18.874	340216	0.04	140484	0.07	2.42	Bis(tridecyl) phthalate
83	19.455	931902	0.11	188959	0.10	4.93	Methyl (Z)-5,11,14,17-eicosatetraenoate
84	19.743	1389373	0.16	510326	0.27	2.72	Z-12-Pentacosene
85	20.089	932053	0.11	265380	0.14	3.51	Acetic acid, 4-methylphenyl ester
86	20.454	381200	0.04	144284	0.08	2.64	Octadecanal
87	21.421	3312455	0.38	755114	0.40	4.39	Methyl (Z)-5,11,14,17-eicosatetraenoate
88	21.520	3567969	0.41	729441	0.39	4.89	Oxalic acid, dodecyl 2-methylphenyl ester
89	21.659	2555305	0.30	568755	0.30	4.49	2,3-Dimethyldodecane
90	21.939	42956870	4.97	8280196	4.38	5.19	rotundene
91	22.084	34776243	4.02	6089487	3.22	5.71	Benzene, 1-(1,1-dimethylpropoxy)-4-methyl-
92	22.674	1918386	0.22	469295	0.25	4.09	Terephthalic acid, 2-ethylhexyl undecyl ester
93	23.961	2297326	0.27	727024	0.38	3.16	D:B-Friedo-18,19-secolup-19-ene, 3,10-epoxy-
94	23.990	1978105	0.23	698061	0.37	2.83	Methyl 4,4-difluororetinoate (all-trans)
95	24.358	39483558	4.57	3474668	1.84	11.36	Squalene
96	24.400	1917848	0.22	3201059	1.69	0.60	3,6-Nonadien-5-one, 2,2,8,8-tetramethyl-
97	24.502	31061891	3.59	4041569	2.14	7.69	Lanosterol
98	24.747	60826293	7.04	5532850	2.93	10.99	Lanosterol
99	24.818	25282978	2.93	5861657	3.10	4.31	Lanosterol
100	24.874	22470278	2.60	5916869	3.13	3.80	Obtusifoliol
101	25.022	57296548	6.63	7284912	3.86	7.87	Lanosterol
102	25.070	12513431	1.45	7074387	3.75	1.77	Rhodopin
103	25.236	127393094	14.74	8897218	4.71	14.32	Lanosterol
104	25.487	562804	0.07	151642	0.08	3.71	2,4a,8,8-Tetramethyldecahydrocyclopropa[d]na
105	26.285	1056764	0.12	144923	0.08	7.29	Pseduosarsasapogenin-5,20-dien
106	26.456	1037246	0.12	165670	0.09	6.26	Cholesta-17(20),24-diene-3,6-diol, (3.beta.,5.al
107	27.161	420765	0.05	125784	0.07	3.35	Octadecane, 1-chloro-
108	27.820	15905686	1.84	1921473	1.02	8.28	Obtusifoliol
109	27.865	9630610	1.11	2100415	1.11	4.59	Ergost-25-ene-3,5,6,12-tetrol, (3.beta.,5.alpha.,
110	27.916	3600061	0.42	2068949	1.10	1.74	Pregnenolone
111	27.976	7155426	0.83	2096806	1.11	3.41	cis-sesquisabinene hydrate
112	28.015	27808368	3.22	2030727	1.08	13.69	3-Isopropyltricyclo[4.3.1.1(2,5)]undec-3-en-10-

Qualitative Analysis Report

Peak#	R.Time	Area	Area%	Height	Height%	A/H	Name
113	30.190	759098	0.09	216628	0.11	3.50	Lupeol
114	30.237	970349	0.11	233631	0.12	4.15	Thunbergol
115	31.095	11325103	1.31	888053	0.47	12.75	1-Heptatriacotanol
116	31.825	15711115	1.82	1447264	0.77	10.86	9,19-Cycloergost-24(28)-en-3-ol, 4,14-dimethy
117	31.845	1736555	0.20	1461887	0.77	1.19	Lanosterol
118	31.880	5613842	0.65	1470461	0.78	3.82	Lanost-7-en-3-one
119	31.925	8271084	0.96	1431585	0.76	5.78	(-)-delta.-Panasinsine
120	33.296	537563	0.06	109286	0.06	4.92	Cholest-7-en-3-one, 4,4-dimethyl-, (5.alpha.)-
121	33.830	175078	0.02	117066	0.06	1.50	.beta.-Humulene
122	33.946	2287966	0.26	271314	0.14	8.43	Lupeol
		864129361	100.00	188856177	100.00		

Library

<< Target >>
Line#:1   R.Time:4.080(Scan#:17) MassPeaks:357
RawMode:Averaged 4.075-4.085(16-18) BasePeak:41.05(82635)
BG Mode:Calc. from Peak Group 1 - Event 1 Scan

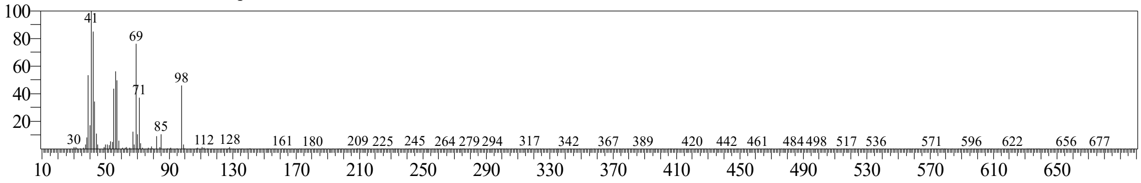

Hit#:1 Entry:6504 Library:NIST11.lib
SI:85 Formula:C8H14O CAS:13609-58-0 MolWeight:126 RetIndex:1072
CompName:Cycloheptanone, 3-methyl-, (R)- $$ Cycloheptanone, 3-methyl-, (+)- $$ 3-Methylcycloheptanone # $$

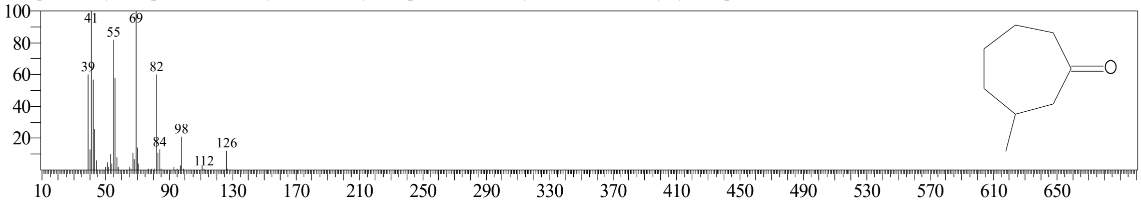

Hit#:2 Entry:4119 Library:NIST11.lib
SI:84 Formula:C7H14O CAS:7045-79-6 MolWeight:114 RetIndex:790
CompName:Oxetane, 2-methyl-4-propyl- $$ 2-Methyl-4-propyloxetane # $$

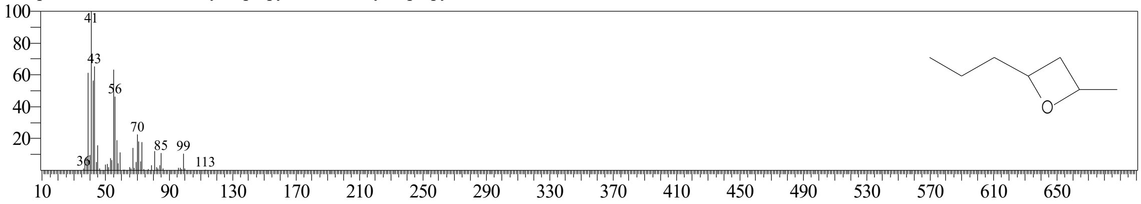

Hit#:3 Entry:1706 Library:NIST11.lib
SI:84 Formula:C7H14 CAS:930-57-4 MolWeight:98 RetIndex:719
CompName:Cyclopropane, butyl- $$ Butane, 1-cyclopropyl- $$ Butylcyclopropane $$

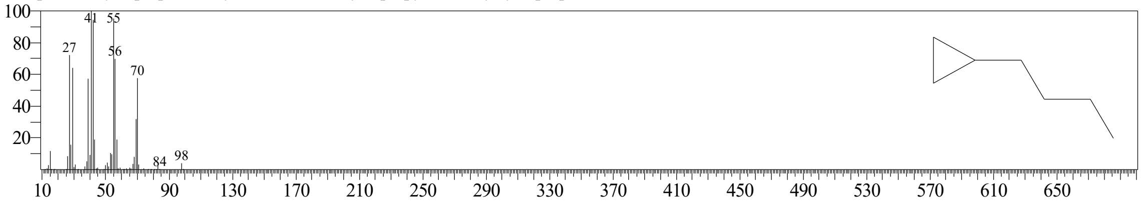

Hit#:4 Entry:1689 Library:NIST11.lib
SI:84 Formula:C6H10O CAS:1757-42-2 MolWeight:98 RetIndex:832
CompName:Cyclopentanone, 3-methyl- $$ 3-Methyl-1-cyclopentanone $$ 3-Methylcyclopentanone $$

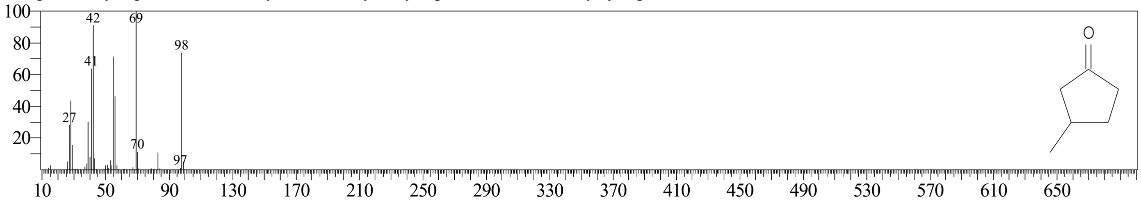

Hit#:5 Entry:1709 Library:NIST11.lib
SI:83 Formula:C7H14 CAS:14686-14-7 MolWeight:98 RetIndex:725
CompName:3-Heptene, (E)- $$ (E)-3-Heptene $$ trans-3-Heptene $$ 3-C7H14 $$ (3E)-3-Heptene # $$

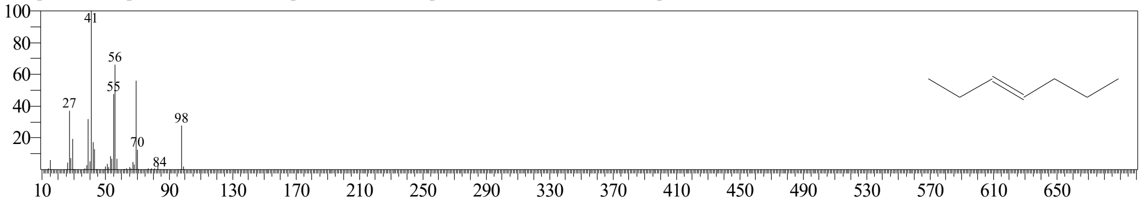

<< Target >>
Line#:2 R.Time:4.140(Scan#:29) MassPeaks:367
RawMode:Averaged 4.135-4.145(28-30) BasePeak:43.05(291279)
BG Mode:Calc. from Peak Group 1 - Event 1 Scan

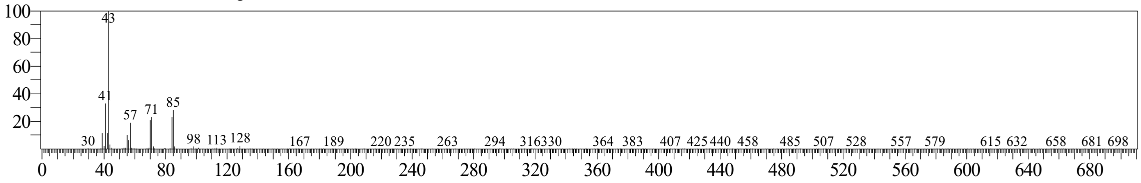

Hit#:1 Entry:7426 Library:NIST11.lib
SI:96 Formula:C9H20 CAS:2216-34-4 MolWeight:128 RetIndex:852
CompName:Octane, 4-methyl- $$ 4-Methyloctane $$ 1,3-Dimethylheptane $$

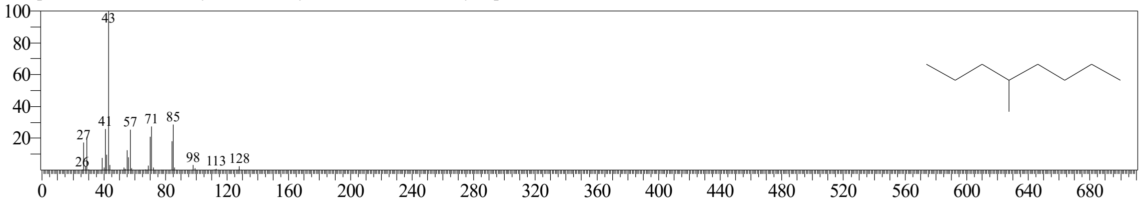

Hit#:2 Entry:7413 Library:NIST11.lib
SI:94 Formula:C9H20 CAS:921-47-1 MolWeight:128 RetIndex:724
CompName:Hexane, 2,3,4-trimethyl- $$ 2,3,4-Trimethylhexane $$

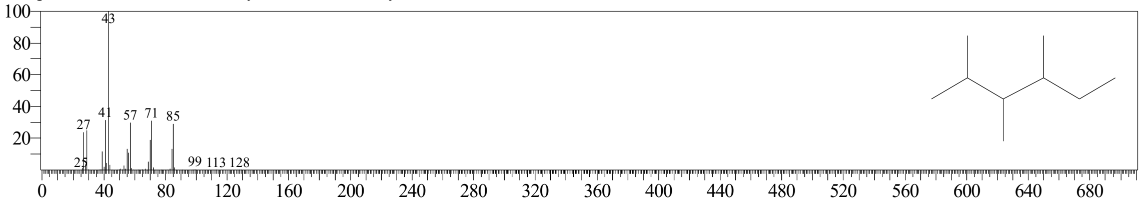

Hit#:3 Entry:4245 Library:NIST11.lib
SI:93 Formula:C8H18 CAS:619-99-8 MolWeight:114 RetIndex:752
CompName:Hexane, 3-ethyl- $$ 3-Ethylhexane $$

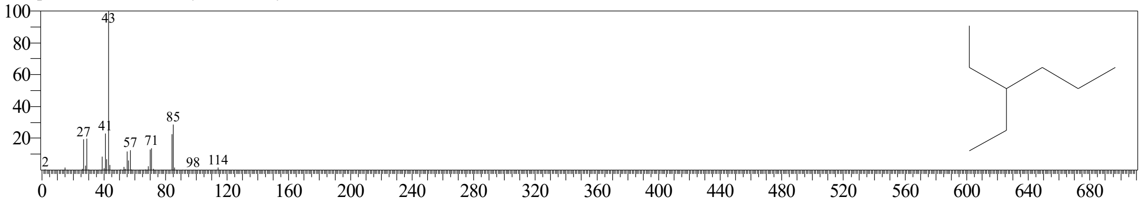

Hit#:4 Entry:7422 Library:NIST11.lib
SI:92 Formula:C9H20 CAS:1069-53-0 MolWeight:128 RetIndex:724
CompName:Hexane, 2,3,5-trimethyl- $$ 2,3,5-Trimethylhexane $$

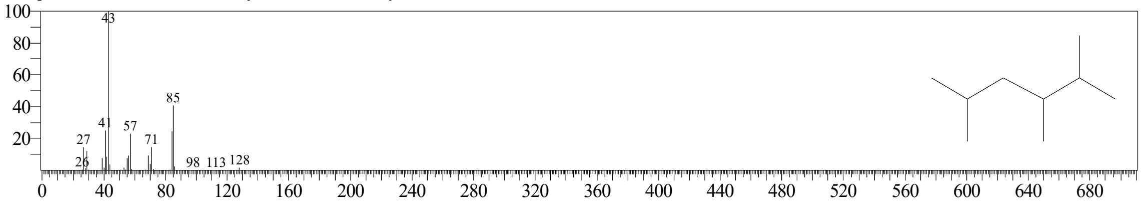

Hit#:5 Entry:7420 Library:NIST11.lib
SI:92 Formula:C9H20 CAS:3074-71-3 MolWeight:128 RetIndex:788
CompName:Heptane, 2,3-dimethyl- $$ 2,3-Dimethylheptane $$

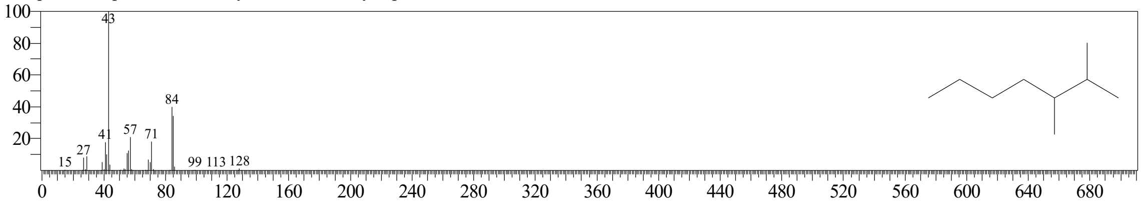

<< Target >>
Line#:3 R.Time:4.230(Scan#:47) MassPeaks:373
RawMode:Averaged 4.225-4.235(46-48) BasePeak:57.05(223336)
BG Mode:Calc. from Peak Group 1 - Event 1 Scan

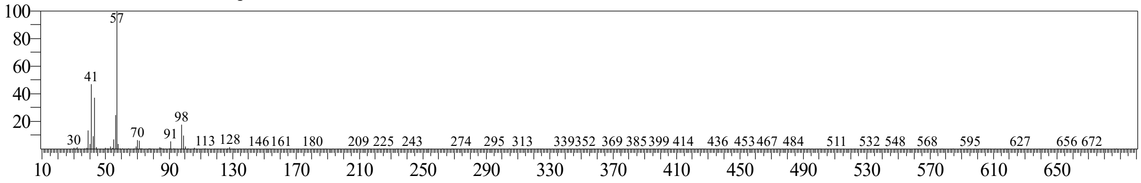

Hit#:1 Entry:12322 Library:NIST11.lib
SI:92 Formula:C10H22 CAS:52896-87-4 MolWeight:142 RetIndex:887
CompName:Heptane, 4-(1-methylethyl)- $$ 4-Isopropylheptane $$

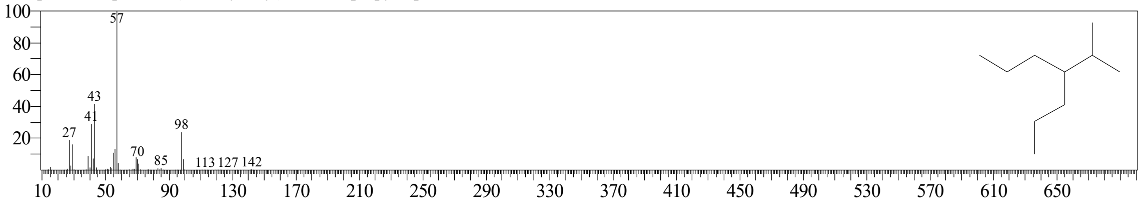

Hit#:2 Entry:7434 Library:NIST11.lib
SI:91 Formula:C9H20 CAS:2216-33-3 MolWeight:128 RetIndex:852
CompName:Octane, 3-methyl- $$ 3-Methyloctane $$ Isononane $$

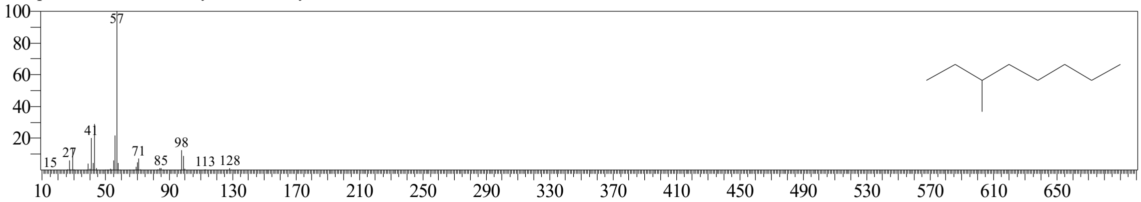

Hit#:3 Entry:7433 Library:NIST11.lib
SI:91 Formula:C9H20 CAS:2216-30-0 MolWeight:128 RetIndex:788
CompName:Heptane, 2,5-dimethyl- $$ 2,5-Dimethylheptane $$

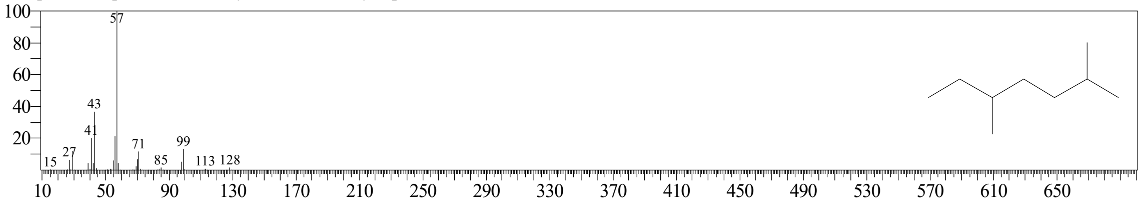

Hit#:4 Entry:29894 Library:NIST11.lib
SI:91 Formula:C7H15Br CAS:1974-05-6 MolWeight:178 RetIndex:949
CompName:Heptane, 3-bromo- $$ 3-Bromoheptane $$

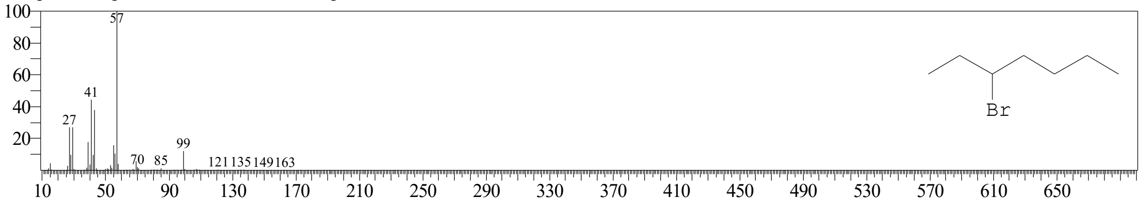

Hit#:5 Entry:7427 Library:NIST11.lib
SI:90 Formula:C9H20 CAS:926-82-9 MolWeight:128 RetIndex:788
CompName:Heptane, 3,5-dimethyl- $$ 3,5-Dimethylheptane $$

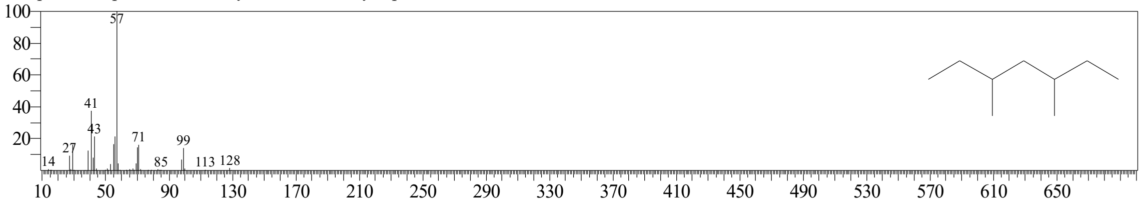

<< Target >>
Line#:4 R.Time:4.315(Scan#:64) MassPeaks:326
RawMode:Averaged 4.310-4.320(63-65) BasePeak:91.05(201954)
BG Mode:Calc. from Peak Group 1 - Event 1 Scan

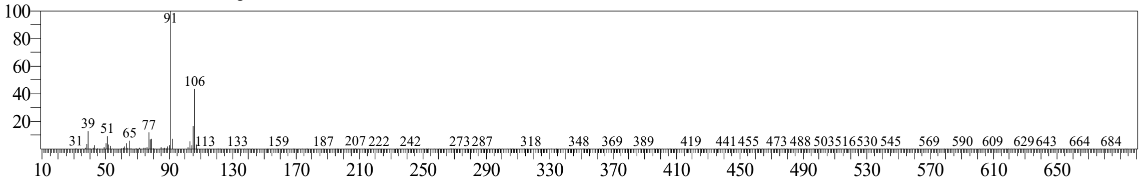

Hit#:1 Entry:2687 Library:NIST11.lib
SI:95 Formula:C8H10 CAS:95-47-6 MolWeight:106 RetIndex:907
CompName:o-Xylene $$ Benzene, 1,2-dimethyl- $$ o-Dimethylbenzene $$ o-Methyltoluene $$ o-Xylol $$ 1,2-Dimethylbenzene $$ 1,2-Xylene $$ 3,4-Xylene $

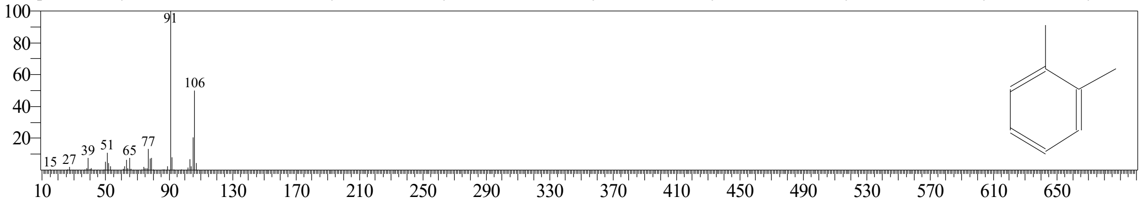

Hit#:2 Entry:2685 Library:NIST11.lib
SI:95 Formula:C8H10 CAS:108-38-3 MolWeight:106 RetIndex:907
CompName:Benzene, 1,3-dimethyl- $$ m-Xylene $$ m-Dimethylbenzene $$ m-Xylol $$ 1,3-Dimethylbenzene $$ 1,3-Xylene $$ 2,4-Xylene $$ m-Methyltoluene

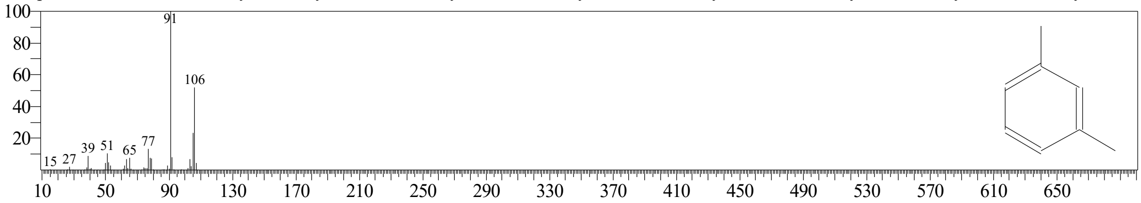

Hit#:3 Entry:2686 Library:NIST11.lib
SI:93 Formula:C8H10 CAS:106-42-3 MolWeight:106 RetIndex:907
CompName:p-Xylene $$ Benzene, 1,4-dimethyl- $$ p-Dimethylbenzene $$ p-Xylol $$ 1,4-Dimethylbenzene $$ 1,4-Xylene $$ p-Methyltoluene $$ para-Xylene $

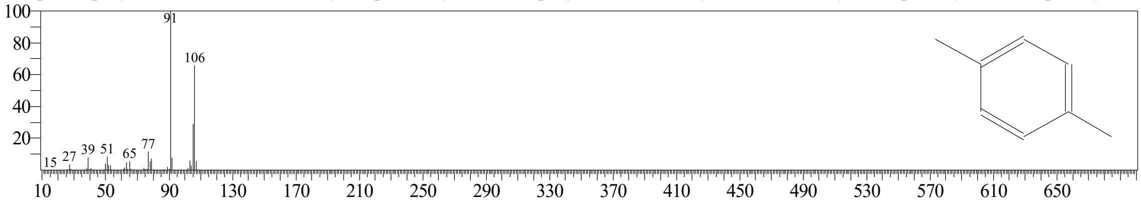

Hit#:4 Entry:2683 Library:NIST11.lib
SI:91 Formula:C8H10 CAS:100-41-4 MolWeight:106 RetIndex:893
CompName:Ethylbenzene $$ Benzene, ethyl- $$ Ethylbenzol $$ EB $$ Phenylethane $$ Aethylbenzol $$ Ethylbenzeen $$ Etilbenzene $$ Etylobenzen $$ NCI-C

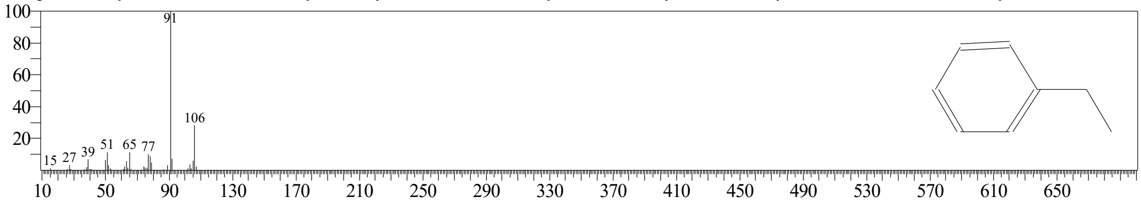

Hit#:5 Entry:2682 Library:NIST11.lib
SI:88 Formula:C8H10 CAS:2175-91-9 MolWeight:106 RetIndex:824
CompName:1,3-Cyclopentadiene, 5-(1-methylethylidene)- $$ 1,3-Cyclopentadiene, 5-isopropylidene- $$ Dimethylfulvene $$ Isopropylidenecyclopentadiene $$

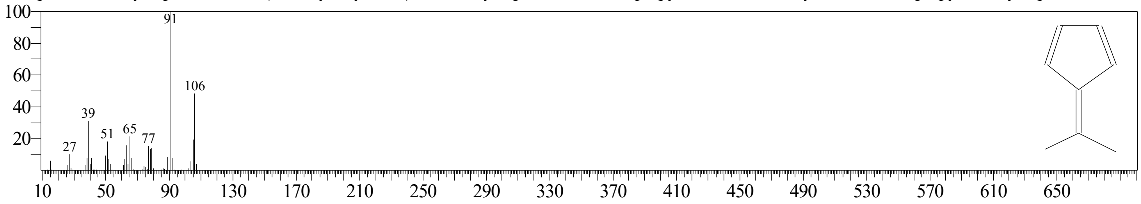

<< Target >>
Line#:5 R.Time:4.550(Scan#:111) MassPeaks:408
RawMode:Averaged 4.545-4.555(110-112) BasePeak:43.10(678743)
BG Mode:Calc. from Peak Group 1 - Event 1 Scan

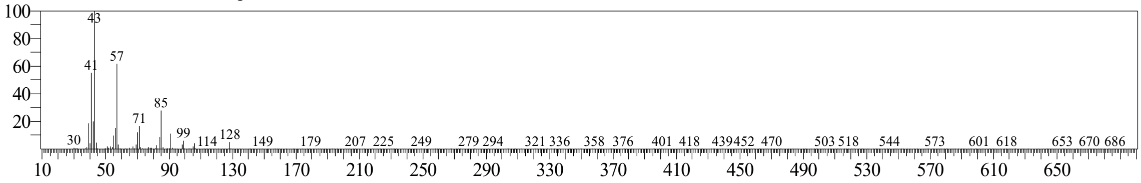

Hit#:1 Entry:7416 Library:NIST11.lib
SI:91 Formula:C9H20 CAS:111-84-2 MolWeight:128 RetIndex:916
CompName:Nonane $$ n-Nonane $$ Shellsol 140 $$ n-C9H20 $$ UN 1920 $$

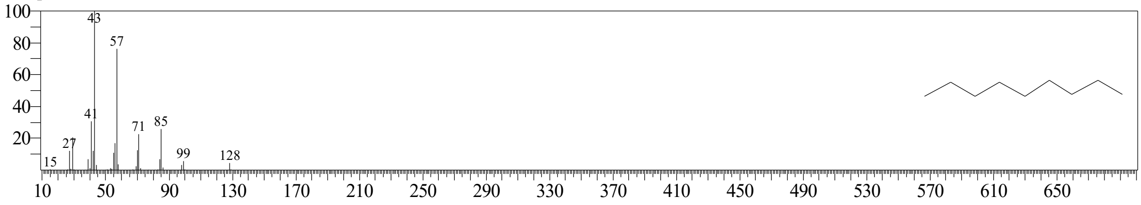

Hit#:2 Entry:4236 Library:NIST11.lib
SI:90 Formula:C8H18 CAS:589-43-5 MolWeight:114 RetIndex:688
CompName:Hexane, 2,4-dimethyl- $$ 2,4-Dimethylhexane $$

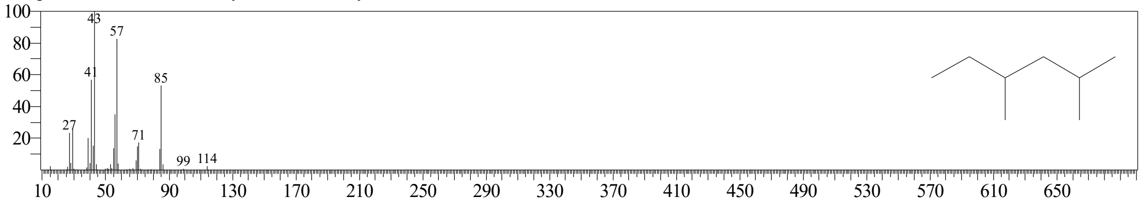

Hit#:3 Entry:12306 Library:NIST11.lib
SI:89 Formula:C10H22 CAS:871-83-0 MolWeight:142 RetIndex:951
CompName:Nonane, 2-methyl- $$ 2-Methylnonane $$

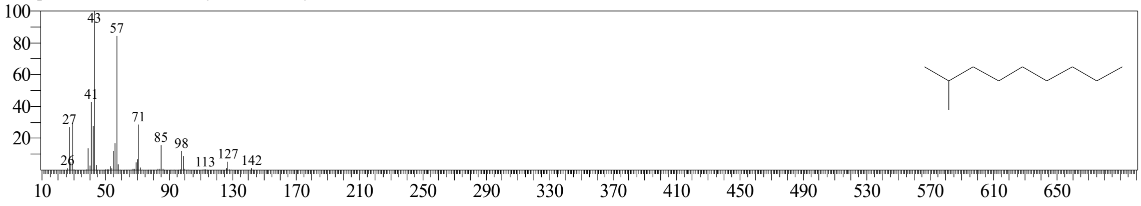

Hit#:4 Entry:12325 Library:NIST11.lib
SI:89 Formula:C10H22 CAS:13475-81-5 MolWeight:142 RetIndex:846
CompName:Hexane, 2,2,3,3-tetramethyl- $$ 2,2,3,3-Tetramethylhexane $$

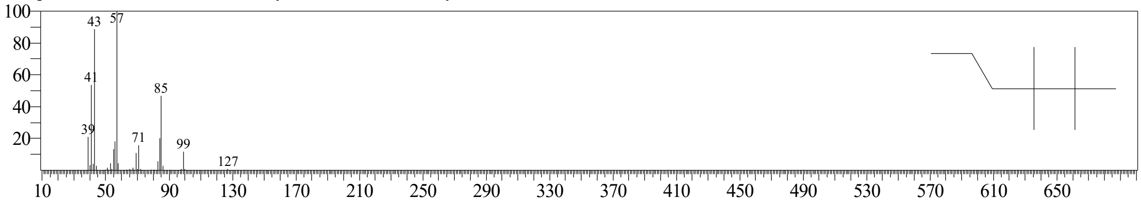

Hit#:5 Entry:12315 Library:NIST11.lib
SI:88 Formula:C10H22 CAS:2613-61-8 MolWeight:142 RetIndex:823
CompName:Heptane, 2,4,6-trimethyl- $$ 2,4,6-Trimethylheptane $$

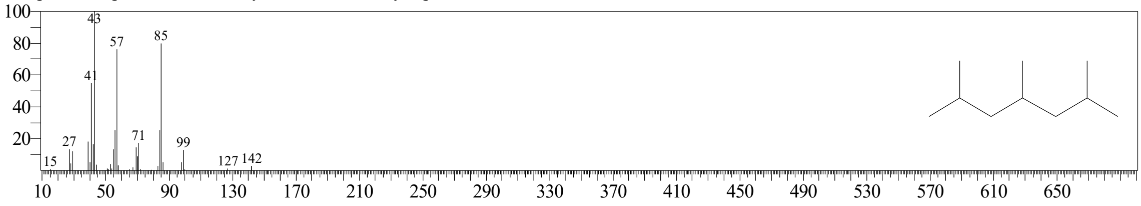

<< Target >>
Line#:6 R.Time:4.740(Scan#:149) MassPeaks:355
RawMode:Averaged 4.735-4.745(148-150) BasePeak:43.05(973338)
BG Mode:Calc. from Peak Group 1 - Event 1 Scan

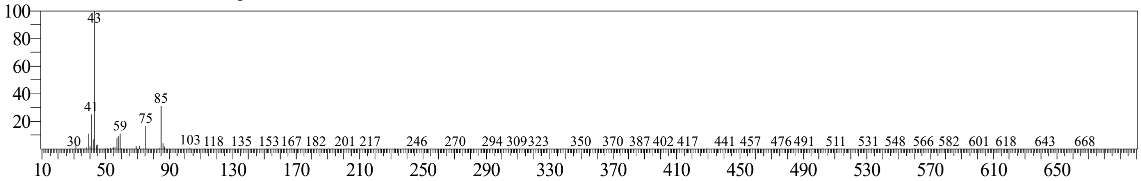

Hit#:1 Entry:7299 Library:NIST11.lib
SI:88 Formula:C8H16O CAS:26118-38-7 MolWeight:128 RetIndex:868
CompName:2-Hexanone, 3,3-dimethyl- $$ 3,3-Dimethyl-2-hexanone # $$

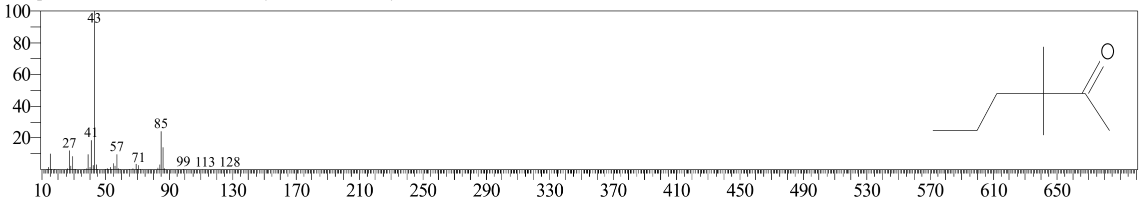

Hit#:2 Entry:8164 Library:NIST11.lib
SI:87 Formula:C6H13NO2 CAS:14255-44-8 MolWeight:131 RetIndex:935
CompName:Hexane, 2-nitro- $$ 2-Nitrohexane # $$

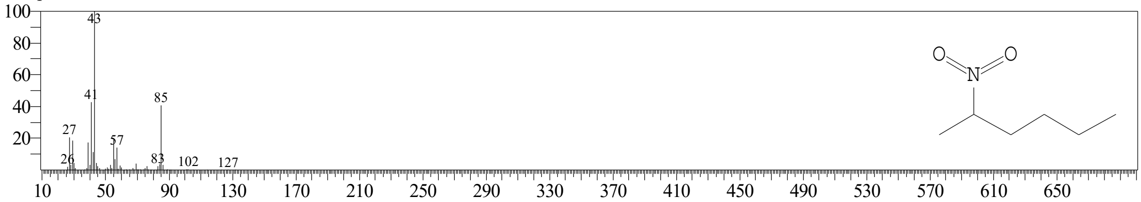

Hit#:3 Entry:51590 Library:NIST11.lib
SI:86 Formula:C5H9IO CAS:3695-29-2 MolWeight:212 RetIndex:1069
CompName:5-Iodopentan-2-one $$ 5-Iodo-2-pentanone # $$

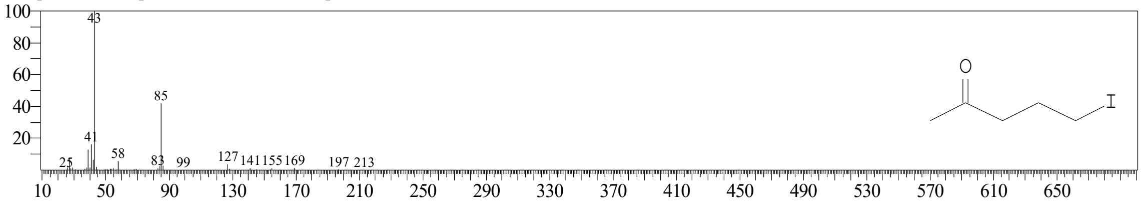

Hit#:4 Entry:4540 Library:NIST11.lib
SI:86 Formula:C6H12O2 CAS:25910-96-7 MolWeight:116 RetIndex:815
CompName:3-Oxetanol, 2,2,3-trimethyl- $$ 2,2,3-Trimethyl-3-oxetanol # $$

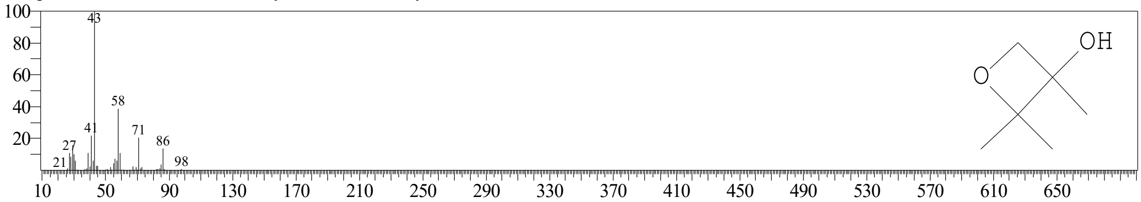

Hit#:5 Entry:4129 Library:NIST11.lib
SI:86 Formula:C7H14O CAS:34061-75-1 MolWeight:114 RetIndex:783
CompName:Ether, 3-butenyl propyl $$ 3-Butenyl propyl ether # $$

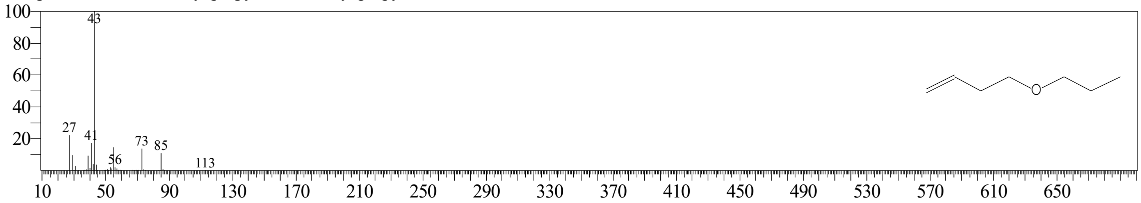

<< Target >>
Line#:7 R.Time:4.940(Scan#:189) MassPeaks:378
RawMode:Averaged 4.935-4.945(188-190) BasePeak:43.05(988043)
BG Mode:Calc. from Peak Group 1 - Event 1 Scan

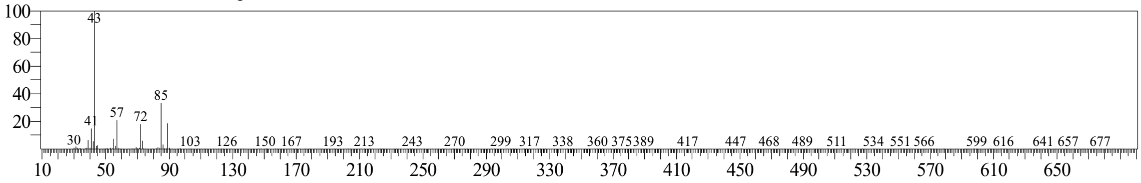

Hit#:1 Entry:3988 Library:NIST11.lib
SI:88 Formula:C6H10O2 CAS:17257-81-7 MolWeight:114 RetIndex:806
CompName:Ethanone, 1-(3-ethyloxiranyl)- $$ 2-Hexanone, 3,4-epoxy- $$ 1-(3-Ethyl-2-oxiranyl)ethanone # $$

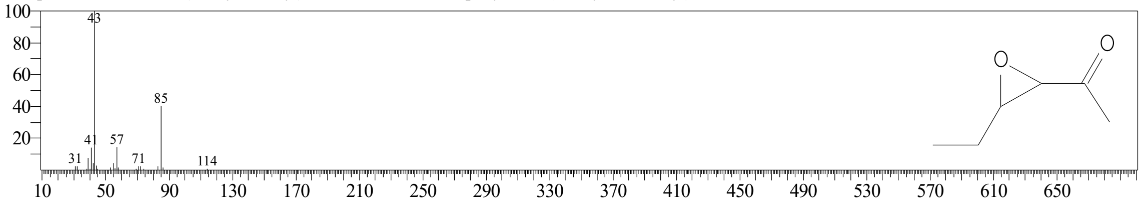

Hit#:2 Entry:7295 Library:NIST11.lib
SI:86 Formula:C8H16O CAS:2371-19-9 MolWeight:128 RetIndex:888
CompName:2-Heptanone, 3-methyl- $$ 3-Methyl-2-heptanone $$ 3-Methylheptan-2-one $$

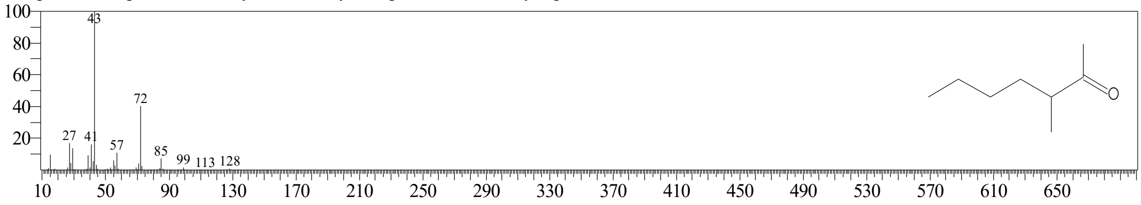

Hit#:3 Entry:18593 Library:NIST11.lib
SI:86 Formula:C10H20O CAS:107995-55-1 MolWeight:156 RetIndex:1081
CompName:Hexane, 1-(3-butenyloxy)- $$ 3-Butenyl hexyl ether # $$

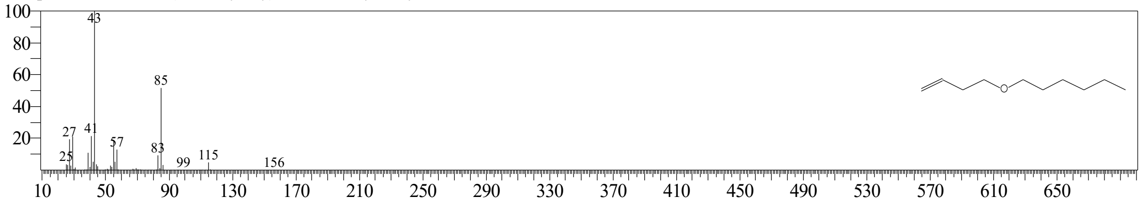

Hit#:4 Entry:12929 Library:NIST11.lib
SI:86 Formula:C8H16O2 CAS:30536-44-8 MolWeight:144 RetIndex:1028
CompName:4-Butoxy-2-butanone

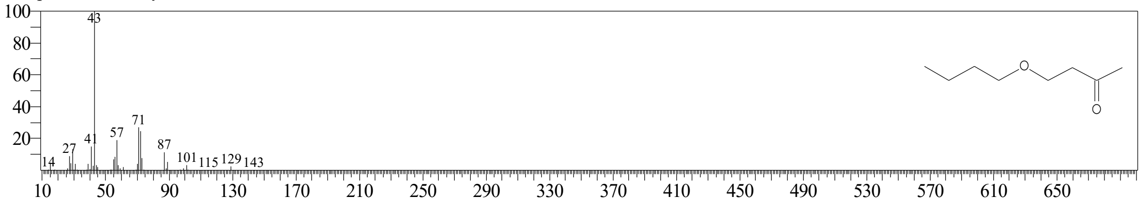

Hit#:5 Entry:7299 Library:NIST11.lib
SI:86 Formula:C8H16O CAS:26118-38-7 MolWeight:128 RetIndex:868
CompName:2-Hexanone, 3,3-dimethyl- $$ 3,3-Dimethyl-2-hexanone # $$

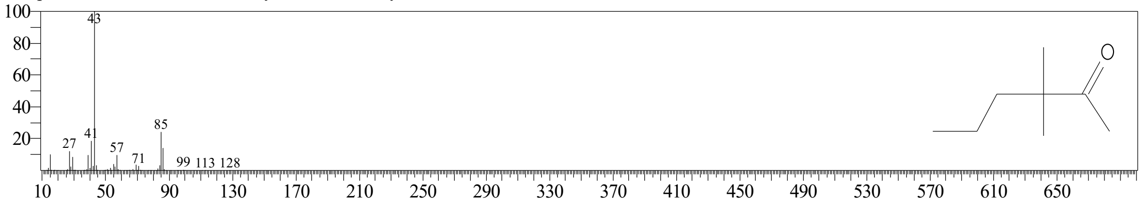

<< Target >>
Line#:8 R.Time:5.230(Scan#:247) MassPeaks:320
RawMode:Averaged 5.225-5.235(246-248) BasePeak:43.05(32149)
BG Mode:Calc. from Peak Group 1 - Event 1 Scan

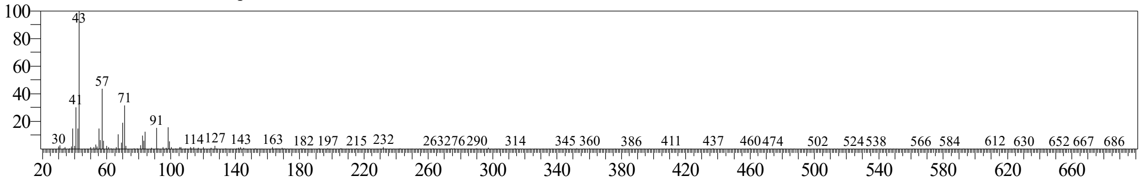

Hit#:1 Entry:12306 Library:NIST11.lib
SI:83 Formula:C10H22 CAS:871-83-0 MolWeight:142 RetIndex:951
CompName:Nonane, 2-methyl- $$ 2-Methylnonane $$

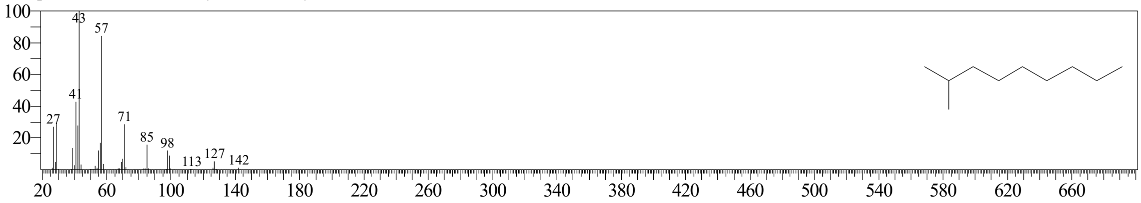

Hit#:2 Entry:7436 Library:NIST11.lib
SI:82 Formula:C9H20 CAS:16747-30-1 MolWeight:128 RetIndex:767
CompName:Hexane, 2,4,4-trimethyl- $$ 2,4,4-Trimethylhexane $$

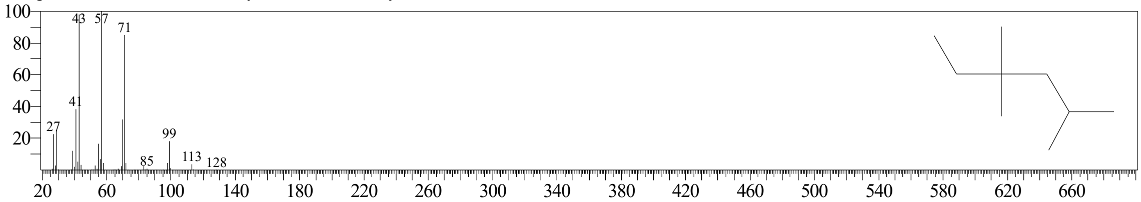

Hit#:3 Entry:12327 Library:NIST11.lib
SI:82 Formula:C10H22 CAS:52896-95-4 MolWeight:142 RetIndex:823
CompName:Heptane, 2,3,4-trimethyl- $$ 2,3,4-Trimethylheptane $$

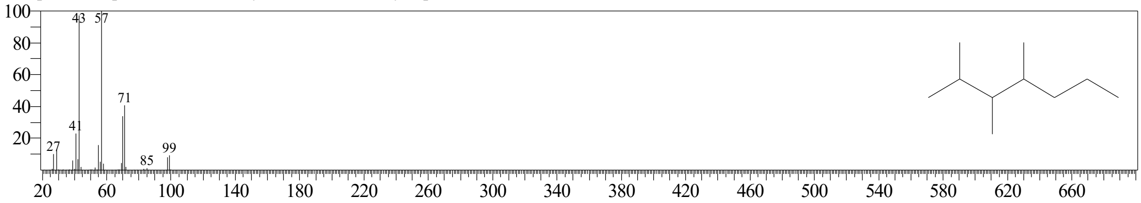

Hit#:4 Entry:7413 Library:NIST11.lib
SI:82 Formula:C9H20 CAS:921-47-1 MolWeight:128 RetIndex:724
CompName:Hexane, 2,3,4-trimethyl- $$ 2,3,4-Trimethylhexane $$

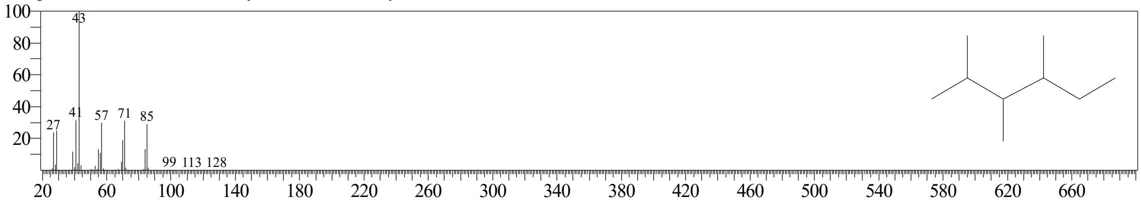

Hit#:5 Entry:43975 Library:NIST11.lib
SI:82 Formula:C12H24O2 CAS:35061-61-1 MolWeight:200 RetIndex:1253
CompName:Propanoic acid, 2-methyl-, 2-ethylhexyl ester $$ Isobutyric acid, 2-ethylhexyl ester $$ 2-Ethylhexyl 2-methylpropanoate # $$

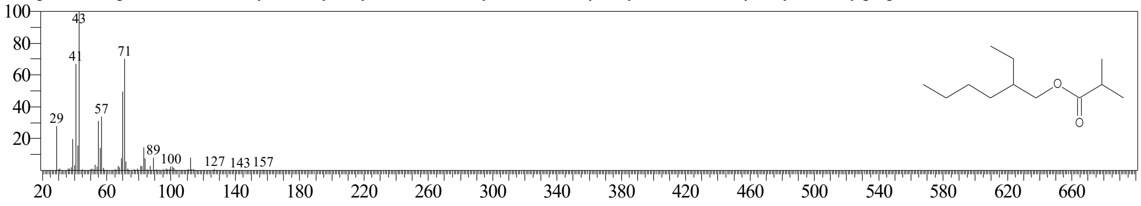

<< Target >>
Line#:9 R.Time:5.270(Scan#:255) MassPeaks:295
RawMode:Averaged 5.265-5.275(254-256) BasePeak:43.05(44421)
BG Mode:Calc. from Peak Group 1 - Event 1 Scan

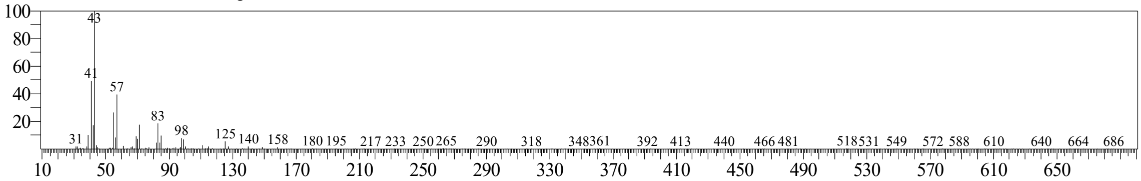

Hit#:1 Entry:96490 Library:NIST11.lib
SI:86 Formula:C15H28O4 CAS:0-00-0 MolWeight:272 RetIndex:1847
CompName:Oxalic acid, decyl propyl ester

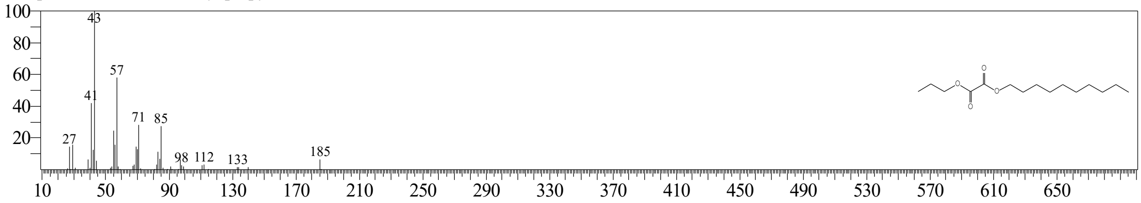

Hit#:2 Entry:150228 Library:NIST11.lib
SI:86 Formula:C20H38O4 CAS:0-00-0 MolWeight:342 RetIndex:2344
CompName:Oxalic acid, dodecyl hexyl ester

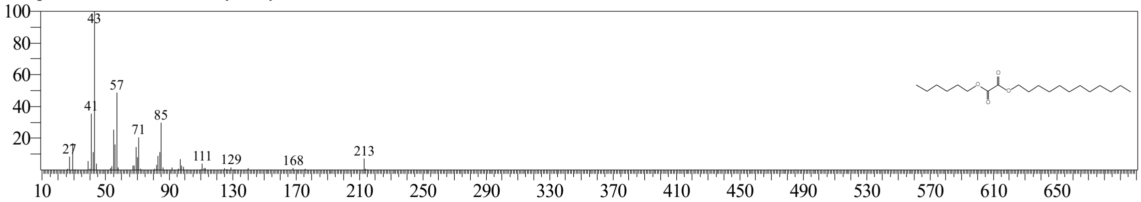

Hit#:3 Entry:26088 Library:NIST11.lib
SI:86 Formula:C12H26 CAS:1632-71-9 MolWeight:170 RetIndex:1086
CompName:Nonane, 4-ethyl-5-methyl- $$ 4-Ethyl-5-methylnonane # $$

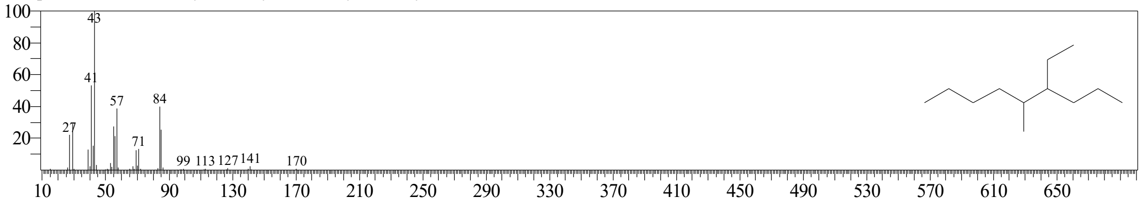

Hit#:4 Entry:51051 Library:NIST11.lib
SI:85 Formula:C15H30 CAS:59920-26-2 MolWeight:210 RetIndex:1287
CompName:2,4,6,8-Tetramethyl-1-undecene

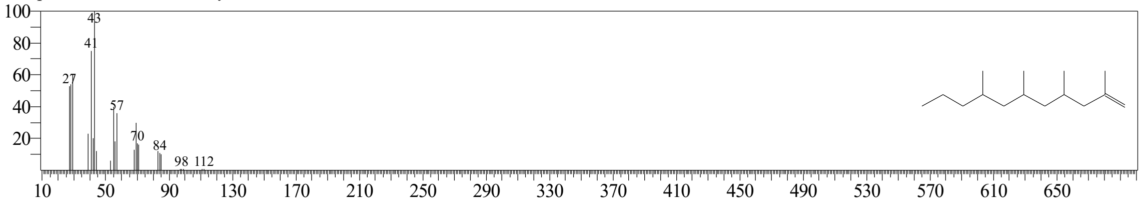

Hit#:5 Entry:12306 Library:NIST11.lib
SI:84 Formula:C10H22 CAS:871-83-0 MolWeight:142 RetIndex:951
CompName:Nonane, 2-methyl- $$ 2-Methylnonane $$

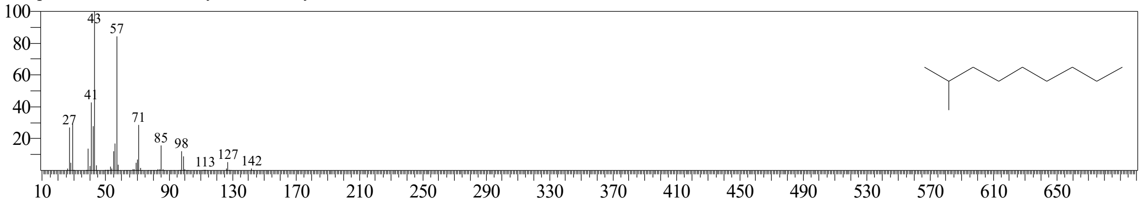

<< Target >>
Line#:10 R.Time:5.340(Scan#:269) MassPeaks:334
RawMode:Averaged 5.335-5.345(268-270) BasePeak:71.05(17723)
BG Mode:Calc. from Peak Group 1 - Event 1 Scan

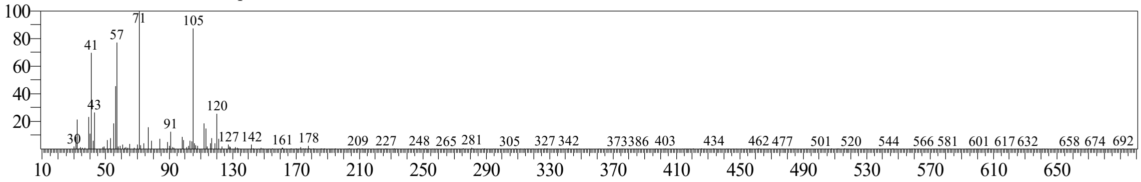

Hit#:1 Entry:38191 Library:NIST11.lib
SI:71 Formula:C8H17Br CAS:999-06-4 MolWeight:192 RetIndex:1049
CompName:Octane, 4-bromo- $$ 4-Bromooctane $$

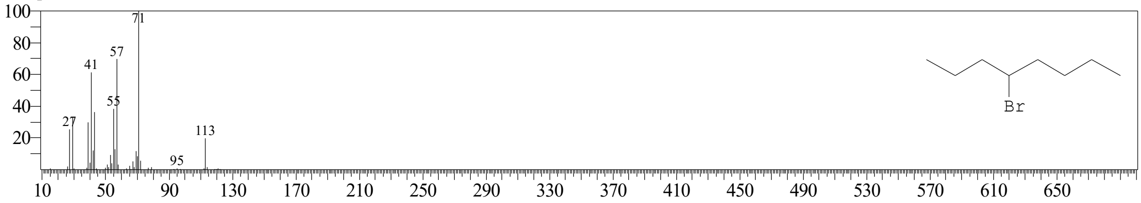

Hit#:2 Entry:12310 Library:NIST11.lib
SI:70 Formula:C10H22 CAS:13475-78-0 MolWeight:142 RetIndex:887
CompName:Heptane, 5-ethyl-2-methyl- $$ 5-Ethyl-2-methylheptane $$ Heptane, 3-ethyl-6-methyl $$

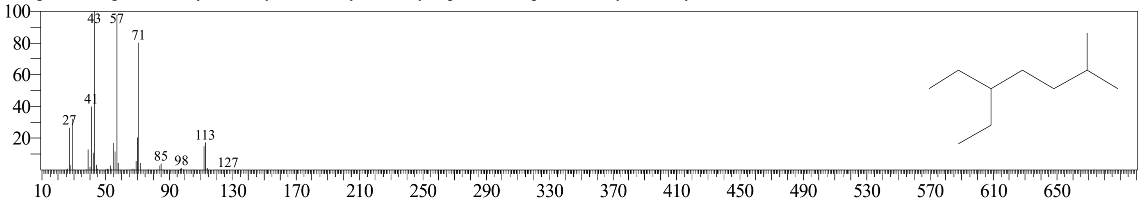

Hit#:3 Entry:6604 Library:NIST11.lib
SI:70 Formula:C8H14O CAS:17123-63-6 MolWeight:126 RetIndex:913
CompName:2,5-Dimethyl-1,5-hexadien-3-ol

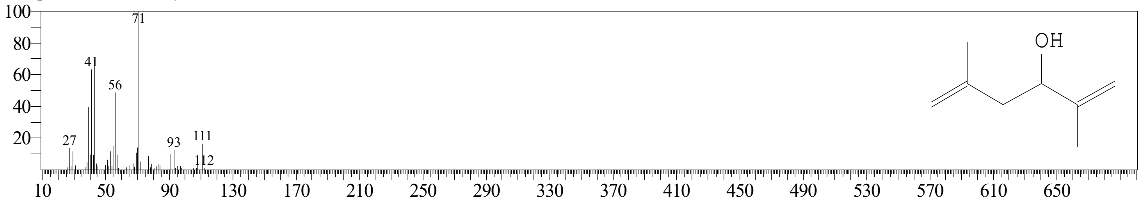

Hit#:4 Entry:71583 Library:NIST11.lib
SI:69 Formula:C8H17I CAS:557-36-8 MolWeight:240 RetIndex:1167
CompName:Octane, 2-iodo- $$ sec-Octyl Iodide $$ 2-Iodooctane $$ 2-Octyl iodide $$

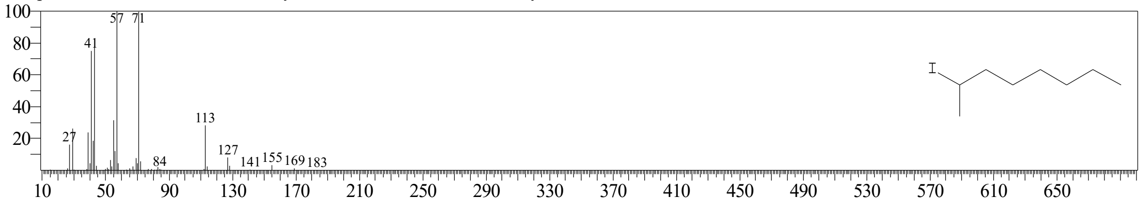

Hit#:5 Entry:38188 Library:NIST11.lib
SI:69 Formula:C8H17Br CAS:999-64-4 MolWeight:192 RetIndex:1049
CompName:3-Bromooctane $$ Octane, 3-bromo- $$

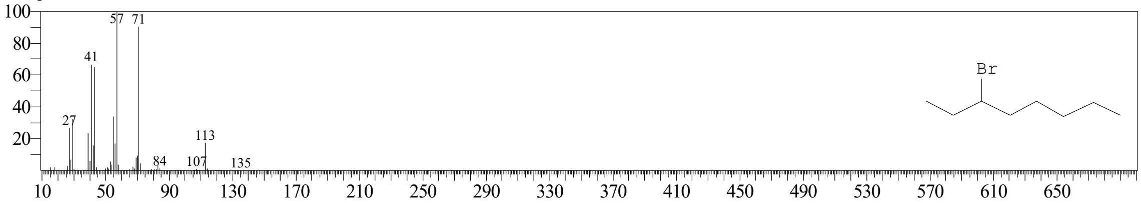

<< Target >>
Line#:11 R.Time:5.460(Scan#:293) MassPeaks:359
RawMode:Averaged 5.455-5.465(292-294) BasePeak:43.05(484290)
BG Mode:Calc. from Peak Group 1 - Event 1 Scan

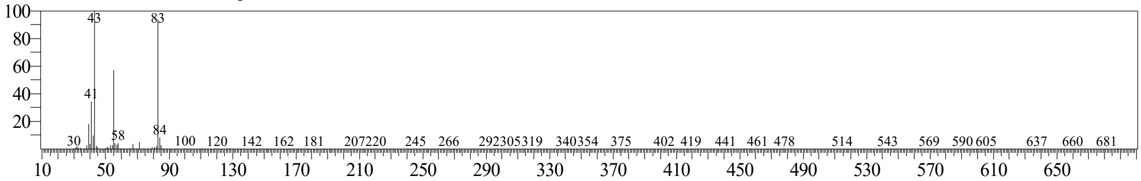

Hit#:1 Entry:6418 Library:NIST11.lib
SI:86 Formula:C7H10O2 CAS:15121-02-5 MolWeight:126 RetIndex:920
CompName:Cyclopentane, 1-acetyl-1,2-epoxy- $$ 1-(6-Oxabicyclo[3.1.0]hex-1-yl)ethanone # $$

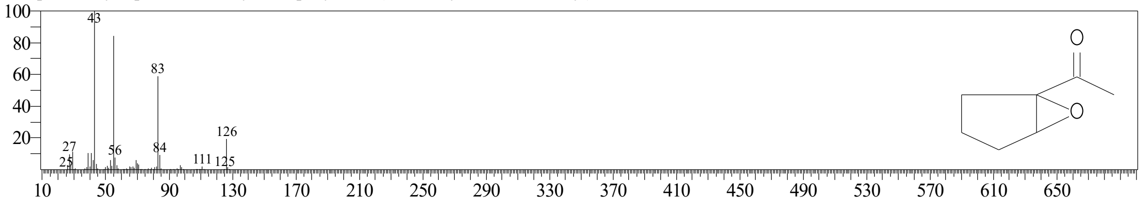

Hit#:2 Entry:6711 Library:NIST11.lib
SI:86 Formula:C9H18 CAS:55702-61-9 MolWeight:126 RetIndex:775
CompName:2-Hexene, 4,4,5-trimethyl- $$ (2E)-4,4,5-Trimethyl-2-hexene # $$

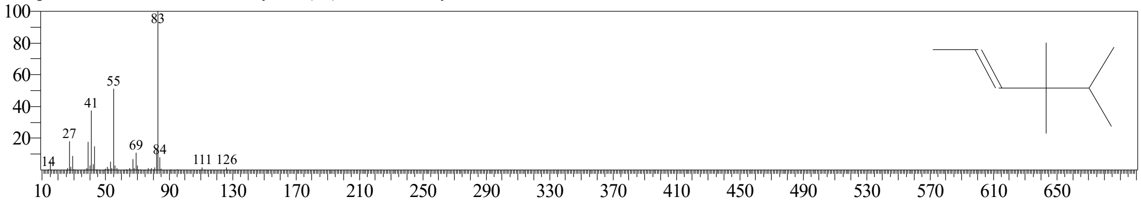

Hit#:3 Entry:63462 Library:NIST11.lib
SI:86 Formula:C12H20O4 CAS:0-00-0 MolWeight:228 RetIndex:1548
CompName:Oxalic acid, cyclohexyl isobutyl ester

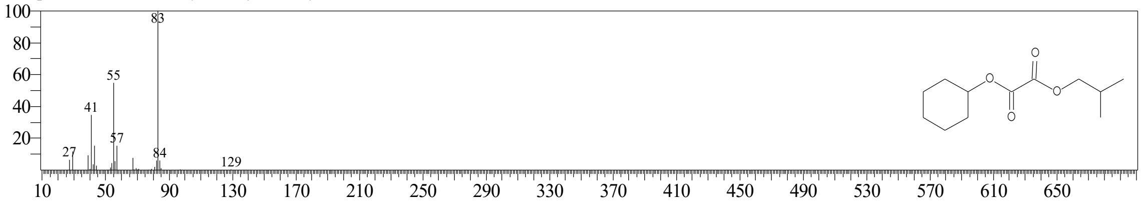

Hit#:4 Entry:1702 Library:NIST11.lib
SI:85 Formula:C6H10O CAS:763-93-9 MolWeight:98 RetIndex:762
CompName:3-Hexen-2-one $$ 1-Butenyl methyl ketone $$ Methyl 1-butenyl ketone $$ Hex-3-en-2-one $$

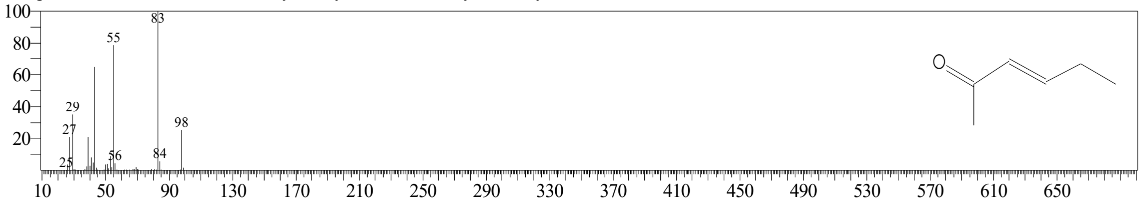

Hit#:5 Entry:53303 Library:NIST11.lib
SI:85 Formula:C11H18O4 CAS:0-00-0 MolWeight:214 RetIndex:1513
CompName:Oxalic acid, cyclohexyl propyl ester

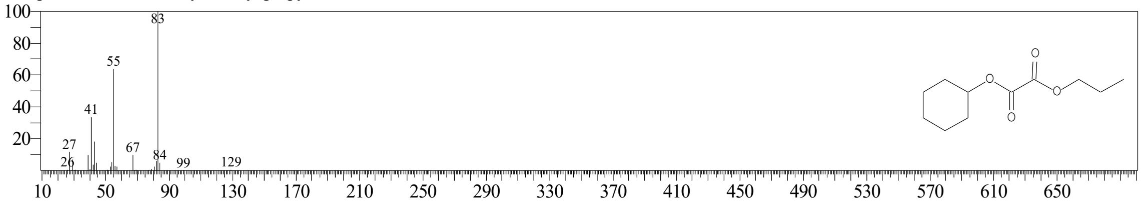

<< Target >>
Line#:12 R.Time:5.570(Scan#:315) MassPeaks:383
RawMode:Averaged 5.565-5.575(314-316) BasePeak:41.05(911169)
BG Mode:Calc. from Peak Group 1 - Event 1 Scan

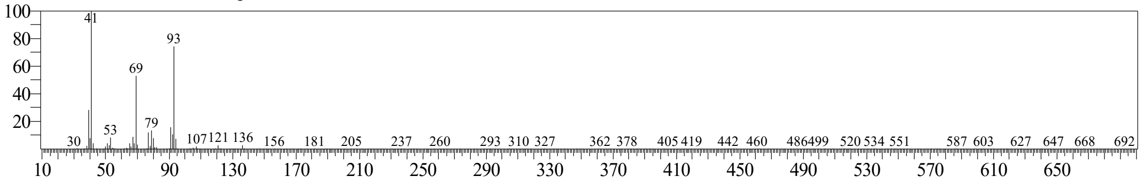

Hit#:1 Entry:9719 Library:NIST11.lib
SI:94 Formula:C10H16 CAS:123-35-3 MolWeight:136 RetIndex:958
CompName:.beta.-Myrcene $$ 1,6-Octadiene, 7-methyl-3-methylene- $$ Myrcene $$ 7-Methyl-3-methylene-1,6-octadiene $$ 7-Methyl-3-methyleneoctadiene-(1

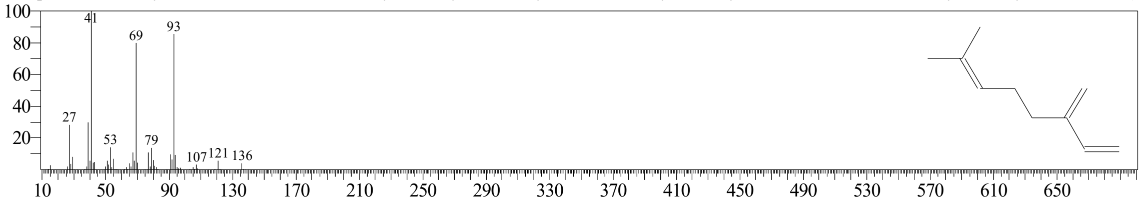

Hit#:2 Entry:9777 Library:NIST11.lib
SI:87 Formula:C10H16 CAS:127-91-3 MolWeight:136 RetIndex:943
CompName:.beta.-Pinene $$ Bicyclo[3.1.1]heptane, 6,6-dimethyl-2-methylene- $$ 2(10)-Pinene $$ Nopinen $$ Nopinene $$ Pseudopinen $$ Pseudopinene $$

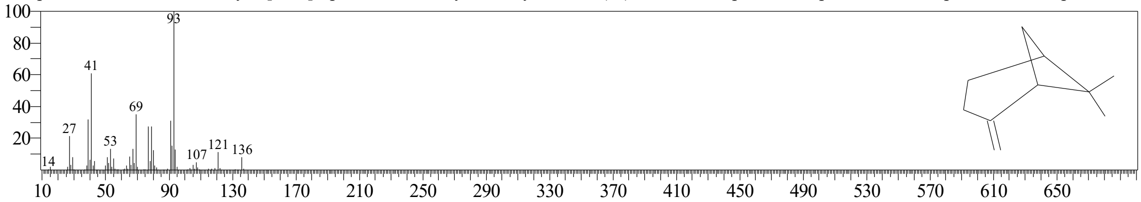

Hit#:3 Entry:9776 Library:NIST11.lib
SI:87 Formula:C10H16 CAS:18172-67-3 MolWeight:136 RetIndex:943
CompName:Bicyclo[3.1.1]heptane, 6,6-dimethyl-2-methylene-, (1S)- $$ 2(10)-Pinene, (1S,5S)-(-)- $$ (-)-.beta.-Pinene $$ (-)-2(10)-Pinene $$ L-.beta.-Pinene $

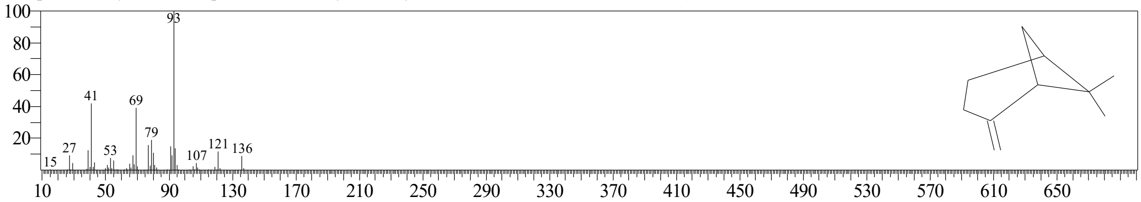

Hit#:4 Entry:43737 Library:NIST11.lib
SI:87 Formula:C10H16S2 CAS:73188-23-5 MolWeight:200 RetIndex:1555
CompName:4-(4-Methylpent-3-enyl)-3,6-dihydro-1,2-dithiin $$ 4-(4-Methyl-3-pentenyl)-3,6-dihydro-1,2-dithiine # $$

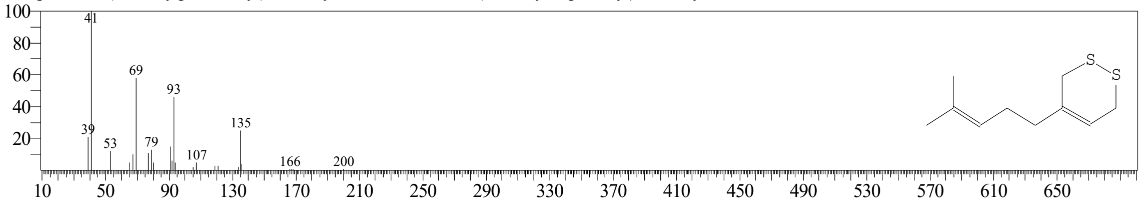

Hit#:5 Entry:54444 Library:NIST11.lib
SI:86 Formula:C10H17Br CAS:35719-26-7 MolWeight:216 RetIndex:1282
CompName:1-Bromo-3,7-dimethyl-2,6-octadiene $$ 2,6-Octadiene, 1-bromo-3,7-dimethyl- $$

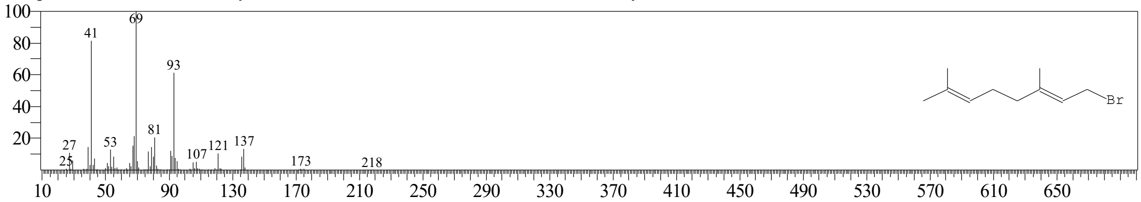

<< Target >>
Line#:13 R.Time:5.665(Scan#:334) MassPeaks:339
RawMode:Averaged 5.660-5.670(333-335) BasePeak:43.05(125224)
BG Mode:Calc. from Peak Group 1 - Event 1 Scan

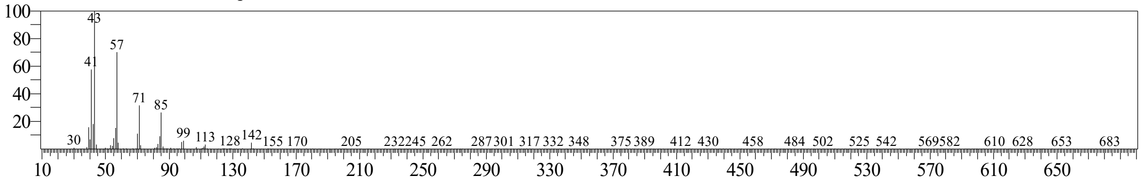

Hit#:1 Entry:7416 Library:NIST11.lib
SI:92 Formula:C9H20 CAS:111-84-2 MolWeight:128 RetIndex:916
CompName:Nonane $$ n-Nonane $$ Shellsol 140 $$ n-C9H20 $$ UN 1920 $$

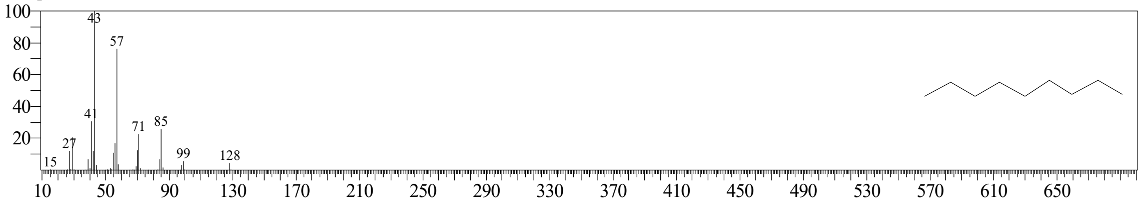

Hit#:2 Entry:12306 Library:NIST11.lib
SI:91 Formula:C10H22 CAS:871-83-0 MolWeight:142 RetIndex:951
CompName:Nonane, 2-methyl- $$ 2-Methylnonane $$

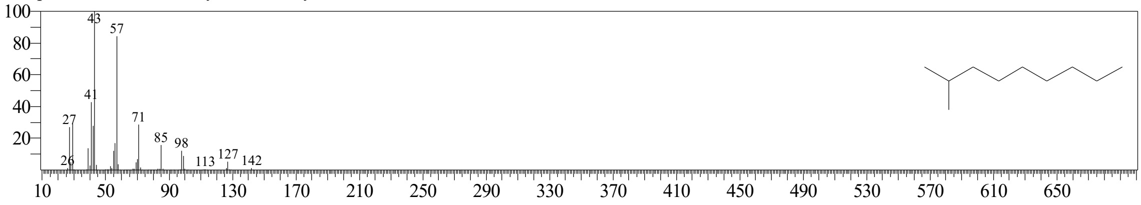

Hit#:3 Entry:26094 Library:NIST11.lib
SI:90 Formula:C12H26 CAS:1002-17-1 MolWeight:170 RetIndex:1086
CompName:Decane, 2,9-dimethyl- $$ 2,9-Dimethyldecane $$

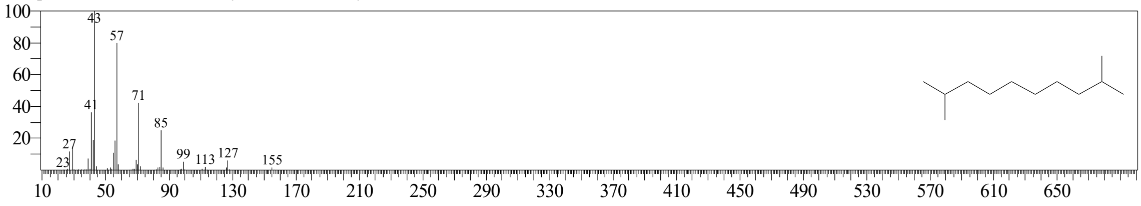

Hit#:4 Entry:4236 Library:NIST11.lib
SI:90 Formula:C8H18 CAS:589-43-5 MolWeight:114 RetIndex:688
CompName:Hexane, 2,4-dimethyl- $$ 2,4-Dimethylhexane $$

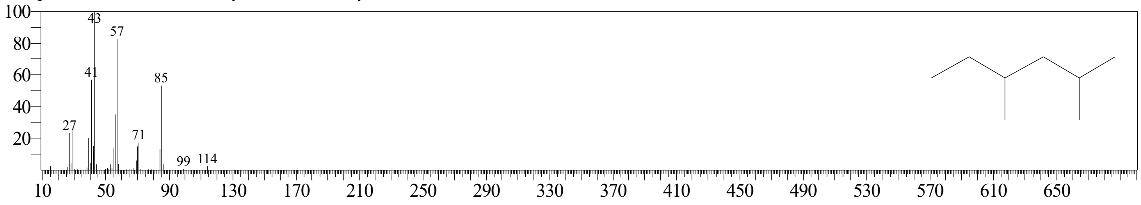

Hit#:5 Entry:12320 Library:NIST11.lib
SI:90 Formula:C10H22 CAS:124-18-5 MolWeight:142 RetIndex:1015
CompName:Decane $$ n-Decane $$ n-C10H22 $$ UN 2247 $$

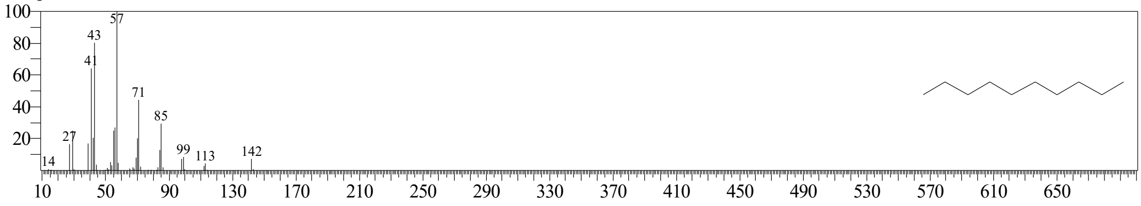

<< Target >>
Line#:14 R.Time:5.710(Scan#:343) MassPeaks:365
RawMode:Averaged 5.705-5.715(342-344) BasePeak:105.10(75569)
BG Mode:Calc. from Peak Group 1 - Event 1 Scan

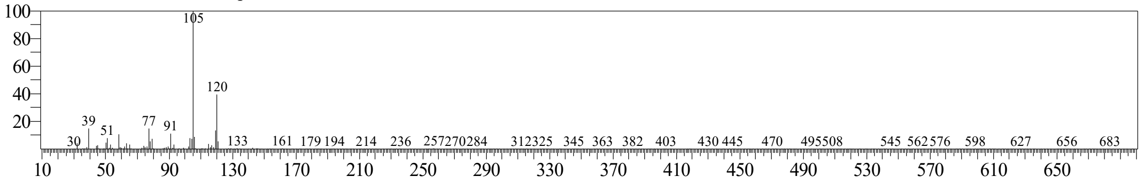

Hit#:1 Entry:5337 Library:NIST11.lib
SI:93 Formula:C9H12 CAS:95-63-6 MolWeight:120 RetIndex:1020
CompName:Benzene, 1,2,4-trimethyl- $$ .psi.-Cumene $$ aS-Trimethylbenzene $$ Pseudocumene $$ Pseudocumol $$ 1,2,4-Trimethylbenzene $$ 1,2,5-Trimeth

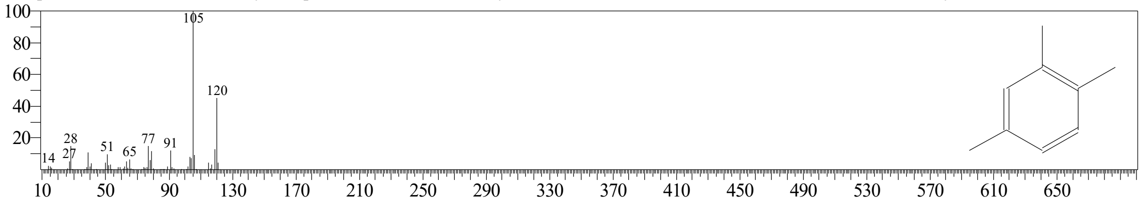

Hit#:2 Entry:5339 Library:NIST11.lib
SI:91 Formula:C9H12 CAS:526-73-8 MolWeight:120 RetIndex:1020
CompName:Benzene, 1,2,3-trimethyl- $$ Hemimellitene $$ 1,2,3-Trimethylbenzene $$ Hemellitol $$ 1,2,3-Trimethyl benzene $$ 1,2,3-Trimethylbenzene $$

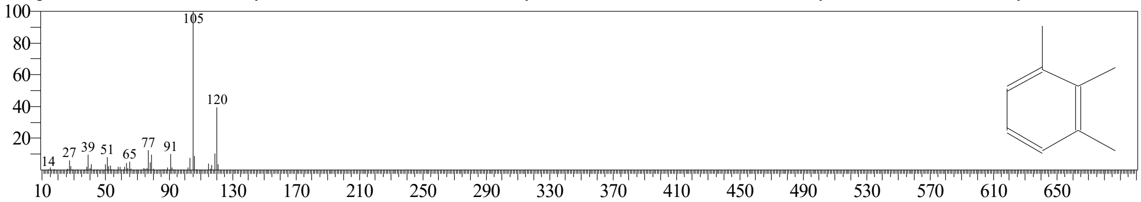

Hit#:3 Entry:5343 Library:NIST11.lib
SI:89 Formula:C9H12 CAS:620-14-4 MolWeight:120 RetIndex:1006
CompName:Benzene, 1-ethyl-3-methyl- $$ Toluene, m-ethyl- $$ m-Ethylmethylbenzene $$ m-Ethyltoluene $$ m-Methylethylbenzene $$ 1-Ethyl-3-methylbenze

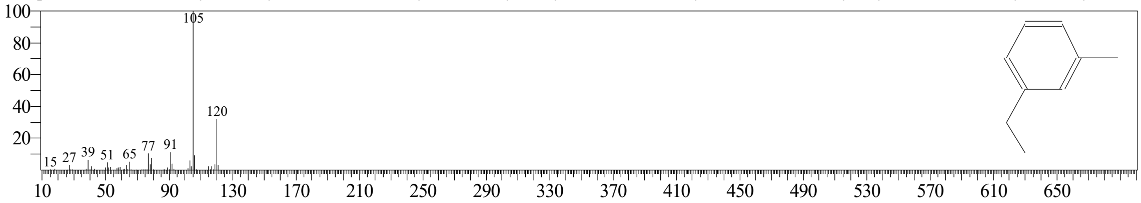

Hit#:4 Entry:5341 Library:NIST11.lib
SI:88 Formula:C9H12 CAS:611-14-3 MolWeight:120 RetIndex:1006
CompName:Benzene, 1-ethyl-2-methyl- $$ Toluene, o-ethyl- $$ o-Ethylmethylbenzene $$ o-Ethyltoluene $$ o-Methylethylbenzene $$ 1-Ethyl-2-methylbenzene

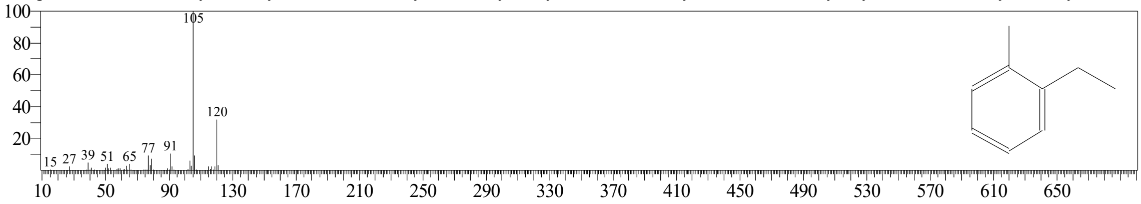

Hit#:5 Entry:5347 Library:NIST11.lib
SI:88 Formula:C9H12 CAS:108-67-8 MolWeight:120 RetIndex:1020
CompName:Mesitylene $$ Benzene, 1,3,5-trimethyl- $$ s-Trimethylbenzene $$ 1,3,5-Trimethylbenzene $$ sym-Trimethylbenzene $$ Fleet-X $$ TMB $$ UN 23

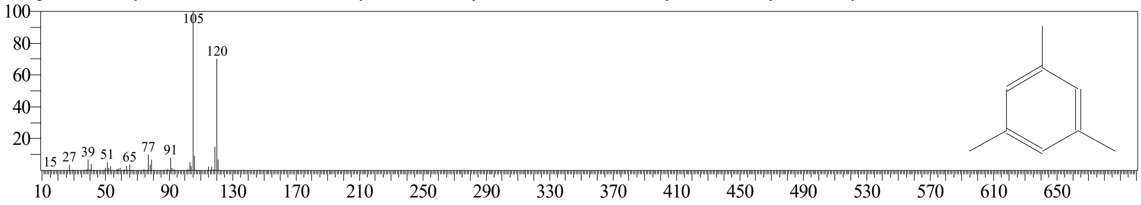

<< Target >>
Line#:15 R.Time:6.020(Scan#:405) MassPeaks:396
RawMode:Averaged 6.015-6.025(404-406) BasePeak:105.00(31667)
BG Mode:Calc. from Peak Group 1 - Event 1 Scan

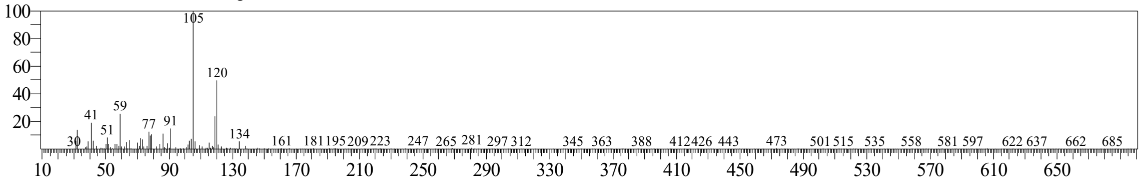

Hit#:1 Entry:5337 Library:NIST11.lib
SI:82 Formula:C9H12 CAS:95-63-6 MolWeight:120 RetIndex:1020
CompName:Benzene, 1,2,4-trimethyl- $$ .psi.-Cumene $$ aS-Trimethylbenzene $$ Pseudocumene $$ Pseudocumol $$ 1,2,4-Trimethylbenzene $$ 1,2,5-Trimeth

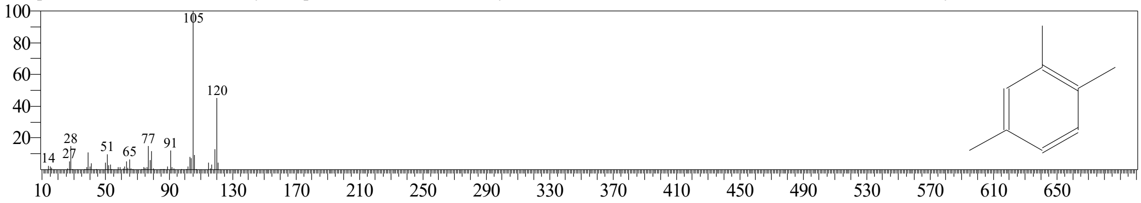

Hit#:2 Entry:5339 Library:NIST11.lib
SI:80 Formula:C9H12 CAS:526-73-8 MolWeight:120 RetIndex:1020
CompName:Benzene, 1,2,3-trimethyl- $$ Hemimellitene $$ 1,2,3-Trimethylbenzene $$ Hemellitol $$ 1,2,3-Trimethyl benzene $$ 1,2,3-Trimethylbenzene $$

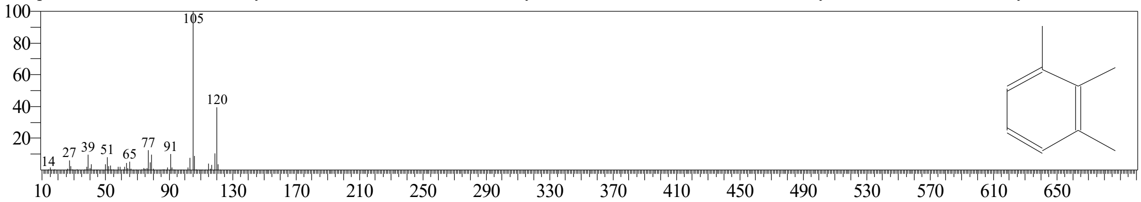

Hit#:3 Entry:5347 Library:NIST11.lib
SI:78 Formula:C9H12 CAS:108-67-8 MolWeight:120 RetIndex:1020
CompName:Mesitylene $$ Benzene, 1,3,5-trimethyl- $$ s-Trimethylbenzene $$ 1,3,5-Trimethylbenzene $$ sym-Trimethylbenzene $$ Fleet-X $$ TMB $$ UN 23

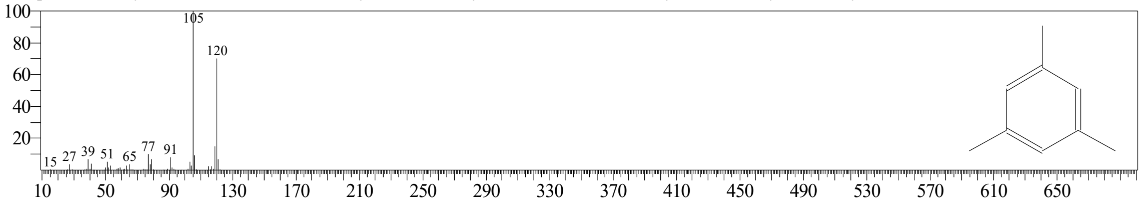

Hit#:4 Entry:5343 Library:NIST11.lib
SI:76 Formula:C9H12 CAS:620-14-4 MolWeight:120 RetIndex:1006
CompName:Benzene, 1-ethyl-3-methyl- $$ Toluene, m-ethyl- $$ m-Ethylmethylbenzene $$ m-Ethyltoluene $$ m-Methylethylbenzene $$ 1-Ethyl-3-methylbenze

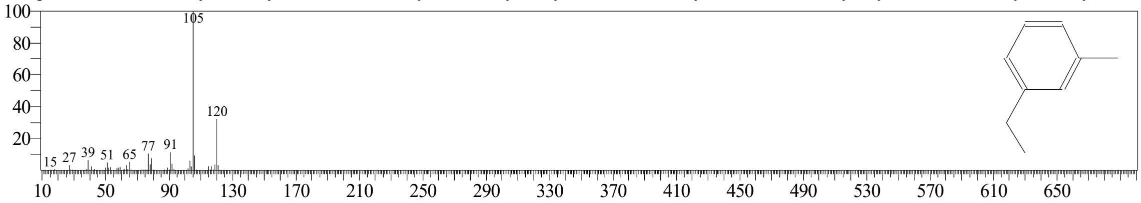

Hit#:5 Entry:5346 Library:NIST11.lib
SI:75 Formula:C9H12 CAS:622-96-8 MolWeight:120 RetIndex:1006
CompName:Benzene, 1-ethyl-4-methyl- $$ Toluene, p-ethyl- $$ p-Ethylmethylbenzene $$ p-Ethyltoluene $$ p-Methylethylbenzene $$ 1-Ethyl-4-methylbenzene

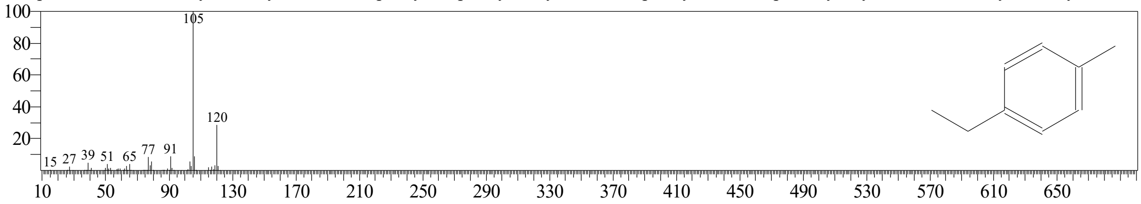

<< Target >>
Line#:16 R.Time:6.090(Scan#:419) MassPeaks:378
RawMode:Averaged 6.085-6.095(418-420) BasePeak:93.10(28392)
BG Mode:Calc. from Peak Group 1 - Event 1 Scan

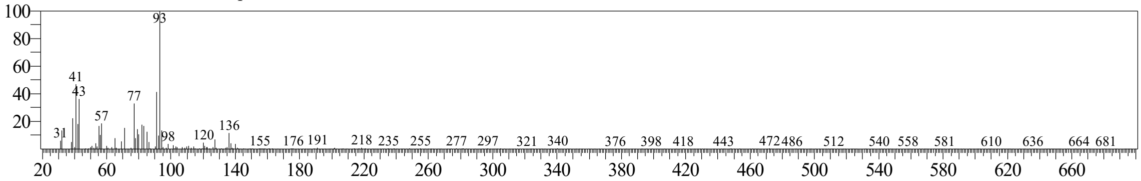

Hit#:1 Entry:9781 Library:NIST11.lib
SI:77 Formula:C10H16 CAS:3387-41-5 MolWeight:136 RetIndex:897
CompName:Bicyclo[3.1.0]hexane, 4-methylene-1-(1-methylethyl)- $$ 4(10)-Thujene $$ Sabinen $$ Sabinene $$ (+)-Sabinene $$ THUJENE, 4(10)- $$ 1-Isopro

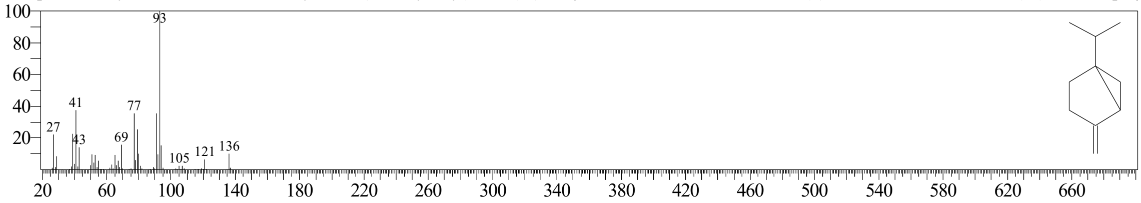

Hit#:2 Entry:17609 Library:NIST11.lib
SI:76 Formula:C10H18O CAS:17699-16-0 MolWeight:154 RetIndex:1041
CompName:Bicyclo[3.1.0]hexan-2-ol, 2-methyl-5-(1-methylethyl)-, (1.alpha.,2.alpha.,5.alpha.)- $$ trans-Sabinene hydrate (trans for Me vs IP) $$ trans-Sabinen

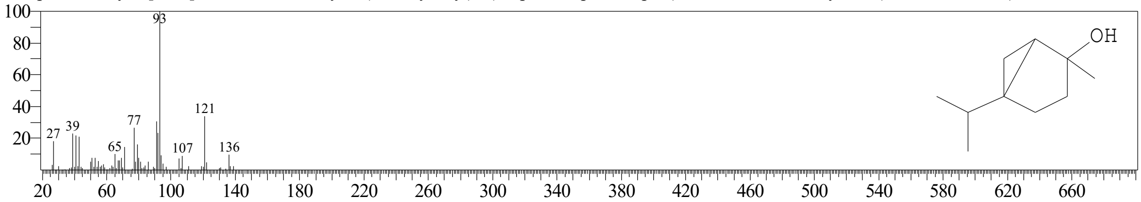

Hit#:3 Entry:9796 Library:NIST11.lib
SI:76 Formula:C10H16 CAS:68998-21-0 MolWeight:136 RetIndex:928
CompName:Cyclopropane, 1,1-dimethyl-2-(3-methyl-1,3-butadienyl)- $$ 1,1-Dimethyl-2-[(1E)-3-methyl-1,3-butadienyl]cyclopropane # $$

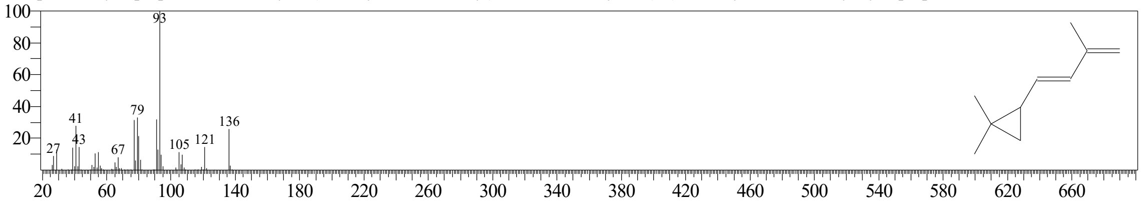

Hit#:4 Entry:30407 Library:NIST11.lib
SI:75 Formula:C12H18O CAS:0-00-0 MolWeight:178 RetIndex:1174
CompName:Bicyclo[3.1.1]hept-3-ene, 4,6,6-trimethyl-2-vinyloxy- $$ 2,6,6-Trimethyl-4-(vinyloxy)bicyclo[3.1.1]hept-2-ene # $$

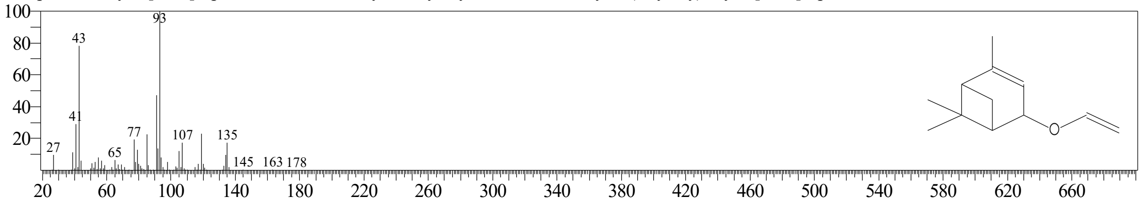

Hit#:5 Entry:41486 Library:NIST11.lib
SI:74 Formula:C12H20O2 CAS:115-95-7 MolWeight:196 RetIndex:1272
CompName:Linalyl acetate $$ 1,6-Octadien-3-ol, 3,7-dimethyl-, acetate $$ Acetic acid linalool ester $$ Bergamiol $$ Bergamol $$ Bergamot mint oil $$ Linaloo

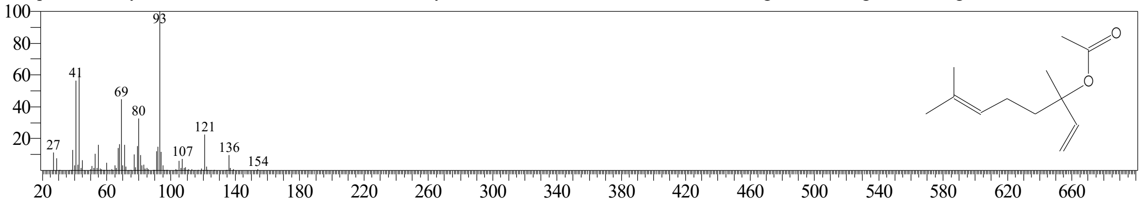

<< Target >>
Line#:17 R.Time:6.390(Scan#:479) MassPeaks:381
RawMode:Averaged 6.385-6.395(478-480) BasePeak:43.05(16764)
BG Mode:Calc. from Peak Group 1 - Event 1 Scan

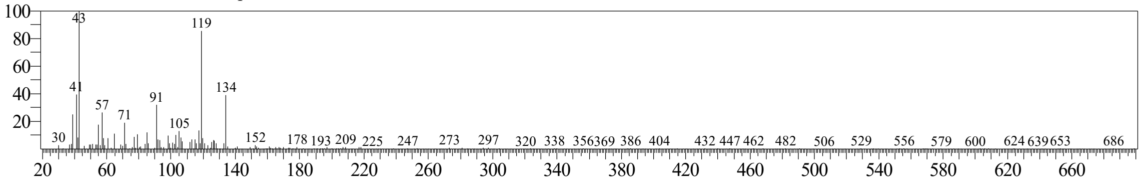

Hit#:1 Entry:38761 Library:NIST11.lib
SI:76 Formula:C13H20O CAS:0-00-0 MolWeight:192 RetIndex:1344
CompName:(+)-3-Carene, 2-(acetylmethyl)- $$ 1-(3,7,7-Trimethylbicyclo[4.1.0]hept-3-en-2-yl)acetone # $$

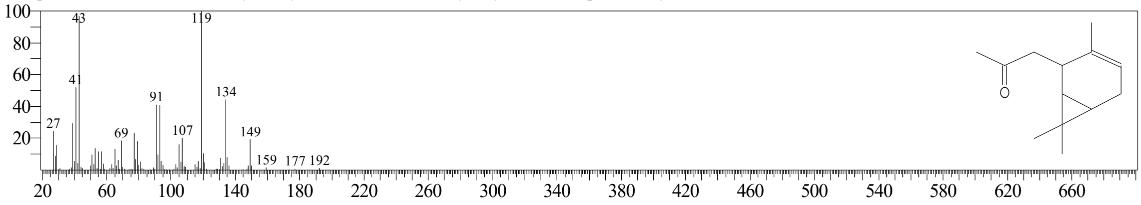

Hit#:2 Entry:38723 Library:NIST11.lib
SI:73 Formula:C13H20O CAS:96294-80-3 MolWeight:192 RetIndex:1339
CompName:.beta.-Pinene, 3-(acetylmethyl)- $$ 1-(6,6-Dimethyl-2-methylenebicyclo[3.1.1]hept-3-yl)acetone # $$

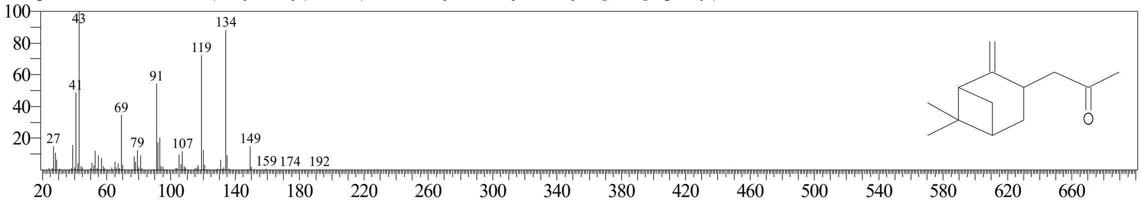

Hit#:3 Entry:69140 Library:NIST11.lib
SI:73 Formula:C14H20O3 CAS:0-00-0 MolWeight:236 RetIndex:1605
CompName:3(10)-Caren-4-ol, acetoacetic acid ester $$ 7,7-Dimethyl-4-methylenebicyclo[4.1.0]hept-3-yl 3-oxobutanoate # $$

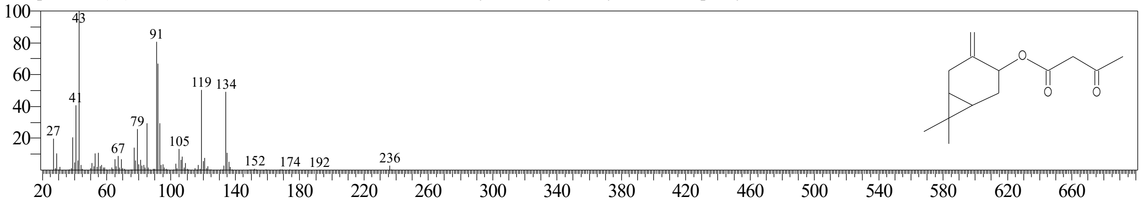

Hit#:4 Entry:122390 Library:NIST11.lib
SI:71 Formula:C18H27NO3 CAS:0-00-0 MolWeight:305 RetIndex:2370
CompName:2(3H)-Oxazolone, dihydro-4-hydroxy-4,5-dimethyl-5-(2-methyl-2-phenylpropyl)-3-propyl-

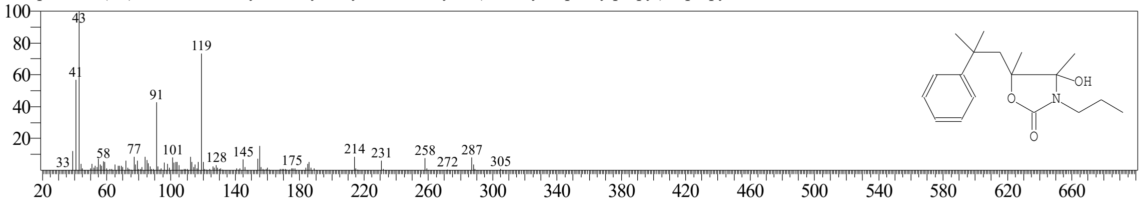

Hit#:5 Entry:30407 Library:NIST11.lib
SI:70 Formula:C12H18O CAS:0-00-0 MolWeight:178 RetIndex:1174
CompName:Bicyclo[3.1.1]hept-3-ene, 4,6,6-trimethyl-2-vinyloxy- $$ 2,6,6-Trimethyl-4-(vinyloxy)bicyclo[3.1.1]hept-2-ene # $$

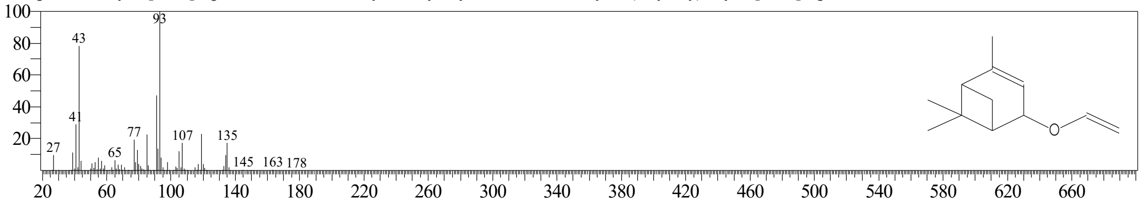

<< Target >>
Line#:18 R.Time:6.770(Scan#:555) MassPeaks:362
RawMode:Averaged 6.765-6.775(554-556) BasePeak:43.05(94355)
BG Mode:Calc. from Peak Group 1 - Event 1 Scan

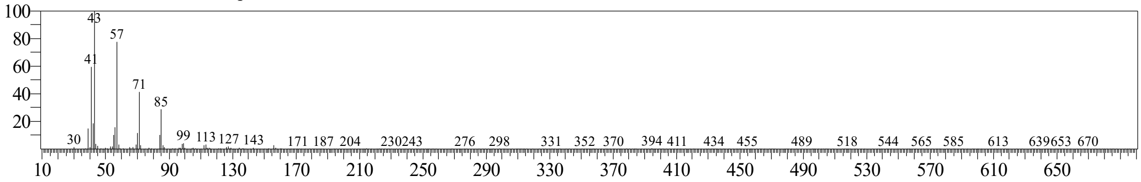

Hit#:1 Entry:26094 Library:NIST11.lib
SI:92 Formula:C12H26 CAS:1002-17-1 MolWeight:170 RetIndex:1086
CompName:Decane, 2,9-dimethyl- $$ 2,9-Dimethyldecane $$

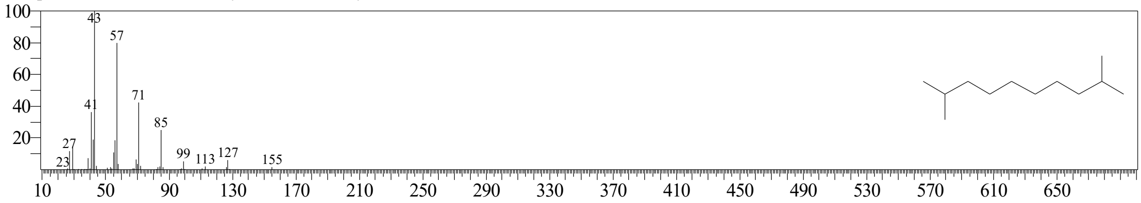

Hit#:2 Entry:18715 Library:NIST11.lib
SI:92 Formula:C11H24 CAS:1120-21-4 MolWeight:156 RetIndex:1115
CompName:Undecane $$ n-Undecane $$ Hendecane $$ n-C11H24 $$ UN 2330 $$

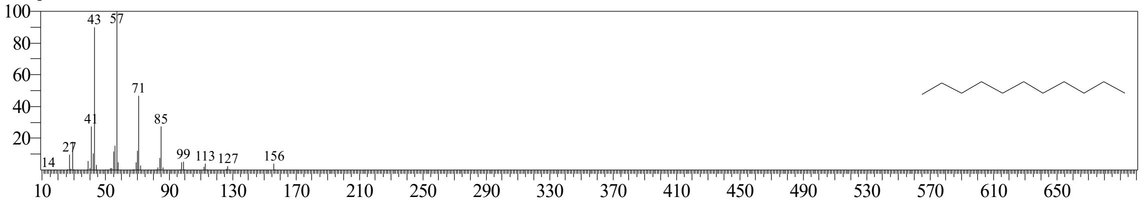

Hit#:3 Entry:12306 Library:NIST11.lib
SI:92 Formula:C10H22 CAS:871-83-0 MolWeight:142 RetIndex:951
CompName:Nonane, 2-methyl- $$ 2-Methylnonane $$

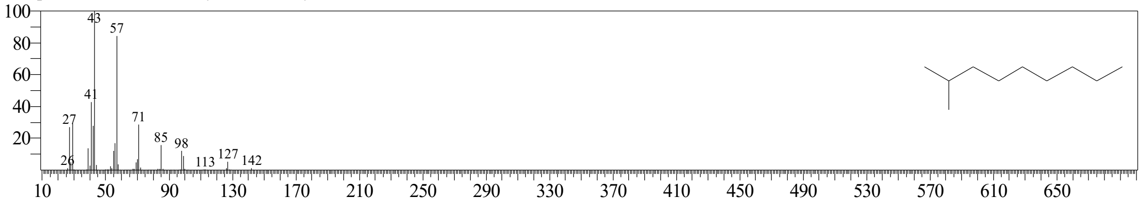

Hit#:4 Entry:7416 Library:NIST11.lib
SI:91 Formula:C9H20 CAS:111-84-2 MolWeight:128 RetIndex:916
CompName:Nonane $$ n-Nonane $$ Shellsol 140 $$ n-C9H20 $$ UN 1920 $$

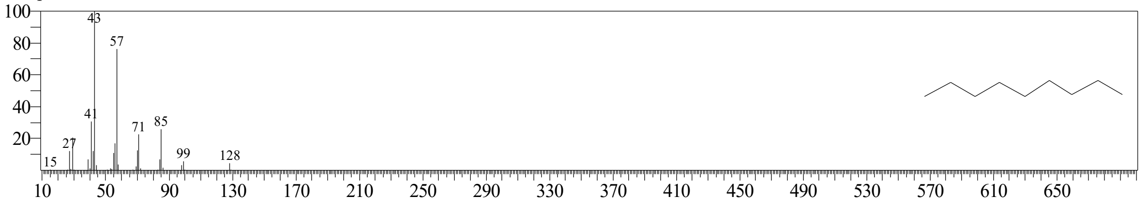

Hit#:5 Entry:34136 Library:NIST11.lib
SI:91 Formula:C13H28 CAS:17301-32-5 MolWeight:184 RetIndex:1185
CompName:Undecane, 4,7-dimethyl- $$ 4,7-Dimethylundecane # $$

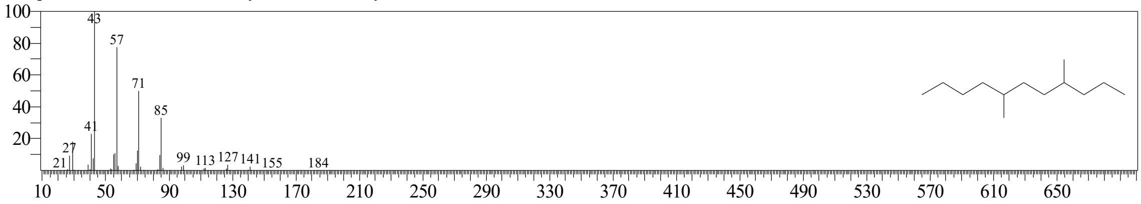

Qualitative Analysis Report

<< Target >>
Line#:19 R.Time:6.945(Scan#:590) MassPeaks:340
RawMode:Averaged 6.940-6.950(589-591) BasePeak:85.15(22894)
BG Mode:Calc. from Peak Group 1 - Event 1 Scan

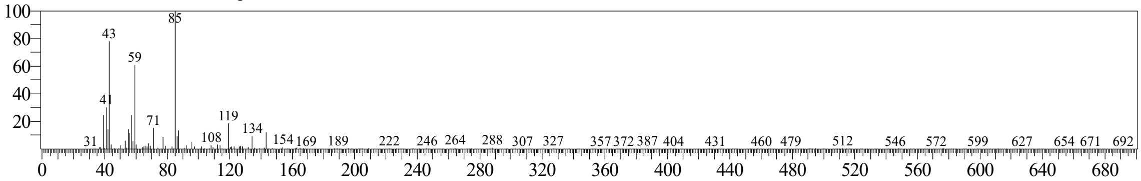

Hit#:1 Entry:2082 Library:NIST11.lib
SI:79 Formula:C6H12O CAS:10473-13-9 MolWeight:100 RetIndex:677
CompName:3-Buten-2-ol, 2,3-dimethyl- $$ CH2=C(CH3)C(CH3)2OH $$ 2,3-Dimethyl-3-buten-2-ol $$ 2,3-Dimethyl-1-buten-3-ol $$ 3-Hydroxy-2,3-dimethyl-

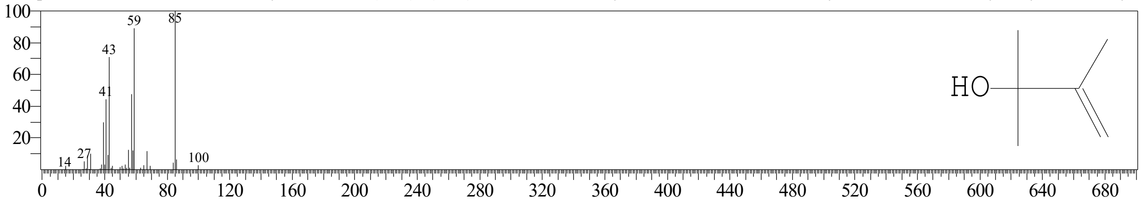

Hit#:2 Entry:12126 Library:NIST11.lib
SI:78 Formula:C8H14O2 CAS:0-00-0 MolWeight:142 RetIndex:993
CompName:Pivaloylacetone, enol form $$ (3Z)-4-Hydroxy-5,5-dimethyl-3-hexen-2-one # $$

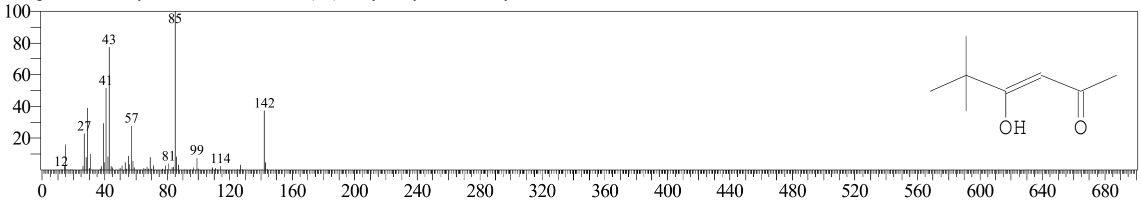

Hit#:3 Entry:26743 Library:NIST11.lib
SI:77 Formula:C8H12O4 CAS:0-00-0 MolWeight:172 RetIndex:1265
CompName:1,3-Dimethyl-4,8-dioxatricyclo[5.1.0.0(3,5)]octane-2,6-diol

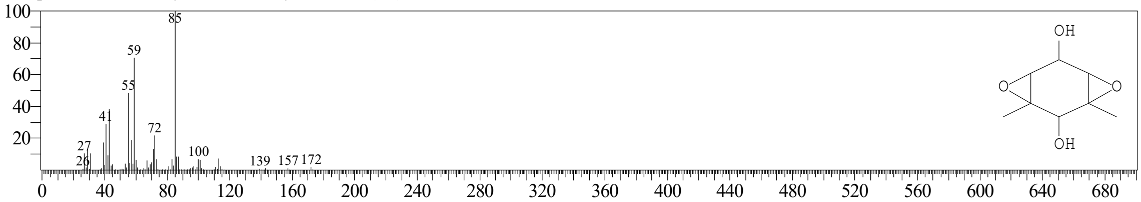

Hit#:4 Entry:12127 Library:NIST11.lib
SI:76 Formula:C8H14O2 CAS:7307-04-2 MolWeight:142 RetIndex:1004
CompName:2,4-Hexanedione, 5,5-dimethyl- $$ Pivaloylacetone, keto form $$ 5,5-Dimethyl-hexan-2,4-dione $$ 5,5-Dimethyl-2,4-hexanedione # $$ 5,5-Dimeth

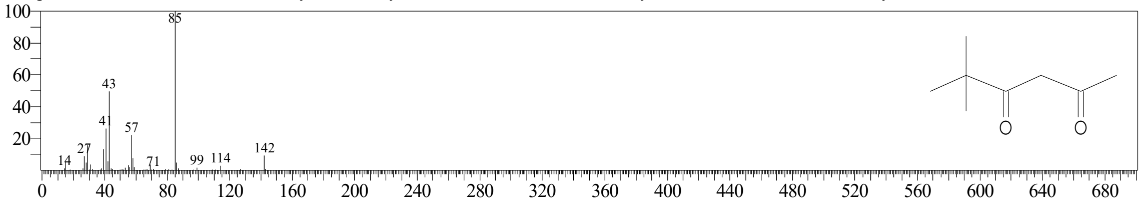

Hit#:5 Entry:14389 Library:NIST11.lib
SI:76 Formula:C7H13ClO CAS:15721-22-9 MolWeight:148 RetIndex:945
CompName:2,2-Dimethylvaleroyl chloride $$ 2,2-Dimethylpentanoyl chloride # $$

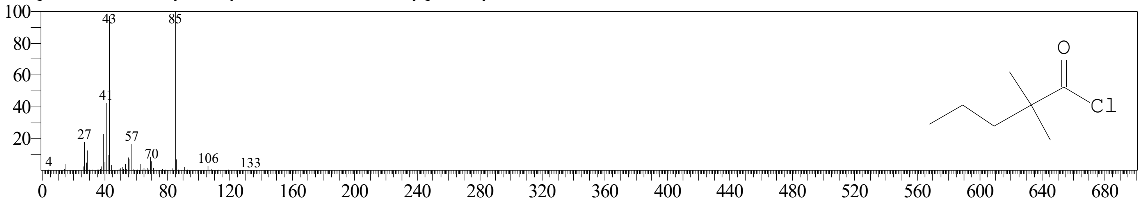

<< Target >>
Line#:20 R.Time:7.220(Scan#:645) MassPeaks:349
RawMode:Averaged 7.215-7.225(644-646) BasePeak:73.10(32421)
BG Mode:Calc. from Peak Group 1 - Event 1 Scan

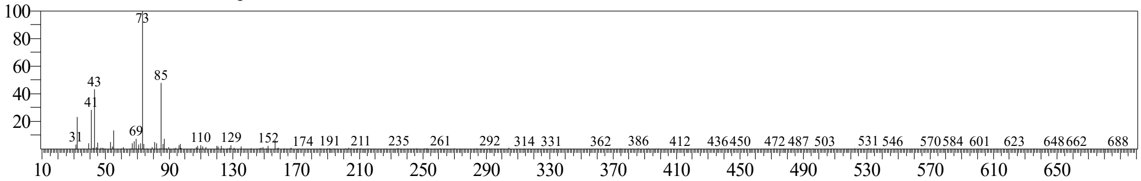

Hit#:1 Entry:36153 Library:NIST11.lib
SI:77 Formula:C10H20O3 CAS:0-00-0 MolWeight:188 RetIndex:1174
CompName:3-Methylpentan-3-yl propyl carbonate

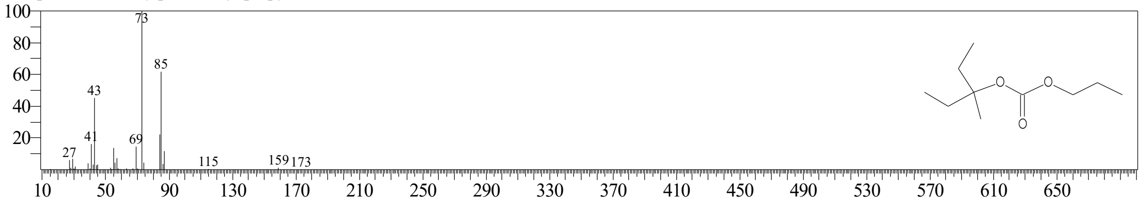

Hit#:2 Entry:72975 Library:NIST11.lib
SI:74 Formula:C15H31NO CAS:36475-50-0 MolWeight:241 RetIndex:1804
CompName:Tetradecanal, O-methyloxime $$ (1E)-Tetradecanal o-methyloxime # $$

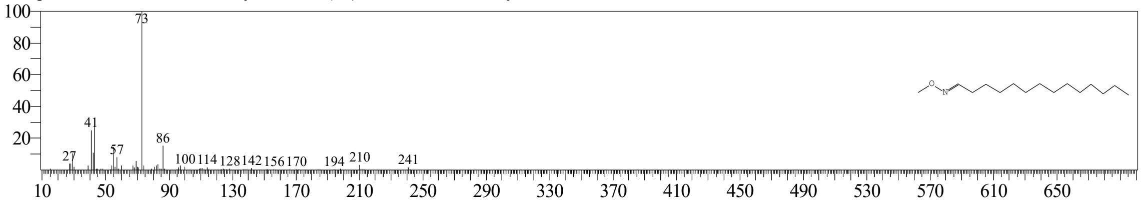

Hit#:3 Entry:13303 Library:NIST11.lib
SI:74 Formula:C7H15NO2 CAS:78-28-4 MolWeight:145 RetIndex:1043
CompName:Emylcamate $$ 3-Pentanol, 3-methyl-, carbamate $$ tert-Hexanol carbamate $$ Diethyl methyl carbinol urethan $$ Emylcamat $$ KABI-925 $$ Met

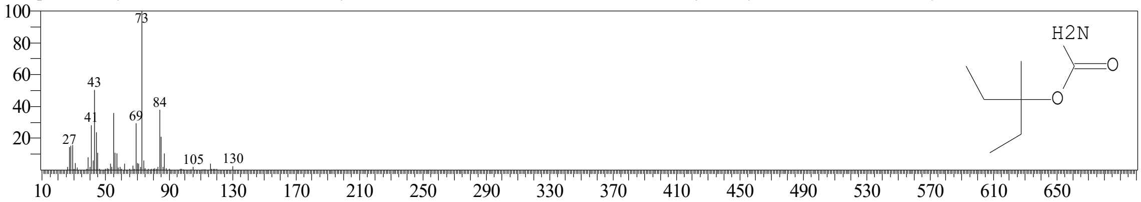

Hit#:4 Entry:2347 Library:NIST11.lib
SI:73 Formula:C6H14O CAS:994-05-8 MolWeight:102 RetIndex:609
CompName:Butane, 2-methoxy-2-methyl- $$ Ether, methyl tert-pentyl $$ tert-Amyl Methyl ether $$ Methyl tert-pentyl ether $$ 1,1-Dimethylpropyl methyl ethe

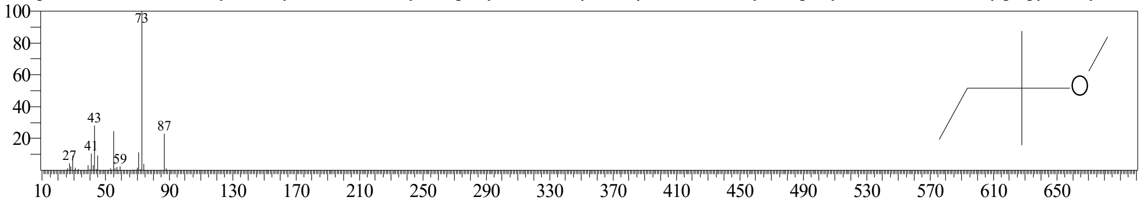

Hit#:5 Entry:28104 Library:NIST11.lib
SI:73 Formula:C10H22O2 CAS:53273-13-5 MolWeight:174 RetIndex:998
CompName:Hexane, 2,5-dimethoxy-2,5-dimethyl- $$ 2,5-Dimethoxy-2,5-dimethylhexane $$

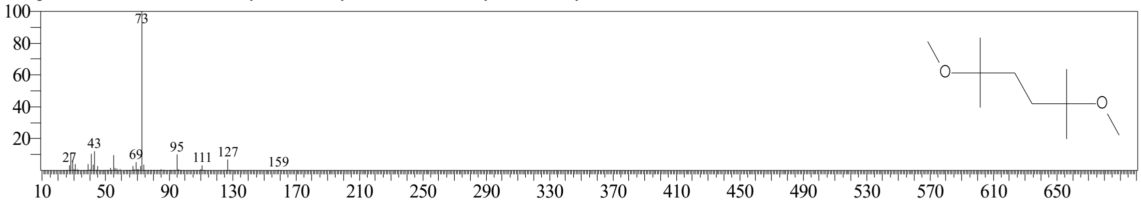

<< Target >>
Line#:21 R.Time:7.515(Scan#:704) MassPeaks:368
RawMode:Averaged 7.510-7.520(703-705) BasePeak:43.05(18351)
BG Mode:Calc. from Peak Group 1 - Event 1 Scan

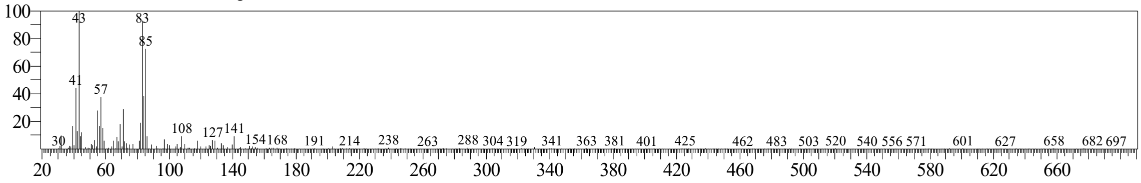

Hit#:1 Entry:34147 Library:NIST11.lib
SI:80 Formula:C13H28 CAS:17312-80-0 MolWeight:184 RetIndex:1185
CompName:Undecane, 2,4-dimethyl- $$ 2,4-Dimethylundecane $$

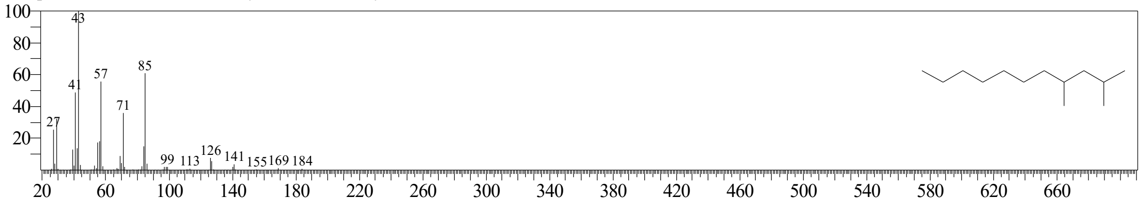

Hit#:2 Entry:181538 Library:NIST11.lib
SI:79 Formula:C24H46O4 CAS:0-00-0 MolWeight:398 RetIndex:2677
CompName:Oxalic acid, hexadecyl isohexyl ester

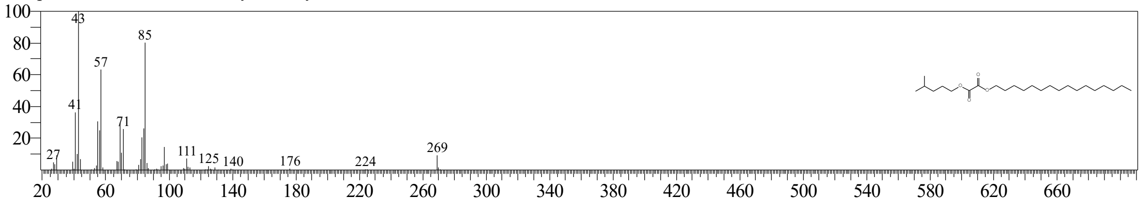

Hit#:3 Entry:8016 Library:NIST11.lib
SI:79 Formula:C8H18O CAS:66576-22-5 MolWeight:130 RetIndex:894
CompName:3-Ethyl-3-methyl-2-pentanol

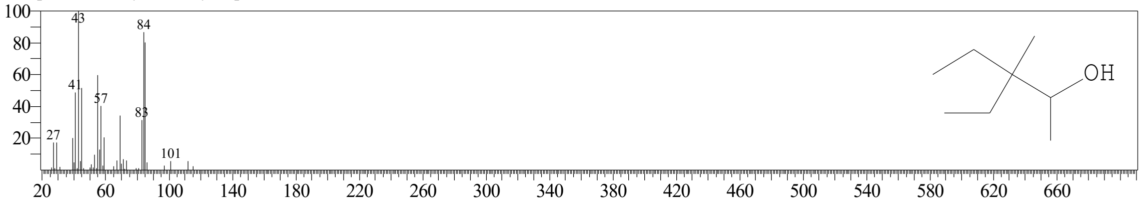

Hit#:4 Entry:26101 Library:NIST11.lib
SI:79 Formula:C12H26 CAS:1636-41-5 MolWeight:170 RetIndex:1086
CompName:Octane, 4,5-diethyl- $$ 4,5-Diethyloctane # $$

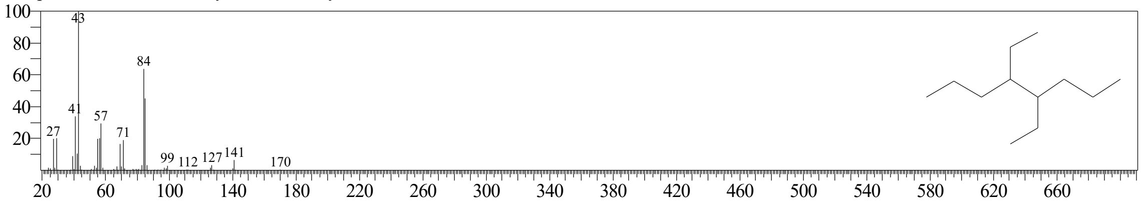

Hit#:5 Entry:129413 Library:NIST11.lib
SI:79 Formula:C18H34O4 CAS:0-00-0 MolWeight:314 RetIndex:1953
CompName:Oxalic acid, 6-ethyloct-3-yl isohexyl ester

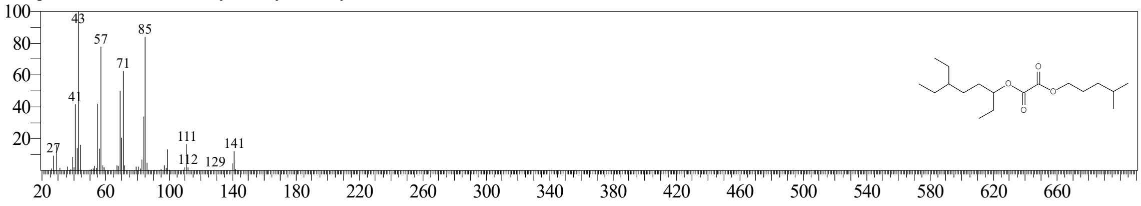

<< Target >>
Line#:22 R.Time:7.705(Scan#:742) MassPeaks:400
RawMode:Averaged 7.700-7.710(741-743) BasePeak:83.05(52888)
BG Mode:Calc. from Peak Group 1 - Event 1 Scan

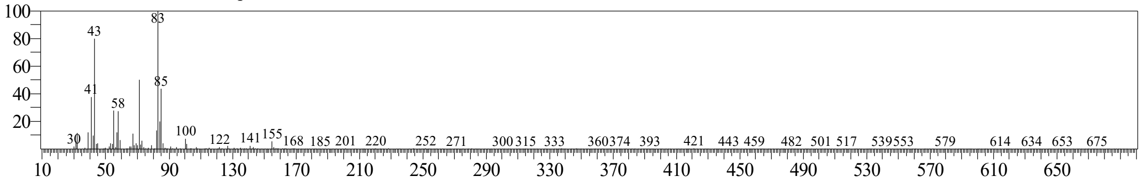

Hit#:1 Entry:33780 Library:NIST11.lib
SI:79 Formula:C10H16O3 CAS:0-00-0 MolWeight:184 RetIndex:1299
CompName:3-Methyl-2-butenoic acid, 2-tetrahydrofurylmethyl ester $$ Tetrahydro-2-furanylmethyl 3-methyl-2-butenoate # $$

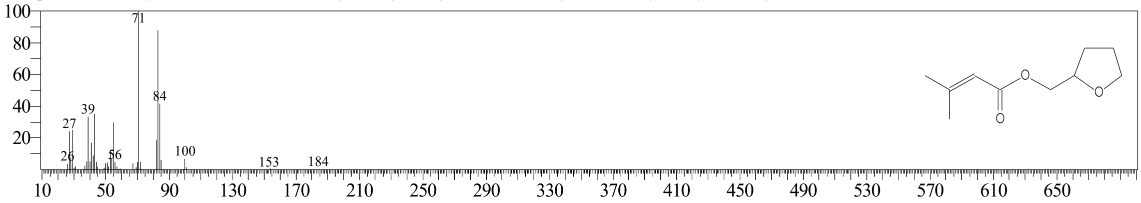

Hit#:2 Entry:27227 Library:NIST11.lib
SI:79 Formula:C11H24O CAS:213547-15-0 MolWeight:172 RetIndex:1213
CompName:4-Methyl-5-decanol

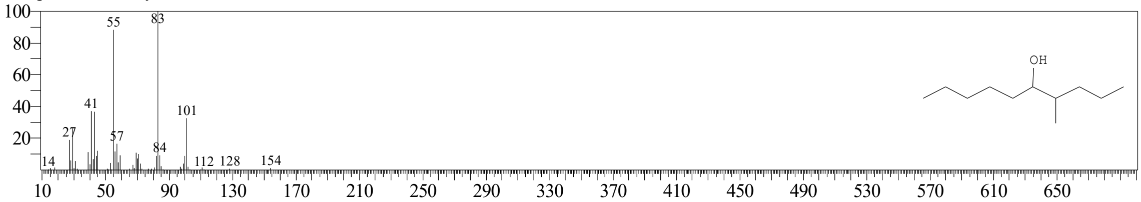

Hit#:3 Entry:27105 Library:NIST11.lib
SI:78 Formula:C10H20O2 CAS:0-00-0 MolWeight:172 RetIndex:1317
CompName:1,1-Hexylenedioxybutane $$ 2-Propyl-1,3-dioxonane # $$

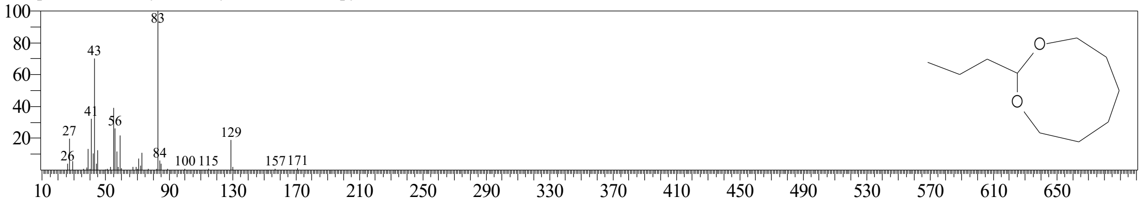

Hit#:4 Entry:13161 Library:NIST11.lib
SI:78 Formula:C9H20O CAS:19549-73-6 MolWeight:144 RetIndex:950
CompName:3-Heptanol, 2,6-dimethyl- $$ 2,6-Dimethyl-3-heptanol $$

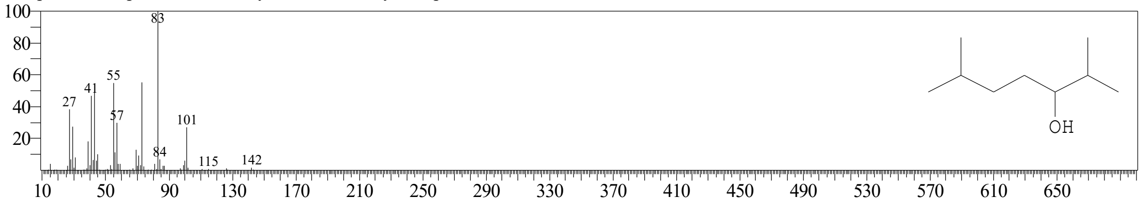

Hit#:5 Entry:72330 Library:NIST11.lib
SI:77 Formula:C15H28O2 CAS:0-00-0 MolWeight:240 RetIndex:1536
CompName:3-Methyl-2-butenoic acid, 6-ethyl-3-octyl ester $$ 1,4-Diethylhexyl 3-methyl-2-butenoate # $$

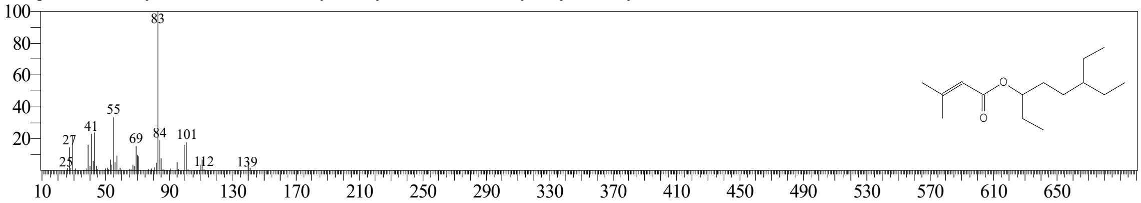

<< Target >>
Line#:23 R.Time:7.815(Scan#:764) MassPeaks:342
RawMode:Averaged 7.810-7.820(763-765) BasePeak:43.10(35312)
BG Mode:Calc. from Peak Group 1 - Event 1 Scan

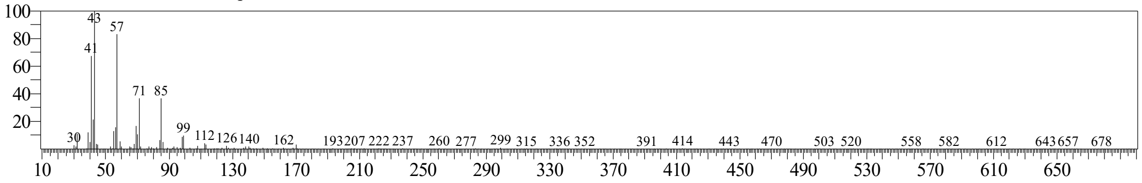

Hit#:1 Entry:12306 Library:NIST11.lib
SI:88 Formula:C10H22 CAS:871-83-0 MolWeight:142 RetIndex:951
CompName:Nonane, 2-methyl- $$ 2-Methylnonane $$

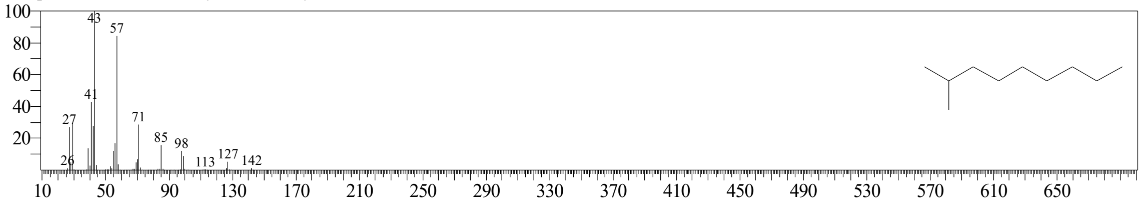

Hit#:2 Entry:18717 Library:NIST11.lib
SI:87 Formula:C11H24 CAS:6975-98-0 MolWeight:156 RetIndex:1051
CompName:Decane, 2-methyl- $$ 2-Methyldecane $$ n-C8H17CH(CH3)2 $$

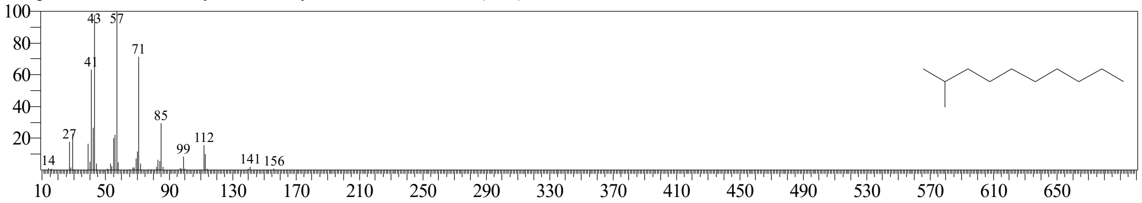

Hit#:3 Entry:52481 Library:NIST11.lib
SI:87 Formula:C15H32 CAS:55030-62-1 MolWeight:212 RetIndex:1384
CompName:Tridecane, 4,8-dimethyl- $$ 4,8-Dimethyltridecane # $$

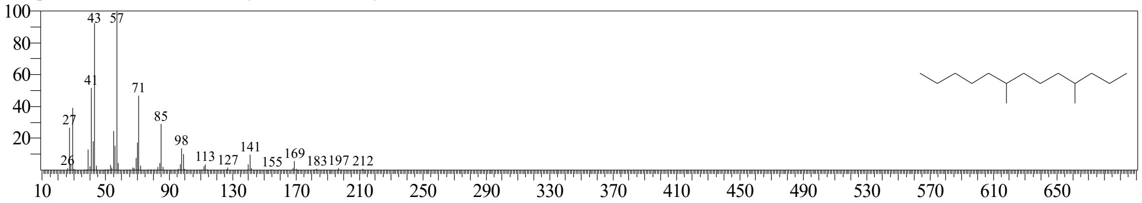

Hit#:4 Entry:34138 Library:NIST11.lib
SI:87 Formula:C13H28 CAS:1560-97-0 MolWeight:184 RetIndex:1249
CompName:Dodecane, 2-methyl- $$ 11-Methyldodecane $$ 2-Methyldodecane $$

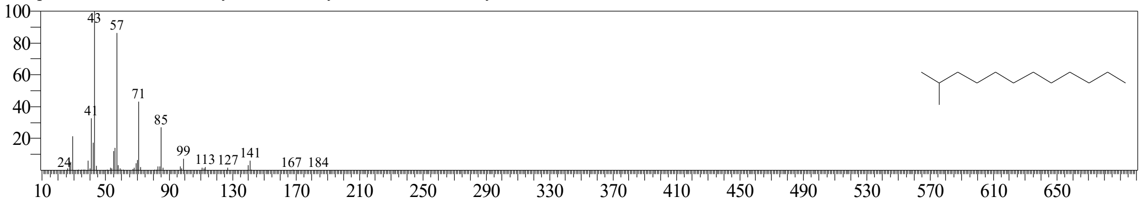

Hit#:5 Entry:4236 Library:NIST11.lib
SI:87 Formula:C8H18 CAS:589-43-5 MolWeight:114 RetIndex:688
CompName:Hexane, 2,4-dimethyl- $$ 2,4-Dimethylhexane $$

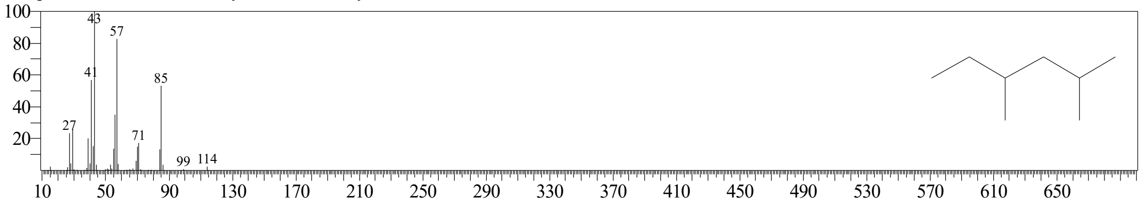

<< Target >>
Line#:24 R.Time:7.925(Scan#:786) MassPeaks:379
RawMode:Averaged 7.920-7.930(785-787) BasePeak:43.05(44396)
BG Mode:Calc. from Peak Group 1 - Event 1 Scan

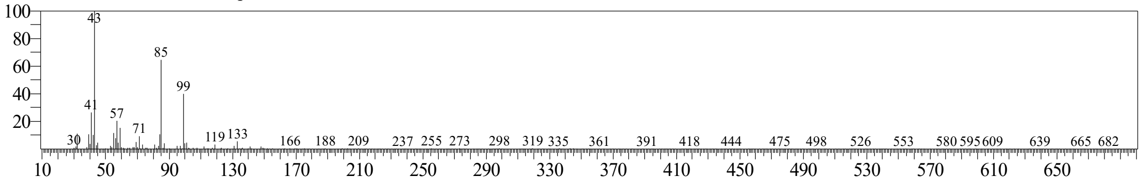

Hit#:1 Entry:33872 Library:NIST11.lib
SI:84 Formula:C11H20O2 CAS:0-00-0 MolWeight:184 RetIndex:1238
CompName:6,6,7-Trimethyl-octane-2,5-dione $$ 6,6,7-Trimethyl-2,5-octanedione # $$

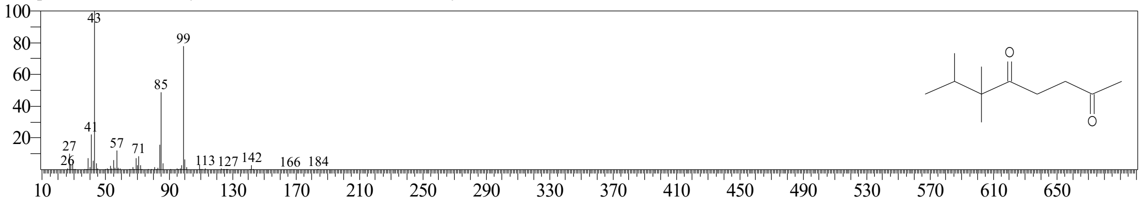

Hit#:2 Entry:18403 Library:NIST11.lib
SI:83 Formula:C9H16O2 CAS:44970-05-0 MolWeight:156 RetIndex:989
CompName:Pentanoic acid, 2,2-dimethyl-, ethenyl ester $$ Vinyl 2,2-dimethylpentanoate # $$

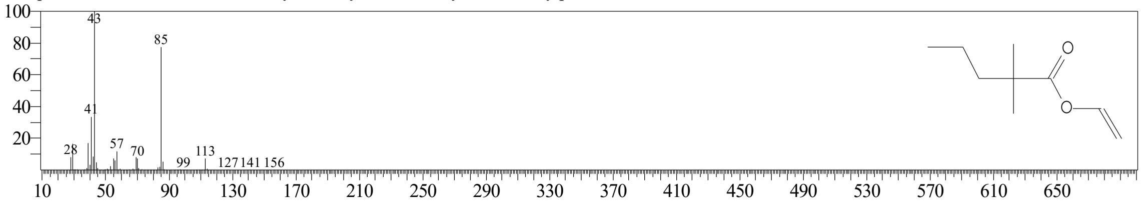

Hit#:3 Entry:35305 Library:NIST11.lib
SI:83 Formula:C12H26O CAS:112-58-3 MolWeight:186 RetIndex:1290
CompName:Hexane, 1,1'-oxybis- $$ Hexyl ether $$ n-Hexyl ether $$ Di-n-hexyl ether $$ Dihexyl ether $$ Bis(1-hexyl)ether $$ Ether, dihexyl $$ 1-(Hexyloxy)h

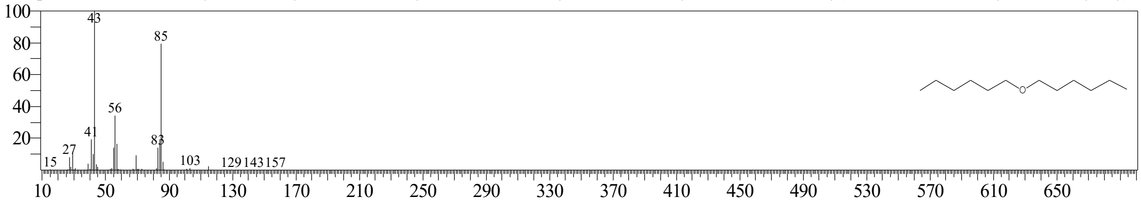

Hit#:4 Entry:7904 Library:NIST11.lib
SI:82 Formula:C7H14O2 CAS:19889-05-3 MolWeight:130 RetIndex:989
CompName:Butanoic acid, 2-ethyl-2-methyl- $$ Butyric acid, 2-ethyl-2-methyl- $$ 2-Ethyl-2-methylbutanoic acid $$ 2-Methyl-2-ethylbutanoic acid $$

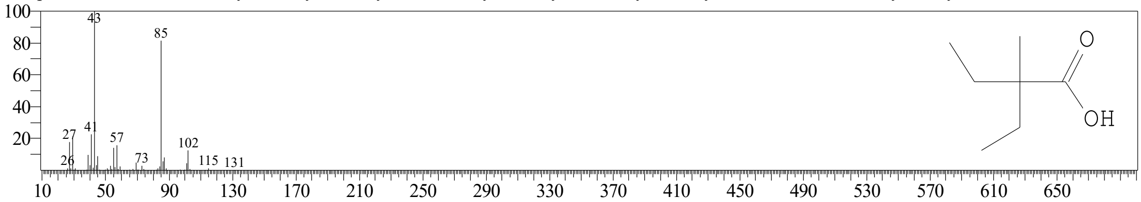

Hit#:5 Entry:18406 Library:NIST11.lib
SI:82 Formula:C9H16O2 CAS:999-05-3 MolWeight:156 RetIndex:1112
CompName:7-Methyloctane-2,4-dione, enol form $$ (3Z)-4-Hydroxy-7-methyl-3-octen-2-one # $$

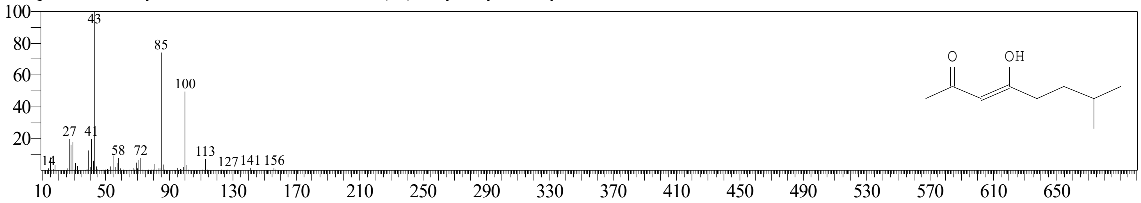

<< Target >>
Line#:25 R.Time:8.155(Scan#:832) MassPeaks:394
RawMode:Averaged 8.150-8.160(831-833) BasePeak:43.05(35576)
BG Mode:Calc. from Peak Group 1 - Event 1 Scan

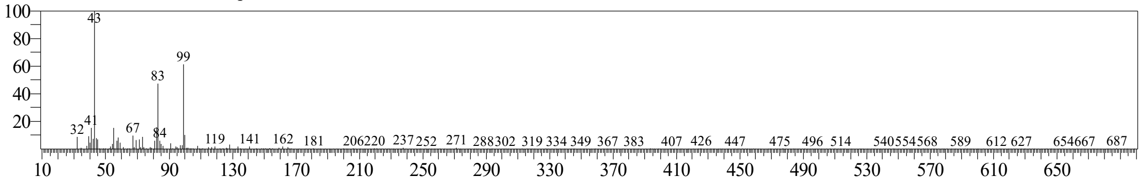

Hit#:1 Entry:12033 Library:NIST11.lib
SI:80 Formula:C8H14O2 CAS:3050-69-9 MolWeight:142 RetIndex:974
CompName:n-Caproic acid vinyl ester $$ Hexanoic acid, ethenyl ester $$ Caproic acid, vinyl ester $$ Hexanoic acid, vinyl ester $$ Vinyl caproate $$ Vinyl hexa

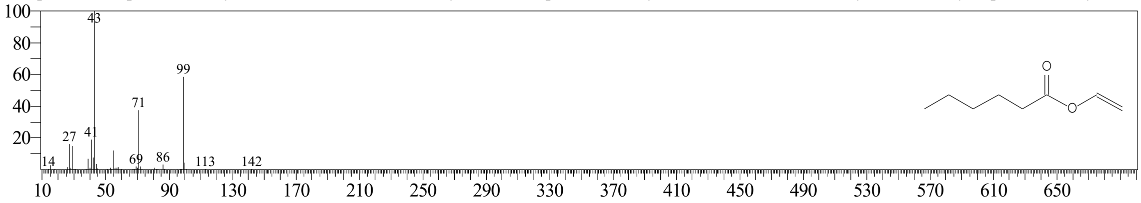

Hit#:2 Entry:25797 Library:NIST11.lib
SI:79 Formula:C10H18O2 CAS:2442-10-6 MolWeight:170 RetIndex:1109
CompName:1-Octen-3-yl-acetate $$ 1-Octen-3-ol, acetate $$ Amyl vinyl carbinol acetate $$ Amyl vinyl carbinyl acetate $$ 1-Pentylallyl acetate $$ 1-Octen-3-yl

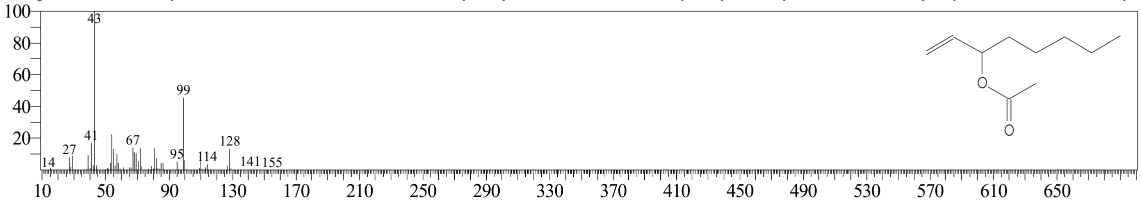

Hit#:3 Entry:32924 Library:NIST11.lib
SI:79 Formula:C12H22O CAS:0-00-0 MolWeight:182 RetIndex:1359
CompName:2-Cyclohexyl-hex-5-en-2-ol $$ 2-Cyclohexyl-5-hexen-2-ol # $$

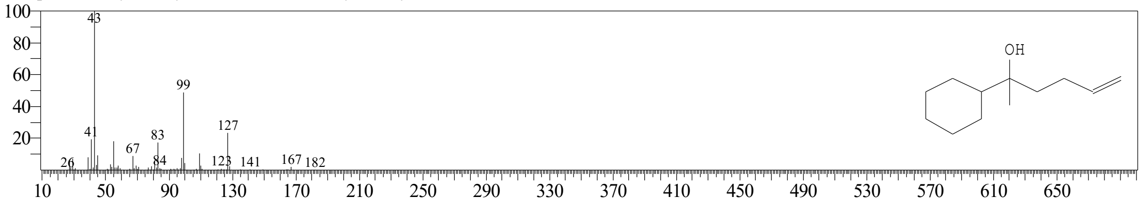

Hit#:4 Entry:33872 Library:NIST11.lib
SI:79 Formula:C11H20O2 CAS:0-00-0 MolWeight:184 RetIndex:1238
CompName:6,6,7-Trimethyl-octane-2,5-dione $$ 6,6,7-Trimethyl-2,5-octanedione # $$

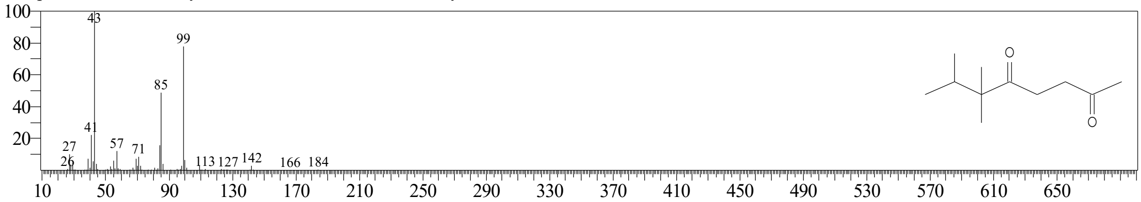

Hit#:5 Entry:4720 Library:NIST11.lib
SI:78 Formula:C3H7N3O2 CAS:352-97-6 MolWeight:117 RetIndex:1076
CompName:Guanidineacetic acid $$ Glycocyamine $$ Guanidinoacetic acid $$ Glucocyamine $$ N-Amidinoglycine $$ Glycine, N-(aminoiminomethyl)- $$ .alp

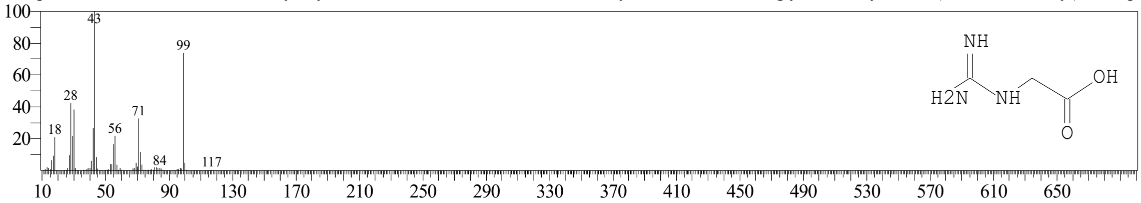

<< Target >>
Line#:26 R.Time:8.500(Scan#:901) MassPeaks:376
RawMode:Averaged 8.495-8.505(900-902) BasePeak:43.05(158397)
BG Mode:Calc. from Peak Group 1 - Event 1 Scan

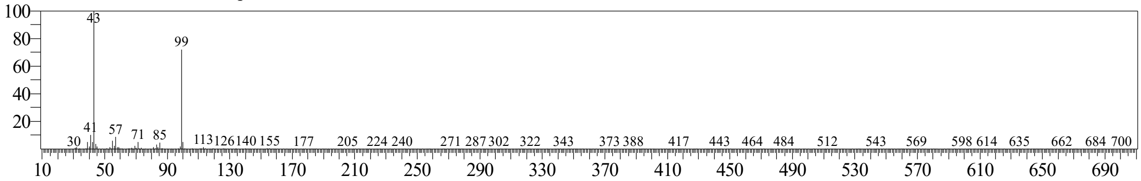

Hit#:1 Entry:12033 Library:NIST11.lib
SI:89 Formula:C8H14O2 CAS:3050-69-9 MolWeight:142 RetIndex:974
CompName:n-Caproic acid vinyl ester $$ Hexanoic acid, ethenyl ester $$ Caproic acid, vinyl ester $$ Hexanoic acid, vinyl ester $$ Vinyl caproate $$ Vinyl hexa

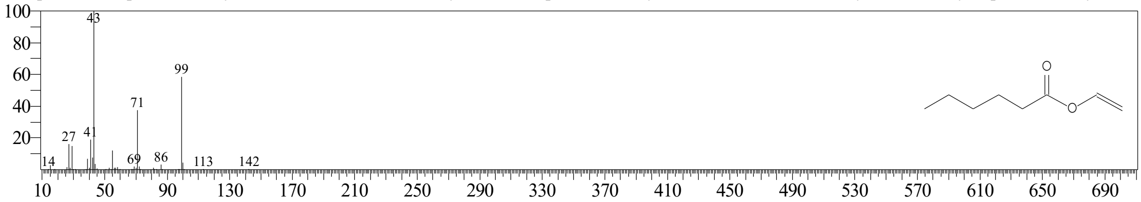

Hit#:2 Entry:33872 Library:NIST11.lib
SI:88 Formula:C11H20O2 CAS:0-00-0 MolWeight:184 RetIndex:1238
CompName:6,6,7-Trimethyl-octane-2,5-dione $$ 6,6,7-Trimethyl-2,5-octanedione # $$

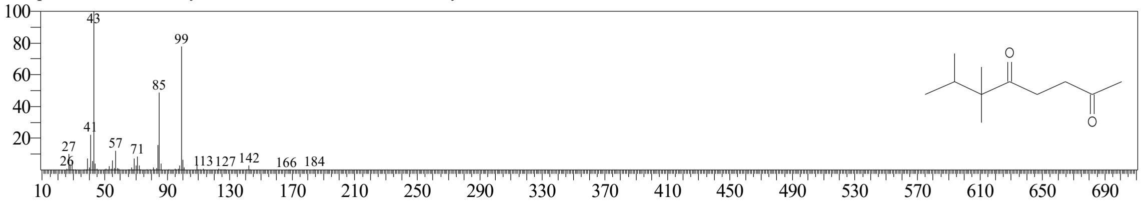

Hit#:3 Entry:19146 Library:NIST11.lib
SI:88 Formula:C7H10O4 CAS:57681-51-3 MolWeight:158 RetIndex:1226
CompName:2(3H)-Furanone, 5-(acetyloxy)dihydro-5-methyl- $$ 2-Methyl-5-oxotetrahydro-2-furanyl acetate # $$

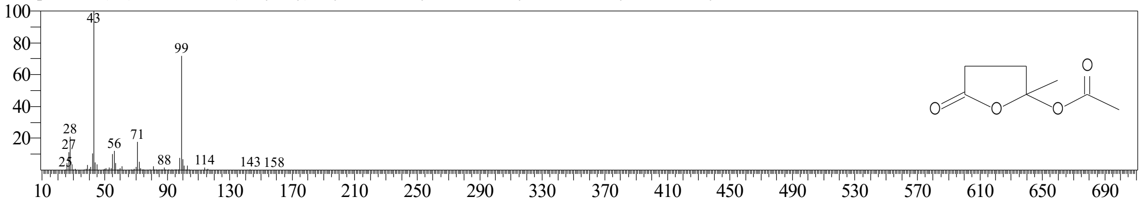

Hit#:4 Entry:4482 Library:NIST11.lib
SI:87 Formula:C5H8O3 CAS:28525-83-9 MolWeight:116 RetIndex:1045
CompName:2-Pentenoic acid, 4-hydroxy- $$ (2E)-4-Hydroxy-2-pentenoic acid # $$

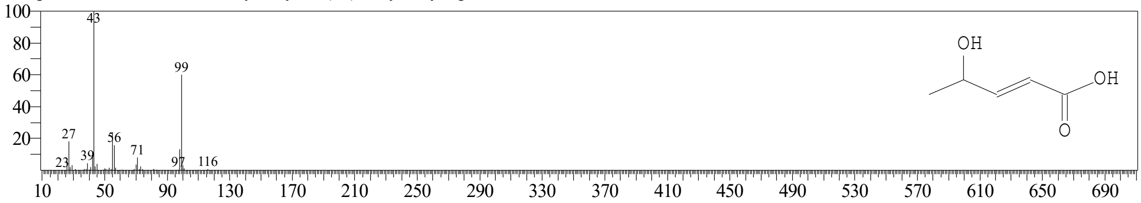

Hit#:5 Entry:18502 Library:NIST11.lib
SI:87 Formula:C9H16O2 CAS:10200-21-2 MolWeight:156 RetIndex:1194
CompName:2(3H)-Furanone, dihydro-5-methyl-5-(2-methylpropyl)- $$ 5-Isobutyl-5-methyldihydro-2(3H)-furanone # $$

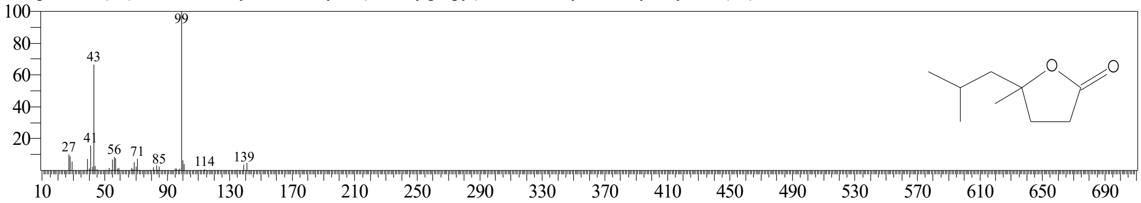

<< Target >>
Line#:27 R.Time:8.670(Scan#:935) MassPeaks:364
RawMode:Averaged 8.665-8.675(934-936) BasePeak:43.00(74882)
BG Mode:Calc. from Peak Group 1 - Event 1 Scan

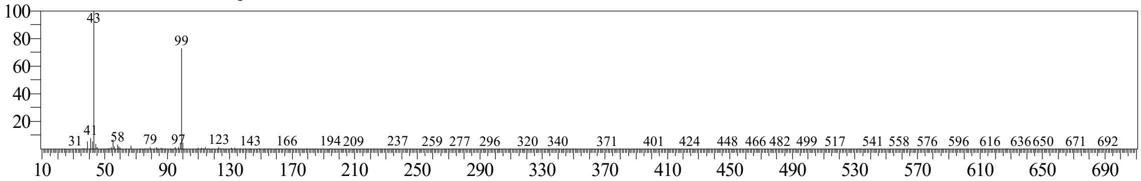

Hit#:1 Entry:4482 Library:NIST11.lib
SI:84 Formula:C5H8O3 CAS:28525-83-9 MolWeight:116 RetIndex:1045
CompName:2-Pentenoic acid, 4-hydroxy- $$ (2E)-4-Hydroxy-2-pentenoic acid # $$

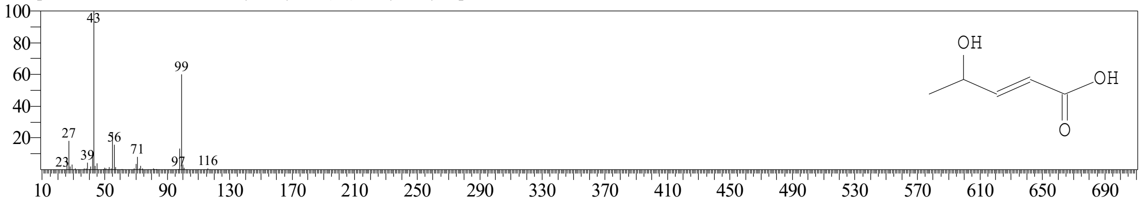

Hit#:2 Entry:19146 Library:NIST11.lib
SI:84 Formula:C7H10O4 CAS:57681-51-3 MolWeight:158 RetIndex:1226
CompName:2(3H)-Furanone, 5-(acetyloxy)dihydro-5-methyl- $$ 2-Methyl-5-oxotetrahydro-2-furanyl acetate # $$

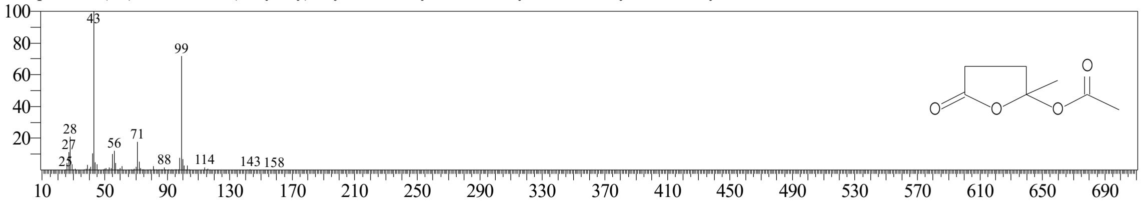

Hit#:3 Entry:12034 Library:NIST11.lib
SI:84 Formula:C8H14O2 CAS:90112-64-4 MolWeight:142 RetIndex:1064
CompName:Cyclobutene-3,4-diol, tetramethyl- $$ 1,2,3,4-Tetramethyl-3-cyclobutene-1,2-diol # $$

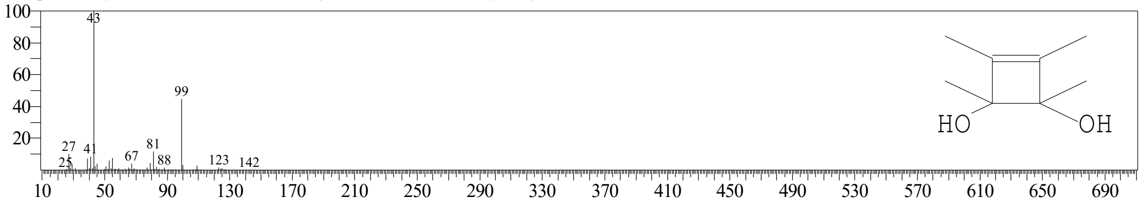

Hit#:4 Entry:12033 Library:NIST11.lib
SI:83 Formula:C8H14O2 CAS:3050-69-9 MolWeight:142 RetIndex:974
CompName:n-Caproic acid vinyl ester $$ Hexanoic acid, ethenyl ester $$ Caproic acid, vinyl ester $$ Hexanoic acid, vinyl ester $$ Vinyl caproate $$ Vinyl hexa

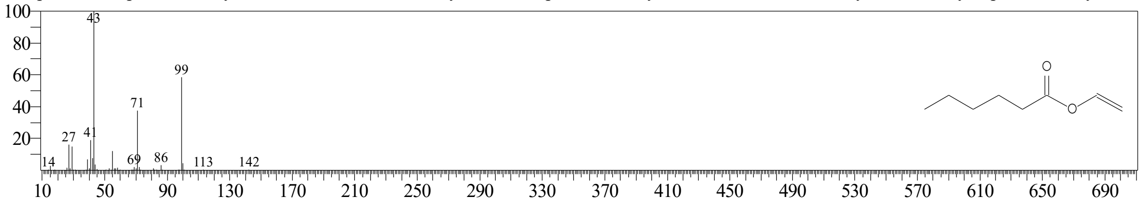

Hit#:5 Entry:11084 Library:NIST11.lib
SI:82 Formula:C8H12O2 CAS:0-00-0 MolWeight:140 RetIndex:900
CompName:1,5-Hexadien-3-ol, acetate

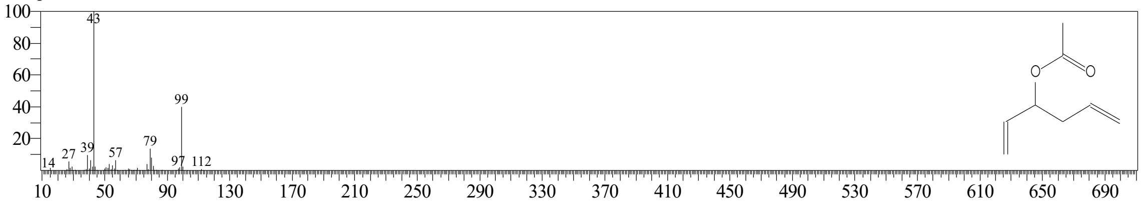

<< Target >>
Line#:28 R.Time:8.805(Scan#:962) MassPeaks:393
RawMode:Averaged 8.800-8.810(961-963) BasePeak:43.05(37702)
BG Mode:Calc. from Peak Group 1 - Event 1 Scan

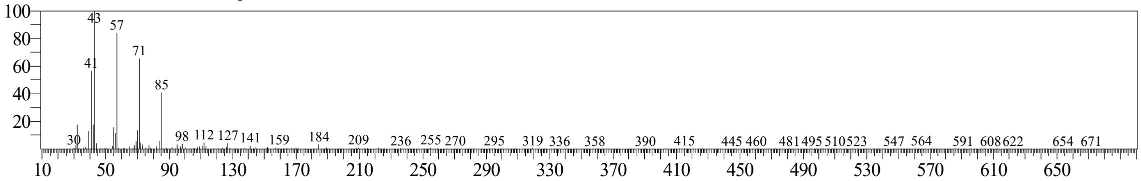

Hit#:1 Entry:26099 Library:NIST11.lib
SI:88 Formula:C12H26 CAS:17312-44-6 MolWeight:170 RetIndex:1086
CompName:2,3-Dimethyldecane $$ Decane, 2,3-dimethyl $$

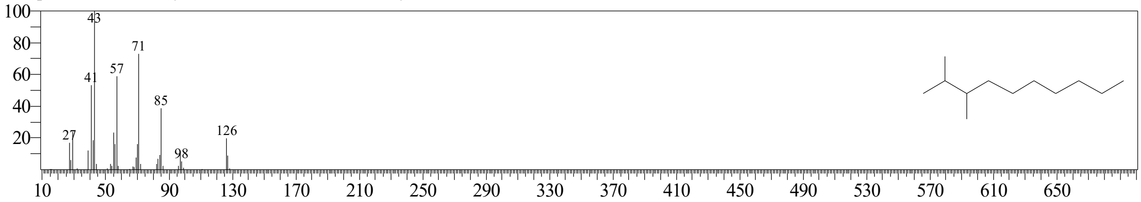

Hit#:2 Entry:26089 Library:NIST11.lib
SI:88 Formula:C12H26 CAS:17312-54-8 MolWeight:170 RetIndex:1086
CompName:Decane, 3,7-dimethyl- $$ 3,7-Dimethyldecane # $$

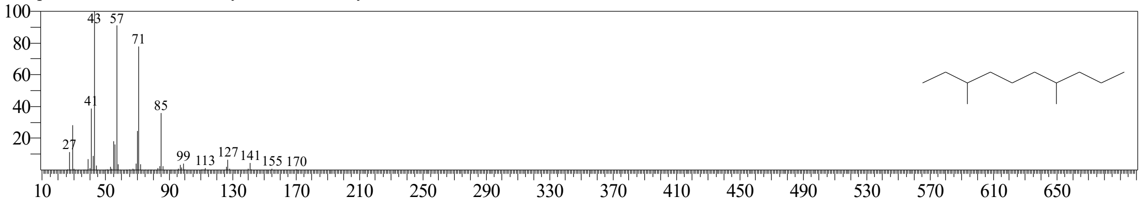

Hit#:3 Entry:26091 Library:NIST11.lib
SI:88 Formula:C12H26 CAS:13150-81-7 MolWeight:170 RetIndex:1086
CompName:2,6-Dimethyldecane

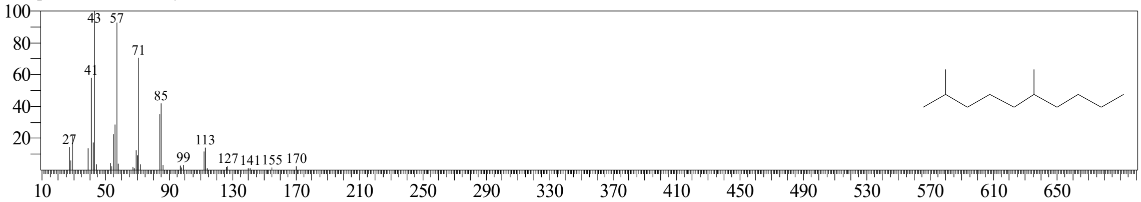

Hit#:4 Entry:18707 Library:NIST11.lib
SI:88 Formula:C11H24 CAS:62016-30-2 MolWeight:156 RetIndex:966
CompName:Octane, 2,3,3-trimethyl- $$ 2,3,3-Trimethyloctane # $$

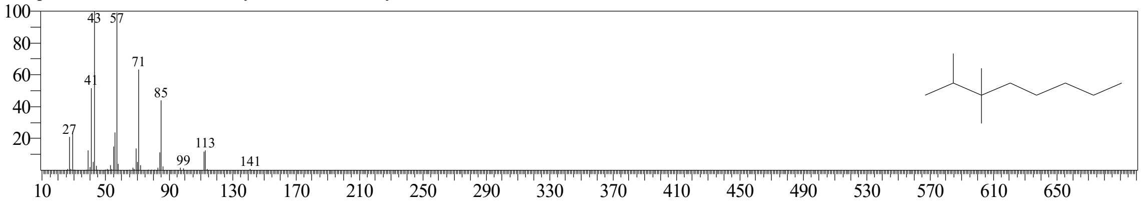

Hit#:5 Entry:18708 Library:NIST11.lib
SI:88 Formula:C11H24 CAS:62016-18-6 MolWeight:156 RetIndex:986
CompName:Octane, 5-ethyl-2-methyl- $$ 5-Ethyl-2-methyloctane # $$

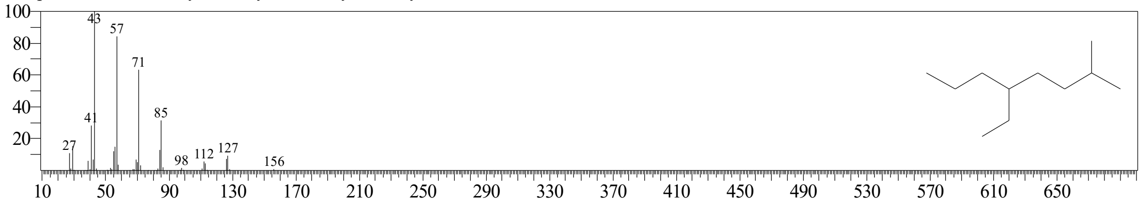

Qualitative Analysis Report

<< Target >>
Line#:29 R.Time:9.725(Scan#:1146) MassPeaks:364
RawMode:Averaged 9.720-9.730(1145-1147) BasePeak:43.05(32745)
BG Mode:Calc. from Peak Group 1 - Event 1 Scan

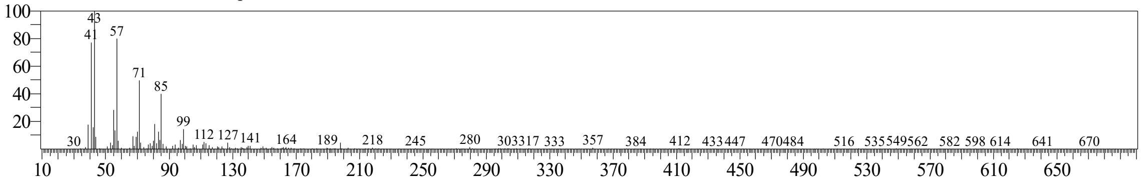

Hit#:1 Entry:24835 Library:NIST11.lib
SI:87 Formula:C12H24 CAS:54410-98-9 MolWeight:168 RetIndex:1012
CompName:1-Nonene, 4,6,8-trimethyl- $$ 4,6,8-Trimethyl-1-nonene # $$

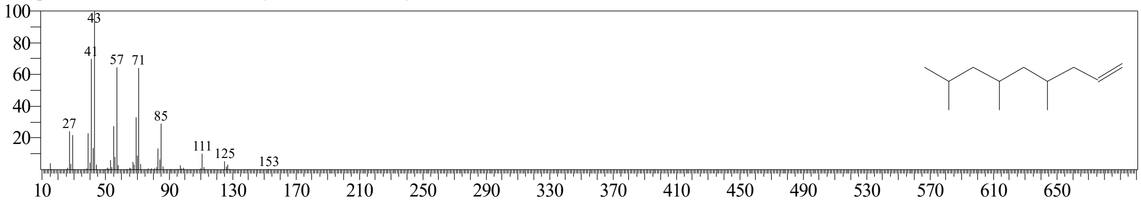

Hit#:2 Entry:104531 Library:NIST11.lib
SI:87 Formula:C20H42 CAS:1560-86-7 MolWeight:282 RetIndex:1945
CompName:Nonadecane, 2-methyl- $$ 2-Methylnonadecane $$

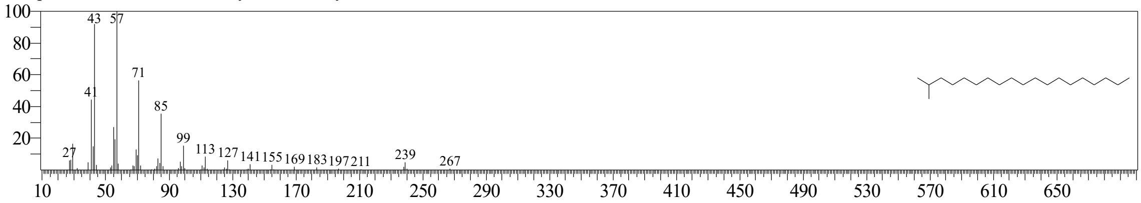

Hit#:3 Entry:62481 Library:NIST11.lib
SI:87 Formula:C16H34 CAS:55045-09-5 MolWeight:226 RetIndex:1548
CompName:Tridecane, 7-propyl- $$ 7-n-Propyltridecane $$ 7-Propyltridecane # $$

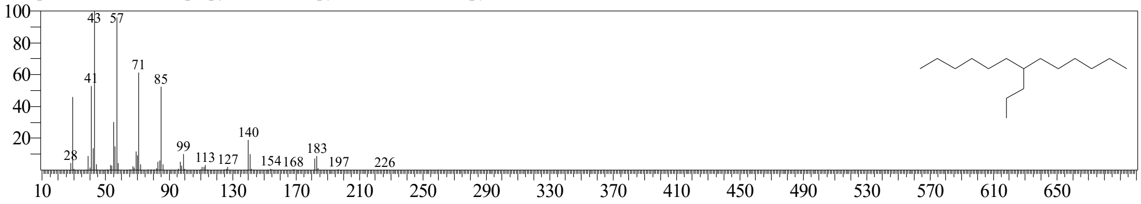

Hit#:4 Entry:18717 Library:NIST11.lib
SI:87 Formula:C11H24 CAS:6975-98-0 MolWeight:156 RetIndex:1051
CompName:Decane, 2-methyl- $$ 2-Methyldecane $$ n-C8H17CH(CH3)2 $$

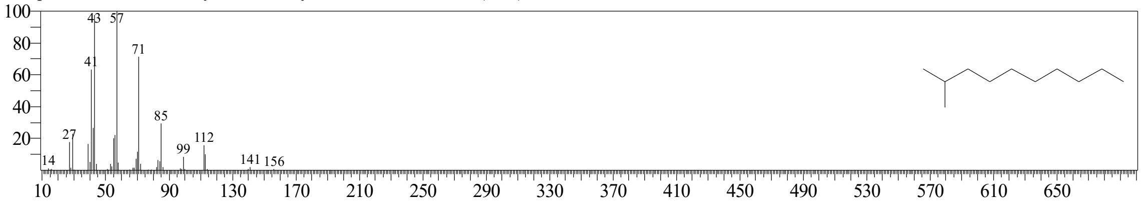

Hit#:5 Entry:19561 Library:NIST11.lib
SI:87 Formula:C10H22O CAS:10042-59-8 MolWeight:158 RetIndex:1194
CompName:1-Heptanol, 2-propyl- $$ 2-Propylheptanol $$ 2-Propyl-1-heptanol # $$

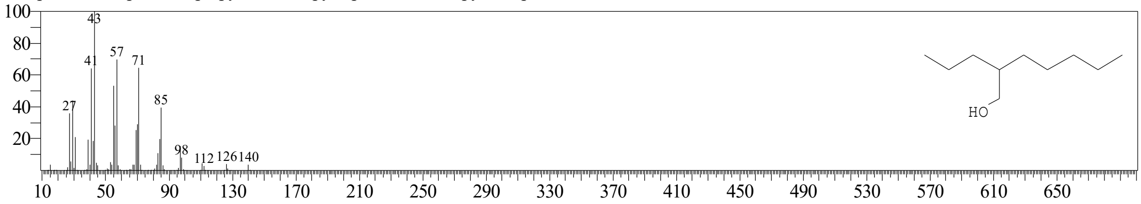

<< Target >>
Line#:30 R.Time:10.030(Scan#:1207) MassPeaks:390
RawMode:Averaged 10.025-10.035(1206-1208) BasePeak:41.10(180709)
BG Mode:Calc. from Peak Group 1 - Event 1 Scan

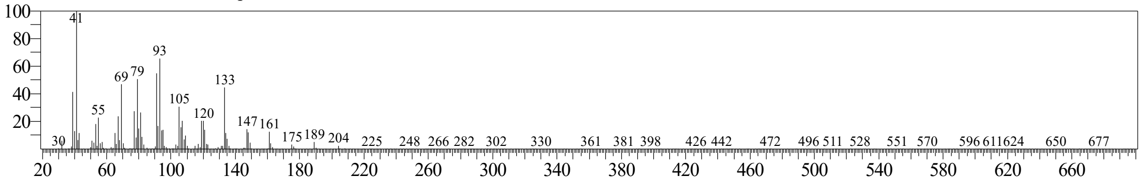

Hit#:1 Entry:46586 Library:NIST11.lib
SI:93 Formula:C15H24 CAS:118-65-0 MolWeight:204 RetIndex:1494
CompName:Bicyclo[7.2.0]undec-4-ene, 4,11,11-trimethyl-8-methylene-,[1R-(1R*,4Z,9S*)]- $$ Isocaryophyllene $$ 4,11,11-Trimethyl-8-methylenebicyclo[7.2.(

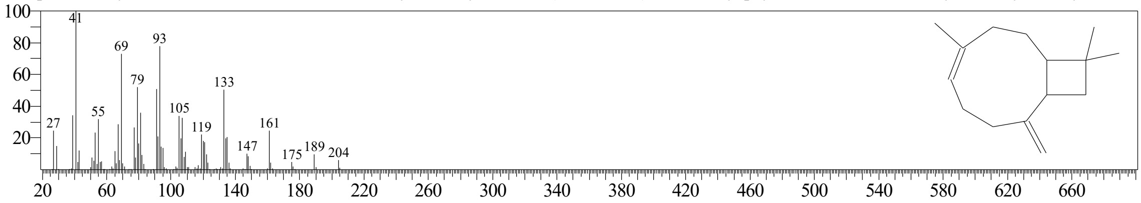

Hit#:2 Entry:46612 Library:NIST11.lib
SI:88 Formula:C15H24 CAS:0-00-0 MolWeight:204 RetIndex:1407
CompName:Bicyclo[5.2.0]nonane, 4-methylene-2,8,8-trimethyl-2-vinyl- $$ 2,8,8-Trimethyl-4-methylene-2-vinylbicyclo[5.2.0]nonane # $$

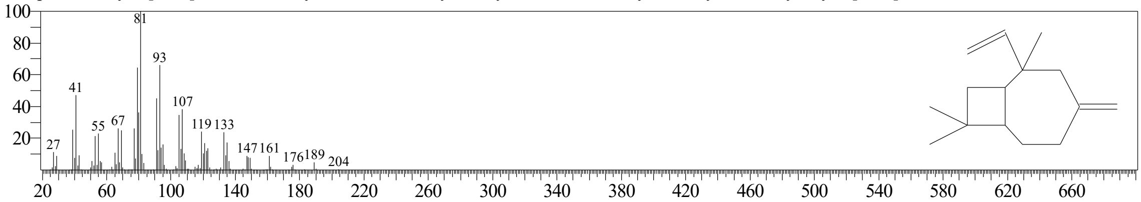

Hit#:3 Entry:46618 Library:NIST11.lib
SI:88 Formula:C15H24 CAS:13877-93-5 MolWeight:204 RetIndex:1494
CompName:Bicyclo[7.2.0]undec-4-ene, 4,11,11-trimethyl-8-methylene- $$ Bicyclo[7.2.0]undec-4-ene, 4,11,11-trimethyl-8-methylene-, (Z)- $$ cis-4,11,11-Trim

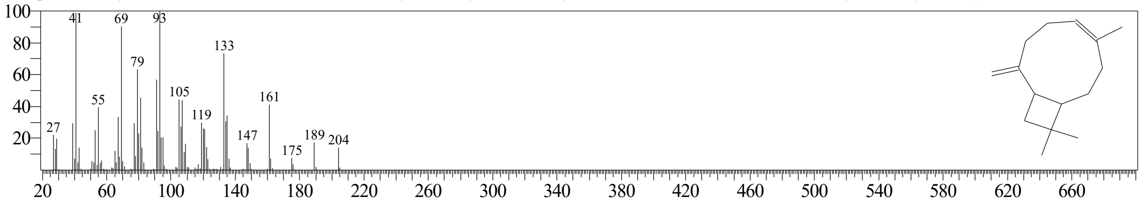

Hit#:4 Entry:46619 Library:NIST11.lib
SI:88 Formula:C15H24 CAS:29837-07-8 MolWeight:204 RetIndex:1518
CompName:cis-.alpha.-Bisabolene $$ 4-[(1Z)-1,5-Dimethyl-1,4-hexadienyl]-1-methyl-1-cyclohexene $$ .alpha.-Bisabolene (Z) $$ (Z)-.alpha.-Bisabolene $$ Cy

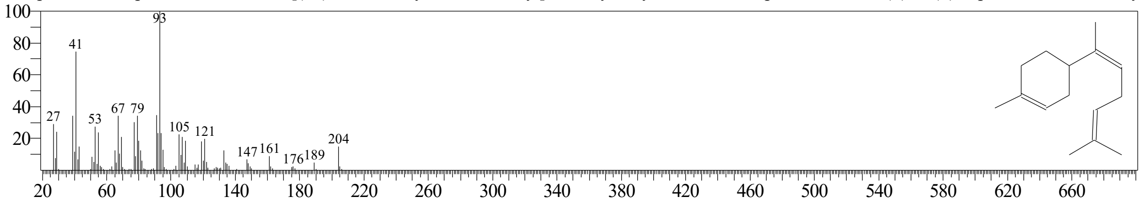

Hit#:5 Entry:46518 Library:NIST11.lib
SI:86 Formula:C13H20N2 CAS:0-00-0 MolWeight:204 RetIndex:0
CompName:1,4-Methanocycloocta[d]pyridazine, 1,4,4a,5,6,9,10,10a-octahydro-11,11-dimethyl-, (1.alpha.,4.alpha.,4aalpha.,10a.alpha.)-

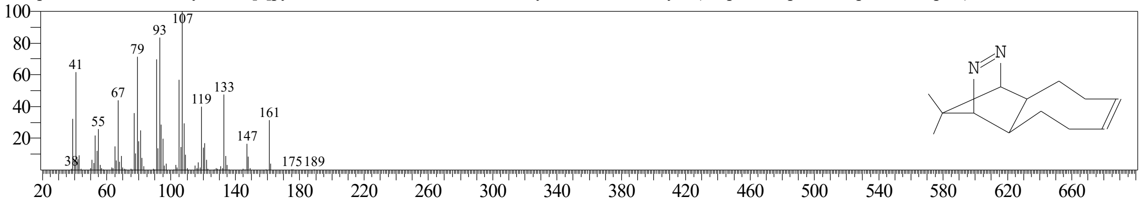

<< Target >>
Line#:31 R.Time:10.350(Scan#:1271) MassPeaks:437
RawMode:Averaged 10.345-10.355(1270-1272) BasePeak:93.05(222983)
BG Mode:Calc. from Peak Group 1 - Event 1 Scan

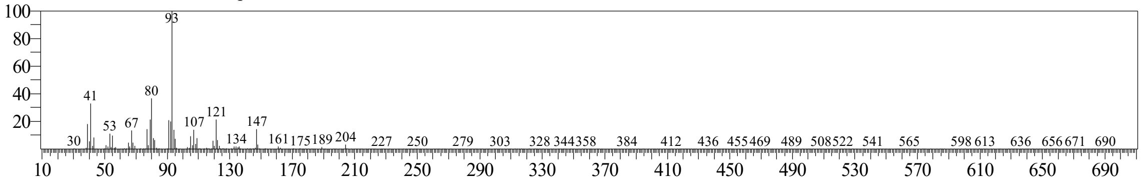

Hit#:1 Entry:46627 Library:NIST11.lib
SI:94 Formula:C15H24 CAS:6753-98-6 MolWeight:204 RetIndex:1579
CompName:Humulene $$ .alpha.-Caryophyllene $$ 1,4,8-Cycloundecatriene, 2,6,6,9-tetramethyl-, (E,E,E)- $$ .alpha.-Humulene $$ Cycloundeca-1,4,8-triene,2,

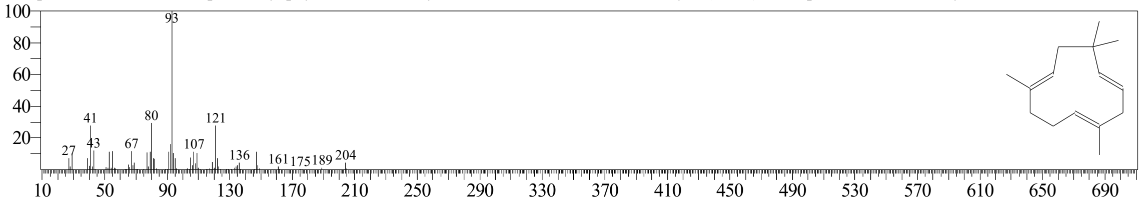

Hit#:2 Entry:46629 Library:NIST11.lib
SI:91 Formula:C15H24 CAS:0-00-0 MolWeight:204 RetIndex:1579
CompName:1,4,7,-Cycloundecatriene, 1,5,9,9-tetramethyl-, Z,Z,Z- $$ 1,5,9,9-Tetramethyl-1,4,7-cycloundecatriene # $$

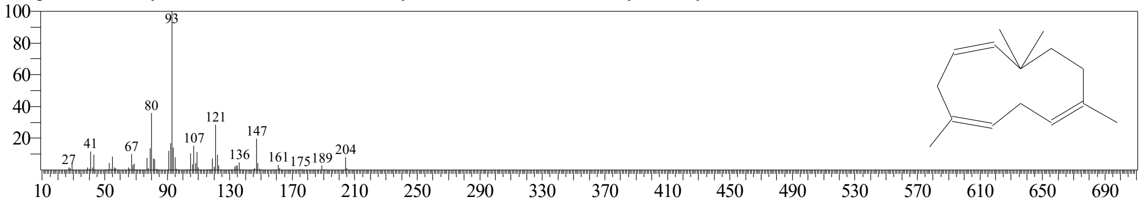

Hit#:3 Entry:9810 Library:NIST11.lib
SI:87 Formula:C10H16 CAS:498-15-7 MolWeight:136 RetIndex:948
CompName:(+)-3-Carene $$ Bicyclo[4.1.0]hept-3-ene, 3,7,7-trimethyl-, (1S)- $$ 3,7,7-Trimethylbicyclo[4.1.0]hept-3-ene-, (1S,6R)- $$ Isodiprene $$ (+)-.delta.

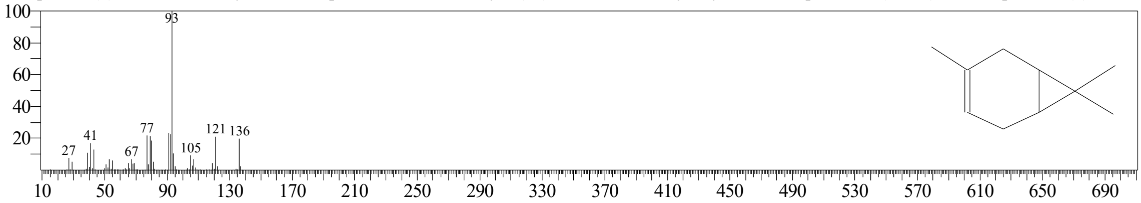

Hit#:4 Entry:9782 Library:NIST11.lib
SI:87 Formula:C10H16 CAS:488-97-1 MolWeight:136 RetIndex:729
CompName:Tricyclo[2.2.1.0(2,6)]heptane, 1,3,3-trimethyl- $$ Cyclofenchene $$ Tricyclo[2.2.1.02,6]heptane, 1,3,3-trimethyl- $$

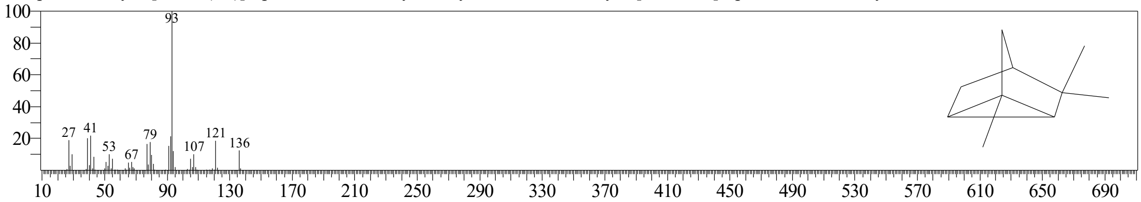

Hit#:5 Entry:9779 Library:NIST11.lib
SI:87 Formula:C10H16 CAS:3338-55-4 MolWeight:136 RetIndex:976
CompName:1,3,6-Octatriene, 3,7-dimethyl-, (Z)- $$ .beta.-cis-Ocimene $$ cis-.beta.-Ocimene $$ cis-3,7-Dimethyl-1,3,6-octatriene $$ Ocimene, cis-.beta.- $$ (Z

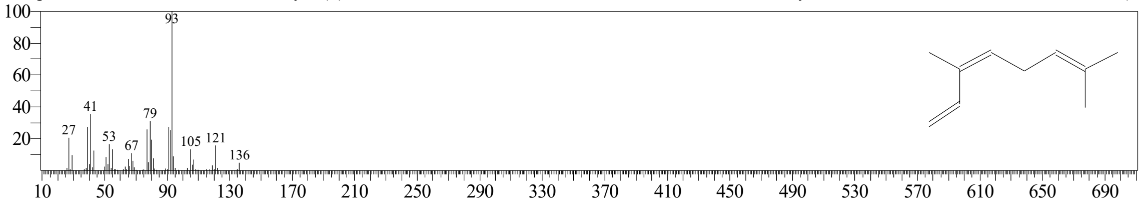

<< Target >>
Line#:32 R.Time:10.565(Scan#:1314) MassPeaks:409
RawMode:Averaged 10.560-10.570(1313-1315) BasePeak:161.10(115326)
BG Mode:Calc. from Peak Group 1 - Event 1 Scan

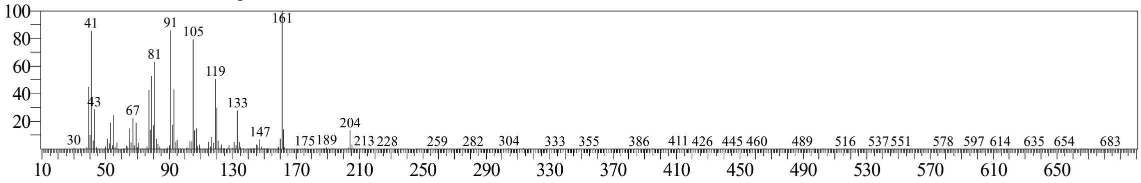

Hit#:1 Entry:46722 Library:NIST11.lib
SI:93 Formula:C15H24 CAS:23986-74-5 MolWeight:204 RetIndex:1515
CompName:1,6-Cyclodecadiene, 1-methyl-5-methylene-8-(1-methylethyl)-, [S-(E,E)]- $$ Germacrene D $$ 8-Isopropyl-1-methyl-5-methylene-1,6-cyclodecadie

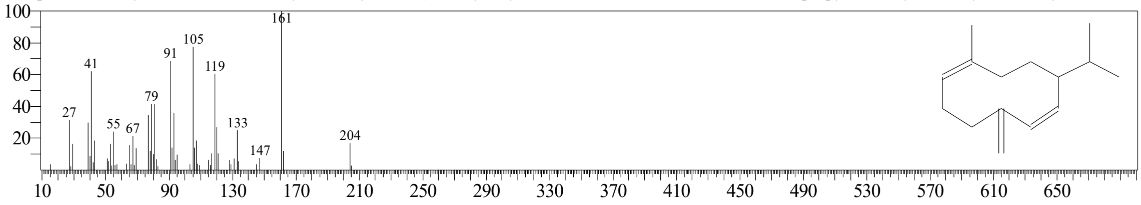

Hit#:2 Entry:46691 Library:NIST11.lib
SI:91 Formula:C15H24 CAS:0-00-0 MolWeight:204 RetIndex:1216
CompName:.beta.-ylangene

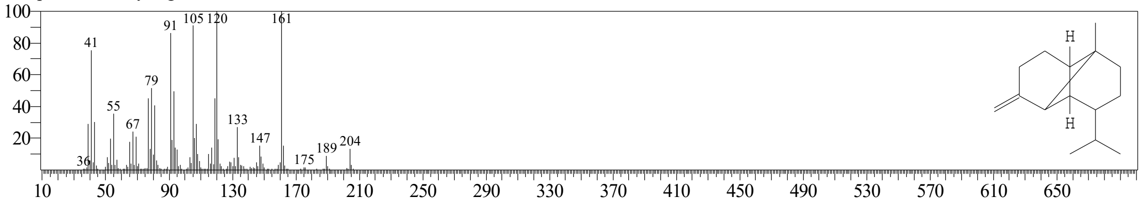

Hit#:3 Entry:46720 Library:NIST11.lib
SI:89 Formula:C15H24 CAS:0-00-0 MolWeight:204 RetIndex:1216
CompName:.beta.-copaene

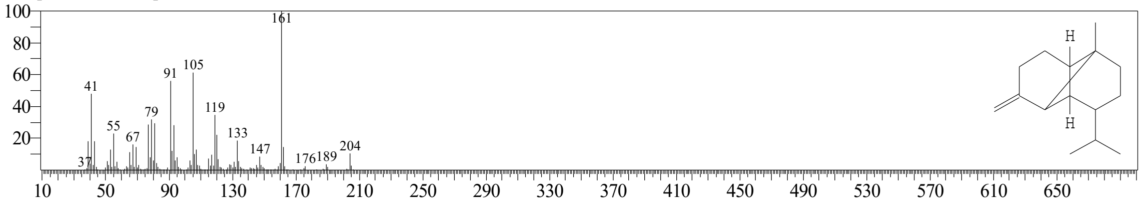

Hit#:4 Entry:29328 Library:NIST11.lib
SI:86 Formula:C13H20 CAS:0-00-0 MolWeight:176 RetIndex:1251
CompName:Bicyclo[2.2.1]heptane, 2-cyclopropylidene-1,7,7-trimethyl- $$ 2-Cyclopropylidene-1,7,7-trimethylbicyclo[2.2.1]heptane # $$

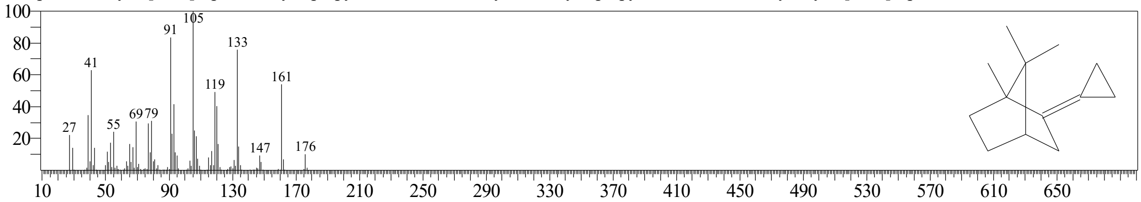

Hit#:5 Entry:46595 Library:NIST11.lib
SI:86 Formula:C15H24 CAS:0-00-0 MolWeight:204 RetIndex:1281
CompName:Bicyclo[4.1.0]-3-heptene, 2-isopropenyl-5-isopropyl-7,7-dimethyl- $$ 2-Isopropenyl-5-isopropyl-7,7-dimethylbicyclo[4.1.0]hept-3-ene # $$

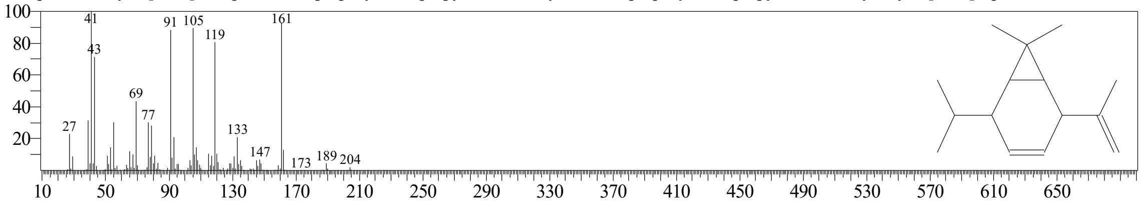

<< Target >>
Line#:33 R.Time:11.125(Scan#:1426) MassPeaks:409
RawMode:Averaged 11.120-11.130(1425-1427) BasePeak:93.05(31011)
BG Mode:Calc. from Peak Group 1 - Event 1 Scan

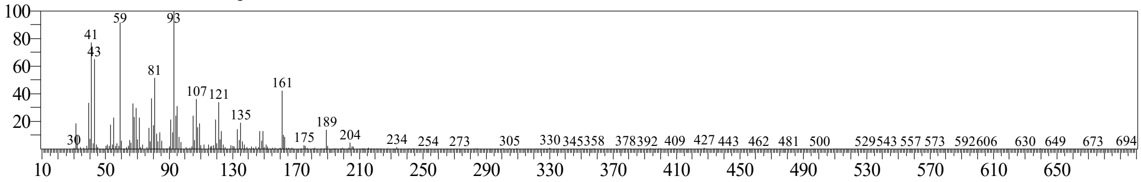

Hit#:1 Entry:59376 Library:NIST11.lib
SI:91 Formula:C15H26O CAS:639-99-6 MolWeight:222 RetIndex:1522
CompName:Cyclohexanemethanol, 4-ethenyl-.alpha.,.alpha.,4-trimethyl-3-(1-methylethenyl)-, [1R-(1.alpha.,3.alpha.,4.beta.)]- $$ o-Menth-8-ene-4-methanol, .a

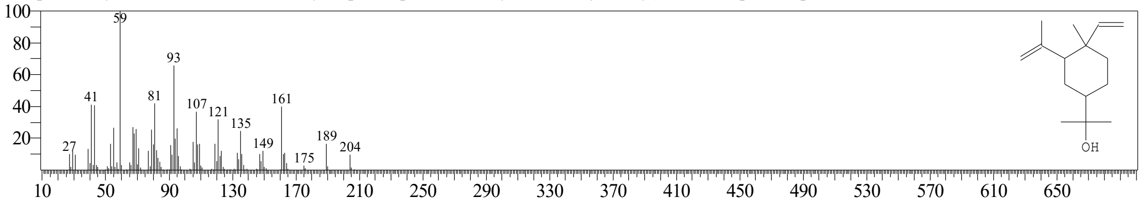

Hit#:2 Entry:59377 Library:NIST11.lib
SI:88 Formula:C15H26O CAS:21657-90-9 MolWeight:222 RetIndex:1694
CompName:3,7-Cyclodecadiene-1-methanol, .alpha.,.alpha.,4,8-tetramethyl-, [s-(Z,Z)] $$ Hedycaryol $$ 2-(4,8-Dimethyl-3,7-cyclodecadien-1-yl)-2-propanol #

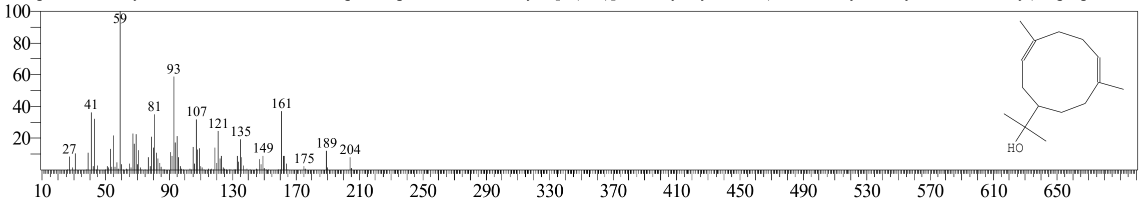

Hit#:3 Entry:59430 Library:NIST11.lib
SI:84 Formula:C15H26O CAS:22451-73-6 MolWeight:222 RetIndex:1614
CompName:5-Azulenemethanol, 1,2,3,3a,4,5,6,7-octahydro-.alpha.,.alpha.,3,8-tetramethyl-, [3S-(3.alpha.,3a.beta.,5.alpha.)]- $$ Guai-1(10)-en-11-ol $$ Bulnesc

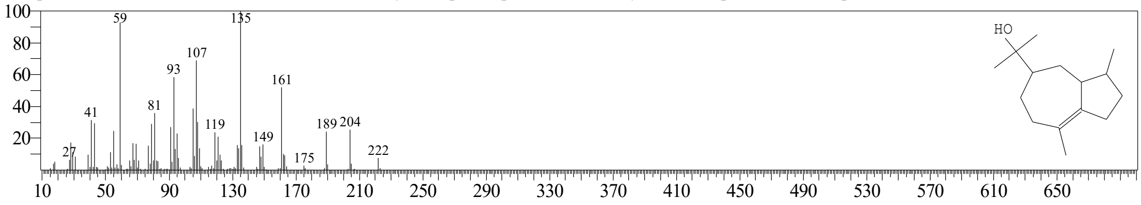

Hit#:4 Entry:59382 Library:NIST11.lib
SI:82 Formula:C15H26O CAS:13822-35-0 MolWeight:222 RetIndex:1614
CompName:5-Azulenemethanol, 1,2,3,4,5,6,7,8-octahydro-.alpha.,.alpha.,3,8-tetramethyl- $$ 2-(3,8-Dimethyl-1,2,3,4,5,6,7,8-octahydro-5-azulenyl)-2-propanol

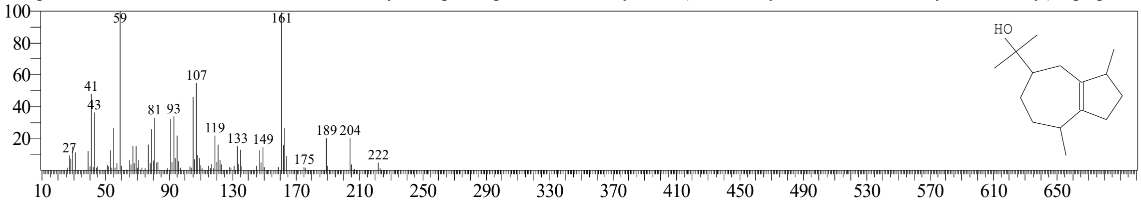

Hit#:5 Entry:59427 Library:NIST11.lib
SI:82 Formula:C15H26O CAS:0-00-0 MolWeight:222 RetIndex:1598
CompName:.alpha.-acorenol

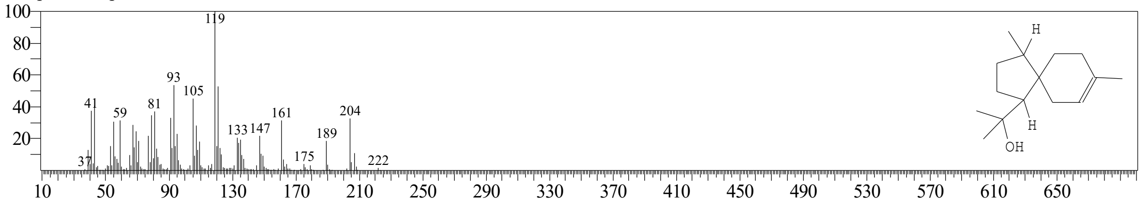

<< Target >>
Line#:34 R.Time:11.350(Scan#:1471) MassPeaks:358
RawMode:Averaged 11.345-11.355(1470-1472) BasePeak:41.05(23122)
BG Mode:Calc. from Peak Group 1 - Event 1 Scan

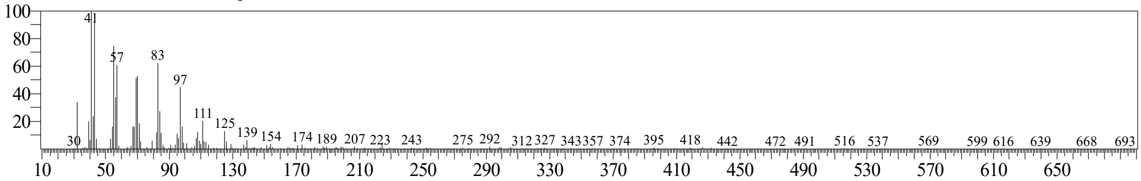

Hit#:1 Entry:51049 Library:NIST11.lib
SI:89 Formula:C15H30 CAS:13360-61-7 MolWeight:210 RetIndex:1502
CompName:1-Pentadecene $$ Pentadecene,1- $$ Pentadec-1-ene $$

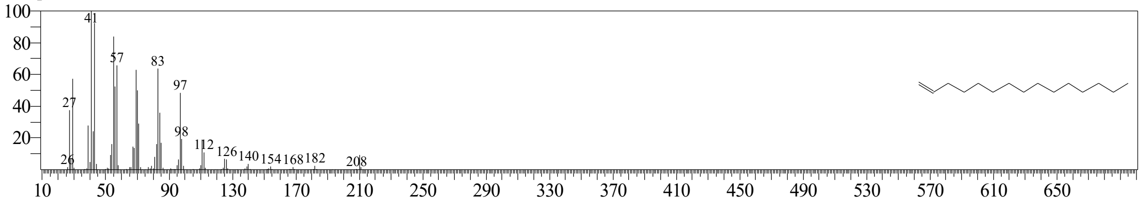

Hit#:2 Entry:70935 Library:NIST11.lib
SI:89 Formula:C17H34 CAS:6765-39-5 MolWeight:238 RetIndex:1701
CompName:1-Heptadecene $$ Hexahydroaplotaxene $$ Heptadec-1-ene $$ NSC 77132 $$ Heptadecene (Related) $$

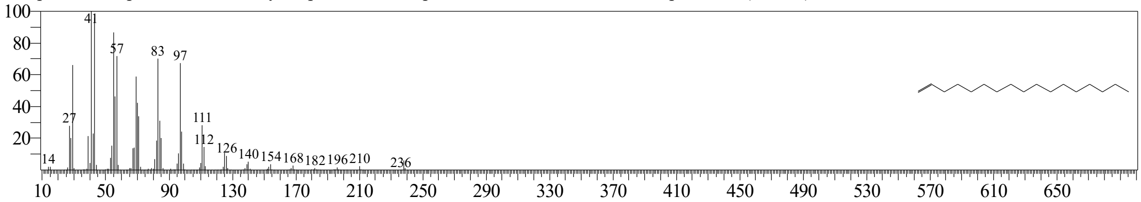

Hit#:3 Entry:60964 Library:NIST11.lib
SI:89 Formula:C16H32 CAS:629-73-2 MolWeight:224 RetIndex:1602
CompName:Cetene $$ 1-Hexadecene $$ .alpha.-Hexadecene $$ n-Hexadec-1-ene $$ 1-Cetene $$ Hexadecylene-1 $$ Hexadec-1-ene $$ Hexadecene-1 $$ Neode

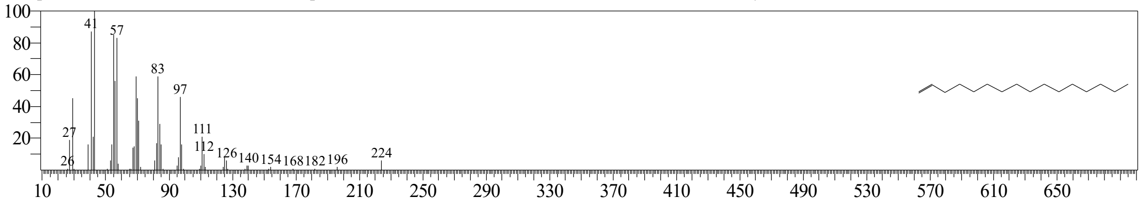

Hit#:4 Entry:60963 Library:NIST11.lib
SI:89 Formula:C16H32 CAS:34303-81-6 MolWeight:224 RetIndex:1620
CompName:3-Hexadecene, (Z)- $$ (3Z)-3-Hexadecene $$ cis-3-Hexadecene $$ (Z)-3-Hexadecene $$

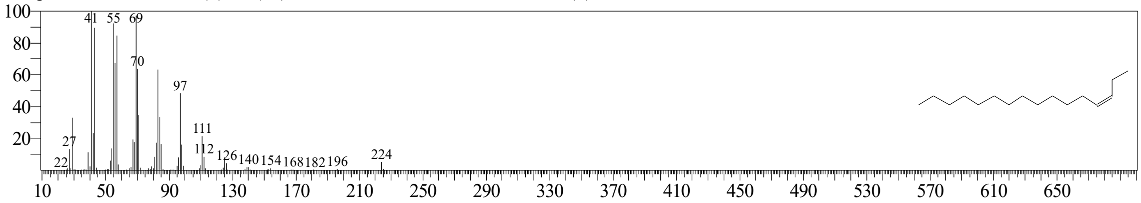

Hit#:5 Entry:24832 Library:NIST11.lib
SI:88 Formula:C12H24 CAS:112-41-4 MolWeight:168 RetIndex:1204
CompName:1-Dodecene $$ .alpha.-Dodecene $$ n-Dodec-1-ene $$ Adacene 12 $$ Dodec-1-ene $$ .alpha.-Dodecylene $$ Dodecylene .alpha.- $$ Dodecene-1 $

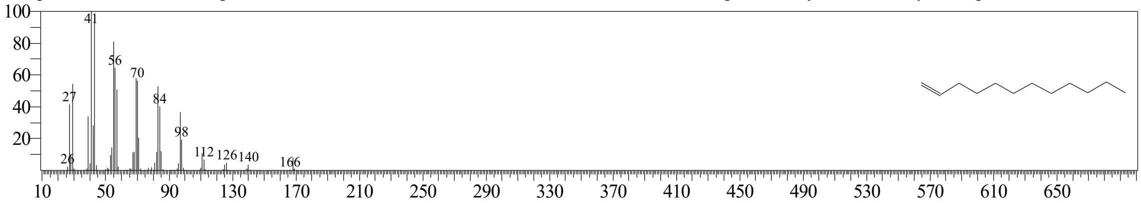

<< Target >>
Line#:35 R.Time:11.450(Scan#:1491) MassPeaks:391
RawMode:Averaged 11.445-11.455(1490-1492) BasePeak:41.05(45417)
BG Mode:Calc. from Peak Group 1 - Event 1 Scan

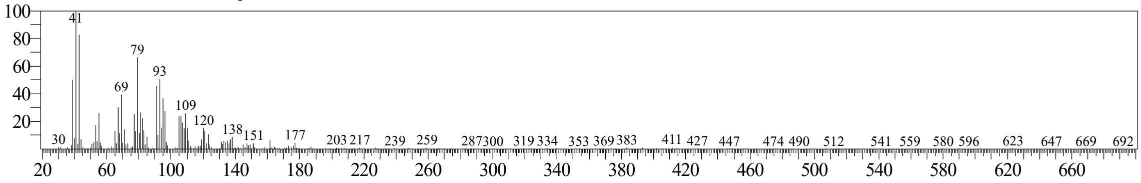

Hit#:1 Entry:57732 Library:NIST11.lib
SI:91 Formula:C15H24O CAS:1139-30-6 MolWeight:220 RetIndex:1507
CompName:Caryophyllene oxide $$ 5-Oxatricyclo[8.2.0.0(4,6)-]dodecane, 4,12,12-trimethyl-9-methylene-, [1R-(1R*,4R*,6R*,10S*)]- $$ 5-Oxatricyclo(8.2.0.

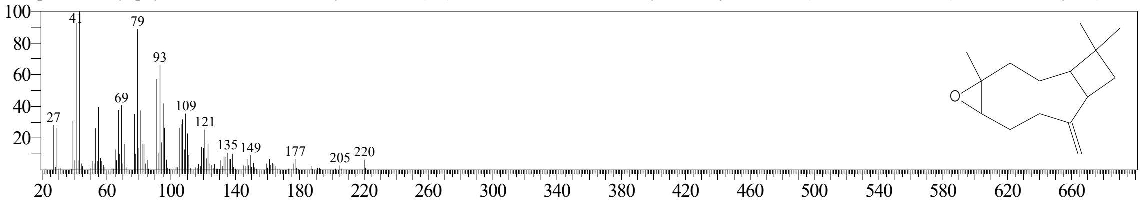

Hit#:2 Entry:16479 Library:NIST11.lib
SI:83 Formula:C10H16O CAS:35907-10-9 MolWeight:152 RetIndex:1201
CompName:p-Mentha-1(7),8-dien-2-ol $$ Cyclohexanol, 2-methylene-5-(1-methylethenyl)- $$ 5-Isopropenyl-2-methylenecyclohexanol $$ Isocarveol $$ 1(7),8-

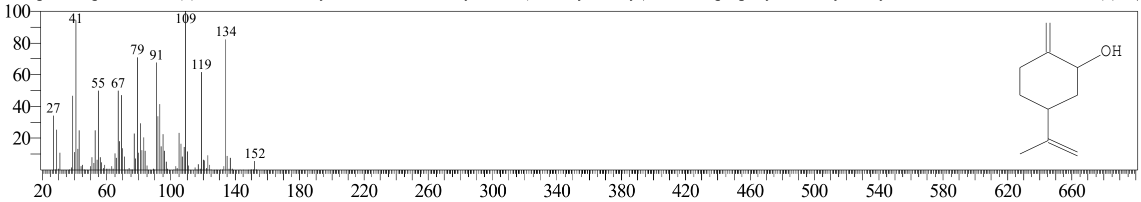

Hit#:3 Entry:16312 Library:NIST11.lib
SI:82 Formula:C10H16O CAS:0-00-0 MolWeight:152 RetIndex:1201
CompName:cis-p-mentha-1(7),8-dien-2-ol

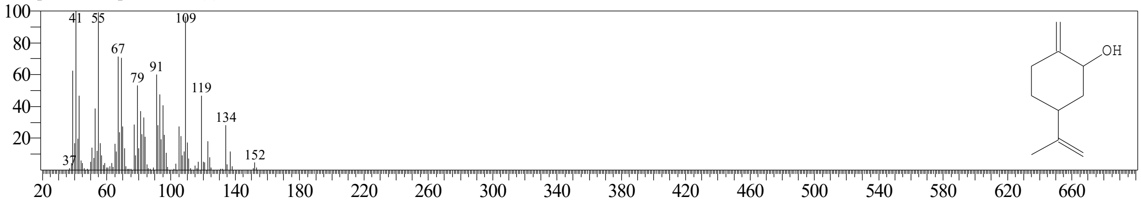

Hit#:4 Entry:37595 Library:NIST11.lib
SI:82 Formula:C14H22 CAS:62338-27-6 MolWeight:190 RetIndex:1404
CompName:1,5,9,11-Tridecatetraene, 12-methyl-, (E,E)- $$ (5E,9E)-12-Methyl-1,5,9,11-tridecatetraene # $$

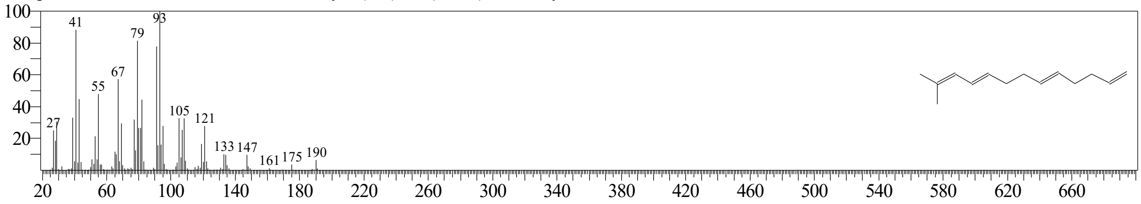

Hit#:5 Entry:23589 Library:NIST11.lib
SI:82 Formula:C11H18O CAS:0-00-0 MolWeight:166 RetIndex:1307
CompName:Bicyclo[4.1.0]heptane,-3-cyclopropyl,-7-hydroxymethyl, (cis) $$ (3-Cyclopropylbicyclo[4.1.0]hept-7-yl)methanol # $$

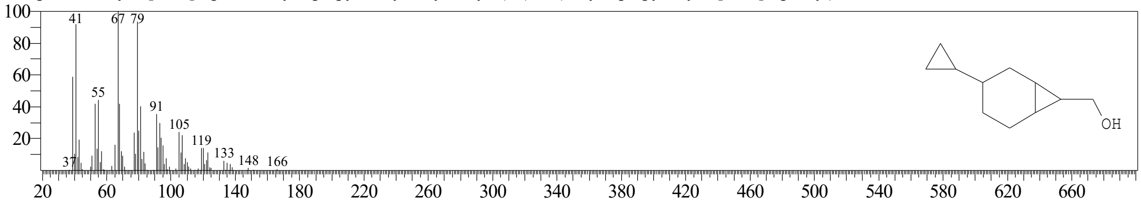

<< Target >>
Line#:36 R.Time:11.580(Scan#:1517) MassPeaks:393
RawMode:Averaged 11.575-11.585(1516-1518) BasePeak:43.05(32666)
BG Mode:Calc. from Peak Group 1 - Event 1 Scan

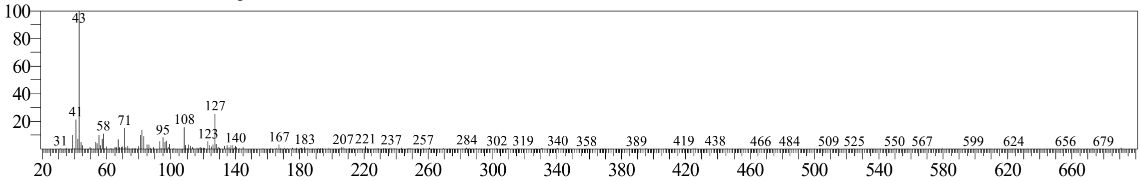

Hit#:1 Entry:18581 Library:NIST11.lib
SI:78 Formula:C10H20O CAS:118452-32-7 MolWeight:156 RetIndex:1023
CompName:Octan-2-one, 3,6-dimethyl- $$ 3,6-Dimethyl-2-octanone # $$

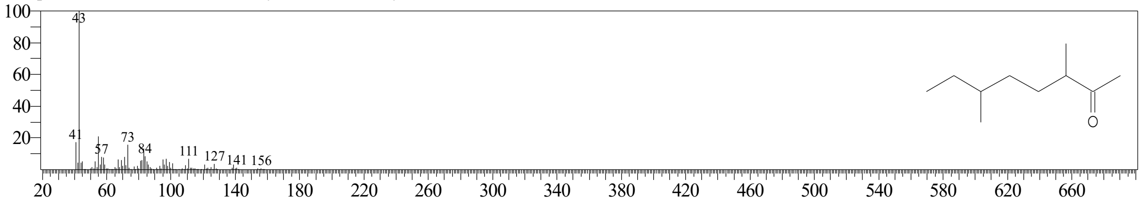

Hit#:2 Entry:82678 Library:NIST11.lib
SI:77 Formula:C16H30O2 CAS:18650-13-0 MolWeight:254 RetIndex:1884
CompName:2,15-Hexadecanedione

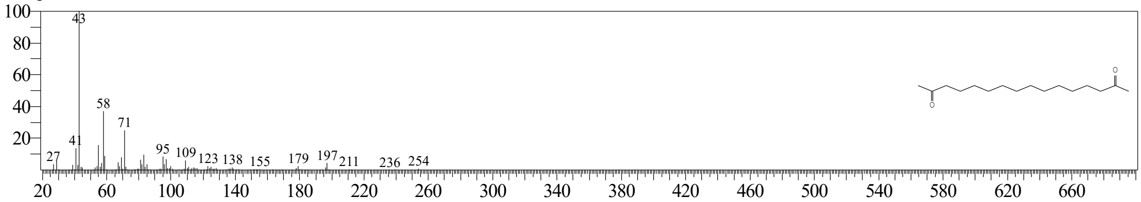

Hit#:3 Entry:43832 Library:NIST11.lib
SI:77 Formula:C11H20O3 CAS:34647-45-5 MolWeight:200 RetIndex:1370
CompName:2H-Pyran-3-ol, tetrahydro-3-methyl-6-propyl-, acetate $$ 2-Propyl-5-acetoxy-5-methyltetrahydropyran $$ 3-Methyl-6-propyltetrahydro-2H-pyran-3-

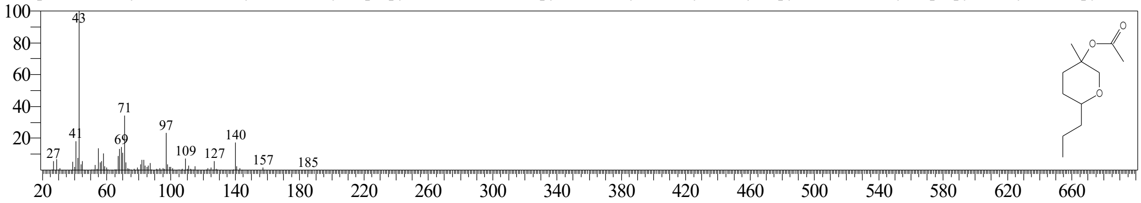

Hit#:4 Entry:23582 Library:NIST11.lib
SI:76 Formula:C11H18O CAS:0-00-0 MolWeight:166 RetIndex:1208
CompName:9-Decen-2-one, 5-methylene- $$ 5-Methylene-9-decen-2-one # $$

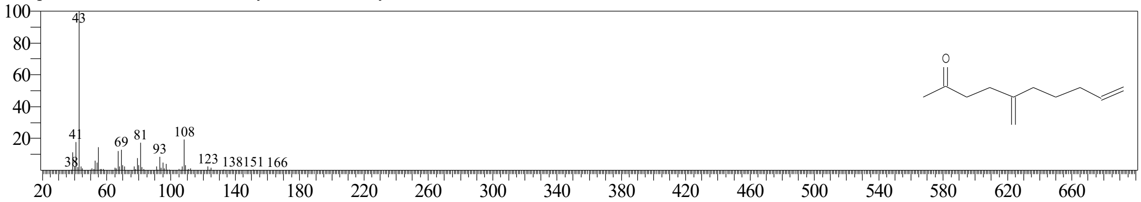

Hit#:5 Entry:6519 Library:NIST11.lib
SI:76 Formula:C8H14O CAS:3664-60-6 MolWeight:126 RetIndex:943
CompName:7-Octen-2-one

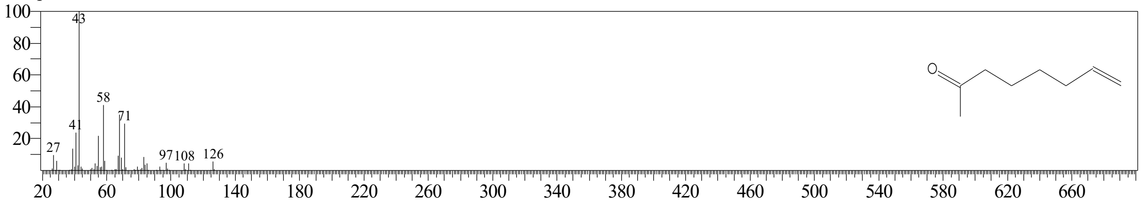

<< Target >>
Line#:37 R.Time:11.680(Scan#:1537) MassPeaks:395
RawMode:Averaged 11.675-11.685(1536-1538) BasePeak:43.05(26037)
BG Mode:Calc. from Peak Group 1 - Event 1 Scan

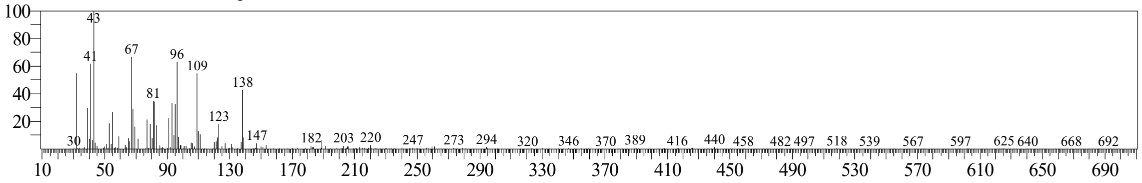

Hit#:1 Entry:57741 Library:NIST11.lib
SI:80 Formula:C15H24O CAS:19888-34-7 MolWeight:220 RetIndex:1592
CompName:12-Oxabicyclo[9.1.0]dodeca-3,7-diene, 1,5,5,8-tetramethyl-, [1R-(1R*,3E,7E,11R*)]- $$ 1,5,5,8-Tetramethyl-12-oxabicyclo[9.1.0]dodeca-3,7-dien

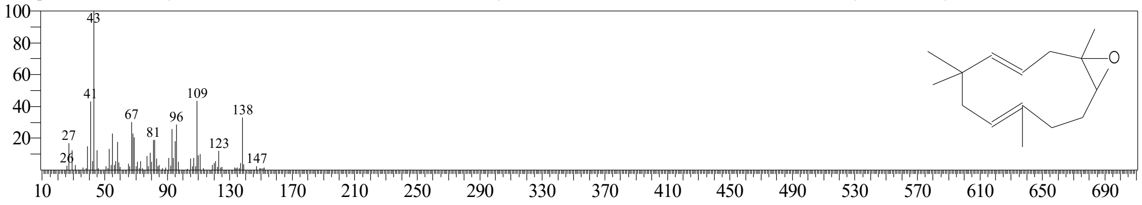

Hit#:2 Entry:10522 Library:NIST11.lib
SI:78 Formula:C10H18 CAS:493-02-7 MolWeight:138 RetIndex:1101
CompName:Naphthalene, decahydro-, trans- $$ trans-Bicyclo[4.4.0]Decane $$ trans-Decahydronaphthalene $$ trans-Decalin $$ trans-Perhydronaphthalene $$ I

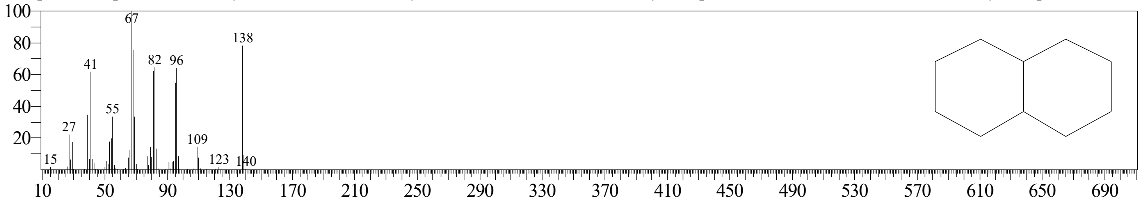

Hit#:3 Entry:10593 Library:NIST11.lib
SI:78 Formula:C10H18 CAS:176-63-6 MolWeight:138 RetIndex:1114
CompName:Spiro[4.5]decane $$ Spiro[4.5]decan $$ Spiro(4,5)decane $$

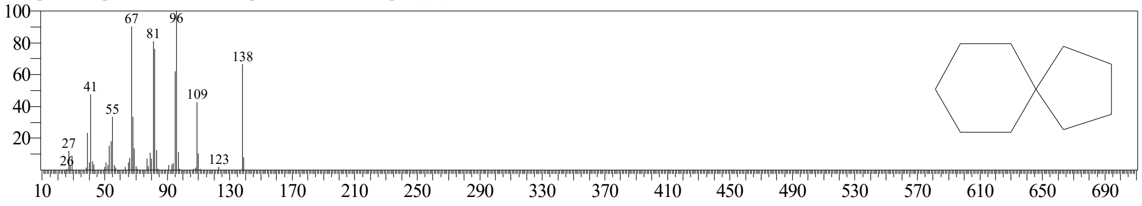

Hit#:4 Entry:62277 Library:NIST11.lib
SI:78 Formula:C14H26O2 CAS:16676-96-3 MolWeight:226 RetIndex:1588
CompName:5-Dodecen-1-ol, acetate, (Z)- $$ Z-5-Dodecen-1-yl acetate $$ (5Z)-5-Dodecenyl acetate $$ Z-5-dodecenyl acetate $$ 5-Dodecen-1-ol, acetate, (5Z)-

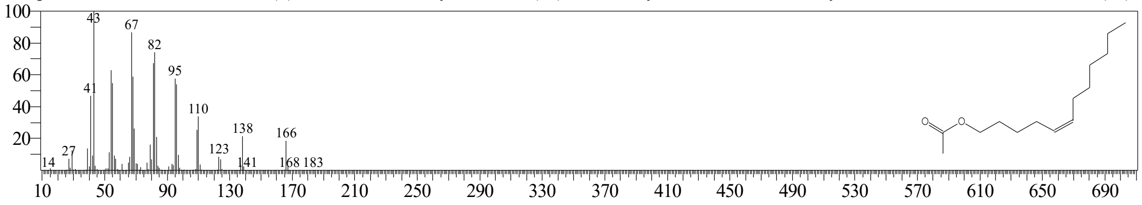

Hit#:5 Entry:42683 Library:NIST11.lib
SI:78 Formula:C12H22O2 CAS:13857-03-9 MolWeight:198 RetIndex:1389
CompName:Z-7-Decen-1-yl acetate $$ (7Z)-7-Decenyl acetate $$ (Z)-7-Decenyl acetate $$

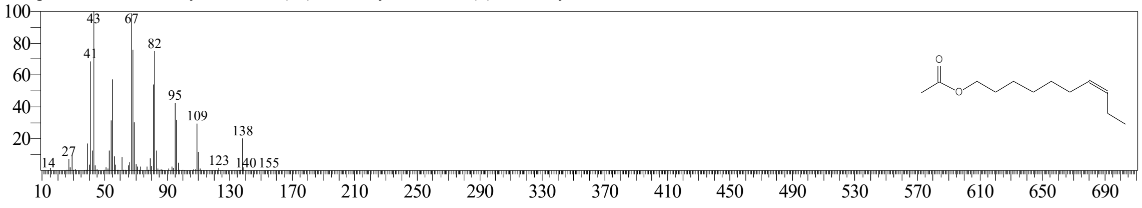

<< Target >>
Line#:38 R.Time:12.050(Scan#:1611) MassPeaks:395
RawMode:Averaged 12.045-12.055(1610-1612) BasePeak:59.00(22109)
BG Mode:Calc. from Peak Group 1 - Event 1 Scan

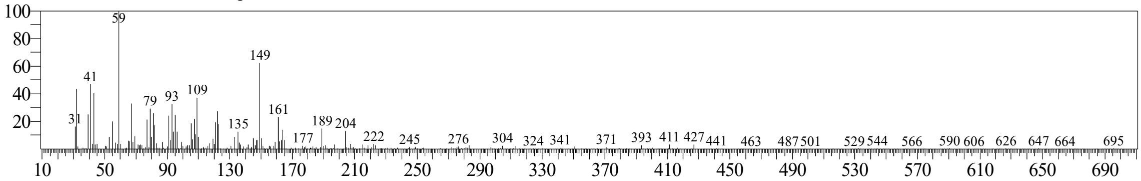

Hit#:1 Entry:59380 Library:NIST11.lib
SI:83 Formula:C15H26O CAS:473-15-4 MolWeight:222 RetIndex:1593
CompName:2-Naphthalenemethanol, decahydro-.alpha.,.alpha.,4a-trimethyl-8-methylene-, [2R-(2.alpha.,4a.alpha.,8a.beta.)]- $$ Eudesm-4(14)-en-11-ol $$ .beta

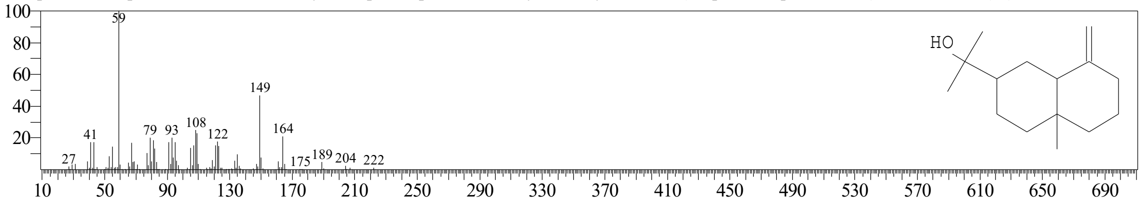

Hit#:2 Entry:59376 Library:NIST11.lib
SI:82 Formula:C15H26O CAS:639-99-6 MolWeight:222 RetIndex:1522
CompName:Cyclohexanemethanol, 4-ethenyl-.alpha.,.alpha.,4-trimethyl-3-(1-methylethenyl)-, [1R-(1.alpha.,3.alpha.,4.beta.)]- $$ o-Menth-8-ene-4-methanol, .a

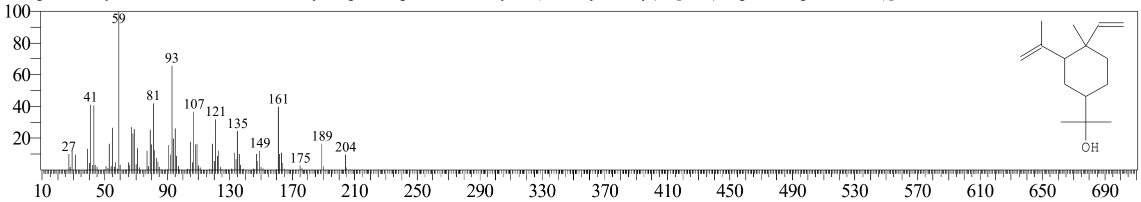

Hit#:3 Entry:59377 Library:NIST11.lib
SI:82 Formula:C15H26O CAS:21657-90-9 MolWeight:222 RetIndex:1694
CompName:3,7-Cyclodecadiene-1-methanol, .alpha.,.alpha.,4,8-tetramethyl-, [s-(Z,Z)] $$ Hedycaryol $$ 2-(4,8-Dimethyl-3,7-cyclodecadien-1-yl)-2-propanol #

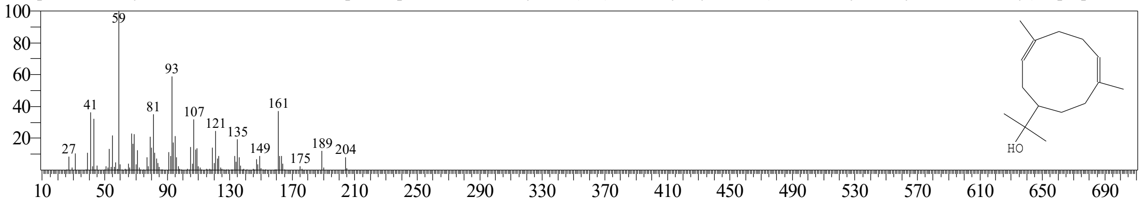

Hit#:4 Entry:59382 Library:NIST11.lib
SI:81 Formula:C15H26O CAS:13822-35-0 MolWeight:222 RetIndex:1614
CompName:5-Azulenemethanol, 1,2,3,4,5,6,7,8-octahydro-.alpha.,.alpha.,3,8-tetramethyl- $$ 2-(3,8-Dimethyl-1,2,3,4,5,6,7,8-octahydro-5-azulenyl)-2-propanol

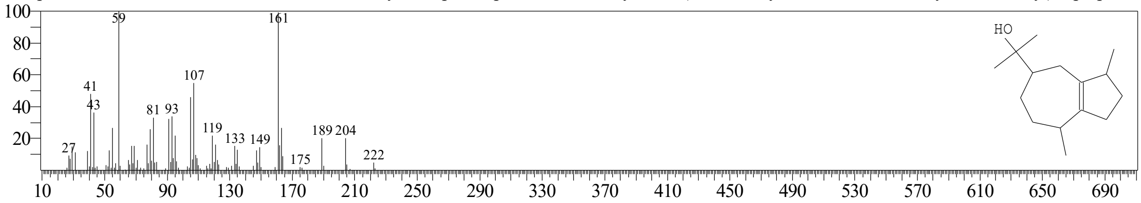

Hit#:5 Entry:59379 Library:NIST11.lib
SI:81 Formula:C15H26O CAS:63891-61-2 MolWeight:222 RetIndex:1598
CompName:2-Naphthalenemethanol, 2,3,4,4a,5,6,7,8-octahydro-.alpha.,.alpha.,4a,8-tetramethyl-, [2R-(2.alpha.,4a.beta.,8.beta.)]- $$ 2-(4a,8-Dimethyl-2,3,4,4a,

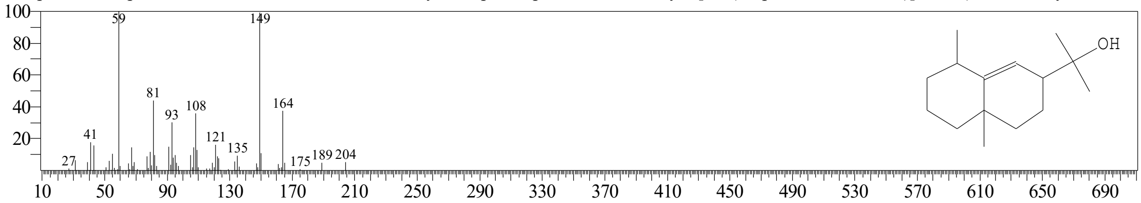

<< Target >>
Line#:39 R.Time:12.350(Scan#:1671) MassPeaks:401
RawMode:Averaged 12.345-12.355(1670-1672) BasePeak:41.10(80130)
BG Mode:Calc. from Peak Group 1 - Event 1 Scan

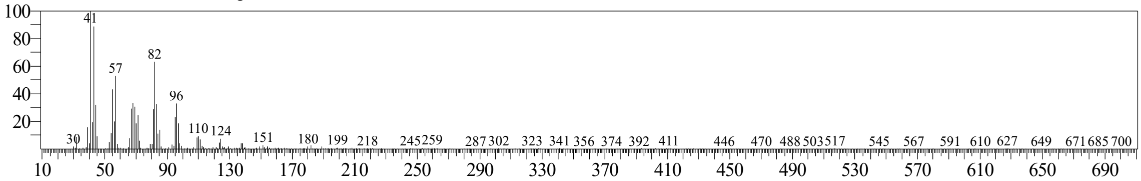

Hit#:1 Entry:93586 Library:NIST11.lib
SI:93 Formula:C18H36O CAS:638-66-4 MolWeight:268 RetIndex:1999
CompName:Octadecanal $$ Stearaldehyde $$ Octadecyl aldehyde $$ Stearyl aldehyde $$ n-Octadecanal $$

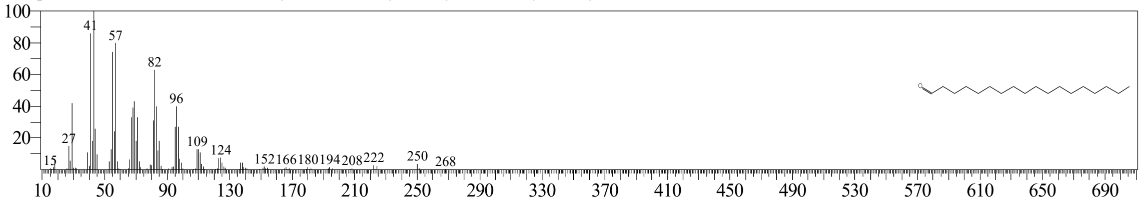

Hit#:2 Entry:26030 Library:NIST11.lib
SI:91 Formula:C11H22O CAS:112-44-7 MolWeight:170 RetIndex:1303
CompName:Undecanal $$ n-Undecanal $$ n-Undecyl aldehyde $$ Hendecanal $$ Hendecanaldehyde $$ Undecyl aldehyde $$ Undecylic aldehyde $$ 1-Undecan

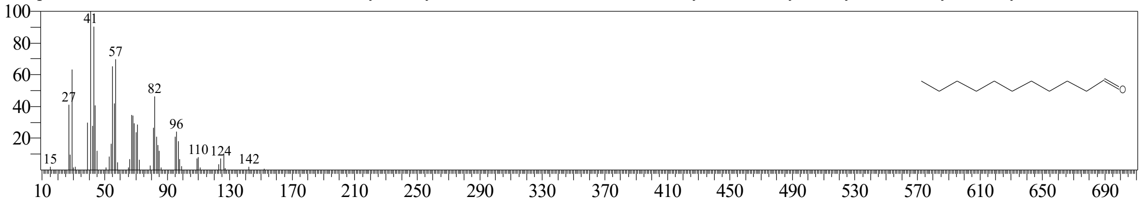

Hit#:3 Entry:52426 Library:NIST11.lib
SI:91 Formula:C14H28O CAS:0-00-0 MolWeight:212 RetIndex:1664
CompName:E-2-Tetradecen-1-ol $$ (2E)-2-Tetradecen-1-ol # $$

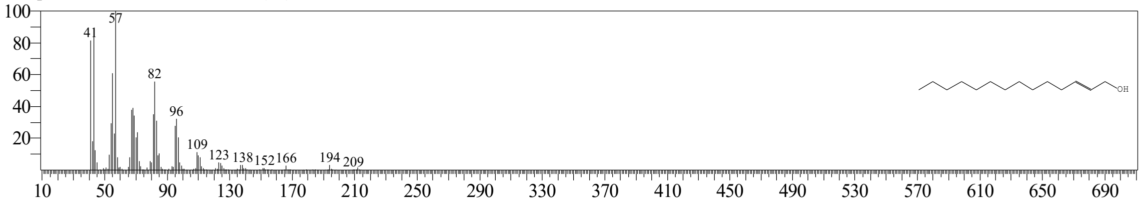

Hit#:4 Entry:42885 Library:NIST11.lib
SI:91 Formula:C13H26O CAS:10486-19-8 MolWeight:198 RetIndex:1502
CompName:Tridecanal $$ n-Tridecylaldehyde $$ Tridecanaldehyde $$ Tridecyl aldehyde $$ 1-Tridecanal $$ Tridecane aldehyde $$ n-Tridecanal $$

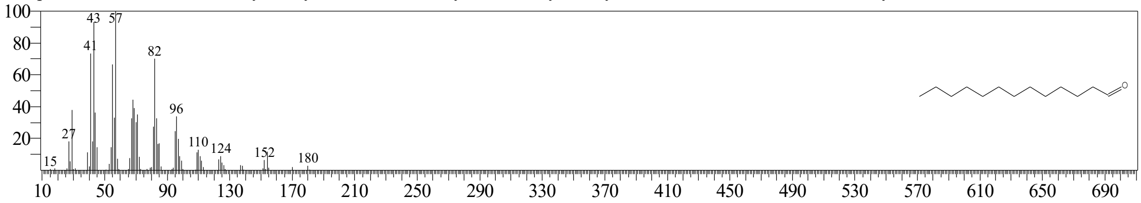

Hit#:5 Entry:34060 Library:NIST11.lib
SI:90 Formula:C12H24O CAS:112-54-9 MolWeight:184 RetIndex:1402
CompName:Dodecanal $$ Lauraldehyde $$ n-Dodecanal $$ n-Dodecyl aldehyde $$ n-Dodecylic aldehyde $$ n-Lauraldehyde $$ Aldehyde C-12 $$ Dodecanalde

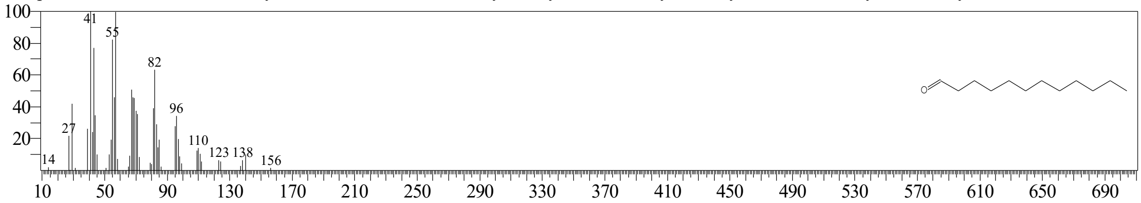

<< Target >>
Line#:40 R.Time:12.765(Scan#:1754) MassPeaks:401
RawMode:Averaged 12.760-12.770(1753-1755) BasePeak:41.05(14956)
BG Mode:Calc. from Peak Group 1 - Event 1 Scan

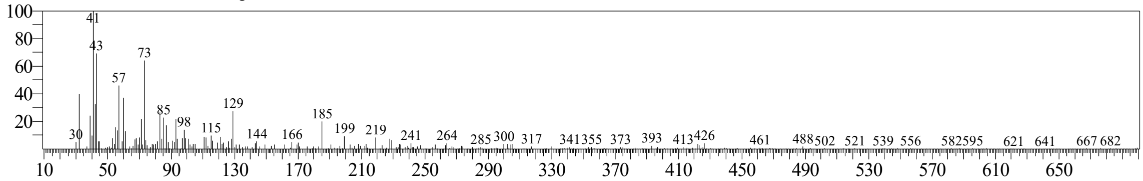

Hit#:1 Entry:63810 Library:NIST11.lib
SI:77 Formula:C14H28O2 CAS:544-63-8 MolWeight:228 RetIndex:1769
CompName:Tetradecanoic acid $$ Myristic acid $$ n-Tetradecanoic acid $$ n-Tetradecoic acid $$ Neo-Fat 14 $$ Univol U 316S $$ 1-Tridecanecarboxylic acid $

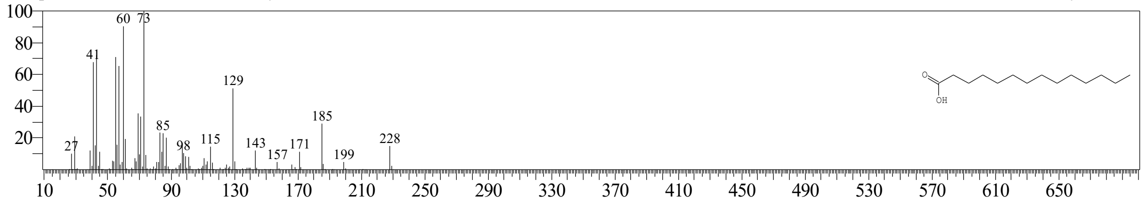

Hit#:2 Entry:53599 Library:NIST11.lib
SI:76 Formula:C13H26O2 CAS:638-53-9 MolWeight:214 RetIndex:1670
CompName:Tridecanoic acid $$ n-Tridecanoic acid $$ n-Tridecoic acid $$ Tridecylic acid $$

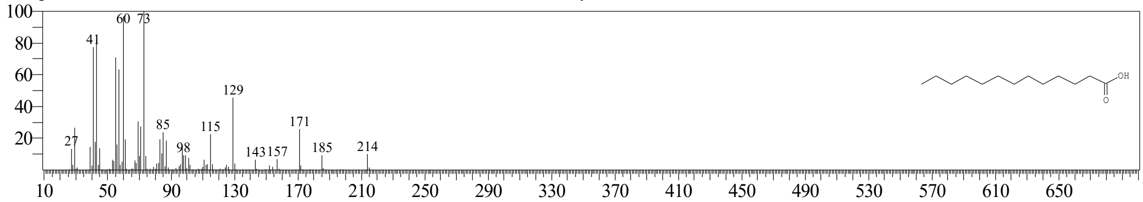

Hit#:3 Entry:128151 Library:NIST11.lib
SI:76 Formula:C20H40O2 CAS:506-30-9 MolWeight:312 RetIndex:2366
CompName:Eicosanoic acid $$ Arachic acid $$ Arachidic acid $$ Icosanoic acid $$ n-Eicosanoic acid $$ Arachidic acid (synthetic) $$

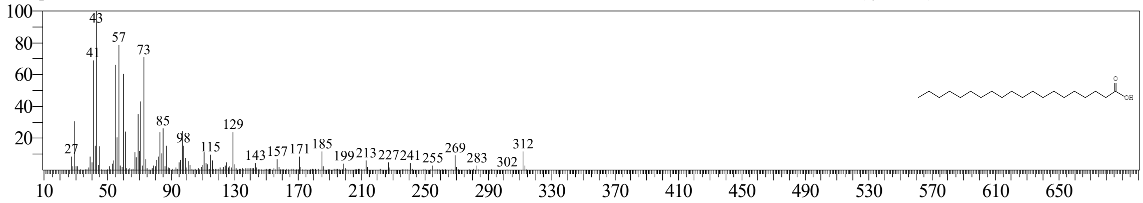

Hit#:4 Entry:73851 Library:NIST11.lib
SI:75 Formula:C15H30O2 CAS:1002-84-2 MolWeight:242 RetIndex:1869
CompName:Pentadecanoic acid $$ Pentadecylic acid $$ n-Pentadecanoic acid $$ n-Pentadecylic acid $$

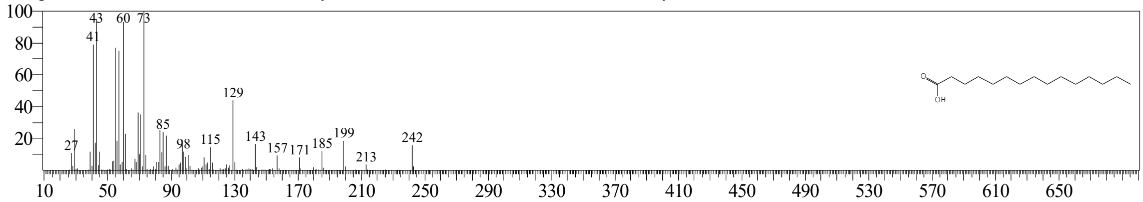

Hit#:5 Entry:84362 Library:NIST11.lib
SI:75 Formula:C16H32O2 CAS:57-10-3 MolWeight:256 RetIndex:1968
CompName:n-Hexadecanoic acid $$ Hexadecanoic acid $$ n-Hexadecoic acid $$ Palmitic acid $$ Pentadecanecarboxylic acid $$ 1-Pentadecanecarboxylic acid

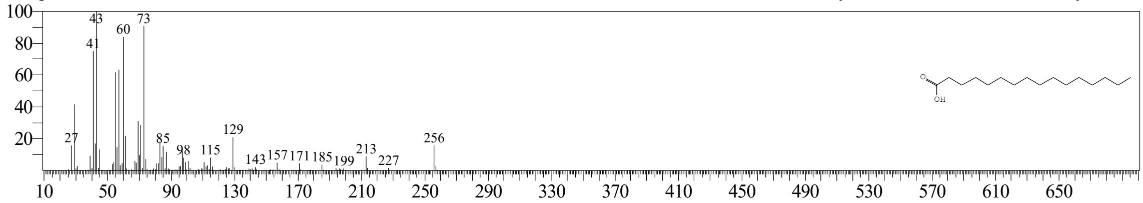

<< Target >>
Line#:41 R.Time:12.855(Scan#:1772) MassPeaks:371
RawMode:Averaged 12.850-12.860(1771-1773) BasePeak:41.10(62049)
BG Mode:Calc. from Peak Group 1 - Event 1 Scan

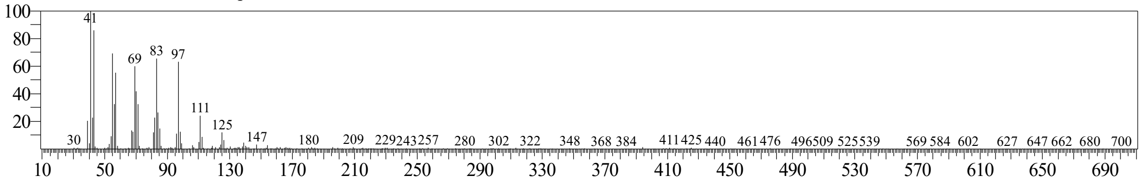

Hit#:1 Entry:70935 Library:NIST11.lib
SI:94 Formula:C17H34 CAS:6765-39-5 MolWeight:238 RetIndex:1701
CompName:1-Heptadecene $$ Hexahydroaplotaxene $$ Heptadec-1-ene $$ NSC 77132 $$ Heptadecene (Related) $$

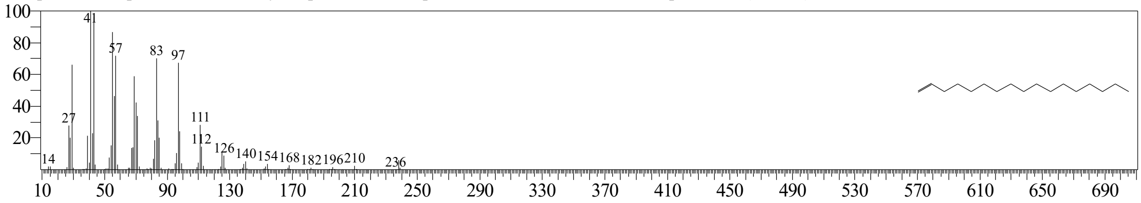

Hit#:2 Entry:51049 Library:NIST11.lib
SI:93 Formula:C15H30 CAS:13360-61-7 MolWeight:210 RetIndex:1502
CompName:1-Pentadecene $$ Pentadecene,1- $$ Pentadec-1-ene $$

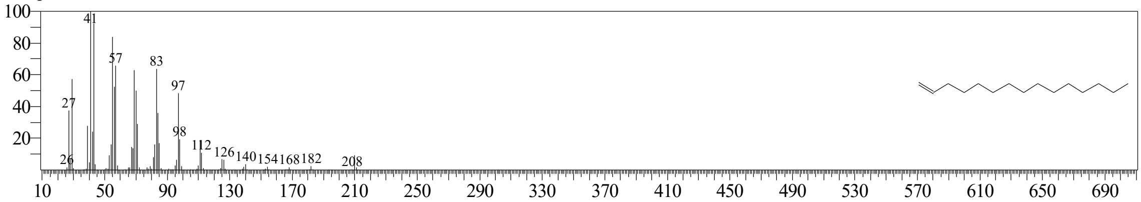

Hit#:3 Entry:81317 Library:NIST11.lib
SI:93 Formula:C17H32O CAS:0-00-0 MolWeight:252 RetIndex:1907
CompName:E-15-Heptadecenal $$ (15E)-15-Heptadecenal # $$

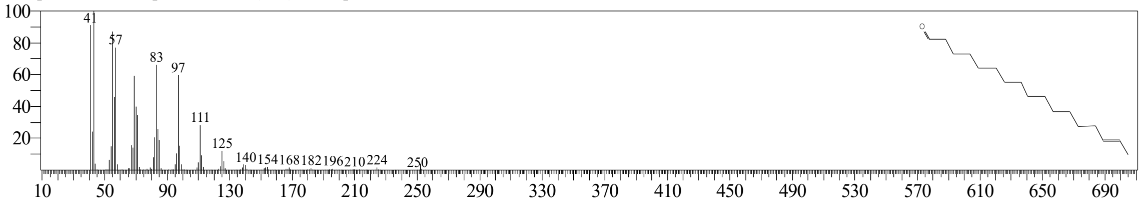

Hit#:4 Entry:70890 Library:NIST11.lib
SI:93 Formula:C16H30O CAS:330207-53-9 MolWeight:238 RetIndex:1808
CompName:E-14-Hexadecenal $$ (14E)-14-Hexadecenal $$ 14-Hexadecenal, (E)- $$

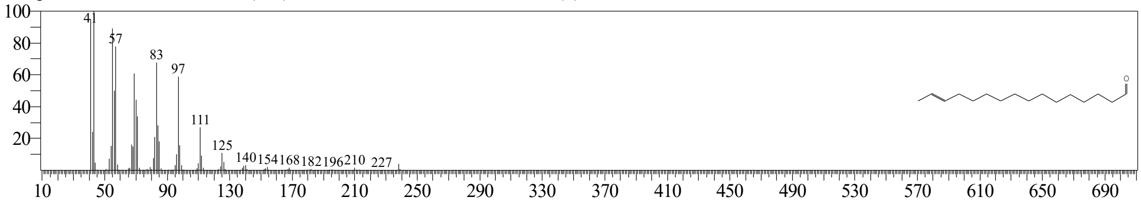

Hit#:5 Entry:102910 Library:NIST11.lib
SI:93 Formula:C20H40 CAS:296-56-0 MolWeight:280 RetIndex:2398
CompName:Cycloeicosane $$ Cycloicosane # $$

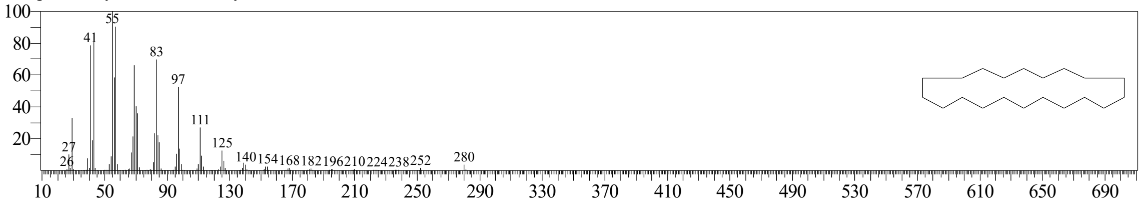

<< Target >>
Line#:42 R.Time:13.160(Scan#:1833) MassPeaks:357
RawMode:Averaged 13.155-13.165(1832-1834) BasePeak:41.05(12586)
BG Mode:Calc. from Peak Group 1 - Event 1 Scan

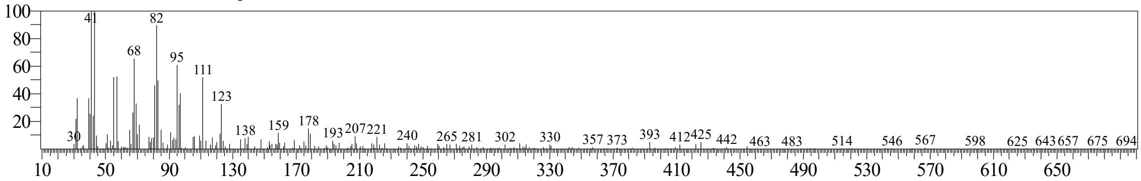

Hit#:1 Entry:163432 Library:NIST11.lib
SI:77 Formula:C26H50 CAS:55334-11-7 MolWeight:362 RetIndex:2653
CompName:1,1'-Bicyclopentyl, 2-hexadecyl- $$ 1-Cyclopentyl-2-n-hexadecylcyclopentane $$

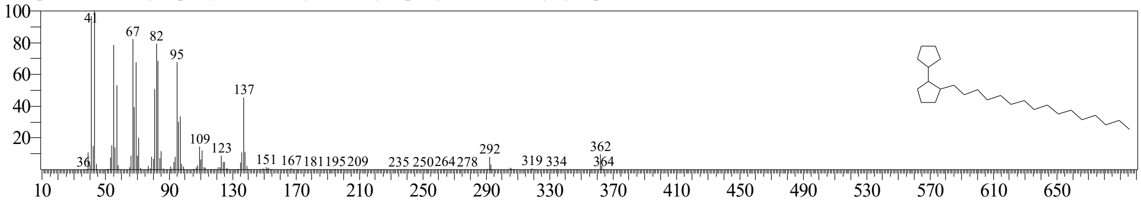

Hit#:2 Entry:115518 Library:NIST11.lib
SI:77 Formula:C20H40O CAS:102608-53-7 MolWeight:296 RetIndex:2045
CompName:3,7,11,15-Tetramethyl-2-hexadecen-1-ol $$ 2-Hexadecen-1-ol, 3,7,11,15-tetramethyl $$

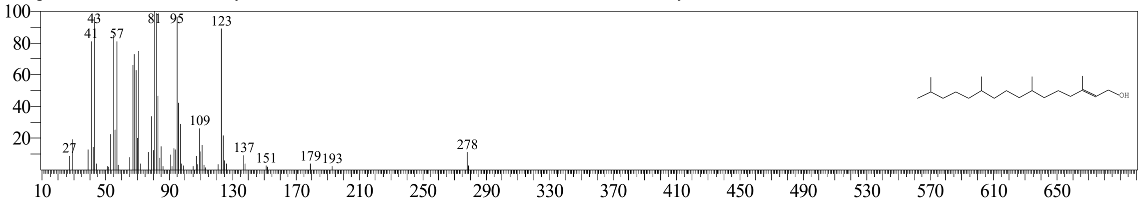

Hit#:3 Entry:62268 Library:NIST11.lib
SI:76 Formula:C14H26O2 CAS:141-16-2 MolWeight:226 RetIndex:1501
CompName:Citronellyl butyrate $$ Butanoic acid, 3,7-dimethyl-6-octenyl ester $$ Butyric acid, 3,7-dimethyl-6-octenyl ester $$ Citronellyl n-butyrate $$ Natura

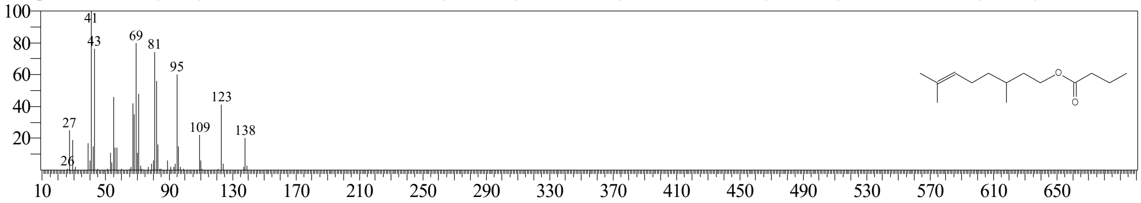

Hit#:4 Entry:79872 Library:NIST11.lib
SI:76 Formula:C18H34 CAS:629-89-0 MolWeight:250 RetIndex:1808
CompName:1-Octadecyne

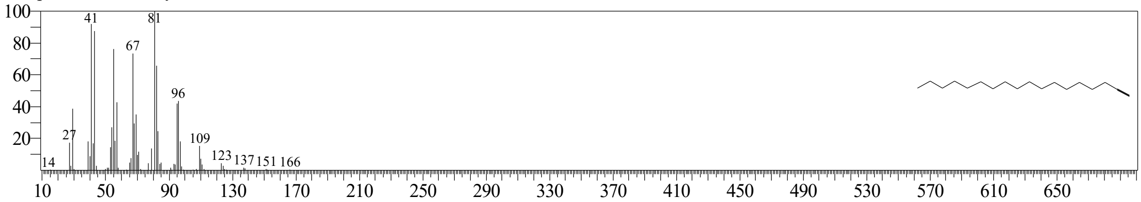

Hit#:5 Entry:59496 Library:NIST11.lib
SI:75 Formula:C16H30 CAS:629-74-3 MolWeight:222 RetIndex:1609
CompName:1-Hexadecyne

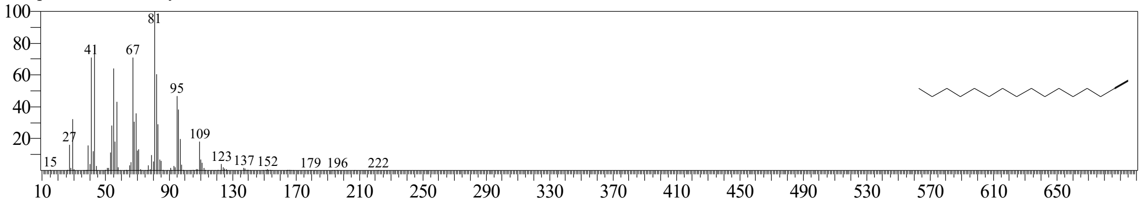

<< Target >>
Line#:43 R.Time:13.315(Scan#:1864) MassPeaks:366
RawMode:Averaged 13.310-13.320(1863-1865) BasePeak:43.10(34896)
BG Mode:Calc. from Peak Group 1 - Event 1 Scan

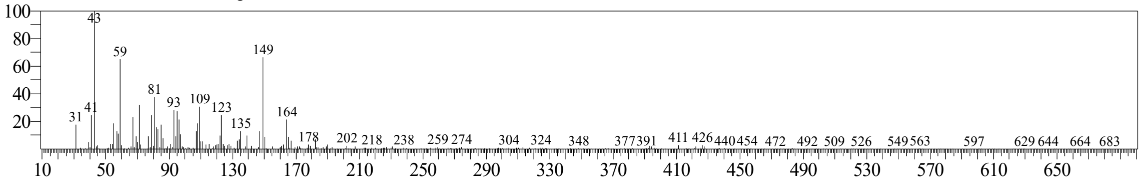

Hit#:1 Entry:59380 Library:NIST11.lib
SI:76 Formula:C15H26O CAS:473-15-4 MolWeight:222 RetIndex:1593
CompName:2-Naphthalenemethanol, decahydro-.alpha.,.alpha.,4a-trimethyl-8-methylene-, [2R-(2.alpha.,4a.alpha.,8a.beta.)]- $$ Eudesm-4(14)-en-11-ol $$ .beta

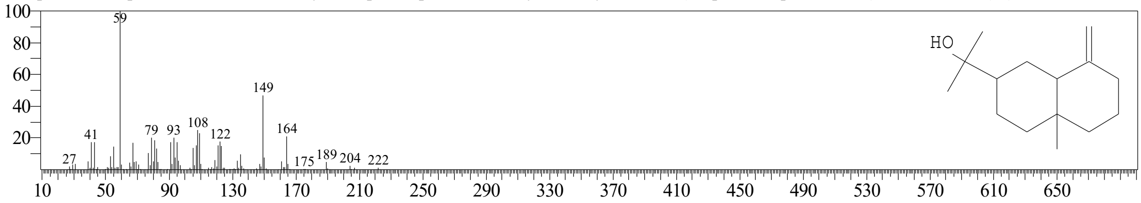

Hit#:2 Entry:60792 Library:NIST11.lib
SI:76 Formula:C14H24O2 CAS:72294-84-9 MolWeight:224 RetIndex:1497
CompName:3-Buten-2-ol, 2-methyl-4-(1,3,3-trimethyl-7-oxabicyclo[4.1.0]hept-2-yl)- $$ (3E)-2-Methyl-4-(1,3,3-trimethyl-7-oxabicyclo[4.1.0]hept-2-yl)-3-bute

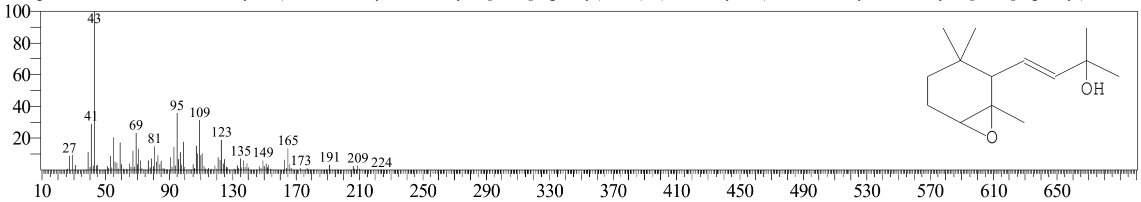

Hit#:3 Entry:60908 Library:NIST11.lib
SI:74 Formula:C15H28O CAS:69239-72-1 MolWeight:224 RetIndex:1770
CompName:5,9-Dimethyl-2-(1-methylethylidene)-1-cyclodecanol $$ 5,9-Dimethyl-2-(1-methylethylidene)cyclodecanol # $$

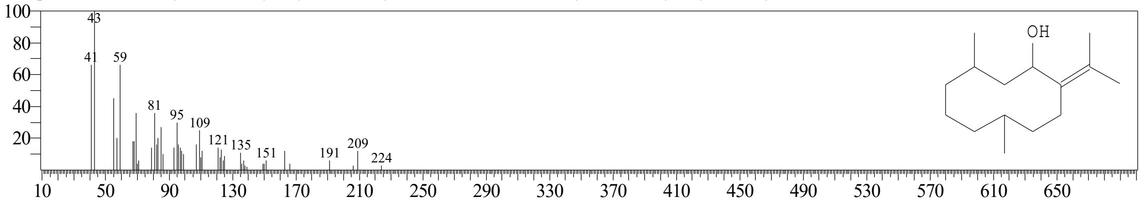

Hit#:4 Entry:59379 Library:NIST11.lib
SI:74 Formula:C15H26O CAS:63891-61-2 MolWeight:222 RetIndex:1598
CompName:2-Naphthalenemethanol, 2,3,4,4a,5,6,7,8-octahydro-.alpha.,.alpha.,4a,8-tetramethyl-, [2R-(2.alpha.,4a.beta.,8.beta.)]- $$ 2-(4a,8-Dimethyl-2,3,4,4a,

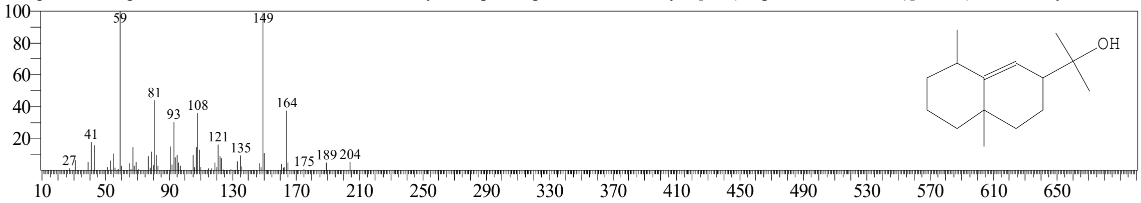

Hit#:5 Entry:72318 Library:NIST11.lib
SI:73 Formula:C15H28O2 CAS:60714-01-4 MolWeight:240 RetIndex:1766
CompName:2-Methyl-5-(2,6,6-trimethyl-cyclohex-1-enyl)-pentane-2,3-diol $$ 2-Methyl-5-(2,6,6-trimethyl-1-cyclohexen-1-yl)-2,3-pentanediol # $$

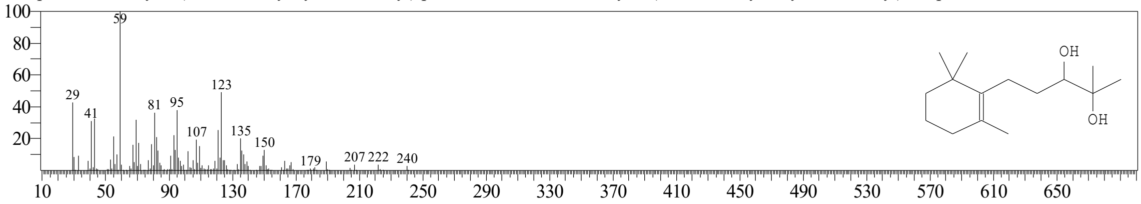

<< Target >>
Line#:44 R.Time:13.440(Scan#:1889) MassPeaks:389
RawMode:Averaged 13.435-13.445(1888-1890) BasePeak:41.10(14926)
BG Mode:Calc. from Peak Group 1 - Event 1 Scan

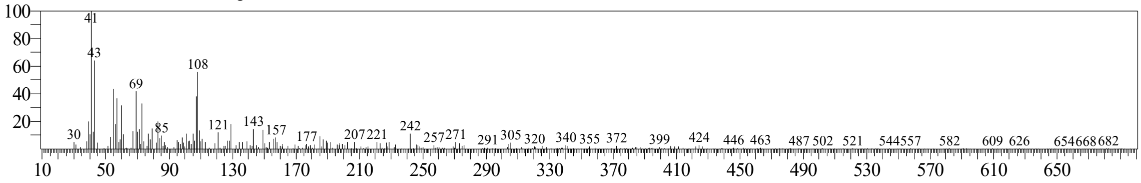

Hit#:1 Entry:89750 Library:NIST11.lib
SI:68 Formula:C11H21BrO2 CAS:18294-93-4 MolWeight:264 RetIndex:1703
CompName:Undecanoic acid, 10-bromo- $$ 10-Bromoundecanoic acid # $$

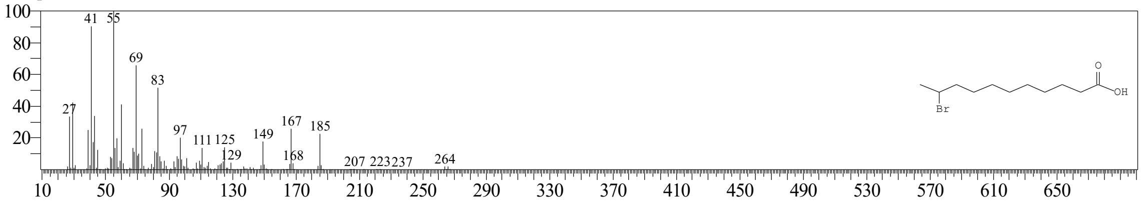

Hit#:2 Entry:73851 Library:NIST11.lib
SI:68 Formula:C15H30O2 CAS:1002-84-2 MolWeight:242 RetIndex:1869
CompName:Pentadecanoic acid $$ Pentadecylic acid $$ n-Pentadecanoic acid $$ n-Pentadecylic acid $$

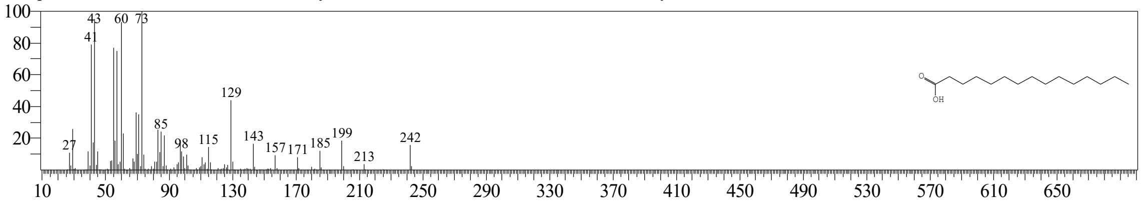

Hit#:3 Entry:102327 Library:NIST11.lib
SI:68 Formula:C14H16O2S2 CAS:0-00-0 MolWeight:280 RetIndex:0
CompName:3-Methyl-4-(phenylthio)-2-prop-2-enyl-2,5-dihydrothiophene 1,1-dioxide

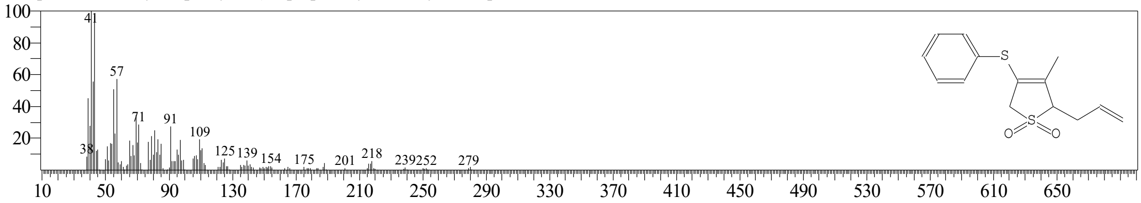

Hit#:4 Entry:24745 Library:NIST11.lib
SI:67 Formula:C11H20O CAS:53448-07-0 MolWeight:168 RetIndex:1311
CompName:2-Undecenal, E- $$ (2E)-2-Undecenal $$ 2(E)-Undecenal $$ 2-Undecenal, (E)- $$ 2-Undecenal, trans $$ (E)-2-Undecenal $$ (E)-Undec-2-enal $$ tr

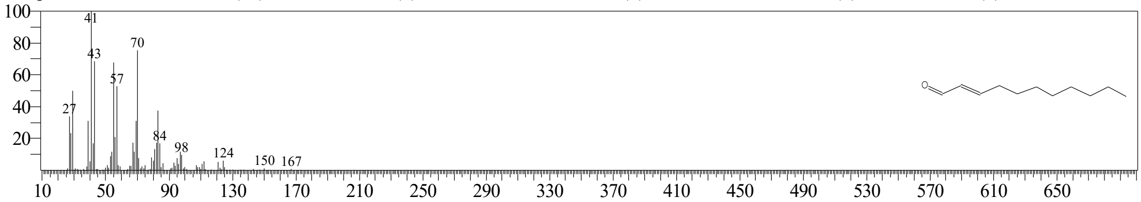

Hit#:5 Entry:84362 Library:NIST11.lib
SI:67 Formula:C16H32O2 CAS:57-10-3 MolWeight:256 RetIndex:1968
CompName:n-Hexadecanoic acid $$ Hexadecanoic acid $$ n-Hexadecoic acid $$ Palmitic acid $$ Pentadecanecarboxylic acid $$ 1-Pentadecanecarboxylic acid

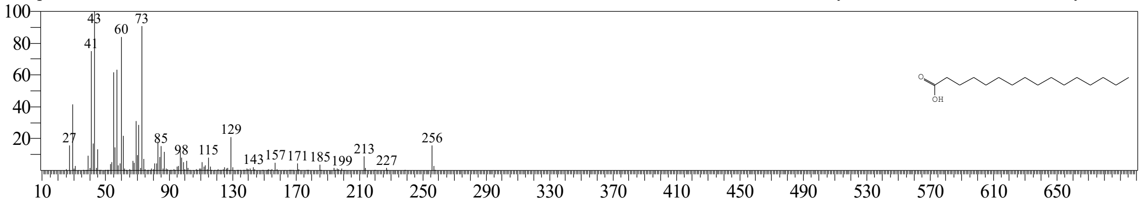

<< Target >>
Line#:45 R.Time:13.600(Scan#:1921) MassPeaks:344
RawMode:Averaged 13.595-13.605(1920-1922) BasePeak:41.05(9533)
BG Mode:Calc. from Peak Group 1 - Event 1 Scan

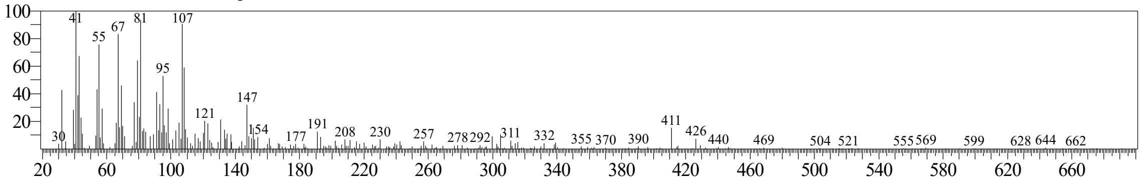

Hit#:1 Entry:156918 Library:NIST11.lib
SI:77 Formula:C21H36O4 CAS:18465-99-1 MolWeight:352 RetIndex:2705
CompName:9,12,15-Octadecatrienoic acid, 2,3-dihydroxypropyl ester, (Z,Z,Z)- $$ Linolenin, 1-mono- $$ .alpha.-Glyceryl linolenate $$ 2,3-Dihydroxypropyl (1

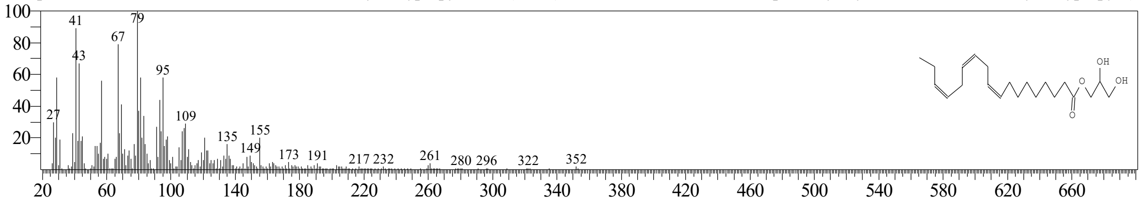

Hit#:2 Entry:69416 Library:NIST11.lib
SI:77 Formula:C16H28O CAS:56829-23-3 MolWeight:236 RetIndex:1816
CompName:cis,cis-7,10,-Hexadecadienal $$ (7Z,10Z)-7,10-Hexadecadienal # $$

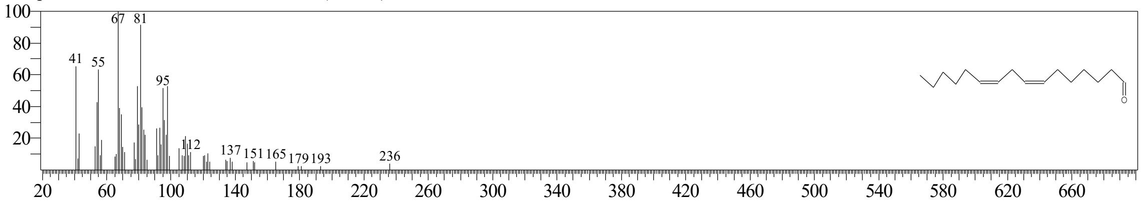

Hit#:3 Entry:67943 Library:NIST11.lib
SI:77 Formula:C16H26O CAS:56797-43-4 MolWeight:234 RetIndex:1824
CompName:cis,cis,cis-7,10,13-Hexadecatrienal $$ (7Z,10Z,13Z)-7,10,13-Hexadecatrienal # $$

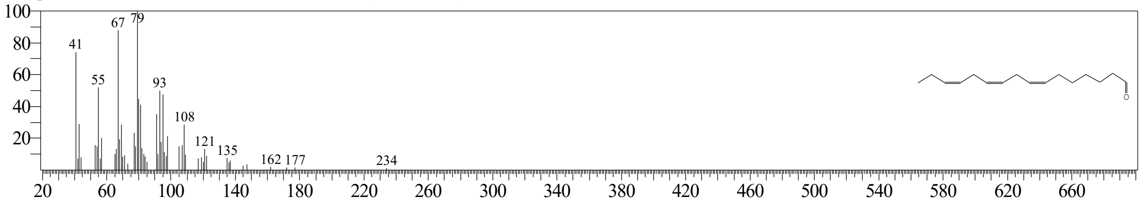

Hit#:4 Entry:156919 Library:NIST11.lib
SI:76 Formula:C21H36O4 CAS:55268-58-1 MolWeight:352 RetIndex:2721
CompName:Linolenic acid, 2-hydroxy-1-(hydroxymethyl)ethyl ester (Z,Z,Z)- $$ 2-Hydroxy-1-(hydroxymethyl)ethyl (9E,12E,15E)-9,12,15-octadecatrienoate # $

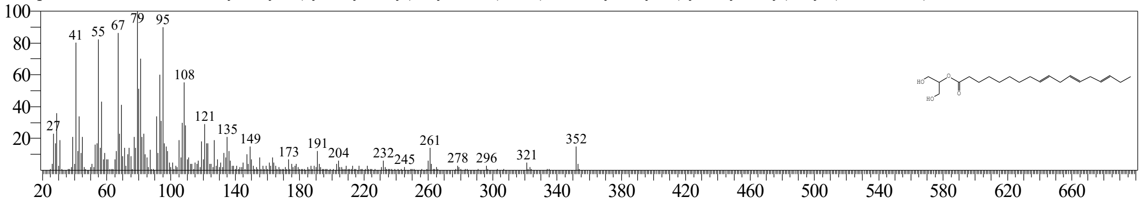

Hit#:5 Entry:144988 Library:NIST11.lib
SI:76 Formula:C23H42O CAS:0-00-0 MolWeight:334 RetIndex:2539
CompName:1-Heptadec-1-ynyl-cyclohexanol $$ 1-(1-Heptadecynyl)cyclohexanol # $$

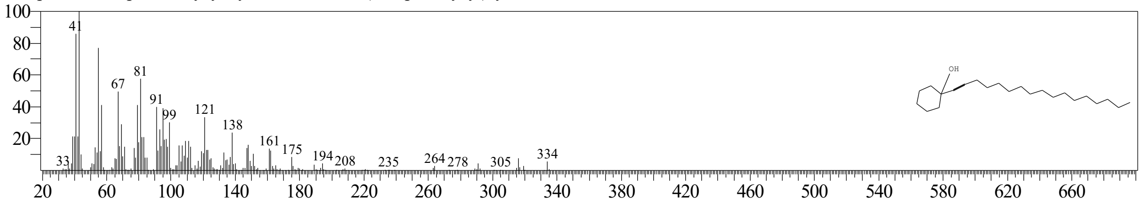

<< Target >>
Line#:46 R.Time:13.645(Scan#:1930) MassPeaks:382
RawMode:Averaged 13.640-13.650(1929-1931) BasePeak:79.10(48701)
BG Mode:Calc. from Peak Group 1 - Event 1 Scan

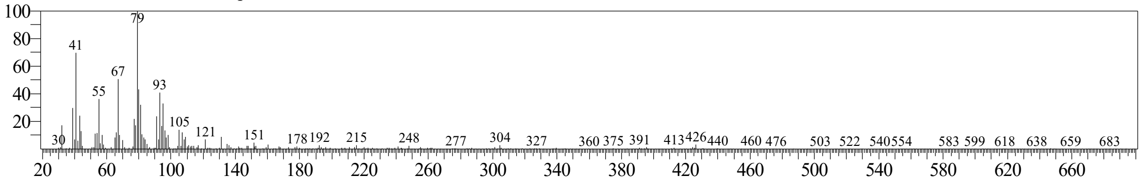

Hit#:1 Entry:89012 Library:NIST11.lib
SI:85 Formula:C18H30O CAS:26537-71-3 MolWeight:262 RetIndex:2023
CompName:9,12,15-Octadecatrienal $$ (9E,12E,15E)-9,12,15-Octadecatrienal # $$

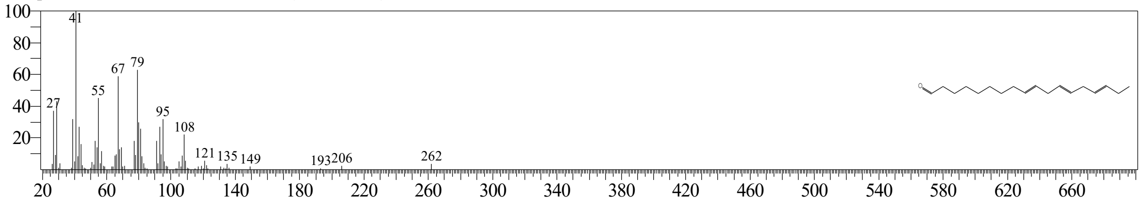

Hit#:2 Entry:90503 Library:NIST11.lib
SI:85 Formula:C18H32O CAS:506-44-5 MolWeight:264 RetIndex:2077
CompName:9,12,15-Octadecatrien-1-ol, (Z,Z,Z)- $$ (9E,12E,15E)-9,12,15-Octadecatrien-1-ol # $$ (Z,Z,Z)-9,12,15-Octadecatrien-1-ol $$

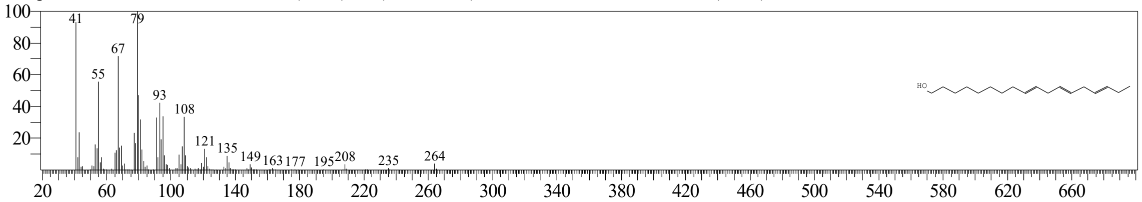

Hit#:3 Entry:67943 Library:NIST11.lib
SI:85 Formula:C16H26O CAS:56797-43-4 MolWeight:234 RetIndex:1824
CompName:cis,cis,cis-7,10,13-Hexadecatrienal $$ (7Z,10Z,13Z)-7,10,13-Hexadecatrienal # $$

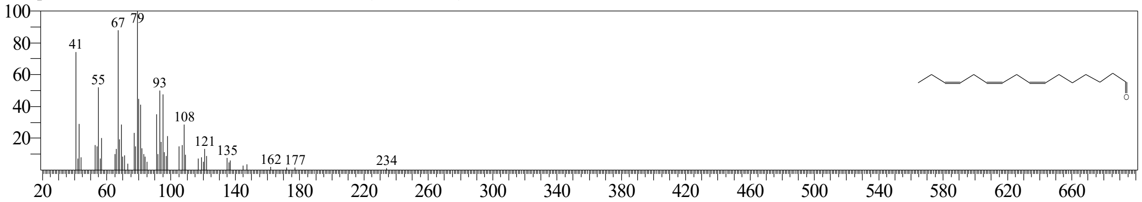

Hit#:4 Entry:38810 Library:NIST11.lib
SI:84 Formula:C14H24 CAS:74744-44-8 MolWeight:192 RetIndex:1438
CompName:3-Tetradecen-5-yne, (E)- $$ (3E)-3-Tetradecen-5-yne # $$

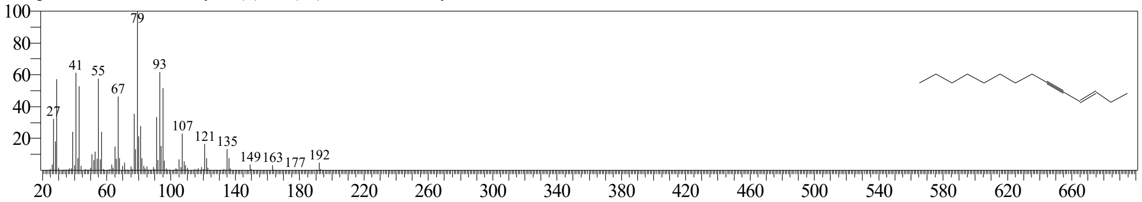

Hit#:5 Entry:38809 Library:NIST11.lib
SI:84 Formula:C14H24 CAS:74663-68-6 MolWeight:192 RetIndex:1438
CompName:3-Tetradecen-5-yne, (Z)- $$ (3Z)-3-Tetradecen-5-yne # $$

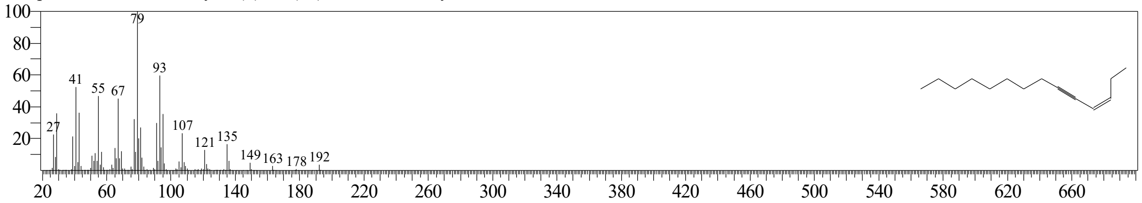

<< Target >>
Line#:47 R.Time:13.790(Scan#:1959) MassPeaks:430
RawMode:Averaged 13.785-13.795(1958-1960) BasePeak:74.05(300545)
BG Mode:Calc. from Peak Group 1 - Event 1 Scan

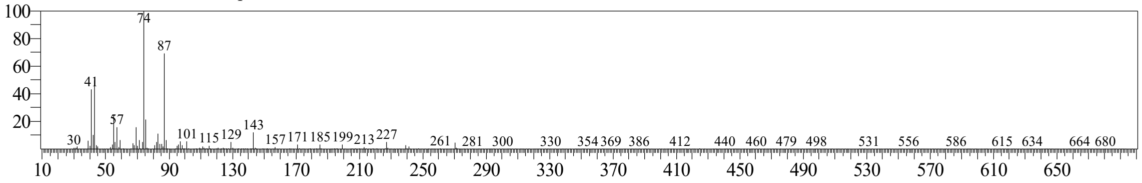

Hit#:1 Entry:95189 Library:NIST11.lib
SI:94 Formula:C17H34O2 CAS:5129-60-2 MolWeight:270 RetIndex:1814
CompName:Pentadecanoic acid, 14-methyl-, methyl ester $$ Methyl 14-methylpentadecanoate # $$ Methyl isohexadecanoate $$

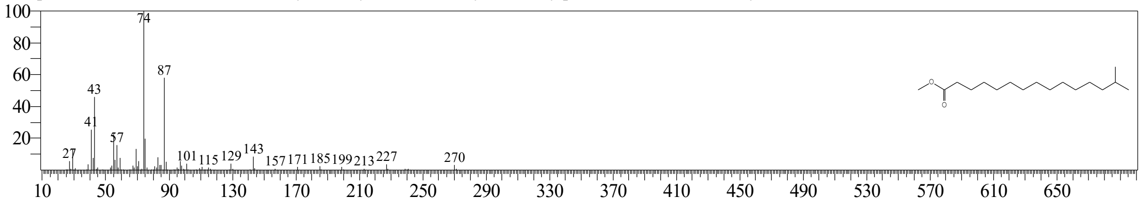

Hit#:2 Entry:106180 Library:NIST11.lib
SI:93 Formula:C18H36O2 CAS:6929-04-0 MolWeight:284 RetIndex:1914
CompName:Hexadecanoic acid, 15-methyl-, methyl ester $$ Methyl isoheptadecanoate $$ Methyl 15-methylhexadecanoate $$

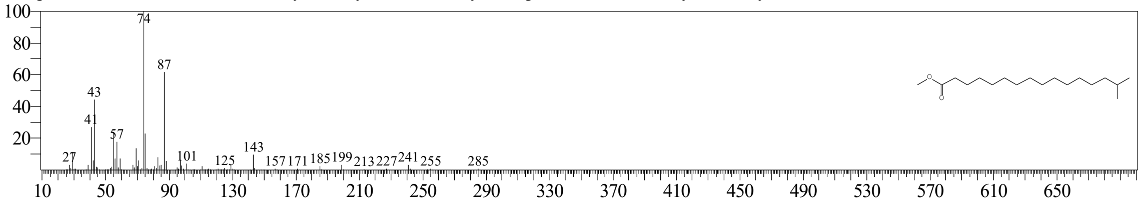

Hit#:3 Entry:95188 Library:NIST11.lib
SI:91 Formula:C17H34O2 CAS:112-39-0 MolWeight:270 RetIndex:1878
CompName:Hexadecanoic acid, methyl ester $$ Palmitic acid, methyl ester $$ n-Hexadecanoic acid methyl ester $$ Metholene 2216 $$ Methyl hexadecanoate $

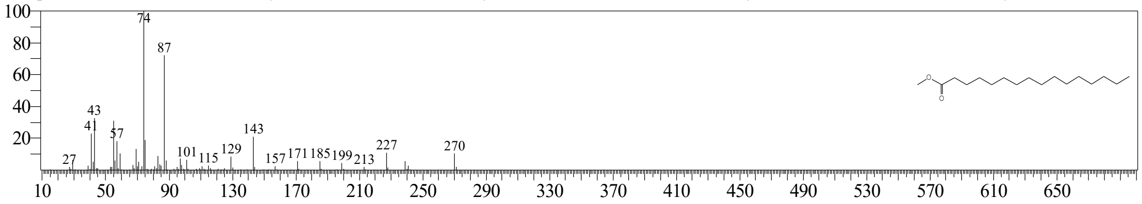

Hit#:4 Entry:73852 Library:NIST11.lib
SI:91 Formula:C15H30O2 CAS:5129-58-8 MolWeight:242 RetIndex:1615
CompName:Tridecanoic acid, 12-methyl-, methyl ester $$ Methyl isomyristate $$ Methyl 12-methyltridecanoate $$

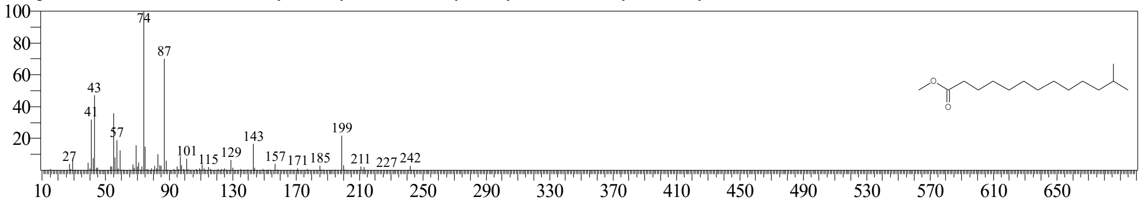

Hit#:5 Entry:84388 Library:NIST11.lib
SI:90 Formula:C16H32O2 CAS:7132-64-1 MolWeight:256 RetIndex:1779
CompName:Pentadecanoic acid, methyl ester $$ Methyl n-pentadecanoate $$ Methyl pentadecanoate $$ n-Pentadecanoic acid methyl ester $$

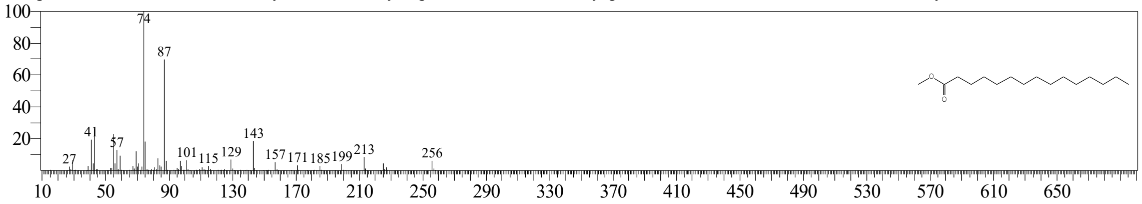

<< Target >>
Line#:48 R.Time:13.980(Scan#:1997) MassPeaks:393
RawMode:Averaged 13.975-13.985(1996-1998) BasePeak:41.05(177648)
BG Mode:Calc. from Peak Group 1 - Event 1 Scan

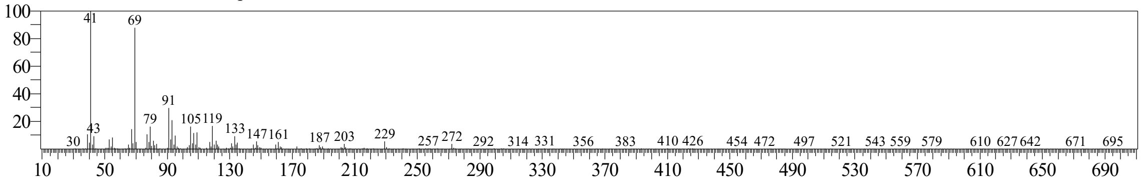

Hit#:1 Entry:46578 Library:NIST11.lib
SI:84 Formula:C15H24 CAS:77129-48-7 MolWeight:204 RetIndex:1440
CompName:1,6,10-Dodecatriene, 7,11-dimethyl-3-methylene- $$ 7,11-Dimethyl-3-methylene-1,6,10-dodecatriene $$

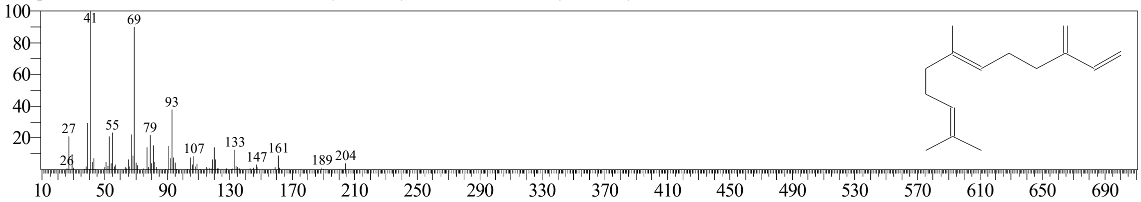

Hit#:2 Entry:96991 Library:NIST11.lib
SI:84 Formula:C20H32 CAS:38259-79-9 MolWeight:272 RetIndex:1958
CompName:2,6,11,15-Tetramethyl-hexadeca-2,6,8,10,14-pentaene $$ (6E,8E,10E)-2,6,11,15-Tetramethyl-2,6,8,10,14-hexadecapentaene # $$

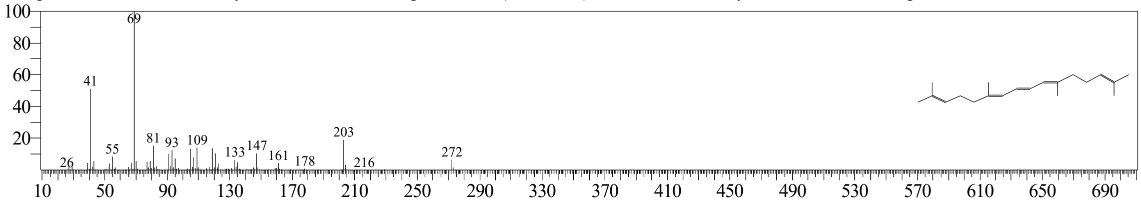

Hit#:3 Entry:110903 Library:NIST11.lib
SI:83 Formula:C20H34O CAS:1113-21-9 MolWeight:290 RetIndex:2046
CompName:1,6,10,14-Hexadecatetraen-3-ol, 3,7,11,15-tetramethyl-, (E,E)- $$ (6E,10E)-3,7,11,15-Tetramethyl-1,6,10,14-hexadecatetraen-3-ol # $$ Geranyl lina

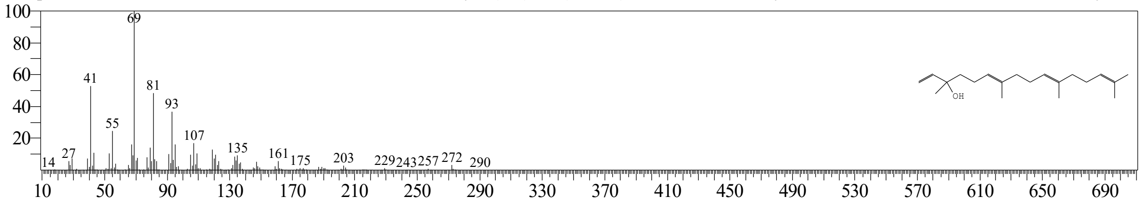

Hit#:4 Entry:57745 Library:NIST11.lib
SI:83 Formula:C15H24O CAS:2387-68-0 MolWeight:220 RetIndex:1572
CompName:6,10-Dodecadien-1-yn-3-ol, 3,7,11-trimethyl- $$ Deca-4,8-dienol, 1-ethynyl-1,5,9-trimethyl- $$ (6E)-3,7,11-Trimethyl-6,10-dodecadien-1-yn-3-ol #

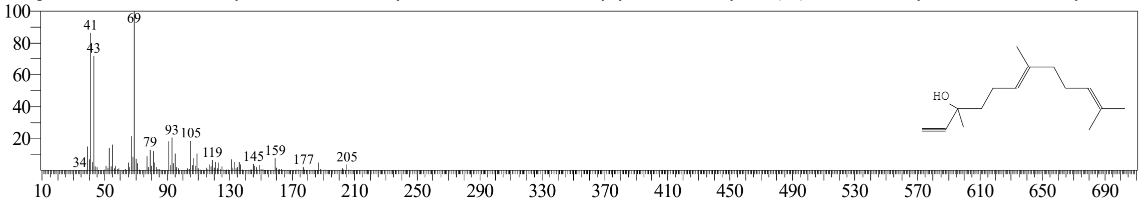

Hit#:5 Entry:96989 Library:NIST11.lib
SI:83 Formula:C20H32 CAS:77898-97-6 MolWeight:272 RetIndex:1940
CompName:(E,E,E)-3,7,11,15-Tetramethylhexadeca-1,3,6,10,14-pentaene $$ (3E,6E,10E)-3,7,11,15-Tetramethyl-1,3,6,10,14-hexadecapentaene $$ (E,E,E)- .alp

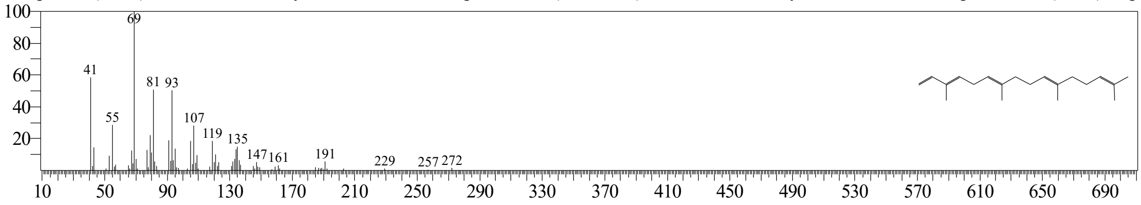

<< Target >>
Line#:49 R.Time:14.105(Scan#:2022) MassPeaks:420
RawMode:Averaged 14.100-14.110(2021-2023) BasePeak:43.05(339916)
BG Mode:Calc. from Peak Group 1 - Event 1 Scan

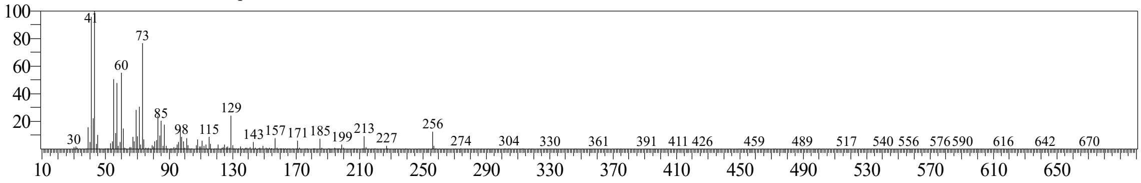

Hit#:1 Entry:84362 Library:NIST11.lib
SI:93 Formula:C16H32O2 CAS:57-10-3 MolWeight:256 RetIndex:1968
CompName:n-Hexadecanoic acid $$ Hexadecanoic acid $$ n-Hexadecoic acid $$ Palmitic acid $$ Pentadecanecarboxylic acid $$ 1-Pentadecanecarboxylic acid

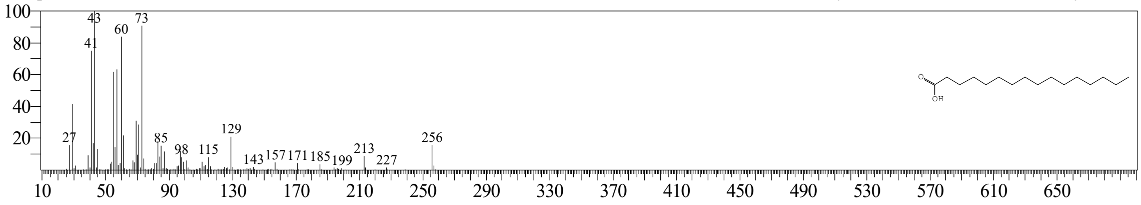

Hit#:2 Entry:128151 Library:NIST11.lib
SI:90 Formula:C20H40O2 CAS:506-30-9 MolWeight:312 RetIndex:2366
CompName:Eicosanoic acid $$ Arachic acid $$ Arachidic acid $$ Icosanoic acid $$ n-Eicosanoic acid $$ Arachidic acid (synthetic) $$

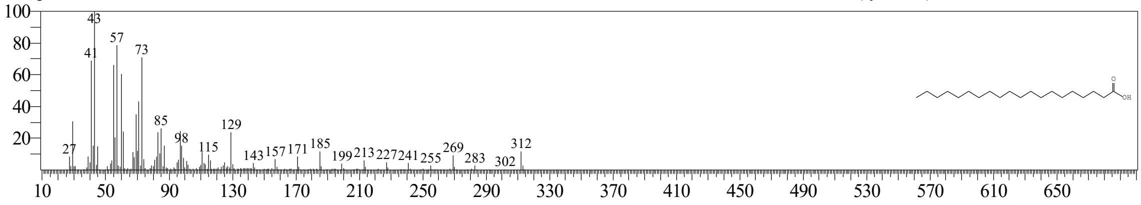

Hit#:3 Entry:73851 Library:NIST11.lib
SI:89 Formula:C15H30O2 CAS:1002-84-2 MolWeight:242 RetIndex:1869
CompName:Pentadecanoic acid $$ Pentadecylic acid $$ n-Pentadecanoic acid $$ n-Pentadecylic acid $$

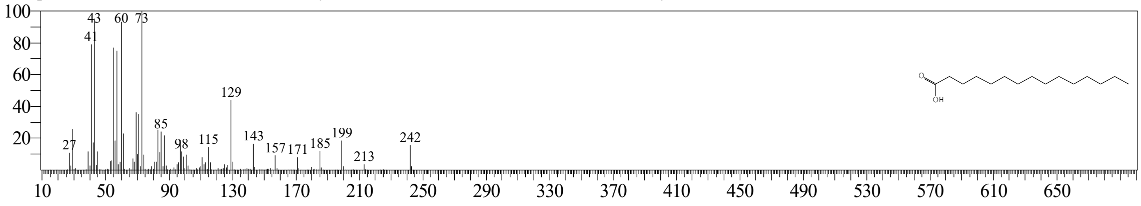

Hit#:4 Entry:53599 Library:NIST11.lib
SI:89 Formula:C13H26O2 CAS:638-53-9 MolWeight:214 RetIndex:1670
CompName:Tridecanoic acid $$ n-Tridecanoic acid $$ n-Tridecoic acid $$ Tridecylic acid $$

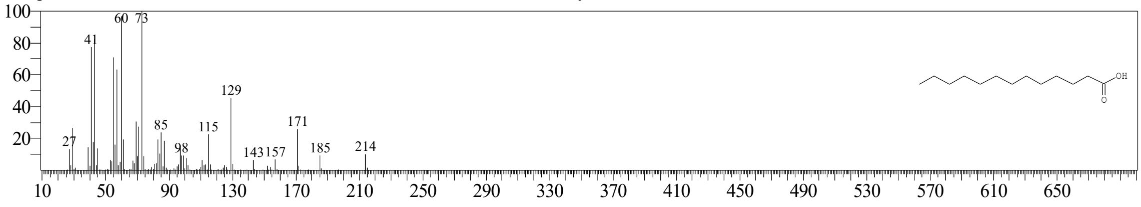

Hit#:5 Entry:117133 Library:NIST11.lib
SI:89 Formula:C19H38O2 CAS:646-30-0 MolWeight:298 RetIndex:2266
CompName:Nonadecanoic acid $$ n-Nonadecanoic acid $$

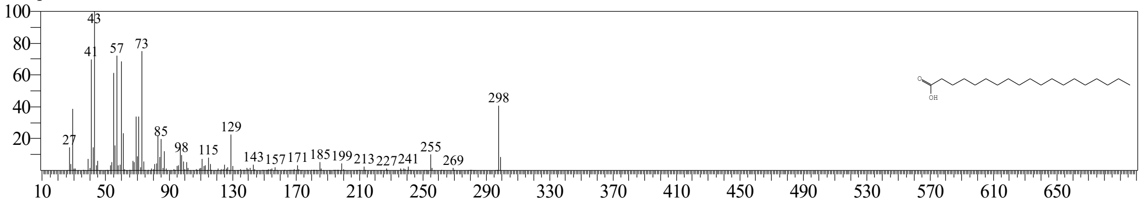

Qualitative Analysis Report

<< Target >>
Line#:50 R.Time:14.230(Scan#:2047) MassPeaks:434
RawMode:Averaged 14.225-14.235(2046-2048) BasePeak:41.10(178371)
BG Mode:Calc. from Peak Group 1 - Event 1 Scan

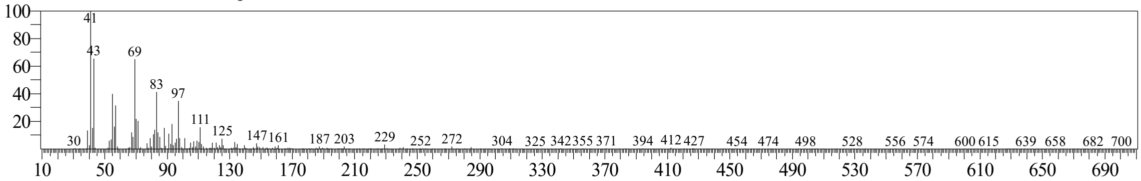

Hit#:1 Entry:41639 Library:NIST11.lib
SI:85 Formula:C14H28 CAS:41446-68-8 MolWeight:196 RetIndex:1421
CompName:3-Tetradecene, (E)- $$ (3E)-3-Tetradecene $$ (E)-3-Tetradecene $$ trans-3-Tetradecene $$

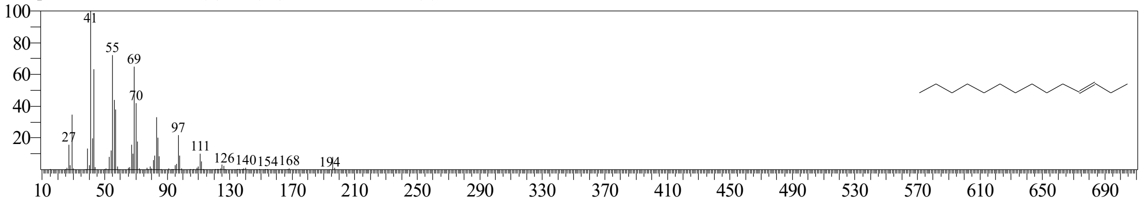

Hit#:2 Entry:113654 Library:NIST11.lib
SI:84 Formula:C16H32Cl2 CAS:7735-39-9 MolWeight:294 RetIndex:2062
CompName:Hexadecane, 1,16-dichloro- $$ 1,16-Dichlorohexadecane # $$

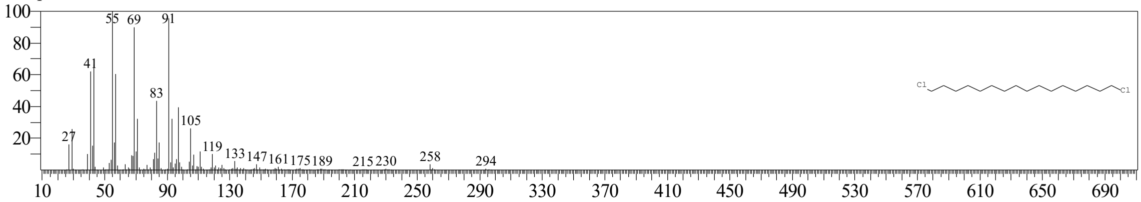

Hit#:3 Entry:72461 Library:NIST11.lib
SI:83 Formula:C16H32O CAS:0-00-0 MolWeight:240 RetIndex:1862
CompName:E-2-Hexadecacen-1-ol $$ (2E)-2-Hexadecen-1-ol # $$

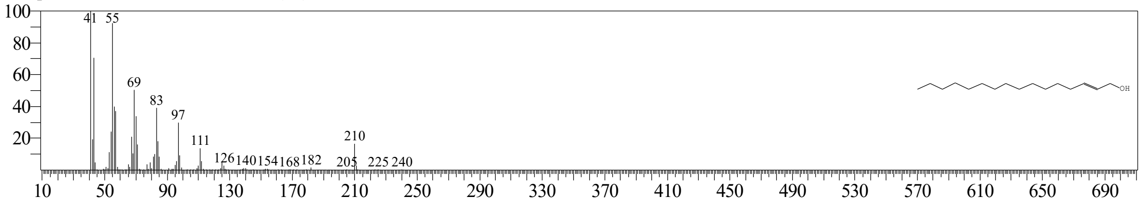

Hit#:4 Entry:45176 Library:NIST11.lib
SI:83 Formula:C12H26S CAS:10059-13-9 MolWeight:202 RetIndex:1433
CompName:2-Undecanethiol, 2-methyl- $$ 1,1-Dimethyl-decyl-mercaptan $$ 2-Methylundecyl-2-thiol $$ 1,1-Dimethyldecyl hydrosulfide # $$ 2-Methyl-2-und

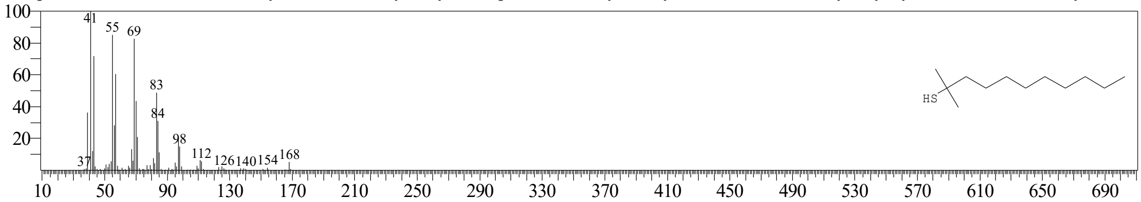

Hit#:5 Entry:41641 Library:NIST11.lib
SI:83 Formula:C14H28 CAS:41446-78-0 MolWeight:196 RetIndex:1421
CompName:4-Tetradecene, (E)- $$ trans-4-Tetradecene $$ (4E)-4-Tetradecene $$ (E)-4-Tetradecene $$

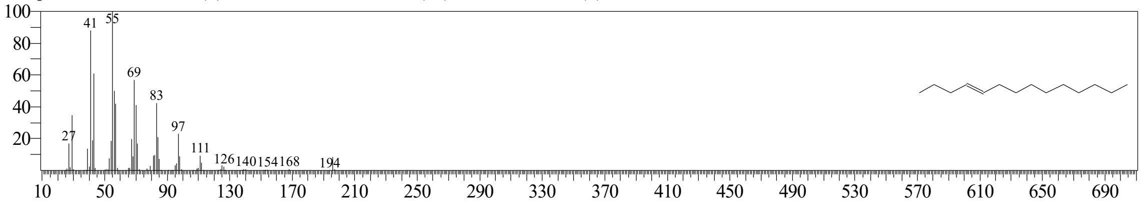

<< Target >>
Line#:51 R.Time:14.475(Scan#:2096) MassPeaks:372
RawMode:Averaged 14.470-14.480(2095-2097) BasePeak:108.05(15006)
BG Mode:Calc. from Peak Group 1 - Event 1 Scan

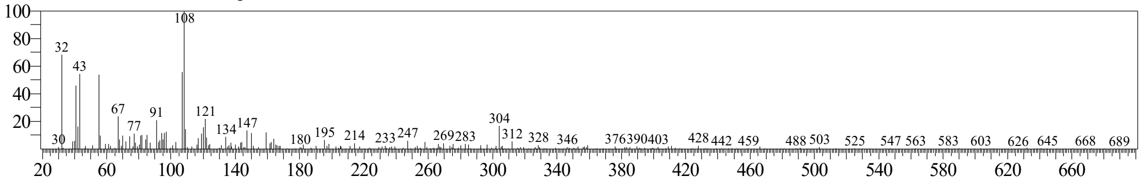

Hit#:1 Entry:15539 Library:NIST11.lib
SI:63 Formula:C11H18 CAS:115948-98-6 MolWeight:150 RetIndex:1027
CompName:2,2-Dimethyl-3-vinyl-bicyclo[2.2.1]heptane

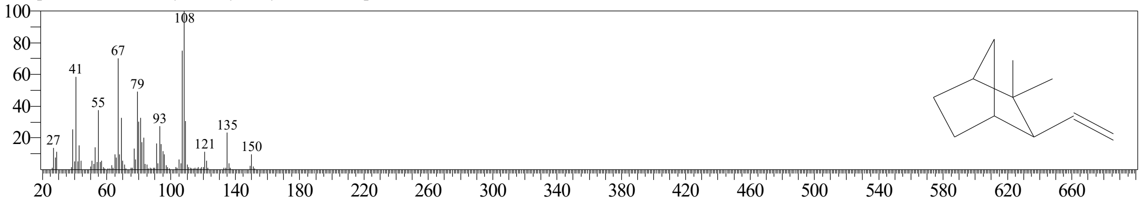

Hit#:2 Entry:143347 Library:NIST11.lib
SI:63 Formula:C22H36O2 CAS:54550-12-8 MolWeight:332 RetIndex:2170
CompName:Androstan-3-ol, 9-methyl-, acetate, (3.beta.,5.alpha.)- $$ 9-Methylandrostan-3-yl acetate # $$

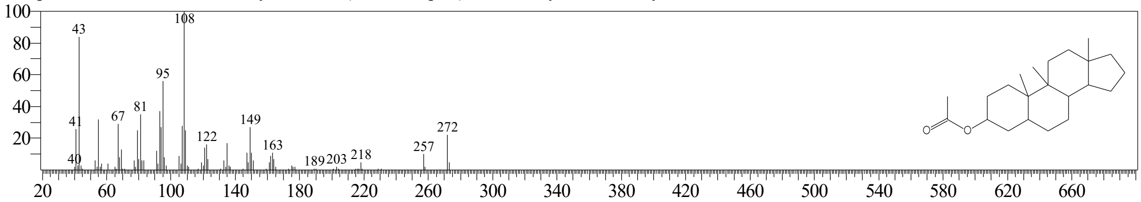

Hit#:3 Entry:123533 Library:NIST11.lib
SI:63 Formula:C20H34O2 CAS:0-00-0 MolWeight:306 RetIndex:2179
CompName:11-(3-Ethenylcyclopentyl)undec-10-enoic acid, ethyl ester $$ Ethyl (10Z)-11-(3-vinylcyclopentyl)-10-undecenoate # $$

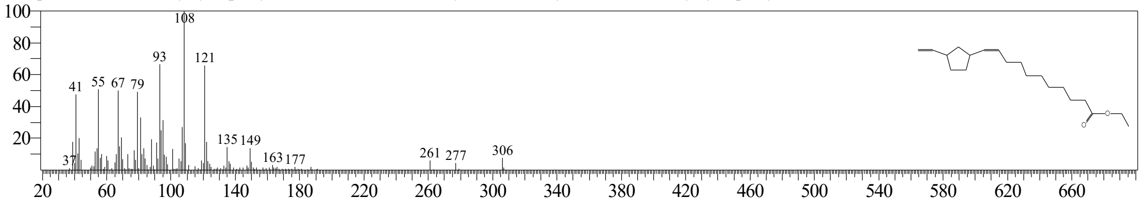

Hit#:4 Entry:46672 Library:NIST11.lib
SI:63 Formula:C15H24 CAS:0-00-0 MolWeight:204 RetIndex:1450
CompName:rotundene

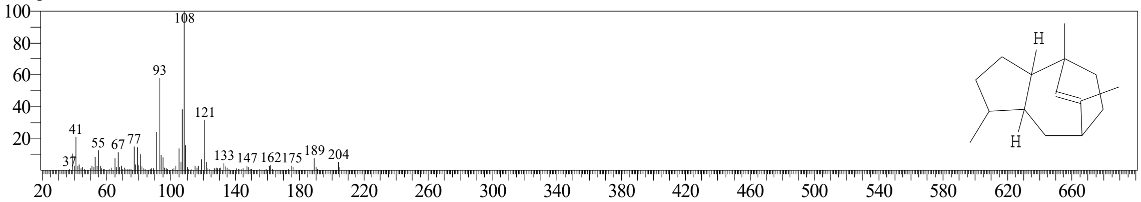

Hit#:5 Entry:135718 Library:NIST11.lib
SI:62 Formula:C21H38O2 CAS:0-00-0 MolWeight:322 RetIndex:2292
CompName:Z,Z-4,6-Nonadecadien-1-ol acetate $$ (4Z,6Z)-4,6-Nonadecadienyl acetate # $$

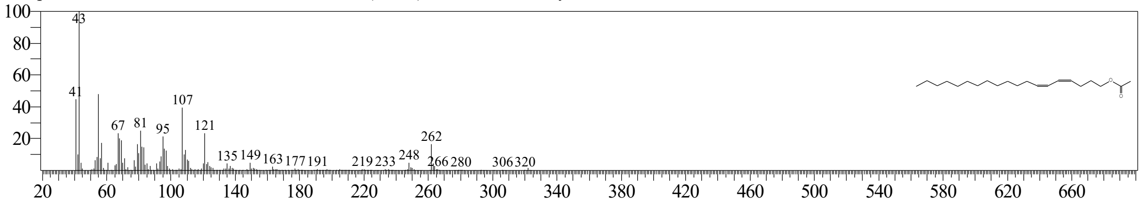

<< Target >>
Line#:52 R.Time:14.645(Scan#:2130) MassPeaks:355
RawMode:Averaged 14.640-14.650(2129-2131) BasePeak:41.05(6212)
BG Mode:Calc. from Peak Group 1 - Event 1 Scan

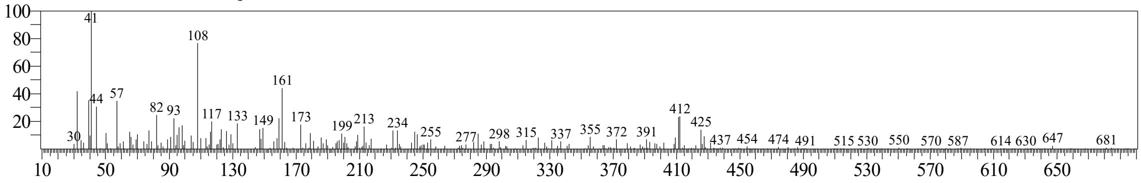

Hit#:1 Entry:78716 Library:NIST11.lib
SI:44 Formula:C14H19NO3 CAS:313957-60-7 MolWeight:249 RetIndex:1962
CompName:Oxazolidin-2-one, 3-tert-butyl-5-phenoxymethyl- $$ 3-tert-Butyl-5-(phenoxymethyl)-1,3-oxazolidin-2-one # $$

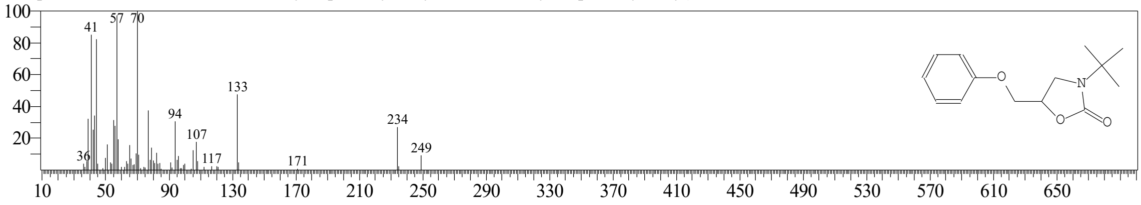

Hit#:2 Entry:25772 Library:NIST11.lib
SI:43 Formula:C10H18O2 CAS:10359-41-8 MolWeight:170 RetIndex:1326
CompName:Bicyclo[2.2.1]heptane-2,5-diol, 1,7,7-trimethyl-, (2-endo,5-exo)- $$ 1,7,7-Trimethylbicyclo[2.2.1]heptane-2,5-diol # $$

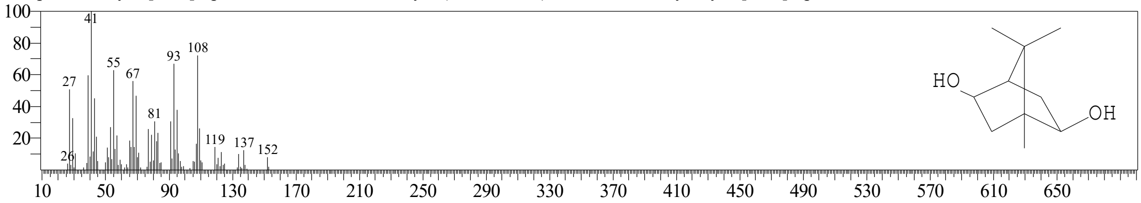

Hit#:3 Entry:54954 Library:NIST11.lib
SI:42 Formula:C15H20O CAS:17910-09-7 MolWeight:216 RetIndex:1532
CompName:Benzofuran, 6-ethenyl-4,5,6,7-tetrahydro-3,6-dimethyl-5-isopropenyl-, trans- $$ 5-Isopropenyl-3,6-dimethyl-6-vinyl-4,5,6,7-tetrahydro-1-benzofura

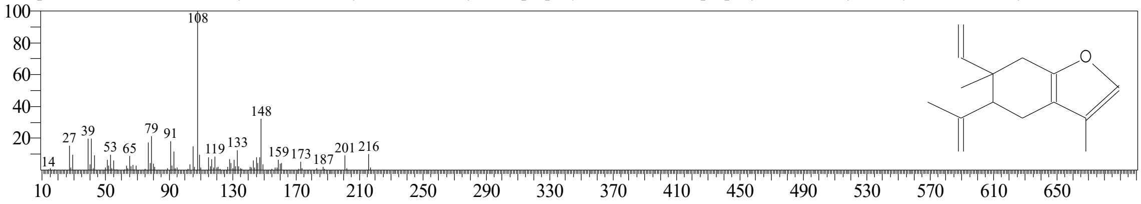

Hit#:4 Entry:39680 Library:NIST11.lib
SI:42 Formula:C10H10O4 CAS:14447-07-5 MolWeight:194 RetIndex:1347
CompName:2-Butynedioic acid, di-2-propenyl ester $$ Acetylenedicarboxylic acid, diallyl ester $$ Diallyl 2-butynedioate # $$

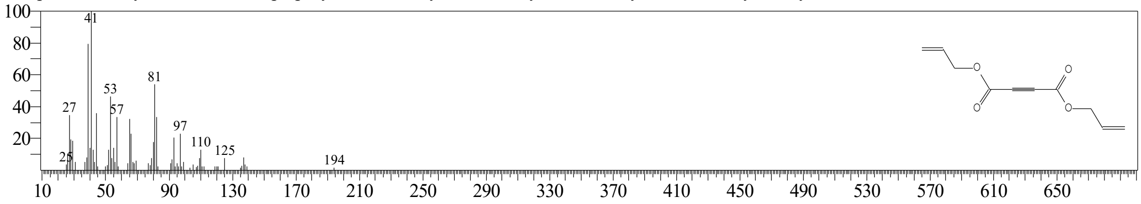

Hit#:5 Entry:57288 Library:NIST11.lib
SI:42 Formula:C11H16N4O CAS:0-00-0 MolWeight:220 RetIndex:2228
CompName:1H-[1,2,4]Triazole-3-carboxylic acid (2-cyclohex-1-enyl-ethyl)-amide $$ N-[2-(1-Cyclohexen-1-yl)ethyl]-1H-1,2,4-triazole-3-carboxamide # $$

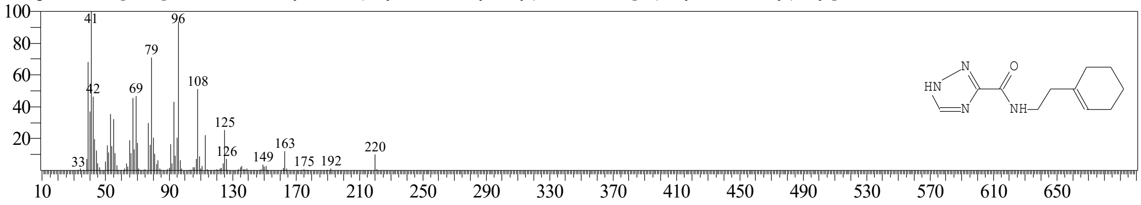

Qualitative Analysis Report

<< Target >>
Line#:53 R.Time:14.660(Scan#:2133) MassPeaks:370
RawMode:Averaged 14.655-14.665(2132-2134) BasePeak:41.05(8220)
BG Mode:Calc. from Peak Group 1 - Event 1 Scan

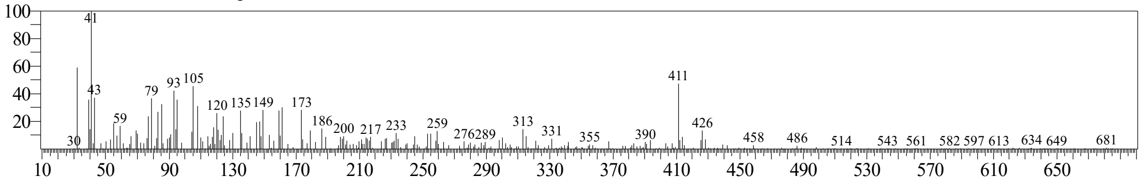

Hit#:1 Entry:205488 Library:NIST11.lib
SI:57 Formula:C30H52O6 CAS:56053-00-0 MolWeight:508 RetIndex:3493
CompName:Ergostane-3,5,6,12,25-pentol, 25-acetate, (3.beta.,5.alpha.,6.beta.,12.beta.)- $$ 3,5,6,12-Tetrahydroxyergostan-25-yl acetate # $$

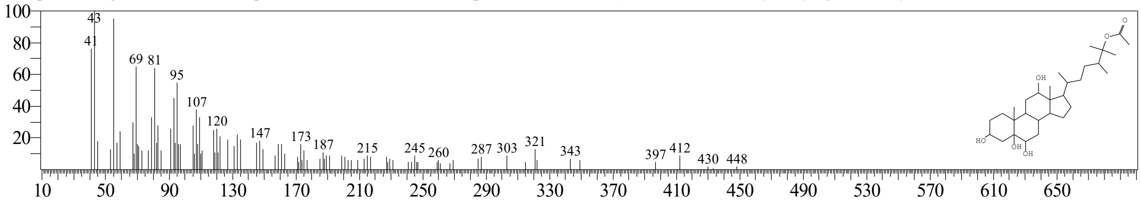

Hit#:2 Entry:57712 Library:NIST11.lib
SI:57 Formula:C15H24O CAS:0-00-0 MolWeight:220 RetIndex:1281
CompName:Isoaromadendrene epoxide $$ 1,3b,6,6-Tetramethyldecahydro-1H-cyclopropa[7,8]azuleno[4,5-b]oxirene # $$

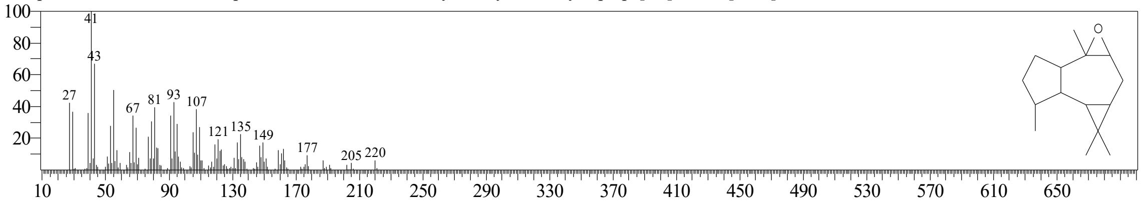

Hit#:3 Entry:191101 Library:NIST11.lib
SI:56 Formula:C30H50O CAS:469-39-6 MolWeight:426 RetIndex:2760
CompName:9,19-Cycloergost-24(28)-en-3-ol, 4,14-dimethyl-, (3.beta.,4.alpha.,5.alpha.)- $$ 9,19-Cyclo-5.alpha.,9.beta.-ergost-24(28)-en-3.beta.-ol, 4.alpha.,14

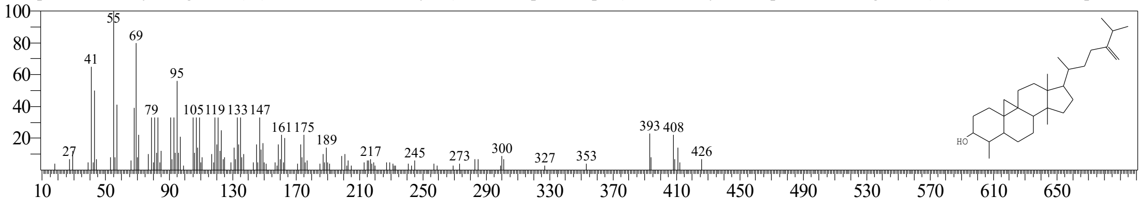

Hit#:4 Entry:137177 Library:NIST11.lib
SI:56 Formula:C23H32O CAS:0-00-0 MolWeight:324 RetIndex:2561
CompName:2-[4-methyl-6-(2,6,6-trimethylcyclohex-1-enyl)hexa-1,3,5-trienyl]cyclohex-1-en-1-carboxaldehyde $$ 2-[(1E,3E,5E)-4-Methyl-6-(2,6,6-trimethyl-1

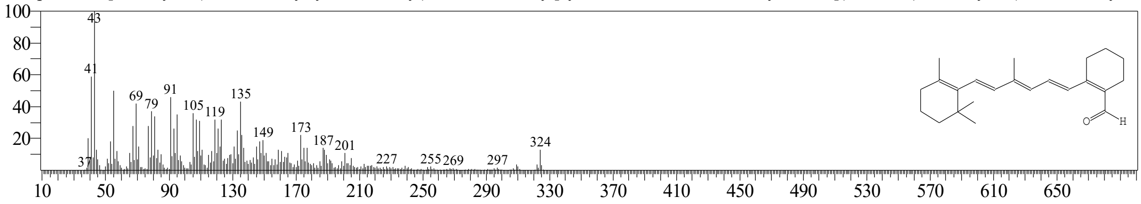

Hit#:5 Entry:90127 Library:NIST11.lib
SI:56 Formula:C15H20O4 CAS:2199-06-6 MolWeight:264 RetIndex:1954
CompName:Trichothec-9-en-8-one, 12,13-epoxy-4-hydroxy-, (4.beta.)-

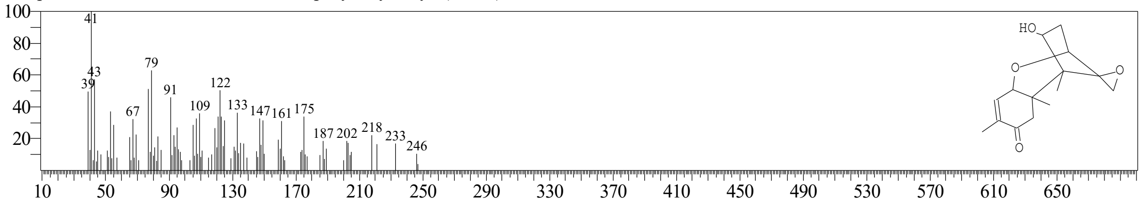

Qualitative Analysis Report

<< Target >>
Line#:54 R.Time:14.750(Scan#:2151) MassPeaks:383
RawMode:Averaged 14.745-14.755(2150-2152) BasePeak:43.05(10961)
BG Mode:Calc. from Peak Group 1 - Event 1 Scan

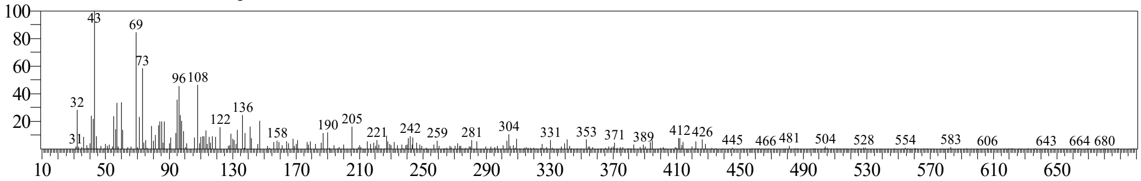

Hit#:1 Entry:133742 Library:NIST11.lib
SI:64 Formula:C15H29BrO2 CAS:74685-41-9 MolWeight:320 RetIndex:2101
CompName:Pentadecanoic acid, 14-bromo- $$ 14-Bromopentadecanoic acid # $$

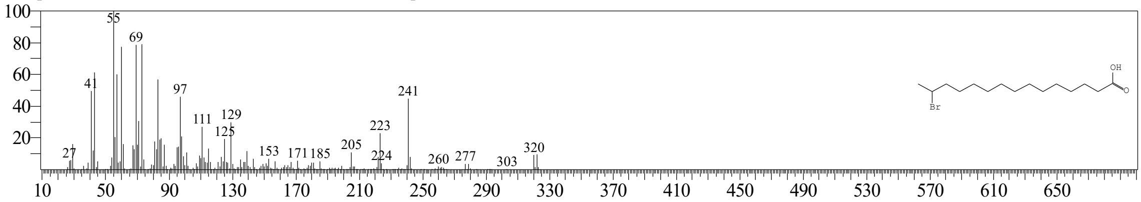

Hit#:2 Entry:51953 Library:NIST11.lib
SI:64 Formula:C11H16O4 CAS:117132-08-8 MolWeight:212 RetIndex:1610
CompName:9,9-Dimethoxybicyclo[3.3.1]nona-2,4-dione $$ 9,9-Dimethoxybicyclo[3.3.1]nonane-2,4-dione # $$

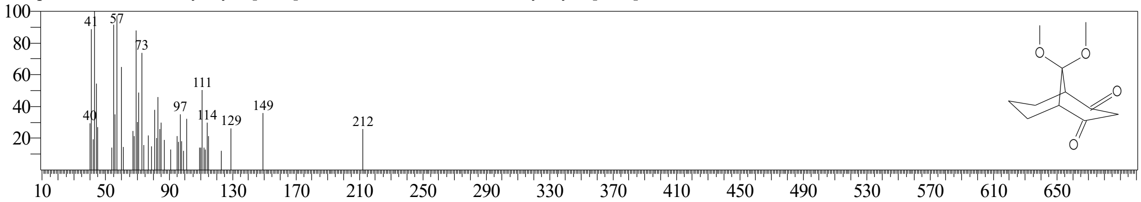

Hit#:3 Entry:128151 Library:NIST11.lib
SI:64 Formula:C20H40O2 CAS:506-30-9 MolWeight:312 RetIndex:2366
CompName:Eicosanoic acid $$ Arachic acid $$ Arachidic acid $$ Icosanoic acid $$ n-Eicosanoic acid $$ Arachidic acid (synthetic) $$

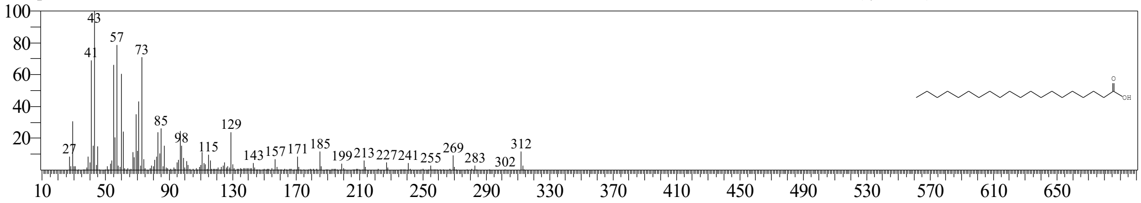

Hit#:4 Entry:166821 Library:NIST11.lib
SI:64 Formula:C24H48O2 CAS:557-59-5 MolWeight:368 RetIndex:2763
CompName:Tetracosanoic acid $$ Lignoceric acid $$

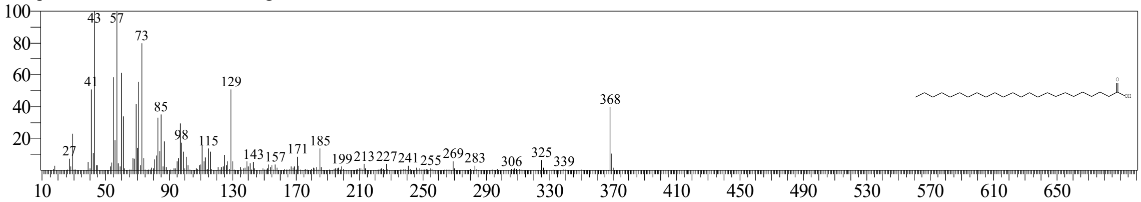

Hit#:5 Entry:211367 Library:NIST11.lib
SI:64 Formula:C38H68O8 CAS:28474-90-0 MolWeight:652 RetIndex:4765
CompName:l-(+)-Ascorbic acid 2,6-dihexadecanoate

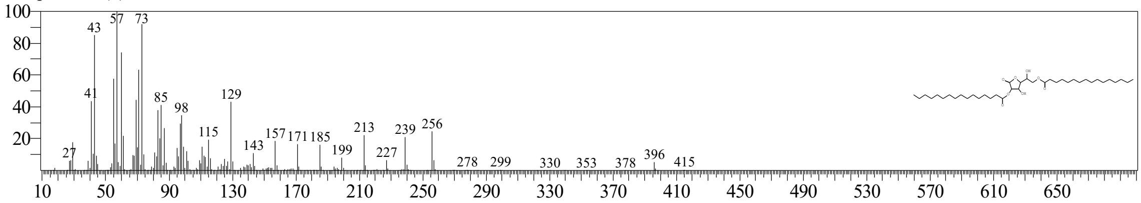

<< Target >>
Line#:55 R.Time:14.895(Scan#:2180) MassPeaks:375
RawMode:Averaged 14.890-14.900(2179-2181) BasePeak:67.05(36553)
BG Mode:Calc. from Peak Group 1 - Event 1 Scan

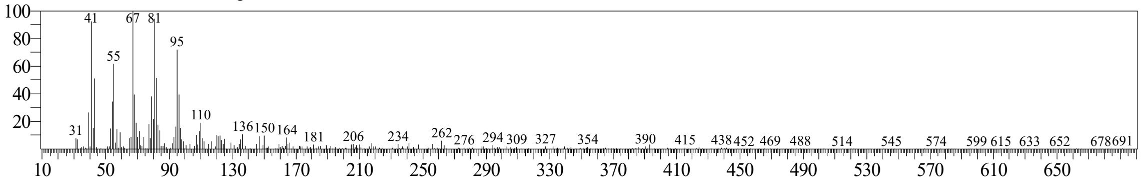

Hit#:1 Entry:113947 Library:NIST11.lib
SI:87 Formula:C19H34O2 CAS:2462-85-3 MolWeight:294 RetIndex:2093
CompName:9,12-Octadecadienoic acid, methyl ester $$ 9,12-Octadecenoic acid, methyl ester $$ Methyl octadeca-9,12-dienoate $$ Methyl 9,12-octadecadienoa

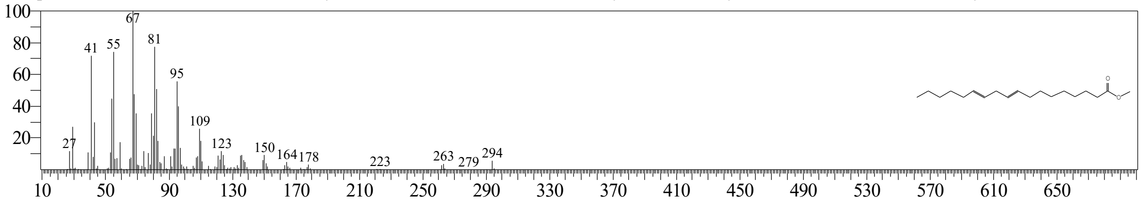

Hit#:2 Entry:135723 Library:NIST11.lib
SI:87 Formula:C21H38O2 CAS:2463-02-7 MolWeight:322 RetIndex:2292
CompName:11,14-Eicosadienoic acid, methyl ester $$ Methyl (11E,14E)-11,14-icosadienoate # $$

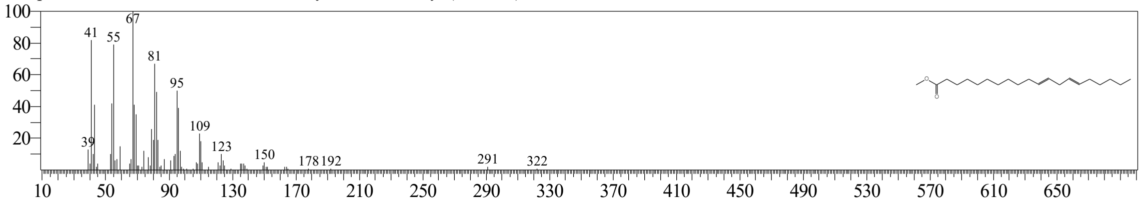

Hit#:3 Entry:81243 Library:NIST11.lib
SI:86 Formula:C16H28O2 CAS:123-69-3 MolWeight:252 RetIndex:2246
CompName:Oxacycloheptadec-8-en-2-one, (8Z) $$ Ambrettolid $$ Ambrettolide $$ Musk ambrette $$ Musk ambrette, natural $$ Musk natural $$ 7-Hexadecen

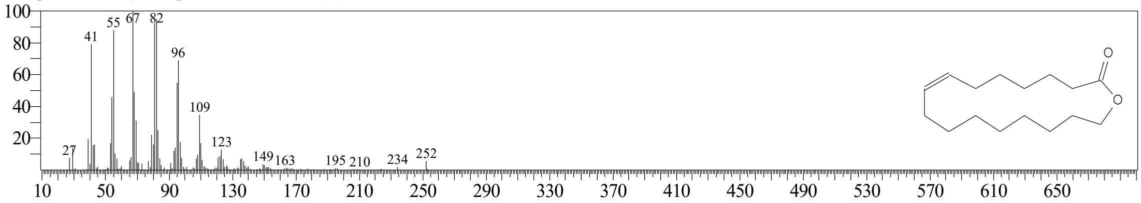

Hit#:4 Entry:113946 Library:NIST11.lib
SI:85 Formula:C19H34O2 CAS:56554-62-2 MolWeight:294 RetIndex:2093
CompName:10,13-Octadecadienoic acid, methyl ester $$ Methyl (10E,13E)-10,13-octadecadienoate # $$

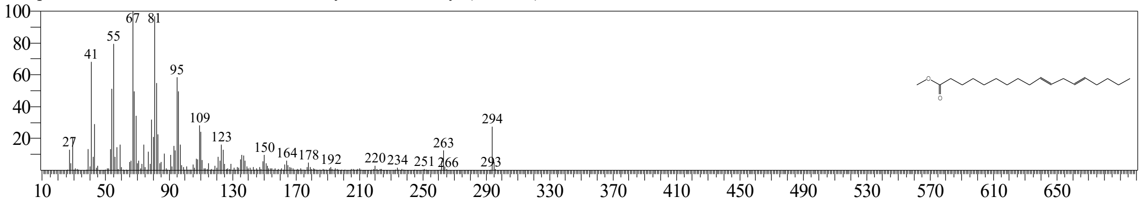

Hit#:5 Entry:113931 Library:NIST11.lib
SI:85 Formula:C19H34O2 CAS:2566-97-4 MolWeight:294 RetIndex:2093
CompName:9,12-Octadecadienoic acid, methyl ester, (E,E)- $$ Linolelaidic acid, methyl ester $$ Methyl linolelaidate $$ Methyl trans,trans-9,12-octadecadieno

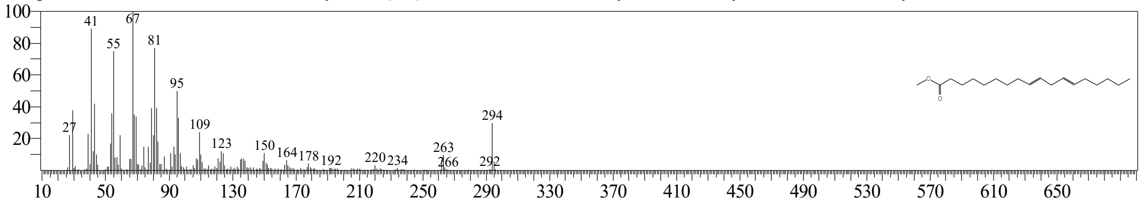

Qualitative Analysis Report

<< Target >>
Line#:56 R.Time:14.940(Scan#:2189) MassPeaks:421
RawMode:Averaged 14.935-14.945(2188-2190) BasePeak:79.05(170220)
BG Mode:Calc. from Peak Group 1 - Event 1 Scan

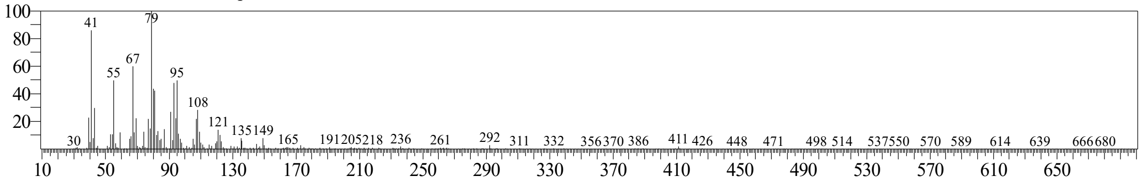

Hit#:1 Entry:132823 Library:NIST11.lib
SI:90 Formula:C21H34O2 CAS:59149-01-8 MolWeight:318 RetIndex:2308
CompName:Methyl (Z)-5,11,14,17-eicosatetraenoate $$ Methyl (5Z,11Z,14Z,17Z)-5,11,14,17-icosatetraenoate # $$

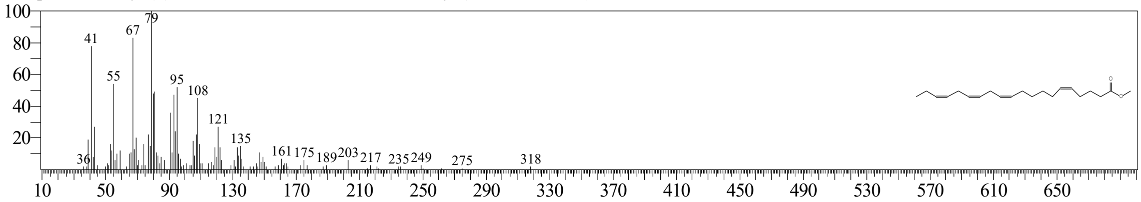

Hit#:2 Entry:90440 Library:NIST11.lib
SI:90 Formula:C17H28O2 CAS:56554-30-4 MolWeight:264 RetIndex:1902
CompName:7,10,13-Hexadecatrienoic acid, methyl ester $$ Methyl (7E,10E,13E)-7,10,13-hexadecatrienoate # $$

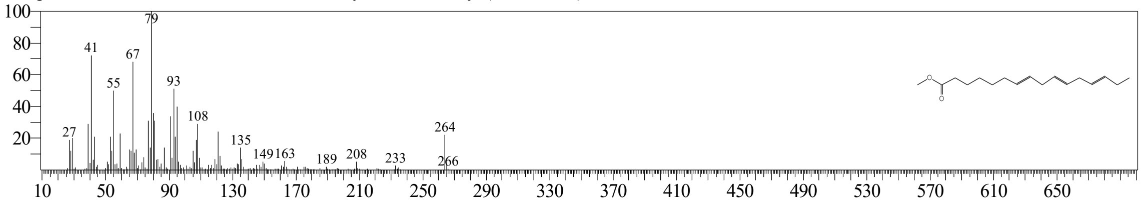

Hit#:3 Entry:112450 Library:NIST11.lib
SI:89 Formula:C19H32O2 CAS:301-00-8 MolWeight:292 RetIndex:2101
CompName:9,12,15-Octadecatrienoic acid, methyl ester, (Z,Z,Z)- $$ Linolenic acid, methyl ester $$ Methyl all-cis-9,12,15-octadecatrienoate $$ Methyl linolen

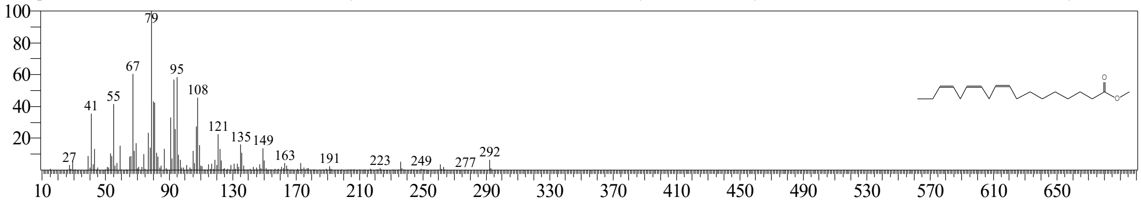

Hit#:4 Entry:101390 Library:NIST11.lib
SI:89 Formula:C18H30O2 CAS:463-40-1 MolWeight:278 RetIndex:2191
CompName:9,12,15-Octadecatrienoic acid, (Z,Z,Z)- $$ Linolenic acid $$ .alpha.-Linolenic acid $$ All-cis-9,12,15-Octadecatrienoic acid $$ cis,cis,cis-9,12,15-

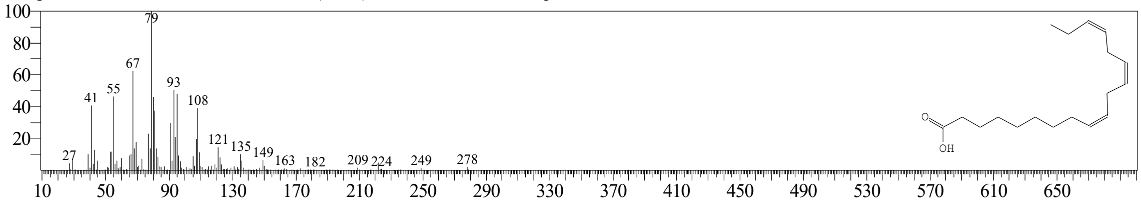

Hit#:5 Entry:90503 Library:NIST11.lib
SI:89 Formula:C18H32O CAS:506-44-5 MolWeight:264 RetIndex:2077
CompName:9,12,15-Octadecatrien-1-ol, (Z,Z,Z)- $$ (9E,12E,15E)-9,12,15-Octadecatrien-1-ol # $$ (Z,Z,Z)-9,12,15-Octadecatrien-1-ol $$

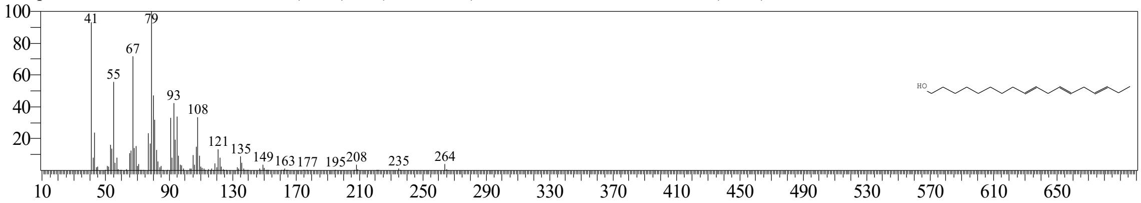

<< Target >>
Line#:57 R.Time:15.015(Scan#:2204) MassPeaks:384
RawMode:Averaged 15.010-15.020(2203-2205) BasePeak:71.05(340586)
BG Mode:Calc. from Peak Group 1 - Event 1 Scan

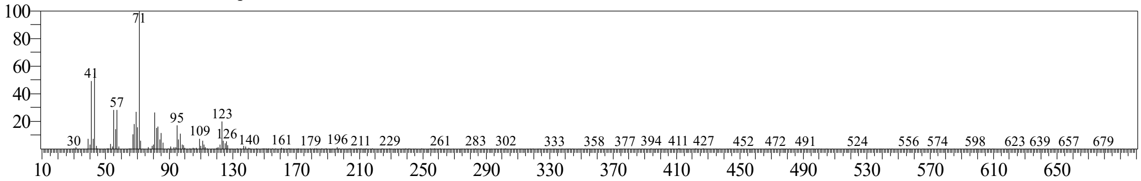

Hit#:1 Entry:115516 Library:NIST11.lib
SI:95 Formula:C20H40O CAS:150-86-7 MolWeight:296 RetIndex:2045
CompName:Phytol $$ 2-Hexadecen-1-ol, 3,7,11,15-tetramethyl-, [R-[R*,R*-(E)]]- $$ trans-Phytol $$ 3,7,11,15-Tetramethyl-2-hexadecen-1-ol-, (2E,7R,11R)- $

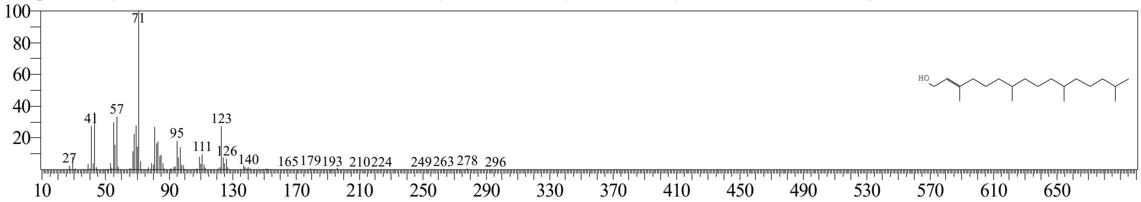

Hit#:2 Entry:84384 Library:NIST11.lib
SI:87 Formula:C16H32O2 CAS:0-00-0 MolWeight:256 RetIndex:1936
CompName:13-Methylpentadec-14-ene-1,13-diol $$ 13-Methyl-14-pentadecene-1,13-diol # $$

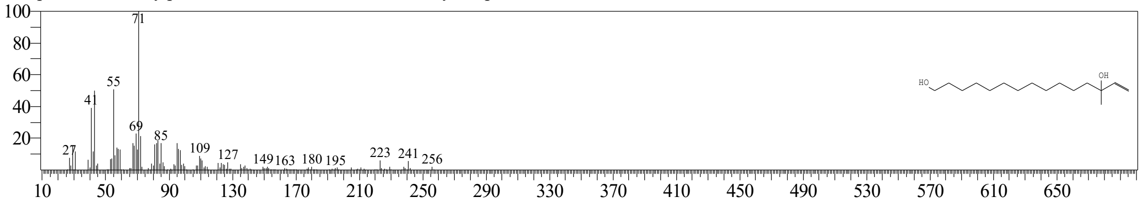

Hit#:3 Entry:18678 Library:NIST11.lib
SI:87 Formula:C10H20O CAS:491-01-0 MolWeight:156 RetIndex:1164
CompName:Cyclohexanol, 5-methyl-2-(1-methylethyl)-, (1.alpha.,2.alpha.,5.beta.)- $$ Menthol, trans-1,3,trans-1,4- $$ Neo-Menthol $$ Neomenthol $$ 2-Isopr

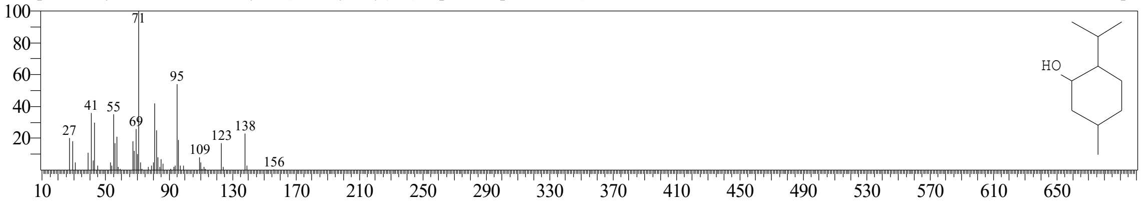

Hit#:4 Entry:18670 Library:NIST11.lib
SI:87 Formula:C10H20O CAS:23283-97-8 MolWeight:156 RetIndex:1164
CompName:Cyclohexanol, 5-methyl-2-(1-methylethyl)-, [1S-(1.alpha.,2.beta.,5.beta.)]- $$ (1S,2R,5R)-(+)-Isomenthol $$ 2-Isopropyl-5-methylcyclohexanol # $

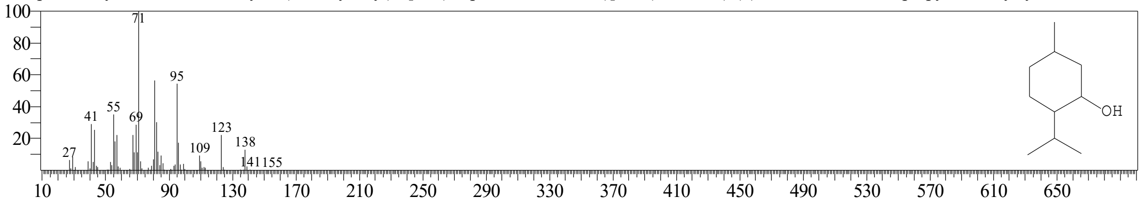

Hit#:5 Entry:18679 Library:NIST11.lib
SI:86 Formula:C10H20O CAS:490-99-3 MolWeight:156 RetIndex:1164
CompName:Cyclohexanol, 5-methyl-2-(1-methylethyl)-, (1.alpha.,2.beta.,5.beta.)- $$ Menthol, trans-1,3,cis-1,4- $$ Isomenthol $$ Isomentol $$ 2-Isopropyl-5-n

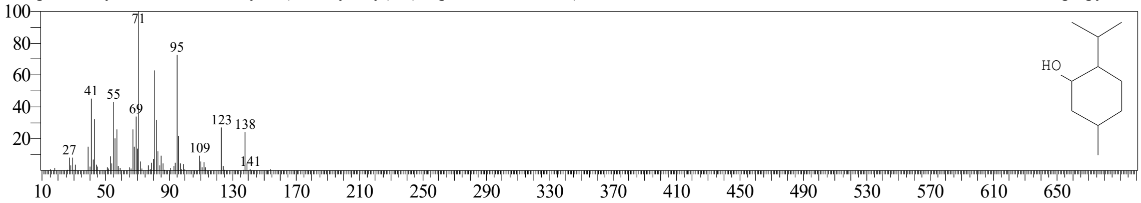

<< Target >>
Line#:58 R.Time:15.265(Scan#:2254) MassPeaks:446
RawMode:Averaged 15.260-15.270(2253-2255) BasePeak:41.05(166042)
BG Mode:Calc. from Peak Group 1 - Event 1 Scan

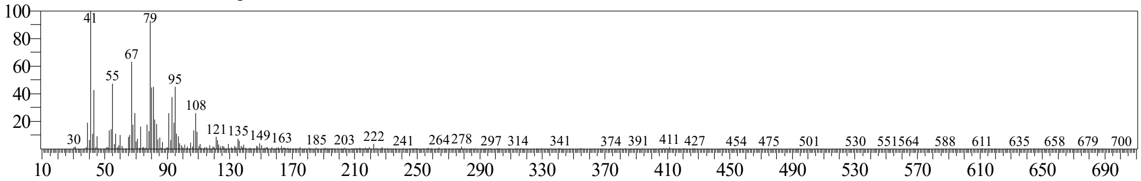

Hit#:1 Entry:101390 Library:NIST11.lib
SI:90 Formula:C18H30O2 CAS:463-40-1 MolWeight:278 RetIndex:2191
CompName:9,12,15-Octadecatrienoic acid, (Z,Z,Z)- $$ Linolenic acid $$ .alpha.-Linolenic acid $$ All-cis-9,12,15-Octadecatrienoic acid $$ cis,cis,cis-9,12,15-(

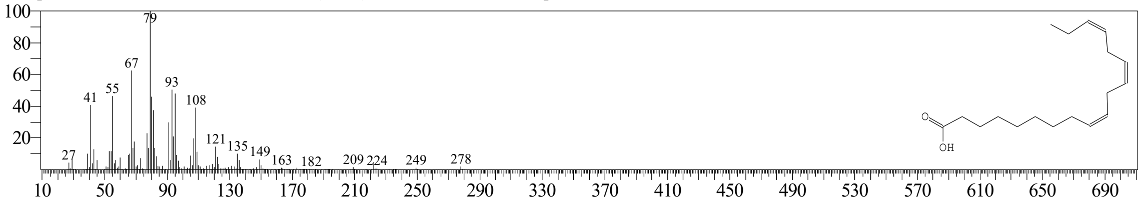

Hit#:2 Entry:90503 Library:NIST11.lib
SI:89 Formula:C18H32O CAS:506-44-5 MolWeight:264 RetIndex:2077
CompName:9,12,15-Octadecatrien-1-ol, (Z,Z,Z)- $$ (9E,12E,15E)-9,12,15-Octadecatrien-1-ol # $$ (Z,Z,Z)-9,12,15-Octadecatrien-1-ol $$

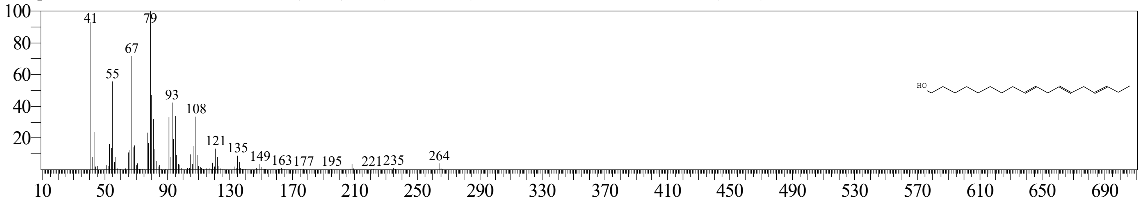

Hit#:3 Entry:67943 Library:NIST11.lib
SI:89 Formula:C16H26O CAS:56797-43-4 MolWeight:234 RetIndex:1824
CompName:cis,cis,cis-7,10,13-Hexadecatrienal $$ (7Z,10Z,13Z)-7,10,13-Hexadecatrienal # $$

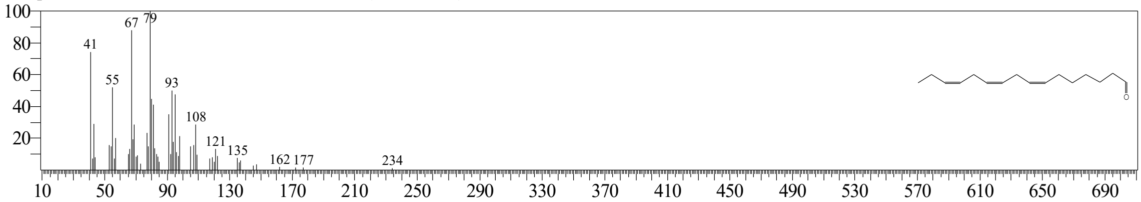

Hit#:4 Entry:123525 Library:NIST11.lib
SI:87 Formula:C20H34O2 CAS:1783-84-2 MolWeight:306 RetIndex:2390
CompName:8,11,14-Eicosatrienoic acid, (Z,Z,Z)- $$ cis-8,11,14-Eicosatrienoic Acid $$ Dihomo-.gamma.-linolenic acid $$ (8E,11E,14E)-8,11,14-Icosatrienoic

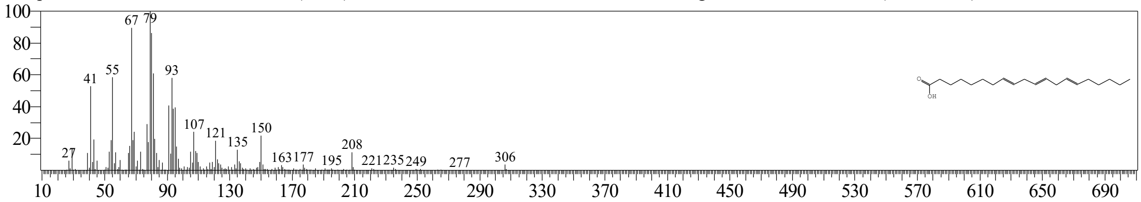

Hit#:5 Entry:89012 Library:NIST11.lib
SI:86 Formula:C18H30O CAS:26537-71-3 MolWeight:262 RetIndex:2023
CompName:9,12,15-Octadecatrienal $$ (9E,12E,15E)-9,12,15-Octadecatrienal # $$

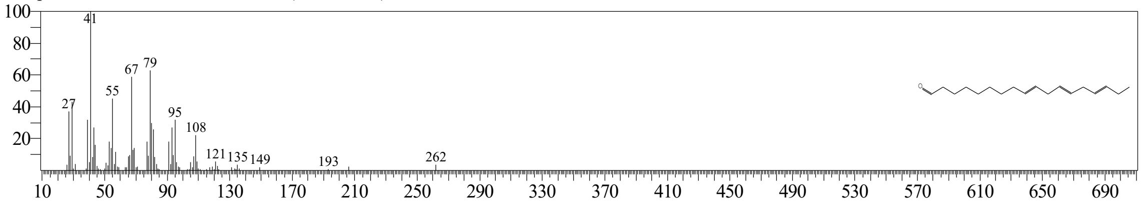

<< Target >>
Line#:59 R.Time:15.385(Scan#:2278) MassPeaks:395
RawMode:Averaged 15.380-15.390(2277-2279) BasePeak:43.05(40409)
BG Mode:Calc. from Peak Group 1 - Event 1 Scan

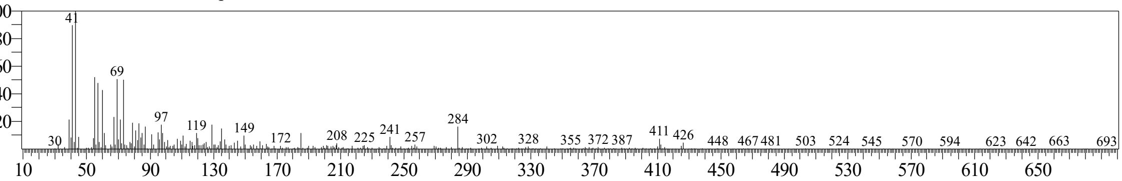

Hit#:1 Entry:169171 Library:NIST11.lib
SI:79 Formula:C22H44O4 CAS:106-11-6 MolWeight:372 RetIndex:2694
CompName:Octadecanoic acid, 2-(2-hydroxyethoxy)ethyl ester $$ Aqua Cera $$ Atlas G 2146 $$ Cerasynt $$ Cerasynt Special $$ Clindrol SDG $$ Diethylene

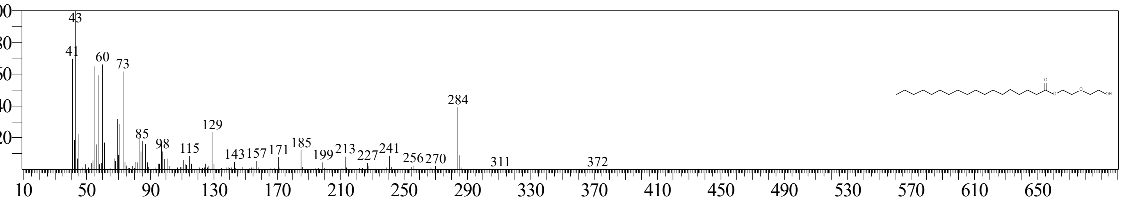

Hit#:2 Entry:128151 Library:NIST11.lib
SI:79 Formula:C20H40O2 CAS:506-30-9 MolWeight:312 RetIndex:2366
CompName:Eicosanoic acid $$ Arachic acid $$ Arachidic acid $$ Icosanoic acid $$ n-Eicosanoic acid $$ Arachidic acid (synthetic) $$

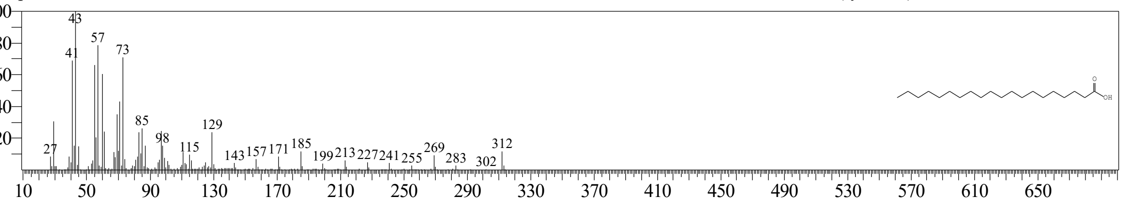

Hit#:3 Entry:84362 Library:NIST11.lib
SI:78 Formula:C16H32O2 CAS:57-10-3 MolWeight:256 RetIndex:1968
CompName:n-Hexadecanoic acid $$ Hexadecanoic acid $$ n-Hexadecoic acid $$ Palmitic acid $$ Pentadecanecarboxylic acid $$ 1-Pentadecanecarboxylic acid

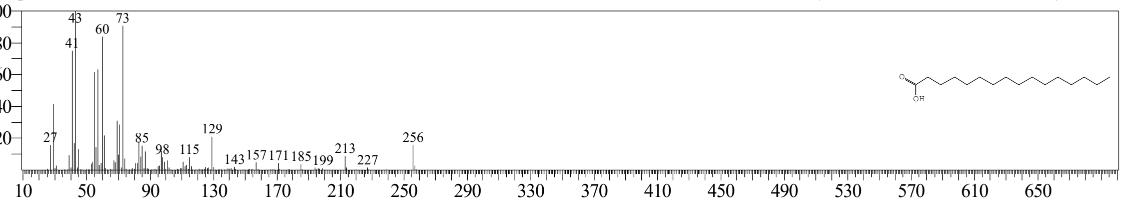

Hit#:4 Entry:106158 Library:NIST11.lib
SI:78 Formula:C18H36O2 CAS:57-11-4 MolWeight:284 RetIndex:2167
CompName:Octadecanoic acid $$ Stearic acid $$ n-Octadecanoic acid $$ Humko Industrene R $$ Hydrofol Acid 150 $$ Hystrene S-97 $$ Hystrene T-70 $$ Hys

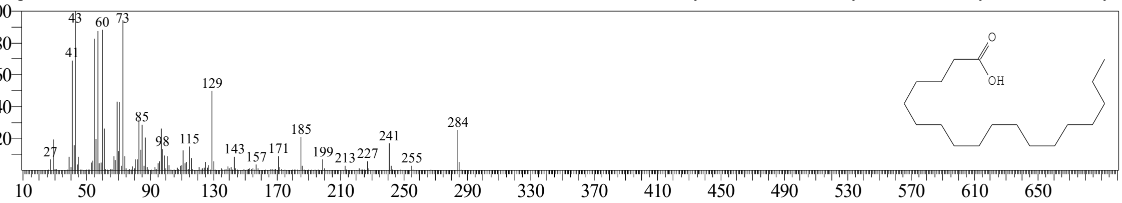

Hit#:5 Entry:117133 Library:NIST11.lib
SI:77 Formula:C19H38O2 CAS:646-30-0 MolWeight:298 RetIndex:2266
CompName:Nonadecanoic acid $$ n-Nonadecanoic acid $$

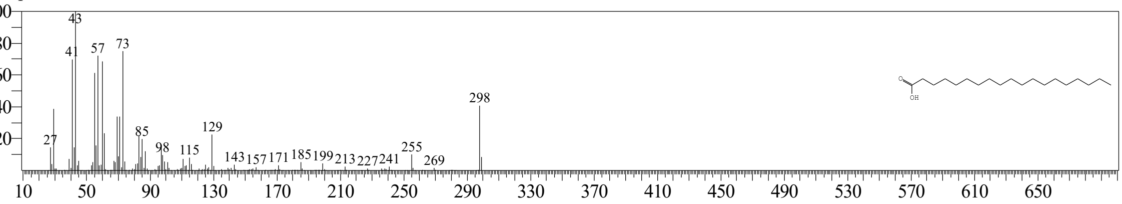

<< Target >>
Line#:60 R.Time:15.470(Scan#:2295) MassPeaks:409
RawMode:Averaged 15.465-15.475(2294-2296) BasePeak:56.05(182039)
BG Mode:Calc. from Peak Group 1 - Event 1 Scan

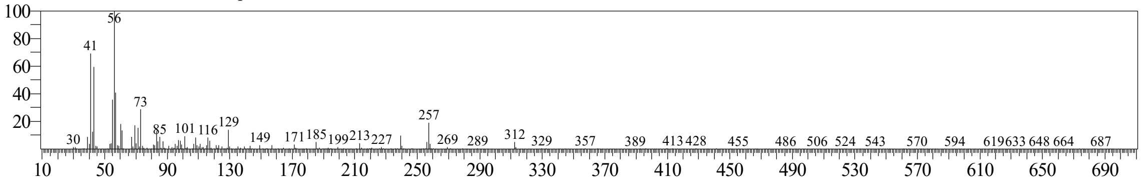

Hit#:1 Entry:128159 Library:NIST11.lib
SI:89 Formula:C20H40O2 CAS:111-06-8 MolWeight:312 RetIndex:2177
CompName:Hexadecanoic acid, butyl ester $$ Palmitic acid, butyl ester $$ n-Butyl hexadecanoate $$ n-Butyl palmitate $$ Butyl palmitate $$ Butyl ester of hex[illegible]

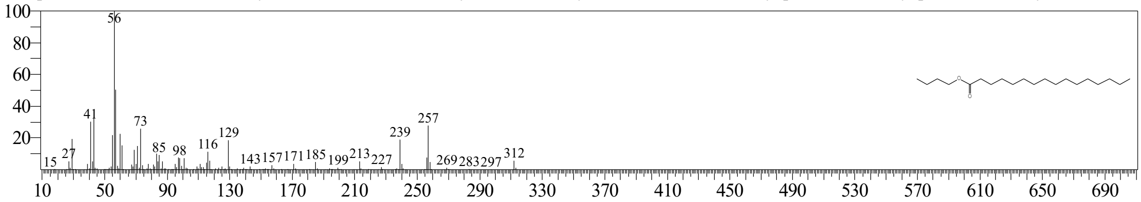

Hit#:2 Entry:128158 Library:NIST11.lib
SI:89 Formula:C20H40O2 CAS:31158-91-5 MolWeight:312 RetIndex:2092
CompName:Hexadecanoic acid, 1,1-dimethylethyl ester $$ tert-Butyl palmitate # $$

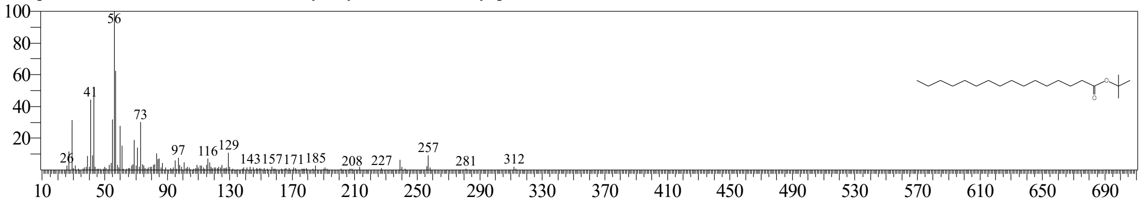

Hit#:3 Entry:106162 Library:NIST11.lib
SI:87 Formula:C18H36O2 CAS:110-36-1 MolWeight:284 RetIndex:1978
CompName:Butyl myristate $$ n-Butyl myristate $$ Myristic acid n-butyl ester $$ Tetradecanoic acid, butyl ester $$ Bumyr $$ Myristic acid, butyl ester $$ Buty[illegible]

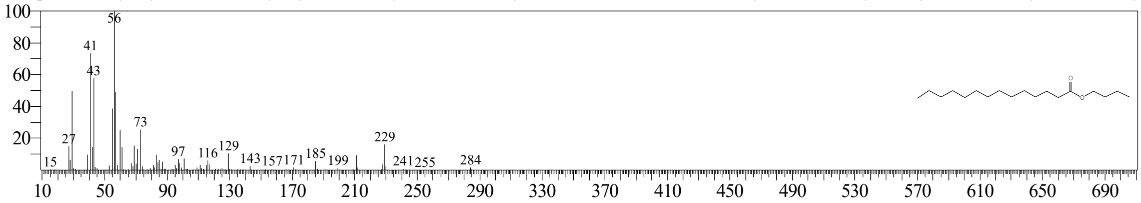

Hit#:4 Entry:128162 Library:NIST11.lib
SI:87 Formula:C20H40O2 CAS:110-34-9 MolWeight:312 RetIndex:2112
CompName:Hexadecanoic acid, 2-methylpropyl ester $$ 2-methylpropyl hexadecanoate $$ isobutyl hexadecanoate $$

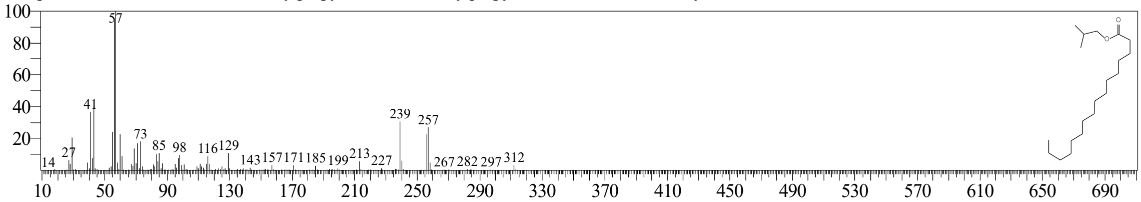

Hit#:5 Entry:149085 Library:NIST11.lib
SI:81 Formula:C22H44O2 CAS:646-13-9 MolWeight:340 RetIndex:2311
CompName:Octadecanoic acid, 2-methylpropyl ester $$ Stearic acid, isobutyl ester $$ Isobutyl stearate $$ Emerest 2324 $$ Estol 1476 $$ Kemester 5415 $$ Ke[illegible]

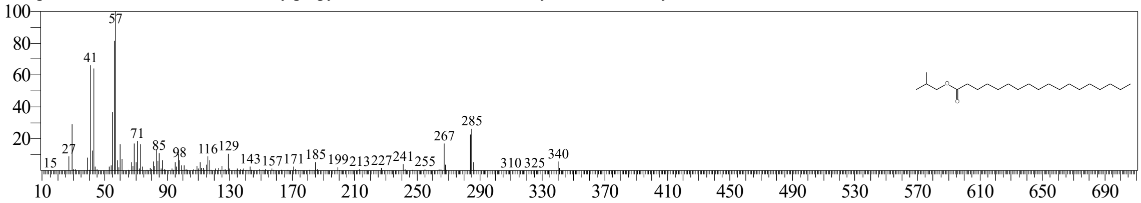

<< Target >>
Line#:61 R.Time:15.520(Scan#:2305) MassPeaks:366
RawMode:Averaged 15.515-15.525(2304-2306) BasePeak:43.10(71724)
BG Mode:Calc. from Peak Group 1 - Event 1 Scan

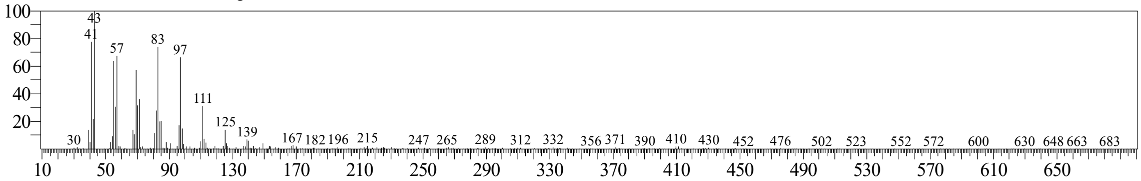

Hit#:1 Entry:70935 Library:NIST11.lib
SI:93 Formula:C17H34 CAS:6765-39-5 MolWeight:238 RetIndex:1701
CompName:1-Heptadecene $$ Hexahydroaplotaxene $$ Heptadec-1-ene $$ NSC 77132 $$ Heptadecene (Related) $$

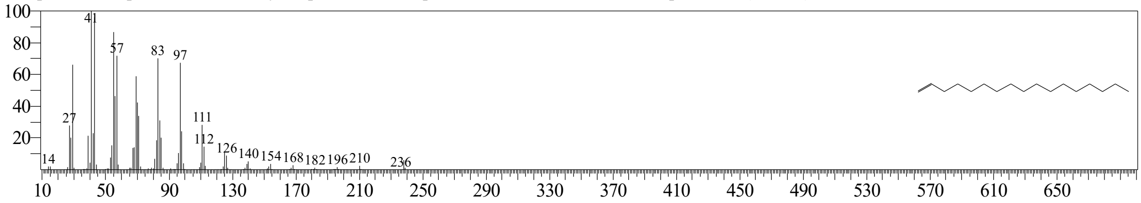

Hit#:2 Entry:81347 Library:NIST11.lib
SI:93 Formula:C18H36 CAS:112-88-9 MolWeight:252 RetIndex:1801
CompName:1-Octadecene $$ .alpha.-Octadecene $$ Octadecylene, .alpha.- $$ Neodene 18 $$ Octadec-1-ene $$ Octadecene-1 $$ Octadeca-1-ene $$

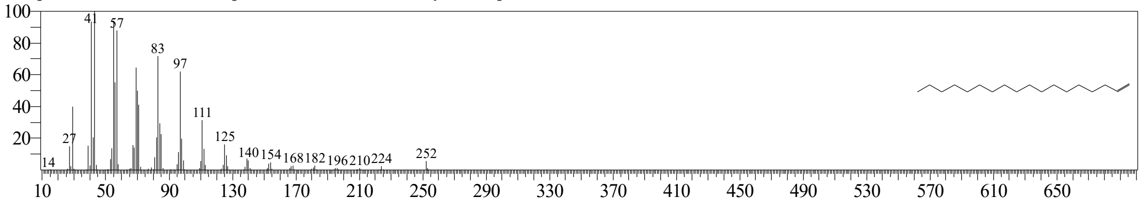

Hit#:3 Entry:102910 Library:NIST11.lib
SI:92 Formula:C20H40 CAS:296-56-0 MolWeight:280 RetIndex:2398
CompName:Cycloeicosane $$ Cycloicosane # $$

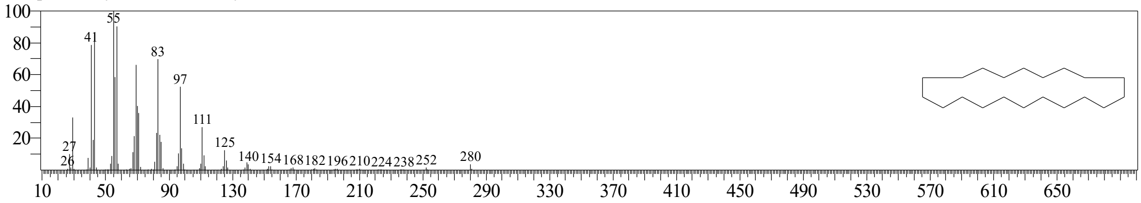

Hit#:4 Entry:81317 Library:NIST11.lib
SI:92 Formula:C17H32O CAS:0-00-0 MolWeight:252 RetIndex:1907
CompName:E-15-Heptadecenal $$ (15E)-15-Heptadecenal # $$

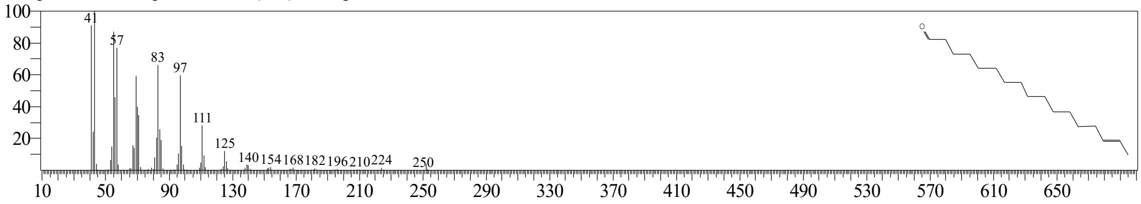

Hit#:5 Entry:147159 Library:NIST11.lib
SI:92 Formula:C18H33F3O2 CAS:6222-03-3 MolWeight:338 RetIndex:1812
CompName:Trifluoroacetoxy hexadecane $$ Hexadecyl trifluoroacetate $$ Hexadecyl 2,2,2-trifluoroacetate $$ 1-Hexadecanol, trifluoroacetate $$ Acetic acid, tr

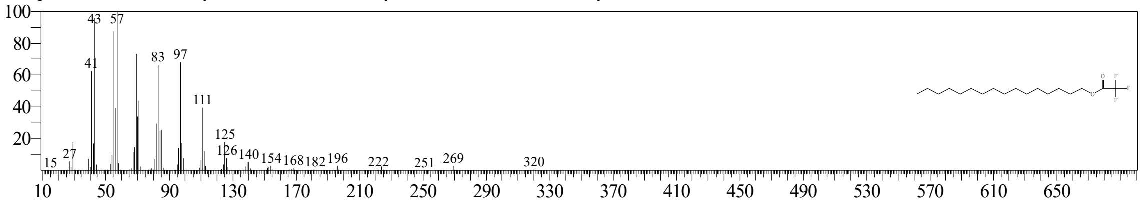

<< Target >>
Line#:62 R.Time:15.615(Scan#:2324) MassPeaks:341
RawMode:Averaged 15.610-15.620(2323-2325) BasePeak:149.10(3442)
BG Mode:Calc. from Peak Group 1 - Event 1 Scan

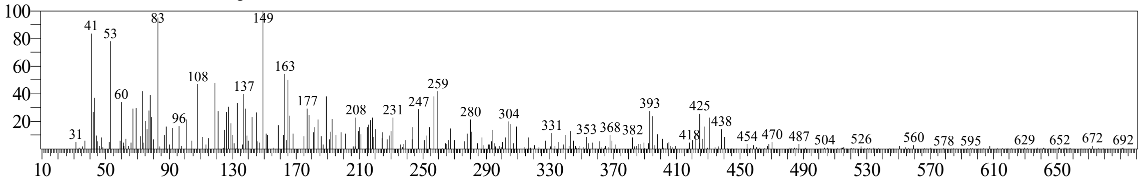

Hit#:1 Entry:49478 Library:NIST11.lib
SI:44 Formula:C13H20O2 CAS:0-00-0 MolWeight:208 RetIndex:1397
CompName:Cyclopentanecarboxylic acid, 3-methylene-2,2-dimethyl-5-[(E)-1-propenyl]-, methyl ester $$ Methyl 2,2-dimethyl-3-methylene-5-[(1E)-1-propenyl]

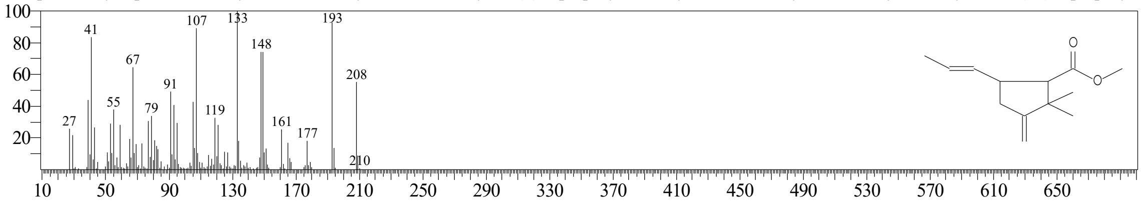

Hit#:2 Entry:206509 Library:NIST11.lib
SI:44 Formula:C27H36O10 CAS:64807-01-8 MolWeight:520 RetIndex:3239
CompName:4H-Cyclopropa[5',6']benz[1',2':7,8]azuleno[5,6-b]oxiren-4-one, 8,8a-bis(acetyloxy)-2a-[(acetyloxy)methyl]-1,1a,1b,1c,2a,3,3a,6a,6b,7,8,8a-dodecah

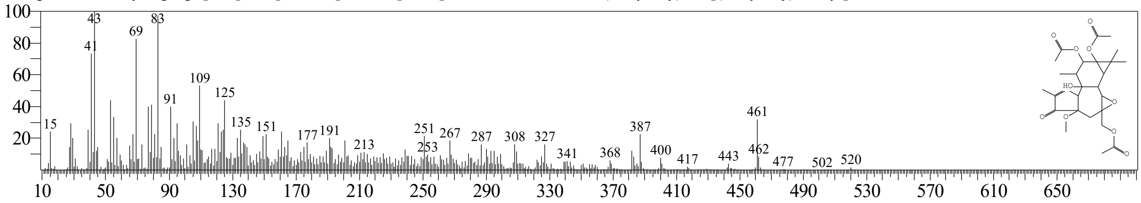

Hit#:3 Entry:209817 Library:NIST11.lib
SI:44 Formula:C28H37ClO11 CAS:77573-36-5 MolWeight:584 RetIndex:3723
CompName:5H-Cyclopropa[3,4]benz[1,2-e]azulen-5-one, 3,9,9a-tris(acetyloxy)-3-[(acetyloxy)methyl]-2-chloro-1,1a,1b,2,3,4,4a,7a,7b,8,9,9a-dodecahydro-4a,7

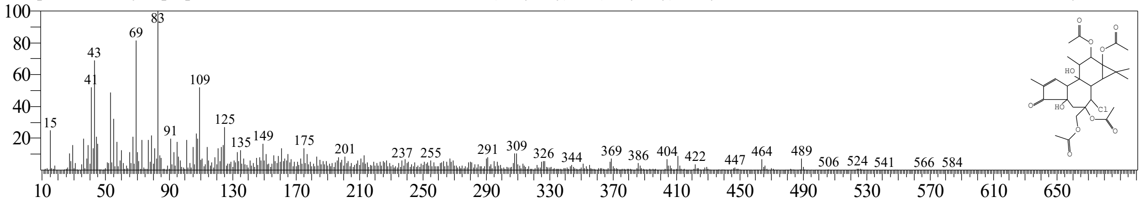

Hit#:4 Entry:57565 Library:NIST11.lib
SI:44 Formula:C14H20O2 CAS:23585-24-2 MolWeight:220 RetIndex:1522
CompName:Tricyclo[5.1.0.0(3,5)]octane-2,6-dione, 1,3,4,5,7,8-hexamethyl-, stereoisomer

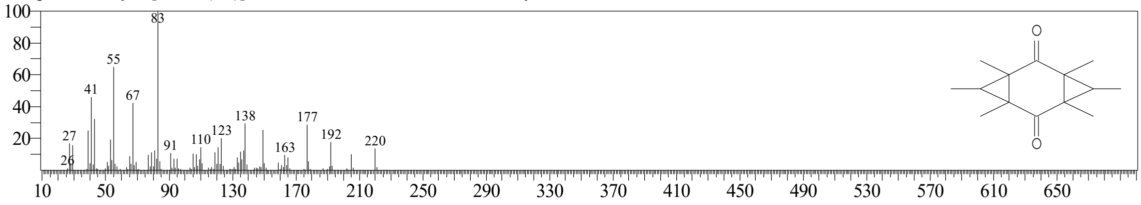

Hit#:5 Entry:66596 Library:NIST11.lib
SI:43 Formula:C16H24O CAS:0-00-0 MolWeight:232 RetIndex:1688
CompName:Bicyclo[5.1.0]octan-2-one, 4,6-diisopropylidene-8,8-dimethyl- $$ 8,8-Dimethyl-4,6-bis(1-methylethylidene)bicyclo[5.1.0]octan-2-one # $$

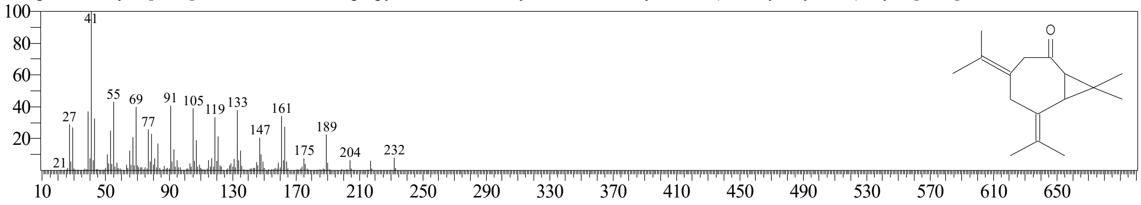

Qualitative Analysis Report

<< Target >>
Line#:63 R.Time:15.655(Scan#:2332) MassPeaks:358
RawMode:Averaged 15.650-15.660(2331-2333) BasePeak:108.05(6715)
BG Mode:Calc. from Peak Group 1 - Event 1 Scan

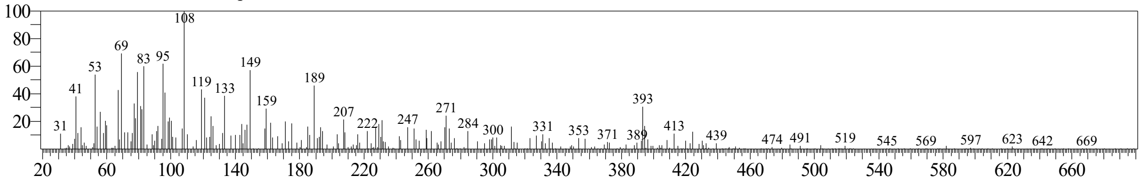

Hit#:1 Entry:46664 Library:NIST11.lib
SI:52 Formula:C15H24 CAS:0-00-0 MolWeight:204 RetIndex:1475
CompName:Cycloheptane, 4-methylene-1-methyl-2-(2-methyl-1-propen-1-yl)-1-vinyl- $$ 1-Methyl-4-methylene-2-(2-methyl-1-propenyl)-1-vinylcycloheptane #

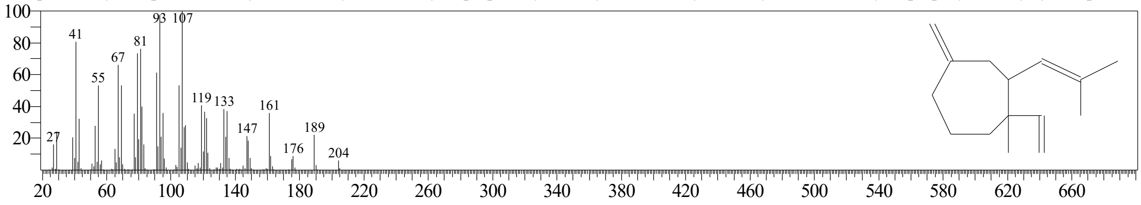

Hit#:2 Entry:46668 Library:NIST11.lib
SI:51 Formula:C15H24 CAS:116-04-1 MolWeight:204 RetIndex:1574
CompName:.beta.-Humulene $$ 1,4,4-Trimethyl-8-methylene-1,5-cycloundecadiene # $$

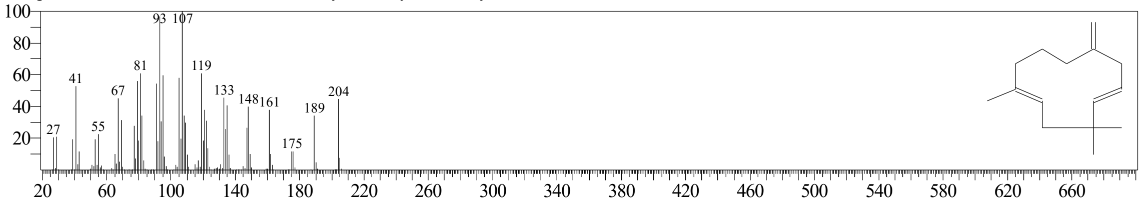

Hit#:3 Entry:118922 Library:NIST11.lib
SI:51 Formula:C21H32O CAS:54550-04-8 MolWeight:300 RetIndex:2057
CompName:Androst-1-en-3-one, 4,4-dimethyl-, (5.alpha.)- $$ 4,4-Dimethylandrost-1-en-3-one # $$

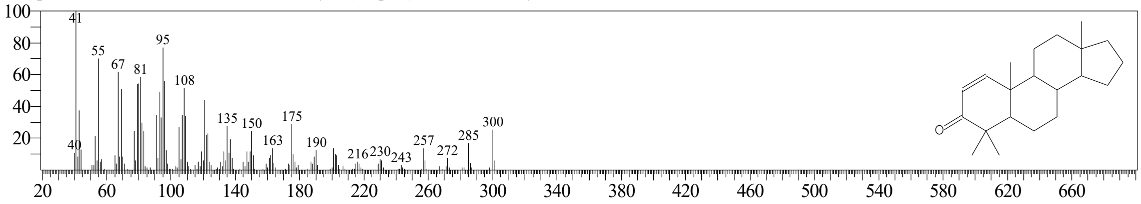

Hit#:4 Entry:76882 Library:NIST11.lib
SI:51 Formula:C18H30 CAS:0-00-0 MolWeight:246 RetIndex:1649
CompName:d-Norandrostane (5.alpha.,14.alpha.) $$ 8a,10a-Dimethylhexadecahydrocyclobuta[a]phenanthrene # $$

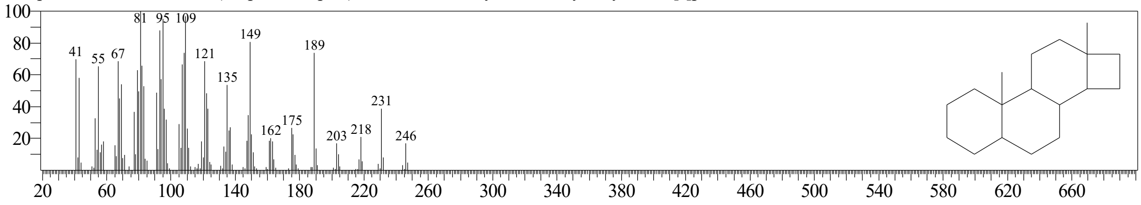

Hit#:5 Entry:15429 Library:NIST11.lib
SI:51 Formula:C10H14O CAS:17429-25-3 MolWeight:150 RetIndex:1208
CompName:5H-Inden-5-one, 1,2,3,3a,4,7a-hexahydro-7a-methyl-, trans- $$ 5(4H)-Indanone, 3a,7a-dihydro-7a-methyl-, trans- $$ 7a-Methyl-1,2,3,3a,4,7a-hexah

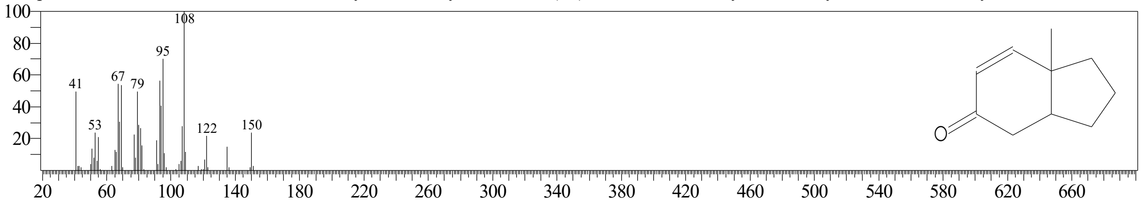

<< Target >>
Line#:64 R.Time:15.780(Scan#:2357) MassPeaks:367
RawMode:Averaged 15.775-15.785(2356-2358) BasePeak:55.05(9388)
BG Mode:Calc. from Peak Group 1 - Event 1 Scan

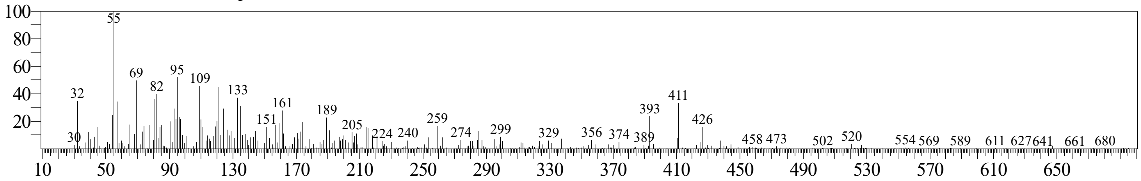

Hit#:1 Entry:191101 Library:NIST11.lib
SI:56 Formula:C30H50O CAS:469-39-6 MolWeight:426 RetIndex:2760
CompName:9,19-Cycloergost-24(28)-en-3-ol, 4,14-dimethyl-, (3.beta.,4.alpha.,5.alpha.)- $$ 9,19-Cyclo-5.alpha.,9.beta.-ergost-24(28)-en-3.beta.-ol, 4.alpha.,14

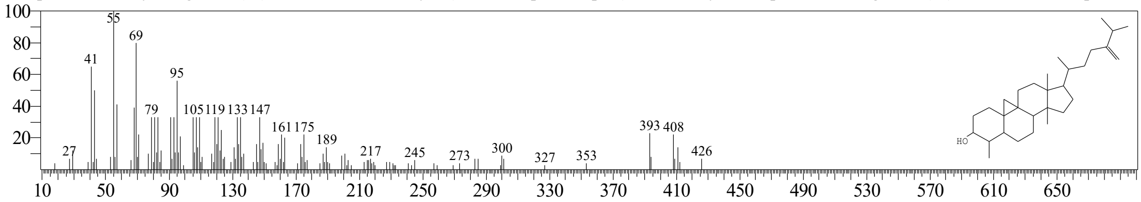

Hit#:2 Entry:196536 Library:NIST11.lib
SI:56 Formula:C28H48O4 CAS:56052-97-2 MolWeight:448 RetIndex:3179
CompName:Ergost-25-ene-3,5,6,12-tetrol, (3.beta.,5.alpha.,6.beta.,12.beta.)- $$ Ergost-25(27)-ene-3,5,6,12-tetrol # $$

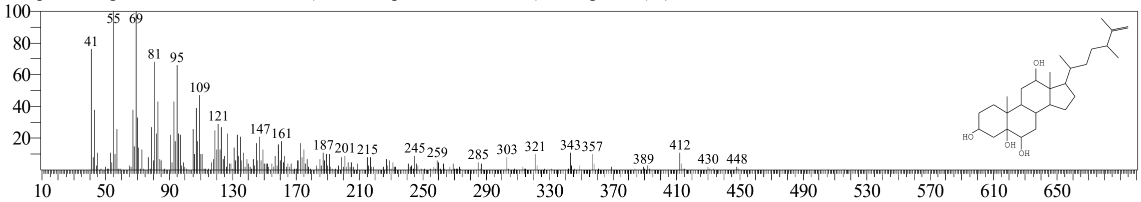

Hit#:3 Entry:199030 Library:NIST11.lib
SI:55 Formula:C31H56O2 CAS:0-00-0 MolWeight:460 RetIndex:3230
CompName:i-Propyl 5,9,19-octacosatrienoate

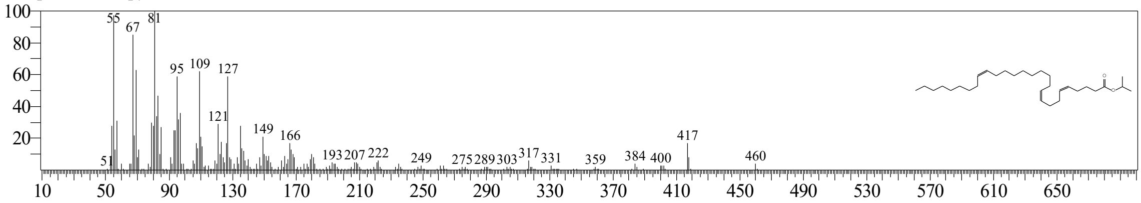

Hit#:4 Entry:59406 Library:NIST11.lib
SI:55 Formula:C15H26O CAS:27551-75-3 MolWeight:222 RetIndex:1593
CompName:1,4-Methanoazulen-9-ol, decahydro-1,5,5,8a-tetramethyl-, [1R-(1.alpha. ..

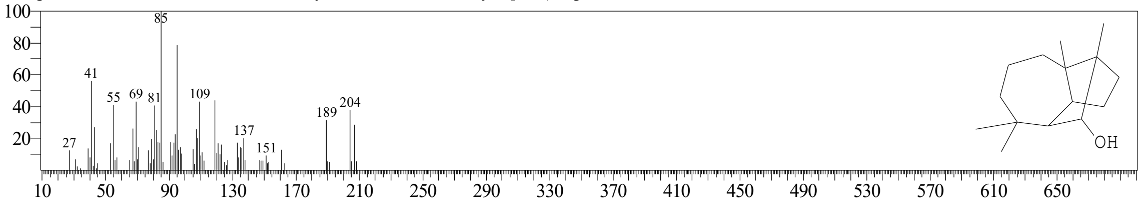

Hit#:5 Entry:197367 Library:NIST11.lib
SI:55 Formula:C33H56 CAS:0-00-0 MolWeight:452 RetIndex:2885
CompName:1,1,6-trimethyl-3-methylene-2-(3,6,9,13-tetramethyl-6-ethenye-10,14-dimethylene-pentadec-4-enyl)cyclohexane

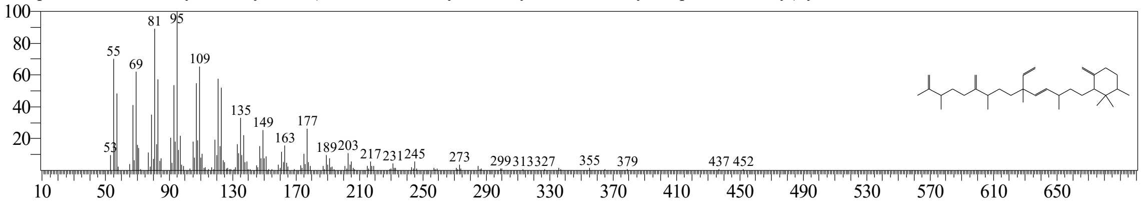

<< Target >>
Line#:65 R.Time:15.915(Scan#:2384) MassPeaks:398
RawMode:Averaged 15.910-15.920(2383-2385) BasePeak:108.05(19337)
BG Mode:Calc. from Peak Group 1 - Event 1 Scan

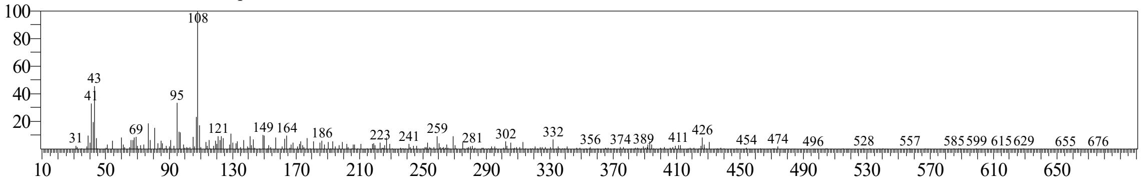

Hit#:1 Entry:48461 Library:NIST11.lib
SI:62 Formula:C11H17N3O CAS:0-00-0 MolWeight:207 RetIndex:1793
CompName:1-Acetyl-4-amino-5-isobutyl-2,5-dihydro-1H-pyrrole-3-carbonitrile $$ Cl-4838 $$

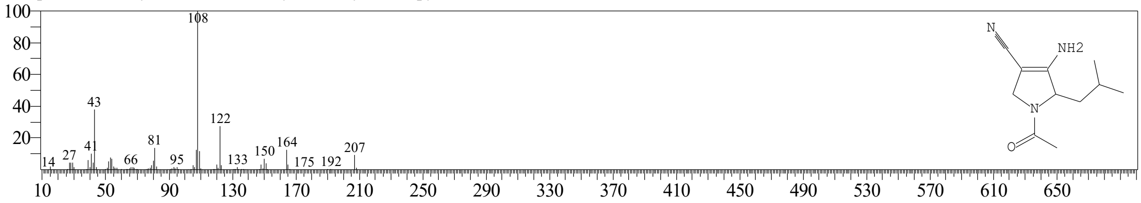

Hit#:2 Entry:143347 Library:NIST11.lib
SI:61 Formula:C22H36O2 CAS:54550-12-8 MolWeight:332 RetIndex:2170
CompName:Androstan-3-ol, 9-methyl-, acetate, (3.beta.,5.alpha.)- $$ 9-Methylandrostan-3-yl acetate # $$

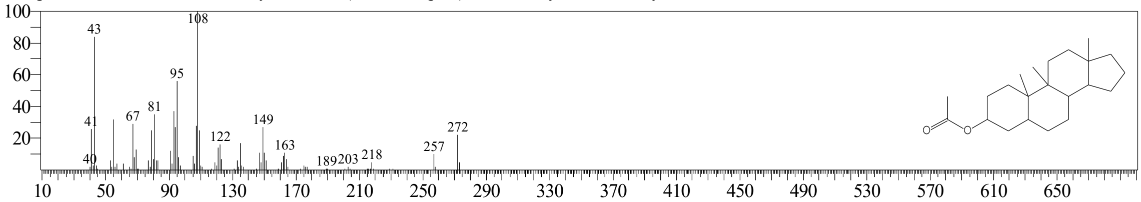

Hit#:3 Entry:38517 Library:NIST11.lib
SI:60 Formula:C11H16N2O CAS:0-00-0 MolWeight:192 RetIndex:1555
CompName:1-(5,6-Dimethyl-2-pyrazinyl)-3-methyl-1-butanone $$ 1-(3,5,6-Trimethyl-2-pyrazinyl)-1-butanone # $$

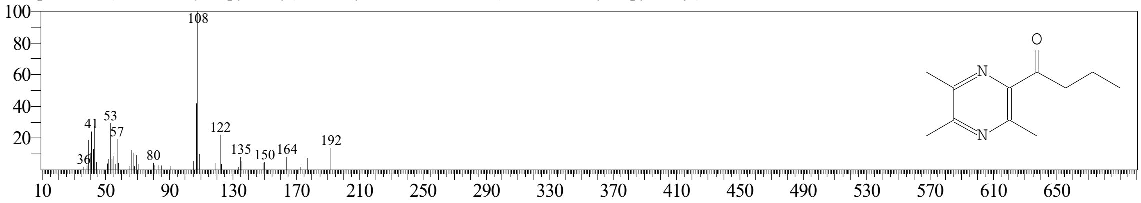

Hit#:4 Entry:22424 Library:NIST11.lib
SI:60 Formula:C11H16O CAS:0-00-0 MolWeight:164 RetIndex:1154
CompName:1,2-Epoxycyclooct-3-ene, 5,5-dimethyl-8-methylene- $$ 4,4-Dimethyl-7-methylene-9-oxabicyclo[6.1.0]non-2-ene # $$

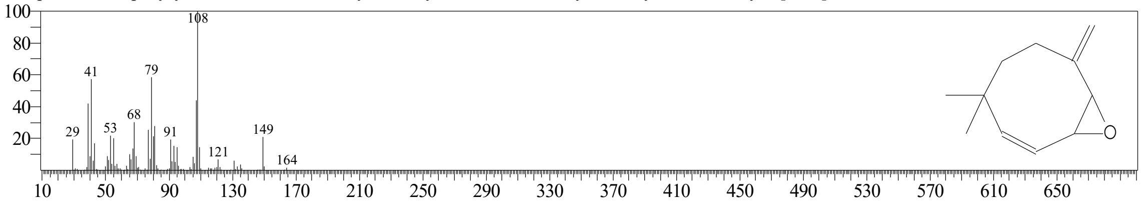

Hit#:5 Entry:30506 Library:NIST11.lib
SI:60 Formula:C13H22 CAS:0-00-0 MolWeight:178 RetIndex:1303
CompName:1,10-Dimethyl-2-methylene-trans-decalin $$ 1,8a-Dimethyl-2-methylenedecahydronaphthalene # $$

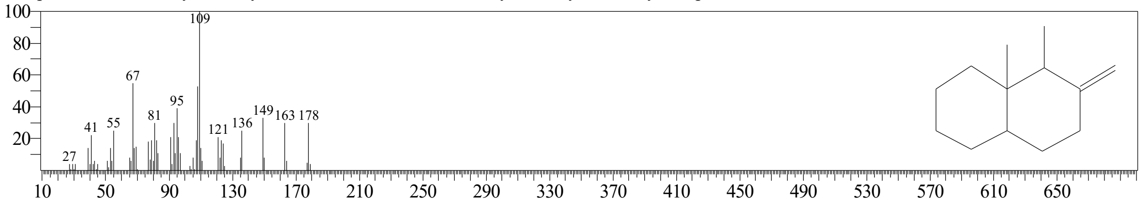

<< Target >>
Line#:66 R.Time:16.210(Scan#:2443) MassPeaks:374
RawMode:Averaged 16.205-16.215(2442-2444) BasePeak:41.05(12884)
BG Mode:Calc. from Peak Group 1 - Event 1 Scan

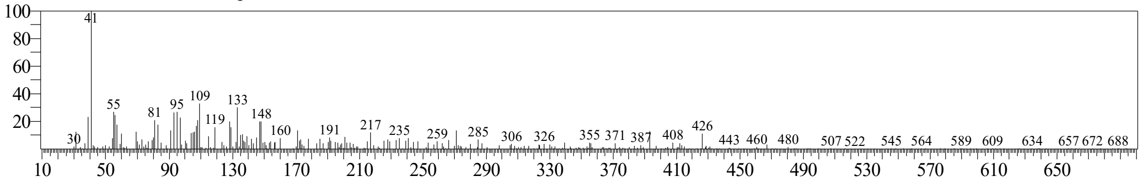

Hit#:1 Entry:208642 Library:NIST11.lib
SI:56 Formula:C40H58O CAS:105-92-0 MolWeight:554 RetIndex:4025
CompName:Rhodopin $$ .psi.,.psi.-Carotene, 1,2-dihydro-1-hydroxy- $$ Lycopene, 1,2-dihydro-1-hydroxy- $$ Lycopene, 1,2-dihydro-1-hydroxy-, all-trans- $$

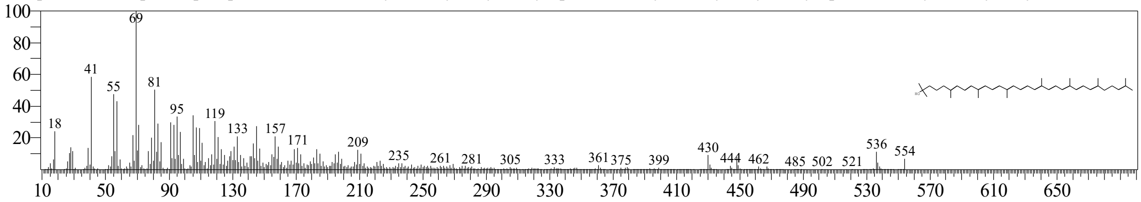

Hit#:2 Entry:96989 Library:NIST11.lib
SI:55 Formula:C20H32 CAS:77898-97-6 MolWeight:272 RetIndex:1940
CompName:(E,E,E)-3,7,11,15-Tetramethylhexadeca-1,3,6,10,14-pentaene $$ (3E,6E,10E)-3,7,11,15-Tetramethyl-1,3,6,10,14-hexadecapentaene $$ (E,E,E)- .alp

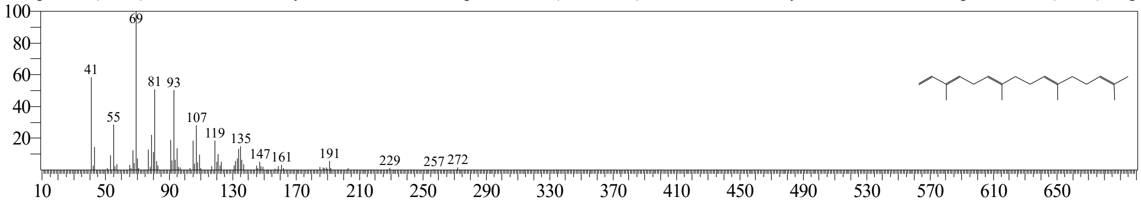

Hit#:3 Entry:59344 Library:NIST11.lib
SI:55 Formula:C15H26O CAS:16981-75-2 MolWeight:222 RetIndex:1543
CompName:Illudol $$ 3,6,6,7b-Tetramethyldecahydro-1H-cyclobuta[e]inden-3-ol # $$

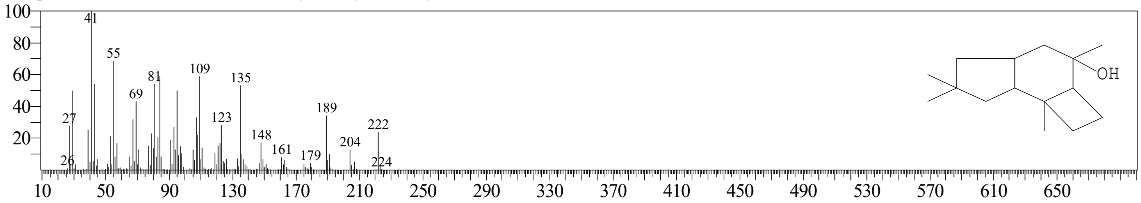

Hit#:4 Entry:57712 Library:NIST11.lib
SI:55 Formula:C15H24O CAS:0-00-0 MolWeight:220 RetIndex:1281
CompName:Isoaromadendrene epoxide $$ 1,3b,6,6-Tetramethyldecahydro-1H-cyclopropa[7,8]azuleno[4,5-b]oxirene # $$

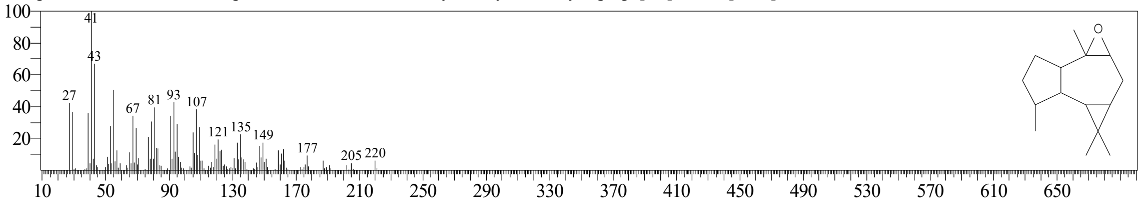

Hit#:5 Entry:46662 Library:NIST11.lib
SI:55 Formula:C15H24 CAS:0-00-0 MolWeight:204 RetIndex:1403
CompName:Tricyclo[7.2.0.0(3,8)]undec-4-ene, 4,8,11,11-tetramethyl- $$ 1,1,2b,6-Tetramethyl-2,2a,2b,3,4,6a,7,7a-octahydro-1H-cyclobuta[a]indene # $$

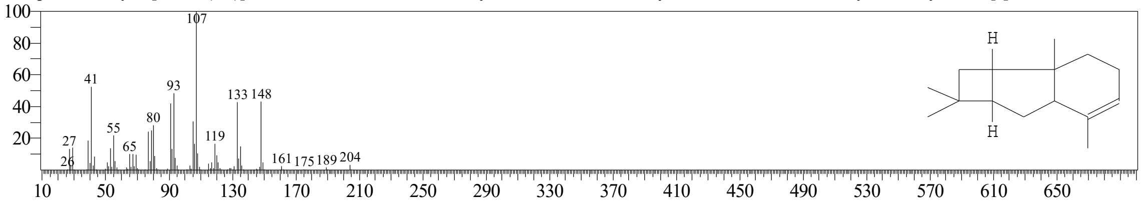

<< Target >>
Line#:67 R.Time:16.345(Scan#:2470) MassPeaks:367
RawMode:Averaged 16.340-16.350(2469-2471) BasePeak:108.00(22924)
BG Mode:Calc. from Peak Group 1 - Event 1 Scan

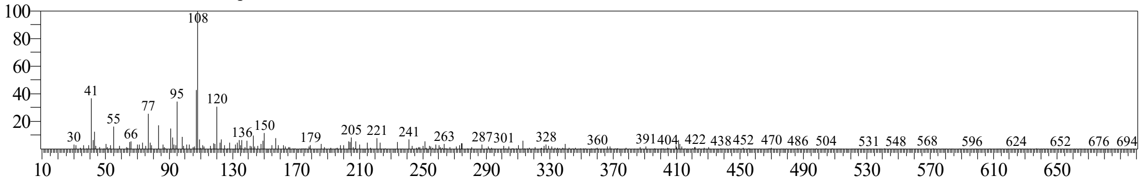

Hit#:1 Entry:83648 Library:NIST11.lib
SI:59 Formula:C11H13BrO2 CAS:0-00-0 MolWeight:256 RetIndex:1669
CompName:4-Bromobutanoic acid, 2-methylphenyl ester $$ 2-Methylphenyl 4-bromobutanoate # $$

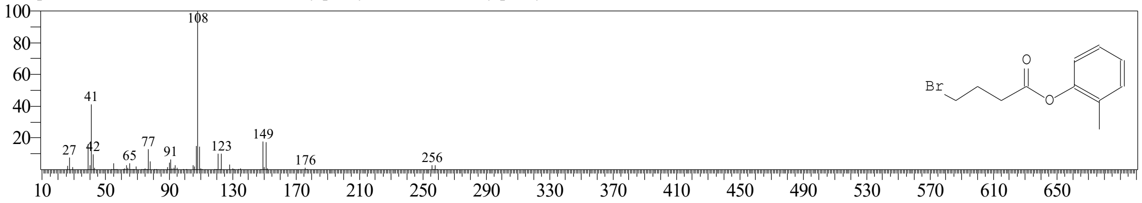

Hit#:2 Entry:118361 Library:NIST11.lib
SI:59 Formula:C16H20N4O2 CAS:0-00-0 MolWeight:300 RetIndex:2470
CompName:2-Acetyl-5,5'-dimethyl-1-phenyl-2,3,4,5,2',4'-hexahydro-1H-[3,4']bipyrazolyl-3'-one $$ 4-(2-Acetyl-5-methyl-1-phenyl-3-pyrazolidinyl)-5-methyl-2,

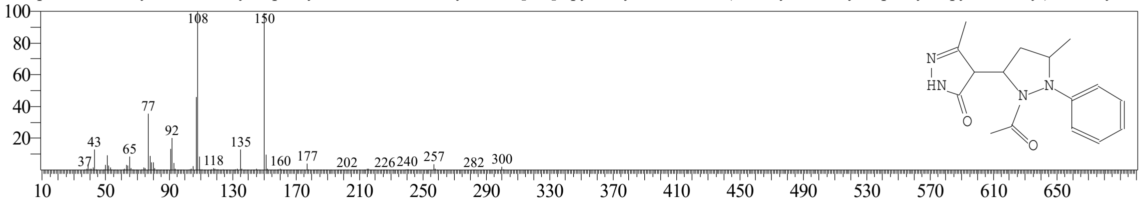

Hit#:3 Entry:15430 Library:NIST11.lib
SI:58 Formula:C10H14O CAS:24545-81-1 MolWeight:150 RetIndex:1073
CompName:Bicyclo[3.1.0]hex-3-en-2-one, 4-methyl-1-(1-methylethyl)- $$ 3-Thujen-2-one $$ Umbellulon $$ Umbellulone $$ THUJEN-2-ONE $$ 1-Isopropyl-4

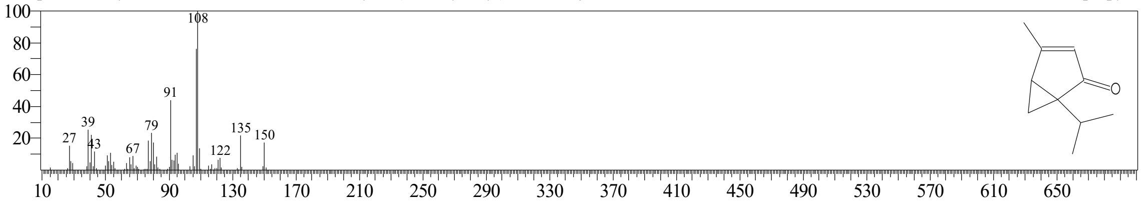

Hit#:4 Entry:61915 Library:NIST11.lib
SI:58 Formula:C12H15ClO2 CAS:0-00-0 MolWeight:226 RetIndex:1697
CompName:5-Chloropentanoic acid, 2-methylphenyl ester $$ 2-Methylphenyl 5-chloropentanoate # $$

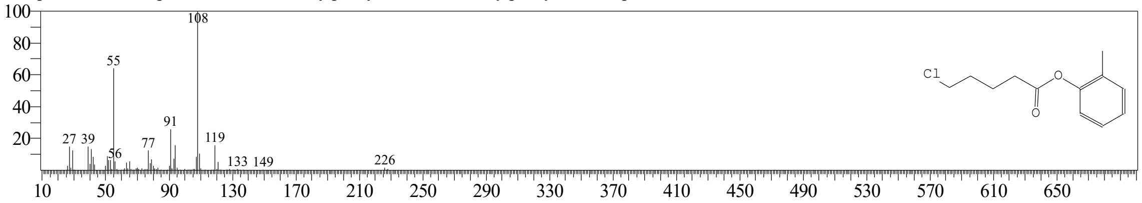

Hit#:5 Entry:123909 Library:NIST11.lib
SI:58 Formula:C17H25NO4 CAS:0-00-0 MolWeight:307 RetIndex:2321
CompName:.beta.-Alanine, N-benzyloxycarbonyl-, hexyl ester

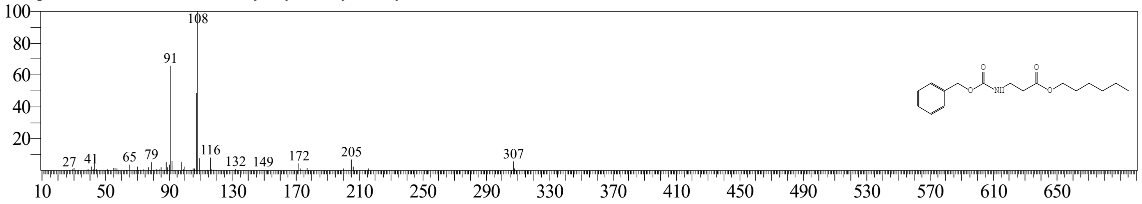

Qualitative Analysis Report

<< Target >>
Line#:68 R.Time:16.410(Scan#:2483) MassPeaks:358
RawMode:Averaged 16.405-16.415(2482-2484) BasePeak:108.05(230578)
BG Mode:Calc. from Peak Group 1 - Event 1 Scan

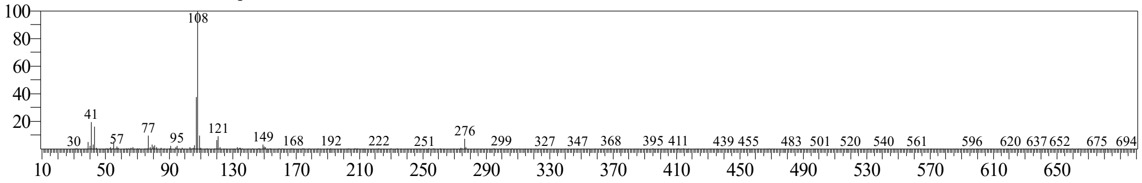

Hit#:1 Entry:15237 Library:NIST11.lib
SI:82 Formula:C9H10O2 CAS:140-39-6 MolWeight:150 RetIndex:1174
CompName:Acetic acid, 4-methylphenyl ester $$ Acetic acid, p-tolyl ester $$ p-Acetoxytoluene $$ p-Cresol acetate $$ p-Cresyl acetate $$ p-Methylphenyl aceta

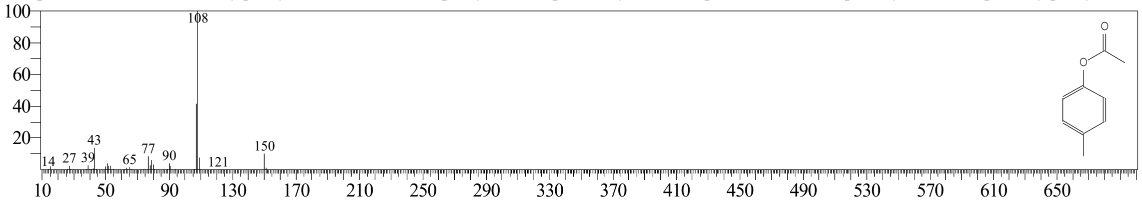

Hit#:2 Entry:30439 Library:NIST11.lib
SI:82 Formula:C12H18O CAS:85709-98-4 MolWeight:178 RetIndex:1296
CompName:Benzene, 1-(1,1-dimethylpropoxy)-4-methyl- $$ Toluene, 4-(1,1-dimethylpropoxy)- $$ 1-Methyl-4-(tert-pentyloxy)benzene # $$

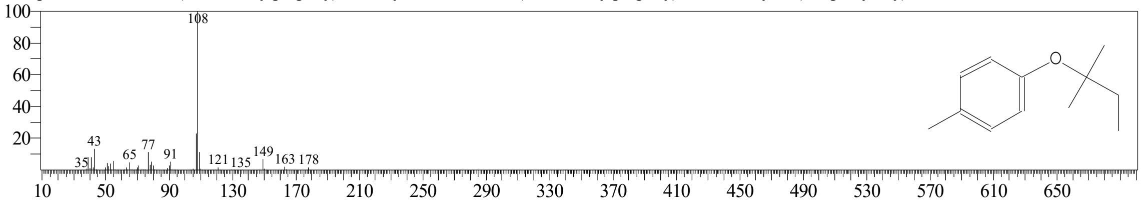

Hit#:3 Entry:15236 Library:NIST11.lib
SI:81 Formula:C9H10O2 CAS:122-46-3 MolWeight:150 RetIndex:1174
CompName:m-Cresyl acetate $$ Acetic acid, m-tolyl ester $$ m-Acetoxytoluene $$ m-Cresol acetate $$ m-Methylphenyl acetate $$ m-Tolyl acetate $$ Cresatin $

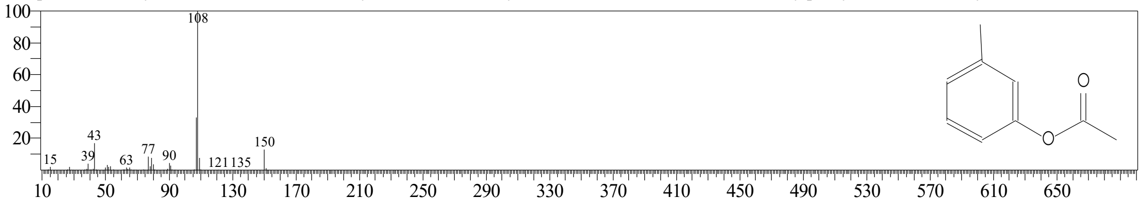

Hit#:4 Entry:22341 Library:NIST11.lib
SI:80 Formula:C10H16N2 CAS:91010-41-2 MolWeight:164 RetIndex:1228
CompName:2-Isoamyl-6-methylpyrazine $$ 2-Isopentyl-6-methylpyrazine # $$ 2-Methyl-6-isopentylpyrazine $$ 6-Methyl-2-(3-methylbutyl)-pyrazine $$ Pyrazi

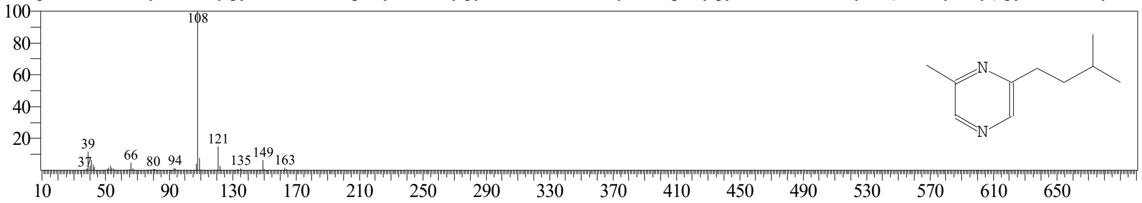

Hit#:5 Entry:39885 Library:NIST11.lib
SI:80 Formula:C11H14O3 CAS:0-00-0 MolWeight:194 RetIndex:1448
CompName:Carbonic acid, propyl 3-methylphenyl ester

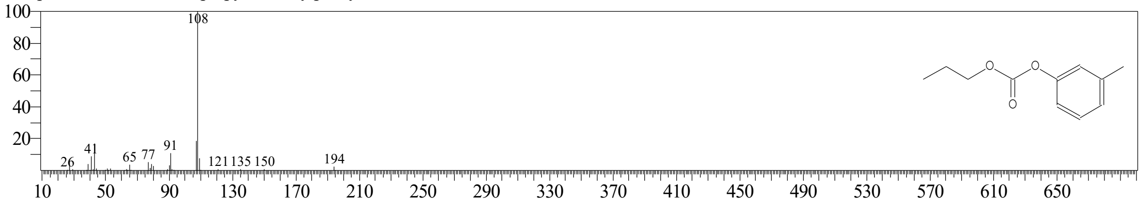

<< Target >>
Line#:69 R.Time:16.510(Scan#:2503) MassPeaks:342
RawMode:Averaged 16.505-16.515(2502-2504) BasePeak:123.10(13020)
BG Mode:Calc. from Peak Group 1 - Event 1 Scan

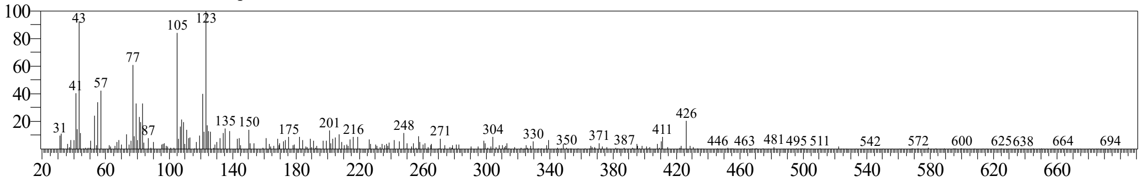

Hit#:1 Entry:31716 Library:NIST11.lib
SI:62 Formula:C12H20O CAS:211563-96-1 MolWeight:180 RetIndex:1322
CompName:Cyclohexene, 1,5,5-trimethyl-6-acetylmethyl- $$ 1-(2,6,6-Trimethyl-2-cyclohexen-1-yl)acetone # $$

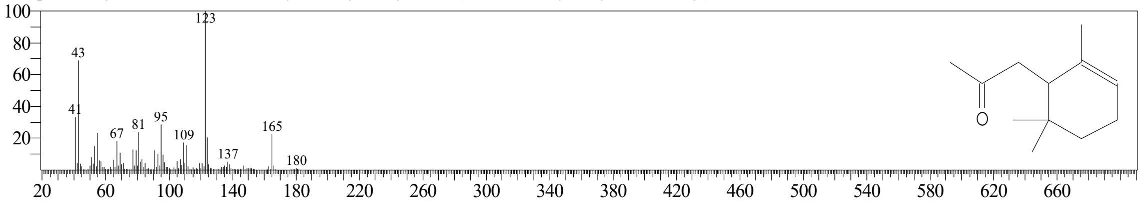

Hit#:2 Entry:68462 Library:NIST11.lib
SI:61 Formula:C15H25NO CAS:24703-24-0 MolWeight:235 RetIndex:1738
CompName:Cyclopropa[d]naphthalen-3-one, octahydro-2,4a,8,8-tetramethyl-, oxime $$ (3Z)-2,4a,8,8-Tetramethyloctahydrocyclopropa[d]naphthalen-3(1H)-one

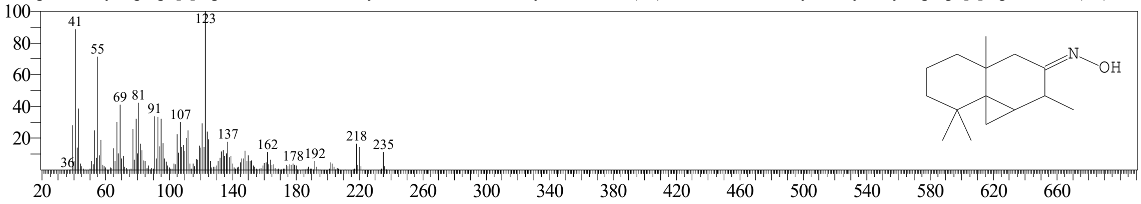

Hit#:3 Entry:57783 Library:NIST11.lib
SI:61 Formula:C15H24O CAS:0-00-0 MolWeight:220 RetIndex:1293
CompName:Tricyclo[6.3.0.0(5,7)]undecane, 1,8-epoxy-2,6,6,9-tetramethyl-

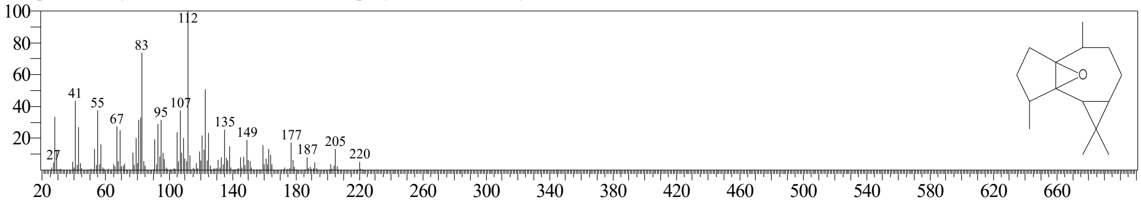

Hit#:4 Entry:59214 Library:NIST11.lib
SI:60 Formula:C14H22O2 CAS:97371-44-3 MolWeight:222 RetIndex:1518
CompName:3-Buten-2-one, 3-methyl-4-(1,3,3-trimethyl-7-oxabicyclo[4.1.0]heptan-1-yl)- $$ 3-Methyl-4-(1,3,3-trimethyl-7-oxa-bicyclo[4.1.0]hept-2-yl)-but-3-e

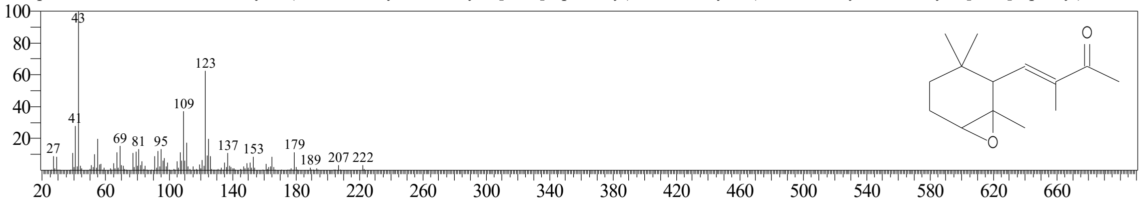

Hit#:5 Entry:191096 Library:NIST11.lib
SI:60 Formula:C30H50O CAS:55515-18-9 MolWeight:426 RetIndex:2788
CompName:Lanost-7-en-3-one, (9.beta.,13.alpha.,14.beta.,17.alpha.)- $$ Lanost-7-en-3-one # $$

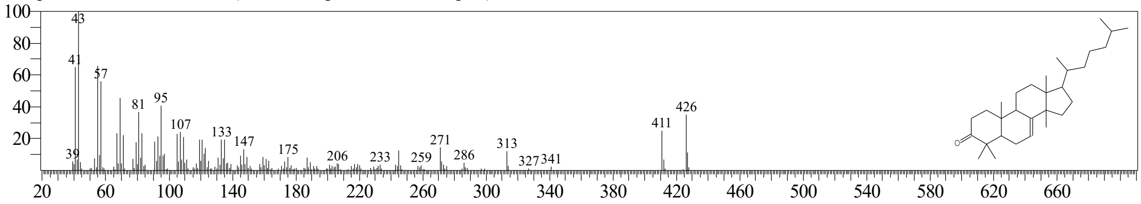

Qualitative Analysis Report

<< Target >>
Line#:70 R.Time:16.610(Scan#:2523) MassPeaks:372
RawMode:Averaged 16.605-16.615(2522-2524) BasePeak:41.05(17277)
BG Mode:Calc. from Peak Group 1 - Event 1 Scan

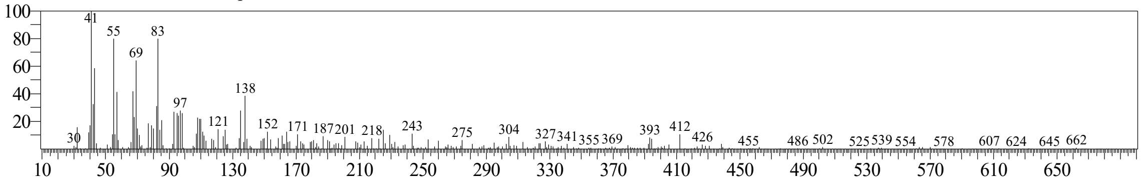

Hit#:1 Entry:147505 Library:NIST11.lib
SI:79 Formula:C22H42O2 CAS:506-33-2 MolWeight:338 RetIndex:2572
CompName:(E)-13-Docosenoic acid $$ 13-Docosenoic acid, (E)- $$ trans-13-Docosenoic acid $$ Brassidic acid $$ (13E)-13-Docosenoic acid # $$

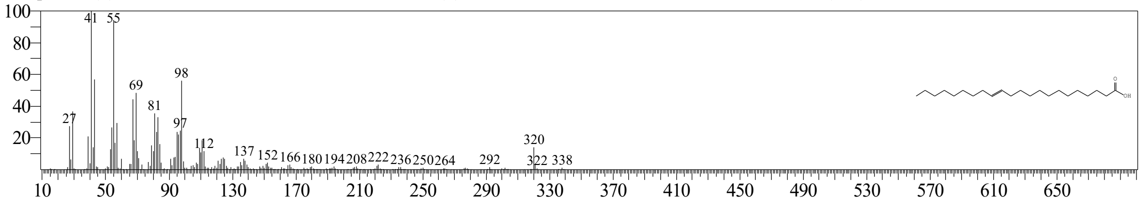

Hit#:2 Entry:91949 Library:NIST11.lib
SI:79 Formula:C18H34O CAS:2423-10-1 MolWeight:266 RetIndex:2007
CompName:9-Octadecenal, (Z)- $$ Olealdehyde $$ cis-9-Octadecenal $$ Oleylaldehyde $$ Z-9-Octadecenal $$ (9Z)-9-Octadecenal # $$

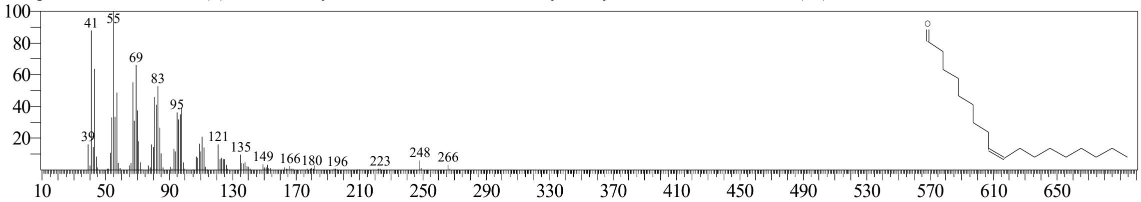

Hit#:3 Entry:70898 Library:NIST11.lib
SI:78 Formula:C16H30O CAS:56219-04-6 MolWeight:238 RetIndex:1808
CompName:cis-9-Hexadecenal $$ 9-Hexadecenal, (Z)- $$ (Z)-9-Hexadecenal $$ Z-9-Hexadecenal $$ (9Z)-9-Hexadecenal # $$

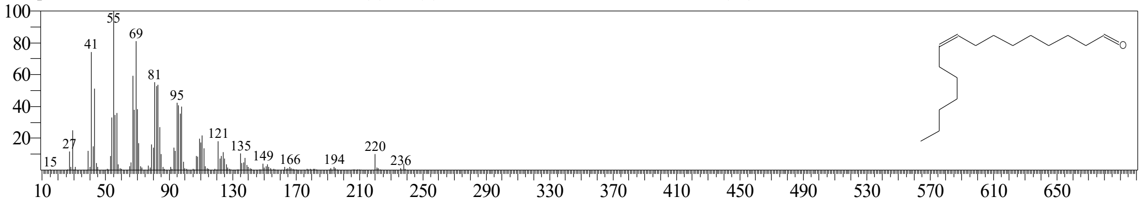

Hit#:4 Entry:70892 Library:NIST11.lib
SI:78 Formula:C16H30O CAS:56797-40-1 MolWeight:238 RetIndex:1808
CompName:7-Hexadecenal, (Z)- $$ (Z)-7-Hexadecenal $$ Z-7-Hexadecenal $$ (7Z)-7-Hexadecenal # $$

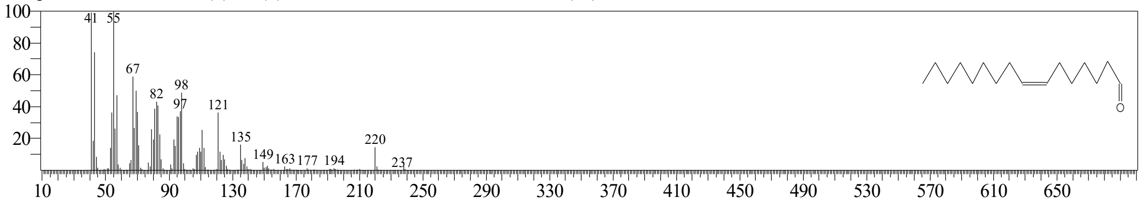

Hit#:5 Entry:50988 Library:NIST11.lib
SI:78 Formula:C14H26O CAS:65128-96-3 MolWeight:210 RetIndex:1609
CompName:7-Tetradecenal, (Z)- $$ Z-7-Tetradecenal $$ (7Z)-7-Tetradecenal # $$

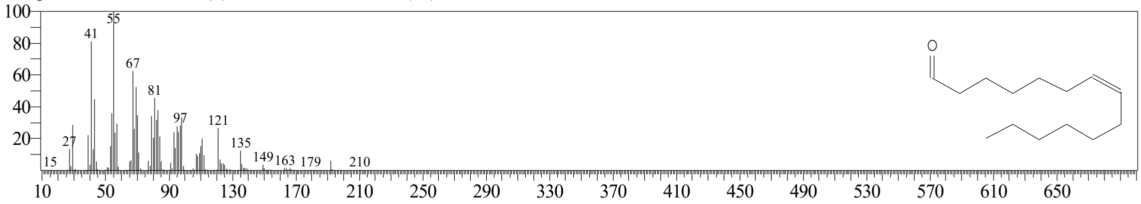

<< Target >>
Line#:71 R.Time:16.815(Scan#:2564) MassPeaks:362
RawMode:Averaged 16.810-16.820(2563-2565) BasePeak:83.05(19355)
BG Mode:Calc. from Peak Group 1 - Event 1 Scan

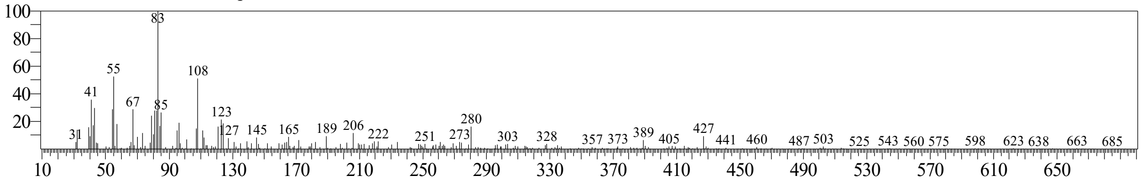

Hit#:1 Entry:24571 Library:NIST11.lib
SI:66 Formula:C10H16O2 CAS:80648-29-9 MolWeight:168 RetIndex:1388
CompName:6-Methyloctahydrocoumarin $$ 6-Methyloctahydro-2H-chromen-2-one # $$ 2H-1-Benzopyran-2-one, octahydro-6-methyl- $$ Coumarin, octahydro

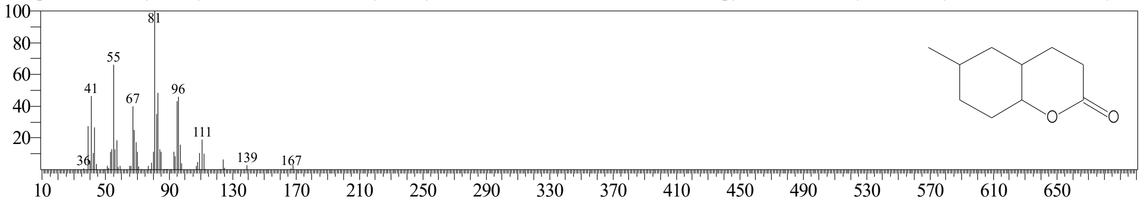

Hit#:2 Entry:32960 Library:NIST11.lib
SI:66 Formula:C12H22O CAS:0-00-0 MolWeight:182 RetIndex:1377
CompName:Cyclooctene, 3-butoxy- $$ 3-Butoxy-1-cyclooctene # $$

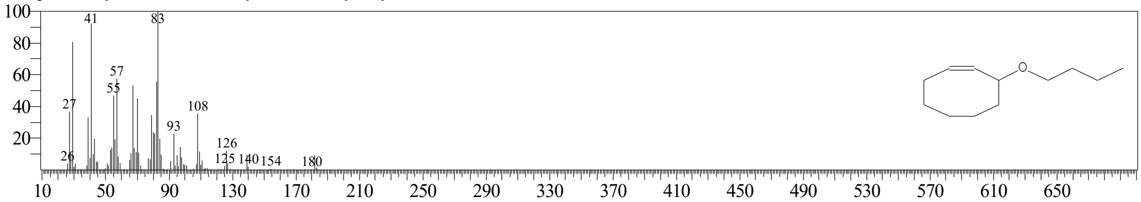

Hit#:3 Entry:22338 Library:NIST11.lib
SI:66 Formula:C10H16N2 CAS:1871-96-1 MolWeight:164 RetIndex:1506
CompName:Decanedinitrile $$ Sebaconitrile $$ Octane-1,8-dicarbonitrile $$ Sebacic acid dinitrile $$ 1,8-Dicyanooctane $$ Octamethylene dicyanide $$ Oktan

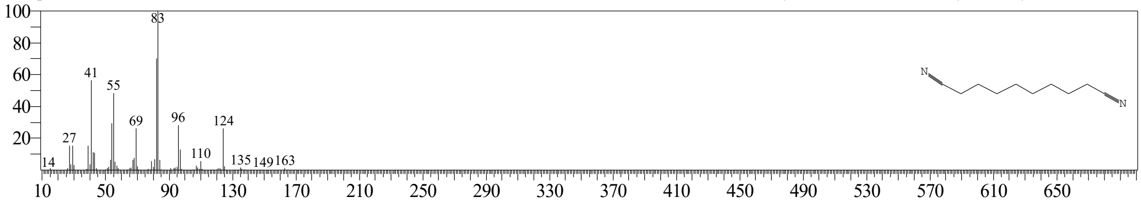

Hit#:4 Entry:59500 Library:NIST11.lib
SI:65 Formula:C16H30 CAS:55012-77-6 MolWeight:222 RetIndex:1636
CompName:1,1'-Bicyclohexyl, 2-(2-methylpropyl)-, trans-

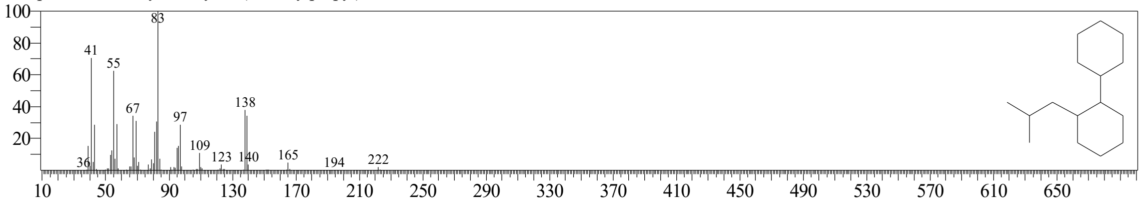

Hit#:5 Entry:49650 Library:NIST11.lib
SI:65 Formula:C15H28 CAS:50991-16-7 MolWeight:208 RetIndex:1537
CompName:1,1'-Bicyclohexyl, 2-(1-methylethyl)-, trans- $$ Bicyclohexyl, 2-isopropyl-, trans- $$

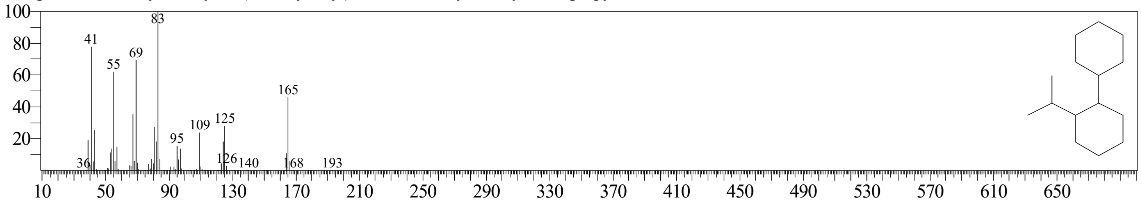

<< Target >>
Line#:72 R.Time:16.900(Scan#:2581) MassPeaks:410
RawMode:Averaged 16.895-16.905(2580-2582) BasePeak:79.05(65232)
BG Mode:Calc. from Peak Group 1 - Event 1 Scan

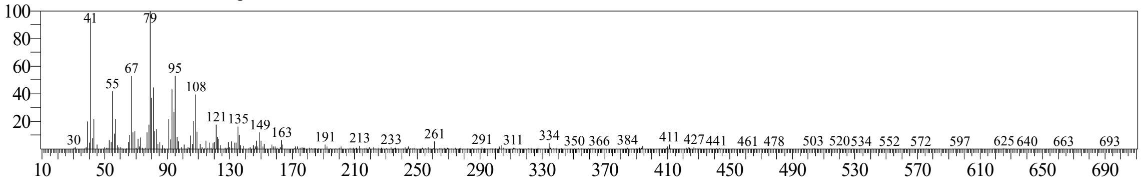

Hit#:1 Entry:132823 Library:NIST11.lib
SI:89 Formula:C21H34O2 CAS:59149-01-8 MolWeight:318 RetIndex:2308
CompName:Methyl (Z)-5,11,14,17-eicosatetraenoate $$ Methyl (5Z,11Z,14Z,17Z)-5,11,14,17-icosatetraenoate # $$

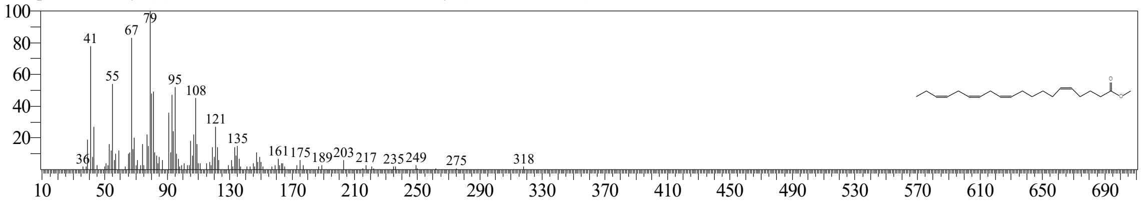

Hit#:2 Entry:101390 Library:NIST11.lib
SI:89 Formula:C18H30O2 CAS:463-40-1 MolWeight:278 RetIndex:2191
CompName:9,12,15-Octadecatrienoic acid, (Z,Z,Z)- $$ Linolenic acid $$ .alpha.-Linolenic acid $$ All-cis-9,12,15-Octadecatrienoic acid $$ cis,cis,cis-9,12,15-(

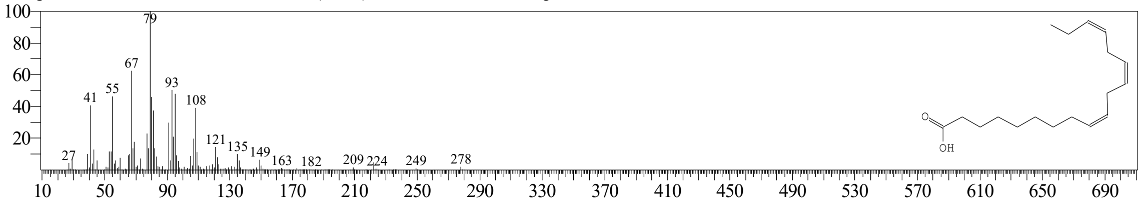

Hit#:3 Entry:90503 Library:NIST11.lib
SI:88 Formula:C18H32O CAS:506-44-5 MolWeight:264 RetIndex:2077
CompName:9,12,15-Octadecatrien-1-ol, (Z,Z,Z)- $$ (9E,12E,15E)-9,12,15-Octadecatrien-1-ol # $$ (Z,Z,Z)-9,12,15-Octadecatrien-1-ol $$

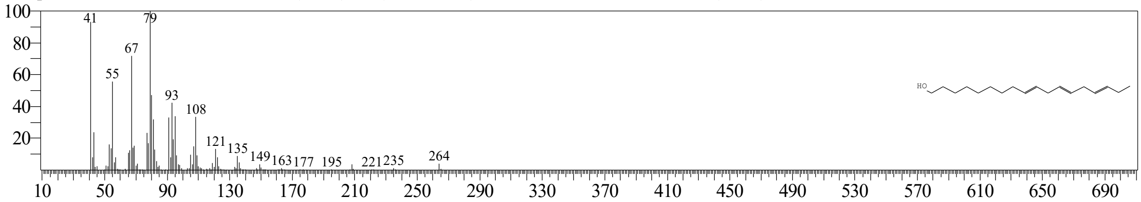

Hit#:4 Entry:67981 Library:NIST11.lib
SI:87 Formula:C17H30 CAS:74744-55-1 MolWeight:234 RetIndex:1736
CompName:3-Heptadecen-5-yne, (Z)- $$ (3Z)-3-Heptadecen-5-yne # $$

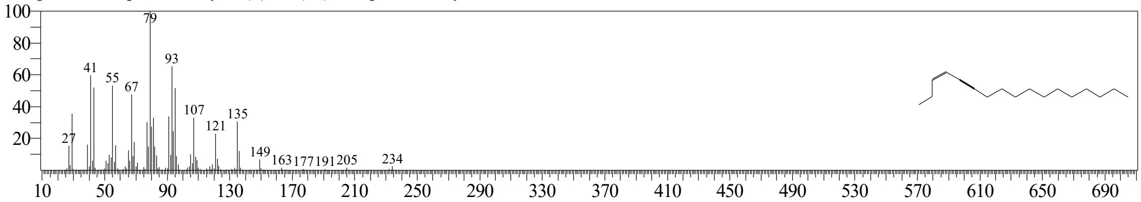

Hit#:5 Entry:112450 Library:NIST11.lib
SI:87 Formula:C19H32O2 CAS:301-00-8 MolWeight:292 RetIndex:2101
CompName:9,12,15-Octadecatrienoic acid, methyl ester, (Z,Z,Z)- $$ Linolenic acid, methyl ester $$ Methyl all-cis-9,12,15-octadecatrienoate $$ Methyl linolena

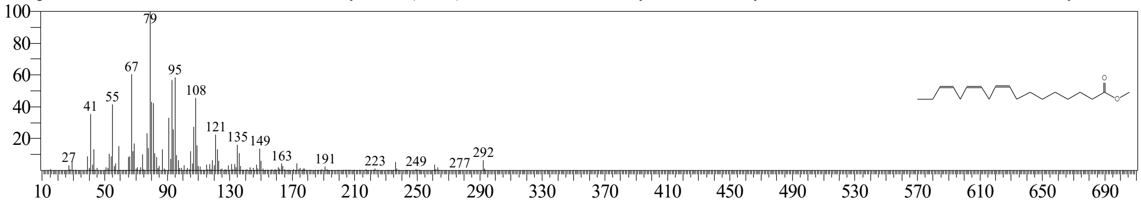

<< Target >>
Line#:73 R.Time:17.110(Scan#:2623) MassPeaks:416
RawMode:Averaged 17.105-17.115(2622-2624) BasePeak:56.05(15861)
BG Mode:Calc. from Peak Group 1 - Event 1 Scan

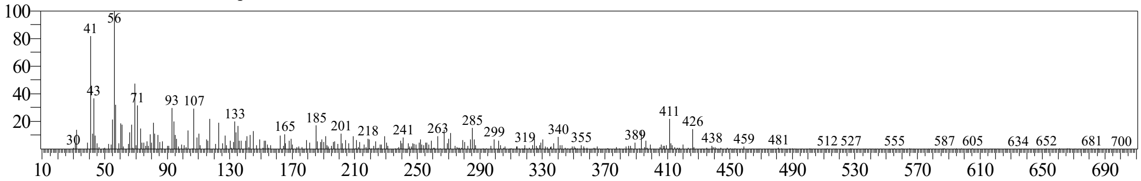

Hit#:1 Entry:149085 Library:NIST11.lib
SI:59 Formula:C22H44O2 CAS:646-13-9 MolWeight:340 RetIndex:2311
CompName:Octadecanoic acid, 2-methylpropyl ester $$ Stearic acid, isobutyl ester $$ Isobutyl stearate $$ Emerest 2324 $$ Estol 1476 $$ Kemester 5415 $$ Ke

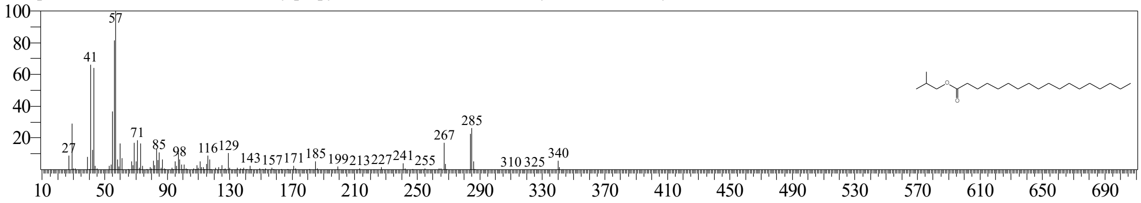

Hit#:2 Entry:106162 Library:NIST11.lib
SI:59 Formula:C18H36O2 CAS:110-36-1 MolWeight:284 RetIndex:1978
CompName:Butyl myristate $$ n-Butyl myristate $$ Myristic acid n-butyl ester $$ Tetradecanoic acid, butyl ester $$ Bumyr $$ Myristic acid, butyl ester $$ Buty

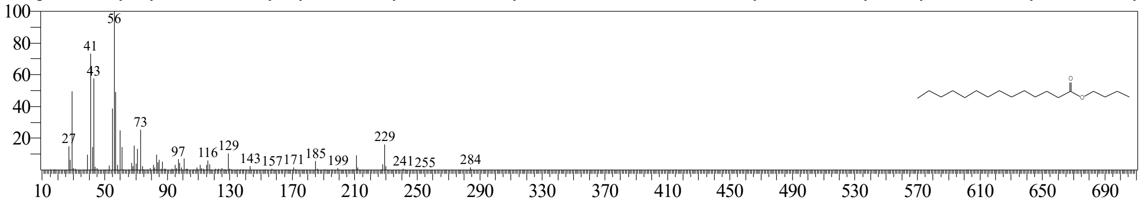

Hit#:3 Entry:191096 Library:NIST11.lib
SI:59 Formula:C30H50O CAS:55515-18-9 MolWeight:426 RetIndex:2788
CompName:Lanost-7-en-3-one, (9.beta.,13.alpha.,14.beta.,17.alpha.)- $$ Lanost-7-en-3-one # $$

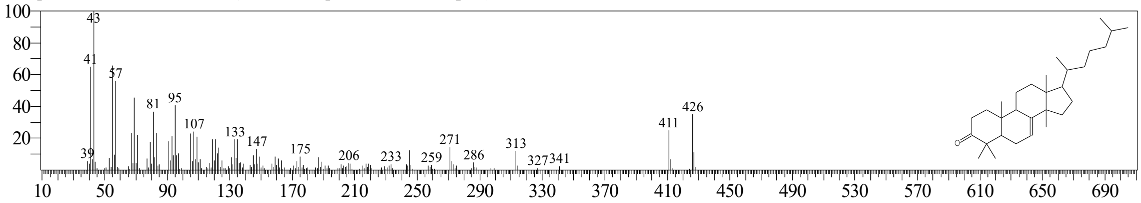

Hit#:4 Entry:149079 Library:NIST11.lib
SI:58 Formula:C22H44O2 CAS:123-95-5 MolWeight:340 RetIndex:2375
CompName:Octadecanoic acid, butyl ester $$ Stearic acid, butyl ester $$ n-Butyl octadecanoate $$ n-Butyl stearate $$ Apex 4 $$ Butyl octadecanoate $$ Butyl s

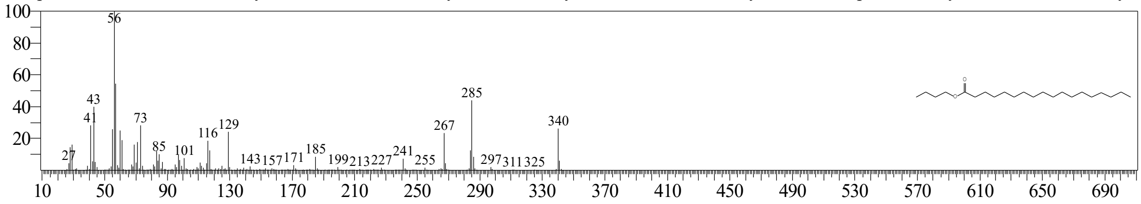

Hit#:5 Entry:62263 Library:NIST11.lib
SI:58 Formula:C14H26O2 CAS:0-00-0 MolWeight:226 RetIndex:1570
CompName:Butyl 9-decenoate

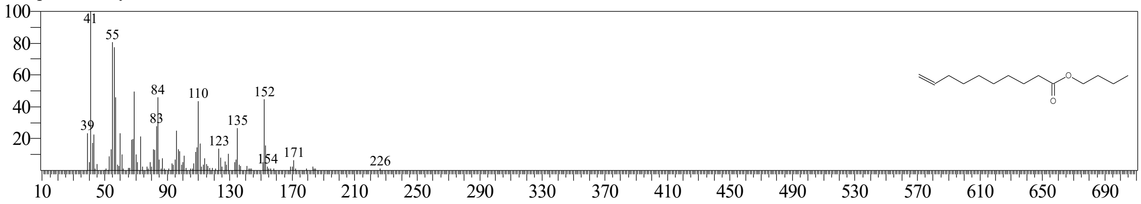

<< Target >>
Line#:74 R.Time:17.185(Scan#:2638) MassPeaks:365
RawMode:Averaged 17.180-17.190(2637-2639) BasePeak:43.10(33341)
BG Mode:Calc. from Peak Group 1 - Event 1 Scan

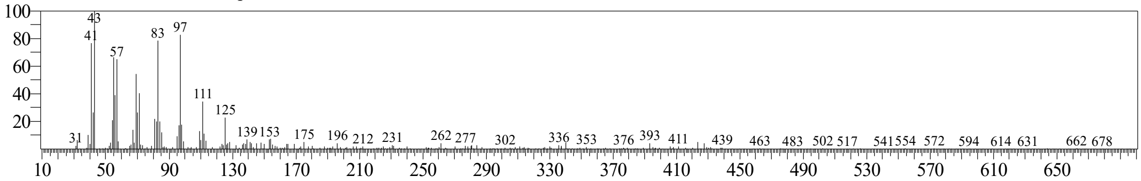

Hit#:1 Entry:114020 Library:NIST11.lib
SI:89 Formula:C21H42 CAS:95008-11-0 MolWeight:294 RetIndex:2117
CompName:10-Heneicosene (c,t) $$ (10E)-10-Henicosene # $$

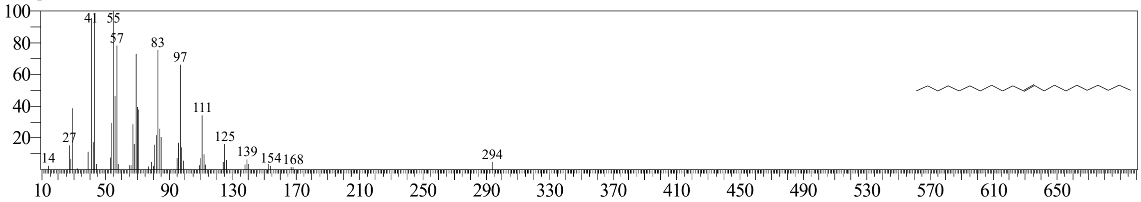

Hit#:2 Entry:164490 Library:NIST11.lib
SI:89 Formula:C26H52 CAS:18835-33-1 MolWeight:364 RetIndex:2596
CompName:1-Hexacosene

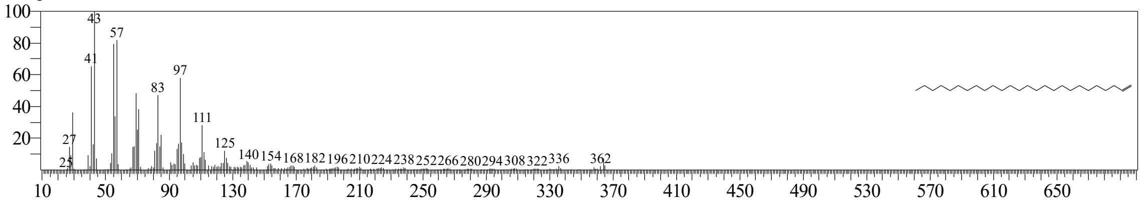

Hit#:3 Entry:135759 Library:NIST11.lib
SI:89 Formula:C23H46 CAS:18835-32-0 MolWeight:322 RetIndex:2298
CompName:1-Tricosene $$ Tricosene-1 $$

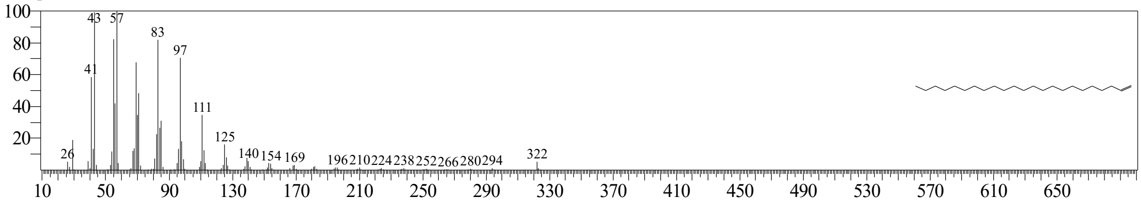

Hit#:4 Entry:128253 Library:NIST11.lib
SI:89 Formula:C21H44O CAS:15594-90-8 MolWeight:312 RetIndex:2351
CompName:1-Heneicosanol $$ Henicosan-1-ol $$ Heneicosyl alcohol $$

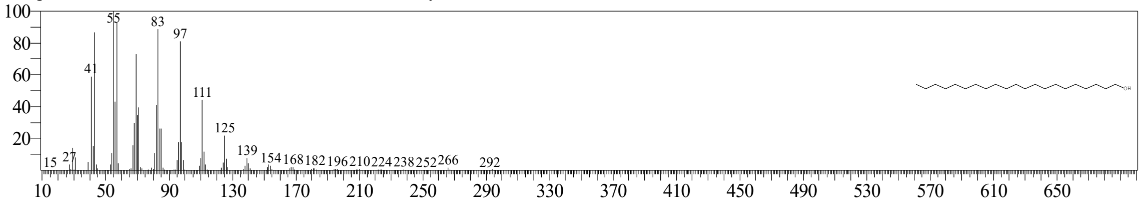

Hit#:5 Entry:70935 Library:NIST11.lib
SI:88 Formula:C17H34 CAS:6765-39-5 MolWeight:238 RetIndex:1701
CompName:1-Heptadecene $$ Hexahydroaplotaxene $$ Heptadec-1-ene $$ NSC 77132 $$ Heptadecene (Related) $$

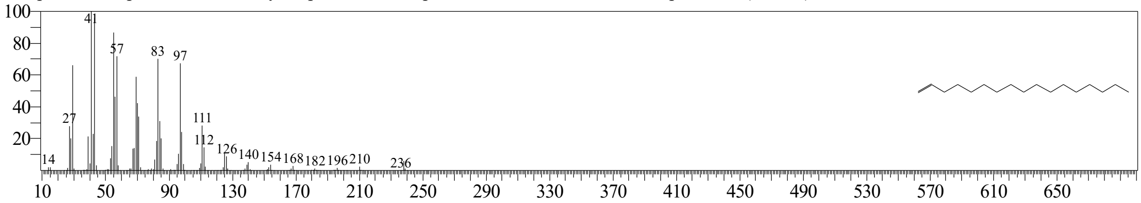

<< Target >>
Line#:75 R.Time:17.375(Scan#:2676) MassPeaks:372
RawMode:Averaged 17.370-17.380(2675-2677) BasePeak:108.00(55219)
BG Mode:Calc. from Peak Group 1 - Event 1 Scan

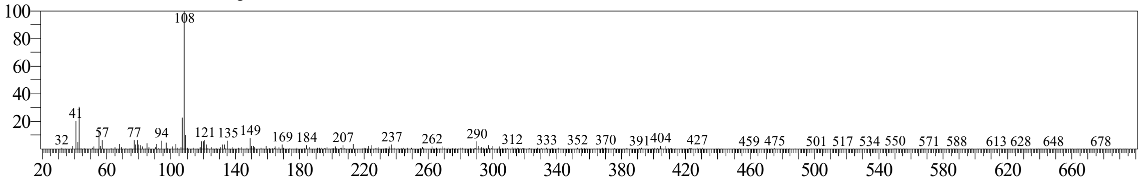

Hit#:1 Entry:90155 Library:NIST11.lib
SI:73 Formula:C15H20O4 CAS:0-00-0 MolWeight:264 RetIndex:1937
CompName:Oxalic acid, hexyl 2-methylphenyl ester

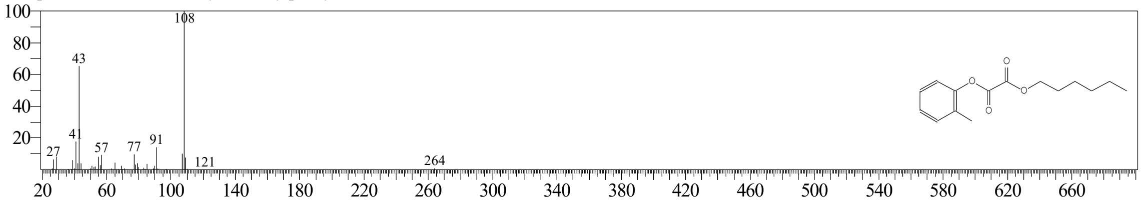

Hit#:2 Entry:79486 Library:NIST11.lib
SI:73 Formula:C14H18O4 CAS:0-00-0 MolWeight:250 RetIndex:1838
CompName:Oxalic acid, 2-methylphenyl pentyl ester

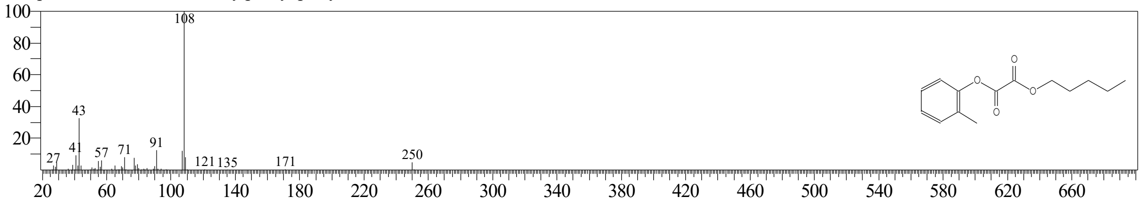

Hit#:3 Entry:58886 Library:NIST11.lib
SI:72 Formula:C12H14O4 CAS:0-00-0 MolWeight:222 RetIndex:1639
CompName:Oxalic acid, 2-methylphenyl propyl ester

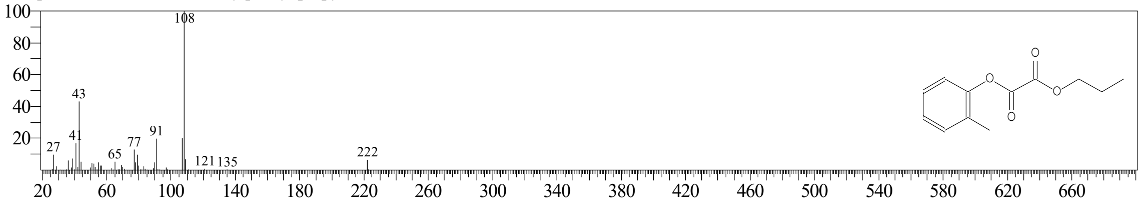

Hit#:4 Entry:123190 Library:NIST11.lib
SI:72 Formula:C18H26O4 CAS:0-00-0 MolWeight:306 RetIndex:2235
CompName:Oxalic acid, 2-methylphenyl nonyl ester

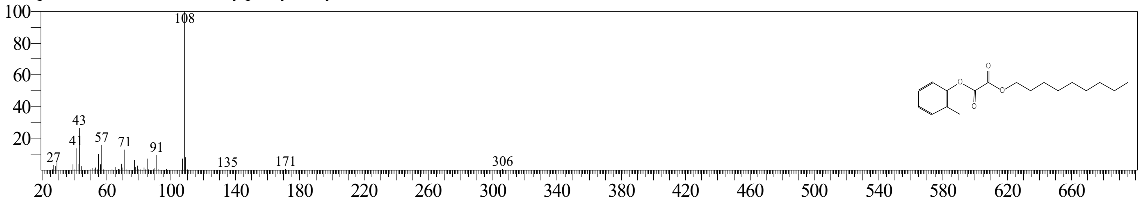

Hit#:5 Entry:154416 Library:NIST11.lib
SI:72 Formula:C21H32O4 CAS:0-00-0 MolWeight:348 RetIndex:2534
CompName:Oxalic acid, dodecyl 2-methylphenyl ester

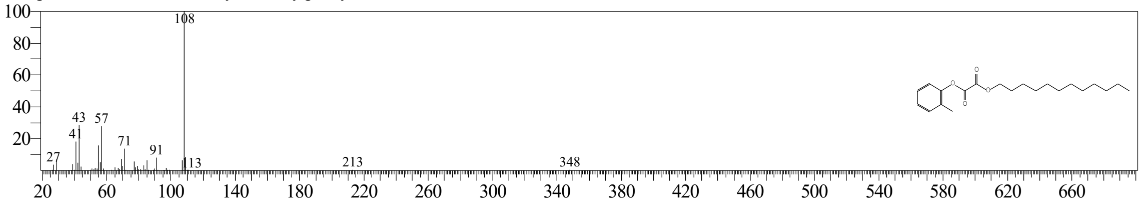

<< Target >>
Line#:76 R.Time:17.740(Scan#:2749) MassPeaks:348
RawMode:Averaged 17.735-17.745(2748-2750) BasePeak:43.05(10058)
BG Mode:Calc. from Peak Group 1 - Event 1 Scan

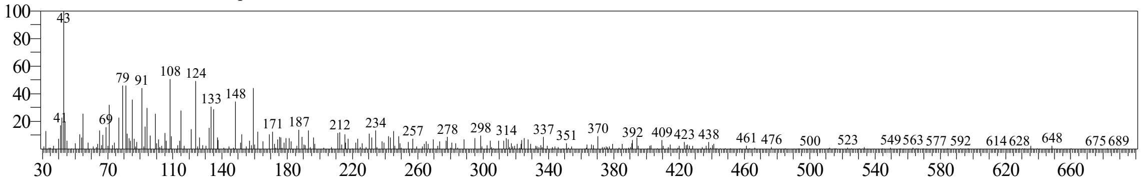

Hit#:1 Entry:23580 Library:NIST11.lib
SI:62 Formula:C11H18O CAS:56362-33-5 MolWeight:166 RetIndex:1278
CompName:Ethanone, 1-(octahydro-1H-inden-1-yl)-, (1.alpha.,3a.beta.,7a.alpha.)- $$ 1-Octahydro-1H-inden-1-ylethanone, (1.alpha.,3a.beta.,7a.alpha.)- $$

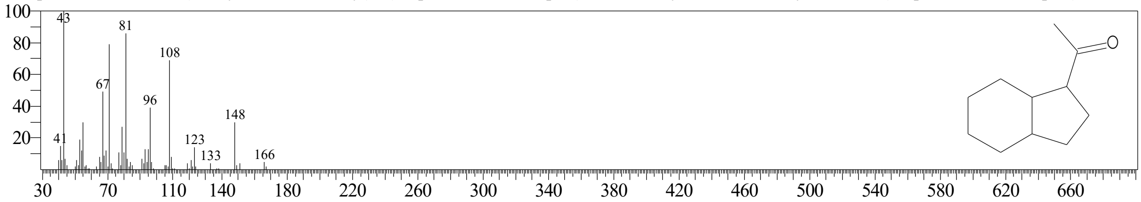

Hit#:2 Entry:40039 Library:NIST11.lib
SI:61 Formula:C12H18O2 CAS:77670-93-0 MolWeight:194 RetIndex:1504
CompName:Epianastrephin, (-) $$ 4,7a-Dimethyl-4-vinylhexahydro-1-benzofuran-2(3H)-one # $$

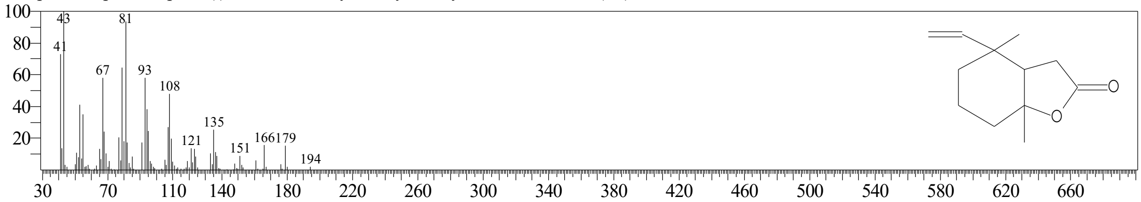

Hit#:3 Entry:23579 Library:NIST11.lib
SI:60 Formula:C11H18O CAS:56362-32-4 MolWeight:166 RetIndex:1278
CompName:Ethanone, 1-(octahydro-1H-inden-1-yl)-, (1.alpha.,3a.alpha.,7a.beta.)- $$ 1-Octahydro-1H-inden-1-ylethanone, (1.alpha.,3a.alpha.,7a.beta.)- $$

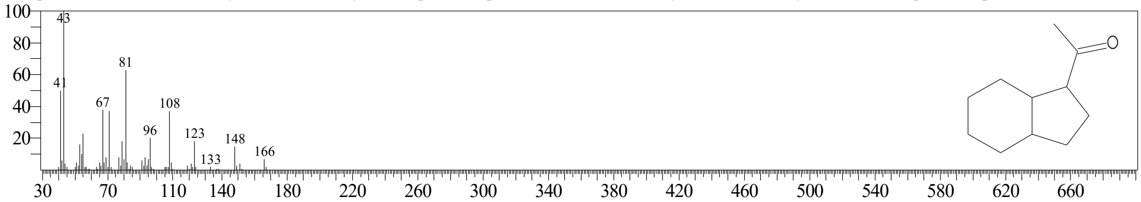

Hit#:4 Entry:184207 Library:NIST11.lib
SI:59 Formula:C27H48O2 CAS:0-00-0 MolWeight:404 RetIndex:2774
CompName:Cholestan-3.beta.,16.alpha.-diol $$ Cholestane-3,16-diol # $$

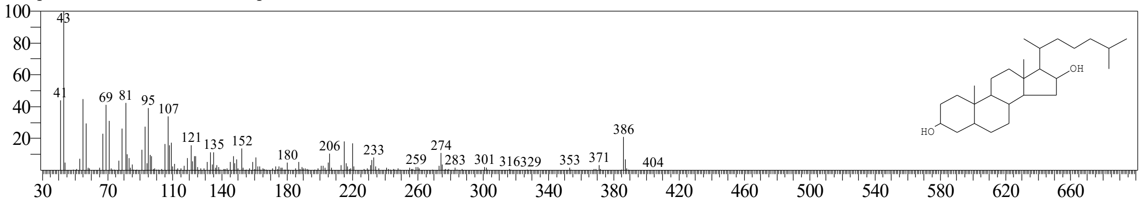

Hit#:5 Entry:131293 Library:NIST11.lib
SI:58 Formula:C21H32O2 CAS:6022-48-6 MolWeight:316 RetIndex:2237
CompName:5.alpha.-Pregnane-12,20-dione $$ Pregnane-12,20-dione # $$

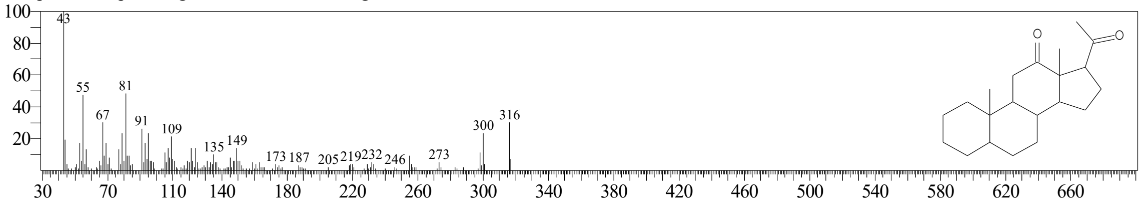

<< Target >>
Line#:77 R.Time:17.935(Scan#:2788) MassPeaks:356
RawMode:Averaged 17.930-17.940(2787-2789) BasePeak:108.00(18390)
BG Mode:Calc. from Peak Group 1 - Event 1 Scan

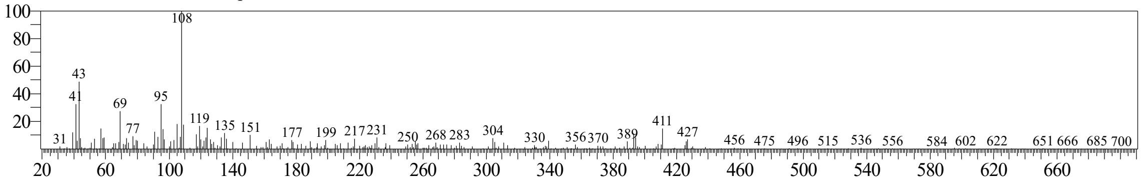

Hit#:1 Entry:40107 Library:NIST11.lib
SI:62 Formula:C12H18O2 CAS:55627-02-6 MolWeight:194 RetIndex:1271
CompName:Bicyclo[2.2.1]heptane-3-methylene-2,2-dimethyl-5-ol acetate $$ 5,5-Dimethyl-6-methylenebicyclo[2.2.1]hept-2-yl acetate # $$

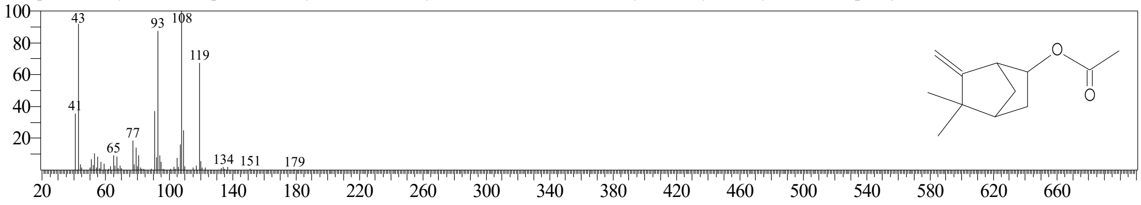

Hit#:2 Entry:89013 Library:NIST11.lib
SI:60 Formula:C18H30O CAS:0-00-0 MolWeight:262 RetIndex:1825
CompName:Sclareoloxide(Cis-B/C) $$ 3,4a,7,7,10a-Pentamethyl-4a,5,6,6a,7,8,9,10,10a,10b-decahydro-1H-benzo[f]chromene # $$

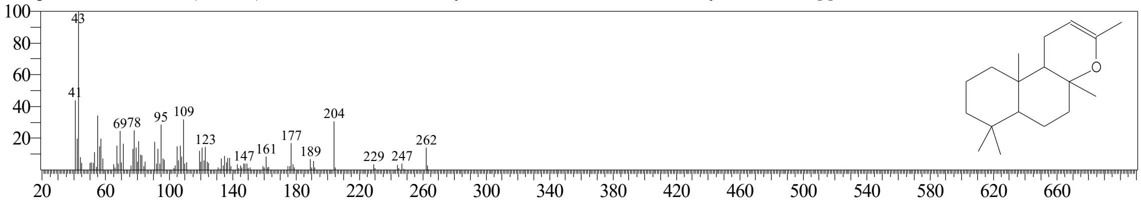

Hit#:3 Entry:49452 Library:NIST11.lib
SI:60 Formula:C13H20O2 CAS:52210-15-8 MolWeight:208 RetIndex:1627
CompName:2-Cyclohexen-1-one, 4-(3-hydroxy-1-butenyl)-3,5,5-trimethyl-, [R-[R*,R*-(E)]]- $$ 2-Cyclohexen-1-one, 4-[(1E,3R)-3-hydroxy-1-buten-1-yl]-3,5,

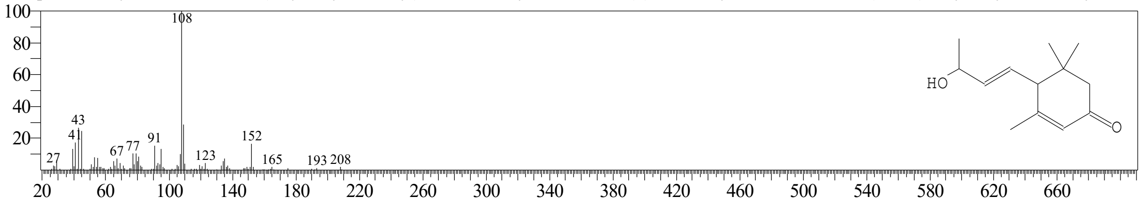

Hit#:4 Entry:16471 Library:NIST11.lib
SI:59 Formula:C10H16O CAS:0-00-0 MolWeight:152 RetIndex:1120
CompName:1,7,7-Trimethylbicyclo[2.2.1]hept-5-en-2-ol

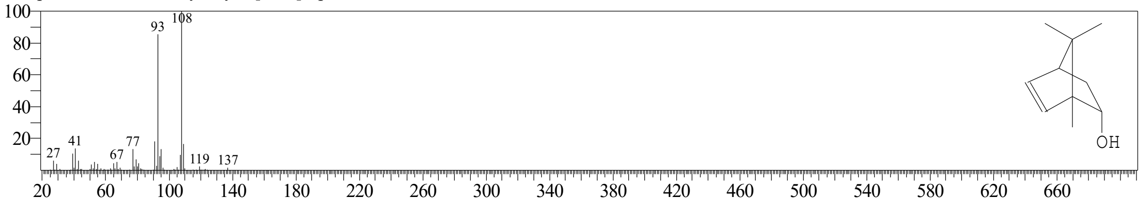

Hit#:5 Entry:143347 Library:NIST11.lib
SI:59 Formula:C22H36O2 CAS:54550-12-8 MolWeight:332 RetIndex:2170
CompName:Androstan-3-ol, 9-methyl-, acetate, (3.beta.,5.alpha.)- $$ 9-Methylandrostan-3-yl acetate # $$

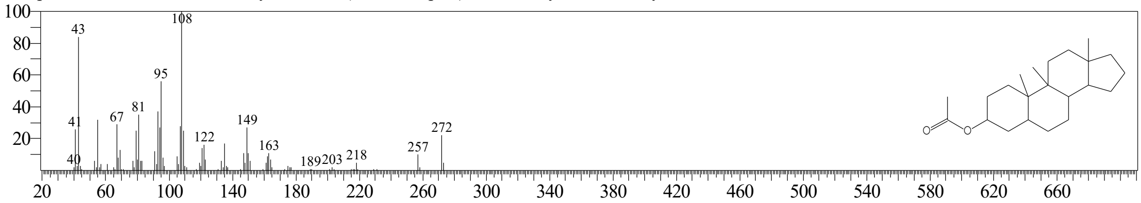

<< Target >>
Line#:78 R.Time:18.045(Scan#:2810) MassPeaks:365
RawMode:Averaged 18.040-18.050(2809-2811) BasePeak:108.05(10334)
BG Mode:Calc. from Peak Group 1 - Event 1 Scan

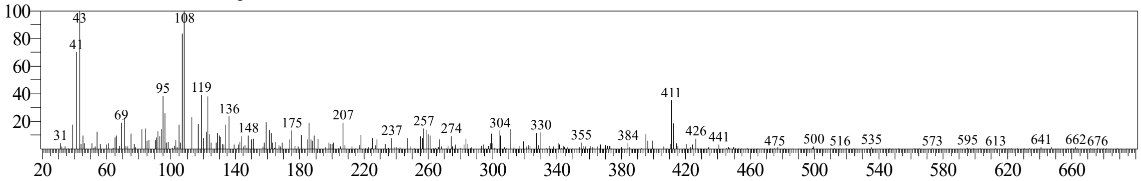

Hit#:1 Entry:31701 Library:NIST11.lib
SI:52 Formula:C12H20O CAS:130876-99-2 MolWeight:180 RetIndex:1320
CompName:5,8-Decadien-2-one, 5,9-dimethyl-, (E)- $$ (5E)-5,9-Dimethyl-5,8-decadien-2-one # $$

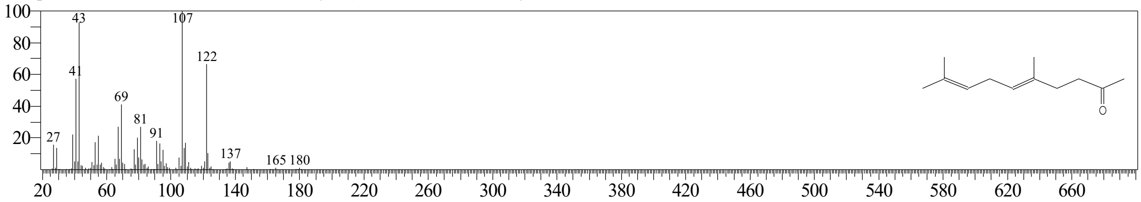

Hit#:2 Entry:59367 Library:NIST11.lib
SI:52 Formula:C15H26O CAS:489-41-8 MolWeight:222 RetIndex:1530
CompName:(-)-Globulol $$ 1H-Cycloprop[e]azulen-4-ol, decahydro-1,1,4,7-tetramethyl-, [1aR-(1a.alpha.,4.alpha.,4a.alpha.,7.alpha.,7a.beta.,7b.alpha.)]- $$ 1,1

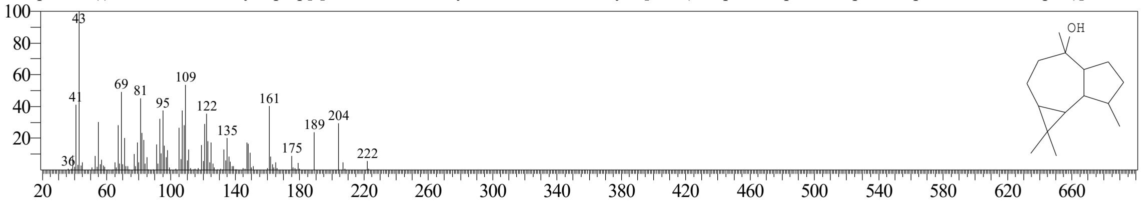

Hit#:3 Entry:59440 Library:NIST11.lib
SI:51 Formula:C15H26O CAS:19435-97-3 MolWeight:222 RetIndex:1580
CompName:1-Naphthalenol, 1,2,3,4,4a,7,8,8a-octahydro-1,6-dimethyl-4-(1-methylethyl)-, [1R-(1.alpha.,4.beta.,4a.beta.,8a.beta.)]- $$ 1.beta.-Cadin-4-en-10-ol

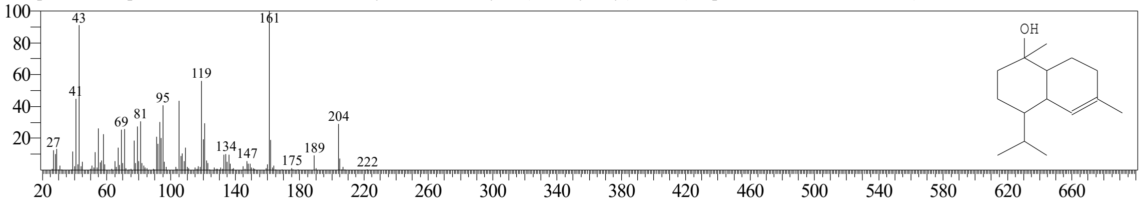

Hit#:4 Entry:59361 Library:NIST11.lib
SI:51 Formula:C15H26O CAS:51371-47-2 MolWeight:222 RetIndex:1530
CompName:Globulol $$ 1,1,4,7-Tetramethyldecahydro-1H-cyclopropa[e]azulen-4-ol, (1a.alpha.,4.alpha.,4a.alpha.,7.alpha.,7a.beta.,7b.alpha.)- $$ (.+/-.)-Globul

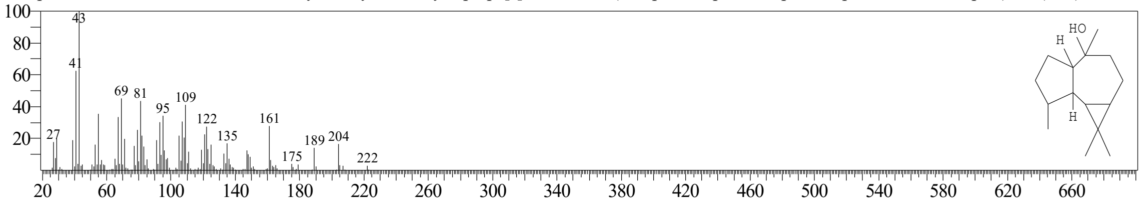

Hit#:5 Entry:101320 Library:NIST11.lib
SI:51 Formula:C17H26O3 CAS:0-00-0 MolWeight:278 RetIndex:2018
CompName:Acetic acid, 3-hydroxy-7-isopropenyl-1,4a-dimethyl-2,3,4,4a,5,6,7,8-octahydronaphthalen-2-yl ester $$ 3-Hydroxy-7-isopropenyl-1,4a-dimethyl-2,3

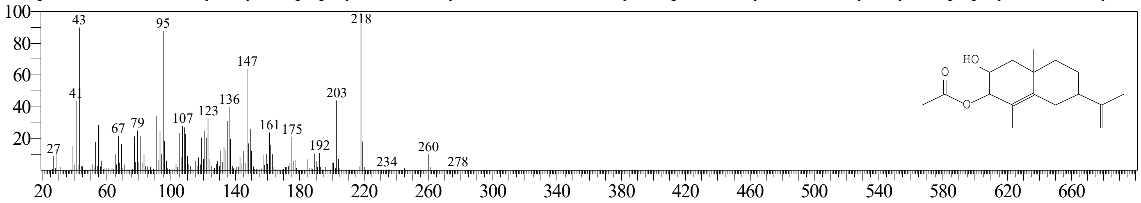

<< Target >>
Line#:79 R.Time:18.370(Scan#:2875) MassPeaks:383
RawMode:Averaged 18.365-18.375(2874-2876) BasePeak:43.10(41925)
BG Mode:Calc. from Peak Group 1 - Event 1 Scan

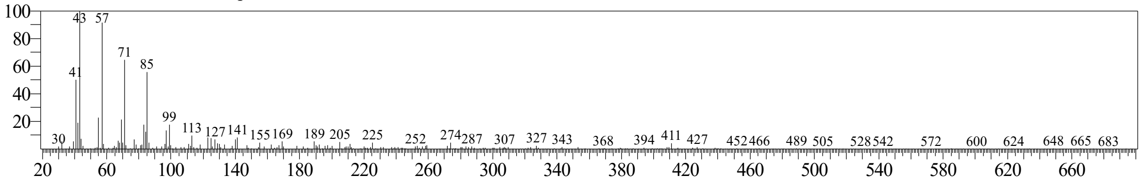

Hit#:1 Entry:196907 Library:NIST11.lib
SI:86 Formula:C32H66 CAS:55401-55-3 MolWeight:450 RetIndex:3138
CompName:Docosane, 11-decyl- $$ 11-n-Decyldocosane $$ 11-Decyldocosane # $$

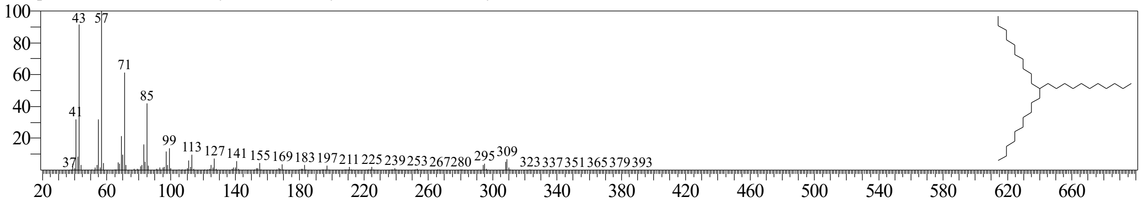

Hit#:2 Entry:93613 Library:NIST11.lib
SI:85 Formula:C19H40 CAS:1560-88-9 MolWeight:268 RetIndex:1846
CompName:Octadecane, 2-methyl- $$ 2-Methyloctadecane $$

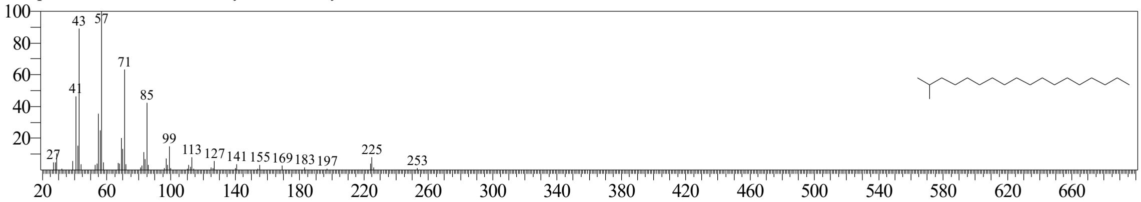

Hit#:3 Entry:165576 Library:NIST11.lib
SI:85 Formula:C26H54 CAS:13475-76-8 MolWeight:366 RetIndex:2542
CompName:Docosane, 11-butyl- $$ 11-Butyldocosane $$ Hexadecane, 5-decyl $$ 11-n-Butyldocosane $$

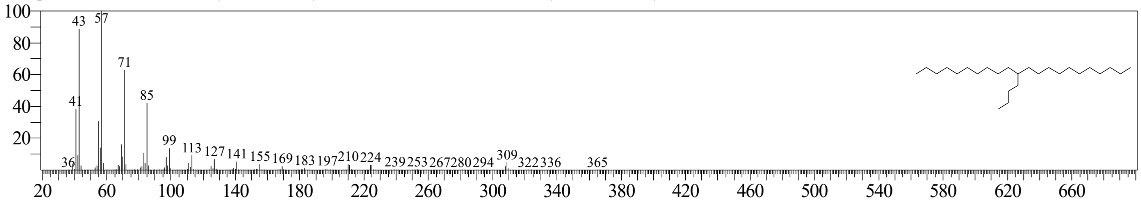

Hit#:4 Entry:165568 Library:NIST11.lib
SI:85 Formula:C26H54 CAS:55282-11-6 MolWeight:366 RetIndex:2477
CompName:Heneicosane, 11-(1-ethylpropyl)- $$ 11-(1-Ethylpropyl)heneicosane $$ 11-(3'-n-Pentyl)heneicosane $$ 11-(1-Ethylpropyl)henicosane # $$

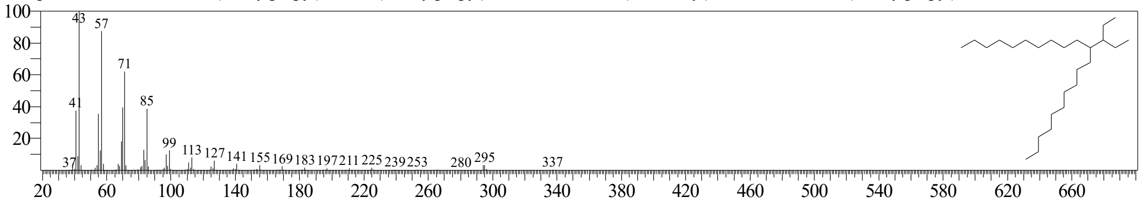

Hit#:5 Entry:201892 Library:NIST11.lib
SI:84 Formula:C34H70 CAS:55429-84-0 MolWeight:478 RetIndex:3337
CompName:Tetracosane, 11-decyl- $$ 11-n-Decyltetracosane $$ 11-Decyltetracosane # $$

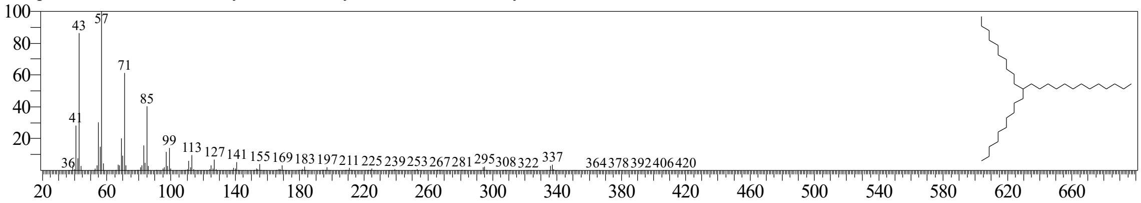

<< Target >>
Line#:80 R.Time:18.470(Scan#:2895) MassPeaks:435
RawMode:Averaged 18.465-18.475(2894-2896) BasePeak:108.00(4162216)
BG Mode:Calc. from Peak Group 1 - Event 1 Scan

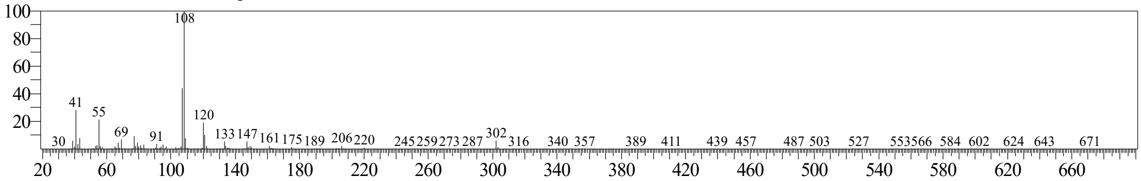

Hit#:1 Entry:46672 Library:NIST11.lib
SI:77 Formula:C15H24 CAS:0-00-0 MolWeight:204 RetIndex:1450
CompName:rotundene

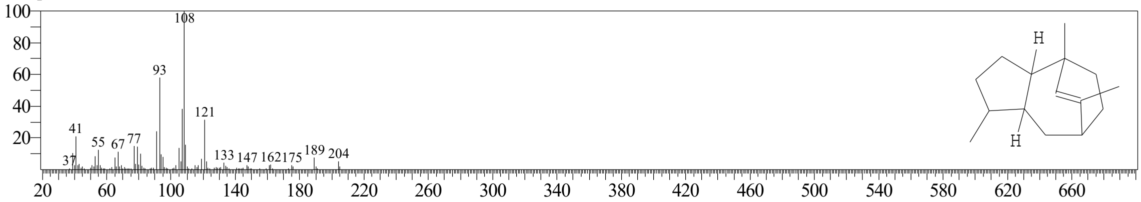

Hit#:2 Entry:22527 Library:NIST11.lib
SI:77 Formula:C12H20 CAS:74792-98-6 MolWeight:164 RetIndex:1065
CompName:Spiro[2.4]heptane, 1,2,4,5-tetramethyl-6-methylene- $$ 1,2,4,5-Tetramethyl-6-methylenespiro[2.4]heptane # $$

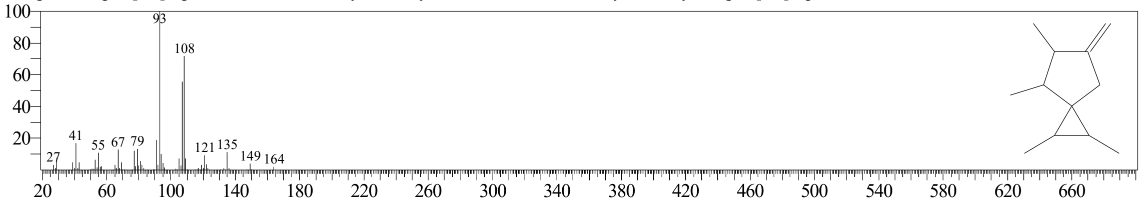

Hit#:3 Entry:29267 Library:NIST11.lib
SI:77 Formula:C12H16O CAS:156089-29-1 MolWeight:176 RetIndex:1388
CompName:Benzene, 1-methyl-4-(2-pentenyloxy)- $$ 1-Methyl-4-[(2E)-2-pentenyloxy]benzene # $$

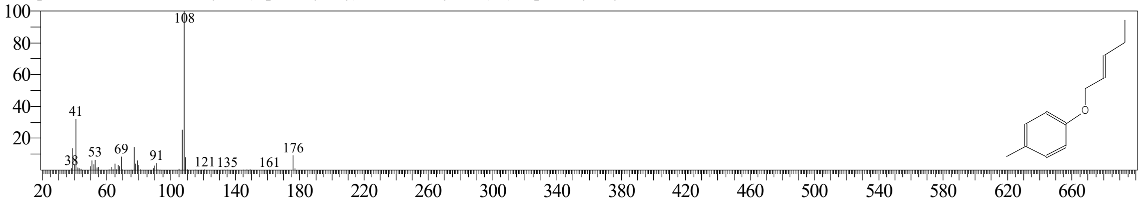

Hit#:4 Entry:9568 Library:NIST11.lib
SI:76 Formula:C8H12N2 CAS:29460-93-3 MolWeight:136 RetIndex:1015
CompName:2-(1-Methylpropyl)pyrazine $$ 2-Sec-butylpyrazine $$ 1-Methylpropylpyrazine $$ Pyrazine, (1-methylpropyl) $$ sec-Butylpyrazine $$

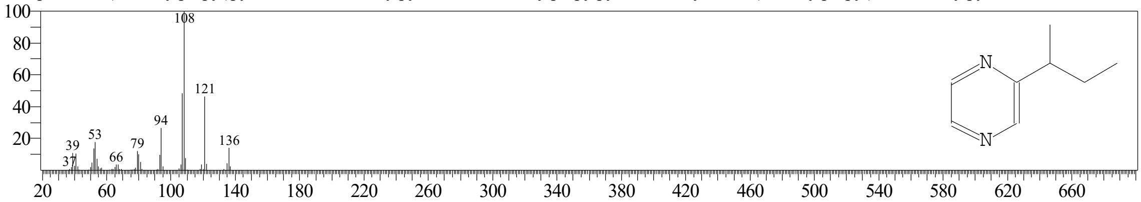

Hit#:5 Entry:22341 Library:NIST11.lib
SI:76 Formula:C10H16N2 CAS:91010-41-2 MolWeight:164 RetIndex:1228
CompName:2-Isoamyl-6-methylpyrazine $$ 2-Isopentyl-6-methylpyrazine # $$ 2-Methyl-6-isopentylpyrazine $$ 6-Methyl-2-(3-methylbutyl)-pyrazine $$ Pyrazi

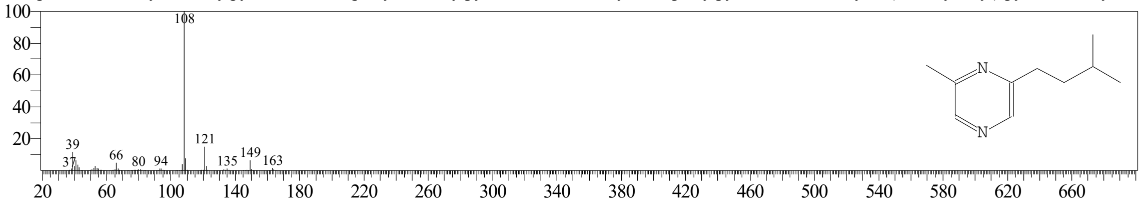

<< Target >>
Line#:81 R.Time:18.590(Scan#:2919) MassPeaks:428
RawMode:Averaged 18.585-18.595(2918-2920) BasePeak:108.05(7402616)
BG Mode:Calc. from Peak Group 1 - Event 1 Scan

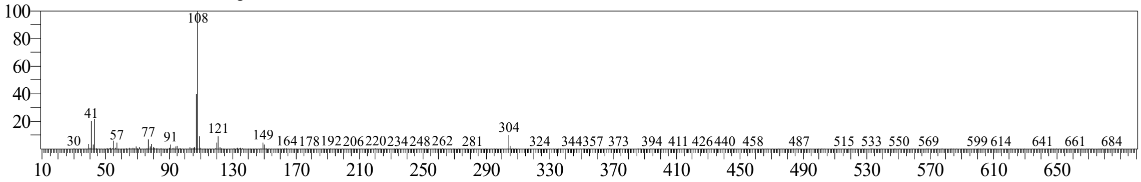

Hit#:1 Entry:121943 Library:NIST11.lib
SI:84 Formula:C21H36O CAS:501-24-6 MolWeight:304 RetIndex:2406
CompName:Phenol, 3-pentadecyl- $$ Phenol, m-pentadecyl- $$ m-Pentadecylphenol $$ Anacardol, tetrahydro- $$ Cyclogallipharaol $$ Hydrocardanol $$ Hydrc

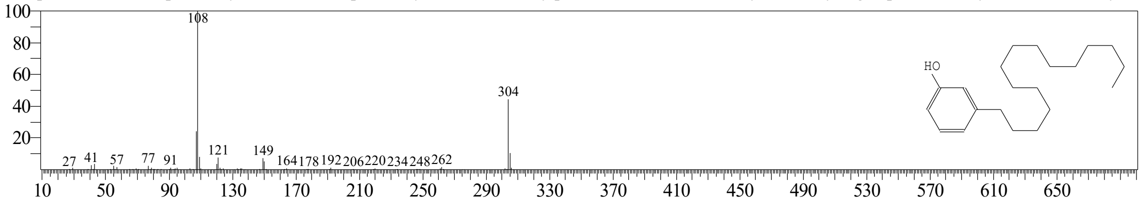

Hit#:2 Entry:30439 Library:NIST11.lib
SI:82 Formula:C12H18O CAS:85709-98-4 MolWeight:178 RetIndex:1296
CompName:Benzene, 1-(1,1-dimethylpropoxy)-4-methyl- $$ Toluene, 4-(1,1-dimethylpropoxy)- $$ 1-Methyl-4-(tert-pentyloxy)benzene # $$

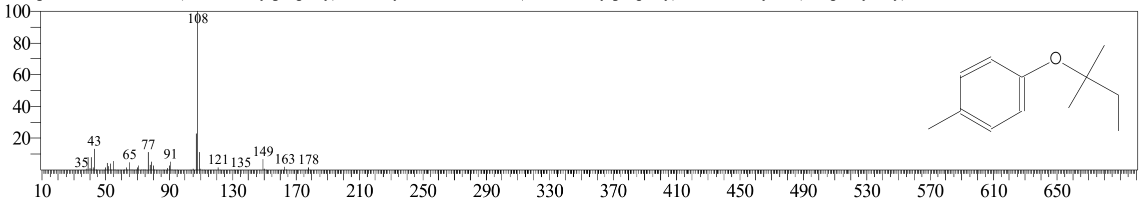

Hit#:3 Entry:15237 Library:NIST11.lib
SI:82 Formula:C9H10O2 CAS:140-39-6 MolWeight:150 RetIndex:1174
CompName:Acetic acid, 4-methylphenyl ester $$ Acetic acid, p-tolyl ester $$ p-Acetoxytoluene $$ p-Cresol acetate $$ p-Cresyl acetate $$ p-Methylphenyl aceta

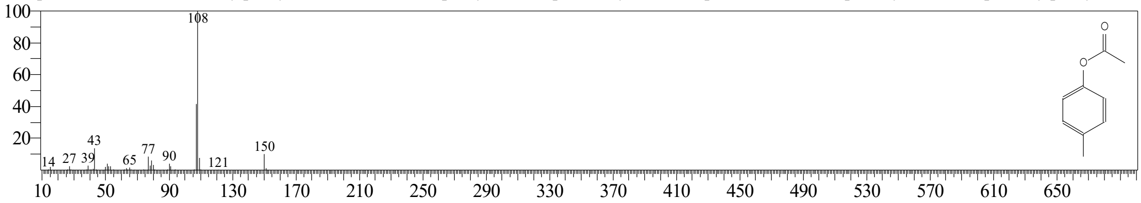

Hit#:4 Entry:79486 Library:NIST11.lib
SI:82 Formula:C14H18O4 CAS:0-00-0 MolWeight:250 RetIndex:1838
CompName:Oxalic acid, 2-methylphenyl pentyl ester

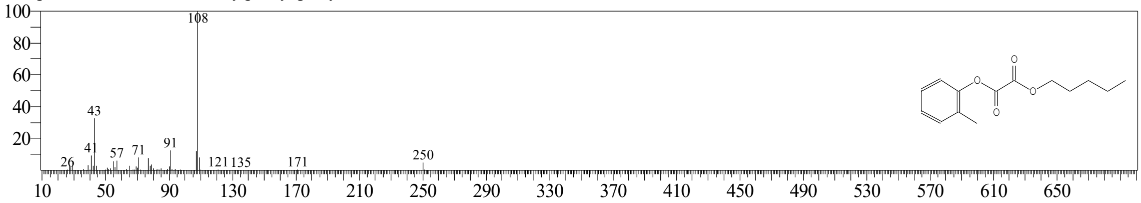

Hit#:5 Entry:59043 Library:NIST11.lib
SI:81 Formula:C13H18O3 CAS:0-00-0 MolWeight:222 RetIndex:1563
CompName:Carbonic acid, neopentyl 3-methylphenyl ester

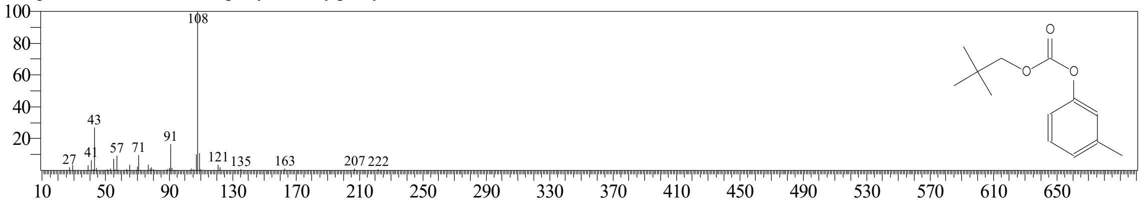

Qualitative Analysis Report

<< Target >>
Line#:82 R.Time:18.875(Scan#:2976) MassPeaks:352
RawMode:Averaged 18.870-18.880(2975-2977) BasePeak:41.10(17407)
BG Mode:Calc. from Peak Group 1 - Event 1 Scan

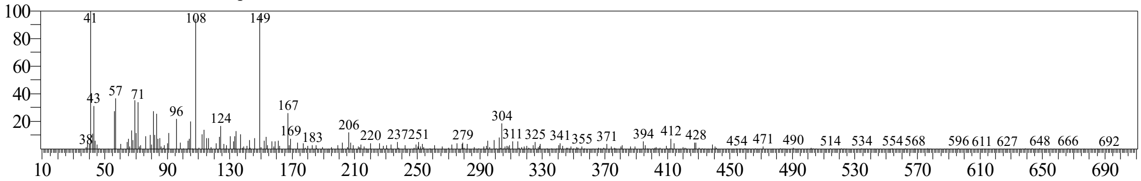

Hit#:1 Entry:207205 Library:NIST11.lib
SI:61 Formula:C34H58O4 CAS:119-06-2 MolWeight:530 RetIndex:3826
CompName:Bis(tridecyl) phthalate $$ 1,2-Benzenedicarboxylic acid, ditridecyl ester $$ Phthalic acid, ditridecyl ester $$ Ditridecyl phthalate $$ Polycizer 962-I

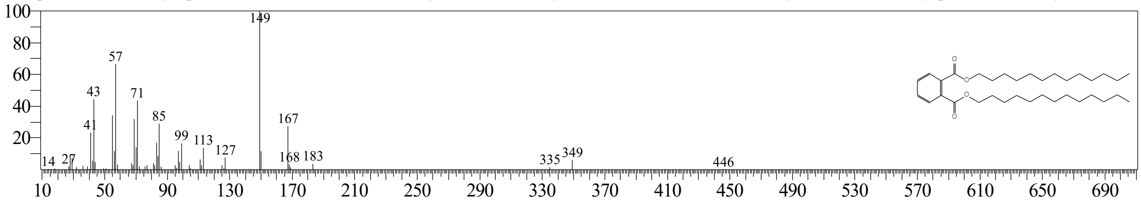

Hit#:2 Entry:162890 Library:NIST11.lib
SI:60 Formula:C18H35BrO2 CAS:0-00-0 MolWeight:362 RetIndex:2274
CompName:4-Bromobutanoic acid, tetradecyl ester $$ Tetradecyl 4-bromobutanoate # $$

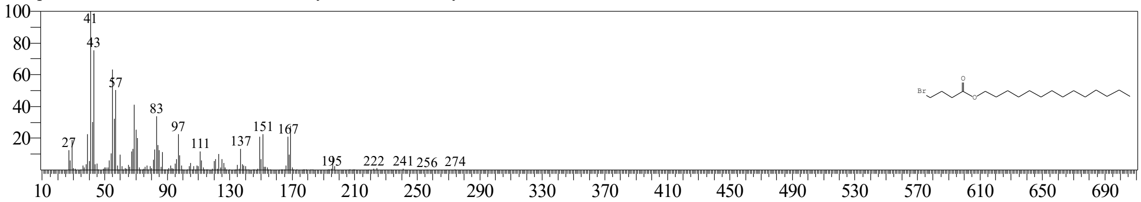

Hit#:3 Entry:159219 Library:NIST11.lib
SI:60 Formula:C19H17FN2O2S CAS:0-00-0 MolWeight:356 RetIndex:3063
CompName:3-(2,5-Dimethylanilinomethyl)-5-(3-fluorobenzylidene)-2,4-thiazolidinedione $$ (5E)-3-[(2,5-Dimethylanilino)methyl]-5-(3-fluorobenzylidene)-1,3

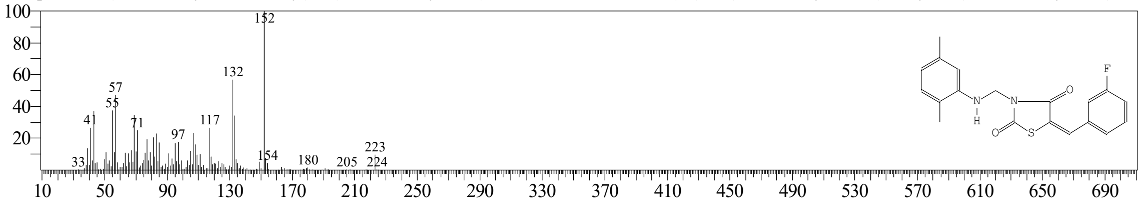

Hit#:4 Entry:41581 Library:NIST11.lib
SI:59 Formula:C13H24O CAS:7069-41-2 MolWeight:196 RetIndex:1510
CompName:2-Tridecenal, (E)- $$ (2E)-2-Tridecenal $$ (E)-2-Tridecenal $$ (E)-Tridec-2-enal $$

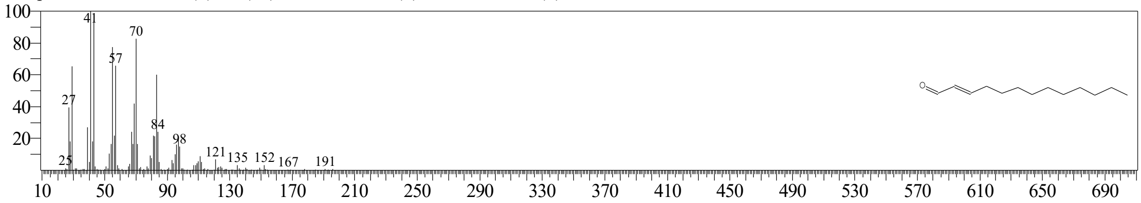

Hit#:5 Entry:171111 Library:NIST11.lib
SI:59 Formula:C19H37BrO2 CAS:0-00-0 MolWeight:376 RetIndex:2373
CompName:4-Bromobutanoic acid, pentadecyl ester $$ Pentadecyl 4-bromobutanoate # $$

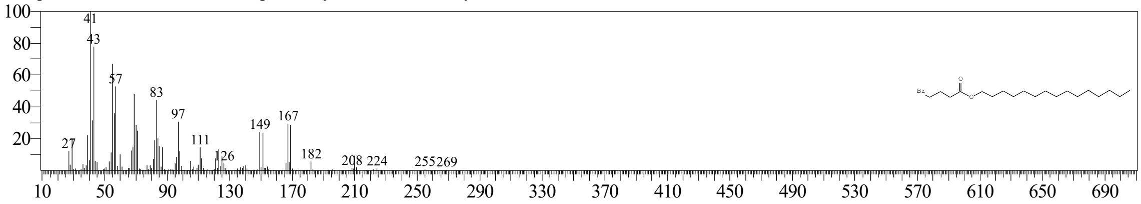

<< Target >>
Line#:83 R.Time:19.455(Scan#:3092) MassPeaks:381
RawMode:Averaged 19.450-19.460(3091-3093) BasePeak:107.05(14073)
BG Mode:Calc. from Peak Group 1 - Event 1 Scan

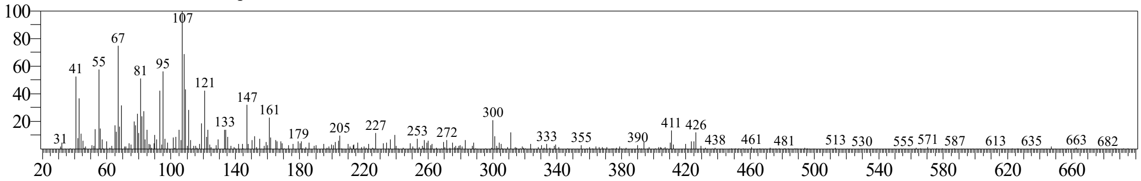

Hit#:1 Entry:132823 Library:NIST11.lib
SI:74 Formula:C21H34O2 CAS:59149-01-8 MolWeight:318 RetIndex:2308
CompName:Methyl (Z)-5,11,14,17-eicosatetraenoate $$ Methyl (5Z,11Z,14Z,17Z)-5,11,14,17-icosatetraenoate # $$

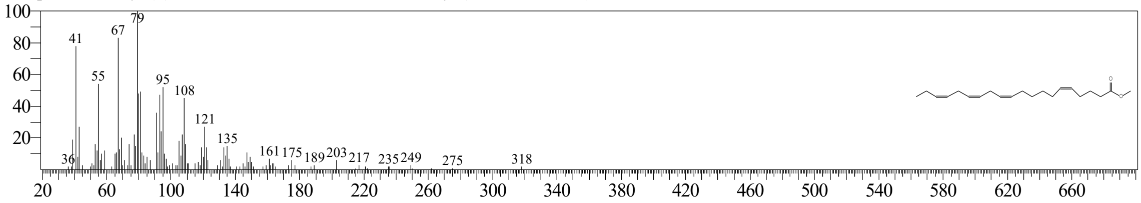

Hit#:2 Entry:59420 Library:NIST11.lib
SI:74 Formula:C15H26O CAS:95975-84-1 MolWeight:222 RetIndex:1530
CompName:4aH-cycloprop[e]azulen-4a-ol, decahydro-1,1,4,7-tetramethyl- $$ 1,1,4,7-Tetramethyldecahydro-4ah-cyclopropa[e]azulen-4a-ol # $$

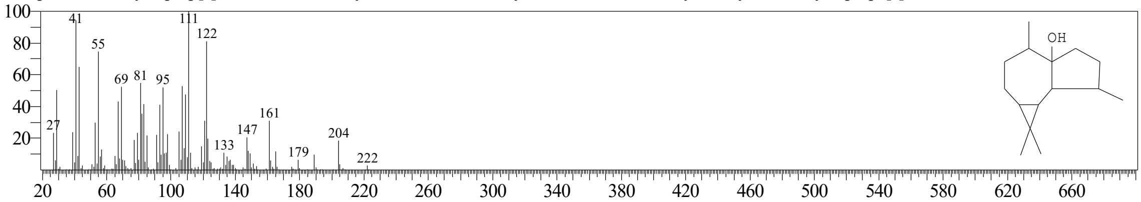

Hit#:3 Entry:46664 Library:NIST11.lib
SI:74 Formula:C15H24 CAS:0-00-0 MolWeight:204 RetIndex:1475
CompName:Cycloheptane, 4-methylene-1-methyl-2-(2-methyl-1-propen-1-yl)-1-vinyl- $$ 1-Methyl-4-methylene-2-(2-methyl-1-propenyl)-1-vinylcycloheptane #

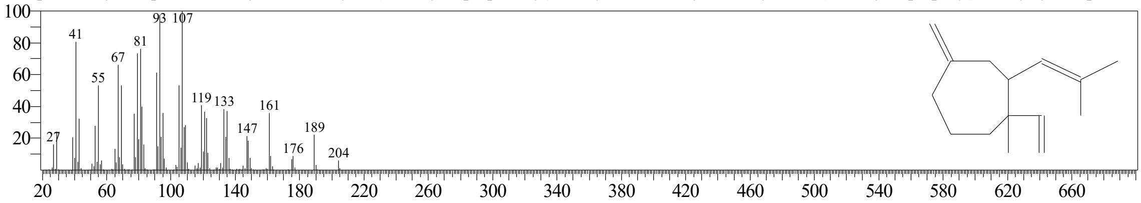

Hit#:4 Entry:59362 Library:NIST11.lib
SI:72 Formula:C15H26O CAS:577-27-5 MolWeight:222 RetIndex:1530
CompName:Ledol $$ 1H-Cycloprop[e]azulen-4-ol, decahydro-1,1,4,7-tetramethyl-, [1ar-(1a.alpha.,4.alpha.,4a.beta.,7.alpha.,7a.beta.,7b.alpha.)]- $$ 1H-Cyclopr

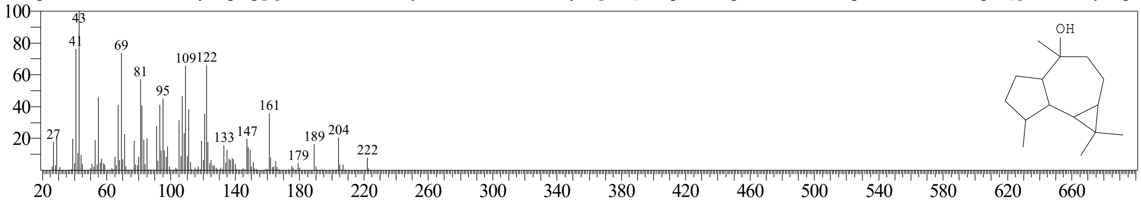

Hit#:5 Entry:59359 Library:NIST11.lib
SI:72 Formula:C15H26O CAS:552-02-3 MolWeight:222 RetIndex:1530
CompName:1H-Cycloprop[e]azulen-4-ol, decahydro-1,1,4,7-tetramethyl-, [1aR-(1a.alpha.,4.beta.,4a.beta.,7.alpha.,7a.beta.,7b.alpha.)]- $$ Viridiflorol $$ 1,1,4,7

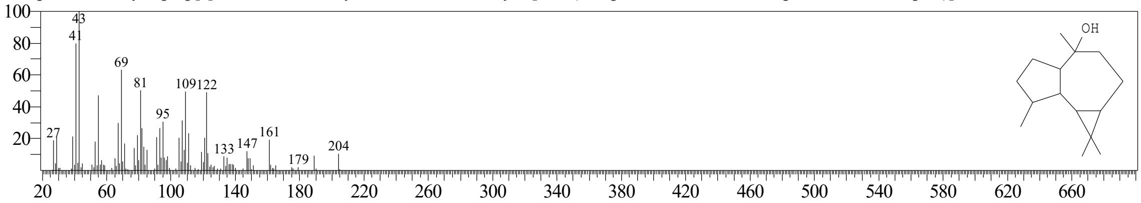

<< Target >>
Line#:84 R.Time:19.745(Scan#:3150) MassPeaks:376
RawMode:Averaged 19.740-19.750(3149-3151) BasePeak:43.10(48902)
BG Mode:Calc. from Peak Group 1 - Event 1 Scan

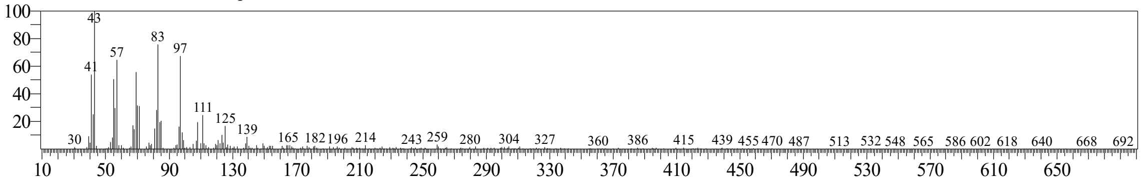

Hit#:1 Entry:155811 Library:NIST11.lib
SI:88 Formula:C25H50 CAS:0-00-0 MolWeight:350 RetIndex:2514
CompName:Z-12-Pentacosene $$ (12Z)-12-Pentacosene # $$

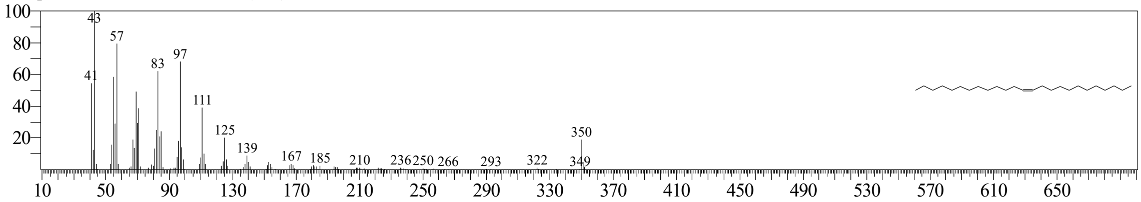

Hit#:2 Entry:147159 Library:NIST11.lib
SI:88 Formula:C18H33F3O2 CAS:6222-03-3 MolWeight:338 RetIndex:1812
CompName:Trifluoroacetoxy hexadecane $$ Hexadecyl trifluoroacetate $$ Hexadecyl 2,2,2-trifluoroacetate $$ 1-Hexadecanol, trifluoroacetate $$ Acetic acid, tr

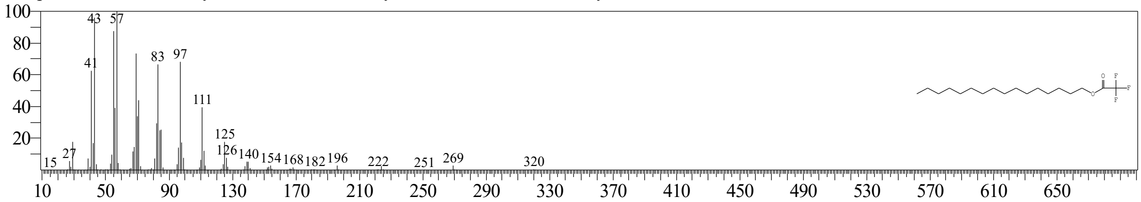

Hit#:3 Entry:114020 Library:NIST11.lib
SI:88 Formula:C21H42 CAS:95008-11-0 MolWeight:294 RetIndex:2117
CompName:10-Heneicosene (c,t) $$ (10E)-10-Henicosene # $$

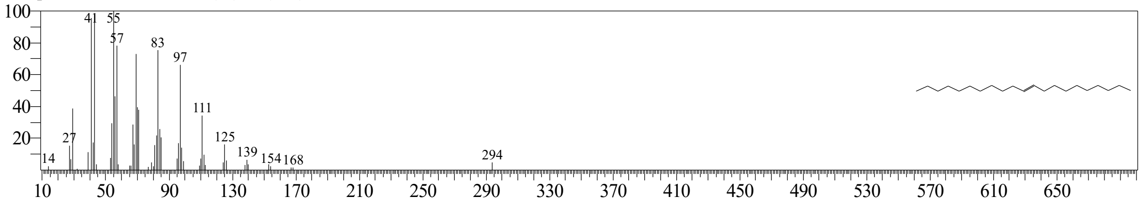

Hit#:4 Entry:197101 Library:NIST11.lib
SI:88 Formula:C21H35F7O2 CAS:959085-66-6 MolWeight:452 RetIndex:1833
CompName:Heptadecyl heptafluorobutyrate $$ Heptadecyl 2,2,3,3,4,4,4-heptafluorobutanoate $$ 1-Heptadecanol, heptafluorobutyrate $$ Heptadecyl perfluorob

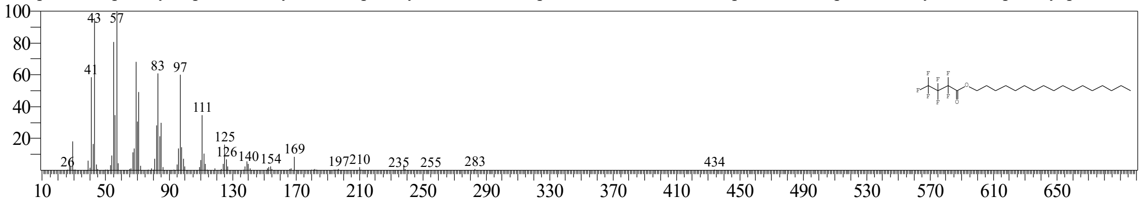

Hit#:5 Entry:82687 Library:NIST11.lib
SI:88 Formula:C16H30O2 CAS:56221-91-1 MolWeight:254 RetIndex:1769
CompName:13-Tetradecen-1-ol acetate $$ 13-Tetradecenyl acetate # $$

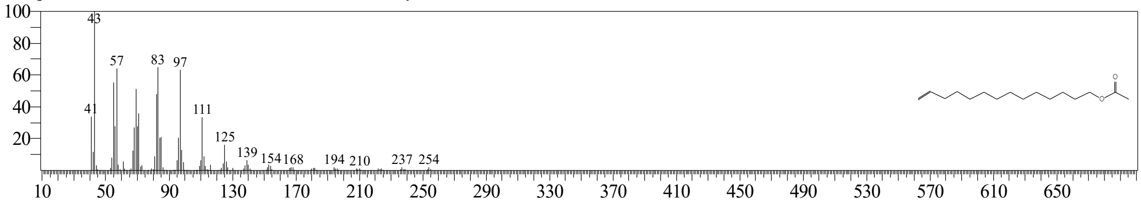

<< Target >>
Line#:85 R.Time:20.090(Scan#:3219) MassPeaks:390
RawMode:Averaged 20.085-20.095(3218-3220) BasePeak:108.00(88817)
BG Mode:Calc. from Peak Group 1 - Event 1 Scan

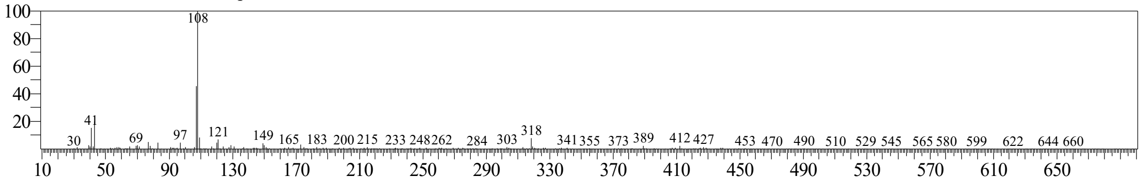

Hit#:1 Entry:15237 Library:NIST11.lib
SI:75 Formula:C9H10O2 CAS:140-39-6 MolWeight:150 RetIndex:1174
CompName:Acetic acid, 4-methylphenyl ester $$ Acetic acid, p-tolyl ester $$ p-Acetoxytoluene $$ p-Cresol acetate $$ p-Cresyl acetate $$ p-Methylphenyl aceta

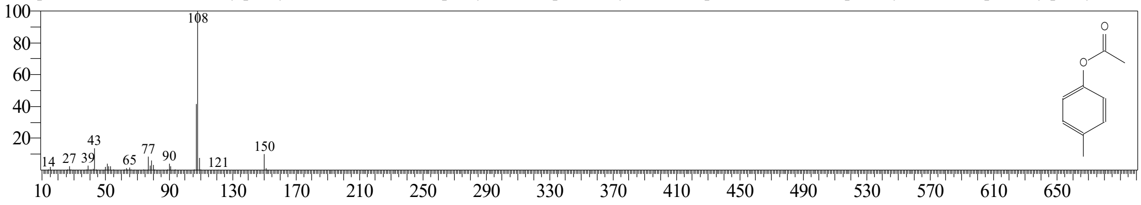

Hit#:2 Entry:39885 Library:NIST11.lib
SI:74 Formula:C11H14O3 CAS:0-00-0 MolWeight:194 RetIndex:1448
CompName:Carbonic acid, propyl 3-methylphenyl ester

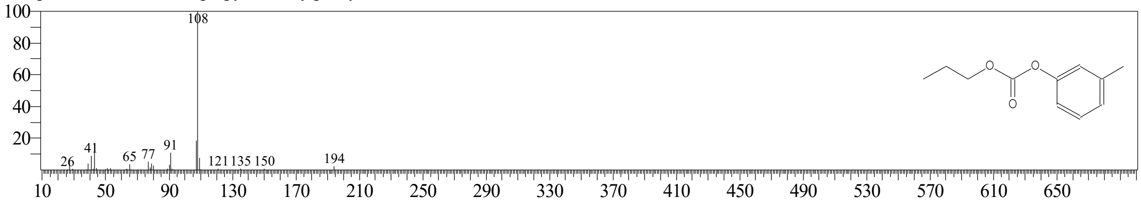

Hit#:3 Entry:30439 Library:NIST11.lib
SI:73 Formula:C12H18O CAS:85709-98-4 MolWeight:178 RetIndex:1296
CompName:Benzene, 1-(1,1-dimethylpropoxy)-4-methyl- $$ Toluene, 4-(1,1-dimethylpropoxy)- $$ 1-Methyl-4-(tert-pentyloxy)benzene # $$

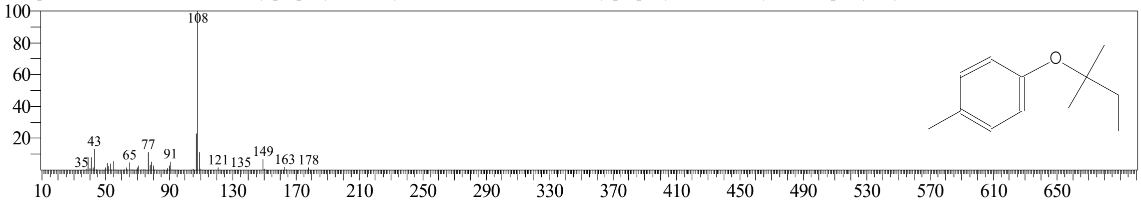

Hit#:4 Entry:112148 Library:NIST11.lib
SI:73 Formula:C17H24O4 CAS:0-00-0 MolWeight:292 RetIndex:2136
CompName:Oxalic acid, 2-methylphenyl octyl ester

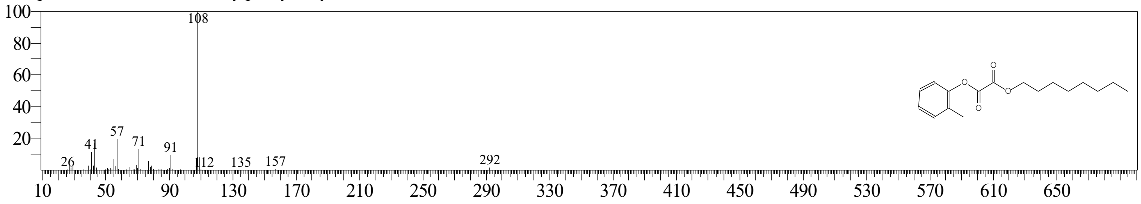

Hit#:5 Entry:79486 Library:NIST11.lib
SI:73 Formula:C14H18O4 CAS:0-00-0 MolWeight:250 RetIndex:1838
CompName:Oxalic acid, 2-methylphenyl pentyl ester

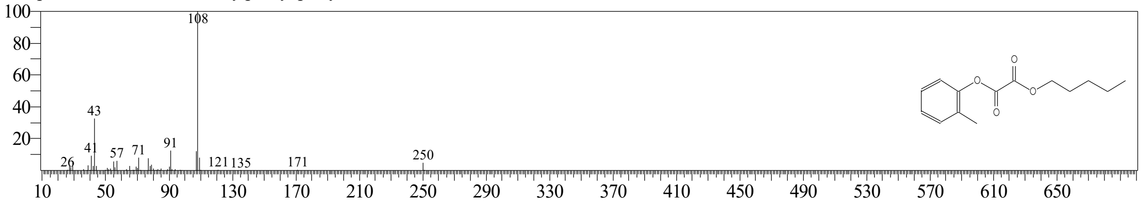

<< Target >>
Line#:86 R.Time:20.455(Scan#:3292) MassPeaks:373
RawMode:Averaged 20.450-20.460(3291-3293) BasePeak:41.05(12271)
BG Mode:Calc. from Peak Group 1 - Event 1 Scan

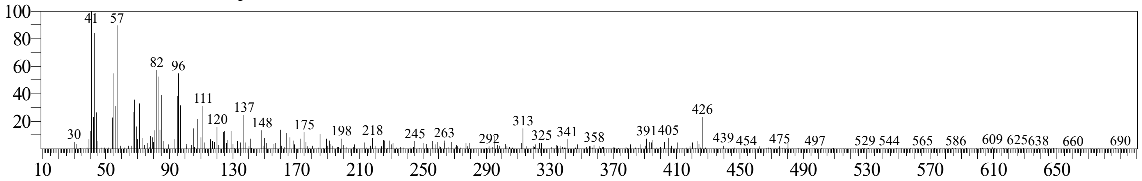

Hit#:1 Entry:93586 Library:NIST11.lib
SI:77 Formula:C18H36O CAS:638-66-4 MolWeight:268 RetIndex:1999
CompName:Octadecanal $$ Stearaldehyde $$ Octadecyl aldehyde $$ Stearyl aldehyde $$ n-Octadecanal $$

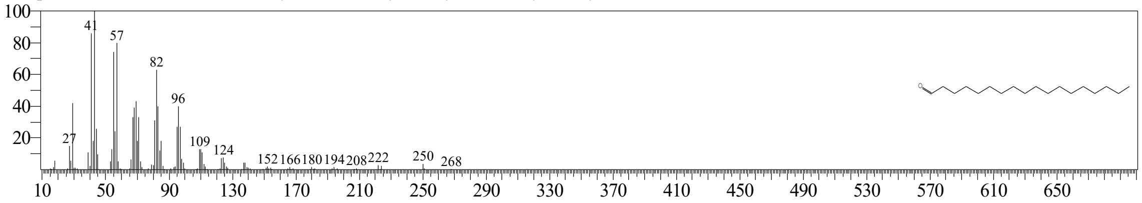

Hit#:2 Entry:93594 Library:NIST11.lib
SI:76 Formula:C18H36O CAS:0-00-0 MolWeight:268 RetIndex:2061
CompName:Z-2-Octadecen-1-ol $$ (2Z)-2-Octadecen-1-ol # $$

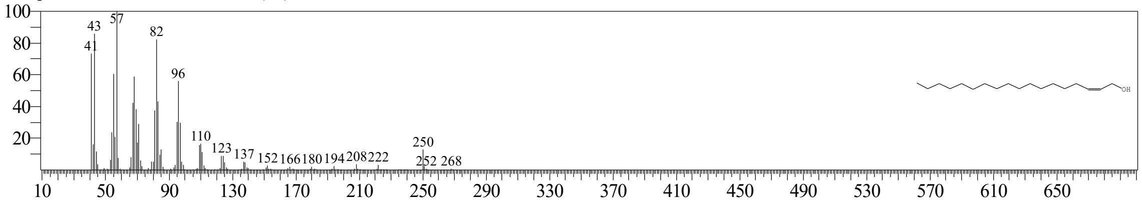

Hit#:3 Entry:82829 Library:NIST11.lib
SI:76 Formula:C17H34O CAS:0-00-0 MolWeight:254 RetIndex:1899
CompName:Heptadecanal

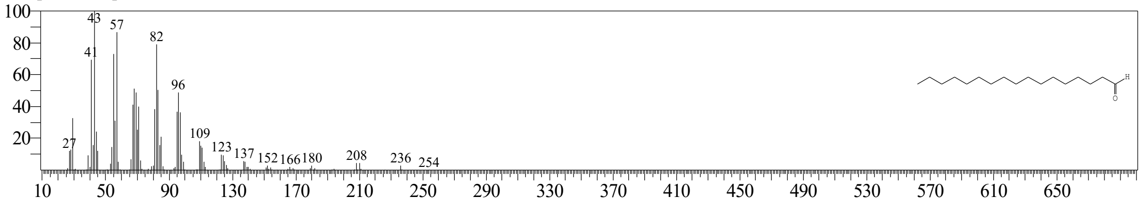

Hit#:4 Entry:52426 Library:NIST11.lib
SI:76 Formula:C14H28O CAS:0-00-0 MolWeight:212 RetIndex:1664
CompName:E-2-Tetradecen-1-ol $$ (2E)-2-Tetradecen-1-ol # $$

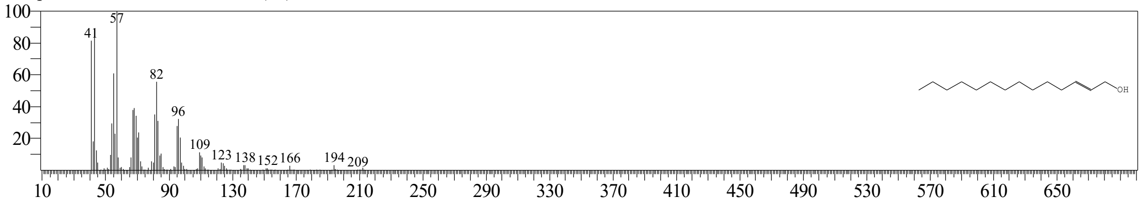

Hit#:5 Entry:72472 Library:NIST11.lib
SI:75 Formula:C16H32O CAS:629-80-1 MolWeight:240 RetIndex:1800
CompName:Hexadecanal $$ Palmitaldehyde $$ 1-Hexadecanal $$ n-Hexadecanal $$

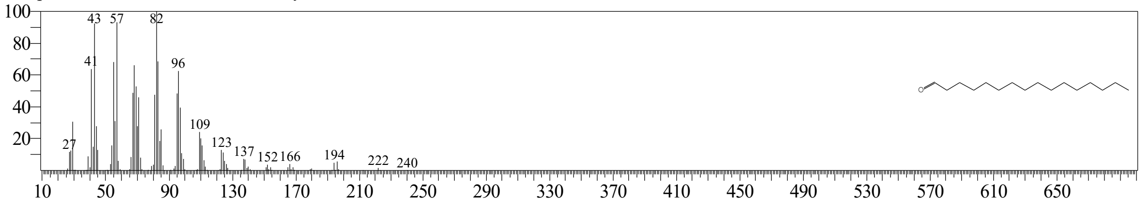

<< Target >>
Line#:87 R.Time:21.420(Scan#:3485) MassPeaks:406
RawMode:Averaged 21.415-21.425(3484-3486) BasePeak:107.05(39305)
BG Mode:Calc. from Peak Group 1 - Event 1 Scan

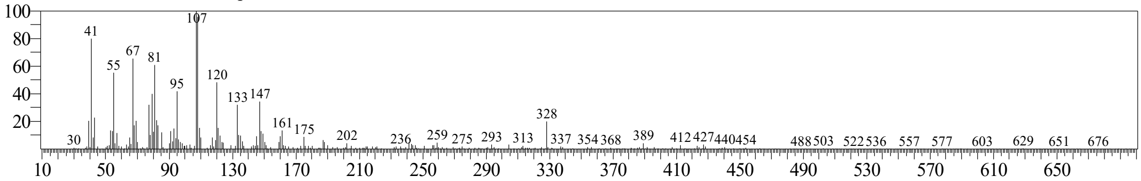

Hit#:1 Entry:132823 Library:NIST11.lib
SI:80 Formula:C21H34O2 CAS:59149-01-8 MolWeight:318 RetIndex:2308
CompName:Methyl (Z)-5,11,14,17-eicosatetraenoate $$ Methyl (5Z,11Z,14Z,17Z)-5,11,14,17-icosatetraenoate # $$

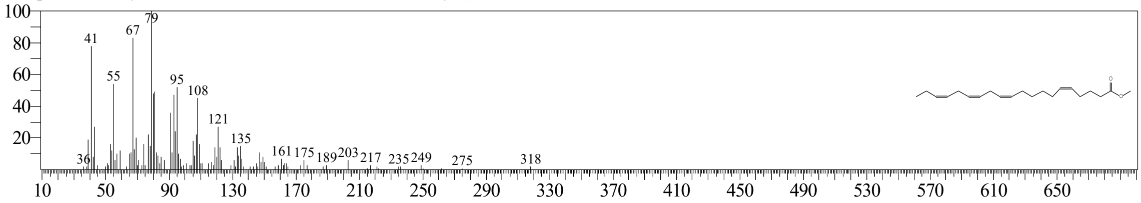

Hit#:2 Entry:46664 Library:NIST11.lib
SI:80 Formula:C15H24 CAS:0-00-0 MolWeight:204 RetIndex:1475
CompName:Cycloheptane, 4-methylene-1-methyl-2-(2-methyl-1-propen-1-yl)-1-vinyl- $$ 1-Methyl-4-methylene-2-(2-methyl-1-propenyl)-1-vinylcycloheptane #

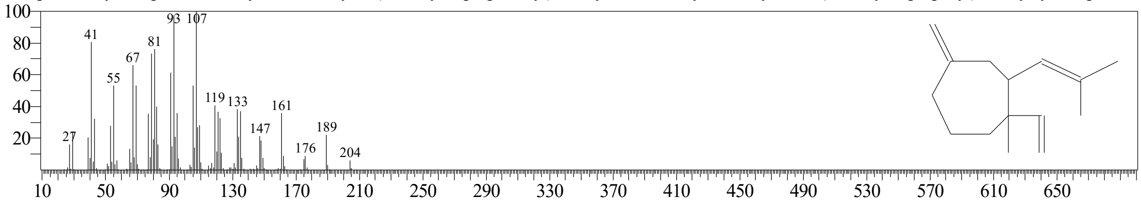

Hit#:3 Entry:37592 Library:NIST11.lib
SI:78 Formula:C14H22 CAS:62338-54-9 MolWeight:190 RetIndex:1485
CompName:1,5-Cycloundecadiene, 8,8-dimethyl-9-methylene- $$ 8,8-Dimethyl-9-methylene-1,5-cycloundecadiene # $$

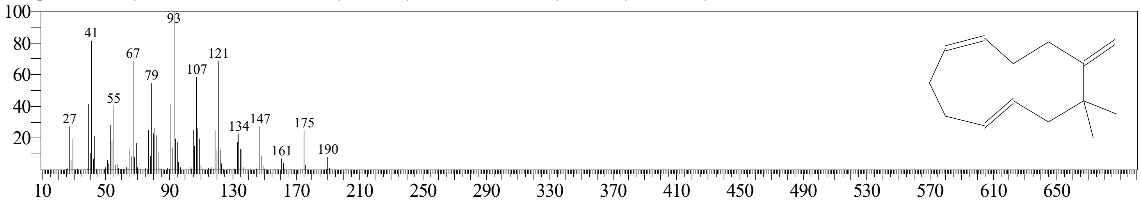

Hit#:4 Entry:46667 Library:NIST11.lib
SI:78 Formula:C15H24 CAS:3691-11-0 MolWeight:204 RetIndex:1490
CompName:Azulene, 1,2,3,5,6,7,8,8a-octahydro-1,4-dimethyl-7-(1-methylethenyl)-, [1S-(1.alpha.,7.alpha.,8a.beta.)]- $$ Guaia-1(10),11-diene $$ .alpha.-Bulnes

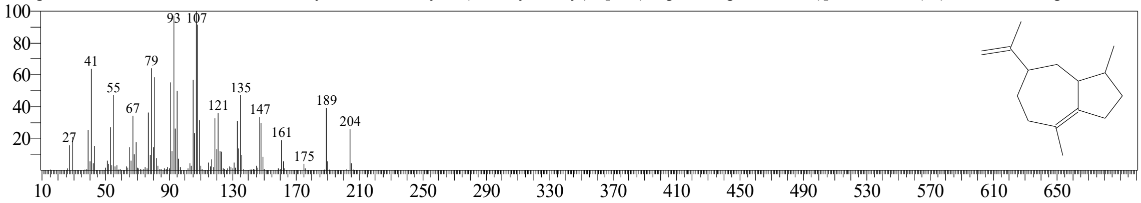

Hit#:5 Entry:146224 Library:NIST11.lib
SI:78 Formula:C22H40O2 CAS:56599-50-9 MolWeight:336 RetIndex:2453
CompName:2H-Pyran, 2-(7-heptadecynyloxy)tetrahydro- $$ 2-(7-Heptadecynyloxy)tetrahydro-2H-pyran # $$

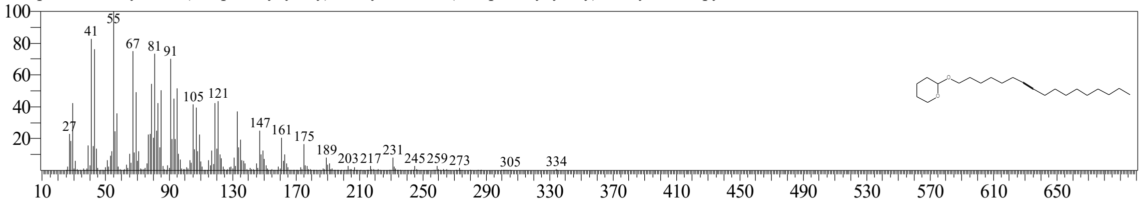

Qualitative Analysis Report

<< Target >>
Line#:88   R.Time:21.520(Scan#:3505) MassPeaks:365
RawMode:Averaged 21.515-21.525(3504-3506) BasePeak:108.05(116178)
BG Mode:Calc. from Peak Group 1 - Event 1 Scan

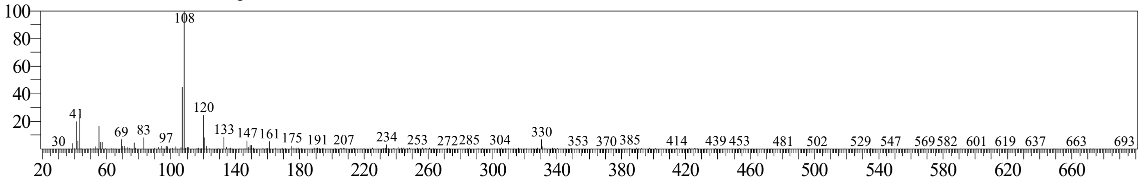

Hit#:1 Entry:154416 Library:NIST11.lib
SI:72 Formula:C21H32O4 CAS:0-00-0 MolWeight:348 RetIndex:2534
CompName:Oxalic acid, dodecyl 2-methylphenyl ester

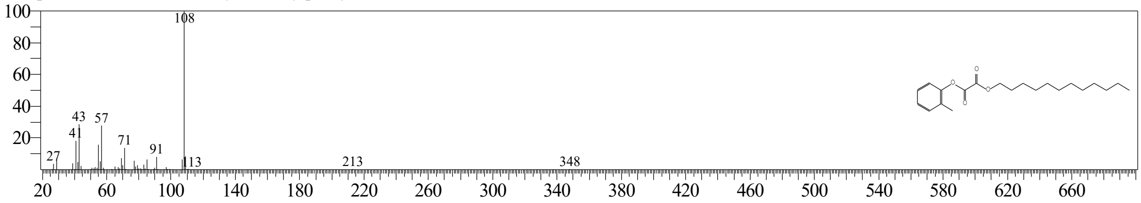

Hit#:2 Entry:144667 Library:NIST11.lib
SI:72 Formula:C20H30O4 CAS:0-00-0 MolWeight:334 RetIndex:2434
CompName:Oxalic acid, 2-methylphenyl undecyl ester

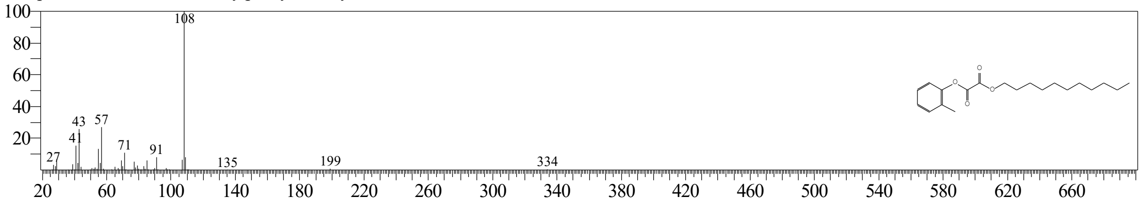

Hit#:3 Entry:163171 Library:NIST11.lib
SI:71 Formula:C22H34O4 CAS:0-00-0 MolWeight:362 RetIndex:2633
CompName:Oxalic acid, 2-methylphenyl tridecyl ester

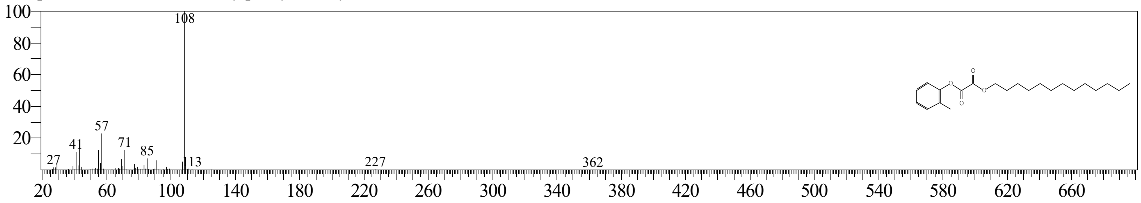

Hit#:4 Entry:123190 Library:NIST11.lib
SI:70 Formula:C18H26O4 CAS:0-00-0 MolWeight:306 RetIndex:2235
CompName:Oxalic acid, 2-methylphenyl nonyl ester

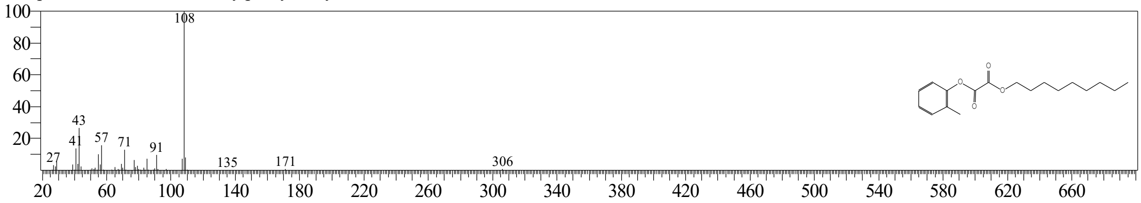

Hit#:5 Entry:171380 Library:NIST11.lib
SI:70 Formula:C23H36O4 CAS:0-00-0 MolWeight:376 RetIndex:2732
CompName:Oxalic acid, 2-methylphenyl tetradecyl ester

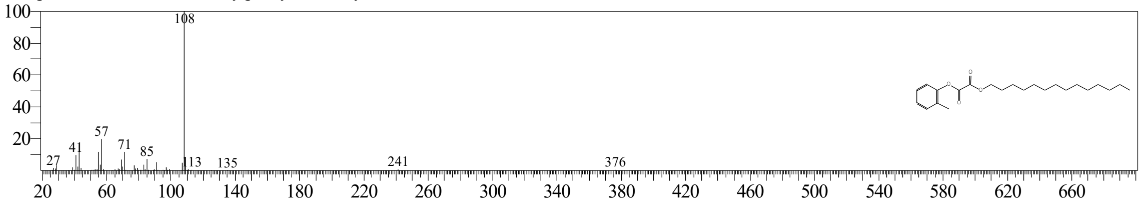

<< Target >>
Line#:89 R.Time:21.660(Scan#:3533) MassPeaks:342
RawMode:Averaged 21.655-21.665(3532-3534) BasePeak:43.10(80809)
BG Mode:Calc. from Peak Group 1 - Event 1 Scan

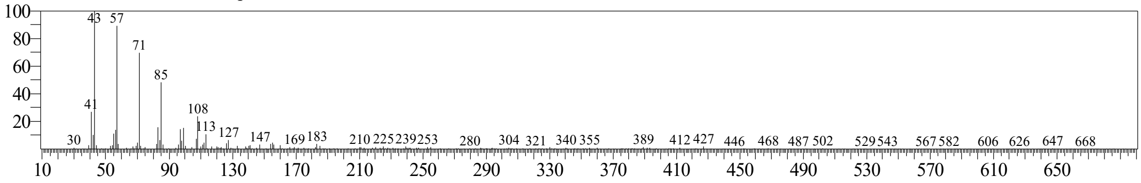

Hit#:1 Entry:42951 Library:NIST11.lib
SI:87 Formula:C14H30 CAS:6117-98-2 MolWeight:198 RetIndex:1285
CompName:2,3-Dimethyldodecane

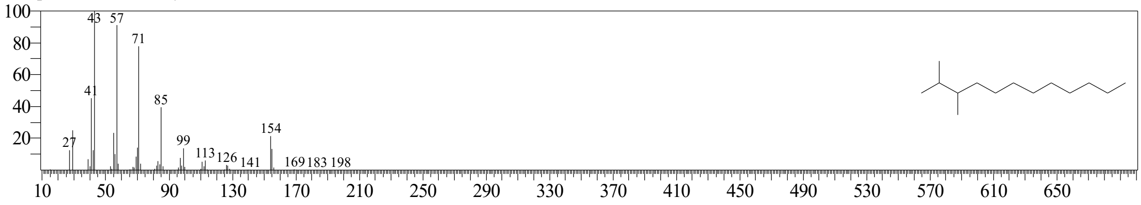

Hit#:2 Entry:26090 Library:NIST11.lib
SI:87 Formula:C12H26 CAS:7045-71-8 MolWeight:170 RetIndex:1150
CompName:Undecane, 2-methyl- $$ 2-Methylundecane $$

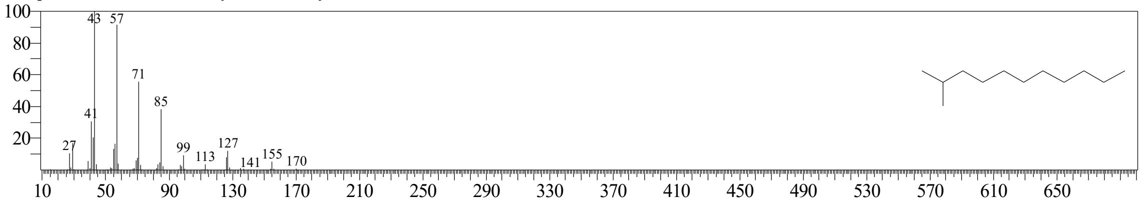

Hit#:3 Entry:42960 Library:NIST11.lib
SI:87 Formula:C14H30 CAS:1560-96-9 MolWeight:198 RetIndex:1349
CompName:Tridecane, 2-methyl- $$ 2-Methyltridecane $$ 2-Methyl-n-tridecane $$

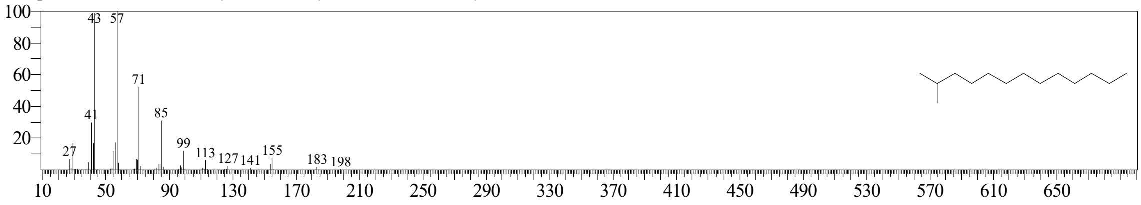

Hit#:4 Entry:62482 Library:NIST11.lib
SI:87 Formula:C16H34 CAS:55045-10-8 MolWeight:226 RetIndex:1548
CompName:Tridecane, 6-propyl- $$ 6-Propyltridecane # $$

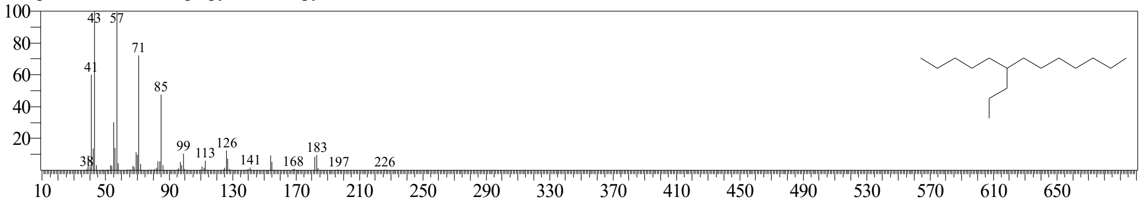

Hit#:5 Entry:201892 Library:NIST11.lib
SI:87 Formula:C34H70 CAS:55429-84-0 MolWeight:478 RetIndex:3337
CompName:Tetracosane, 11-decyl- $$ 11-n-Decyltetracosane $$ 11-Decyltetracosane # $$

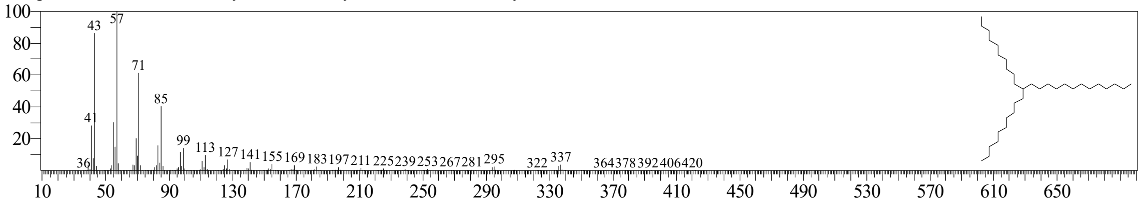

<< Target >>
Line#:90 R.Time:21.940(Scan#:3589) MassPeaks:425
RawMode:Averaged 21.935-21.945(3588-3590) BasePeak:108.05(1767013)
BG Mode:Calc. from Peak Group 1 - Event 1 Scan

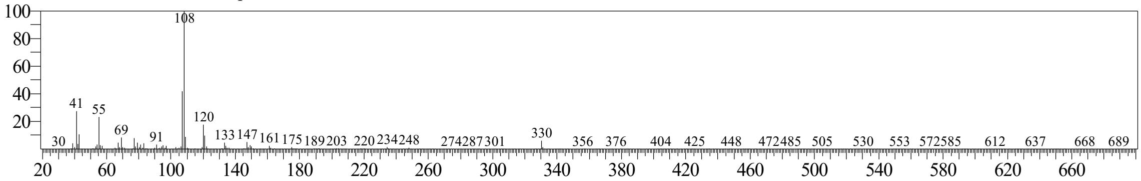

Hit#:1 Entry:46672 Library:NIST11.lib
SI:77 Formula:C15H24 CAS:0-00-0 MolWeight:204 RetIndex:1450
CompName:rotundene

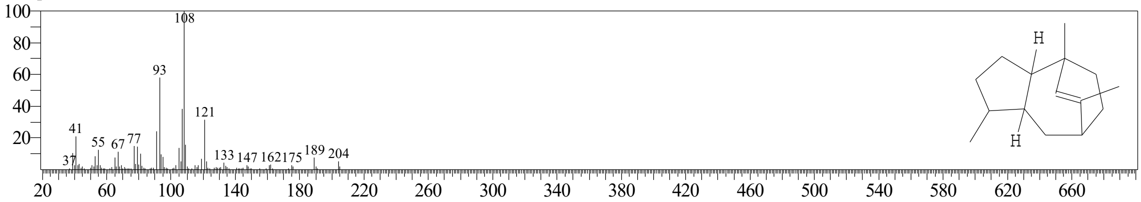

Hit#:2 Entry:22527 Library:NIST11.lib
SI:77 Formula:C12H20 CAS:74792-98-6 MolWeight:164 RetIndex:1065
CompName:Spiro[2.4]heptane, 1,2,4,5-tetramethyl-6-methylene- $$ 1,2,4,5-Tetramethyl-6-methylenespiro[2.4]heptane # $$

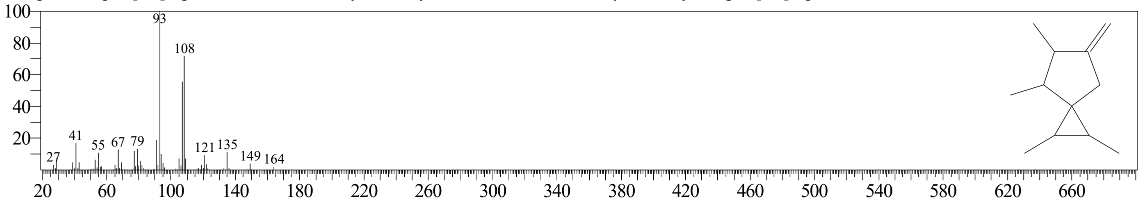

Hit#:3 Entry:29267 Library:NIST11.lib
SI:77 Formula:C12H16O CAS:156089-29-1 MolWeight:176 RetIndex:1388
CompName:Benzene, 1-methyl-4-(2-pentenyloxy)- $$ 1-Methyl-4-[(2E)-2-pentenyloxy]benzene # $$

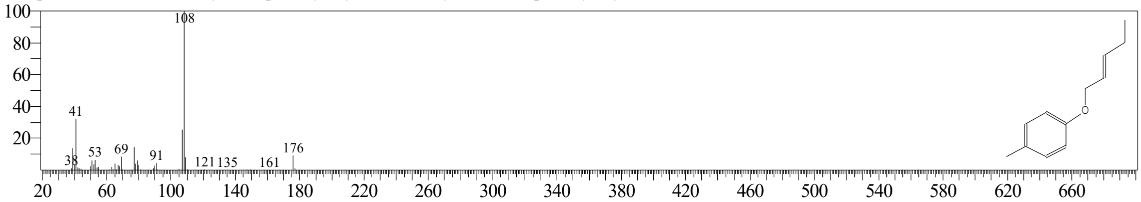

Hit#:4 Entry:22341 Library:NIST11.lib
SI:75 Formula:C10H16N2 CAS:91010-41-2 MolWeight:164 RetIndex:1228
CompName:2-Isoamyl-6-methylpyrazine $$ 2-Isopentyl-6-methylpyrazine # $$ 2-Methyl-6-isopentylpyrazine $$ 6-Methyl-2-(3-methylbutyl)-pyrazine $$ Pyrazi

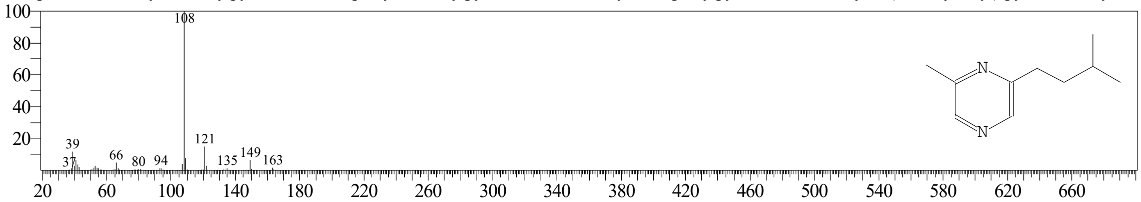

Hit#:5 Entry:9568 Library:NIST11.lib
SI:75 Formula:C8H12N2 CAS:29460-93-3 MolWeight:136 RetIndex:1015
CompName:2-(1-Methylpropyl)pyrazine $$ 2-Sec-butylpyrazine $$ 1-Methylpropylpyrazine $$ Pyrazine, (1-methylpropyl) $$ sec-Butylpyrazine $$

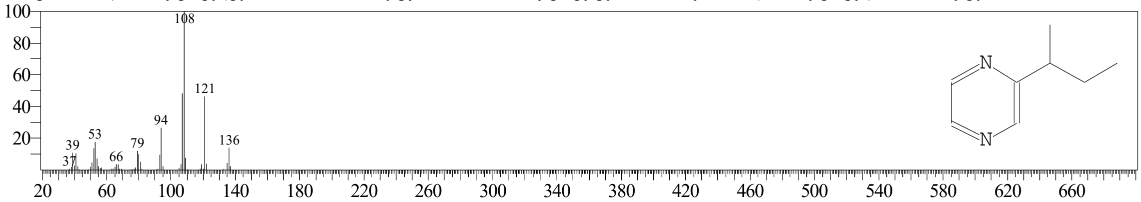

Qualitative Analysis Report

<< Target >>
Line#:91 R.Time:22.085(Scan#:3618) MassPeaks:401
RawMode:Averaged 22.080-22.090(3617-3619) BasePeak:108.05(1482402)
BG Mode:Calc. from Peak Group 1 - Event 1 Scan

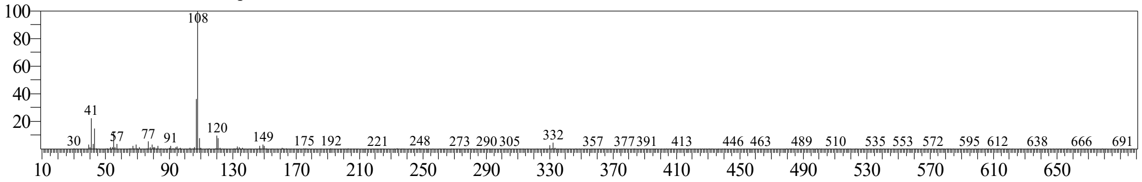

Hit#:1 Entry:30439 Library:NIST11.lib
SI:81 Formula:C12H18O CAS:85709-98-4 MolWeight:178 RetIndex:1296
CompName:Benzene, 1-(1,1-dimethylpropoxy)-4-methyl- $$ Toluene, 4-(1,1-dimethylpropoxy)- $$ 1-Methyl-4-(tert-pentyloxy)benzene # $$

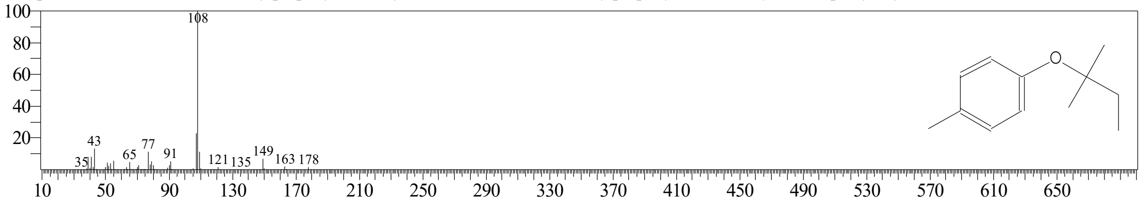

Hit#:2 Entry:67849 Library:NIST11.lib
SI:80 Formula:C15H22O2 CAS:59558-23-5 MolWeight:234 RetIndex:1770
CompName:para-Tolyl octanoate $$ p-Cresyl caprylate $$ n-Octanoic acid p-tolyl ester $$ Octanoic acid, 4-methylphenyl ester $$ p-Cresyl capryrate $$ p-Cresy

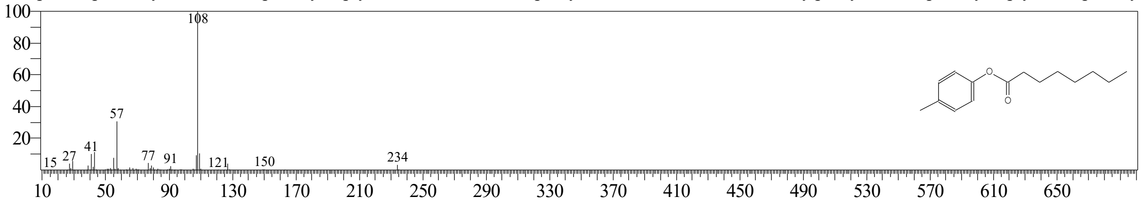

Hit#:3 Entry:101114 Library:NIST11.lib
SI:80 Formula:C16H22O4 CAS:0-00-0 MolWeight:278 RetIndex:2037
CompName:Oxalic acid, heptyl 2-methylphenyl ester

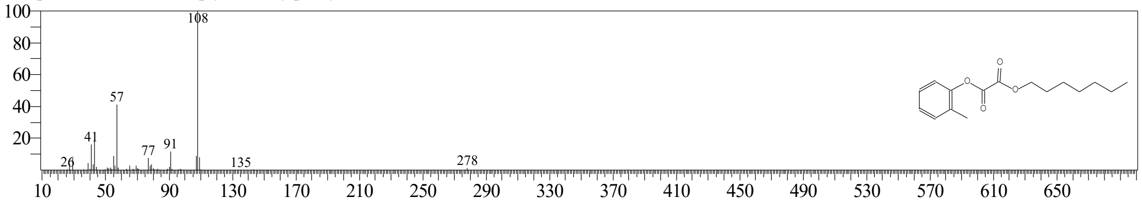

Hit#:4 Entry:15237 Library:NIST11.lib
SI:79 Formula:C9H10O2 CAS:140-39-6 MolWeight:150 RetIndex:1174
CompName:Acetic acid, 4-methylphenyl ester $$ Acetic acid, p-tolyl ester $$ p-Acetoxytoluene $$ p-Cresol acetate $$ p-Cresyl acetate $$ p-Methylphenyl aceta

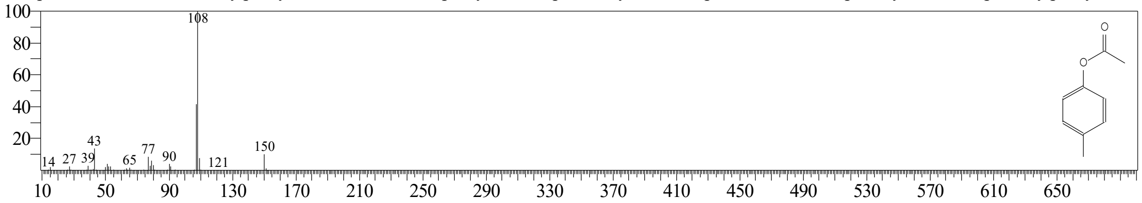

Hit#:5 Entry:112148 Library:NIST11.lib
SI:79 Formula:C17H24O4 CAS:0-00-0 MolWeight:292 RetIndex:2136
CompName:Oxalic acid, 2-methylphenyl octyl ester

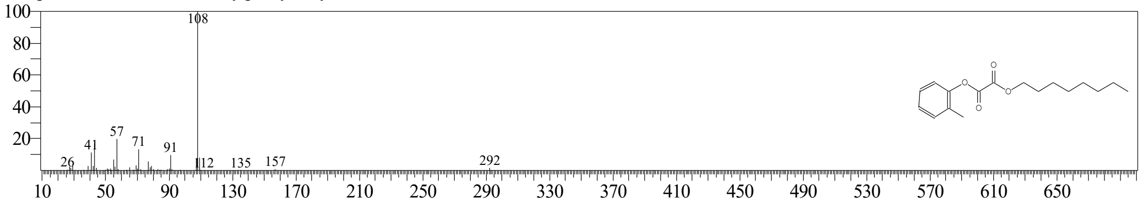

Qualitative Analysis Report

<< Target >>
Line#:92 R.Time:22.675(Scan#:3736) MassPeaks:375
RawMode:Averaged 22.670-22.680(3735-3737) BasePeak:70.05(70957)
BG Mode:Calc. from Peak Group 1 - Event 1 Scan

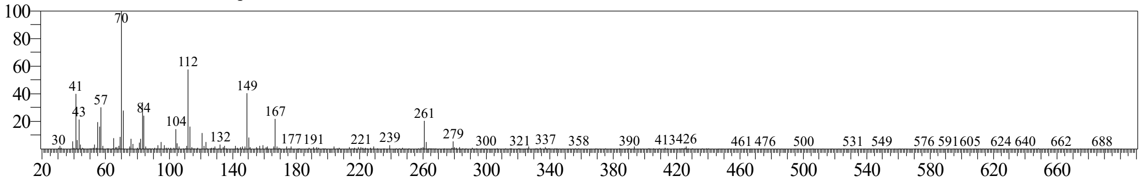

Hit#:1 Entry:192641 Library:NIST11.lib
SI:82 Formula:C27H44O4 CAS:0-00-0 MolWeight:432 RetIndex:3066
CompName:Terephthalic acid, 2-ethylhexyl undecyl ester

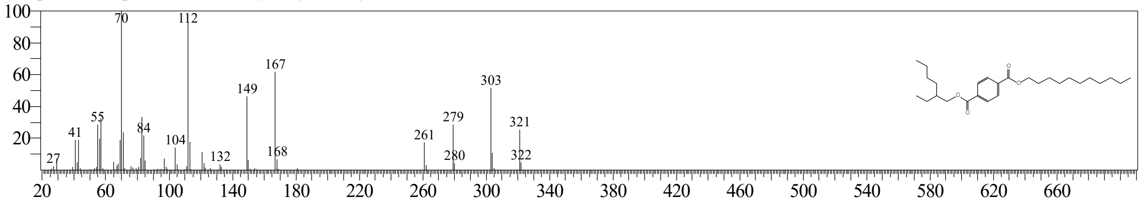

Hit#:2 Entry:196090 Library:NIST11.lib
SI:81 Formula:C28H46O4 CAS:0-00-0 MolWeight:446 RetIndex:3165
CompName:Terephthalic acid, dodecyl 2-ethylhexyl ester

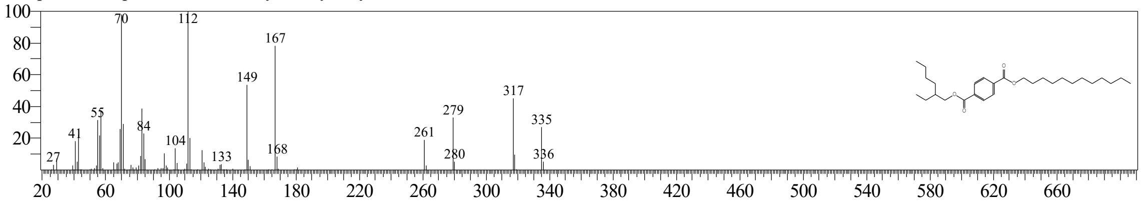

Hit#:3 Entry:178294 Library:NIST11.lib
SI:80 Formula:C24H38O4 CAS:6422-86-2 MolWeight:390 RetIndex:2704
CompName:1,4-Benzenedicarboxylic acid, bis(2-ethylhexyl) ester $$ Terephthalic acid, bis(2-ethylhexyl)ester $$ Bis(2-ethylhexyl) terephthalate $$ Kodaflex dc

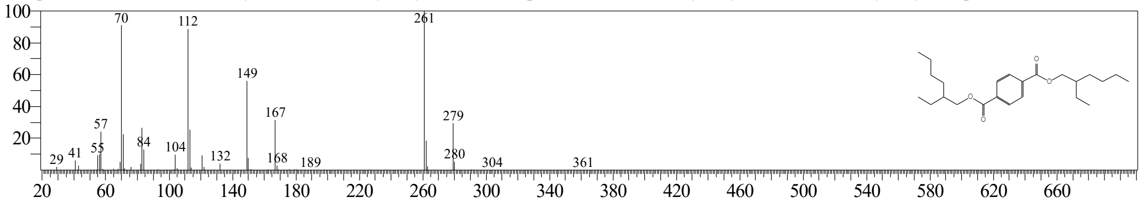

Hit#:4 Entry:188655 Library:NIST11.lib
SI:80 Formula:C26H42O4 CAS:0-00-0 MolWeight:418 RetIndex:2967
CompName:Terephthalic acid, decyl 2-ethylhexyl ester

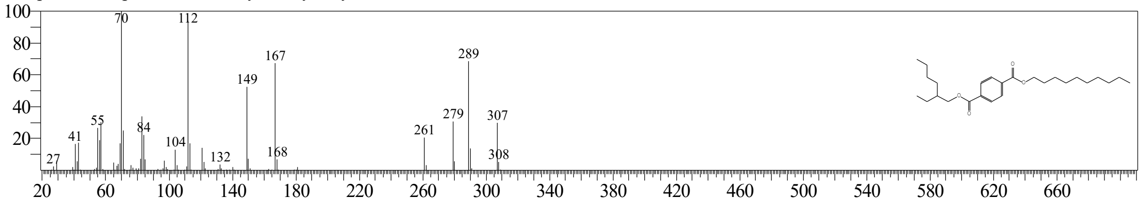

Hit#:5 Entry:178293 Library:NIST11.lib
SI:80 Formula:C24H38O4 CAS:0-00-0 MolWeight:390 RetIndex:2768
CompName:Terephthalic acid, 2-ethylhexyl octyl ester

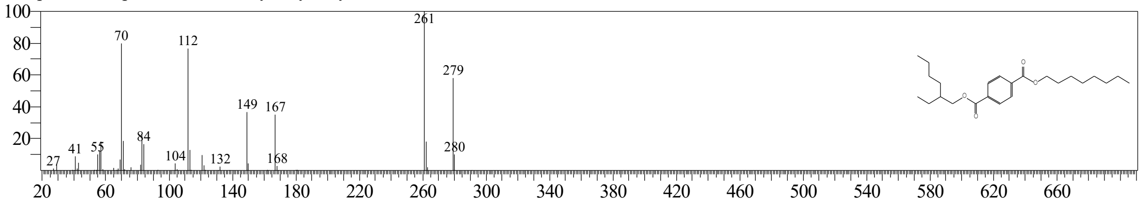

<< Target >>
Line#:93 R.Time:23.960(Scan#:3993) MassPeaks:368
RawMode:Averaged 23.955-23.965(3992-3994) BasePeak:411.40(4304)
BG Mode:Calc. from Peak Group 1 - Event 1 Scan

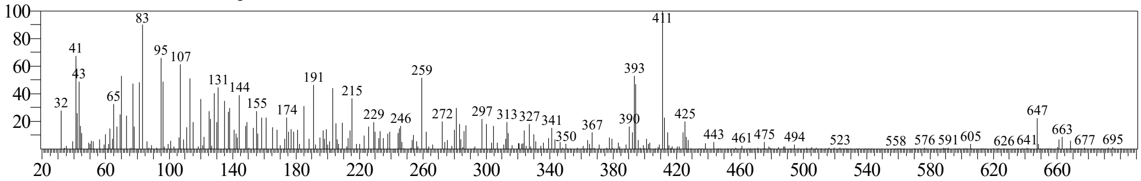

Hit#:1 Entry:191114 Library:NIST11.lib
SI:43 Formula:C30H50O CAS:35060-26-5 MolWeight:426 RetIndex:2737
CompName:D:B-Friedo-18,19-secolup-19-ene, 3,10-epoxy-, (3.beta.,10.beta.)- $$ Baccharis oxide $$

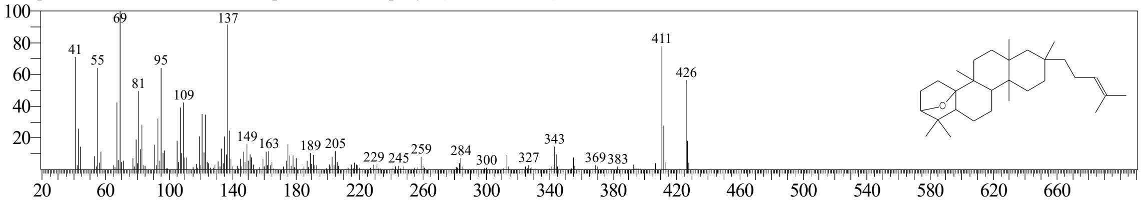

Hit#:2 Entry:127941 Library:NIST11.lib
SI:41 Formula:C19H20O4 CAS:0-00-0 MolWeight:312 RetIndex:2584
CompName:2,2,4,4-Tetramethyl-6-(1-oxo-3-phenylprop-2-enyl)-cyclohexane-1,3,5-trione

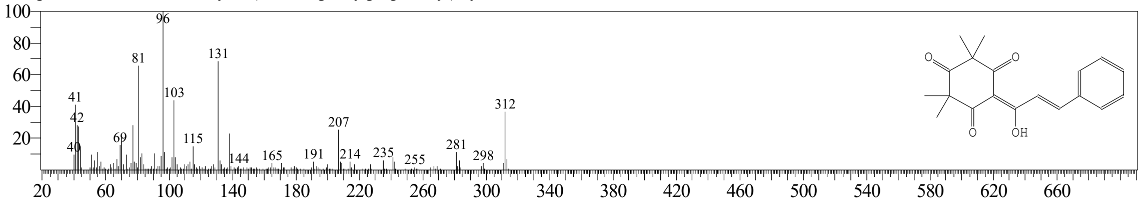

Hit#:3 Entry:191140 Library:NIST11.lib
SI:40 Formula:C30H50O CAS:5985-80-8 MolWeight:426 RetIndex:2788
CompName:Lanost-7-en-3-one

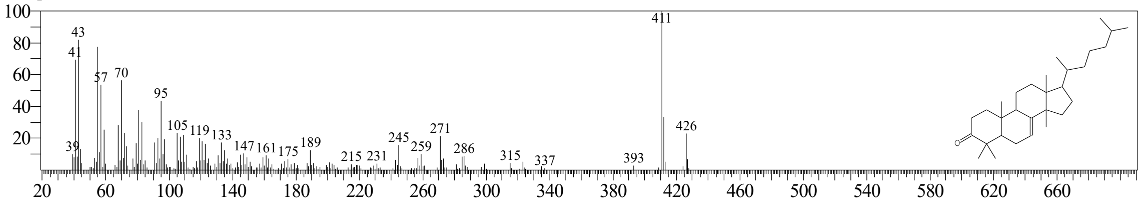

Hit#:4 Entry:191107 Library:NIST11.lib
SI:40 Formula:C30H50O CAS:79-63-0 MolWeight:426 RetIndex:2882
CompName:Lanosterol $$ Lanosta-8,24-dien-3-ol, (3.beta.)- $$ Lanosta-8,24-dien-3.beta.-ol $$ Botalan base 138 $$ Cholesta-8,24-dien-3-ol, 4,4,14-trimethyl-,

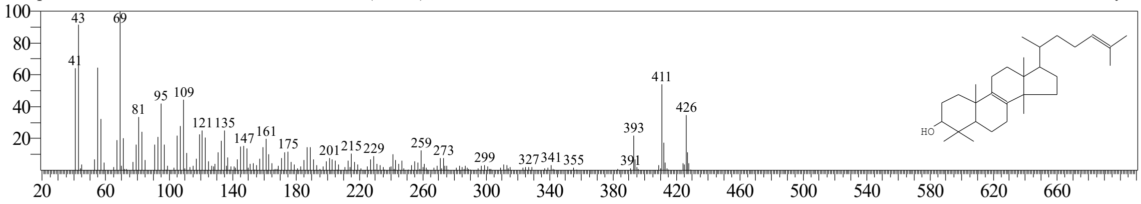

Hit#:5 Entry:24789 Library:NIST11.lib
SI:39 Formula:C11H20O CAS:0-00-0 MolWeight:168 RetIndex:1241
CompName:(3,7,7-Trimethyl-bicyclo[2.2.1]hept-2-yl)-methanol

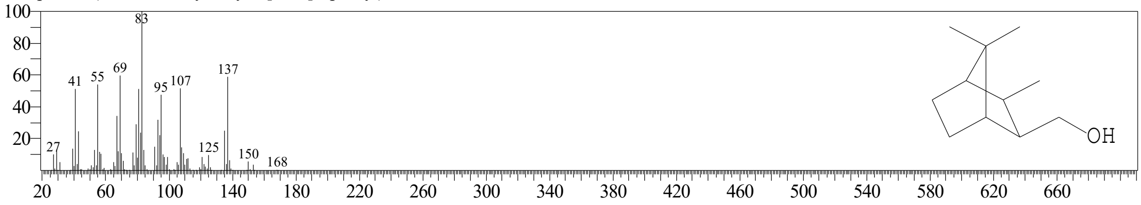

<< Target >>
Line#:94 R.Time:23.990(Scan#:3999) MassPeaks:371
RawMode:Averaged 23.985-23.995(3998-4000) BasePeak:69.05(6251)
BG Mode:Calc. from Peak Group 1 - Event 1 Scan

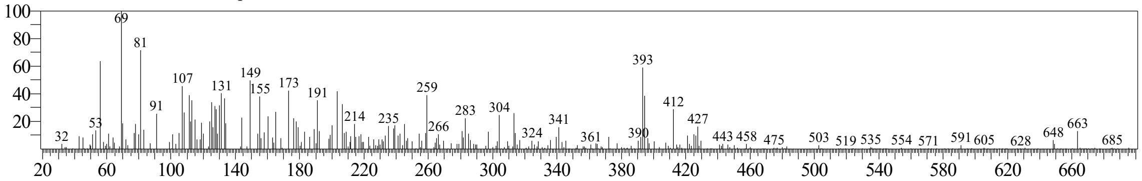

Hit#:1 Entry:155667 Library:NIST11.lib
SI:39 Formula:C21H28F2O2 CAS:90660-19-8 MolWeight:350 RetIndex:2144
CompName:Methyl 4,4-difluororetinoate (all-trans)

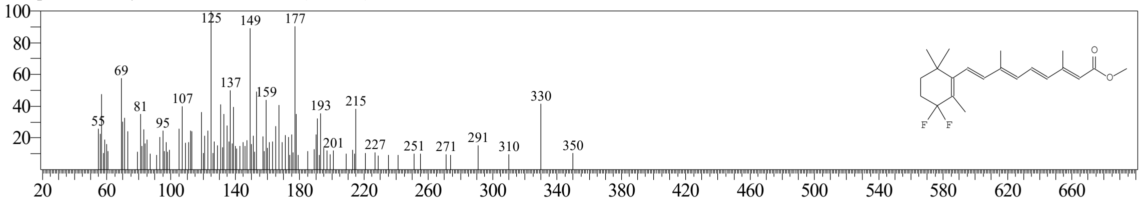

Hit#:2 Entry:47986 Library:NIST11.lib
SI:38 Formula:C14H22O CAS:125257-67-2 MolWeight:206 RetIndex:1553
CompName:1,2,3,4,5,6,7,8-Octahydro-2-naphthol, 4-methylene-2,5,5-trimethyl- $$ 2,5,5-Trimethyl-4-methylene-1,2,3,4,5,6,7,8-octahydro-2-naphthalenol # $$

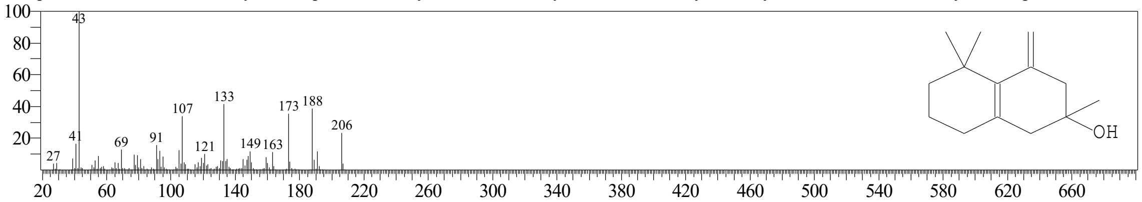

Hit#:3 Entry:84013 Library:NIST11.lib
SI:38 Formula:C14H24O4 CAS:0-00-0 MolWeight:256 RetIndex:2127
CompName:2,2-Dimethyl-6-methylene-1-[3,5-dihydroxy-1-pentenyl]cyclohexan-1-perhydrol $$ (4E)-5-(1-Hydroperoxy-2,2-dimethyl-6-methylenecyclohexyl)-4

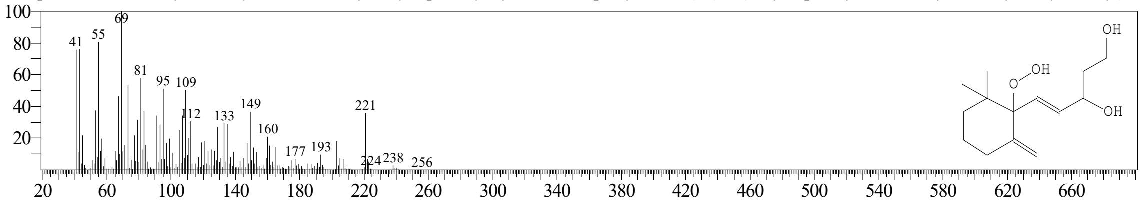

Hit#:4 Entry:196536 Library:NIST11.lib
SI:36 Formula:C28H48O4 CAS:56052-97-2 MolWeight:448 RetIndex:3179
CompName:Ergost-25-ene-3,5,6,12-tetrol, (3.beta.,5.alpha.,6.beta.,12.beta.)- $$ Ergost-25(27)-ene-3,5,6,12-tetrol # $$

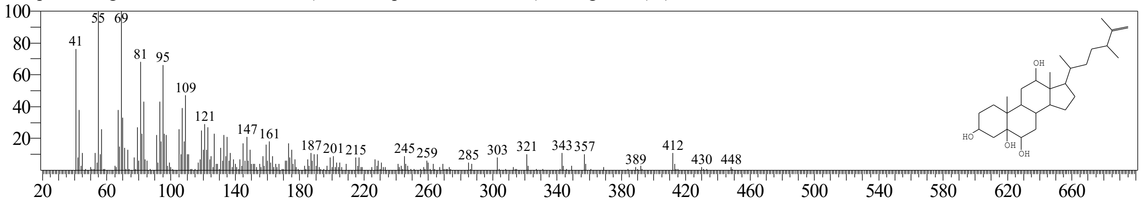

Hit#:5 Entry:47985 Library:NIST11.lib
SI:36 Formula:C14H22O CAS:125257-63-8 MolWeight:206 RetIndex:1473
CompName:Bicyclo[4.3.0]nonan-1-ol, 7,9-bis(methylene)-2,2,6-trimethyl- $$ 4,4,7a-Trimethyl-1,3-dimethyleneoctahydro-3ah-inden-3a-ol # $$

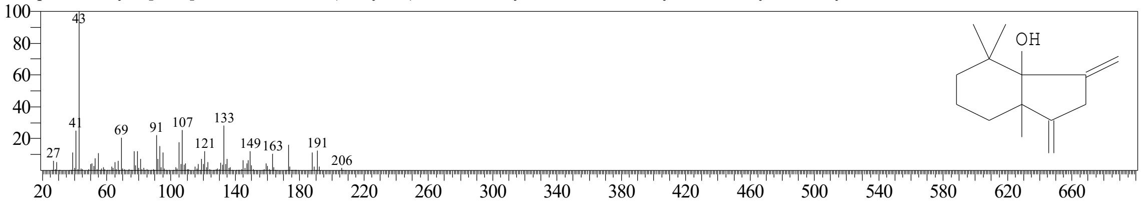

<< Target >>
Line#:95 R.Time:24.355(Scan#:4072) MassPeaks:361
RawMode:Averaged 24.350-24.360(4071-4073) BasePeak:69.10(165513)
BG Mode:Calc. from Peak Group 1 - Event 1 Scan

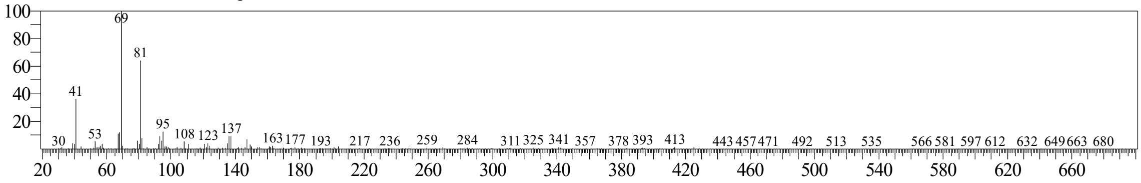

Hit#:1 Entry:186078 Library:NIST11.lib
SI:84 Formula:C30H50 CAS:111-02-4 MolWeight:410 RetIndex:2914
CompName:Squalene $$ 2,6,10,14,18,22-Tetracosahexaene, 2,6,10,15,19,23-hexamethyl-, (all-E)- $$ all-trans-Squalene $$ trans-Squalene $$ Spinacen $$ Spin

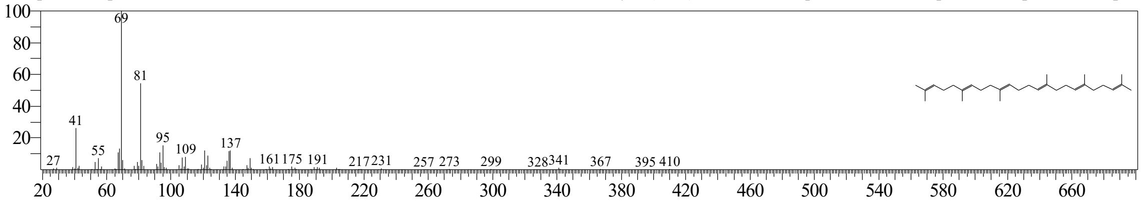

Hit#:2 Entry:78353 Library:NIST11.lib
SI:83 Formula:C17H28O CAS:66408-55-7 MolWeight:248 RetIndex:1855
CompName:4,8,12-Tetradecatrienal, 5,9,13-trimethyl- $$ (4E,8E)-5,9,13-Trimethyl-4,8,12-tetradecatrienal # $$

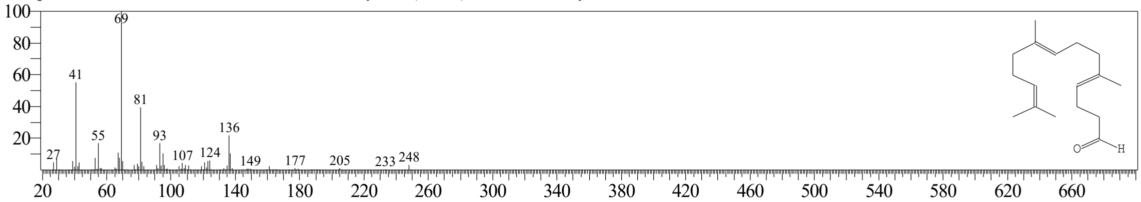

Hit#:3 Entry:38802 Library:NIST11.lib
SI:83 Formula:C14H24 CAS:62951-96-6 MolWeight:192 RetIndex:1350
CompName:1,5,9-Undecatriene, 2,6,10-trimethyl-, (Z)- $$ (5Z)-2,6,10-Trimethyl-1,5,9-undecatriene # $$

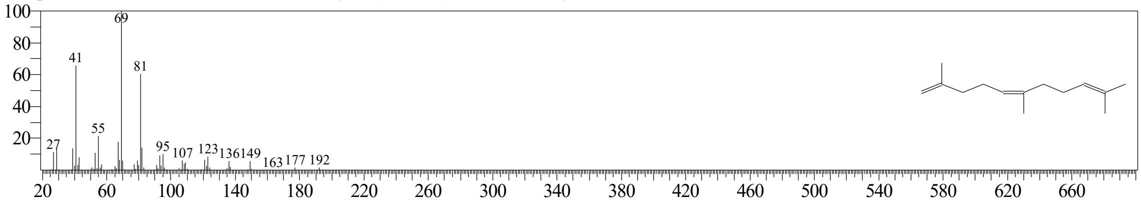

Hit#:4 Entry:65746 Library:NIST11.lib
SI:82 Formula:C16H25N CAS:6006-01-5 MolWeight:231 RetIndex:1812
CompName:3,7,11-Tridecatrienenitrile, 4,8,12-trimethyl- $$ Farnesyl cyanide $$ (3E,7E)-4,8,12-Trimethyl-3,7,11-tridecatrienenitrile # $$

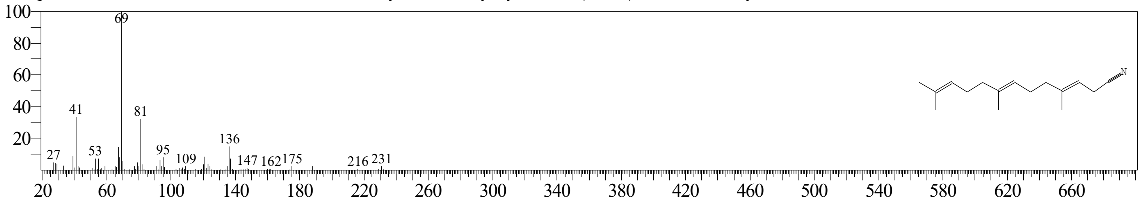

Hit#:5 Entry:175446 Library:NIST11.lib
SI:82 Formula:C27H44O CAS:0-00-0 MolWeight:384 RetIndex:2819
CompName:Docosa-2,6,10,14,18-pentaen-22-al, 2,6,10,15,18-pentamethyl-, all-trans $$ (4E,8E,12E,16E)-4,8,13,17,21-Pentamethyl-4,8,12,16,20-docosapentae

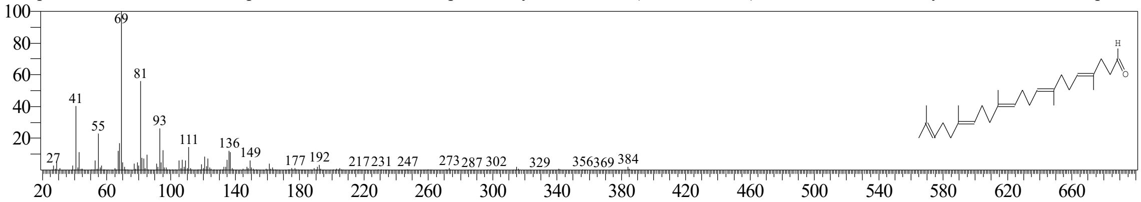

Qualitative Analysis Report

<< Target >>
Line#:96 R.Time:24.400(Scan#:4081) MassPeaks:301
RawMode:Averaged 24.395-24.405(4080-4082) BasePeak:147.10(4267)
BG Mode:Calc. from Peak Group 1 - Event 1 Scan

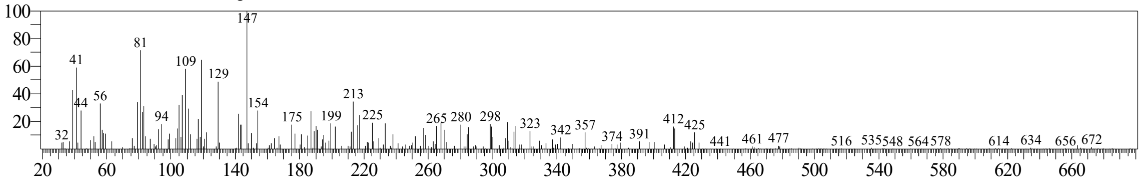

Hit#:1 Entry:37520 Library:NIST11.lib
SI:48 Formula:C13H18O CAS:35845-67-1 MolWeight:190 RetIndex:1315
CompName:3,6-Nonadien-5-one, 2,2,8,8-tetramethyl- $$ 3,6-Nonadiyn-5-one, 2,2,8,8-tetramethyl- $$ 2,2,8,8-Tetramethyl-3,6-nonadiyn-5-one # $$

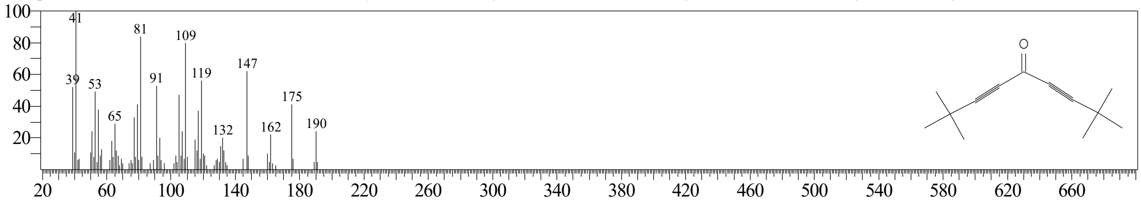

Hit#:2 Entry:69273 Library:NIST11.lib
SI:45 Formula:C15H24O2 CAS:51276-18-7 MolWeight:236 RetIndex:1926
CompName:5,6-Azulenedimethanol, 1,2,3,3a,8,8a-hexahydro-2,2,8-trimethyl-, (3a.alpha.,8.beta.,8a.alpha.)- $$ Vellerdiol $$

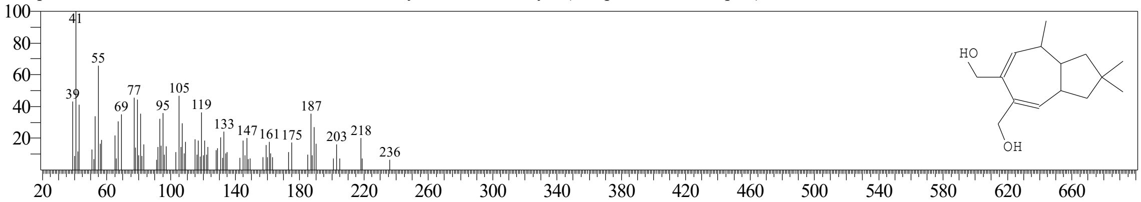

Hit#:3 Entry:76897 Library:NIST11.lib
SI:44 Formula:C18H30 CAS:78578-97-9 MolWeight:246 RetIndex:1365
CompName:Tetracyclo[6.1.0.0(2,4).0(5,7)]nonane, 3,6,9-triethyl-3,6,9-trimethyl-

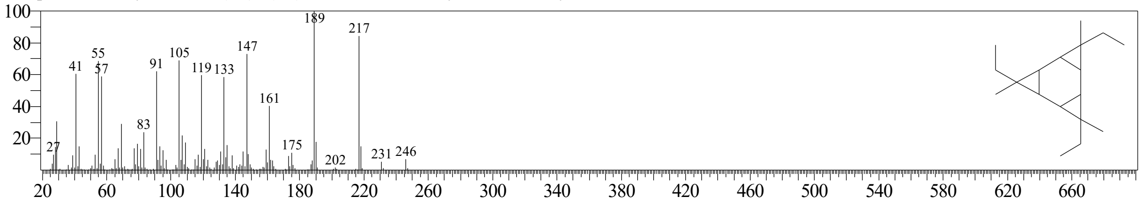

Hit#:4 Entry:31726 Library:NIST11.lib
SI:44 Formula:C12H20O CAS:4808-01-9 MolWeight:180 RetIndex:1465
CompName:2-Propen-1-ol, 3-(2,6,6-trimethyl-1-cyclohexen-1-yl)- $$ .beta.-Citrylideneethanol $$ (2E)-3-(2,6,6-Trimethyl-1-cyclohexen-1-yl)-2-propen-1-ol # $

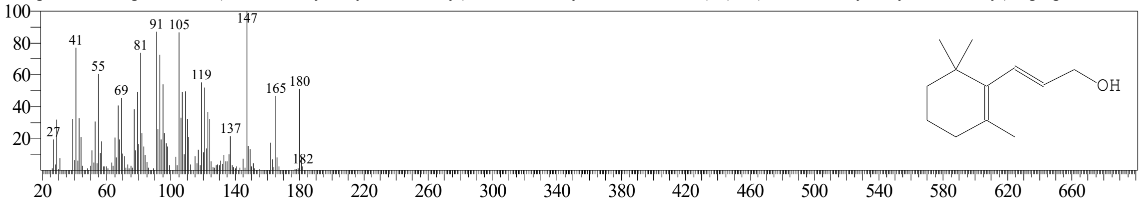

Hit#:5 Entry:76820 Library:NIST11.lib
SI:44 Formula:C16H22O2 CAS:0-00-0 MolWeight:246 RetIndex:1833
CompName:3,3-Dimethyl-1-(5-phenyl-tetrahydrofuran-2-yl)-butan-2-one $$ 3,3-Dimethyl-1-(5-phenyltetrahydro-2-furanyl)-2-butanone # $$

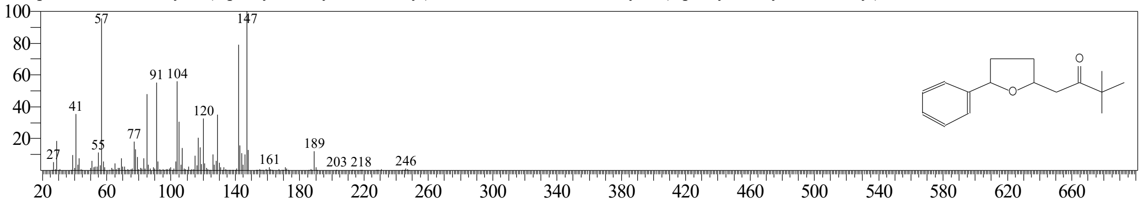

Qualitative Analysis Report

<< Target >>
Line#:97 R.Time:24.500(Scan#:4101) MassPeaks:387
RawMode:Averaged 24.495-24.505(4100-4102) BasePeak:109.10(25275)
BG Mode:Calc. from Peak Group 1 - Event 1 Scan

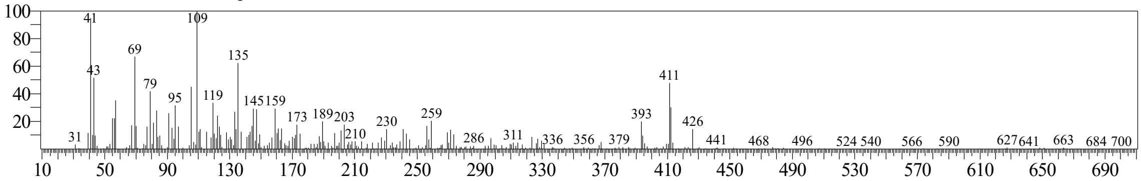

Hit#:1 Entry:191107 Library:NIST11.lib
SI:74 Formula:C30H50O CAS:79-63-0 MolWeight:426 RetIndex:2882
CompName:Lanosterol $$ Lanosta-8,24-dien-3-ol, (3.beta.)- $$ Lanosta-8,24-dien-3.beta.-ol $$ Botalan base 138 $$ Cholesta-8,24-dien-3-ol, 4,4,14-trimethyl-,

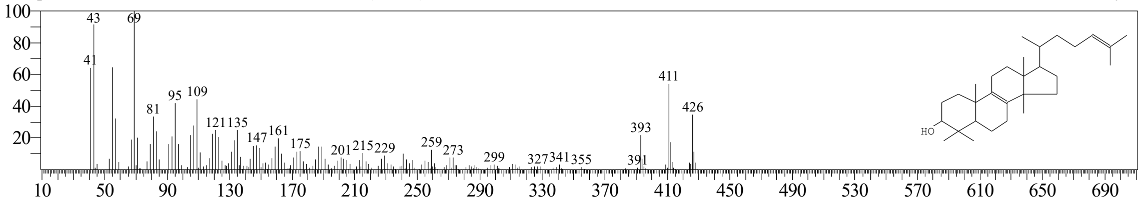

Hit#:2 Entry:191106 Library:NIST11.lib
SI:69 Formula:C30H50O CAS:469-38-5 MolWeight:426 RetIndex:2816
CompName:9,19-Cyclolanost-24-en-3-ol, (3.beta.)- $$ 9,19-Cyclo-9.beta.-lanost-24-en-3.beta.-ol $$ Cycloartenol $$ Handianol $$ 1-(1,5-Dimethyl-4-hexenyl)-

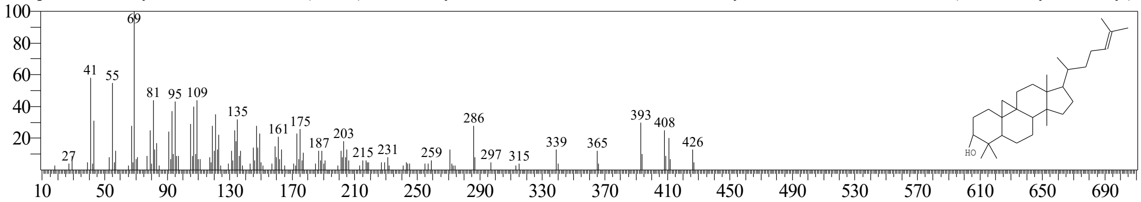

Hit#:3 Entry:191101 Library:NIST11.lib
SI:69 Formula:C30H50O CAS:469-39-6 MolWeight:426 RetIndex:2760
CompName:9,19-Cycloergost-24(28)-en-3-ol, 4,14-dimethyl-, (3.beta.,4.alpha.,5.alpha.)- $$ 9,19-Cyclo-5.alpha.,9.beta.-ergost-24(28)-en-3.beta.-ol, 4.alpha.,14

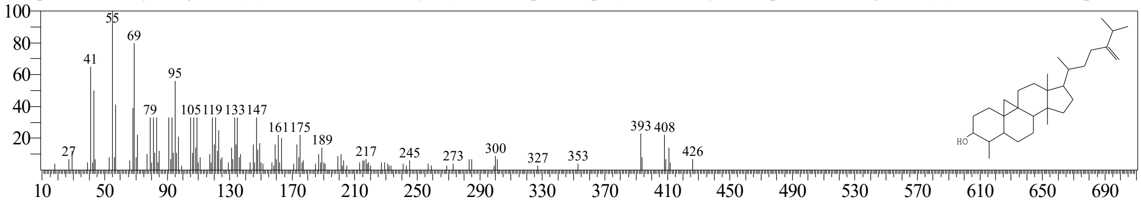

Hit#:4 Entry:191141 Library:NIST11.lib
SI:69 Formula:C30H50O CAS:16910-32-0 MolWeight:426 RetIndex:2826
CompName:Obtusifoliol $$ Ergosta-8,24(28)-dien-3-ol, 4,14-dimethyl-, (3.beta.,4.alpha.,5.alpha.)- $$ 5.alpha.-Ergosta-8,24(28)-dien-3.beta.-ol, 4.alpha.,14-din

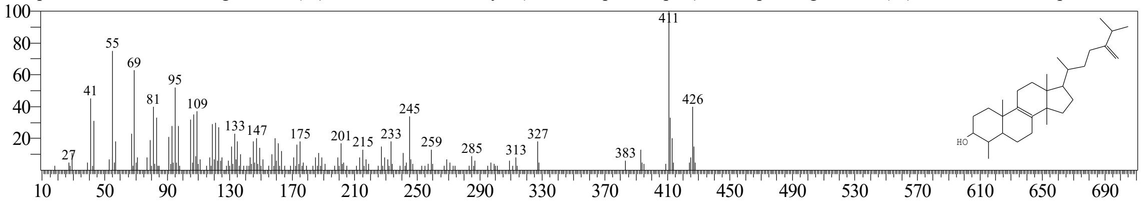

Hit#:5 Entry:195684 Library:NIST11.lib
SI:68 Formula:C30H52O2 CAS:26525-84-8 MolWeight:444 RetIndex:2923
CompName:9,19-Cyclo-9.beta.-lanostane-3.beta.,25-diol $$ Cycloartane-3.beta.,25-diol $$ 1-(5-Hydroxy-1,5-dimethylhexyl)-3a,6,6,12a-tetramethyltetradecahy

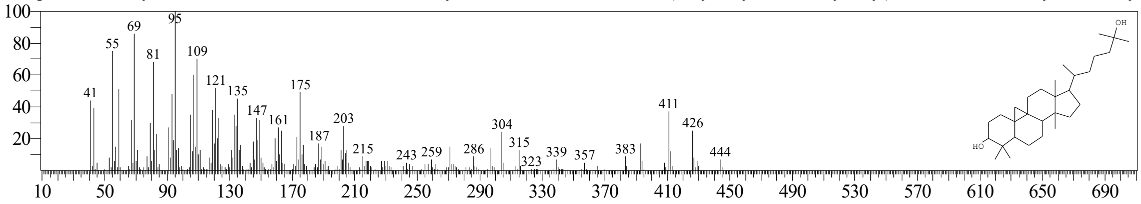

Qualitative Analysis Report

<< Target >>
Line#:98 R.Time:24.745(Scan#:4150) MassPeaks:370
RawMode:Averaged 24.740-24.750(4149-4151) BasePeak:55.05(23781)
BG Mode:Calc. from Peak Group 1 - Event 1 Scan

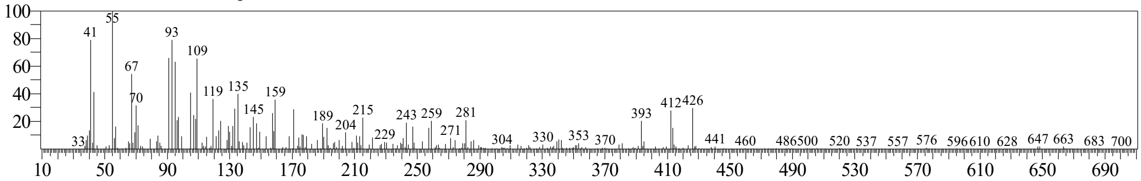

Hit#:1 Entry:191107 Library:NIST11.lib
SI:64 Formula:C30H50O CAS:79-63-0 MolWeight:426 RetIndex:2882
CompName:Lanosterol $$ Lanosta-8,24-dien-3-ol, (3.beta.)- $$ Lanosta-8,24-dien-3.beta.-ol $$ Botalan base 138 $$ Cholesta-8,24-dien-3-ol, 4,4,14-trimethyl-,

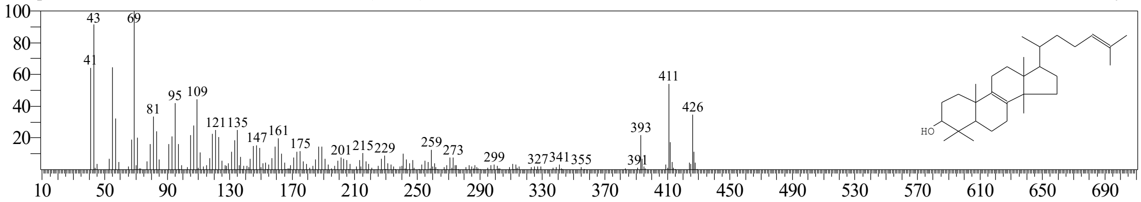

Hit#:2 Entry:196536 Library:NIST11.lib
SI:63 Formula:C28H48O4 CAS:56052-97-2 MolWeight:448 RetIndex:3179
CompName:Ergost-25-ene-3,5,6,12-tetrol, (3.beta.,5.alpha.,6.beta.,12.beta.)- $$ Ergost-25(27)-ene-3,5,6,12-tetrol # $$

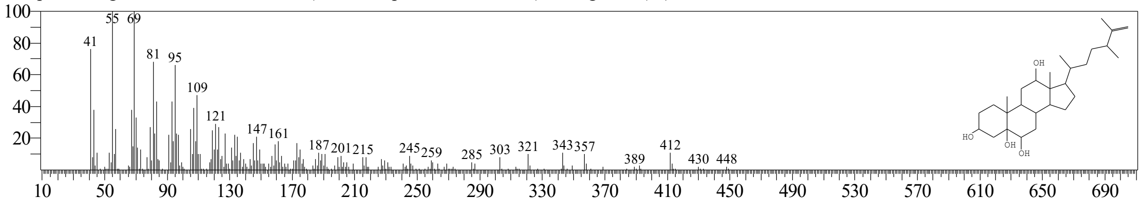

Hit#:3 Entry:69283 Library:NIST11.lib
SI:62 Formula:C15H24O2 CAS:0-00-0 MolWeight:236 RetIndex:1729
CompName:Murolan-3,9(11)-diene-10-peroxy $$ 8-Isopropyl-5-methyl-2-methylene-1,2,3,4,4a,5,8,8a-octahydro-1-naphthalenyl hydroperoxide # $$

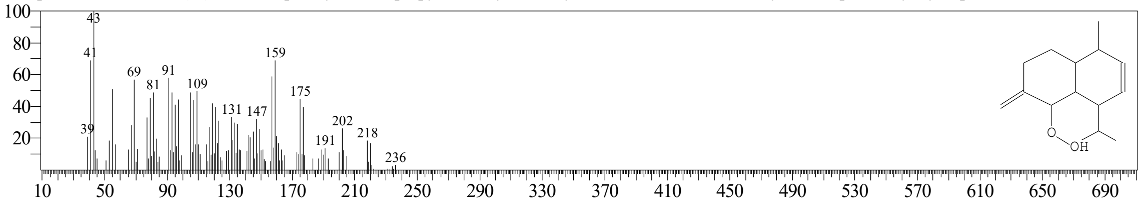

Hit#:4 Entry:186823 Library:NIST11.lib
SI:62 Formula:C29H48O CAS:83-48-7 MolWeight:412 RetIndex:2739
CompName:Stigmasterol $$ Stigmasta-5,22-dien-3-ol, (3.beta.,22E)- $$ Stigmasta-5,22-dien-3.beta.-ol $$ .beta.-Stigmasterol $$ (24S)-5,22-Stigmastadien-3.bet

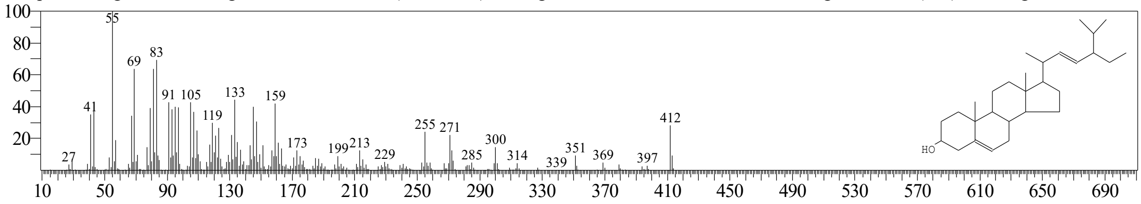

Hit#:5 Entry:186819 Library:NIST11.lib
SI:62 Formula:C29H48O CAS:2604-90-2 MolWeight:412 RetIndex:2714
CompName:Cholest-7-en-3-one, 4,4-dimethyl-, (5.alpha.)- $$ 5.alpha.-Cholest-7-en-3-one, 4,4-dimethyl- $$ 4,4-Dimethylcholest-7-en-3-one # $$

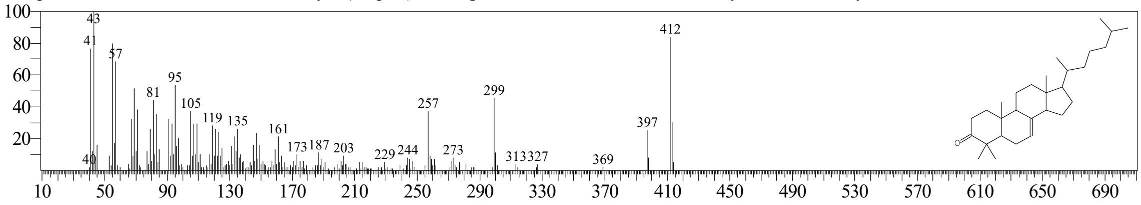

<< Target >>
Line#:99 R.Time:24.820(Scan#:4165) MassPeaks:355
RawMode:Averaged 24.815-24.825(4164-4166) BasePeak:69.05(17833)
BG Mode:Calc. from Peak Group 1 - Event 1 Scan

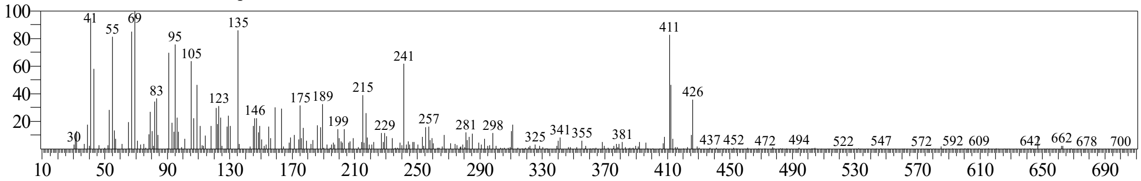

Hit#:1 Entry:191107 Library:NIST11.lib
SI:65 Formula:C30H50O CAS:79-63-0 MolWeight:426 RetIndex:2882
CompName:Lanosterol $$ Lanosta-8,24-dien-3-ol, (3.beta.)- $$ Lanosta-8,24-dien-3.beta.-ol $$ Botalan base 138 $$ Cholesta-8,24-dien-3-ol, 4,4,14-trimethyl-,

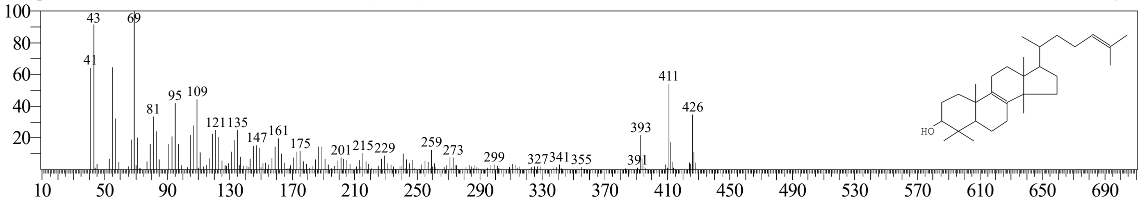

Hit#:2 Entry:191114 Library:NIST11.lib
SI:64 Formula:C30H50O CAS:35060-26-5 MolWeight:426 RetIndex:2737
CompName:D:B-Friedo-18,19-secolup-19-ene, 3,10-epoxy-, (3.beta.,10.beta.)- $$ Baccharis oxide $$

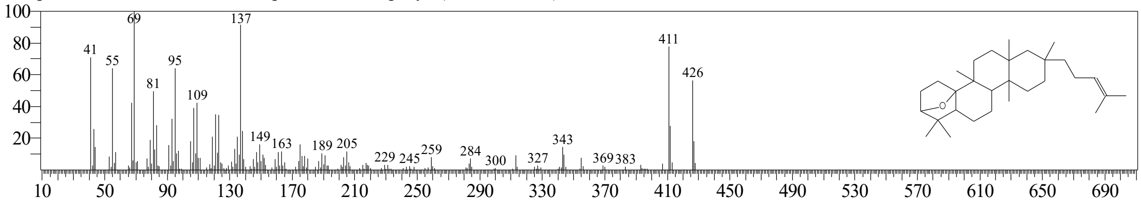

Hit#:3 Entry:191141 Library:NIST11.lib
SI:63 Formula:C30H50O CAS:16910-32-0 MolWeight:426 RetIndex:2826
CompName:Obtusifoliol $$ Ergosta-8,24(28)-dien-3-ol, 4,14-dimethyl-, (3.beta.,4.alpha.,5.alpha.)- $$ 5.alpha.-Ergosta-8,24(28)-dien-3.beta.-ol, 4.alpha.,14-din

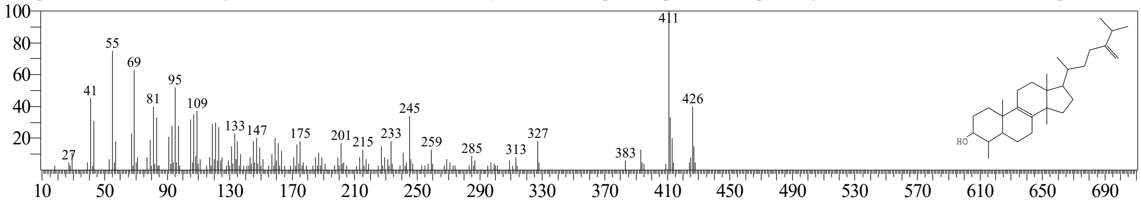

Hit#:4 Entry:48079 Library:NIST11.lib
SI:62 Formula:C15H26 CAS:0-00-0 MolWeight:206 RetIndex:1426
CompName:Caparratriene

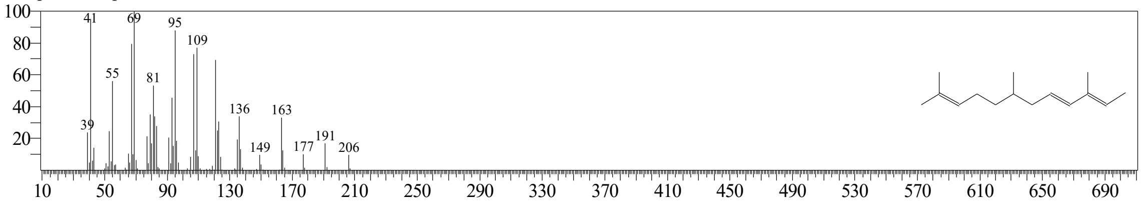

Hit#:5 Entry:48080 Library:NIST11.lib
SI:62 Formula:C15H26 CAS:172549-29-0 MolWeight:206 RetIndex:1426
CompName:(2Z,4E)-3,7,11-Trimethyl-2,4,10-dodecatriene

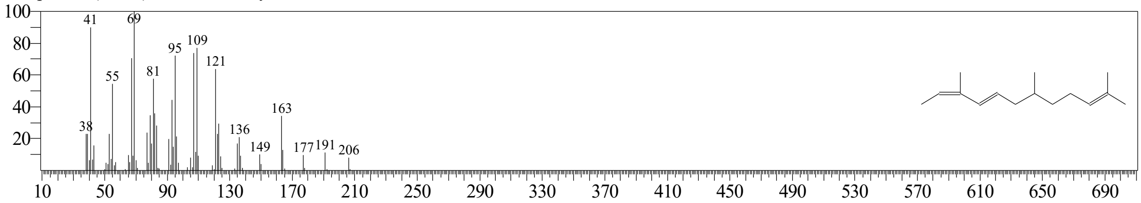

Qualitative Analysis Report

<< Target >>
Line#:100 R.Time:24.875(Scan#:4176) MassPeaks:365
RawMode:Averaged 24.870-24.880(4175-4177) BasePeak:411.30(17696)
BG Mode:Calc. from Peak Group 1 - Event 1 Scan

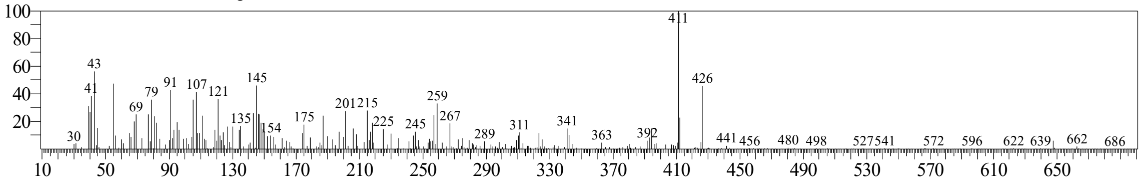

Hit#:1 Entry:191141 Library:NIST11.lib
SI:59 Formula:C30H50O CAS:16910-32-0 MolWeight:426 RetIndex:2826
CompName:Obtusifoliol $$ Ergosta-8,24(28)-dien-3-ol, 4,14-dimethyl-, (3.beta.,4.alpha.,5.alpha.)- $$ 5.alpha.-Ergosta-8,24(28)-dien-3.beta.-ol, 4.alpha.,14-din

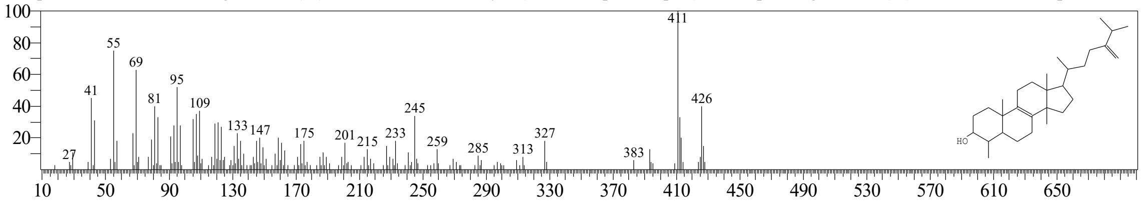

Hit#:2 Entry:191145 Library:NIST11.lib
SI:58 Formula:C30H50O CAS:0-00-0 MolWeight:426 RetIndex:2857
CompName:4.alpha.,14-Dimethyl-5.alpha.-ergosta-8,24(28)-dien-3.beta.-ol $$ 4,14-Dimethylergosta-5,24-dien-3-ol # $$

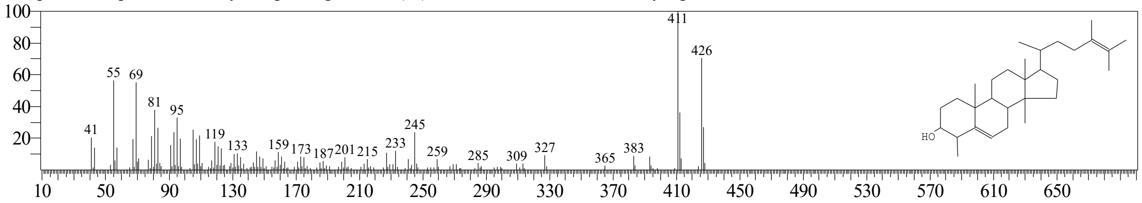

Hit#:3 Entry:191107 Library:NIST11.lib
SI:57 Formula:C30H50O CAS:79-63-0 MolWeight:426 RetIndex:2882
CompName:Lanosterol $$ Lanosta-8,24-dien-3-ol, (3.beta.)- $$ Lanosta-8,24-dien-3.beta.-ol $$ Botalan base 138 $$ Cholesta-8,24-dien-3-ol, 4,4,14-trimethyl-,

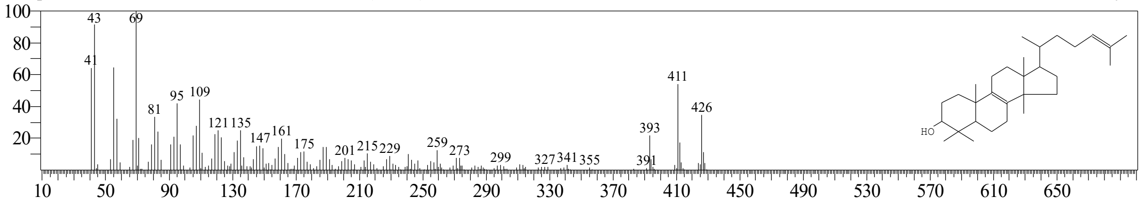

Hit#:4 Entry:191140 Library:NIST11.lib
SI:57 Formula:C30H50O CAS:5985-80-8 MolWeight:426 RetIndex:2788
CompName:Lanost-7-en-3-one

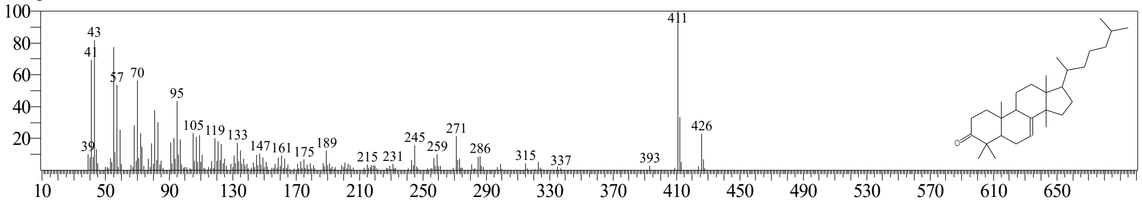

Hit#:5 Entry:131287 Library:NIST11.lib
SI:57 Formula:C21H32O2 CAS:10178-35-5 MolWeight:316 RetIndex:2176
CompName:1-Naphthalenecarboxylic acid, decahydro-1,4a-dimethyl-6-methylene-5-(3-methyl-2,4-pentadienyl)-, methyl ester, [1S-[1.alpha.,4a.alpha.,5.alpha.(Z

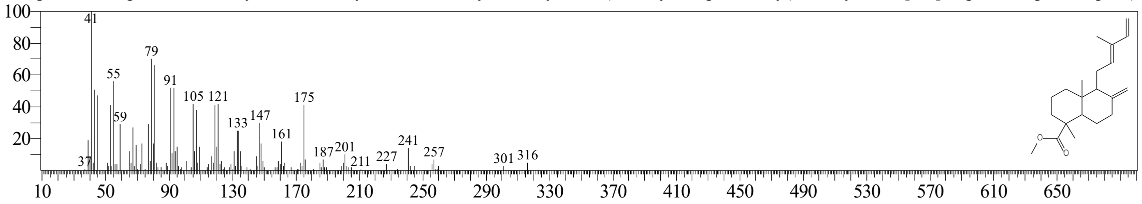

Qualitative Analysis Report

<< Target >>
Line#:101 R.Time:25.020(Scan#:4205) MassPeaks:398
RawMode:Averaged 25.015-25.025(4204-4206) BasePeak:69.05(76265)
BG Mode:Calc. from Peak Group 1 - Event 1 Scan

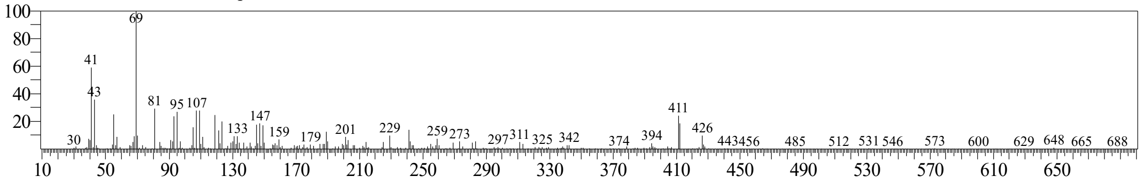

Hit#:1 Entry:191107 Library:NIST11.lib
SI:74 Formula:C30H50O CAS:79-63-0 MolWeight:426 RetIndex:2882
CompName:Lanosterol $$ Lanosta-8,24-dien-3-ol, (3.beta.)- $$ Lanosta-8,24-dien-3.beta.-ol $$ Botalan base 138 $$ Cholesta-8,24-dien-3-ol, 4,4,14-trimethyl-,

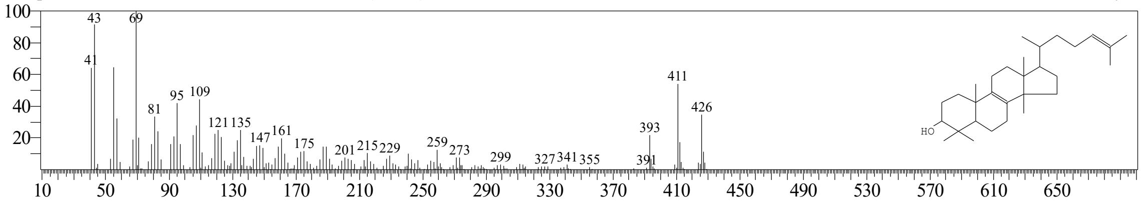

Hit#:2 Entry:200398 Library:NIST11.lib
SI:68 Formula:C32H52O2 CAS:1259-10-5 MolWeight:468 RetIndex:2956
CompName:9,19-Cyclolanost-24-en-3-ol, acetate, (3.beta.)- $$ 9,19-Cyclo-9.beta.-lanost-24-en-3.beta.-ol, acetate $$ Cycloartenol acetate $$ Cycloartenyl aceta

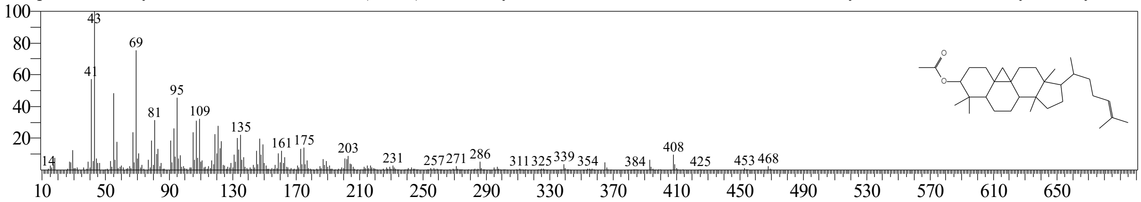

Hit#:3 Entry:191111 Library:NIST11.lib
SI:68 Formula:C30H50O CAS:54159-46-5 MolWeight:426 RetIndex:3058
CompName:1,6,10,14,18,22-Tetracosahexaen-3-ol, 2,6,10,15,19,23-hexamethyl-, (all-E)- $$ (6E,10E,14E,18E)-2,6,10,15,19,23-Hexamethyl-1,6,10,14,18,22-te

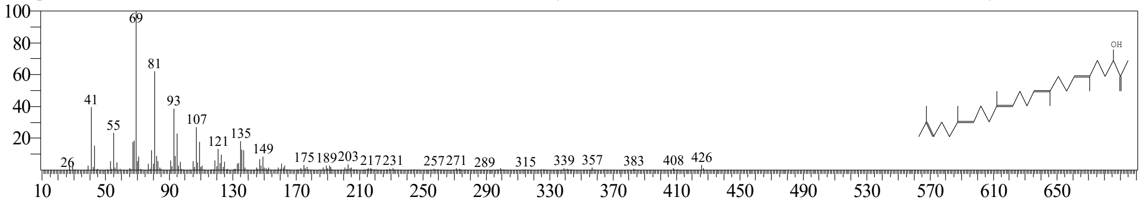

Hit#:4 Entry:194758 Library:NIST11.lib
SI:68 Formula:C30H48O2 CAS:0-00-0 MolWeight:440 RetIndex:3018
CompName:2-(3-Hydroxy-4,4,10,13,14-pentamethyl-2,3,4,5,6,7,10,11,12,13,14,15,16,17-tetradecahydro-1H-cyclopenta[a]phenanthren-17-yl)-6-methyl-5-hepte

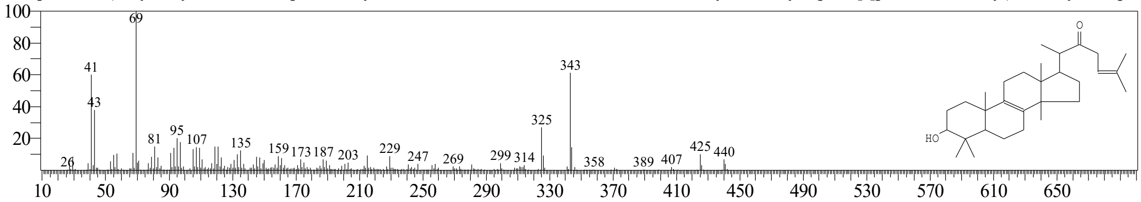

Hit#:5 Entry:96989 Library:NIST11.lib
SI:68 Formula:C20H32 CAS:77898-97-6 MolWeight:272 RetIndex:1940
CompName:(E,E,E)-3,7,11,15-Tetramethylhexadeca-1,3,6,10,14-pentaene $$ (3E,6E,10E)-3,7,11,15-Tetramethyl-1,3,6,10,14-hexadecapentaene $$ (E,E,E)- .alp

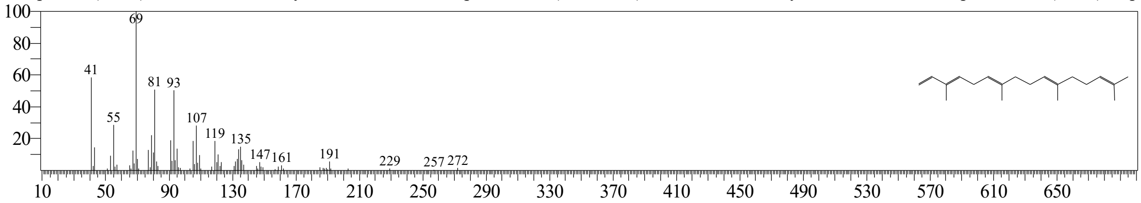

Qualitative Analysis Report

<< Target >>
Line#:102 R.Time:25.070(Scan#:4215) MassPeaks:351
RawMode:Averaged 25.065-25.075(4214-4216) BasePeak:107.10(15130)
BG Mode:Calc. from Peak Group 1 - Event 1 Scan

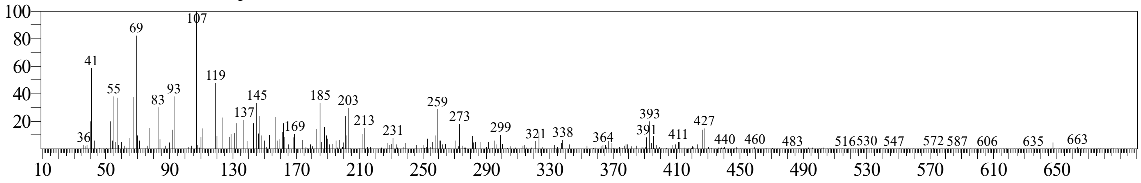

Hit#:1 Entry:208642 Library:NIST11.lib
SI:55 Formula:C40H58O CAS:105-92-0 MolWeight:554 RetIndex:4025
CompName:Rhodopin $$ .psi.,.psi.-Carotene, 1,2-dihydro-1-hydroxy- $$ Lycopene, 1,2-dihydro-1-hydroxy- $$ Lycopene, 1,2-dihydro-1-hydroxy-, all-trans- $$

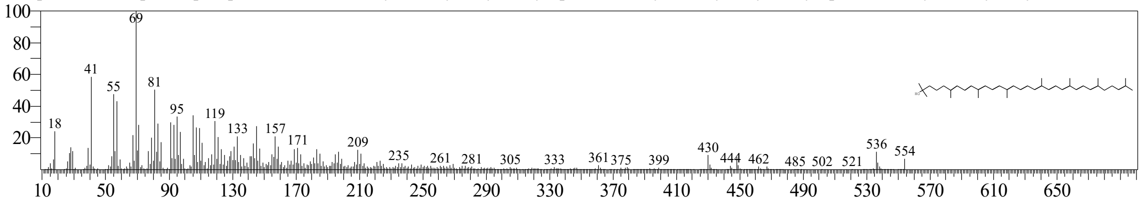

Hit#:2 Entry:69278 Library:NIST11.lib
SI:54 Formula:C15H24O2 CAS:0-00-0 MolWeight:236 RetIndex:1904
CompName:Bicyclo[4.4.0]dec-2-ene-4-ol, 2-methyl-9-(prop-1-en-3-ol-2-yl)- $$ 6-[1-(Hydroxymethyl)vinyl]-4,8a-dimethyl-1,2,4a,5,6,7,8,8a-octahydro-2-naphtl

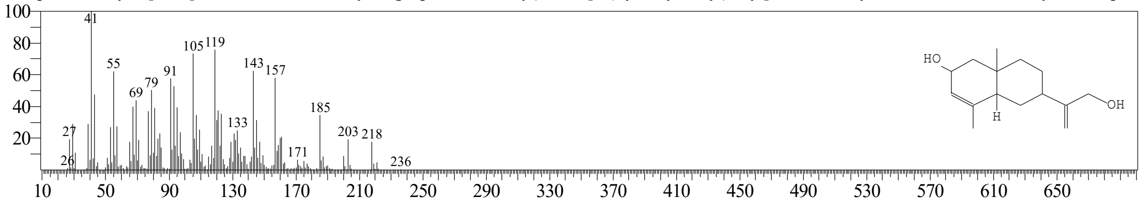

Hit#:3 Entry:140323 Library:NIST11.lib
SI:52 Formula:C21H28O3 CAS:0-00-0 MolWeight:328 RetIndex:2706
CompName:2-(7-Hydroxymethyl-3,11-dimethyl-dodeca-2,6,10-trienyl)-[1,4]benzoquinone $$ 2-[(2E,6Z)-7-(Hydroxymethyl)-3,11-dimethyl-2,6,10-dodecatrieny

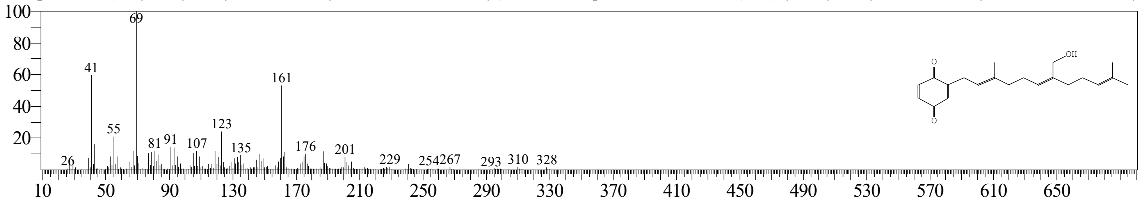

Hit#:4 Entry:96984 Library:NIST11.lib
SI:52 Formula:C20H32 CAS:75349-96-1 MolWeight:272 RetIndex:1463
CompName:Pentacyclo[9.1.0.0(2,4).0(5,7).0(8,10)]dodecane, 3,3,6,6,9,9,12,12-octamethyl-, anti,anti,anti-

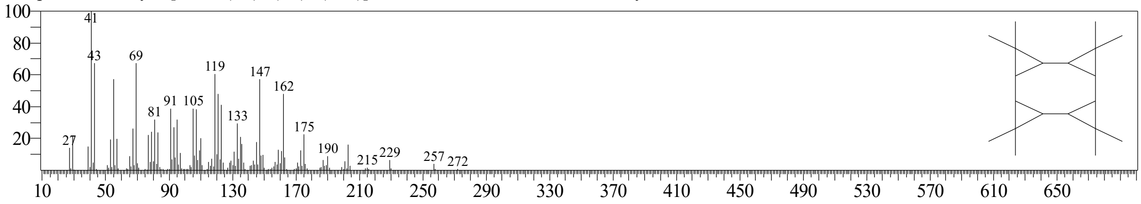

Hit#:5 Entry:207596 Library:NIST11.lib
SI:52 Formula:C40H56 CAS:36516-73-1 MolWeight:536 RetIndex:3949
CompName:.beta.,.epsilon.-Carotene, neo $$ 4',5'-Didehydro-5',6'-dihydro-beta,beta-carotene # $$

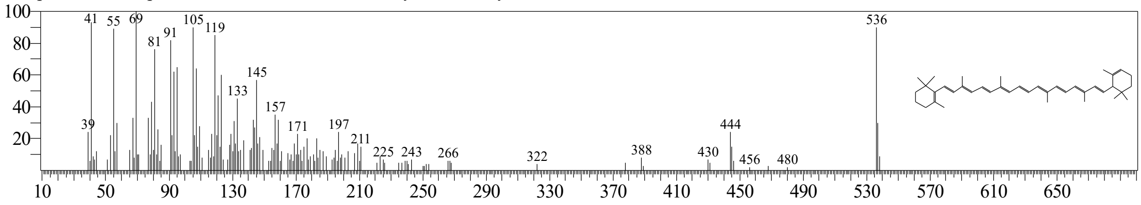

<< Target >>
Line#:103 R.Time:25.235(Scan#:4248) MassPeaks:509
RawMode:Averaged 25.230-25.240(4247-4249) BasePeak:69.10(365495)
BG Mode:Calc. from Peak Group 1 - Event 1 Scan

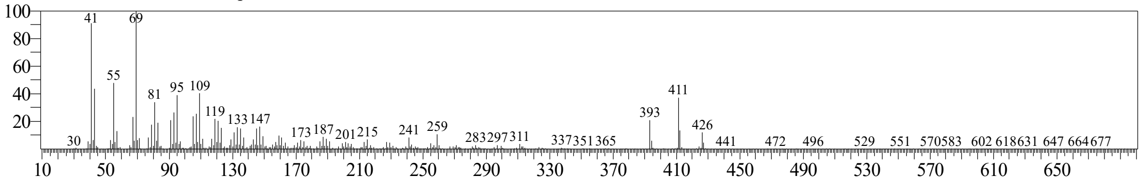

Hit#:1 Entry:191107 Library:NIST11.lib
SI:86 Formula:C30H50O CAS:79-63-0 MolWeight:426 RetIndex:2882
CompName:Lanosterol $$ Lanosta-8,24-dien-3-ol, (3.beta.)- $$ Lanosta-8,24-dien-3.beta.-ol $$ Botalan base 138 $$ Cholesta-8,24-dien-3-ol, 4,4,14-trimethyl-,

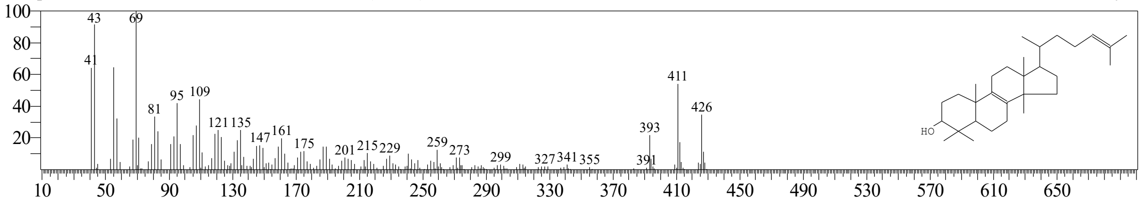

Hit#:2 Entry:200403 Library:NIST11.lib
SI:79 Formula:C32H52O2 CAS:2671-68-3 MolWeight:468 RetIndex:3022
CompName:Lanosta-8,24-dien-3-ol, acetate, (3.beta.)- $$ Lanosta-8,24-dien-3.beta.-ol, acetate $$ Lanosta-8,24-dien-3.beta.-yl acetate $$ Lanosterol acetate $$ I

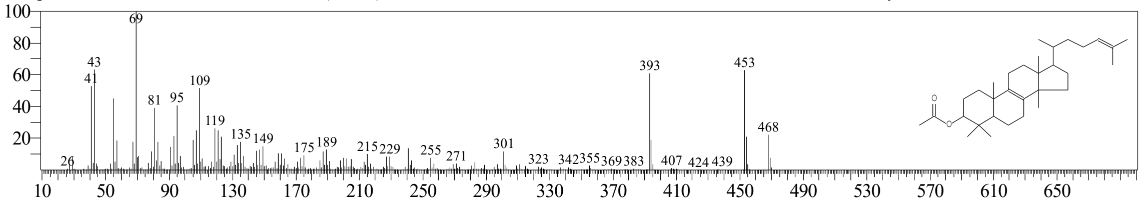

Hit#:3 Entry:191096 Library:NIST11.lib
SI:79 Formula:C30H50O CAS:55515-18-9 MolWeight:426 RetIndex:2788
CompName:Lanost-7-en-3-one, (9.beta.,13.alpha.,14.beta.,17.alpha.)- $$ Lanost-7-en-3-one # $$

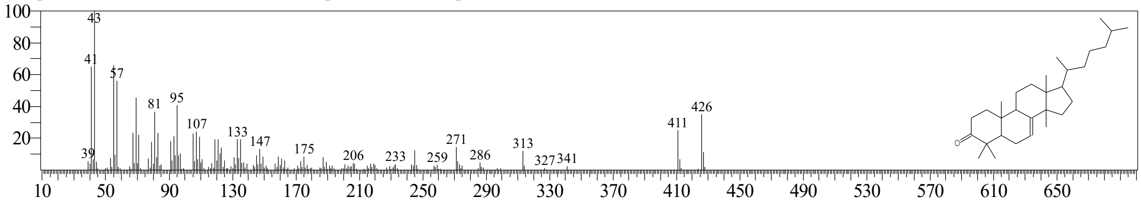

Hit#:4 Entry:200398 Library:NIST11.lib
SI:77 Formula:C32H52O2 CAS:1259-10-5 MolWeight:468 RetIndex:2956
CompName:9,19-Cyclolanost-24-en-3-ol, acetate, (3.beta.)- $$ 9,19-Cyclo-9.beta.-lanost-24-en-3.beta.-ol, acetate $$ Cycloartenol acetate $$ Cycloartenyl aceta

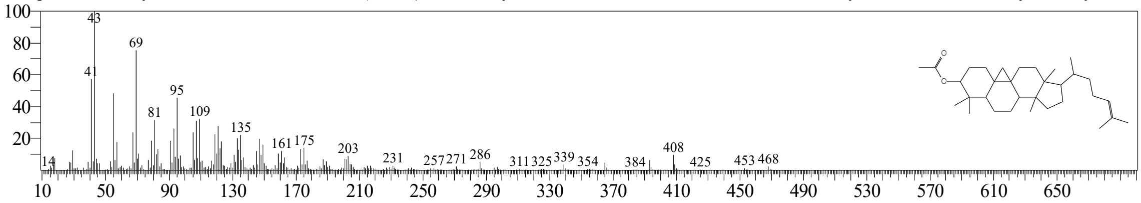

Hit#:5 Entry:191145 Library:NIST11.lib
SI:77 Formula:C30H50O CAS:0-00-0 MolWeight:426 RetIndex:2857
CompName:4.alpha.,14-Dimethyl-5.alpha.-ergosta-8,24(28)-dien-3.beta.-ol $$ 4,14-Dimethylergosta-5,24-dien-3-ol # $$

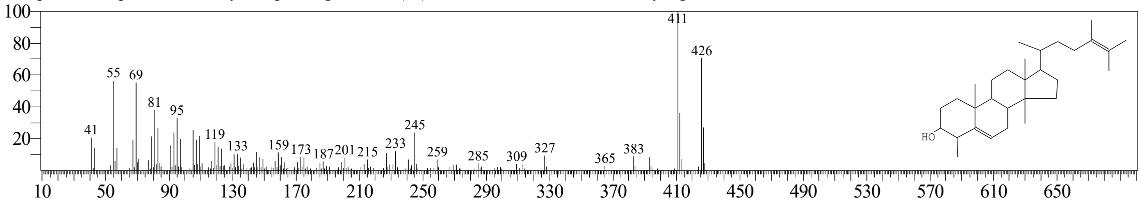

Qualitative Analysis Report

<< Target >>
Line#:104 R.Time:25.485(Scan#:4298) MassPeaks:393
RawMode:Averaged 25.480-25.490(4297-4299) BasePeak:111.05(9172)
BG Mode:Calc. from Peak Group 1 - Event 1 Scan

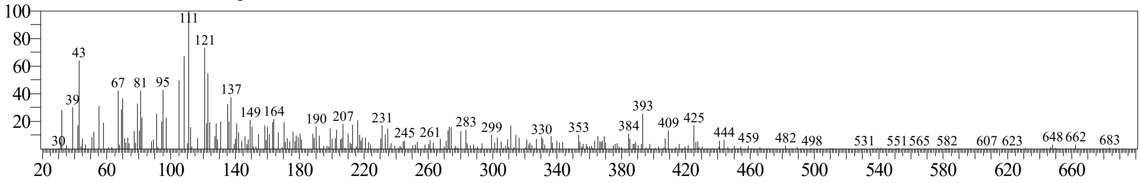

Hit#:1 Entry:48100 Library:NIST11.lib
SI:58 Formula:C15H26 CAS:74022-04-1 MolWeight:206 RetIndex:1405
CompName:2,4a,8,8-Tetramethyldecahydrocyclopropa[d]naphthalene

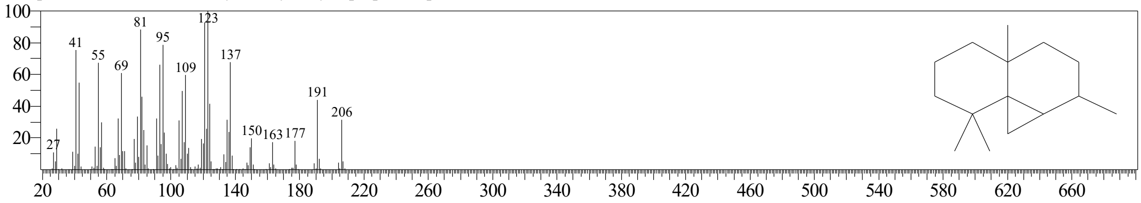

Hit#:2 Entry:109384 Library:NIST11.lib
SI:56 Formula:C21H36 CAS:0-00-0 MolWeight:288 RetIndex:2262
CompName:Trispiro[4.2.4.2.4.2.]heneicosane $$ Trispiro[4.2.4.2.4.2]henicosane # $$

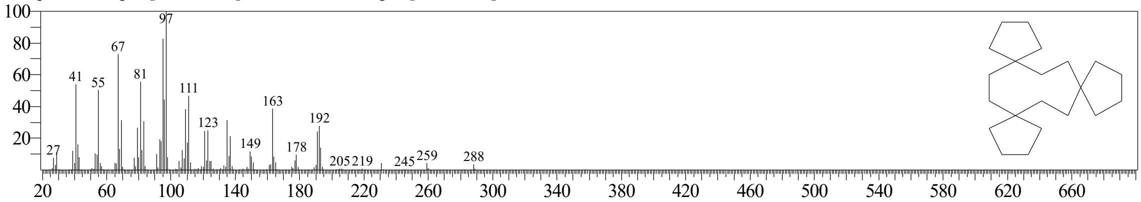

Hit#:3 Entry:191689 Library:NIST11.lib
SI:56 Formula:C30H52O CAS:24739-08-0 MolWeight:428 RetIndex:2921
CompName:C(14a)-Homo-27-nor-14.beta.-gammaceran-3.alpha.-ol

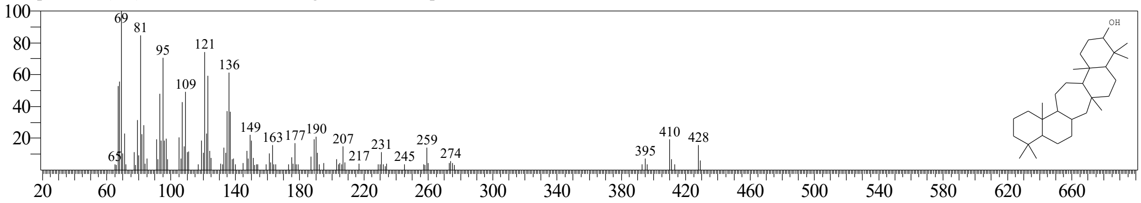

Hit#:4 Entry:195682 Library:NIST11.lib
SI:56 Formula:C30H52O2 CAS:0-00-0 MolWeight:444 RetIndex:3183
CompName:2,6,10,15,19,23-Hexamethyl-tetracosa-2,10,14,18,22-pentaene-6,7-diol $$ (10E,14E,18Z)-2,6,10,15,19,23-Hexamethyl-2,10,14,18,22-tetracosapen

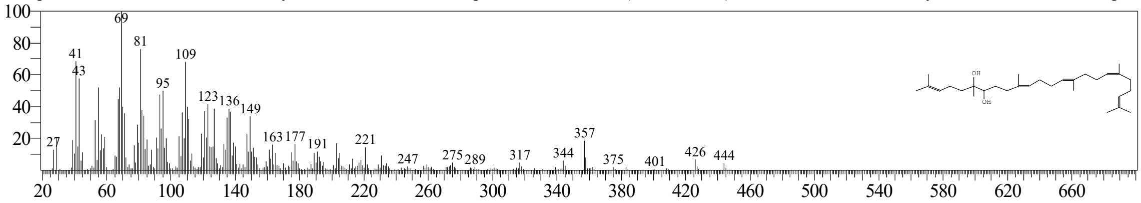

Hit#:5 Entry:78379 Library:NIST11.lib
SI:56 Formula:C18H32 CAS:54411-95-9 MolWeight:248 RetIndex:1692
CompName:1H-Indene, 1-(1,5-dimethyl-2-hexenyl)octahydro-7a-methyl-, [1R-[1.alpha.(1R*,2Z),3a.beta.,7a.alpha.]]- $$ 1-[(2E)-1,5-Dimethyl-2-hexenyl]-7a-m

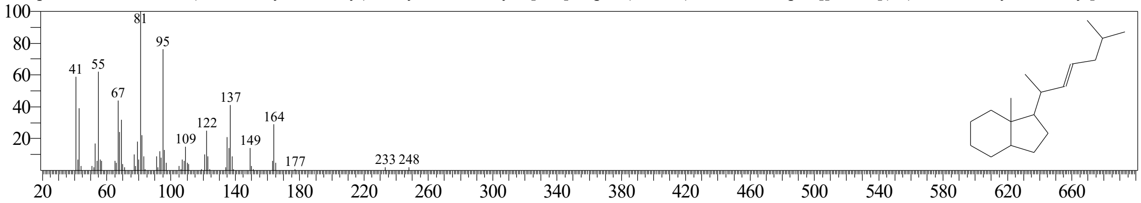

Qualitative Analysis Report

<< Target >>
Line#:105 R.Time:26.285(Scan#:4458) MassPeaks:391
RawMode:Averaged 26.280-26.290(4457-4459) BasePeak:43.10(12879)
BG Mode:Calc. from Peak Group 1 - Event 1 Scan

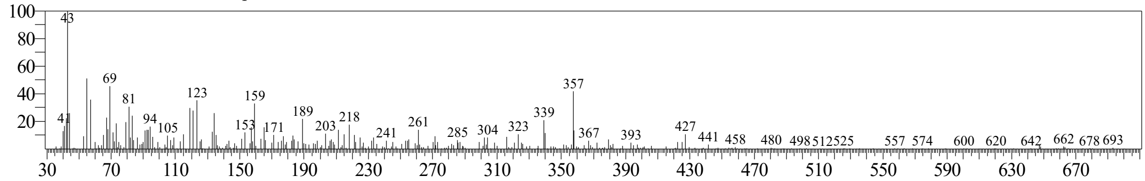

Hit#:1 Entry:187456 Library:NIST11.lib
SI:63 Formula:C27H42O3 CAS:0-00-0 MolWeight:414 RetIndex:2956
CompName:Pseduosarsasapogenin-5,20-dien $$ Furosta-5,20(22)-dien-3,26-diol # $$

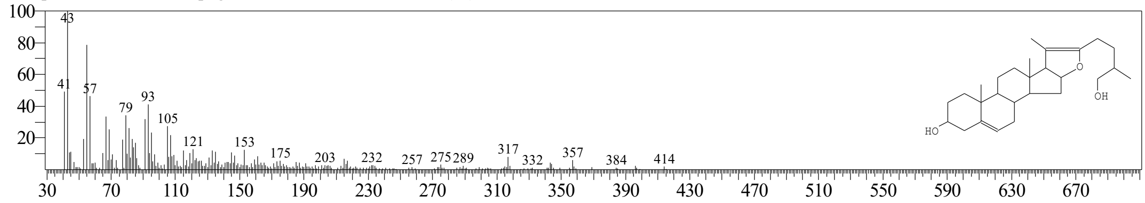

Hit#:2 Entry:90425 Library:NIST11.lib
SI:60 Formula:C17H28O2 CAS:2306-78-7 MolWeight:264 RetIndex:1754
CompName:Nerolidyl acetate $$ 1,6,10-Dodecatrien-3-ol, 3,7,11-trimethyl-, acetate $$ Nerolidol, acetate $$ 3,7,11-Trimethyl-1,6,10-dodecatrien-3-yl acetate $$

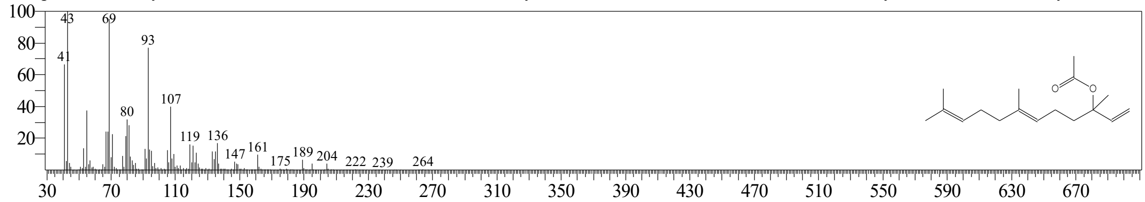

Hit#:3 Entry:192203 Library:NIST11.lib
SI:60 Formula:C28H46O3 CAS:0-00-0 MolWeight:430 RetIndex:2996
CompName:Pregnan-16-one, 20-[[3-hydroxy-6-methyl]oxacyclohex-3-yl]methyl-

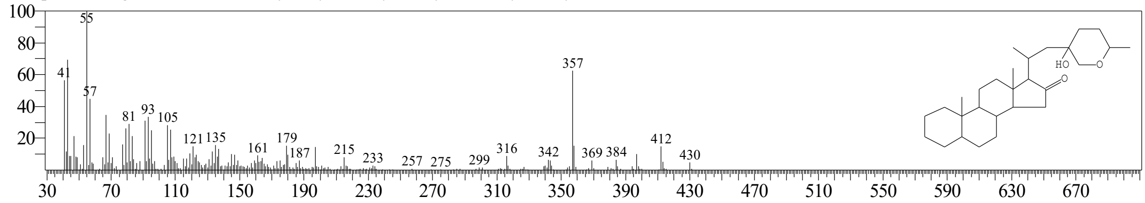

Hit#:4 Entry:191096 Library:NIST11.lib
SI:60 Formula:C30H50O CAS:55515-18-9 MolWeight:426 RetIndex:2788
CompName:Lanost-7-en-3-one, (9.beta.,13.alpha.,14.beta.,17.alpha.)- $$ Lanost-7-en-3-one # $$

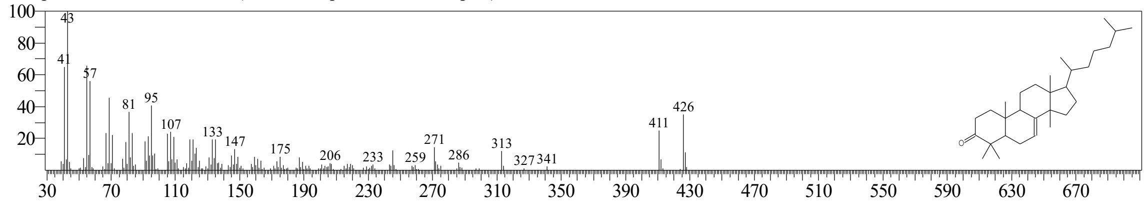

Hit#:5 Entry:153209 Library:NIST11.lib
SI:60 Formula:C22H34O3 CAS:55902-84-6 MolWeight:346 RetIndex:2338
CompName:Kauran-18-al, 17-(acetyloxy)-, (4.beta.)- $$ 18-Oxokauran-17-yl acetate # $$

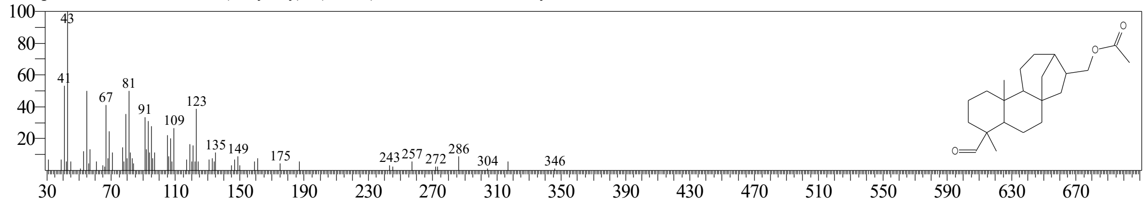

Qualitative Analysis Report

<< Target >>
Line#:106 R.Time:26.455(Scan#:4492) MassPeaks:388
RawMode:Averaged 26.450-26.460(4491-4493) BasePeak:313.25(17177)
BG Mode:Calc. from Peak Group 1 - Event 1 Scan

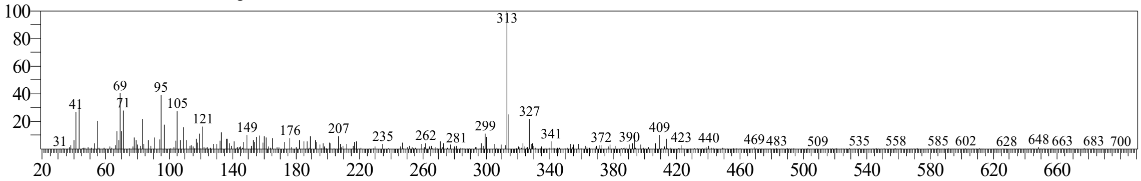

Hit#:1 Entry:182652 Library:NIST11.lib
SI:63 Formula:C27H44O2 CAS:55924-02-2 MolWeight:400 RetIndex:2888
CompName:Cholesta-17(20),24-diene-3,6-diol, (3.beta.,5.alpha.,6.alpha.)- $$ (17E)-Cholesta-17,24-diene-3,6-diol # $$

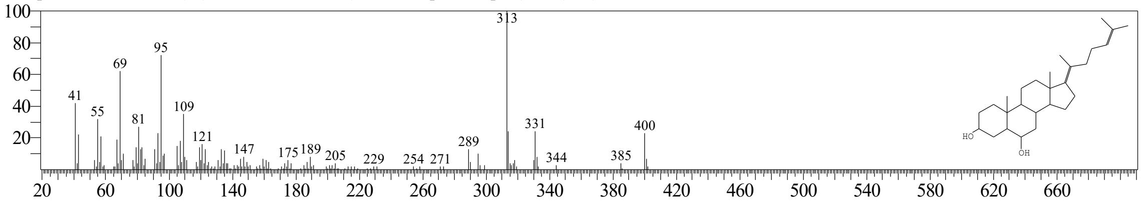

Hit#:2 Entry:197852 Library:NIST11.lib
SI:56 Formula:C31H50O2 CAS:17808-71-8 MolWeight:454 RetIndex:2881
CompName:5.alpha.-Stigmasta-7,16-dien-3.beta.-ol, acetate $$ Stigmasta-7,16-dien-3-yl acetate # $$

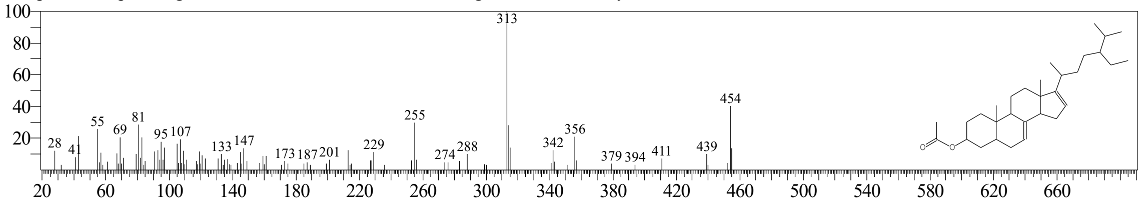

Hit#:3 Entry:197363 Library:NIST11.lib
SI:55 Formula:C31H48O2 CAS:17808-70-7 MolWeight:452 RetIndex:2913
CompName:5.alpha.-Stigmasta-7,16,25-trien-3.beta.-ol, acetate $$ Stigmasta-7,16,25(27)-trien-3-yl acetate # $$

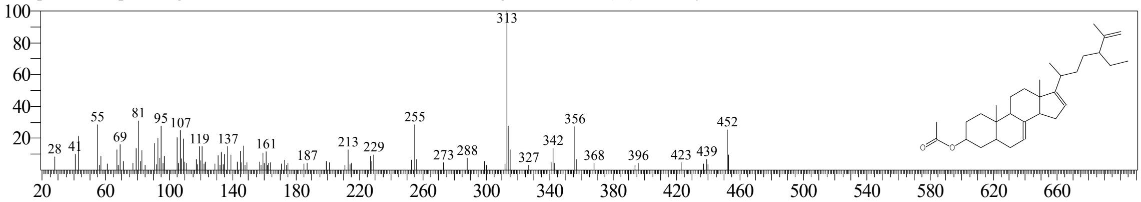

Hit#:4 Entry:191135 Library:NIST11.lib
SI:55 Formula:C30H50O CAS:0-00-0 MolWeight:426 RetIndex:2750
CompName:Cyclopenta[a]cyclopropa[e]phenanthren-7-one, perhydro-1-(1,5-dimethylhexyl)-3a,6,6,12a-tetramethyl $$ 1-(1,5-Dimethylhexyl)-3a,6,6,12a-tetram

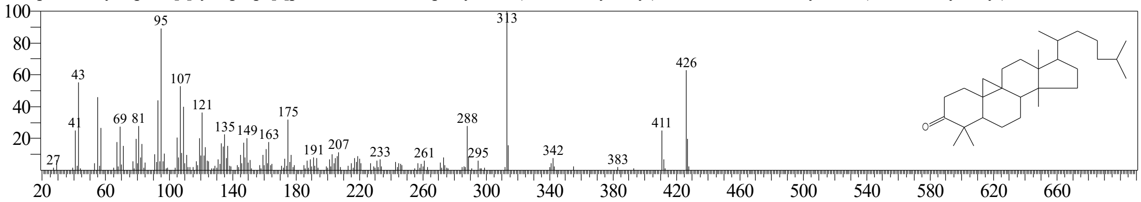

Hit#:5 Entry:194753 Library:NIST11.lib
SI:54 Formula:C30H48O2 CAS:1449-60-1 MolWeight:440 RetIndex:2779
CompName:Ergosta-7,22-dien-3-ol, acetate, (3.beta.,5.alpha.)- $$ Ergosta-7,22-dien-3.beta.-ol, acetate $$ 5.alpha.-Ergost-7,22-dien-3.beta.-ol acetate $$ 7,22-E

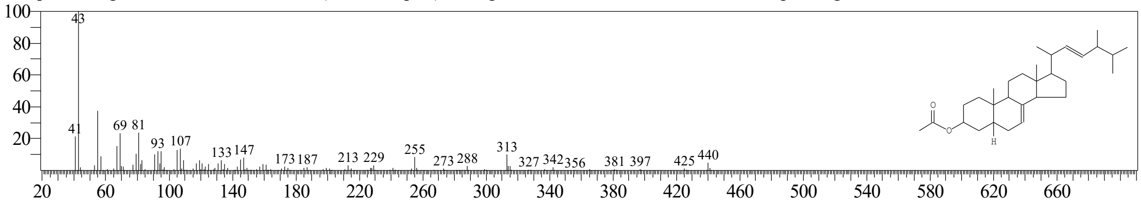

<< Target >>
Line#:107 R.Time:27.160(Scan#:4633) MassPeaks:376
RawMode:Averaged 27.155-27.165(4632-4634) BasePeak:57.15(19160)
BG Mode:Calc. from Peak Group 1 - Event 1 Scan

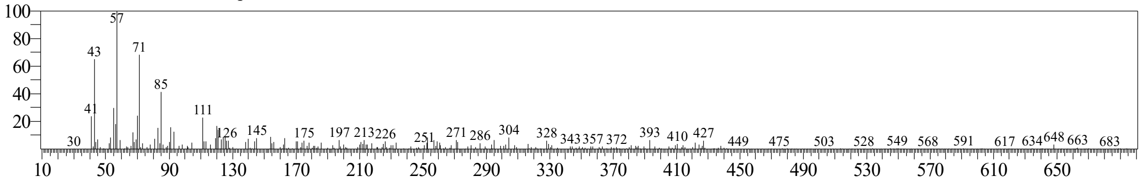

Hit#:1 Entry:109236 Library:NIST11.lib
SI:73 Formula:C18H37Cl CAS:3386-33-2 MolWeight:288 RetIndex:2036
CompName:Octadecane, 1-chloro- $$ n-Octadecyl chloride $$ Octadecyl chloride $$ 1-Chlorooctadecane $$ Chlorooctadecane $$

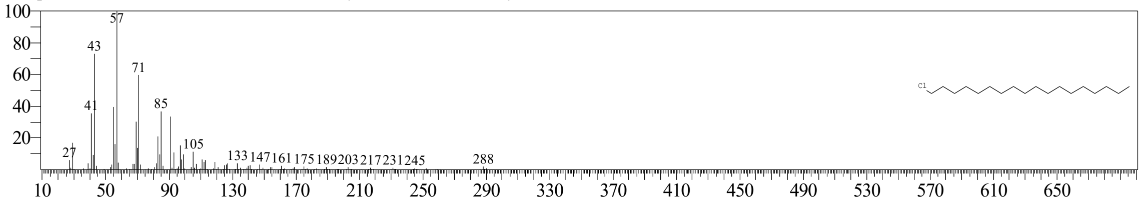

Hit#:2 Entry:87431 Library:NIST11.lib
SI:72 Formula:C16H33Cl CAS:4860-03-1 MolWeight:260 RetIndex:1837
CompName:Hexadecane, 1-chloro- $$ Cetyl chloride $$ Hexadecyl chloride $$ 1-Chlorohexadecane $$ n-Hexadecyl chloride $$ Palmityl chloride $$

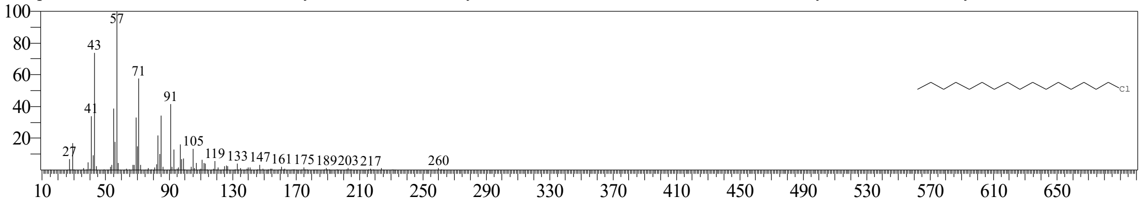

Hit#:3 Entry:120301 Library:NIST11.lib
SI:72 Formula:C19H39Cl CAS:62016-76-6 MolWeight:302 RetIndex:2135
CompName:Nonadecane, 1-chloro- $$ 1-Chlorononadecane # $$

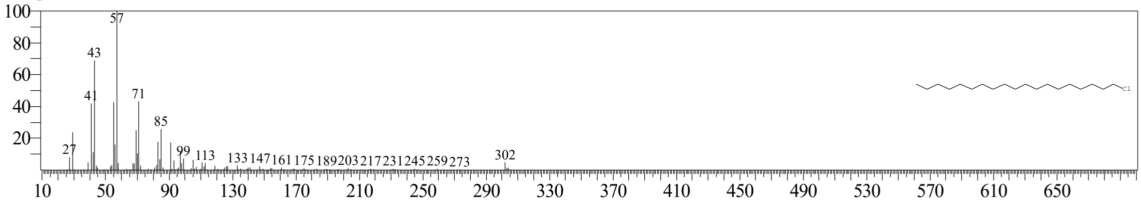

Hit#:4 Entry:115545 Library:NIST11.lib
SI:72 Formula:C21H44 CAS:54833-48-6 MolWeight:296 RetIndex:1852
CompName:Heptadecane, 2,6,10,15-tetramethyl- $$ 2,6,10,15-Tetramethylheptadecane # $$

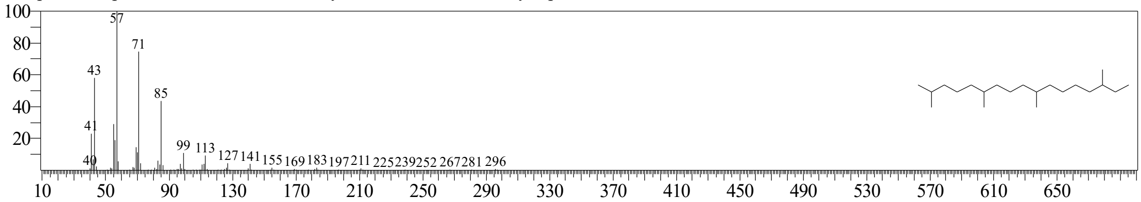

Hit#:5 Entry:187487 Library:NIST11.lib
SI:71 Formula:C27H55Cl CAS:62016-79-9 MolWeight:414 RetIndex:2930
CompName:Heptacosane, 1-chloro- $$ 1-Chloroheptacosane # $$

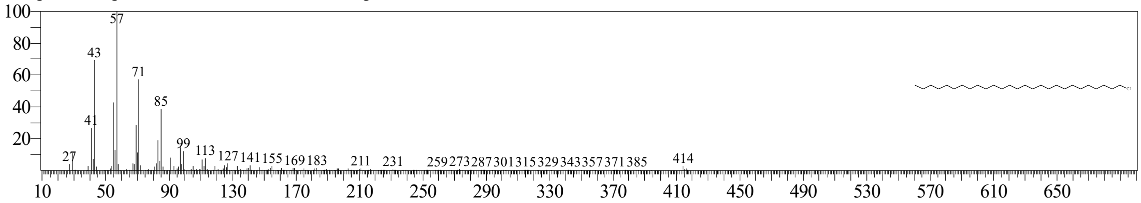

Qualitative Analysis Report

<< Target >>
Line#:108 R.Time:27.820(Scan#:4765) MassPeaks:351
RawMode:Averaged 27.815-27.825(4764-4766) BasePeak:411.30(10530)
BG Mode:Calc. from Peak Group 1 - Event 1 Scan

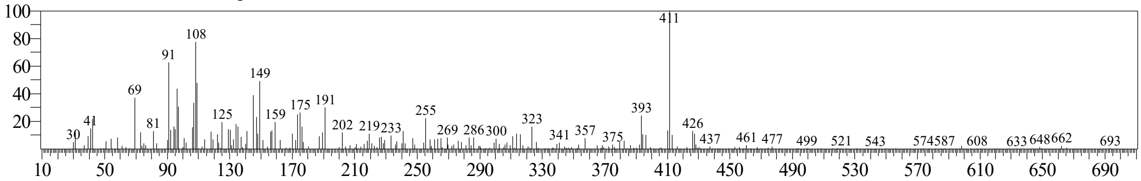

Hit#:1 Entry:191141 Library:NIST11.lib
SI:48 Formula:C30H50O CAS:16910-32-0 MolWeight:426 RetIndex:2826
CompName:Obtusifoliol $$ Ergosta-8,24(28)-dien-3-ol, 4,14-dimethyl-, (3.beta.,4.alpha.,5.alpha.)- $$ 5.alpha.-Ergosta-8,24(28)-dien-3.beta.-ol, 4.alpha.,14-din

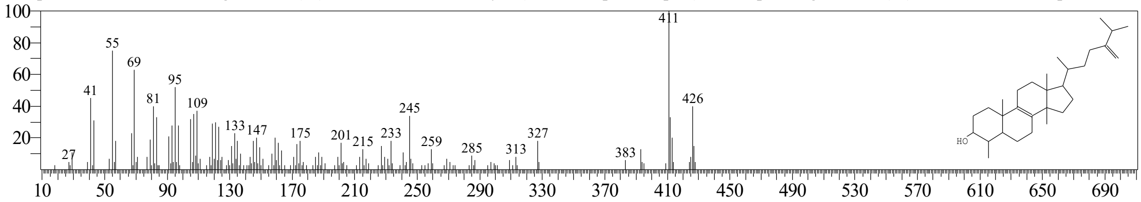

Hit#:2 Entry:191142 Library:NIST11.lib
SI:48 Formula:C30H50O CAS:0-00-0 MolWeight:426 RetIndex:2882
CompName:Tirucallol

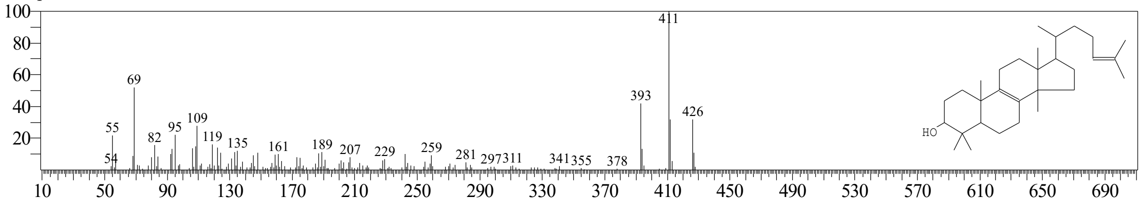

Hit#:3 Entry:195684 Library:NIST11.lib
SI:48 Formula:C30H52O2 CAS:26525-84-8 MolWeight:444 RetIndex:2923
CompName:9,19-Cyclo-9.beta.-lanostane-3.beta.,25-diol $$ Cycloartane-3.beta.,25-diol $$ 1-(5-Hydroxy-1,5-dimethylhexyl)-3a,6,6,12a-tetramethyltetradecahy

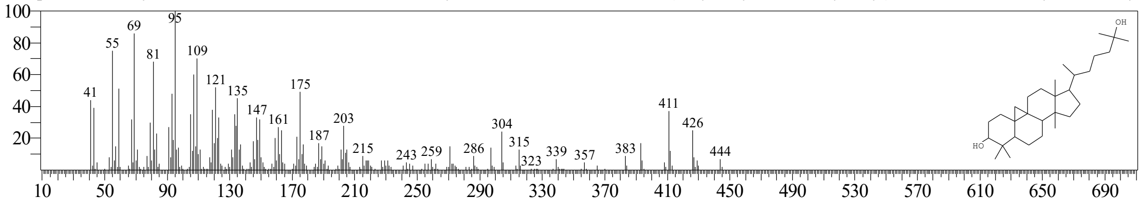

Hit#:4 Entry:123076 Library:NIST11.lib
SI:47 Formula:C17H22O5 CAS:69745-81-9 MolWeight:306 RetIndex:2412
CompName:Cyclodeca[b]furan-2(3H)-one, 9-(acetyloxy)-3a,4,5,8,9,11a-hexahydro-4-hydroxy-6,10-dimethyl-3-methylene- $$ 4-Hydroxy-6,10-dimethyl-3-meth

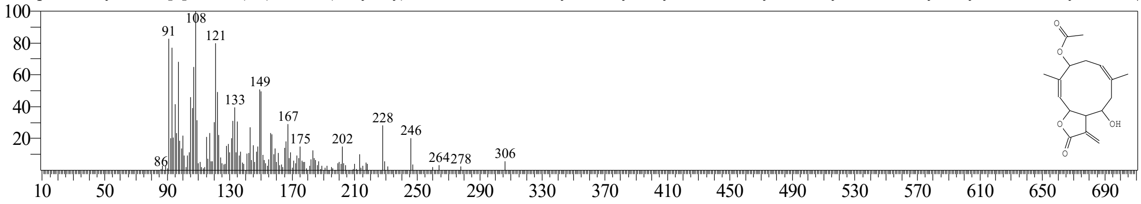

Hit#:5 Entry:191106 Library:NIST11.lib
SI:47 Formula:C30H50O CAS:469-38-5 MolWeight:426 RetIndex:2816
CompName:9,19-Cyclolanost-24-en-3-ol, (3.beta.)- $$ 9,19-Cyclo-9.beta.-lanost-24-en-3.beta.-ol $$ Cycloartenol $$ Handianol $$ 1-(1,5-Dimethyl-4-hexenyl)-

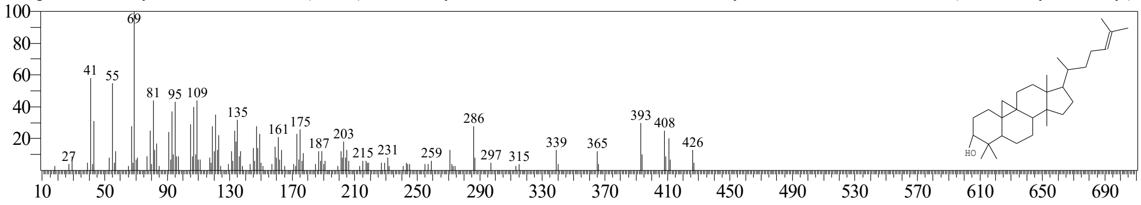

<< Target >>
Line#:109 R.Time:27.865(Scan#:4774) MassPeaks:374
RawMode:Averaged 27.860-27.870(4773-4775) BasePeak:69.10(16054)
BG Mode:Calc. from Peak Group 1 - Event 1 Scan

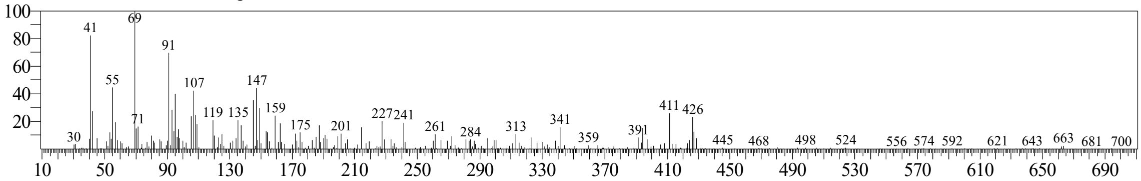

Hit#:1 Entry:196536 Library:NIST11.lib
SI:61 Formula:C28H48O4 CAS:56052-97-2 MolWeight:448 RetIndex:3179
CompName:Ergost-25-ene-3,5,6,12-tetrol, (3.beta.,5.alpha.,6.beta.,12.beta.)- $$ Ergost-25(27)-ene-3,5,6,12-tetrol # $$

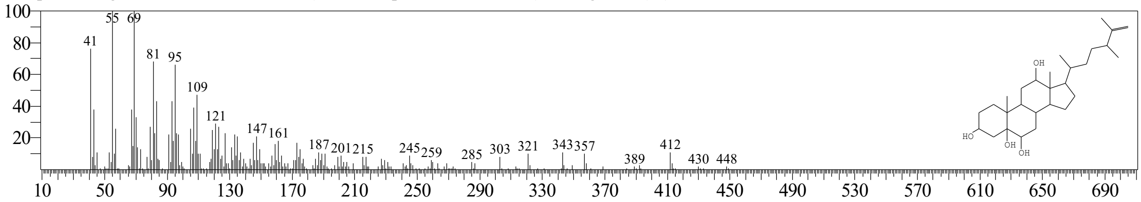

Hit#:2 Entry:191107 Library:NIST11.lib
SI:61 Formula:C30H50O CAS:79-63-0 MolWeight:426 RetIndex:2882
CompName:Lanosterol $$ Lanosta-8,24-dien-3-ol, (3.beta.)- $$ Lanosta-8,24-dien-3.beta.-ol $$ Botalan base 138 $$ Cholesta-8,24-dien-3-ol, 4,4,14-trimethyl-,

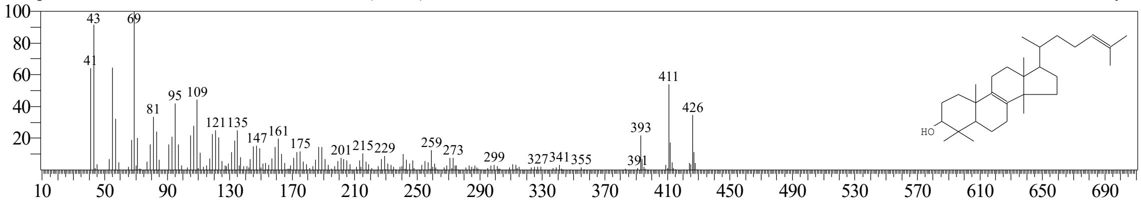

Hit#:3 Entry:191141 Library:NIST11.lib
SI:59 Formula:C30H50O CAS:16910-32-0 MolWeight:426 RetIndex:2826
CompName:Obtusifoliol $$ Ergosta-8,24(28)-dien-3-ol, 4,14-dimethyl-, (3.beta.,4.alpha.,5.alpha.)- $$ 5.alpha.-Ergosta-8,24(28)-dien-3.beta.-ol, 4.alpha.,14-din

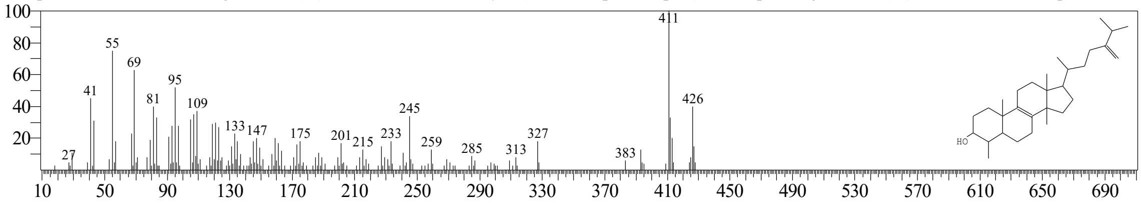

Hit#:4 Entry:191101 Library:NIST11.lib
SI:59 Formula:C30H50O CAS:469-39-6 MolWeight:426 RetIndex:2760
CompName:9,19-Cycloergost-24(28)-en-3-ol, 4,14-dimethyl-, (3.beta.,4.alpha.,5.alpha.)- $$ 9,19-Cyclo-5.alpha.,9.beta.-ergost-24(28)-en-3.beta.-ol, 4.alpha.,14

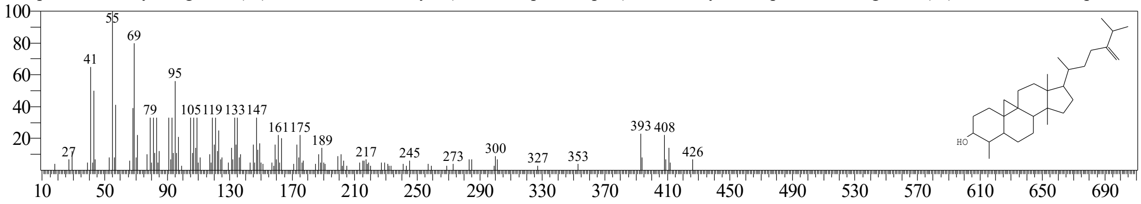

Hit#:5 Entry:96989 Library:NIST11.lib
SI:58 Formula:C20H32 CAS:77898-97-6 MolWeight:272 RetIndex:1940
CompName:(E,E,E)-3,7,11,15-Tetramethylhexadeca-1,3,6,10,14-pentaene $$ (3E,6E,10E)-3,7,11,15-Tetramethyl-1,3,6,10,14-hexadecapentaene $$ (E,E,E)- .alp

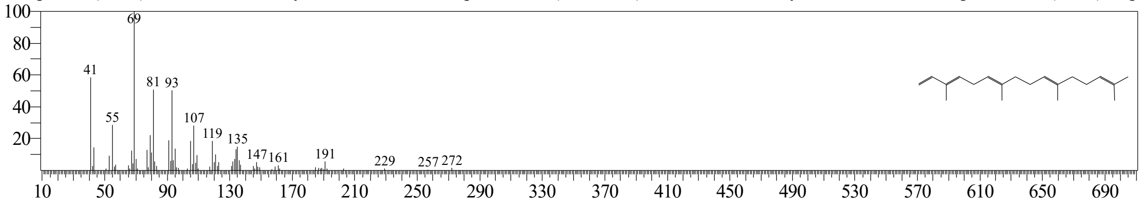

<< Target >>
Line#:110 R.Time:27.915(Scan#:4784) MassPeaks:356
RawMode:Averaged 27.910-27.920(4783-4785) BasePeak:43.05(10851)
BG Mode:Calc. from Peak Group 1 - Event 1 Scan

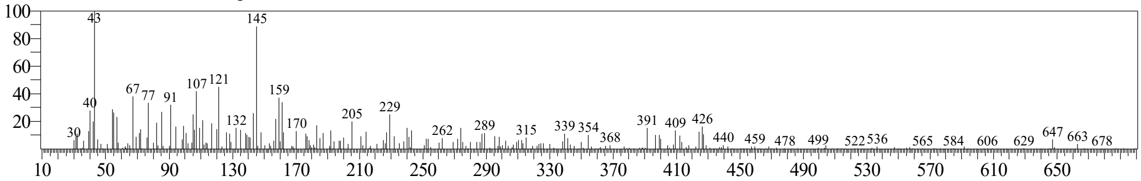

Hit#:1 Entry:131298 Library:NIST11.lib
SI:54 Formula:C21H32O2 CAS:145-13-1 MolWeight:316 RetIndex:2264
CompName:Pregnenolone $$ Pregn-5-en-20-one, 3-hydroxy-, (3.beta.)- $$ Pregn-5-en-20-one, 3.beta.-hydroxy- $$ Arthenolone $$ Bina-Skin $$ Enelone $$ Na

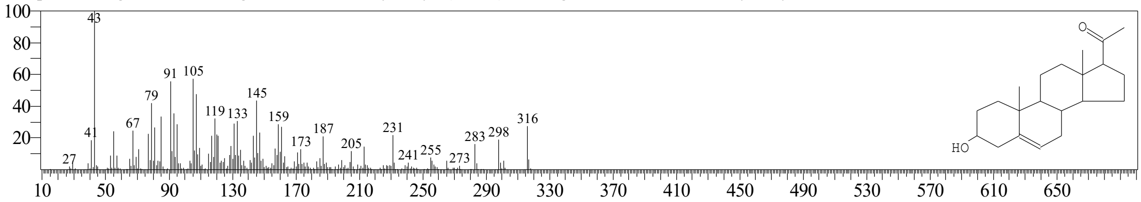

Hit#:2 Entry:176730 Library:NIST11.lib
SI:50 Formula:C23H33NO4 CAS:0-00-0 MolWeight:387 RetIndex:2758
CompName:1-Phenanthrenepropanenitrile, 7,.alpha.-diacetoxy-2-methylene-4b-methylperhydro- $$ 2-[7-(Acetyloxy)-4b-methyl-2-methylenetetradecahydro-1-p

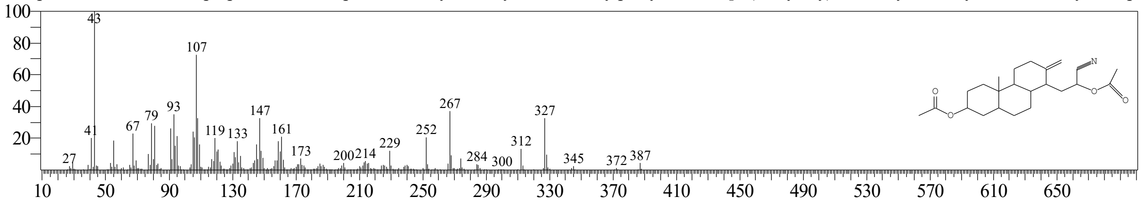

Hit#:3 Entry:131299 Library:NIST11.lib
SI:50 Formula:C21H32O2 CAS:38372-24-6 MolWeight:316 RetIndex:2264
CompName:Pregn-5-en-20-one, 3-hydroxy- $$ 5-Pregnene-3-ol-20-one $$ 3-Hydroxypregn-5-en-20-one # $$

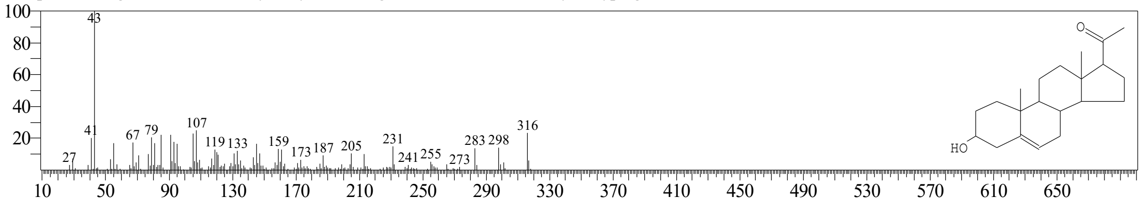

Hit#:4 Entry:57734 Library:NIST11.lib
SI:49 Formula:C15H24O CAS:77171-55-2 MolWeight:220 RetIndex:1536
CompName:(-)-Spathulenol $$ 1H-Cycloprop[e]azulen-7-ol, decahydro-1,1,7-trimethyl-4-methylene-, [1aS-(1a.alpha.,4a.alpha.,7.beta.)] $$ Ent-Spathulenol $$

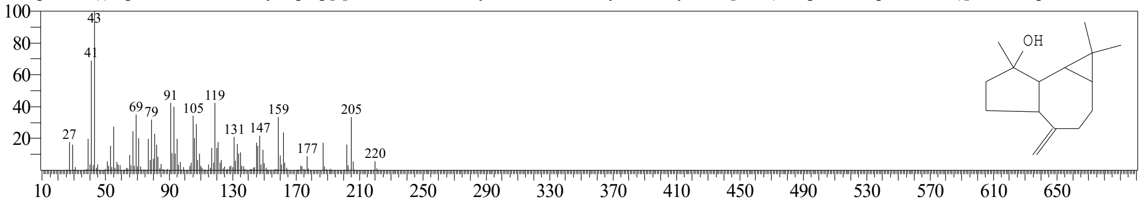

Hit#:5 Entry:141843 Library:NIST11.lib
SI:49 Formula:C22H34O2 CAS:0-00-0 MolWeight:330 RetIndex:2213
CompName:5-Pregnen-3.beta.-ol-20-one, methyl ether

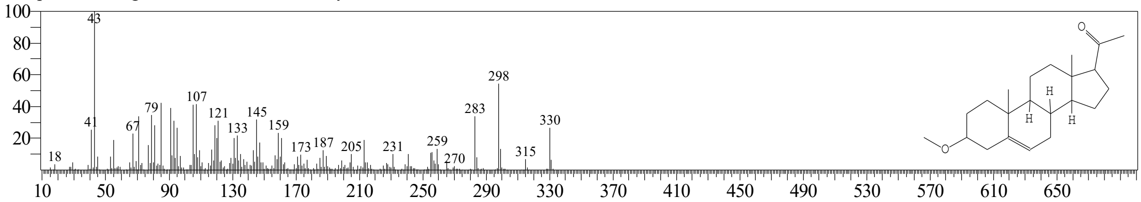

<< Target >>
Line#:111 R.Time:27.975(Scan#:4796) MassPeaks:361
RawMode:Averaged 27.970-27.980(4795-4797) BasePeak:41.05(10950)
BG Mode:Calc. from Peak Group 1 - Event 1 Scan

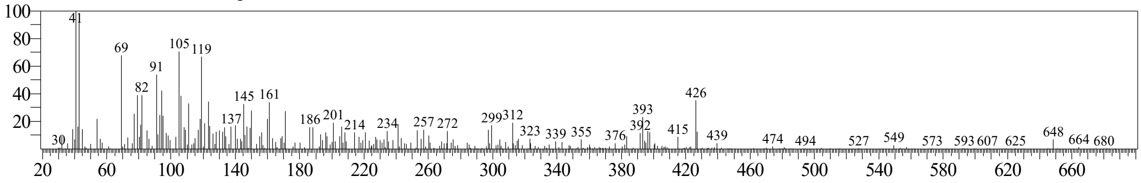

Hit#:1 Entry:59354 Library:NIST11.lib
SI:62 Formula:C15H26O CAS:0-00-0 MolWeight:222 RetIndex:1523
CompName:cis-sesquisabinene hydrate

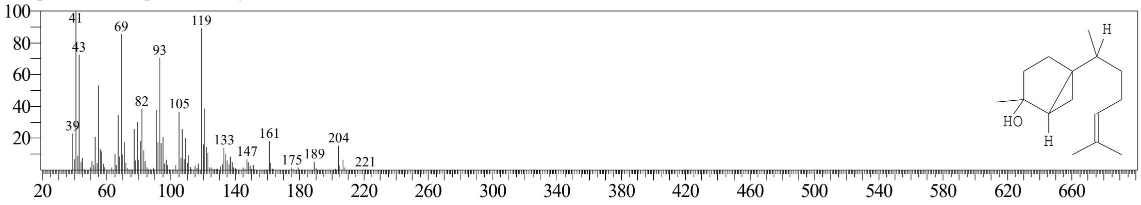

Hit#:2 Entry:59425 Library:NIST11.lib
SI:61 Formula:C15H26O CAS:0-00-0 MolWeight:222 RetIndex:1523
CompName:trans-Sesquisabinene hydrate

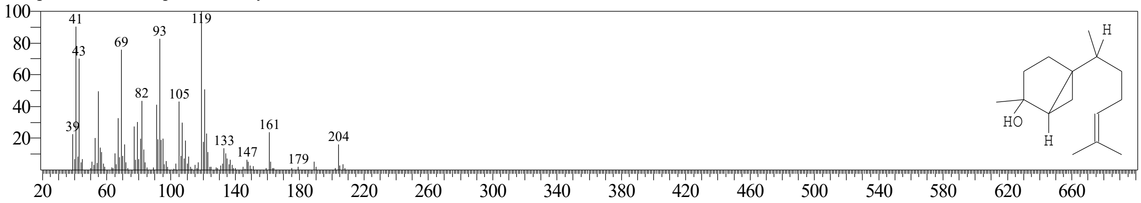

Hit#:3 Entry:59395 Library:NIST11.lib
SI:60 Formula:C15H26O CAS:0-00-0 MolWeight:222 RetIndex:1523
CompName:7-epi-cis-sesquisabinene hydrate

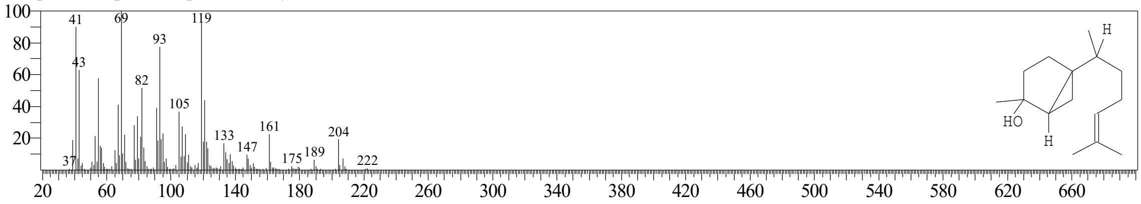

Hit#:4 Entry:112110 Library:NIST11.lib
SI:59 Formula:C17H24O4 CAS:0-00-0 MolWeight:292 RetIndex:2244
CompName:Acetic acid, 6-(1-hydroxymethyl-vinyl)-4,8a-dimethyl-3-oxo-1,2,3,5,6,7,8,8a-octahydronaphthalen-2-yl ester $$ 6-[1-(Hydroxymethyl)vinyl]-4,8a-d

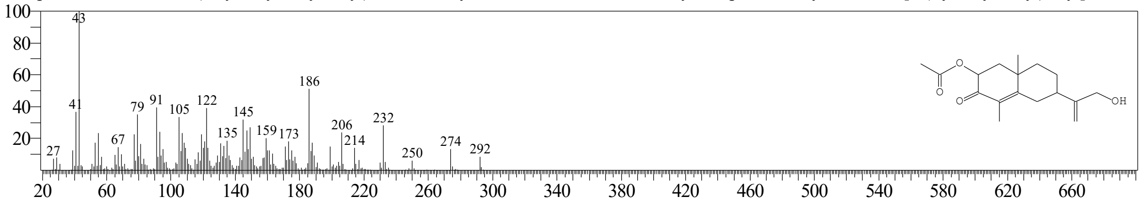

Hit#:5 Entry:59426 Library:NIST11.lib
SI:59 Formula:C15H26O CAS:0-00-0 MolWeight:222 RetIndex:1523
CompName:7-epi-trans-sesquisabinene hydrate

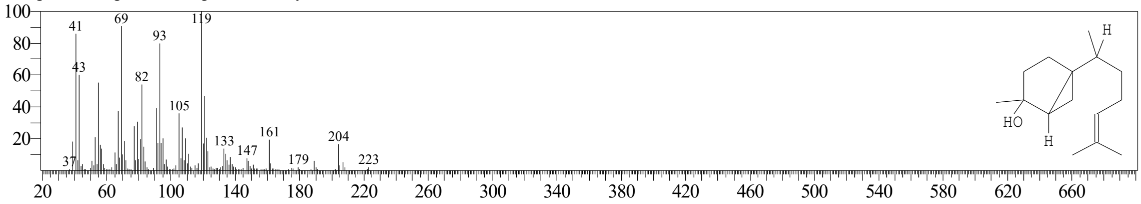

Qualitative Analysis Report

<< Target >>
Line#:112 R.Time:28.015(Scan#:4804) MassPeaks:399
RawMode:Averaged 28.010-28.020(4803-4805) BasePeak:41.10(16796)
BG Mode:Calc. from Peak Group 1 - Event 1 Scan

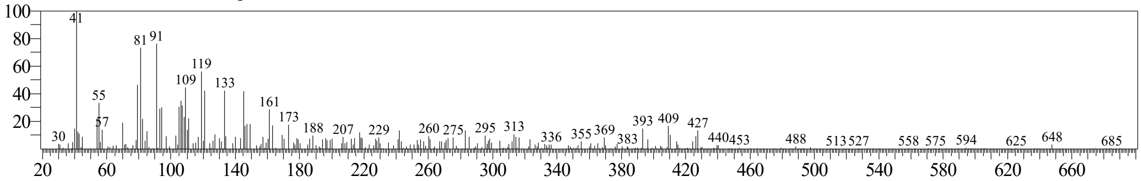

Hit#:1 Entry:47991 Library:NIST11.lib
SI:65 Formula:C14H22O CAS:0-00-0 MolWeight:206 RetIndex:1395
CompName:3-Isopropyltricyclo[4.3.1.1(2,5)]undec-3-en-10-ol

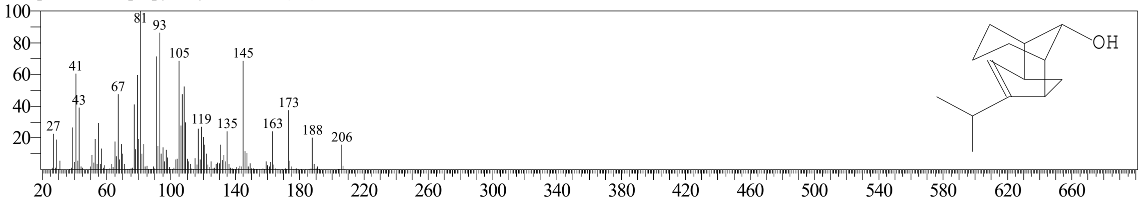

Hit#:2 Entry:170434 Library:NIST11.lib
SI:63 Formula:C25H42O2 CAS:66990-32-7 MolWeight:374 RetIndex:2897
CompName:10-12-Pentacosadiynoic acid $$ 10,12-Pentacosadiynoic acid $$

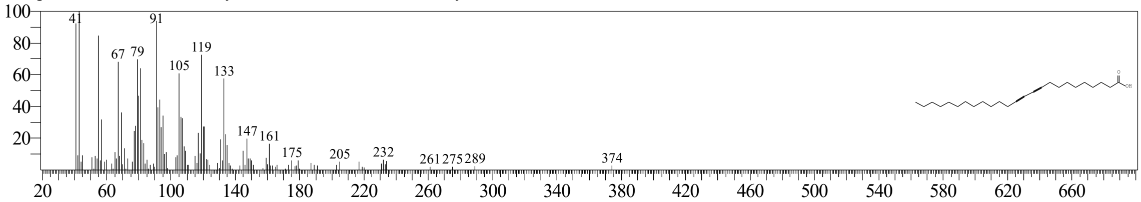

Hit#:3 Entry:146224 Library:NIST11.lib
SI:63 Formula:C22H40O2 CAS:56599-50-9 MolWeight:336 RetIndex:2453
CompName:2H-Pyran, 2-(7-heptadecynyloxy)tetrahydro- $$ 2-(7-Heptadecynyloxy)tetrahydro-2H-pyran # $$

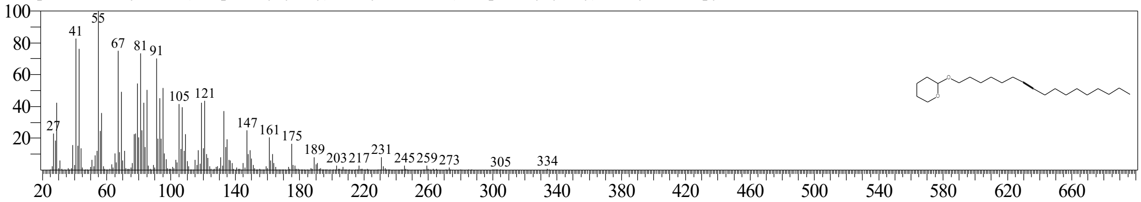

Hit#:4 Entry:46518 Library:NIST11.lib
SI:63 Formula:C13H20N2 CAS:0-00-0 MolWeight:204 RetIndex:0
CompName:1,4-Methanocycloocta[d]pyridazine, 1,4,4a,5,6,9,10,10a-octahydro-11,11-dimethyl-, (1.alpha.,4.alpha.,4a.alpha.,10a.alpha.)-

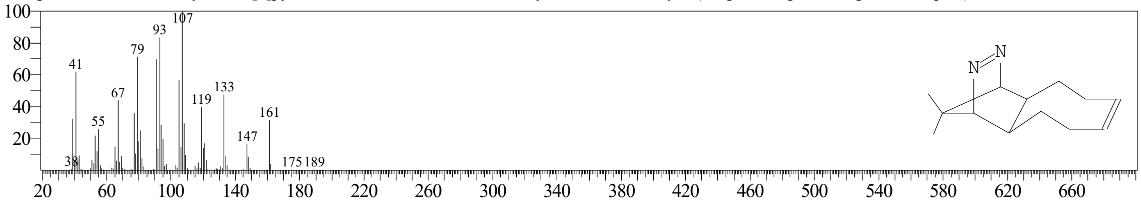

Hit#:5 Entry:47992 Library:NIST11.lib
SI:63 Formula:C14H22O CAS:0-00-0 MolWeight:206 RetIndex:1379
CompName:2-Isopropyl-tricyclo[4.3.1.1(2,5)]undec-3-en-10-ol

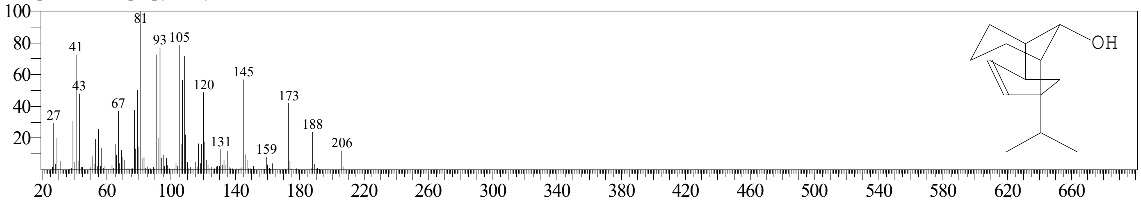

<< Target >>
Line#:113 R.Time:30.190(Scan#:5239) MassPeaks:335
RawMode:Averaged 30.185-30.195(5238-5240) BasePeak:107.05(5892)
BG Mode:Calc. from Peak Group 1 - Event 1 Scan

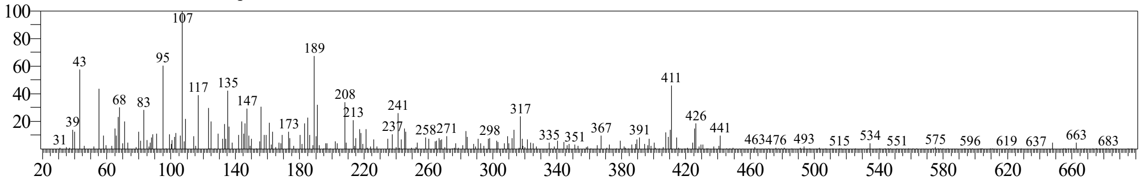

Hit#:1 Entry:191097 Library:NIST11.lib
SI:48 Formula:C30H50O CAS:545-47-1 MolWeight:426 RetIndex:2848
CompName:Lupeol $$ Lup-20(29)-en-3-ol, (3.beta.)- $$ Lup-20(29)-en-3.beta.-ol $$ .beta.-Viscol $$ Clerodol $$ Fagarasterol $$ Fagarsterol $$ Lupenol $$ Mo

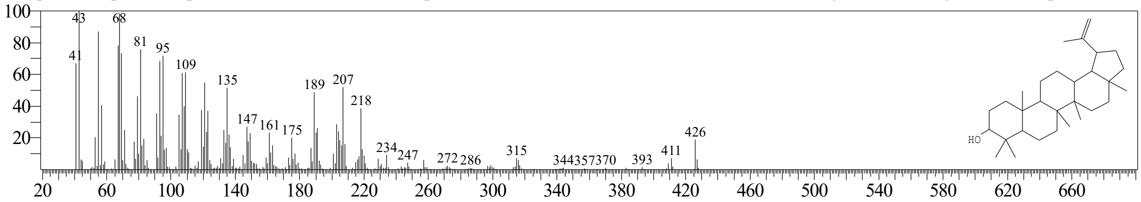

Hit#:2 Entry:116555 Library:NIST11.lib
SI:47 Formula:C15H22O6 CAS:34114-99-3 MolWeight:298 RetIndex:2401
CompName:T-2 Tetraol $$ Trichothec-9-ene-3,4,8,15-tetrol, 12,13-epoxy-, (3.alpha.,4.beta.,8.alpha.)- $$ Trichothec-9-ene-3.alpha.,4.beta.,8.alpha.,15-tetrol, 12,

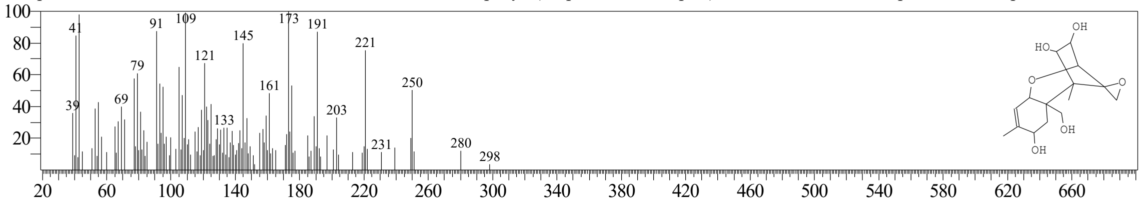

Hit#:3 Entry:207105 Library:NIST11.lib
SI:47 Formula:C34H56O4 CAS:0-00-0 MolWeight:528 RetIndex:3330
CompName:Acetic acid, 13-acetoxy-4,4,6a,6b,8a,11,12,14b-octamethyldocosahydropicen-3-yl ester $$ 12-(Acetyloxy)ursan-3-yl acetate # $$

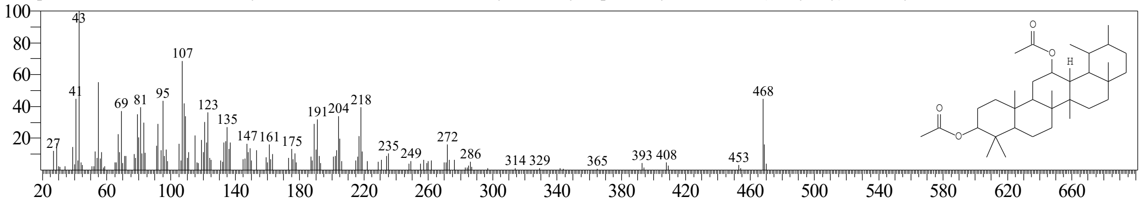

Hit#:4 Entry:123079 Library:NIST11.lib
SI:47 Formula:C17H22O5 CAS:31299-06-6 MolWeight:306 RetIndex:2327
CompName:Azuleno[4,5-b]furan-2,9-dione, 9a-[(acetyloxy)methyl]decahydro-6-methyl-3-methylene-, [3as-(3a.alpha.,6.beta.,6a.alpha.,9a.beta.,9b.alpha.)]- $$ 1

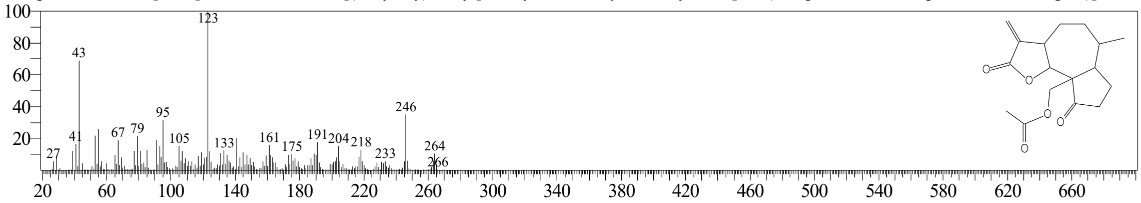

Hit#:5 Entry:191148 Library:NIST11.lib
SI:47 Formula:C30H50O CAS:0-00-0 MolWeight:426 RetIndex:2836
CompName:6a,14a-Methanopicene, perhydro-1,2,4a,6b,9,9,12a-heptamethyl-10-hydroxy-

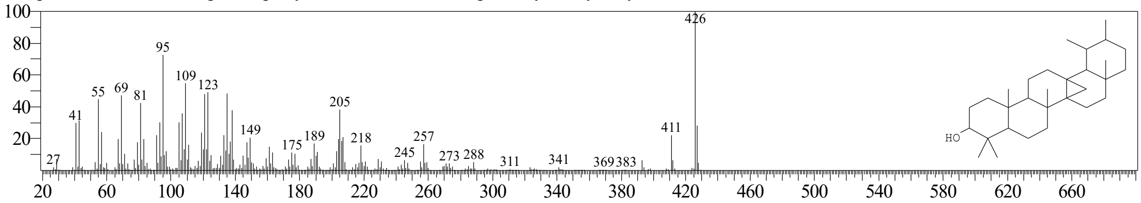

<< Target >>
Line#:114 R.Time:30.235(Scan#:5248) MassPeaks:357
RawMode:Averaged 30.230-30.240(5247-5249) BasePeak:95.05(11003)
BG Mode:Calc. from Peak Group 1 - Event 1 Scan

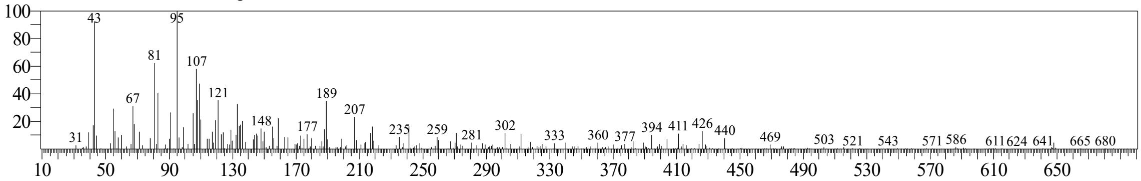

Hit#:1 Entry:110901 Library:NIST11.lib
SI:62 Formula:C20H34O CAS:25269-17-4 MolWeight:290 RetIndex:2211
CompName:Thunbergol $$ 4-Isopropyl-1,7,11-trimethyl-2,7,11-cyclotetradecatrien-1-ol # $$

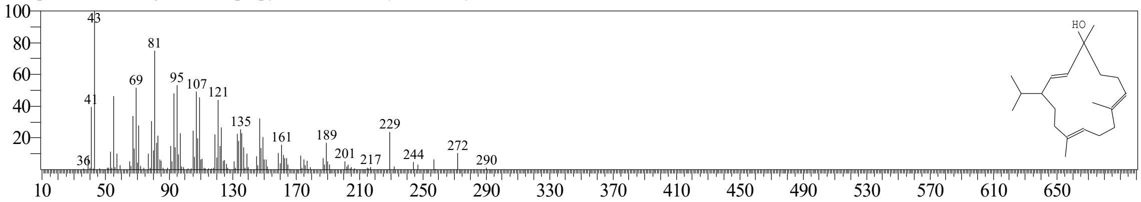

Hit#:2 Entry:200397 Library:NIST11.lib
SI:60 Formula:C32H52O2 CAS:10376-42-8 MolWeight:468 RetIndex:2900
CompName:9,19-Cycloergost-24(28)-en-3-ol, 4,14-dimethyl-, acetate, (3.beta.,4.alpha.,5.alpha.)- $$ 1-(4-Isopropyl-1-methyl-4-pentenyl)-3a,6,12a-trimethyltetra

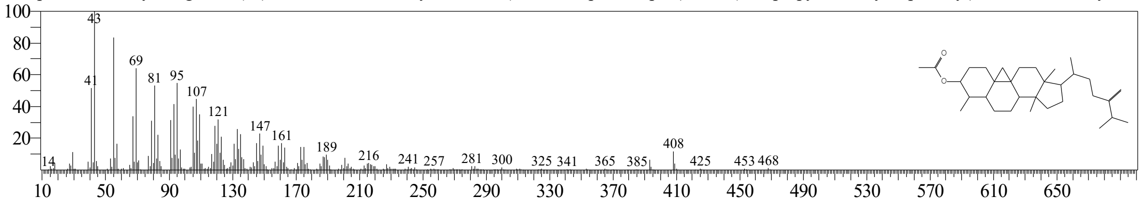

Hit#:3 Entry:191097 Library:NIST11.lib
SI:59 Formula:C30H50O CAS:545-47-1 MolWeight:426 RetIndex:2848
CompName:Lupeol $$ Lup-20(29)-en-3-ol, (3.beta.)- $$ Lup-20(29)-en-3.beta.-ol $$ .beta.-Viscol $$ Clerodol $$ Fagarasterol $$ Fagarsterol $$ Lupenol $$ Mo

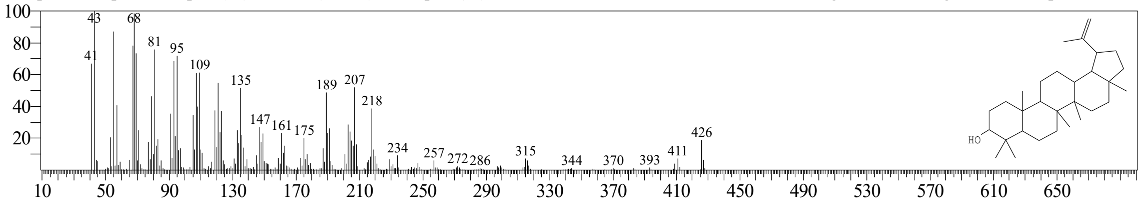

Hit#:4 Entry:89016 Library:NIST11.lib
SI:58 Formula:C18H30O CAS:0-00-0 MolWeight:262 RetIndex:1825
CompName:Sclareoloxide(Cis-A/B) $$ 3,4a,7,7,10a-Pentamethyl-4a,5,6,6a,7,8,9,10,10a,10b-decahydro-1H-benzo[f]chromene # $$

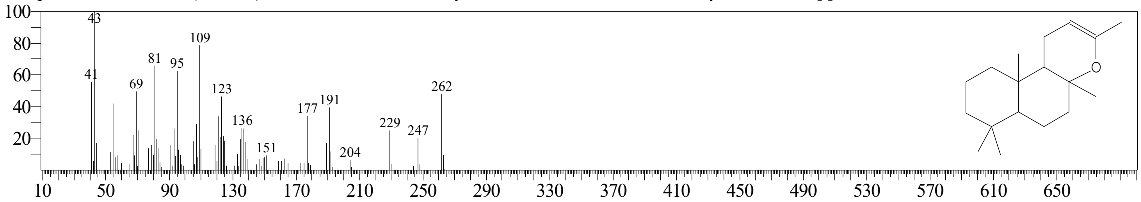

Hit#:5 Entry:59367 Library:NIST11.lib
SI:58 Formula:C15H26O CAS:489-41-8 MolWeight:222 RetIndex:1530
CompName:(-)-Globulol $$ 1H-Cycloprop[e]azulen-4-ol, decahydro-1,1,4,7-tetramethyl-, [1aR-(1a.alpha.,4.alpha.,4a.alpha.,7.alpha.,7a.beta.,7b.alpha.)]- $$ 1,1

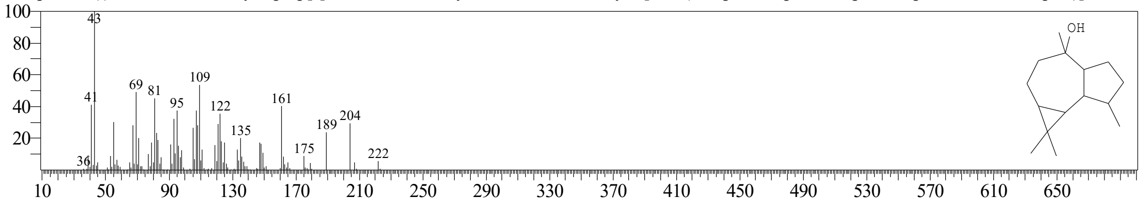

<< Target >>
Line#:115 R.Time:31.095(Scan#:5420) MassPeaks:424
RawMode:Averaged 31.090-31.100(5419-5421) BasePeak:41.10(41598)
BG Mode:Calc. from Peak Group 1 - Event 1 Scan

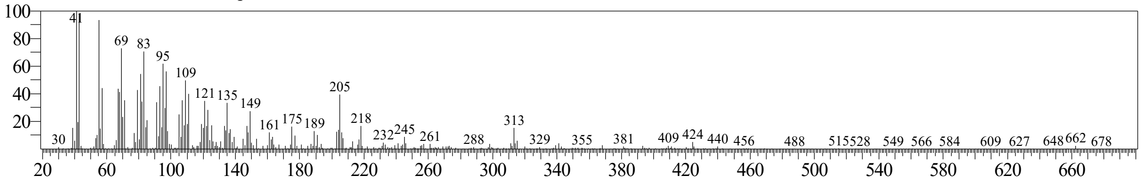

Hit#:1 Entry:207593 Library:NIST11.lib
SI:81 Formula:C37H76O CAS:105794-58-9 MolWeight:536 RetIndex:3942
CompName:1-Heptatriacotanol $$ 1-Heptatriacontanol # $$

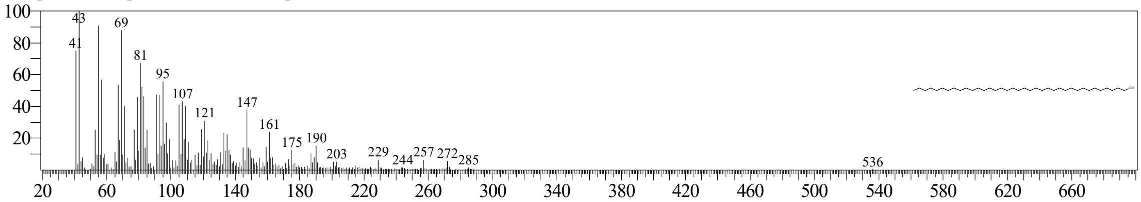

Hit#:2 Entry:113929 Library:NIST11.lib
SI:81 Formula:C19H34O2 CAS:0-00-0 MolWeight:294 RetIndex:2241
CompName:E,E,Z-1,3,12-Nonadecatriene-5,14-diol $$ (3E,12Z)-1,3,12-Nonadecatriene-5,14-diol # $$

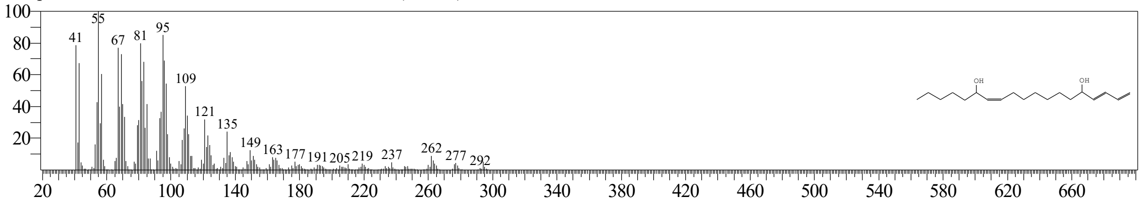

Hit#:3 Entry:91766 Library:NIST11.lib
SI:80 Formula:C16H26O3 CAS:19780-11-1 MolWeight:266 RetIndex:2159
CompName:2-Dodecen-1-yl(-)succinic anhydride $$ 2,5-Furandione, 3-dodecenyl- $$ n-Dodecenylsuccinic anhydride $$ Dodecenyl succinic anhydride $$ 2,5-l

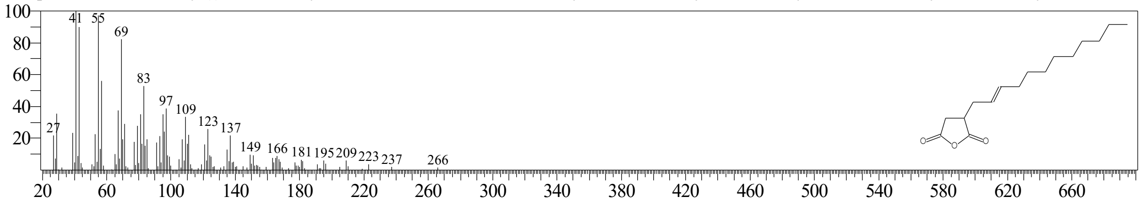

Hit#:4 Entry:195680 Library:NIST11.lib
SI:80 Formula:C30H52O2 CAS:0-00-0 MolWeight:444 RetIndex:3350
CompName:Tricyclo[20.8.0.0(7,16)]triacontane, 1(22),7(16)-diepoxy-

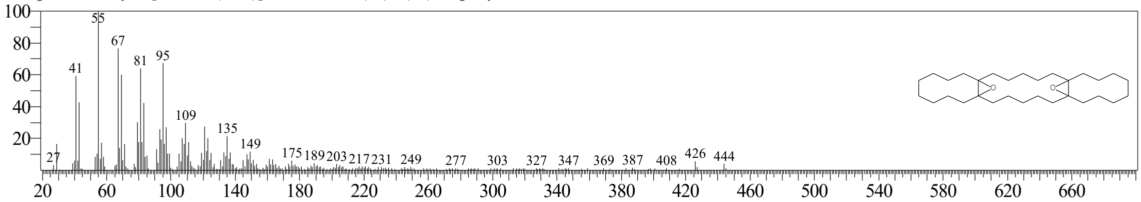

Hit#:5 Entry:187511 Library:NIST11.lib
SI:80 Formula:C29H50O CAS:2097-85-0 MolWeight:414 RetIndex:2704
CompName:Cholestan-3-one, 4,4-dimethyl-, (5.alpha.)- $$ 5.alpha.-Cholestan-3-one, 4,4-dimethyl- $$ 4,4-Dimethyl-5-.alpha.-cholestane-3-one $$ 4,4-Dimethy

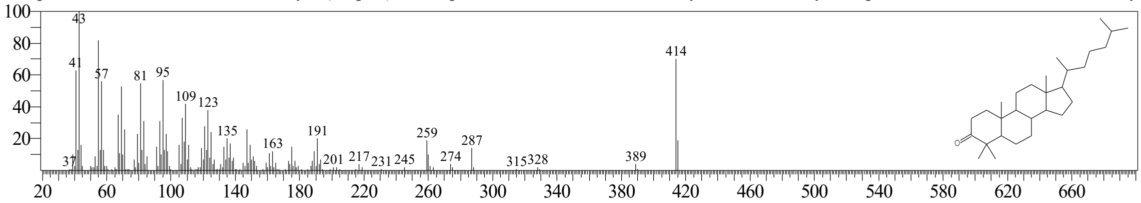

<< Target >>
Line#:116 R.Time:31.825(Scan#:5566) MassPeaks:354
RawMode:Averaged 31.820-31.830(5565-5567) BasePeak:109.10(5769)
BG Mode:Calc. from Peak Group 1 - Event 1 Scan

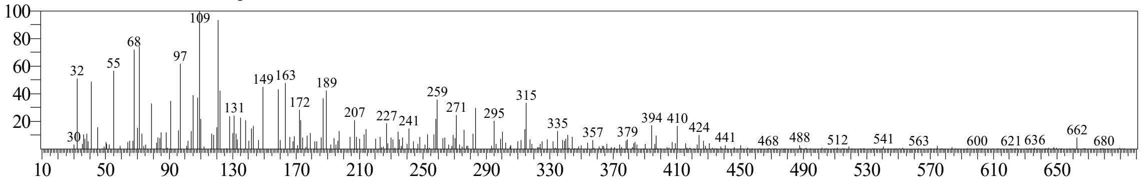

Hit#:1 Entry:191101 Library:NIST11.lib
SI:45 Formula:C30H50O CAS:469-39-6 MolWeight:426 RetIndex:2760
CompName:9,19-Cycloergost-24(28)-en-3-ol, 4,14-dimethyl-, (3.beta.,4.alpha.,5.alpha.)- $$ 9,19-Cyclo-5.alpha.,9.beta.-ergost-24(28)-en-3.beta.-ol, 4.alpha.,14

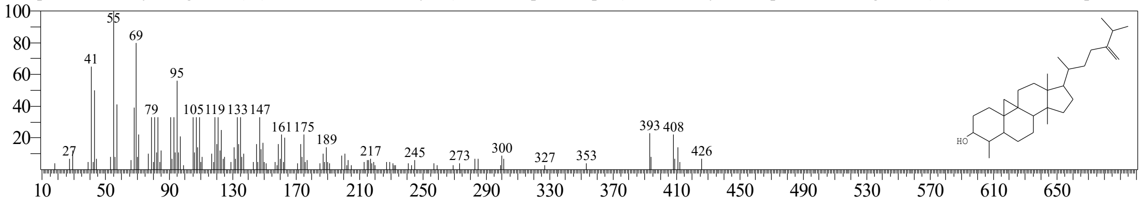

Hit#:2 Entry:207479 Library:NIST11.lib
SI:45 Formula:C36H54OS CAS:0-00-0 MolWeight:534 RetIndex:3742
CompName:17-(1,5-Dimethyl-3-phenylthiohex-4-enyl)-4,4,10,13,14-pentamethyl-2,3,4,5,6,7,10,11,12,13,14,15,16,17-tetradecahydro-1H-cyclopent(a)phenanth

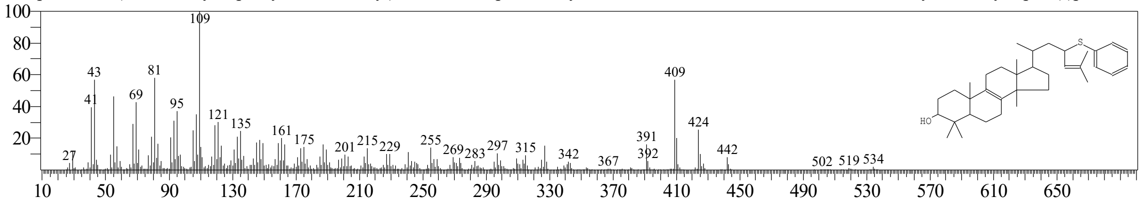

Hit#:3 Entry:132819 Library:NIST11.lib
SI:44 Formula:C21H34O2 CAS:7217-07-4 MolWeight:318 RetIndex:2290
CompName:4-Pregnene-3.beta.,20.beta.-diol $$ Pregn-4-ene-3,20-diol # $$

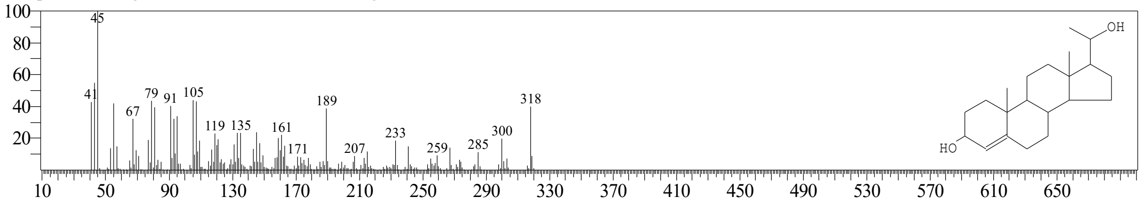

Hit#:4 Entry:196536 Library:NIST11.lib
SI:44 Formula:C28H48O4 CAS:56052-97-2 MolWeight:448 RetIndex:3179
CompName:Ergost-25-ene-3,5,6,12-tetrol, (3.beta.,5.alpha.,6.beta.,12.beta.)- $$ Ergost-25(27)-ene-3,5,6,12-tetrol # $$

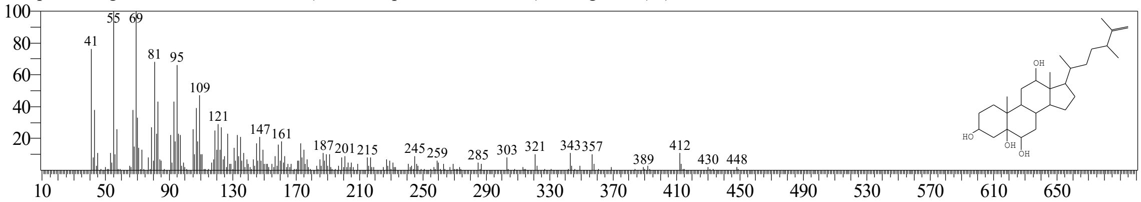

Hit#:5 Entry:143239 Library:NIST11.lib
SI:44 Formula:C21H32O3 CAS:0-00-0 MolWeight:332 RetIndex:2206
CompName:Isosteviol methyl ester $$ Methyl 12-hydroxyatis-16-en-18-oate # $$

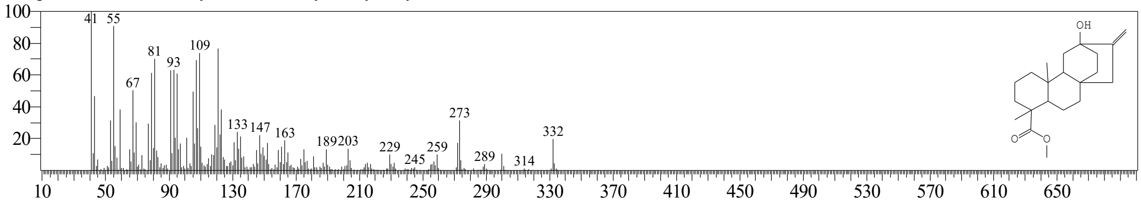

Qualitative Analysis Report

<< Target >>
Line#:117 R.Time:31.845(Scan#:5570) MassPeaks:349
RawMode:Averaged 31.840-31.850(5569-5571) BasePeak:69.10(5805)
BG Mode:Calc. from Peak Group 1 - Event 1 Scan

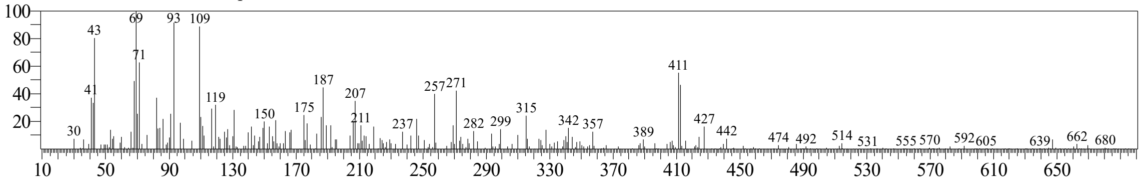

Hit#:1 Entry:191107 Library:NIST11.lib
SI:49 Formula:C30H50O CAS:79-63-0 MolWeight:426 RetIndex:2882
CompName:Lanosterol $$ Lanosta-8,24-dien-3-ol, (3.beta.)- $$ Lanosta-8,24-dien-3.beta.-ol $$ Botalan base 138 $$ Cholesta-8,24-dien-3-ol, 4,4,14-trimethyl-,

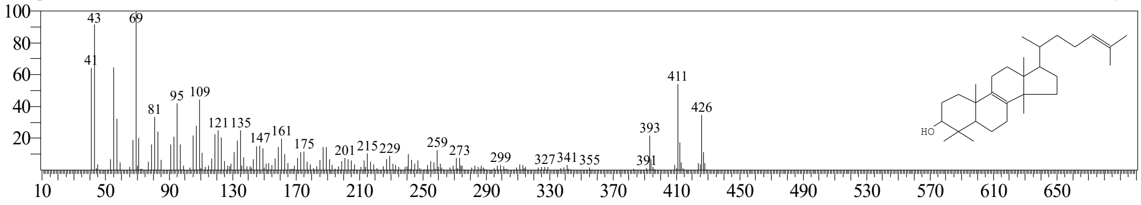

Hit#:2 Entry:88684 Library:NIST11.lib
SI:47 Formula:C14H27ClO2 CAS:74306-07-3 MolWeight:262 RetIndex:1805
CompName:3-Chlropropionic acid, undecyl ester $$ Undecyl 3-chloropropanoate # $$ Propanoic acid, 3-chloro, undecyl ester $$

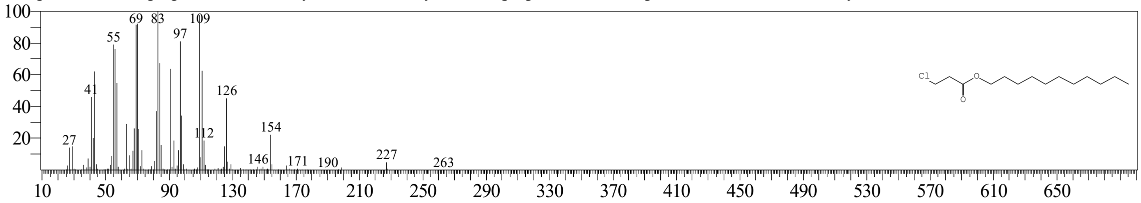

Hit#:3 Entry:195682 Library:NIST11.lib
SI:47 Formula:C30H52O2 CAS:0-00-0 MolWeight:444 RetIndex:3183
CompName:2,6,10,15,19,23-Hexamethyl-tetracosa-2,10,14,18,22-pentaene-6,7-diol $$ (10E,14E,18Z)-2,6,10,15,19,23-Hexamethyl-2,10,14,18,22-tetracosapen

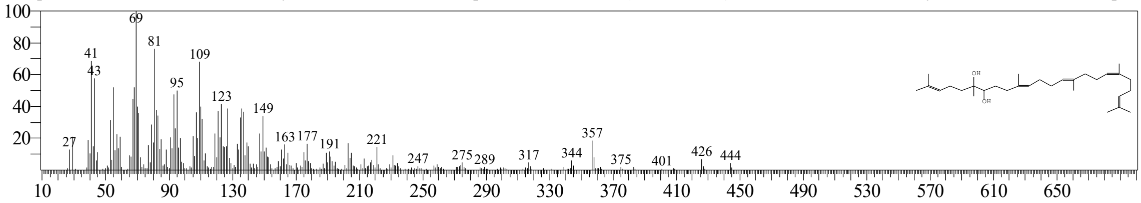

Hit#:4 Entry:24551 Library:NIST11.lib
SI:46 Formula:C10H16O2 CAS:5286-38-4 MolWeight:168 RetIndex:1171
CompName:7-Oxabicyclo[4.1.0]heptan-2-one, 6-methyl-3-(1-methylethyl)- $$ p-Menthan-3-one, 1,2-epoxy- $$ Piperitone oxide $$ Piperitone 1-oxide $$ Piper

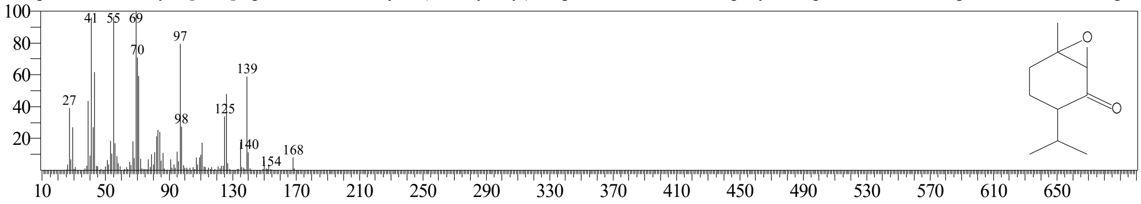

Hit#:5 Entry:60919 Library:NIST11.lib
SI:46 Formula:C15H28O CAS:20576-57-2 MolWeight:224 RetIndex:1661
CompName:2,10-Dodecadien-1-ol, 3,7,11-trimethyl-, (Z)- $$ (2Z)-3,7,11-Trimethyl-2,10-dodecadien-1-ol $$ (Z)-6,7-Dihydrofarnesol $$ 6,7-Dihydro-2-cis-farn

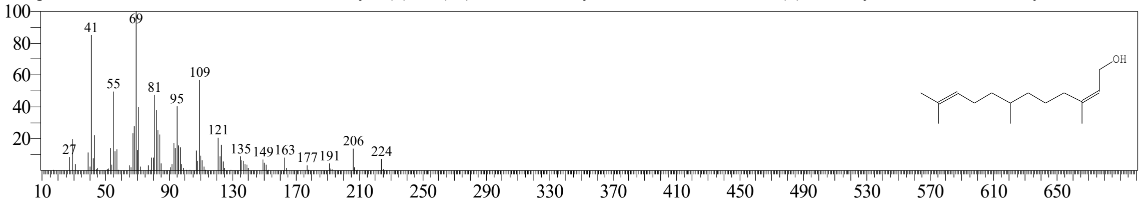

<< Target >>
Line#:118 R.Time:31.880(Scan#:5577) MassPeaks:331
RawMode:Averaged 31.875-31.885(5576-5578) BasePeak:411.30(7761)
BG Mode:Calc. from Peak Group 1 - Event 1 Scan

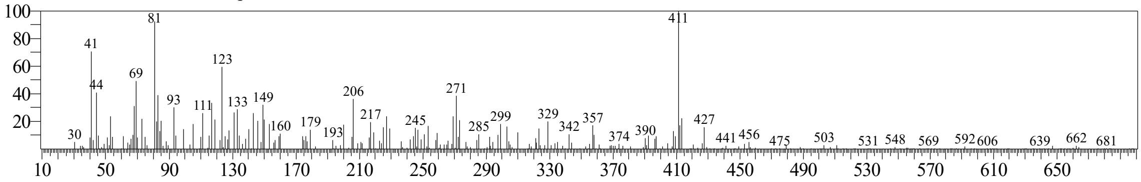

Hit#:1 Entry:191140 Library:NIST11.lib
SI:45 Formula:C30H50O CAS:5985-80-8 MolWeight:426 RetIndex:2788
CompName:Lanost-7-en-3-one

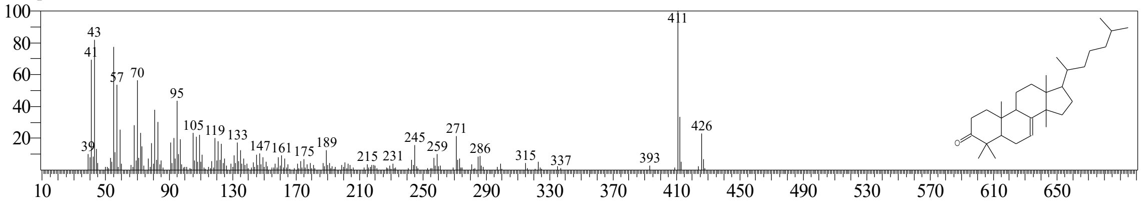

Hit#:2 Entry:191145 Library:NIST11.lib
SI:43 Formula:C30H50O CAS:0-00-0 MolWeight:426 RetIndex:2857
CompName:4.alpha.,14-Dimethyl-5.alpha.-ergosta-8,24(28)-dien-3.beta.-ol $$ 4,14-Dimethylergosta-5,24-dien-3-ol # $$

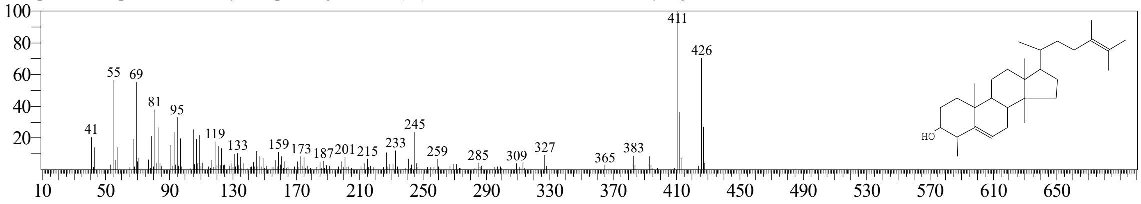

Hit#:3 Entry:191141 Library:NIST11.lib
SI:43 Formula:C30H50O CAS:16910-32-0 MolWeight:426 RetIndex:2826
CompName:Obtusifoliol $$ Ergosta-8,24(28)-dien-3-ol, 4,14-dimethyl-, (3.beta.,4.alpha.,5.alpha.)- $$ 5.alpha.-Ergosta-8,24(28)-dien-3.beta.-ol, 4.alpha.,14-din

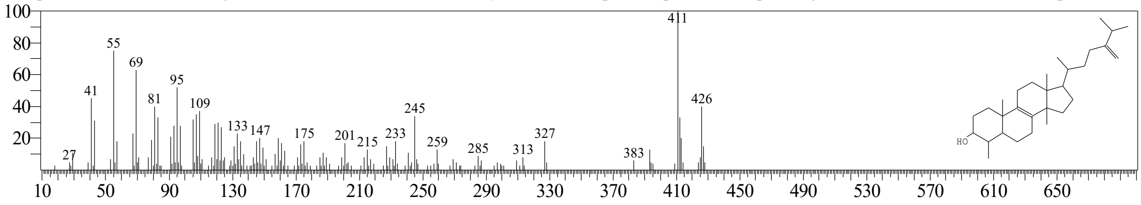

Hit#:4 Entry:201289 Library:NIST11.lib
SI:42 Formula:C30H50O2S CAS:55283-37-9 MolWeight:474 RetIndex:3260
CompName:Cholest-2-ene-2-carbothioic acid, 3-hydroxy-, O-ethyl ester, (5.alpha.)- $$ o-Ethyl 3-hydroxycholest-2-ene-2-carbothioate # $$

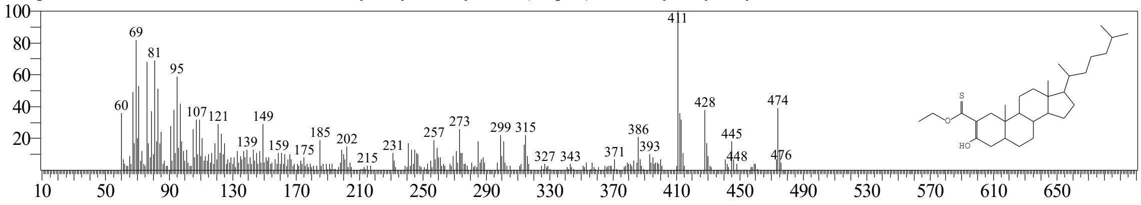

Hit#:5 Entry:138299 Library:NIST11.lib
SI:41 Formula:C18H30O5 CAS:108533-24-0 MolWeight:326 RetIndex:2021
CompName:2-Butyloxycarbonyloxy-1,1,10-trimethyl-6,9-epidioxydecalin

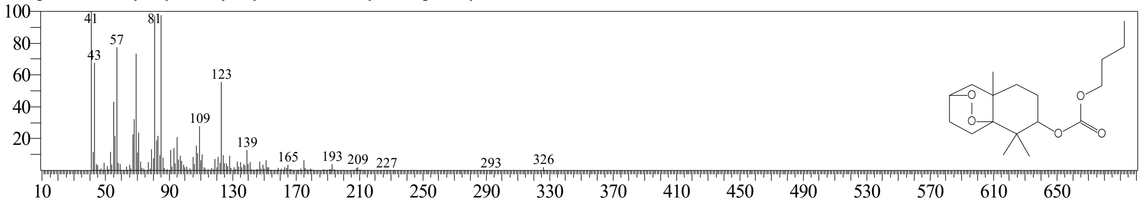

<< Target >>
Line#:119 R.Time:31.925(Scan#:5586) MassPeaks:355
RawMode:Averaged 31.920-31.930(5585-5587) BasePeak:133.05(9409)
BG Mode:Calc. from Peak Group 1 - Event 1 Scan

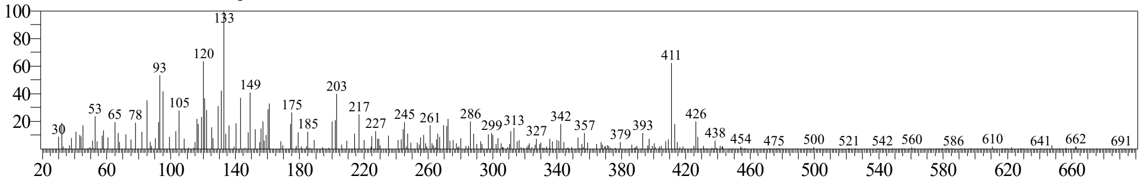

Hit#:1 Entry:46701 Library:NIST11.lib
SI:42 Formula:C15H24 CAS:0-00-0 MolWeight:204 RetIndex:1387
CompName:(-)-delta.-Panasinsine $$ 2,2,4a,8-Tetramethyl-1,2,2a,3,4,4a,7,8-octahydrocyclobuta[c]indene # $$

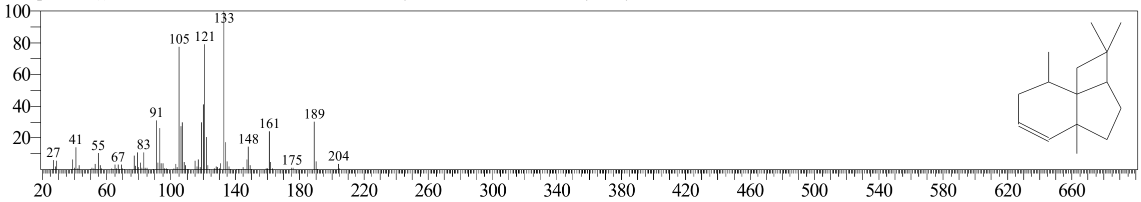

Hit#:2 Entry:46424 Library:NIST11.lib
SI:42 Formula:C12H21BN2 CAS:136704-98-8 MolWeight:204 RetIndex:0
CompName:Boron, (1,3-butanediiminato-N,N')-1,5-cyclooctanediyl-, (t-4)-

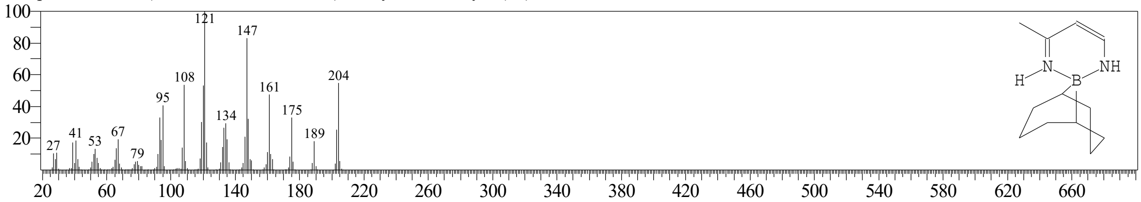

Hit#:3 Entry:46696 Library:NIST11.lib
SI:41 Formula:C15H24 CAS:0-00-0 MolWeight:204 RetIndex:1512
CompName:Thujopsene-(I2) $$ 1,1,8,9a-Tetramethyl-2,3,5,6,7,9a-hexahydro-1H-benzo[a]cycloheptene # $$

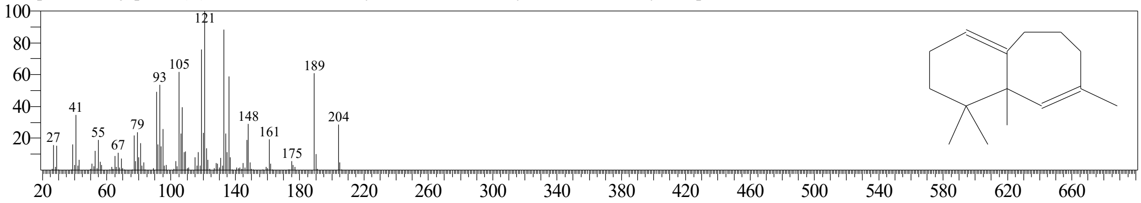

Hit#:4 Entry:118812 Library:NIST11.lib
SI:41 Formula:C19H24O3 CAS:968-93-4 MolWeight:300 RetIndex:2278
CompName:Testolactone $$ D-Homo-17a-oxaandrosta-1,4-diene-3,17-dione $$ .DELTA.1-Dehydrotestolactone $$ .DELTA.1-Dehydrotestololactone $$ .DELTA

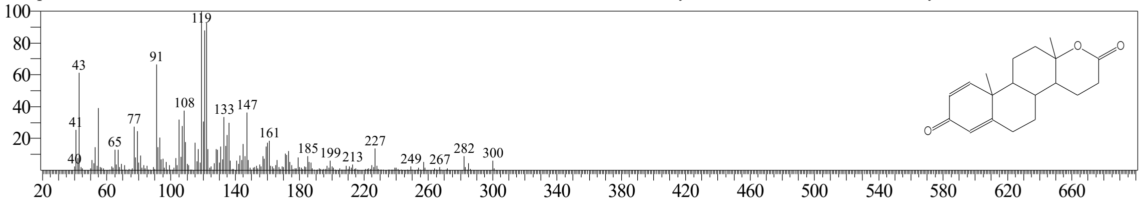

Hit#:5 Entry:56368 Library:NIST11.lib
SI:41 Formula:C15H22O CAS:102681-49-2 MolWeight:218 RetIndex:1438
CompName:2H-Cyclopropa[g]benzofuran, 4,5,5a,6,6a,6b-hexahydro-4,4,6b-trimethyl-2-(1-methylethenyl)- $$ 2-Isopropenyl-4,4,6b-trimethyl-4,5,5a,6,6a,6b-he

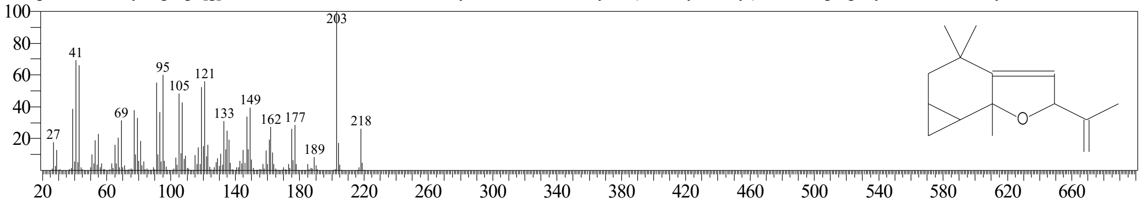

<< Target >>
Line#:120 R.Time:33.295(Scan#:5860) MassPeaks:371
RawMode:Averaged 33.290-33.300(5859-5861) BasePeak:43.05(7888)
BG Mode:Calc. from Peak Group 1 - Event 1 Scan

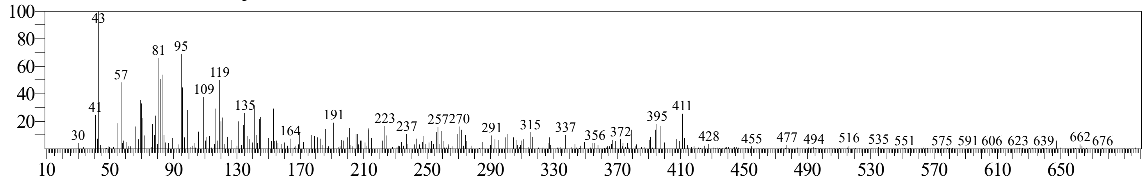

Hit#:1 Entry:186819 Library:NIST11.lib
SI:56 Formula:C29H48O CAS:2604-90-2 MolWeight:412 RetIndex:2714
CompName:Cholest-7-en-3-one, 4,4-dimethyl-, (5.alpha.)- $$ 5.alpha.-Cholest-7-en-3-one, 4,4-dimethyl- $$ 4,4-Dimethylcholest-7-en-3-one # $$

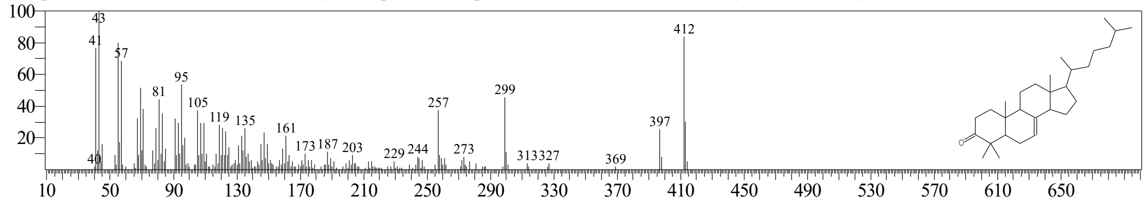

Hit#:2 Entry:207593 Library:NIST11.lib
SI:55 Formula:C37H76O CAS:105794-58-9 MolWeight:536 RetIndex:3942
CompName:1-Heptatriacotanol $$ 1-Heptatriacontanol # $$

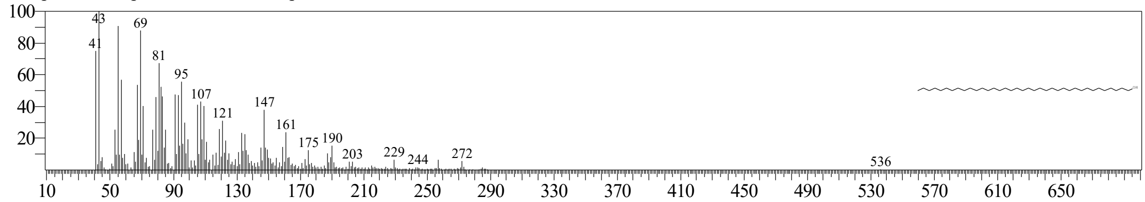

Hit#:3 Entry:191101 Library:NIST11.lib
SI:54 Formula:C30H50O CAS:469-39-6 MolWeight:426 RetIndex:2760
CompName:9,19-Cycloergost-24(28)-en-3-ol, 4,14-dimethyl-, (3.beta.,4.alpha.,5.alpha.)- $$ 9,19-Cyclo-5.alpha.,9.beta.-ergost-24(28)-en-3.beta.-ol, 4.alpha.,14

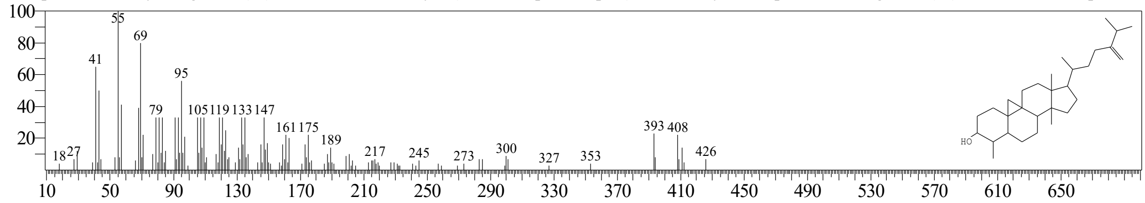

Hit#:4 Entry:200397 Library:NIST11.lib
SI:54 Formula:C32H52O2 CAS:10376-42-8 MolWeight:468 RetIndex:2900
CompName:9,19-Cycloergost-24(28)-en-3-ol, 4,14-dimethyl-, acetate, (3.beta.,4.alpha.,5.alpha.)- $$ 1-(4-Isopropyl-1-methyl-4-pentenyl)-3a,6,12a-trimethyltetra

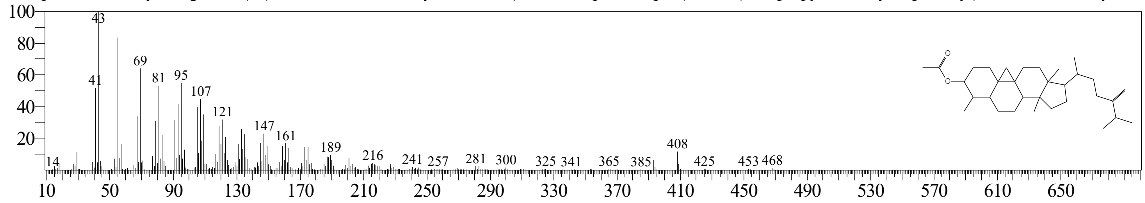

Hit#:5 Entry:128998 Library:NIST11.lib
SI:54 Formula:C13H21Cl3O2 CAS:0-00-0 MolWeight:314 RetIndex:1876
CompName:Trichloroacetic acid, undec-2-enyl ester

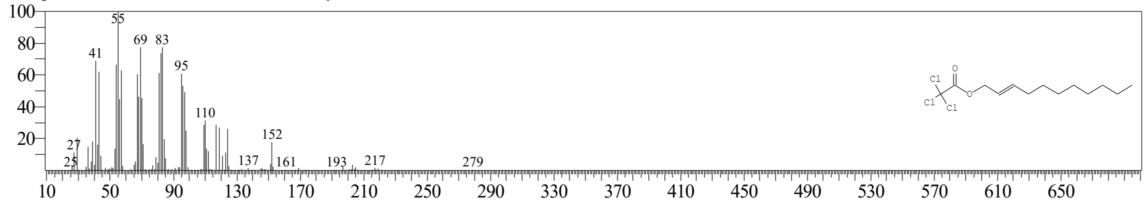

Qualitative Analysis Report

<< Target >>
Line#:121 R.Time:33.830(Scan#:5967) MassPeaks:348
RawMode:Averaged 33.825-33.835(5966-5968) BasePeak:107.10(7642)
BG Mode:Calc. from Peak Group 1 - Event 1 Scan

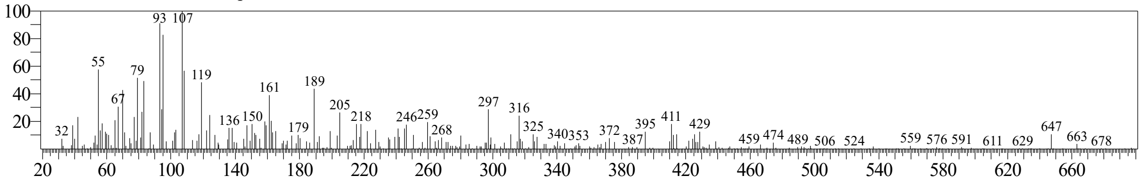

Hit#:1 Entry:46668 Library:NIST11.lib
SI:53 Formula:C15H24 CAS:116-04-1 MolWeight:204 RetIndex:1574
CompName:.beta.-Humulene $$ 1,4,4-Trimethyl-8-methylene-1,5-cycloundecadiene # $$

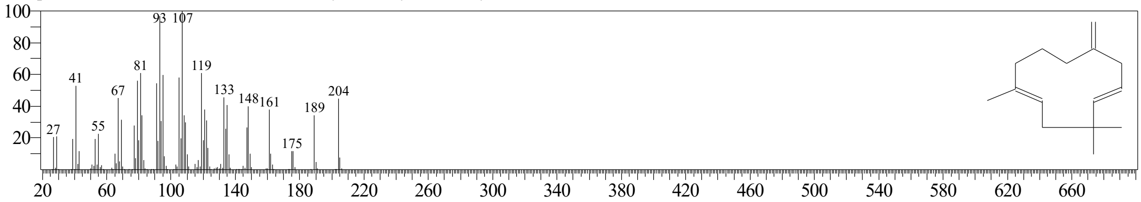

Hit#:2 Entry:46664 Library:NIST11.lib
SI:53 Formula:C15H24 CAS:0-00-0 MolWeight:204 RetIndex:1475
CompName:Cycloheptane, 4-methylene-1-methyl-2-(2-methyl-1-propen-1-yl)-1-vinyl- $$ 1-Methyl-4-methylene-2-(2-methyl-1-propenyl)-1-vinylcycloheptane #

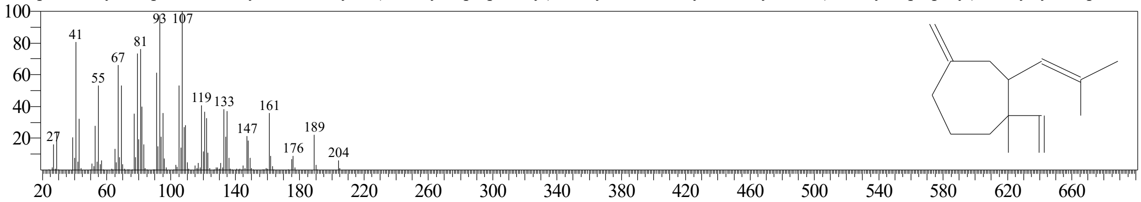

Hit#:3 Entry:46719 Library:NIST11.lib
SI:52 Formula:C15H24 CAS:475-20-7 MolWeight:204 RetIndex:1398
CompName:Longifolene $$ 1,4-Methanoazulene, decahydro-4,8,8-trimethyl-9-methylene-, [1S-(1.alpha.,3a.beta.,4.alpha.,8a.beta.)]- $$ 1,4-Methanoazulene, de

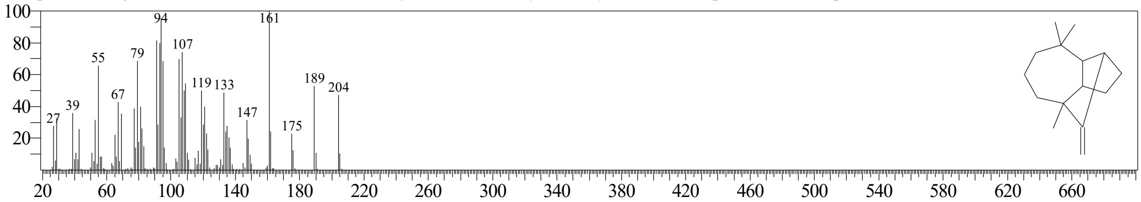

Hit#:4 Entry:46698 Library:NIST11.lib
SI:52 Formula:C15H24 CAS:0-00-0 MolWeight:204 RetIndex:1391
CompName:Tricyclo[6.3.0.0(2,4)]undec-8-ene, 3,3,7,11-tetramethyl- $$ 1,1,4,7-Tetramethyl-1a,2,3,4,6,7,7a,7b-octahydro-1H-cyclopropa[e]azulene # $$

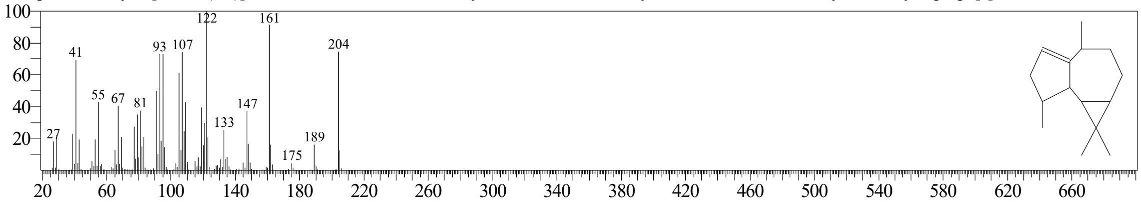

Hit#:5 Entry:46626 Library:NIST11.lib
SI:52 Formula:C15H24 CAS:0-00-0 MolWeight:204 RetIndex:1456
CompName:Bicyclo[5.3.0]decane, 2-methylene-5-(1-methylvinyl)-8-methyl- $$ 7-Isopropenyl-1-methyl-4-methylenedecahydroazulene # $$

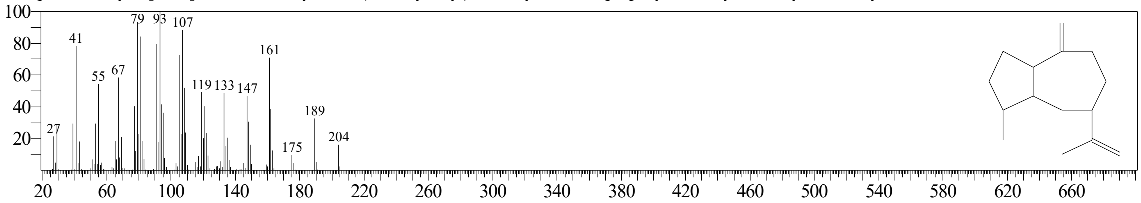

<< Target >>
Line#:122 R.Time:33.945(Scan#:5990) MassPeaks:373
RawMode:Averaged 33.940-33.950(5989-5991) BasePeak:95.05(10793)
BG Mode:Calc. from Peak Group 1 - Event 1 Scan

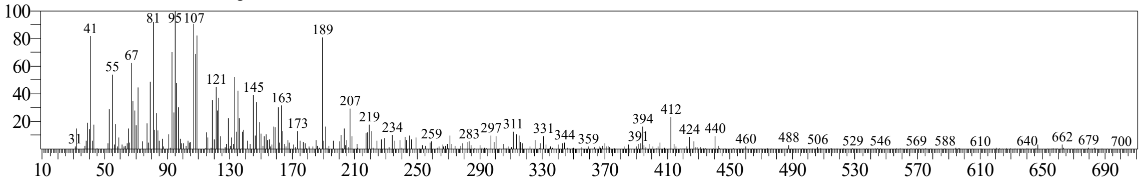

Hit#:1 Entry:191097 Library:NIST11.lib
SI:76 Formula:C30H50O CAS:545-47-1 MolWeight:426 RetIndex:2848
CompName:Lupeol $$ Lup-20(29)-en-3-ol, (3.beta.)- $$ Lup-20(29)-en-3.beta.-ol $$ .beta.-Viscol $$ Clerodol $$ Fagarasterol $$ Fagarsterol $$ Lupenol $$ Mo

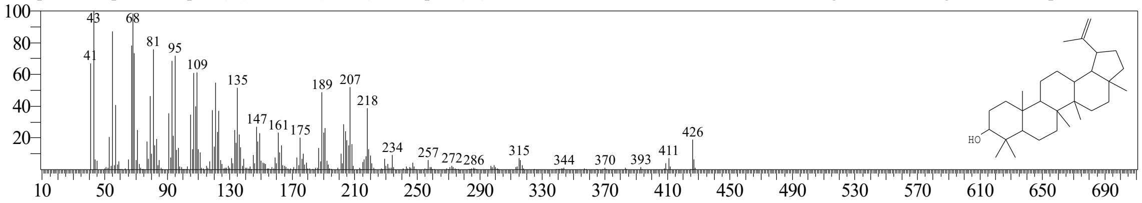

Hit#:2 Entry:76882 Library:NIST11.lib
SI:75 Formula:C18H30 CAS:0-00-0 MolWeight:246 RetIndex:1649
CompName:d-Norandrostane (5.alpha.,14.alpha.) $$ 8a,10a-Dimethylhexadecahydrocyclobuta[a]phenanthrene # $$

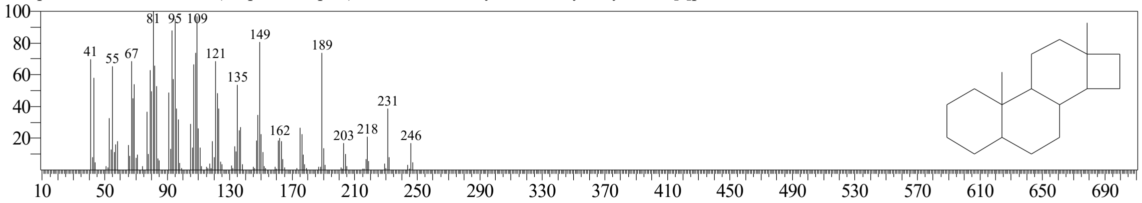

Hit#:3 Entry:123529 Library:NIST11.lib
SI:75 Formula:C20H34O2 CAS:7220-78-2 MolWeight:306 RetIndex:2400
CompName:4,8,13-Cyclotetradecatriene-1,3-diol, 1,5,9-trimethyl-12-(1-methylethyl)- $$ FW 306 $$ 4,8,13-Duvatriene-1,3-Diol $$ 12-Isopropyl-1,5,9-trimethy

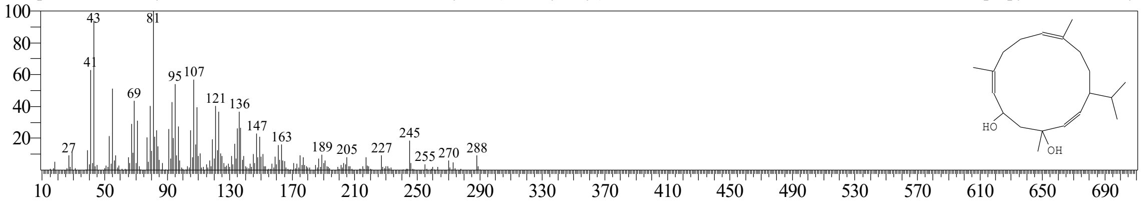

Hit#:4 Entry:143389 Library:NIST11.lib
SI:75 Formula:C23H40O CAS:106932-90-5 MolWeight:332 RetIndex:2385
CompName:Oct-5-en-2-ol, 8-(1,4,4a,5,6,7,8,8a-octahydro-2, 5, 5, 8a-tetramethylnaphth-1-yl)-6-methyl- $$ (5E)-8-(2,5,5,8a-Tetramethyl-1,4,4a,5,6,7,8,8a-octah

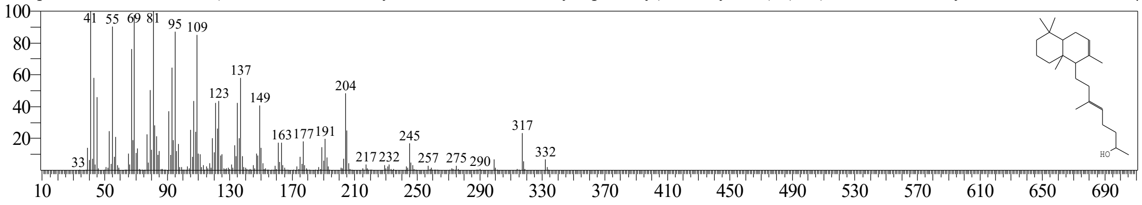

Hit#:5 Entry:110901 Library:NIST11.lib
SI:74 Formula:C20H34O CAS:25269-17-4 MolWeight:290 RetIndex:2211
CompName:Thunbergol $$ 4-Isopropyl-1,7,11-trimethyl-2,7,11-cyclotetradecatrien-1-ol # $$

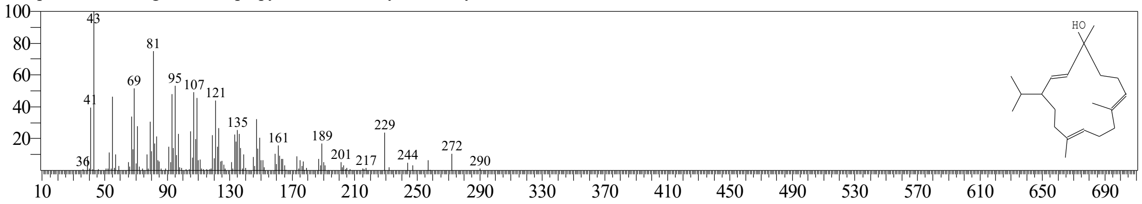

Supplement: Supplementary file 1 [file pharmaceuticals-19-01635-s001.zip › File S1—GC-MS analysis/Ozoroa paniculosa DCM (Ramile).pdf]
