# Supplementary material for: Phyto-Mediated Synthesized Gold Nanoparticles (AuNPs) from Ozoroa paniculosa Leaf Extracts: Chemical Characterization and Antidiabetic Evaluation
Source: Pharmaceuticals (Basel). 2026 Oct 15;19(10):1635. doi: 10.3390/ph19101635 (PMC13708053; doi:10.3390/ph19101635)

Qualitative Analysis Report

Sample Information

Sample Type	: Unknown
Sample Name	: C:\GCMSData\Raw Data\Ext - UniZulu\16-E-C.qgd
Sample ID	: Prince
Vial #	: 98
Method File	: C:\GCMSData\general - split - extended.qgm
Tuning File	: C:\GCMSsolution\System\Tune1\Normal Conc - 2025 04.qgt

Chromatogram OPH hex

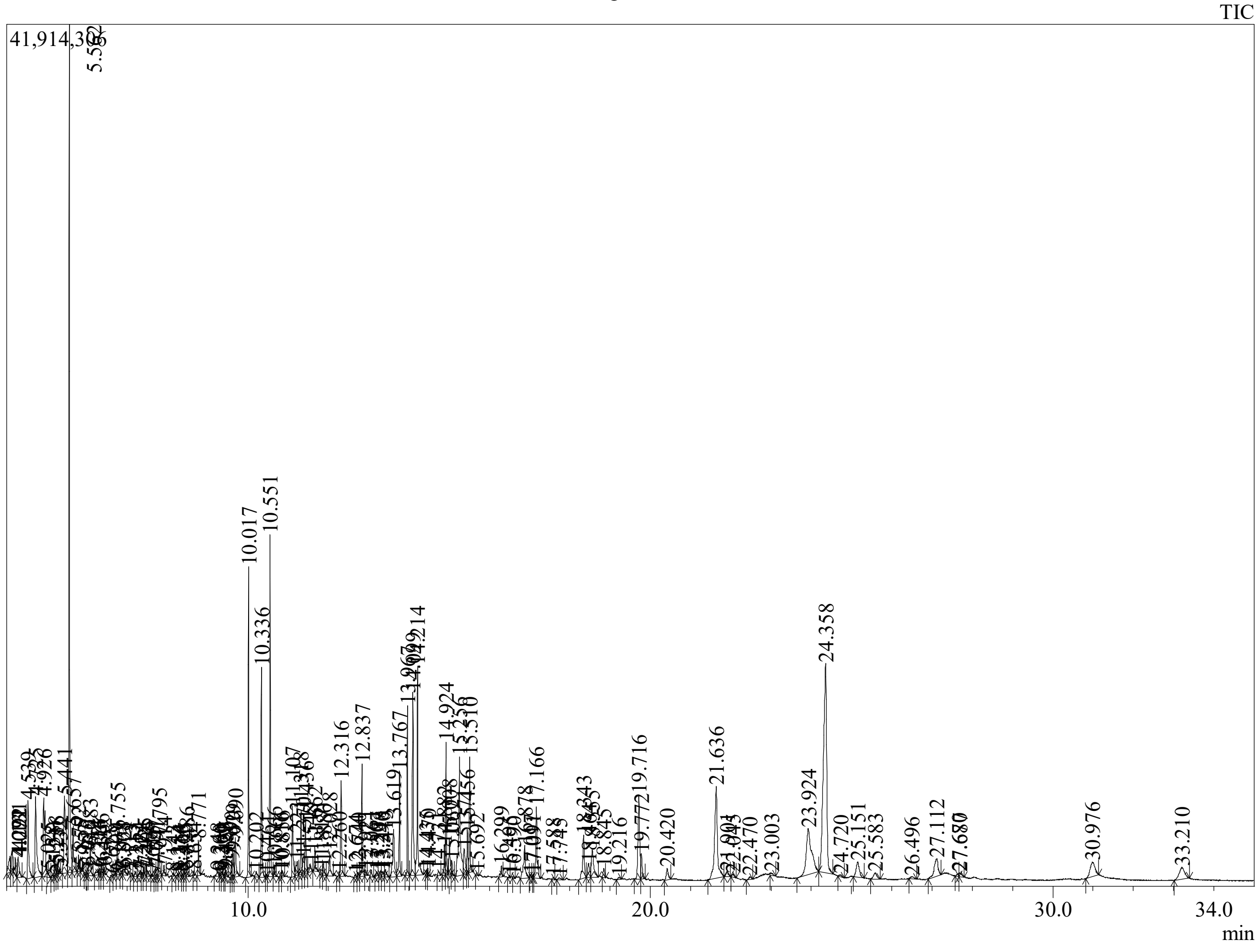

Peak Report TIC

Peak#	R.Time	Area	Area%	Height	Height%	A/H	Name
1	4.072	2621706	0.48	669406	0.26	3.92	Cycloheptanone, 3-methyl-, (R)-
2	4.131	2252997	0.41	1026245	0.40	2.20	Octane, 4-methyl-
3	4.221	1278704	0.24	830741	0.33	1.54	Heptane, 4-(1-methylethyl)-
4	4.292	2638246	0.49	981232	0.39	2.69	o-Xylene
5	4.539	6641508	1.22	3784723	1.49	1.75	Hexane, 2,4-dimethyl-
6	4.725	10409215	1.92	3932133	1.55	2.65	2-Hexanone, 3,3-dimethyl-
7	4.926	12150461	2.24	3809981	1.50	3.19	Ethanone, 1-(3-ethyloxiranyl)-
8	5.025	127588	0.02	124148	0.05	1.03	Hexanoic acid, 4-octyl ester
9	5.138	290170	0.05	142588	0.06	2.04	Decane
10	5.218	990145	0.18	544466	0.21	1.82	Nonane, 2-methyl-
11	5.256	642954	0.12	490025	0.19	1.31	Nonane, 2-methyl-
12	5.332	294632	0.05	238349	0.09	1.24	Nonane, 3-methyl-
13	5.441	12993107	2.39	3759814	1.48	3.46	2-Hexene, 4,4,5-trimethyl-
14	5.562	57208864	10.53	41233873	16.22	1.39	.beta.-Myrcene
15	5.657	4353460	0.80	2227310	0.88	1.95	Nonane
16	5.792	1298708	0.24	806673	0.32	1.61	.alpha.-Phellandrene
17	5.855	167459	0.03	170808	0.07	0.98	Sulfurous acid, hexyl pentadecyl ester
18	5.953	178678	0.03	133283	0.05	1.34	1-Octadecanesulphonyl chloride
19	6.012	680435	0.13	361882	0.14	1.88	Benzene, 1,2-diethyl-
20	6.083	3244690	0.60	1384490	0.54	2.34	Bicyclo[3.1.0]hexane, 4-methylene-1-(1-methyl
21	6.274	752854	0.14	286389	0.11	2.63	Hexane, 2,2,3,3-tetramethyl-
22	6.316	350111	0.06	317235	0.12	1.10	Heptane, 5-ethyl-2-methyl-
23	6.366	1057764	0.19	596591	0.23	1.77	1-Nonene, 4,6,8-trimethyl-
24	6.432	533858	0.10	362777	0.14	1.47	Decane, 3-methyl-
25	6.625	379329	0.07	109031	0.04	3.48	Benzene, 1-ethyl-2,3-dimethyl-
26	6.709	247215	0.05	128667	0.05	1.92	.beta.-curcumene

Qualitative Analysis Report

Peak#	R.Time	Area	Area%	Height	Height%	A/H	Name
27	6.755	3007205	0.55	2127836	0.84	1.41	Decane, 2,9-dimethyl-
28	6.868	434982	0.08	219645	0.09	1.98	1,6-Octadien-3-ol, 3,7-dimethyl-
29	6.921	987689	0.18	287154	0.11	3.44	2-Octen-1-ol, 3,7-dimethyl-, isobutyrate, (Z)-
30	7.113	332598	0.06	147768	0.06	2.25	Spiro[3.5]nona-5,7-dien-1-one, 5,9,9-trimethyl-
31	7.212	534112	0.10	222784	0.09	2.40	trans-2-Undecen-1-ol
32	7.324	641434	0.12	277584	0.11	2.31	1-Undecene, 4-methyl-
33	7.371	236455	0.04	181960	0.07	1.30	Undecane, 4-methyl-
34	7.425	656789	0.12	442165	0.17	1.49	Undecane, 2,4-dimethyl-
35	7.496	757127	0.14	289831	0.11	2.61	Tridecane, 7-propyl-
36	7.615	107492	0.02	114030	0.04	0.94	Decane, 4-ethyl-
37	7.697	624850	0.12	418022	0.16	1.49	3-Methyl-2-butenoic acid, 2-tetrahydrofurylmet
38	7.741	1172838	0.22	393066	0.15	2.98	2(3H)-Furanone, dihydro-5-methyl-5-(2-methyl
39	7.795	3177647	0.59	1933589	0.76	1.64	Dodecane, 2-methyl-
40	7.914	1098676	0.20	527732	0.21	2.08	Tridecane, 4,8-dimethyl-
41	8.143	293539	0.05	168527	0.07	1.74	n-Caproic acid vinyl ester
42	8.254	145778	0.03	107360	0.04	1.36	Cyclopentane, 1-butyl-2-pentyl-
43	8.316	323080	0.06	138909	0.05	2.33	2-Bromononane
44	8.366	226306	0.04	194544	0.08	1.16	Undecane, 4,8-dimethyl-
45	8.425	606389	0.11	256620	0.10	2.36	Undecane, 2,10-dimethyl-
46	8.486	1428213	0.26	1031591	0.41	1.38	n-Caproic acid vinyl ester
47	8.654	276458	0.05	230356	0.09	1.20	1,5-Hexadien-3-ol, acetate
48	8.771	2882872	0.53	1732929	0.68	1.66	Decane, 2-methyl-
49	9.248	721997	0.13	239294	0.09	3.02	Cyclopentane, heneicosyl-
50	9.300	177024	0.03	145752	0.06	1.21	Undecane, 4-methyl-
51	9.360	359622	0.07	232761	0.09	1.55	Tridecane, 2-methyl-
52	9.421	289802	0.05	202469	0.08	1.43	Tridecane, 4,8-dimethyl-
53	9.455	407162	0.07	355807	0.14	1.14	Dodecane, 2,6,11-trimethyl-
54	9.590	1229238	0.23	852142	0.34	1.44	.alfa.-Copaene
55	9.629	2195917	0.40	1226295	0.48	1.79	1-Dodecene
56	9.690	3956683	0.73	1904208	0.75	2.08	Octane, 2,3,3-trimethyl-
57	10.017	19138494	3.52	15096612	5.94	1.27	Bicyclo[7.2.0]undec-4-ene, 4,11,11-trimethyl-8
58	10.202	831452	0.15	266050	0.10	3.13	Undecane, 4,8-dimethyl-
59	10.336	13389338	2.47	10231178	4.02	1.31	Humulene
60	10.461	190581	0.04	130078	0.05	1.47	1,6-Cyclodecadiene, 1-methyl-5-methylene-8-(
61	10.551	22122674	4.07	16634114	6.54	1.33	1,6-Cyclodecadiene, 1-methyl-5-methylene-8-(
62	10.666	825284	0.15	506847	0.20	1.63	Bicyclogermacrene
63	10.816	403582	0.07	233951	0.09	1.73	cis-muurola-3,5-diene
64	10.856	602136	0.11	314156	0.12	1.92	4,8,12-Tetradecatrien-1-ol, 5,9,13-trimethyl-
65	11.107	5932694	1.09	3439114	1.35	1.73	Cyclohexanemethanol, 4-ethenyl-.alpha.,.alpha
66	11.223	1828237	0.34	680378	0.27	2.69	1,5-Cyclodecadiene, 1,5-dimethyl-8-(1-methyle
67	11.318	4928602	0.91	3051100	1.20	1.62	1-Pentadecene
68	11.370	2066635	0.38	809928	0.32	2.55	1-Hydroxy-1,7-dimethyl-4-isopropyl-2,7-cycloc
69	11.436	4343494	0.80	2539506	1.00	1.71	Caryophyllene oxide
70	11.548	1345454	0.25	382537	0.15	3.52	Octadecanal
71	11.662	2032293	0.37	1216574	0.48	1.67	3-Oxatricyclo[4.1.1.0(2,4)]octane, 2,7,7-trimetl
72	11.817	611790	0.11	242279	0.10	2.53	8-epi-.gama.-eudesmol
73	11.880	1227779	0.23	559300	0.22	2.20	1-Naphthalenol, 1,2,3,4,4a,7,8,8a-octahydro-1,
74	12.028	2681576	0.49	1194452	0.47	2.25	2-Naphthalenemethanol, decahydro-.alpha.,.alp
75	12.260	513692	0.09	253008	0.10	2.03	Ledene oxide-(II)
76	12.316	8598809	1.58	4678414	1.84	1.84	Octadecanal
77	12.674	320161	0.06	150770	0.06	2.12	1-Tridecene
78	12.740	505494	0.09	237815	0.09	2.13	Tridecanoic acid
79	12.837	7614812	1.40	5480797	2.16	1.39	E-15-Heptadecenal
80	12.921	632863	0.12	315318	0.12	2.01	2-Dodecenal, (E)-
81	13.061	787417	0.14	336807	0.13	2.34	Octadecanal
82	13.207	342783	0.06	212123	0.08	1.62	2-Undecanone, 6,10-dimethyl-
83	13.298	541390	0.10	267741	0.11	2.02	2-Naphthalenemethanol, 2,3,4,4a,5,6,7,8-octah
84	13.340	748073	0.14	233454	0.09	3.20	6-Isopropenyl-4,8a-dimethyl-1,2,3,5,6,7,8,8a-o
85	13.413	982692	0.18	350324	0.14	2.81	Pentadecanoic acid
86	13.619	6905826	1.27	2319059	0.91	2.98	9,12,15-Octadecatrien-1-ol, (Z,Z,Z)-
87	13.767	8385418	1.54	5126994	2.02	1.64	Pentadecanoic acid, 14-methyl-, methyl ester
88	13.967	9917041	1.83	8253234	3.25	1.20	1,6,10-Dodecatriene, 7,11-dimethyl-3-methylen
89	14.099	17104197	3.15	8914026	3.51	1.92	n-Hexadecanoic acid
90	14.214	15562860	2.87	10166527	4.00	1.53	3-Tetradecene, (E)-
91	14.435	295133	0.05	234938	0.09	1.26	Hexadecanoic acid, 15-methyl-, methyl ester
92	14.470	419834	0.08	345839	0.14	1.21	1,6,10,14-Hexadecatetraen-3-ol, 3,7,11,15-tetra
93	14.731	545161	0.10	226142	0.09	2.41	Eicosanoic acid
94	14.882	1954238	0.36	1457218	0.57	1.34	9,12-Octadecadienoic acid, methyl ester
95	14.924	8220222	1.51	6418361	2.52	1.28	9,12,15-Octadecatrienoic acid, methyl ester, (Z,
96	15.008	3746230	0.69	1734633	0.68	2.16	Phytol
97	15.067	1043932	0.19	702307	0.28	1.49	Hexadecanoic acid, 15-methyl-, methyl ester
98	15.256	17486967	3.22	5763472	2.27	3.03	9,12,15-Octadecatrienoic acid, (Z,Z,Z)-
99	15.371	3529127	0.65	1264940	0.50	2.79	Octadecanoic acid, 2-(2-hydroxyethoxy)ethyl e
100	15.456	3527884	0.65	2130055	0.84	1.66	Hexadecanoic acid, butyl ester
101	15.510	8076241	1.49	5639450	2.22	1.43	E-15-Heptadecenal
102	15.692	255988	0.05	129445	0.05	1.98	4-Chlorobutyric acid, pentadecyl ester
103	16.299	1080010	0.20	534370	0.21	2.02	Heptadecane, 9-octyl-
104	16.495	147192	0.03	106906	0.04	1.38	Benzoic acid, tridecyl ester
105	16.590	192208	0.04	112520	0.04	1.71	Pentadecanal-
106	16.878	5283625	0.97	1599607	0.63	3.30	9,12,15-Octadecatrienoic acid, (Z,Z,Z)-
107	17.017	318956	0.06	168591	0.07	1.89	5,9,13-Pentadecatrien-2-one, 6,10,14-trimethyl-
108	17.091	267800	0.05	195122	0.08	1.37	Octadecanoic acid, butyl ester
109	17.166	8604320	1.58	3456429	1.36	2.49	E-15-Heptadecenal
110	17.588	237459	0.04	123204	0.05	1.93	Octadecanal
111	17.745	460598	0.08	100503	0.04	4.58	
112	18.343	5787784	1.07	2105744	0.83	2.75	Heptadecane, 9-octyl-

Qualitative Analysis Report

Peak#	R.Time	Area	Area%	Height	Height%	A/H	Name
113	18.464	2304943	0.42	701212	0.28	3.29	Benzene, 1-methyl-4-(2-pentenyloxy)-
114	18.555	5184989	0.95	1337809	0.53	3.88	Phenol, 3-pentadecyl-
115	18.845	1156337	0.21	452309	0.18	2.56	Diisooctyl phthalate
116	19.216	341059	0.06	116777	0.05	2.92	Hexadecanoic acid, 1-[[[(2-aminoethoxy)hydro:
117	19.716	12142929	2.24	4087165	1.61	2.97	E-15-Heptadecenal
118	19.772	3002870	0.55	1233000	0.48	2.44	Dodecane, 4-methyl-
119	20.420	1936657	0.36	552208	0.22	3.51	Octadecanal
120	21.636	21867773	4.03	4479868	1.76	4.88	Eicosane
121	21.901	838930	0.15	214924	0.08	3.90	2-Isoamyl-6-methylpyrazine
122	22.045	464453	0.09	132196	0.05	3.51	Oxalic acid, heptyl 2-methylphenyl ester
123	22.470	608916	0.11	159981	0.06	3.81	Octadecanal
124	23.003	504290	0.09	126410	0.05	3.99	Acetic acid, 2-(1-piperidyl)-, [4-aminofurazanyl
125	23.924	20287542	3.74	2240339	0.88	9.06	1-Hexacosene
126	24.358	58467999	10.76	10155781	3.99	5.76	Squalene
127	24.720	374359	0.07	101919	0.04	3.67	Urs-12-ene
128	25.151	4214631	0.78	742185	0.29	5.68	Octadecanal
129	25.583	1844890	0.34	291049	0.11	6.34	4,8,13-Cyclotetradecatriene-1,3-diol, 1,5,9-trim
130	26.496	640763	0.12	120657	0.05	5.31	Naphthalene, 1,2,3,4,4a,5,6,8a-octahydro-4a,8-
131	27.112	5938040	1.09	876436	0.34	6.78	Eicosane
132	27.670	540758	0.10	178194	0.07	3.03	Z,Z-6,28-Heptatriactontadien-2-one
133	27.687	709096	0.13	182285	0.07	3.89	1-Cyclopentylethanol, chlorodifluoroacetate
134	30.976	5791626	1.07	725788	0.29	7.98	Heptadecyl heptafluorobutyrate
135	33.210	5447772	1.00	606219	0.24	8.99	Octadecanal
		543132986	100.00	254244662	100.00		

Library

<< Target >>
Line#:1 R.Time:4.070(Scan#:15) MassPeaks:353
RawMode:Averaged 4.065-4.075(14-16) BasePeak:41.05(64346)
BG Mode:Calc. from Peak Group 1 - Event 1 Scan

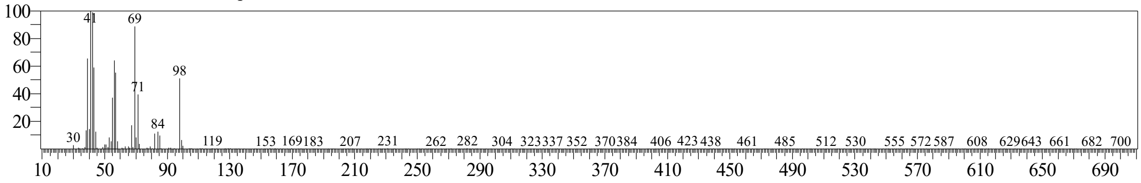

Hit#:1 Entry:6504 Library:NIST11.lib
SI:84 Formula:C8H14O CAS:13609-58-0 MolWeight:126 RetIndex:1072
CompName:Cycloheptanone, 3-methyl-, (R)- $$ Cycloheptanone, 3-methyl-, (+)- $$ 3-Methylcycloheptanone # $$

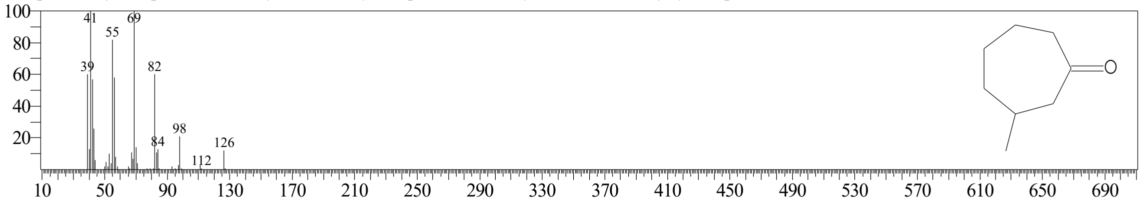

Hit#:2 Entry:4119 Library:NIST11.lib
SI:83 Formula:C7H14O CAS:7045-79-6 MolWeight:114 RetIndex:790
CompName:Oxetane, 2-methyl-4-propyl- $$ 2-Methyl-4-propyloxetane # $$

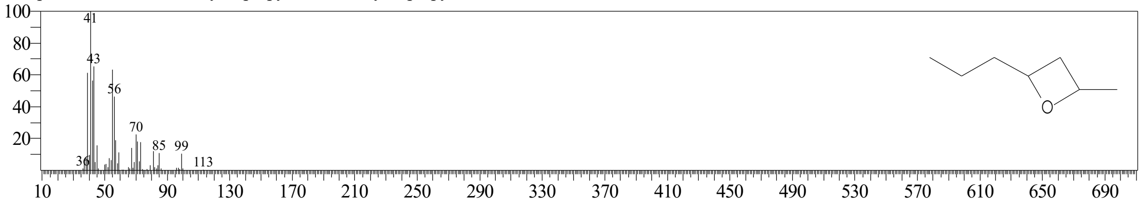

Hit#:3 Entry:1689 Library:NIST11.lib
SI:83 Formula:C6H10O CAS:1757-42-2 MolWeight:98 RetIndex:832
CompName:Cyclopentanone, 3-methyl- $$ 3-Methyl-1-cyclopentanone $$ 3-Methylcyclopentanone $$

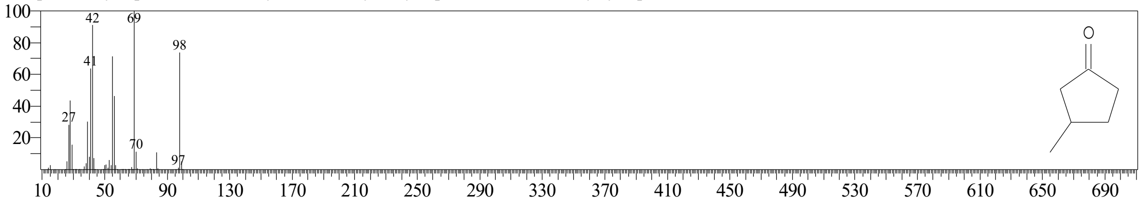

Hit#:4 Entry:41283 Library:NIST11.lib
SI:82 Formula:C10H22B2O2 CAS:0-00-0 MolWeight:196 RetIndex:0
CompName:2,5-Dibora-1,4-dioxane, 2,3,5,6-tetraethyl- $$ 2,3,5,6-Tetraethyl-1,4,2,5-dioxadiborinane # $$

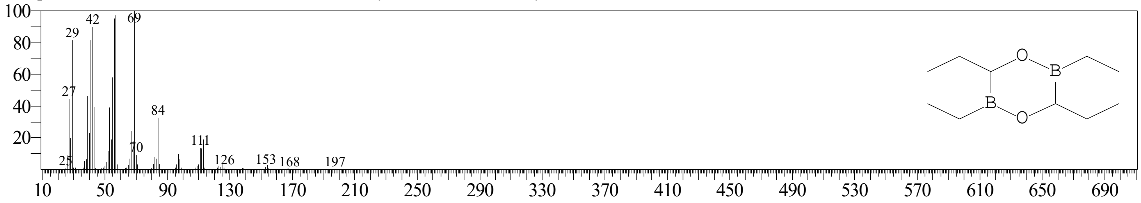

Hit#:5 Entry:11426 Library:NIST11.lib
SI:82 Formula:C10H20 CAS:0-00-0 MolWeight:140 RetIndex:999
CompName:Cyclobutane, 1-butyl-2-ethyl- $$ 1-Butyl-2-ethylcyclobutane # $$

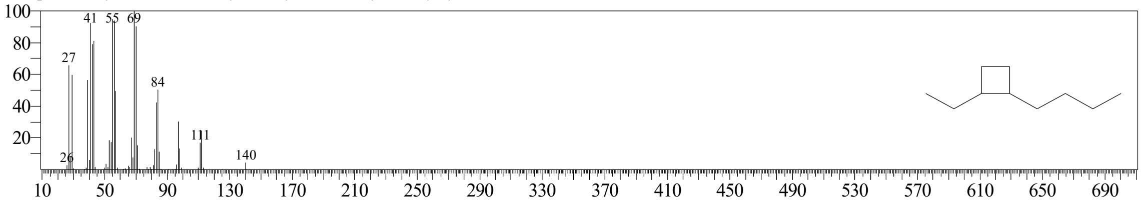

Qualitative Analysis Report

<< Target >>
Line#:2 R.Time:4.130(Scan#:27) MassPeaks:343
RawMode:Averaged 4.125-4.135(26-28) BasePeak:43.05(253066)
BG Mode:Calc. from Peak Group 1 - Event 1 Scan

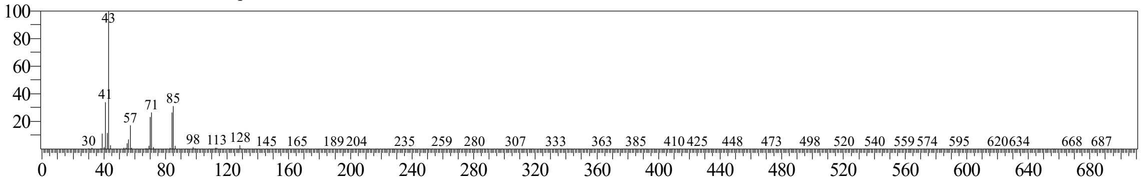

Hit#:1 Entry:7426 Library:NIST11.lib
SI:95 Formula:C9H20 CAS:2216-34-4 MolWeight:128 RetIndex:852
CompName:Octane, 4-methyl- $$ 4-Methyloctane $$ 1,3-Dimethylheptane $$

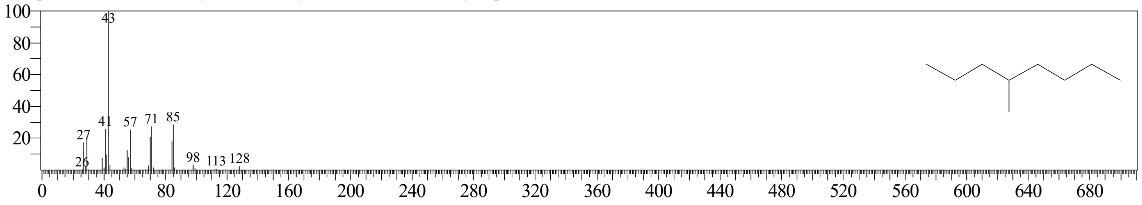

Hit#:2 Entry:7413 Library:NIST11.lib
SI:93 Formula:C9H20 CAS:921-47-1 MolWeight:128 RetIndex:724
CompName:Hexane, 2,3,4-trimethyl- $$ 2,3,4-Trimethylhexane $$

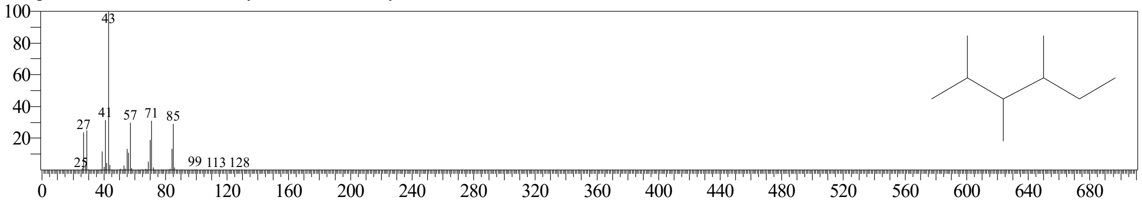

Hit#:3 Entry:4245 Library:NIST11.lib
SI:92 Formula:C8H18 CAS:619-99-8 MolWeight:114 RetIndex:752
CompName:Hexane, 3-ethyl- $$ 3-Ethylhexane $$

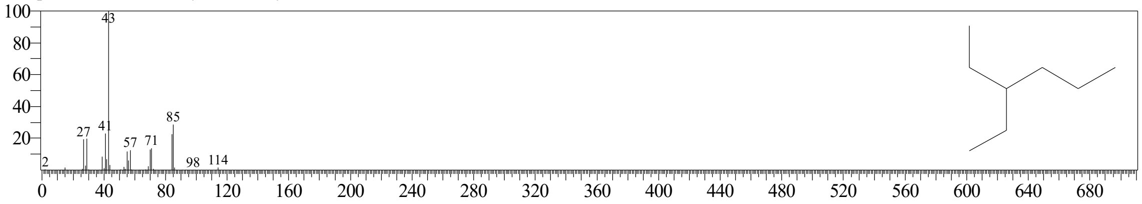

Hit#:4 Entry:7422 Library:NIST11.lib
SI:92 Formula:C9H20 CAS:1069-53-0 MolWeight:128 RetIndex:724
CompName:Hexane, 2,3,5-trimethyl- $$ 2,3,5-Trimethylhexane $$

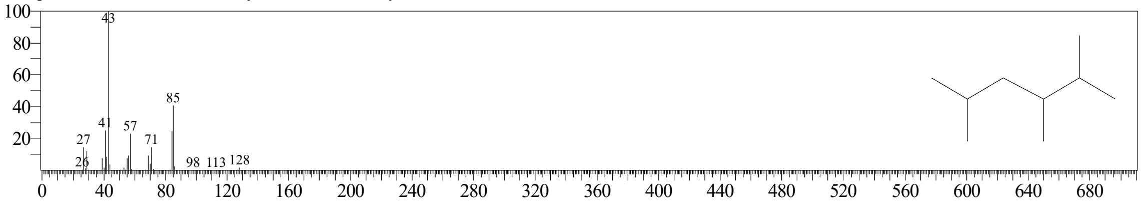

Hit#:5 Entry:7424 Library:NIST11.lib
SI:92 Formula:C9H20 CAS:2213-23-2 MolWeight:128 RetIndex:788
CompName:Heptane, 2,4-dimethyl- $$ 2,4-Dimethylheptane $$

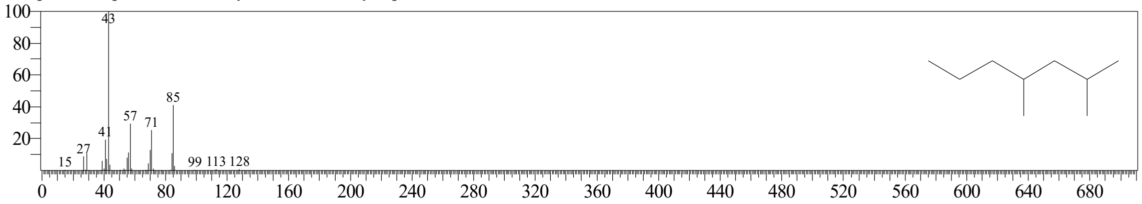

<< Target >>
Line#:3 R.Time:4.220(Scan#:45) MassPeaks:391
RawMode:Averaged 4.215-4.225(44-46) BasePeak:57.10(224421)
BG Mode:Calc. from Peak Group 1 - Event 1 Scan

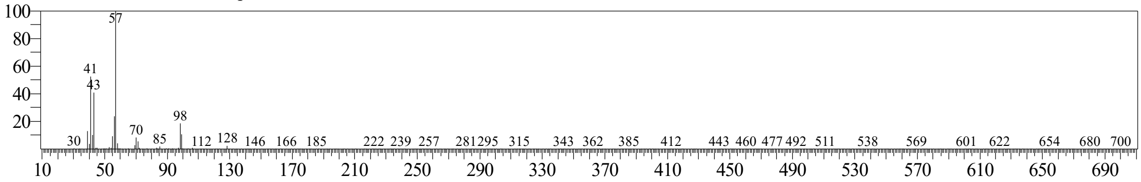

Hit#:1 Entry:12322 Library:NIST11.lib
SI:93 Formula:C10H22 CAS:52896-87-4 MolWeight:142 RetIndex:887
CompName:Heptane, 4-(1-methylethyl)- $$ 4-Isopropylheptane $$

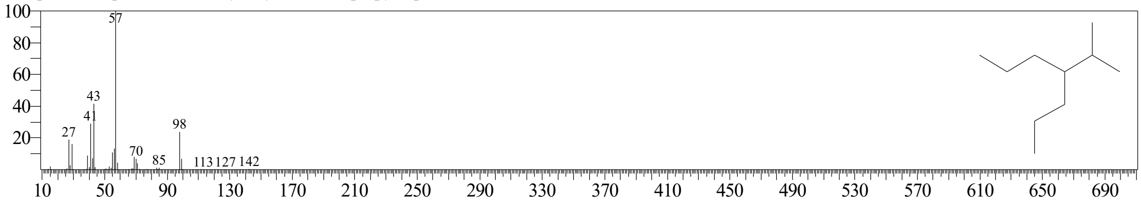

Hit#:2 Entry:7433 Library:NIST11.lib
SI:91 Formula:C9H20 CAS:2216-30-0 MolWeight:128 RetIndex:788
CompName:Heptane, 2,5-dimethyl- $$ 2,5-Dimethylheptane $$

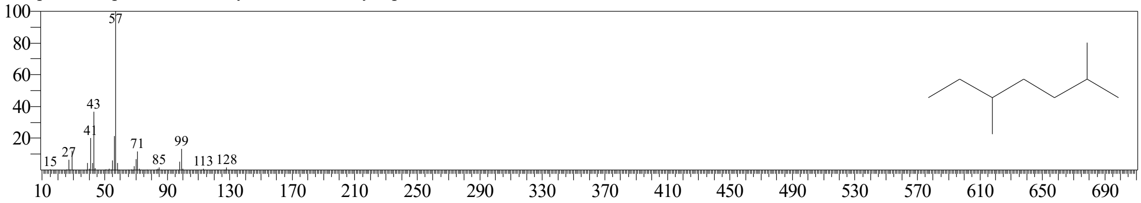

Hit#:3 Entry:29894 Library:NIST11.lib
SI:91 Formula:C7H15Br CAS:1974-05-6 MolWeight:178 RetIndex:949
CompName:Heptane, 3-bromo- $$ 3-Bromoheptane $$

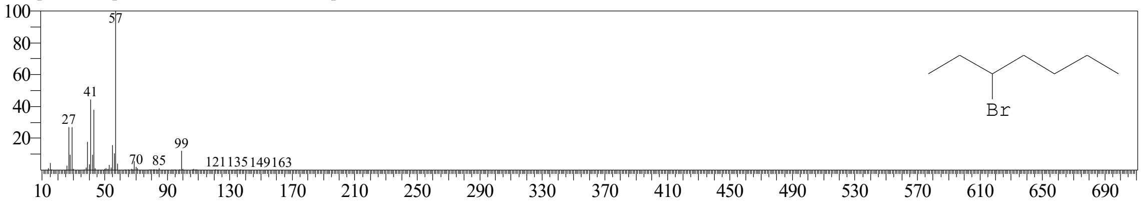

Hit#:4 Entry:7434 Library:NIST11.lib
SI:91 Formula:C9H20 CAS:2216-33-3 MolWeight:128 RetIndex:852
CompName:Octane, 3-methyl- $$ 3-Methyloctane $$ Isononane $$

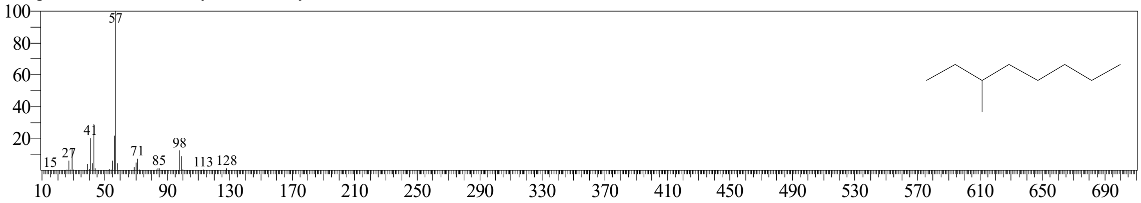

Hit#:5 Entry:7427 Library:NIST11.lib
SI:91 Formula:C9H20 CAS:926-82-9 MolWeight:128 RetIndex:788
CompName:Heptane, 3,5-dimethyl- $$ 3,5-Dimethylheptane $$

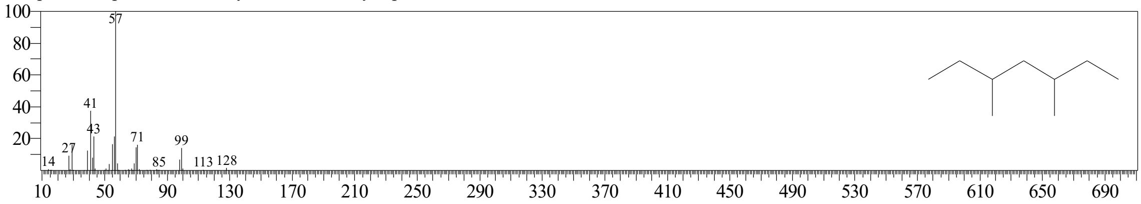

Qualitative Analysis Report

<< Target >>
Line#:4 R.Time:4.290(Scan#:59) MassPeaks:396
RawMode:Averaged 4.285-4.295(58-60) BasePeak:91.05(338374)
BG Mode:Calc. from Peak Group 1 - Event 1 Scan

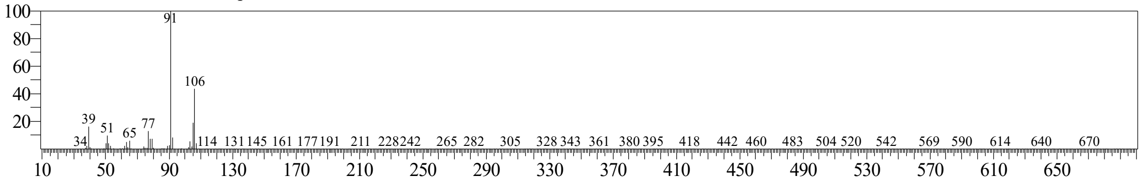

Hit#:1 Entry:2687 Library:NIST11.lib
SI:97 Formula:C8H10 CAS:95-47-6 MolWeight:106 RetIndex:907
CompName:o-Xylene $$ Benzene, 1,2-dimethyl- $$ o-Dimethylbenzene $$ o-Methyltoluene $$ o-Xylol $$ 1,2-Dimethylbenzene $$ 1,2-Xylene $$ 3,4-Xylene $

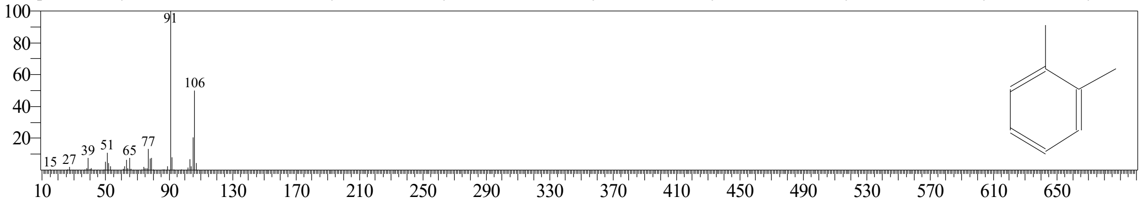

Hit#:2 Entry:2685 Library:NIST11.lib
SI:96 Formula:C8H10 CAS:108-38-3 MolWeight:106 RetIndex:907
CompName:Benzene, 1,3-dimethyl- $$ m-Xylene $$ m-Dimethylbenzene $$ m-Xylol $$ 1,3-Dimethylbenzene $$ 1,3-Xylene $$ 2,4-Xylene $$ m-Methyltoluene

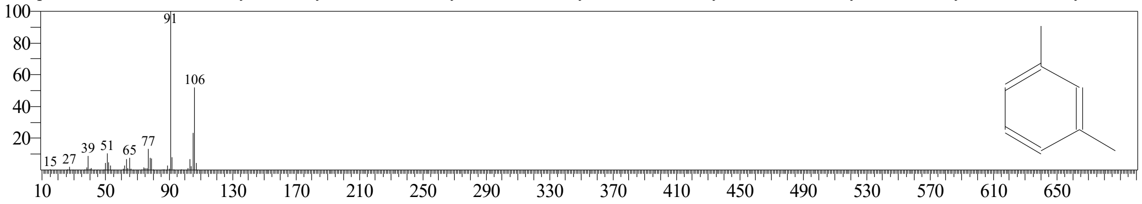

Hit#:3 Entry:2686 Library:NIST11.lib
SI:95 Formula:C8H10 CAS:106-42-3 MolWeight:106 RetIndex:907
CompName:p-Xylene $$ Benzene, 1,4-dimethyl- $$ p-Dimethylbenzene $$ p-Xylol $$ 1,4-Dimethylbenzene $$ 1,4-Xylene $$ p-Methyltoluene $$ para-Xylene

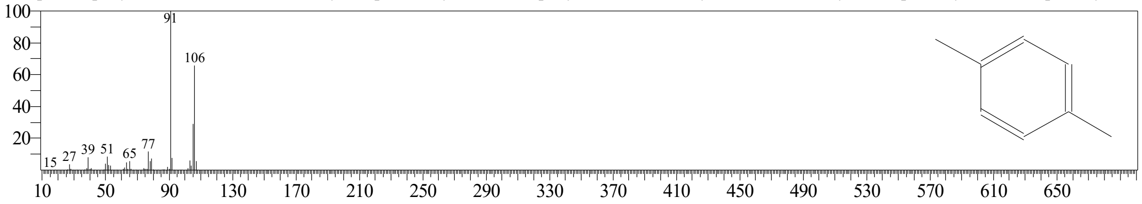

Hit#:4 Entry:2683 Library:NIST11.lib
SI:92 Formula:C8H10 CAS:100-41-4 MolWeight:106 RetIndex:893
CompName:Ethylbenzene $$ Benzene, ethyl- $$ Ethylbenzol $$ EB $$ Phenylethane $$ Aethylbenzol $$ Ethylbenzeen $$ Etilbenzene $$ Etylobenzen $$ NCI-C

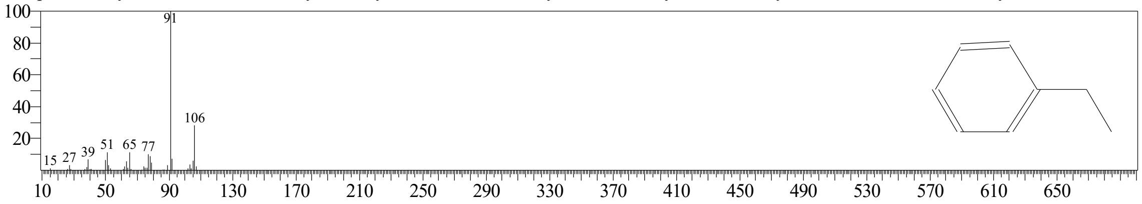

Hit#:5 Entry:2682 Library:NIST11.lib
SI:90 Formula:C8H10 CAS:2175-91-9 MolWeight:106 RetIndex:824
CompName:1,3-Cyclopentadiene, 5-(1-methylethylidene)- $$ 1,3-Cyclopentadiene, 5-isopropylidene- $$ Dimethylfulvene $$ Isopropylidenecyclopentadiene $$

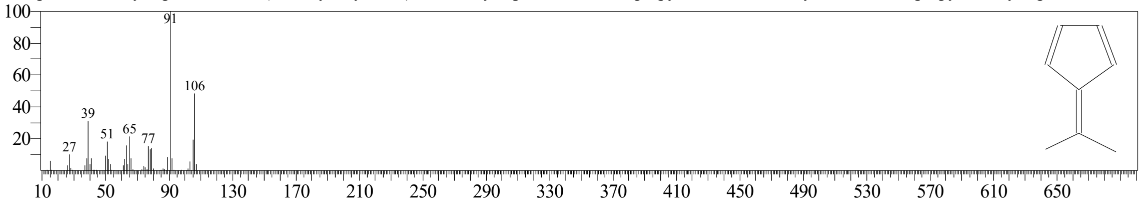

<< Target >>
Line#:5 R.Time:4.540(Scan#:109) MassPeaks:392
RawMode:Averaged 4.535-4.545(108-110) BasePeak:43.05(714198)
BG Mode:Calc. from Peak Group 1 - Event 1 Scan

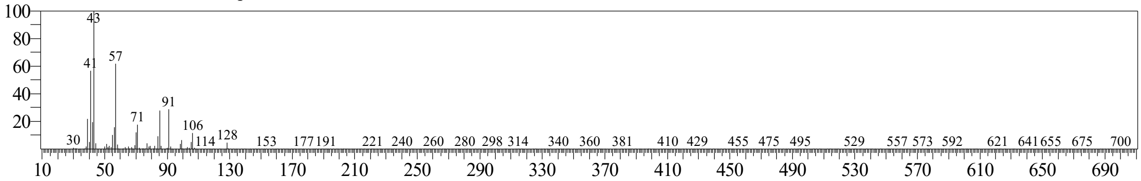

Hit#:1 Entry:4236 Library:NIST11.lib
SI:87 Formula:C8H18 CAS:589-43-5 MolWeight:114 RetIndex:688
CompName:Hexane, 2,4-dimethyl- $$ 2,4-Dimethylhexane $$

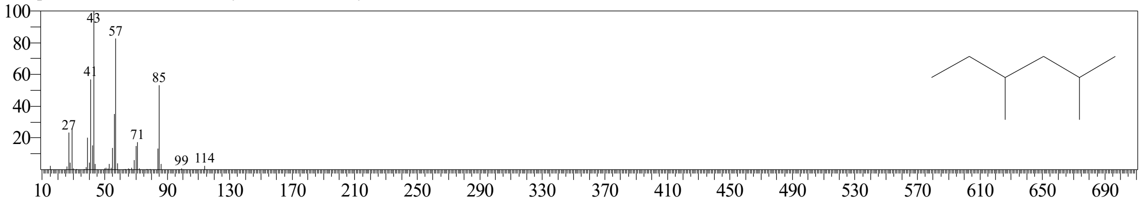

Hit#:2 Entry:7416 Library:NIST11.lib
SI:86 Formula:C9H20 CAS:111-84-2 MolWeight:128 RetIndex:916
CompName:Nonane $$ n-Nonane $$ Shellsol 140 $$ n-C9H20 $$ UN 1920 $$

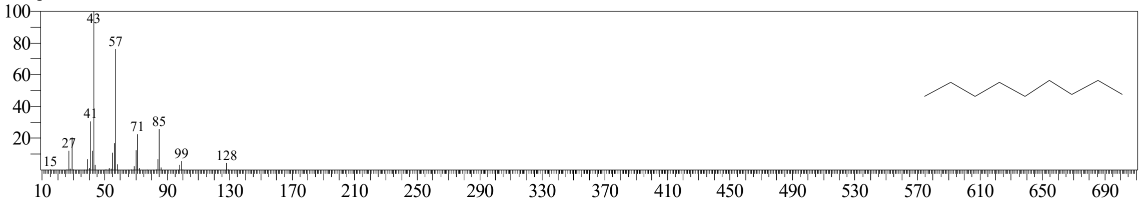

Hit#:3 Entry:12306 Library:NIST11.lib
SI:86 Formula:C10H22 CAS:871-83-0 MolWeight:142 RetIndex:951
CompName:Nonane, 2-methyl- $$ 2-Methylnonane $$

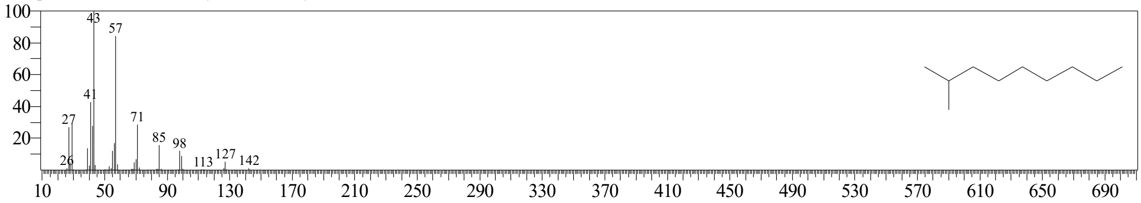

Hit#:4 Entry:12325 Library:NIST11.lib
SI:85 Formula:C10H22 CAS:13475-81-5 MolWeight:142 RetIndex:846
CompName:Hexane, 2,2,3,3-tetramethyl- $$ 2,2,3,3-Tetramethylhexane $$

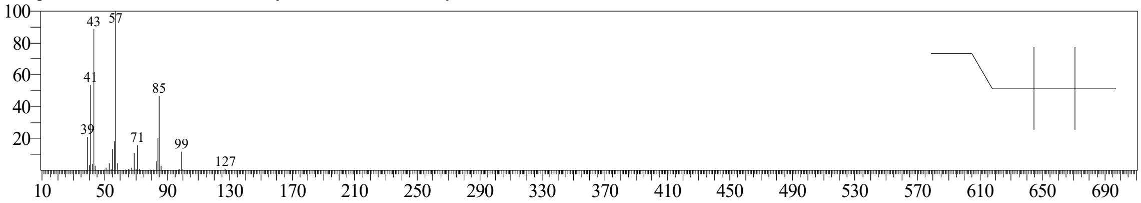

Hit#:5 Entry:92779 Library:NIST11.lib
SI:85 Formula:C10H21I CAS:0-00-0 MolWeight:268 RetIndex:1365
CompName:1-Iodo-2-methylnonane

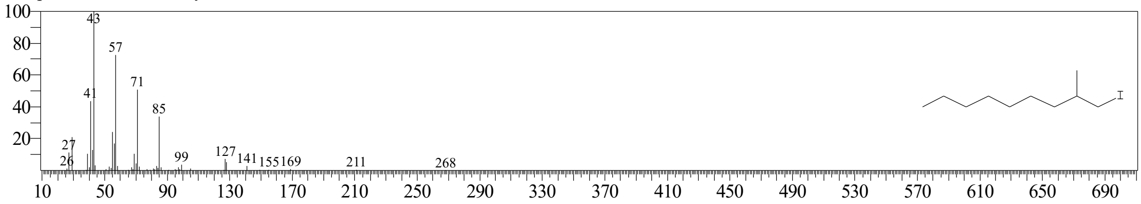

<< Target >>
Line#:6 R.Time:4.725(Scan#:146) MassPeaks:364
RawMode:Averaged 4.720-4.730(145-147) BasePeak:43.05(1428448)
BG Mode:Calc. from Peak Group 1 - Event 1 Scan

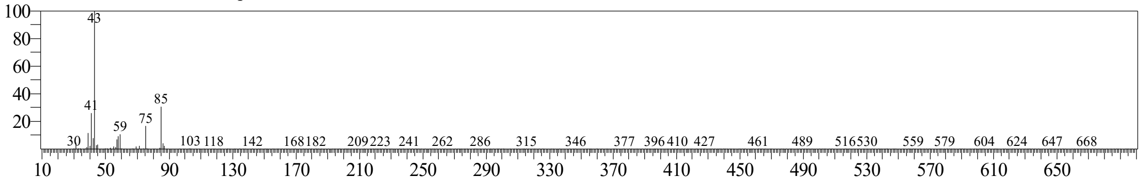

Hit#:1 Entry:7299 Library:NIST11.lib
SI:88 Formula:C8H16O CAS:26118-38-7 MolWeight:128 RetIndex:868
CompName:2-Hexanone, 3,3-dimethyl- $$ 3,3-Dimethyl-2-hexanone # $$

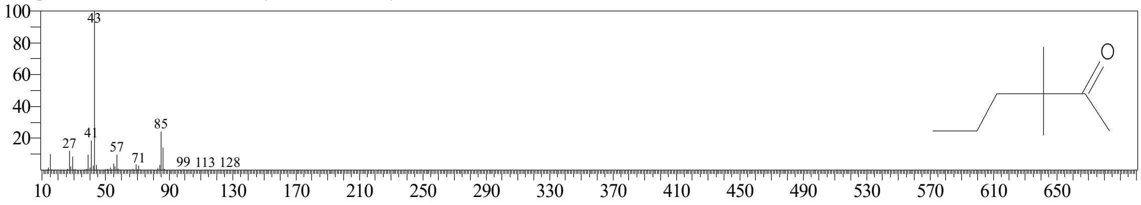

Hit#:2 Entry:8164 Library:NIST11.lib
SI:87 Formula:C6H13NO2 CAS:14255-44-8 MolWeight:131 RetIndex:935
CompName:Hexane, 2-nitro- $$ 2-Nitrohexane # $$

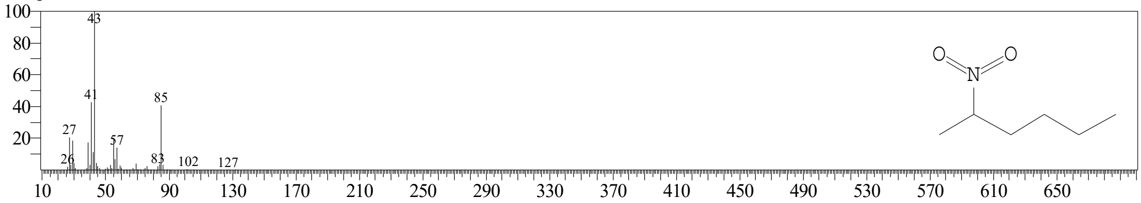

Hit#:3 Entry:4540 Library:NIST11.lib
SI:86 Formula:C6H12O2 CAS:25910-96-7 MolWeight:116 RetIndex:815
CompName:3-Oxetanol, 2,2,3-trimethyl- $$ 2,2,3-Trimethyl-3-oxetanol # $$

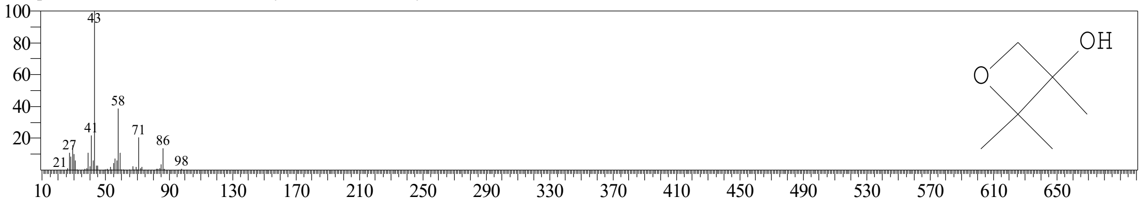

Hit#:4 Entry:51590 Library:NIST11.lib
SI:86 Formula:C5H9IO CAS:3695-29-2 MolWeight:212 RetIndex:1069
CompName:5-Iodopentan-2-one $$ 5-Iodo-2-pentanone # $$

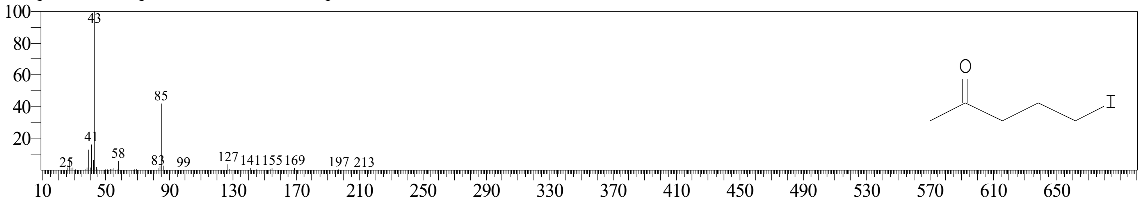

Hit#:5 Entry:4129 Library:NIST11.lib
SI:86 Formula:C7H14O CAS:34061-75-1 MolWeight:114 RetIndex:783
CompName:Ether, 3-butenyl propyl $$ 3-Butenyl propyl ether # $$

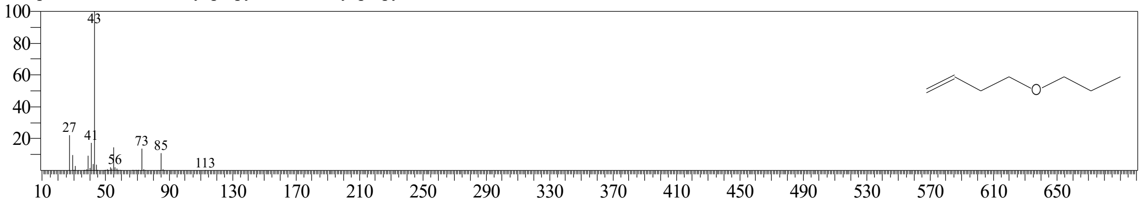

<< Target >>
Line#:7 R.Time:4.925(Scan#:186) MassPeaks:397
RawMode:Averaged 4.920-4.930(185-187) BasePeak:43.05(1312475)
BG Mode:Calc. from Peak Group 1 - Event 1 Scan

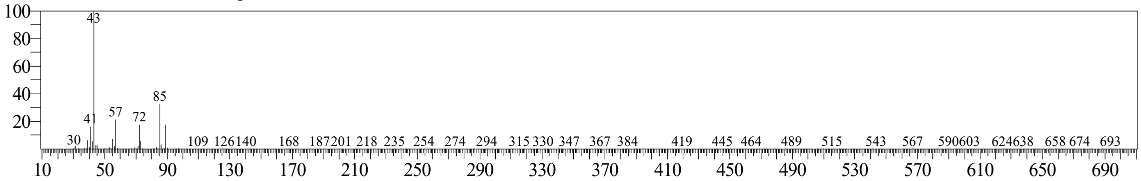

Hit#:1 Entry:3988 Library:NIST11.lib
SI:88 Formula:C6H10O2 CAS:17257-81-7 MolWeight:114 RetIndex:806
CompName:Ethanone, 1-(3-ethyloxiranyl)- $$ 2-Hexanone, 3,4-epoxy- $$ 1-(3-Ethyl-2-oxiranyl)ethanone # $$

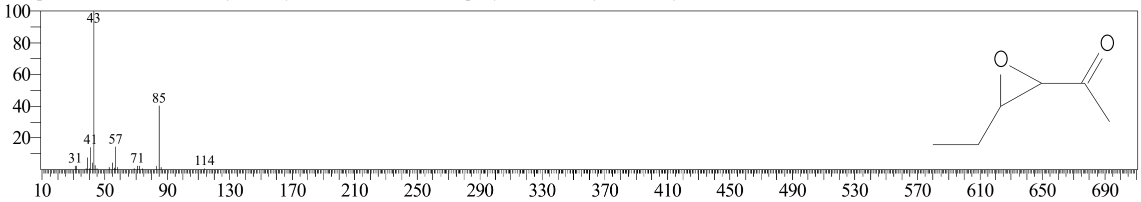

Hit#:2 Entry:7295 Library:NIST11.lib
SI:87 Formula:C8H16O CAS:2371-19-9 MolWeight:128 RetIndex:888
CompName:2-Heptanone, 3-methyl- $$ 3-Methyl-2-heptanone $$ 3-Methylheptan-2-one $$

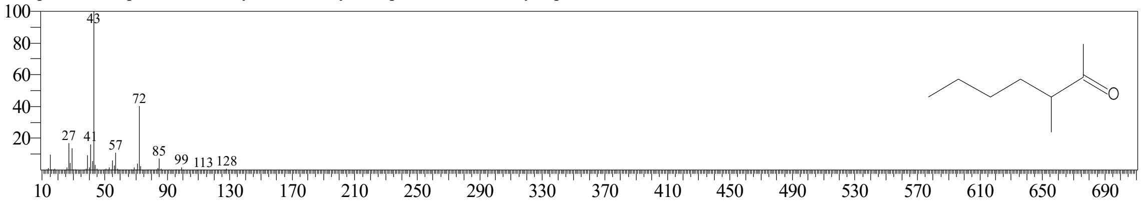

Hit#:3 Entry:7299 Library:NIST11.lib
SI:86 Formula:C8H16O CAS:26118-38-7 MolWeight:128 RetIndex:868
CompName:2-Hexanone, 3,3-dimethyl- $$ 3,3-Dimethyl-2-hexanone # $$

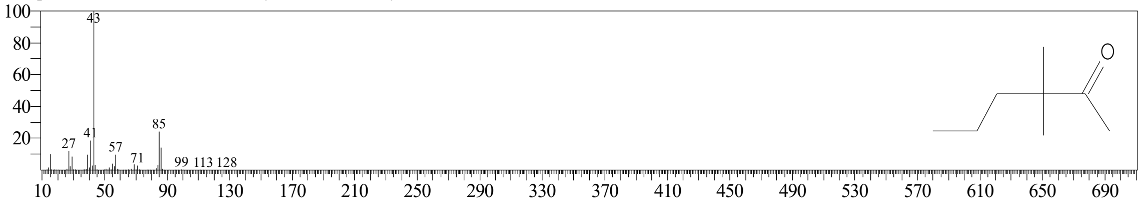

Hit#:4 Entry:7296 Library:NIST11.lib
SI:86 Formula:C8H16O CAS:19550-10-8 MolWeight:128 RetIndex:824
CompName:2-Hexanone, 3,4-dimethyl- $$ 3,4-Dimethyl-2-hexanone $$

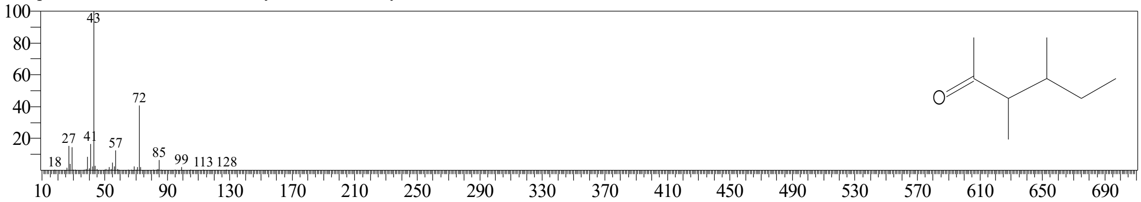

Hit#:5 Entry:18593 Library:NIST11.lib
SI:86 Formula:C10H20O CAS:107995-55-1 MolWeight:156 RetIndex:1081
CompName:Hexane, 1-(3-butenyloxy)- $$ 3-Butenyl hexyl ether # $$

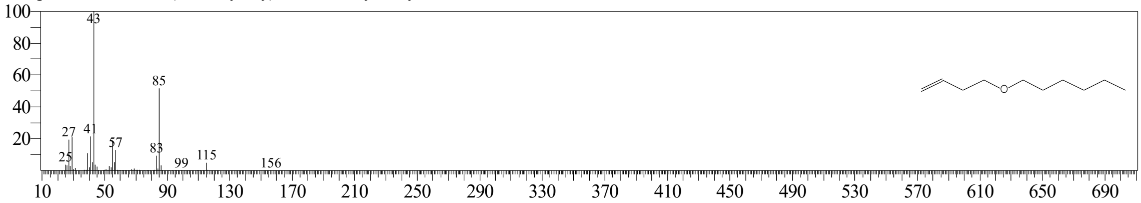

<< Target >>
Line#:8 R.Time:5.025(Scan#:206) MassPeaks:349
RawMode:Averaged 5.020-5.030(205-207) BasePeak:43.05(13971)
BG Mode:Calc. from Peak Group 1 - Event 1 Scan

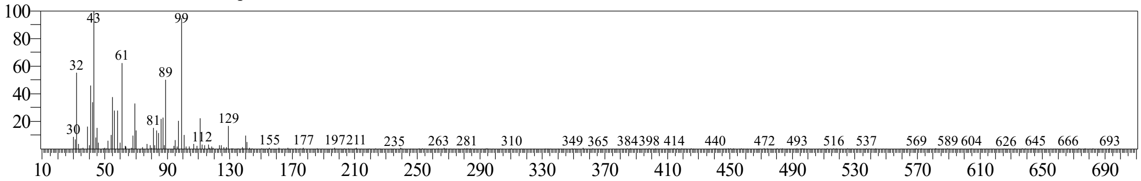

Hit#:1 Entry:63822 Library:NIST11.lib
SI:70 Formula:C14H28O2 CAS:0-00-0 MolWeight:228 RetIndex:1516
CompName:Hexanoic acid, 4-octyl ester $$ 1-Propylpentyl hexanoate # $$

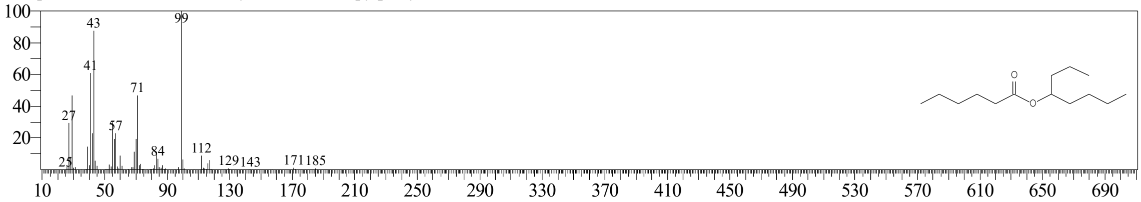

Hit#:2 Entry:82670 Library:NIST11.lib
SI:70 Formula:C16H30O2 CAS:180252-09-9 MolWeight:254 RetIndex:1769
CompName:Hexanoic acid, 9-decen-1-yl ester $$ 9-Decenyl hexanoate # $$

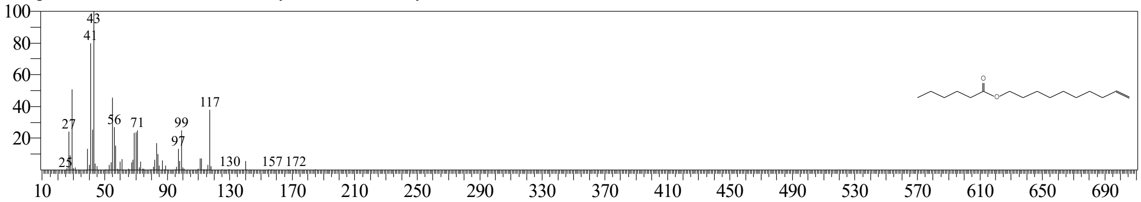

Hit#:3 Entry:128189 Library:NIST11.lib
SI:68 Formula:C20H40O2 CAS:55193-21-0 MolWeight:312 RetIndex:2112
CompName:Hexanoic acid, 2-tetradecyl ester $$ 1-Methyltridecyl hexanoate # $$

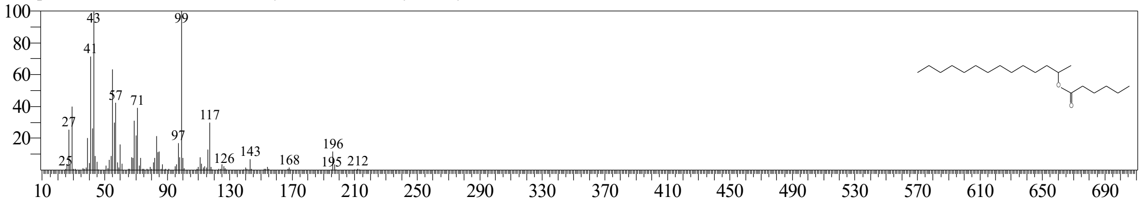

Hit#:4 Entry:43973 Library:NIST11.lib
SI:68 Formula:C12H24O2 CAS:112-17-4 MolWeight:200 RetIndex:1381
CompName:Acetic acid, decyl ester $$ n-Decyl acetate $$ Acetate C 10 $$ Decyl acetate $$ 1-Decanol acetate $$ n-Decyl ethanoate $$ Decyl ester of acetic acid

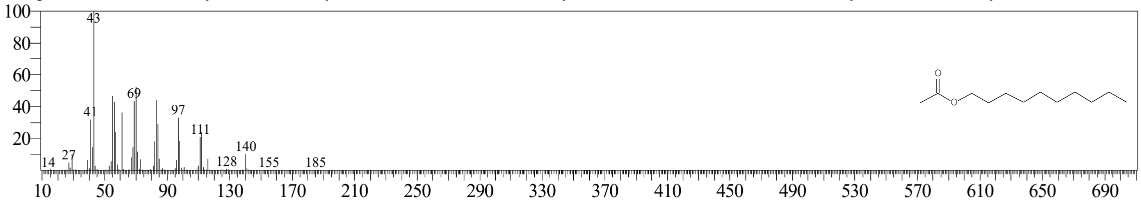

Hit#:5 Entry:35144 Library:NIST11.lib
SI:67 Formula:C11H22O2 CAS:143-13-5 MolWeight:186 RetIndex:1282
CompName:Acetic acid, nonyl ester $$ n-Nonanyl acetate $$ n-Nonyl acetate $$ n-Nonyl ethanoate $$ Acetic acid n-nonyl ester $$ Nonanol acetate $$ Nonyl ac

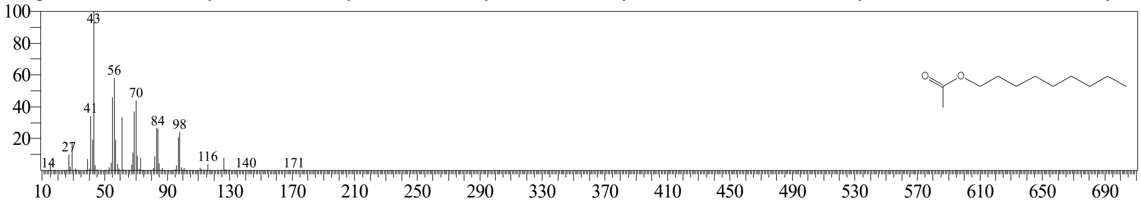

<< Target >>
Line#:9 R.Time:5.140(Scan#:229) MassPeaks:331
RawMode:Averaged 5.135-5.145(228-230) BasePeak:57.10(20532)
BG Mode:Calc. from Peak Group 1 - Event 1 Scan

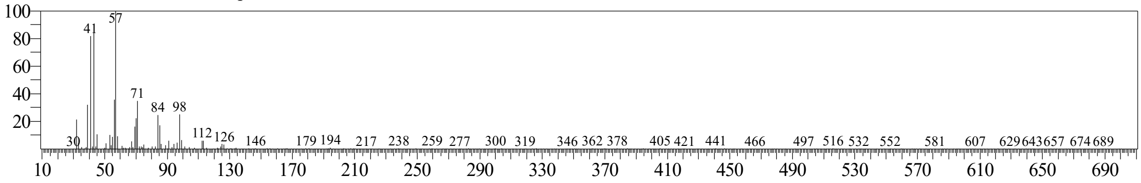

Hit#:1 Entry:12320 Library:NIST11.lib
SI:84 Formula:C10H22 CAS:124-18-5 MolWeight:142 RetIndex:1015
CompName:Decane $$ n-Decane $$ n-C10H22 $$ UN 2247 $$

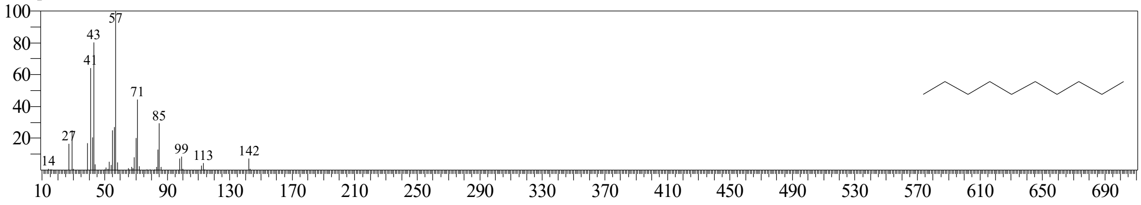

Hit#:2 Entry:42959 Library:NIST11.lib
SI:82 Formula:C14H30 CAS:192823-15-7 MolWeight:198 RetIndex:1156
CompName:Decane, 2,3,5,8-tetramethyl- $$ 2,3,5,8-Tetramethyldecane # $$

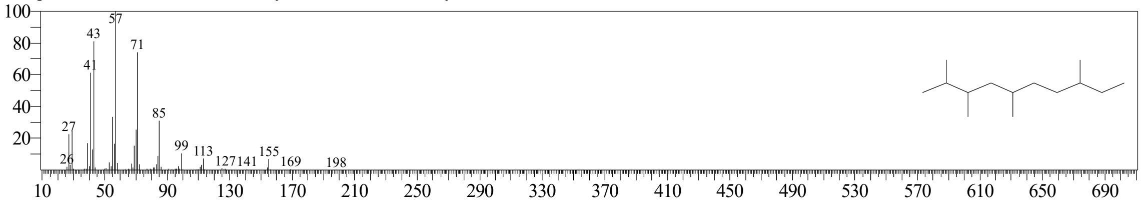

Hit#:3 Entry:12323 Library:NIST11.lib
SI:82 Formula:C10H22 CAS:15869-86-0 MolWeight:142 RetIndex:951
CompName:Octane, 4-ethyl- $$ 4-Ethyloctane $$

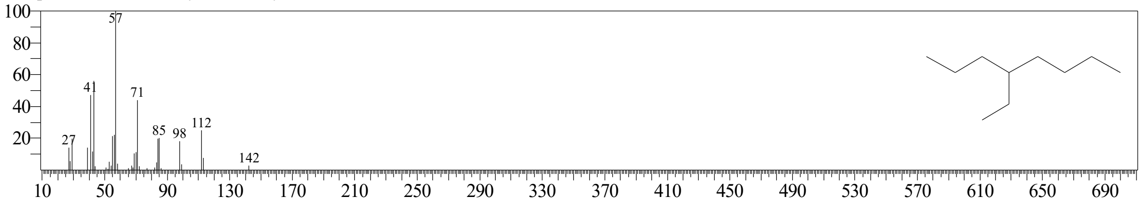

Hit#:4 Entry:18707 Library:NIST11.lib
SI:82 Formula:C11H24 CAS:62016-30-2 MolWeight:156 RetIndex:966
CompName:Octane, 2,3,3-trimethyl- $$ 2,3,3-Trimethyloctane # $$

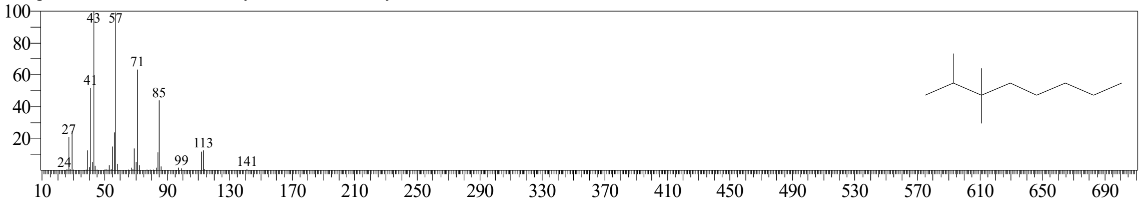

Hit#:5 Entry:12325 Library:NIST11.lib
SI:82 Formula:C10H22 CAS:13475-81-5 MolWeight:142 RetIndex:846
CompName:Hexane, 2,2,3,3-tetramethyl- $$ 2,2,3,3-Tetramethylhexane $$

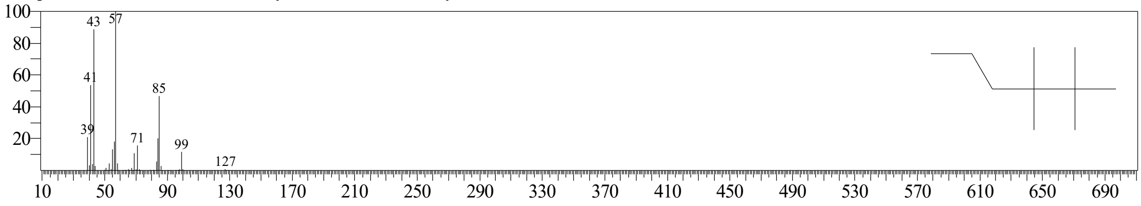

<< Target >>
Line#:10 R.Time:5.220(Scan#:245) MassPeaks:397
RawMode:Averaged 5.215-5.225(244-246) BasePeak:43.10(86383)
BG Mode:Calc. from Peak Group 1 - Event 1 Scan

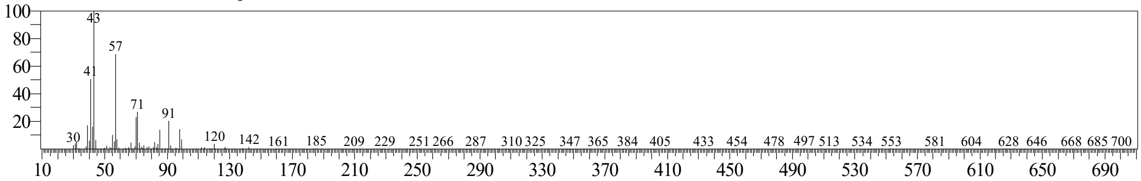

Hit#:1 Entry:12306 Library:NIST11.lib
SI:88 Formula:C10H22 CAS:871-83-0 MolWeight:142 RetIndex:951
CompName:Nonane, 2-methyl- $$ 2-Methylnonane $$

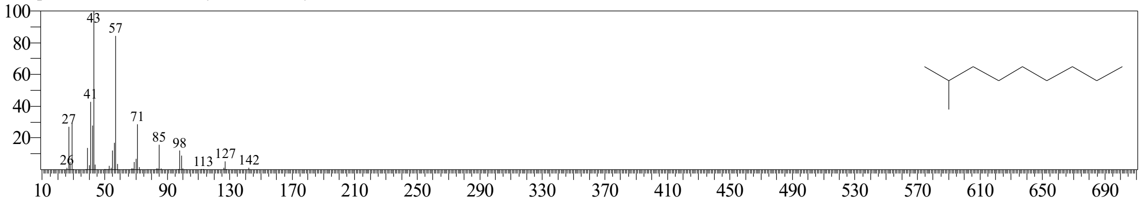

Hit#:2 Entry:26098 Library:NIST11.lib
SI:85 Formula:C12H26 CAS:52670-34-5 MolWeight:170 RetIndex:958
CompName:Octane, 2,3,6,7-tetramethyl- $$ 2,3,6,7-Tetramethyloctane # $$

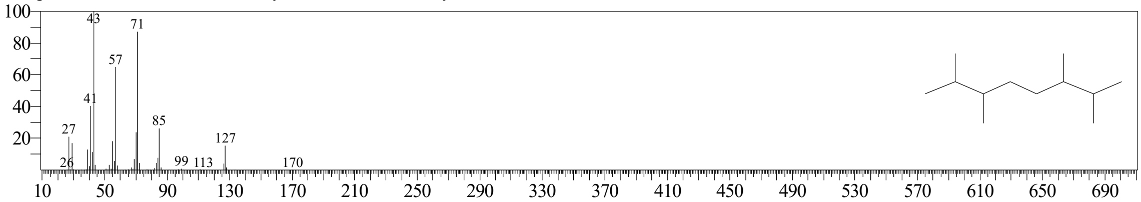

Hit#:3 Entry:4236 Library:NIST11.lib
SI:84 Formula:C8H18 CAS:589-43-5 MolWeight:114 RetIndex:688
CompName:Hexane, 2,4-dimethyl- $$ 2,4-Dimethylhexane $$

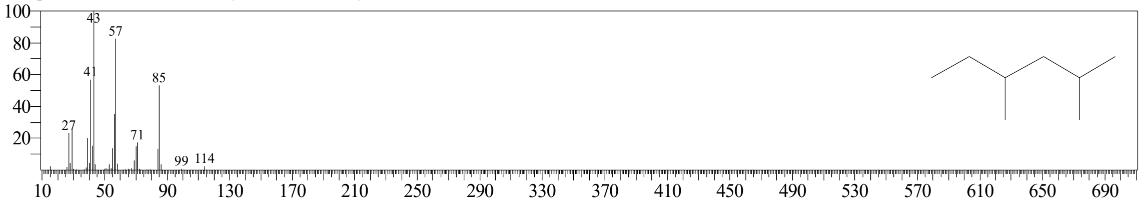

Hit#:4 Entry:18711 Library:NIST11.lib
SI:84 Formula:C11H24 CAS:2847-72-5 MolWeight:156 RetIndex:1051
CompName:Decane, 4-methyl- $$ 4-Methyldecane $$

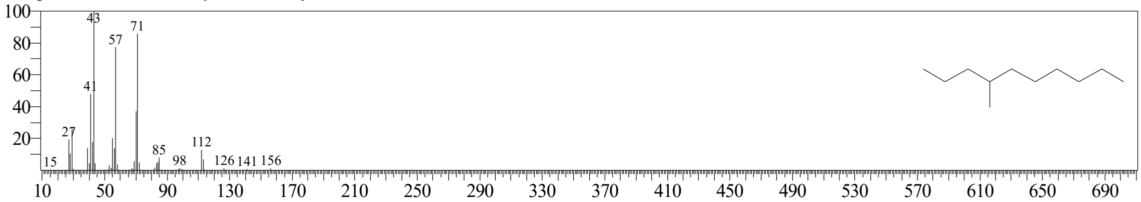

Hit#:5 Entry:93609 Library:NIST11.lib
SI:84 Formula:C19H40 CAS:10544-96-4 MolWeight:268 RetIndex:1846
CompName:Octadecane, 6-methyl- $$ 6-Methyloctadecane # $$

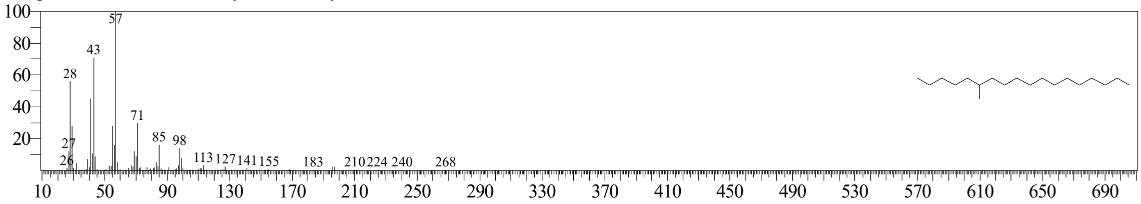

<< Target >>
Line#:11 R.Time:5.255(Scan#:252) MassPeaks:353
RawMode:Averaged 5.250-5.260(251-253) BasePeak:43.05(68279)
BG Mode:Calc. from Peak Group 1 - Event 1 Scan

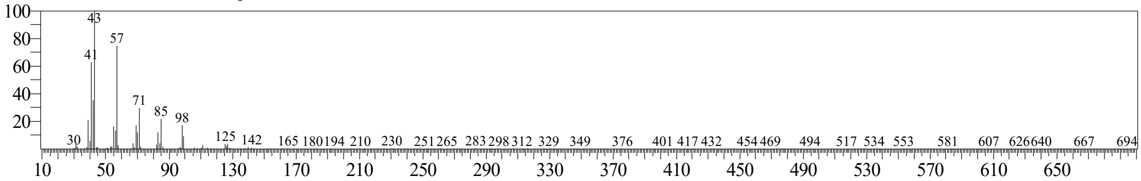

Hit#:1 Entry:12306 Library:NIST11.lib
SI:91 Formula:C10H22 CAS:871-83-0 MolWeight:142 RetIndex:951
CompName:Nonane, 2-methyl- $$ 2-Methylnonane $$

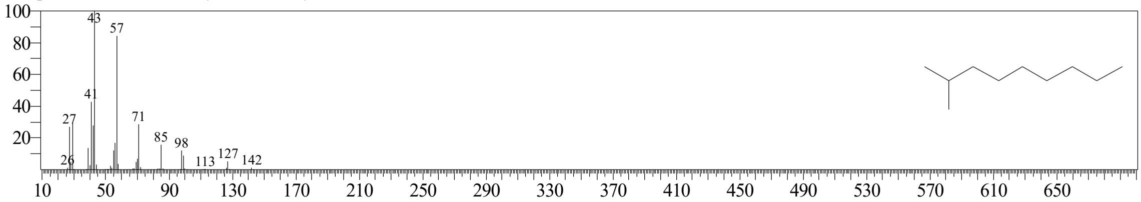

Hit#:2 Entry:24835 Library:NIST11.lib
SI:90 Formula:C12H24 CAS:54410-98-9 MolWeight:168 RetIndex:1012
CompName:1-Nonene, 4,6,8-trimethyl- $$ 4,6,8-Trimethyl-1-nonene # $$

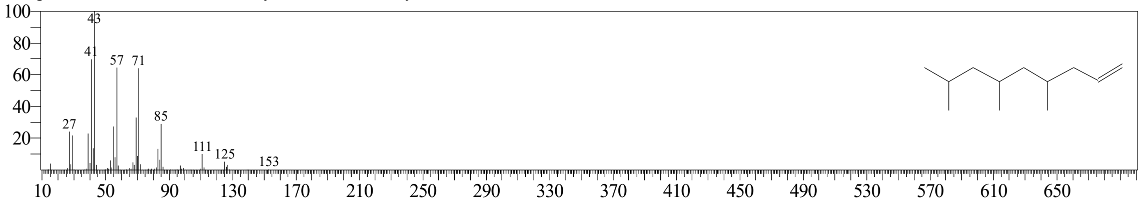

Hit#:3 Entry:26094 Library:NIST11.lib
SI:88 Formula:C12H26 CAS:1002-17-1 MolWeight:170 RetIndex:1086
CompName:Decane, 2,9-dimethyl- $$ 2,9-Dimethyldecane $$

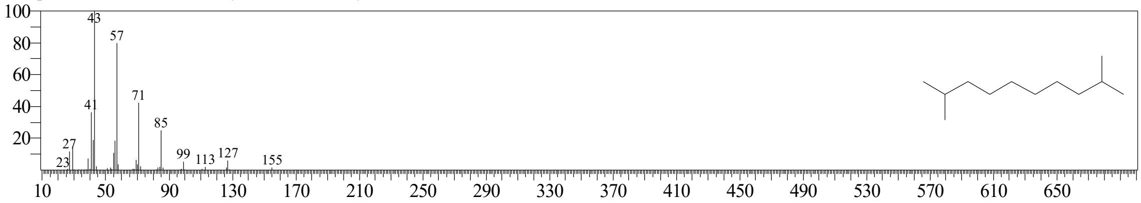

Hit#:4 Entry:96490 Library:NIST11.lib
SI:88 Formula:C15H28O4 CAS:0-00-0 MolWeight:272 RetIndex:1847
CompName:Oxalic acid, decyl propyl ester

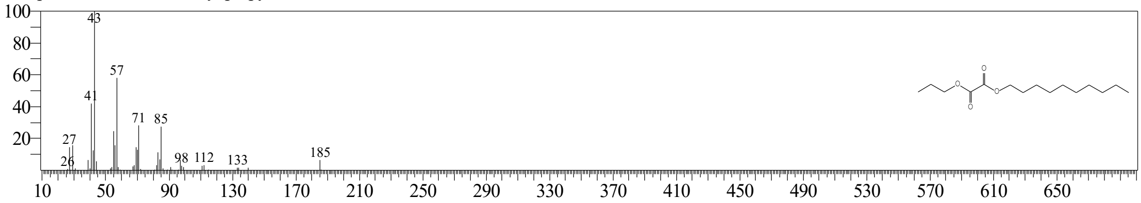

Hit#:5 Entry:62478 Library:NIST11.lib
SI:88 Formula:C16H34 CAS:1560-93-6 MolWeight:226 RetIndex:1548
CompName:Pentadecane, 2-methyl- $$ 2-Methylpentadecane $$

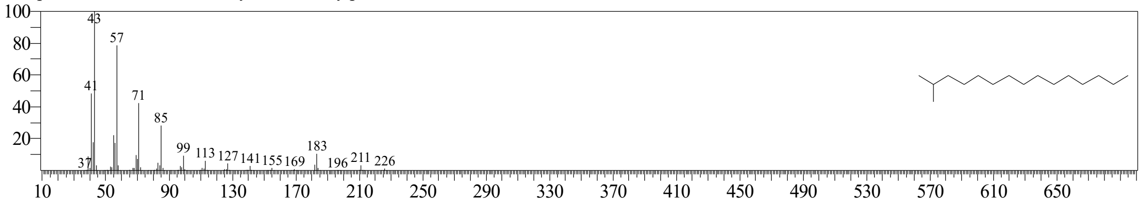

<< Target >>
Line#:12 R.Time:5.330(Scan#:267) MassPeaks:320
RawMode:Averaged 5.325-5.335(266-268) BasePeak:57.05(48340)
BG Mode:Calc. from Peak Group 1 - Event 1 Scan

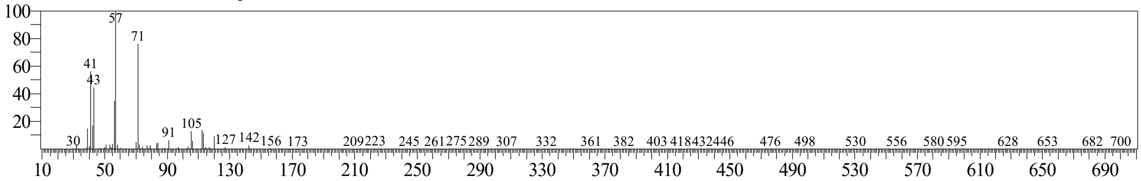

Hit#:1 Entry:12338 Library:NIST11.lib
SI:86 Formula:C10H22 CAS:5911-04-6 MolWeight:142 RetIndex:951
CompName:Nonane, 3-methyl- $$ 3-Methylnonane $$ 3-Methylnonane, (DL)- $$

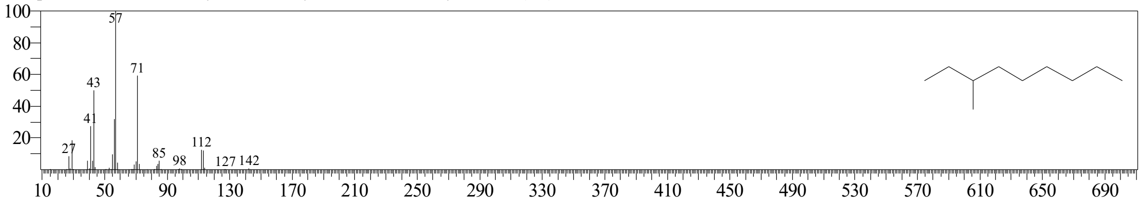

Hit#:2 Entry:12326 Library:NIST11.lib
SI:85 Formula:C10H22 CAS:52896-90-9 MolWeight:142 RetIndex:887
CompName:Heptane, 3-ethyl-5-methyl- $$ 3-Ethyl-5-methylheptane $$ Heptane, 5-ethyl-3-methyl $$

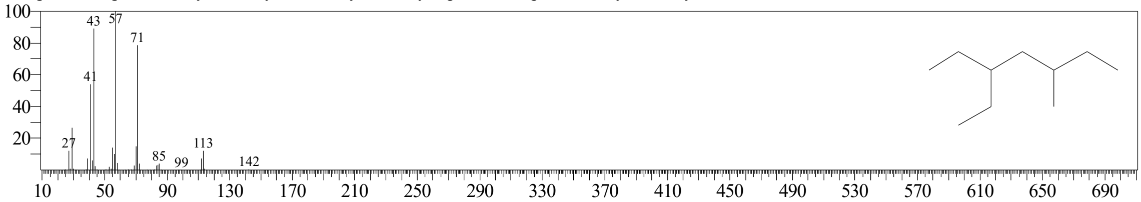

Hit#:3 Entry:12310 Library:NIST11.lib
SI:84 Formula:C10H22 CAS:13475-78-0 MolWeight:142 RetIndex:887
CompName:Heptane, 5-ethyl-2-methyl- $$ 5-Ethyl-2-methylheptane $$ Heptane, 3-ethyl-6-methyl $$

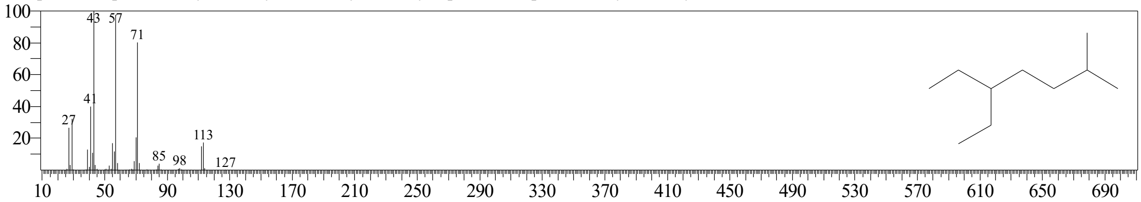

Hit#:4 Entry:12339 Library:NIST11.lib
SI:84 Formula:C10H22 CAS:15869-94-0 MolWeight:142 RetIndex:887
CompName:Octane, 3,6-dimethyl- $$ 3,6-Dimethyloctane $$

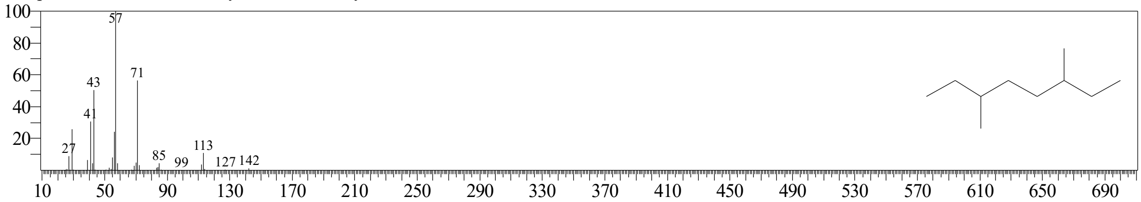

Hit#:5 Entry:38188 Library:NIST11.lib
SI:84 Formula:C8H17Br CAS:999-64-4 MolWeight:192 RetIndex:1049
CompName:3-Bromooctane $$ Octane, 3-bromo- $$

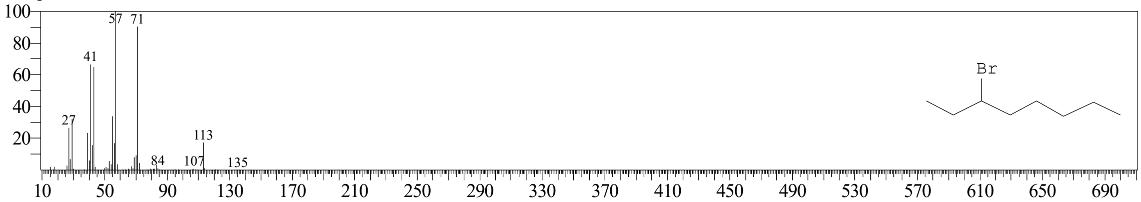

Qualitative Analysis Report

<< Target >>
Line#:13 R.Time:5.440(Scan#:289) MassPeaks:372
RawMode:Averaged 5.435-5.445(288-290) BasePeak:43.05(874743)
BG Mode:Calc. from Peak Group 1 - Event 1 Scan

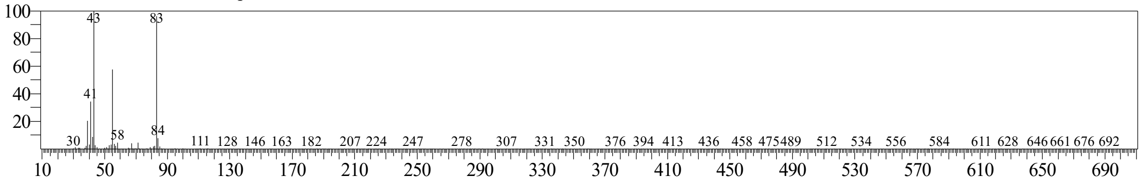

Hit#:1 Entry:6711 Library:NIST11.lib
SI:87 Formula:C9H18 CAS:55702-61-9 MolWeight:126 RetIndex:775
CompName:2-Hexene, 4,4,5-trimethyl- $$ (2E)-4,4,5-Trimethyl-2-hexene # $$

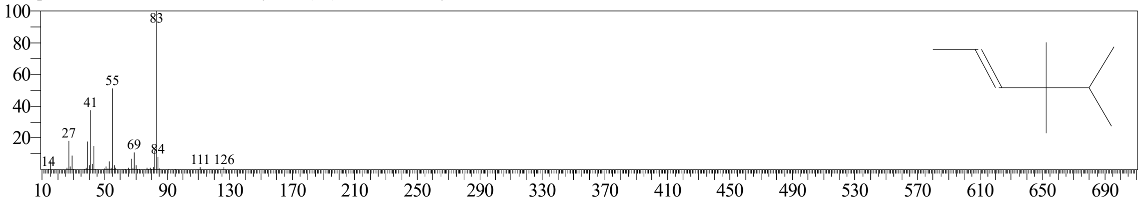

Hit#:2 Entry:6418 Library:NIST11.lib
SI:87 Formula:C7H10O2 CAS:15121-02-5 MolWeight:126 RetIndex:920
CompName:Cyclopentane, 1-acetyl-1,2-epoxy- $$ 1-(6-Oxabicyclo[3.1.0]hex-1-yl)ethanone # $$

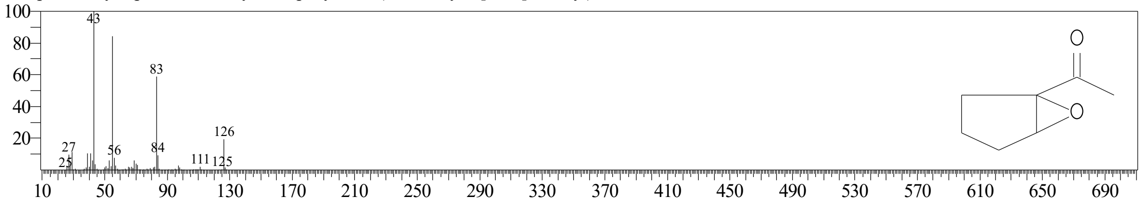

Hit#:3 Entry:63462 Library:NIST11.lib
SI:87 Formula:C12H20O4 CAS:0-00-0 MolWeight:228 RetIndex:1548
CompName:Oxalic acid, cyclohexyl isobutyl ester

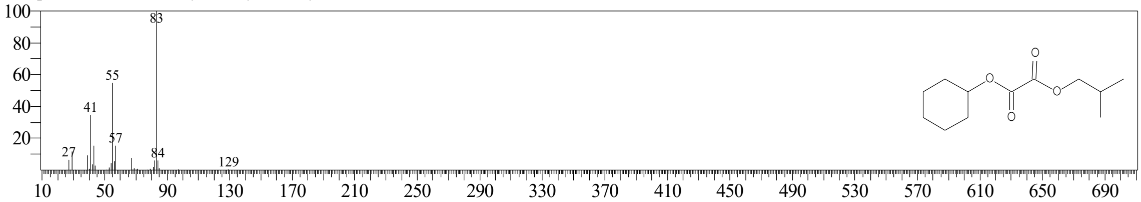

Hit#:4 Entry:1702 Library:NIST11.lib
SI:86 Formula:C6H10O CAS:763-93-9 MolWeight:98 RetIndex:762
CompName:3-Hexen-2-one $$ 1-Butenyl methyl ketone $$ Methyl 1-butenyl ketone $$ Hex-3-en-2-one $$

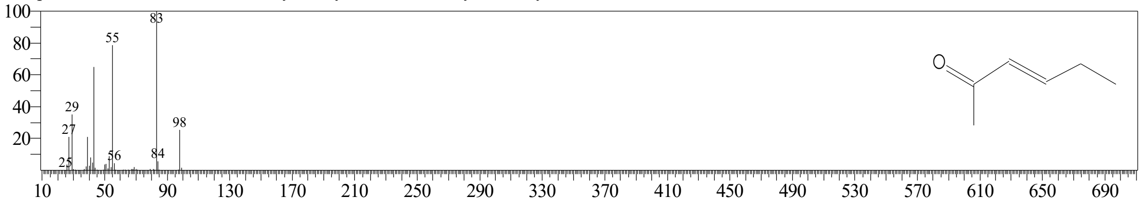

Hit#:5 Entry:53303 Library:NIST11.lib
SI:86 Formula:C11H18O4 CAS:0-00-0 MolWeight:214 RetIndex:1513
CompName:Oxalic acid, cyclohexyl propyl ester

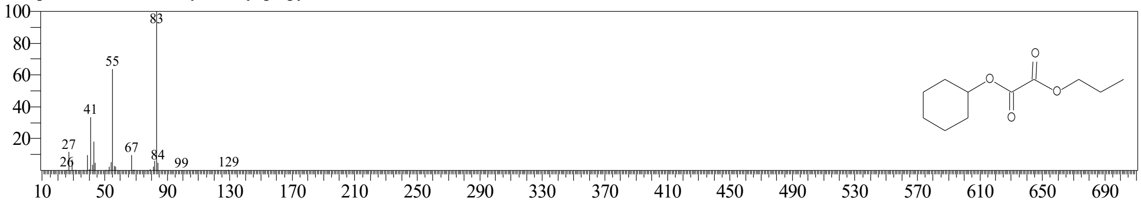

<< Target >>
Line#:14 R.Time:5.560(Scan#:313) MassPeaks:411
RawMode:Averaged 5.555-5.565(312-314) BasePeak:41.05(7954796)
BG Mode:Calc. from Peak Group 1 - Event 1 Scan

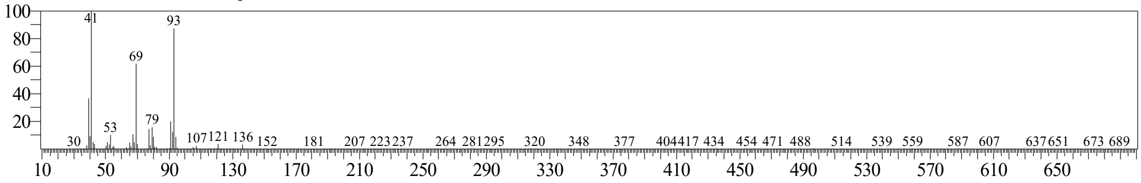

Hit#:1 Entry:9719 Library:NIST11.lib
SI:95 Formula:C10H16 CAS:123-35-3 MolWeight:136 RetIndex:958
CompName:.beta.-Myrcene $$ 1,6-Octadiene, 7-methyl-3-methylene- $$ Myrcene $$ 7-Methyl-3-methylene-1,6-octadiene $$ 7-Methyl-3-methyleneoctadiene-(1

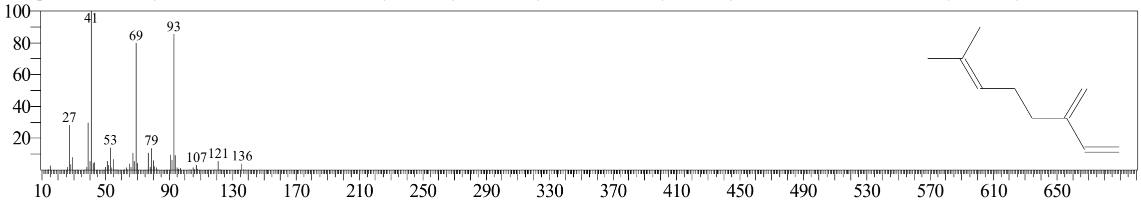

Hit#:2 Entry:9777 Library:NIST11.lib
SI:90 Formula:C10H16 CAS:127-91-3 MolWeight:136 RetIndex:943
CompName:.beta.-Pinene $$ Bicyclo[3.1.1]heptane, 6,6-dimethyl-2-methylene- $$ 2(10)-Pinene $$ Nopinen $$ Nopinene $$ Pseudopinen $$ Pseudopinene $$

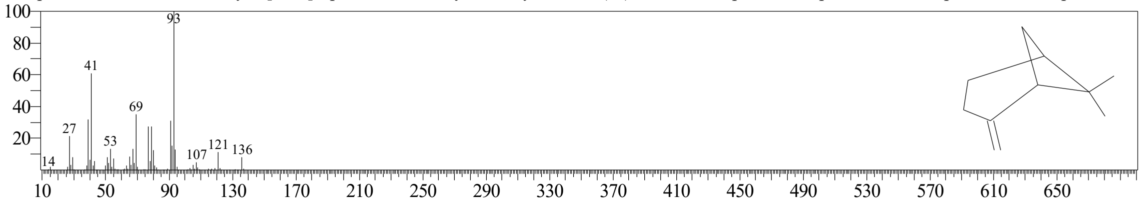

Hit#:3 Entry:9776 Library:NIST11.lib
SI:88 Formula:C10H16 CAS:18172-67-3 MolWeight:136 RetIndex:943
CompName:Bicyclo[3.1.1]heptane, 6,6-dimethyl-2-methylene-, (1S)- $$ 2(10)-Pinene, (1S,5S)-(-)- $$ (-)-.beta.-Pinene $$ (-)-2(10)-Pinene $$ L-.beta.-Pinene $$

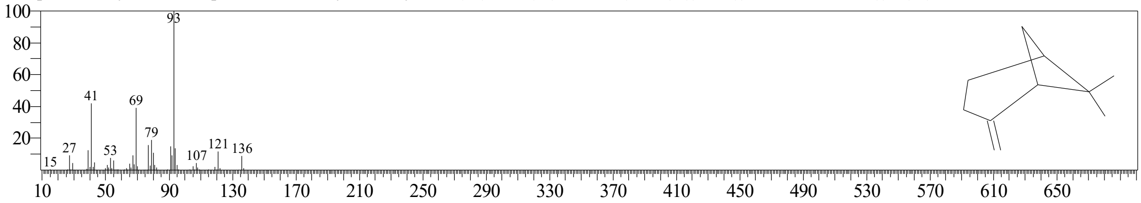

Hit#:4 Entry:54444 Library:NIST11.lib
SI:87 Formula:C10H17Br CAS:35719-26-7 MolWeight:216 RetIndex:1282
CompName:1-Bromo-3,7-dimethyl-2,6-octadiene $$ 2,6-Octadiene, 1-bromo-3,7-dimethyl- $$

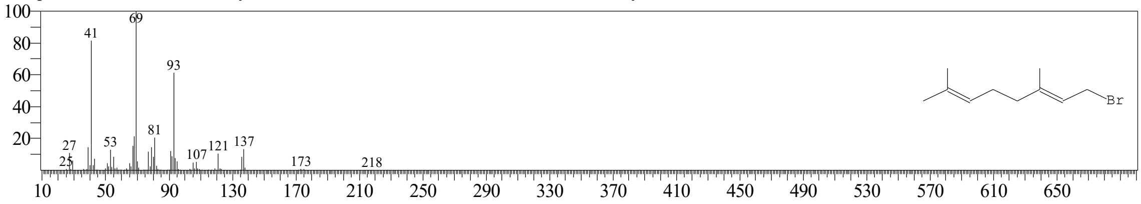

Hit#:5 Entry:9781 Library:NIST11.lib
SI:85 Formula:C10H16 CAS:3387-41-5 MolWeight:136 RetIndex:897
CompName:Bicyclo[3.1.0]hexane, 4-methylene-1-(1-methylethyl)- $$ 4(10)-Thujene $$ Sabinen $$ Sabinene $$ (+)-Sabinene $$ THUJENE, 4(10)- $$ 1-Isopro

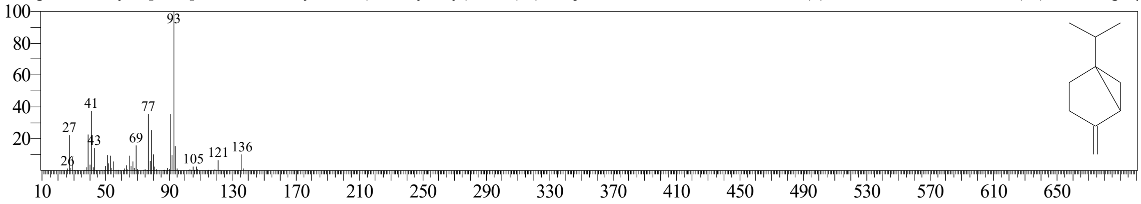

<< Target >>
Line#:15 R.Time:5.655(Scan#:332) MassPeaks:356
RawMode:Averaged 5.650-5.660(331-333) BasePeak:43.05(403178)
BG Mode:Calc. from Peak Group 1 - Event 1 Scan

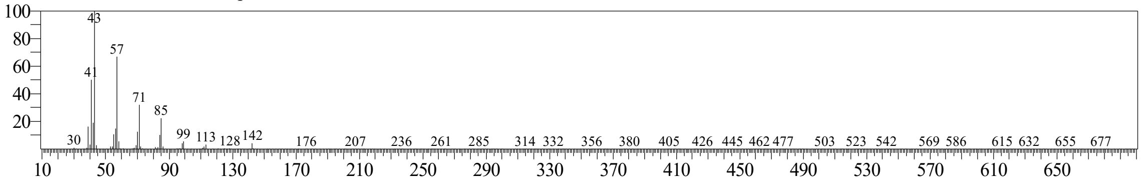

Hit#:1 Entry:7416 Library:NIST11.lib
SI:93 Formula:C9H20 CAS:111-84-2 MolWeight:128 RetIndex:916
CompName:Nonane $$ n-Nonane $$ Shellsol 140 $$ n-C9H20 $$ UN 1920 $$

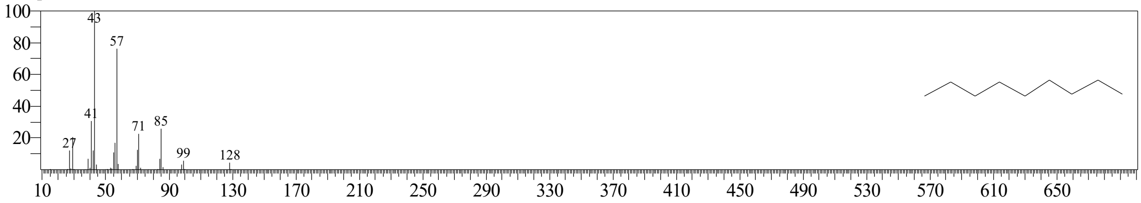

Hit#:2 Entry:12306 Library:NIST11.lib
SI:93 Formula:C10H22 CAS:871-83-0 MolWeight:142 RetIndex:951
CompName:Nonane, 2-methyl- $$ 2-Methylnonane $$

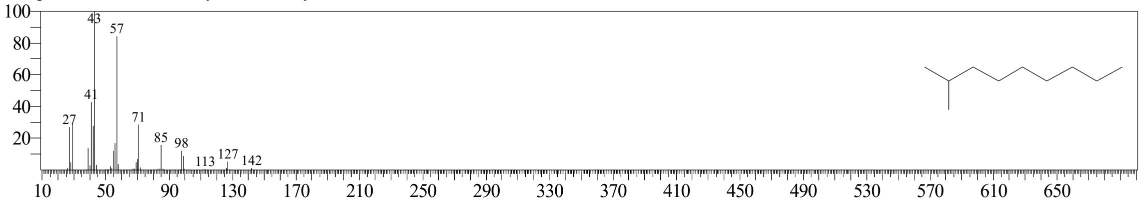

Hit#:3 Entry:34138 Library:NIST11.lib
SI:92 Formula:C13H28 CAS:1560-97-0 MolWeight:184 RetIndex:1249
CompName:Dodecane, 2-methyl- $$ 11-Methyldodecane $$ 2-Methyldodecane $$

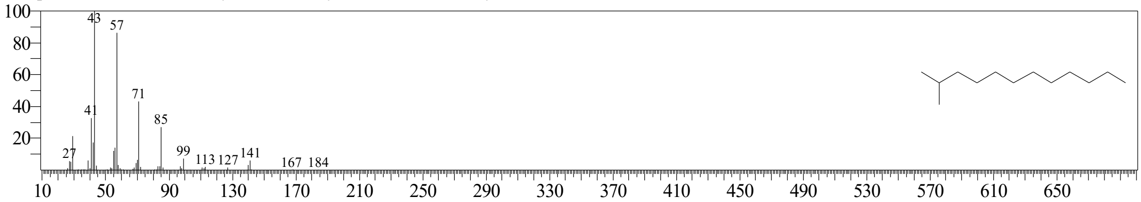

Hit#:4 Entry:26094 Library:NIST11.lib
SI:92 Formula:C12H26 CAS:1002-17-1 MolWeight:170 RetIndex:1086
CompName:Decane, 2,9-dimethyl- $$ 2,9-Dimethyldecane $$

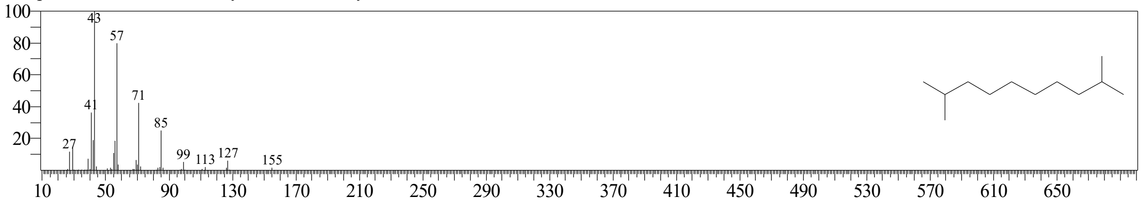

Hit#:5 Entry:7414 Library:NIST11.lib
SI:91 Formula:C9H20 CAS:3221-61-2 MolWeight:128 RetIndex:852
CompName:Octane, 2-methyl- $$ 2-Methyloctane $$ Isononane $$

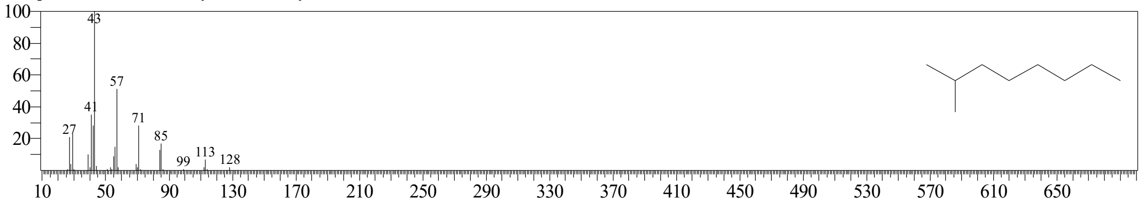

Qualitative Analysis Report

<< Target >>
Line#:16 R.Time:5.790(Scan#:359) MassPeaks:366
RawMode:Averaged 5.785-5.795(358-360) BasePeak:93.10(202583)
BG Mode:Calc. from Peak Group 1 - Event 1 Scan

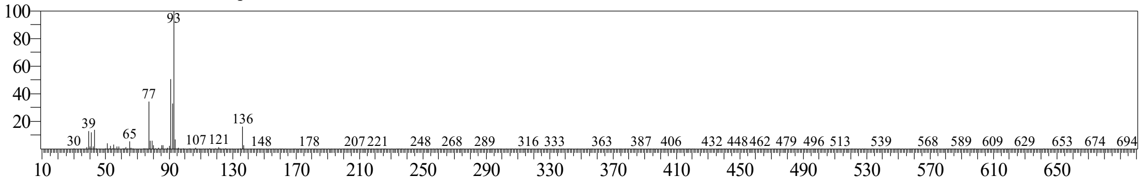

Hit#:1 Entry:9805 Library:NIST11.lib
SI:92 Formula:C10H16 CAS:99-83-2 MolWeight:136 RetIndex:969
CompName:.alpha.-Phellandrene $$ 1,3-Cyclohexadiene, 2-methyl-5-(1-methylethyl)- $$ .alpha.-Fellandrene $$ p-Mentha-1,5-diene $$ 5-Isopropyl-2-methyl-1,

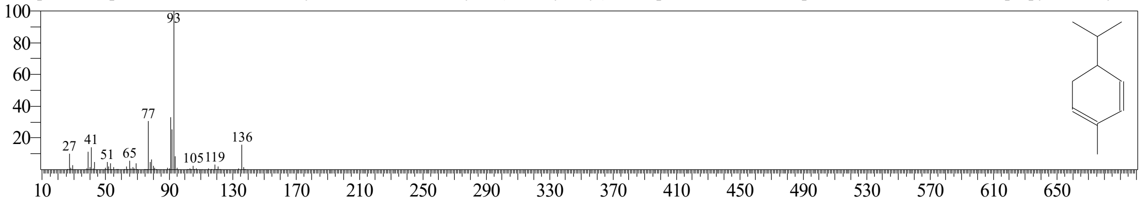

Hit#:2 Entry:9791 Library:NIST11.lib
SI:92 Formula:C10H16 CAS:2867-05-2 MolWeight:136 RetIndex:902
CompName:Bicyclo[3.1.0]hex-2-ene, 2-methyl-5-(1-methylethyl)- $$ 3-Thujene $$ .alpha.-Thujene $$ Origanene $$ 5-Isopropyl-2-methylbicyclo[3.1.0]hex-2-e

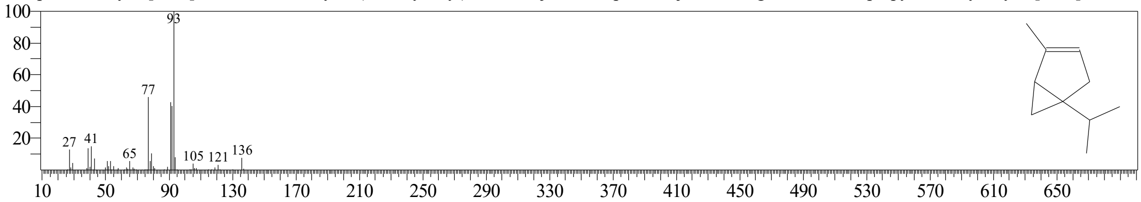

Hit#:3 Entry:9789 Library:NIST11.lib
SI:91 Formula:C10H16 CAS:28634-89-1 MolWeight:136 RetIndex:873
CompName:Bicyclo[3.1.0]hex-2-ene, 4-methyl-1-(1-methylethyl)- $$ 2-Thujene $$ .beta.-Thujene $$ 1-Isopropyl-4-methylbicyclo[3.1.0]hex-2-ene $$

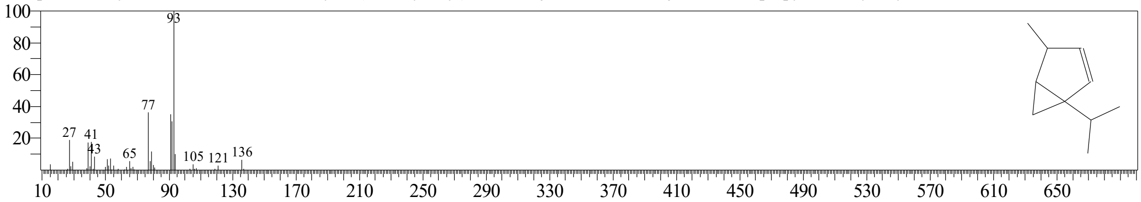

Hit#:4 Entry:9790 Library:NIST11.lib
SI:87 Formula:C10H16 CAS:555-10-2 MolWeight:136 RetIndex:964
CompName:.beta.-Phellandrene $$ Cyclohexene, 3-methylene-6-(1-methylethyl)- $$ p-Mentha-1(7),2-diene $$ Phellandrene, .beta. $$ 3-Isopropyl-6-methylene-

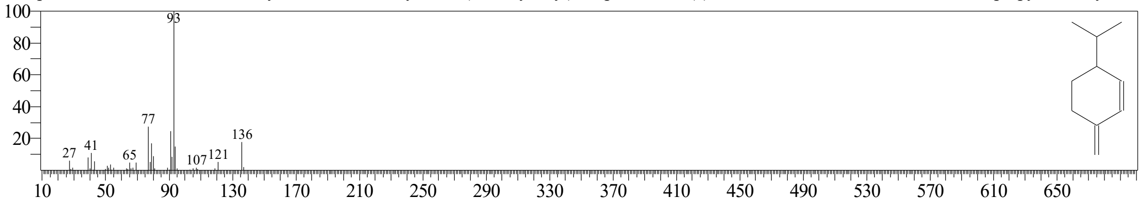

Hit#:5 Entry:9812 Library:NIST11.lib
SI:86 Formula:C10H16 CAS:4889-83-2 MolWeight:136 RetIndex:948
CompName:Bicyclo[3.1.1]hept-2-ene, 3,6,6-trimethyl- $$ 2-Norpinene, 3,6,6-trimethyl- $$ 3-Methyl-apopinene $$ 3,6,6-Trimethylbicyclo[3.1.1]hept-2-ene # $

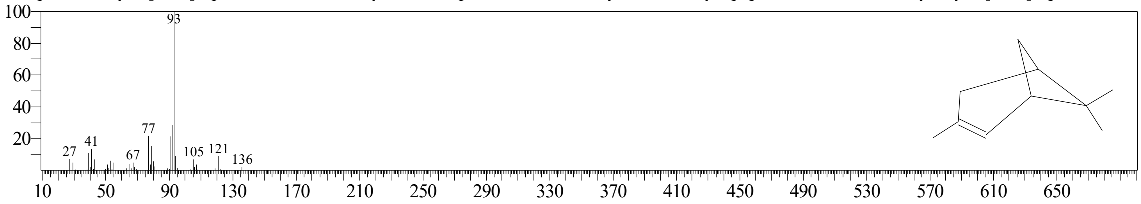

<< Target >>
Line#:17 R.Time:5.855(Scan#:372) MassPeaks:386
RawMode:Averaged 5.850-5.860(371-373) BasePeak:43.05(13092)
BG Mode:Calc. from Peak Group 1 - Event 1 Scan

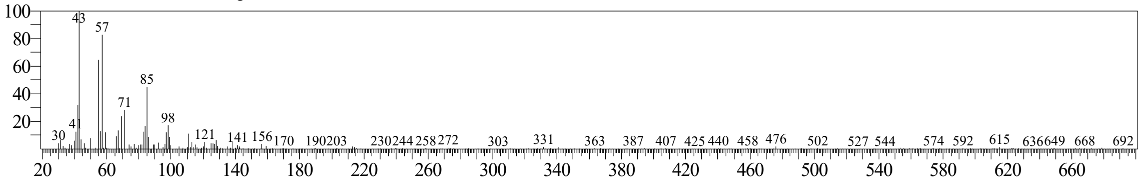

Hit#:1 Entry:171262 Library:NIST11.lib
SI:80 Formula:C21H44O3S CAS:0-00-0 MolWeight:376 RetIndex:2732
CompName:Sulfurous acid, hexyl pentadecyl ester

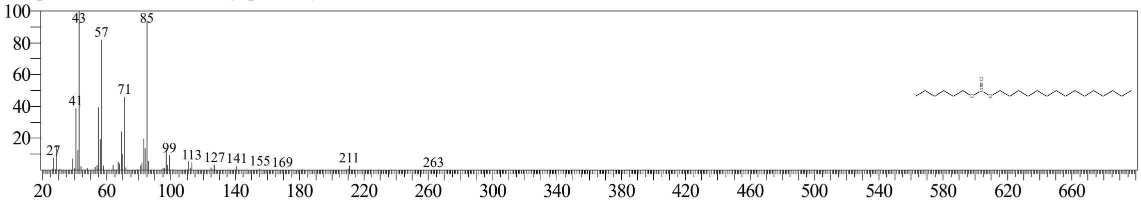

Hit#:2 Entry:190888 Library:NIST11.lib
SI:80 Formula:C26H50O4 CAS:0-00-0 MolWeight:426 RetIndex:2940
CompName:Oxalic acid, hexyl octadecyl ester

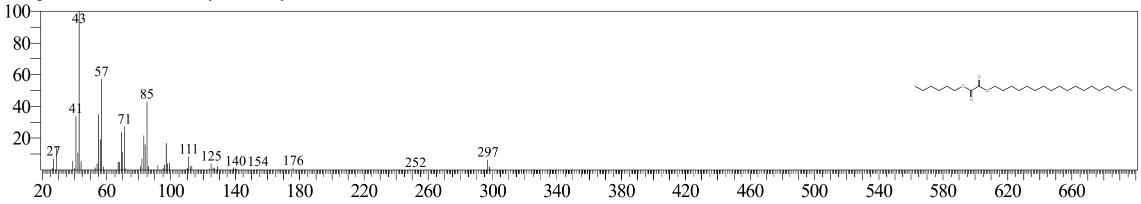

Hit#:3 Entry:186586 Library:NIST11.lib
SI:80 Formula:C25H48O4 CAS:0-00-0 MolWeight:412 RetIndex:2841
CompName:Oxalic acid, heptadecyl hexyl ester

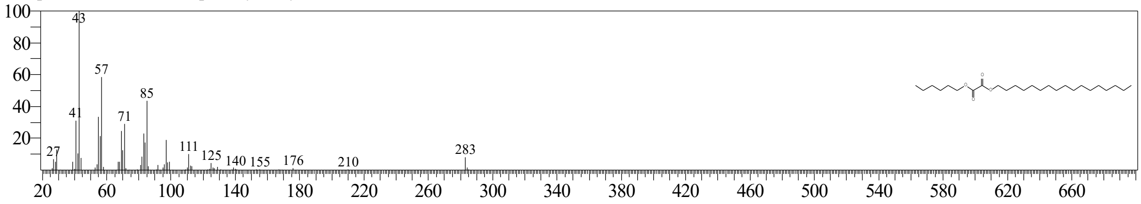

Hit#:4 Entry:175157 Library:NIST11.lib
SI:79 Formula:C23H44O4 CAS:0-00-0 MolWeight:384 RetIndex:2642
CompName:Oxalic acid, hexyl pentadecyl ester

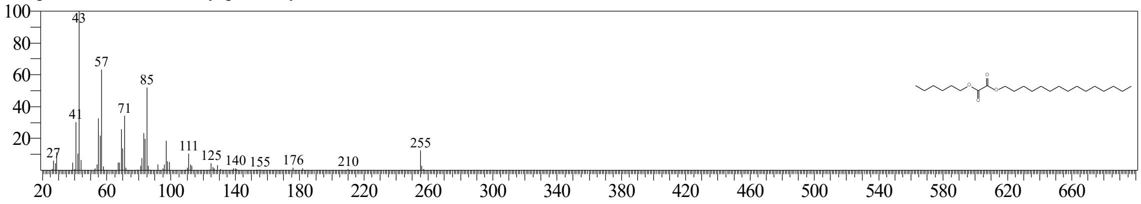

Hit#:5 Entry:195056 Library:NIST11.lib
SI:79 Formula:C22H45Cl3Si CAS:7325-84-0 MolWeight:442 RetIndex:2647
CompName:Silane, trichlorodocosyl- $$ Docosyltrichlorosilane $$ Trichloro(docosyl)silane # $$

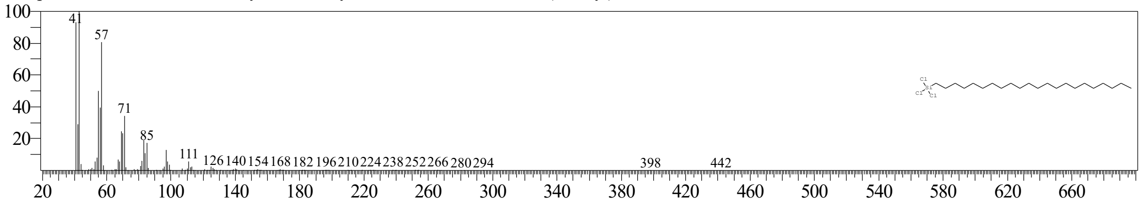

Qualitative Analysis Report

<< Target >>
Line#:18 R.Time:5.895(Scan#:380) MassPeaks:364
RawMode:Averaged 5.890-5.900(379-381) BasePeak:43.05(97874)
BG Mode:Calc. from Peak Group 1 - Event 1 Scan

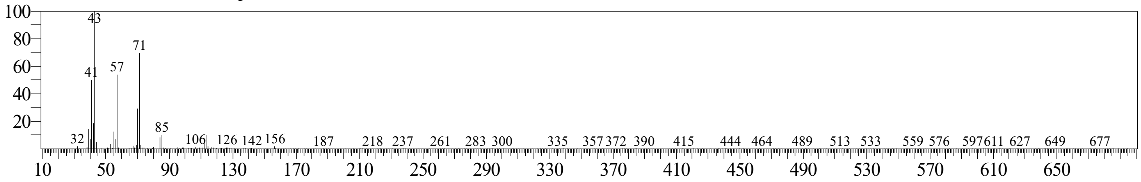

Hit#:1 Entry:18711 Library:NIST11.lib
SI:92 Formula:C11H24 CAS:2847-72-5 MolWeight:156 RetIndex:1051
CompName:Decane, 4-methyl- $$ 4-Methyldecane $$

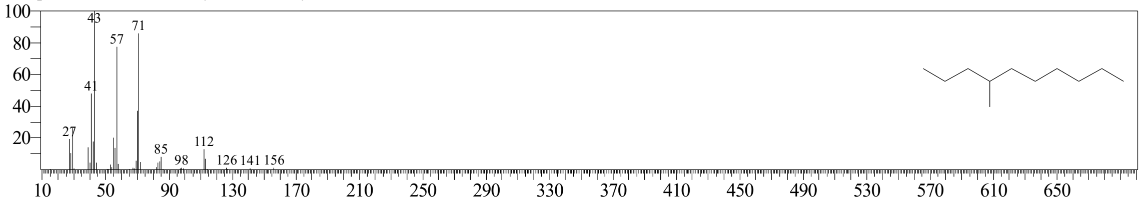

Hit#:2 Entry:26097 Library:NIST11.lib
SI:91 Formula:C12H26 CAS:2980-69-0 MolWeight:170 RetIndex:1150
CompName:Undecane, 4-methyl- $$ 4-Methylundecane $$

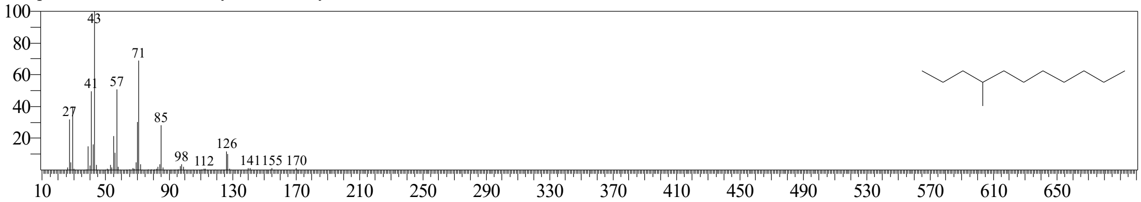

Hit#:3 Entry:18706 Library:NIST11.lib
SI:91 Formula:C11H24 CAS:17302-28-2 MolWeight:156 RetIndex:986
CompName:Nonane, 2,6-dimethyl- $$ 2,6-Dimethylnonane $$

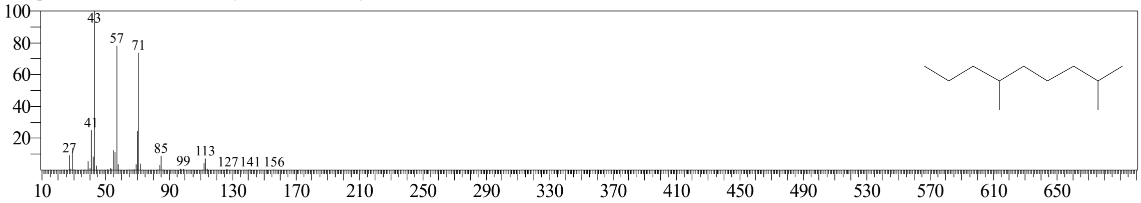

Hit#:4 Entry:34145 Library:NIST11.lib
SI:90 Formula:C13H28 CAS:17301-33-6 MolWeight:184 RetIndex:1185
CompName:Undecane, 4,8-dimethyl- $$ 4,8-Dimethylundecane # $$

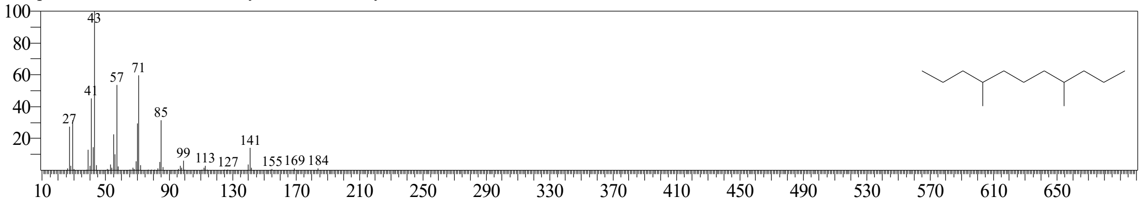

Hit#:5 Entry:12310 Library:NIST11.lib
SI:90 Formula:C10H22 CAS:13475-78-0 MolWeight:142 RetIndex:887
CompName:Heptane, 5-ethyl-2-methyl- $$ 5-Ethyl-2-methylheptane $$ Heptane, 3-ethyl-6-methyl $$

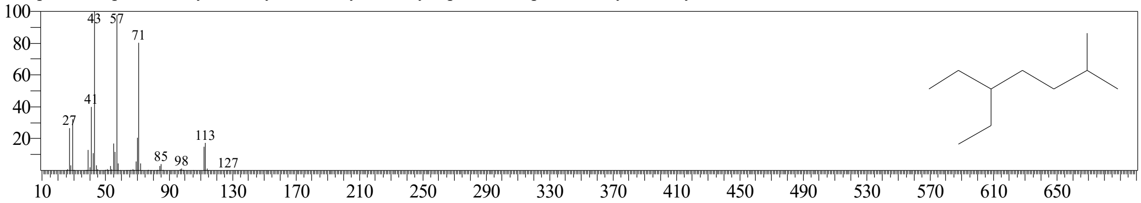

<< Target >>
Line#:19 R.Time:5.955(Scan#:392) MassPeaks:352
RawMode:Averaged 5.950-5.960(391-393) BasePeak:41.05(16471)
BG Mode:Calc. from Peak Group 1 - Event 1 Scan

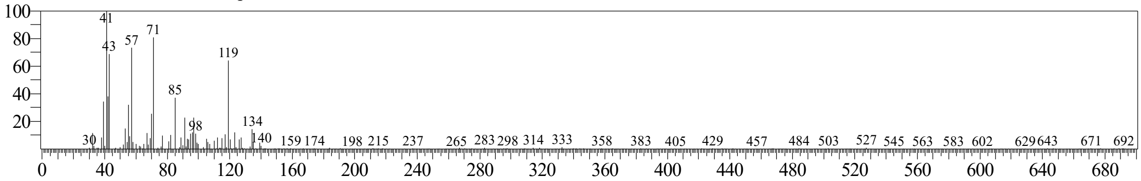

Hit#:1 Entry:156678 Library:NIST11.lib
SI:76 Formula:C18H37ClO2S CAS:0-00-0 MolWeight:352 RetIndex:2493
CompName:1-Octadecanesulphonyl chloride

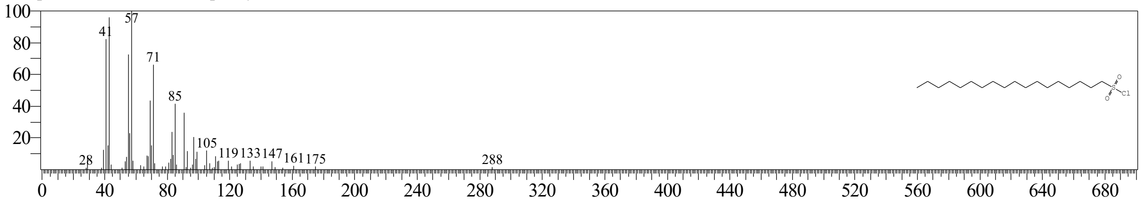

Hit#:2 Entry:47433 Library:NIST11.lib
SI:75 Formula:C9H19Br CAS:2216-35-5 MolWeight:206 RetIndex:1148
CompName:2-Bromononane $$ sec-Nonyl bromide $$ Nonane, 2-bromo- $$

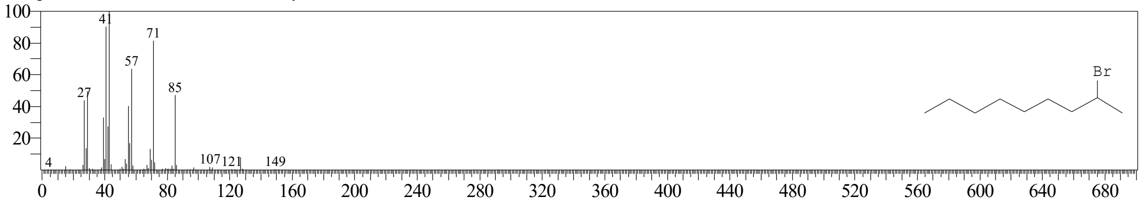

Hit#:3 Entry:131260 Library:NIST11.lib
SI:75 Formula:C20H41Cl CAS:42217-02-7 MolWeight:316 RetIndex:2234
CompName:1-Chloroeicosane $$ Eicosane, 1-chloro- $$ 1-Chloroicosane # $$

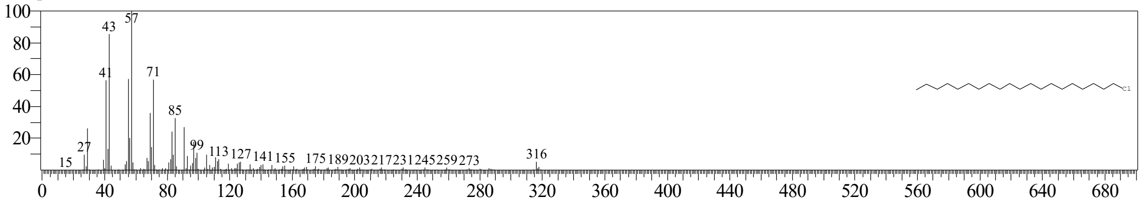

Hit#:4 Entry:109236 Library:NIST11.lib
SI:73 Formula:C18H37Cl CAS:3386-33-2 MolWeight:288 RetIndex:2036
CompName:Octadecane, 1-chloro- $$ n-Octadecyl chloride $$ Octadecyl chloride $$ 1-Chlorooctadecane $$ Chlorooctadecane $$

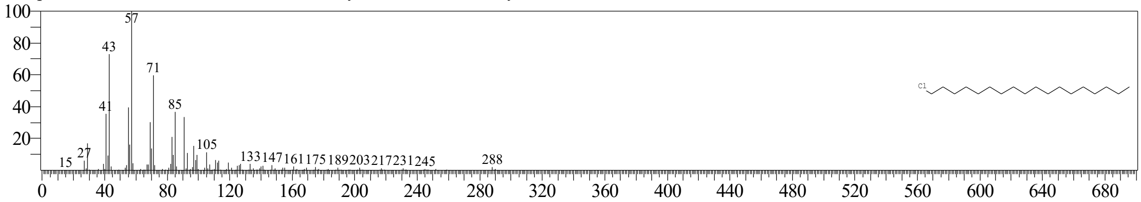

Hit#:5 Entry:37395 Library:NIST11.lib
SI:73 Formula:C11H23Cl CAS:2473-03-2 MolWeight:190 RetIndex:1340
CompName:1-Chloroundecane $$ n-Undecyl chloride $$ Undecane, 1-chloro- $$ Undecyl chloride $$

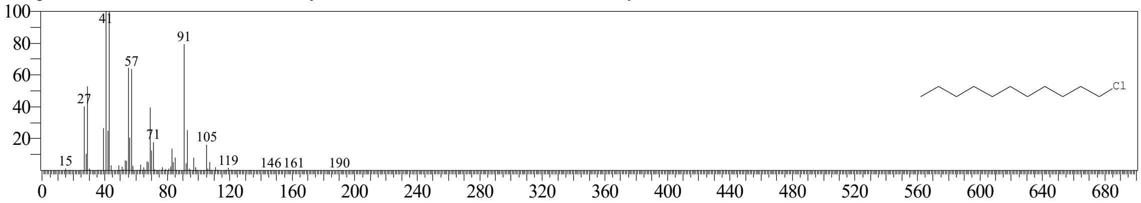

Qualitative Analysis Report

<< Target >>
Line#:20 R.Time:6.010(Scan#:403) MassPeaks:373
RawMode:Averaged 6.005-6.015(402-404) BasePeak:105.05(28078)
BG Mode:Calc. from Peak Group 1 - Event 1 Scan

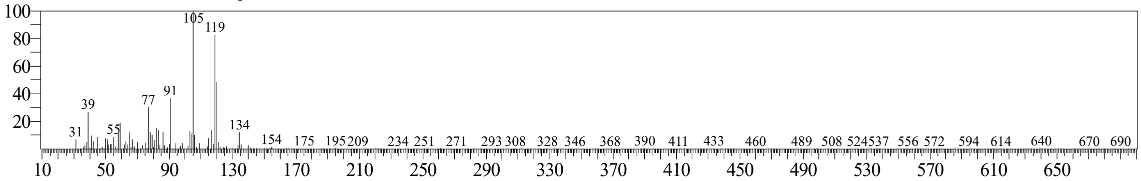

Hit#:1 Entry:9104 Library:NIST11.lib
SI:76 Formula:C10H14 CAS:135-01-3 MolWeight:134 RetIndex:1106
CompName:Benzene, 1,2-diethyl- $$ Benzene, o-diethyl- $$ o-Diethylbenzene $$ 1,2-Diethylbenzene $$ ortho-Diethylbenzene $$ 1,2-Diethylbenzene $$

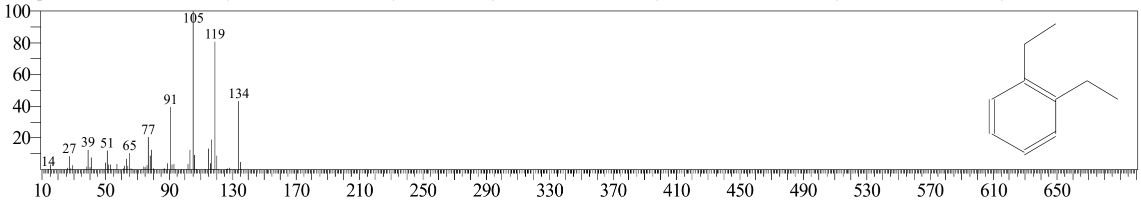

Hit#:2 Entry:9116 Library:NIST11.lib
SI:75 Formula:C10H14 CAS:141-93-5 MolWeight:134 RetIndex:1106
CompName:Benzene, 1,3-diethyl- $$ Benzene, m-diethyl- $$ m-Diethylbenzene $$ 1,3-Diethylbenzene $$ meta-Diethylbenzene $$

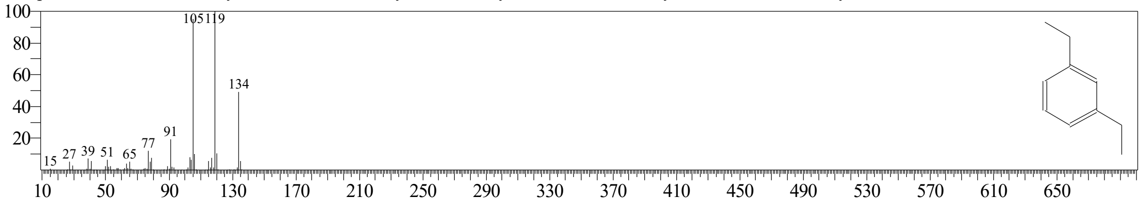

Hit#:3 Entry:5345 Library:NIST11.lib
SI:75 Formula:C9H12 CAS:14296-81-2 MolWeight:120 RetIndex:920
CompName:Cyclohexane, 1,2,4-tris(methylene)- $$ 1,2,4-Trimethylenecyclohexane # $$

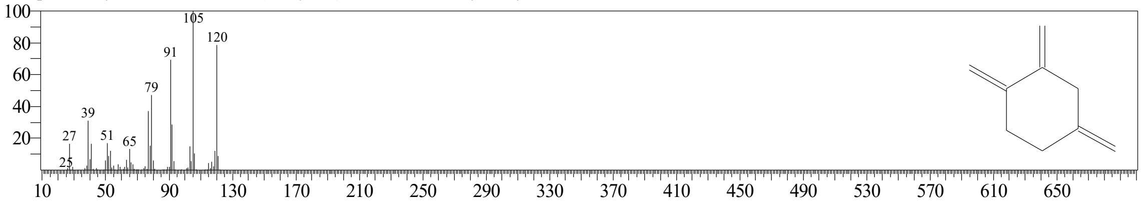

Hit#:4 Entry:5334 Library:NIST11.lib
SI:74 Formula:C9H12 CAS:76003-40-2 MolWeight:120 RetIndex:868
CompName:1-Hepten-5-yne, 2-methyl-3-methylene- $$ 2-Methyl-3-methylene-1-hepten-5-yne # $$

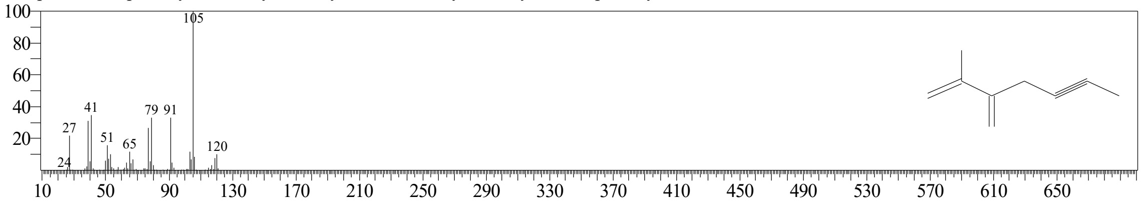

Hit#:5 Entry:5337 Library:NIST11.lib
SI:73 Formula:C9H12 CAS:95-63-6 MolWeight:120 RetIndex:1020
CompName:Benzene, 1,2,4-trimethyl- $$ .psi.-Cumene $$ aS-Trimethylbenzene $$ Pseudocumene $$ Pseudocumol $$ 1,2,4-Trimethylbenzene $$ 1,2,5-Trimeth

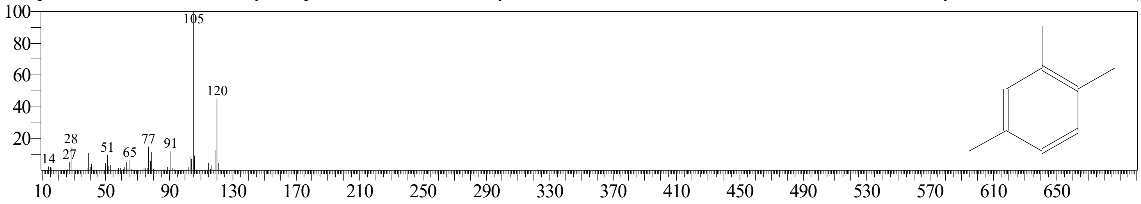

Qualitative Analysis Report

<< Target >>
Line#:21 R.Time:6.085(Scan#:418) MassPeaks:341
RawMode:Averaged 6.080-6.090(417-419) BasePeak:93.10(247119)
BG Mode:Calc. from Peak Group 1 - Event 1 Scan

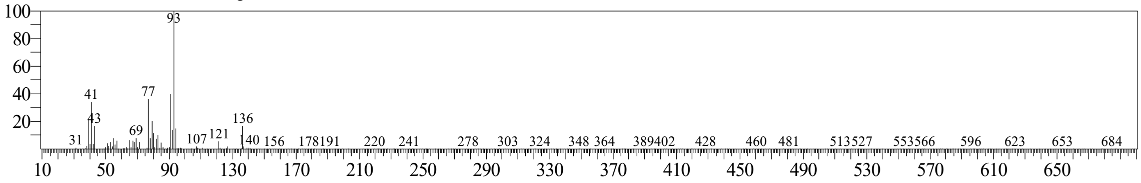

Hit#:1 Entry:9781 Library:NIST11.lib
SI:90 Formula:C10H16 CAS:3387-41-5 MolWeight:136 RetIndex:897
CompName:Bicyclo[3.1.0]hexane, 4-methylene-1-(1-methylethyl)- $$ 4(10)-Thujene $$ Sabinen $$ Sabinene $$ (+)-Sabinene $$ THUJENE, 4(10)- $$ 1-Isopro[illegible]

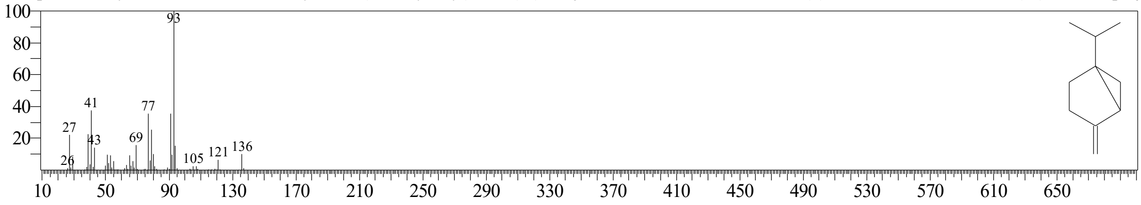

Hit#:2 Entry:9808 Library:NIST11.lib
SI:86 Formula:C10H16 CAS:508-32-7 MolWeight:136 RetIndex:729
CompName:Tricyclo[2.2.1.0(2,6)]heptane, 1,7,7-trimethyl- $$ Tricyclene $$ 1,7,7-Trimethyltricyclo[2.2.1.0,2,6]heptane $$ 1,7,7-Trimethyltricyclo[2.2.1.0(2,6)][illegible]

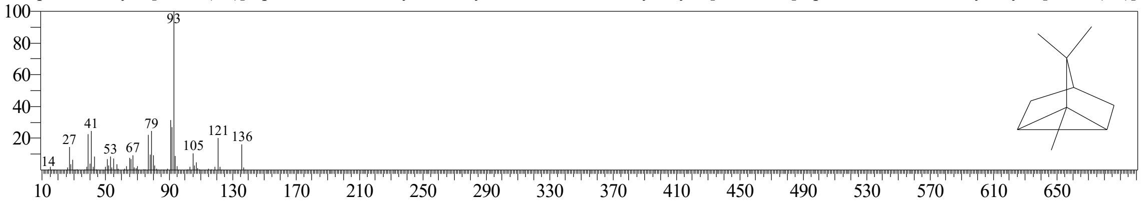

Hit#:3 Entry:9796 Library:NIST11.lib
SI:86 Formula:C10H16 CAS:68998-21-0 MolWeight:136 RetIndex:928
CompName:Cyclopropane, 1,1-dimethyl-2-(3-methyl-1,3-butadienyl)- $$ 1,1-Dimethyl-2-[(1E)-3-methyl-1,3-butadienyl]cyclopropane # $$

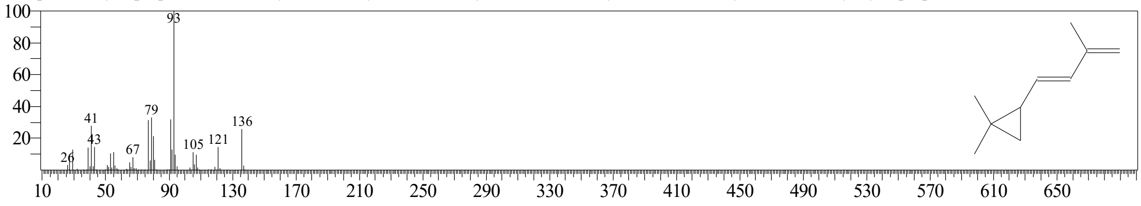

Hit#:4 Entry:22339 Library:NIST11.lib
SI:86 Formula:C10H16N2 CAS:87143-58-6 MolWeight:164 RetIndex:0
CompName:3,5-Methanocyclopentapyrazole, 3,3a,4,5,6,6a-hexahydro-3a,4,4-trimethyl-

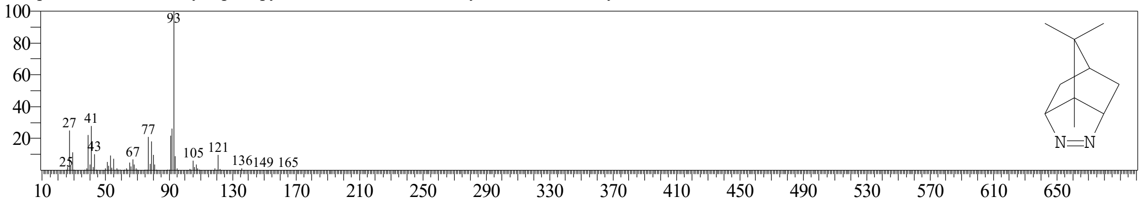

Hit#:5 Entry:9777 Library:NIST11.lib
SI:86 Formula:C10H16 CAS:127-91-3 MolWeight:136 RetIndex:943
CompName:.beta.-Pinene $$ Bicyclo[3.1.1]heptane, 6,6-dimethyl-2-methylene- $$ 2(10)-Pinene $$ Nopinen $$ Nopinene $$ Pseudopinen $$ Pseudopinene $$ [illegible]

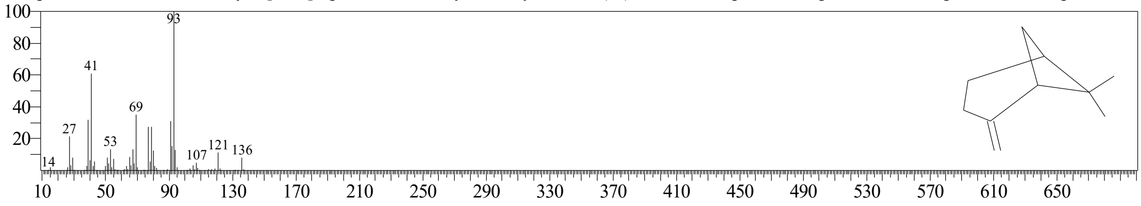

<< Target >>
Line#:22 R.Time:6.275(Scan#:456) MassPeaks:410
RawMode:Averaged 6.270-6.280(455-457) BasePeak:57.10(49476)
BG Mode:Calc. from Peak Group 1 - Event 1 Scan

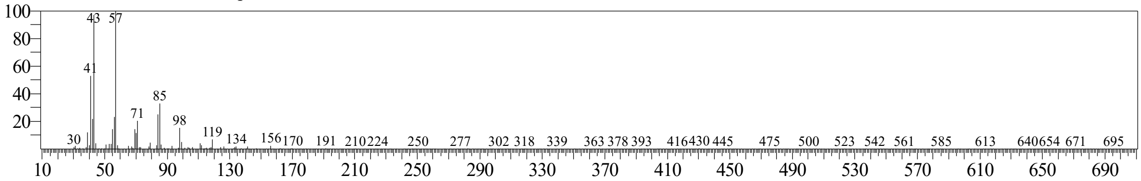

Hit#:1 Entry:12325 Library:NIST11.lib
SI:89 Formula:C10H22 CAS:13475-81-5 MolWeight:142 RetIndex:846
CompName:Hexane, 2,2,3,3-tetramethyl- $$ 2,2,3,3-Tetramethylhexane $$

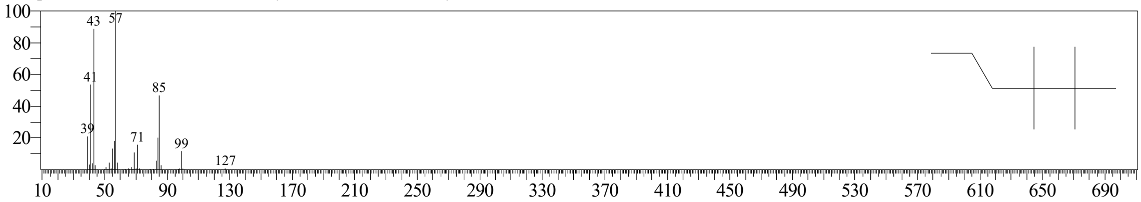

Hit#:2 Entry:12315 Library:NIST11.lib
SI:89 Formula:C10H22 CAS:2613-61-8 MolWeight:142 RetIndex:823
CompName:Heptane, 2,4,6-trimethyl- $$ 2,4,6-Trimethylheptane $$

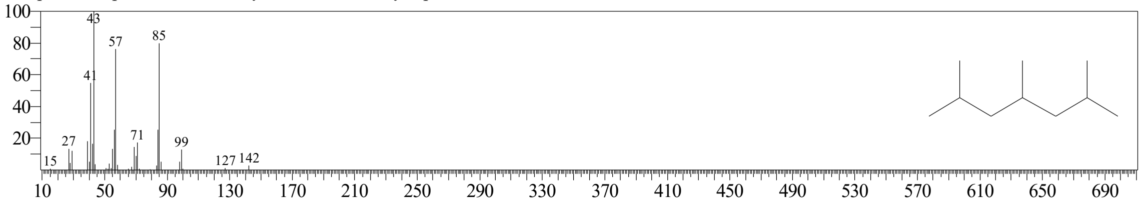

Hit#:3 Entry:7428 Library:NIST11.lib
SI:88 Formula:C9H20 CAS:16747-33-4 MolWeight:128 RetIndex:767
CompName:Pentane, 3-ethyl-2,3-dimethyl- $$ 3-Ethyl-2,3-dimethylpentane $$

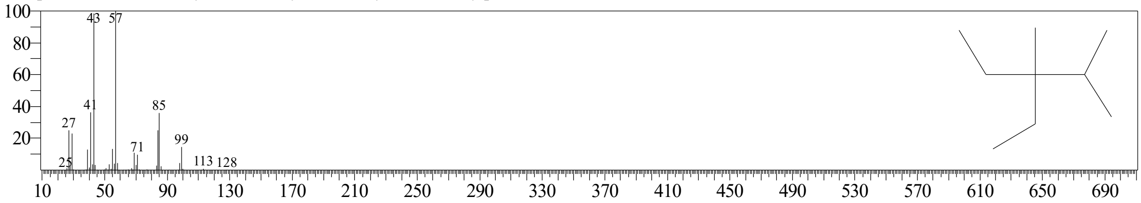

Hit#:4 Entry:4236 Library:NIST11.lib
SI:88 Formula:C8H18 CAS:589-43-5 MolWeight:114 RetIndex:688
CompName:Hexane, 2,4-dimethyl- $$ 2,4-Dimethylhexane $$

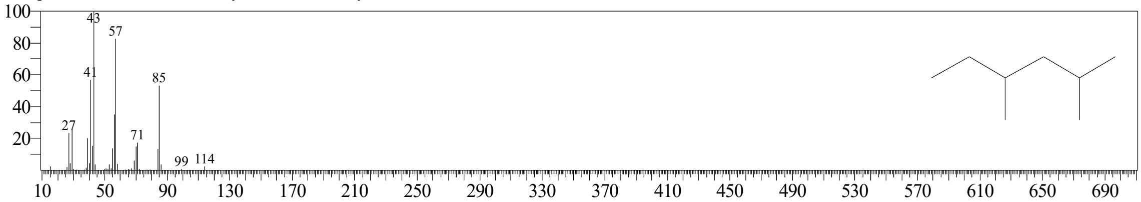

Hit#:5 Entry:12320 Library:NIST11.lib
SI:88 Formula:C10H22 CAS:124-18-5 MolWeight:142 RetIndex:1015
CompName:Decane $$ n-Decane $$ n-C10H22 $$ UN 2247 $$

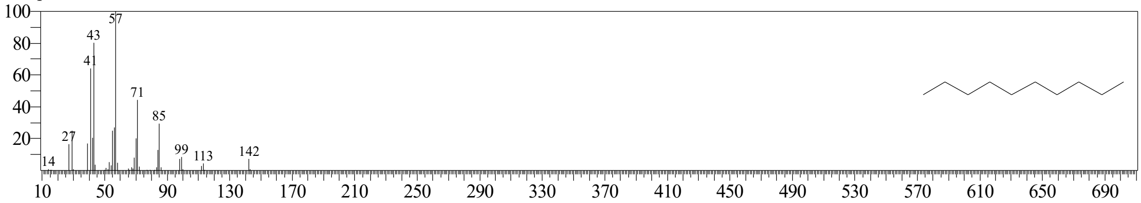

<< Target >>
Line#:23 R.Time:6.315(Scan#:464) MassPeaks:359
RawMode:Averaged 6.310-6.320(463-465) BasePeak:43.05(55028)
BG Mode:Calc. from Peak Group 1 - Event 1 Scan

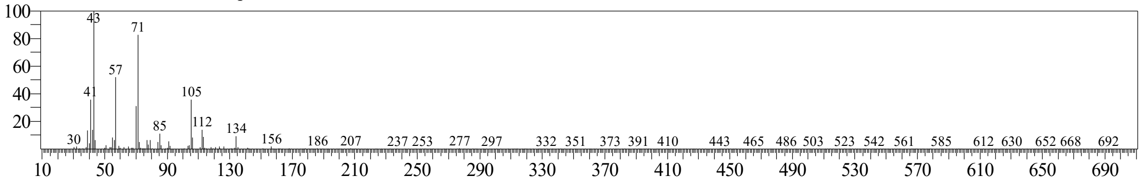

Hit#:1 Entry:12310 Library:NIST11.lib
SI:85 Formula:C10H22 CAS:13475-78-0 MolWeight:142 RetIndex:887
CompName:Heptane, 5-ethyl-2-methyl- $$ 5-Ethyl-2-methylheptane $$ Heptane, 3-ethyl-6-methyl $$

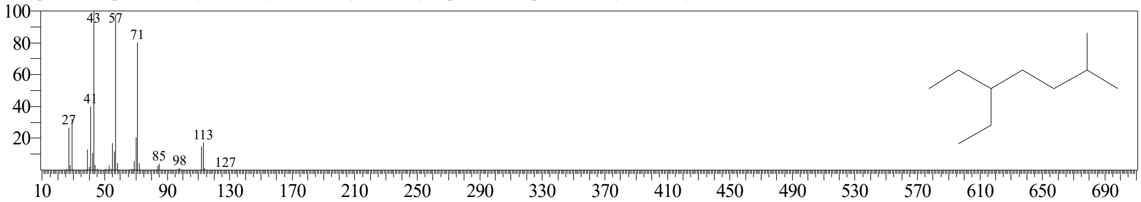

Hit#:2 Entry:18711 Library:NIST11.lib
SI:84 Formula:C11H24 CAS:2847-72-5 MolWeight:156 RetIndex:1051
CompName:Decane, 4-methyl- $$ 4-Methyldecane $$

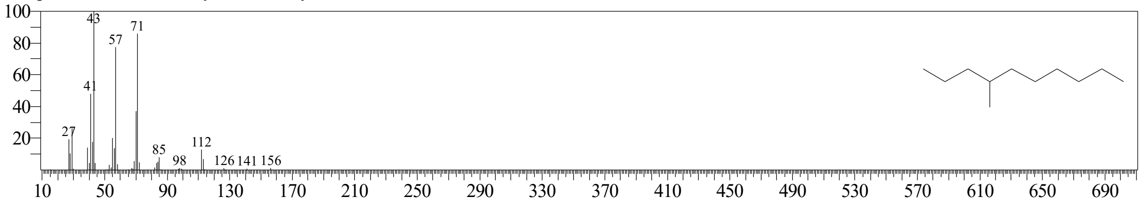

Hit#:3 Entry:12309 Library:NIST11.lib
SI:83 Formula:C10H22 CAS:5881-17-4 MolWeight:142 RetIndex:951
CompName:Octane, 3-ethyl- $$ 3-Ethyloctane $$

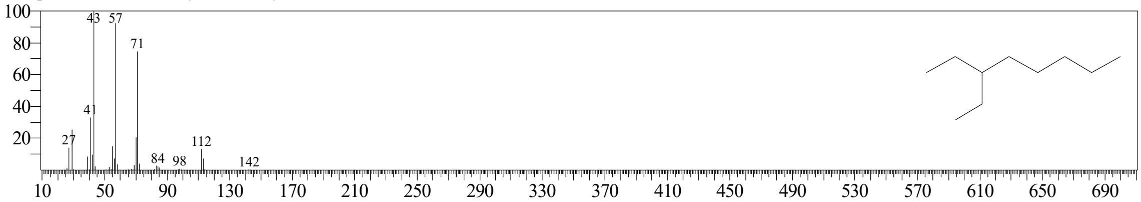

Hit#:4 Entry:12312 Library:NIST11.lib
SI:83 Formula:C10H22 CAS:1189-99-7 MolWeight:142 RetIndex:867
CompName:Heptane, 2,5,5-trimethyl- $$ 2,5,5-Trimethylheptane $$

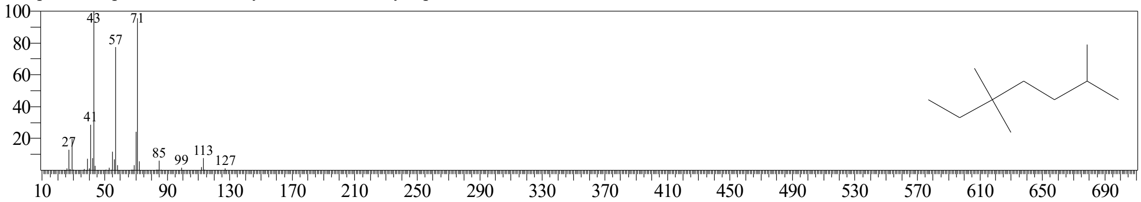

Hit#:5 Entry:18706 Library:NIST11.lib
SI:82 Formula:C11H24 CAS:17302-28-2 MolWeight:156 RetIndex:986
CompName:Nonane, 2,6-dimethyl- $$ 2,6-Dimethylnonane $$

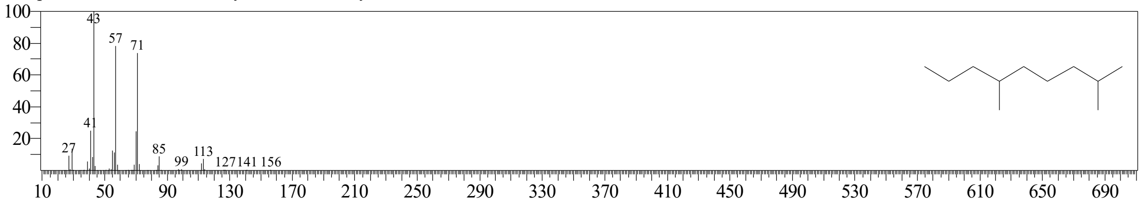

<< Target >>
Line#:24 R.Time:6.365(Scan#:474) MassPeaks:381
RawMode:Averaged 6.360-6.370(473-475) BasePeak:43.10(88046)
BG Mode:Calc. from Peak Group 1 - Event 1 Scan

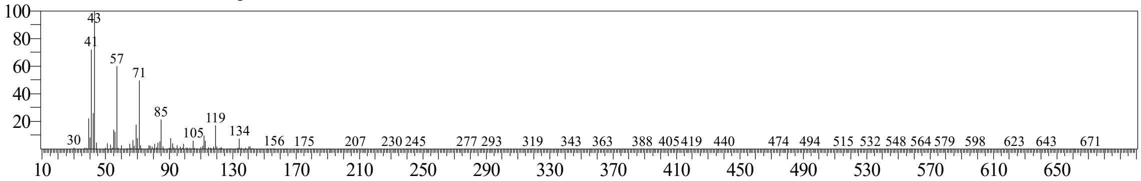

Hit#:1 Entry:24835 Library:NIST11.lib
SI:86 Formula:C12H24 CAS:54410-98-9 MolWeight:168 RetIndex:1012
CompName:1-Nonene, 4,6,8-trimethyl- $$ 4,6,8-Trimethyl-1-nonene # $$

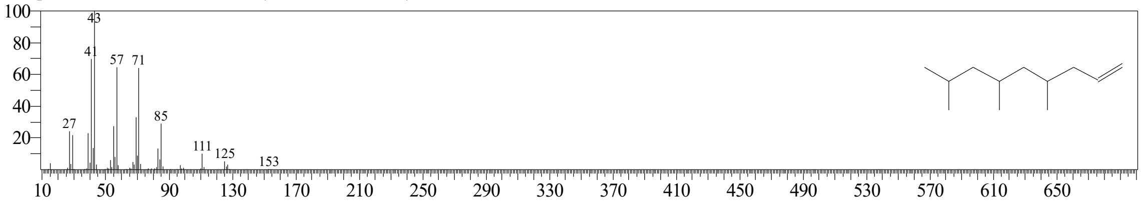

Hit#:2 Entry:18717 Library:NIST11.lib
SI:86 Formula:C11H24 CAS:6975-98-0 MolWeight:156 RetIndex:1051
CompName:Decane, 2-methyl- $$ 2-Methyldecane $$ n-C8H17CH(CH3)2 $$

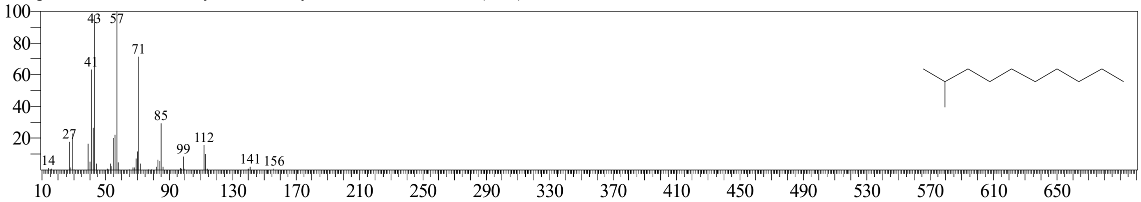

Hit#:3 Entry:18707 Library:NIST11.lib
SI:84 Formula:C11H24 CAS:62016-30-2 MolWeight:156 RetIndex:966
CompName:Octane, 2,3,3-trimethyl- $$ 2,3,3-Trimethyloctane # $$

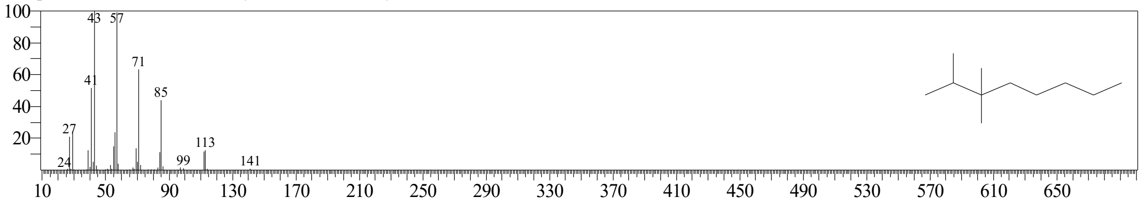

Hit#:4 Entry:92779 Library:NIST11.lib
SI:83 Formula:C10H21I CAS:0-00-0 MolWeight:268 RetIndex:1365
CompName:1-Iodo-2-methylnonane

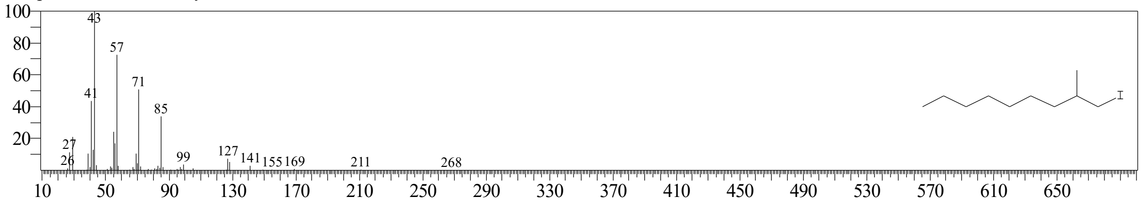

Hit#:5 Entry:34145 Library:NIST11.lib
SI:83 Formula:C13H28 CAS:17301-33-6 MolWeight:184 RetIndex:1185
CompName:Undecane, 4,8-dimethyl- $$ 4,8-Dimethylundecane # $$

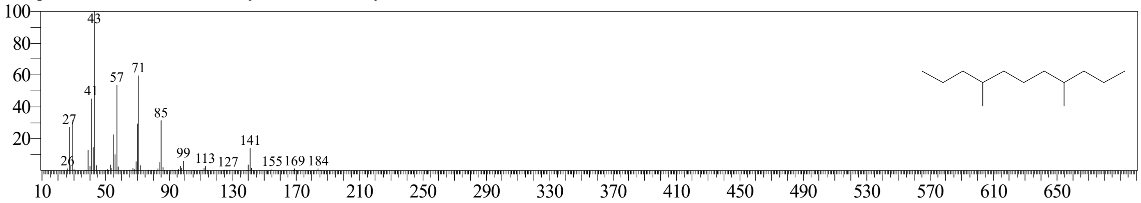

<< Target >>
Line#:25 R.Time:6.430(Scan#:487) MassPeaks:366
RawMode:Averaged 6.425-6.435(486-488) BasePeak:41.05(43288)
BG Mode:Calc. from Peak Group 1 - Event 1 Scan

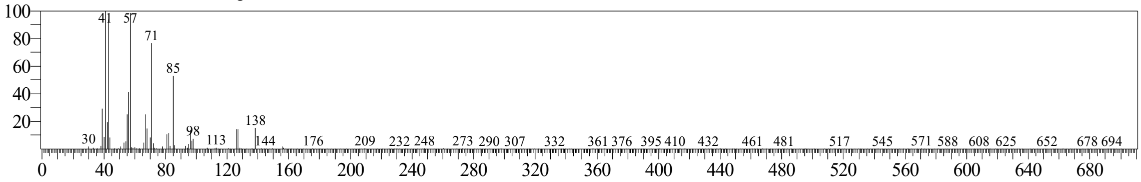

Hit#:1 Entry:18725 Library:NIST11.lib
SI:87 Formula:C11H24 CAS:13151-34-3 MolWeight:156 RetIndex:1051
CompName:Decane, 3-methyl- $$ 3-Methyldecane $$ 2-Ethylnonane $$

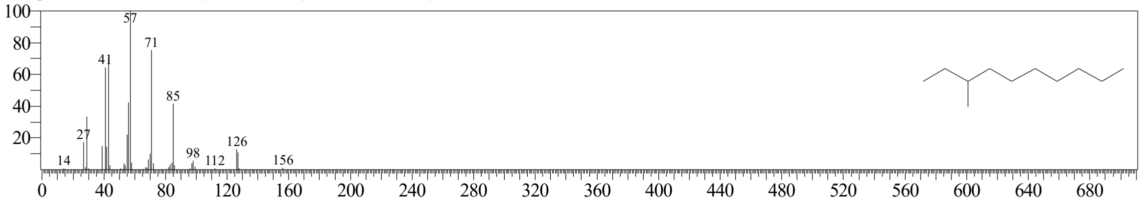

Hit#:2 Entry:47433 Library:NIST11.lib
SI:86 Formula:C9H19Br CAS:2216-35-5 MolWeight:206 RetIndex:1148
CompName:2-Bromononane $$ sec-Nonyl bromide $$ Nonane, 2-bromo- $$

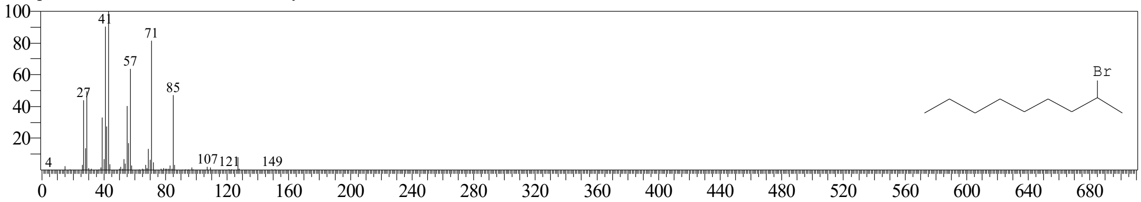

Hit#:3 Entry:34183 Library:NIST11.lib
SI:85 Formula:C13H28 CAS:62185-54-0 MolWeight:184 RetIndex:1185
CompName:Nonane, 5-(1-methylpropyl)- $$ 5-Sec-butylnonane # $$

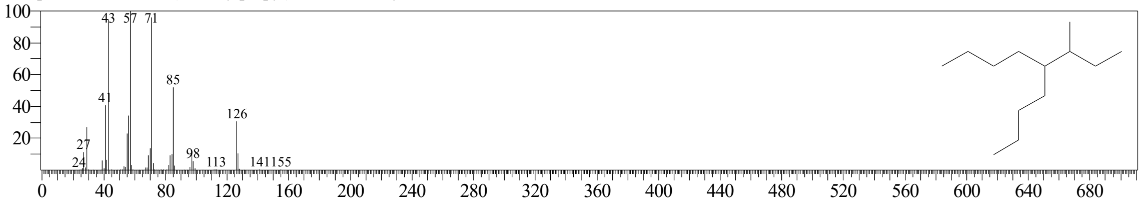

Hit#:4 Entry:34182 Library:NIST11.lib
SI:84 Formula:C13H28 CAS:62185-53-9 MolWeight:184 RetIndex:1185
CompName:Nonane, 5-(2-methylpropyl)- $$ 5-Isobutylnonane # $$

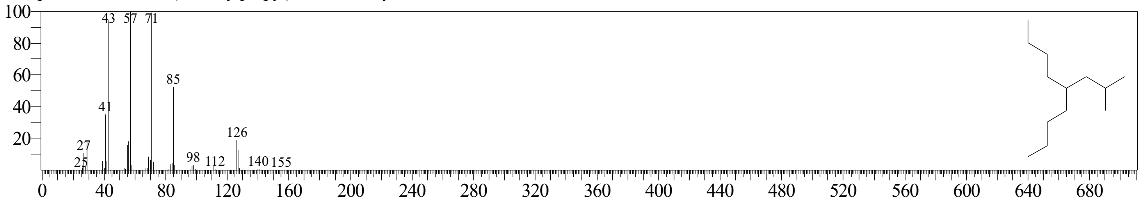

Hit#:5 Entry:34132 Library:NIST11.lib
SI:84 Formula:C13H28 CAS:17312-78-6 MolWeight:184 RetIndex:1185
CompName:Undecane, 3,4-dimethyl- $$ 3,4-Dimethylundecane # $$

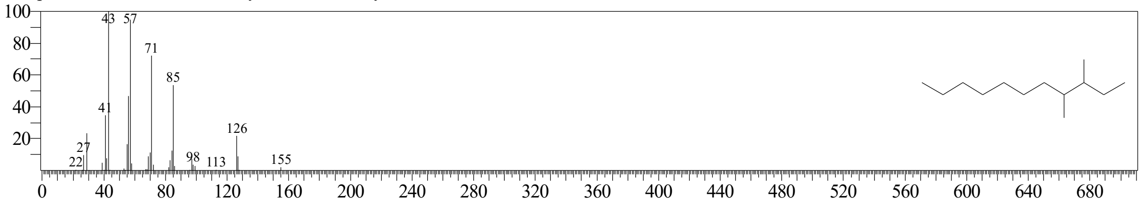

<< Target >>
Line#:26 R.Time:6.475(Scan#:496) MassPeaks:356
RawMode:Averaged 6.470-6.480(495-497) BasePeak:105.00(7976)
BG Mode:Calc. from Peak Group 1 - Event 1 Scan

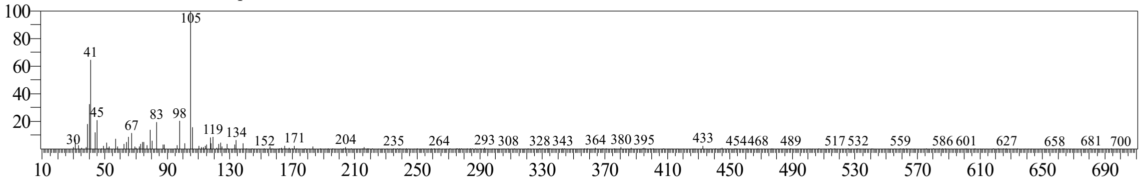

Hit#:1 Entry:126544 Library:NIST11.lib
SI:66 Formula:C21H26O2 CAS:39000-49-2 MolWeight:310 RetIndex:2288
CompName:2,4-Di-tert-butylphenyl benzoate

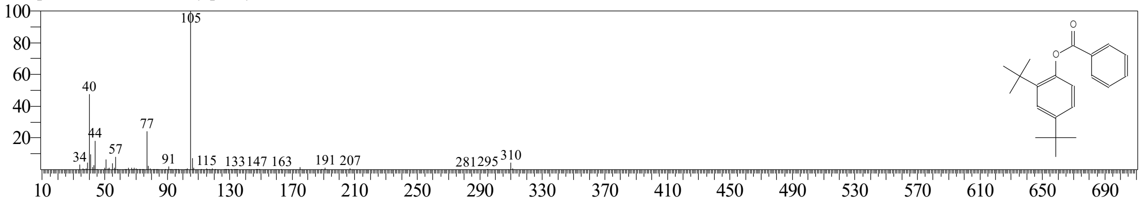

Hit#:2 Entry:109139 Library:NIST11.lib
SI:63 Formula:C17H17ClO2 CAS:52196-47-1 MolWeight:288 RetIndex:2330
CompName:3-tert-Butyl-5-chloro-2-hydroxybenzophenone $$ (3-tert-Butyl-5-chloro-2-hydroxyphenyl)(phenyl)methanone # $$

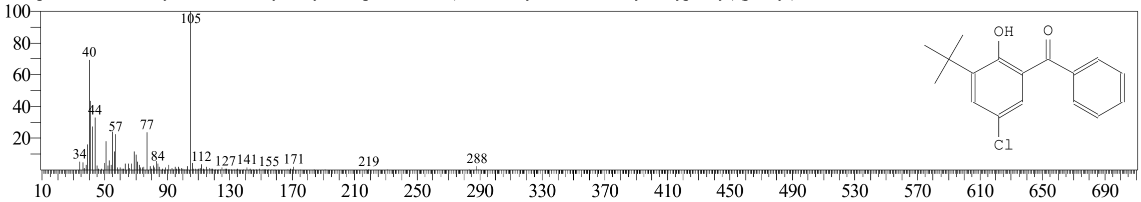

Hit#:3 Entry:36298 Library:NIST11.lib
SI:62 Formula:C12H12O2 CAS:150967-24-1 MolWeight:188 RetIndex:1415
CompName:Benzoic acid, 2-methylene-3-butenyl ester $$ 2-Methylene-3-butenyl benzoate # $$

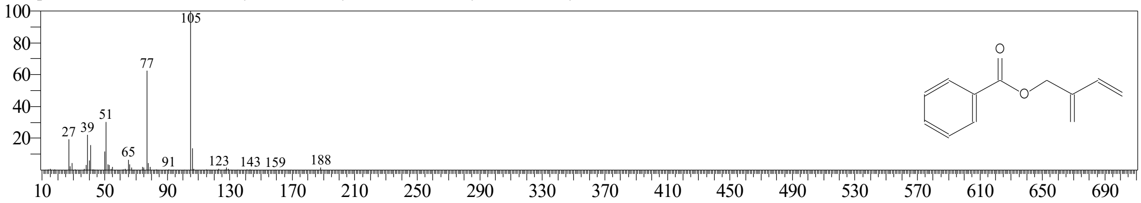

Hit#:4 Entry:80726 Library:NIST11.lib
SI:62 Formula:C12H12O2S2 CAS:153837-88-8 MolWeight:252 RetIndex:2087
CompName:Thiophene, 2-(4-methylbenzylsulfonyl)- $$ 2-[(4-Methylbenzyl)sulfonyl]thiophene # $$

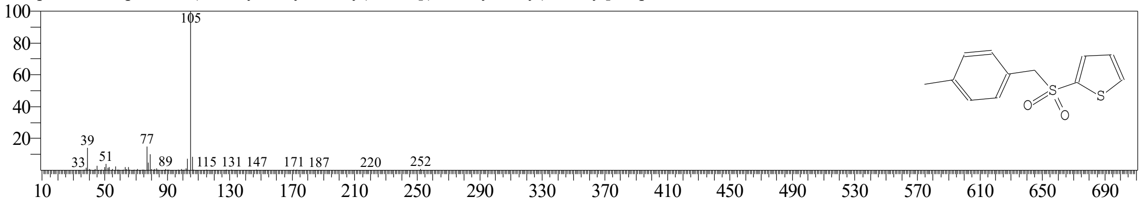

Hit#:5 Entry:57586 Library:NIST11.lib
SI:62 Formula:C14H20O2 CAS:0-00-0 MolWeight:220 RetIndex:1508
CompName:Benzoic acid, 4,4-dimethylpent-2-yl ester

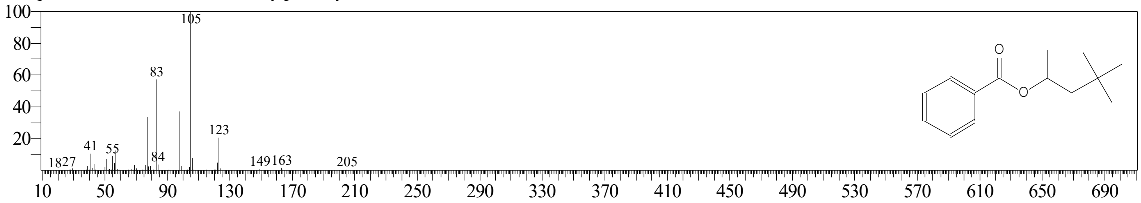

<< Target >>
Line#:27 R.Time:6.540(Scan#:509) MassPeaks:344
RawMode:Averaged 6.535-6.545(508-510) BasePeak:41.10(5426)
BG Mode:Calc. from Peak Group 1 - Event 1 Scan

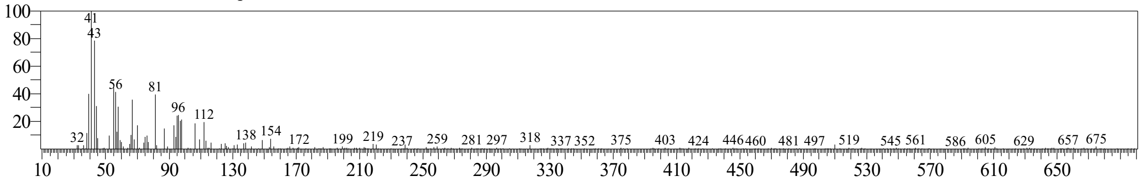

Hit#:1 Entry:17455 Library:NIST11.lib
SI:71 Formula:C10H18O CAS:69668-93-5 MolWeight:154 RetIndex:1195
CompName:3-Decyn-2-ol

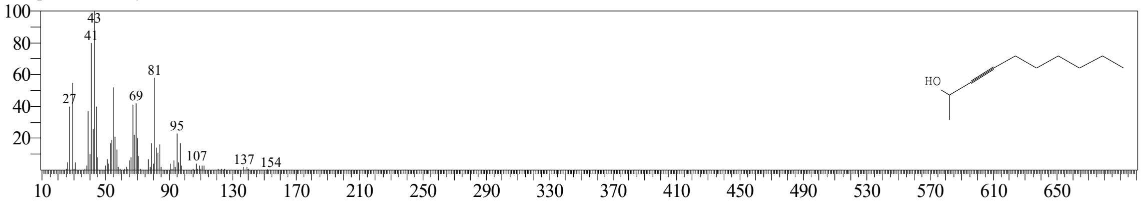

Hit#:2 Entry:60905 Library:NIST11.lib
SI:70 Formula:C15H28O CAS:2834-00-6 MolWeight:224 RetIndex:1772
CompName:2-Pentadecyn-1-ol

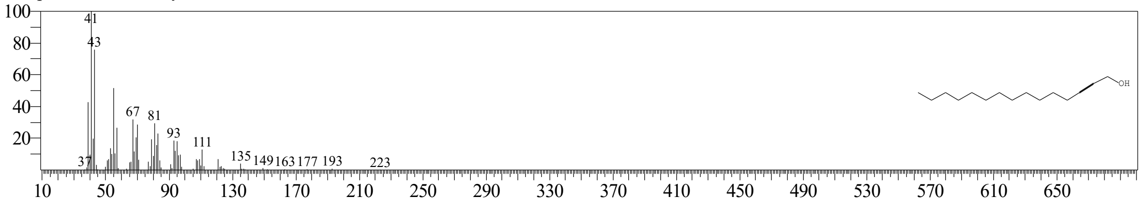

Hit#:3 Entry:40289 Library:NIST11.lib
SI:70 Formula:C14H26 CAS:765-10-6 MolWeight:194 RetIndex:1411
CompName:1-Tetradecyne $$ 1-C14H26 $$

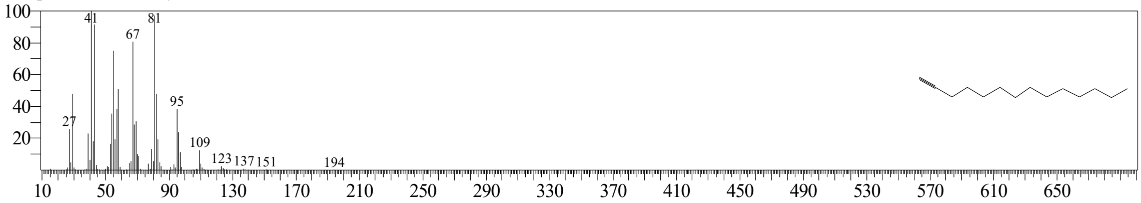

Hit#:4 Entry:86123 Library:NIST11.lib
SI:70 Formula:C10H17N3O3S CAS:349572-30-1 MolWeight:259 RetIndex:2234
CompName:Sulfonamide, N-cyclohexyl-N'-(5-methylisoxazol-3-yl)- $$ N-Cyclohexyl-N'-(5-methyl-3-isoxazolyl)sulfamide # $$

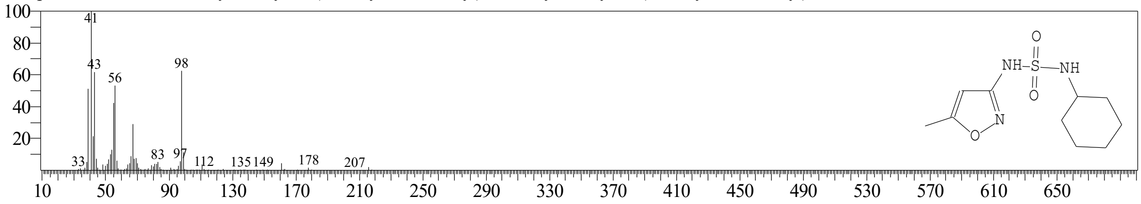

Hit#:5 Entry:10479 Library:NIST11.lib
SI:70 Formula:C10H18 CAS:764-93-2 MolWeight:138 RetIndex:1013
CompName:1-Decyne $$ Octylacetylene $$ 1-C10H18 $$

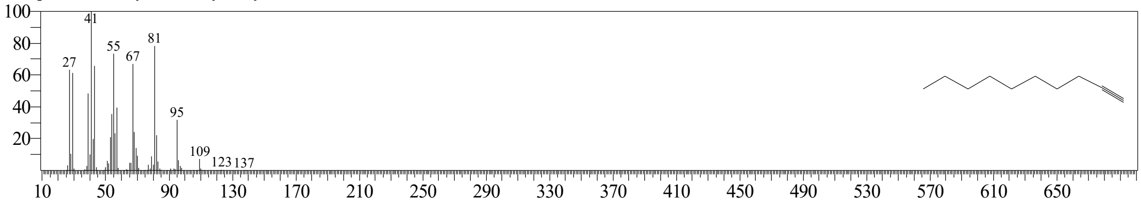

Qualitative Analysis Report

<< Target >>
Line#:28 R.Time:6.625(Scan#:526) MassPeaks:362
RawMode:Averaged 6.620-6.630(525-527) BasePeak:119.00(21649)
BG Mode:Calc. from Peak Group 1 - Event 1 Scan

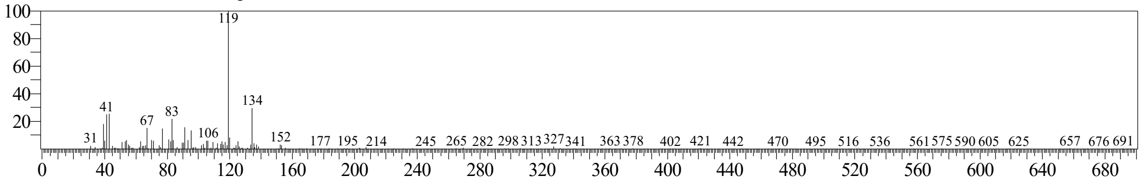

Hit#:1 Entry:9120 Library:NIST11.lib
SI:72 Formula:C10H14 CAS:933-98-2 MolWeight:134 RetIndex:1119
CompName:Benzene, 1-ethyl-2,3-dimethyl- $$ o-Xylene, 3-ethyl- $$ 1-Ethyl-2,3-dimethylbenzene $$ 1,2-Dimethyl-3-ethylbenzene $$ 3-Ethyl-o-xylene $$ 1,2-[illegible]

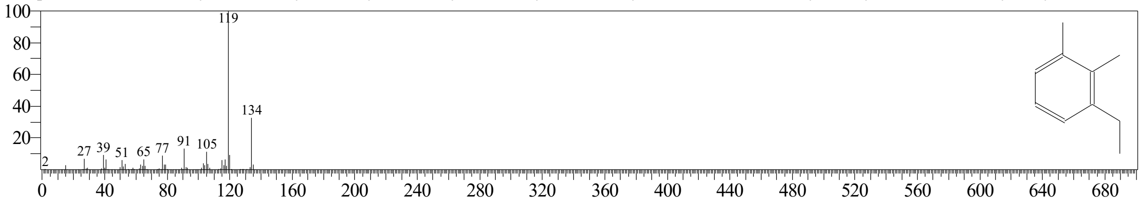

Hit#:2 Entry:40117 Library:NIST11.lib
SI:72 Formula:C12H18O2 CAS:0-00-0 MolWeight:194 RetIndex:1276
CompName:Bicyclo[3.1.1]hept-2-en-4-ol, 2,6,6-trimethyl-, acetate $$ 4,6,6-Trimethylbicyclo[3.1.1]hept-3-en-2-yl acetate # $$

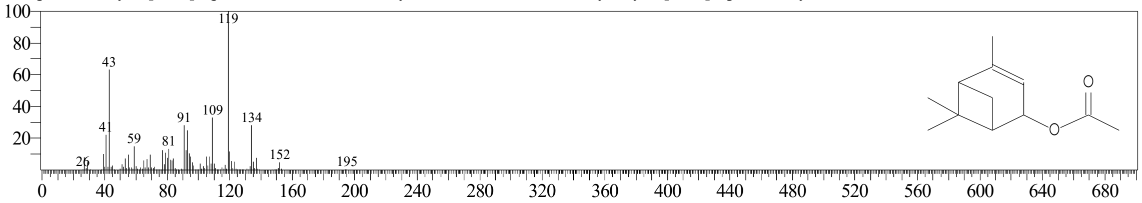

Hit#:3 Entry:29273 Library:NIST11.lib
SI:72 Formula:C12H16O CAS:91531-36-1 MolWeight:176 RetIndex:1354
CompName:Spiro[3.5]nona-5,7-dien-1-one, 5,9,9-trimethyl- $$ 5,9,9-Trimethylspiro[3.5]nona-5,7-dien-1-one # $$

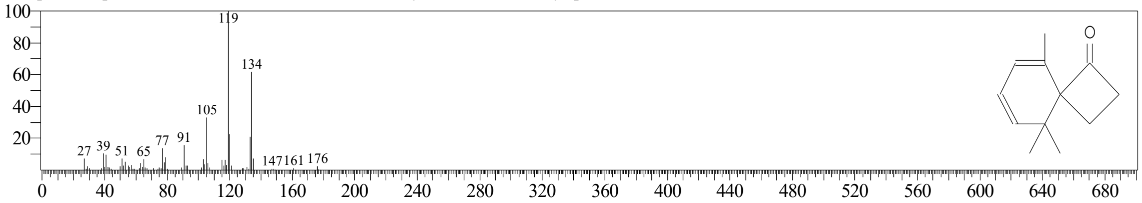

Hit#:4 Entry:9127 Library:NIST11.lib
SI:72 Formula:C10H14 CAS:76089-59-3 MolWeight:134 RetIndex:989
CompName:1,3-Cyclopentadiene, 1,2,3,4-tetramethyl-5-methylene- $$ 1,2,3,4-Tetramethyl-5-methylene-1,3-cyclopentadiene # $$ 1,2,3,4-Tetramethylfulvene $$

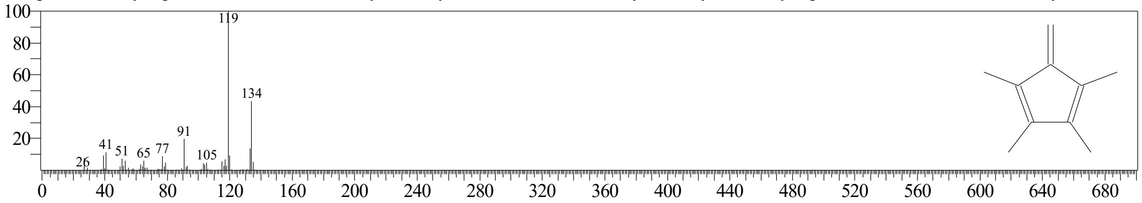

Hit#:5 Entry:9117 Library:NIST11.lib
SI:72 Formula:C10H14 CAS:2870-04-4 MolWeight:134 RetIndex:1119
CompName:Benzene, 2-ethyl-1,3-dimethyl- $$ m-Xylene, 2-ethyl- $$ 1,3-Dimethyl-2-ethylbenzene $$ 2-Ethyl-1,3-dimethylbenzene $$ 1,3-Dimethyl-2-ethyl be[illegible]

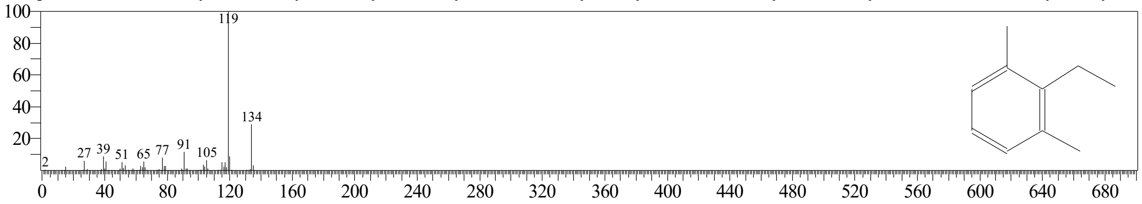

<< Target >>
Line#:29 R.Time:6.710(Scan#:543) MassPeaks:384
RawMode:Averaged 6.705-6.715(542-544) BasePeak:119.05(16751)
BG Mode:Calc. from Peak Group 1 - Event 1 Scan

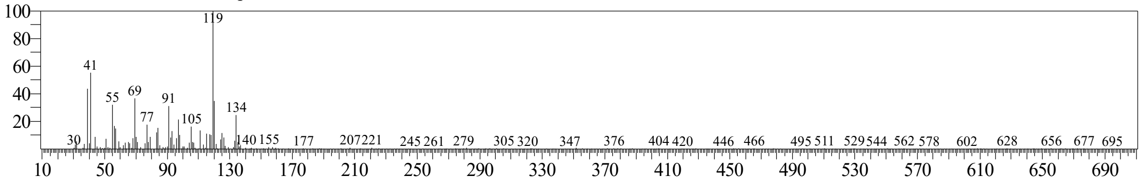

Hit#:1 Entry:46675 Library:NIST11.lib
SI:71 Formula:C15H24 CAS:0-00-0 MolWeight:204 RetIndex:1480
CompName:.beta.-curcumene

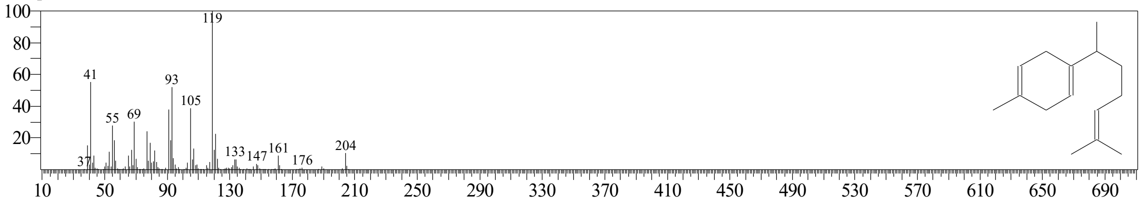

Hit#:2 Entry:21454 Library:NIST11.lib
SI:71 Formula:C12H18 CAS:130930-56-2 MolWeight:162 RetIndex:1111
CompName:1,7,7-Trimethyl-2-vinylbicyclo[2.2.1]hept-2-ene

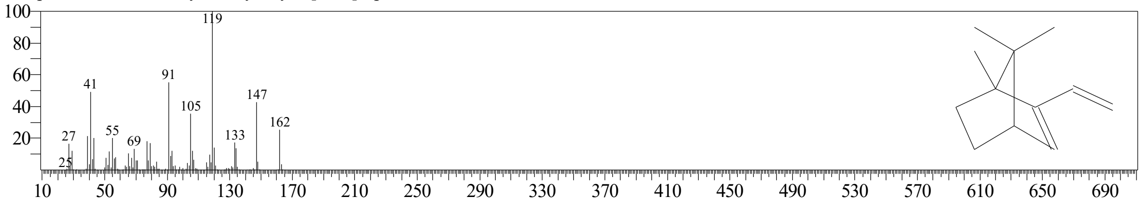

Hit#:3 Entry:46678 Library:NIST11.lib
SI:70 Formula:C15H24 CAS:55123-21-2 MolWeight:204 RetIndex:1425
CompName:Bicyclo[3.1.1]heptane, 6-methyl-2-methylene-6-(4-methyl-3-pentenyl)-, [1R-(1.alpha.,5.alpha.,6.beta.)]- $$ 6-Methyl-2-methylene-6-(4-methyl-3-pe

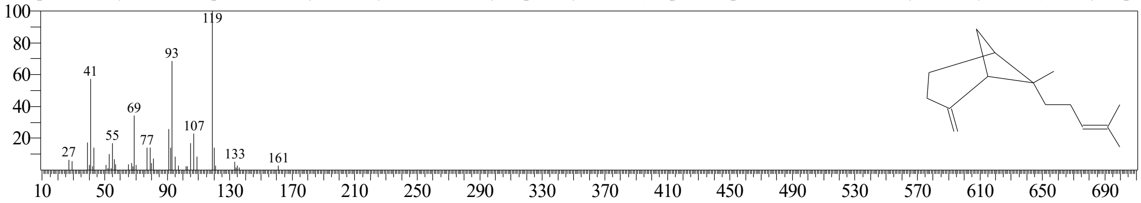

Hit#:4 Entry:16498 Library:NIST11.lib
SI:69 Formula:C10H16O CAS:99-48-9 MolWeight:152 RetIndex:1206
CompName:Carveol $$ p-Mentha-6,8-dien-2-ol $$ 2-Cyclohexen-1-ol, 2-methyl-5-(1-methylethenyl)- $$ p-Mentha-1,8-dien-6-ol $$ 1-Methyl-4-isopropenyl-6-

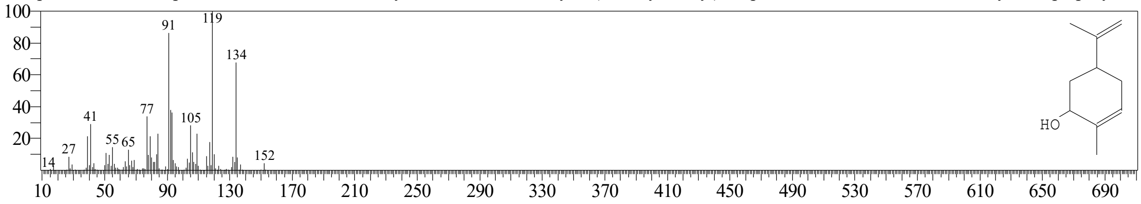

Hit#:5 Entry:21287 Library:NIST11.lib
SI:69 Formula:C10H14N2 CAS:0-00-0 MolWeight:162 RetIndex:0
CompName:1,4-Methano-1H-cyclopenta[d]pyridazine, 4,4a,5,7a-tetrahydro-8,8-dimethyl-, (1.alpha.,4.alpha.,4a.alpha.,7a.alpha.)-

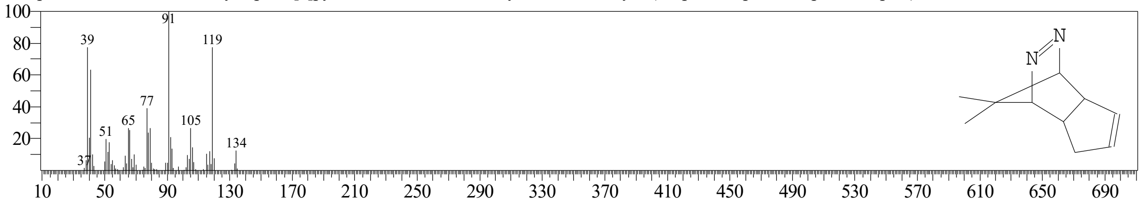

<< Target >>
Line#:30 R.Time:6.755(Scan#:552) MassPeaks:367
RawMode:Averaged 6.750-6.760(551-553) BasePeak:43.05(430960)
BG Mode:Calc. from Peak Group 1 - Event 1 Scan

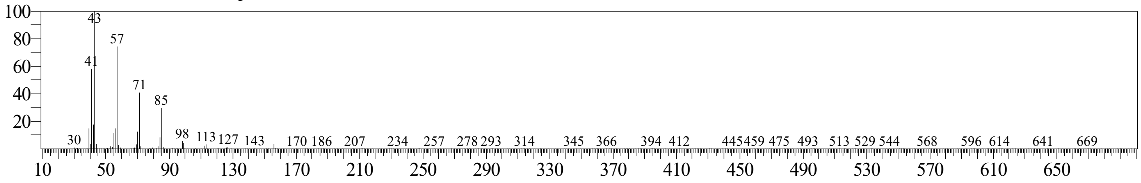

Hit#:1 Entry:26094 Library:NIST11.lib
SI:93 Formula:C12H26 CAS:1002-17-1 MolWeight:170 RetIndex:1086
CompName:Decane, 2,9-dimethyl- $$ 2,9-Dimethyldecane $$

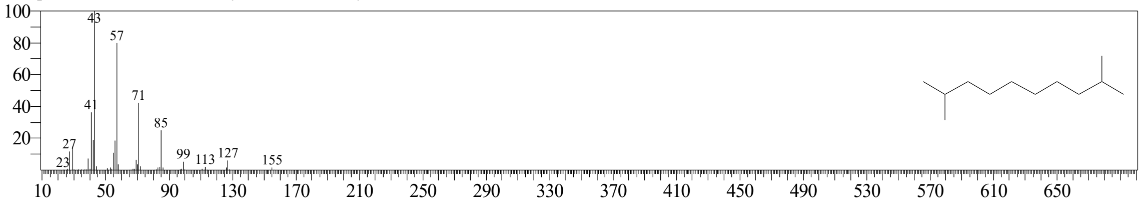

Hit#:2 Entry:18715 Library:NIST11.lib
SI:93 Formula:C11H24 CAS:1120-21-4 MolWeight:156 RetIndex:1115
CompName:Undecane $$ n-Undecane $$ Hendecane $$ n-C11H24 $$ UN 2330 $$

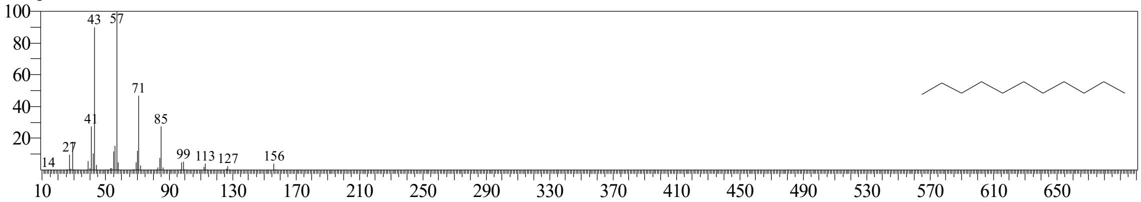

Hit#:3 Entry:12306 Library:NIST11.lib
SI:92 Formula:C10H22 CAS:871-83-0 MolWeight:142 RetIndex:951
CompName:Nonane, 2-methyl- $$ 2-Methylnonane $$

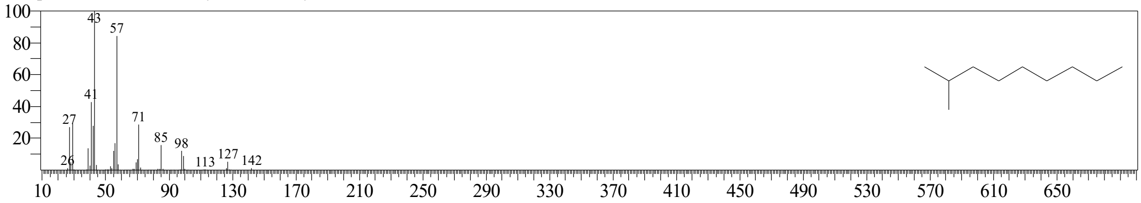

Hit#:4 Entry:34136 Library:NIST11.lib
SI:92 Formula:C13H28 CAS:17301-32-5 MolWeight:184 RetIndex:1185
CompName:Undecane, 4,7-dimethyl- $$ 4,7-Dimethylundecane # $$

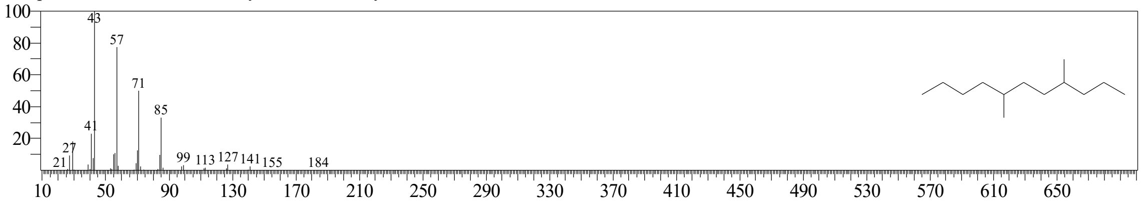

Hit#:5 Entry:26093 Library:NIST11.lib
SI:92 Formula:C12H26 CAS:2801-84-5 MolWeight:170 RetIndex:1086
CompName:Decane, 2,4-dimethyl- $$ 2,4-Dimethyldecane $$

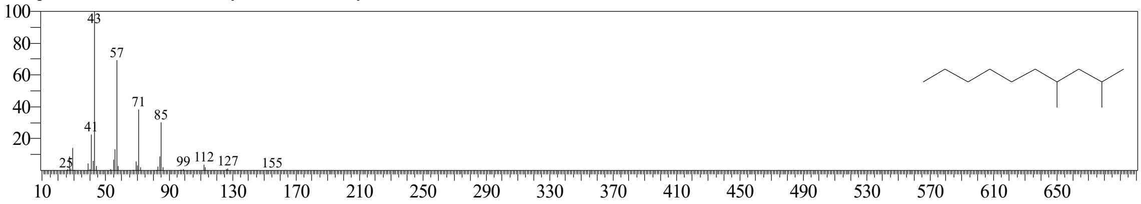

<< Target >>
Line#:31 R.Time:6.870(Scan#:575) MassPeaks:351
RawMode:Averaged 6.865-6.875(574-576) BasePeak:41.05(16077)
BG Mode:Calc. from Peak Group 1 - Event 1 Scan

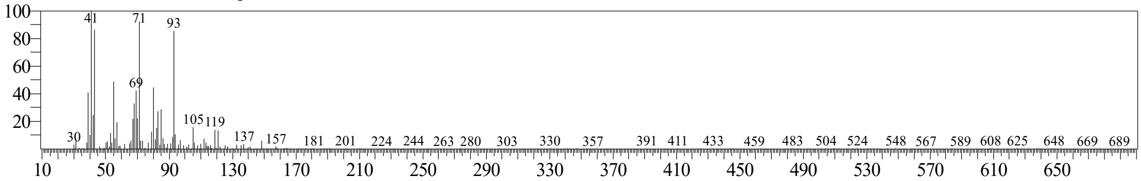

Hit#:1 Entry:17562 Library:NIST11.lib
SI:82 Formula:C10H18O CAS:78-70-6 MolWeight:154 RetIndex:1082
CompName:1,6-Octadien-3-ol, 3,7-dimethyl- $$ .beta.-Linalool $$ Linalol $$ Linalool $$ Linalyl alcohol $$ 2,6-Dimethyl-2,7-octadien-6-ol $$ allo-Ocimenol $

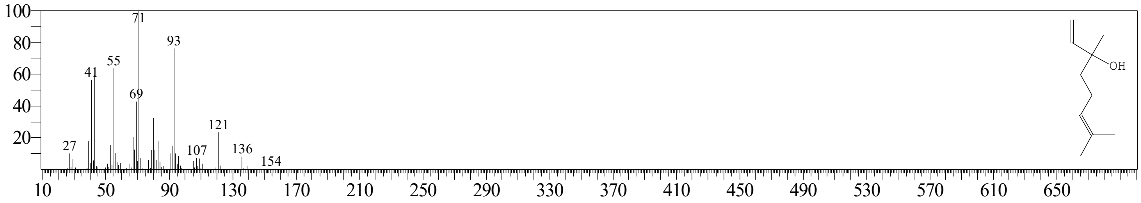

Hit#:2 Entry:97575 Library:NIST11.lib
SI:80 Formula:C17H23NO2 CAS:7149-26-0 MolWeight:273 RetIndex:2157
CompName:1,6-Octadien-3-ol, 3,7-dimethyl-, 2-aminobenzoate $$ Anthranilic acid, 1,5-dimethyl-1-vinyl-4-hexenyl ester $$ Linalyl anthranilate $$ Anthranilic a

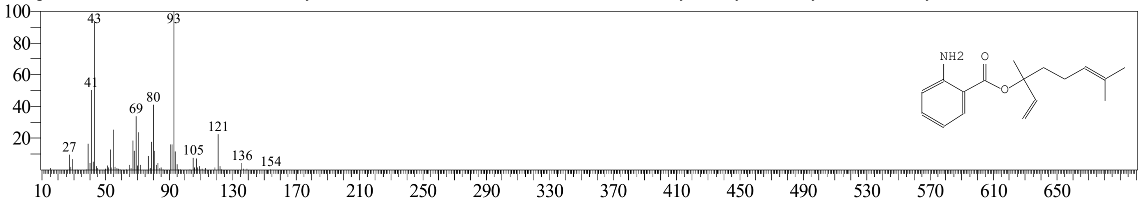

Hit#:3 Entry:60905 Library:NIST11.lib
SI:78 Formula:C15H28O CAS:2834-00-6 MolWeight:224 RetIndex:1772
CompName:2-Pentadecyn-1-ol

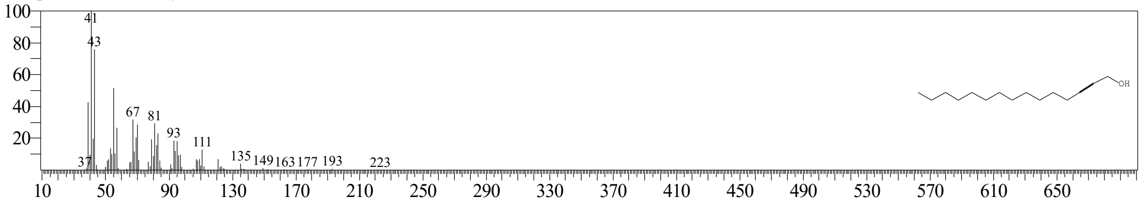

Hit#:4 Entry:17428 Library:NIST11.lib
SI:78 Formula:C10H18O CAS:2497-25-8 MolWeight:154 RetIndex:1212
CompName:2-Decenal, (Z)- $$ (Z)-2-Decenal $$ (2Z)-2-Decenal # $$ 2Z-Decenal $$ (Z)-Dec-2-enal $$

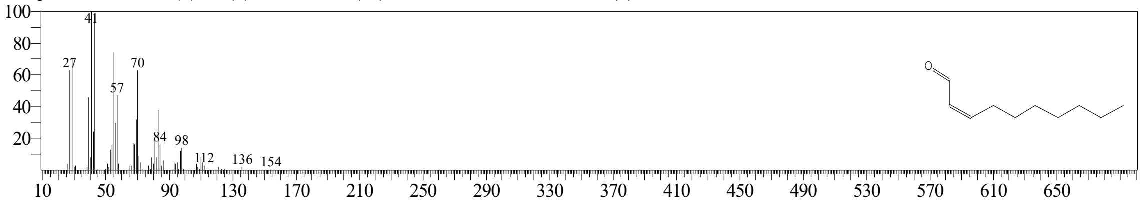

Hit#:5 Entry:19718 Library:NIST11.lib
SI:78 Formula:C7H13NO3 CAS:104313-51-1 MolWeight:159 RetIndex:1327
CompName:2-Nitrohept-2-en-1-ol $$ (2Z)-2-Nitro-2-hepten-1-ol # $$

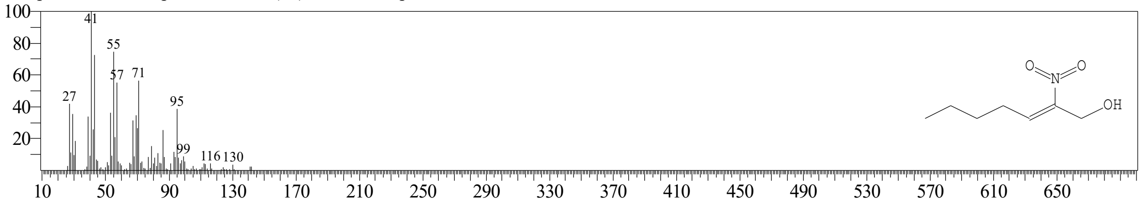

<< Target >>
Line#:32 R.Time:6.920(Scan#:585) MassPeaks:346
RawMode:Averaged 6.915-6.925(584-586) BasePeak:43.00(23071)
BG Mode:Calc. from Peak Group 1 - Event 1 Scan

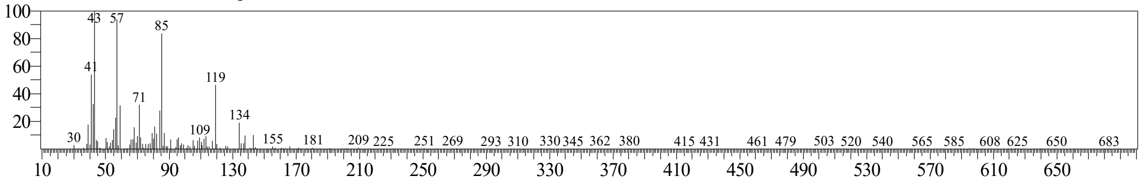

Hit#:1 Entry:62287 Library:NIST11.lib
SI:76 Formula:C14H26O2 CAS:0-00-0 MolWeight:226 RetIndex:1437
CompName:2-Octen-1-ol, 3,7-dimethyl-, isobutyrate, (Z)- $$ (2Z)-3,7-Dimethyl-2-octenyl 2-methylpropanoate # $$

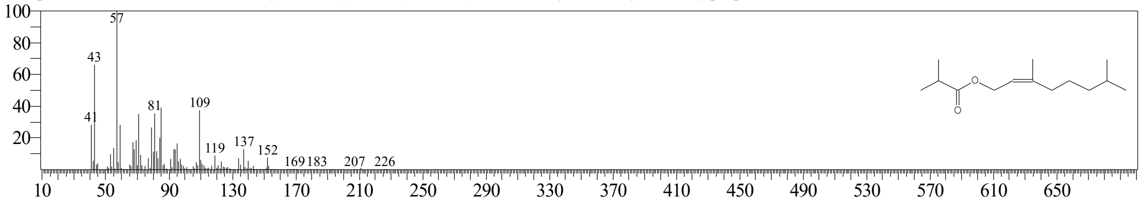

Hit#:2 Entry:18707 Library:NIST11.lib
SI:74 Formula:C11H24 CAS:62016-30-2 MolWeight:156 RetIndex:966
CompName:Octane, 2,3,3-trimethyl- $$ 2,3,3-Trimethyloctane # $$

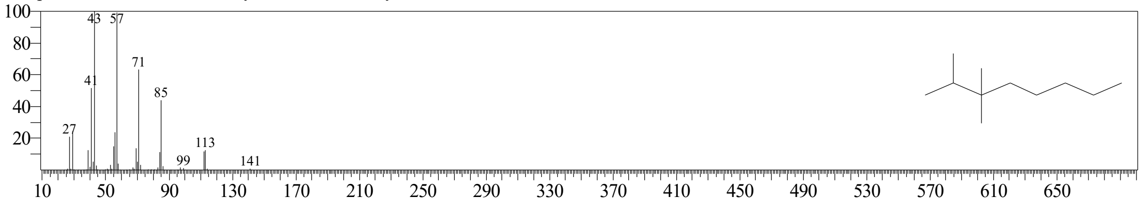

Hit#:3 Entry:100964 Library:NIST11.lib
SI:74 Formula:C14H30O3S CAS:0-00-0 MolWeight:278 RetIndex:2036
CompName:Sulfurous acid, hexyl octyl ester

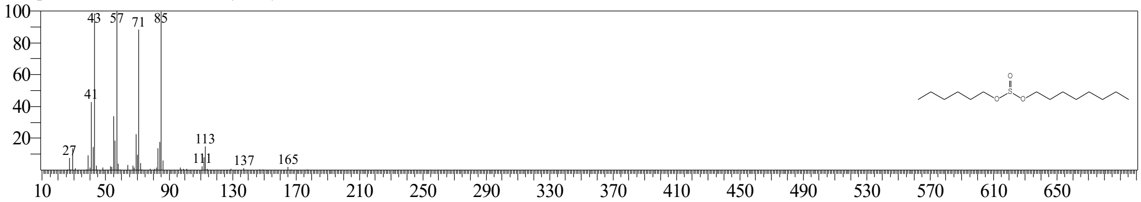

Hit#:4 Entry:53722 Library:NIST11.lib
SI:74 Formula:C14H30O CAS:17071-54-4 MolWeight:214 RetIndex:1489
CompName:Hexyl octyl ether $$ 1-(Hexyloxy)octane # $$

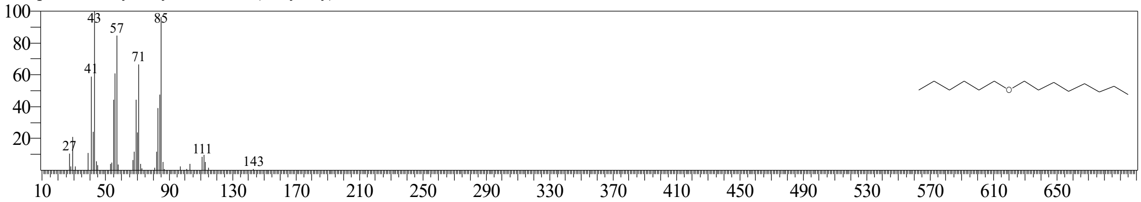

Hit#:5 Entry:18614 Library:NIST11.lib
SI:74 Formula:C10H20O CAS:18409-18-2 MolWeight:156 RetIndex:1266
CompName:2-Decen-1-ol, (E)- $$ trans-2-Decenol $$ Dec-2-en-1-ol $$ (2E)-2-Decen-1-ol $$ (E)-2-Decen-1-ol $$ trans-2-Decen-1-ol $$

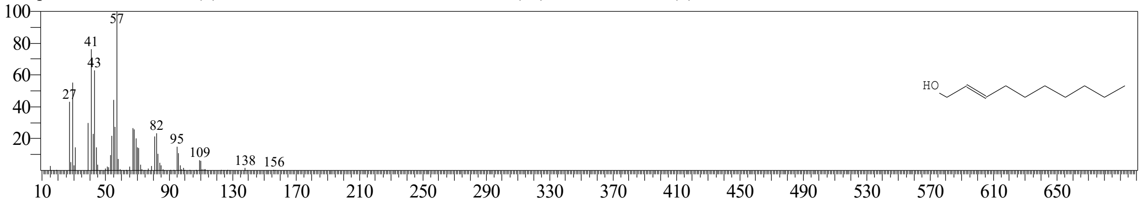

<< Target >>
Line#:33 R.Time:7.035(Scan#:608) MassPeaks:352
RawMode:Averaged 7.030-7.040(607-609) BasePeak:41.05(23996)
BG Mode:Calc. from Peak Group 1 - Event 1 Scan

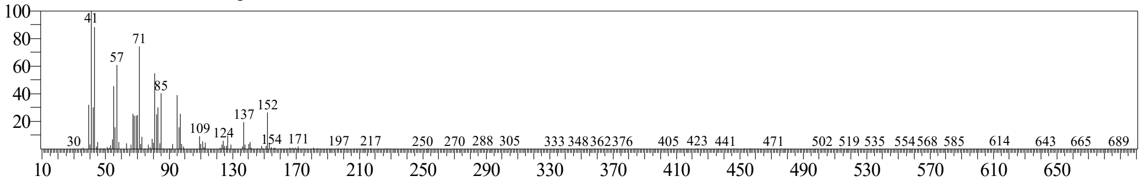

Hit#:1 Entry:52414 Library:NIST11.lib
SI:86 Formula:C14H28O CAS:3234-28-4 MolWeight:212 RetIndex:1503
CompName:Oxirane, dodecyl- $$ 1,2-Epoxytetradecane $$ 2-Dodecyloxirane # $$

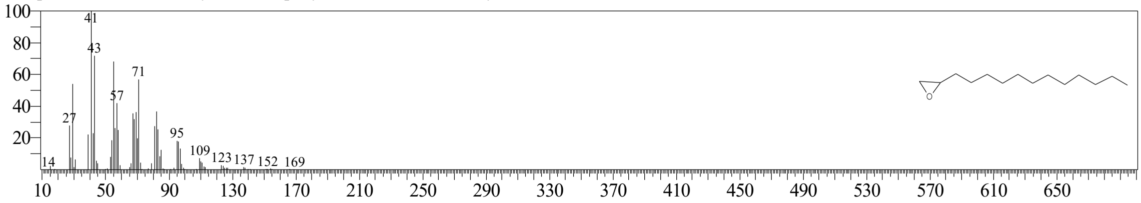

Hit#:2 Entry:72460 Library:NIST11.lib
SI:84 Formula:C16H32O CAS:7320-37-8 MolWeight:240 RetIndex:1702
CompName:Oxirane, tetradecyl- $$ Hexadecane, 1,2-epoxy- $$ Hexadecylene oxide $$ 1,2-Epoxyhexadecane $$ 1,2-Hexadecane oxide $$ 1,2-Hexadecene epox

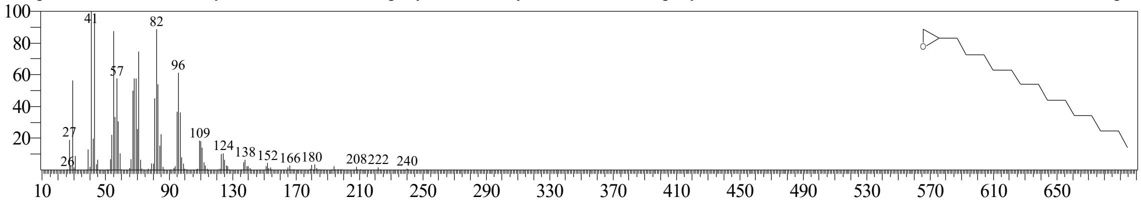

Hit#:3 Entry:42882 Library:NIST11.lib
SI:84 Formula:C13H26O CAS:74962-98-4 MolWeight:198 RetIndex:1564
CompName:2-Tridecen-1-ol, (E)- $$ trans-2-Tridecen-1-ol $$ (2E)-2-Tridecen-1-ol $$ E-2-Tridecen-1-ol $$

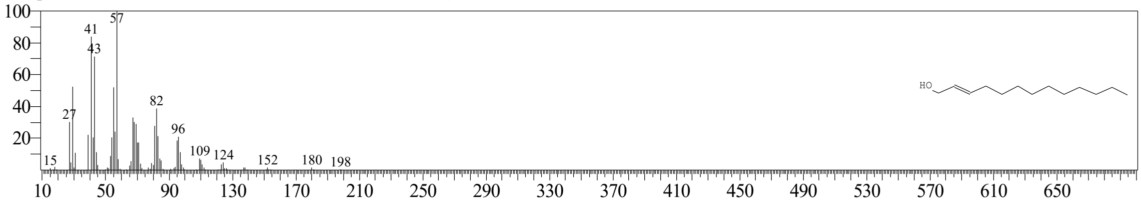

Hit#:4 Entry:62419 Library:NIST11.lib
SI:84 Formula:C15H30O CAS:18633-25-5 MolWeight:226 RetIndex:1603
CompName:Oxirane, tridecyl- $$ 2-Tridecyloxirane # $$

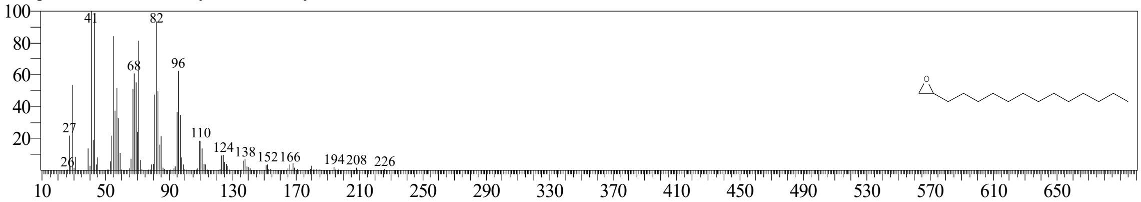

Hit#:5 Entry:93586 Library:NIST11.lib
SI:84 Formula:C18H36O CAS:638-66-4 MolWeight:268 RetIndex:1999
CompName:Octadecanal $$ Stearaldehyde $$ Octadecyl aldehyde $$ Stearyl aldehyde $$ n-Octadecanal $$

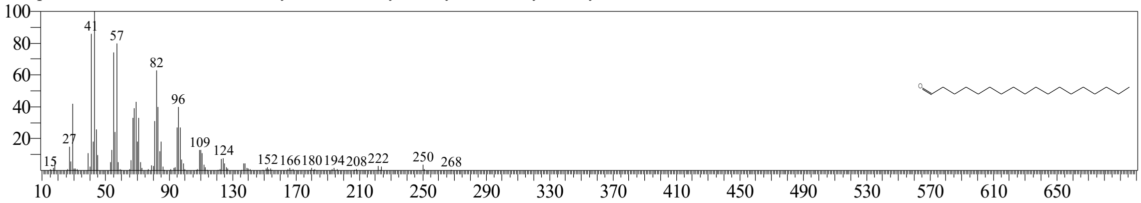

<< Target >>
Line#:34 R.Time:7.115(Scan#:624) MassPeaks:340
RawMode:Averaged 7.110-7.120(623-625) BasePeak:119.10(25409)
BG Mode:Calc. from Peak Group 1 - Event 1 Scan

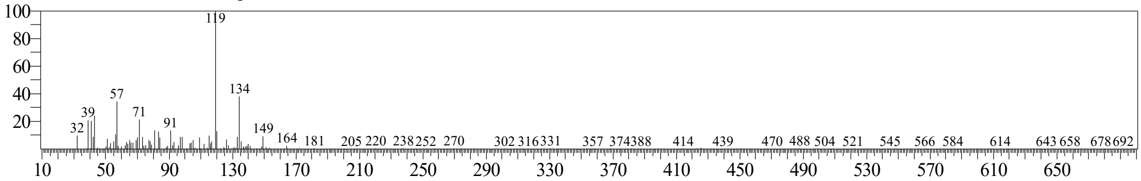

Hit#:1 Entry:29273 Library:NIST11.lib
SI:73 Formula:C12H16O CAS:91531-36-1 MolWeight:176 RetIndex:1354
CompName:Spiro[3.5]nona-5,7-dien-1-one, 5,9,9-trimethyl- $$ 5,9,9-Trimethylspiro[3.5]nona-5,7-dien-1-one # $$

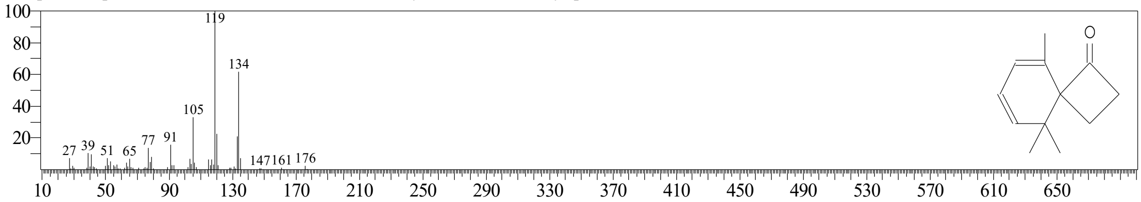

Hit#:2 Entry:9118 Library:NIST11.lib
SI:71 Formula:C10H14 CAS:99-87-6 MolWeight:134 RetIndex:1042
CompName:p-Cymene $$ Benzene, 1-methyl-4-(1-methylethyl)- $$ p-Cimene $$ p-Cymol $$ p-Isopropyltoluene $$ p-Methylisopropylbenzene $$ Camphogen $

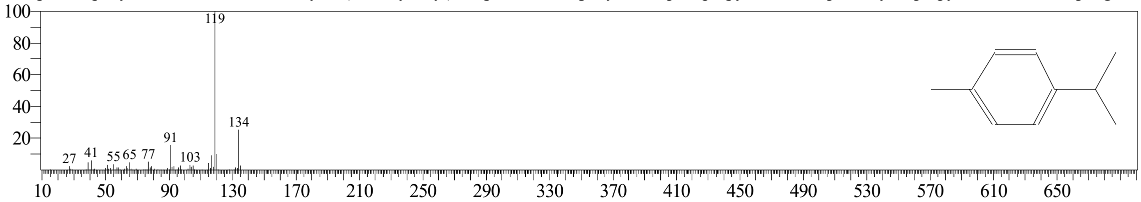

Hit#:3 Entry:21454 Library:NIST11.lib
SI:69 Formula:C12H18 CAS:130930-56-2 MolWeight:162 RetIndex:1111
CompName:1,7,7-Trimethyl-2-vinylbicyclo[2.2.1]hept-2-ene

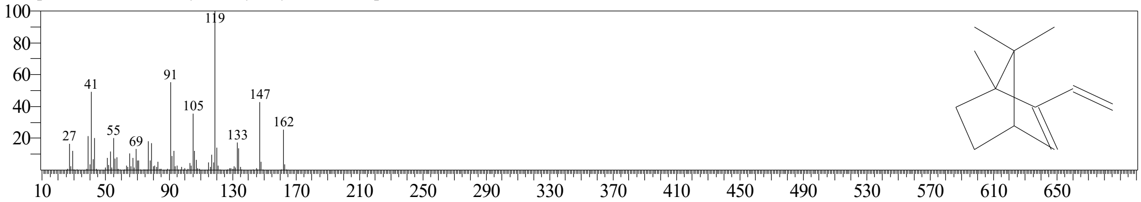

Hit#:4 Entry:25970 Library:NIST11.lib
SI:69 Formula:C10H18O2 CAS:61465-23-4 MolWeight:170 RetIndex:1308
CompName:(+-)-trans-1-Isopropenyl-4-methyl-1,4-cyclohexanediol $$ 1-Isopropenyl-4-methyl-1,4-cyclohexanediol # $$

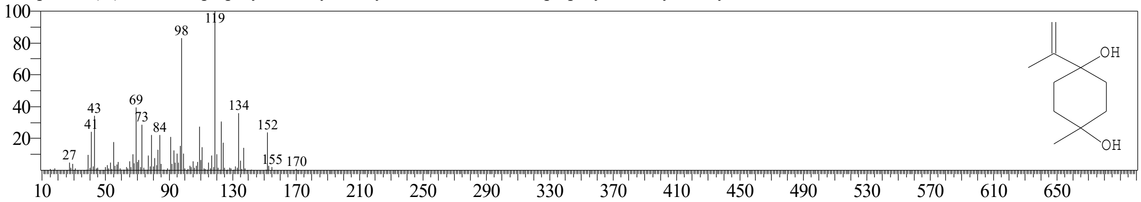

Hit#:5 Entry:38761 Library:NIST11.lib
SI:69 Formula:C13H20O CAS:0-00-0 MolWeight:192 RetIndex:1344
CompName:(+)-3-Carene, 2-(acetylmethyl)- $$ 1-(3,7,7-Trimethylbicyclo[4.1.0]hept-3-en-2-yl)acetone # $$

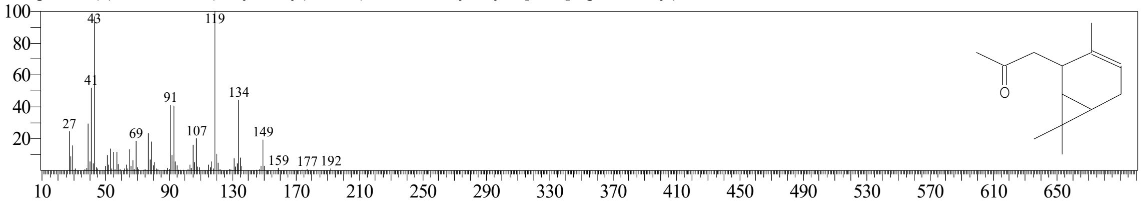

<< Target >>
Line#:35 R.Time:7.210(Scan#:643) MassPeaks:356
RawMode:Averaged 7.205-7.215(642-644) BasePeak:73.05(30595)
BG Mode:Calc. from Peak Group 1 - Event 1 Scan

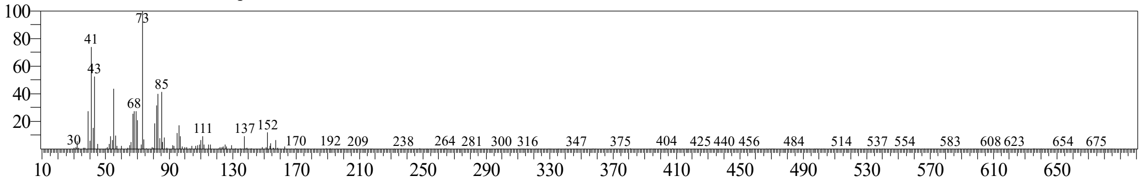

Hit#:1 Entry:26041 Library:NIST11.lib
SI:78 Formula:C11H22O CAS:75039-84-8 MolWeight:170 RetIndex:1365
CompName:trans-2-Undecen-1-ol $$ (2E)-2-Undecen-1-ol $$ (E)-2-Undecen-1-ol $$ trans-Undec-2-en-1-ol $$

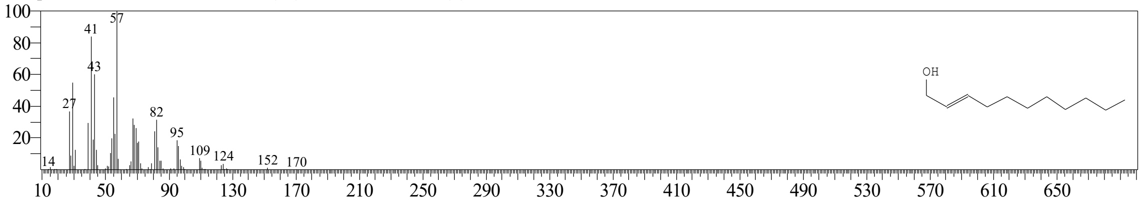

Hit#:2 Entry:33864 Library:NIST11.lib
SI:78 Formula:C11H20O2 CAS:4189-02-0 MolWeight:184 RetIndex:1479
CompName:2-Undecenoic acid $$ Undec-2-enoic acid $$

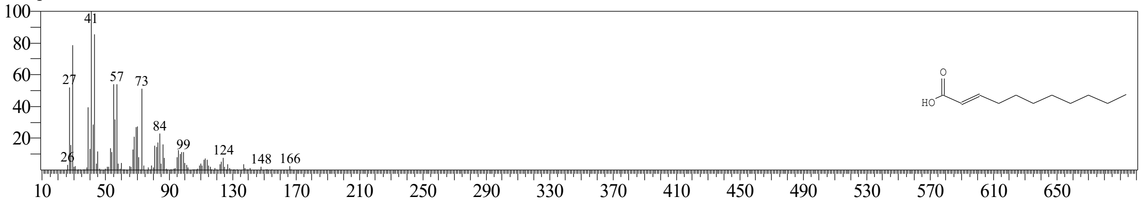

Hit#:3 Entry:25775 Library:NIST11.lib
SI:78 Formula:C10H18O2 CAS:334-49-6 MolWeight:170 RetIndex:1380
CompName:trans-2-Decenoic acid $$ 2-Decenoic acid, (E)- $$ E-2-Decenoic acid $$

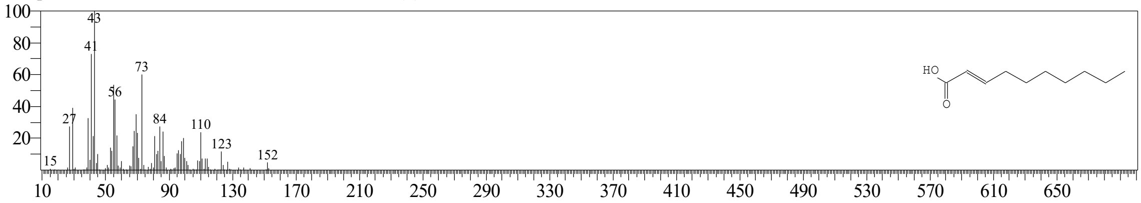

Hit#:4 Entry:33865 Library:NIST11.lib
SI:77 Formula:C11H20O2 CAS:15790-94-0 MolWeight:184 RetIndex:1479
CompName:trans-2-undecenoic acid $$ (2E)-2-Undecenoic acid # $$

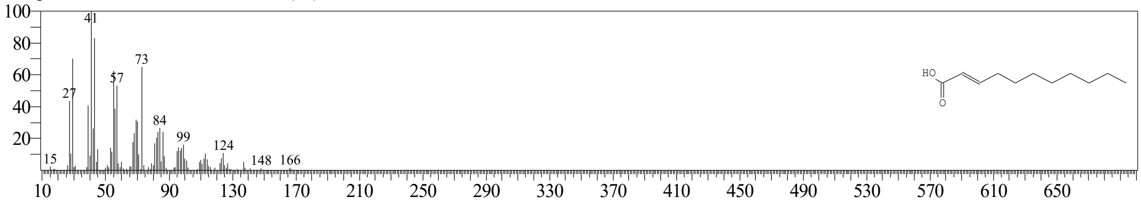

Hit#:5 Entry:72139 Library:NIST11.lib
SI:77 Formula:C14H24O3 CAS:74685-36-2 MolWeight:240 RetIndex:2137
CompName:Oxacyclotetradecane-2,11-dione, 13-methyl- $$ 13-Methyloxacyclotetradecane-2,11-dione # $$

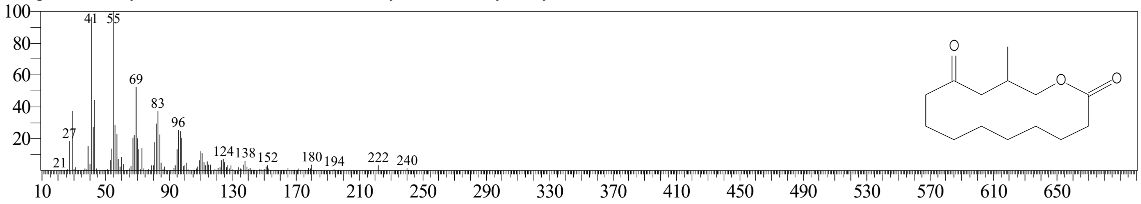

<< Target >>
Line#:36 R.Time:7.325(Scan#:666) MassPeaks:349
RawMode:Averaged 7.320-7.330(665-667) BasePeak:43.05(49292)
BG Mode:Calc. from Peak Group 1 - Event 1 Scan

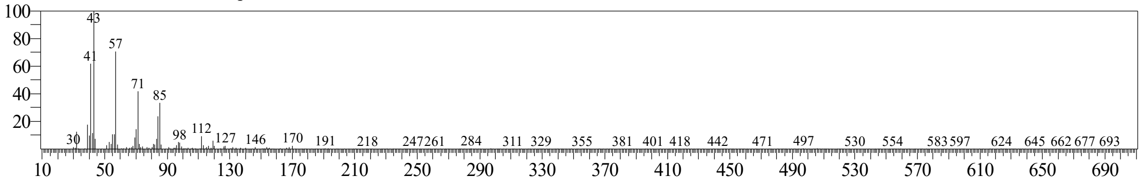

Hit#:1 Entry:24840 Library:NIST11.lib
SI:88 Formula:C12H24 CAS:74630-39-0 MolWeight:168 RetIndex:1140
CompName:1-Undecene, 4-methyl- $$ 4-Methyl-1-undecene # $$

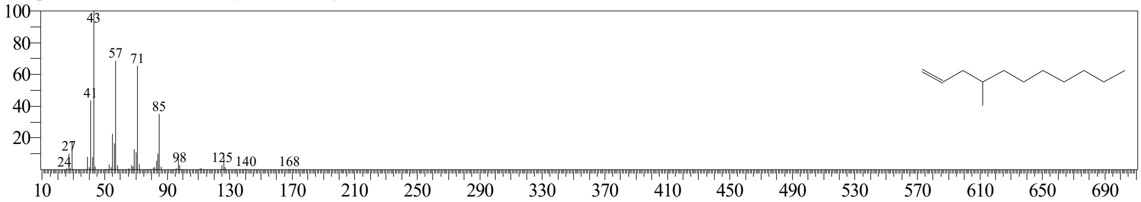

Hit#:2 Entry:26110 Library:NIST11.lib
SI:87 Formula:C12H26 CAS:62185-21-1 MolWeight:170 RetIndex:958
CompName:Octane, 3,4,5,6-tetramethyl- $$ 3,4,5,6-Tetramethyloctane # $$

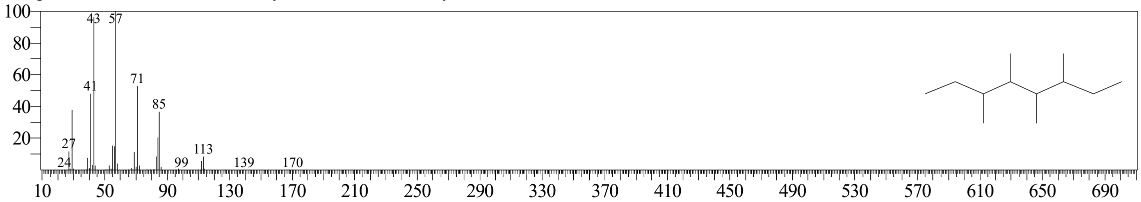

Hit#:3 Entry:26099 Library:NIST11.lib
SI:87 Formula:C12H26 CAS:17312-44-6 MolWeight:170 RetIndex:1086
CompName:2,3-Dimethyldecane $$ Decane, 2,3-dimethyl $$

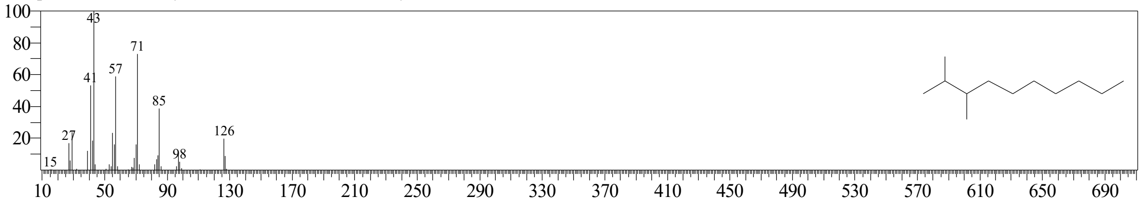

Hit#:4 Entry:26095 Library:NIST11.lib
SI:87 Formula:C12H26 CAS:1632-70-8 MolWeight:170 RetIndex:1150
CompName:Undecane, 5-methyl- $$ 5-Methylundecane $$ Methylundecane $$

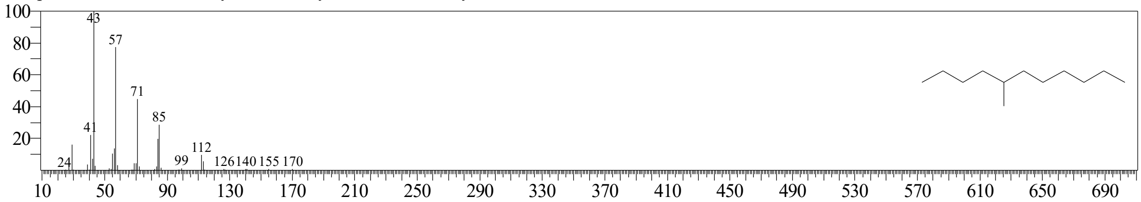

Hit#:5 Entry:18717 Library:NIST11.lib
SI:87 Formula:C11H24 CAS:6975-98-0 MolWeight:156 RetIndex:1051
CompName:Decane, 2-methyl- $$ 2-Methyldecane $$ n-C8H17CH(CH3)2 $$

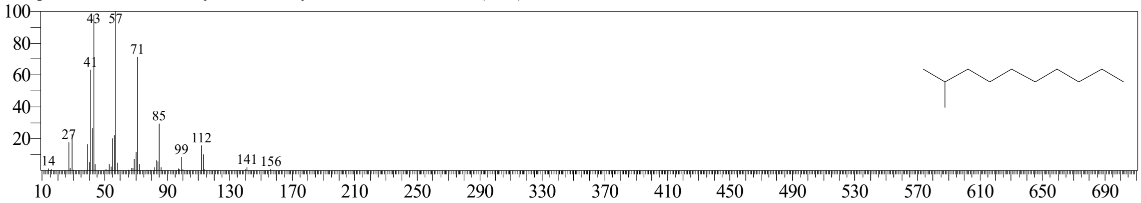

<< Target >>
Line#:37 R.Time:7.370(Scan#:675) MassPeaks:373
RawMode:Averaged 7.365-7.375(674-676) BasePeak:43.05(35278)
BG Mode:Calc. from Peak Group 1 - Event 1 Scan

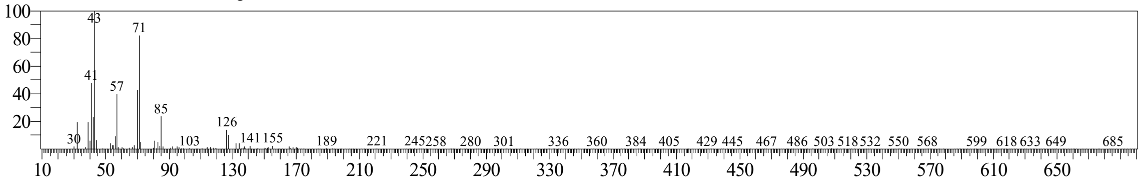

Hit#:1 Entry:26097 Library:NIST11.lib
SI:88 Formula:C12H26 CAS:2980-69-0 MolWeight:170 RetIndex:1150
CompName:Undecane, 4-methyl- $$ 4-Methylundecane $$

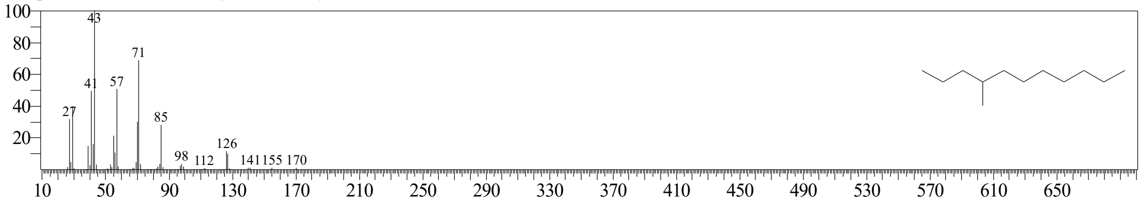

Hit#:2 Entry:26098 Library:NIST11.lib
SI:86 Formula:C12H26 CAS:52670-34-5 MolWeight:170 RetIndex:958
CompName:Octane, 2,3,6,7-tetramethyl- $$ 2,3,6,7-Tetramethyloctane # $$

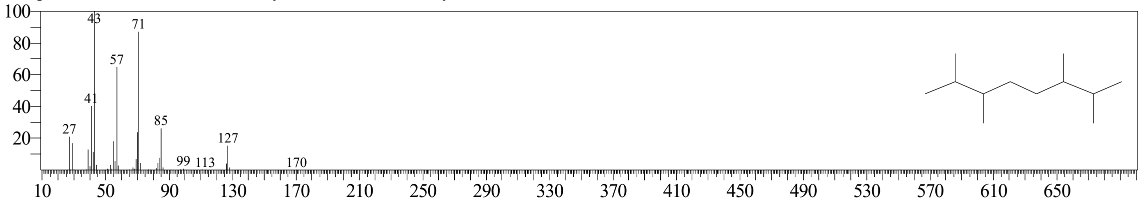

Hit#:3 Entry:26099 Library:NIST11.lib
SI:85 Formula:C12H26 CAS:17312-44-6 MolWeight:170 RetIndex:1086
CompName:2,3-Dimethyldecane $$ Decane, 2,3-dimethyl $$

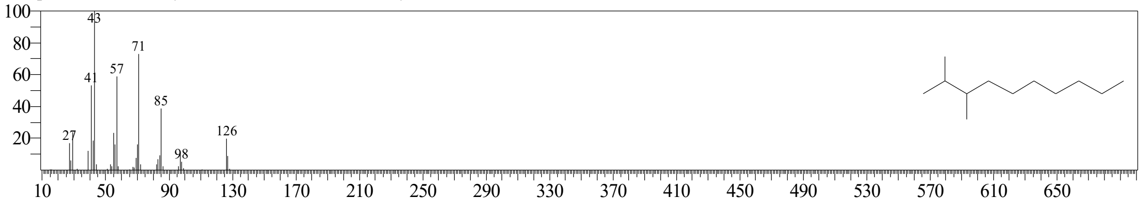

Hit#:4 Entry:18711 Library:NIST11.lib
SI:84 Formula:C11H24 CAS:2847-72-5 MolWeight:156 RetIndex:1051
CompName:Decane, 4-methyl- $$ 4-Methyldecane $$

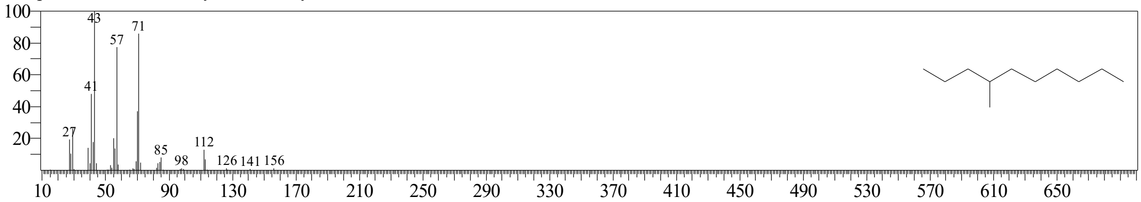

Hit#:5 Entry:18710 Library:NIST11.lib
SI:84 Formula:C11H24 CAS:62016-19-7 MolWeight:156 RetIndex:986
CompName:Octane, 6-ethyl-2-methyl- $$ 6-Ethyl-2-methyloctane # $$

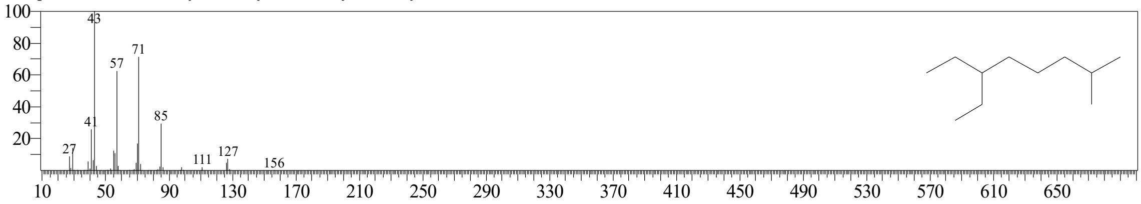

<< Target >>
Line#:38 R.Time:7.425(Scan#:686) MassPeaks:372
RawMode:Averaged 7.420-7.430(685-687) BasePeak:43.05(81742)
BG Mode:Calc. from Peak Group 1 - Event 1 Scan

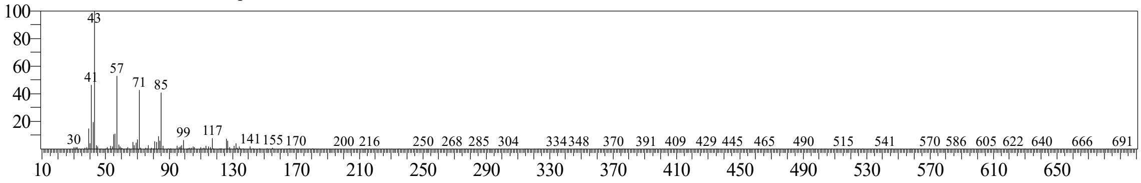

Hit#:1 Entry:34147 Library:NIST11.lib
SI:89 Formula:C13H28 CAS:17312-80-0 MolWeight:184 RetIndex:1185
CompName:Undecane, 2,4-dimethyl- $$ 2,4-Dimethylundecane $$

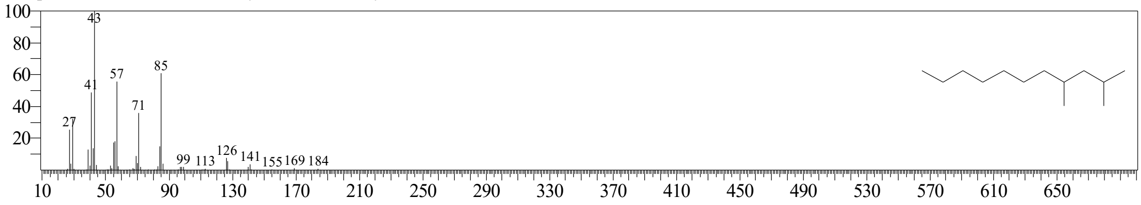

Hit#:2 Entry:92779 Library:NIST11.lib
SI:89 Formula:C10H21I CAS:0-00-0 MolWeight:268 RetIndex:1365
CompName:1-Iodo-2-methylnonane

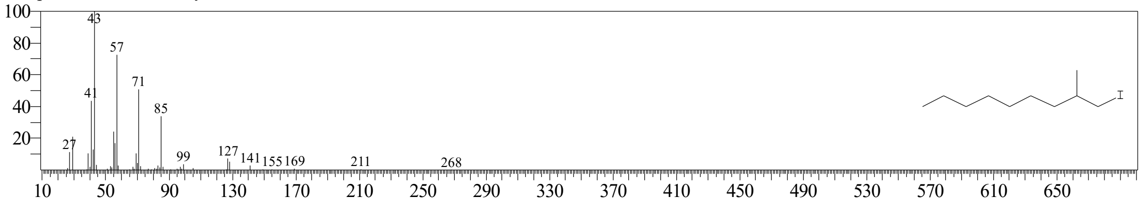

Hit#:3 Entry:26097 Library:NIST11.lib
SI:88 Formula:C12H26 CAS:2980-69-0 MolWeight:170 RetIndex:1150
CompName:Undecane, 4-methyl- $$ 4-Methylundecane $$

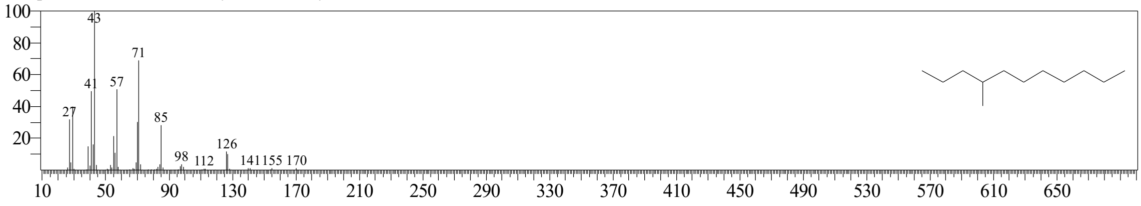

Hit#:4 Entry:26096 Library:NIST11.lib
SI:88 Formula:C12H26 CAS:1636-44-8 MolWeight:170 RetIndex:1150
CompName:Decane, 4-ethyl- $$ 4-Ethyldecane # $$

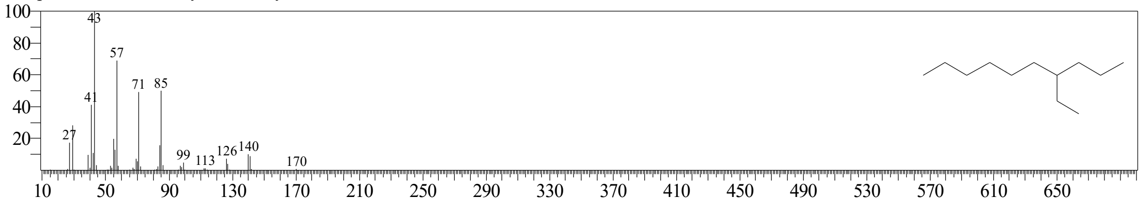

Hit#:5 Entry:26099 Library:NIST11.lib
SI:88 Formula:C12H26 CAS:17312-44-6 MolWeight:170 RetIndex:1086
CompName:2,3-Dimethyldecane $$ Decane, 2,3-dimethyl $$

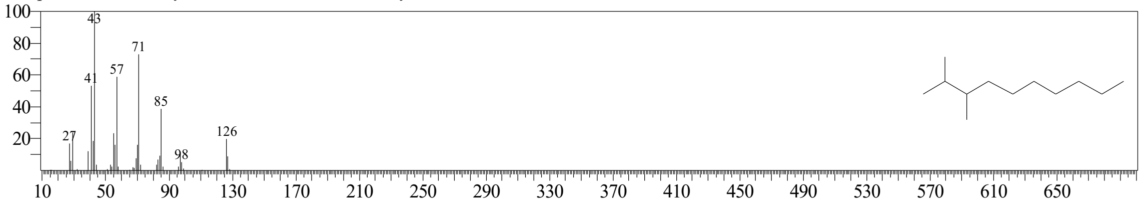

<< Target >>
Line#:39 R.Time:7.495(Scan#:700) MassPeaks:394
RawMode:Averaged 7.490-7.500(699-701) BasePeak:43.10(44812)
BG Mode:Calc. from Peak Group 1 - Event 1 Scan

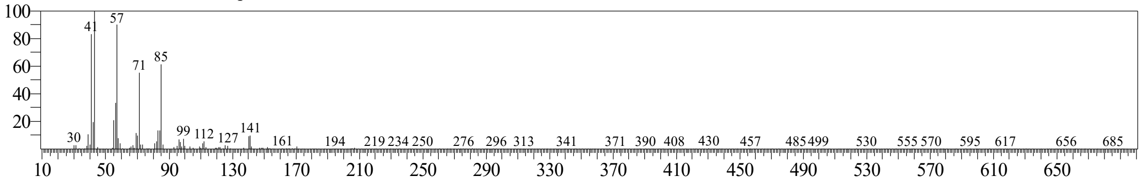

Hit#:1 Entry:62481 Library:NIST11.lib
SI:89 Formula:C16H34 CAS:55045-09-5 MolWeight:226 RetIndex:1548
CompName:Tridecane, 7-propyl- $$ 7-n-Propyltridecane $$ 7-Propyltridecane # $$

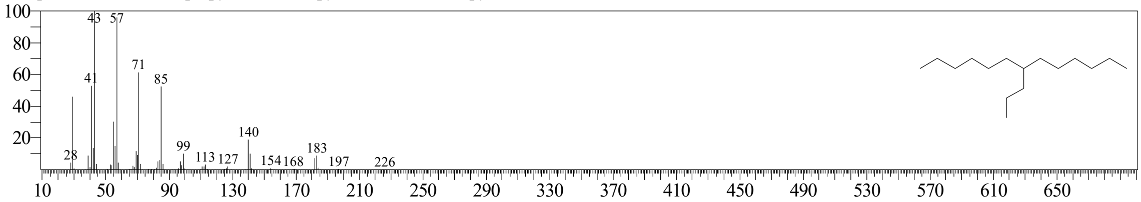

Hit#:2 Entry:34130 Library:NIST11.lib
SI:88 Formula:C13H28 CAS:17312-77-5 MolWeight:184 RetIndex:1185
CompName:Undecane, 2,3-dimethyl- $$ 2,3-Dimethylundecane $$

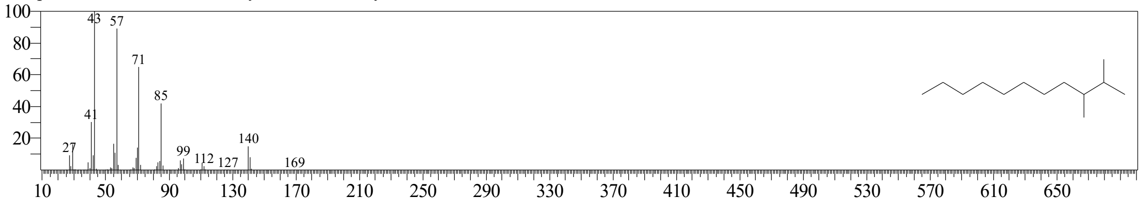

Hit#:3 Entry:26096 Library:NIST11.lib
SI:88 Formula:C12H26 CAS:1636-44-8 MolWeight:170 RetIndex:1150
CompName:Decane, 4-ethyl- $$ 4-Ethyldecane # $$

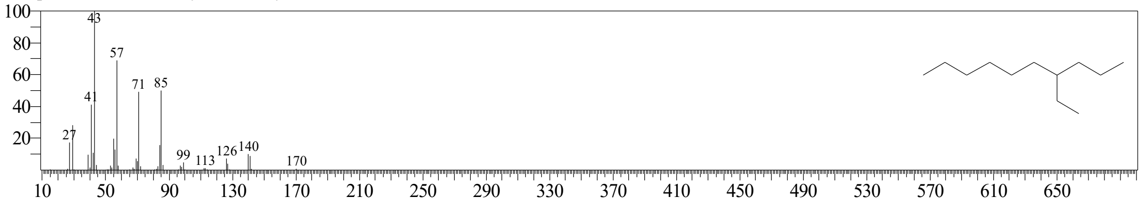

Hit#:4 Entry:34133 Library:NIST11.lib
SI:87 Formula:C13H28 CAS:17301-27-8 MolWeight:184 RetIndex:1185
CompName:Undecane, 2,10-dimethyl- $$ 2,10-Dimethylundecane # $$

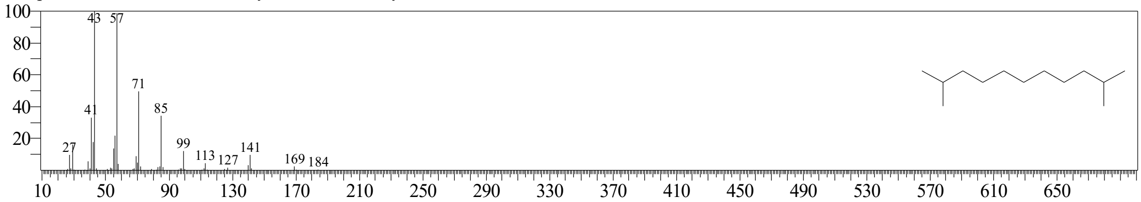

Hit#:5 Entry:82867 Library:NIST11.lib
SI:87 Formula:C18H38 CAS:26741-18-4 MolWeight:254 RetIndex:1746
CompName:9-methylheptadecane

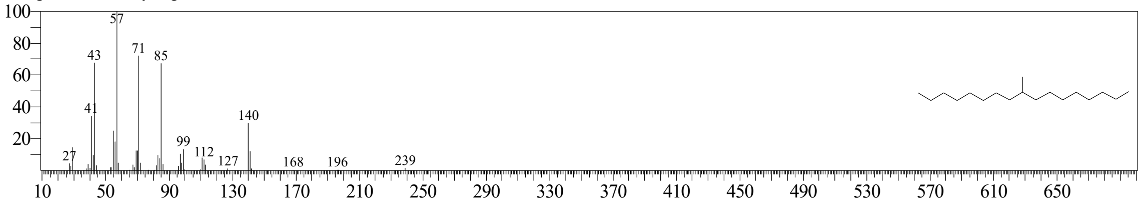

<< Target >>
Line#:40 R.Time:7.615(Scan#:724) MassPeaks:378
RawMode:Averaged 7.610-7.620(723-725) BasePeak:43.05(20098)
BG Mode:Calc. from Peak Group 1 - Event 1 Scan

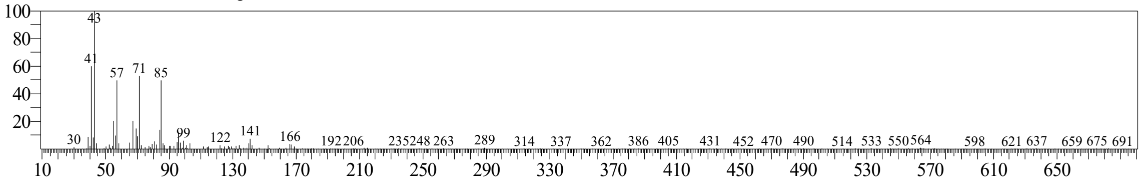

Hit#:1 Entry:26096 Library:NIST11.lib
SI:87 Formula:C12H26 CAS:1636-44-8 MolWeight:170 RetIndex:1150
CompName:Decane, 4-ethyl- $$ 4-Ethyldecane # $$

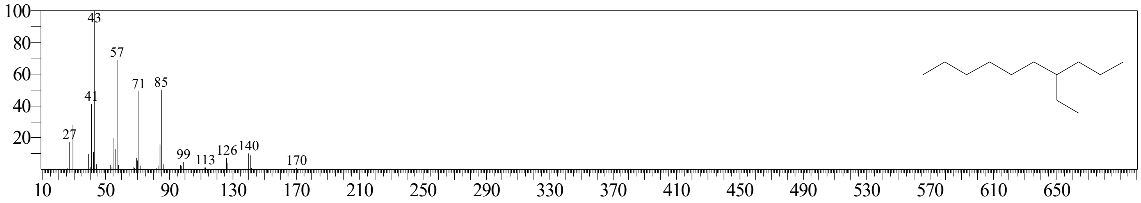

Hit#:2 Entry:34145 Library:NIST11.lib
SI:86 Formula:C13H28 CAS:17301-33-6 MolWeight:184 RetIndex:1185
CompName:Undecane, 4,8-dimethyl- $$ 4,8-Dimethylundecane # $$

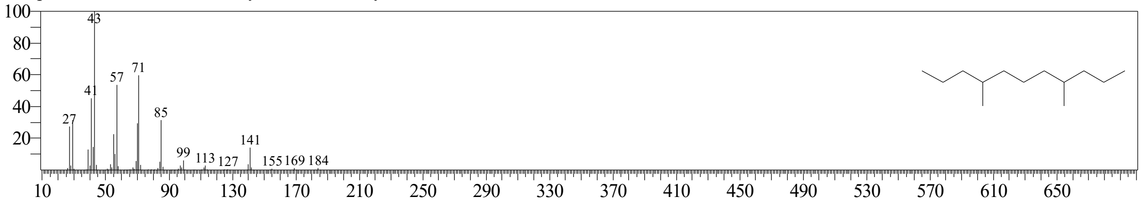

Hit#:3 Entry:34147 Library:NIST11.lib
SI:86 Formula:C13H28 CAS:17312-80-0 MolWeight:184 RetIndex:1185
CompName:Undecane, 2,4-dimethyl- $$ 2,4-Dimethylundecane $$

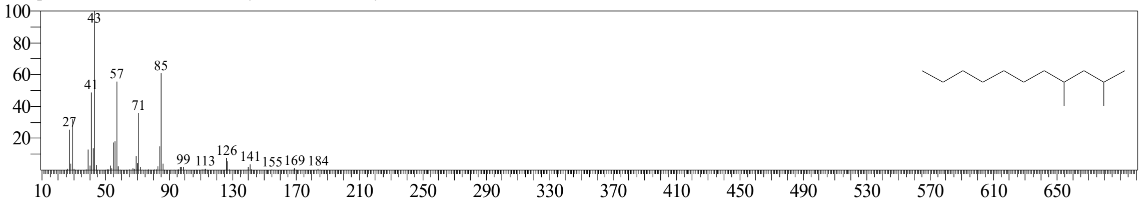

Hit#:4 Entry:92779 Library:NIST11.lib
SI:85 Formula:C10H21I CAS:0-00-0 MolWeight:268 RetIndex:1365
CompName:1-Iodo-2-methylnonane

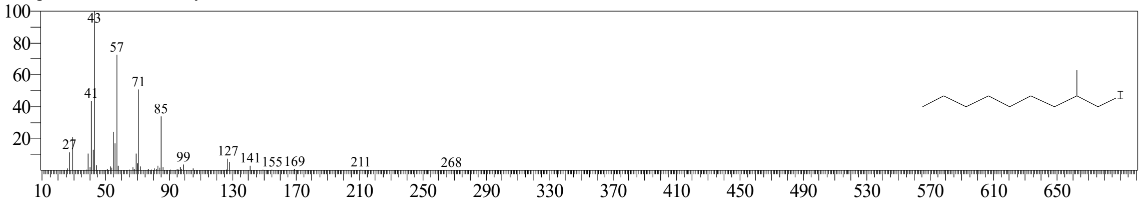

Hit#:5 Entry:24840 Library:NIST11.lib
SI:85 Formula:C12H24 CAS:74630-39-0 MolWeight:168 RetIndex:1140
CompName:1-Undecene, 4-methyl- $$ 4-Methyl-1-undecene # $$

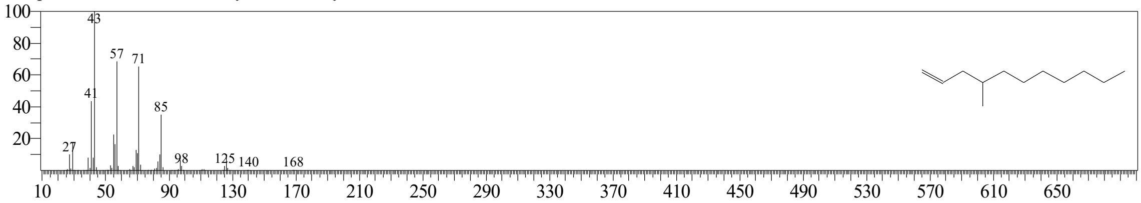

<< Target >>
Line#:41 R.Time:7.640(Scan#:729) MassPeaks:374
RawMode:Averaged 7.635-7.645(728-730) BasePeak:83.05(6138)
BG Mode:Calc. from Peak Group 1 - Event 1 Scan

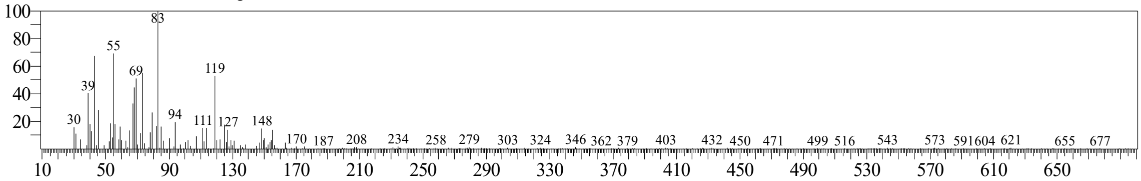

Hit#:1 Entry:17582 Library:NIST11.lib
SI:64 Formula:C10H18O CAS:0-00-0 MolWeight:154 RetIndex:1233
CompName:2,4-Pentadien-1-ol, 3-pentyl-, (2Z)- $$ (2E)-3-Pentyl-2,4-pentadien-1-ol # $$

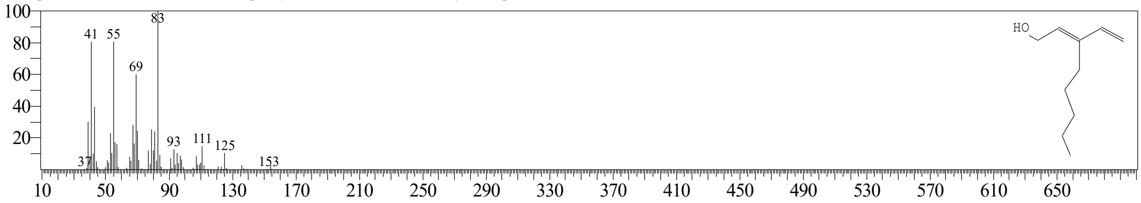

Hit#:2 Entry:20042 Library:NIST11.lib
SI:64 Formula:C7H12O4 CAS:623-82-5 MolWeight:160 RetIndex:1366
CompName:(R)-(+)-3-Methyladipic acid $$ (+)-3-Methyladipic acid $$ Hexanedioic acid, 3-methyl-, (R)- $$ 3-Methylhexanedioic acid # $$

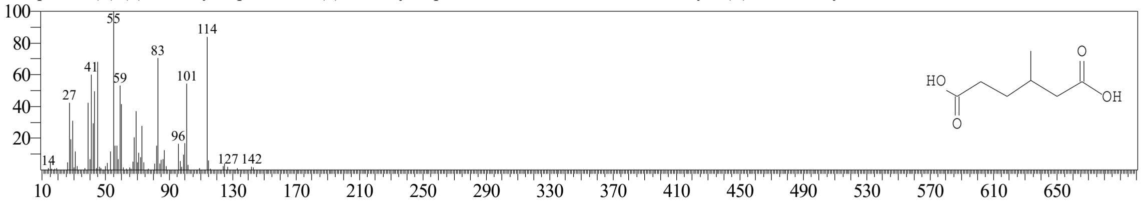

Hit#:3 Entry:7106 Library:NIST11.lib
SI:63 Formula:C7H12O2 CAS:1119-60-4 MolWeight:128 RetIndex:1064
CompName:6-Heptenoic acid

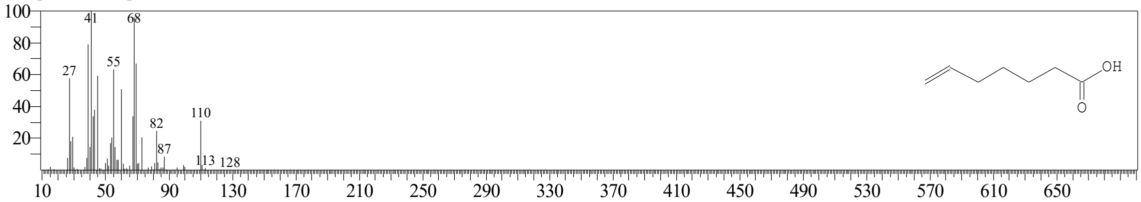

Hit#:4 Entry:12116 Library:NIST11.lib
SI:63 Formula:C8H14O2 CAS:34998-29-3 MolWeight:142 RetIndex:887
CompName:4-Pentenoic acid, 2,4-dimethyl-, methyl ester $$ Methyl 2,4-dimethyl-4-pentenoate # $$

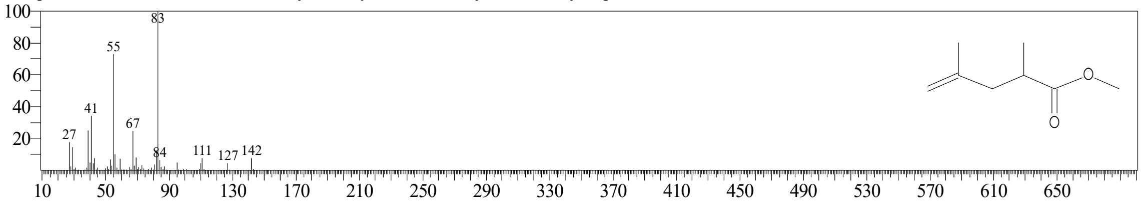

Hit#:5 Entry:82667 Library:NIST11.lib
SI:62 Formula:C16H30O2 CAS:6053-49-2 MolWeight:254 RetIndex:2011
CompName:Cyclopentaneundecanoic acid $$ 11-Cyclopentylundecanoic acid # $$

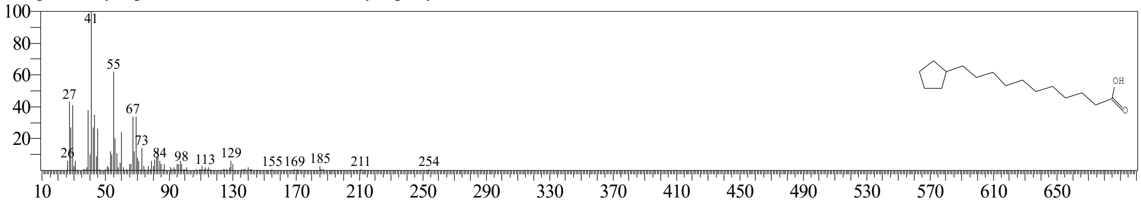

<< Target >>
Line#:42 R.Time:7.695(Scan#:740) MassPeaks:337
RawMode:Averaged 7.690-7.700(739-741) BasePeak:83.10(52628)
BG Mode:Calc. from Peak Group 1 - Event 1 Scan

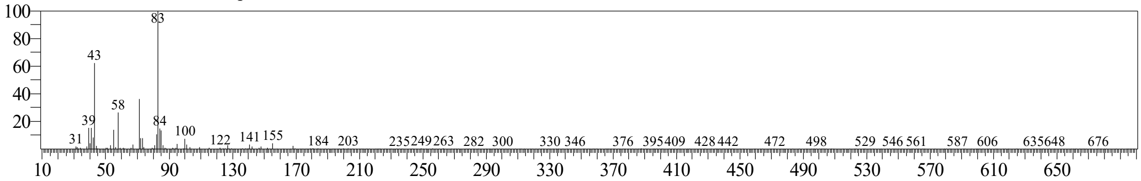

Hit#:1 Entry:33780 Library:NIST11.lib
SI:79 Formula:C10H16O3 CAS:0-00-0 MolWeight:184 RetIndex:1299
CompName:3-Methyl-2-butenoic acid, 2-tetrahydrofurylmethyl ester $$ Tetrahydro-2-furanylmethyl 3-methyl-2-butenoate # $$

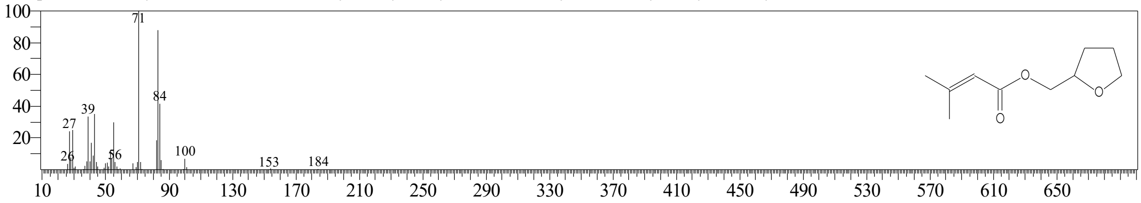

Hit#:2 Entry:25904 Library:NIST11.lib
SI:77 Formula:C10H18O2 CAS:150462-84-3 MolWeight:170 RetIndex:1104
CompName:3-Methyl-2-butenoic acid, 2-pentyl ester $$ 1-Methylbutyl 3-methyl-2-butenoate # $$

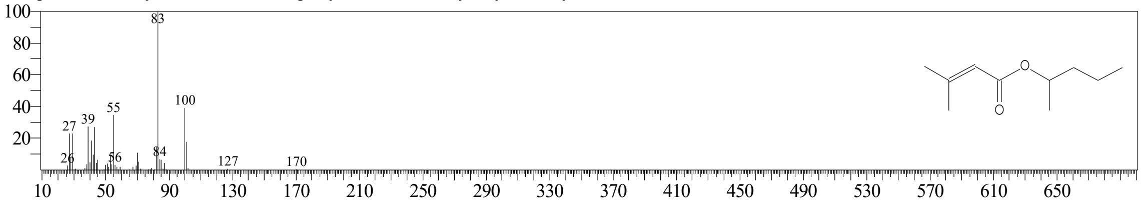

Hit#:3 Entry:12118 Library:NIST11.lib
SI:76 Formula:C8H14O2 CAS:61692-76-0 MolWeight:142 RetIndex:905
CompName:2-Butenoic acid, 2-methyl-, 1-methylethyl ester, (Z)- $$ Isopropyl (2Z)-2-methyl-2-butenoate $$

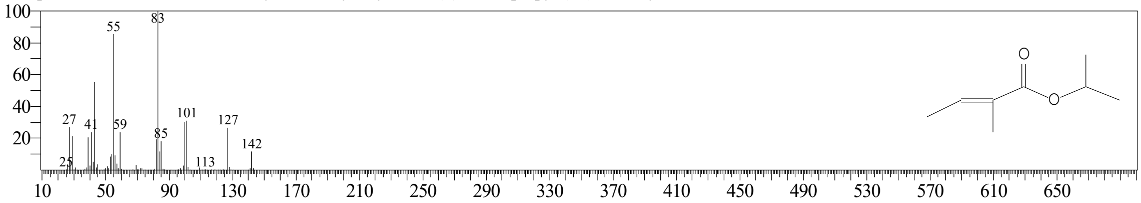

Hit#:4 Entry:12119 Library:NIST11.lib
SI:76 Formula:C8H14O2 CAS:1733-25-1 MolWeight:142 RetIndex:905
CompName:Isopropyl tiglate $$ 2-Butenoic acid, 2-methyl-, 1-methylethyl ester, (E)- $$ 2-Butenoic acid, 2-methyl, 1-isopropyl ester (E)- $$ Isopropyl 2-methyl

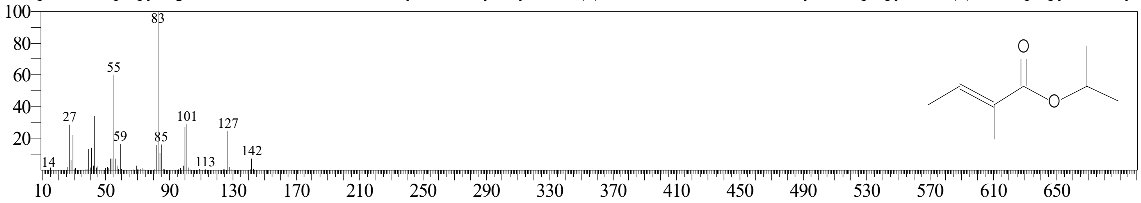

Hit#:5 Entry:27105 Library:NIST11.lib
SI:76 Formula:C10H20O2 CAS:0-00-0 MolWeight:172 RetIndex:1317
CompName:1,1-Hexylenedioxybutane $$ 2-Propyl-1,3-dioxonane # $$

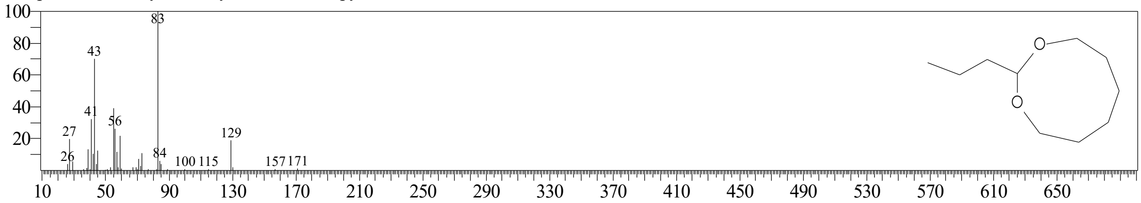

Qualitative Analysis Report

<< Target >>
Line#:43 R.Time:7.740(Scan#:749) MassPeaks:330
RawMode:Averaged 7.735-7.745(748-750) BasePeak:99.10(63844)
BG Mode:Calc. from Peak Group 1 - Event 1 Scan

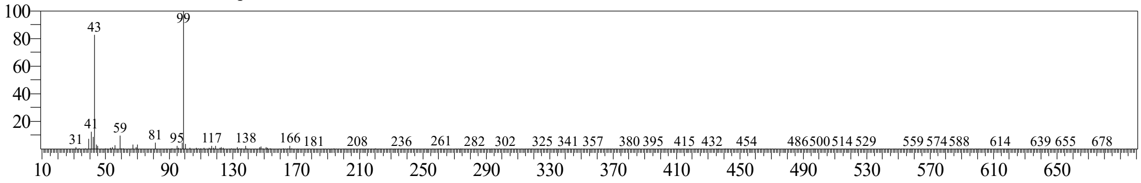

Hit#:1 Entry:18502 Library:NIST11.lib
SI:81 Formula:C9H16O2 CAS:10200-21-2 MolWeight:156 RetIndex:1194
CompName:2(3H)-Furanone, dihydro-5-methyl-5-(2-methylpropyl)- $$ 5-Isobutyl-5-methyldihydro-2(3H)-furanone # $$

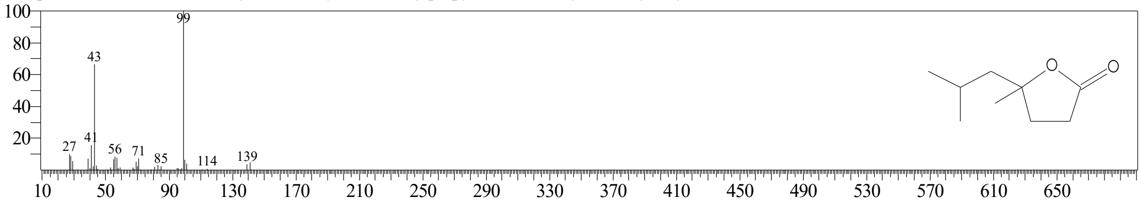

Hit#:2 Entry:1770 Library:NIST11.lib
SI:79 Formula:C2H5N5 CAS:38104-45-9 MolWeight:99 RetIndex:1130
CompName:3,4-Diamino-1,2,4(4H)-triazole $$ 3,4-Diamino-1,2,4-triazole $$ 4H-1,2,4-Triazole-3,4-diamine # $$

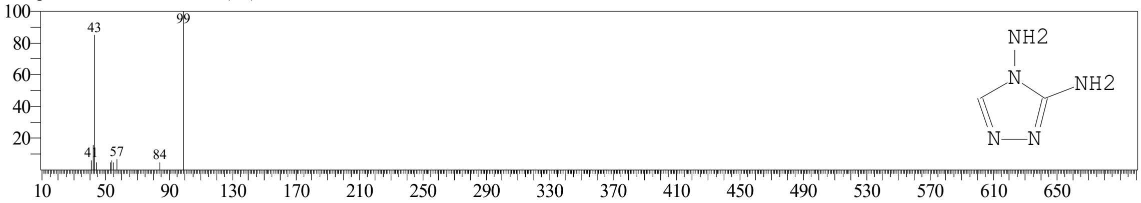

Hit#:3 Entry:11476 Library:NIST11.lib
SI:79 Formula:C4H7N5O CAS:0-00-0 MolWeight:141 RetIndex:1454
CompName:1H-1,2,4-Triazole-3,5-diamine, 1-acetyl-

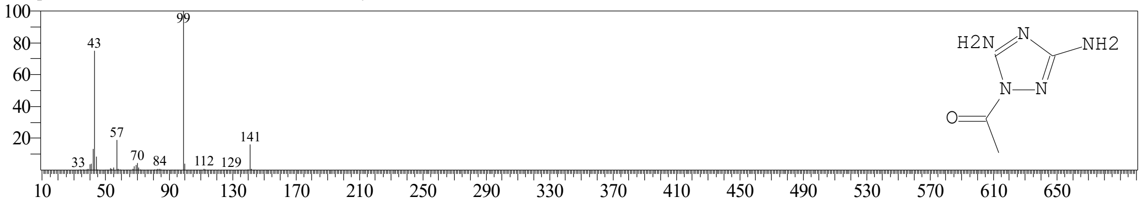

Hit#:4 Entry:1771 Library:NIST11.lib
SI:79 Formula:C2H5N5 CAS:1455-77-2 MolWeight:99 RetIndex:1311
CompName:3,5-Diamino-1,2,4-triazole $$ Guanazole $$ 1H-1,2,4-Triazole-3,5-diamine $$ MC 51762 $$ NSC 1895 $$ 1,2,4-Triazolidine, 3,5-diimino- $$ 3,5-

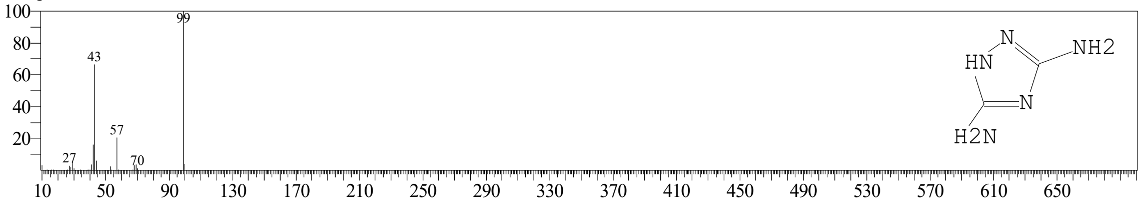

Hit#:5 Entry:1778 Library:NIST11.lib
SI:78 Formula:C3H5N3O CAS:503-86-6 MolWeight:99 RetIndex:1229
CompName:4H-Imidazol-4-one, 2-amino-1,5-dihydro- $$ Glycocyanidine $$ 2-Amino-1,5-dihydro-4H-imidazol-4-one # $$

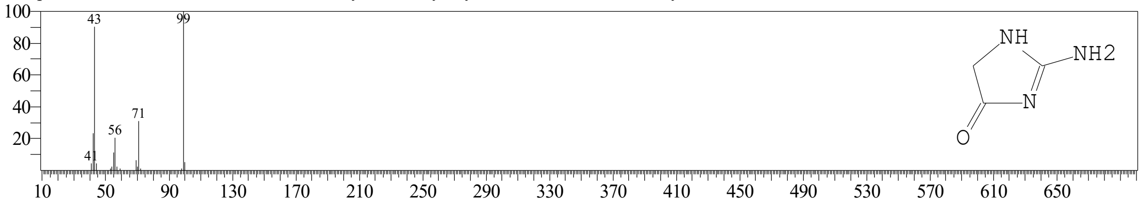

<< Target >>
Line#:44 R.Time:7.795(Scan#:760) MassPeaks:350
RawMode:Averaged 7.790-7.800(759-761) BasePeak:43.05(356198)
BG Mode:Calc. from Peak Group 1 - Event 1 Scan

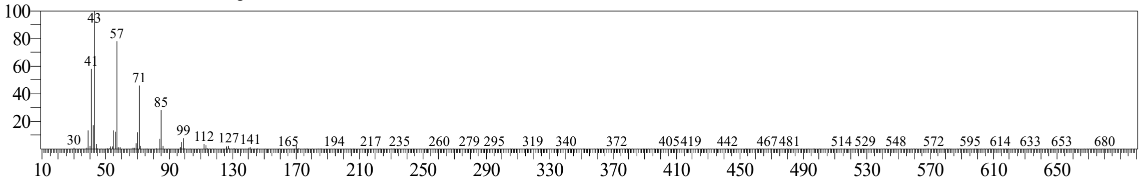

Hit#:1 Entry:34138 Library:NIST11.lib
SI:93 Formula:C13H28 CAS:1560-97-0 MolWeight:184 RetIndex:1249
CompName:Dodecane, 2-methyl- $$ 11-Methyldodecane $$ 2-Methyldodecane $$

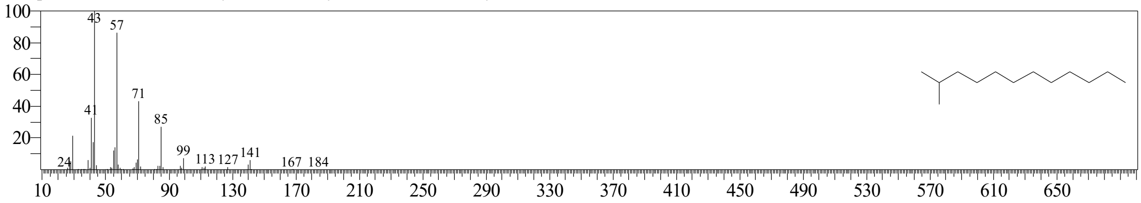

Hit#:2 Entry:26094 Library:NIST11.lib
SI:93 Formula:C12H26 CAS:1002-17-1 MolWeight:170 RetIndex:1086
CompName:Decane, 2,9-dimethyl- $$ 2,9-Dimethyldecane $$

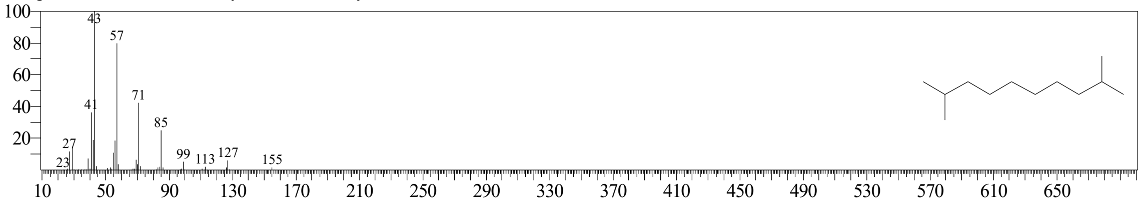

Hit#:3 Entry:52481 Library:NIST11.lib
SI:93 Formula:C15H32 CAS:55030-62-1 MolWeight:212 RetIndex:1384
CompName:Tridecane, 4,8-dimethyl- $$ 4,8-Dimethyltridecane # $$

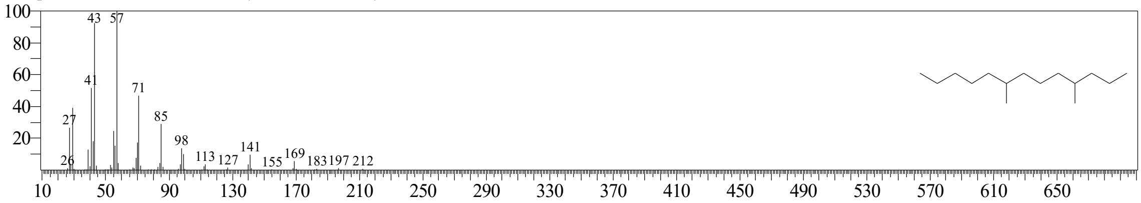

Hit#:4 Entry:34136 Library:NIST11.lib
SI:93 Formula:C13H28 CAS:17301-32-5 MolWeight:184 RetIndex:1185
CompName:Undecane, 4,7-dimethyl- $$ 4,7-Dimethylundecane # $$

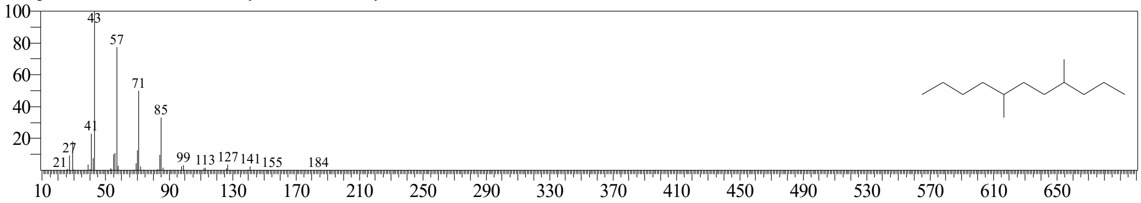

Hit#:5 Entry:18715 Library:NIST11.lib
SI:92 Formula:C11H24 CAS:1120-21-4 MolWeight:156 RetIndex:1115
CompName:Undecane $$ n-Undecane $$ Hendecane $$ n-C11H24 $$ UN 2330 $$

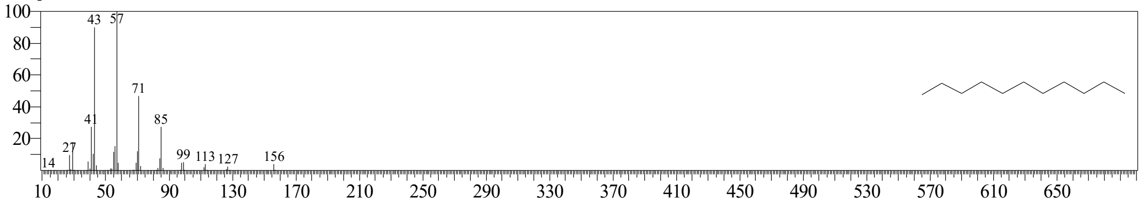

Qualitative Analysis Report

<< Target >>
Line#:45 R.Time:7.915(Scan#:784) MassPeaks:381
RawMode:Averaged 7.910-7.920(783-785) BasePeak:43.05(84111)
BG Mode:Calc. from Peak Group 1 - Event 1 Scan

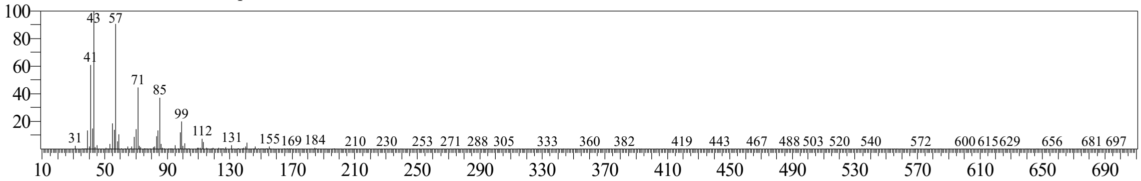

Hit#:1 Entry:52481 Library:NIST11.lib
SI:90 Formula:C15H32 CAS:55030-62-1 MolWeight:212 RetIndex:1384
CompName:Tridecane, 4,8-dimethyl- $$ 4,8-Dimethyltridecane # $$

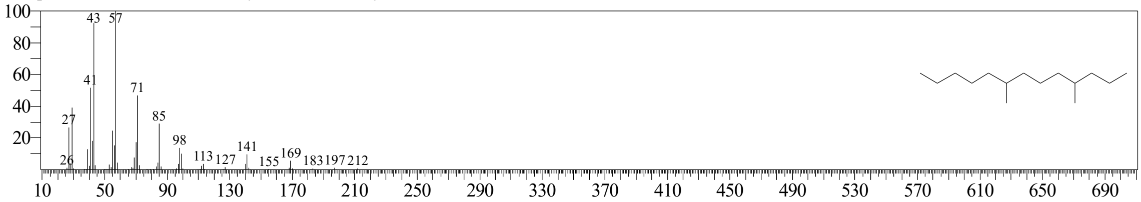

Hit#:2 Entry:18717 Library:NIST11.lib
SI:89 Formula:C11H24 CAS:6975-98-0 MolWeight:156 RetIndex:1051
CompName:Decane, 2-methyl- $$ 2-Methyldecane $$ n-C8H17CH(CH3)2 $$

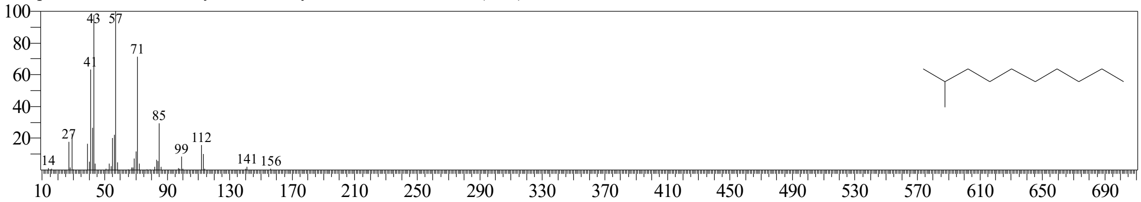

Hit#:3 Entry:26110 Library:NIST11.lib
SI:89 Formula:C12H26 CAS:62185-21-1 MolWeight:170 RetIndex:958
CompName:Octane, 3,4,5,6-tetramethyl- $$ 3,4,5,6-Tetramethyloctane # $$

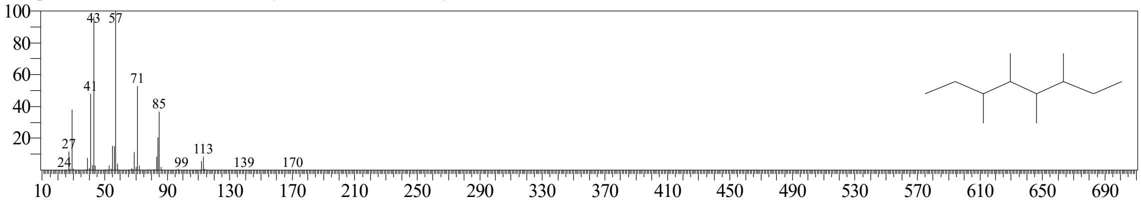

Hit#:4 Entry:12320 Library:NIST11.lib
SI:89 Formula:C10H22 CAS:124-18-5 MolWeight:142 RetIndex:1015
CompName:Decane $$ n-Decane $$ n-C10H22 $$ UN 2247 $$

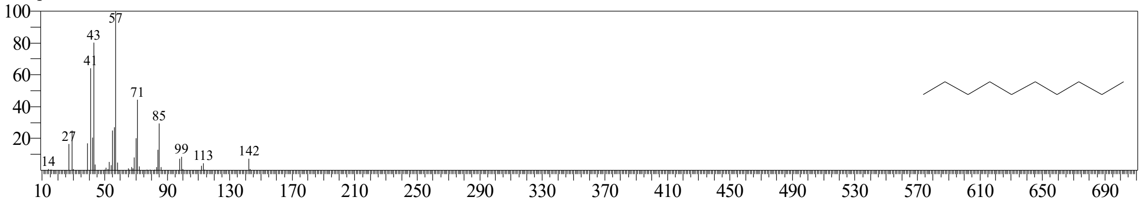

Hit#:5 Entry:18707 Library:NIST11.lib
SI:89 Formula:C11H24 CAS:62016-30-2 MolWeight:156 RetIndex:966
CompName:Octane, 2,3,3-trimethyl- $$ 2,3,3-Trimethyloctane # $$

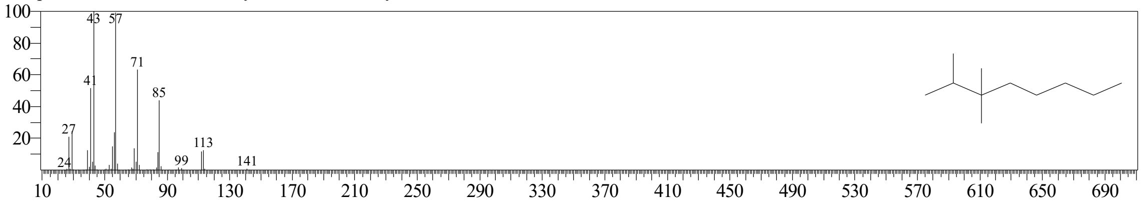

Qualitative Analysis Report

<< Target >>
Line#:46 R.Time:7.995(Scan#:800) MassPeaks:337
RawMode:Averaged 7.990-8.000(799-801) BasePeak:43.00(17668)
BG Mode:Calc. from Peak Group 1 - Event 1 Scan

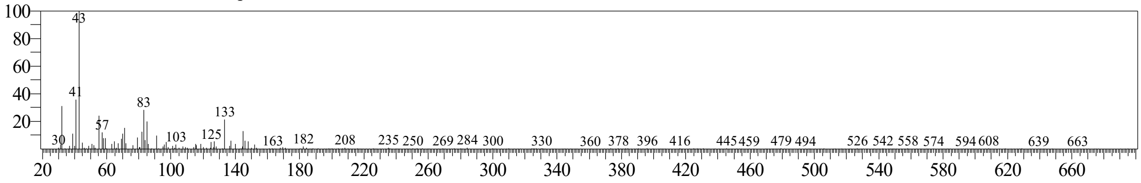

Hit#:1 Entry:43829 Library:NIST11.lib
SI:71 Formula:C11H20O3 CAS:0-00-0 MolWeight:200 RetIndex:1199
CompName:Acetic acid, (3-allyloxy-1,1-dimethylbutyl) ester $$ 3-(Allyloxy)-1,1-dimethylbutyl acetate # $$

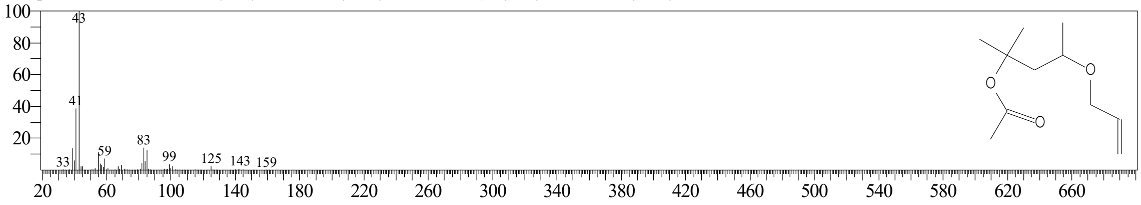

Hit#:2 Entry:11072 Library:NIST11.lib
SI:71 Formula:C8H12O2 CAS:60415-94-3 MolWeight:140 RetIndex:1167
CompName:2-Acetonylcyclopentanone $$ 2-(2-Oxopropyl)-1-cyclopentanone $$ Cyclopentanone, 2-(2-oxopropyl)- $$ 2-(2-Oxopropyl)cyclopentanone $$ Cycl

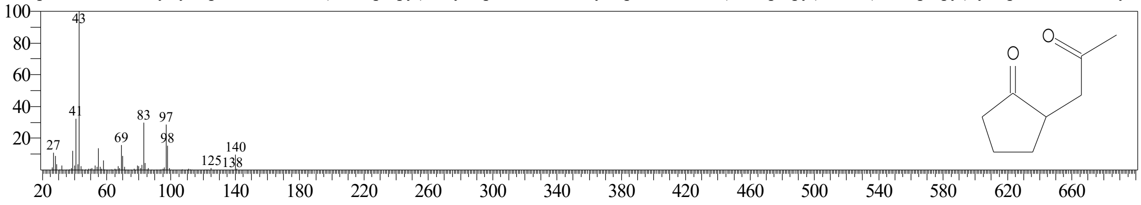

Hit#:3 Entry:26098 Library:NIST11.lib
SI:70 Formula:C12H26 CAS:52670-34-5 MolWeight:170 RetIndex:958
CompName:Octane, 2,3,6,7-tetramethyl- $$ 2,3,6,7-Tetramethyloctane # $$

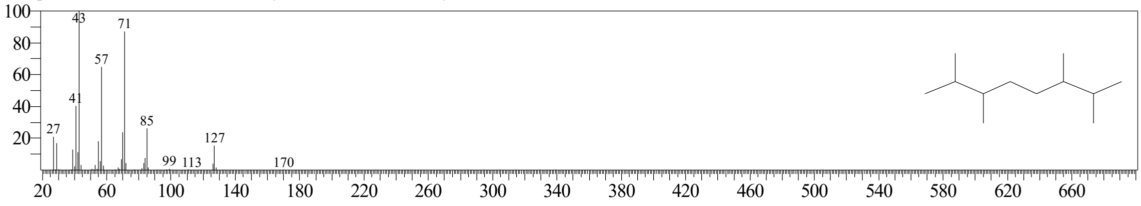

Hit#:4 Entry:4656 Library:NIST11.lib
SI:70 Formula:C7H16O CAS:111768-02-6 MolWeight:116 RetIndex:832
CompName:1-Pentanol, 2,4-dimethyl-, (.+/-.)- $$ 2,4-Dimethyl-1-pentanol $$

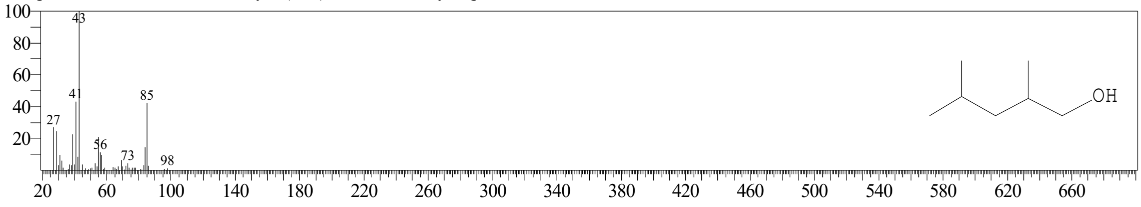

Hit#:5 Entry:96490 Library:NIST11.lib
SI:69 Formula:C15H28O4 CAS:0-00-0 MolWeight:272 RetIndex:1847
CompName:Oxalic acid, decyl propyl ester

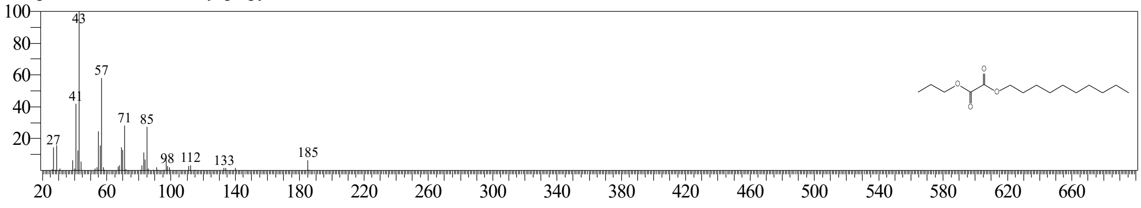

<< Target >>
Line#:47 R.Time:8.075(Scan#:816) MassPeaks:363
RawMode:Averaged 8.070-8.080(815-817) BasePeak:43.10(11291)
BG Mode:Calc. from Peak Group 1 - Event 1 Scan

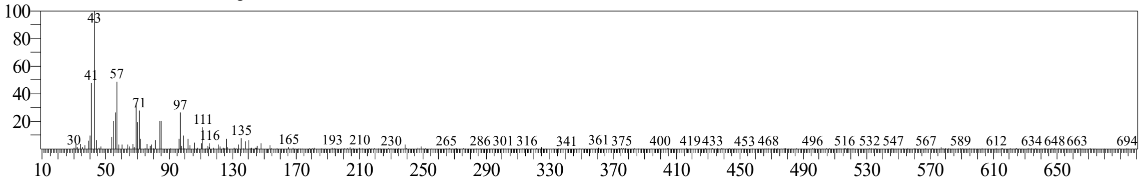

Hit#:1 Entry:129412 Library:NIST11.lib
SI:79 Formula:C18H34O4 CAS:0-00-0 MolWeight:314 RetIndex:2145
CompName:Oxalic acid, propyl tridecyl ester

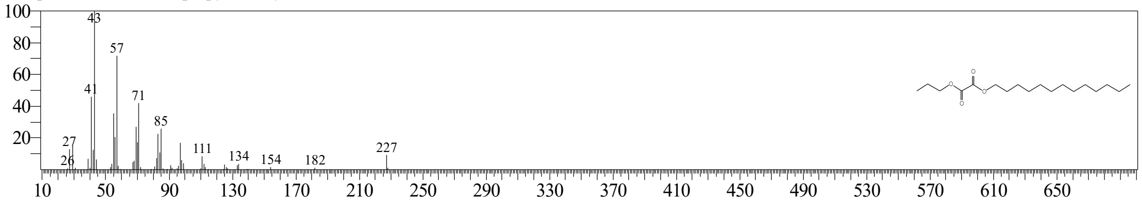

Hit#:2 Entry:19561 Library:NIST11.lib
SI:79 Formula:C10H22O CAS:10042-59-8 MolWeight:158 RetIndex:1194
CompName:1-Heptanol, 2-propyl- $$ 2-Propylheptanol $$ 2-Propyl-1-heptanol # $$

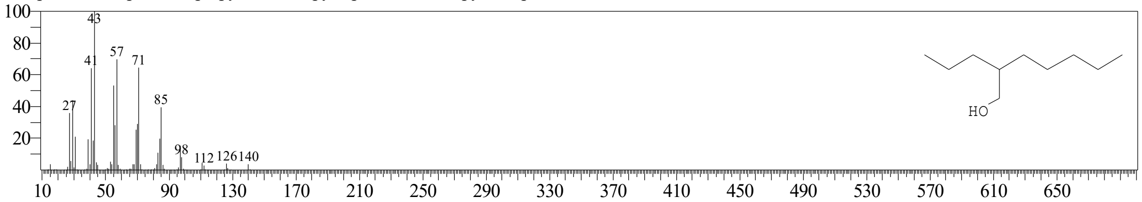

Hit#:3 Entry:107435 Library:NIST11.lib
SI:79 Formula:C16H30O4 CAS:0-00-0 MolWeight:286 RetIndex:1946
CompName:Oxalic acid, propyl undecyl ester

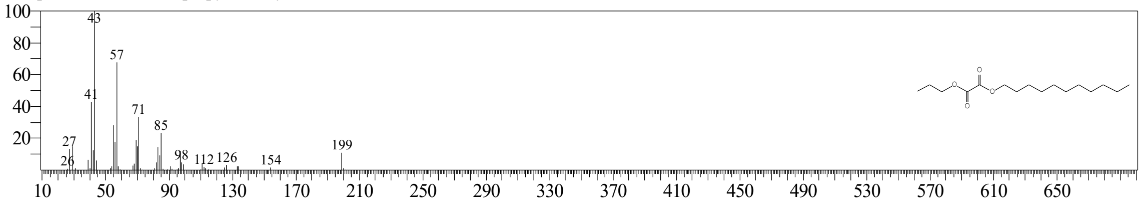

Hit#:4 Entry:175156 Library:NIST11.lib
SI:79 Formula:C23H44O4 CAS:0-00-0 MolWeight:384 RetIndex:2642
CompName:Oxalic acid, octadecyl propyl ester

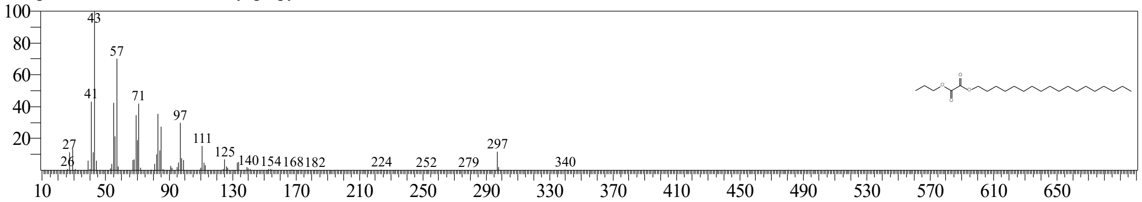

Hit#:5 Entry:24841 Library:NIST11.lib
SI:78 Formula:C12H24 CAS:74630-42-5 MolWeight:168 RetIndex:1140
CompName:1-Undecene, 7-methyl- $$ 7-Methyl-1-undecene # $$

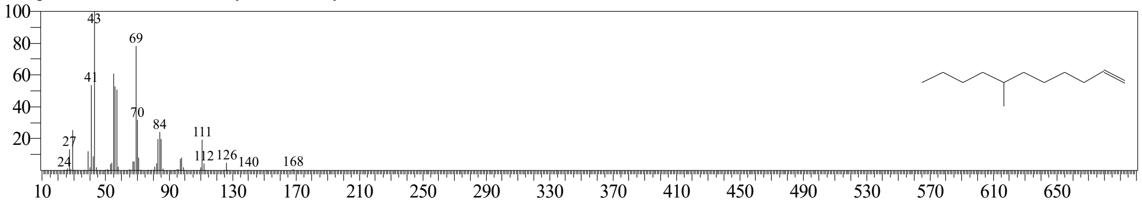

<< Target >>
Line#:48 R.Time:8.145(Scan#:830) MassPeaks:324
RawMode:Averaged 8.140-8.150(829-831) BasePeak:43.05(41787)
BG Mode:Calc. from Peak Group 1 - Event 1 Scan

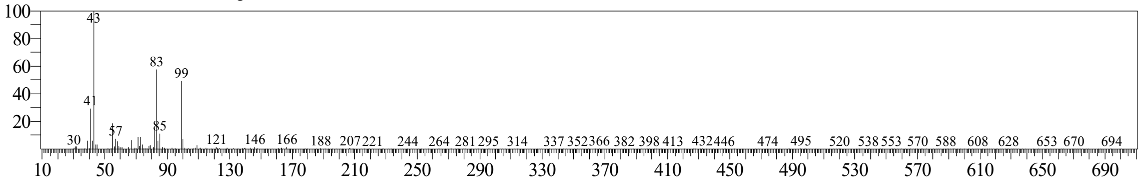

Hit#:1 Entry:12033 Library:NIST11.lib
SI:80 Formula:C8H14O2 CAS:3050-69-9 MolWeight:142 RetIndex:974
CompName:n-Caproic acid vinyl ester $$ Hexanoic acid, ethenyl ester $$ Caproic acid, vinyl ester $$ Hexanoic acid, vinyl ester $$ Vinyl caproate $$ Vinyl hexa

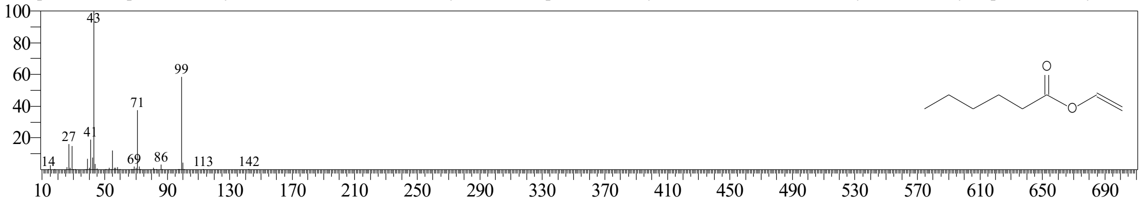

Hit#:2 Entry:33872 Library:NIST11.lib
SI:79 Formula:C11H20O2 CAS:0-00-0 MolWeight:184 RetIndex:1238
CompName:6,6,7-Trimethyl-octane-2,5-dione $$ 6,6,7-Trimethyl-2,5-octanedione # $$

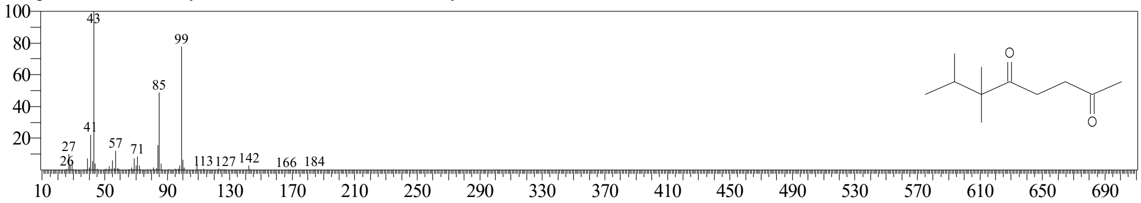

Hit#:3 Entry:32924 Library:NIST11.lib
SI:79 Formula:C12H22O CAS:0-00-0 MolWeight:182 RetIndex:1359
CompName:2-Cyclohexyl-hex-5-en-2-ol $$ 2-Cyclohexyl-5-hexen-2-ol # $$

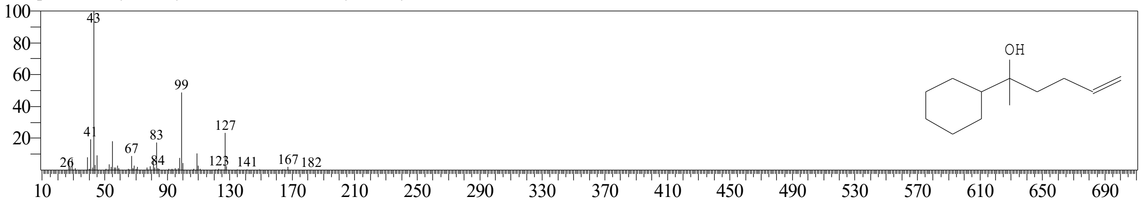

Hit#:4 Entry:43829 Library:NIST11.lib
SI:79 Formula:C11H20O3 CAS:0-00-0 MolWeight:200 RetIndex:1199
CompName:Acetic acid, (3-allyloxy-1,1-dimethylbutyl) ester $$ 3-(Allyloxy)-1,1-dimethylbutyl acetate # $$

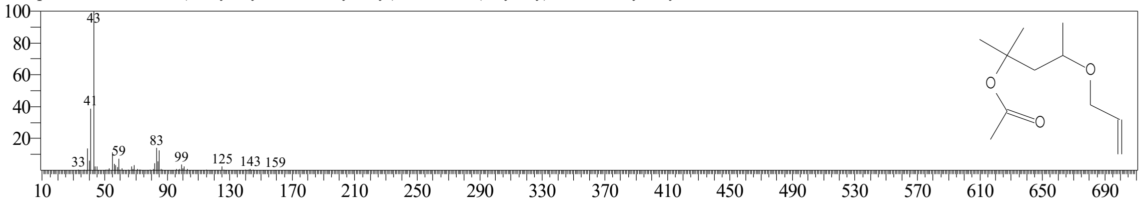

Hit#:5 Entry:42695 Library:NIST11.lib
SI:79 Formula:C12H22O2 CAS:22427-95-8 MolWeight:198 RetIndex:1509
CompName:3,7-Decadiene-5,6-diol, 4,7-dimethyl- $$ (3E,7E)-4,7-Dimethyl-3,7-decadiene-5,6-diol # $$

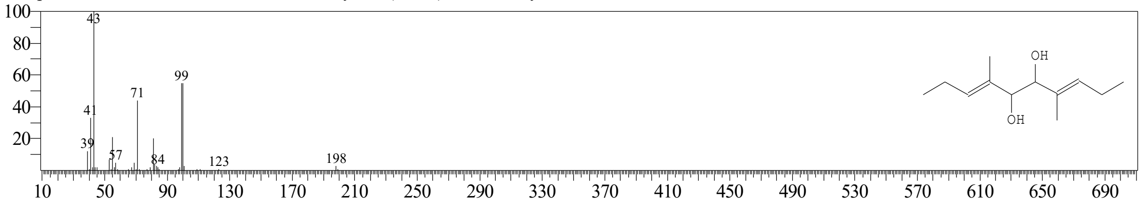

Qualitative Analysis Report

<< Target >>
Line#:49 R.Time:8.175(Scan#:836) MassPeaks:318
RawMode:Averaged 8.170-8.180(835-837) BasePeak:43.05(17543)
BG Mode:Calc. from Peak Group 1 - Event 1 Scan

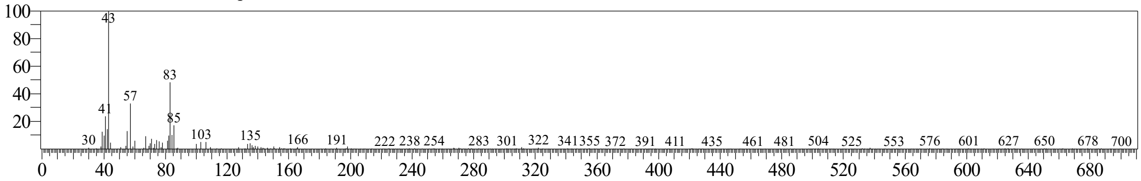

Hit#:1 Entry:7418 Library:NIST11.lib
SI:77 Formula:C9H20 CAS:16789-46-1 MolWeight:128 RetIndex:788
CompName:Hexane, 3-ethyl-2-methyl- $$ 3-Ethyl-2-methylhexane $$ 2-Methyl-3-ethylhexane $$

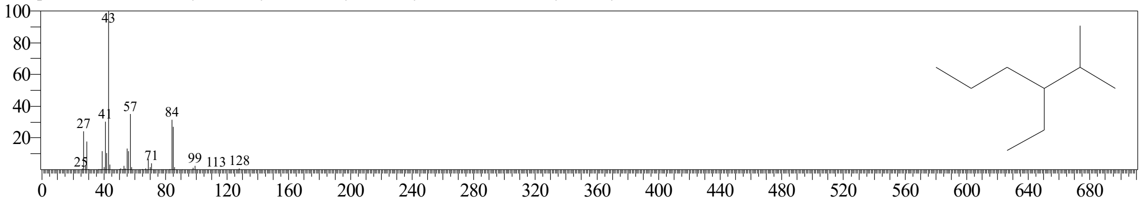

Hit#:2 Entry:7413 Library:NIST11.lib
SI:77 Formula:C9H20 CAS:921-47-1 MolWeight:128 RetIndex:724
CompName:Hexane, 2,3,4-trimethyl- $$ 2,3,4-Trimethylhexane $$

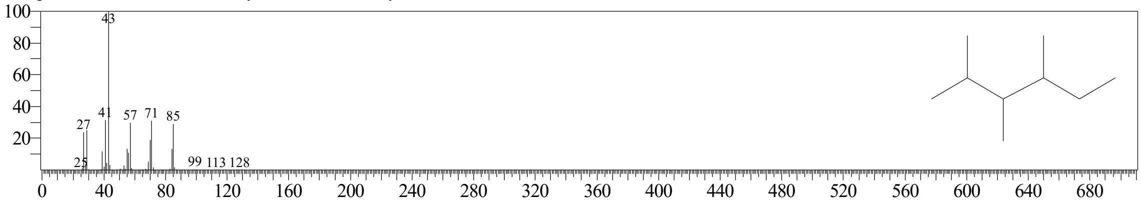

Hit#:3 Entry:4243 Library:NIST11.lib
SI:76 Formula:C8H18 CAS:560-21-4 MolWeight:114 RetIndex:668
CompName:Pentane, 2,3,3-trimethyl- $$ 2,3,3-Trimethylpentane $$ C2H5C(CH3)2CH(CH3)2 $$

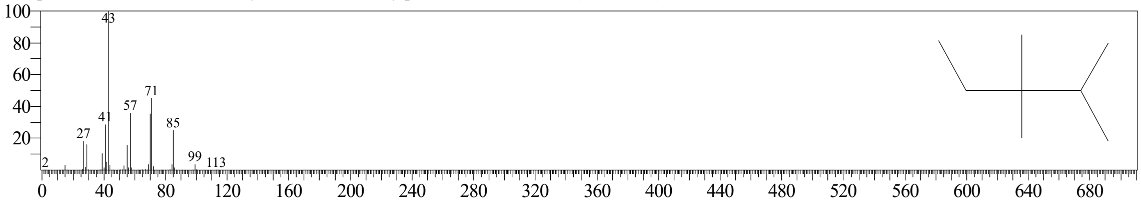

Hit#:4 Entry:6651 Library:NIST11.lib
SI:76 Formula:C9H18 CAS:13151-12-7 MolWeight:126 RetIndex:842
CompName:1-Octene, 4-methyl- $$ 4-Methyl-1-octene # $$

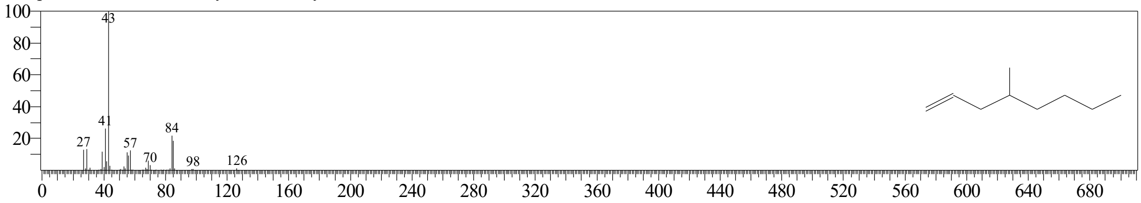

Hit#:5 Entry:4240 Library:NIST11.lib
SI:76 Formula:C8H18 CAS:609-26-7 MolWeight:114 RetIndex:688
CompName:Pentane, 3-ethyl-2-methyl- $$ 2-Methyl-3-ethylpentane $$ 3-Ethyl-2-methylpentane $$

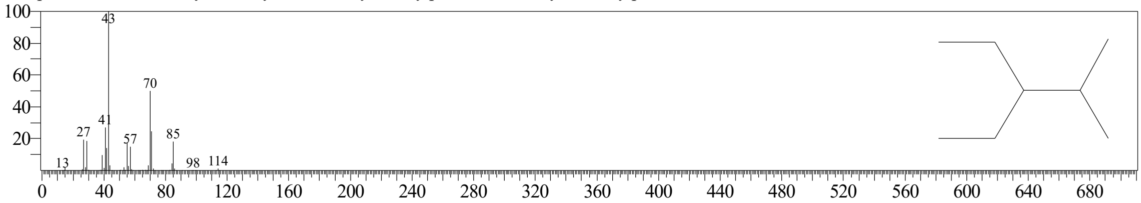

<< Target >>
Line#:50 R.Time:8.255(Scan#:852) MassPeaks:352
RawMode:Averaged 8.250-8.260(851-853) BasePeak:32.10(10678)
BG Mode:Calc. from Peak Group 1 - Event 1 Scan

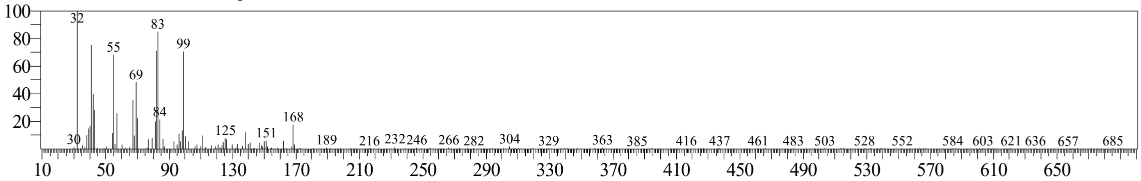

Hit#:1 Entry:41666 Library:NIST11.lib
SI:74 Formula:C14H28 CAS:61142-52-7 MolWeight:196 RetIndex:1418
CompName:Cyclopentane, 1-butyl-2-pentyl- $$ 1-Butyl-2-pentylcyclopentane # $$

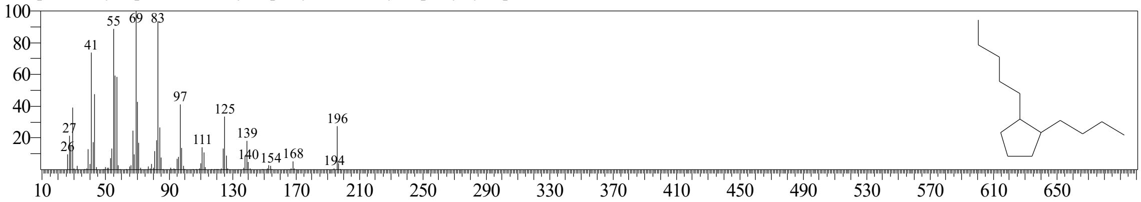

Hit#:2 Entry:24847 Library:NIST11.lib
SI:74 Formula:C12H24 CAS:7206-29-3 MolWeight:168 RetIndex:1222
CompName:6-Dodecene, (Z)- $$ (6Z)-6-Dodecene $$ cis-6-Dodecene $$ (Z)-6-Dodecene $$

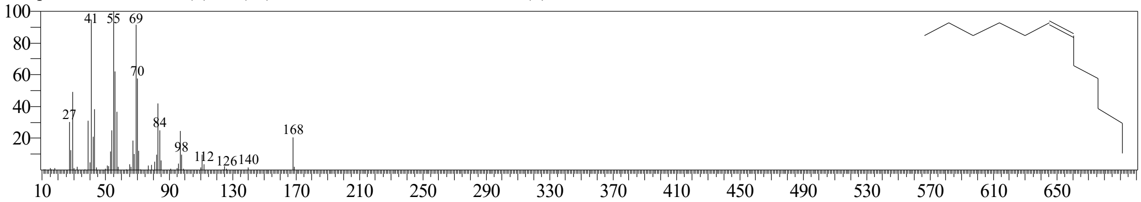

Hit#:3 Entry:24853 Library:NIST11.lib
SI:74 Formula:C12H24 CAS:66553-50-2 MolWeight:168 RetIndex:1155
CompName:Cyclopentane, 1-methyl-2-(4-methylpentyl)-, trans- $$ trans-1-Methyl-2-(4-methylpentyl)cyclopentane $$ 1-Methyl-2-(4-methylpentyl)cyclopentan

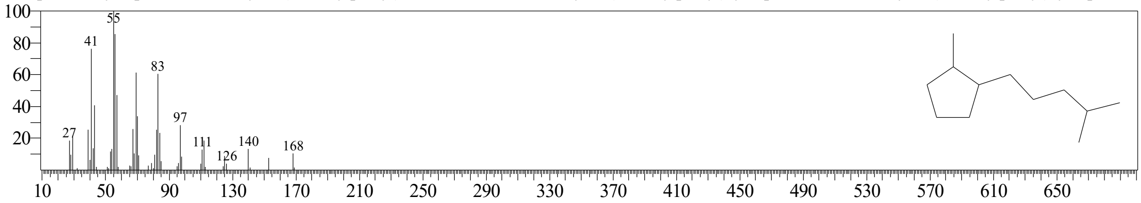

Hit#:4 Entry:6664 Library:NIST11.lib
SI:73 Formula:C9H18 CAS:3728-57-2 MolWeight:126 RetIndex:921
CompName:Cyclopentane, 1-methyl-2-propyl- $$ 1-Methyl-2-propylcyclopentane # $$

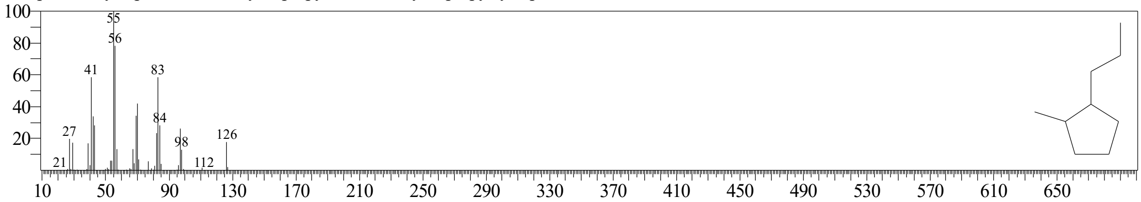

Hit#:5 Entry:92916 Library:NIST11.lib
SI:73 Formula:C12H25ClO2S CAS:10147-40-7 MolWeight:268 RetIndex:1897
CompName:1-Dodecanesulfonyl chloride $$ 1-Dodecanesulphonyl chloride $$

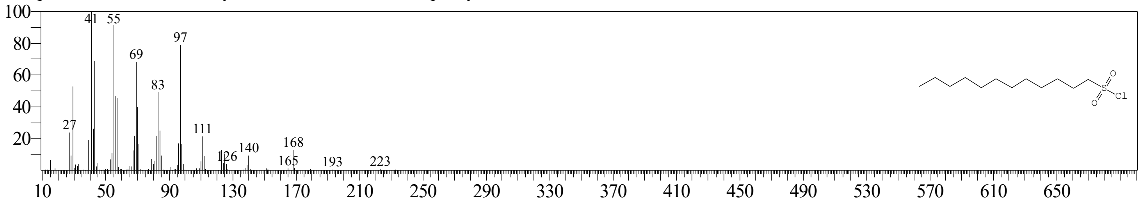

Qualitative Analysis Report

<< Target >>
Line#:51 R.Time:8.315(Scan#:864) MassPeaks:337
RawMode:Averaged 8.310-8.320(863-865) BasePeak:43.10(23617)
BG Mode:Calc. from Peak Group 1 - Event 1 Scan

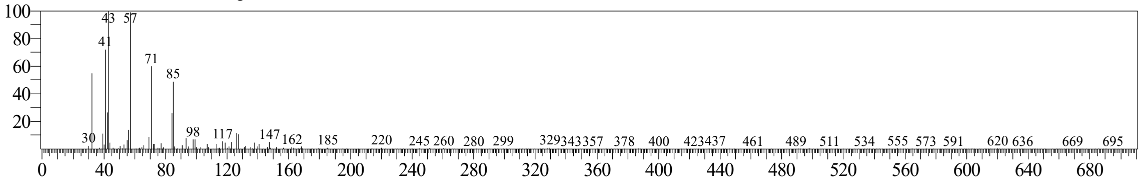

Hit#:1 Entry:47433 Library:NIST11.lib
SI:81 Formula:C9H19Br CAS:2216-35-5 MolWeight:206 RetIndex:1148
CompName:2-Bromononane $$ sec-Nonyl bromide $$ Nonane, 2-bromo- $$

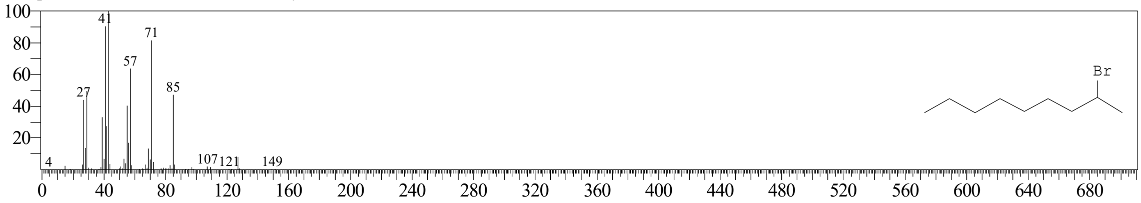

Hit#:2 Entry:26091 Library:NIST11.lib
SI:81 Formula:C12H26 CAS:13150-81-7 MolWeight:170 RetIndex:1086
CompName:2,6-Dimethyldecane

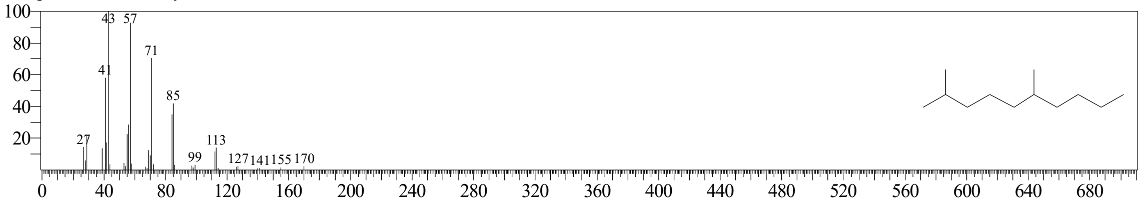

Hit#:3 Entry:18707 Library:NIST11.lib
SI:80 Formula:C11H24 CAS:62016-30-2 MolWeight:156 RetIndex:966
CompName:Octane, 2,3,3-trimethyl- $$ 2,3,3-Trimethyloctane # $$

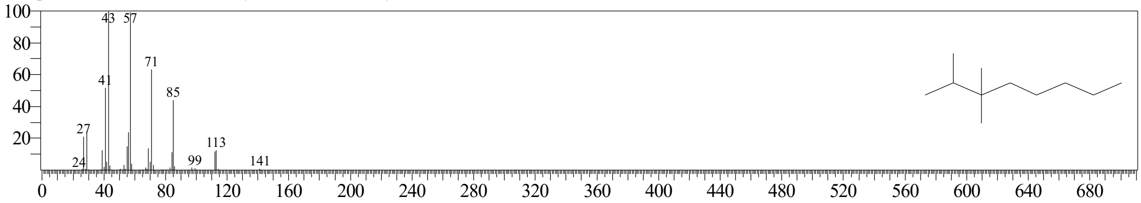

Hit#:4 Entry:26099 Library:NIST11.lib
SI:80 Formula:C12H26 CAS:17312-44-6 MolWeight:170 RetIndex:1086
CompName:2,3-Dimethyldecane $$ Decane, 2,3-dimethyl $$

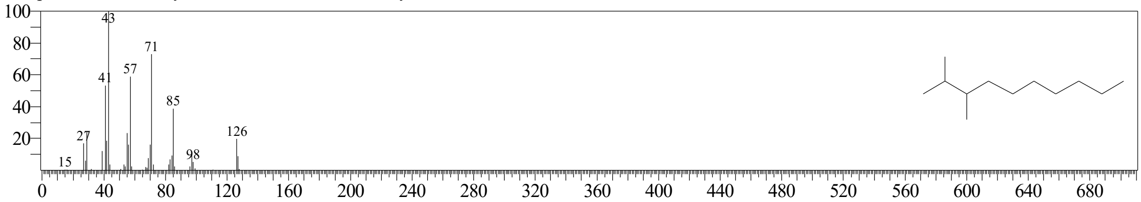

Hit#:5 Entry:26090 Library:NIST11.lib
SI:80 Formula:C12H26 CAS:7045-71-8 MolWeight:170 RetIndex:1150
CompName:Undecane, 2-methyl- $$ 2-Methylundecane $$

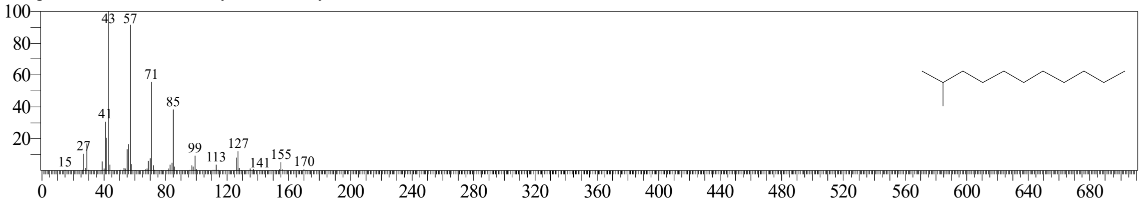

<< Target >>
Line#:52 R.Time:8.365(Scan#:874) MassPeaks:361
RawMode:Averaged 8.360-8.370(873-875) BasePeak:43.10(39568)
BG Mode:Calc. from Peak Group 1 - Event 1 Scan

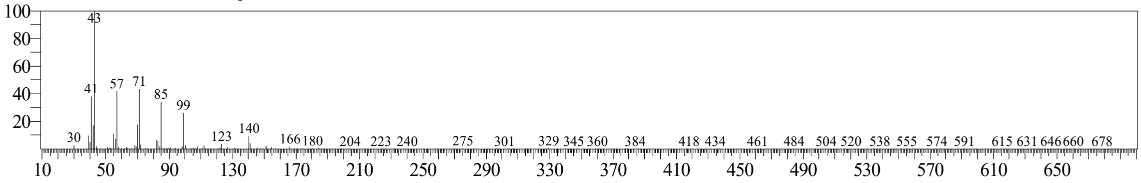

Hit#:1 Entry:34145 Library:NIST11.lib
SI:88 Formula:C13H28 CAS:17301-33-6 MolWeight:184 RetIndex:1185
CompName:Undecane, 4,8-dimethyl- $$ 4,8-Dimethylundecane # $$

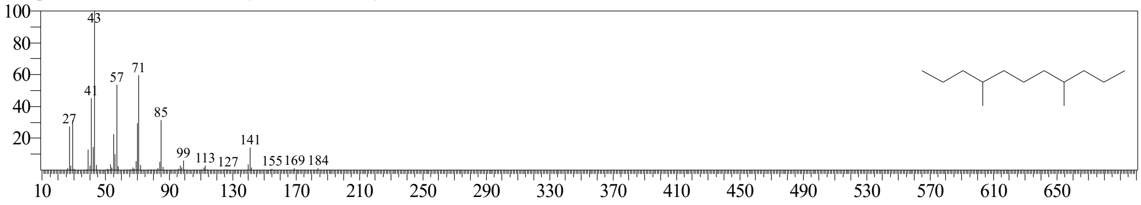

Hit#:2 Entry:34138 Library:NIST11.lib
SI:87 Formula:C13H28 CAS:1560-97-0 MolWeight:184 RetIndex:1249
CompName:Dodecane, 2-methyl- $$ 11-Methyldodecane $$ 2-Methyldodecane $$

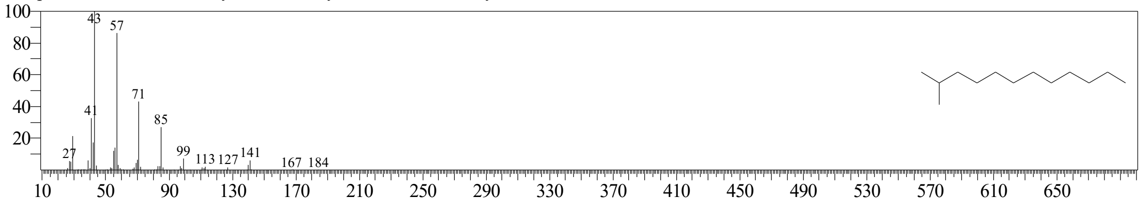

Hit#:3 Entry:34133 Library:NIST11.lib
SI:87 Formula:C13H28 CAS:17301-27-8 MolWeight:184 RetIndex:1185
CompName:Undecane, 2,10-dimethyl- $$ 2,10-Dimethylundecane # $$

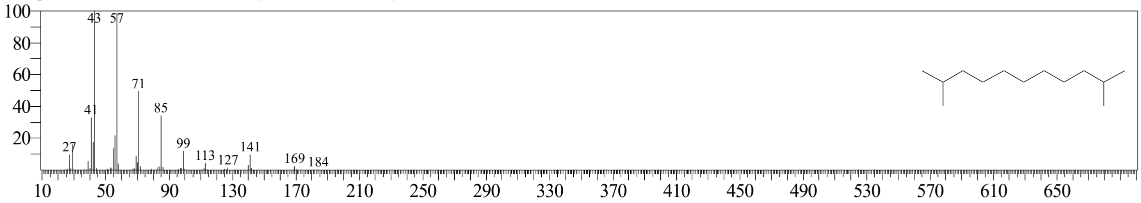

Hit#:4 Entry:34136 Library:NIST11.lib
SI:86 Formula:C13H28 CAS:17301-32-5 MolWeight:184 RetIndex:1185
CompName:Undecane, 4,7-dimethyl- $$ 4,7-Dimethylundecane # $$

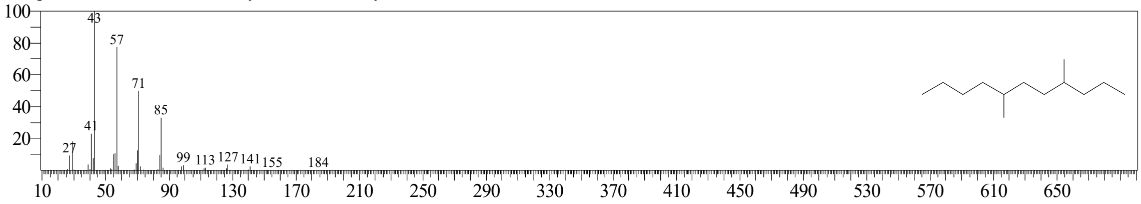

Hit#:5 Entry:26096 Library:NIST11.lib
SI:86 Formula:C12H26 CAS:1636-44-8 MolWeight:170 RetIndex:1150
CompName:Decane, 4-ethyl- $$ 4-Ethyldecane # $$

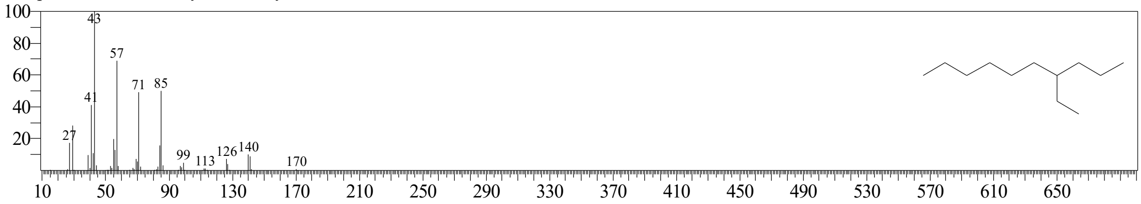

<< Target >>
Line#:53 R.Time:8.425(Scan#:886) MassPeaks:359
RawMode:Averaged 8.420-8.430(885-887) BasePeak:43.10(35747)
BG Mode:Calc. from Peak Group 1 - Event 1 Scan

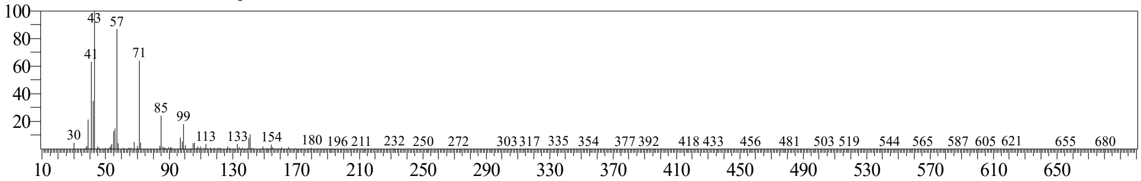

Hit#:1 Entry:34133 Library:NIST11.lib
SI:86 Formula:C13H28 CAS:17301-27-8 MolWeight:184 RetIndex:1185
CompName:Undecane, 2,10-dimethyl- $$ 2,10-Dimethylundecane # $$

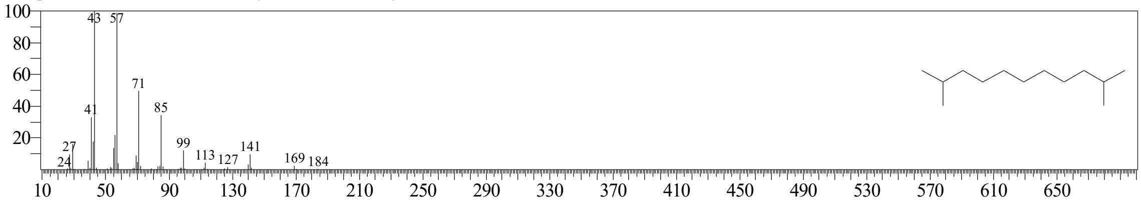

Hit#:2 Entry:18717 Library:NIST11.lib
SI:86 Formula:C11H24 CAS:6975-98-0 MolWeight:156 RetIndex:1051
CompName:Decane, 2-methyl- $$ 2-Methyldecane $$ n-C8H17CH(CH3)2 $$

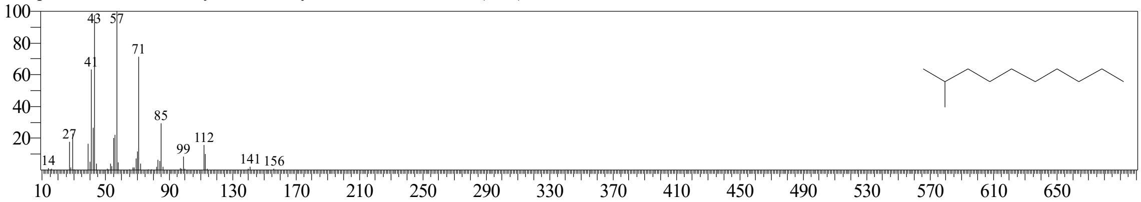

Hit#:3 Entry:52481 Library:NIST11.lib
SI:86 Formula:C15H32 CAS:55030-62-1 MolWeight:212 RetIndex:1384
CompName:Tridecane, 4,8-dimethyl- $$ 4,8-Dimethyltridecane # $$

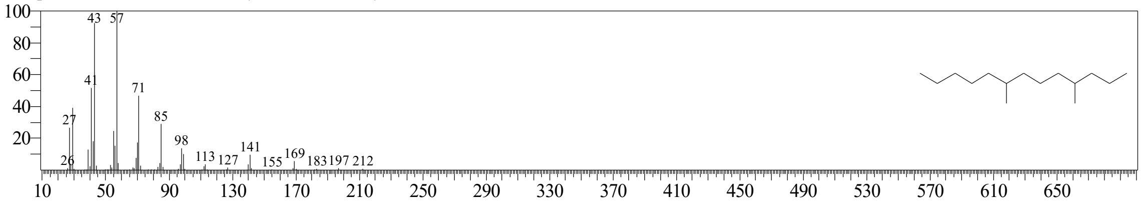

Hit#:4 Entry:34138 Library:NIST11.lib
SI:86 Formula:C13H28 CAS:1560-97-0 MolWeight:184 RetIndex:1249
CompName:Dodecane, 2-methyl- $$ 11-Methyldodecane $$ 2-Methyldodecane $$

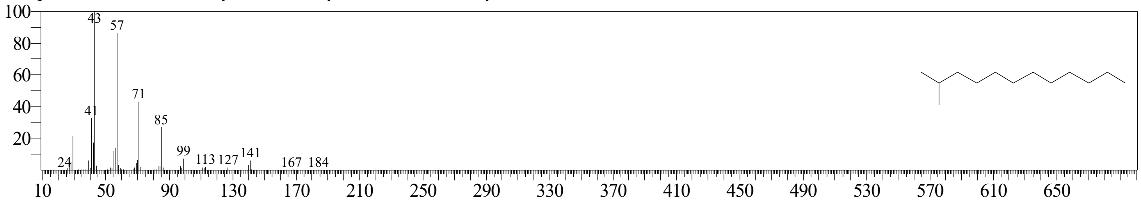

Hit#:5 Entry:26089 Library:NIST11.lib
SI:86 Formula:C12H26 CAS:17312-54-8 MolWeight:170 RetIndex:1086
CompName:Decane, 3,7-dimethyl- $$ 3,7-Dimethyldecane # $$

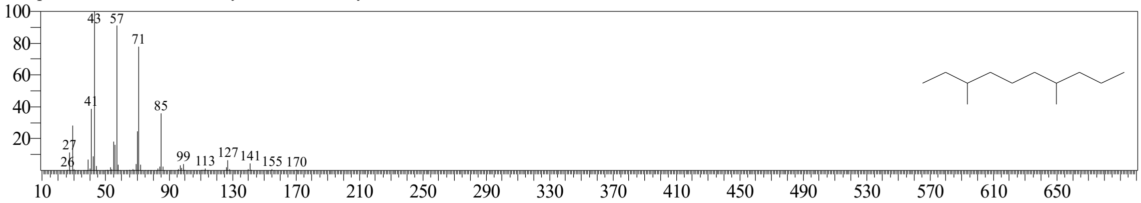

<< Target >>
Line#:54 R.Time:8.485(Scan#:898) MassPeaks:365
RawMode:Averaged 8.480-8.490(897-899) BasePeak:43.05(218461)
BG Mode:Calc. from Peak Group 1 - Event 1 Scan

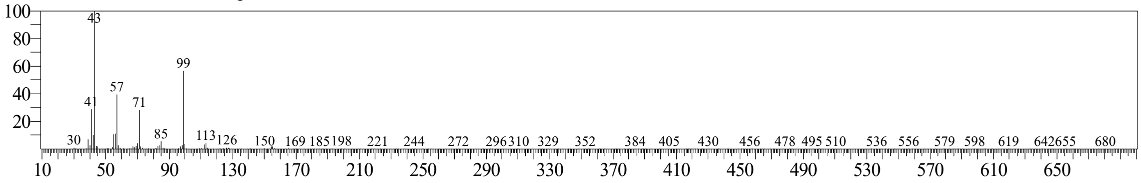

Hit#:1 Entry:12033 Library:NIST11.lib
SI:88 Formula:C8H14O2 CAS:3050-69-9 MolWeight:142 RetIndex:974
CompName:n-Caproic acid vinyl ester $$ Hexanoic acid, ethenyl ester $$ Caproic acid, vinyl ester $$ Hexanoic acid, vinyl ester $$ Vinyl caproate $$ Vinyl hexa

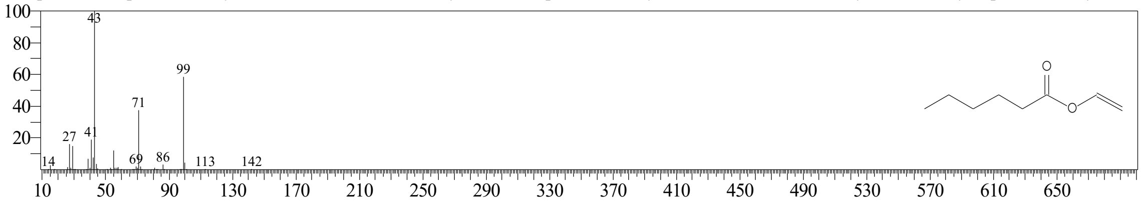

Hit#:2 Entry:12305 Library:NIST11.lib
SI:87 Formula:C10H22 CAS:1072-16-8 MolWeight:142 RetIndex:887
CompName:Octane, 2,7-dimethyl- $$ Diisoamyl $$ Diisopentyl $$ 2,7-Dimethyloctane $$

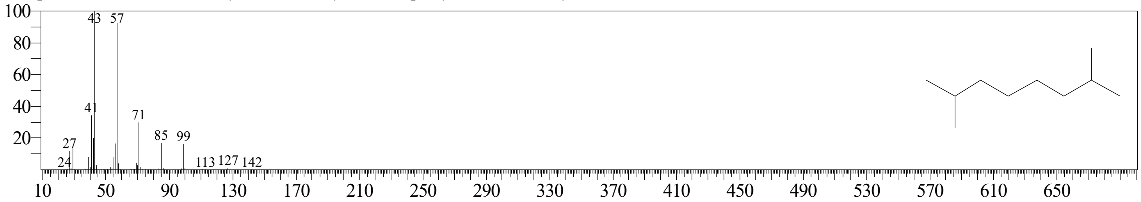

Hit#:3 Entry:7415 Library:NIST11.lib
SI:86 Formula:C9H20 CAS:1072-05-5 MolWeight:128 RetIndex:788
CompName:Heptane, 2,6-dimethyl- $$ 2,6-Dimethylheptane $$

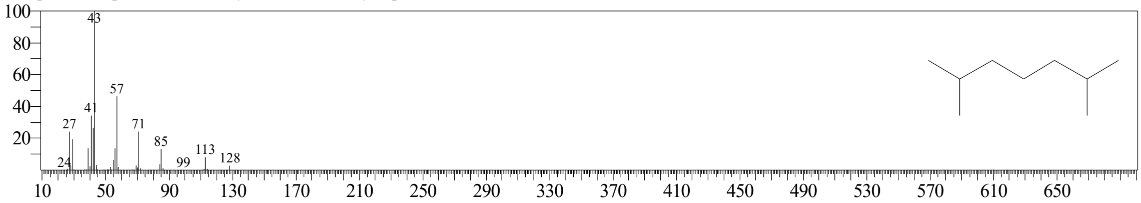

Hit#:4 Entry:26094 Library:NIST11.lib
SI:86 Formula:C12H26 CAS:1002-17-1 MolWeight:170 RetIndex:1086
CompName:Decane, 2,9-dimethyl- $$ 2,9-Dimethyldecane $$

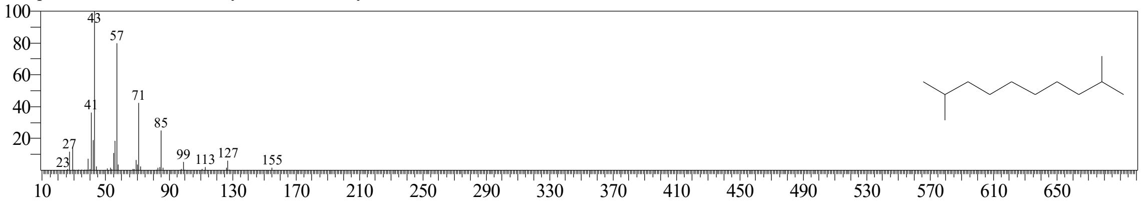

Hit#:5 Entry:26093 Library:NIST11.lib
SI:86 Formula:C12H26 CAS:2801-84-5 MolWeight:170 RetIndex:1086
CompName:Decane, 2,4-dimethyl- $$ 2,4-Dimethyldecane $$

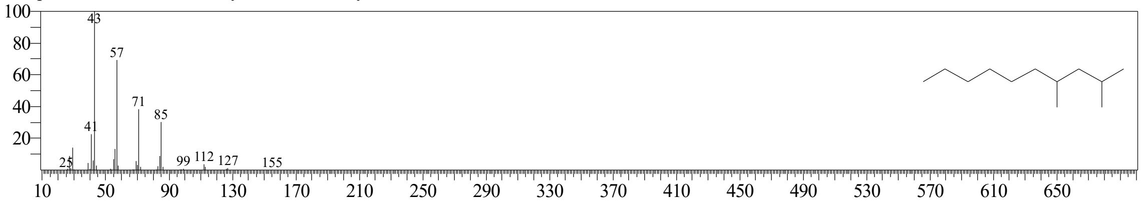

<< Target >>
Line#:55 R.Time:8.615(Scan#:924) MassPeaks:373
RawMode:Averaged 8.610-8.620(923-925) BasePeak:43.05(21810)
BG Mode:Calc. from Peak Group 1 - Event 1 Scan

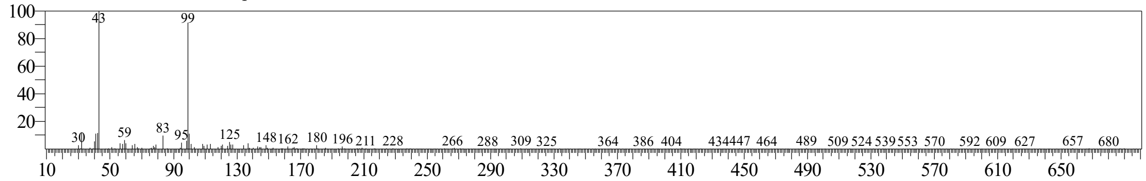

Hit#:1 Entry:11487 Library:NIST11.lib
SI:71 Formula:C5H7N3O2 CAS:55819-76-6 MolWeight:141 RetIndex:1564
CompName:1-Acetyl-2-cyanoacetylhydrazine $$ N'-(2-Cyanoacetyl)acetohydrazide # $$

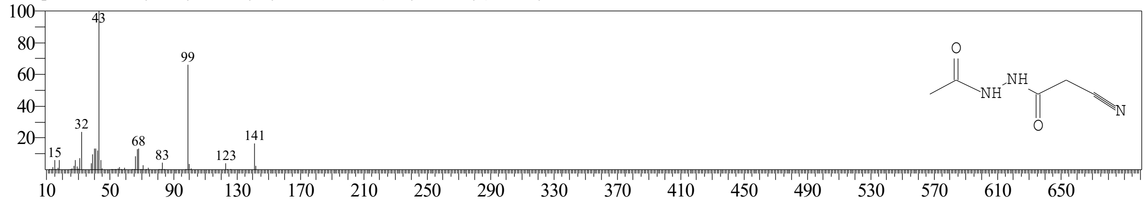

Hit#:2 Entry:18502 Library:NIST11.lib
SI:70 Formula:C9H16O2 CAS:10200-21-2 MolWeight:156 RetIndex:1194
CompName:2(3H)-Furanone, dihydro-5-methyl-5-(2-methylpropyl)- $$ 5-Isobutyl-5-methyldihydro-2(3H)-furanone # $$

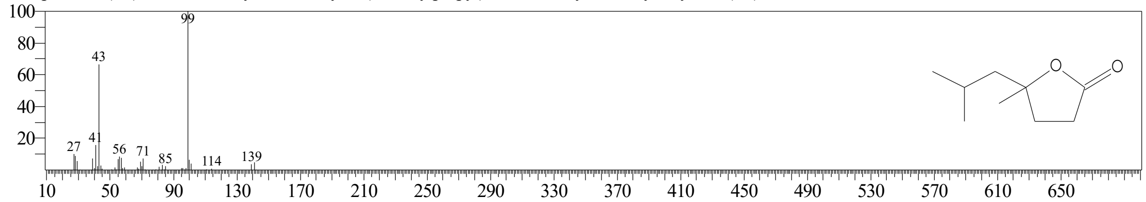

Hit#:3 Entry:11476 Library:NIST11.lib
SI:69 Formula:C4H7N5O CAS:0-00-0 MolWeight:141 RetIndex:1454
CompName:1H-1,2,4-Triazole-3,5-diamine, 1-acetyl-

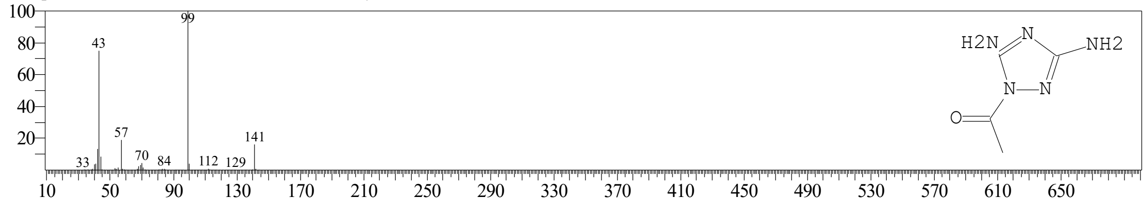

Hit#:4 Entry:19146 Library:NIST11.lib
SI:69 Formula:C7H10O4 CAS:57681-51-3 MolWeight:158 RetIndex:1226
CompName:2(3H)-Furanone, 5-(acetyloxy)dihydro-5-methyl- $$ 2-Methyl-5-oxotetrahydro-2-furanyl acetate # $$

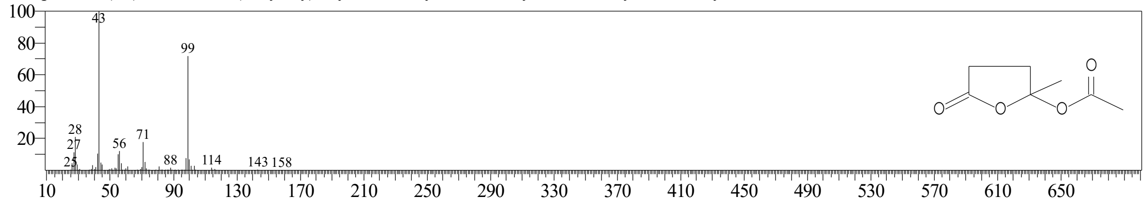

Hit#:5 Entry:26658 Library:NIST11.lib
SI:68 Formula:C7H13BO4 CAS:58163-59-0 MolWeight:172 RetIndex:0
CompName:1,3,2-Dioxaborolane-4-methanol, 2-ethyl-, acetate $$ (2-Ethyl-1,3,2-dioxaborolan-4-yl)methyl acetate # $$

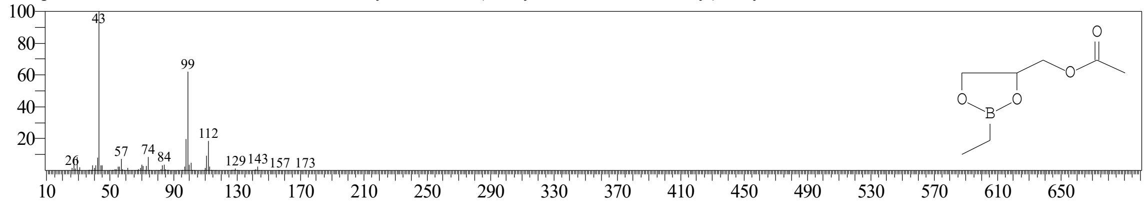

<< Target >>
Line#:56 R.Time:8.655(Scan#:932) MassPeaks:344
RawMode:Averaged 8.650-8.660(931-933) BasePeak:43.00(93081)
BG Mode:Calc. from Peak Group 1 - Event 1 Scan

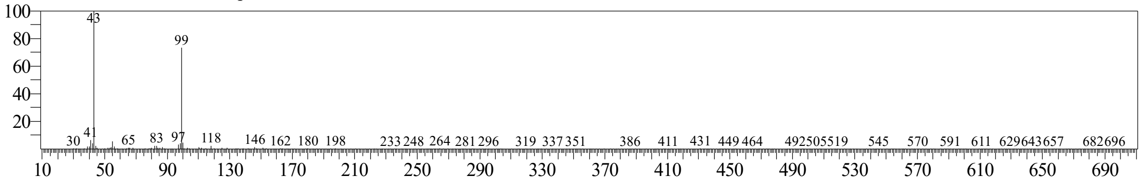

Hit#:1 Entry:11084 Library:NIST11.lib
SI:83 Formula:C8H12O2 CAS:0-00-0 MolWeight:140 RetIndex:900
CompName:1,5-Hexadien-3-ol, acetate

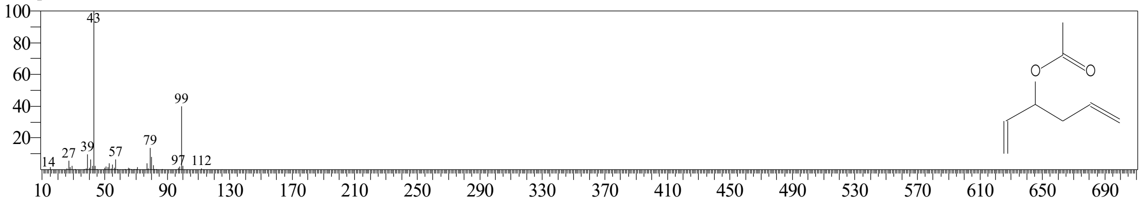

Hit#:2 Entry:4482 Library:NIST11.lib
SI:83 Formula:C5H8O3 CAS:28525-83-9 MolWeight:116 RetIndex:1045
CompName:2-Pentenoic acid, 4-hydroxy- $$ (2E)-4-Hydroxy-2-pentenoic acid # $$

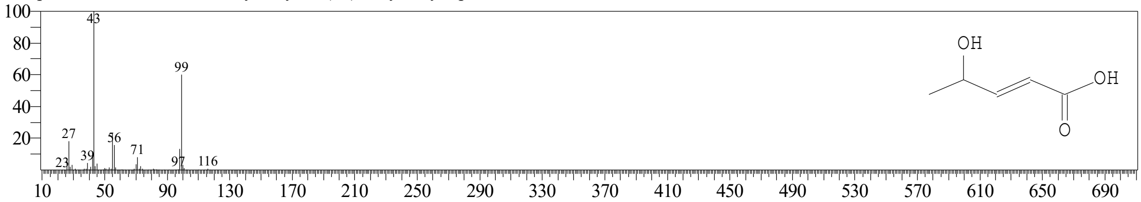

Hit#:3 Entry:26658 Library:NIST11.lib
SI:83 Formula:C7H13BO4 CAS:58163-59-0 MolWeight:172 RetIndex:0
CompName:1,3,2-Dioxaborolane-4-methanol, 2-ethyl-, acetate $$ (2-Ethyl-1,3,2-dioxaborolan-4-yl)methyl acetate # $$

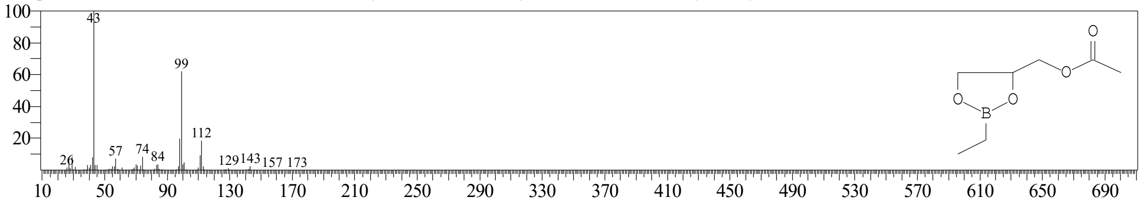

Hit#:4 Entry:19146 Library:NIST11.lib
SI:82 Formula:C7H10O4 CAS:57681-51-3 MolWeight:158 RetIndex:1226
CompName:2(3H)-Furanone, 5-(acetyloxy)dihydro-5-methyl- $$ 2-Methyl-5-oxotetrahydro-2-furanyl acetate # $$

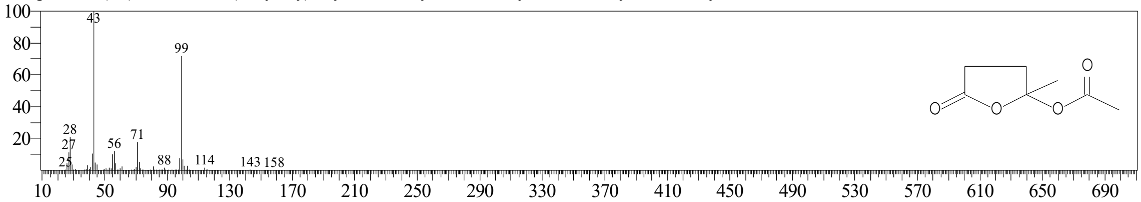

Hit#:5 Entry:1770 Library:NIST11.lib
SI:82 Formula:C2H5N5 CAS:38104-45-9 MolWeight:99 RetIndex:1130
CompName:3,4-Diamino-1,2,4(4H)-triazole $$ 3,4-Diamino-1,2,4-triazole $$ 4H-1,2,4-Triazole-3,4-diamine # $$

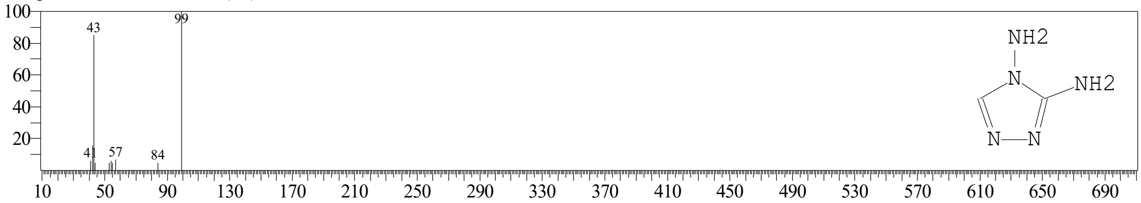

<< Target >>
Line#:57 R.Time:8.700(Scan#:941) MassPeaks:354
RawMode:Averaged 8.695-8.705(940-942) BasePeak:57.00(9125)
BG Mode:Calc. from Peak Group 1 - Event 1 Scan

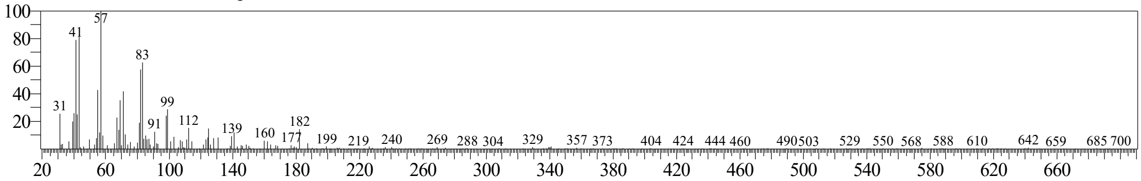

Hit#:1 Entry:91996 Library:NIST11.lib
SI:76 Formula:C19H38 CAS:13151-92-3 MolWeight:266 RetIndex:1909
CompName:Tridecane, 7-cyclohexyl- $$ 7-Cyclohexyltridecane $$ (1-Hexylheptyl)cyclohexane # $$ Cyclohexane, 1-hexylheptyl $$

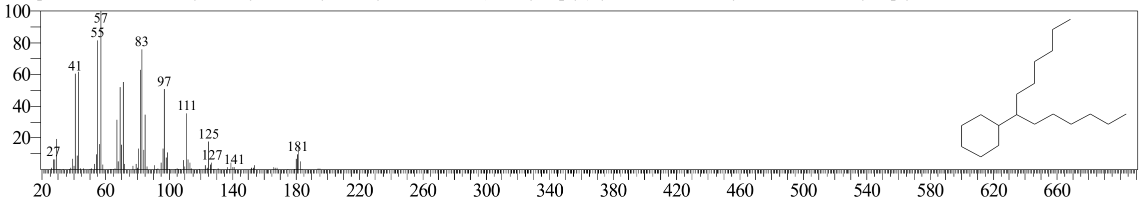

Hit#:2 Entry:91998 Library:NIST11.lib
SI:76 Formula:C19H38 CAS:13151-90-1 MolWeight:266 RetIndex:1909
CompName:Tridecane, 5-cyclohexyl- $$ (1-Butylnonyl)cyclohexane $$ Cyclohexane, 1-butylnonyl $$

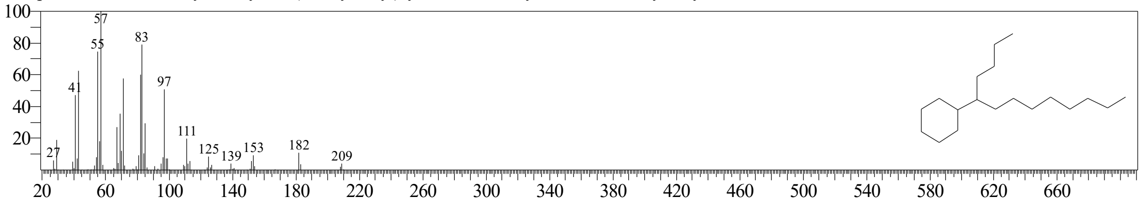

Hit#:3 Entry:81360 Library:NIST11.lib
SI:76 Formula:C18H36 CAS:13151-83-2 MolWeight:252 RetIndex:1810
CompName:Dodecane, 3-cyclohexyl- $$ (1-Ethyldecyl)cyclohexane # $$ Cyclohexane, 1-ethyldecyl $$

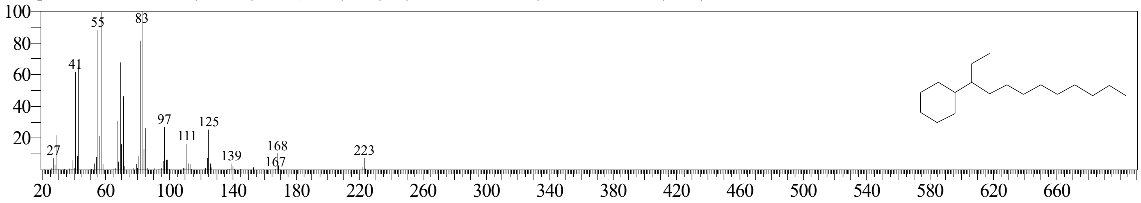

Hit#:4 Entry:92002 Library:NIST11.lib
SI:76 Formula:C19H38 CAS:13151-88-7 MolWeight:266 RetIndex:1909
CompName:Tridecane, 3-cyclohexyl- $$ (1-Ethylundecyl)cyclohexane $$ Cyclohexane, 1-ethylundecyl $$

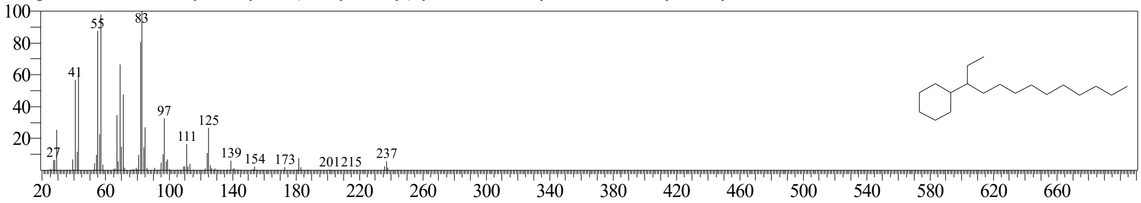

Hit#:5 Entry:70943 Library:NIST11.lib
SI:76 Formula:C17H34 CAS:13151-78-5 MolWeight:238 RetIndex:1710
CompName:Undecane, 3-cyclohexyl- $$ (1-Ethylnonyl)cyclohexane # $$ Cyclohexane, 1-ethylnonyl $$

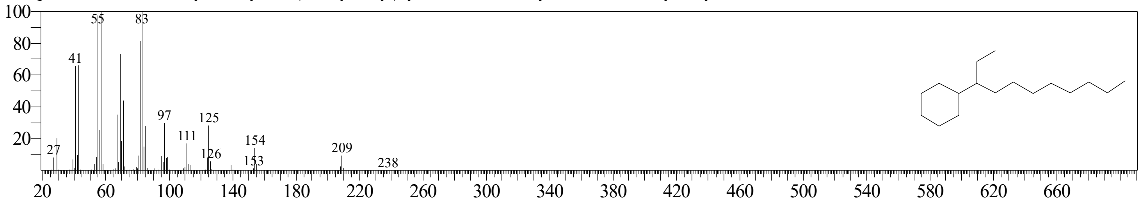

<< Target >>
Line#:58 R.Time:8.770(Scan#:955) MassPeaks:364
RawMode:Averaged 8.765-8.775(954-956) BasePeak:43.10(319544)
BG Mode:Calc. from Peak Group 1 - Event 1 Scan

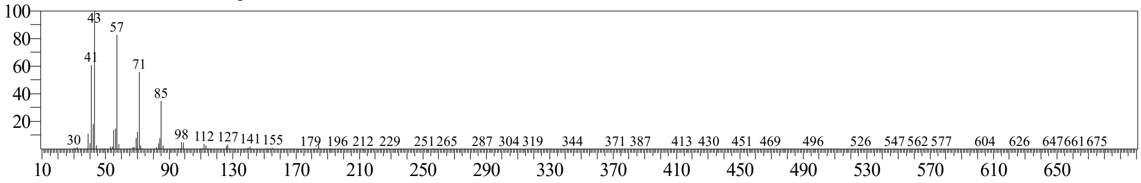

Hit#:1 Entry:18717 Library:NIST11.lib
SI:93 Formula:C11H24 CAS:6975-98-0 MolWeight:156 RetIndex:1051
CompName:Decane, 2-methyl- $$ 2-Methyldecane $$ n-C8H17CH(CH3)2 $$

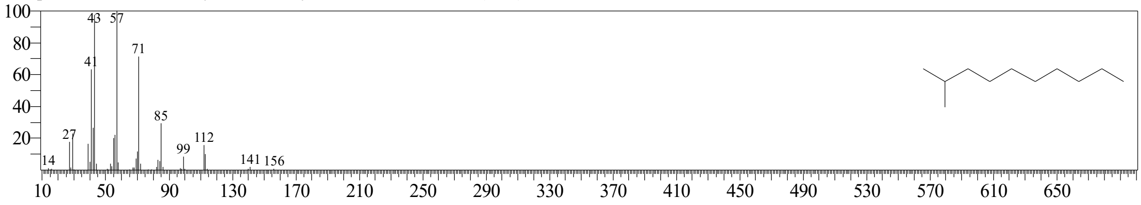

Hit#:2 Entry:26090 Library:NIST11.lib
SI:93 Formula:C12H26 CAS:7045-71-8 MolWeight:170 RetIndex:1150
CompName:Undecane, 2-methyl- $$ 2-Methylundecane $$

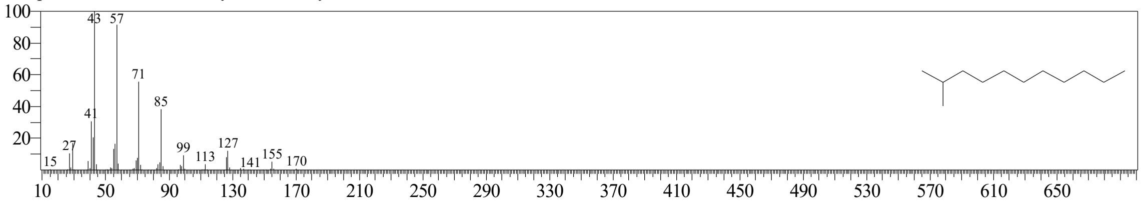

Hit#:3 Entry:26094 Library:NIST11.lib
SI:92 Formula:C12H26 CAS:1002-17-1 MolWeight:170 RetIndex:1086
CompName:Decane, 2,9-dimethyl- $$ 2,9-Dimethyldecane $$

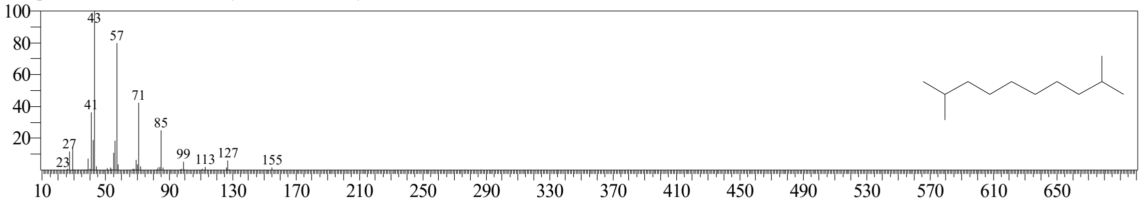

Hit#:4 Entry:34150 Library:NIST11.lib
SI:92 Formula:C13H28 CAS:629-50-5 MolWeight:184 RetIndex:1313
CompName:Tridecane $$ n-Tridecane $$ Tridecane, n- $$

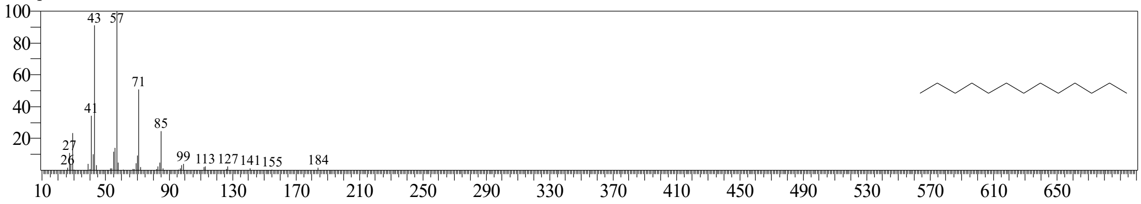

Hit#:5 Entry:18708 Library:NIST11.lib
SI:92 Formula:C11H24 CAS:62016-18-6 MolWeight:156 RetIndex:986
CompName:Octane, 5-ethyl-2-methyl- $$ 5-Ethyl-2-methyloctane # $$

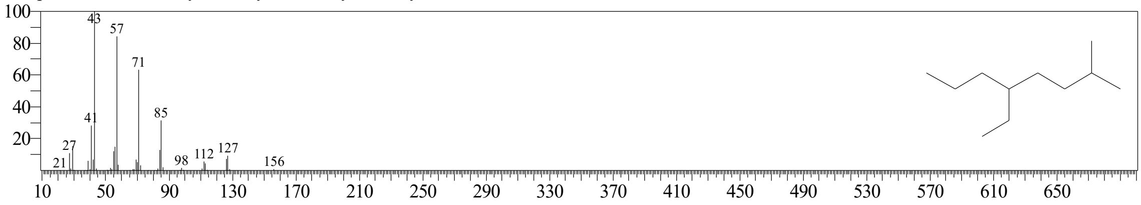

<< Target >>
Line#:59 R.Time:8.860(Scan#:973) MassPeaks:339
RawMode:Averaged 8.855-8.865(972-974) BasePeak:41.05(7510)
BG Mode:Calc. from Peak Group 1 - Event 1 Scan

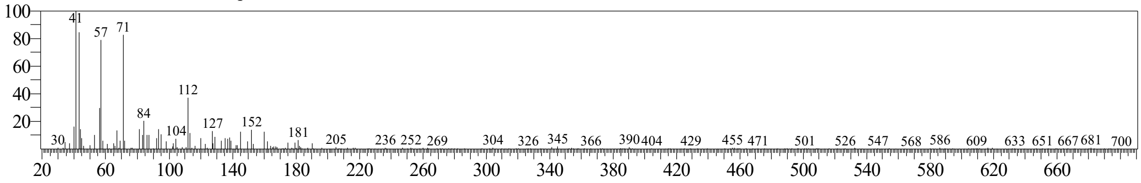

Hit#:1 Entry:17724 Library:NIST11.lib
SI:68 Formula:C11H22 CAS:13151-29-6 MolWeight:154 RetIndex:1041
CompName:1-Decene, 4-methyl- $$ 4-Methyl-1-decene # $$

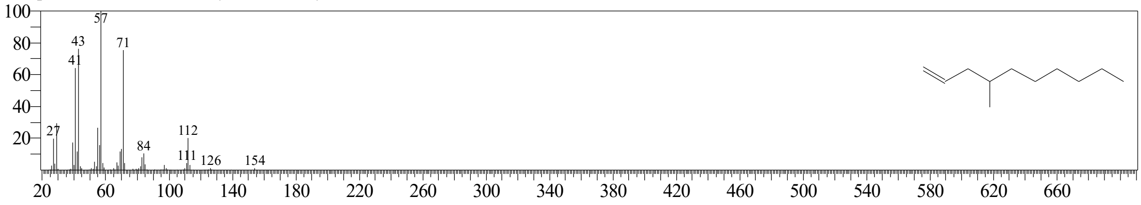

Hit#:2 Entry:18716 Library:NIST11.lib
SI:68 Formula:C11H24 CAS:2884-06-2 MolWeight:156 RetIndex:986
CompName:Nonane, 2,3-dimethyl- $$ 2,3-Dimethylnonane $$

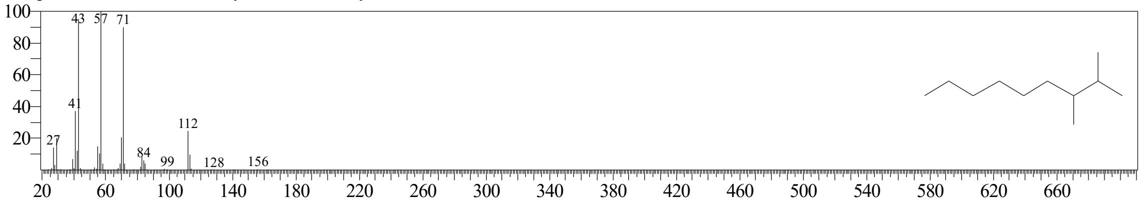

Hit#:3 Entry:26110 Library:NIST11.lib
SI:68 Formula:C12H26 CAS:62185-21-1 MolWeight:170 RetIndex:958
CompName:Octane, 3,4,5,6-tetramethyl- $$ 3,4,5,6-Tetramethyloctane # $$

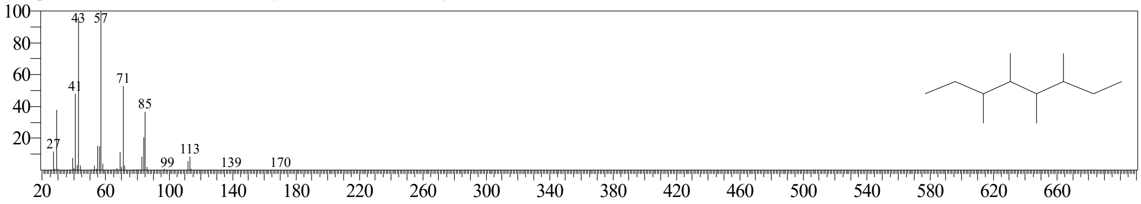

Hit#:4 Entry:18713 Library:NIST11.lib
SI:68 Formula:C11H24 CAS:62016-34-6 MolWeight:156 RetIndex:922
CompName:Octane, 2,3,7-trimethyl- $$ 2,3,7-Trimethyloctane # $$

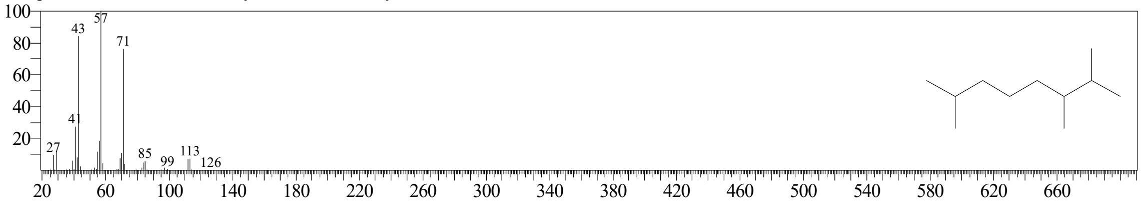

Hit#:5 Entry:42964 Library:NIST11.lib
SI:68 Formula:C14H30 CAS:26730-14-3 MolWeight:198 RetIndex:1349
CompName:Tridecane, 7-methyl- $$ 7-Methyltridecane $$

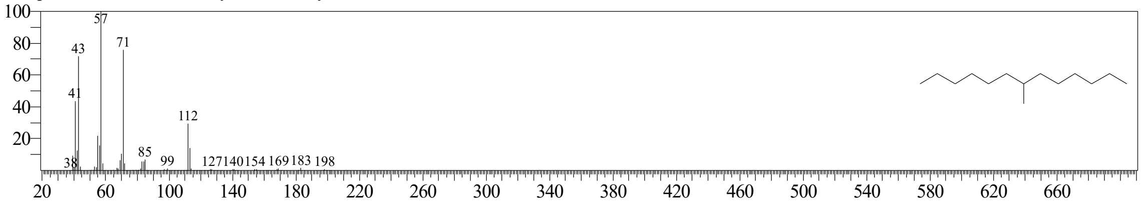

Qualitative Analysis Report

<< Target >>
Line#:60 R.Time:8.900(Scan#:981) MassPeaks:355
RawMode:Averaged 8.895-8.905(980-982) BasePeak:43.05(8331)
BG Mode:Calc. from Peak Group 1 - Event 1 Scan

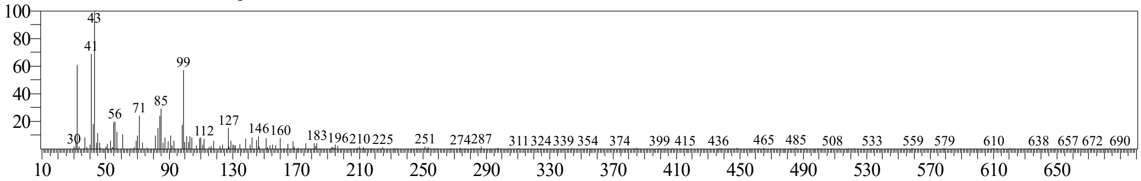

Hit#:1 Entry:63822 Library:NIST11.lib
SI:68 Formula:C14H28O2 CAS:0-00-0 MolWeight:228 RetIndex:1516
CompName:Hexanoic acid, 4-octyl ester $$ 1-Propylpentyl hexanoate # $$

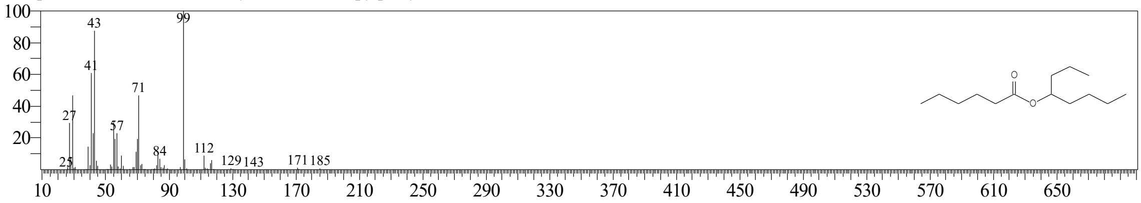

Hit#:2 Entry:35054 Library:NIST11.lib
SI:67 Formula:C10H22N2O CAS:28023-80-5 MolWeight:186 RetIndex:1410
CompName:N-Isopentyl-N-nitroso-pentylamine $$ Pentylamine, N-isopentyl-N-nitroso- $$ 1-Isopentyl-2-oxo-1-pentylhydrazine $$ N-(3-methylbutyl)-N-nitros

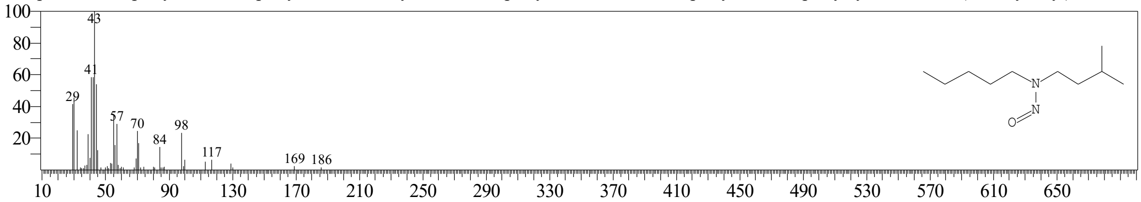

Hit#:3 Entry:82670 Library:NIST11.lib
SI:67 Formula:C16H30O2 CAS:180252-09-9 MolWeight:254 RetIndex:1769
CompName:Hexanoic acid, 9-decen-1-yl ester $$ 9-Decenyl hexanoate # $$

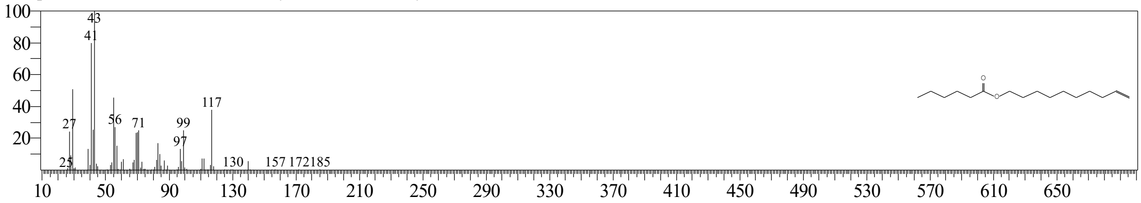

Hit#:4 Entry:18500 Library:NIST11.lib
SI:66 Formula:C9H16O2 CAS:39638-67-0 MolWeight:156 RetIndex:1245
CompName:trans-3-Methyl-4-octanolide $$ 5-Butyl-4-methyldihydro-2(3H)-furanone, trans- $$ trans-Whiskey lactone $$ trans-Whiskeylactone $$ (E)-Whiskyl

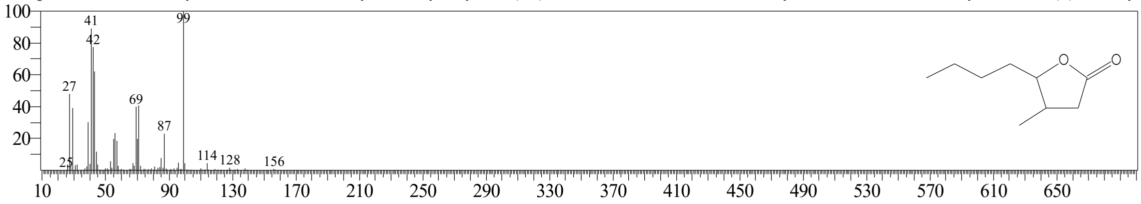

Hit#:5 Entry:2320 Library:NIST11.lib
SI:66 Formula:C6H14O CAS:19550-30-2 MolWeight:102 RetIndex:732
CompName:1-Butanol, 2,3-dimethyl- $$ 2,3-Dimethyl-1-butanol $$ 2,3-Methylbutanol $$ 2,3-Dimethylbutan-1-ol $$

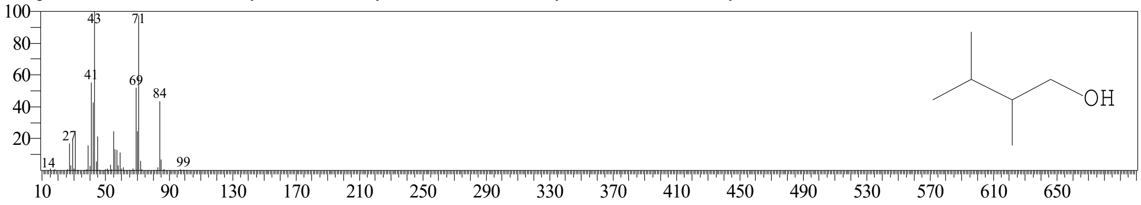

<< Target >>
Line#:61 R.Time:8.970(Scan#:995) MassPeaks:374
RawMode:Averaged 8.965-8.975(994-996) BasePeak:32.10(5819)
BG Mode:Calc. from Peak Group 1 - Event 1 Scan

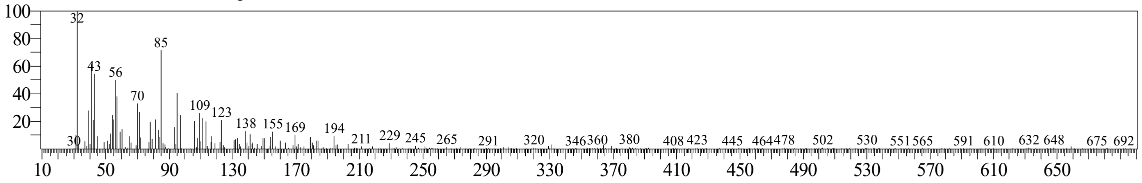

Hit#:1 Entry:137860 Library:NIST11.lib
SI:63 Formula:C12H24Br2 CAS:55334-42-4 MolWeight:326 RetIndex:1742
CompName:Dodecane, 1,2-dibromo- $$ 1,2-Dibromododecane $$

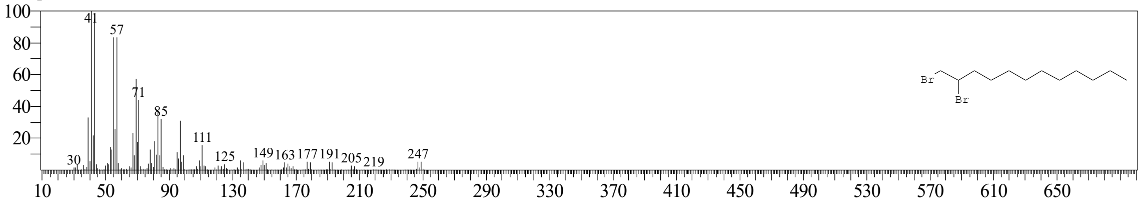

Hit#:2 Entry:96894 Library:NIST11.lib
SI:63 Formula:C17H33Cl CAS:56554-78-0 MolWeight:272 RetIndex:1944
CompName:7-Heptadecene, 1-chloro- $$ (7E)-1-Chloro-7-heptadecene # $$

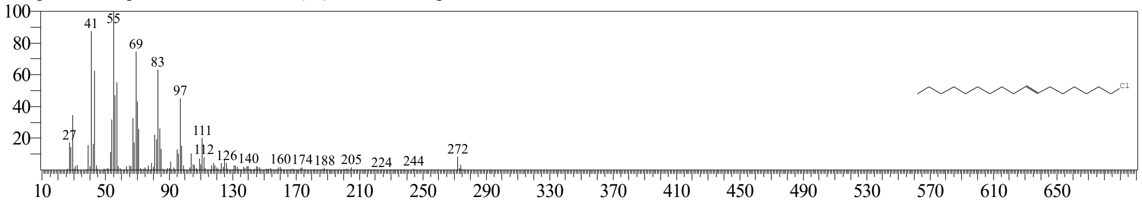

Hit#:3 Entry:12097 Library:NIST11.lib
SI:63 Formula:C8H14O2 CAS:4723-10-8 MolWeight:142 RetIndex:1075
CompName:1,5-Hexadiene-3,4-diol, 2,5-dimethyl- $$ 2,5-Dimethyl-1,5-hexadiene-3,4-diol $$

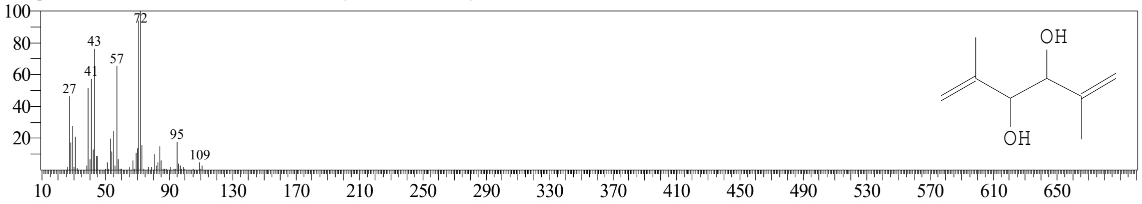

Hit#:4 Entry:41643 Library:NIST11.lib
SI:63 Formula:C14H28 CAS:41446-62-2 MolWeight:196 RetIndex:1421
CompName:5-Tetradecene, (Z)- $$ (5Z)-5-Tetradecene $$ cis-5-Tetradecene $$ (Z)-5-Tetradecene $$

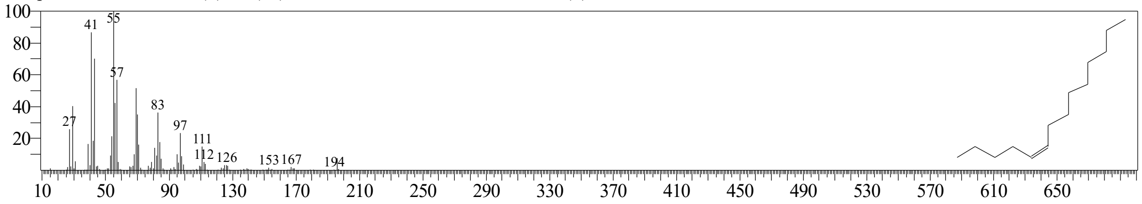

Hit#:5 Entry:13115 Library:NIST11.lib
SI:63 Formula:C9H20O CAS:18450-74-3 MolWeight:144 RetIndex:1030
CompName:1-Heptanol, 2,4-dimethyl-, (2S,4R)-(-)- $$ 2,4-Dimethyl-1-heptanol # $$

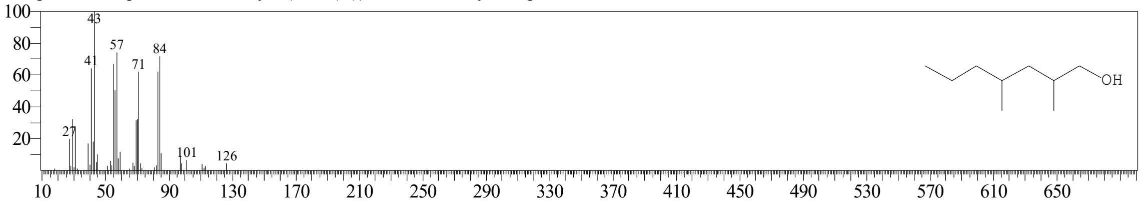

<< Target >>
Line#:62 R.Time:9.025(Scan#:1006) MassPeaks:343
RawMode:Averaged 9.020-9.030(1005-1007) BasePeak:43.00(8224)
BG Mode:Calc. from Peak Group 1 - Event 1 Scan

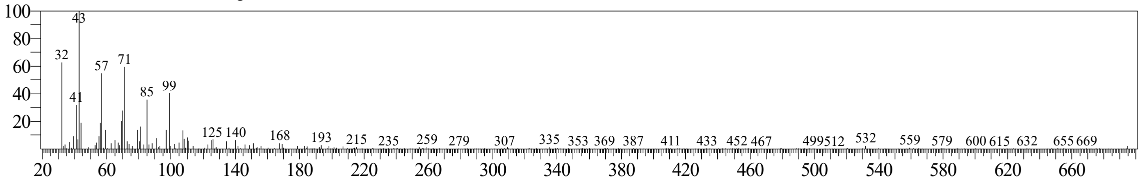

Hit#:1 Entry:34131 Library:NIST11.lib
SI:75 Formula:C13H28 CAS:17301-25-6 MolWeight:184 RetIndex:1185
CompName:Undecane, 2,8-dimethyl- $$ 2,8-Dimethylundecane # $$

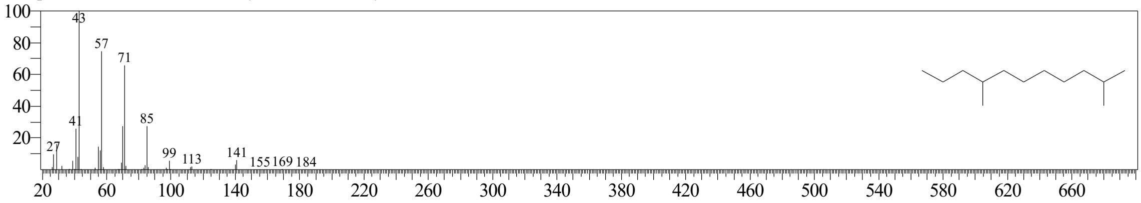

Hit#:2 Entry:18710 Library:NIST11.lib
SI:74 Formula:C11H24 CAS:62016-19-7 MolWeight:156 RetIndex:986
CompName:Octane, 6-ethyl-2-methyl- $$ 6-Ethyl-2-methyloctane # $$

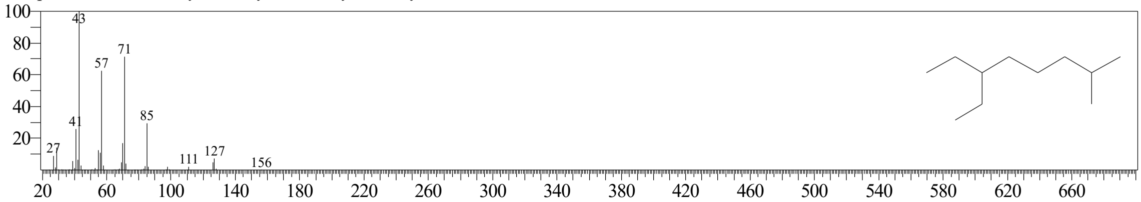

Hit#:3 Entry:42952 Library:NIST11.lib
SI:74 Formula:C14H30 CAS:26730-12-1 MolWeight:198 RetIndex:1349
CompName:Tridecane, 4-methyl- $$ 4-Methyltridecane $$

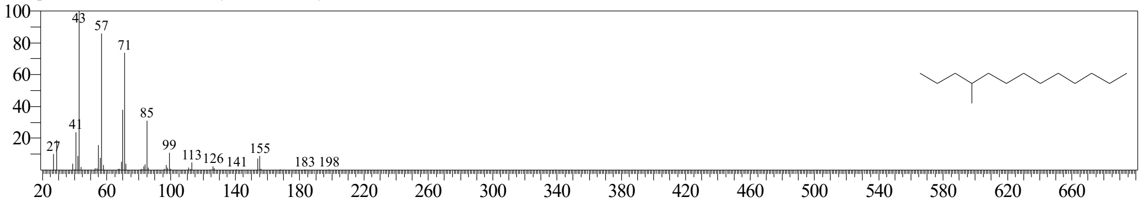

Hit#:4 Entry:24840 Library:NIST11.lib
SI:74 Formula:C12H24 CAS:74630-39-0 MolWeight:168 RetIndex:1140
CompName:1-Undecene, 4-methyl- $$ 4-Methyl-1-undecene # $$

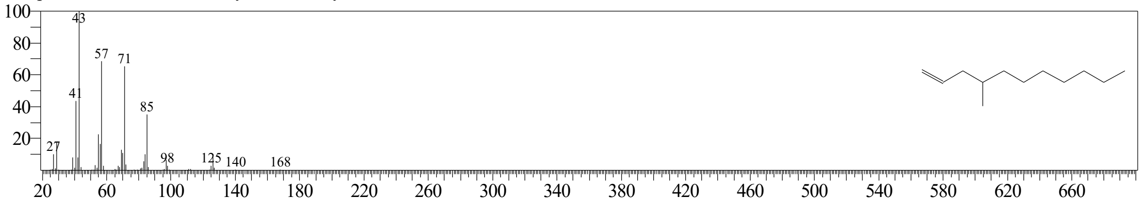

Hit#:5 Entry:104531 Library:NIST11.lib
SI:74 Formula:C20H42 CAS:1560-86-7 MolWeight:282 RetIndex:1945
CompName:Nonadecane, 2-methyl- $$ 2-Methylnonadecane $$

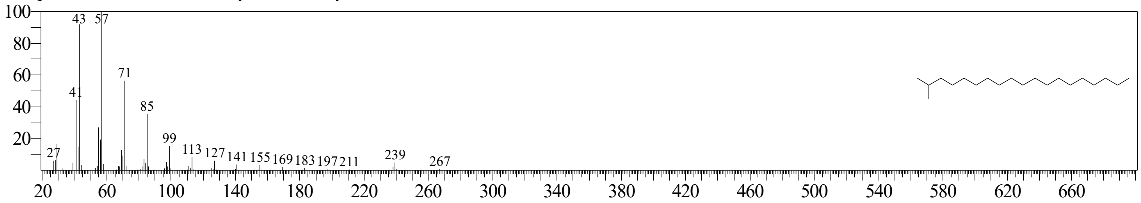

<< Target >>
Line#:63 R.Time:9.080(Scan#:1017) MassPeaks:372
RawMode:Averaged 9.075-9.085(1016-1018) BasePeak:32.10(7963)
BG Mode:Calc. from Peak Group 1 - Event 1 Scan

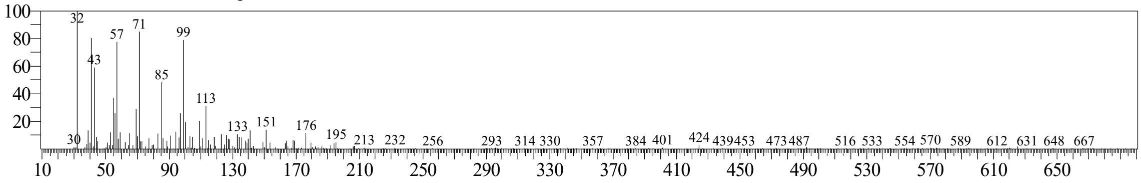

Hit#:1 Entry:72488 Library:NIST11.lib
SI:70 Formula:C17H36 CAS:6008-17-9 MolWeight:240 RetIndex:1627
CompName:5,5-Dibutylnonane

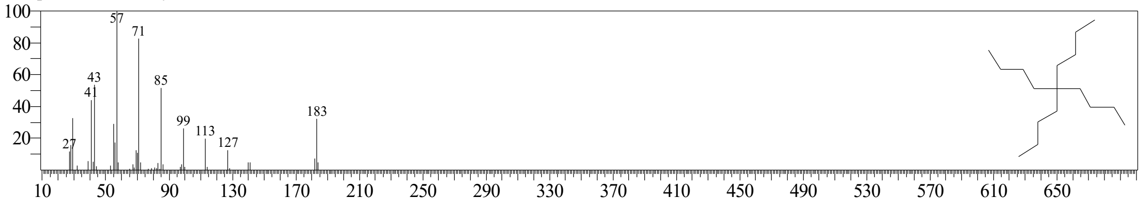

Hit#:2 Entry:93613 Library:NIST11.lib
SI:70 Formula:C19H40 CAS:1560-88-9 MolWeight:268 RetIndex:1846
CompName:Octadecane, 2-methyl- $$ 2-Methyloctadecane $$

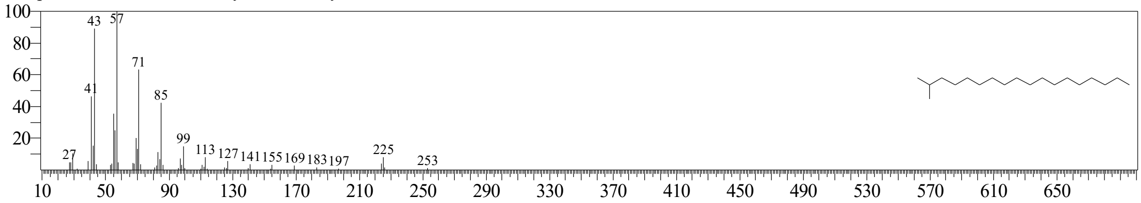

Hit#:3 Entry:99570 Library:NIST11.lib
SI:69 Formula:C14H29Br CAS:74036-95-6 MolWeight:276 RetIndex:1645
CompName:2-Bromotetradecane

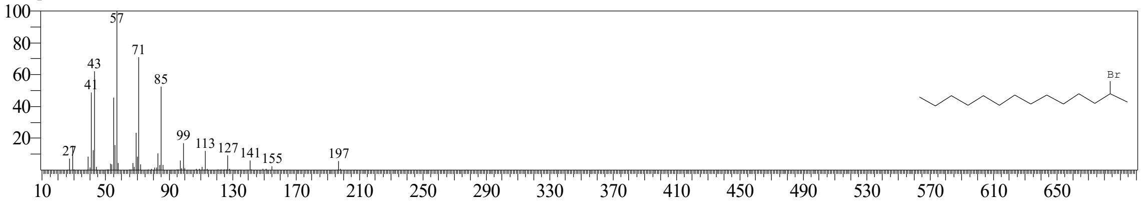

Hit#:4 Entry:104539 Library:NIST11.lib
SI:69 Formula:C20H42 CAS:56862-62-5 MolWeight:282 RetIndex:1945
CompName:10-Methylnonadecane $$ Nonadecane, 10-methyl $$

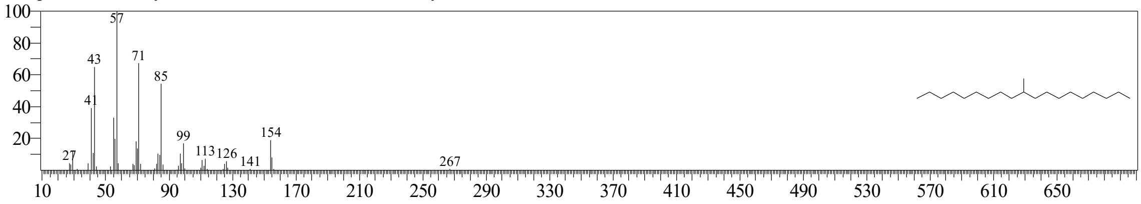

Hit#:5 Entry:34191 Library:NIST11.lib
SI:68 Formula:C13H28 CAS:17312-72-0 MolWeight:184 RetIndex:1229
CompName:4,4-Dipropylheptane

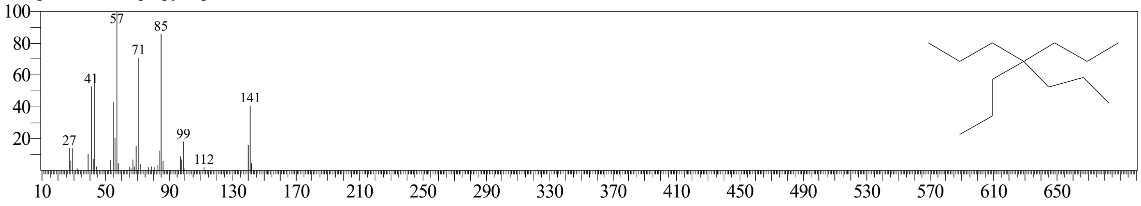

<< Target >>
Line#:64 R.Time:9.145(Scan#:1030) MassPeaks:327
RawMode:Averaged 9.140-9.150(1029-1031) BasePeak:32.10(11536)
BG Mode:Calc. from Peak Group 1 - Event 1 Scan

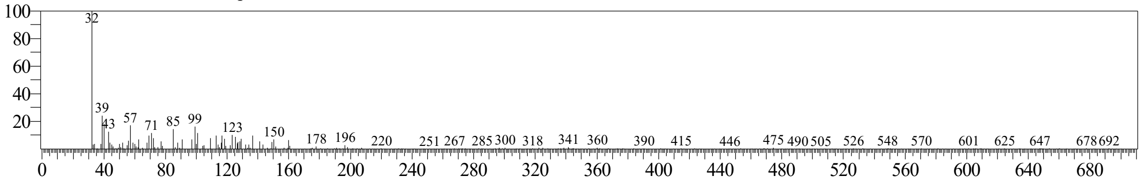

Hit#:1 Entry:95054 Library:NIST11.lib
SI:58 Formula:C16H34N2O CAS:2619-88-7 MolWeight:270 RetIndex:2255
CompName:16-Hexadecanoyl hydrazide $$ Hexadecanohydrazide # $$

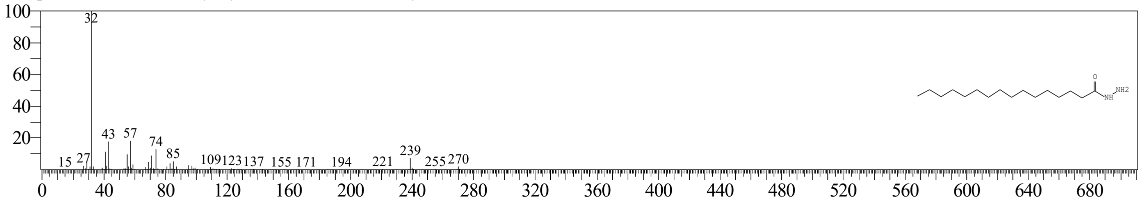

Hit#:2 Entry:117059 Library:NIST11.lib
SI:57 Formula:C18H38N2O CAS:4130-54-5 MolWeight:298 RetIndex:2454
CompName:Stearic acid hydrazide $$ Stearic hydrazide $$ Octadecanoic acid, hydrazide $$ Stearohydrazide $$ Stearoylhydrazine $$ Octadecanohydrazide # $$

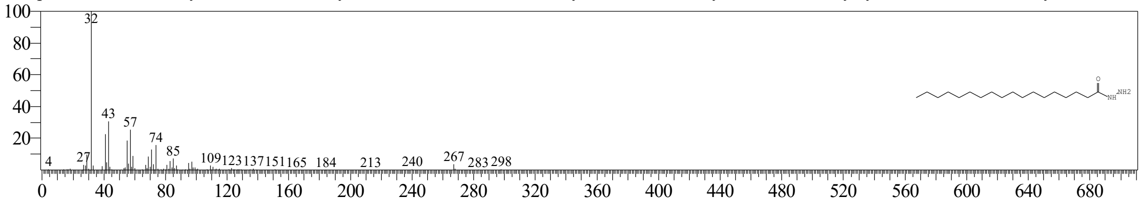

Hit#:3 Entry:8281 Library:NIST11.lib
SI:57 Formula:C3H8N4O2 CAS:3815-86-9 MolWeight:132 RetIndex:1606
CompName:Malonic acid, dihydrazide $$ Propanedioyl dihydrazide $$ Malonhydrazide $$ Malonic acid hydrazide $$ Malonic dihydrazide $$ Malonyl dihydra

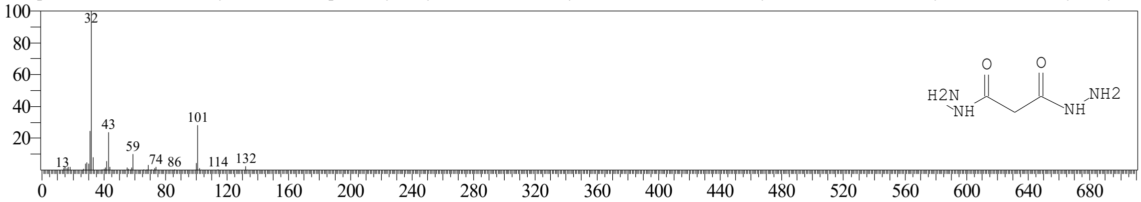

Hit#:4 Entry:8355 Library:NIST11.lib
SI:57 Formula:C5H12N2O2 CAS:0-00-0 MolWeight:132 RetIndex:1253
CompName:3-Hydroxy-3-methyl-butyric acid, hydrazide $$ 3-Hydroxy-3-methylbutanohydrazide # $$

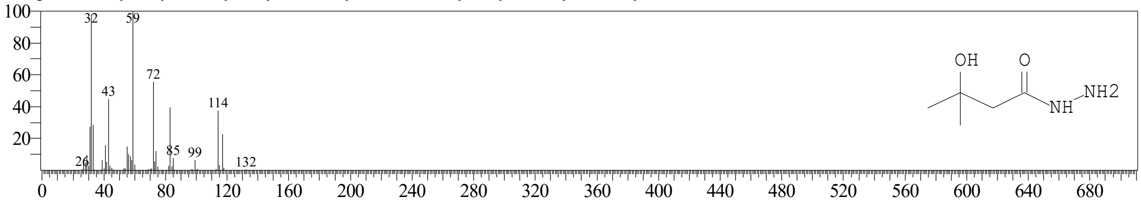

Hit#:5 Entry:1772 Library:NIST11.lib
SI:56 Formula:C3H5N3O CAS:140-87-4 MolWeight:99 RetIndex:1208
CompName:Cyacetacide $$ Cyanoacetohydrazide $$ 2-Cyanoacetylhydrazide $$ Cyanoacethydrazide $$ Cyanoacetic acid hydrazide $$ Acetic acid, cyano-, hyd

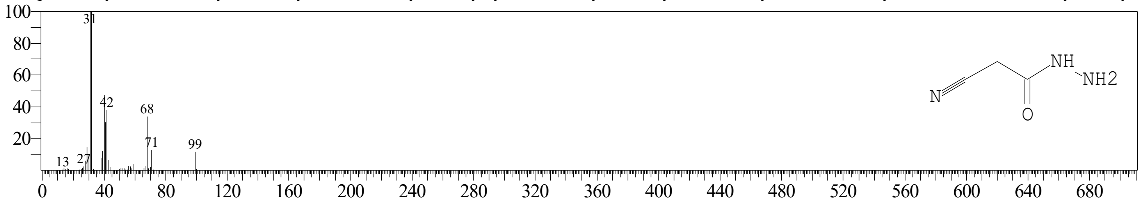

Qualitative Analysis Report

<< Target >>
Line#:65 R.Time:9.250(Scan#:1051) MassPeaks:374
RawMode:Averaged 9.245-9.255(1050-1052) BasePeak:43.05(29033)
BG Mode:Calc. from Peak Group 1 - Event 1 Scan

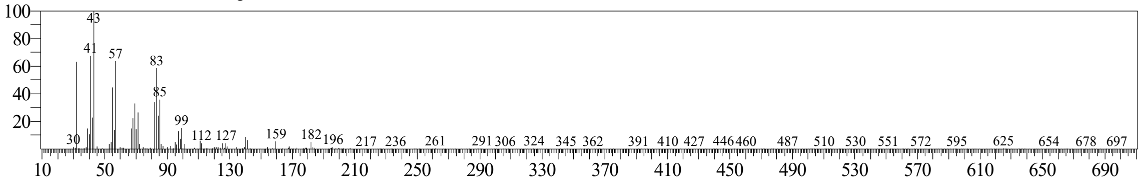

Hit#:1 Entry:164497 Library:NIST11.lib
SI:82 Formula:C26H52 CAS:6703-82-8 MolWeight:364 RetIndex:2649
CompName:Cyclopentane, heneicosyl- $$ Heneicosane, 1-cyclopentyl- $$ n-Heneicosylcyclopentane $$ Henicosylcyclopentane # $$

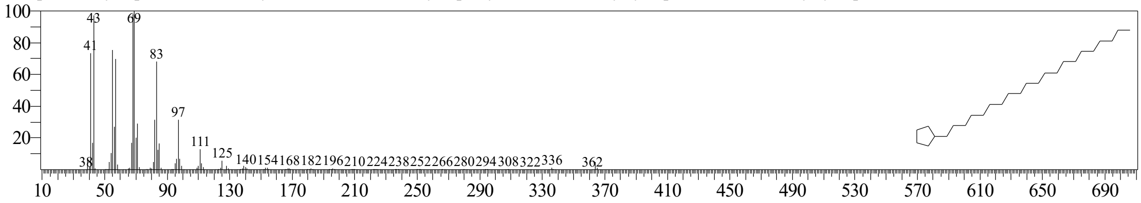

Hit#:2 Entry:92904 Library:NIST11.lib
SI:82 Formula:C12H22Cl2O2 CAS:83005-00-9 MolWeight:268 RetIndex:1727
CompName:Dichloroacetic acid, decyl ester $$ Decyl dichloroacetate # $$

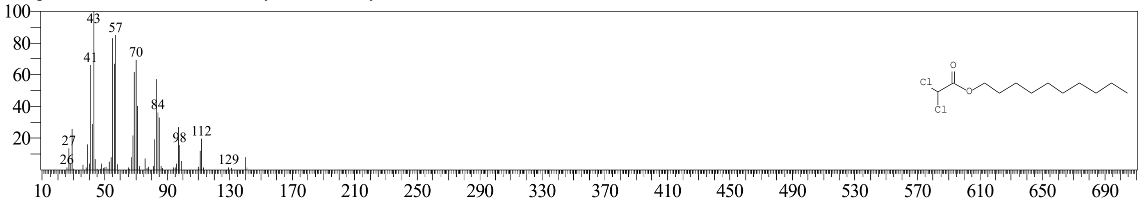

Hit#:3 Entry:24902 Library:NIST11.lib
SI:82 Formula:C12H24 CAS:55170-80-4 MolWeight:168 RetIndex:1117
CompName:1-Decene, 2,4-dimethyl- $$ 2,4-Dimethyl-1-decene # $$

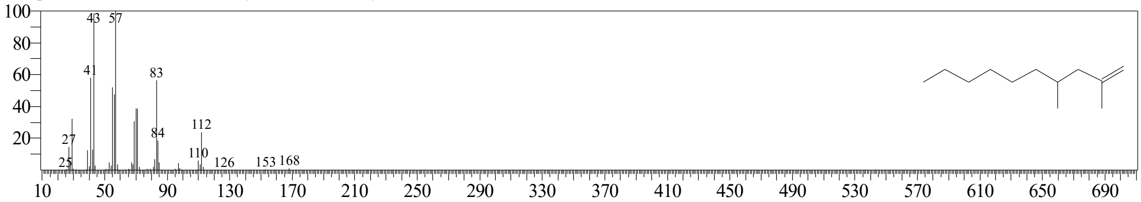

Hit#:4 Entry:24855 Library:NIST11.lib
SI:82 Formula:C12H24 CAS:74663-85-7 MolWeight:168 RetIndex:1216
CompName:Cyclopropane, nonyl- $$ Nonylcyclopropane # $$ n-Nonyl-cyclopropane $$

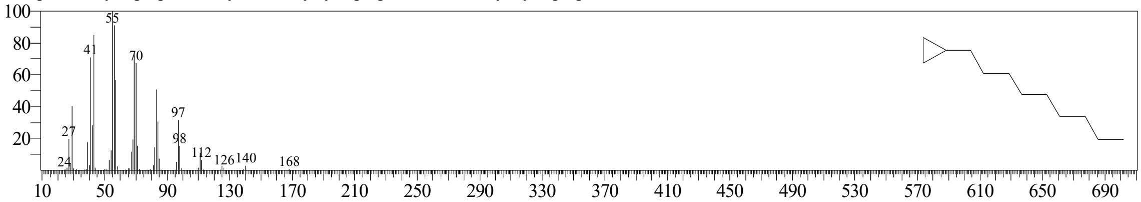

Hit#:5 Entry:53721 Library:NIST11.lib
SI:81 Formula:C14H30O CAS:112-72-1 MolWeight:214 RetIndex:1656
CompName:1-Tetradecanol $$ n-Tetradecan-1-ol $$ n-Tetradecanol $$ n-Tetradecyl alcohol $$ Alfol 14 $$ Lanette K $$ Lanette Wax KS $$ Loxanol V $$ Myris

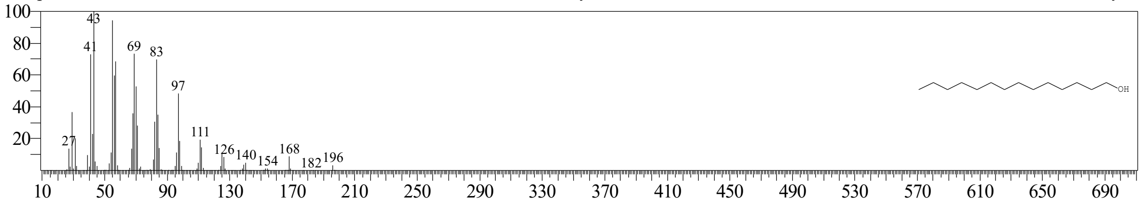

Qualitative Analysis Report

<< Target >>
Line#:66 R.Time:9.300(Scan#:1061) MassPeaks:332
RawMode:Averaged 9.295-9.305(1060-1062) BasePeak:43.05(26646)
BG Mode:Calc. from Peak Group 1 - Event 1 Scan

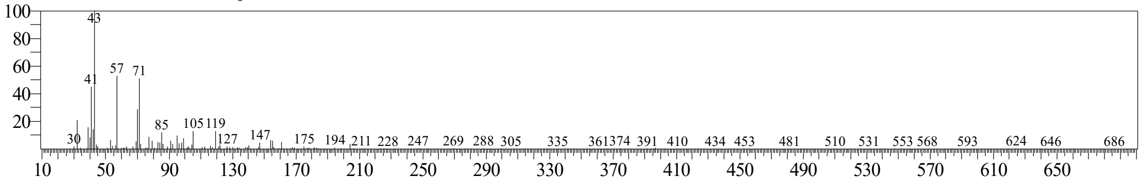

Hit#:1 Entry:26097 Library:NIST11.lib
SI:79 Formula:C12H26 CAS:2980-69-0 MolWeight:170 RetIndex:1150
CompName:Undecane, 4-methyl- $$ 4-Methylundecane $$

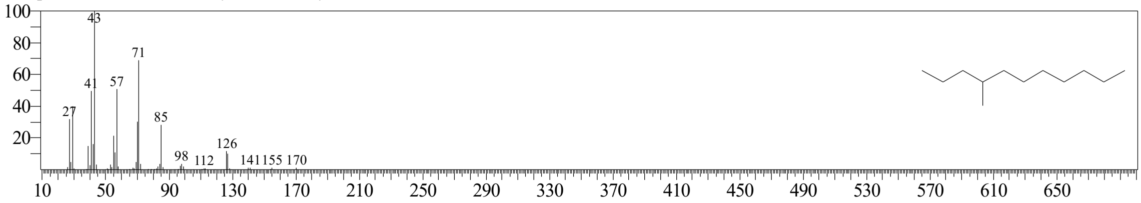

Hit#:2 Entry:34145 Library:NIST11.lib
SI:79 Formula:C13H28 CAS:17301-33-6 MolWeight:184 RetIndex:1185
CompName:Undecane, 4,8-dimethyl- $$ 4,8-Dimethylundecane # $$

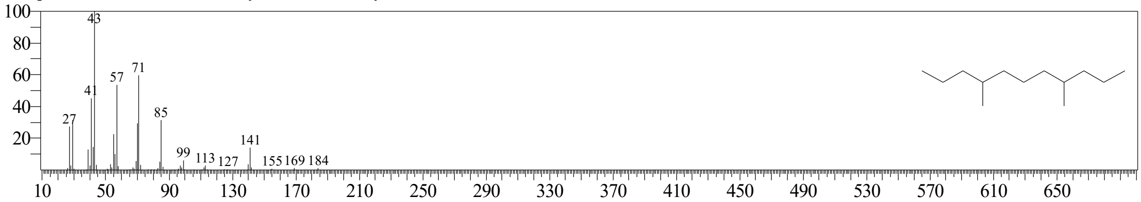

Hit#:3 Entry:34131 Library:NIST11.lib
SI:78 Formula:C13H28 CAS:17301-25-6 MolWeight:184 RetIndex:1185
CompName:Undecane, 2,8-dimethyl- $$ 2,8-Dimethylundecane # $$

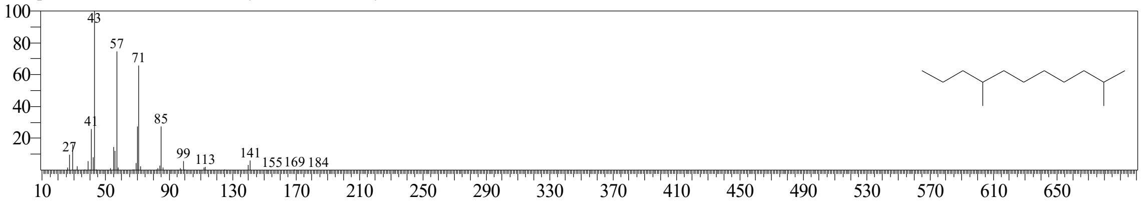

Hit#:4 Entry:26098 Library:NIST11.lib
SI:77 Formula:C12H26 CAS:52670-34-5 MolWeight:170 RetIndex:958
CompName:Octane, 2,3,6,7-tetramethyl- $$ 2,3,6,7-Tetramethyloctane # $$

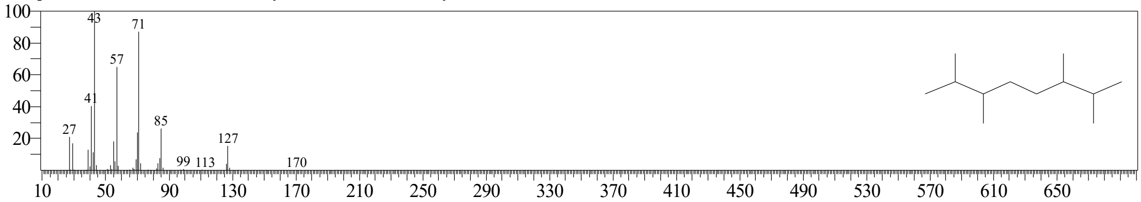

Hit#:5 Entry:4239 Library:NIST11.lib
SI:77 Formula:C8H18 CAS:563-16-6 MolWeight:114 RetIndex:732
CompName:Hexane, 3,3-dimethyl- $$ 3,3-Dimethylhexane $$

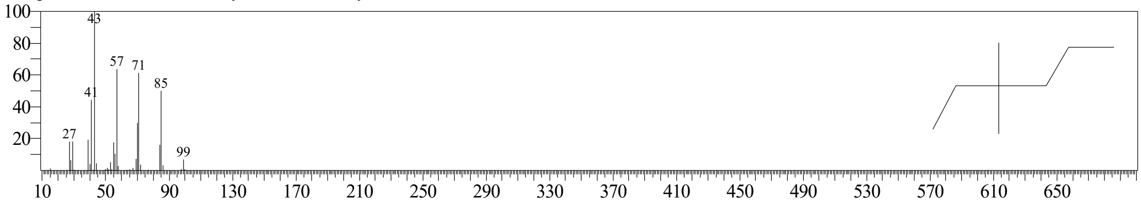

<< Target >>
Line#:67 R.Time:9.360(Scan#:1073) MassPeaks:348
RawMode:Averaged 9.355-9.365(1072-1074) BasePeak:43.05(46556)
BG Mode:Calc. from Peak Group 1 - Event 1 Scan

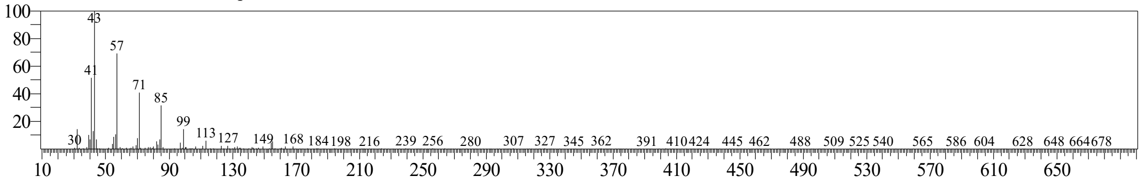

Hit#:1 Entry:42960 Library:NIST11.lib
SI:87 Formula:C14H30 CAS:1560-96-9 MolWeight:198 RetIndex:1349
CompName:Tridecane, 2-methyl- $$ 2-Methyltridecane $$ 2-Methyl-n-tridecane $$

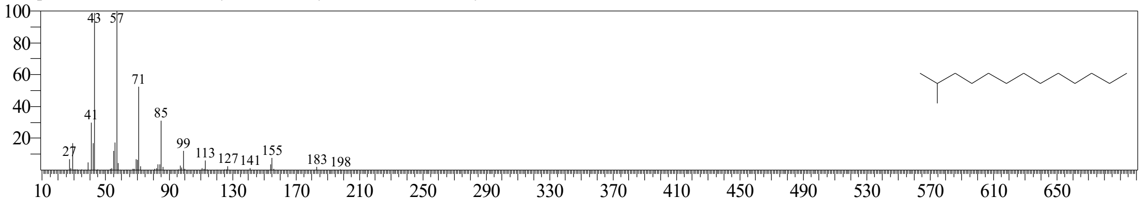

Hit#:2 Entry:26094 Library:NIST11.lib
SI:86 Formula:C12H26 CAS:1002-17-1 MolWeight:170 RetIndex:1086
CompName:Decane, 2,9-dimethyl- $$ 2,9-Dimethyldecane $$

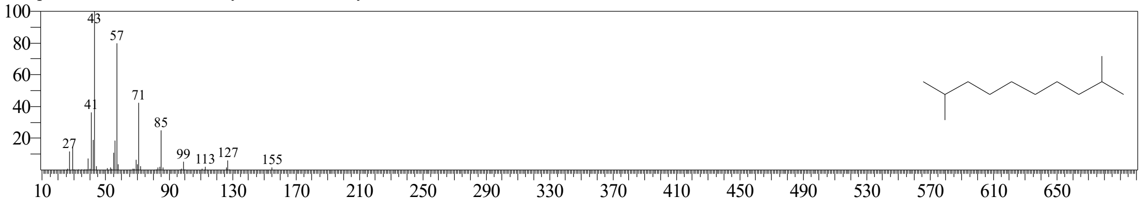

Hit#:3 Entry:62478 Library:NIST11.lib
SI:86 Formula:C16H34 CAS:1560-93-6 MolWeight:226 RetIndex:1548
CompName:Pentadecane, 2-methyl- $$ 2-Methylpentadecane $$

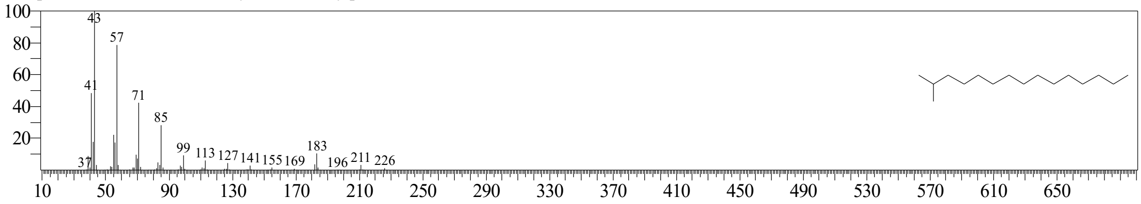

Hit#:4 Entry:34138 Library:NIST11.lib
SI:86 Formula:C13H28 CAS:1560-97-0 MolWeight:184 RetIndex:1249
CompName:Dodecane, 2-methyl- $$ 11-Methyldodecane $$ 2-Methyldodecane $$

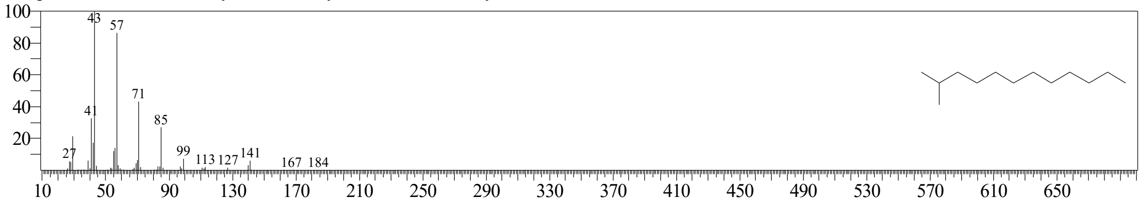

Hit#:5 Entry:103707 Library:NIST11.lib
SI:86 Formula:C11H23I CAS:4282-44-4 MolWeight:282 RetIndex:1529
CompName:1-Iodoundecane $$ Undecane, 1-iodo- $$

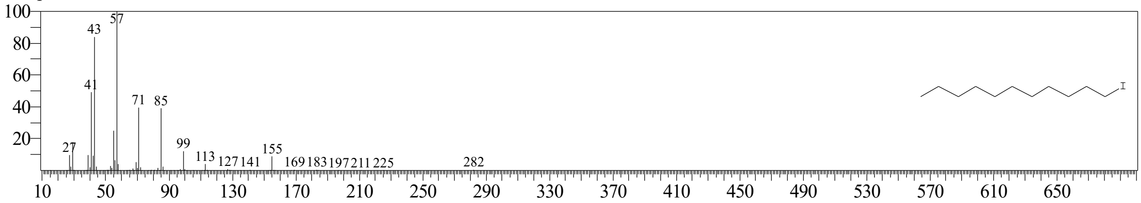

<< Target >>
Line#:68 R.Time:9.420(Scan#:1085) MassPeaks:350
RawMode:Averaged 9.415-9.425(1084-1086) BasePeak:57.10(20299)
BG Mode:Calc. from Peak Group 1 - Event 1 Scan

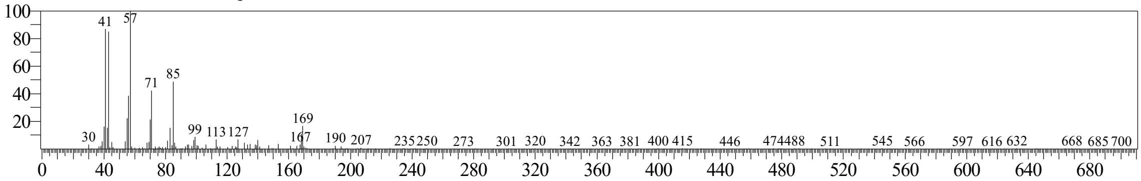

Hit#:1 Entry:52481 Library:NIST11.lib
SI:83 Formula:C15H32 CAS:55030-62-1 MolWeight:212 RetIndex:1384
CompName:Tridecane, 4,8-dimethyl- $$ 4,8-Dimethyltridecane # $$

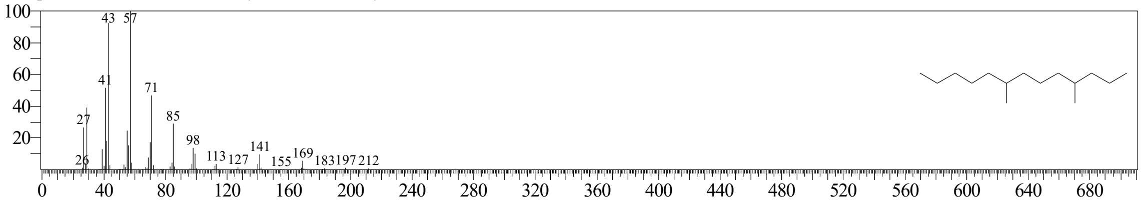

Hit#:2 Entry:52484 Library:NIST11.lib
SI:83 Formula:C15H32 CAS:1560-95-8 MolWeight:212 RetIndex:1448
CompName:Tetradecane, 2-methyl- $$ 2-Methyltetradecane $$

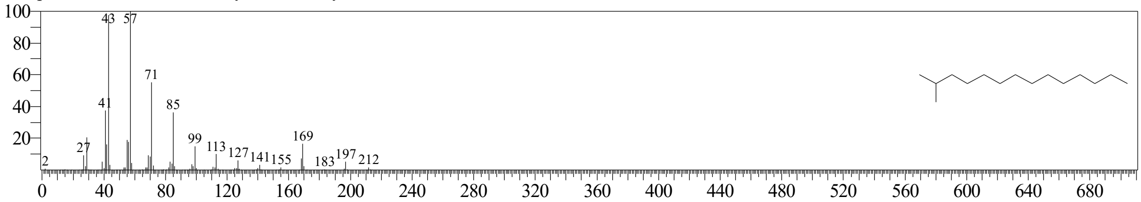

Hit#:3 Entry:114729 Library:NIST11.lib
SI:83 Formula:C12H25I CAS:73105-67-6 MolWeight:296 RetIndex:1564
CompName:1-Iodo-2-methylundecane

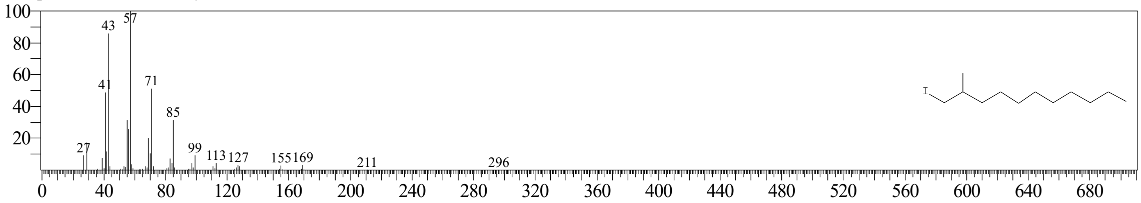

Hit#:4 Entry:82861 Library:NIST11.lib
SI:82 Formula:C18H38 CAS:593-45-3 MolWeight:254 RetIndex:1810
CompName:Octadecane $$ n-Octadecane $$ Octadecan $$

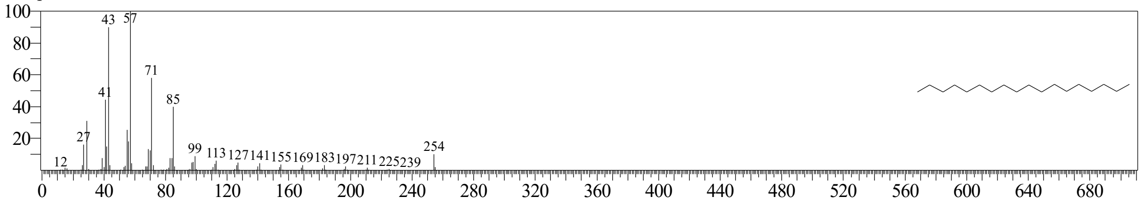

Hit#:5 Entry:115539 Library:NIST11.lib
SI:82 Formula:C21H44 CAS:54833-23-7 MolWeight:296 RetIndex:2045
CompName:Eicosane, 10-methyl- $$ 10-Methylicosane # $$ 10-Methyleicosane $$

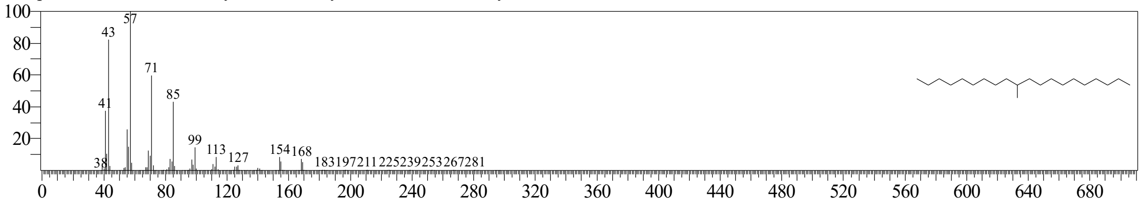

Qualitative Analysis Report

<< Target >>
Line#:69 R.Time:9.455(Scan#:1092) MassPeaks:377
RawMode:Averaged 9.450-9.460(1091-1093) BasePeak:71.10(44646)
BG Mode:Calc. from Peak Group 1 - Event 1 Scan

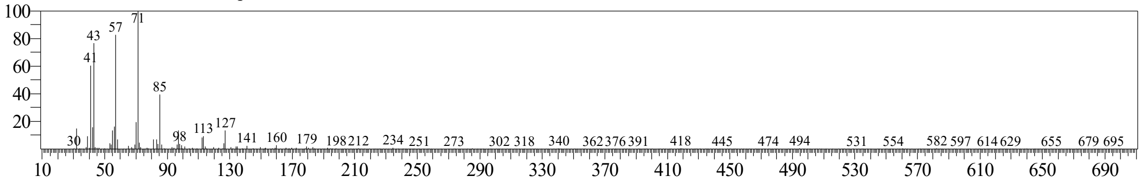

Hit#:1 Entry:52487 Library:NIST11.lib
SI:89 Formula:C15H32 CAS:31295-56-4 MolWeight:212 RetIndex:1320
CompName:Dodecane, 2,6,11-trimethyl- $$ 2,6,11-Trimethyldodecane $$

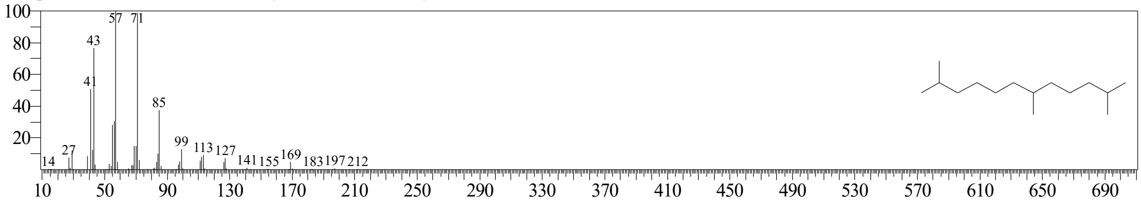

Hit#:2 Entry:34198 Library:NIST11.lib
SI:88 Formula:C13H28 CAS:17312-63-9 MolWeight:184 RetIndex:1249
CompName:Nonane, 5-butyl- $$ 5-Butylnonane $$ 5-n-Butylnonane $$

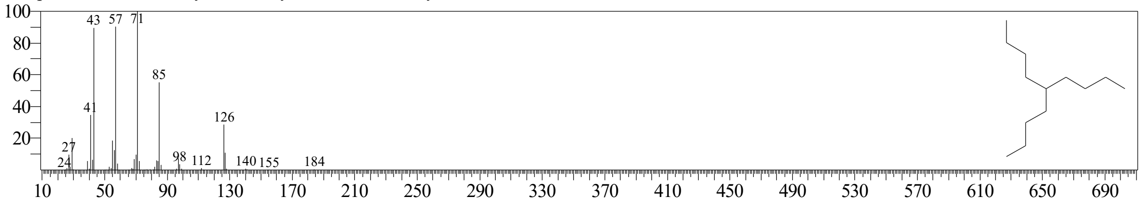

Hit#:3 Entry:18725 Library:NIST11.lib
SI:88 Formula:C11H24 CAS:13151-34-3 MolWeight:156 RetIndex:1051
CompName:Decane, 3-methyl- $$ 3-Methyldecane $$ 2-Ethylnonane $$

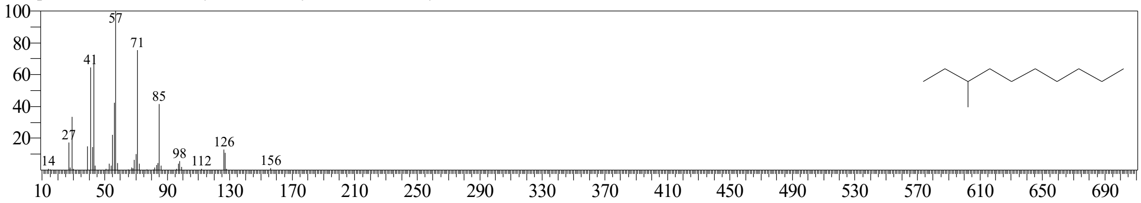

Hit#:4 Entry:26089 Library:NIST11.lib
SI:88 Formula:C12H26 CAS:17312-54-8 MolWeight:170 RetIndex:1086
CompName:Decane, 3,7-dimethyl- $$ 3,7-Dimethyldecane # $$

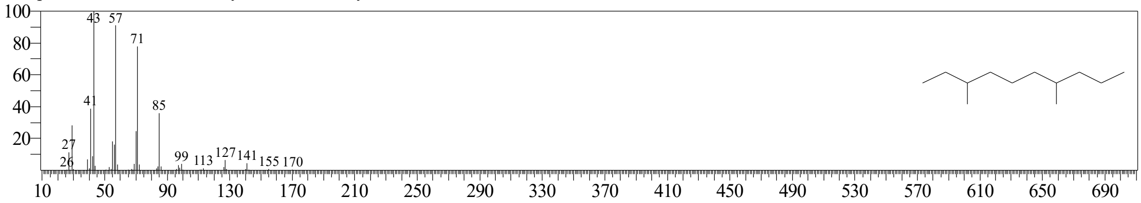

Hit#:5 Entry:34188 Library:NIST11.lib
SI:88 Formula:C13H28 CAS:17312-74-2 MolWeight:184 RetIndex:1229
CompName:Decane, 5-ethyl-5-methyl- $$ 5-Ethyl-5-methyldecane # $$

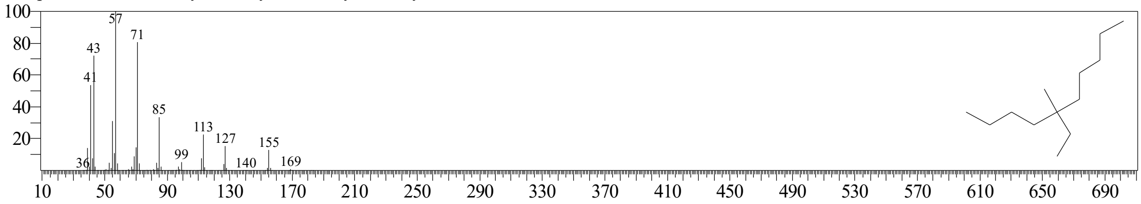

<< Target >>
Line#:70 R.Time:9.590(Scan#:1119) MassPeaks:397
RawMode:Averaged 9.585-9.595(1118-1120) BasePeak:105.10(56920)
BG Mode:Calc. from Peak Group 1 - Event 1 Scan

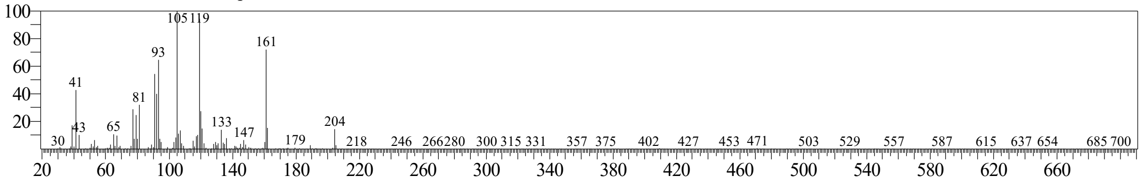

Hit#:1 Entry:46735 Library:NIST11.lib
SI:91 Formula:C15H24 CAS:0-00-0 MolWeight:204 RetIndex:1221
CompName:.alfa.-Copaene

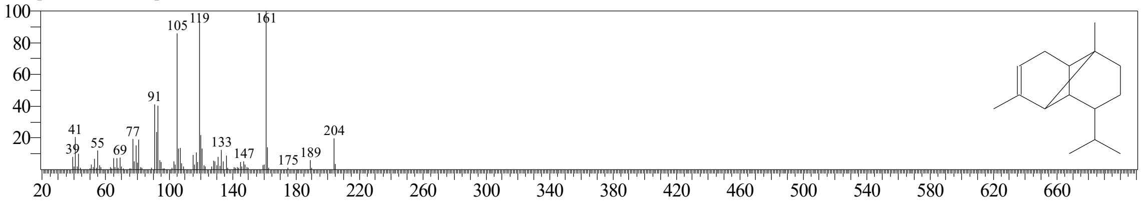

Hit#:2 Entry:46736 Library:NIST11.lib
SI:89 Formula:C15H24 CAS:3856-25-5 MolWeight:204 RetIndex:1221
CompName:Copaene $$ Tricyclo[4.4.0.02,7]dec-3-ene, 1,3-dimethyl-8-(1-methylethyl)-, stereoisomer $$ Tricyclo[4.4.0.02,7]dec-3-ene, 8-isopropyl-1,3-dimethy

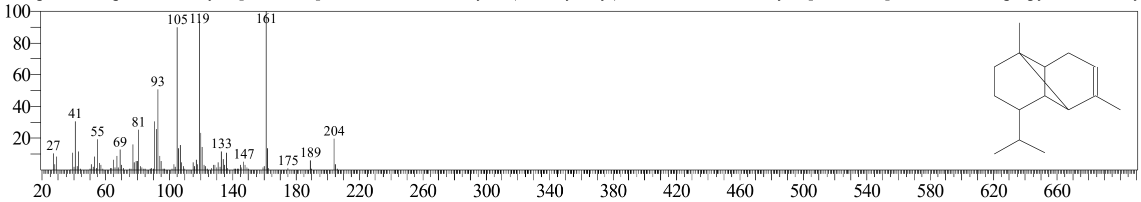

Hit#:3 Entry:46654 Library:NIST11.lib
SI:89 Formula:C15H24 CAS:14912-44-8 MolWeight:204 RetIndex:1221
CompName:Ylangene $$ Tricyclo[4.4.0.0(2,7)]dec-3-ene, 8-isopropyl-1,3-dimethyl-, (1S,2R,6R,7R,8S)-(+)- $$ Tricyclo[4.4.0.0(2,7)]dec-3-ene, 1,3-dimethyl-8-

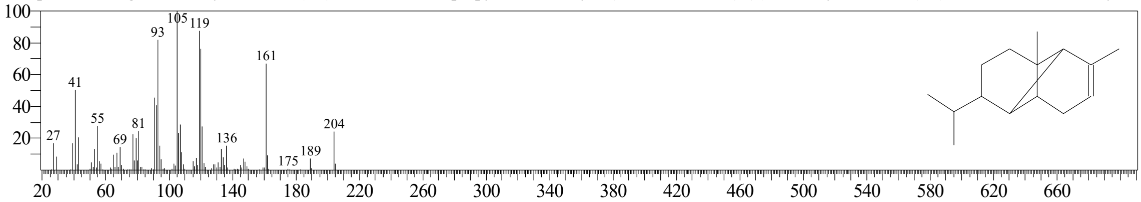

Hit#:4 Entry:46653 Library:NIST11.lib
SI:88 Formula:C15H24 CAS:0-00-0 MolWeight:204 RetIndex:1221
CompName:.alpha.-ylangene

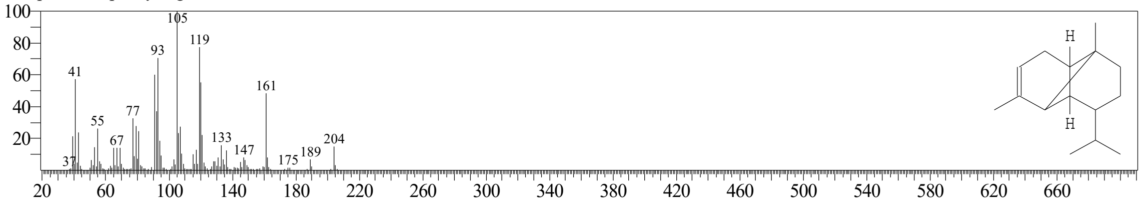

Hit#:5 Entry:46726 Library:NIST11.lib
SI:86 Formula:C15H24 CAS:17699-14-8 MolWeight:204 RetIndex:1344
CompName:.alpha.-Cubebene $$ 1H-Cyclopenta[1,3]cyclopropa[1,2]benzene, 3a,3b,4,5,6,7-hexahydro-3,7-dimethyl-4-(1-methylethyl)-, [3aS-(3a.alpha.,3b.beta

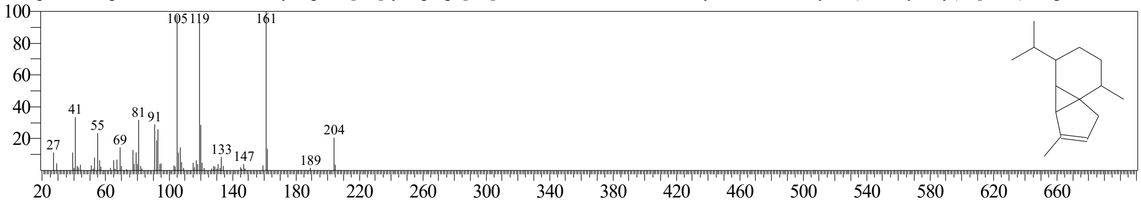

<< Target >>
Line#:71 R.Time:9.630(Scan#:1127) MassPeaks:361
RawMode:Averaged 9.625-9.635(1126-1128) BasePeak:41.05(117532)
BG Mode:Calc. from Peak Group 1 - Event 1 Scan

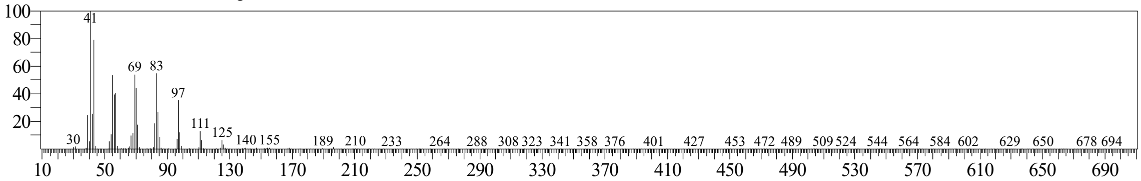

Hit#:1 Entry:24832 Library:NIST11.lib
SI:94 Formula:C12H24 CAS:112-41-4 MolWeight:168 RetIndex:1204
CompName:1-Dodecene $$ .alpha.-Dodecene $$ n-Dodec-1-ene $$ Adacene 12 $$ Dodec-1-ene $$ .alpha.-Dodecylene $$ Dodecylene .alpha.- $$ Dodecene-1 $

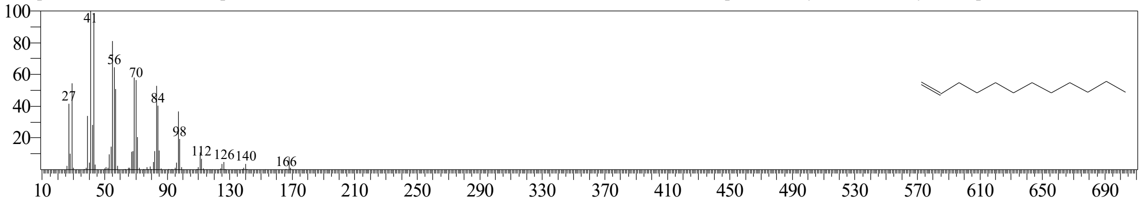

Hit#:2 Entry:41639 Library:NIST11.lib
SI:93 Formula:C14H28 CAS:41446-68-8 MolWeight:196 RetIndex:1421
CompName:3-Tetradecene, (E)- $$ (3E)-3-Tetradecene $$ (E)-3-Tetradecene $$ trans-3-Tetradecene $$

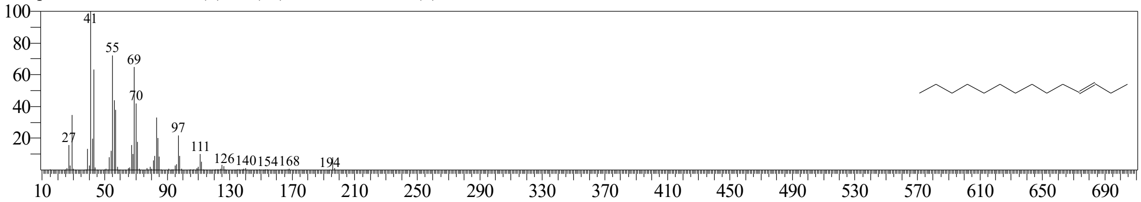

Hit#:3 Entry:51049 Library:NIST11.lib
SI:92 Formula:C15H30 CAS:13360-61-7 MolWeight:210 RetIndex:1502
CompName:1-Pentadecene $$ Pentadecene,1- $$ Pentadec-1-ene $$

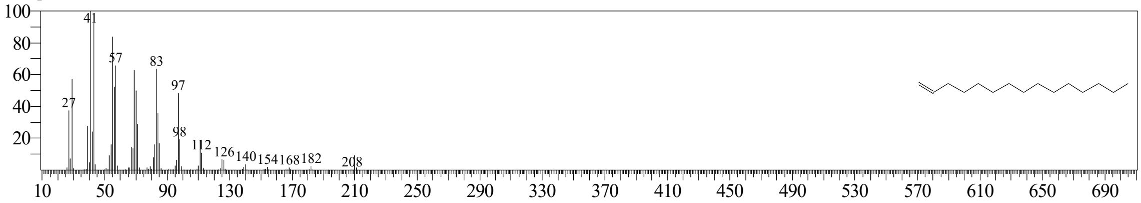

Hit#:4 Entry:41638 Library:NIST11.lib
SI:92 Formula:C14H28 CAS:41446-67-7 MolWeight:196 RetIndex:1421
CompName:3-Tetradecene, (Z)- $$ (3Z)-3-Tetradecene $$ cis-3-Tetradecene $$ (Z)-3-Tetradecene $$

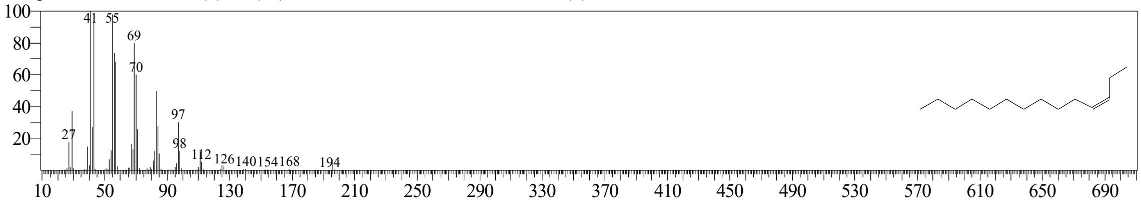

Hit#:5 Entry:41642 Library:NIST11.lib
SI:92 Formula:C14H28 CAS:35953-54-9 MolWeight:196 RetIndex:1421
CompName:2-Tetradecene, (E)- $$ (2E)-2-Tetradecene $$ trans-2-Tetradecene $$ (E)-2-Tetradecene $$ 2-Tetradecene, (2E)- $$ 2-Tetradecene, trans- $$

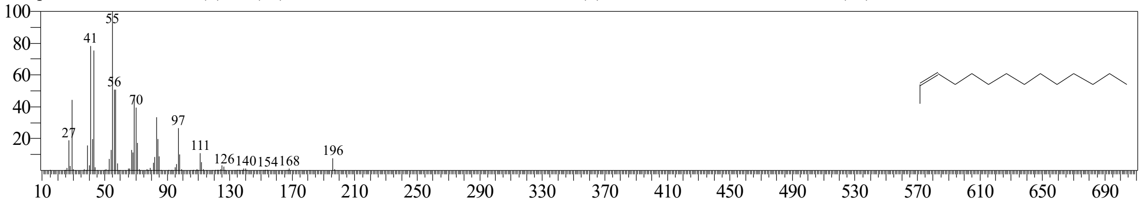

<< Target >>
Line#:72 R.Time:9.690(Scan#:1139) MassPeaks:414
RawMode:Averaged 9.685-9.695(1138-1140) BasePeak:43.05(248285)
BG Mode:Calc. from Peak Group 1 - Event 1 Scan

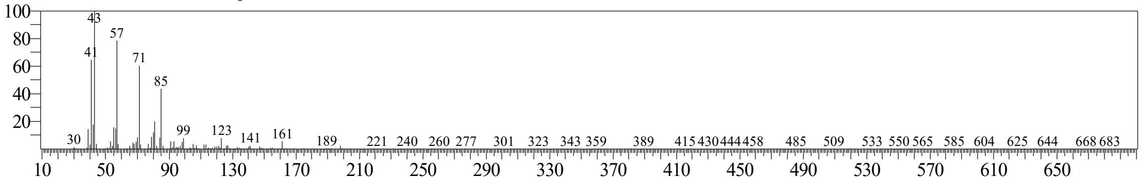

Hit#:1 Entry:18707 Library:NIST11.lib
SI:87 Formula:C11H24 CAS:62016-30-2 MolWeight:156 RetIndex:966
CompName:Octane, 2,3,3-trimethyl- $$ 2,3,3-Trimethyloctane # $$

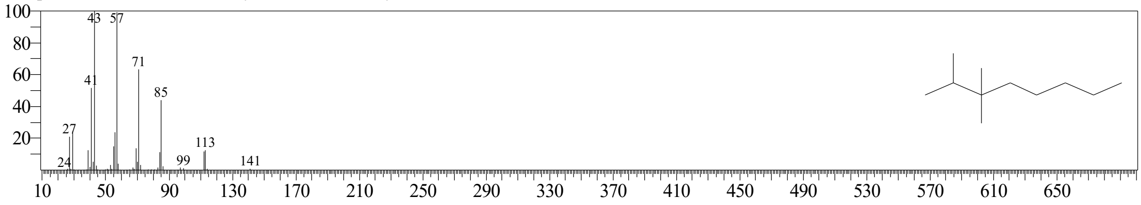

Hit#:2 Entry:92779 Library:NIST11.lib
SI:86 Formula:C10H21I CAS:0-00-0 MolWeight:268 RetIndex:1365
CompName:1-Iodo-2-methylnonane

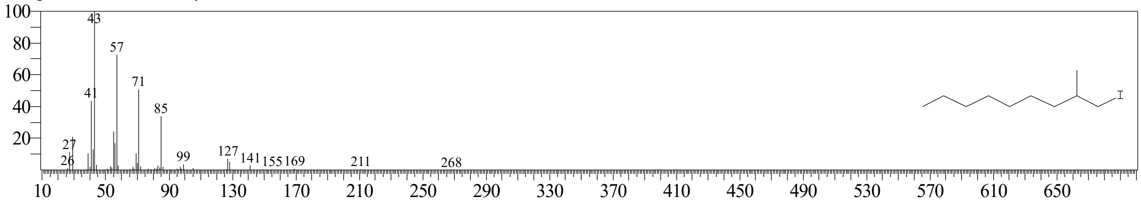

Hit#:3 Entry:18717 Library:NIST11.lib
SI:86 Formula:C11H24 CAS:6975-98-0 MolWeight:156 RetIndex:1051
CompName:Decane, 2-methyl- $$ 2-Methyldecane $$ n-C8H17CH(CH3)2 $$

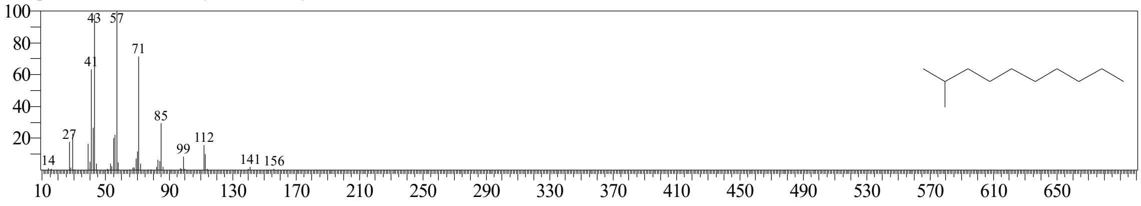

Hit#:4 Entry:24840 Library:NIST11.lib
SI:86 Formula:C12H24 CAS:74630-39-0 MolWeight:168 RetIndex:1140
CompName:1-Undecene, 4-methyl- $$ 4-Methyl-1-undecene # $$

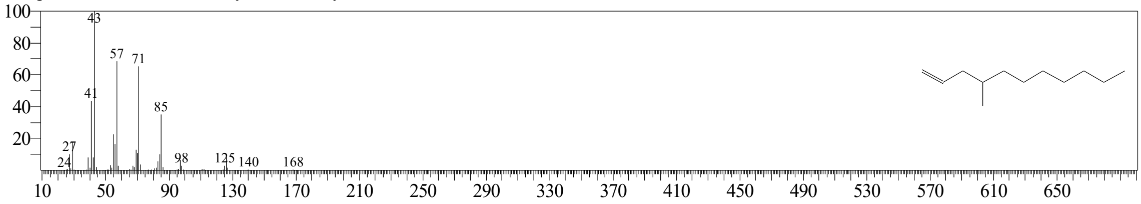

Hit#:5 Entry:26089 Library:NIST11.lib
SI:86 Formula:C12H26 CAS:17312-54-8 MolWeight:170 RetIndex:1086
CompName:Decane, 3,7-dimethyl- $$ 3,7-Dimethyldecane # $$

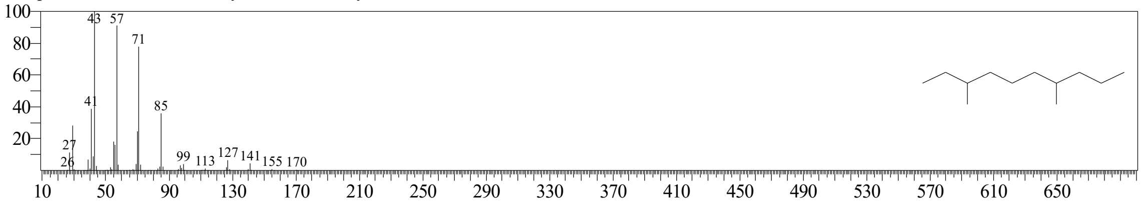

<< Target >>
Line#:73 R.Time:10.015(Scan#:1204) MassPeaks:388
RawMode:Averaged 10.010-10.020(1203-1205) BasePeak:41.10(1392041)
BG Mode:Calc. from Peak Group 1 - Event 1 Scan

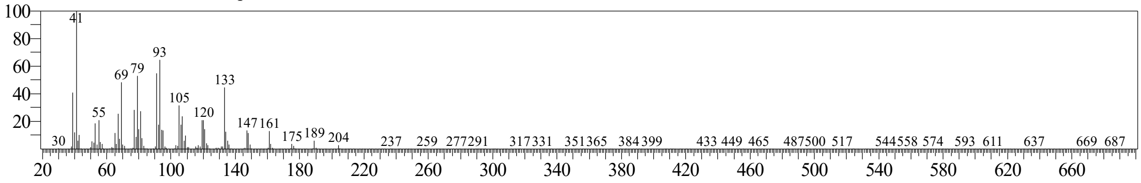

Hit#:1 Entry:46586 Library:NIST11.lib
SI:94 Formula:C15H24 CAS:118-65-0 MolWeight:204 RetIndex:1494
CompName:Bicyclo[7.2.0]undec-4-ene, 4,11,11-trimethyl-8-methylene-,[1R-(1R*,4Z,9S*)]- $$ Isocaryophyllene $$ 4,11,11-Trimethyl-8-methylenebicyclo[7.2.(

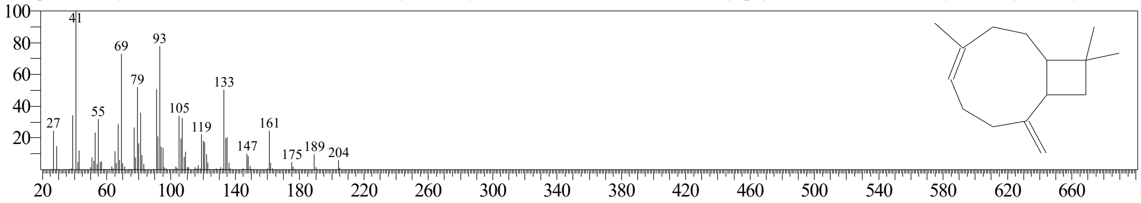

Hit#:2 Entry:46619 Library:NIST11.lib
SI:88 Formula:C15H24 CAS:29837-07-8 MolWeight:204 RetIndex:1518
CompName:cis-.alpha.-Bisabolene $$ 4-[(1Z)-1,5-Dimethyl-1,4-hexadienyl]-1-methyl-1-cyclohexene $$ .alpha.-Bisabolene (Z) $$ (Z)-.alpha.-Bisabolene $$ Cy

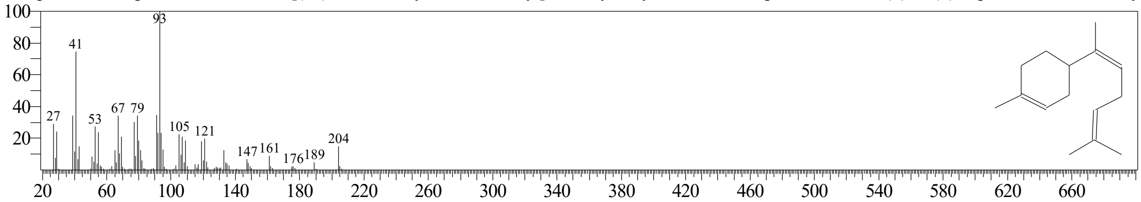

Hit#:3 Entry:46618 Library:NIST11.lib
SI:88 Formula:C15H24 CAS:13877-93-5 MolWeight:204 RetIndex:1494
CompName:Bicyclo[7.2.0]undec-4-ene, 4,11,11-trimethyl-8-methylene- $$ Bicyclo[7.2.0]undec-4-ene, 4,11,11-trimethyl-8-methylene-, (Z)- $$ cis-4,11,11-Trim

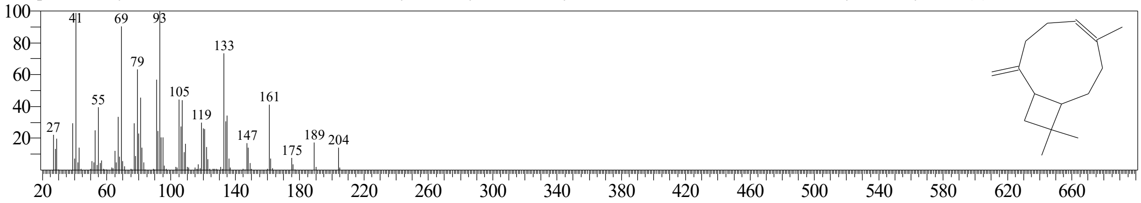

Hit#:4 Entry:46612 Library:NIST11.lib
SI:88 Formula:C15H24 CAS:0-00-0 MolWeight:204 RetIndex:1407
CompName:Bicyclo[5.2.0]nonane, 4-methylene-2,8,8-trimethyl-2-vinyl- $$ 2,8,8-Trimethyl-4-methylene-2-vinylbicyclo[5.2.0]nonane # $$

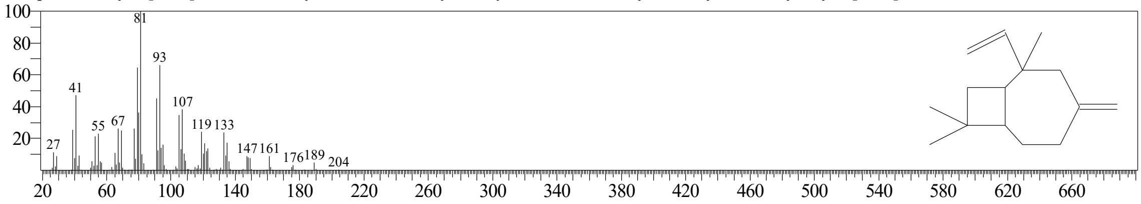

Hit#:5 Entry:46518 Library:NIST11.lib
SI:87 Formula:C13H20N2 CAS:0-00-0 MolWeight:204 RetIndex:0
CompName:1,4-Methanocycloocta[d]pyridazine, 1,4,4a,5,6,9,10,10a-octahydro-11,11-dimethyl-, (1.alpha.,4.alpha.,4a.alpha.,10a.alpha.)-

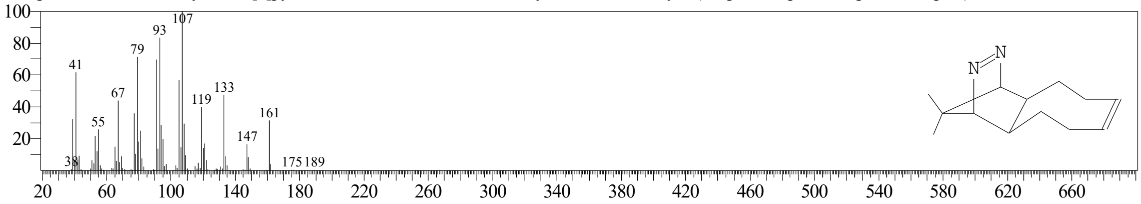

<< Target >>
Line#:74 R.Time:10.105(Scan#:1222) MassPeaks:358
RawMode:Averaged 10.100-10.110(1221-1223) BasePeak:57.15(7596)
BG Mode:Calc. from Peak Group 1 - Event 1 Scan

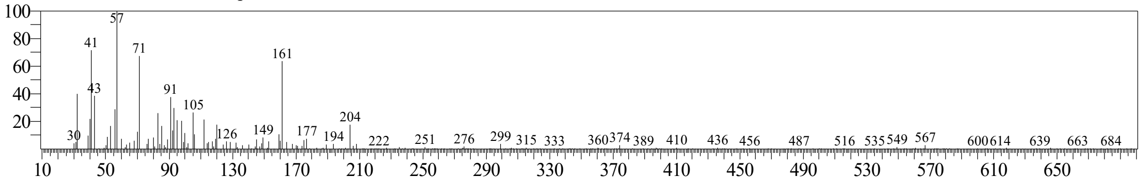

Hit#:1 Entry:46596 Library:NIST11.lib
SI:63 Formula:C15H24 CAS:49826-80-4 MolWeight:204 RetIndex:1525
CompName:Benzene, (2-methyloctyl)- $$ (2-Methyloctyl)benzene # $$

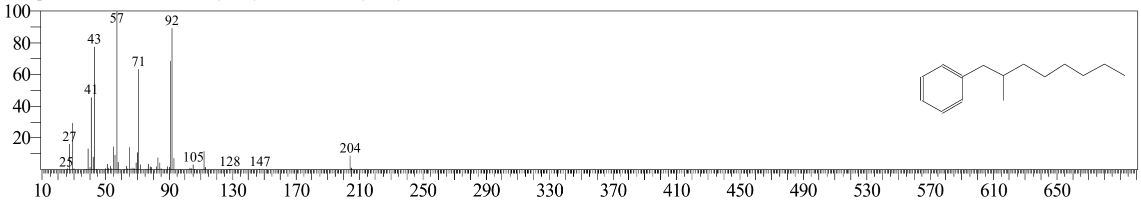

Hit#:2 Entry:66519 Library:NIST11.lib
SI:63 Formula:C14H29Cl CAS:2425-54-9 MolWeight:232 RetIndex:1638
CompName:Tetradecane, 1-chloro- $$ Myristyl chloride $$ Tetradecyl chloride $$ 1-Chlorotetradecane $$ n-Tetradecyl chloride $$

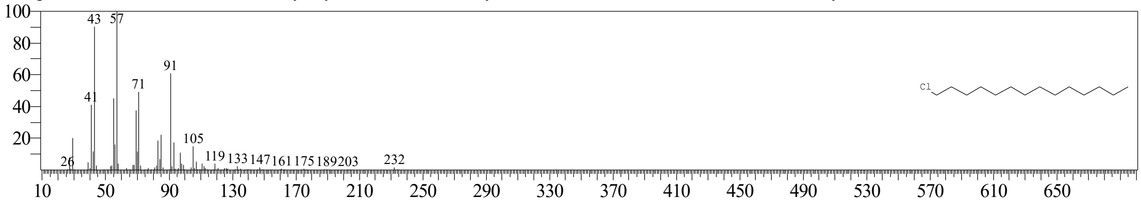

Hit#:3 Entry:87431 Library:NIST11.lib
SI:62 Formula:C16H33Cl CAS:4860-03-1 MolWeight:260 RetIndex:1837
CompName:Hexadecane, 1-chloro- $$ Cetyl chloride $$ Hexadecyl chloride $$ 1-Chlorohexadecane $$ n-Hexadecyl chloride $$ Palmityl chloride $$

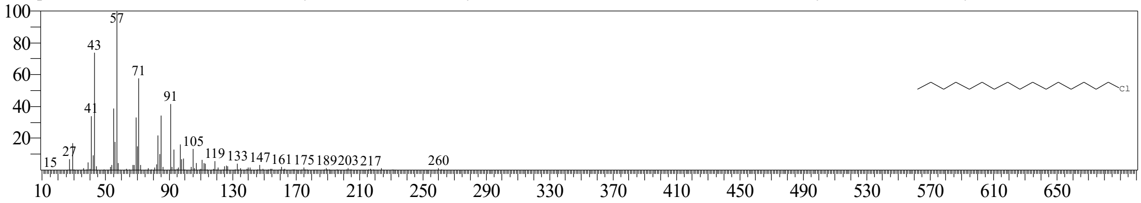

Hit#:4 Entry:76879 Library:NIST11.lib
SI:62 Formula:C18H30 CAS:55134-08-2 MolWeight:246 RetIndex:1759
CompName:Benzene, (2,3-dimethyldecyl)- $$ (2,3-Dimethyldecyl)benzene # $$

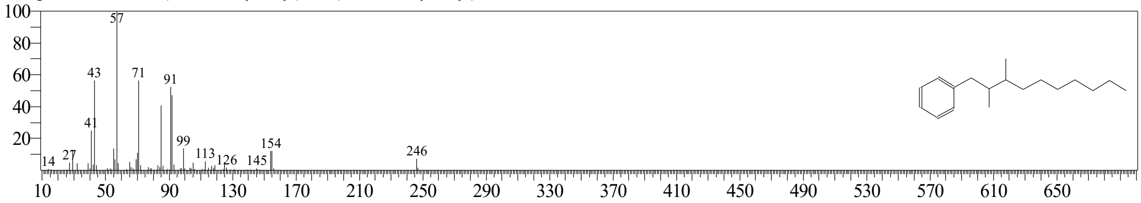

Hit#:5 Entry:156678 Library:NIST11.lib
SI:61 Formula:C18H37ClO2S CAS:0-00-0 MolWeight:352 RetIndex:2493
CompName:1-Octadecanesulphonyl chloride

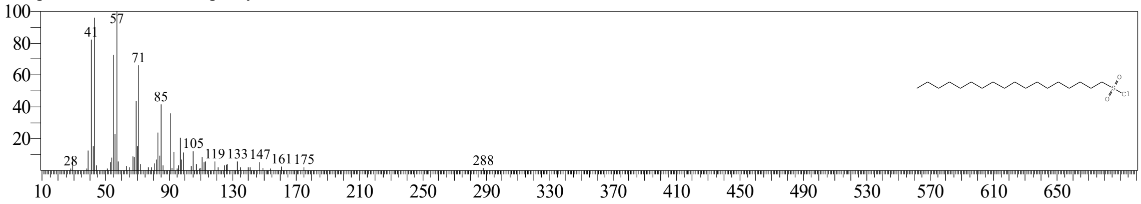

<< Target >>
Line#:75 R.Time:10.200(Scan#:1241) MassPeaks:386
RawMode:Averaged 10.195-10.205(1240-1242) BasePeak:43.05(43642)
BG Mode:Calc. from Peak Group 1 - Event 1 Scan

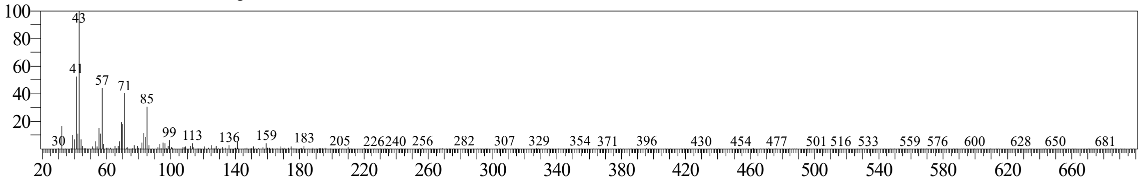

Hit#:1 Entry:34145 Library:NIST11.lib
SI:85 Formula:C13H28 CAS:17301-33-6 MolWeight:184 RetIndex:1185
CompName:Undecane, 4,8-dimethyl- $$ 4,8-Dimethylundecane # $$

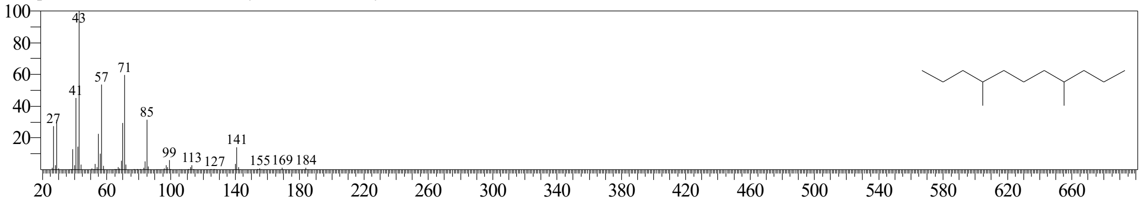

Hit#:2 Entry:27506 Library:NIST11.lib
SI:84 Formula:C10H23NO CAS:29812-79-1 MolWeight:173 RetIndex:1334
CompName:Hydroxylamine, O-decyl- $$ O-Decylhydroxylamine $$

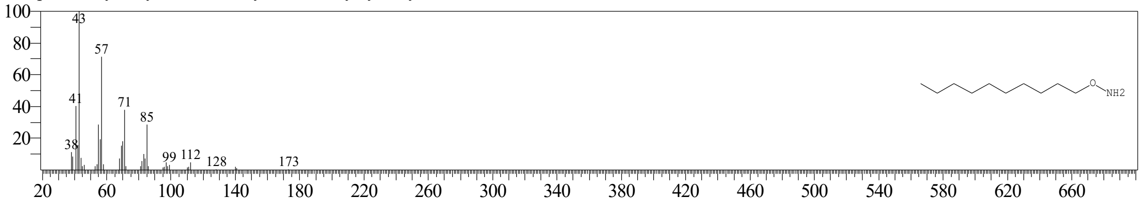

Hit#:3 Entry:92779 Library:NIST11.lib
SI:84 Formula:C10H21I CAS:0-00-0 MolWeight:268 RetIndex:1365
CompName:1-Iodo-2-methylnonane

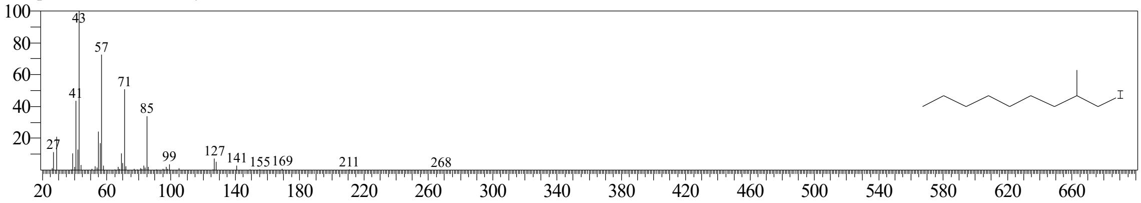

Hit#:4 Entry:62478 Library:NIST11.lib
SI:84 Formula:C16H34 CAS:1560-93-6 MolWeight:226 RetIndex:1548
CompName:Pentadecane, 2-methyl- $$ 2-Methylpentadecane $$

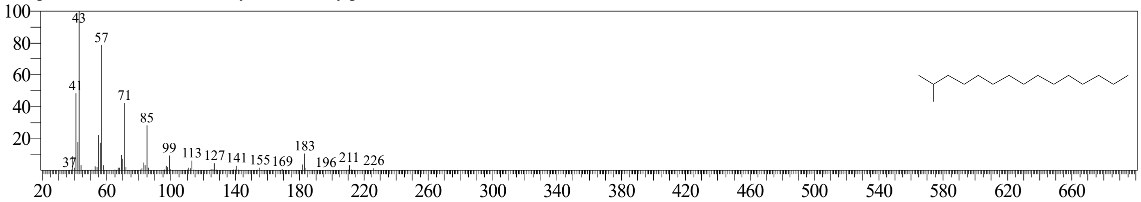

Hit#:5 Entry:52478 Library:NIST11.lib
SI:84 Formula:C15H32 CAS:13286-73-2 MolWeight:212 RetIndex:1448
CompName:Tridecane, 3-ethyl- $$ 3-Ethyltridecane # $$

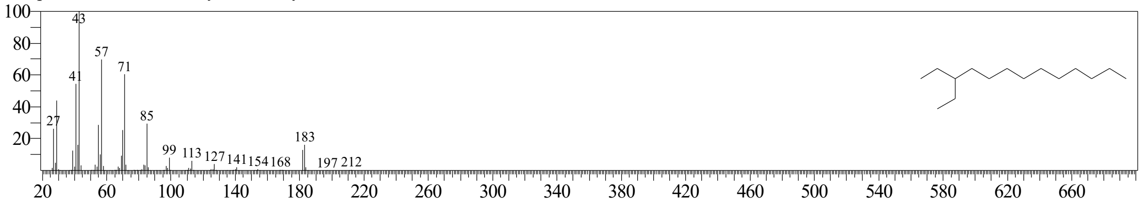

<< Target >>
Line#:76 R.Time:10.335(Scan#:1268) MassPeaks:419
RawMode:Averaged 10.330-10.340(1267-1269) BasePeak:93.05(1789164)
BG Mode:Calc. from Peak Group 1 - Event 1 Scan

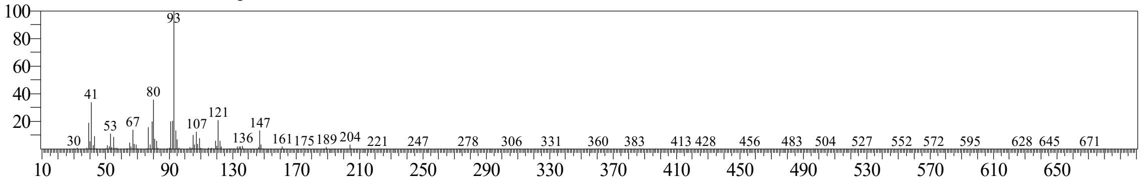

Hit#:1 Entry:46627 Library:NIST11.lib
SI:94 Formula:C15H24 CAS:6753-98-6 MolWeight:204 RetIndex:1579
CompName:Humulene $$ .alpha.-Caryophyllene $$ 1,4,8-Cycloundecatriene, 2,6,6,9-tetramethyl-, (E,E,E)- $$ .alpha.-Humulene $$ Cycloundeca-1,4,8-triene,2,

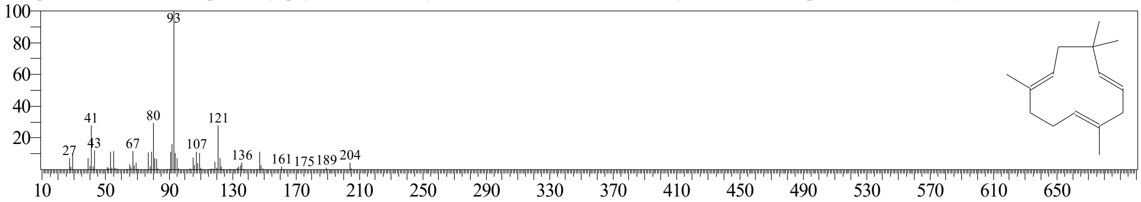

Hit#:2 Entry:46629 Library:NIST11.lib
SI:91 Formula:C15H24 CAS:0-00-0 MolWeight:204 RetIndex:1579
CompName:1,4,7,-Cycloundecatriene, 1,5,9,9-tetramethyl-, Z,Z,Z- $$ 1,5,9,9-Tetramethyl-1,4,7-cycloundecatriene # $$

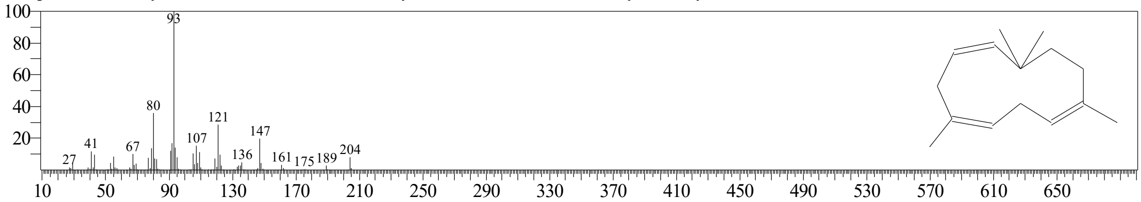

Hit#:3 Entry:9810 Library:NIST11.lib
SI:88 Formula:C10H16 CAS:498-15-7 MolWeight:136 RetIndex:948
CompName:(+)-3-Carene $$ Bicyclo[4.1.0]hept-3-ene, 3,7,7-trimethyl-, (1S)- $$ 3,7,7-Trimethylbicyclo[4.1.0]hept-3-ene-, (1S,6R)- $$ Isodiprene $$ (+)-.delta.

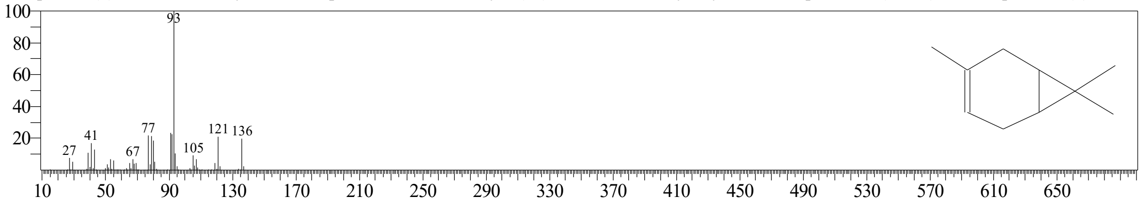

Hit#:4 Entry:9782 Library:NIST11.lib
SI:88 Formula:C10H16 CAS:488-97-1 MolWeight:136 RetIndex:729
CompName:Tricyclo[2.2.1.0(2,6)]heptane, 1,3,3-trimethyl- $$ Cyclofenchene $$ Tricyclo[2.2.1.02,6]heptane, 1,3,3-trimethyl- $$

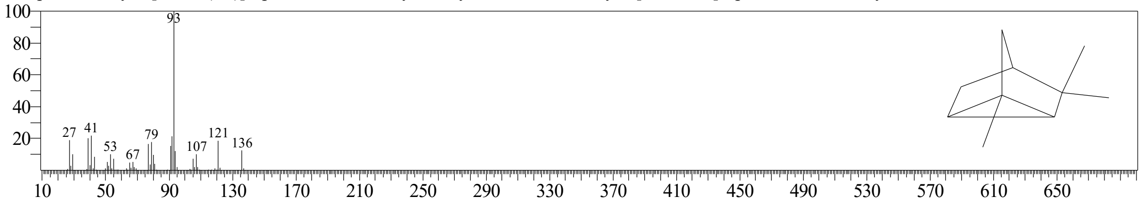

Hit#:5 Entry:9779 Library:NIST11.lib
SI:87 Formula:C10H16 CAS:3338-55-4 MolWeight:136 RetIndex:976
CompName:1,3,6-Octatriene, 3,7-dimethyl-, (Z)- $$ .beta.-cis-Ocimene $$ cis-.beta.-Ocimene $$ cis-3,7-Dimethyl-1,3,6-octatriene $$ Ocimene, cis-.beta.- $$ (Z

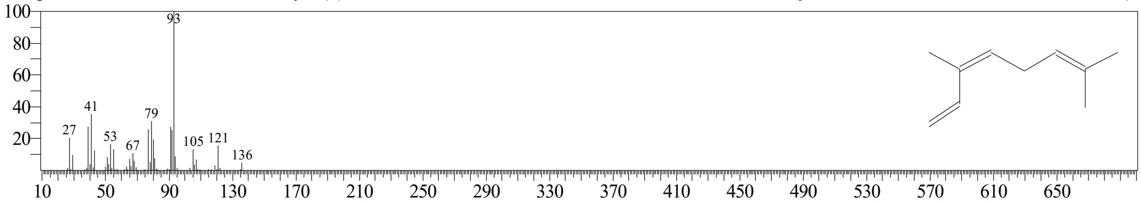

<< Target >>
Line#:77 R.Time:10.460(Scan#:1293) MassPeaks:384
RawMode:Averaged 10.455-10.465(1292-1294) BasePeak:161.10(9471)
BG Mode:Calc. from Peak Group 1 - Event 1 Scan

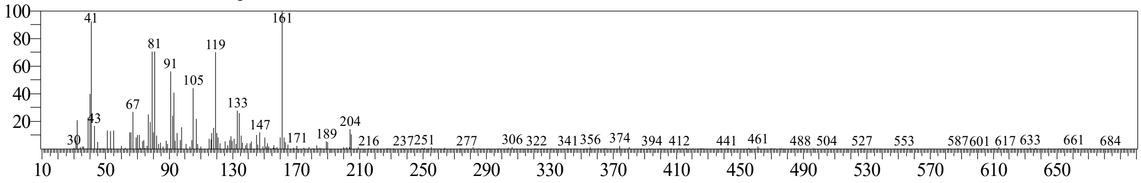

Hit#:1 Entry:46722 Library:NIST11.lib
SI:83 Formula:C15H24 CAS:23986-74-5 MolWeight:204 RetIndex:1515
CompName:1,6-Cyclodecadiene, 1-methyl-5-methylene-8-(1-methylethyl)-, [S-(E,E)]- $$ Germacrene D $$ 8-Isopropyl-1-methyl-5-methylene-1,6-cyclodecadie

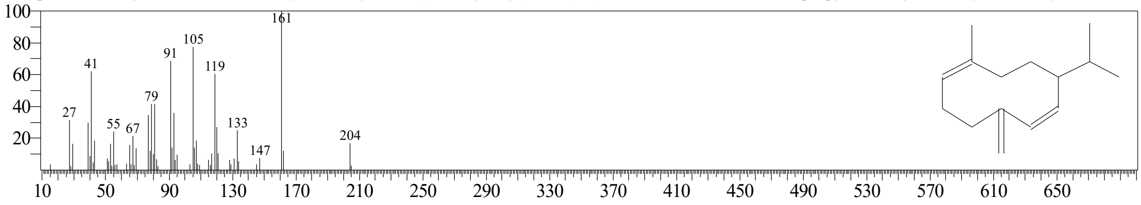

Hit#:2 Entry:46720 Library:NIST11.lib
SI:81 Formula:C15H24 CAS:0-00-0 MolWeight:204 RetIndex:1216
CompName:.beta.-copaene

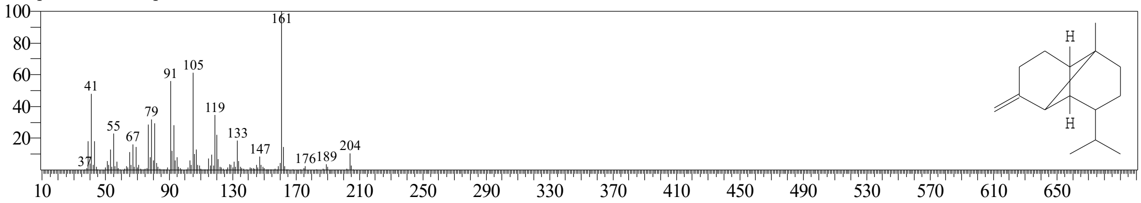

Hit#:3 Entry:46691 Library:NIST11.lib
SI:80 Formula:C15H24 CAS:0-00-0 MolWeight:204 RetIndex:1216
CompName:.beta.-ylangene

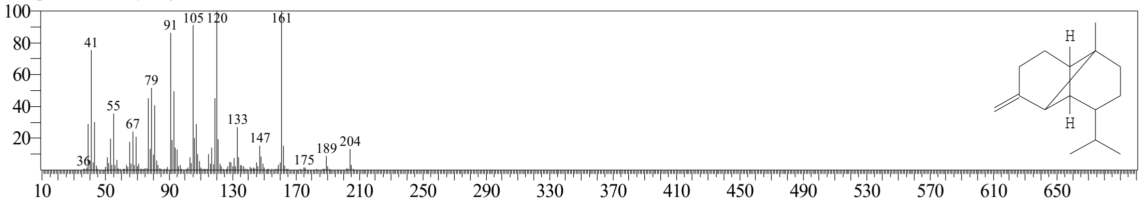

Hit#:4 Entry:46595 Library:NIST11.lib
SI:78 Formula:C15H24 CAS:0-00-0 MolWeight:204 RetIndex:1281
CompName:Bicyclo[4.1.0]-3-heptene, 2-isopropenyl-5-isopropyl-7,7-dimethyl- $$ 2-Isopropenyl-5-isopropyl-7,7-dimethylbicyclo[4.1.0]hept-3-ene # $$

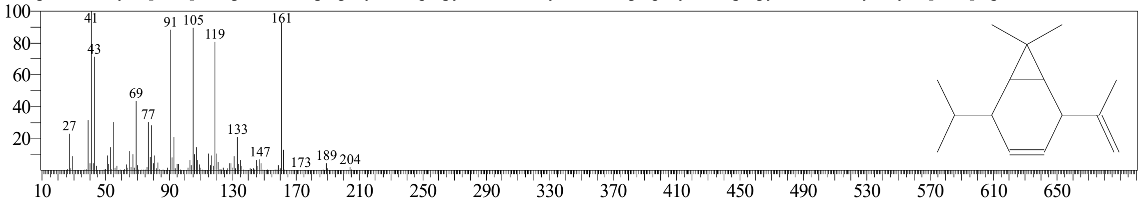

Hit#:5 Entry:46715 Library:NIST11.lib
SI:78 Formula:C15H24 CAS:56348-21-1 MolWeight:204 RetIndex:1257
CompName:Tricyclo[4.1.0.0(2,4)]heptane, 3,3,7,7-tetramethyl-5-(2-methyl-1-propenyl)-

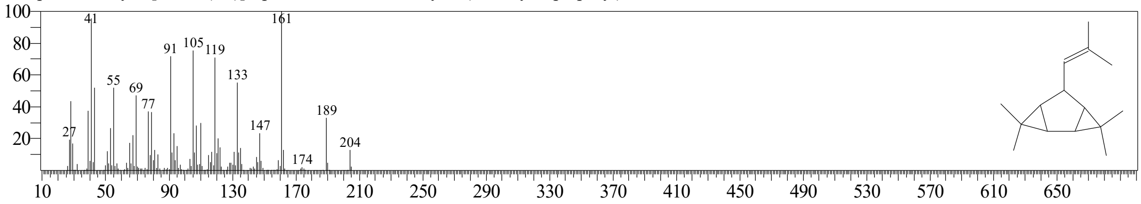

<< Target >>
Line#:78 R.Time:10.550(Scan#:1311) MassPeaks:416
RawMode:Averaged 10.545-10.555(1310-1312) BasePeak:161.10(1298939)
BG Mode:Calc. from Peak Group 1 - Event 1 Scan

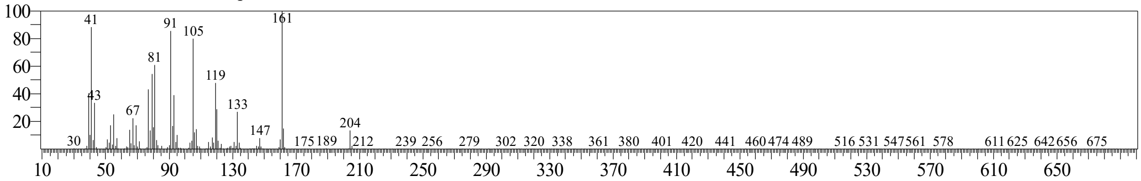

Hit#:1 Entry:46722 Library:NIST11.lib
SI:93 Formula:C15H24 CAS:23986-74-5 MolWeight:204 RetIndex:1515
CompName:1,6-Cyclodecadiene, 1-methyl-5-methylene-8-(1-methylethyl)-, [S-(E,E)]- $$ Germacrene D $$ 8-Isopropyl-1-methyl-5-methylene-1,6-cyclodecadie

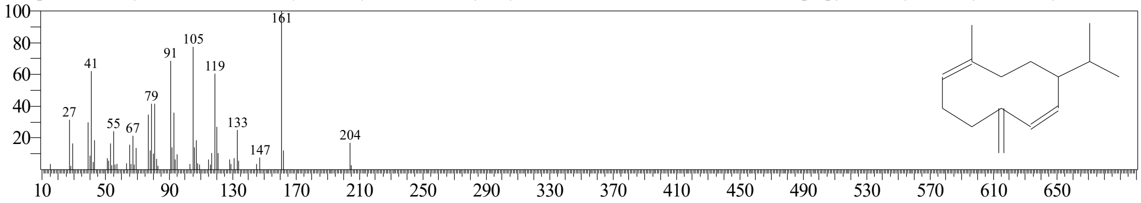

Hit#:2 Entry:46691 Library:NIST11.lib
SI:91 Formula:C15H24 CAS:0-00-0 MolWeight:204 RetIndex:1216
CompName:.beta.-ylangene

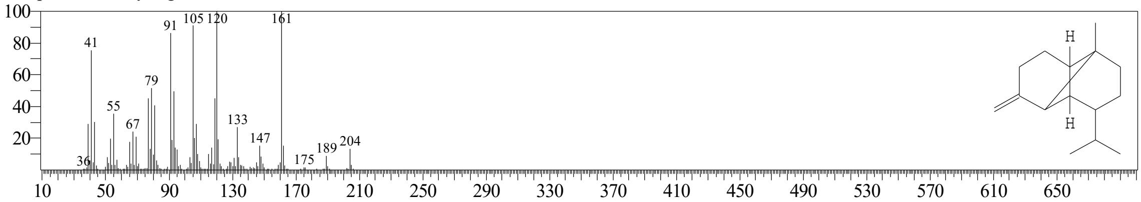

Hit#:3 Entry:46720 Library:NIST11.lib
SI:89 Formula:C15H24 CAS:0-00-0 MolWeight:204 RetIndex:1216
CompName:.beta.-copaene

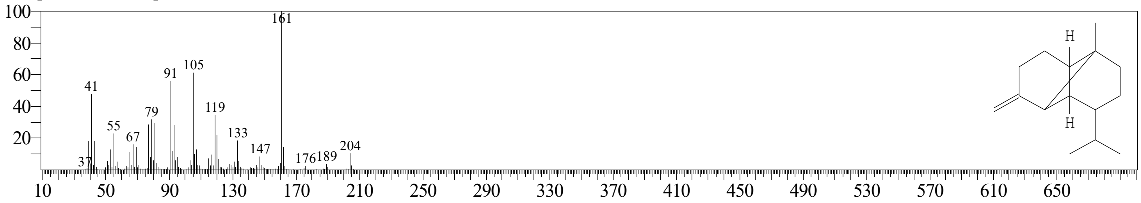

Hit#:4 Entry:29328 Library:NIST11.lib
SI:86 Formula:C13H20 CAS:0-00-0 MolWeight:176 RetIndex:1251
CompName:Bicyclo[2.2.1]heptane, 2-cyclopropylidene-1,7,7-trimethyl- $$ 2-Cyclopropylidene-1,7,7-trimethylbicyclo[2.2.1]heptane # $$

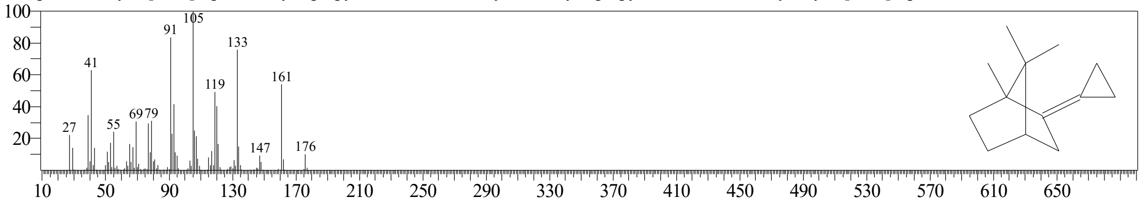

Hit#:5 Entry:46595 Library:NIST11.lib
SI:85 Formula:C15H24 CAS:0-00-0 MolWeight:204 RetIndex:1281
CompName:Bicyclo[4.1.0]-3-heptene, 2-isopropenyl-5-isopropyl-7,7-dimethyl- $$ 2-Isopropenyl-5-isopropyl-7,7-dimethylbicyclo[4.1.0]hept-3-ene # $$

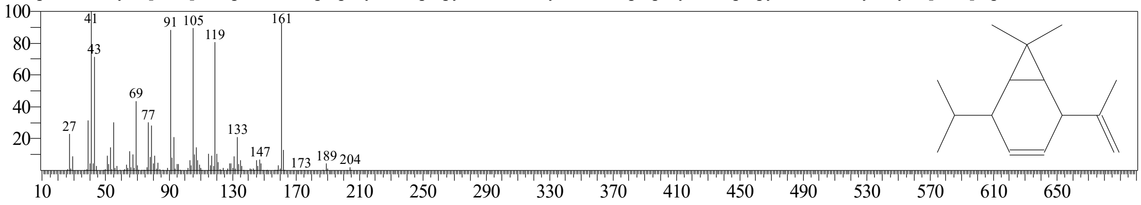

Qualitative Analysis Report

<< Target >>
Line#:79 R.Time:10.665(Scan#:1334) MassPeaks:373
RawMode:Averaged 10.660-10.670(1333-1335) BasePeak:93.10(33340)
BG Mode:Calc. from Peak Group 1 - Event 1 Scan

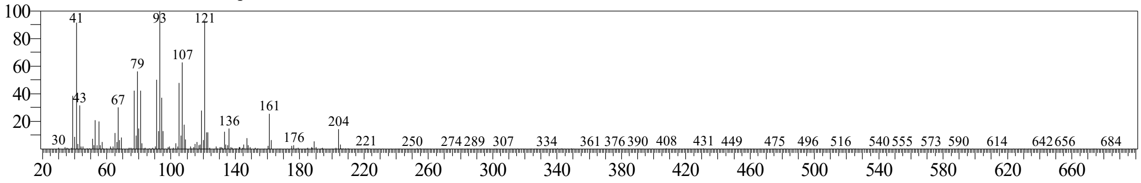

Hit#:1 Entry:46634 Library:NIST11.lib
SI:89 Formula:C15H24 CAS:67650-90-2 MolWeight:204 RetIndex:1499
CompName:Bicyclogermacrene $$ 3,7,11,11-Tetramethylbicyclo[8.1.0]undeca-2,6-diene # $$

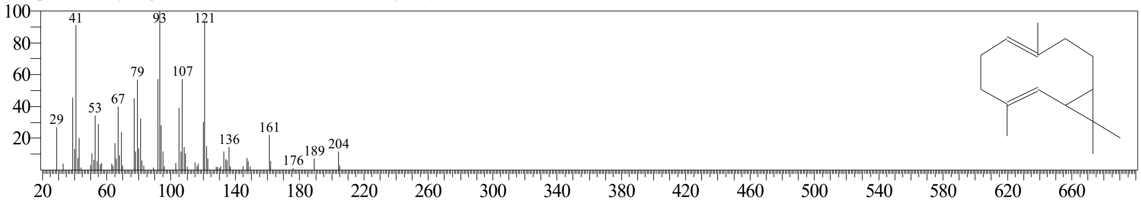

Hit#:2 Entry:46693 Library:NIST11.lib
SI:89 Formula:C15H24 CAS:15423-57-1 MolWeight:204 RetIndex:1603
CompName:1,5-Cyclodecadiene, 1,5-dimethyl-8-(1-methylethylidene)-, (E,E)- $$ Germacrene B $$ Germacra-1(10),4,7(11)-triene $$ Germacra-1(10),4,7(11)-tri

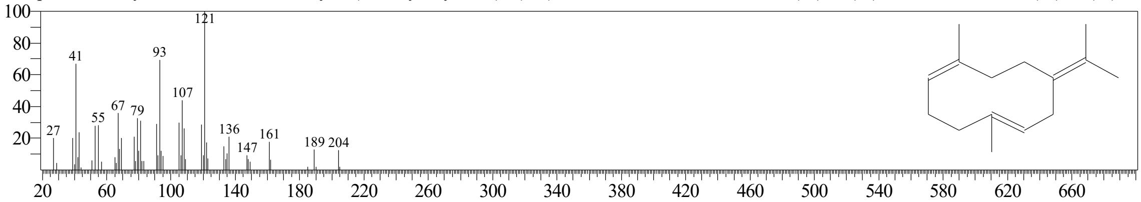

Hit#:3 Entry:46692 Library:NIST11.lib
SI:88 Formula:C15H24 CAS:29873-99-2 MolWeight:204 RetIndex:1431
CompName:.gamma.-Elemene $$ 1-Methyl-2-(1-methylethenyl)-4-(1-methylethylidene)-1-vinylcyclohexane), (1R-trans)- $$ (-)-.gamma.-Elemene $$ o-Menth-8-

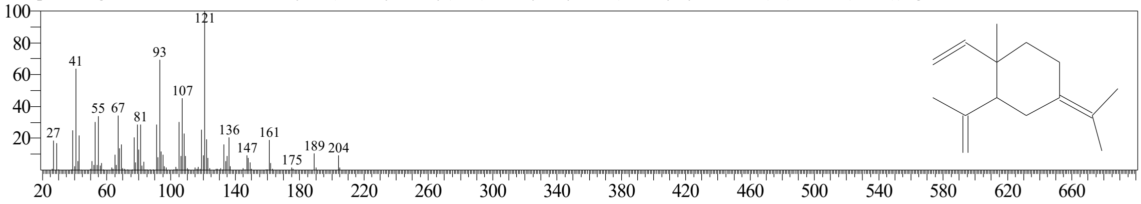

Hit#:4 Entry:9819 Library:NIST11.lib
SI:88 Formula:C10H16 CAS:42123-66-0 MolWeight:136 RetIndex:894
CompName:1,3,6-Heptatriene, 2,5,6-trimethyl- $$ 2,5,6-Trimethyl-1,3,6-heptatriene $$ 2,5,6-Trimethyl-hepta-1,3,6-triene $$ Hepta-1,3,6-triene, 2,5,6-trimethyl

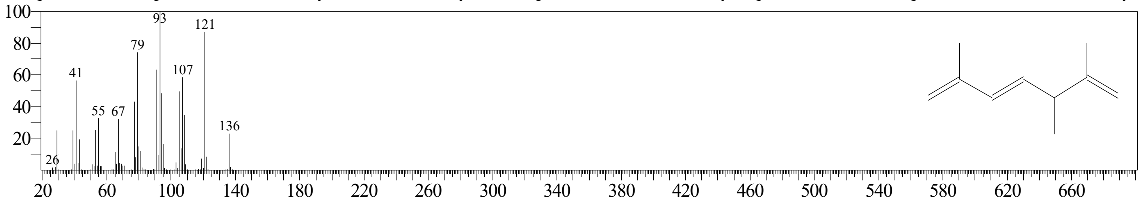

Hit#:5 Entry:61948 Library:NIST11.lib
SI:87 Formula:C12H18O2S CAS:0-00-0 MolWeight:226 RetIndex:0
CompName:3,4-Dimethyl-2,5-diprop-2-enyl-2,5-dihydrothiophene 1,1-dioxide

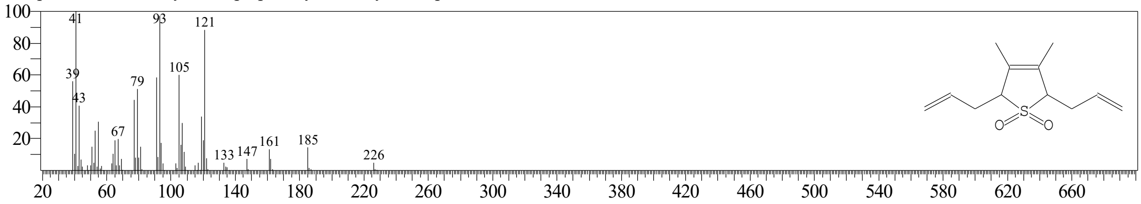

<< Target >>
Line#:80 R.Time:10.815(Scan#:1364) MassPeaks:370
RawMode:Averaged 10.810-10.820(1363-1365) BasePeak:161.20(20287)
BG Mode:Calc. from Peak Group 1 - Event 1 Scan

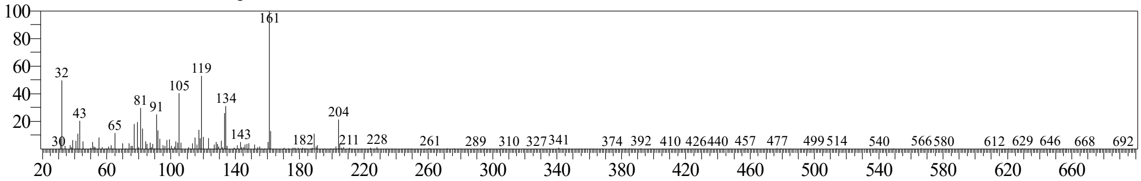

Hit#:1 Entry:46724 Library:NIST11.lib
SI:76 Formula:C15H24 CAS:0-00-0 MolWeight:204 RetIndex:1440
CompName:cis-muurola-3,5-diene

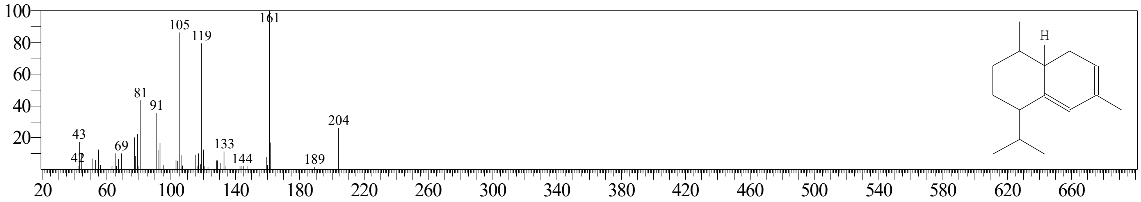

Hit#:2 Entry:46739 Library:NIST11.lib
SI:76 Formula:C15H24 CAS:483-76-1 MolWeight:204 RetIndex:1469
CompName:Naphthalene, 1,2,3,5,6,8a-hexahydro-4,7-dimethyl-1-(1-methylethyl)-, (1S-cis)- $$ Cadina-1(10),4-diene $$ .delta.-Cadinene, (+)- $$ (+)-.delta.-Ca

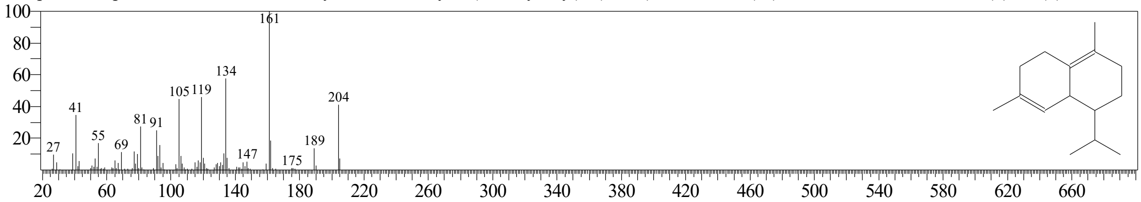

Hit#:3 Entry:46735 Library:NIST11.lib
SI:74 Formula:C15H24 CAS:0-00-0 MolWeight:204 RetIndex:1221
CompName:.alfa.-Copaene

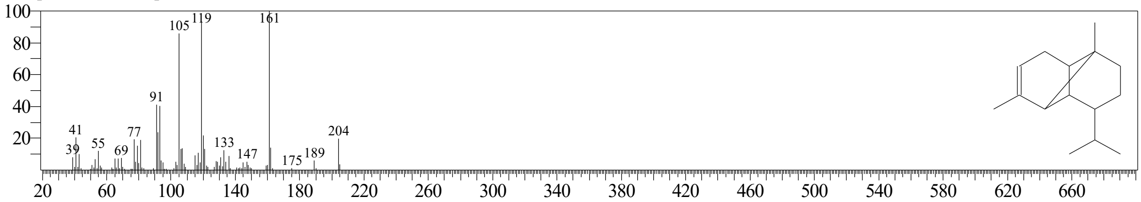

Hit#:4 Entry:46720 Library:NIST11.lib
SI:73 Formula:C15H24 CAS:0-00-0 MolWeight:204 RetIndex:1216
CompName:.beta.-copaene

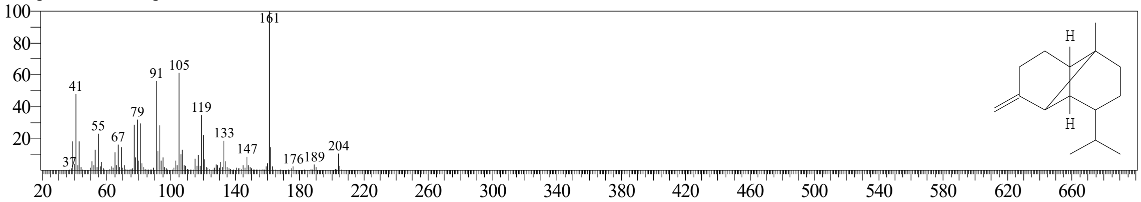

Hit#:5 Entry:46727 Library:NIST11.lib
SI:72 Formula:C15H24 CAS:30021-74-0 MolWeight:204 RetIndex:1435
CompName:.gamma.-Muurolene $$ Naphthalene, 1,2,3,4,4a,5,6,8a-octahydro-7-methyl-4-methylene-1-(1-methylethyl)-, (1.alpha.,4a.alpha.,8a.alpha.)- $$ 1-Isop

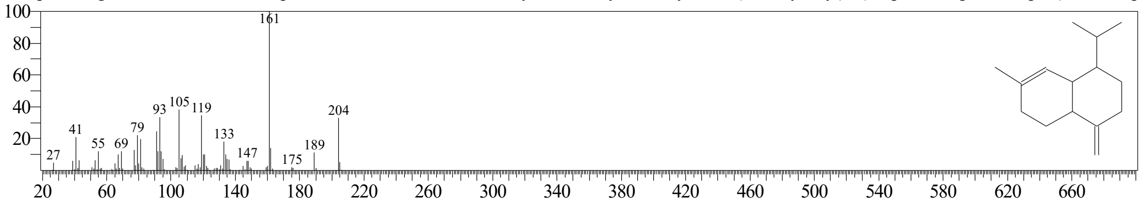

<< Target >>
Line#:81 R.Time:10.855(Scan#:1372) MassPeaks:396
RawMode:Averaged 10.850-10.860(1371-1373) BasePeak:69.10(34377)
BG Mode:Calc. from Peak Group 1 - Event 1 Scan

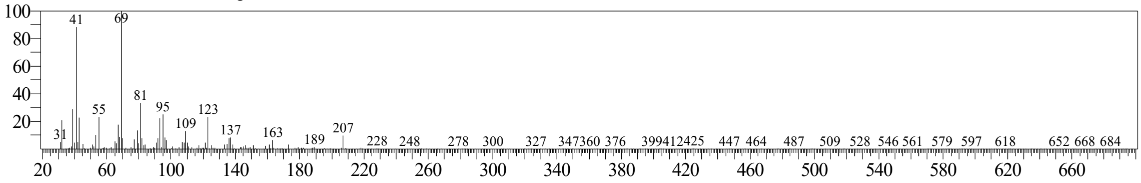

Hit#:1 Entry:79842 Library:NIST11.lib
SI:82 Formula:C17H30O CAS:0-00-0 MolWeight:250 RetIndex:1909
CompName:4,8,12-Tetradecatrien-1-ol, 5,9,13-trimethyl- $$ (4E,8E)-5,9,13-Trimethyl-4,8,12-tetradecatrien-1-ol # $$

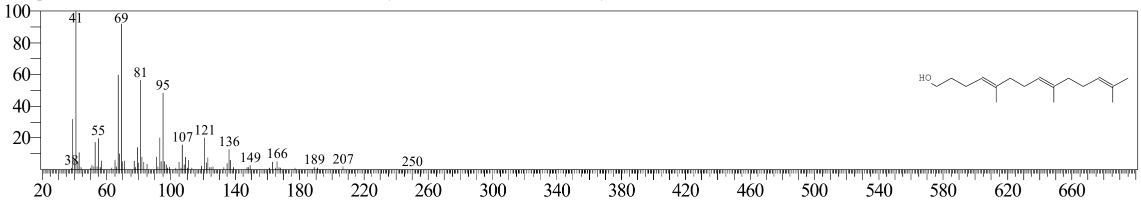

Hit#:2 Entry:49572 Library:NIST11.lib
SI:82 Formula:C14H24O CAS:0-00-0 MolWeight:208 RetIndex:1634
CompName:6,11-Dimethyl-2,6,10-dodecatrien-1-ol $$ (2E,6E)-6,11-Dimethyl-2,6,10-dodecatrien-1-ol # $$

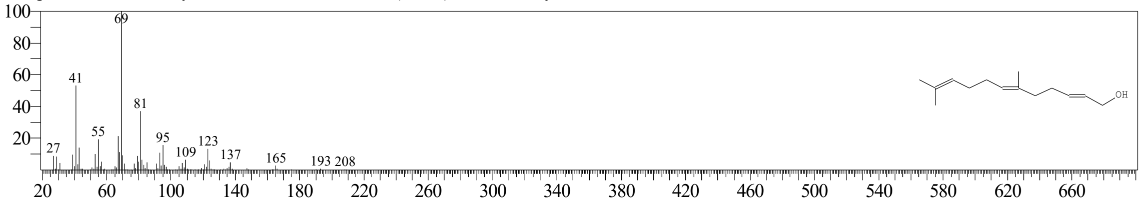

Hit#:3 Entry:38803 Library:NIST11.lib
SI:82 Formula:C14H24 CAS:230646-72-7 MolWeight:192 RetIndex:1227
CompName:1,5,9-Decatriene, 2,3,5,8-tetramethyl- $$ (5E)-2,3,5,8-Tetramethyl-1,5,9-decatriene # $$

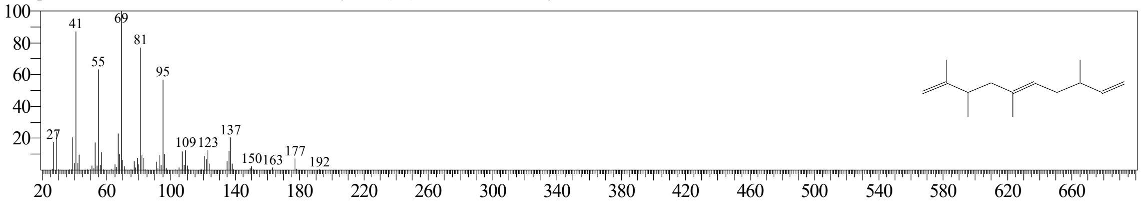

Hit#:4 Entry:24774 Library:NIST11.lib
SI:81 Formula:C11H20O CAS:98678-70-7 MolWeight:168 RetIndex:1280
CompName:Cyclopropanemethanol, 2-methyl-2-(4-methyl-3-pentenyl)- $$ [2-Methyl-2-(4-methyl-3-pentenyl)cyclopropyl]methanol # $$

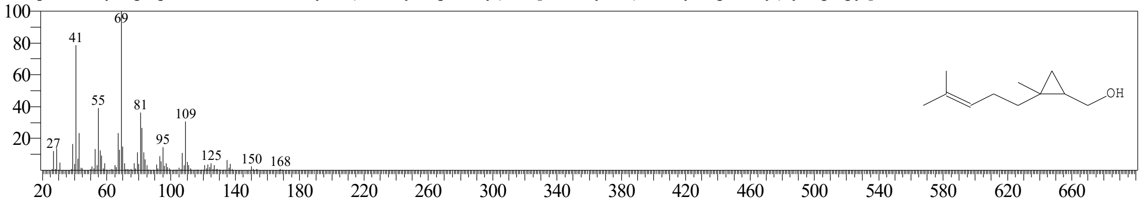

Hit#:5 Entry:59349 Library:NIST11.lib
SI:81 Formula:C15H26O CAS:3790-71-4 MolWeight:222 RetIndex:1710
CompName:2,6,10-Dodecatrien-1-ol, 3,7,11-trimethyl-, (Z,E)- $$ (Z,E)-Farnesol $$ (2Z,6E)-Farnesol $$ cis,trans-Farnesol $$ 2-cis,6-trans-Farnesol $$ (2Z,6E)

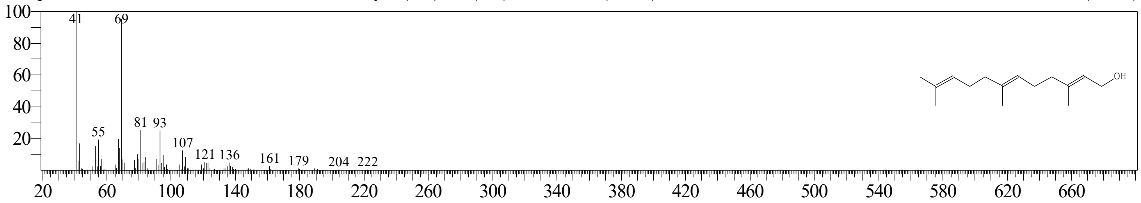

<< Target >>
Line#:82 R.Time:10.965(Scan#:1394) MassPeaks:345
RawMode:Averaged 10.960-10.970(1393-1395) BasePeak:32.10(9876)
BG Mode:Calc. from Peak Group 1 - Event 1 Scan

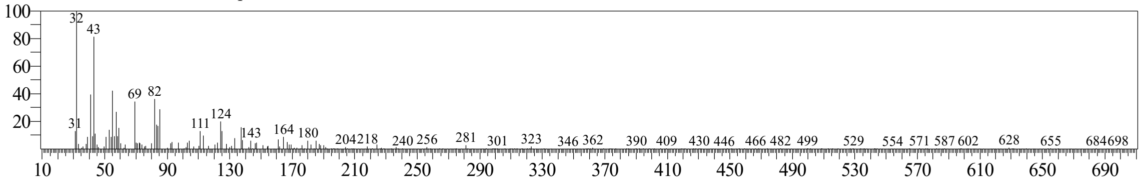

Hit#:1 Entry:13115 Library:NIST11.lib
SI:68 Formula:C9H20O CAS:18450-74-3 MolWeight:144 RetIndex:1030
CompName:1-Heptanol, 2,4-dimethyl-, (2S,4R)-(-)- $$ 2,4-Dimethyl-1-heptanol # $$

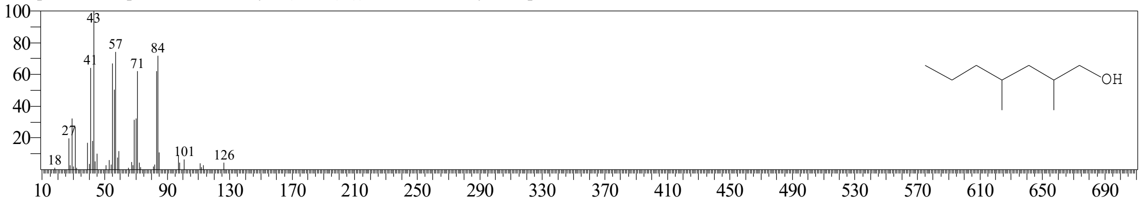

Hit#:2 Entry:4655 Library:NIST11.lib
SI:68 Formula:C7H16O CAS:27522-11-8 MolWeight:116 RetIndex:896
CompName:1-Pentanol, 2-ethyl- $$ 2-Ethyl-1-pentanol $$

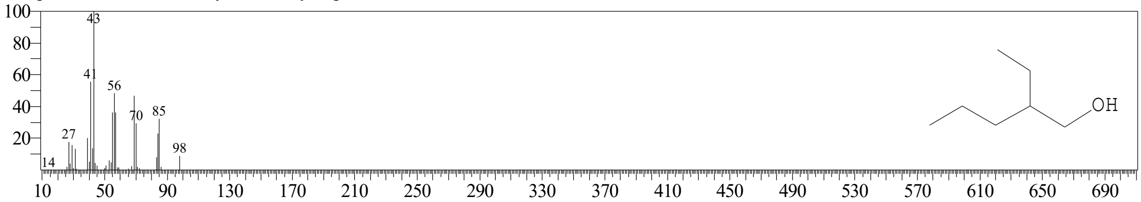

Hit#:3 Entry:24880 Library:NIST11.lib
SI:68 Formula:C12H24 CAS:74630-55-0 MolWeight:168 RetIndex:1158
CompName:4-Undecene, 8-methyl-, (Z)- $$ (4Z)-8-Methyl-4-undecene # $$

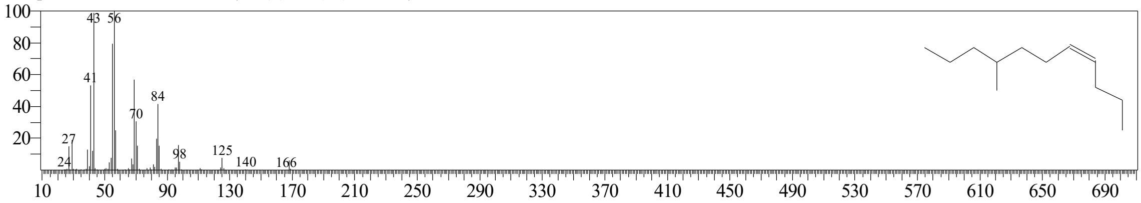

Hit#:4 Entry:24884 Library:NIST11.lib
SI:68 Formula:C12H24 CAS:22370-55-4 MolWeight:168 RetIndex:1140
CompName:1-Undecene, 10-methyl- $$ 10-Methyl-1-undecene # $$

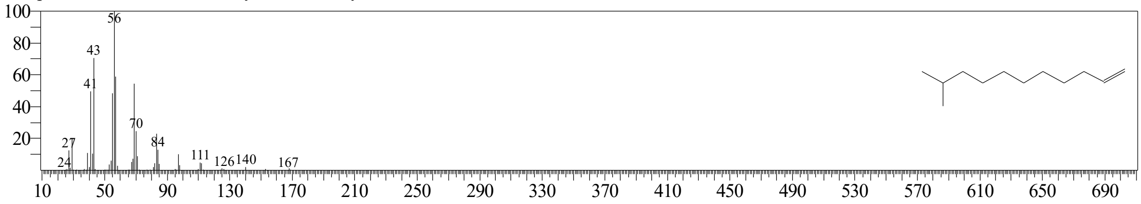

Hit#:5 Entry:107819 Library:NIST11.lib
SI:67 Formula:C18H38O2 CAS:20294-76-2 MolWeight:286 RetIndex:2215
CompName:1,2-Octadecanediol $$ 1,2-Dihydroxyoctadecane $$

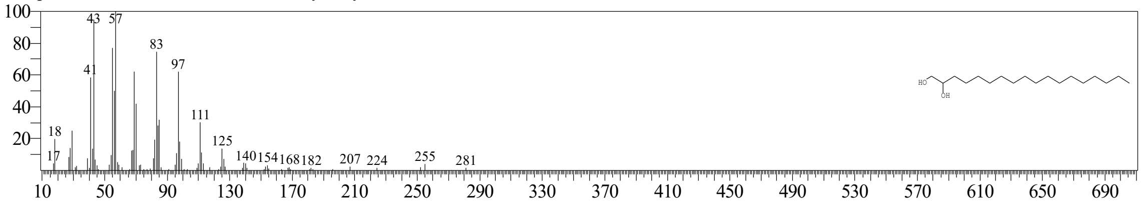

<< Target >>
Line#:83 R.Time:11.105(Scan#:1422) MassPeaks:417
RawMode:Averaged 11.100-11.110(1421-1423) BasePeak:59.05(235518)
BG Mode:Calc. from Peak Group 1 - Event 1 Scan

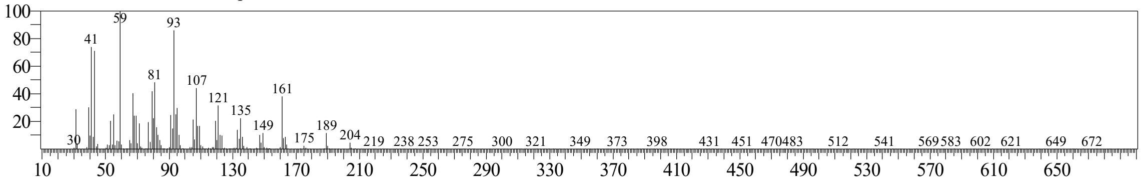

Hit#:1 Entry:59376 Library:NIST11.lib
SI:92 Formula:C15H26O CAS:639-99-6 MolWeight:222 RetIndex:1522
CompName:Cyclohexanemethanol, 4-ethenyl-.alpha.,.alpha.,4-trimethyl-3-(1-methylethenyl)-, [1R-(1.alpha.,3.alpha.,4.beta.)]- $$ o-Menth-8-ene-4-methanol, .a

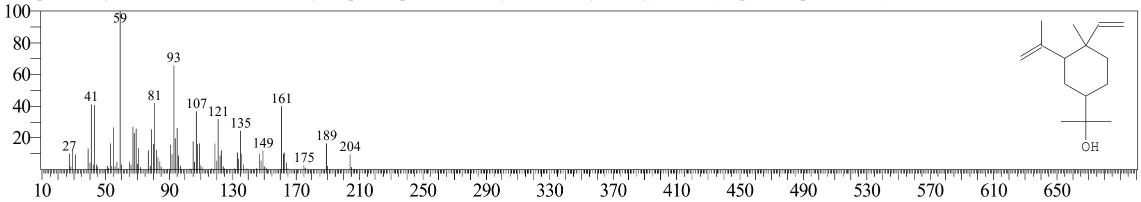

Hit#:2 Entry:59377 Library:NIST11.lib
SI:89 Formula:C15H26O CAS:21657-90-9 MolWeight:222 RetIndex:1694
CompName:3,7-Cyclodecadiene-1-methanol, .alpha.,.alpha.,4,8-tetramethyl-, [s-(Z,Z)] $$ Hedycaryol $$ 2-(4,8-Dimethyl-3,7-cyclodecadien-1-yl)-2-propanol #

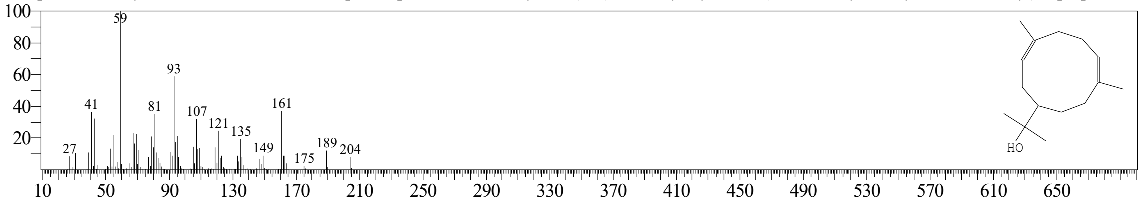

Hit#:3 Entry:59430 Library:NIST11.lib
SI:84 Formula:C15H26O CAS:22451-73-6 MolWeight:222 RetIndex:1614
CompName:5-Azulenemethanol, 1,2,3,3a,4,5,6,7-octahydro-.alpha.,.alpha.,3,8-tetramethyl-, [3S-(3.alpha.,3a.beta.,5.alpha.)]- $$ Guai-1(10)-en-11-ol $$ Bulnes

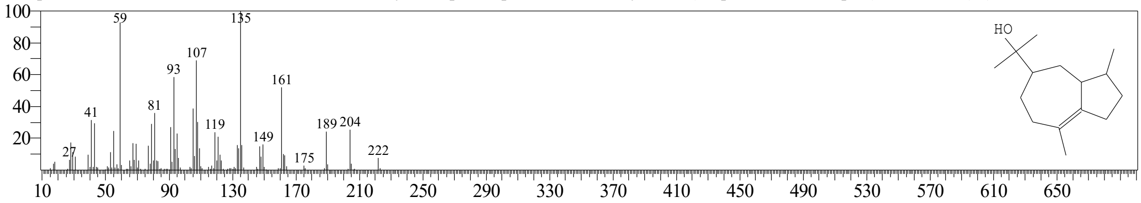

Hit#:4 Entry:59382 Library:NIST11.lib
SI:83 Formula:C15H26O CAS:13822-35-0 MolWeight:222 RetIndex:1614
CompName:5-Azulenemethanol, 1,2,3,4,5,6,7,8-octahydro-.alpha.,.alpha.,3,8-tetramethyl- $$ 2-(3,8-Dimethyl-1,2,3,4,5,6,7,8-octahydro-5-azulenyl)-2-propanol

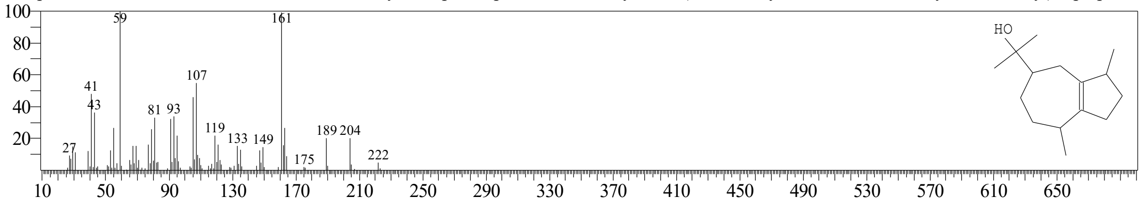

Hit#:5 Entry:59424 Library:NIST11.lib
SI:83 Formula:C15H26O CAS:21284-22-0 MolWeight:222 RetIndex:1580
CompName:Cubenol $$ 1-Isopropyl-4,7-dimethyl-1,3,4,5,6,8a-hexahydro-4a(2H)-naphthalenol-, [1S-(1.alpha.,4.beta.,4a.beta.,8a.alpha.)]- $$ (-)-Cubenol $$ 10

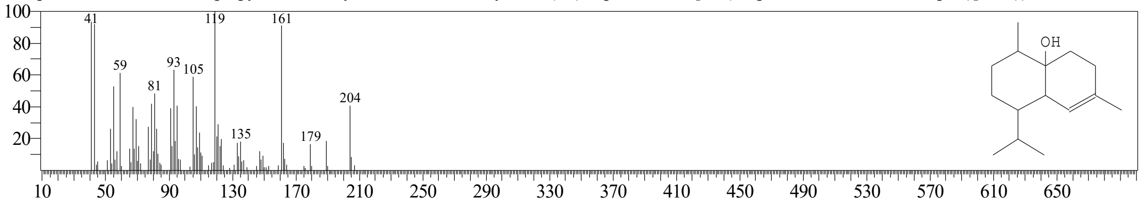

<< Target >>
Line#:84 R.Time:11.225(Scan#:1446) MassPeaks:397
RawMode:Averaged 11.220-11.230(1445-1447) BasePeak:41.10(47353)
BG Mode:Calc. from Peak Group 1 - Event 1 Scan

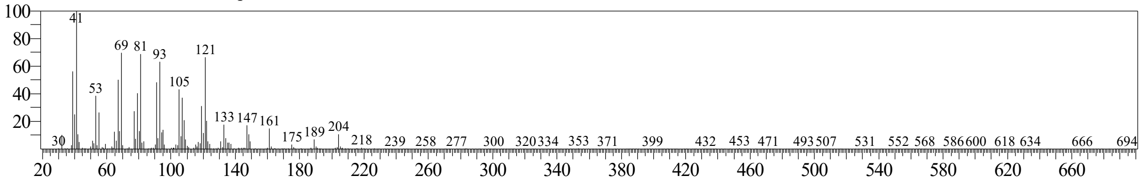

Hit#:1 Entry:46693 Library:NIST11.lib
SI:88 Formula:C15H24 CAS:15423-57-1 MolWeight:204 RetIndex:1603
CompName:1,5-Cyclodecadiene, 1,5-dimethyl-8-(1-methylethylidene)-, (E,E)- $$ Germacrene B $$ Germacra-1(10),4,7(11)-triene $$ Germacra-1(10),4,7(11)-tri

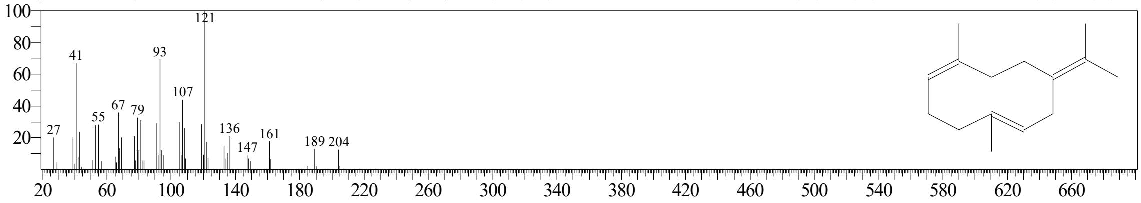

Hit#:2 Entry:46606 Library:NIST11.lib
SI:88 Formula:C15H24 CAS:110823-68-2 MolWeight:204 RetIndex:1398
CompName:Cyclohexane, 1-ethenyl-1-methyl-2,4-bis(1-methylethenyl)- $$ 2,4-Diisopropenyl-1-methyl-1-vinylcyclohexane # $$

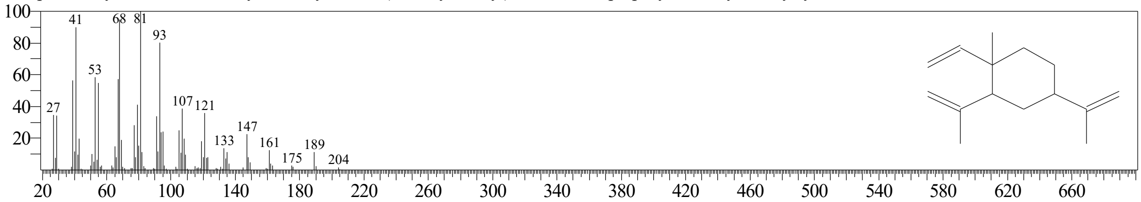

Hit#:3 Entry:46692 Library:NIST11.lib
SI:87 Formula:C15H24 CAS:29873-99-2 MolWeight:204 RetIndex:1431
CompName:.gamma.-Elemene $$ 1-Methyl-2-(1-methylethenyl)-4-(1-methylethylidene)-1-vinylcyclohexane), (1R-trans)- $$ (-)-.gamma.-Elemene $$ o-Menth-8-

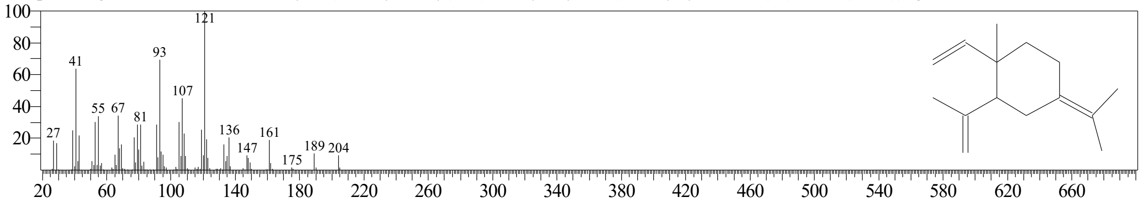

Hit#:4 Entry:46586 Library:NIST11.lib
SI:87 Formula:C15H24 CAS:118-65-0 MolWeight:204 RetIndex:1494
CompName:Bicyclo[7.2.0]undec-4-ene, 4,11,11-trimethyl-8-methylene-,[1R-(1R*,4Z,9S*)]- $$ Isocaryophyllene $$ 4,11,11-Trimethyl-8-methylenebicyclo[7.2.(

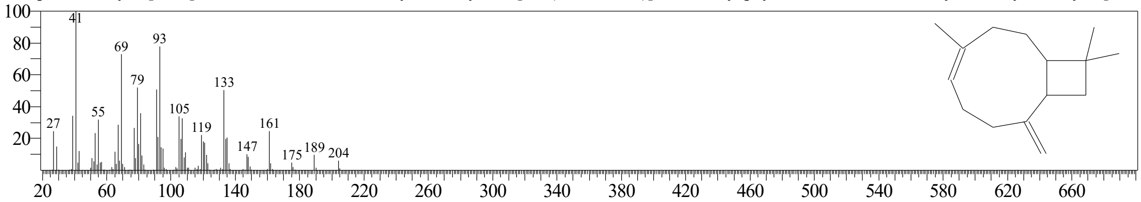

Hit#:5 Entry:46607 Library:NIST11.lib
SI:87 Formula:C15H24 CAS:33880-83-0 MolWeight:204 RetIndex:1398
CompName:Cyclohexane, 1-ethenyl-1-methyl-2,4-bis(1-methylethenyl)-, (1.alpha.,2.beta.,4.beta.)- $$ 2,4-Diisopropenyl-1-methyl-1-vinylcyclohexane # $$ .beta

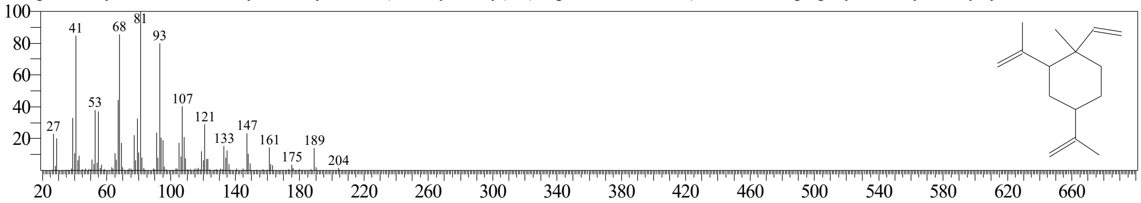

<< Target >>
Line#:85 R.Time:11.320(Scan#:1465) MassPeaks:393
RawMode:Averaged 11.315-11.325(1464-1466) BasePeak:41.05(322032)
BG Mode:Calc. from Peak Group 1 - Event 1 Scan

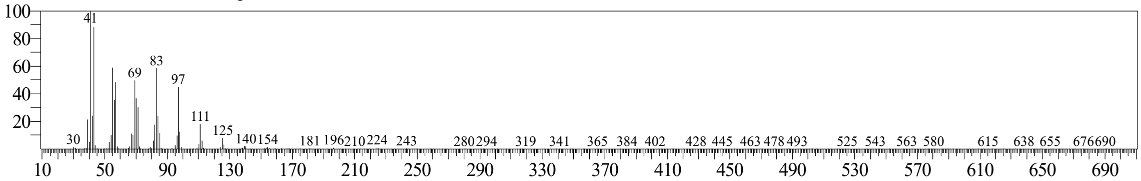

Hit#:1 Entry:51049 Library:NIST11.lib
SI:94 Formula:C15H30 CAS:13360-61-7 MolWeight:210 RetIndex:1502
CompName:1-Pentadecene $$ Pentadecene,1- $$ Pentadec-1-ene $$

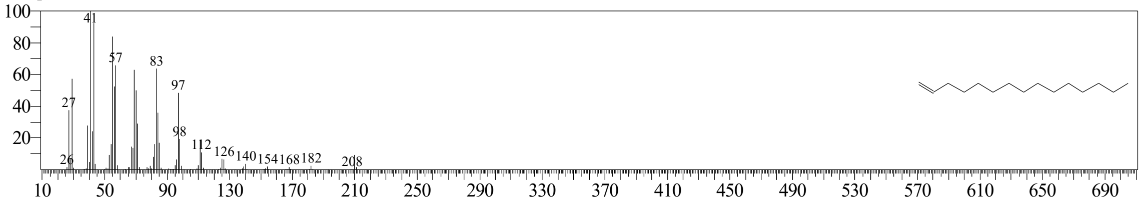

Hit#:2 Entry:60964 Library:NIST11.lib
SI:94 Formula:C16H32 CAS:629-73-2 MolWeight:224 RetIndex:1602
CompName:Cetene $$ 1-Hexadecene $$ .alpha.-Hexadecene $$ n-Hexadec-1-ene $$ 1-Cetene $$ Hexadecylene-1 $$ Hexadec-1-ene $$ Hexadecene-1 $$ Neode

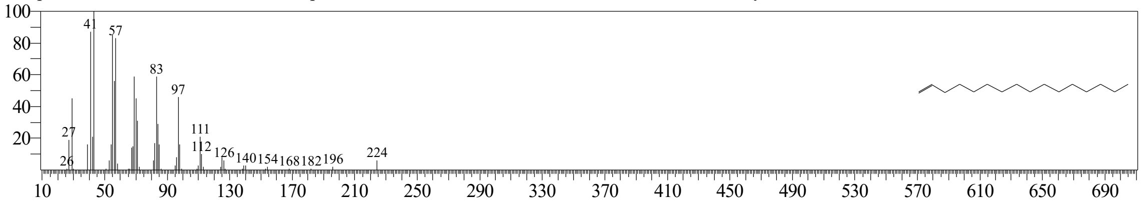

Hit#:3 Entry:70935 Library:NIST11.lib
SI:93 Formula:C17H34 CAS:6765-39-5 MolWeight:238 RetIndex:1701
CompName:1-Heptadecene $$ Hexahydroaplotaxene $$ Heptadec-1-ene $$ NSC 77132 $$ Heptadecene (Related) $$

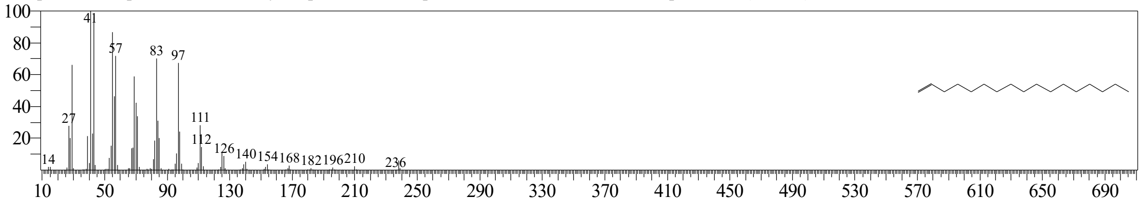

Hit#:4 Entry:72459 Library:NIST11.lib
SI:93 Formula:C16H32O CAS:64437-47-4 MolWeight:240 RetIndex:1862
CompName:Hexadecen-1-ol, trans-9- $$ (9E)-9-Hexadecen-1-ol $$ 9-Hexadecenol, E $$ (E)9-Hexadecen-1-ol $$

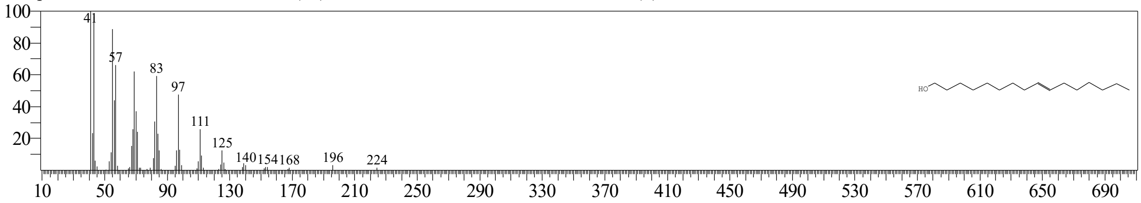

Hit#:5 Entry:24832 Library:NIST11.lib
SI:93 Formula:C12H24 CAS:112-41-4 MolWeight:168 RetIndex:1204
CompName:1-Dodecene $$ .alpha.-Dodecene $$ n-Dodec-1-ene $$ Adacene 12 $$ Dodec-1-ene $$ .alpha.-Dodecylene $$ Dodecylene .alpha.- $$ Dodecene-1 $

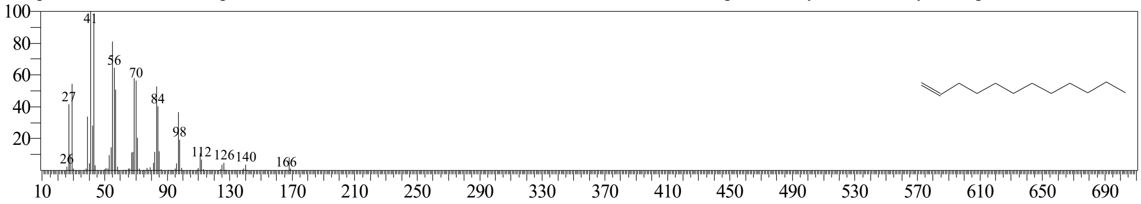

<< Target >>
Line#:86 R.Time:11.370(Scan#:1475) MassPeaks:393
RawMode:Averaged 11.365-11.375(1474-1476) BasePeak:43.05(56394)
BG Mode:Calc. from Peak Group 1 - Event 1 Scan

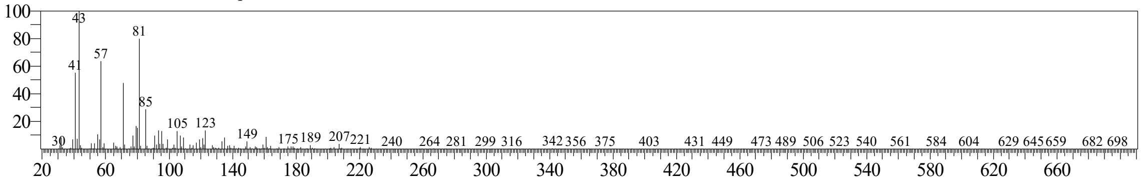

Hit#:1 Entry:59399 Library:NIST11.lib
SI:75 Formula:C15H26O CAS:72120-50-4 MolWeight:222 RetIndex:1660
CompName:1-Hydroxy-1,7-dimethyl-4-isopropyl-2,7-cyclodecadiene $$ Germacrene D-4-ol $$ 4-Isopropyl-1,7-dimethyl-2,7-cyclodecadien-1-ol # $$ Germacre

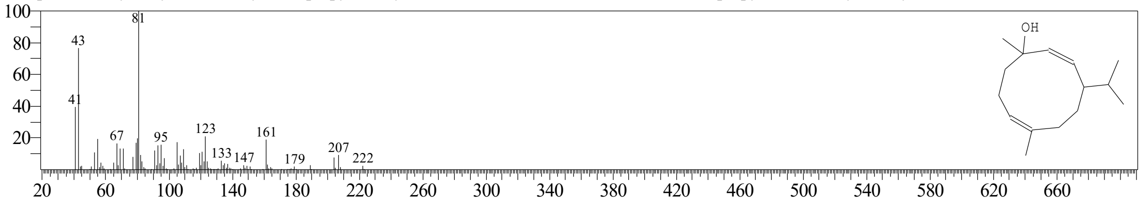

Hit#:2 Entry:112433 Library:NIST11.lib
SI:74 Formula:C19H32O2 CAS:56847-05-3 MolWeight:292 RetIndex:2103
CompName:9-Octadecen-12-ynoic acid, methyl ester $$ Methyl (9E)-9-octadecen-12-ynoate # $$

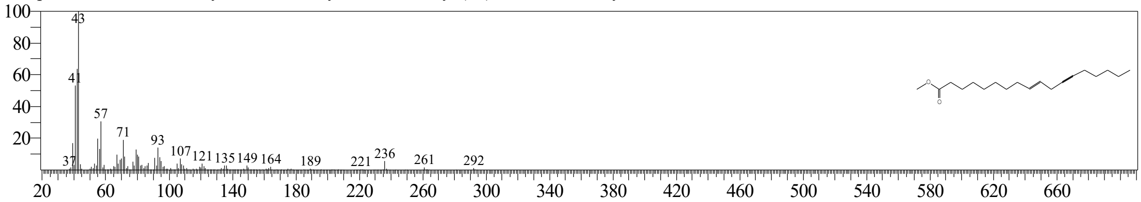

Hit#:3 Entry:70772 Library:NIST11.lib
SI:74 Formula:C15H26O2 CAS:0-00-0 MolWeight:238 RetIndex:1751
CompName:9-(3,3-Dimethyloxiran-2-yl)-2,7-dimethylnona-2,6-dien-1-ol $$ (2E,6E)-9-(3,3-Dimethyl-2-oxiranyl)-2,7-dimethyl-2,6-nonadien-1-ol # $$

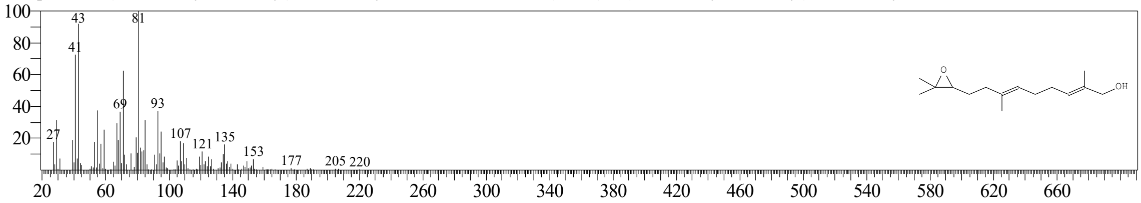

Hit#:4 Entry:92779 Library:NIST11.lib
SI:72 Formula:C10H21I CAS:0-00-0 MolWeight:268 RetIndex:1365
CompName:1-Iodo-2-methylnonane

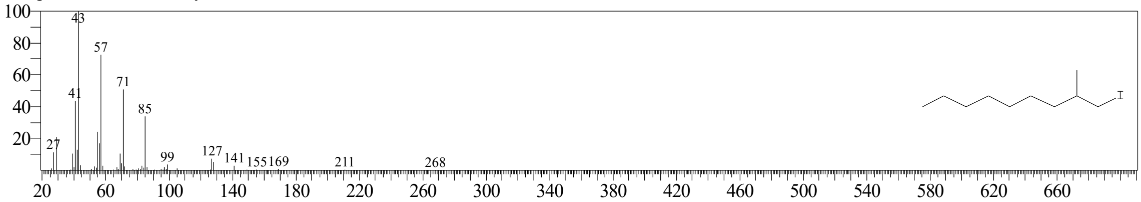

Hit#:5 Entry:70774 Library:NIST11.lib
SI:72 Formula:C15H26O2 CAS:55538-61-9 MolWeight:238 RetIndex:1576
CompName:Pentanoic acid, 1,3,3-trimethylbicyclo[2.2.1]hept-2-yl ester $$ 1,3,3-Trimethylbicyclo[2.2.1]hept-2-yl pentanoate # $$

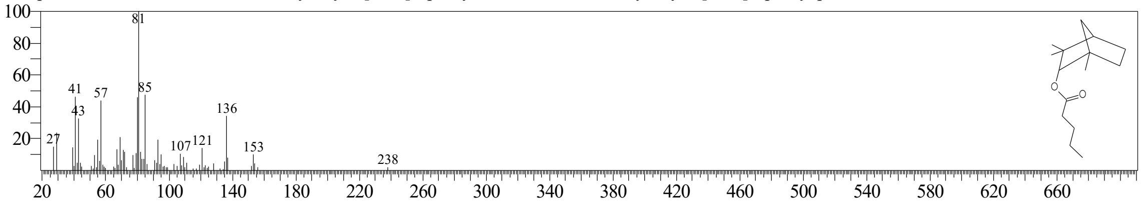

<< Target >>
Line#:87 R.Time:11.435(Scan#:1488) MassPeaks:411
RawMode:Averaged 11.430-11.440(1487-1489) BasePeak:41.10(186437)
BG Mode:Calc. from Peak Group 1 - Event 1 Scan

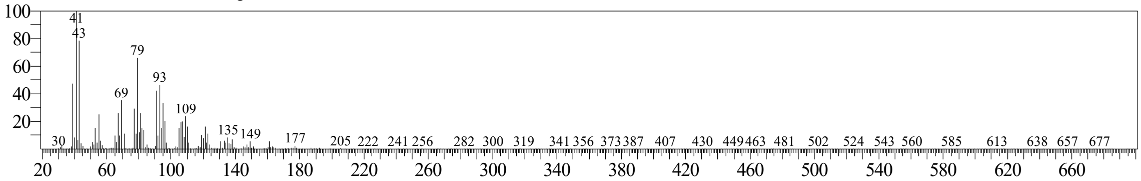

Hit#:1 Entry:57732 Library:NIST11.lib
SI:90 Formula:C15H24O CAS:1139-30-6 MolWeight:220 RetIndex:1507
CompName:Caryophyllene oxide $$ 5-Oxatricyclo[8.2.0.0(4,6)-]dodecane, 4,12,12-trimethyl-9-methylene-, [1R-(1R*,4R*,6R*,10S*)]- $$ 5-Oxatricyclo(8.2.0.

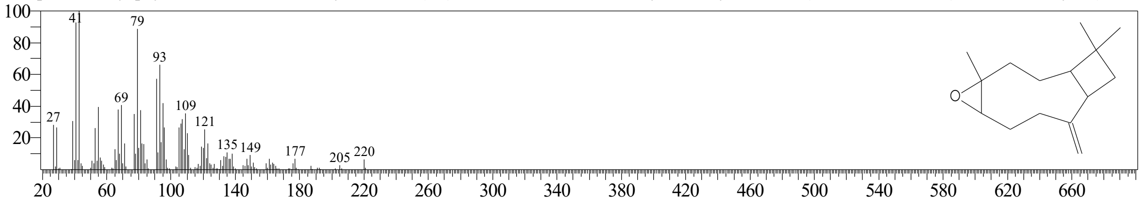

Hit#:2 Entry:57712 Library:NIST11.lib
SI:85 Formula:C15H24O CAS:0-00-0 MolWeight:220 RetIndex:1281
CompName:Isoaromadendrene epoxide $$ 1,3b,6,6-Tetramethyldecahydro-1H-cyclopropa[7,8]azuleno[4,5-b]oxirene # $$

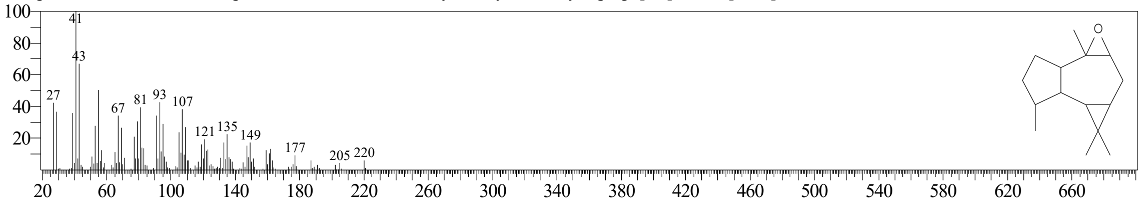

Hit#:3 Entry:48070 Library:NIST11.lib
SI:85 Formula:C15H26 CAS:25491-20-7 MolWeight:206 RetIndex:1393
CompName:1H-3a,7-Methanoazulene, octahydro-1,4,9,9-tetramethyl- $$ Patchoulane $$

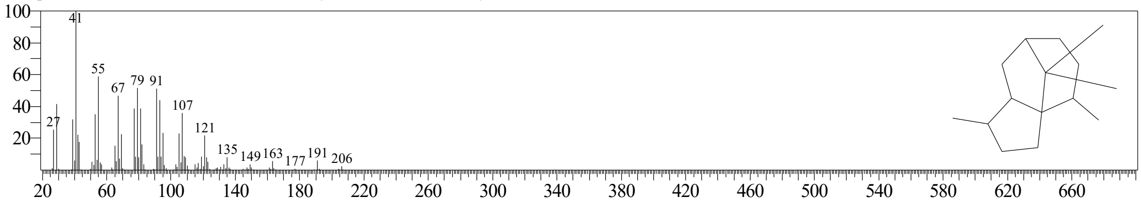

Hit#:4 Entry:67981 Library:NIST11.lib
SI:84 Formula:C17H30 CAS:74744-55-1 MolWeight:234 RetIndex:1736
CompName:3-Heptadecen-5-yne, (Z)- $$ (3Z)-3-Heptadecen-5-yne # $$

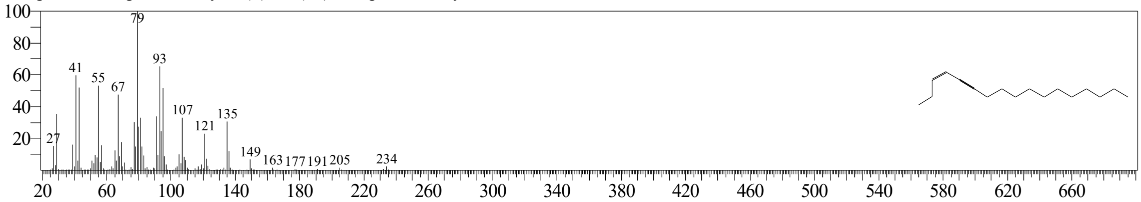

Hit#:5 Entry:86005 Library:NIST11.lib
SI:84 Formula:C18H26O CAS:0-00-0 MolWeight:258 RetIndex:1811
CompName:1,3-Bis-(2-cyclopropyl,2-methylcyclopropyl)-but-2-en-1-one

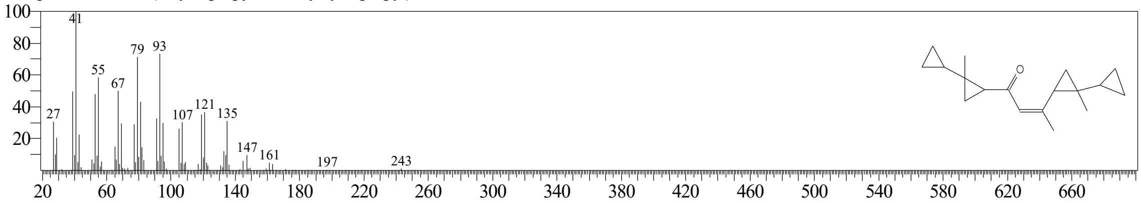

<< Target >>
Line#:88 R.Time:11.550(Scan#:1511) MassPeaks:403
RawMode:Averaged 11.545-11.555(1510-1512) BasePeak:43.10(32632)
BG Mode:Calc. from Peak Group 1 - Event 1 Scan

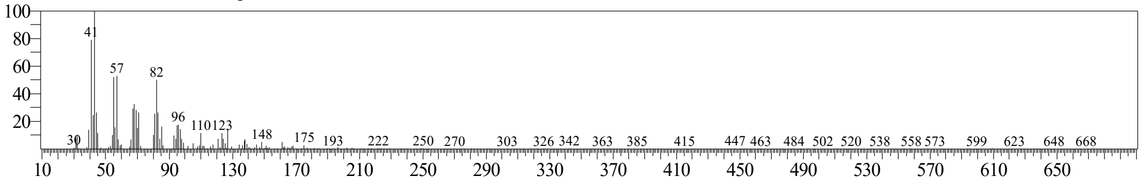

Hit#:1 Entry:93586 Library:NIST11.lib
SI:87 Formula:C18H36O CAS:638-66-4 MolWeight:268 RetIndex:1999
CompName:Octadecanal $$ Stearaldehyde $$ Octadecyl aldehyde $$ Stearyl aldehyde $$ n-Octadecanal $$

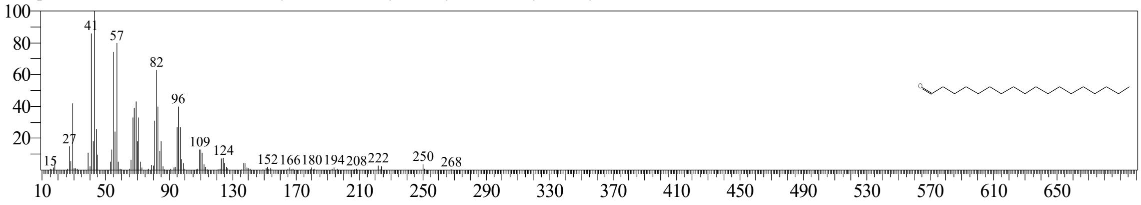

Hit#:2 Entry:26030 Library:NIST11.lib
SI:86 Formula:C11H22O CAS:112-44-7 MolWeight:170 RetIndex:1303
CompName:Undecanal $$ n-Undecanal $$ n-Undecyl aldehyde $$ Hendecanal $$ Hendecanaldehyde $$ Undecyl aldehyde $$ Undecylic aldehyde $$ 1-Undecan

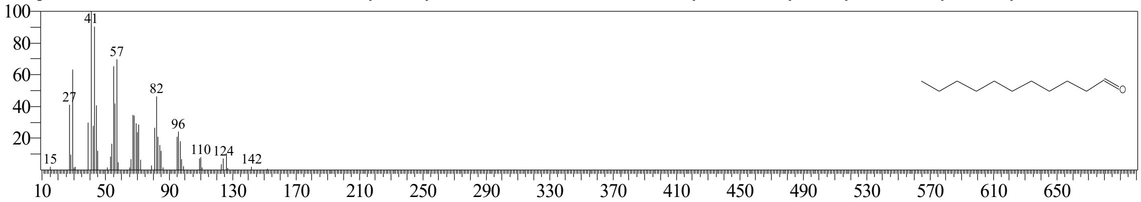

Hit#:3 Entry:42882 Library:NIST11.lib
SI:86 Formula:C13H26O CAS:74962-98-4 MolWeight:198 RetIndex:1564
CompName:2-Tridecen-1-ol, (E)- $$ trans-2-Tridecen-1-ol $$ (2E)-2-Tridecen-1-ol $$ E-2-Tridecen-1-ol $$

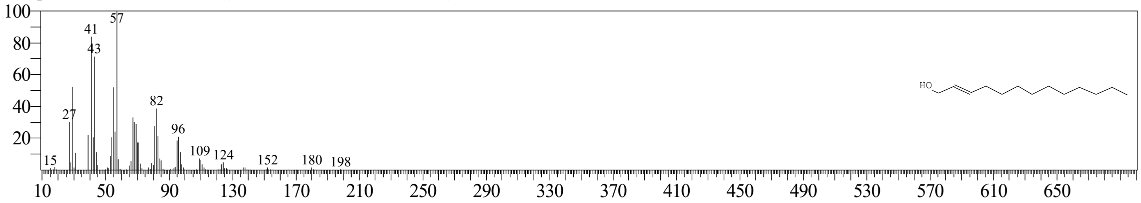

Hit#:4 Entry:52414 Library:NIST11.lib
SI:86 Formula:C14H28O CAS:3234-28-4 MolWeight:212 RetIndex:1503
CompName:Oxirane, dodecyl- $$ 1,2-Epoxytetradecane $$ 2-Dodecyloxirane # $$

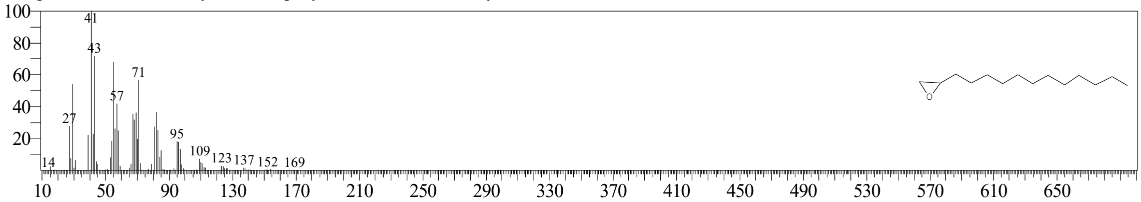

Hit#:5 Entry:52426 Library:NIST11.lib
SI:86 Formula:C14H28O CAS:0-00-0 MolWeight:212 RetIndex:1664
CompName:E-2-Tetradecen-1-ol $$ (2E)-2-Tetradecen-1-ol # $$

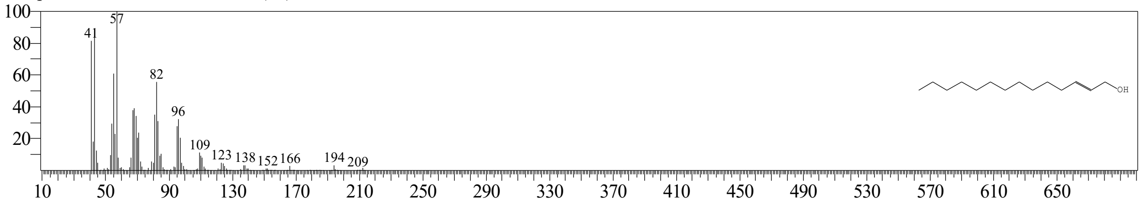

<< Target >>
Line#:89 R.Time:11.660(Scan#:1533) MassPeaks:417
RawMode:Averaged 11.655-11.665(1532-1534) BasePeak:43.05(98636)
BG Mode:Calc. from Peak Group 1 - Event 1 Scan

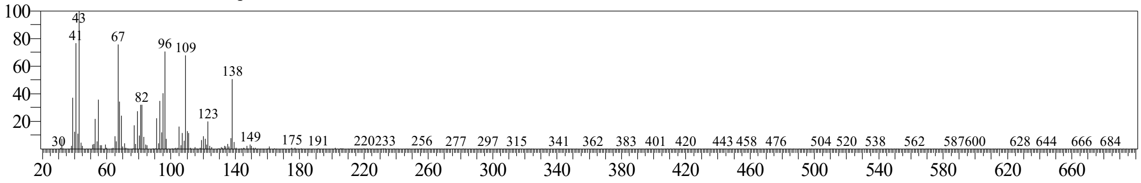

Hit#:1 Entry:16347 Library:NIST11.lib
SI:84 Formula:C10H16O CAS:1686-14-2 MolWeight:152 RetIndex:961
CompName:3-Oxatricyclo[4.1.1.0(2,4)]octane, 2,7,7-trimethyl- $$ Pinane, 2,3-epoxy- $$ .alpha.-Pinene epoxide $$ .alpha.-Pinene oxide $$ 2,3-Epoxypinane $$

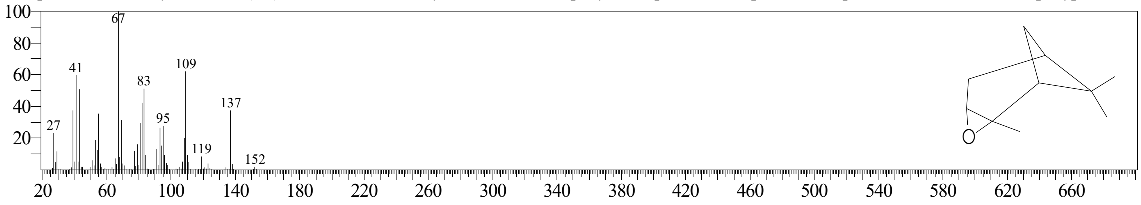

Hit#:2 Entry:10360 Library:NIST11.lib
SI:83 Formula:C9H14O CAS:0-00-0 MolWeight:138 RetIndex:1130
CompName:3-Cyclohexen-1-carboxaldehyde, 3,4-dimethyl- $$ 3,4-Dimethyl-3-cyclohexene-1-carbaldehyde # $$

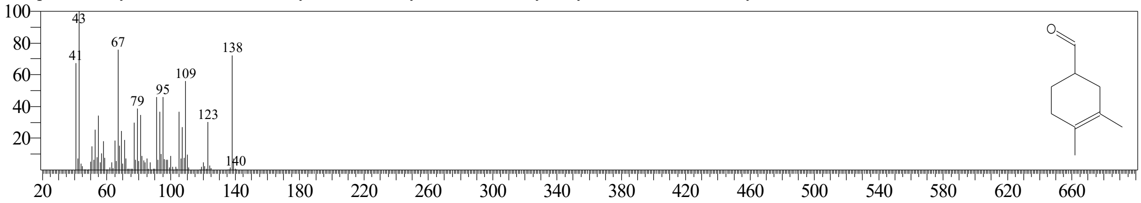

Hit#:3 Entry:16366 Library:NIST11.lib
SI:83 Formula:C10H16O CAS:6931-54-0 MolWeight:152 RetIndex:1020
CompName:Spiro[bicyclo[3.1.1]heptane-2,2'-oxirane], 6,6-dimethyl- $$ .beta.-Pinene oxide $$ Pinane, 2,10-epoxy- $$ .beta.-Pinene epoxide $$ 2,10-Epoxypin

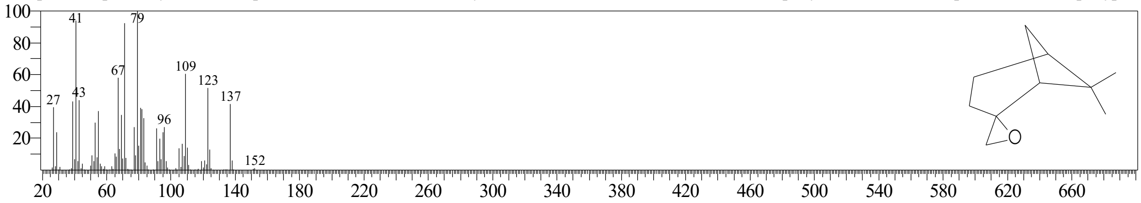

Hit#:4 Entry:24641 Library:NIST11.lib
SI:83 Formula:C10H16O2 CAS:0-00-0 MolWeight:168 RetIndex:1446
CompName:3-Hydroxy-2-(2-methylcyclohex-1-enyl)propionaldehyde $$ 3-Hydroxy-2-(2-methyl-1-cyclohexen-1-yl)propanal # $$

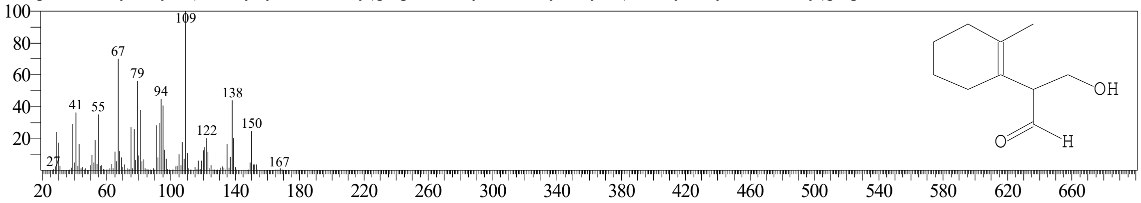

Hit#:5 Entry:57741 Library:NIST11.lib
SI:82 Formula:C15H24O CAS:19888-34-7 MolWeight:220 RetIndex:1592
CompName:12-Oxabicyclo[9.1.0]dodeca-3,7-diene, 1,5,5,8-tetramethyl-, [1R-(1R*,3E,7E,11R*)]- $$ 1,5,5,8-Tetramethyl-12-oxabicyclo[9.1.0]dodeca-3,7-dien

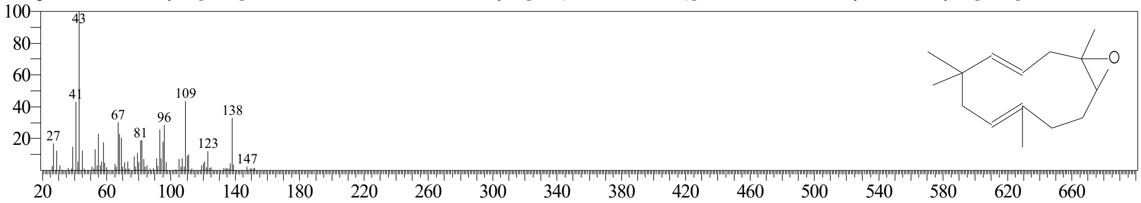

<< Target >>
Line#:90 R.Time:11.815(Scan#:1564) MassPeaks:379
RawMode:Averaged 11.810-11.820(1563-1565) BasePeak:161.10(12479)
BG Mode:Calc. from Peak Group 1 - Event 1 Scan

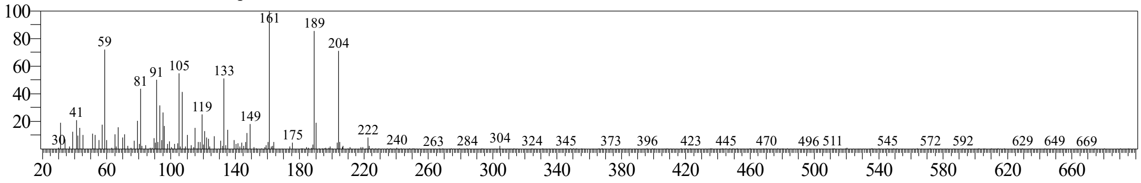

Hit#:1 Entry:59451 Library:NIST11.lib
SI:83 Formula:C15H26O CAS:0-00-0 MolWeight:222 RetIndex:1626
CompName:8-epi-.gama.-eudesmol

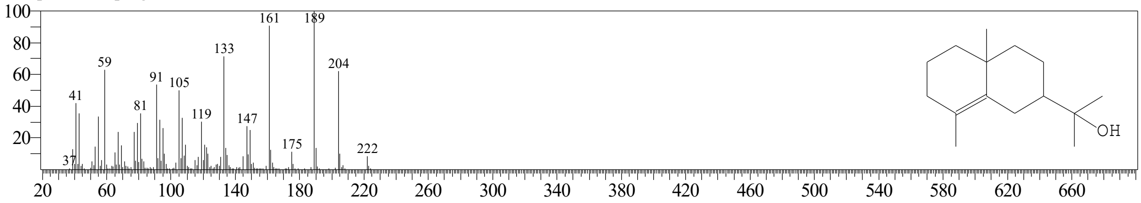

Hit#:2 Entry:59452 Library:NIST11.lib
SI:82 Formula:C15H26O CAS:0-00-0 MolWeight:222 RetIndex:1626
CompName:.gama.-eudesmol

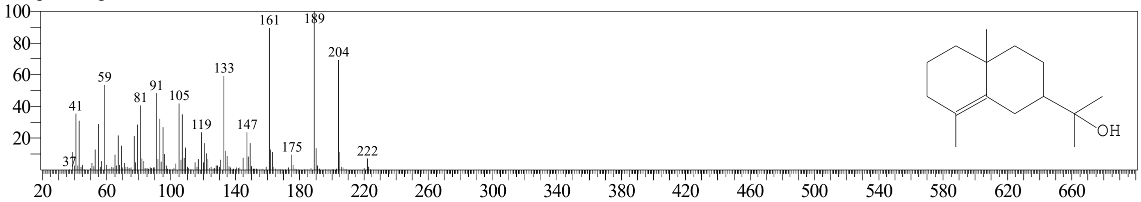

Hit#:3 Entry:59454 Library:NIST11.lib
SI:79 Formula:C15H26O CAS:1209-71-8 MolWeight:222 RetIndex:1626
CompName:2-Naphthalenemethanol, 1,2,3,4,4a,5,6,7-octahydro-.alpha.,.alpha.,4a,8-tetramethyl-, (2R-cis)- $$ .gamma.-Eudesmol $$ .gamma.-Eudesmole $$ [2[illegible]

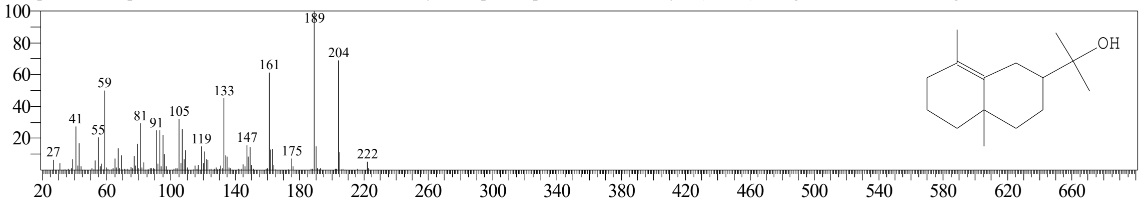

Hit#:4 Entry:46766 Library:NIST11.lib
SI:77 Formula:C15H24 CAS:489-29-2 MolWeight:204 RetIndex:1432
CompName:1H-Cyclopropa[a]naphthalene, 1a,2,3,3a,4,5,6,7b-octahydro-1,1,3a,7-tetramethyl-, [1aR-(1a.alpha.,3a.alpha.,7b.alpha.)]- $$ 1H-Cyclopropa[a]naph[illegible]

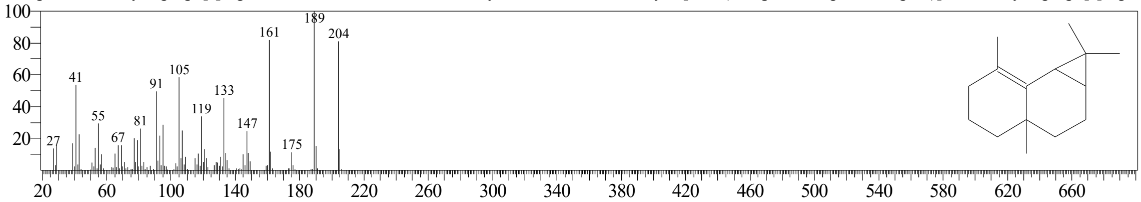

Hit#:5 Entry:59382 Library:NIST11.lib
SI:77 Formula:C15H26O CAS:13822-35-0 MolWeight:222 RetIndex:1614
CompName:5-Azulenemethanol, 1,2,3,4,5,6,7,8-octahydro-.alpha.,.alpha.,3,8-tetramethyl- $$ 2-(3,8-Dimethyl-1,2,3,4,5,6,7,8-octahydro-5-azulenyl)-2-propanol

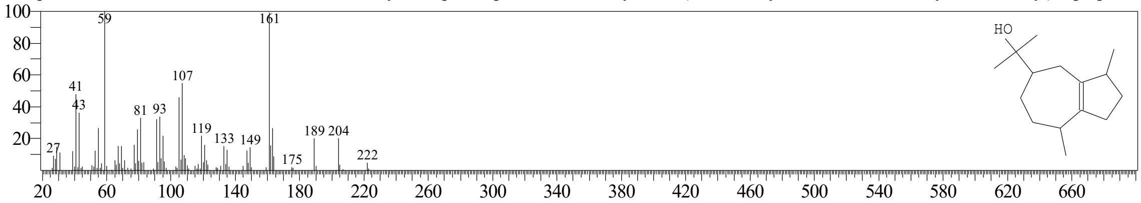

<< Target >>
Line#:91 R.Time:11.880(Scan#:1577) MassPeaks:388
RawMode:Averaged 11.875-11.885(1576-1578) BasePeak:161.15(45881)
BG Mode:Calc. from Peak Group 1 - Event 1 Scan

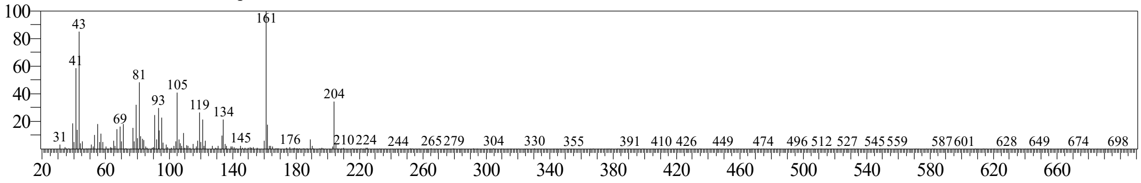

Hit#:1 Entry:59440 Library:NIST11.lib
SI:88 Formula:C15H26O CAS:19435-97-3 MolWeight:222 RetIndex:1580
CompName:1-Naphthalenol, 1,2,3,4,4a,7,8,8a-octahydro-1,6-dimethyl-4-(1-methylethyl)-, [1R-(1.alpha.,4.beta.,4a.beta.,8a.beta.)]- $$ 1.beta.-Cadin-4-en-10-ol

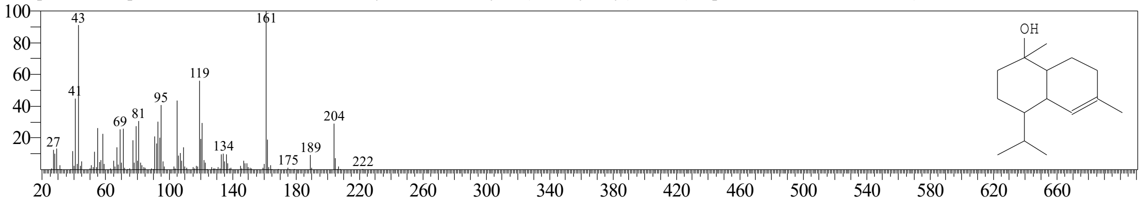

Hit#:2 Entry:59439 Library:NIST11.lib
SI:88 Formula:C15H26O CAS:5937-11-1 MolWeight:222 RetIndex:1580
CompName:.tau.-Cadinol $$ 4-Isopropyl-1,6-dimethyl-1,2,3,4,4a,7,8,8a-octahydro-1-naphthalenol-, (1S-(1alpha,4alpha,4aalpha,8abeta))- $$ .alpha.-epi-Cadino

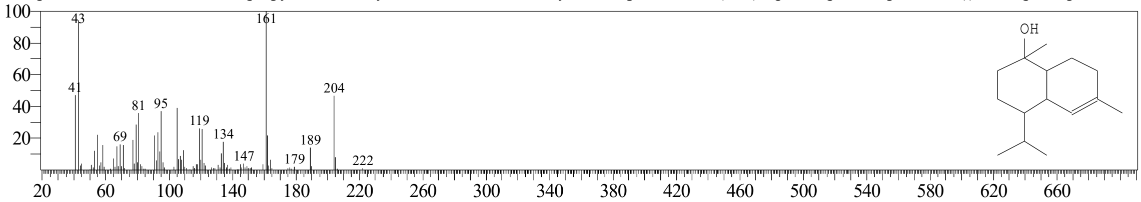

Hit#:3 Entry:59441 Library:NIST11.lib
SI:87 Formula:C15H26O CAS:133645-25-7 MolWeight:222 RetIndex:1580
CompName:1-Naphthalenol, 1,2,3,4,4a,7,8,8a-octahydro-1,6-dimethyl-4-(1-methylethyl)-, [1S-(1.alpha.,4.alpha.,4a.beta.,8a.beta.)]- $$ .delta.-Cedrol $$ 4-Isopr

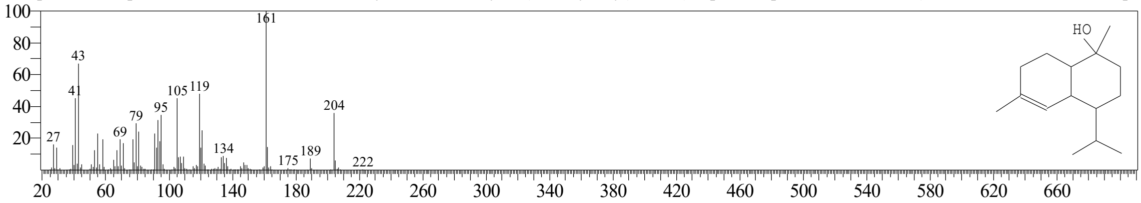

Hit#:4 Entry:59372 Library:NIST11.lib
SI:83 Formula:C15H26O CAS:19912-62-0 MolWeight:222 RetIndex:1580
CompName:.tau.-Muurolol $$ 4-Isopropyl-1,6-dimethyl-1,2,3,4,4a,7,8,8a-octahydro-1-naphthalenol-, [1S-(1.alpha.,4.alpha.,4a.alpha.,8a.alpha.)]- $$ .alpha.-epi

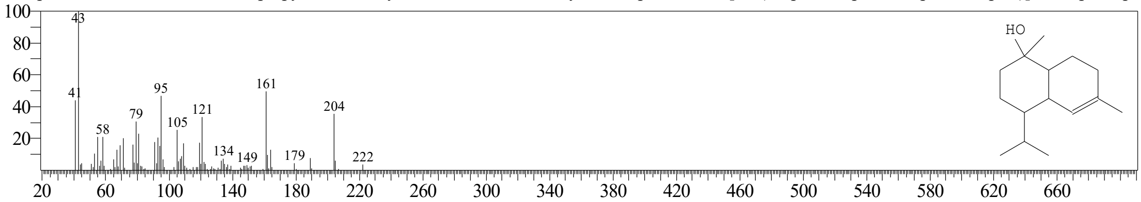

Hit#:5 Entry:59446 Library:NIST11.lib
SI:83 Formula:C15H26O CAS:0-00-0 MolWeight:222 RetIndex:1484
CompName:4-epi-cubedol

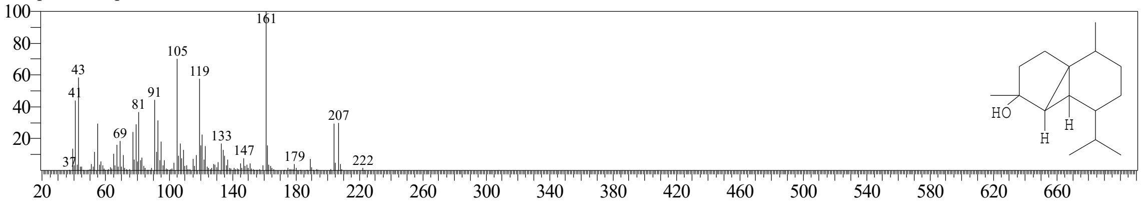

<< Target >>
Line#:92 R.Time:12.030(Scan#:1607) MassPeaks:391
RawMode:Averaged 12.025-12.035(1606-1608) BasePeak:59.05(126337)
BG Mode:Calc. from Peak Group 1 - Event 1 Scan

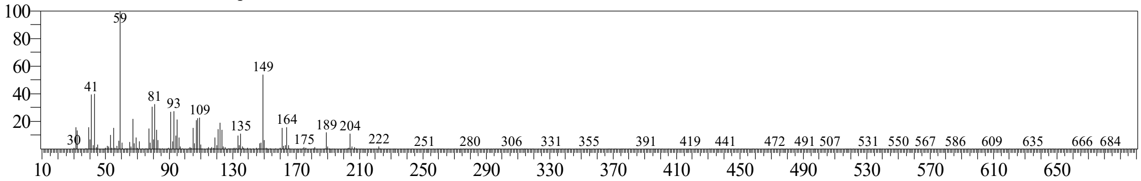

Hit#:1 Entry:59380 Library:NIST11.lib
SI:90 Formula:C15H26O CAS:473-15-4 MolWeight:222 RetIndex:1593
CompName:2-Naphthalenemethanol, decahydro-.alpha.,.alpha.,4a-trimethyl-8-methylene-, [2R-(2.alpha.,4a.alpha.,8a.beta.)]- $$ Eudesm-4(14)-en-11-ol $$ .beta

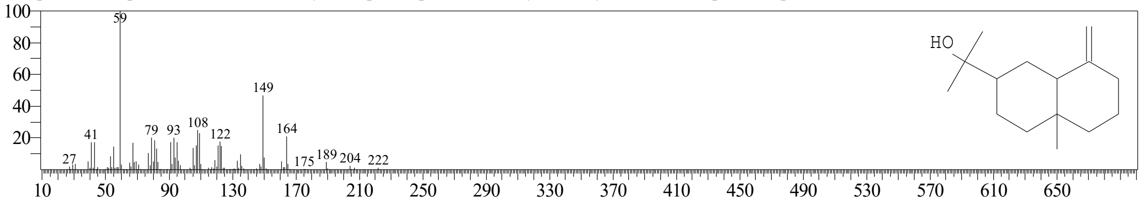

Hit#:2 Entry:59379 Library:NIST11.lib
SI:85 Formula:C15H26O CAS:63891-61-2 MolWeight:222 RetIndex:1598
CompName:2-Naphthalenemethanol, 2,3,4,4a,5,6,7,8-octahydro-.alpha.,.alpha.,4a,8-tetramethyl-, [2R-(2.alpha.,4a.beta.,8.beta.)]- $$ 2-(4a,8-Dimethyl-2,3,4,4a,

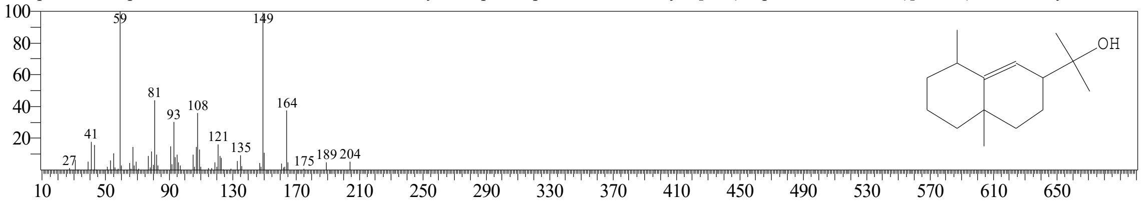

Hit#:3 Entry:59377 Library:NIST11.lib
SI:85 Formula:C15H26O CAS:21657-90-9 MolWeight:222 RetIndex:1694
CompName:3,7-Cyclodecadiene-1-methanol, .alpha.,.alpha.,4,8-tetramethyl-, [s-(Z,Z)] $$ Hedycaryol $$ 2-(4,8-Dimethyl-3,7-cyclodecadien-1-yl)-2-propanol #

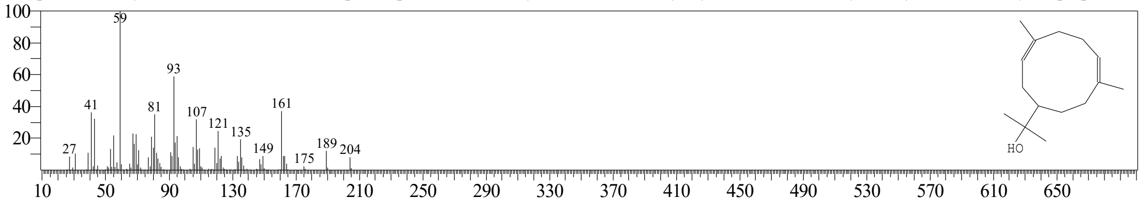

Hit#:4 Entry:59376 Library:NIST11.lib
SI:84 Formula:C15H26O CAS:639-99-6 MolWeight:222 RetIndex:1522
CompName:Cyclohexanemethanol, 4-ethenyl-.alpha.,.alpha.,4-trimethyl-3-(1-methylethenyl)-, [1R-(1.alpha.,3.alpha.,4.beta.)]- $$ o-Menth-8-ene-4-methanol, .a

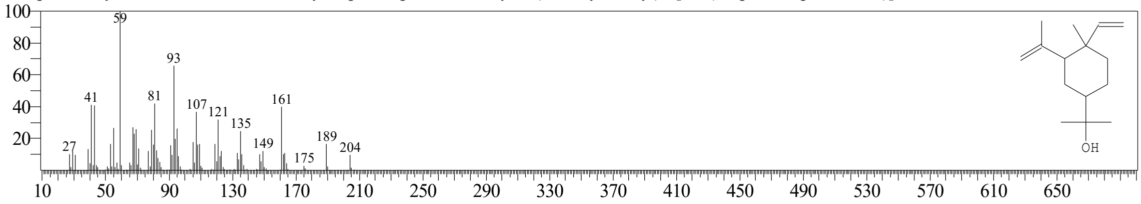

Hit#:5 Entry:59382 Library:NIST11.lib
SI:83 Formula:C15H26O CAS:13822-35-0 MolWeight:222 RetIndex:1614
CompName:5-Azulenemethanol, 1,2,3,4,5,6,7,8-octahydro-.alpha.,.alpha.,3,8-tetramethyl- $$ 2-(3,8-Dimethyl-1,2,3,4,5,6,7,8-octahydro-5-azulenyl)-2-propanol

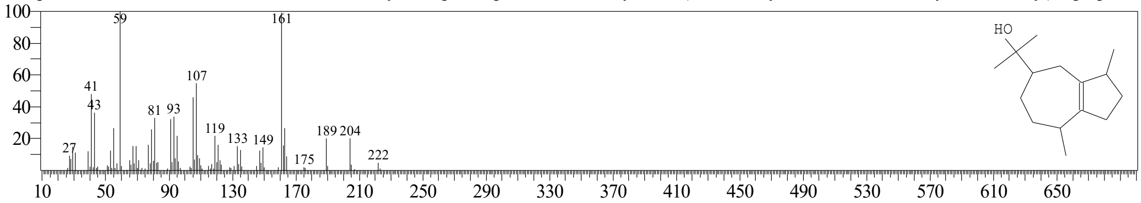

<< Target >>
Line#:93 R.Time:12.150(Scan#:1631) MassPeaks:326
RawMode:Averaged 12.145-12.155(1630-1632) BasePeak:57.10(18173)
BG Mode:Calc. from Peak Group 1 - Event 1 Scan

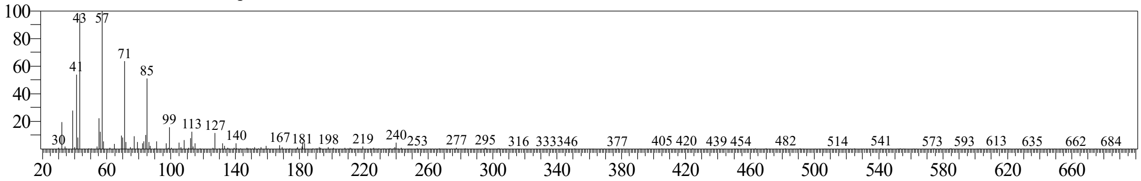

Hit#:1 Entry:18707 Library:NIST11.lib
SI:86 Formula:C11H24 CAS:62016-30-2 MolWeight:156 RetIndex:966
CompName:Octane, 2,3,3-trimethyl- $$ 2,3,3-Trimethyloctane # $$

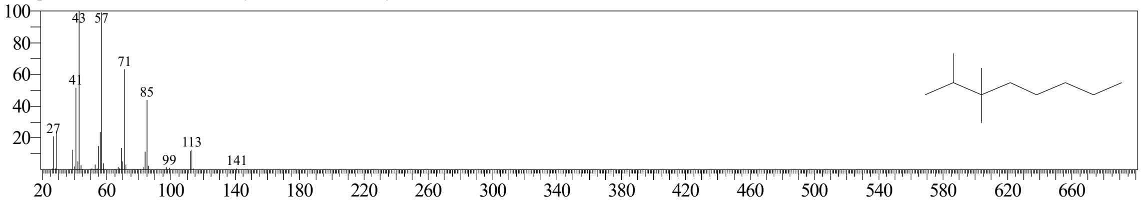

Hit#:2 Entry:62492 Library:NIST11.lib
SI:85 Formula:C16H34 CAS:55045-07-3 MolWeight:226 RetIndex:1483
CompName:Dodecane, 2-methyl-8-propyl- $$ 2-Methyl-8-propyldodecane # $$

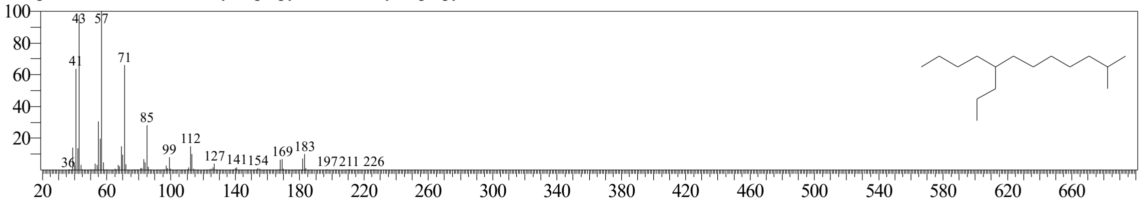

Hit#:3 Entry:62481 Library:NIST11.lib
SI:85 Formula:C16H34 CAS:55045-09-5 MolWeight:226 RetIndex:1548
CompName:Tridecane, 7-propyl- $$ 7-n-Propyltridecane $$ 7-Propyltridecane # $$

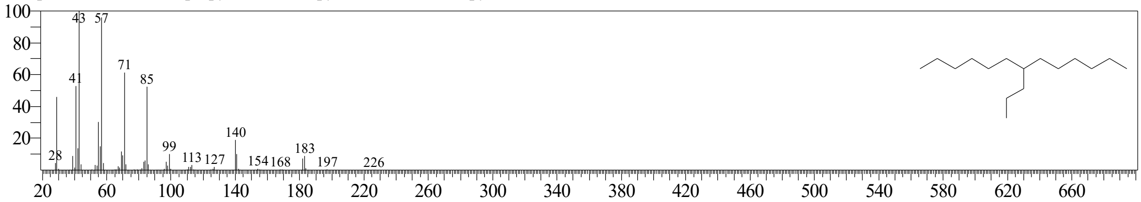

Hit#:4 Entry:104531 Library:NIST11.lib
SI:85 Formula:C20H42 CAS:1560-86-7 MolWeight:282 RetIndex:1945
CompName:Nonadecane, 2-methyl- $$ 2-Methylnonadecane $$

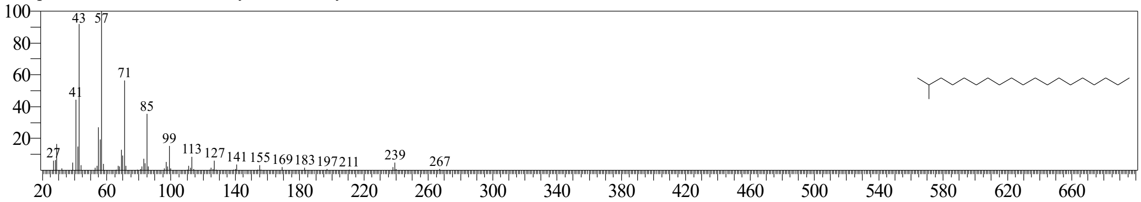

Hit#:5 Entry:62493 Library:NIST11.lib
SI:85 Formula:C16H34 CAS:55045-08-4 MolWeight:226 RetIndex:1483
CompName:Dodecane, 2-methyl-6-propyl- $$ 2-Methyl-6-propyldodecane # $$

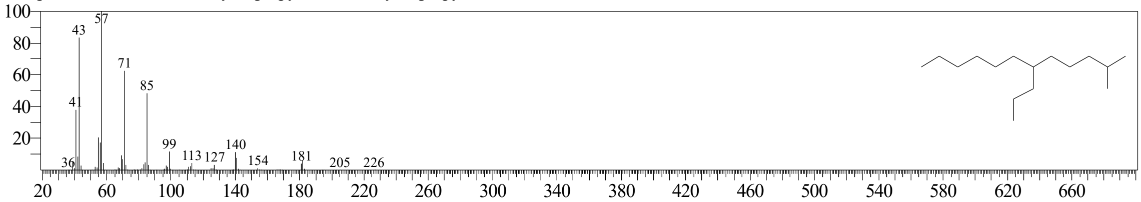

<< Target >>
Line#:94 R.Time:12.185(Scan#:1638) MassPeaks:364
RawMode:Averaged 12.180-12.190(1637-1639) BasePeak:32.10(7113)
BG Mode:Calc. from Peak Group 1 - Event 1 Scan

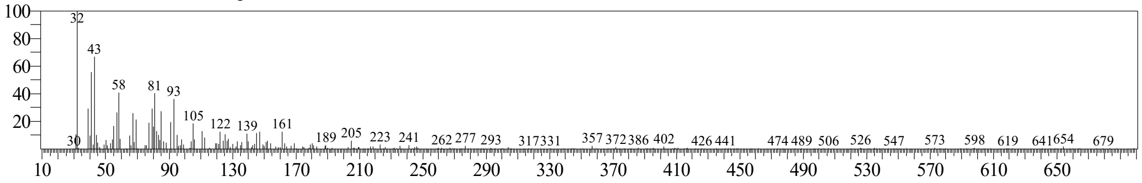

Hit#:1 Entry:17452 Library:NIST11.lib
SI:67 Formula:C10H18O CAS:38092-33-0 MolWeight:154 RetIndex:1100
CompName:trans-2,7-Dimethyl-3,6-octadien-2-ol $$ (3E)-2,7-Dimethyl-3,6-octadien-2-ol $$ 3,6-Octadien-2-ol, 2,7-dimethyl, (E) $$ 3,6-Octadien-2-ol, 2,7-dim

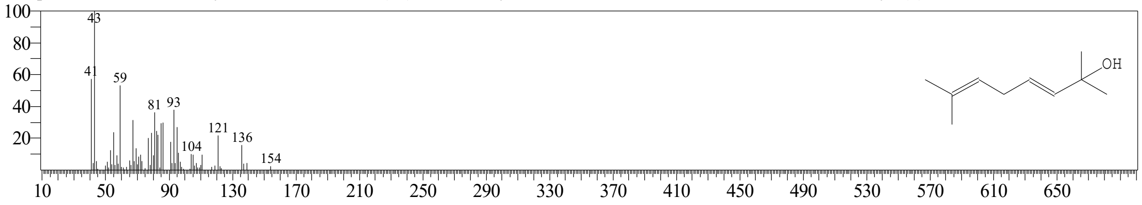

Hit#:2 Entry:24743 Library:NIST11.lib
SI:65 Formula:C11H20O CAS:2774-84-7 MolWeight:168 RetIndex:1355
CompName:10-Undecyn-1-ol

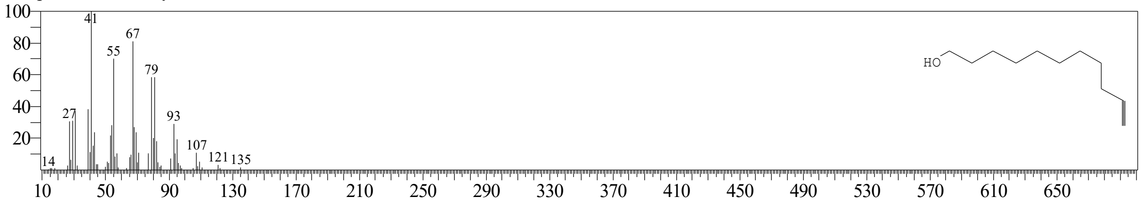

Hit#:3 Entry:11251 Library:NIST11.lib
SI:65 Formula:C9H16O CAS:0-00-0 MolWeight:140 RetIndex:1094
CompName:Nona-3,5-dien-2-ol $$ (3E,5E)-3,5-Nonadien-2-ol # $$

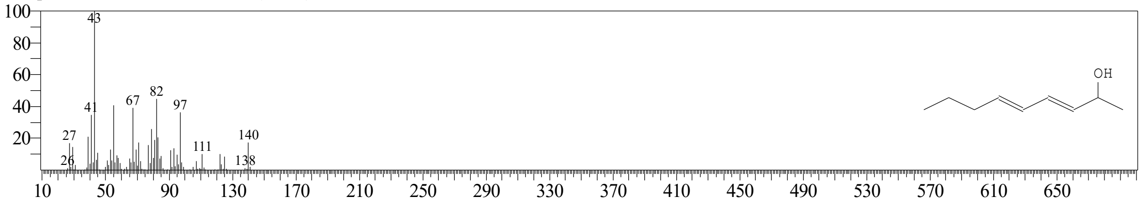

Hit#:4 Entry:24485 Library:NIST11.lib
SI:64 Formula:C10H16O2 CAS:96-08-2 MolWeight:168 RetIndex:1128
CompName:7-Oxabicyclo[4.1.0]heptane, 1-methyl-4-(2-methyloxiranyl)- $$ p-Menthane, 1,2:8,9-diepoxy- $$ .alpha.-Limonene diepoxide $$ Dipentene diepox

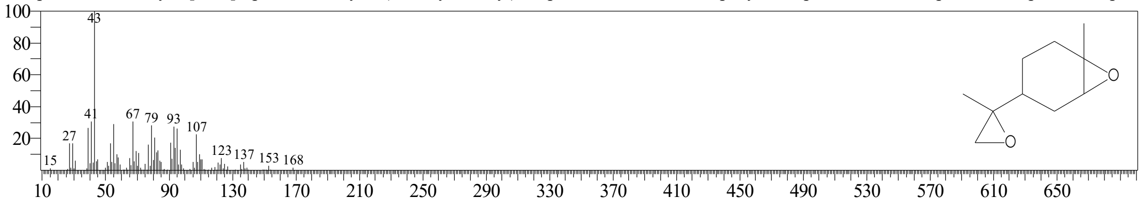

Hit#:5 Entry:17477 Library:NIST11.lib
SI:64 Formula:C10H18O CAS:0-00-0 MolWeight:154 RetIndex:1088
CompName:exo-2,7,7-trimethylbicyclo[2.2.1]heptan-2-ol

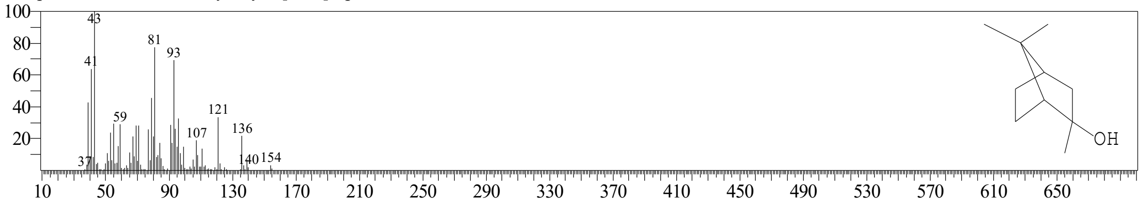

Qualitative Analysis Report

<< Target >>
Line#:95 R.Time:12.260(Scan#:1653) MassPeaks:383
RawMode:Averaged 12.255-12.265(1652-1654) BasePeak:41.05(10968)
BG Mode:Calc. from Peak Group 1 - Event 1 Scan

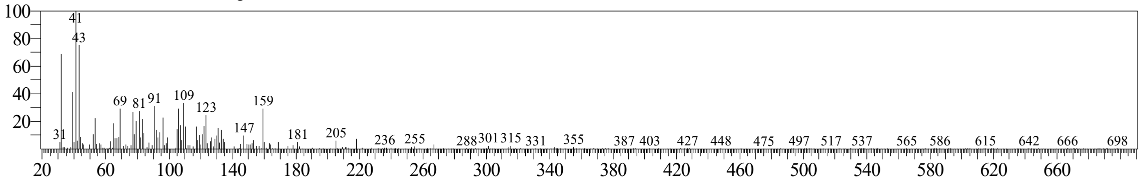

Hit#:1 Entry:57730 Library:NIST11.lib
SI:73 Formula:C15H24O CAS:0-00-0 MolWeight:220 RetIndex:1293
CompName:Ledene oxide-(II) $$ 1,4a,7,7-Tetramethyldecahydrocyclopropa[7,8]azuleno[3a,4-b]oxirene # $$

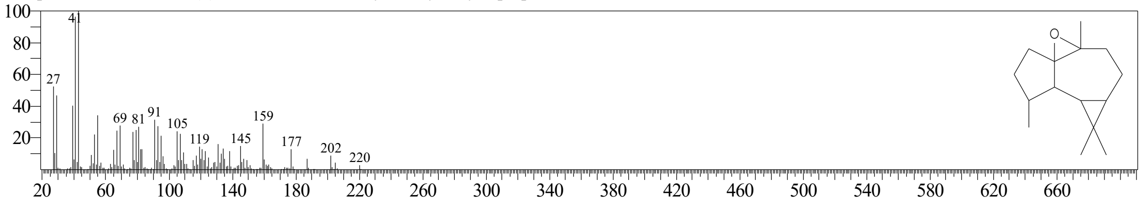

Hit#:2 Entry:57712 Library:NIST11.lib
SI:70 Formula:C15H24O CAS:0-00-0 MolWeight:220 RetIndex:1281
CompName:Isoaromadendrene epoxide $$ 1,3b,6,6-Tetramethyldecahydro-1H-cyclopropa[7,8]azuleno[4,5-b]oxirene # $$

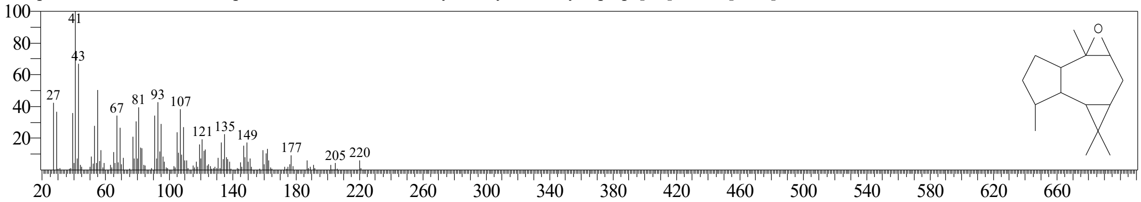

Hit#:3 Entry:57732 Library:NIST11.lib
SI:70 Formula:C15H24O CAS:1139-30-6 MolWeight:220 RetIndex:1507
CompName:Caryophyllene oxide $$ 5-Oxatricyclo[8.2.0.0(4,6)-]dodecane, 4,12,12-trimethyl-9-methylene-, [1R-(1R*,4R*,6R*,10S*)]- $$ 5-Oxatricyclo(8.2.0.

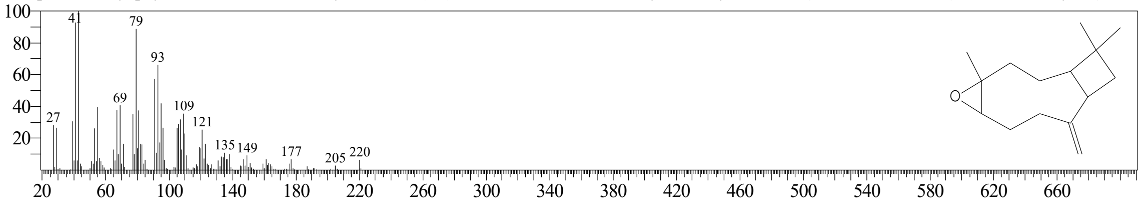

Hit#:4 Entry:23578 Library:NIST11.lib
SI:70 Formula:C11H18O CAS:0-00-0 MolWeight:166 RetIndex:1230
CompName:5-Isopropenyl-1,2-dimethylcyclohex-2-enol $$ 5-Isopropenyl-1,2-dimethyl-2-cyclohexen-1-ol # $$

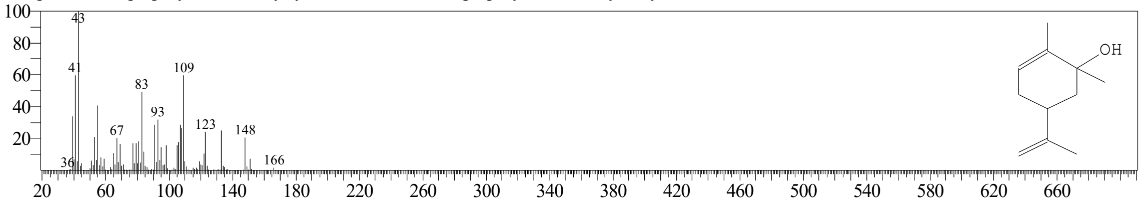

Hit#:5 Entry:57718 Library:NIST11.lib
SI:69 Formula:C15H24O CAS:0-00-0 MolWeight:220 RetIndex:1462
CompName:Aromadendrene oxide-(2)

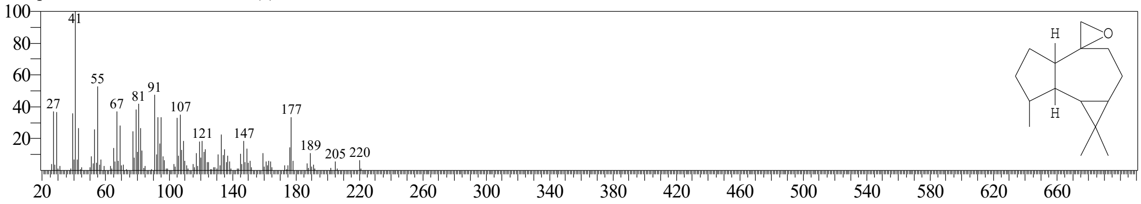

<< Target >>
Line#:96 R.Time:12.315(Scan#:1664) MassPeaks:404
RawMode:Averaged 12.310-12.320(1663-1665) BasePeak:41.10(473872)
BG Mode:Calc. from Peak Group 1 - Event 1 Scan

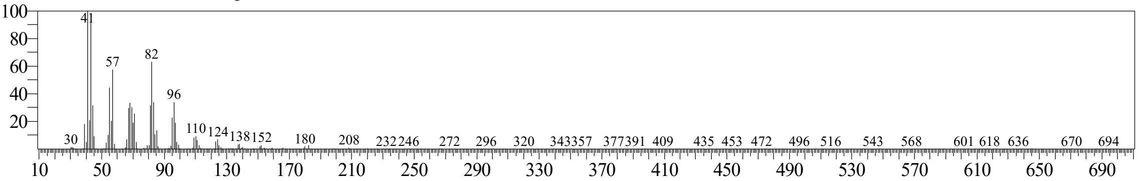

Hit#:1 Entry:93586 Library:NIST11.lib
SI:94 Formula:C18H36O CAS:638-66-4 MolWeight:268 RetIndex:1999
CompName:Octadecanal $$ Stearaldehyde $$ Octadecyl aldehyde $$ Stearyl aldehyde $$ n-Octadecanal $$

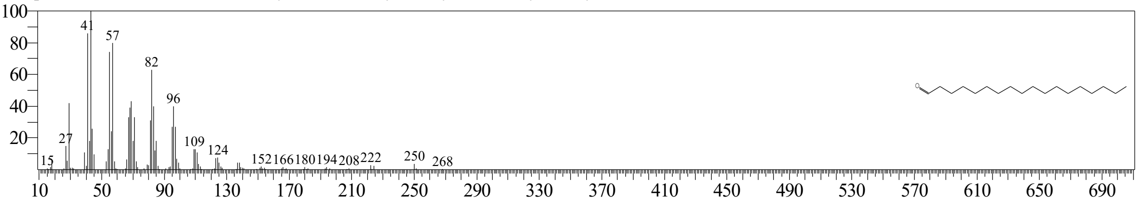

Hit#:2 Entry:26030 Library:NIST11.lib
SI:93 Formula:C11H22O CAS:112-44-7 MolWeight:170 RetIndex:1303
CompName:Undecanal $$ n-Undecanal $$ n-Undecyl aldehyde $$ Hendecanal $$ Hendecanaldehyde $$ Undecyl aldehyde $$ Undecylic aldehyde $$ 1-Undecan

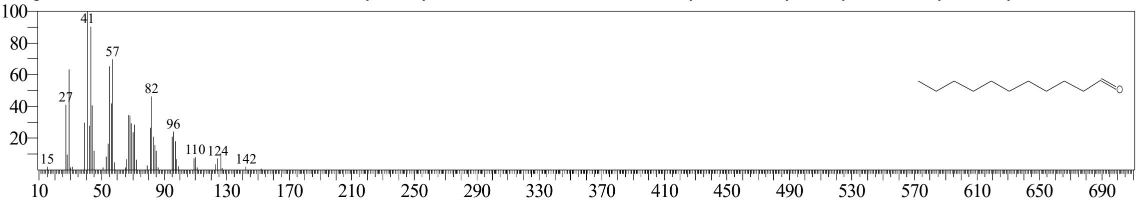

Hit#:3 Entry:42885 Library:NIST11.lib
SI:92 Formula:C13H26O CAS:10486-19-8 MolWeight:198 RetIndex:1502
CompName:Tridecanal $$ n-Tridecylaldehyde $$ Tridecanaldehyde $$ Tridecyl aldehyde $$ 1-Tridecanal $$ Tridecane aldehyde $$ n-Tridecanal $$

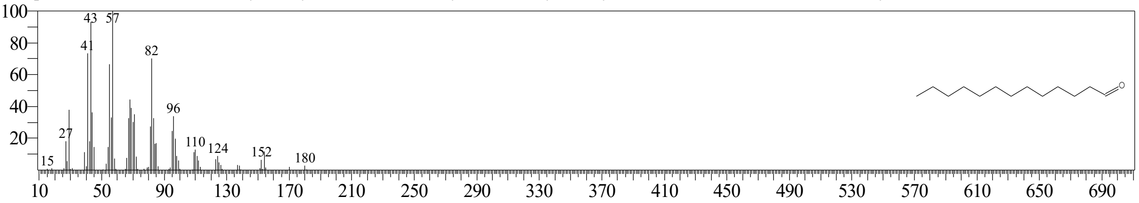

Hit#:4 Entry:52426 Library:NIST11.lib
SI:92 Formula:C14H28O CAS:0-00-0 MolWeight:212 RetIndex:1664
CompName:E-2-Tetradecen-1-ol $$ (2E)-2-Tetradecen-1-ol # $$

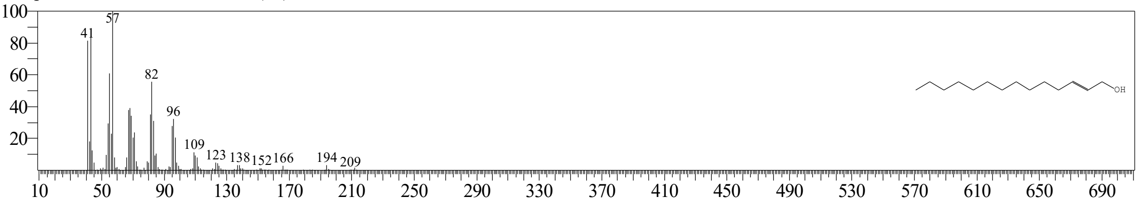

Hit#:5 Entry:34060 Library:NIST11.lib
SI:91 Formula:C12H24O CAS:112-54-9 MolWeight:184 RetIndex:1402
CompName:Dodecanal $$ Lauraldehyde $$ n-Dodecanal $$ n-Dodecyl aldehyde $$ n-Dodecylic aldehyde $$ n-Lauraldehyde $$ Aldehyde C-12 $$ Dodecanalde

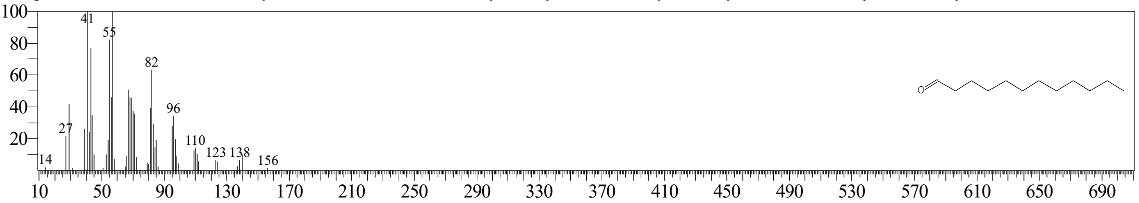

<< Target >>
Line#:97 R.Time:12.445(Scan#:1690) MassPeaks:356
RawMode:Averaged 12.440-12.450(1689-1691) BasePeak:43.05(5826)
BG Mode:Calc. from Peak Group 1 - Event 1 Scan

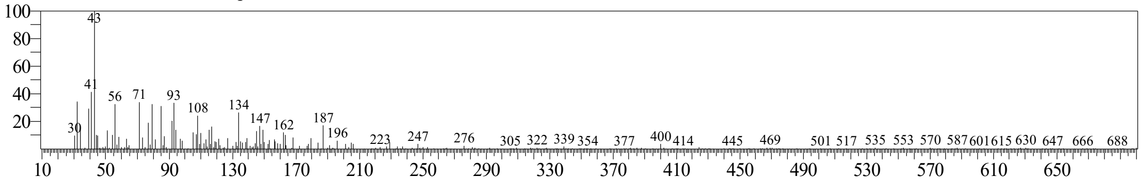

Hit#:1 Entry:30407 Library:NIST11.lib
SI:59 Formula:C12H18O CAS:0-00-0 MolWeight:178 RetIndex:1174
CompName:Bicyclo[3.1.1]hept-3-ene, 4,6,6-trimethyl-2-vinyloxy- $$ 2,6,6-Trimethyl-4-(vinyloxy)bicyclo[3.1.1]hept-2-ene # $$

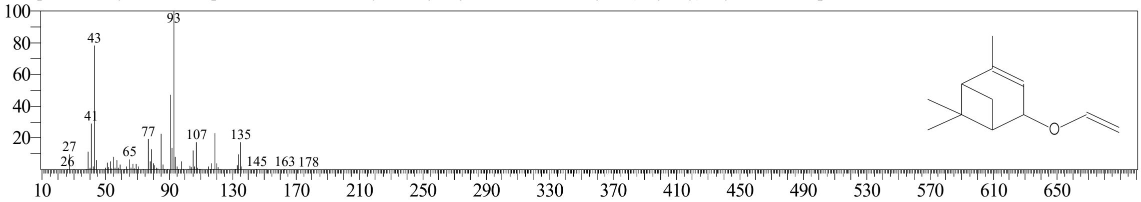

Hit#:2 Entry:69140 Library:NIST11.lib
SI:57 Formula:C14H20O3 CAS:0-00-0 MolWeight:236 RetIndex:1605
CompName:3(10)-Caren-4-ol, acetoacetic acid ester $$ 7,7-Dimethyl-4-methylenebicyclo[4.1.0]hept-3-yl 3-oxobutanoate # $$

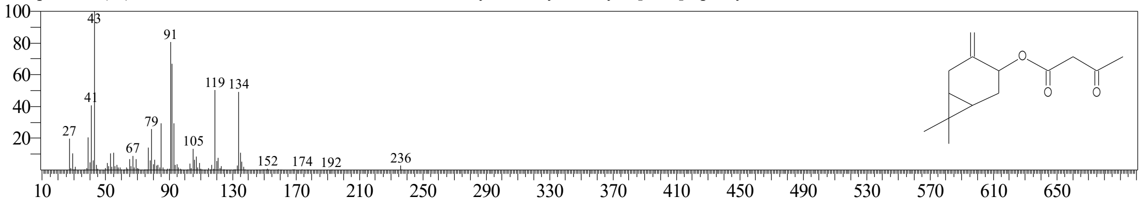

Hit#:3 Entry:176977 Library:NIST11.lib
SI:57 Formula:C19H20N2O7 CAS:62059-48-7 MolWeight:388 RetIndex:2996
CompName:Benzofuran, 7-(2,4-dinitrophenoxy)-2,3-dihydro-2,2-dimethyl-3-(1-methylethoxy)- $$ 7-(2,4-Dinitrophenoxy)-3-isopropoxy-2,2-dimethyl-2,3-dihy

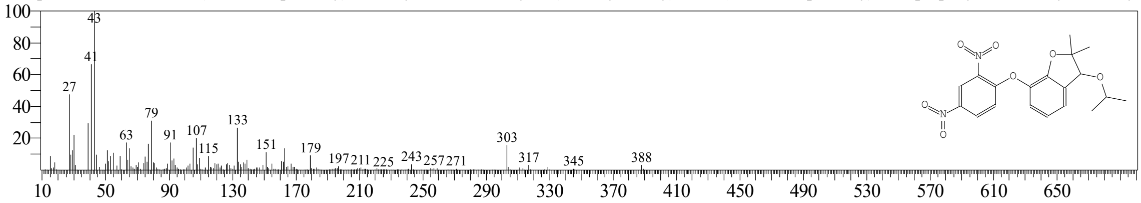

Hit#:4 Entry:38761 Library:NIST11.lib
SI:56 Formula:C13H20O CAS:0-00-0 MolWeight:192 RetIndex:1344
CompName:(+)-3-Carene, 2-(acetylmethyl)- $$ 1-(3,7,7-Trimethylbicyclo[4.1.0]hept-3-en-2-yl)acetone # $$

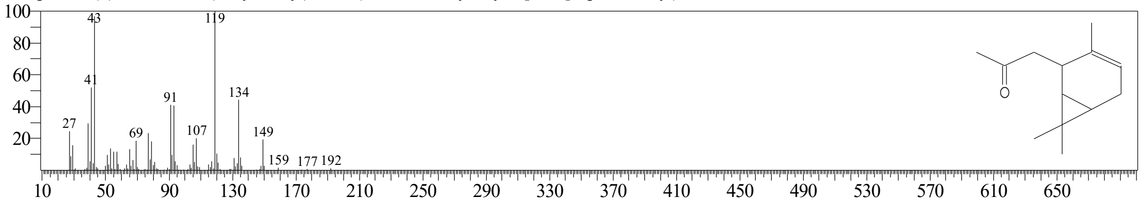

Hit#:5 Entry:41428 Library:NIST11.lib
SI:56 Formula:C12H20O2 CAS:174226-40-5 MolWeight:196 RetIndex:1303
CompName:9,10-Dimethyltricyclo[4.2.1.1(2,5)]decane-9,10-diol

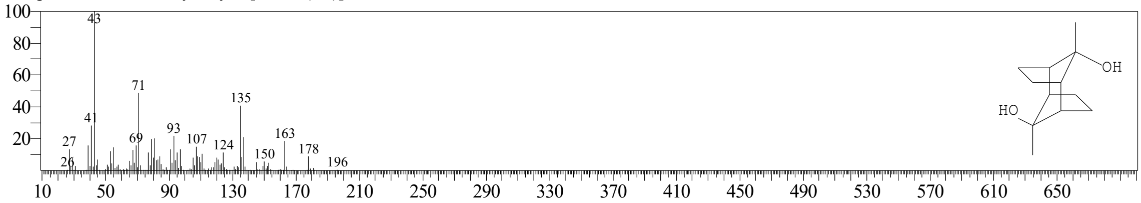

<< Target >>
Line#:98 R.Time:12.505(Scan#:1702) MassPeaks:334
RawMode:Averaged 12.500-12.510(1701-1703) BasePeak:69.05(3038)
BG Mode:Calc. from Peak Group 1 - Event 1 Scan

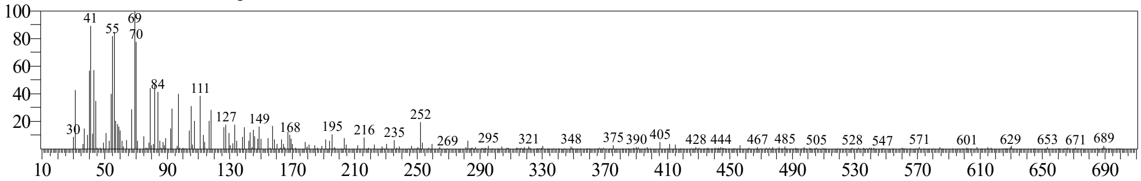

Hit#:1 Entry:27210 Library:NIST11.lib
SI:66 Formula:C11H24O CAS:112-42-5 MolWeight:172 RetIndex:1357
CompName:1-Undecanol $$ Undecyl alcohol $$ n-Undecan-1-ol $$ n-Undecanol $$ n-Undecyl alcohol $$ Hendecanoic alcohol $$ Hendecyl alcohol $$ 1-Hend

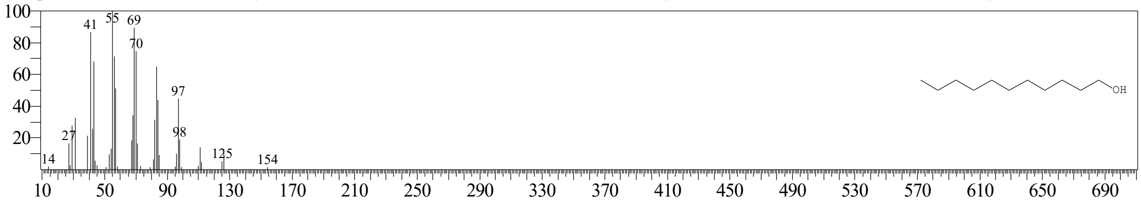

Hit#:2 Entry:19589 Library:NIST11.lib
SI:65 Formula:C10H22O CAS:112-30-1 MolWeight:158 RetIndex:1258
CompName:1-Decanol $$ Decyl alcohol $$ n-Decan-1-ol $$ n-Decanol $$ n-Decyl alcohol $$ Alcohol C10 $$ Alfol 10 $$ Capric alcohol $$ Caprinic alcohol $

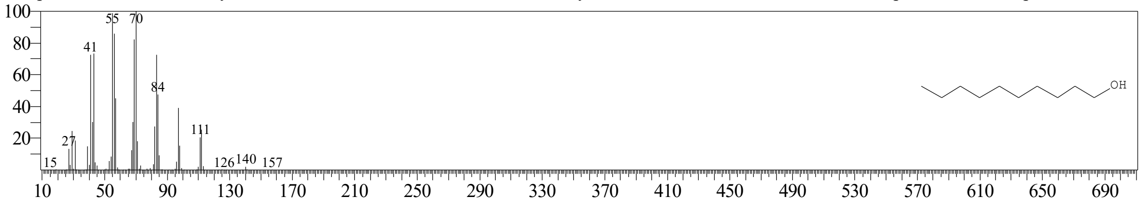

Hit#:3 Entry:11426 Library:NIST11.lib
SI:65 Formula:C10H20 CAS:0-00-0 MolWeight:140 RetIndex:999
CompName:Cyclobutane, 1-butyl-2-ethyl- $$ 1-Butyl-2-ethylcyclobutane # $$

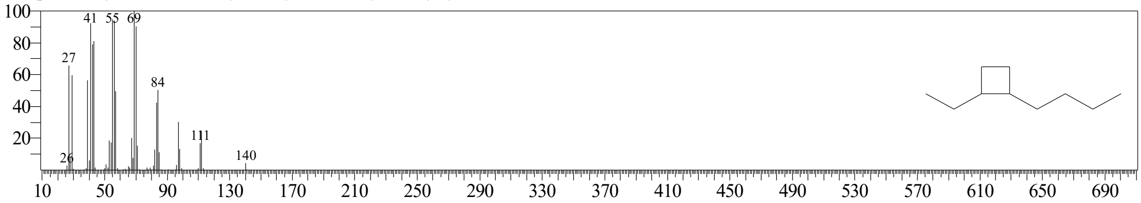

Hit#:4 Entry:35308 Library:NIST11.lib
SI:65 Formula:C12H26O CAS:112-53-8 MolWeight:186 RetIndex:1457
CompName:1-Dodecanol $$ Dodecyl alcohol $$ n-Dodecan-1-ol $$ n-Dodecanol $$ n-Dodecyl alcohol $$ Alcohol C-12 $$ Alfol 12 $$ Dodecanol-1 $$ Dytol J

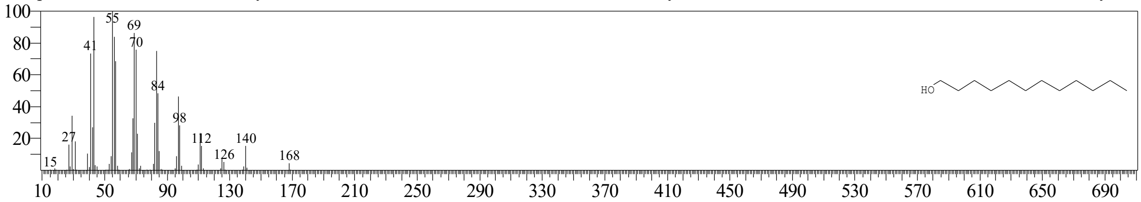

Hit#:5 Entry:24868 Library:NIST11.lib
SI:65 Formula:C12H24 CAS:294-62-2 MolWeight:168 RetIndex:1439
CompName:Cyclododecane

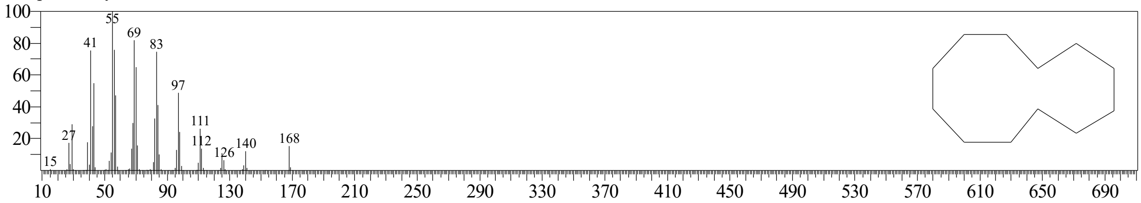

<< Target >>
Line#:99 R.Time:12.550(Scan#:1711) MassPeaks:332
RawMode:Averaged 12.545-12.555(1710-1712) BasePeak:43.00(8379)
BG Mode:Calc. from Peak Group 1 - Event 1 Scan

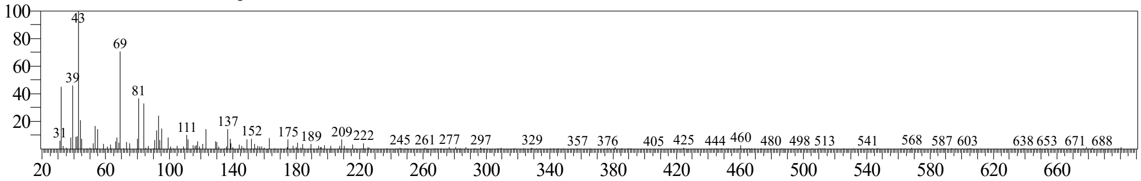

Hit#:1 Entry:41427 Library:NIST11.lib
SI:66 Formula:C12H20O2 CAS:29843-60-5 MolWeight:196 RetIndex:1292
CompName:Isomyrcenyl acetate $$ 2-(2-Methyl-1-propenyl)-5-hexenyl acetate # $$

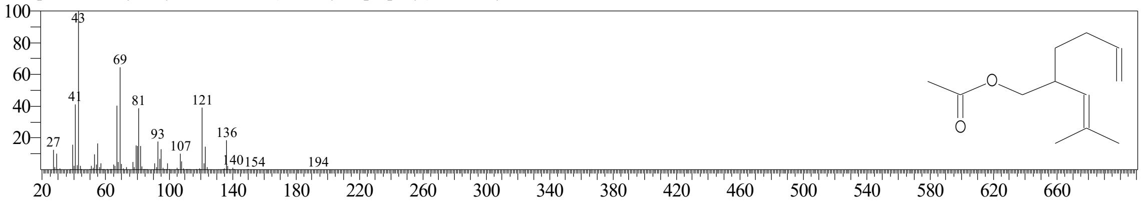

Hit#:2 Entry:17427 Library:NIST11.lib
SI:63 Formula:C10H18O CAS:0-00-0 MolWeight:154 RetIndex:1061
CompName:cis-5-Methyl-2-isopropyl-2-hexen-1-al $$ (2E)-2-Isopropyl-5-methyl-2-hexenal # $$

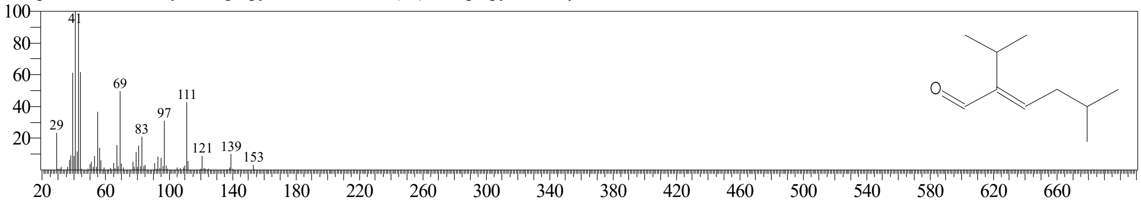

Hit#:3 Entry:62303 Library:NIST11.lib
SI:63 Formula:C14H26O2 CAS:73003-77-7 MolWeight:226 RetIndex:1471
CompName:2,6-Octadiene, 1-(1-ethoxyethoxy)-3,7-dimethyl- $$ (2E)-1-(1-Ethoxyethoxy)-3,7-dimethyl-2,6-octadiene # $$

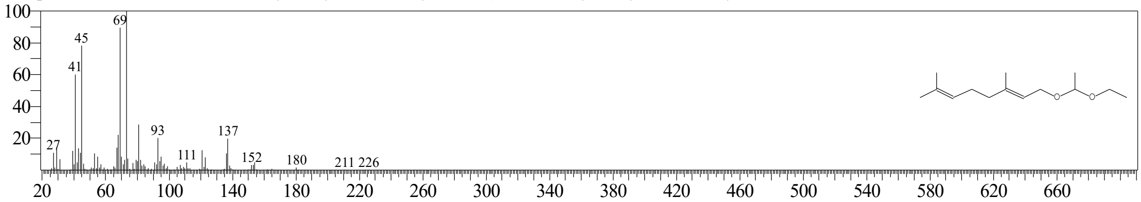

Hit#:4 Entry:17455 Library:NIST11.lib
SI:63 Formula:C10H18O CAS:69668-93-5 MolWeight:154 RetIndex:1195
CompName:3-Decyn-2-ol

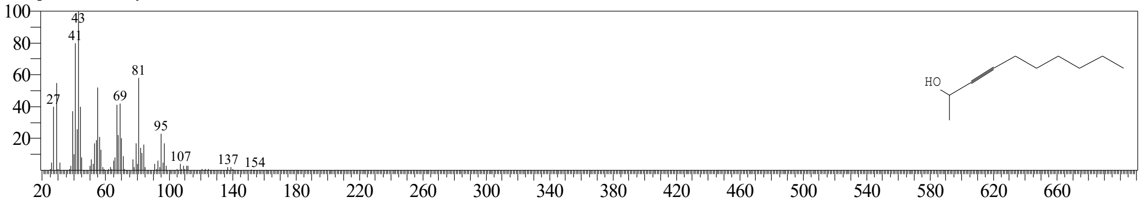

Hit#:5 Entry:89014 Library:NIST11.lib
SI:63 Formula:C18H30O CAS:1117-52-8 MolWeight:262 RetIndex:1902
CompName:5,9,13-Pentadecatrien-2-one, 6,10,14-trimethyl-, (E,E)- $$ Farnesyl acetone $$ (5E,9E)-6,10,14-Trimethyl-5,9,13-pentadecatrien-2-one # $$ 5,9,13-

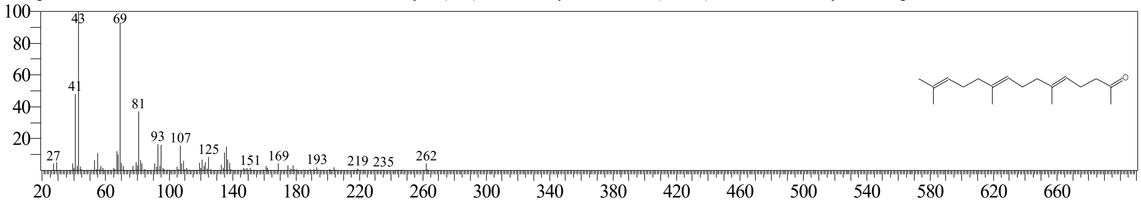

<< Target >>
Line#:100 R.Time:12.675(Scan#:1736) MassPeaks:360
RawMode:Averaged 12.670-12.680(1735-1737) BasePeak:43.00(11520)
BG Mode:Calc. from Peak Group 1 - Event 1 Scan

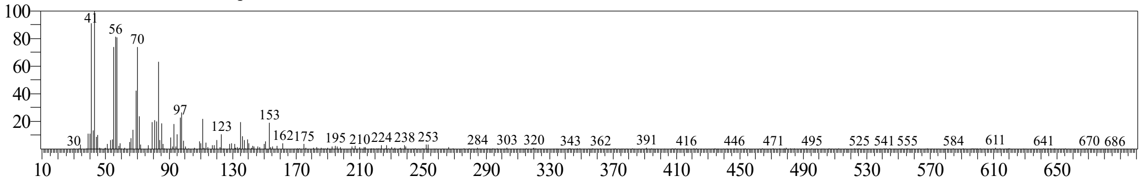

Hit#:1 Entry:32998 Library:NIST11.lib
SI:84 Formula:C13H26 CAS:2437-56-1 MolWeight:182 RetIndex:1304
CompName:1-Tridecene $$ n-Tridec-1-ene $$ 1-C13H26 $$ Tridecene-1 $$ .alpha.-Tridecene $$ Tridec-1-ene $$

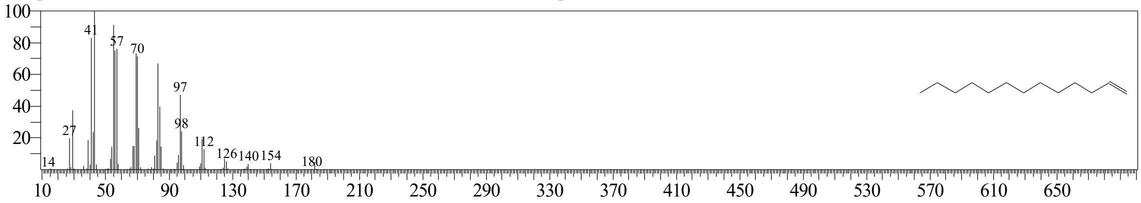

Hit#:2 Entry:41640 Library:NIST11.lib
SI:83 Formula:C14H28 CAS:1120-36-1 MolWeight:196 RetIndex:1403
CompName:1-Tetradecene $$ n-Tetradec-1-ene $$ .alpha.-Tetradecene $$ Neodene 14 $$ Tetradec-1-ene $$ Tetradecene-1 $$

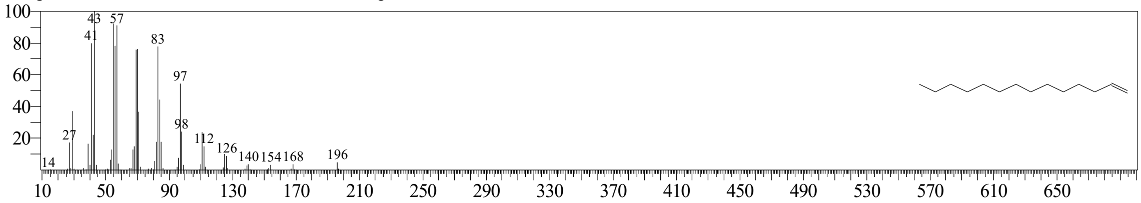

Hit#:3 Entry:91989 Library:NIST11.lib
SI:83 Formula:C19H38 CAS:35354-39-3 MolWeight:266 RetIndex:1854
CompName:cis-2-Methyl-7-octadecene $$ Disparlure olefin $$ (7Z)-2-Methyl-7-octadecene $$ 2-Methyl-7-octadecene, Z $$ 2-Methyl-(Z)-7-octadecene $$ 7-O

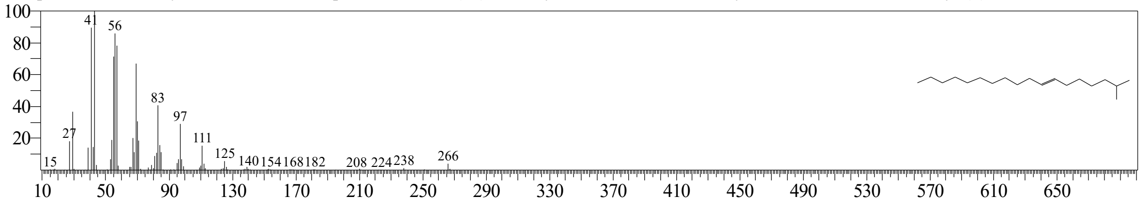

Hit#:4 Entry:41581 Library:NIST11.lib
SI:83 Formula:C13H24O CAS:7069-41-2 MolWeight:196 RetIndex:1510
CompName:2-Tridecenal, (E)- $$ (2E)-2-Tridecenal $$ (E)-2-Tridecenal $$ (E)-Tridec-2-enal $$

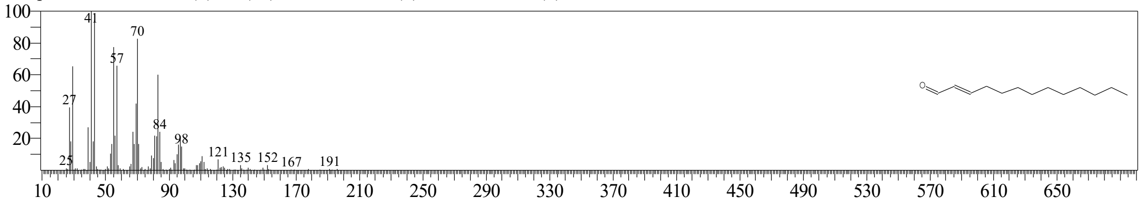

Hit#:5 Entry:102909 Library:NIST11.lib
SI:83 Formula:C20H40 CAS:219750-68-2 MolWeight:280 RetIndex:1953
CompName:2-Methyl-7-nonadecene $$ (7E)-2-Methyl-7-nonadecene # $$

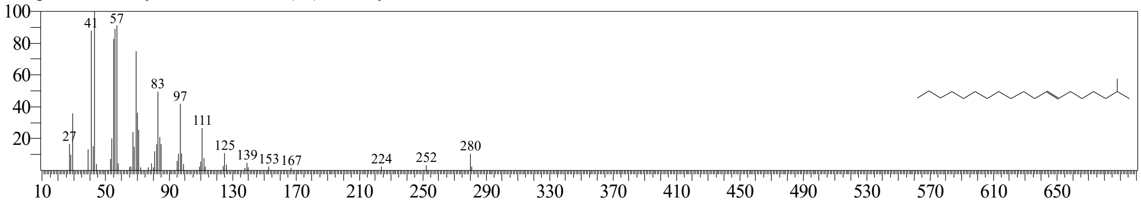

Qualitative Analysis Report

<< Target >>
Line#:101 R.Time:12.740(Scan#:1749) MassPeaks:380
RawMode:Averaged 12.735-12.745(1748-1750) BasePeak:41.10(22036)
BG Mode:Calc. from Peak Group 1 - Event 1 Scan

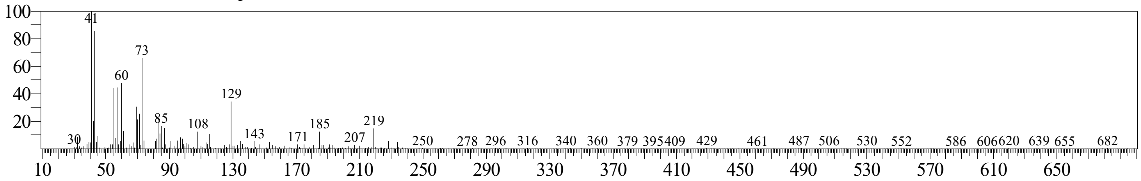

Hit#:1 Entry:53599 Library:NIST11.lib
SI:83 Formula:C13H26O2 CAS:638-53-9 MolWeight:214 RetIndex:1670
CompName:Tridecanoic acid $$ n-Tridecanoic acid $$ n-Tridecoic acid $$ Tridecylic acid $$

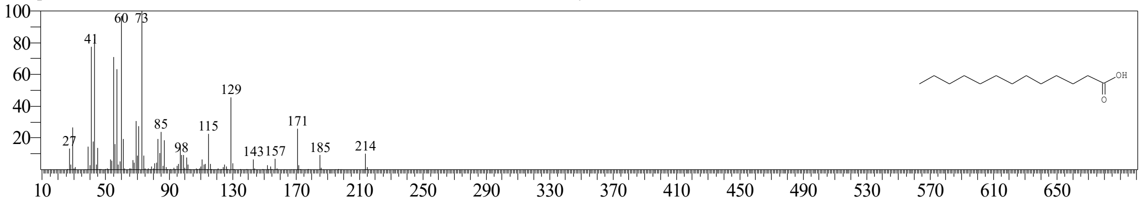

Hit#:2 Entry:84362 Library:NIST11.lib
SI:83 Formula:C16H32O2 CAS:57-10-3 MolWeight:256 RetIndex:1968
CompName:n-Hexadecanoic acid $$ Hexadecanoic acid $$ n-Hexadecoic acid $$ Palmitic acid $$ Pentadecanecarboxylic acid $$ 1-Pentadecanecarboxylic acid

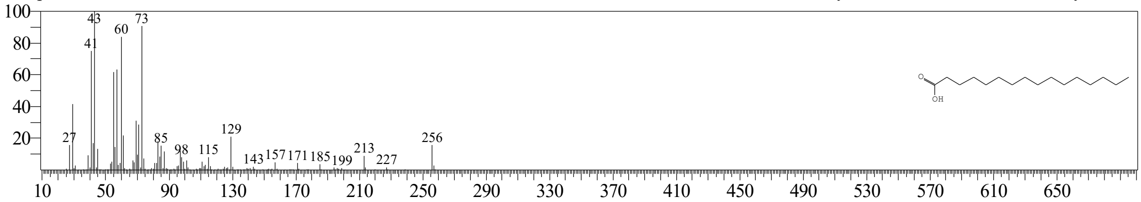

Hit#:3 Entry:63810 Library:NIST11.lib
SI:82 Formula:C14H28O2 CAS:544-63-8 MolWeight:228 RetIndex:1769
CompName:Tetradecanoic acid $$ Myristic acid $$ n-Tetradecanoic acid $$ n-Tetradecoic acid $$ Neo-Fat 14 $$ Univol U 316S $$ 1-Tridecanecarboxylic acid $

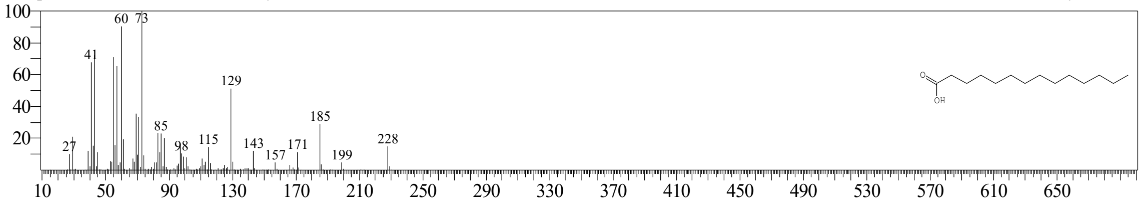

Hit#:4 Entry:117133 Library:NIST11.lib
SI:82 Formula:C19H38O2 CAS:646-30-0 MolWeight:298 RetIndex:2266
CompName:Nonadecanoic acid $$ n-Nonadecanoic acid $$

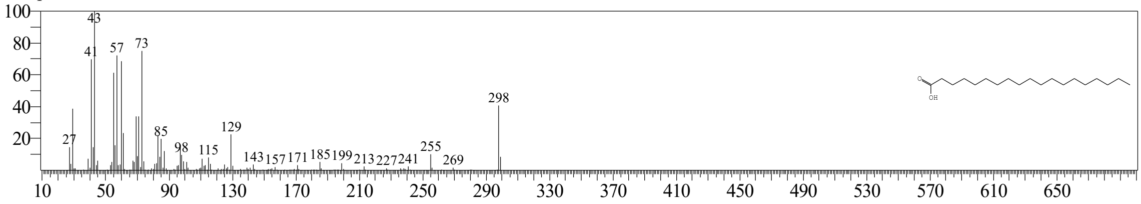

Hit#:5 Entry:128151 Library:NIST11.lib
SI:82 Formula:C20H40O2 CAS:506-30-9 MolWeight:312 RetIndex:2366
CompName:Eicosanoic acid $$ Arachic acid $$ Arachidic acid $$ Icosanoic acid $$ n-Eicosanoic acid $$ Arachidic acid (synthetic) $$

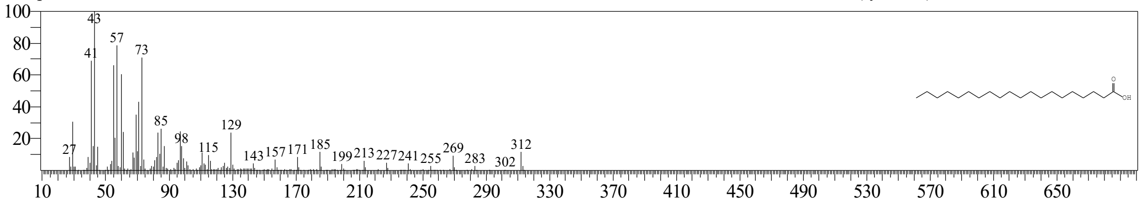

Qualitative Analysis Report

<< Target >>
Line#:102 R.Time:12.835(Scan#:1768) MassPeaks:356
RawMode:Averaged 12.830-12.840(1767-1769) BasePeak:41.10(541712)
BG Mode:Calc. from Peak Group 1 - Event 1 Scan

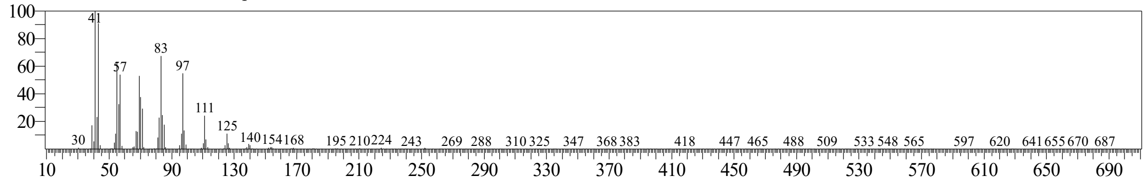

Hit#:1 Entry:81317 Library:NIST11.lib
SI:95 Formula:C17H32O CAS:0-00-0 MolWeight:252 RetIndex:1907
CompName:E-15-Heptadecenal $$ (15E)-15-Heptadecenal # $$

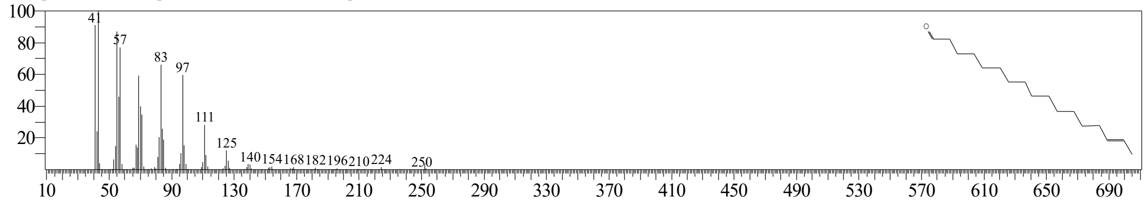

Hit#:2 Entry:70935 Library:NIST11.lib
SI:95 Formula:C17H34 CAS:6765-39-5 MolWeight:238 RetIndex:1701
CompName:1-Heptadecene $$ Hexahydroaplotaxene $$ Heptadec-1-ene $$ NSC 77132 $$ Heptadecene (Related) $$

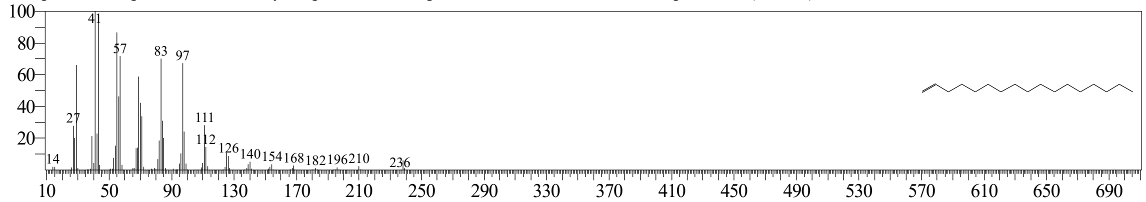

Hit#:3 Entry:72459 Library:NIST11.lib
SI:94 Formula:C16H32O CAS:64437-47-4 MolWeight:240 RetIndex:1862
CompName:Hexadecen-1-ol, trans-9- $$ (9E)-9-Hexadecen-1-ol $$ 9-Hexadecenol, E $$ (E)9-Hexadecen-1-ol $$

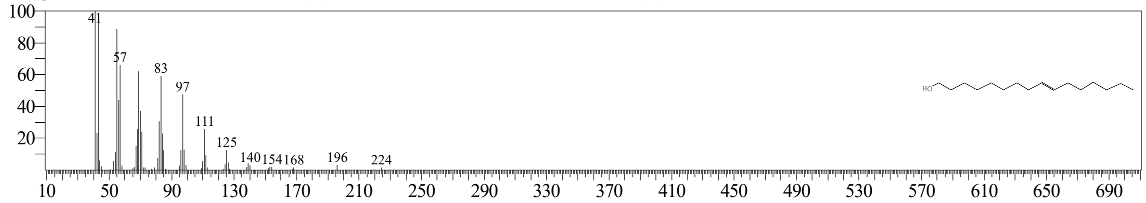

Hit#:4 Entry:70890 Library:NIST11.lib
SI:94 Formula:C16H30O CAS:330207-53-9 MolWeight:238 RetIndex:1808
CompName:E-14-Hexadecenal $$ (14E)-14-Hexadecenal $$ 14-Hexadecenal, (E)- $$

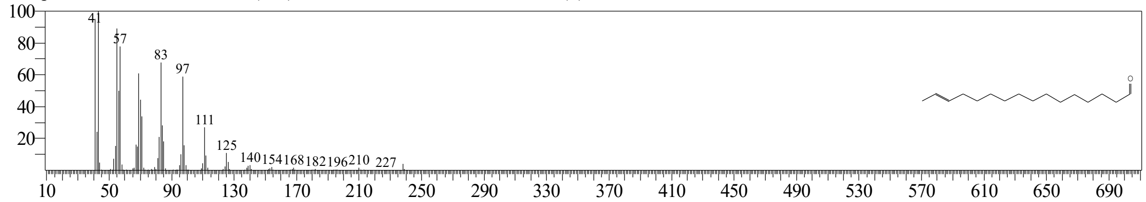

Hit#:5 Entry:51049 Library:NIST11.lib
SI:94 Formula:C15H30 CAS:13360-61-7 MolWeight:210 RetIndex:1502
CompName:1-Pentadecene $$ Pentadecene,1- $$ Pentadec-1-ene $$

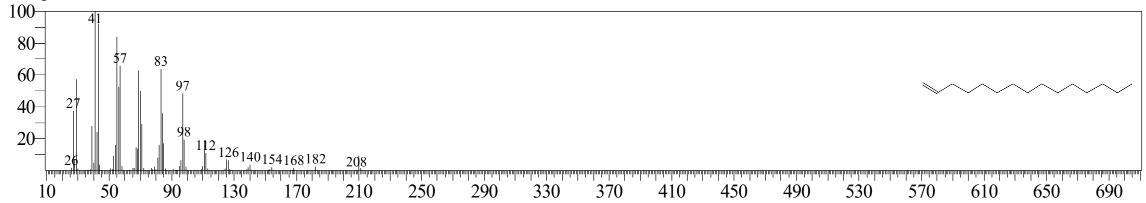

<< Target >>
Line#:103 R.Time:12.920(Scan#:1785) MassPeaks:387
RawMode:Averaged 12.915-12.925(1784-1786) BasePeak:41.05(20778)
BG Mode:Calc. from Peak Group 1 - Event 1 Scan

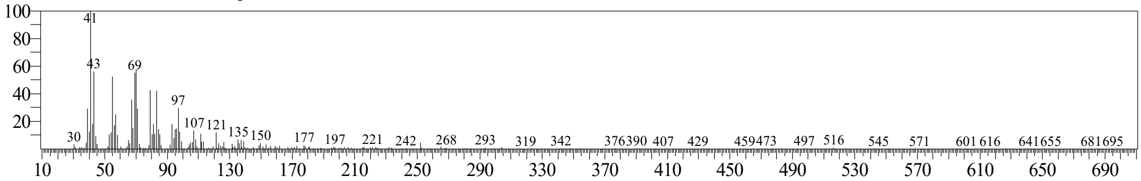

Hit#:1 Entry:32915 Library:NIST11.lib
SI:86 Formula:C12H22O CAS:20407-84-5 MolWeight:182 RetIndex:1410
CompName:2-Dodecenal, (E)- $$ (E)-2-Dodecen-1-al $$ (2E)-2-Dodecenal $$ 2(E)-Dodecenal $$ (E)-2-Dodecenal $$ (E)-Dodec-2-enal $$ trans-2-Dodecenal $

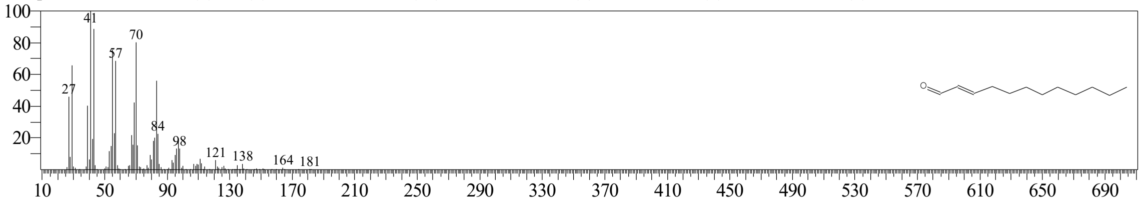

Hit#:2 Entry:41581 Library:NIST11.lib
SI:86 Formula:C13H24O CAS:7069-41-2 MolWeight:196 RetIndex:1510
CompName:2-Tridecenal, (E)- $$ (2E)-2-Tridecenal $$ (E)-2-Tridecenal $$ (E)-Tridec-2-enal $$

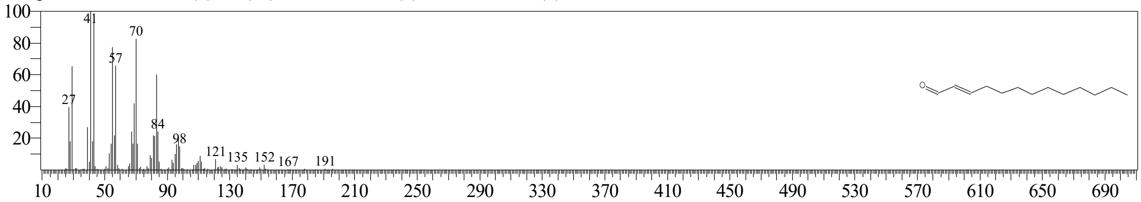

Hit#:3 Entry:60905 Library:NIST11.lib
SI:85 Formula:C15H28O CAS:2834-00-6 MolWeight:224 RetIndex:1772
CompName:2-Pentadecyn-1-ol

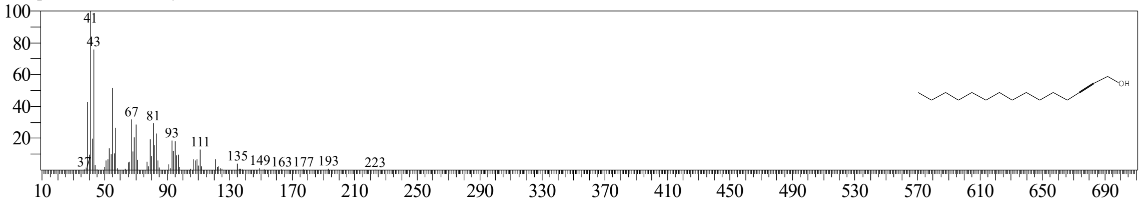

Hit#:4 Entry:91942 Library:NIST11.lib
SI:85 Formula:C18H34O CAS:5090-41-5 MolWeight:266 RetIndex:2007
CompName:9-Octadecenal $$ Octadecenyl aldehyde $$ (9E)-9-Octadecenal # $$

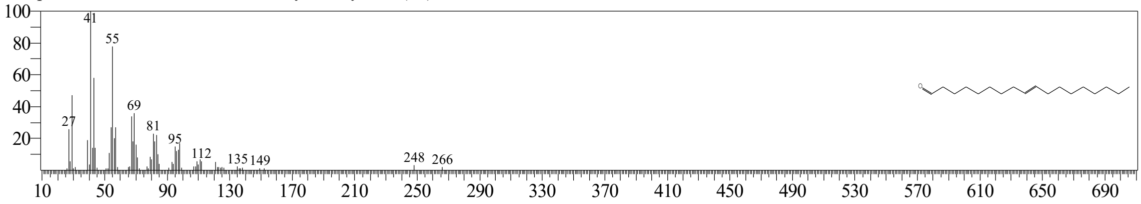

Hit#:5 Entry:32954 Library:NIST11.lib
SI:85 Formula:C12H22O CAS:4826-62-4 MolWeight:182 RetIndex:1410
CompName:2-Dodecenal $$ 2-Dodecen-1-al $$ .beta.-Octyl acrolein $$

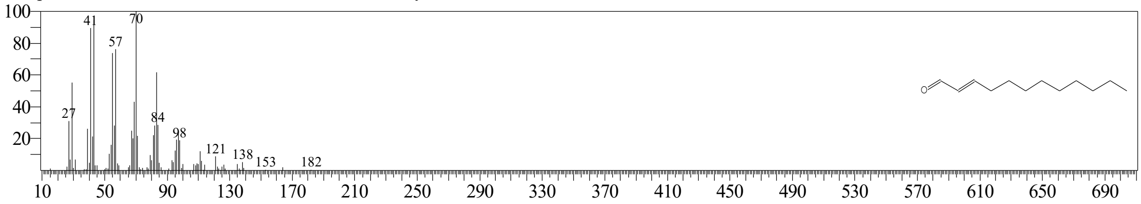

<< Target >>
Line#:104 R.Time:13.005(Scan#:1802) MassPeaks:372
RawMode:Averaged 13.000-13.010(1801-1803) BasePeak:32.10(7475)
BG Mode:Calc. from Peak Group 1 - Event 1 Scan

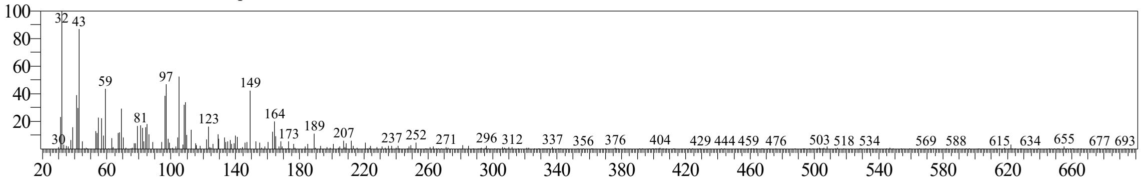

Hit#:1 Entry:75192 Library:NIST11.lib
SI:61 Formula:C14H28O3 CAS:0-00-0 MolWeight:244 RetIndex:1656
CompName:Carbonic acid, decyl propyl ester

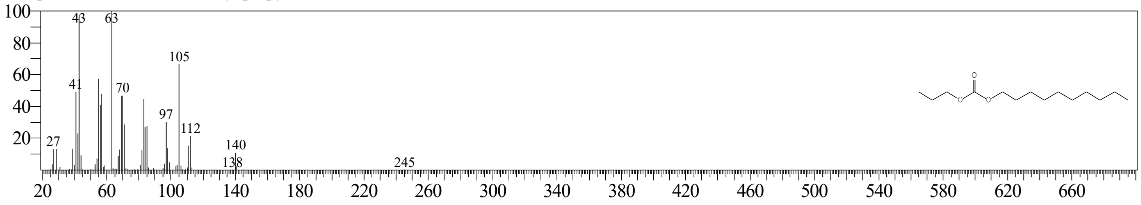

Hit#:2 Entry:75326 Library:NIST11.lib
SI:60 Formula:C15H32O2 CAS:10395-09-2 MolWeight:244 RetIndex:1934
CompName:1,3-Propanediol, 2-dodecyl $$ 2-Dodecyl-1,3-propanediol # $$

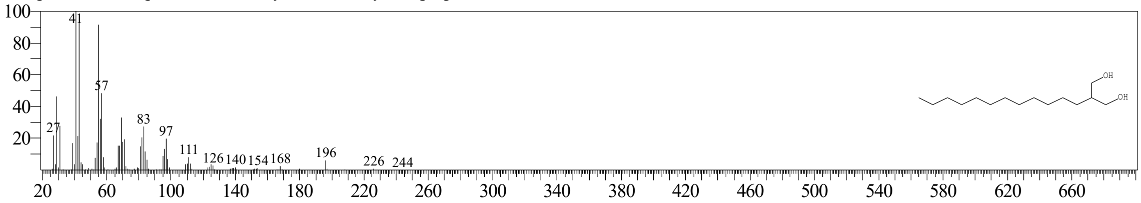

Hit#:3 Entry:150506 Library:NIST11.lib
SI:60 Formula:C21H42O3 CAS:0-00-0 MolWeight:342 RetIndex:2352
CompName:Carbonic acid, heptadecyl propyl ester

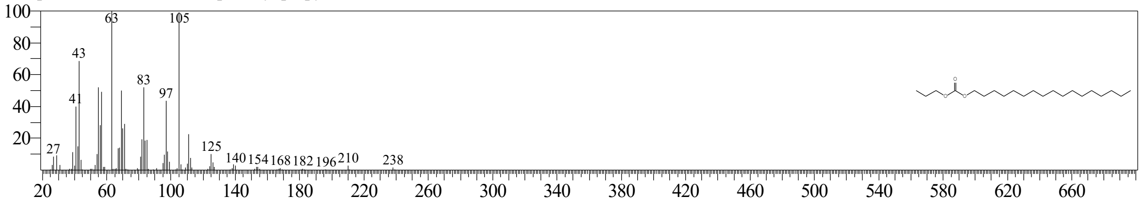

Hit#:4 Entry:34961 Library:NIST11.lib
SI:60 Formula:C10H18OS CAS:33281-91-3 MolWeight:186 RetIndex:1431
CompName:Cyclohexanone, 2-(1-mercapto-1-methylethyl)-5-methyl-, trans- $$ p-Menthon-8-thiol, trans $$ 5-Methyl-2-(1-methyl-1-sulfanylethyl)cyclohexano

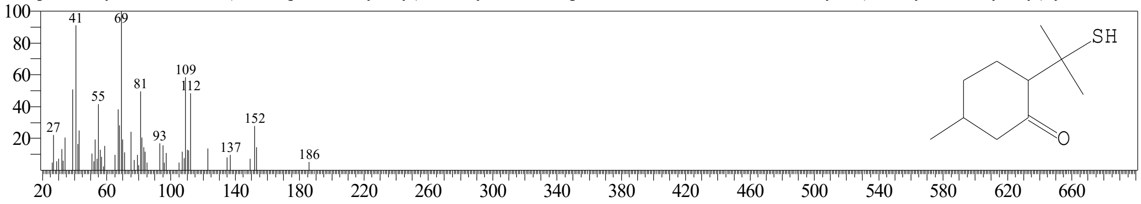

Hit#:5 Entry:28087 Library:NIST11.lib
SI:60 Formula:C10H22O2 CAS:71052-96-5 MolWeight:174 RetIndex:1270
CompName:1-Methoxy-3-hydroxymethyloctane $$ 2-(2-Methoxyethyl)-1-heptanol # $$

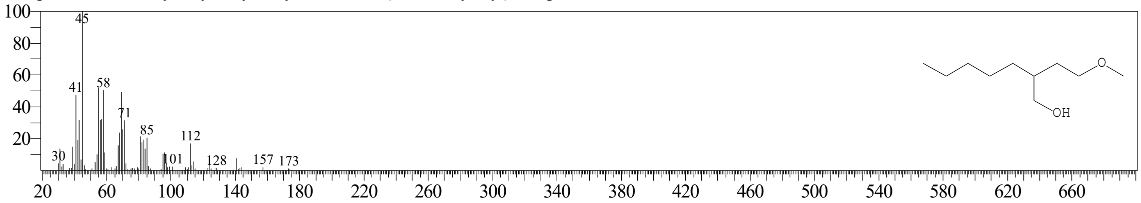

Qualitative Analysis Report

<< Target >>
Line#:105 R.Time:13.060(Scan#:1813) MassPeaks:383
RawMode:Averaged 13.055-13.065(1812-1814) BasePeak:41.05(37123)
BG Mode:Calc. from Peak Group 1 - Event 1 Scan

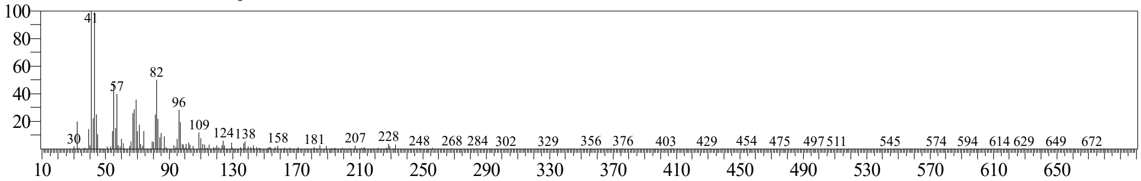

Hit#:1 Entry:93586 Library:NIST11.lib
SI:86 Formula:C18H36O CAS:638-66-4 MolWeight:268 RetIndex:1999
CompName:Octadecanal $$ Stearaldehyde $$ Octadecyl aldehyde $$ Stearyl aldehyde $$ n-Octadecanal $$

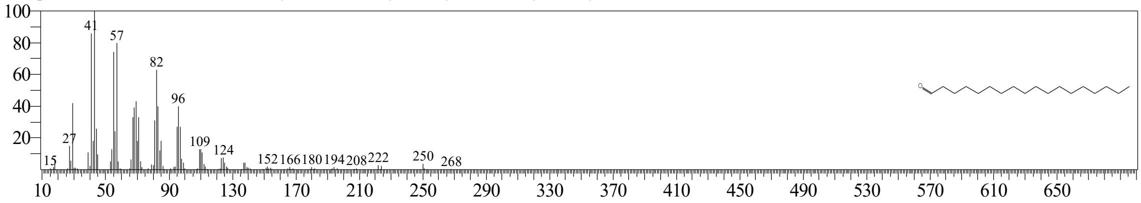

Hit#:2 Entry:26030 Library:NIST11.lib
SI:86 Formula:C11H22O CAS:112-44-7 MolWeight:170 RetIndex:1303
CompName:Undecanal $$ n-Undecanal $$ n-Undecyl aldehyde $$ Hendecanal $$ Hendecanaldehyde $$ Undecyl aldehyde $$ Undecylic aldehyde $$ 1-Undecan

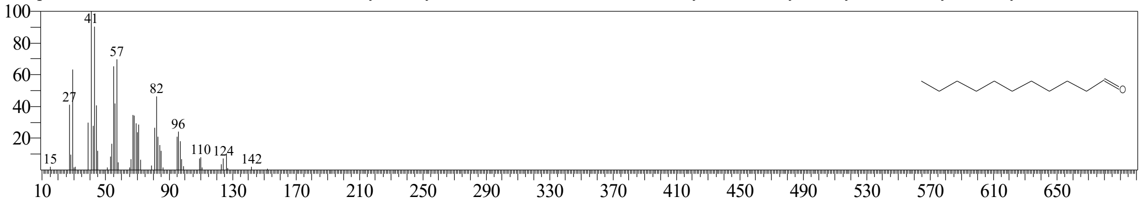

Hit#:3 Entry:42882 Library:NIST11.lib
SI:85 Formula:C13H26O CAS:74962-98-4 MolWeight:198 RetIndex:1564
CompName:2-Tridecen-1-ol, (E)- $$ trans-2-Tridecen-1-ol $$ (2E)-2-Tridecen-1-ol $$ E-2-Tridecen-1-ol $$

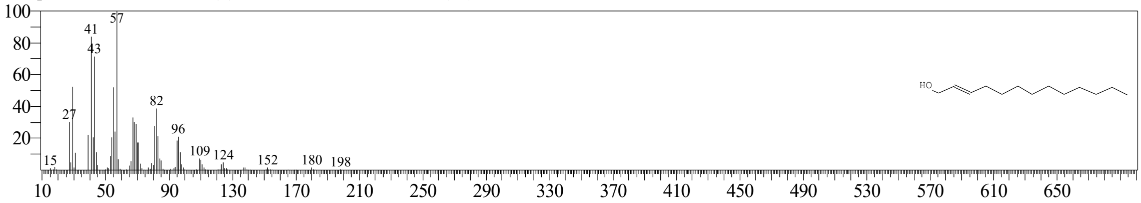

Hit#:4 Entry:52426 Library:NIST11.lib
SI:85 Formula:C14H28O CAS:0-00-0 MolWeight:212 RetIndex:1664
CompName:E-2-Tetradecen-1-ol $$ (2E)-2-Tetradecen-1-ol # $$

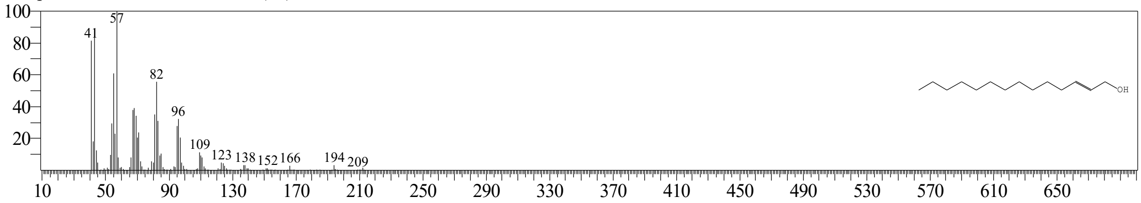

Hit#:5 Entry:52414 Library:NIST11.lib
SI:85 Formula:C14H28O CAS:3234-28-4 MolWeight:212 RetIndex:1503
CompName:Oxirane, dodecyl- $$ 1,2-Epoxytetradecane $$ 2-Dodecyloxirane # $$

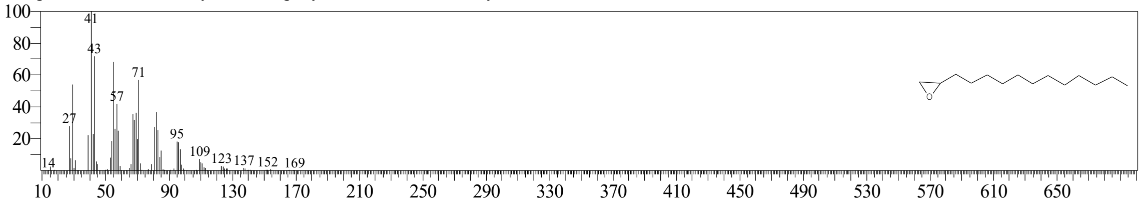

<< Target >>
Line#:106 R.Time:13.145(Scan#:1830) MassPeaks:348
RawMode:Averaged 13.140-13.150(1829-1831) BasePeak:32.10(12643)
BG Mode:Calc. from Peak Group 1 - Event 1 Scan

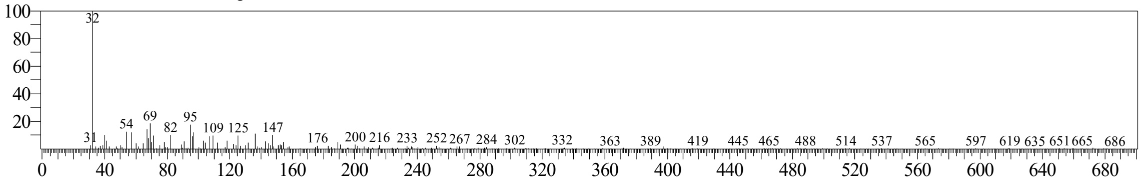

Hit#:1 Entry:128143 Library:NIST11.lib
SI:56 Formula:C20H40O2 CAS:15677-71-1 MolWeight:312 RetIndex:2105
CompName:9-Octadecene, 1,1-dimethoxy-, (Z)- $$ Olealdehyde, dimethyl acetal $$ (9Z)-1,1-Dimethoxy-9-octadecene # $$

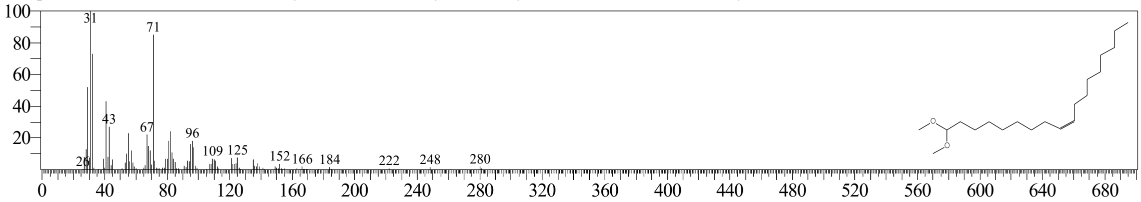

Hit#:2 Entry:95054 Library:NIST11.lib
SI:56 Formula:C16H34N2O CAS:2619-88-7 MolWeight:270 RetIndex:2255
CompName:16-Hexadecanoyl hydrazide $$ Hexadecanohydrazide # $$

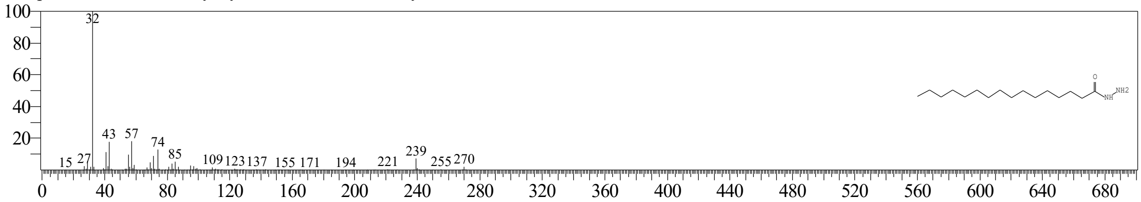

Hit#:3 Entry:117059 Library:NIST11.lib
SI:53 Formula:C18H38N2O CAS:4130-54-5 MolWeight:298 RetIndex:2454
CompName:Stearic acid hydrazide $$ Stearic hydrazide $$ Octadecanoic acid, hydrazide $$ Stearohydrazide $$ Stearoylhydrazine $$ Octadecanohydrazide # $$

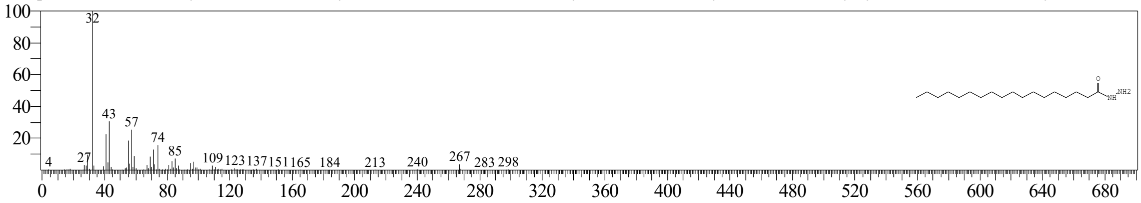

Hit#:4 Entry:23747 Library:NIST11.lib
SI:52 Formula:C5H5N5O2 CAS:0-00-0 MolWeight:167 RetIndex:0
CompName:4-Methyl-3,4-dihydro-[1,2,3]triazolo[4,5-d]pyrimidine-5,7-dione

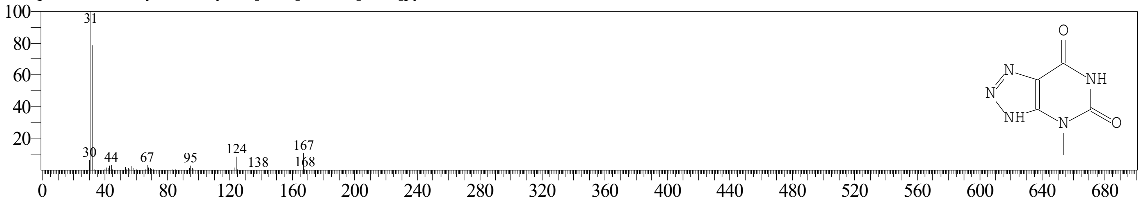

Hit#:5 Entry:1772 Library:NIST11.lib
SI:51 Formula:C3H5N3O CAS:140-87-4 MolWeight:99 RetIndex:1208
CompName:Cyacetacide $$ Cyanoacetohydrazide $$ 2-Cyanoacetylhydrazide $$ Cyanoacethydrazide $$ Cyanoacetic acid hydrazide $$ Acetic acid, cyano-, hyd

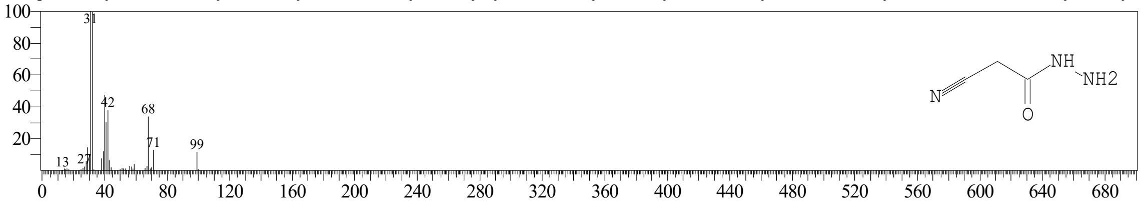

Qualitative Analysis Report

<< Target >>
Line#:107 R.Time:13.205(Scan#:1842) MassPeaks:352
RawMode:Averaged 13.200-13.210(1841-1843) BasePeak:43.10(38653)
BG Mode:Calc. from Peak Group 1 - Event 1 Scan

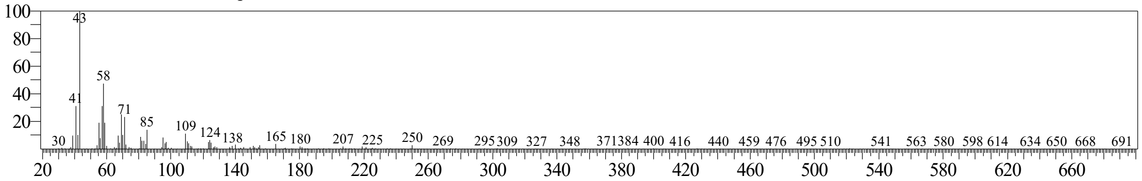

Hit#:1 Entry:42891 Library:NIST11.lib
SI:90 Formula:C13H26O CAS:1604-34-8 MolWeight:198 RetIndex:1321
CompName:2-Undecanone, 6,10-dimethyl- $$ Hexahydropseudoionone $$ Pseudoionone, hexahydro- $$ Tetrahydrogeranylacetone $$ 6,10-Dimethyl-2-undecar

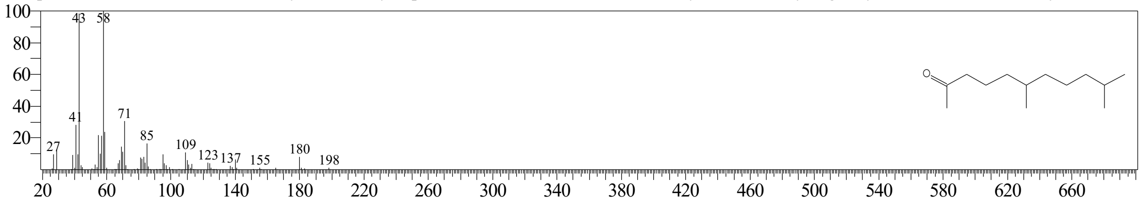

Hit#:2 Entry:93588 Library:NIST11.lib
SI:85 Formula:C18H36O CAS:502-69-2 MolWeight:268 RetIndex:1754
CompName:2-Pentadecanone, 6,10,14-trimethyl- $$ Hexahydrofarnesyl acetone $$ 6,10,14-Trimethyl-2-pentadecanone $$ 6,10,14-Trimethylpentadecan-2-one $

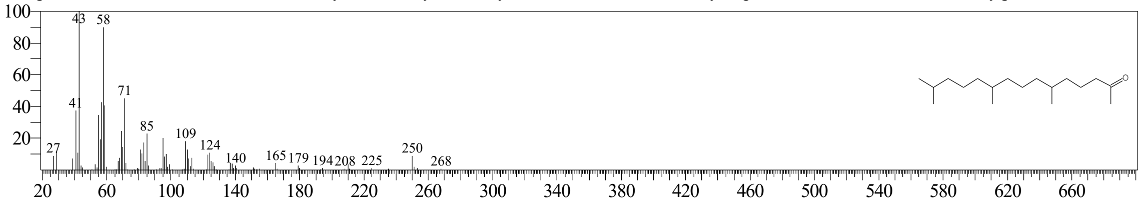

Hit#:3 Entry:52432 Library:NIST11.lib
SI:84 Formula:C14H28O CAS:73105-73-4 MolWeight:212 RetIndex:1485
CompName:6-Methyl-2-tridecanone

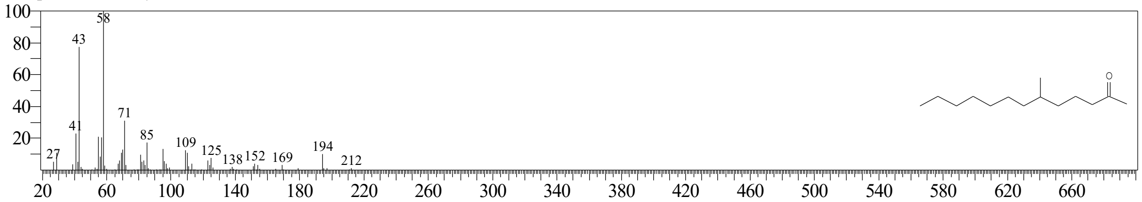

Hit#:4 Entry:104502 Library:NIST11.lib
SI:83 Formula:C19H38O CAS:629-66-3 MolWeight:282 RetIndex:2046
CompName:2-Nonadecanone $$ Methyl heptadecyl ketone $$

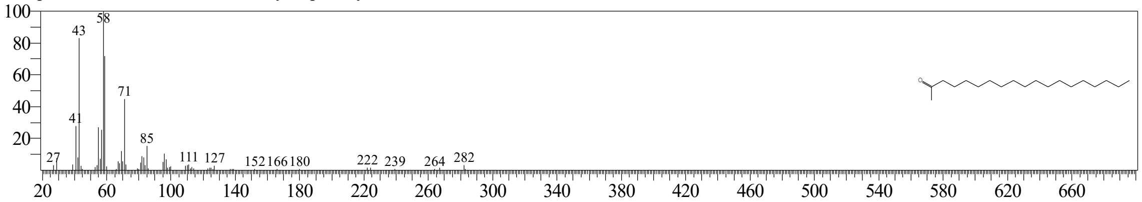

Hit#:5 Entry:34071 Library:NIST11.lib
SI:83 Formula:C12H24O CAS:33933-82-3 MolWeight:184 RetIndex:1222
CompName:2-Decanone, 5,9-dimethyl- $$ 5,9-Dimethyl-2-decanone $$

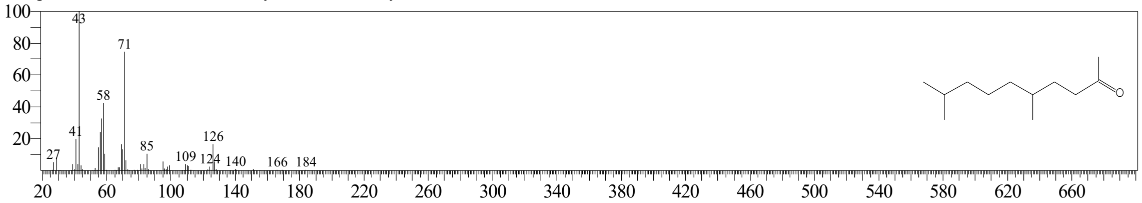

Qualitative Analysis Report

<< Target >>
Line#:108 R.Time:13.300(Scan#:1861) MassPeaks:388
RawMode:Averaged 13.295-13.305(1860-1862) BasePeak:149.15(11331)
BG Mode:Calc. from Peak Group 1 - Event 1 Scan

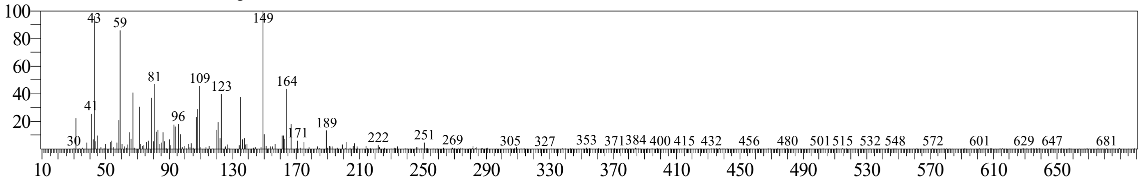

Hit#:1 Entry:59379 Library:NIST11.lib
SI:73 Formula:C15H26O CAS:63891-61-2 MolWeight:222 RetIndex:1598
CompName:2-Naphthalenemethanol, 2,3,4,4a,5,6,7,8-octahydro-.alpha.,.alpha.,4a,8-tetramethyl-, [2R-(2.alpha.,4a.beta.,8.beta.)]- $$ 2-(4a,8-Dimethyl-2,3,4,4a,

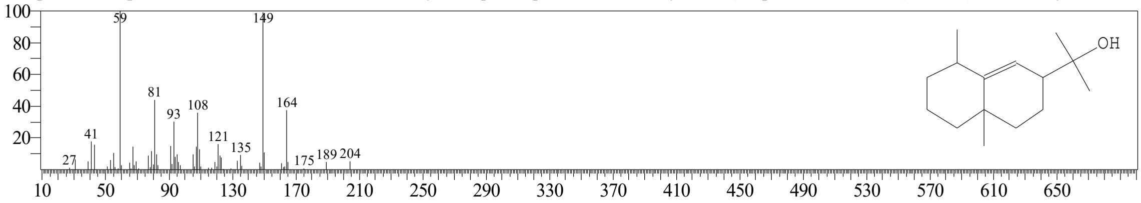

Hit#:2 Entry:59380 Library:NIST11.lib
SI:69 Formula:C15H26O CAS:473-15-4 MolWeight:222 RetIndex:1593
CompName:2-Naphthalenemethanol, decahydro-.alpha.,.alpha.,4a-trimethyl-8-methylene-, [2R-(2.alpha.,4a.alpha.,8a.beta.)]- $$ Eudesm-4(14)-en-11-ol $$ .beta

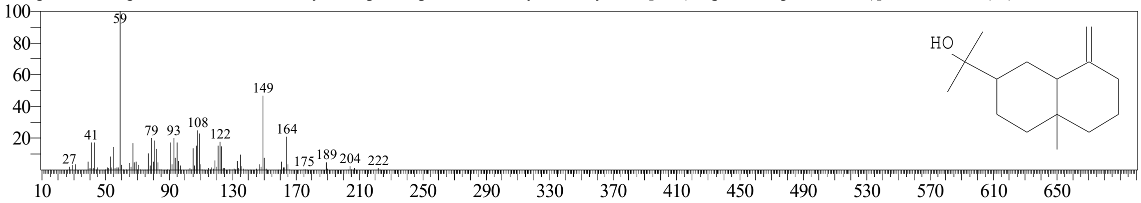

Hit#:3 Entry:59377 Library:NIST11.lib
SI:67 Formula:C15H26O CAS:21657-90-9 MolWeight:222 RetIndex:1694
CompName:3,7-Cyclodecadiene-1-methanol, .alpha.,.alpha.,4,8-tetramethyl-, [s-(Z,Z)] $$ Hedycaryol $$ 2-(4,8-Dimethyl-3,7-cyclodecadien-1-yl)-2-propanol #

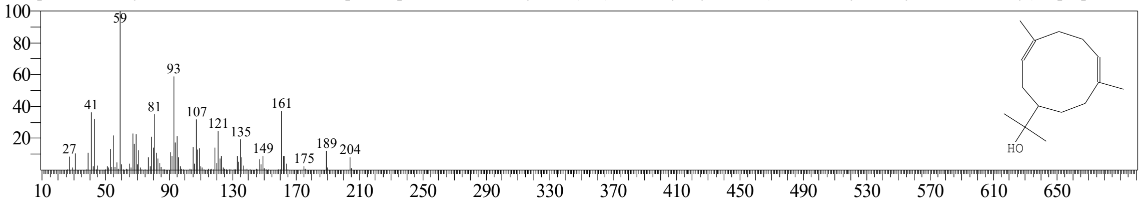

Hit#:4 Entry:59383 Library:NIST11.lib
SI:66 Formula:C15H26O CAS:62192-82-9 MolWeight:222 RetIndex:1614
CompName:1-Naphthalenemethanol, 1,2,3,5,6,7,8,8a-octahydro-.alpha.,.alpha.,4,7-tetramethyl- $$ 2-(4,7-Dimethyl-1,2,3,5,6,7,8,8a-octahydro-1-naphthalenyl)-

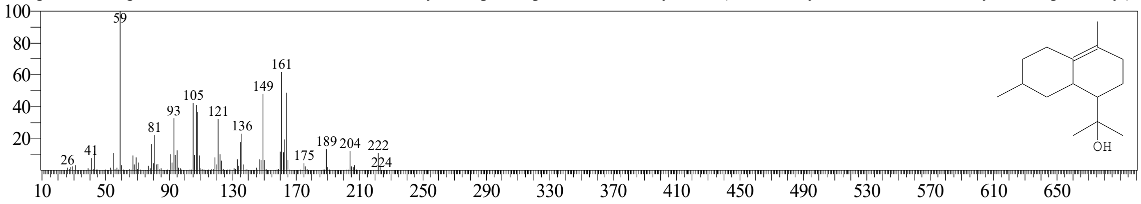

Hit#:5 Entry:59376 Library:NIST11.lib
SI:66 Formula:C15H26O CAS:639-99-6 MolWeight:222 RetIndex:1522
CompName:Cyclohexanemethanol, 4-ethenyl-.alpha.,.alpha.,4-trimethyl-3-(1-methylethenyl)-, [1R-(1.alpha.,3.alpha.,4.beta.)]- $$ o-Menth-8-ene-4-methanol, .a

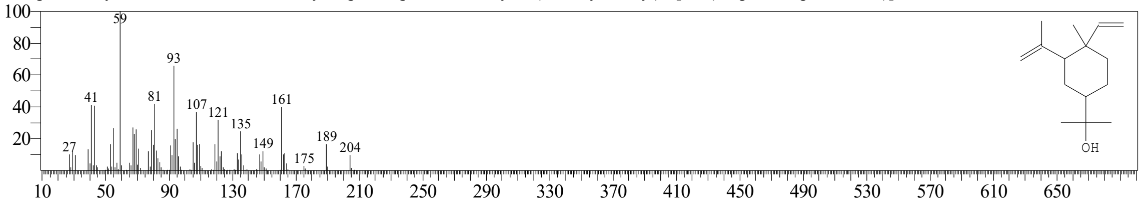

<< Target >>
Line#:109 R.Time:13.340(Scan#:1869) MassPeaks:401
RawMode:Averaged 13.335-13.345(1868-1870) BasePeak:159.20(6025)
BG Mode:Calc. from Peak Group 1 - Event 1 Scan

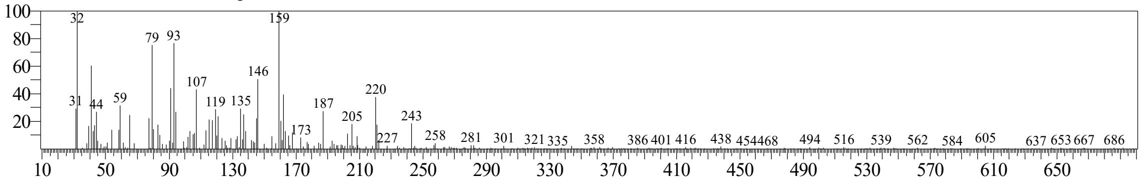

Hit#:1 Entry:57815 Library:NIST11.lib
SI:61 Formula:C15H24O CAS:0-00-0 MolWeight:220 RetIndex:1690
CompName:6-Isopropenyl-4,8a-dimethyl-1,2,3,5,6,7,8,8a-octahydro-naphthalen-2-ol $$ 6-Isopropenyl-4,8a-dimethyl-1,2,3,5,6,7,8,8a-octahydro-2-naphthalenol

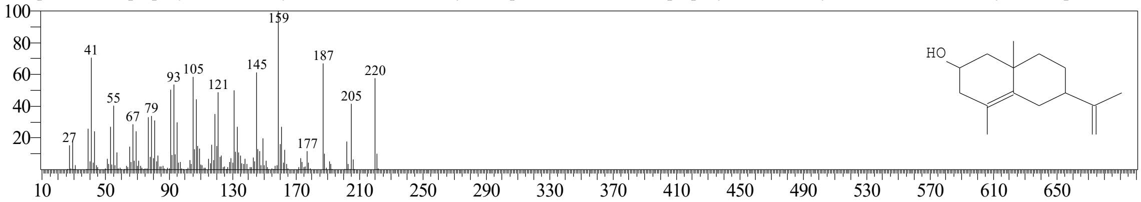

Hit#:2 Entry:57728 Library:NIST11.lib
SI:60 Formula:C15H24O CAS:0-00-0 MolWeight:220 RetIndex:1599
CompName:Tricyclo[5.2.2.0(1,6)]undecan-3-ol, 2-methylene-6,8,8-trimethyl-

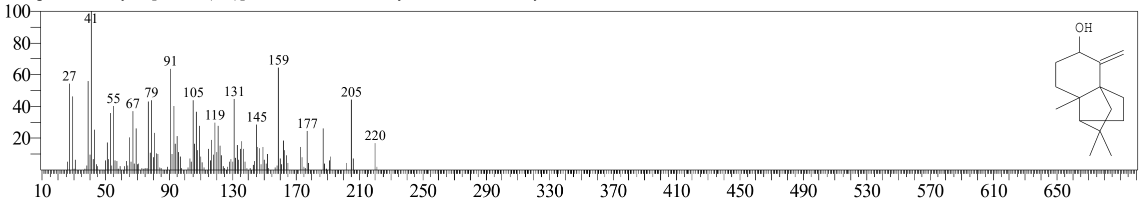

Hit#:3 Entry:57738 Library:NIST11.lib
SI:60 Formula:C15H24O CAS:6750-60-3 MolWeight:220 RetIndex:1536
CompName:1H-Cycloprop[e]azulen-7-ol, decahydro-1,1,7-trimethyl-4-methylene-, [1ar-(1a.alpha.,4a.alpha.,7.beta.,7a.beta.,7b.alpha.)]- $$ Spathulenol $$ 1,1,7

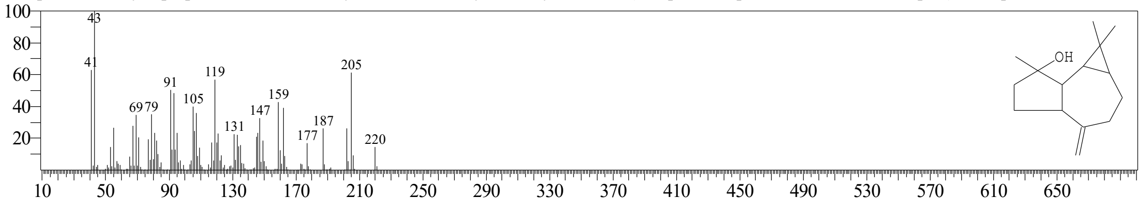

Hit#:4 Entry:57734 Library:NIST11.lib
SI:58 Formula:C15H24O CAS:77171-55-2 MolWeight:220 RetIndex:1536
CompName:(-)-Spathulenol $$ 1H-Cycloprop[e]azulen-7-ol, decahydro-1,1,7-trimethyl-4-methylene-, [1aS-(1a.alpha.,4a.alpha.,7.beta.)] $$ Ent-Spathulenol $$

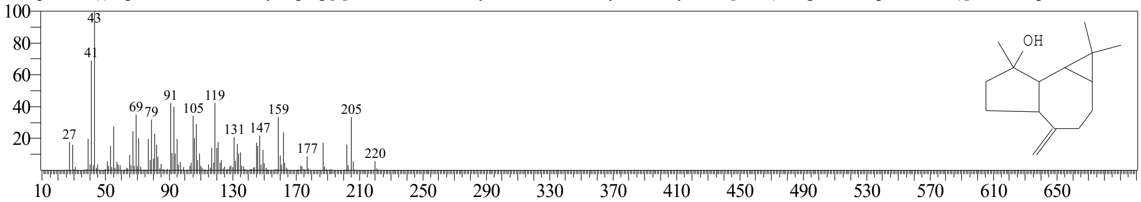

Hit#:5 Entry:57744 Library:NIST11.lib
SI:57 Formula:C15H24O CAS:41370-56-3 MolWeight:220 RetIndex:1377
CompName:Tricyclo[4.4.0.0(2,7)]dec-8-ene-3-methanol, .alpha.,.alpha.,6,8-tetramethyl-, stereoisomer $$ .alpha.-Copaen-11-ol $$ Tricyclo[4.4.0.0(2,7)]dec-8-er

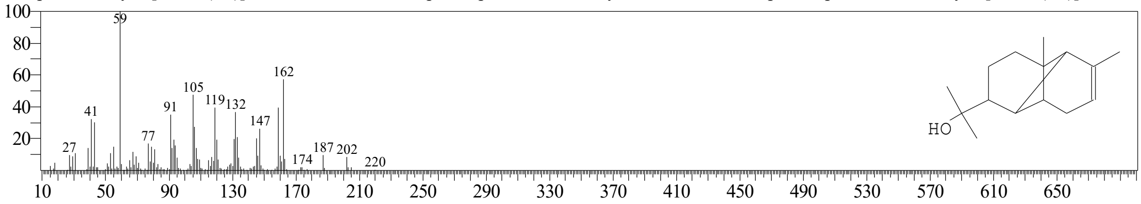

<< Target >>
Line#:110 R.Time:13.415(Scan#:1884) MassPeaks:405
RawMode:Averaged 13.410-13.420(1883-1885) BasePeak:149.00(29826)
BG Mode:Calc. from Peak Group 1 - Event 1 Scan

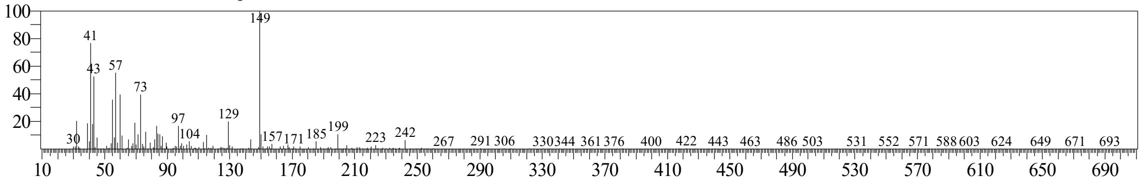

Hit#:1 Entry:73851 Library:NIST11.lib
SI:73 Formula:C15H30O2 CAS:1002-84-2 MolWeight:242 RetIndex:1869
CompName:Pentadecanoic acid $$ Pentadecylic acid $$ n-Pentadecanoic acid $$ n-Pentadecylic acid $$

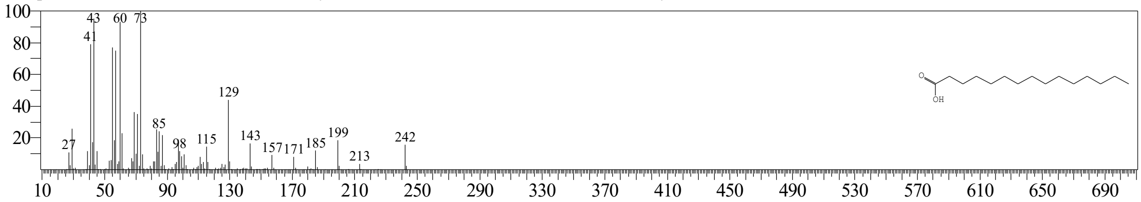

Hit#:2 Entry:84362 Library:NIST11.lib
SI:73 Formula:C16H32O2 CAS:57-10-3 MolWeight:256 RetIndex:1968
CompName:n-Hexadecanoic acid $$ Hexadecanoic acid $$ n-Hexadecoic acid $$ Palmitic acid $$ Pentadecanecarboxylic acid $$ 1-Pentadecanecarboxylic acid

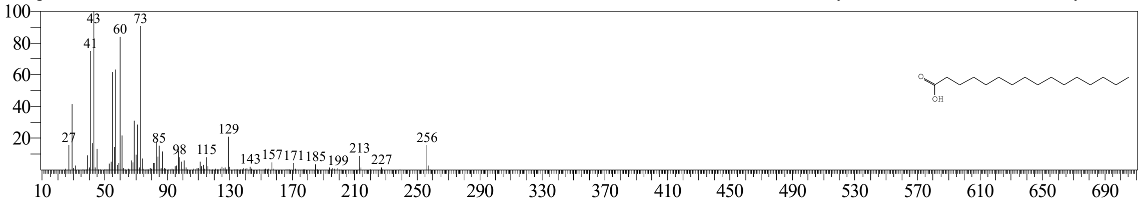

Hit#:3 Entry:53599 Library:NIST11.lib
SI:72 Formula:C13H26O2 CAS:638-53-9 MolWeight:214 RetIndex:1670
CompName:Tridecanoic acid $$ n-Tridecanoic acid $$ n-Tridecoic acid $$ Tridecylic acid $$

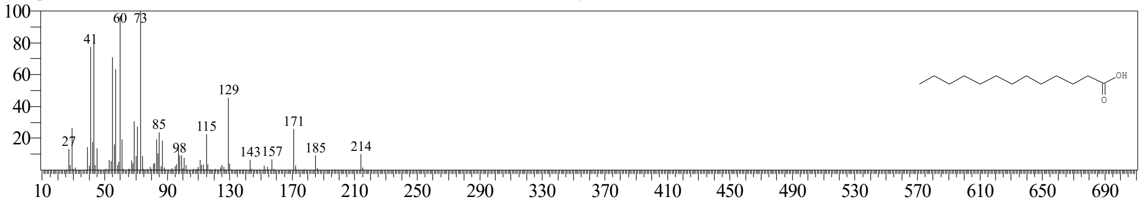

Hit#:4 Entry:63810 Library:NIST11.lib
SI:72 Formula:C14H28O2 CAS:544-63-8 MolWeight:228 RetIndex:1769
CompName:Tetradecanoic acid $$ Myristic acid $$ n-Tetradecanoic acid $$ n-Tetradecoic acid $$ Neo-Fat 14 $$ Univol U 316S $$ 1-Tridecanecarboxylic acid $

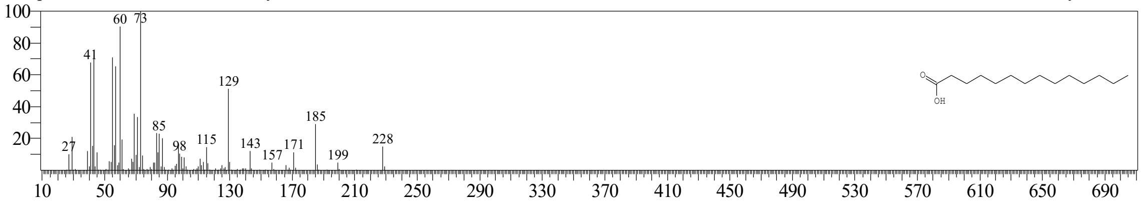

Hit#:5 Entry:117133 Library:NIST11.lib
SI:72 Formula:C19H38O2 CAS:646-30-0 MolWeight:298 RetIndex:2266
CompName:Nonadecanoic acid $$ n-Nonadecanoic acid $$

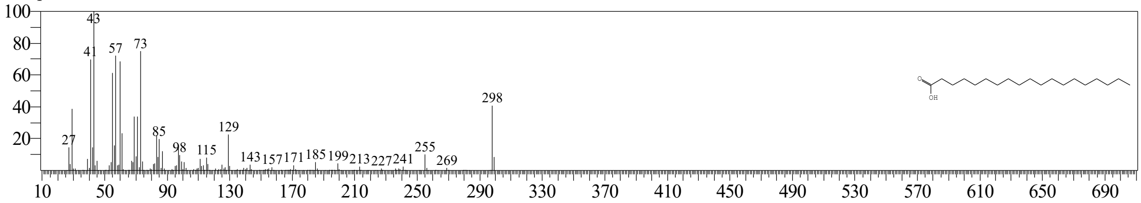

Qualitative Analysis Report

<< Target >>
Line#:111 R.Time:13.620(Scan#:1925) MassPeaks:377
RawMode:Averaged 13.615-13.625(1924-1926) BasePeak:79.05(219189)
BG Mode:Calc. from Peak Group 1 - Event 1 Scan

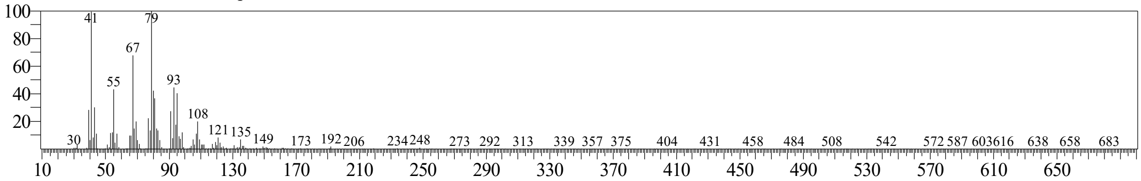

Hit#:1 Entry:90503 Library:NIST11.lib
SI:91 Formula:C18H32O CAS:506-44-5 MolWeight:264 RetIndex:2077
CompName:9,12,15-Octadecatrien-1-ol, (Z,Z,Z)- $$ (9E,12E,15E)-9,12,15-Octadecatrien-1-ol # $$ (Z,Z,Z)-9,12,15-Octadecatrien-1-ol $$

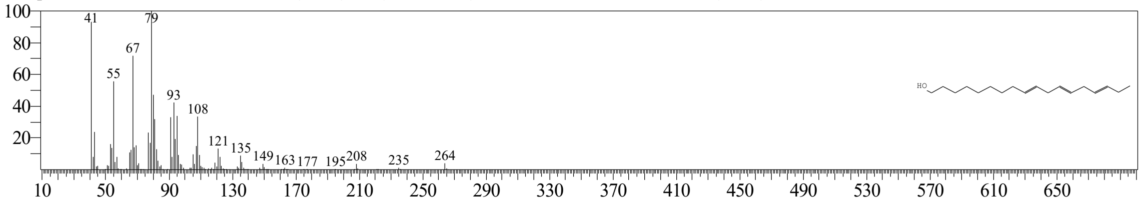

Hit#:2 Entry:67943 Library:NIST11.lib
SI:90 Formula:C16H26O CAS:56797-43-4 MolWeight:234 RetIndex:1824
CompName:cis,cis,cis-7,10,13-Hexadecatrienal $$ (7Z,10Z,13Z)-7,10,13-Hexadecatrienal # $$

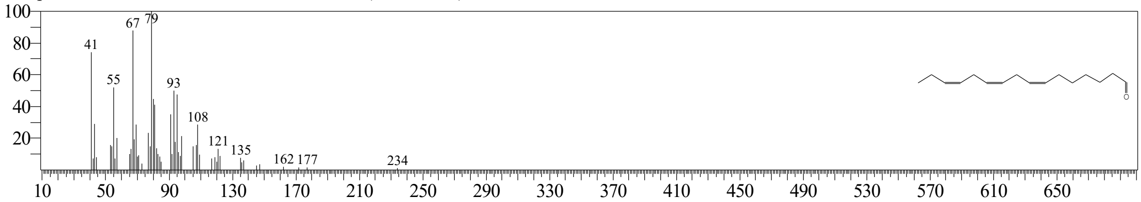

Hit#:3 Entry:89012 Library:NIST11.lib
SI:90 Formula:C18H30O CAS:26537-71-3 MolWeight:262 RetIndex:2023
CompName:9,12,15-Octadecatrienal $$ (9E,12E,15E)-9,12,15-Octadecatrienal # $$

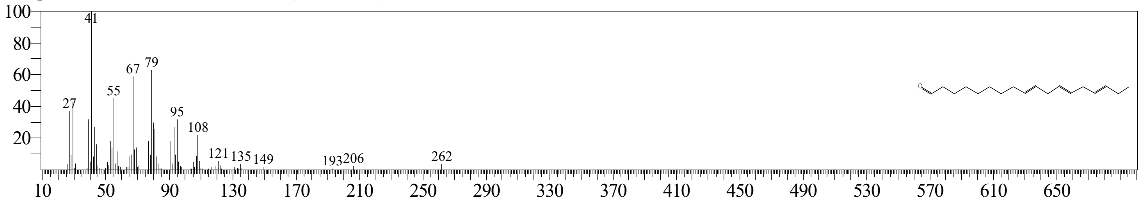

Hit#:4 Entry:101390 Library:NIST11.lib
SI:88 Formula:C18H30O2 CAS:463-40-1 MolWeight:278 RetIndex:2191
CompName:9,12,15-Octadecatrienoic acid, (Z,Z,Z)- $$ Linolenic acid $$ .alpha.-Linolenic acid $$ All-cis-9,12,15-Octadecatrienoic acid $$ cis,cis,cis-9,12,15-(

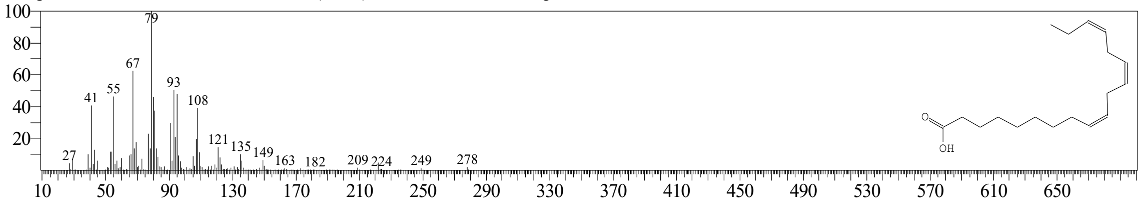

Hit#:5 Entry:38806 Library:NIST11.lib
SI:88 Formula:C14H24 CAS:74752-91-3 MolWeight:192 RetIndex:1420
CompName:1-Tetradecen-3-yne

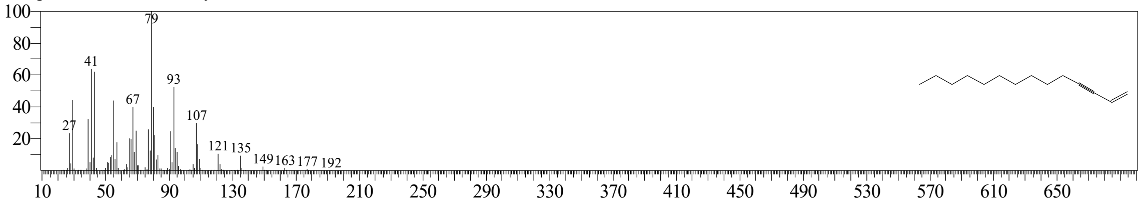

<< Target >>
Line#:112 R.Time:13.765(Scan#:1954) MassPeaks:472
RawMode:Averaged 13.760-13.770(1953-1955) BasePeak:74.05(720709)
BG Mode:Calc. from Peak Group 1 - Event 1 Scan

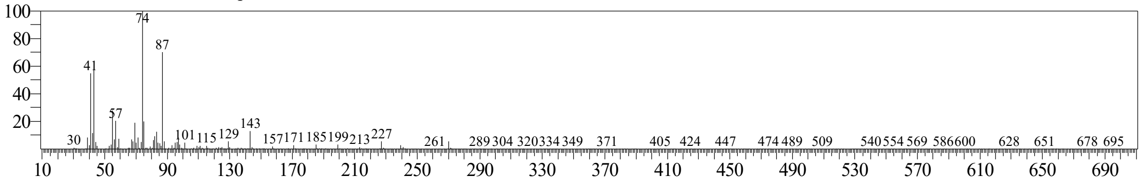

Hit#:1 Entry:95189 Library:NIST11.lib
SI:91 Formula:C17H34O2 CAS:5129-60-2 MolWeight:270 RetIndex:1814
CompName:Pentadecanoic acid, 14-methyl-, methyl ester $$ Methyl 14-methylpentadecanoate # $$ Methyl isohexadecanoate $$

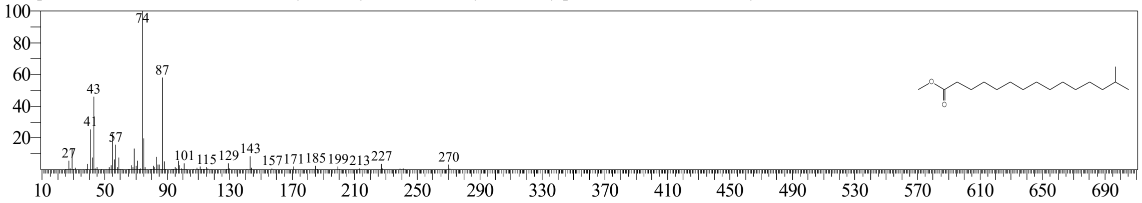

Hit#:2 Entry:115441 Library:NIST11.lib
SI:90 Formula:C19H36O2 CAS:24828-61-3 MolWeight:296 RetIndex:2120
CompName:Cyclopentanetridecanoic acid, methyl ester $$ Methyl dihydrochaulmoograte $$ Methyl 13-cyclopentyltridecanoate # $$

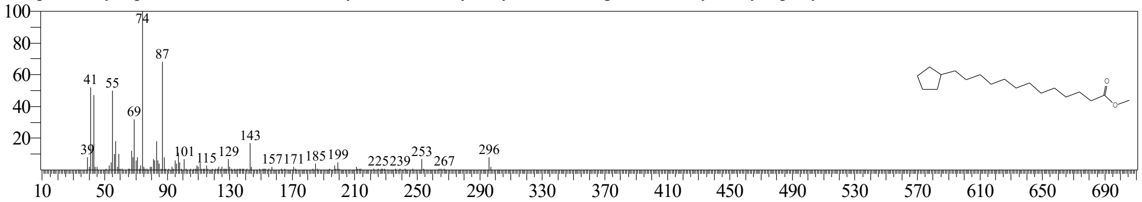

Hit#:3 Entry:73852 Library:NIST11.lib
SI:90 Formula:C15H30O2 CAS:5129-58-8 MolWeight:242 RetIndex:1615
CompName:Tridecanoic acid, 12-methyl-, methyl ester $$ Methyl isomyristate $$ Methyl 12-methyltridecanoate $$

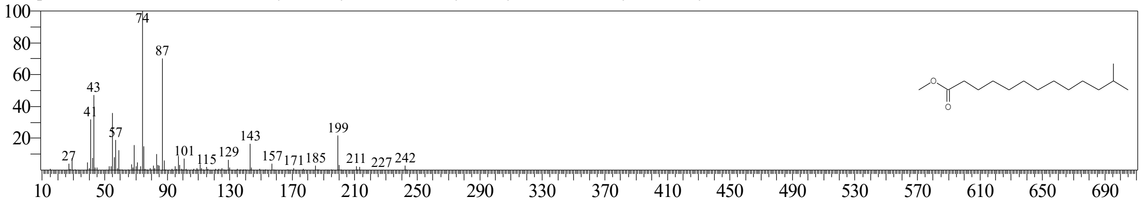

Hit#:4 Entry:106180 Library:NIST11.lib
SI:90 Formula:C18H36O2 CAS:6929-04-0 MolWeight:284 RetIndex:1914
CompName:Hexadecanoic acid, 15-methyl-, methyl ester $$ Methyl isoheptadecanoate $$ Methyl 15-methylhexadecanoate $$

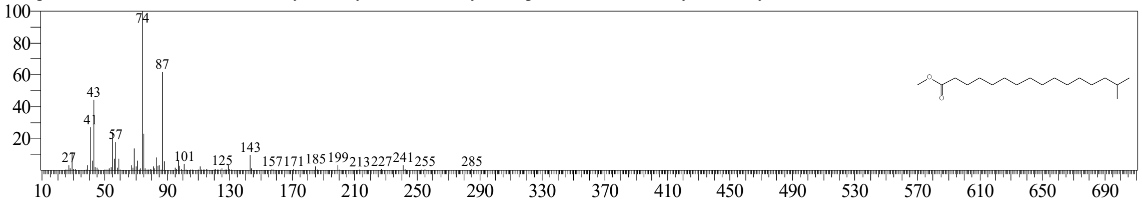

Hit#:5 Entry:95188 Library:NIST11.lib
SI:89 Formula:C17H34O2 CAS:112-39-0 MolWeight:270 RetIndex:1878
CompName:Hexadecanoic acid, methyl ester $$ Palmitic acid, methyl ester $$ n-Hexadecanoic acid methyl ester $$ Metholene 2216 $$ Methyl hexadecanoate $

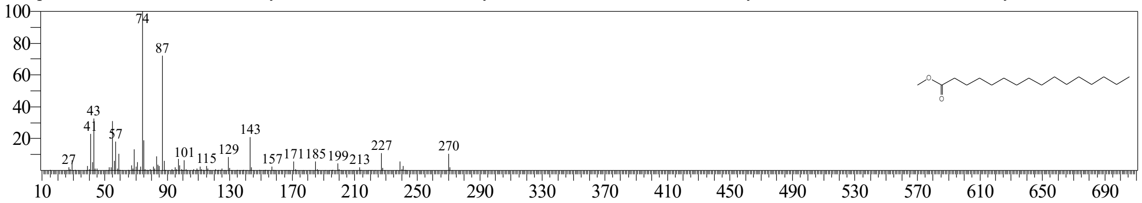

<< Target >>
Line#:113 R.Time:13.845(Scan#:1970) MassPeaks:395
RawMode:Averaged 13.840-13.850(1969-1971) BasePeak:59.10(3736)
BG Mode:Calc. from Peak Group 1 - Event 1 Scan

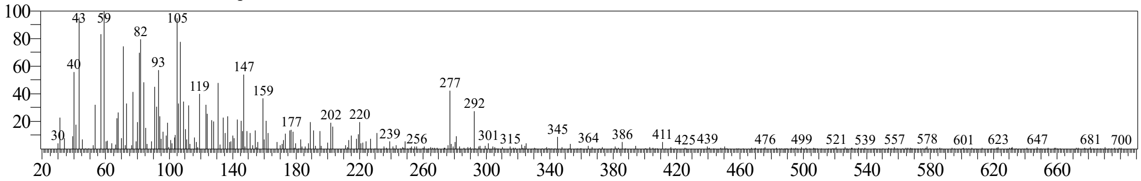

Hit#:1 Entry:116812 Library:NIST11.lib
SI:60 Formula:C17H30O4 CAS:0-00-0 MolWeight:298 RetIndex:2103
CompName:Acetic acid, 10,11-dihydroxy-3,7,11-trimethyl-dodeca-2,6-dienyl ester $$ (2Z,6E)-10,11-Dihydroxy-3,7,11-trimethyl-2,6-dodecadienyl acetate # $$

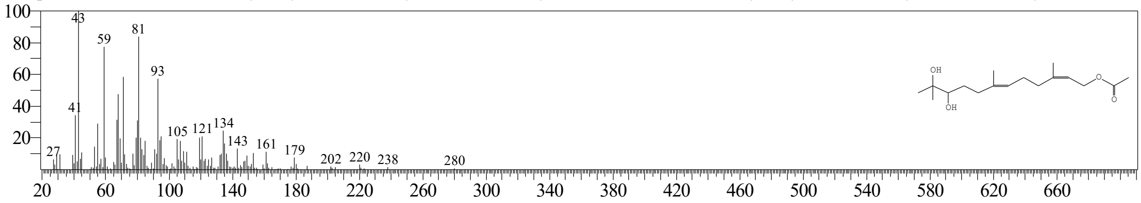

Hit#:2 Entry:57726 Library:NIST11.lib
SI:58 Formula:C15H24O CAS:83637-40-5 MolWeight:220 RetIndex:1540
CompName:Farnesene epoxide, E- $$ 2-[(4E)-5,9-Dimethyl-1-methylene-4,8-decadienyl]oxirane # $$

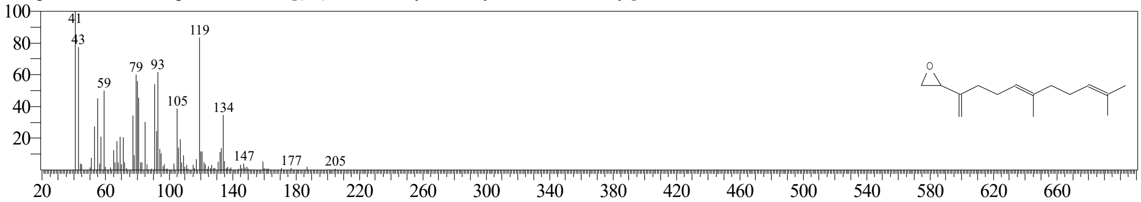

Hit#:3 Entry:207593 Library:NIST11.lib
SI:57 Formula:C37H76O CAS:105794-58-9 MolWeight:536 RetIndex:3942
CompName:1-Heptatriacotanol $$ 1-Heptatriacontanol # $$

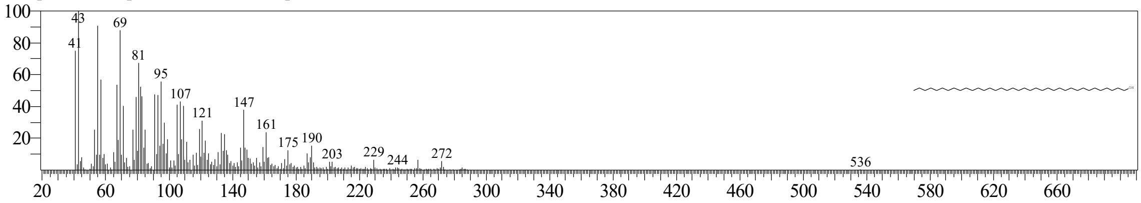

Hit#:4 Entry:59424 Library:NIST11.lib
SI:57 Formula:C15H26O CAS:21284-22-0 MolWeight:222 RetIndex:1580
CompName:Cubenol $$ 1-Isopropyl-4,7-dimethyl-1,3,4,5,6,8a-hexahydro-4a(2H)-naphthalenol-, [1S-(1.alpha.,4.beta.,4a.beta.,8a.alpha.)]- $$ (-)-Cubenol $$ 10

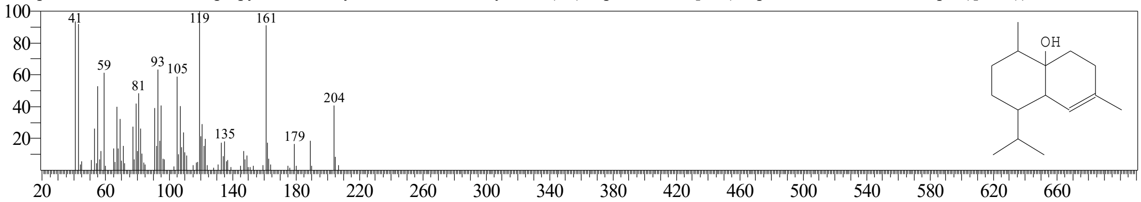

Hit#:5 Entry:101384 Library:NIST11.lib
SI:57 Formula:C18H30O2 CAS:0-00-0 MolWeight:278 RetIndex:1854
CompName:Nerolidyl propionate $$ (4E)-1,5,9-Trimethyl-1-vinyl-4,8-decadienyl propionate # $$

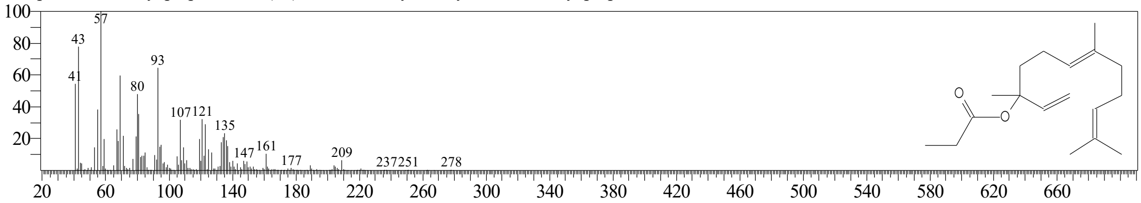

<< Target >>
Line#:114 R.Time:13.965(Scan#:1994) MassPeaks:433
RawMode:Averaged 13.960-13.970(1993-1995) BasePeak:41.05(1308435)
BG Mode:Calc. from Peak Group 1 - Event 1 Scan

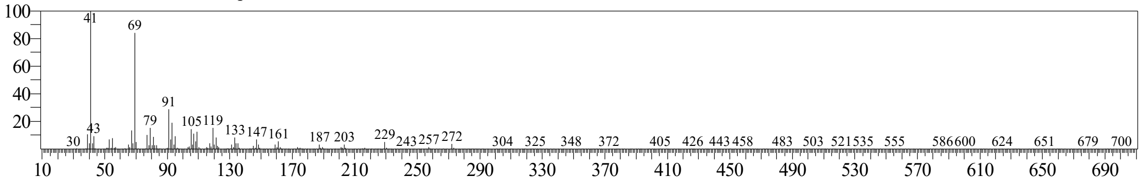

Hit#:1 Entry:46578 Library:NIST11.lib
SI:85 Formula:C15H24 CAS:77129-48-7 MolWeight:204 RetIndex:1440
CompName:1,6,10-Dodecatriene, 7,11-dimethyl-3-methylene- $$ 7,11-Dimethyl-3-methylene-1,6,10-dodecatriene $$

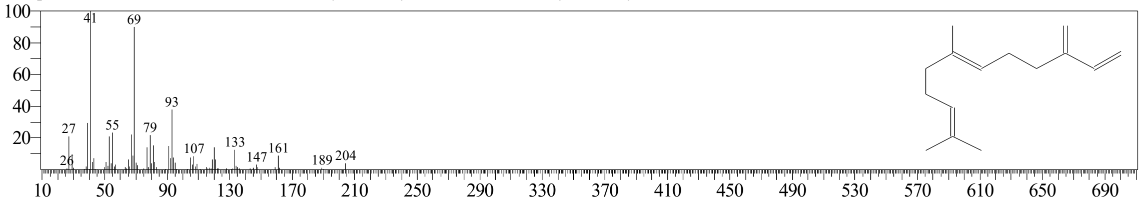

Hit#:2 Entry:96991 Library:NIST11.lib
SI:84 Formula:C20H32 CAS:38259-79-9 MolWeight:272 RetIndex:1958
CompName:2,6,11,15-Tetramethyl-hexadeca-2,6,8,10,14-pentaene $$ (6E,8E,10E)-2,6,11,15-Tetramethyl-2,6,8,10,14-hexadecapentaene # $$

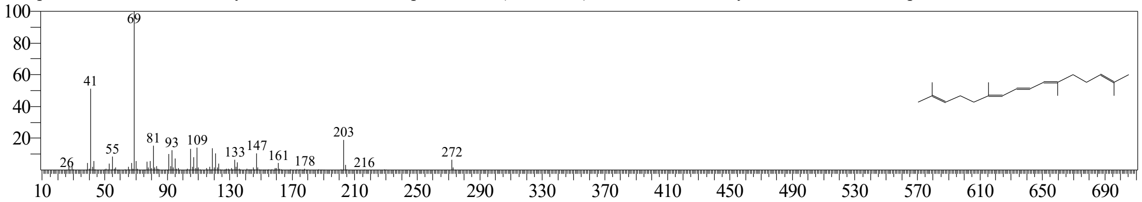

Hit#:3 Entry:110903 Library:NIST11.lib
SI:84 Formula:C20H34O CAS:1113-21-9 MolWeight:290 RetIndex:2046
CompName:1,6,10,14-Hexadecatetraen-3-ol, 3,7,11,15-tetramethyl-, (E,E)- $$ (6E,10E)-3,7,11,15-Tetramethyl-1,6,10,14-hexadecatetraen-3-ol # $$ Geranyl lina

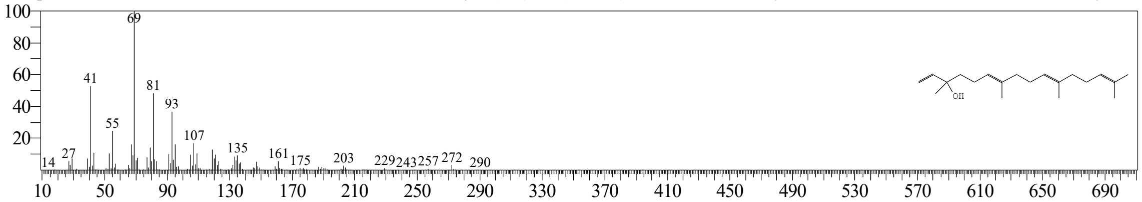

Hit#:4 Entry:46579 Library:NIST11.lib
SI:83 Formula:C15H24 CAS:18794-84-8 MolWeight:204 RetIndex:1440
CompName:(E)-.beta.-Farnesene $$ 1,6,10-Dodecatriene, 7,11-dimethyl-3-methylene-, (E)- $$ 7,11-Dimethyl-3-methylene-1,6,10-dodecatriene, trans $$ (6E)-7,

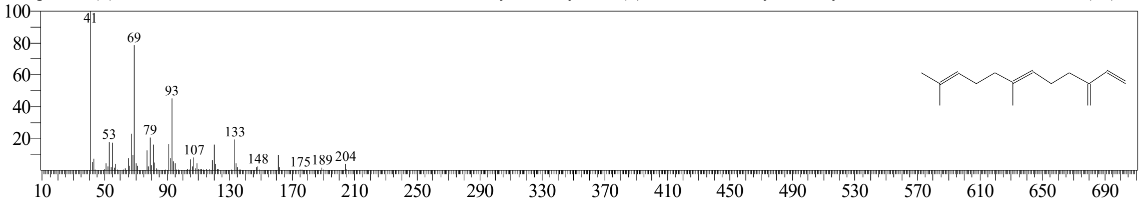

Hit#:5 Entry:46580 Library:NIST11.lib
SI:83 Formula:C15H24 CAS:28973-97-9 MolWeight:204 RetIndex:1440
CompName:cis-.beta.-Farnesene $$ 1,6,10-Dodecatriene, 7,11-dimethyl-3-methylene-, (Z)- $$ (Z)-.beta.-Farnesene $$ (6Z)-7,11-Dimethyl-3-methylene-1,6,10-c

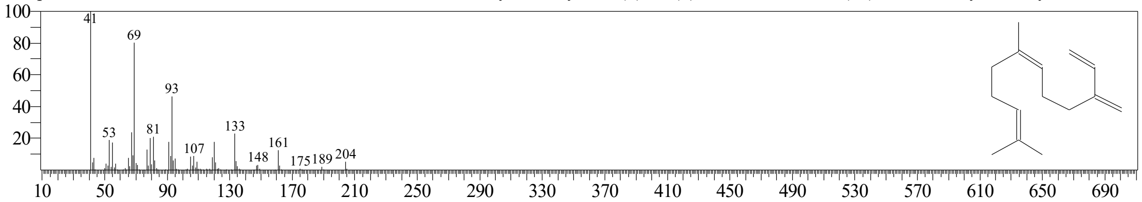

<< Target >>
Line#:115 R.Time:14.100(Scan#:2021) MassPeaks:485
RawMode:Averaged 14.095-14.105(2020-2022) BasePeak:43.10(835177)
BG Mode:Calc. from Peak Group 1 - Event 1 Scan

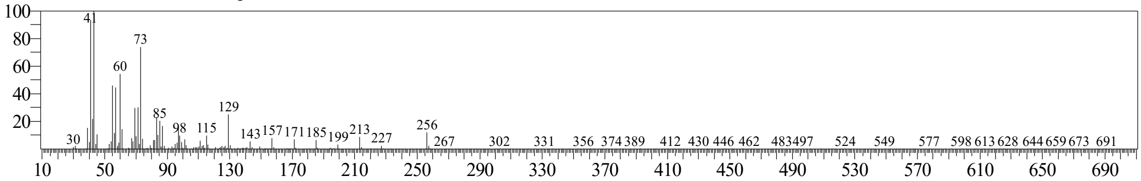

Hit#:1 Entry:84362 Library:NIST11.lib
SI:92 Formula:C16H32O2 CAS:57-10-3 MolWeight:256 RetIndex:1968
CompName:n-Hexadecanoic acid $$ Hexadecanoic acid $$ n-Hexadecoic acid $$ Palmitic acid $$ Pentadecanecarboxylic acid $$ 1-Pentadecanecarboxylic acid

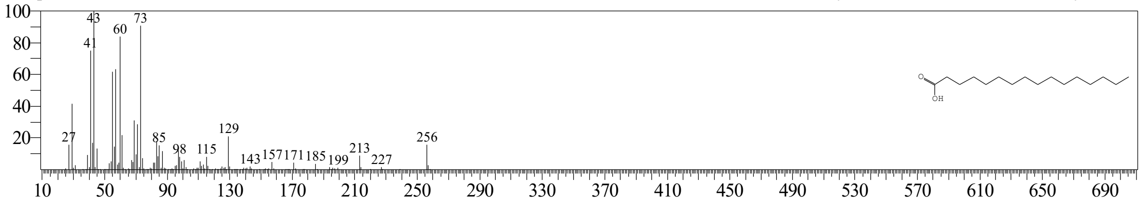

Hit#:2 Entry:128151 Library:NIST11.lib
SI:90 Formula:C20H40O2 CAS:506-30-9 MolWeight:312 RetIndex:2366
CompName:Eicosanoic acid $$ Arachic acid $$ Arachidic acid $$ Icosanoic acid $$ n-Eicosanoic acid $$ Arachidic acid (synthetic) $$

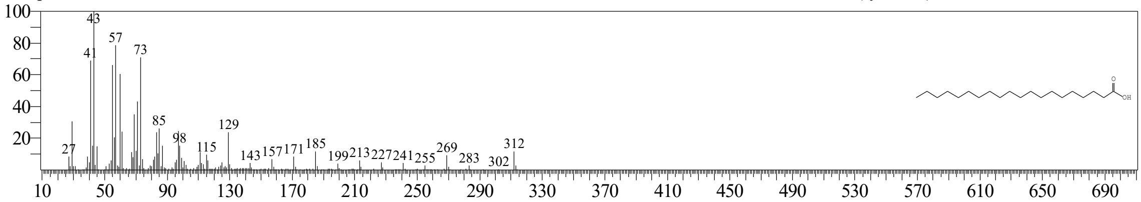

Hit#:3 Entry:53599 Library:NIST11.lib
SI:90 Formula:C13H26O2 CAS:638-53-9 MolWeight:214 RetIndex:1670
CompName:Tridecanoic acid $$ n-Tridecanoic acid $$ n-Tridecoic acid $$ Tridecylic acid $$

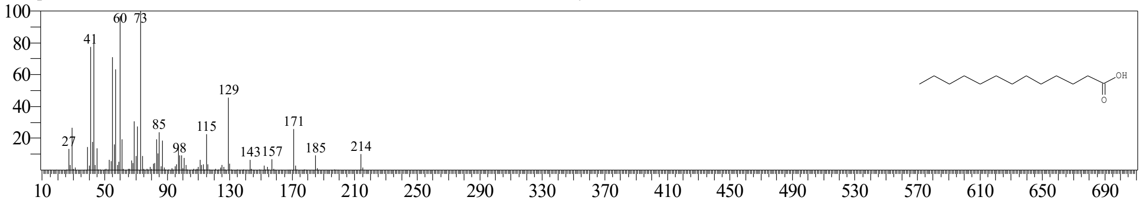

Hit#:4 Entry:73851 Library:NIST11.lib
SI:89 Formula:C15H30O2 CAS:1002-84-2 MolWeight:242 RetIndex:1869
CompName:Pentadecanoic acid $$ Pentadecylic acid $$ n-Pentadecanoic acid $$ n-Pentadecylic acid $$

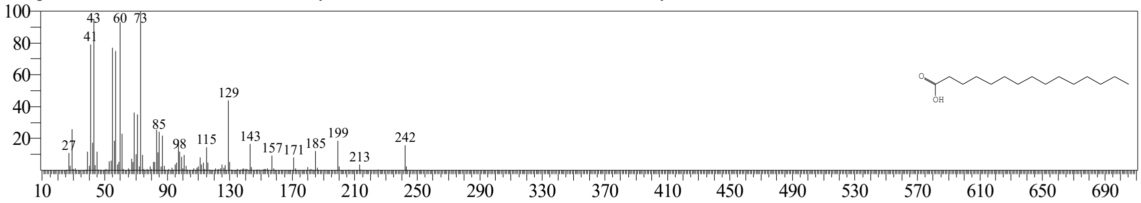

Hit#:5 Entry:169171 Library:NIST11.lib
SI:89 Formula:C22H44O4 CAS:106-11-6 MolWeight:372 RetIndex:2694
CompName:Octadecanoic acid, 2-(2-hydroxyethoxy)ethyl ester $$ Aqua Cera $$ Atlas G 2146 $$ Cerasynt $$ Cerasynt Special $$ Clindrol SDG $$ Diethylene

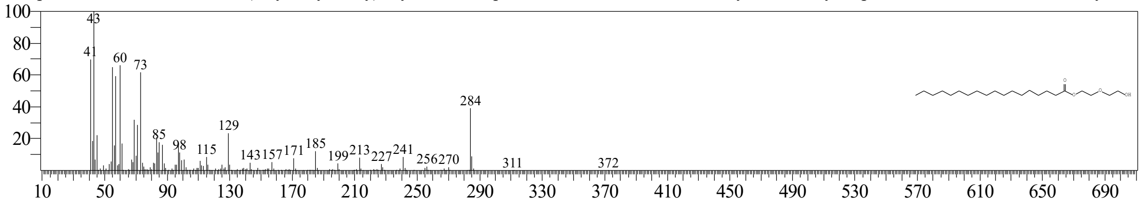

Qualitative Analysis Report

<< Target >>
Line#:116 R.Time:14.215(Scan#:2044) MassPeaks:465
RawMode:Averaged 14.210-14.220(2043-2045) BasePeak:41.05(1061783)
BG Mode:Calc. from Peak Group 1 - Event 1 Scan

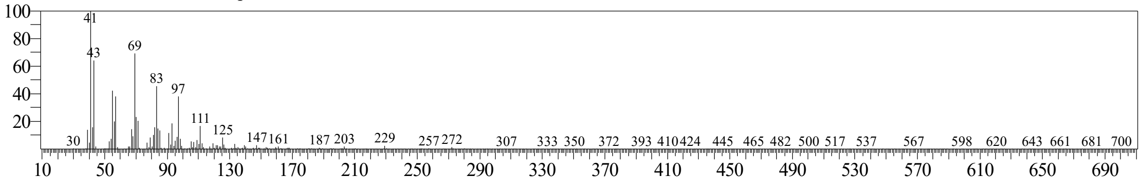

Hit#:1 Entry:41639 Library:NIST11.lib
SI:88 Formula:C14H28 CAS:41446-68-8 MolWeight:196 RetIndex:1421
CompName:3-Tetradecene, (E)- $$ (3E)-3-Tetradecene $$ (E)-3-Tetradecene $$ trans-3-Tetradecene $$

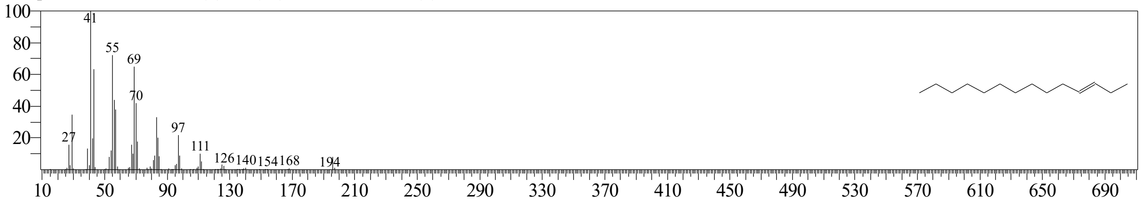

Hit#:2 Entry:45176 Library:NIST11.lib
SI:86 Formula:C12H26S CAS:10059-13-9 MolWeight:202 RetIndex:1433
CompName:2-Undecanethiol, 2-methyl- $$ 1,1-Dimethyl-decyl-mercaptan $$ 2-Methylundecyl-2-thiol $$ 1,1-Dimethyldecyl hydrosulfide # $$ 2-Methyl-2-und

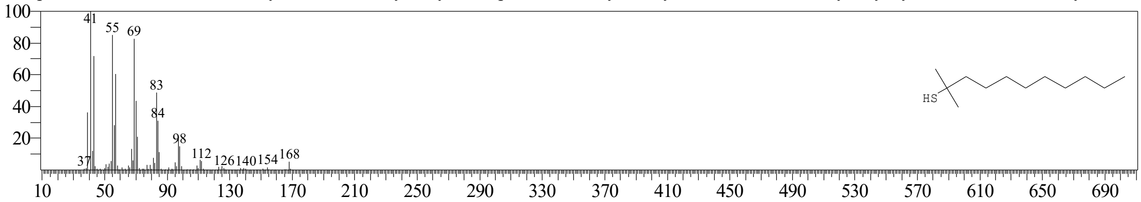

Hit#:3 Entry:41641 Library:NIST11.lib
SI:86 Formula:C14H28 CAS:41446-78-0 MolWeight:196 RetIndex:1421
CompName:4-Tetradecene, (E)- $$ trans-4-Tetradecene $$ (4E)-4-Tetradecene $$ (E)-4-Tetradecene $$

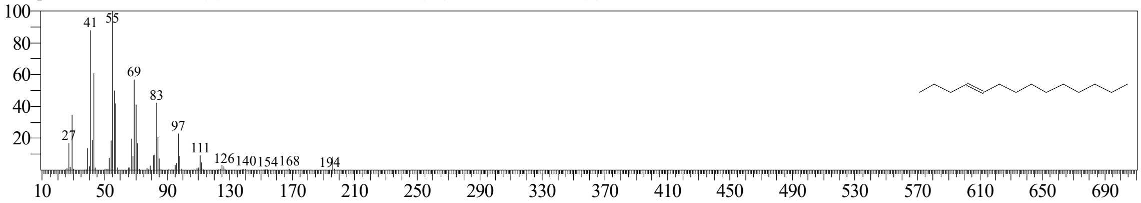

Hit#:4 Entry:72461 Library:NIST11.lib
SI:86 Formula:C16H32O CAS:0-00-0 MolWeight:240 RetIndex:1862
CompName:E-2-Hexadecacen-1-ol $$ (2E)-2-Hexadecen-1-ol # $$

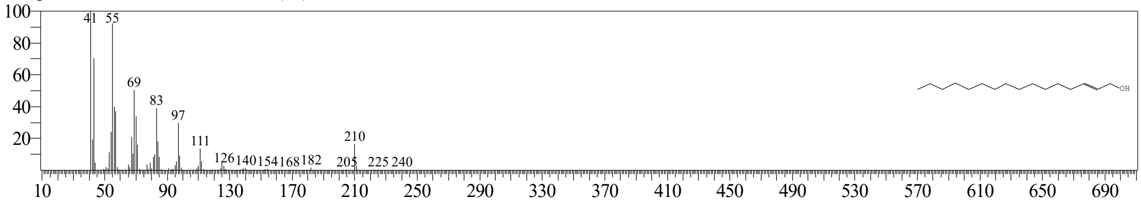

Hit#:5 Entry:91989 Library:NIST11.lib
SI:86 Formula:C19H38 CAS:35354-39-3 MolWeight:266 RetIndex:1854
CompName:cis-2-Methyl-7-octadecene $$ Disparlure olefin $$ (7Z)-2-Methyl-7-octadecene $$ 2-Methyl-7-octadecene, Z $$ 2-Methyl-(Z)-7-octadecene $$ 7-O

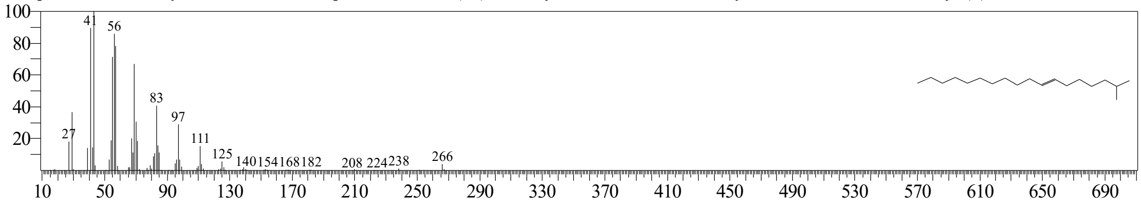

Qualitative Analysis Report

<< Target >>
Line#:117 R.Time:14.435(Scan#:2088) MassPeaks:365
RawMode:Averaged 14.430-14.440(2087-2089) BasePeak:74.00(28546)
BG Mode:Calc. from Peak Group 1 - Event 1 Scan

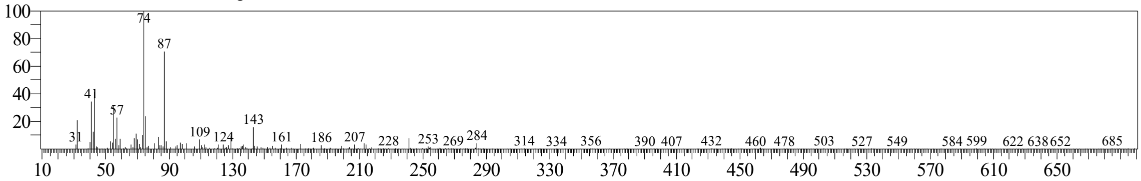

Hit#:1 Entry:106180 Library:NIST11.lib
SI:84 Formula:C18H36O2 CAS:6929-04-0 MolWeight:284 RetIndex:1914
CompName:Hexadecanoic acid, 15-methyl-, methyl ester $$ Methyl isoheptadecanoate $$ Methyl 15-methylhexadecanoate $$

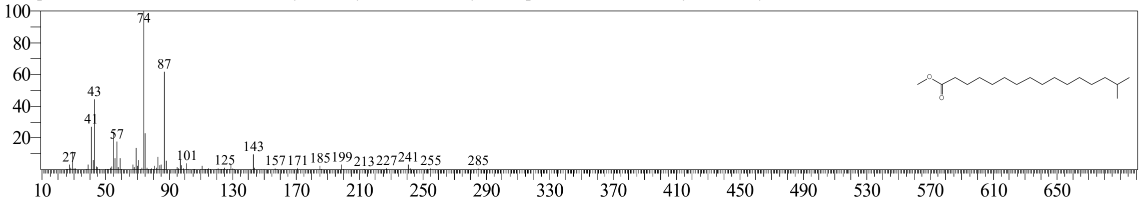

Hit#:2 Entry:106179 Library:NIST11.lib
SI:83 Formula:C18H36O2 CAS:2490-49-5 MolWeight:284 RetIndex:1914
CompName:Hexadecanoic acid, 14-methyl-, methyl ester $$ Methyl 14-methylhexadecanoate $$

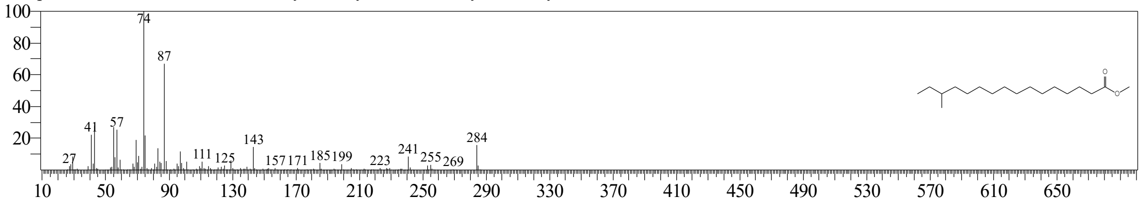

Hit#:3 Entry:73852 Library:NIST11.lib
SI:83 Formula:C15H30O2 CAS:5129-58-8 MolWeight:242 RetIndex:1615
CompName:Tridecanoic acid, 12-methyl-, methyl ester $$ Methyl isomyristate $$ Methyl 12-methyltridecanoate $$

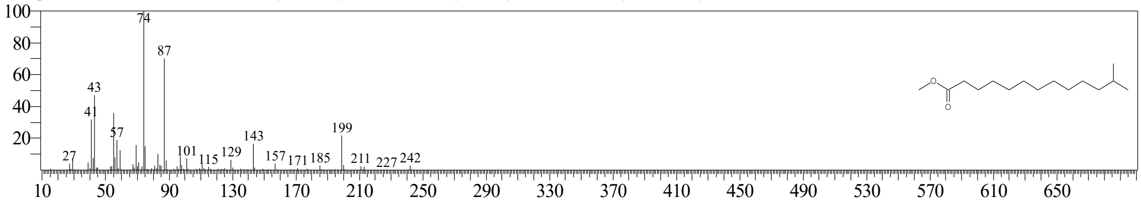

Hit#:4 Entry:95189 Library:NIST11.lib
SI:82 Formula:C17H34O2 CAS:5129-60-2 MolWeight:270 RetIndex:1814
CompName:Pentadecanoic acid, 14-methyl-, methyl ester $$ Methyl 14-methylpentadecanoate # $$ Methyl isohexadecanoate $$

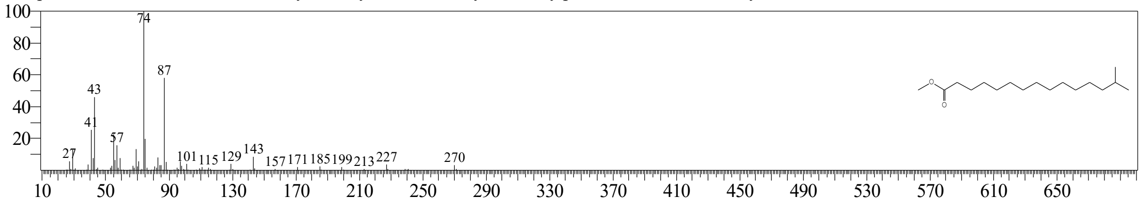

Hit#:5 Entry:138745 Library:NIST11.lib
SI:81 Formula:C21H42O2 CAS:0-00-0 MolWeight:326 RetIndex:2212
CompName:Methyl 18-methylnonadecanoate

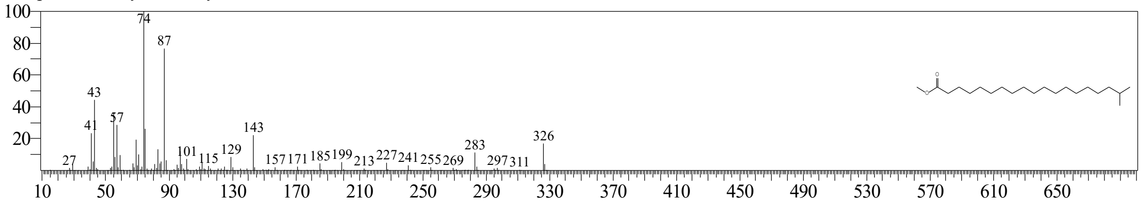

<< Target >>
Line#:118 R.Time:14.470(Scan#:2095) MassPeaks:360
RawMode:Averaged 14.465-14.475(2094-2096) BasePeak:69.10(42489)
BG Mode:Calc. from Peak Group 1 - Event 1 Scan

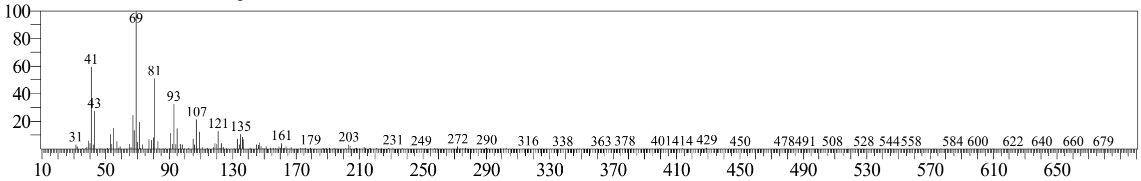

Hit#:1 Entry:110903 Library:NIST11.lib
SI:90 Formula:C20H34O CAS:1113-21-9 MolWeight:290 RetIndex:2046
CompName:1,6,10,14-Hexadecatetraen-3-ol, 3,7,11,15-tetramethyl-, (E,E)- $$ (6E,10E)-3,7,11,15-Tetramethyl-1,6,10,14-hexadecatetraen-3-ol # $$ Geranyl lina

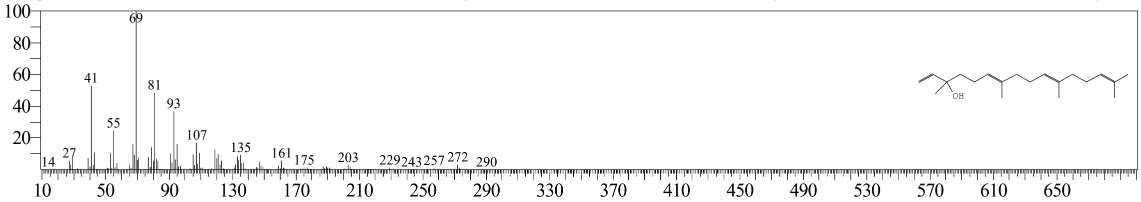

Hit#:2 Entry:110905 Library:NIST11.lib
SI:89 Formula:C20H34O CAS:24034-73-9 MolWeight:290 RetIndex:2192
CompName:trans-Geranylgeraniol $$ 2,6,10,14-Hexadecatetraen-1-ol, 3,7,11,15-tetramethyl-, (E,E,E)- $$ All-trans-Geranylgeraniol $$

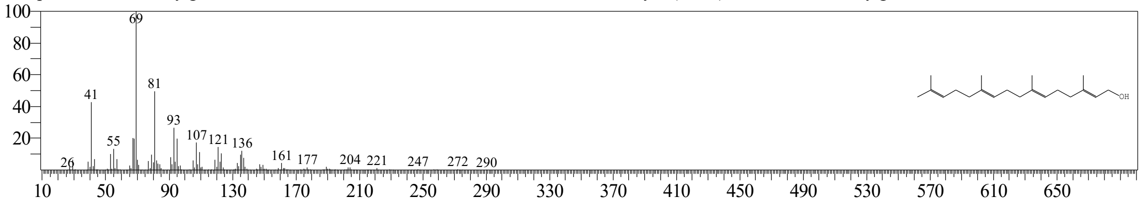

Hit#:3 Entry:59387 Library:NIST11.lib
SI:88 Formula:C15H26O CAS:40716-66-3 MolWeight:222 RetIndex:1564
CompName:1,6,10-Dodecatrien-3-ol, 3,7,11-trimethyl-, (E)- $$ (.+/-.)-trans-Nerolidol $$ (6E)-3,7,11-Trimethyl-1,6,10-dodecatrien-3-ol # $$ (6E)-Nerolidol $$

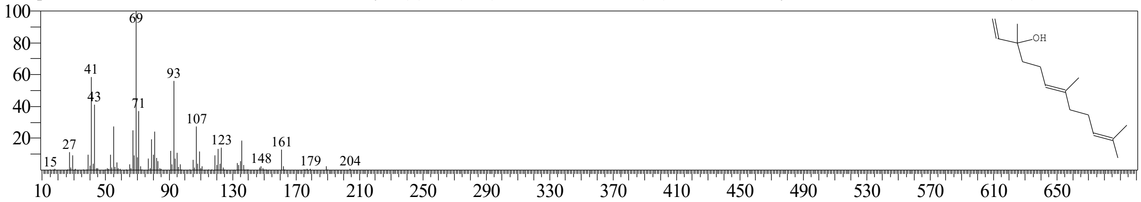

Hit#:4 Entry:110904 Library:NIST11.lib
SI:87 Formula:C20H34O CAS:7614-21-3 MolWeight:290 RetIndex:2192
CompName:Hexadeca-2,6,10,14-tetraen-1-ol, 3,7,11,16-tetramethyl- $$ 3,7,11,15-Tetramethyl-2,6,10,14-hexadecatetraen-1-ol $$ Tetraprenol $$

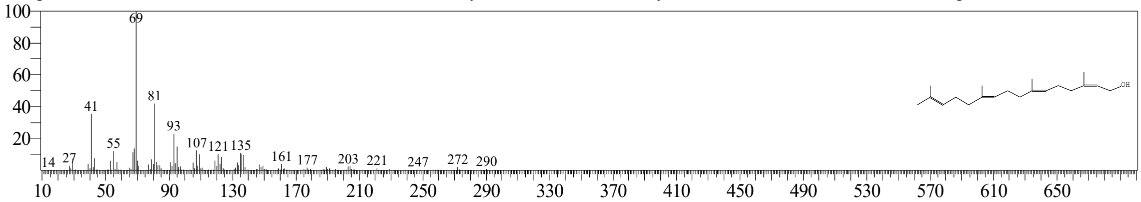

Hit#:5 Entry:110906 Library:NIST11.lib
SI:87 Formula:C20H34O CAS:68931-30-6 MolWeight:290 RetIndex:2046
CompName:3,7,11,15-Tetramethylhexadeca-1,6,10,14-tetraen-3-ol $$ 3,7,11,15-Tetramethyl-1,6,10,14-hexadecatetraen-3-ol $$

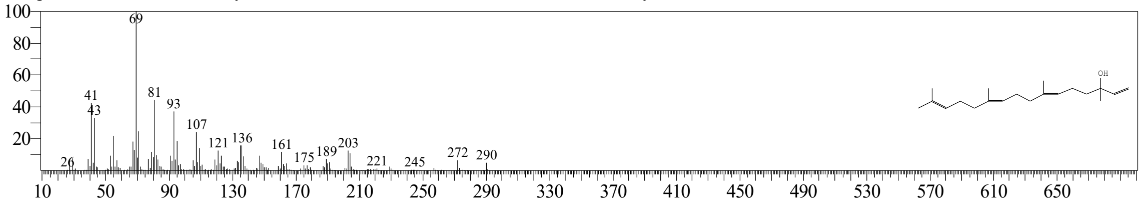

<< Target >>
Line#:119 R.Time:14.620(Scan#:2125) MassPeaks:344
RawMode:Averaged 14.615-14.625(2124-2126) BasePeak:32.10(11991)
BG Mode:Calc. from Peak Group 1 - Event 1 Scan

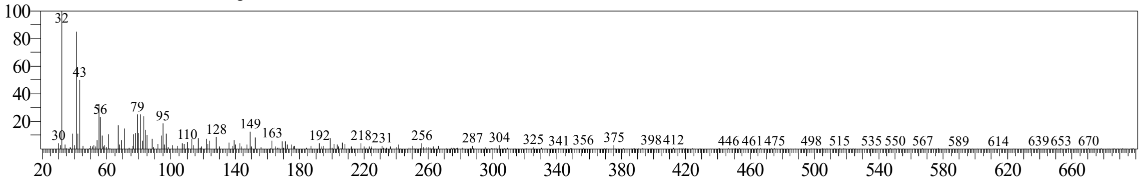

Hit#:1 Entry:24889 Library:NIST11.lib
SI:66 Formula:C12H24 CAS:74630-60-7 MolWeight:168 RetIndex:1158
CompName:4-Undecene, 10-methyl-, (E)- $$ (4E)-10-Methyl-4-undecene # $$

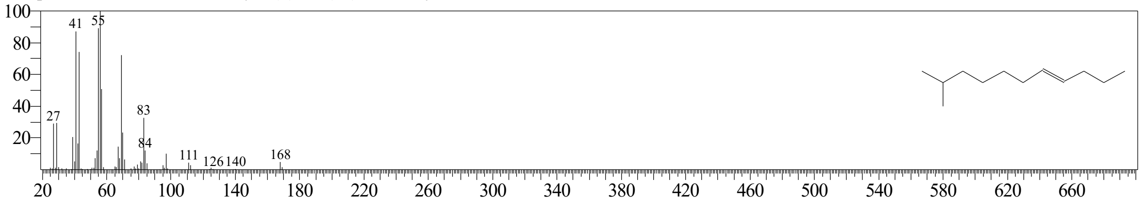

Hit#:2 Entry:134365 Library:NIST11.lib
SI:65 Formula:C21H36O2 CAS:21061-10-9 MolWeight:320 RetIndex:2300
CompName:8,11,14-Eicosatrienoic acid, methyl ester, (Z,Z,Z)- $$ Methyl dihomo-.gamma.-linolenate $$ Methyl (8Z,11Z,14Z)-8,11,14-icosatrienoate # $$ Dihc

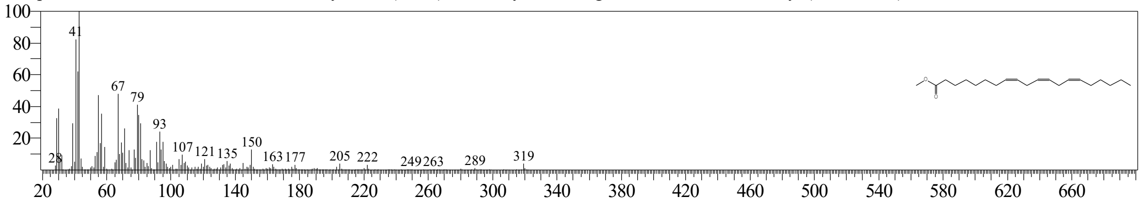

Hit#:3 Entry:102804 Library:NIST11.lib
SI:64 Formula:C18H32O2 CAS:0-00-0 MolWeight:280 RetIndex:1994
CompName:Z,Z-10,12-Hexadecadien-1-ol acetate $$ (10Z,12Z)-10,12-Hexadecadienyl acetate # $$

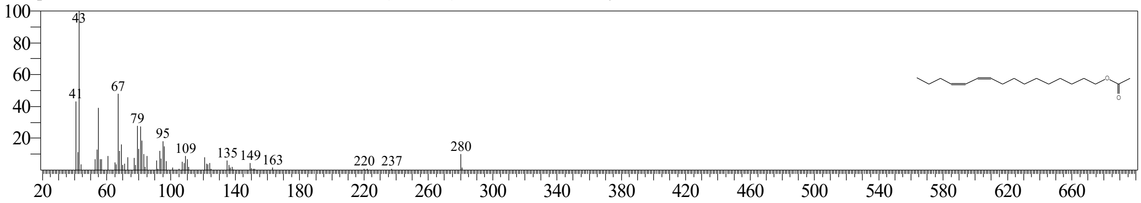

Hit#:4 Entry:60905 Library:NIST11.lib
SI:64 Formula:C15H28O CAS:2834-00-6 MolWeight:224 RetIndex:1772
CompName:2-Pentadecyn-1-ol

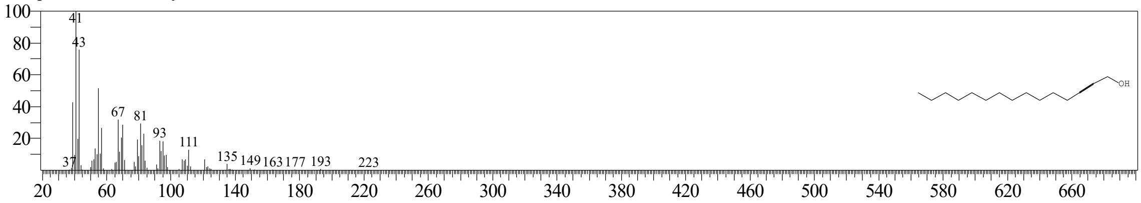

Hit#:5 Entry:137860 Library:NIST11.lib
SI:64 Formula:C12H24Br2 CAS:55334-42-4 MolWeight:326 RetIndex:1742
CompName:Dodecane, 1,2-dibromo- $$ 1,2-Dibromododecane $$

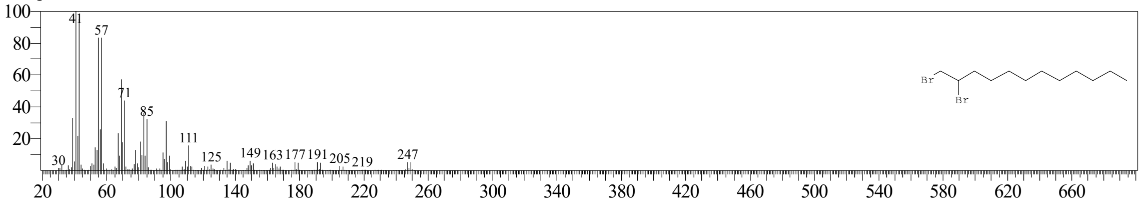

<< Target >>
Line#:120 R.Time:14.730(Scan#:2147) MassPeaks:378
RawMode:Averaged 14.725-14.735(2146-2148) BasePeak:43.10(20614)
BG Mode:Calc. from Peak Group 1 - Event 1 Scan

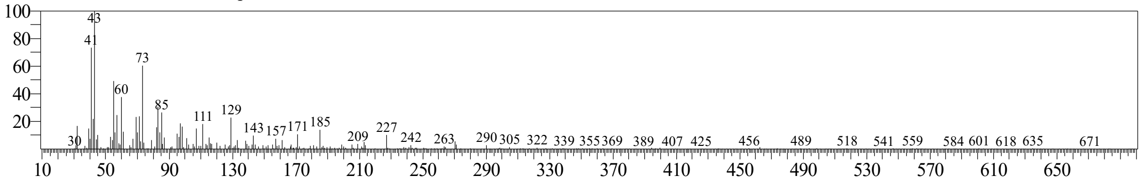

Hit#:1 Entry:128151 Library:NIST11.lib
SI:85 Formula:C20H40O2 CAS:506-30-9 MolWeight:312 RetIndex:2366
CompName:Eicosanoic acid $$ Arachic acid $$ Arachidic acid $$ Icosanoic acid $$ n-Eicosanoic acid $$ Arachidic acid (synthetic) $$

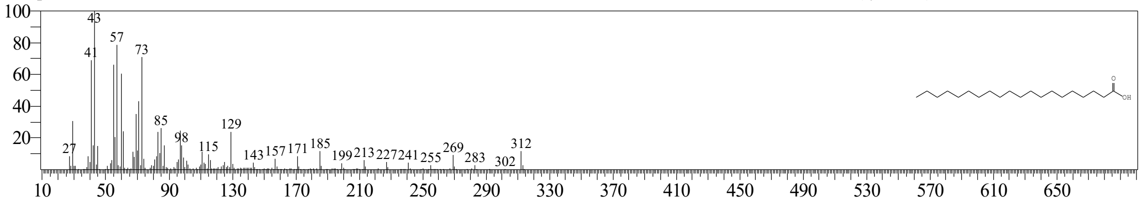

Hit#:2 Entry:169171 Library:NIST11.lib
SI:83 Formula:C22H44O4 CAS:106-11-6 MolWeight:372 RetIndex:2694
CompName:Octadecanoic acid, 2-(2-hydroxyethoxy)ethyl ester $$ Aqua Cera $$ Atlas G 2146 $$ Cerasynt $$ Cerasynt Special $$ Clindrol SDG $$ Diethylene

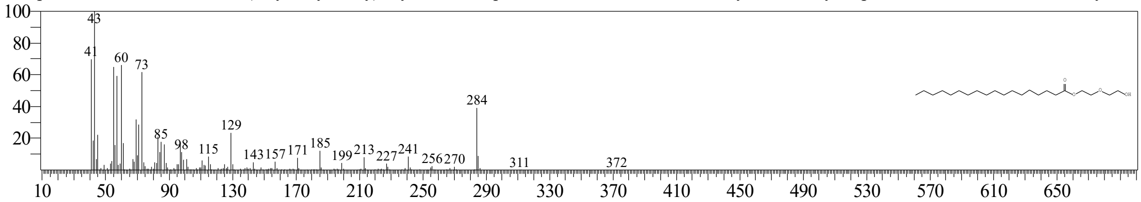

Hit#:3 Entry:84362 Library:NIST11.lib
SI:82 Formula:C16H32O2 CAS:57-10-3 MolWeight:256 RetIndex:1968
CompName:n-Hexadecanoic acid $$ Hexadecanoic acid $$ n-Hexadecoic acid $$ Palmitic acid $$ Pentadecanecarboxylic acid $$ 1-Pentadecanecarboxylic acid

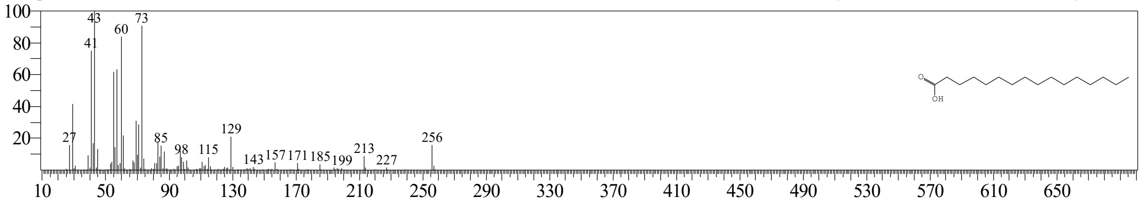

Hit#:4 Entry:73851 Library:NIST11.lib
SI:82 Formula:C15H30O2 CAS:1002-84-2 MolWeight:242 RetIndex:1869
CompName:Pentadecanoic acid $$ Pentadecylic acid $$ n-Pentadecanoic acid $$ n-Pentadecylic acid $$

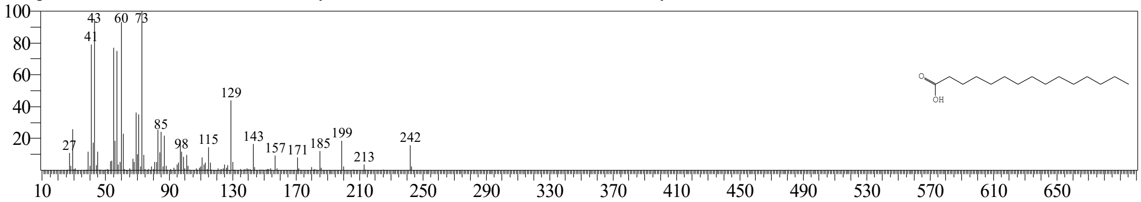

Hit#:5 Entry:53599 Library:NIST11.lib
SI:82 Formula:C13H26O2 CAS:638-53-9 MolWeight:214 RetIndex:1670
CompName:Tridecanoic acid $$ n-Tridecanoic acid $$ n-Tridecoic acid $$ Tridecylic acid $$

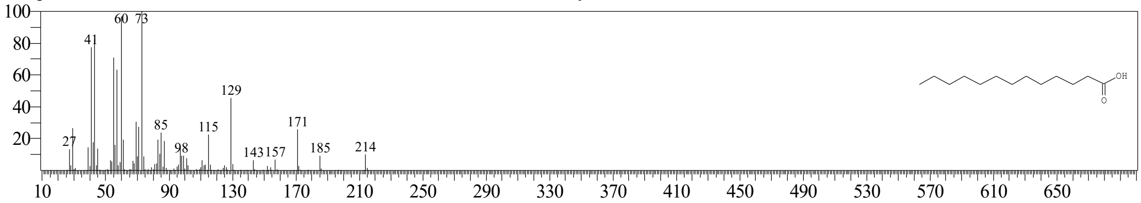

<< Target >>
Line#:121 R.Time:14.880(Scan#:2177) MassPeaks:406
RawMode:Averaged 14.875-14.885(2176-2178) BasePeak:41.10(91743)
BG Mode:Calc. from Peak Group 1 - Event 1 Scan

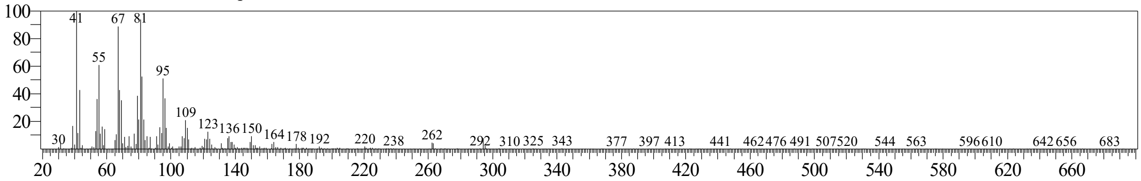

Hit#:1 Entry:113947 Library:NIST11.lib
SI:95 Formula:C19H34O2 CAS:2462-85-3 MolWeight:294 RetIndex:2093
CompName:9,12-Octadecadienoic acid, methyl ester $$ 9,12-Octadecenoic acid, methyl ester $$ Methyl octadeca-9,12-dienoate $$ Methyl 9,12-octadecadienoa

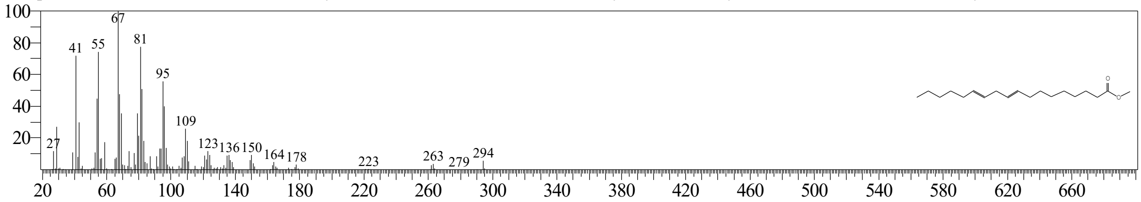

Hit#:2 Entry:113946 Library:NIST11.lib
SI:94 Formula:C19H34O2 CAS:56554-62-2 MolWeight:294 RetIndex:2093
CompName:10,13-Octadecadienoic acid, methyl ester $$ Methyl (10E,13E)-10,13-octadecadienoate # $$

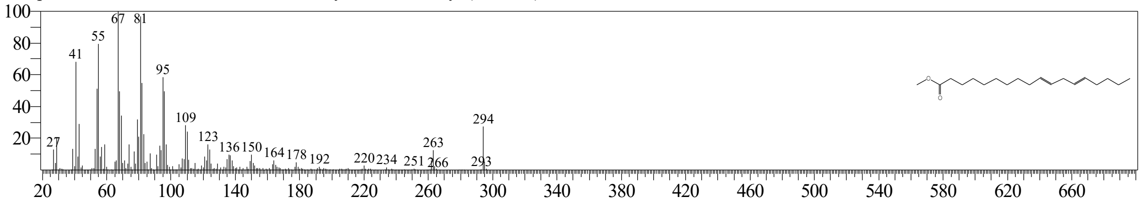

Hit#:3 Entry:113931 Library:NIST11.lib
SI:93 Formula:C19H34O2 CAS:2566-97-4 MolWeight:294 RetIndex:2093
CompName:9,12-Octadecadienoic acid, methyl ester, (E,E)- $$ Linolelaidic acid, methyl ester $$ Methyl linolelaidate $$ Methyl trans,trans-9,12-octadecadieno

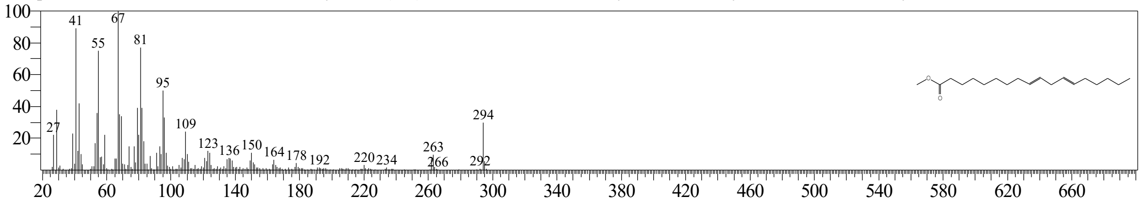

Hit#:4 Entry:135723 Library:NIST11.lib
SI:93 Formula:C21H38O2 CAS:2463-02-7 MolWeight:322 RetIndex:2292
CompName:11,14-Eicosadienoic acid, methyl ester $$ Methyl (11E,14E)-11,14-icosadienoate # $$

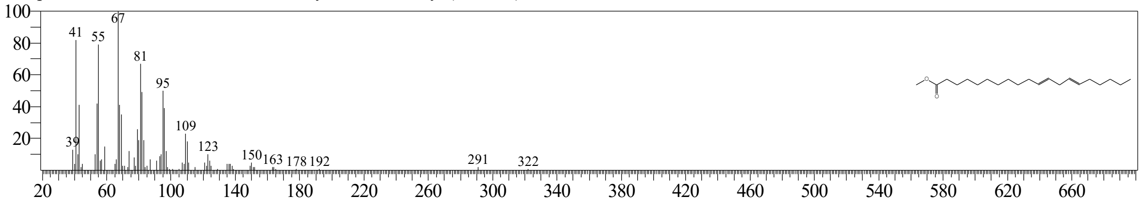

Hit#:5 Entry:113940 Library:NIST11.lib
SI:93 Formula:C19H34O2 CAS:56599-58-7 MolWeight:294 RetIndex:2093
CompName:8,11-Octadecadienoic acid, methyl ester $$ Methyl (8E,11E)-8,11-octadecadienoate # $$

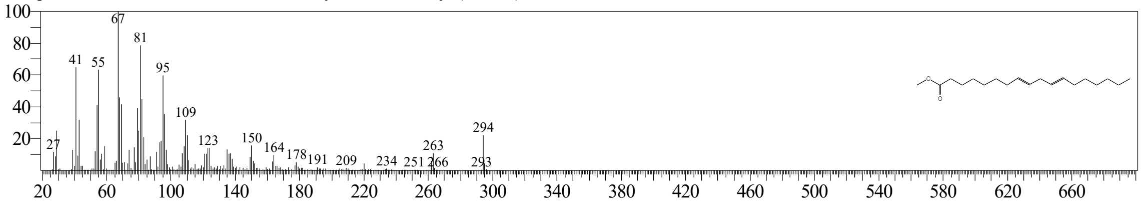

<< Target >>
Line#:122 R.Time:14.925(Scan#:2186) MassPeaks:434
RawMode:Averaged 14.920-14.930(2185-2187) BasePeak:79.10(544651)
BG Mode:Calc. from Peak Group 1 - Event 1 Scan

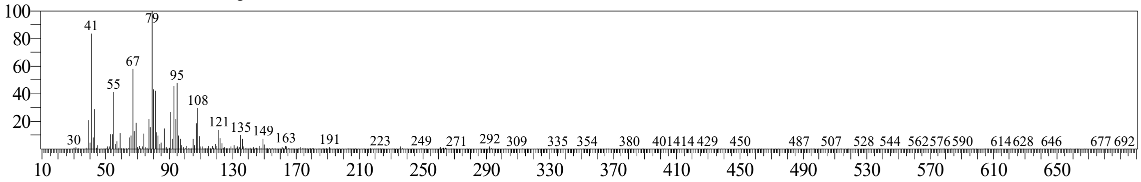

Hit#:1 Entry:112450 Library:NIST11.lib
SI:91 Formula:C19H32O2 CAS:301-00-8 MolWeight:292 RetIndex:2101
CompName:9,12,15-Octadecatrienoic acid, methyl ester, (Z,Z,Z)- $$ Linolenic acid, methyl ester $$ Methyl all-cis-9,12,15-octadecatrienoate $$ Methyl linolen

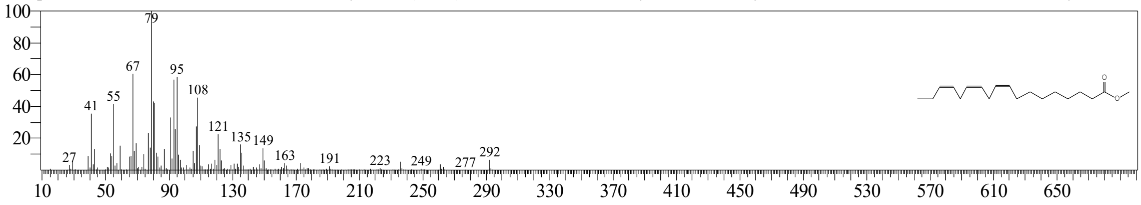

Hit#:2 Entry:101390 Library:NIST11.lib
SI:91 Formula:C18H30O2 CAS:463-40-1 MolWeight:278 RetIndex:2191
CompName:9,12,15-Octadecatrienoic acid, (Z,Z,Z)- $$ Linolenic acid $$ .alpha.-Linolenic acid $$ All-cis-9,12,15-Octadecatrienoic acid $$ cis,cis,cis-9,12,15-(

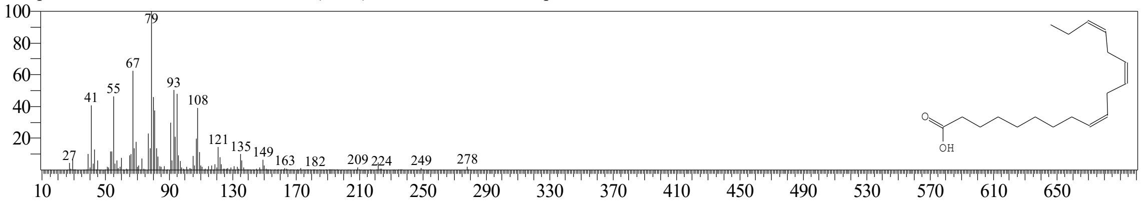

Hit#:3 Entry:132823 Library:NIST11.lib
SI:91 Formula:C21H34O2 CAS:59149-01-8 MolWeight:318 RetIndex:2308
CompName:Methyl (Z)-5,11,14,17-eicosatetraenoate $$ Methyl (5Z,11Z,14Z,17Z)-5,11,14,17-icosatetraenoate # $$

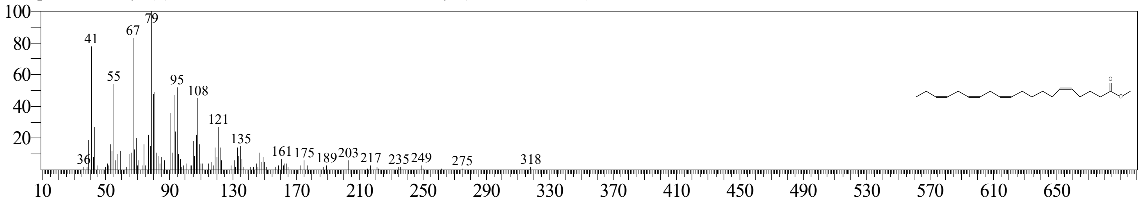

Hit#:4 Entry:90440 Library:NIST11.lib
SI:91 Formula:C17H28O2 CAS:56554-30-4 MolWeight:264 RetIndex:1902
CompName:7,10,13-Hexadecatrienoic acid, methyl ester $$ Methyl (7E,10E,13E)-7,10,13-hexadecatrienoate # $$

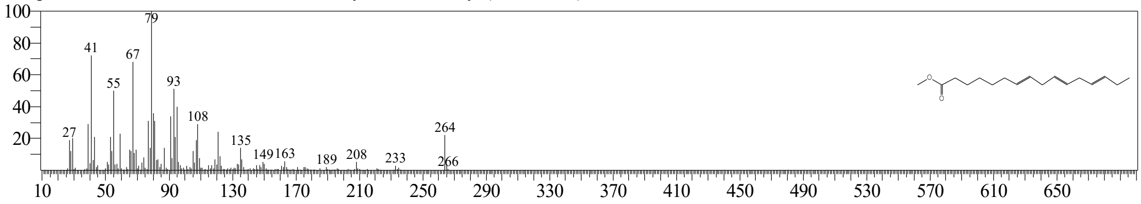

Hit#:5 Entry:90503 Library:NIST11.lib
SI:91 Formula:C18H32O CAS:506-44-5 MolWeight:264 RetIndex:2077
CompName:9,12,15-Octadecatrien-1-ol, (Z,Z,Z)- $$ (9E,12E,15E)-9,12,15-Octadecatrien-1-ol # $$ (Z,Z,Z)-9,12,15-Octadecatrien-1-ol $$

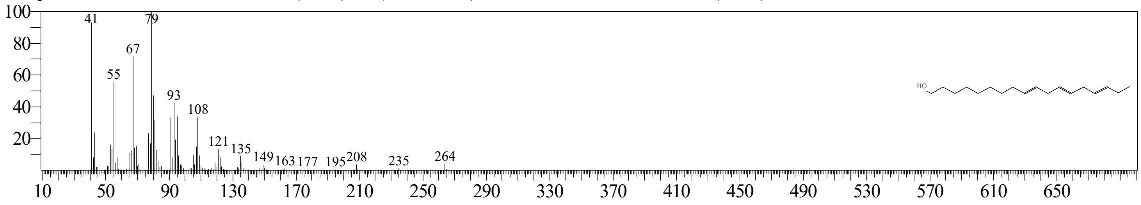

<< Target >>
Line#:123 R.Time:15.010(Scan#:2203) MassPeaks:384
RawMode:Averaged 15.005-15.015(2202-2204) BasePeak:71.05(269771)
BG Mode:Calc. from Peak Group 1 - Event 1 Scan

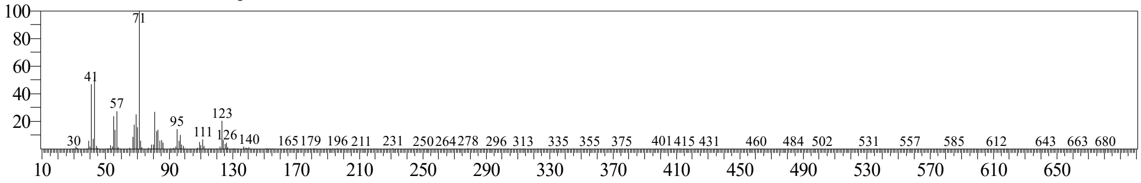

Hit#:1 Entry:115516 Library:NIST11.lib
SI:94 Formula:C20H40O CAS:150-86-7 MolWeight:296 RetIndex:2045
CompName:Phytol $$ 2-Hexadecen-1-ol, 3,7,11,15-tetramethyl-, [R-[R*,R*-(E)]]- $$ trans-Phytol $$ 3,7,11,15-Tetramethyl-2-hexadecen-1-ol-, (2E,7R,11R)- $

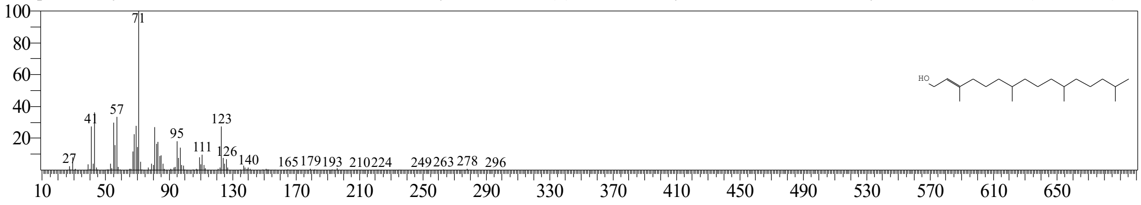

Hit#:2 Entry:84384 Library:NIST11.lib
SI:86 Formula:C16H32O2 CAS:0-00-0 MolWeight:256 RetIndex:1936
CompName:13-Methylpentadec-14-ene-1,13-diol $$ 13-Methyl-14-pentadecene-1,13-diol # $$

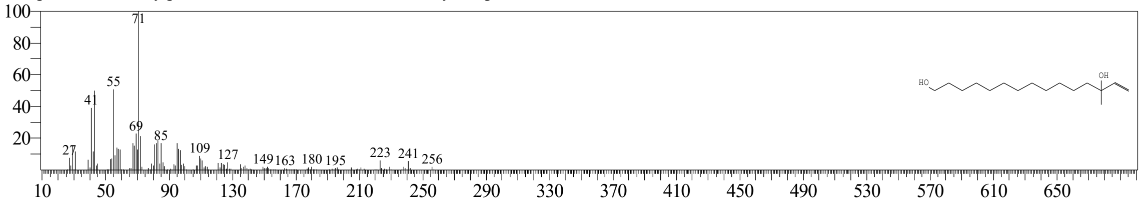

Hit#:3 Entry:18670 Library:NIST11.lib
SI:86 Formula:C10H20O CAS:23283-97-8 MolWeight:156 RetIndex:1164
CompName:Cyclohexanol, 5-methyl-2-(1-methylethyl)-, [1S-(1.alpha.,2.beta.,5.beta.)]- $$ (1S,2R,5R)-(+)-Isomenthol $$ 2-Isopropyl-5-methylcyclohexanol # $

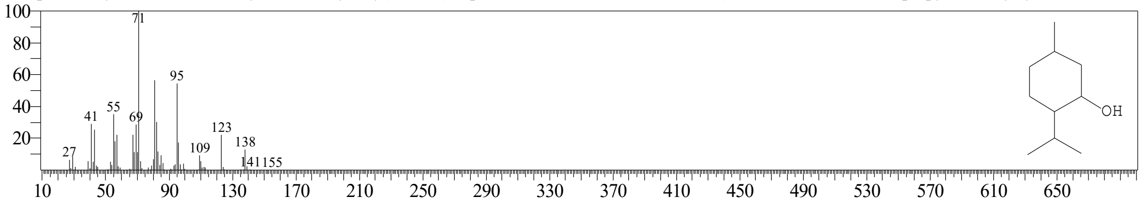

Hit#:4 Entry:18678 Library:NIST11.lib
SI:86 Formula:C10H20O CAS:491-01-0 MolWeight:156 RetIndex:1164
CompName:Cyclohexanol, 5-methyl-2-(1-methylethyl)-, (1.alpha.,2.alpha.,5.beta.)- $$ Menthol, trans-1,3,trans-1,4- $$ Neo-Menthol $$ Neomenthol $$ 2-Isopr

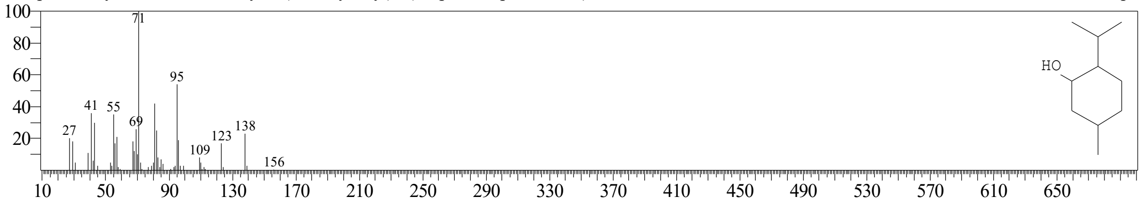

Hit#:5 Entry:17556 Library:NIST11.lib
SI:85 Formula:C10H18O CAS:22460-59-9 MolWeight:154 RetIndex:1071
CompName:1,7-Octadien-3-ol, 2,6-dimethyl- $$ 3,7-Dimethyl-1, 7-octadien-6-ol $$ 2,6-Dimethyl-1,7-octadien-3-ol $$

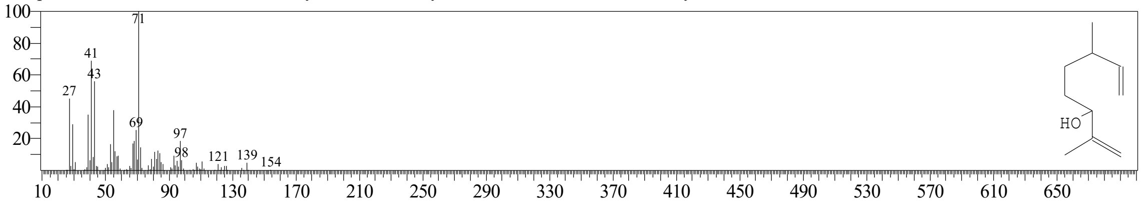

Qualitative Analysis Report

<< Target >>
Line#:124 R.Time:15.065(Scan#:2214) MassPeaks:373
RawMode:Averaged 15.060-15.070(2213-2215) BasePeak:74.00(94638)
BG Mode:Calc. from Peak Group 1 - Event 1 Scan

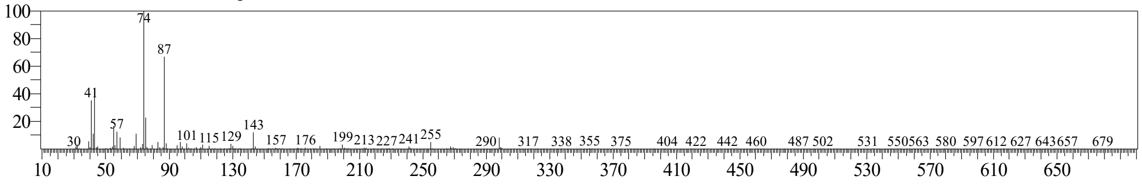

Hit#:1 Entry:106180 Library:NIST11.lib
SI:89 Formula:C18H36O2 CAS:6929-04-0 MolWeight:284 RetIndex:1914
CompName:Hexadecanoic acid, 15-methyl-, methyl ester $$ Methyl isoheptadecanoate $$ Methyl 15-methylhexadecanoate $$

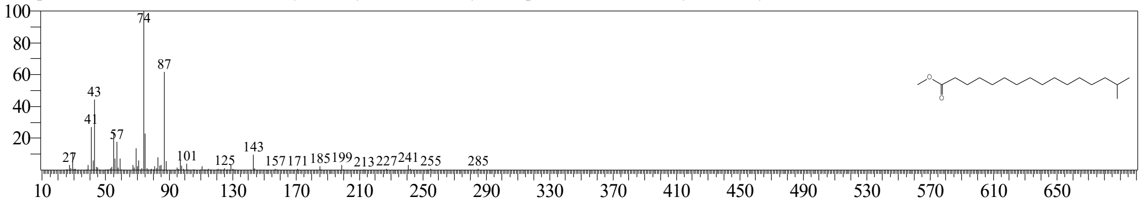

Hit#:2 Entry:95189 Library:NIST11.lib
SI:88 Formula:C17H34O2 CAS:5129-60-2 MolWeight:270 RetIndex:1814
CompName:Pentadecanoic acid, 14-methyl-, methyl ester $$ Methyl 14-methylpentadecanoate # $$ Methyl isohexadecanoate $$

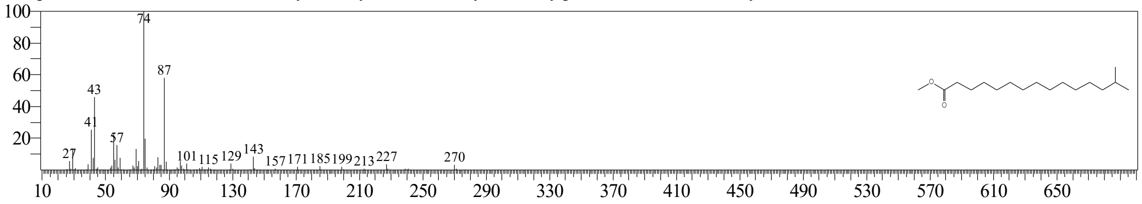

Hit#:3 Entry:117152 Library:NIST11.lib
SI:87 Formula:C19H38O2 CAS:112-61-8 MolWeight:298 RetIndex:2077
CompName:Methyl stearate $$ Octadecanoic acid, methyl ester $$ Stearic acid, methyl ester $$ n-Octadecanoic acid, methyl ester $$ Kemester 9718 $$ Methyl

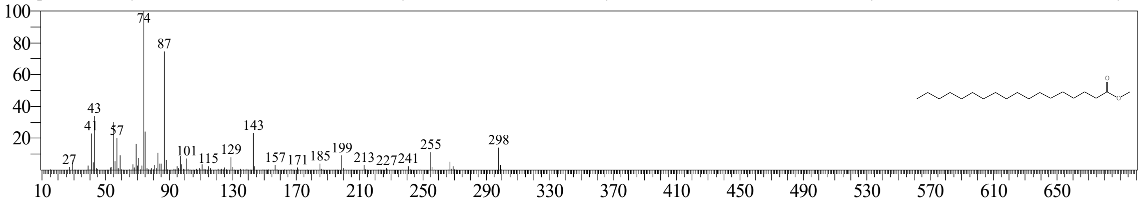

Hit#:4 Entry:73852 Library:NIST11.lib
SI:87 Formula:C15H30O2 CAS:5129-58-8 MolWeight:242 RetIndex:1615
CompName:Tridecanoic acid, 12-methyl-, methyl ester $$ Methyl isomyristate $$ Methyl 12-methyltridecanoate $$

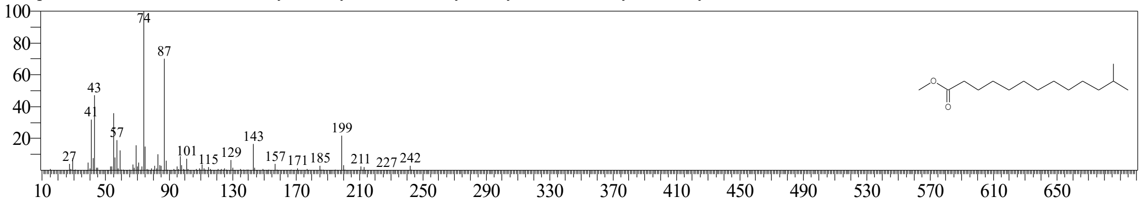

Hit#:5 Entry:106178 Library:NIST11.lib
SI:87 Formula:C18H36O2 CAS:1731-92-6 MolWeight:284 RetIndex:1978
CompName:Heptadecanoic acid, methyl ester $$ Margaric acid methyl ester $$ Methyl heptadecanoate $$ Methyl margarate $$ n-Heptadecanoic acid methyl est

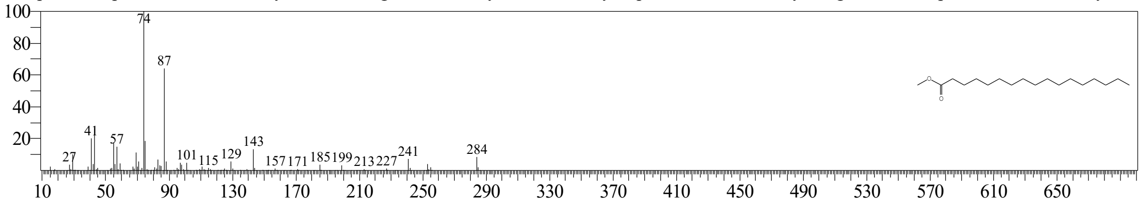

<< Target >>
Line#:125 R.Time:15.255(Scan#:2252) MassPeaks:445
RawMode:Averaged 15.250-15.260(2251-2253) BasePeak:41.10(451954)
BG Mode:Calc. from Peak Group 1 - Event 1 Scan

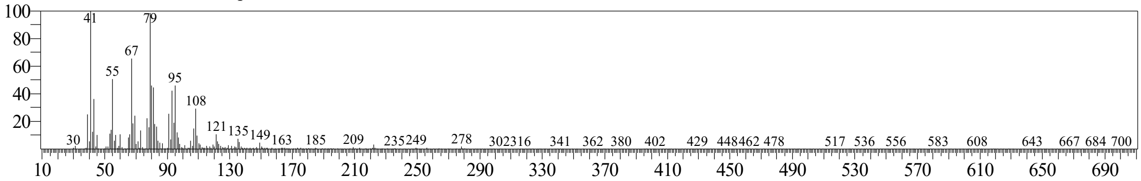

Hit#:1 Entry:101390 Library:NIST11.lib
SI:91 Formula:C18H30O2 CAS:463-40-1 MolWeight:278 RetIndex:2191
CompName:9,12,15-Octadecatrienoic acid, (Z,Z,Z)- $$ Linolenic acid $$ .alpha.-Linolenic acid $$ All-cis-9,12,15-Octadecatrienoic acid $$ cis,cis,cis-9,12,15-(

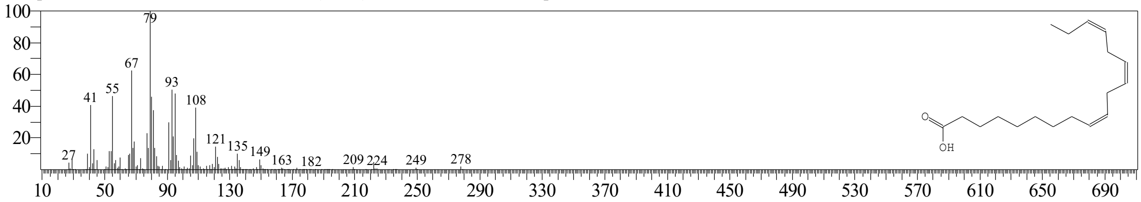

Hit#:2 Entry:90503 Library:NIST11.lib
SI:91 Formula:C18H32O CAS:506-44-5 MolWeight:264 RetIndex:2077
CompName:9,12,15-Octadecatrien-1-ol, (Z,Z,Z)- $$ (9E,12E,15E)-9,12,15-Octadecatrien-1-ol # $$ (Z,Z,Z)-9,12,15-Octadecatrien-1-ol $$

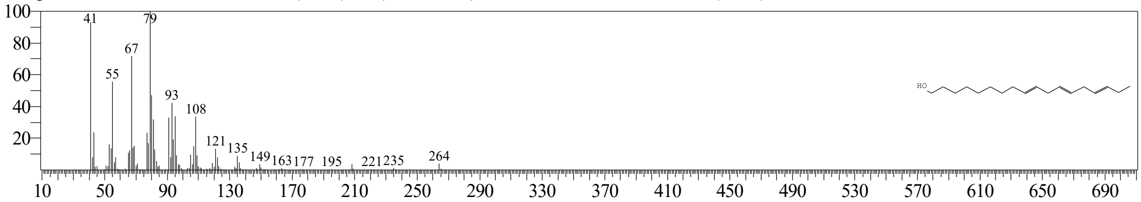

Hit#:3 Entry:67943 Library:NIST11.lib
SI:90 Formula:C16H26O CAS:56797-43-4 MolWeight:234 RetIndex:1824
CompName:cis,cis,cis-7,10,13-Hexadecatrienal $$ (7Z,10Z,13Z)-7,10,13-Hexadecatrienal # $$

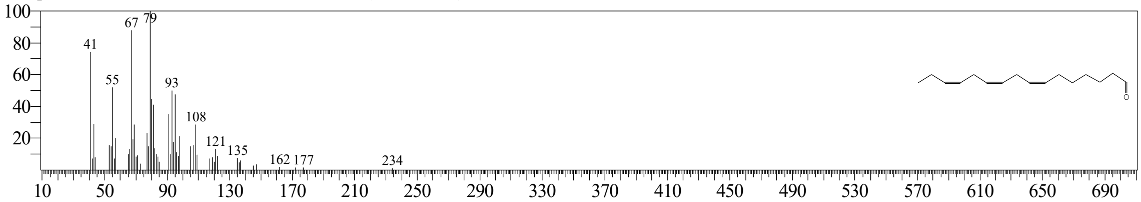

Hit#:4 Entry:123525 Library:NIST11.lib
SI:88 Formula:C20H34O2 CAS:1783-84-2 MolWeight:306 RetIndex:2390
CompName:8,11,14-Eicosatrienoic acid, (Z,Z,Z)- $$ cis-8,11,14-Eicosatrienoic Acid $$ Dihomo-.gamma.-linolenic acid $$ (8E,11E,14E)-8,11,14-Icosatrienoic

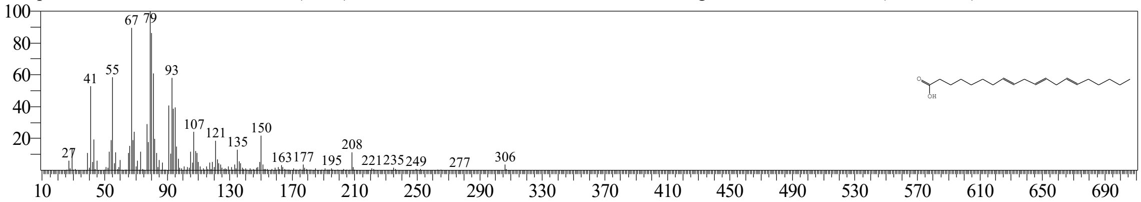

Hit#:5 Entry:101387 Library:NIST11.lib
SI:88 Formula:C18H30O2 CAS:506-26-3 MolWeight:278 RetIndex:2191
CompName:Gamolenic Acid $$ (Z,Z,Z)-6,9,12-Octadecatrienoic acid $$ .gamma.-Linolenic acid $$

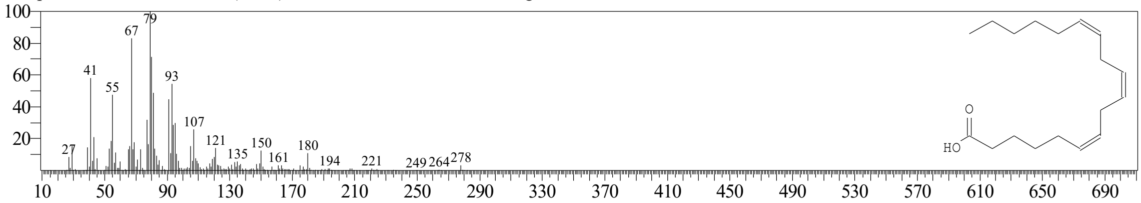

Qualitative Analysis Report

<< Target >>
Line#:126 R.Time:15.370(Scan#:2275) MassPeaks:419
RawMode:Averaged 15.365-15.375(2274-2276) BasePeak:41.05(70208)
BG Mode:Calc. from Peak Group 1 - Event 1 Scan

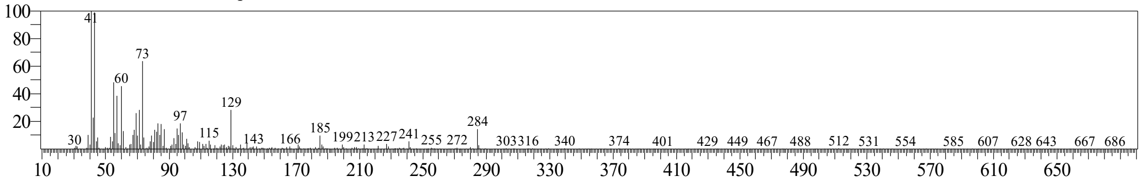

Hit#:1 Entry:169171 Library:NIST11.lib
SI:86 Formula:C22H44O4 CAS:106-11-6 MolWeight:372 RetIndex:2694
CompName:Octadecanoic acid, 2-(2-hydroxyethoxy)ethyl ester $$ Aqua Cera $$ Atlas G 2146 $$ Cerasynt $$ Cerasynt Special $$ Clindrol SDG $$ Diethylene

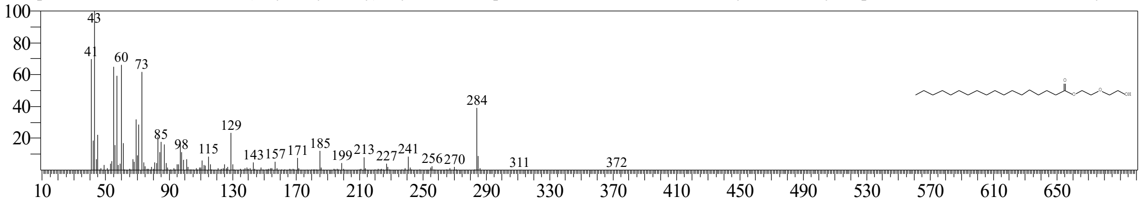

Hit#:2 Entry:84362 Library:NIST11.lib
SI:84 Formula:C16H32O2 CAS:57-10-3 MolWeight:256 RetIndex:1968
CompName:n-Hexadecanoic acid $$ Hexadecanoic acid $$ n-Hexadecoic acid $$ Palmitic acid $$ Pentadecanecarboxylic acid $$ 1-Pentadecanecarboxylic acid

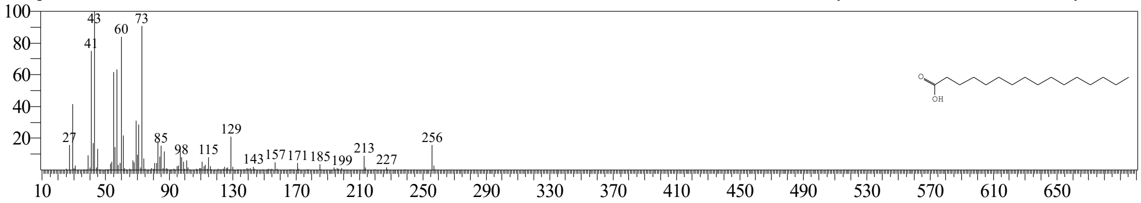

Hit#:3 Entry:128151 Library:NIST11.lib
SI:84 Formula:C20H40O2 CAS:506-30-9 MolWeight:312 RetIndex:2366
CompName:Eicosanoic acid $$ Arachic acid $$ Arachidic acid $$ Icosanoic acid $$ n-Eicosanoic acid $$ Arachidic acid (synthetic) $$

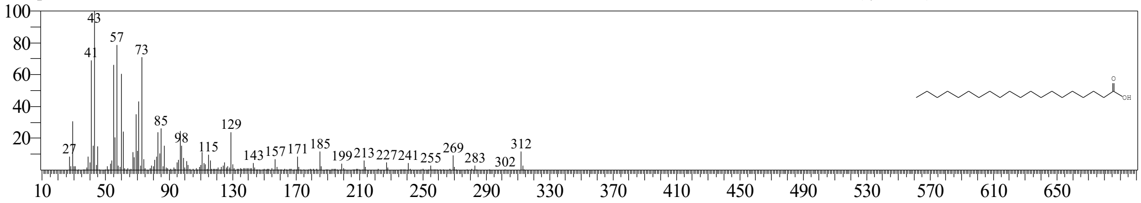

Hit#:4 Entry:117133 Library:NIST11.lib
SI:83 Formula:C19H38O2 CAS:646-30-0 MolWeight:298 RetIndex:2266
CompName:Nonadecanoic acid $$ n-Nonadecanoic acid $$

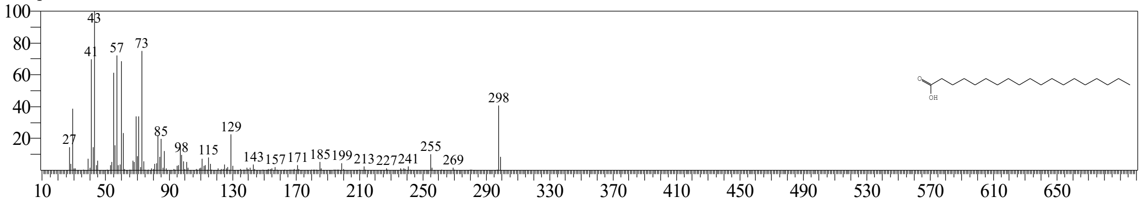

Hit#:5 Entry:106158 Library:NIST11.lib
SI:83 Formula:C18H36O2 CAS:57-11-4 MolWeight:284 RetIndex:2167
CompName:Octadecanoic acid $$ Stearic acid $$ n-Octadecanoic acid $$ Humko Industrene R $$ Hydrofol Acid 150 $$ Hystrene S-97 $$ Hystrene T-70 $$ Hys

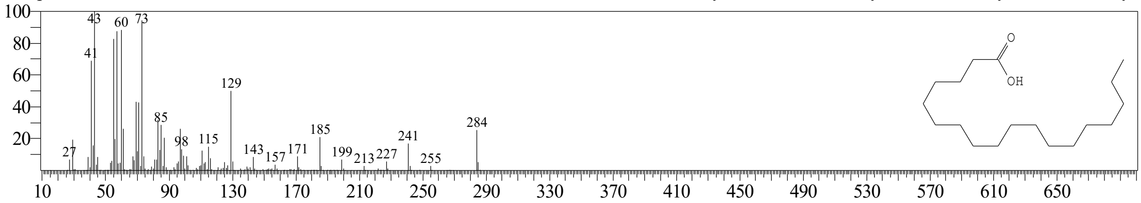

<< Target >>
Line#:127 R.Time:15.455(Scan#:2292) MassPeaks:397
RawMode:Averaged 15.450-15.460(2291-2293) BasePeak:56.05(235891)
BG Mode:Calc. from Peak Group 1 - Event 1 Scan

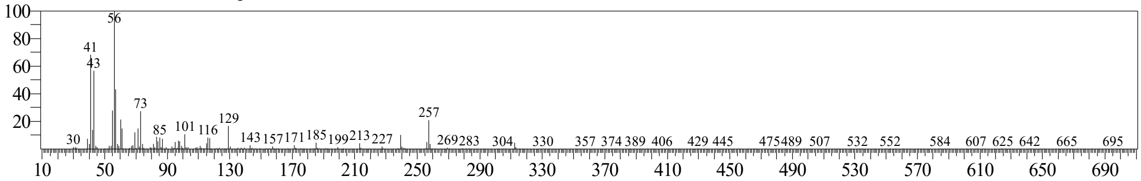

Hit#:1 Entry:128159 Library:NIST11.lib
SI:91 Formula:C20H40O2 CAS:111-06-8 MolWeight:312 RetIndex:2177
CompName:Hexadecanoic acid, butyl ester $$ Palmitic acid, butyl ester $$ n-Butyl hexadecanoate $$ n-Butyl palmitate $$ Butyl palmitate $$ Butyl ester of hex

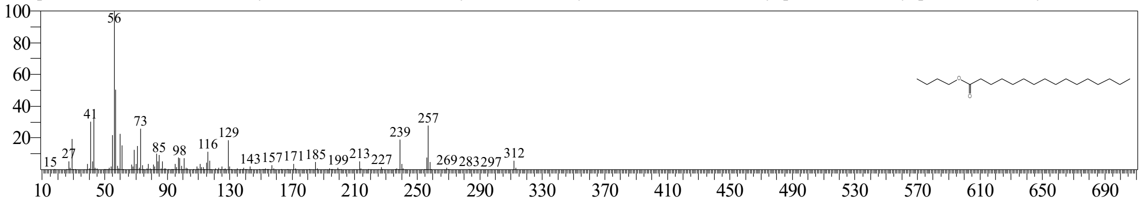

Hit#:2 Entry:128158 Library:NIST11.lib
SI:90 Formula:C20H40O2 CAS:31158-91-5 MolWeight:312 RetIndex:2092
CompName:Hexadecanoic acid, 1,1-dimethylethyl ester $$ tert-Butyl palmitate # $$

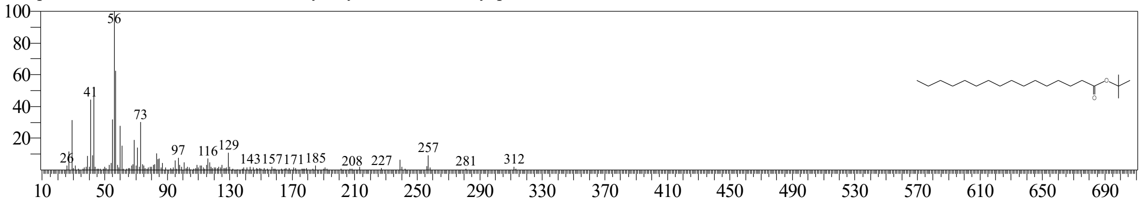

Hit#:3 Entry:106162 Library:NIST11.lib
SI:88 Formula:C18H36O2 CAS:110-36-1 MolWeight:284 RetIndex:1978
CompName:Butyl myristate $$ n-Butyl myristate $$ Myristic acid n-butyl ester $$ Tetradecanoic acid, butyl ester $$ Bumyr $$ Myristic acid, butyl ester $$ Buty

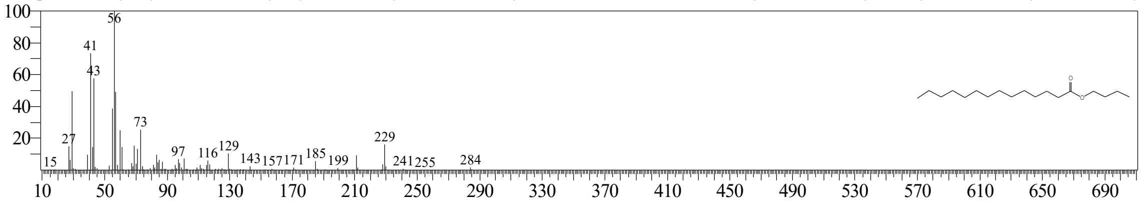

Hit#:4 Entry:128162 Library:NIST11.lib
SI:88 Formula:C20H40O2 CAS:110-34-9 MolWeight:312 RetIndex:2112
CompName:Hexadecanoic acid, 2-methylpropyl ester $$ 2-methylpropyl hexadecanoate $$ isobutyl hexadecanoate $$

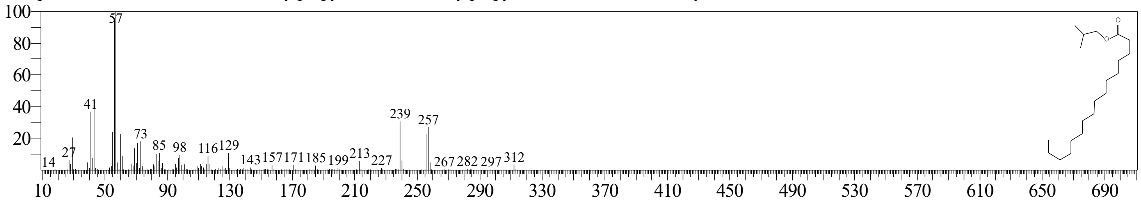

Hit#:5 Entry:63796 Library:NIST11.lib
SI:81 Formula:C14H28O2 CAS:30673-36-0 MolWeight:228 RetIndex:1580
CompName:Butyl caprate $$ n-Capric acid n-butyl ester $$ Decanoic acid, butyl ester $$ Butyl decanoate $$ n-Butyl n-decanoate $$ n-Butyl decanoate $$

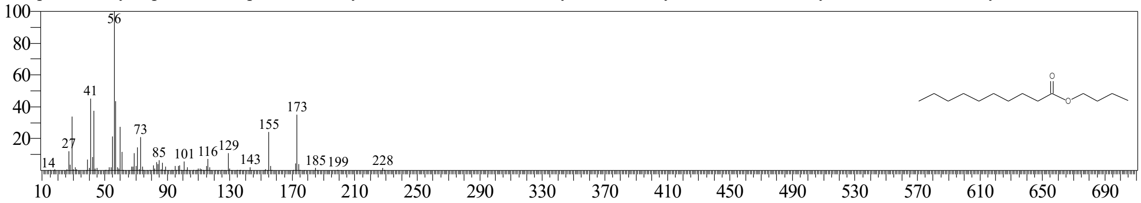

<< Target >>
Line#:128 R.Time:15.510(Scan#:2303) MassPeaks:386
RawMode:Averaged 15.505-15.515(2302-2304) BasePeak:43.05(511596)
BG Mode:Calc. from Peak Group 1 - Event 1 Scan

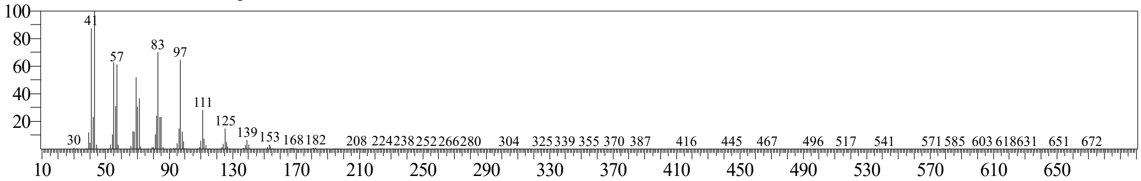

Hit#:1 Entry:81317 Library:NIST11.lib
SI:95 Formula:C17H32O CAS:0-00-0 MolWeight:252 RetIndex:1907
CompName:E-15-Heptadecenal $$ (15E)-15-Heptadecenal # $$

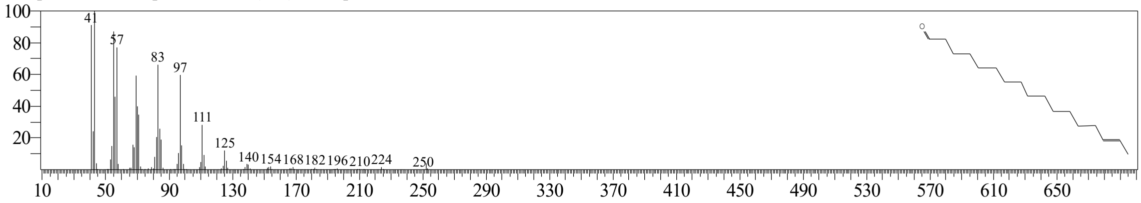

Hit#:2 Entry:70935 Library:NIST11.lib
SI:94 Formula:C17H34 CAS:6765-39-5 MolWeight:238 RetIndex:1701
CompName:1-Heptadecene $$ Hexahydroaplotaxene $$ Heptadec-1-ene $$ NSC 77132 $$ Heptadecene (Related) $$

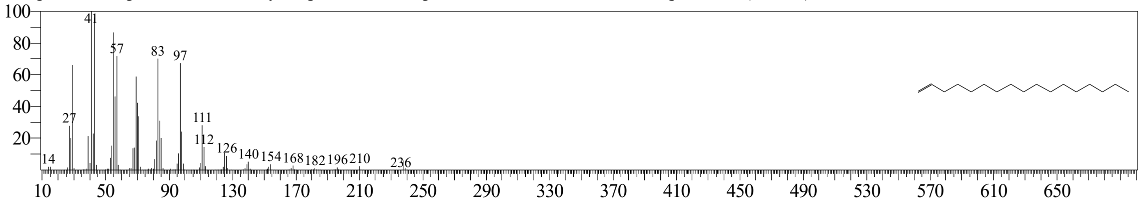

Hit#:3 Entry:70890 Library:NIST11.lib
SI:94 Formula:C16H30O CAS:330207-53-9 MolWeight:238 RetIndex:1808
CompName:E-14-Hexadecenal $$ (14E)-14-Hexadecenal $$ 14-Hexadecenal, (E)- $$

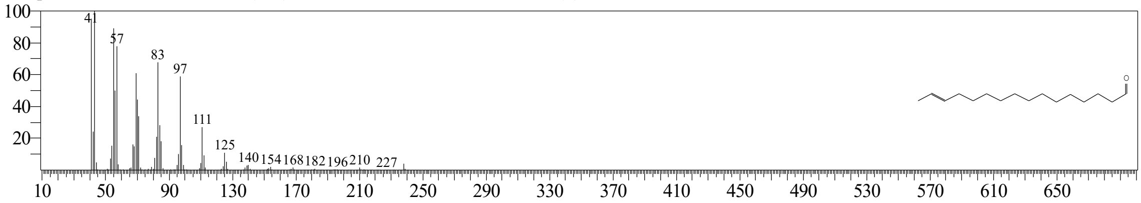

Hit#:4 Entry:81347 Library:NIST11.lib
SI:94 Formula:C18H36 CAS:112-88-9 MolWeight:252 RetIndex:1801
CompName:1-Octadecene $$ .alpha.-Octadecene $$ Octadecylene, .alpha.- $$ Neodene 18 $$ Octadec-1-ene $$ Octadecene-1 $$ Octadeca-1-ene $$

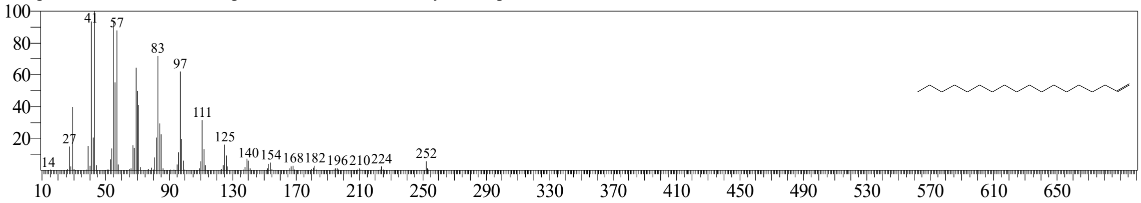

Hit#:5 Entry:114020 Library:NIST11.lib
SI:94 Formula:C21H42 CAS:95008-11-0 MolWeight:294 RetIndex:2117
CompName:10-Heneicosene (c,t) $$ (10E)-10-Henicosene # $$

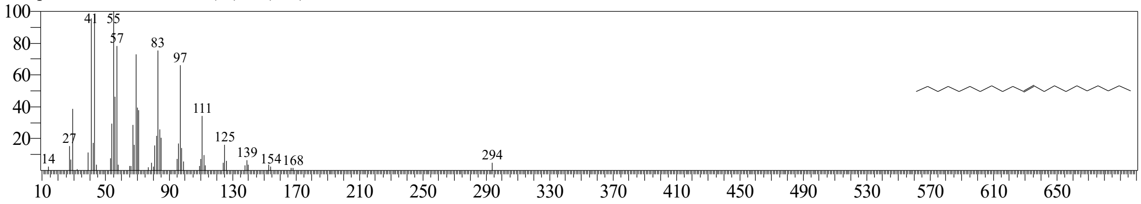

Qualitative Analysis Report

<< Target >>
Line#:129 R.Time:15.605(Scan#:2322) MassPeaks:322
RawMode:Averaged 15.600-15.610(2321-2323) BasePeak:70.05(15273)
BG Mode:Calc. from Peak Group 1 - Event 1 Scan

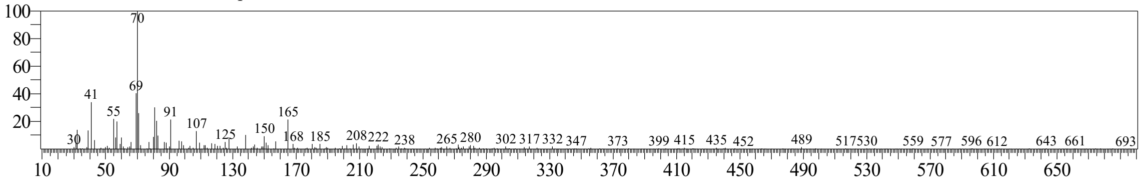

Hit#:1 Entry:24941 Library:NIST11.lib
SI:67 Formula:C12H24 CAS:57024-90-5 MolWeight:168 RetIndex:1199
CompName:2-Undecene, 3-methyl-, (Z)- $$ (2Z)-3-Methyl-2-undecene # $$

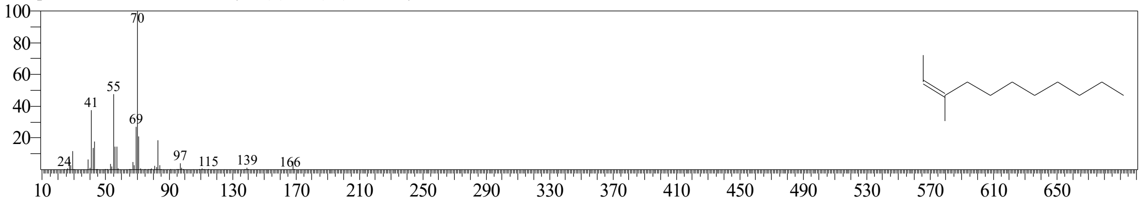

Hit#:2 Entry:42736 Library:NIST11.lib
SI:66 Formula:C12H22O2 CAS:688-84-6 MolWeight:198 RetIndex:1284
CompName:2-Ethylhexyl methacrylate $$ Octyl methacrylate $$ Methacrylic acid 2-ethylhexyl ester $$ 2-Propenoic acid, 2-methyl-, 2-ethylhexyl ester $$ 2-Eth

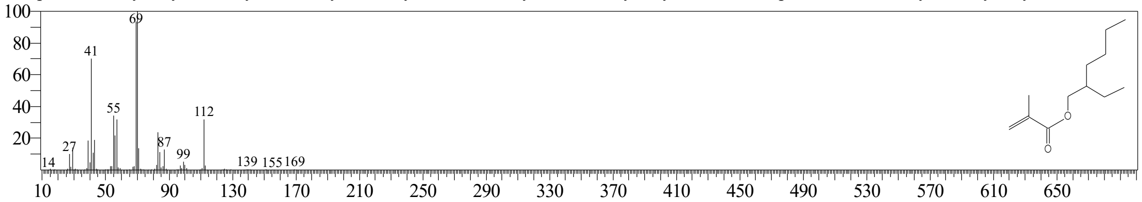

Hit#:3 Entry:24937 Library:NIST11.lib
SI:66 Formula:C12H24 CAS:74630-67-4 MolWeight:168 RetIndex:1158
CompName:5-Undecene, 3-methyl-, (E)- $$ (5E)-3-Methyl-5-undecene # $$

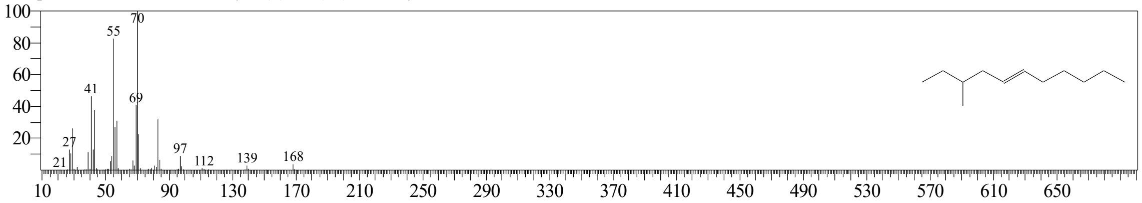

Hit#:4 Entry:17744 Library:NIST11.lib
SI:66 Formula:C11H22 CAS:31797-64-5 MolWeight:154 RetIndex:1094
CompName:Cyclohexane, (1,1-dimethylpropyl)- $$ Cyclohexane, tert-pentyl- $$ tert-Pentylcyclohexane $$ 2-Cyclohexyl-2-methylbutane $$ 2-Methyl-2-cycloh

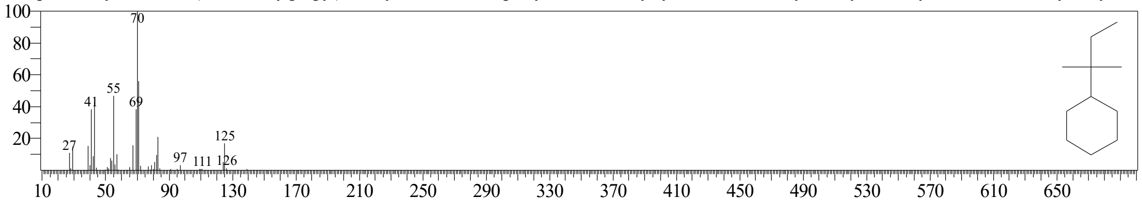

Hit#:5 Entry:24938 Library:NIST11.lib
SI:66 Formula:C12H24 CAS:74630-47-0 MolWeight:168 RetIndex:1199
CompName:2-Undecene, 3-methyl-, (E)- $$ (2E)-3-Methyl-2-undecene # $$

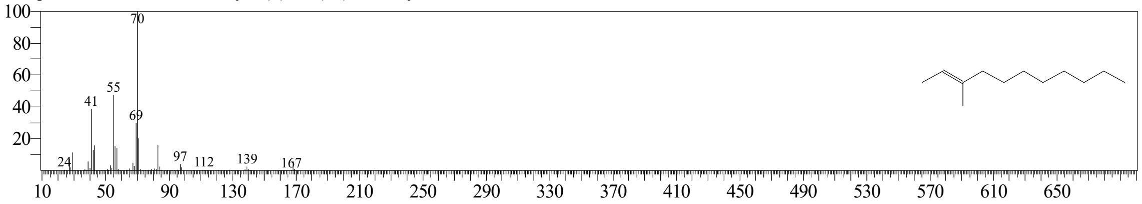

<< Target >>
Line#:130 R.Time:15.690(Scan#:2339) MassPeaks:378
RawMode:Averaged 15.685-15.695(2338-2340) BasePeak:41.00(13190)
BG Mode:Calc. from Peak Group 1 - Event 1 Scan

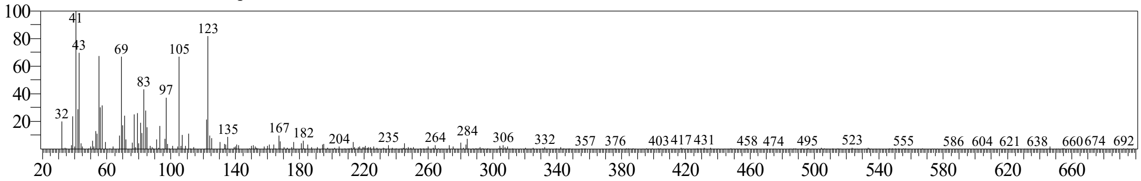

Hit#:1 Entry:143083 Library:NIST11.lib
SI:80 Formula:C19H37ClO2 CAS:0-00-0 MolWeight:332 RetIndex:2302
CompName:4-Chlorobutyric acid, pentadecyl ester

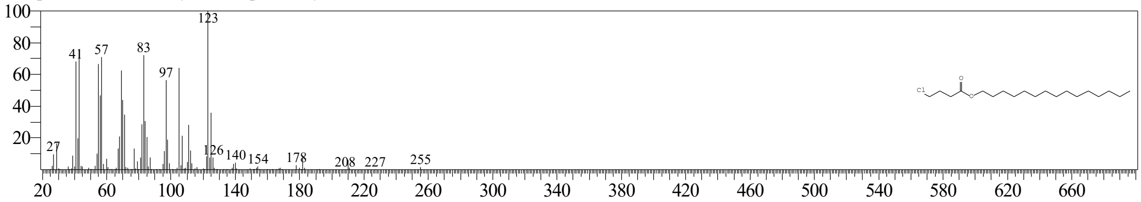

Hit#:2 Entry:170256 Library:NIST11.lib
SI:77 Formula:C22H43ClO2 CAS:0-00-0 MolWeight:374 RetIndex:2601
CompName:4-Chlorobutyric acid, octadecyl ester

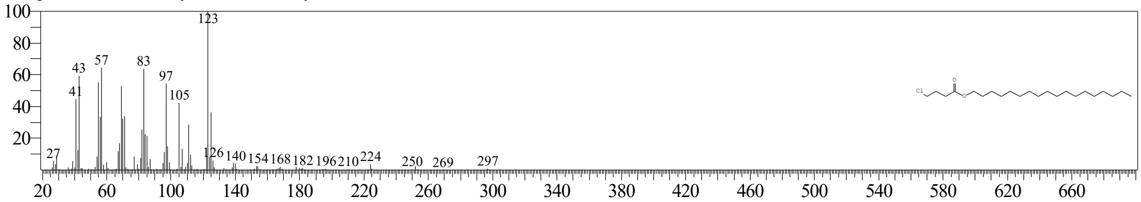

Hit#:3 Entry:133739 Library:NIST11.lib
SI:76 Formula:C15H29BrO2 CAS:0-00-0 MolWeight:320 RetIndex:1912
CompName:Bromoacetic acid, 3-tridecyl ester $$ 1-Ethylundecyl bromoacetate # $$

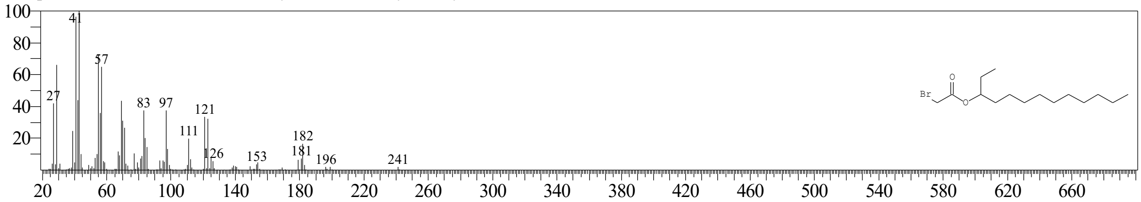

Hit#:4 Entry:91766 Library:NIST11.lib
SI:76 Formula:C16H26O3 CAS:19780-11-1 MolWeight:266 RetIndex:2159
CompName:2-Dodecen-1-yl(-)succinic anhydride $$ 2,5-Furandione, 3-dodecenyl- $$ n-Dodecenylsuccinic anhydride $$ Dodecenyl succinic anhydride $$ 2,5-I

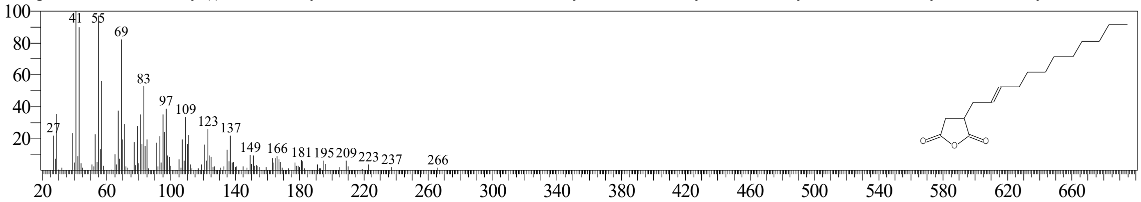

Hit#:5 Entry:133740 Library:NIST11.lib
SI:76 Formula:C15H29BrO2 CAS:0-00-0 MolWeight:320 RetIndex:1912
CompName:Bromoacetic acid, 4-tridecyl ester $$ 1-Propyldecyl bromoacetate # $$

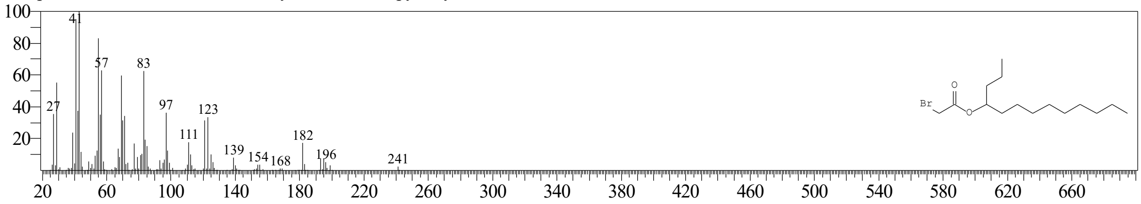

<< Target >>
Line#:131 R.Time:15.785(Scan#:2358) MassPeaks:374
RawMode:Averaged 15.780-15.790(2357-2359) BasePeak:57.05(5224)
BG Mode:Calc. from Peak Group 1 - Event 1 Scan

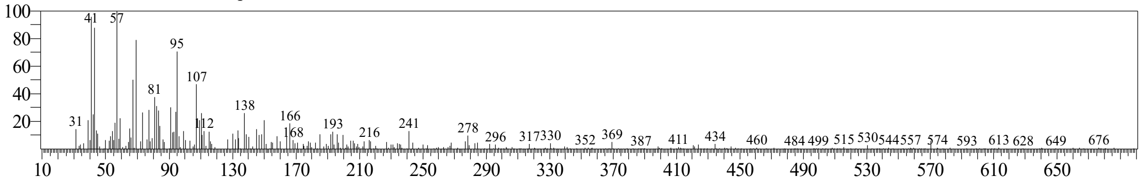

Hit#:1 Entry:42684 Library:NIST11.lib
SI:67 Formula:C12H22O2 CAS:150-84-5 MolWeight:198 RetIndex:1302
CompName:6-Octen-1-ol, 3,7-dimethyl-, acetate $$ Citronellol acetate $$ Citronellyl acetate $$ Natural rhodinol, acetylated $$ 3,7-Dimethyl-6-octenyl acetate $

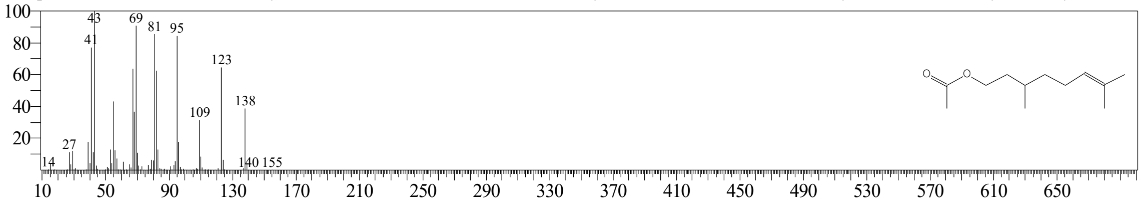

Hit#:2 Entry:17239 Library:NIST11.lib
SI:67 Formula:C9H14O2 CAS:3753-38-6 MolWeight:154 RetIndex:1186
CompName:Bicyclo[2.1.1]hexane-1-carboxylic acid, 5,5-dimethyl- $$ 5,5-Dimethylbicyclo[2.1.1]hexane-1-carboxylic acid # $$

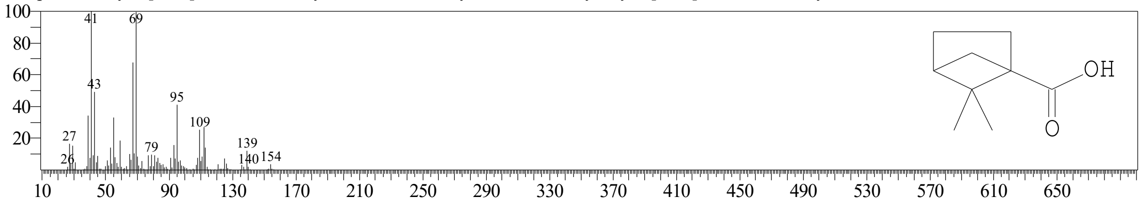

Hit#:3 Entry:31675 Library:NIST11.lib
SI:67 Formula:C12H20O CAS:762-26-5 MolWeight:180 RetIndex:1373
CompName:4,8-Decadienal, 5,9-dimethyl-

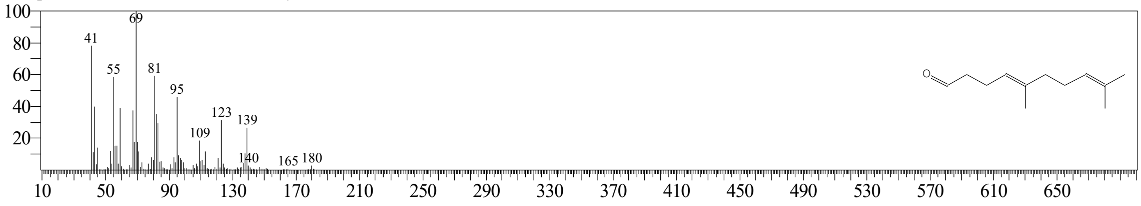

Hit#:4 Entry:32757 Library:NIST11.lib
SI:67 Formula:C11H18O2 CAS:64810-32-8 MolWeight:182 RetIndex:1262
CompName:Ethanone, 1-[3-methyl-3-(4-methyl-3-pentenyl)oxiranyl]- $$ 3,4-Anhydro-1,5-dideoxy-4-(4-methyl-3-pentenyl)pent-2-ulose # $$

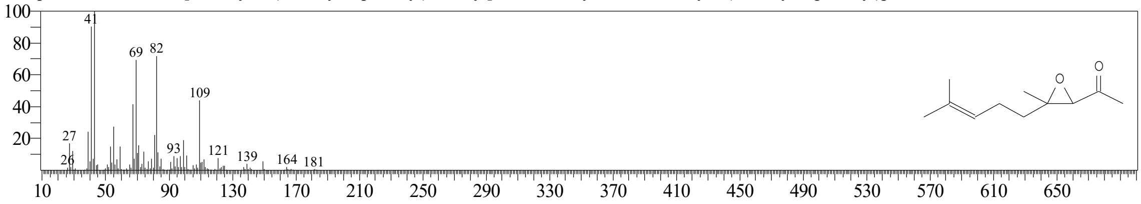

Hit#:5 Entry:18613 Library:NIST11.lib
SI:67 Formula:C10H20O CAS:4194-71-2 MolWeight:156 RetIndex:1266
CompName:2-Decen-1-ol, (Z)- $$ (2Z)-2-Decen-1-ol # $$ cis-2-Decen-1-ol $$ (Z)-2-Decen-1-ol $$

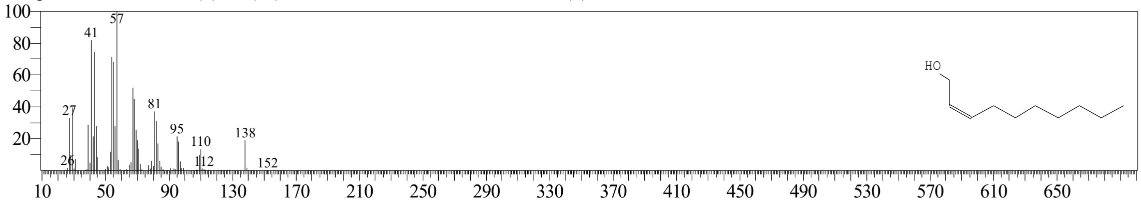

<< Target >>
Line#:132 R.Time:15.885(Scan#:2378) MassPeaks:358
RawMode:Averaged 15.880-15.890(2377-2379) BasePeak:81.00(3750)
BG Mode:Calc. from Peak Group 1 - Event 1 Scan

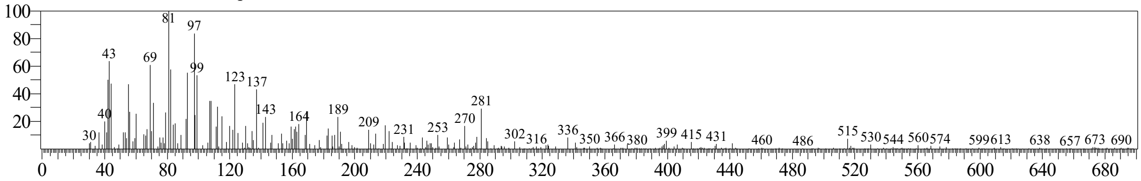

Hit#:1 Entry:17456 Library:NIST11.lib
SI:60 Formula:C10H18O CAS:90026-62-3 MolWeight:154 RetIndex:1125
CompName:Bicyclo[4.1.0]heptan-2-ol, 3,7,7-trimethyl-, (1.alpha.,2.alpha.,3.beta.,6.alpha.)- $$ 3,7,7-Trimethylbicyclo[4.1.0]heptan-2-ol # $$

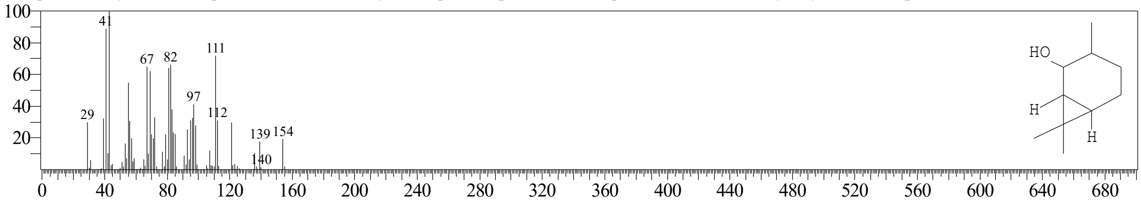

Hit#:2 Entry:144988 Library:NIST11.lib
SI:58 Formula:C23H42O CAS:0-00-0 MolWeight:334 RetIndex:2539
CompName:1-Heptadec-1-ynyl-cyclohexanol $$ 1-(1-Heptadecynyl)cyclohexanol # $$

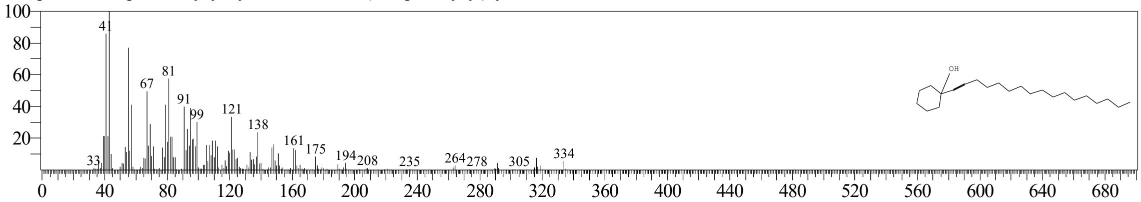

Hit#:3 Entry:79748 Library:NIST11.lib
SI:58 Formula:C16H26O2 CAS:0-00-0 MolWeight:250 RetIndex:1752
CompName:Formic acid, 3,7,11-trimethyl-1,6,10-dodecatrien-3-yl ester $$ (4E)-1,5,9-Trimethyl-1-vinyl-4,8-decadienyl formate # $$

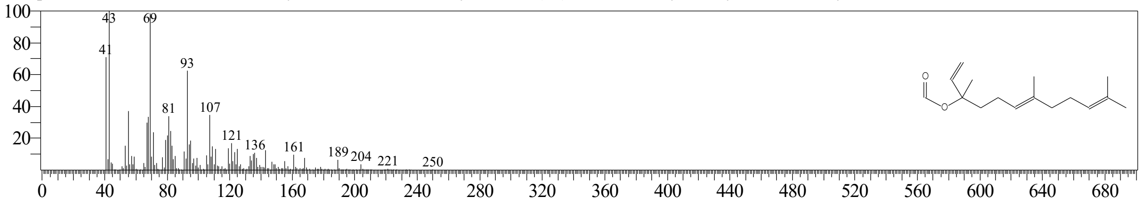

Hit#:4 Entry:11278 Library:NIST11.lib
SI:57 Formula:C9H16O CAS:5432-85-9 MolWeight:140 RetIndex:1087
CompName:4-Isopropylcyclohexanone $$ Cyclohexanone, 4-(1-methylethyl)- $$ Cyclohexanone, 4-isopropyl- $$

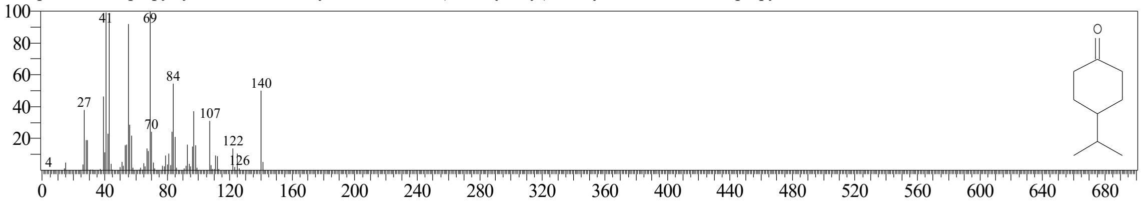

Hit#:5 Entry:102888 Library:NIST11.lib
SI:57 Formula:C19H36O CAS:0-00-0 MolWeight:280 RetIndex:2104
CompName:12-Methyl-E,E-2,13-octadecadien-1-ol $$ (2E,15Z)-14-Methyl-2,15-octadecadien-1-ol # $$

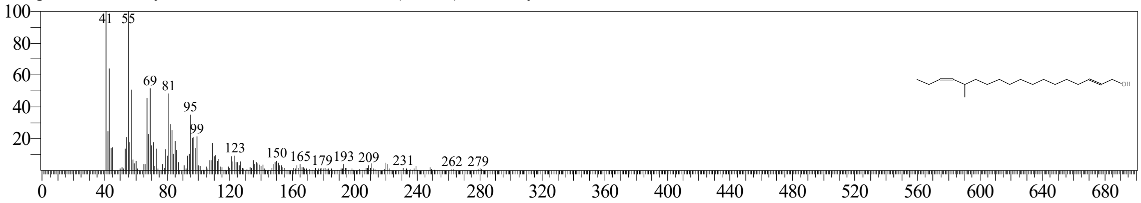

<< Target >>
Line#:133 R.Time:15.955(Scan#:2392) MassPeaks:348
RawMode:Averaged 15.950-15.960(2391-2393) BasePeak:43.05(5696)
BG Mode:Calc. from Peak Group 1 - Event 1 Scan

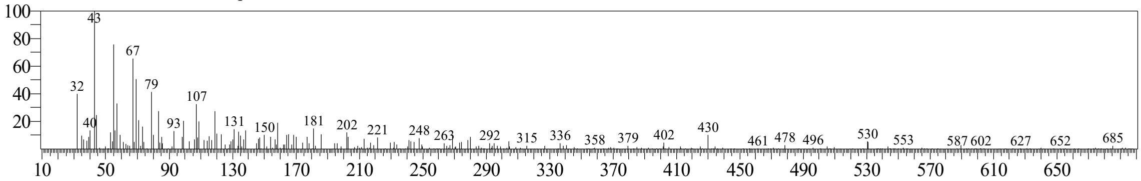

Hit#:1 Entry:187456 Library:NIST11.lib
SI:58 Formula:C27H42O3 CAS:0-00-0 MolWeight:414 RetIndex:2956
CompName:Pseduosarsasapogenin-5,20-dien $$ Furosta-5,20(22)-dien-3,26-diol # $$

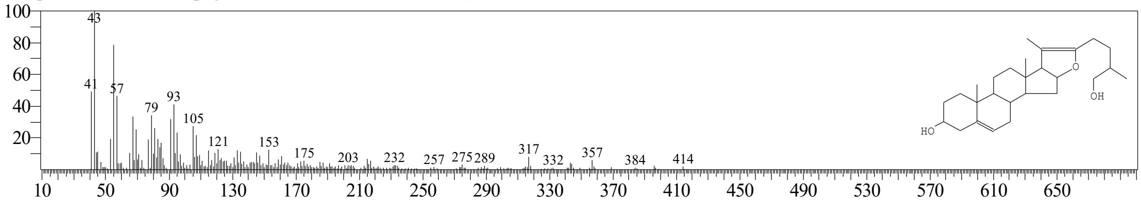

Hit#:2 Entry:165441 Library:NIST11.lib
SI:58 Formula:C22H38O4 CAS:0-00-0 MolWeight:366 RetIndex:2582
CompName:PGH1, methyl ester

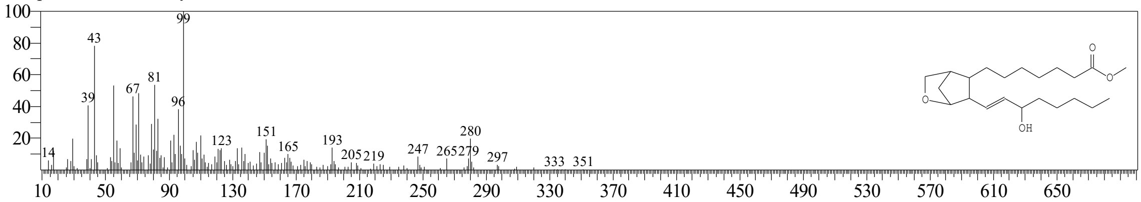

Hit#:3 Entry:208380 Library:NIST11.lib
SI:57 Formula:C28H38O11 CAS:51906-06-0 MolWeight:550 RetIndex:3340
CompName:1b,4a-Epoxy-2H-cyclopenta[3,4]cyclopropa[8,9]cycloundec[1,2-b]oxiren-5(1aH)-one, 2,7,9,10-tetrakis(acetyloxy)decahydro-3,6,8,8,10a-pentameth

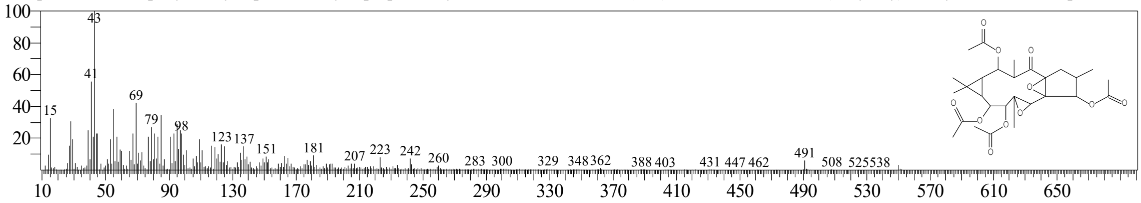

Hit#:4 Entry:144988 Library:NIST11.lib
SI:57 Formula:C23H42O CAS:0-00-0 MolWeight:334 RetIndex:2539
CompName:1-Heptadec-1-ynyl-cyclohexanol $$ 1-(1-Heptadecynyl)cyclohexanol # $$

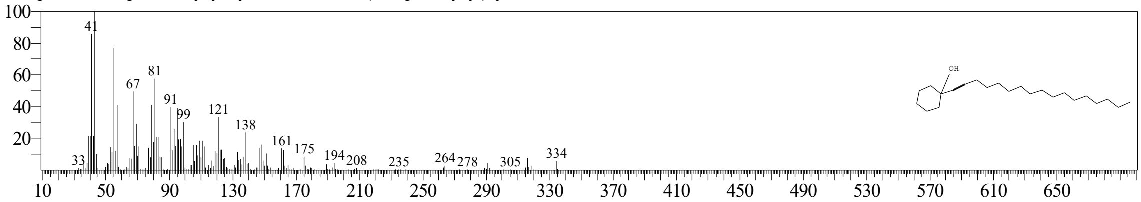

Hit#:5 Entry:207729 Library:NIST11.lib
SI:57 Formula:C37H62O2 CAS:0-00-0 MolWeight:538 RetIndex:3531
CompName:Cholesteryl 3-cyclohexylbutyrate $$ Cholest-5-en-3-yl 3-cyclohexylbutanoate # $$

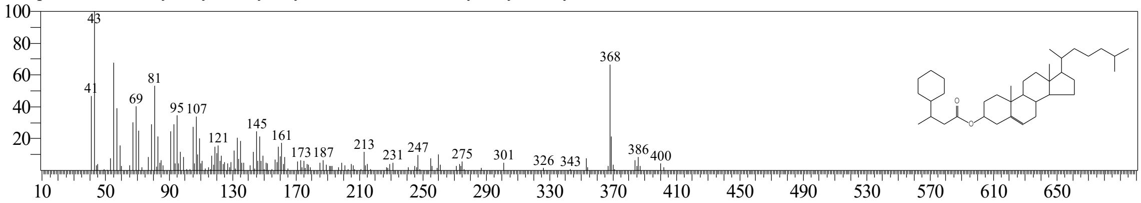

Qualitative Analysis Report

<< Target >>
Line#:134 R.Time:16.030(Scan#:2407) MassPeaks:357
RawMode:Averaged 16.025-16.035(2406-2408) BasePeak:55.10(3024)
BG Mode:Calc. from Peak Group 1 - Event 1 Scan

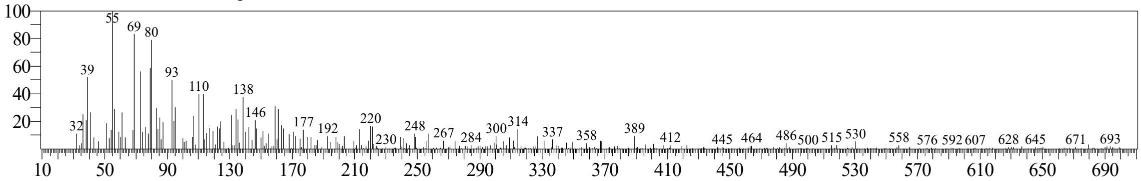

Hit#:1 Entry:179836 Library:NIST11.lib
SI:52 Formula:C24H42O4 CAS:0-00-0 MolWeight:394 RetIndex:2593
CompName:Butanedioic acid, 2,3-bis(8-nonen-1-yl)-, dimethyl ester $$ Dimethyl 2,3-di(8-nonenyl)succinate # $$

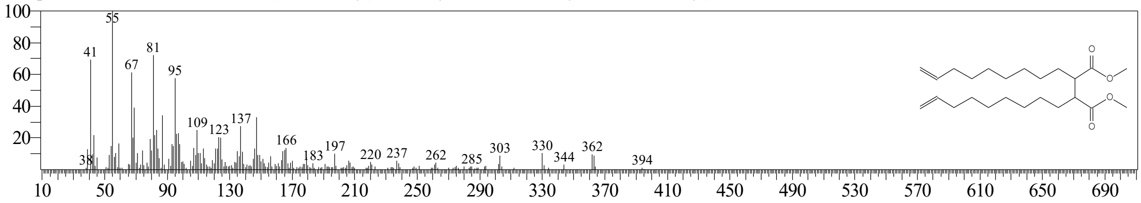

Hit#:2 Entry:30491 Library:NIST11.lib
SI:51 Formula:C13H22 CAS:50746-55-9 MolWeight:178 RetIndex:1187
CompName:Bicyclo[3.1.1]heptane, 2,6,6-trimethyl-3-(2-propenyl)-, (1.alpha.,2.beta.,3.alpha.,5.alpha.)- $$ 3-Allyl-2,6,6-trimethylbicyclo[3.1.1]heptane # $$

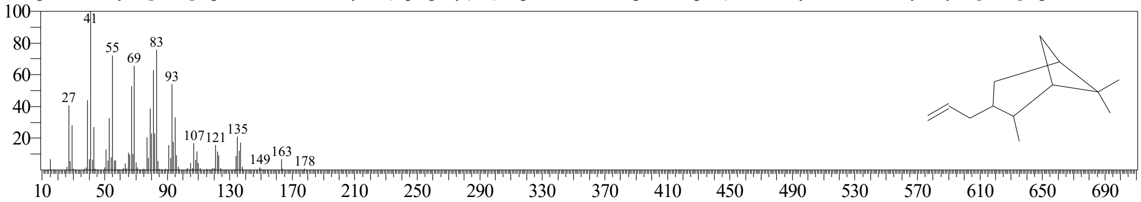

Hit#:3 Entry:59232 Library:NIST11.lib
SI:51 Formula:C14H22O2 CAS:0-00-0 MolWeight:222 RetIndex:1581
CompName:(E)-((3E,6E)-Nona-3,6-dienyl) 2-methylbut-2-enoate

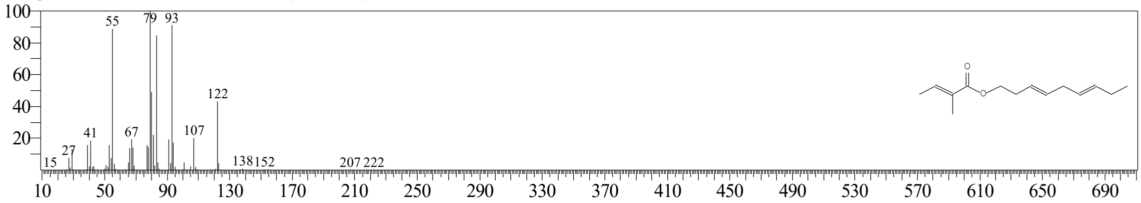

Hit#:4 Entry:57854 Library:NIST11.lib
SI:51 Formula:C16H28 CAS:0-00-0 MolWeight:220 RetIndex:1883
CompName:1,9-Cyclohexadecadiene, (E,E)- $$ 1,9-Cyclohexadecadiene # $$

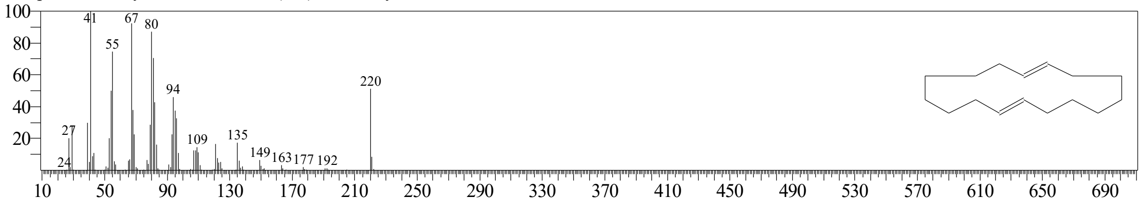

Hit#:5 Entry:57721 Library:NIST11.lib
SI:51 Formula:C15H24O CAS:0-00-0 MolWeight:220 RetIndex:1293
CompName:Calarene epoxide $$ 1,7,7,7b-Tetramethyldecahydrocyclopropa[5,6]naphtho[1,8a-b]oxirene # $$

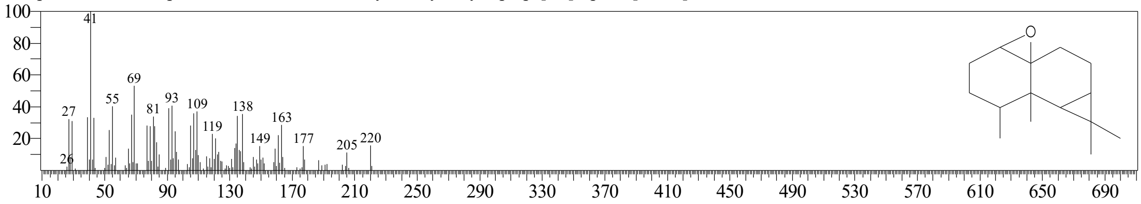

<< Target >>
Line#:135 R.Time:16.105(Scan#:2422) MassPeaks:386
RawMode:Averaged 16.100-16.110(2421-2423) BasePeak:43.10(4066)
BG Mode:Calc. from Peak Group 1 - Event 1 Scan

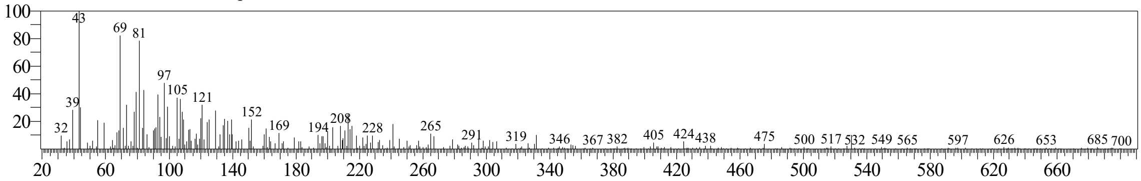

Hit#:1 Entry:123521 Library:NIST11.lib
SI:59 Formula:C20H34O2 CAS:41756-27-8 MolWeight:306 RetIndex:2254
CompName:Sandaracopimar-15-ene-6.beta.,8.beta.-diol $$ Pimar-15-ene-6,8-diol # $$

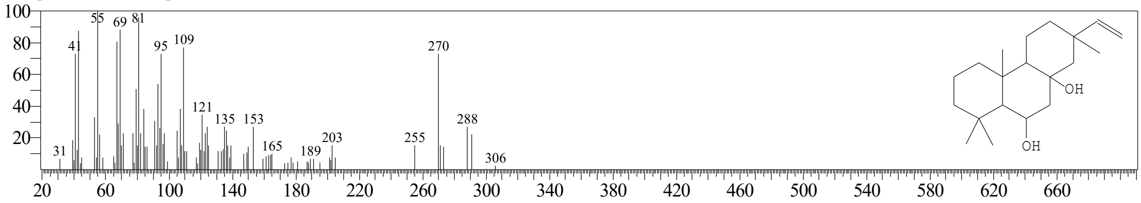

Hit#:2 Entry:102733 Library:NIST11.lib
SI:59 Formula:C17H28O3 CAS:0-00-0 MolWeight:280 RetIndex:2076
CompName:2,6,10-Dodecatriene, 12-acetoxy-6-hydroxymethyl-2,10-dimethyl-, (E,E)- $$ (2E,6Z)-7-(Hydroxymethyl)-3,11-dimethyl-2,6,10-dodecatrienyl acetat

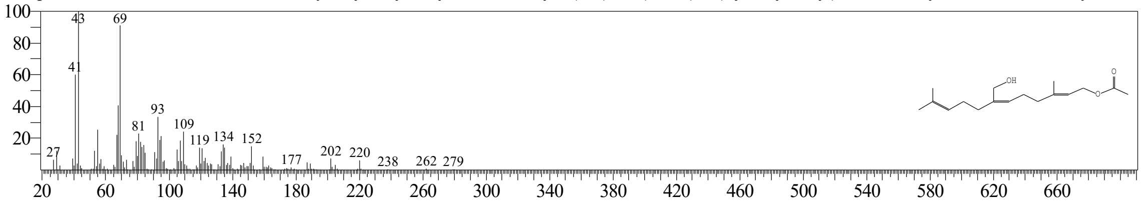

Hit#:3 Entry:79748 Library:NIST11.lib
SI:59 Formula:C16H26O2 CAS:0-00-0 MolWeight:250 RetIndex:1752
CompName:Formic acid, 3,7,11-trimethyl-1,6,10-dodecatrien-3-yl ester $$ (4E)-1,5,9-Trimethyl-1-vinyl-4,8-decadienyl formate # $$

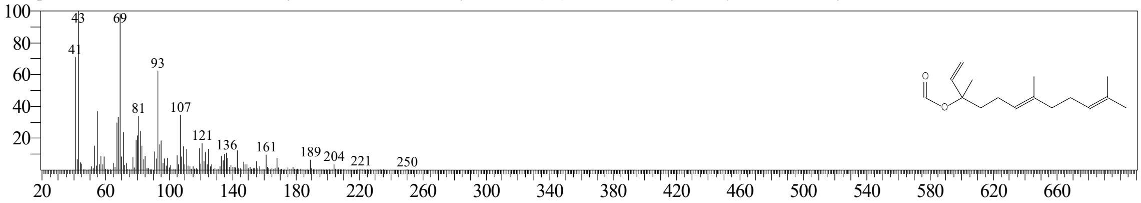

Hit#:4 Entry:17477 Library:NIST11.lib
SI:58 Formula:C10H18O CAS:0-00-0 MolWeight:154 RetIndex:1088
CompName:exo-2,7,7-trimethylbicyclo[2.2.1]heptan-2-ol

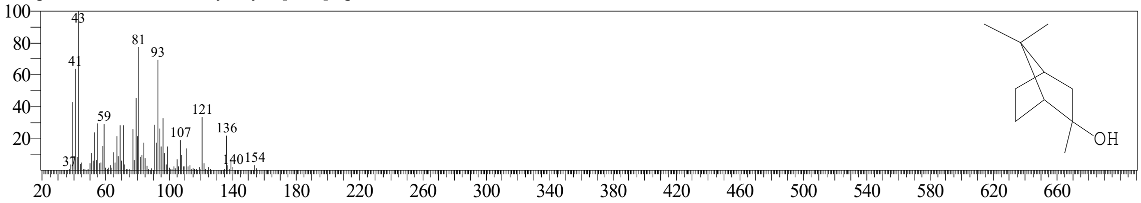

Hit#:5 Entry:49598 Library:NIST11.lib
SI:58 Formula:C14H24O CAS:77143-20-5 MolWeight:208 RetIndex:1457
CompName:4-(2,2,6-Trimethyl-bicyclo[4.1.0]hept-1-yl)-butan-2-one $$ 4-(2,2,6-Trimethylbicyclo[4.1.0]hept-1-yl)-2-butanone # $$

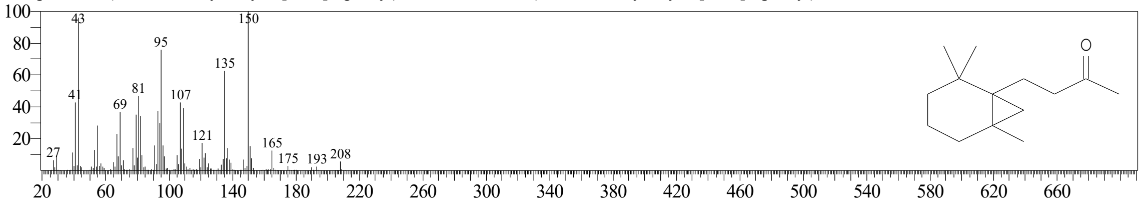

<< Target >>
Line#:136 R.Time:16.195(Scan#:2440) MassPeaks:376
RawMode:Averaged 16.190-16.200(2439-2441) BasePeak:56.15(7499)
BG Mode:Calc. from Peak Group 1 - Event 1 Scan

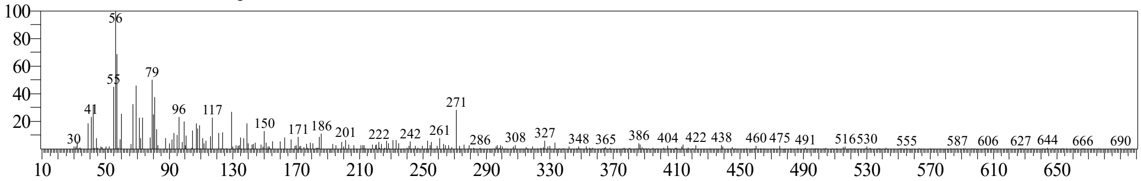

Hit#:1 Entry:138736 Library:NIST11.lib
SI:63 Formula:C21H42O2 CAS:0-00-0 MolWeight:326 RetIndex:2212
CompName:Butyl 15-methylhexadecanoate

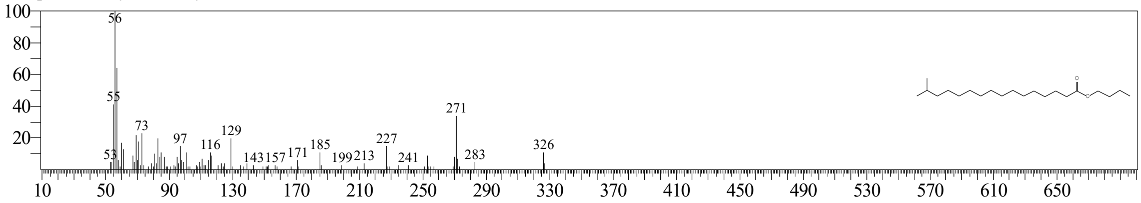

Hit#:2 Entry:101390 Library:NIST11.lib
SI:60 Formula:C18H30O2 CAS:463-40-1 MolWeight:278 RetIndex:2191
CompName:9,12,15-Octadecatrienoic acid, (Z,Z,Z)- $$ Linolenic acid $$ .alpha.-Linolenic acid $$ All-cis-9,12,15-Octadecatrienoic acid $$ cis,cis,cis-9,12,15-(

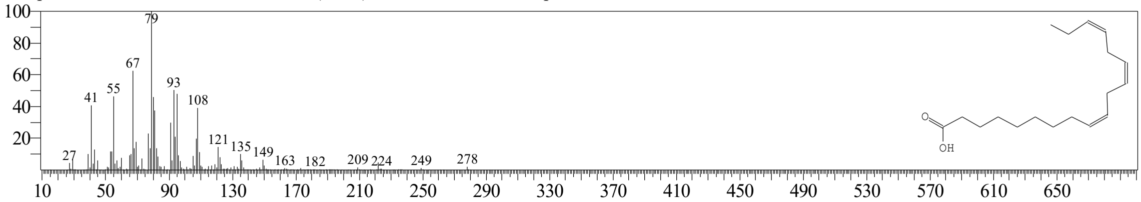

Hit#:3 Entry:102888 Library:NIST11.lib
SI:60 Formula:C19H36O CAS:0-00-0 MolWeight:280 RetIndex:2104
CompName:12-Methyl-E,E-2,13-octadecadien-1-ol $$ (2E,15Z)-14-Methyl-2,15-octadecadien-1-ol # $$

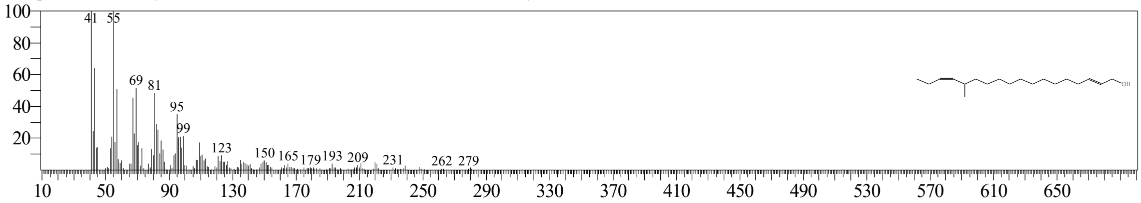

Hit#:4 Entry:15539 Library:NIST11.lib
SI:60 Formula:C11H18 CAS:115948-98-6 MolWeight:150 RetIndex:1027
CompName:2,2-Dimethyl-3-vinyl-bicyclo[2.2.1]heptane

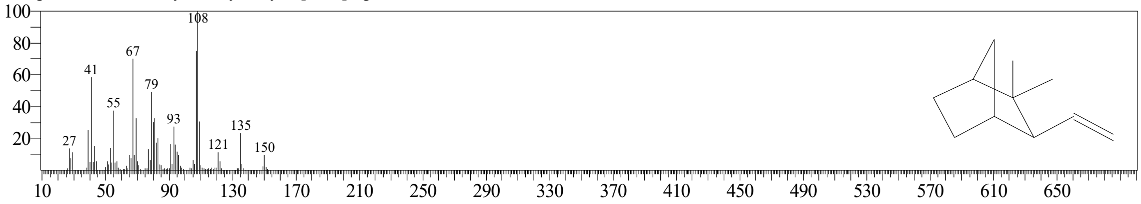

Hit#:5 Entry:144979 Library:NIST11.lib
SI:59 Formula:C22H38O2 CAS:10152-71-3 MolWeight:334 RetIndex:2266
CompName:Cyclopropaneoctanoic acid, 2-[[2-[(2-ethylcyclopropyl)methyl]cyclopropyl]methyl]-, methyl ester $$ Methyl 8-[2-((2-[(2-ethylcyclopropyl)methyl]c

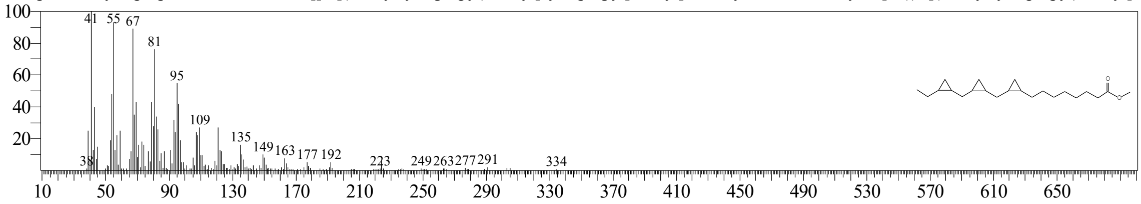

Qualitative Analysis Report

<< Target >>
Line#:137   R.Time:16.300(Scan#:2461) MassPeaks:373
RawMode:Averaged 16.295-16.305(2460-2462) BasePeak:43.10(84830)
BG Mode:Calc. from Peak Group 1 - Event 1 Scan

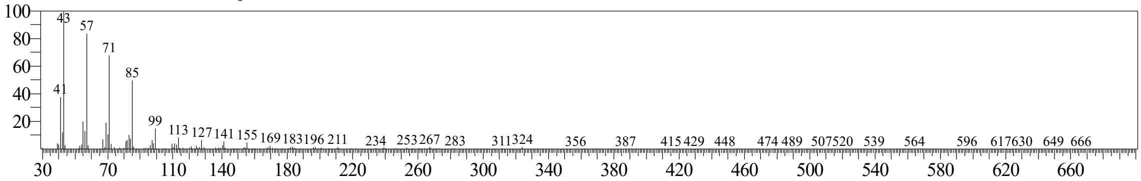

Hit#:1 Entry:157092 Library:NIST11.lib
SI:93 Formula:C25H52 CAS:7225-64-1 MolWeight:352 RetIndex:2442
CompName:Heptadecane, 9-octyl- $$ 9-Octylheptadecane # $$

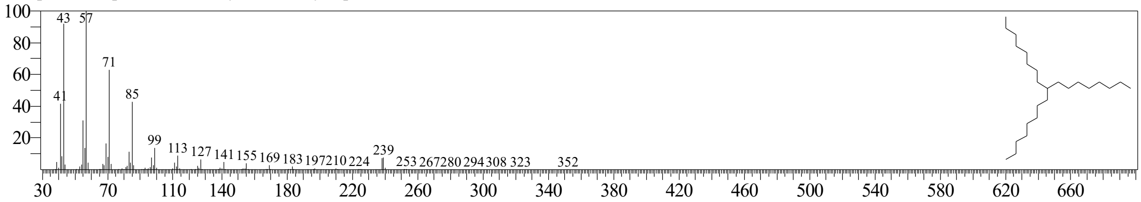

Hit#:2 Entry:115539 Library:NIST11.lib
SI:92 Formula:C21H44 CAS:54833-23-7 MolWeight:296 RetIndex:2045
CompName:Eicosane, 10-methyl- $$ 10-Methylicosane # $$ 10-Methyleicosane $$

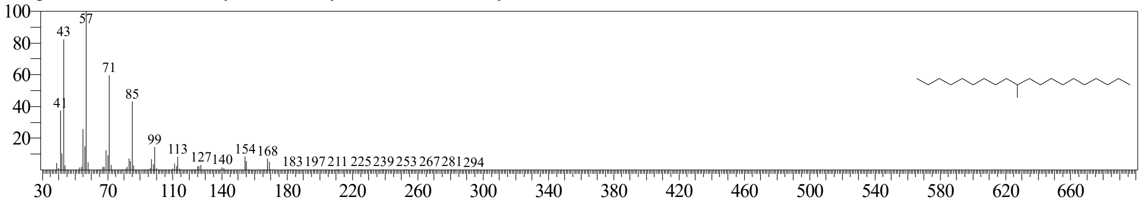

Hit#:3 Entry:165568 Library:NIST11.lib
SI:92 Formula:C26H54 CAS:55282-11-6 MolWeight:366 RetIndex:2477
CompName:Heneicosane, 11-(1-ethylpropyl)- $$ 11-(1-Ethylpropyl)heneicosane $$ 11-(3'-n-Pentyl)heneicosane $$ 11-(1-Ethylpropyl)henicosane # $$

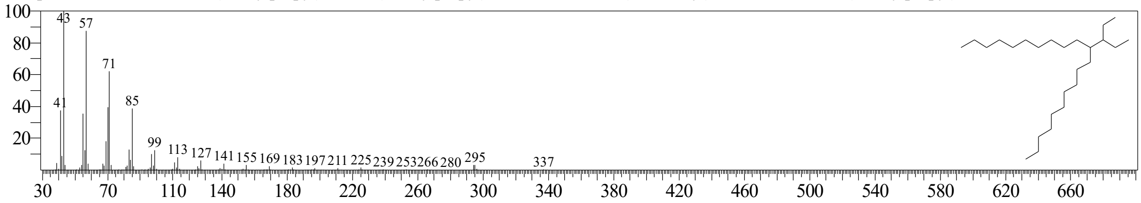

Hit#:4 Entry:165575 Library:NIST11.lib
SI:92 Formula:C26H54 CAS:55333-99-8 MolWeight:366 RetIndex:2542
CompName:Eicosane, 7-hexyl- $$ 7-n-Hexyleicosane $$ 7-Hexylicosane # $$

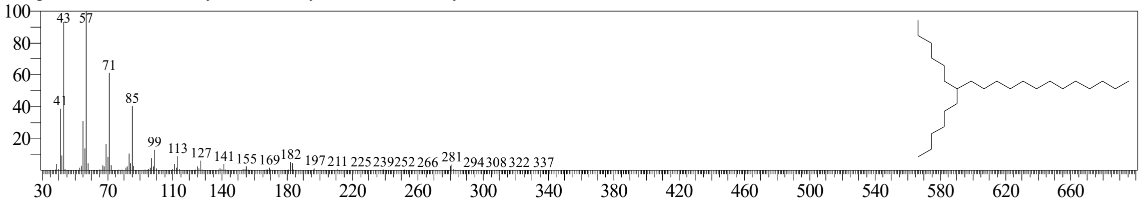

Hit#:5 Entry:93606 Library:NIST11.lib
SI:92 Formula:C19H40 CAS:61868-03-9 MolWeight:268 RetIndex:1782
CompName:Heptadecane, 2,3-dimethyl- $$ 2,3-Dimethylheptadecane # $$

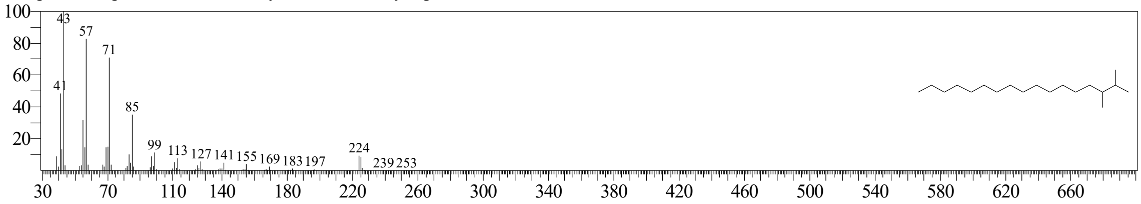

<< Target >>
Line#:138 R.Time:16.365(Scan#:2474) MassPeaks:348
RawMode:Averaged 16.360-16.370(2473-2475) BasePeak:69.10(11974)
BG Mode:Calc. from Peak Group 1 - Event 1 Scan

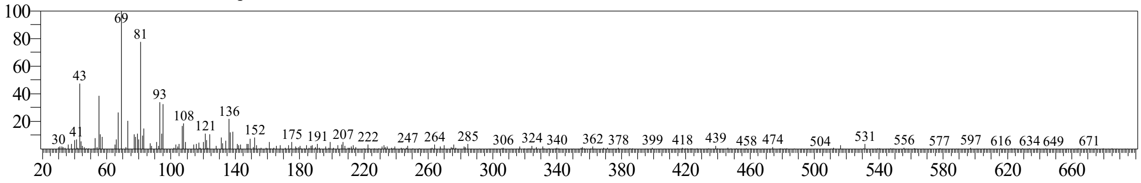

Hit#:1 Entry:38803 Library:NIST11.lib
SI:74 Formula:C14H24 CAS:230646-72-7 MolWeight:192 RetIndex:1227
CompName:1,5,9-Decatriene, 2,3,5,8-tetramethyl- $$ (5E)-2,3,5,8-Tetramethyl-1,5,9-decatriene # $$

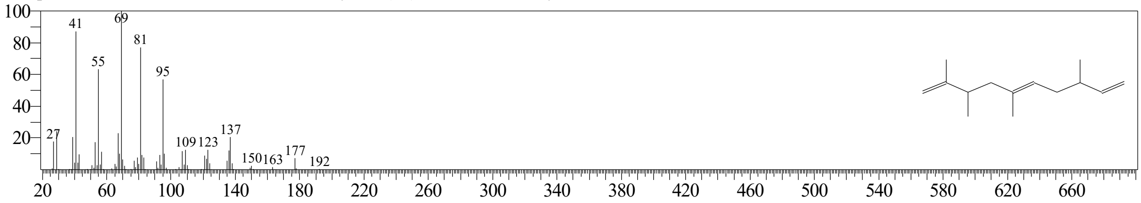

Hit#:2 Entry:191688 Library:NIST11.lib
SI:74 Formula:C30H52O CAS:0-00-0 MolWeight:428 RetIndex:3093
CompName:2,2,4-Trimethyl-3-(3,8,12,16-tetramethyl-heptadeca-3,7,11,15-tetraenyl)-cyclohexanol $$ 2,2,4-Trimethyl-3-[(3E,7E,11E)-3,8,12,16-tetramethyl-3,7

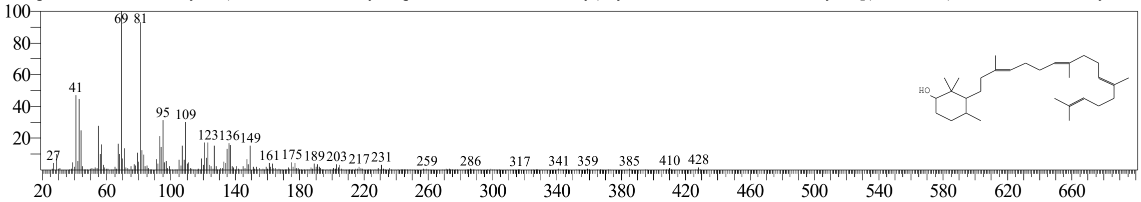

Hit#:3 Entry:60921 Library:NIST11.lib
SI:74 Formula:C15H28O CAS:3625-49-8 MolWeight:224 RetIndex:1574
CompName:6,10-Dodecadien-3-ol, 3,7,11-trimethyl- $$ (6E)-3,7,11-Trimethyl-6,10-dodecadien-3-ol # $$

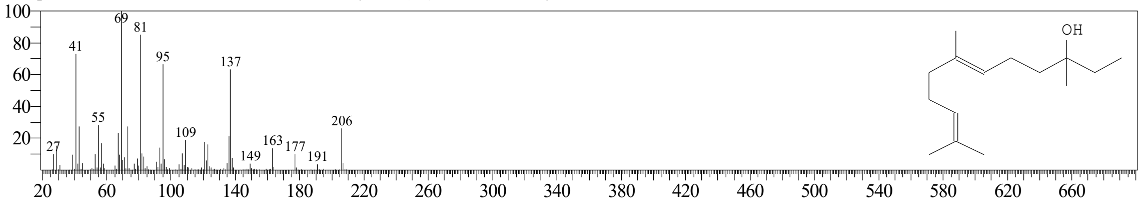

Hit#:4 Entry:91884 Library:NIST11.lib
SI:74 Formula:C17H30O2 CAS:0-00-0 MolWeight:266 RetIndex:1743
CompName:3,7,11,Trimethyl-8,10- dodecedienylacetate

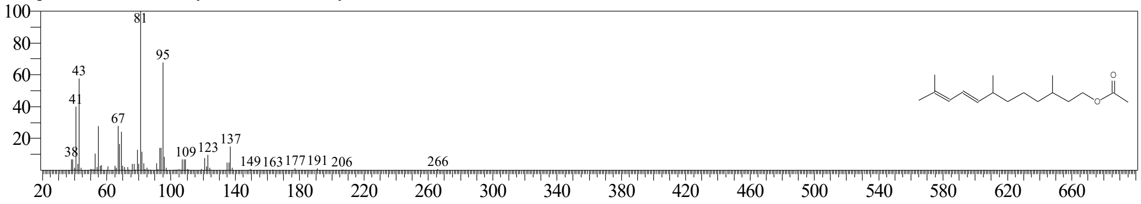

Hit#:5 Entry:191111 Library:NIST11.lib
SI:74 Formula:C30H50O CAS:54159-46-5 MolWeight:426 RetIndex:3058
CompName:1,6,10,14,18,22-Tetracosahexaen-3-ol, 2,6,10,15,19,23-hexamethyl-, (all-E)- $$ (6E,10E,14E,18E)-2,6,10,15,19,23-Hexamethyl-1,6,10,14,18,22-te

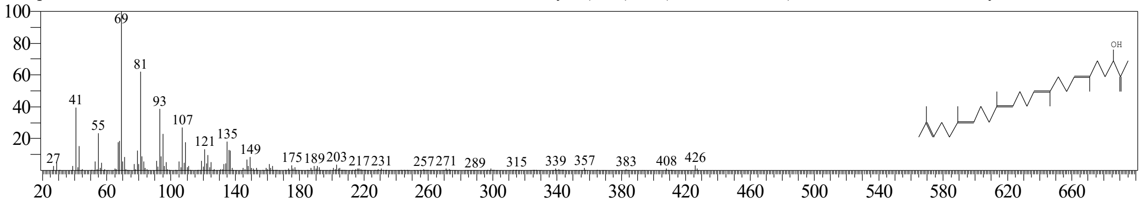

<< Target >>
Line#:139 R.Time:16.420(Scan#:2485) MassPeaks:393
RawMode:Averaged 16.415-16.425(2484-2486) BasePeak:108.10(14276)
BG Mode:Calc. from Peak Group 1 - Event 1 Scan

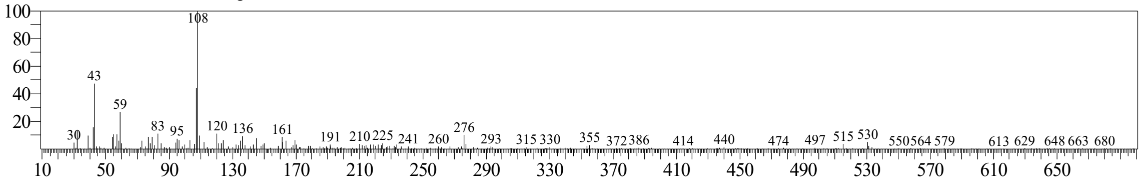

Hit#:1 Entry:23463 Library:NIST11.lib
SI:64 Formula:C10H14O2 CAS:22607-13-2 MolWeight:166 RetIndex:1416
CompName:1,2-Butanediol, 1-phenyl- $$ 1-Phenyl-1,2-butanediol # $$

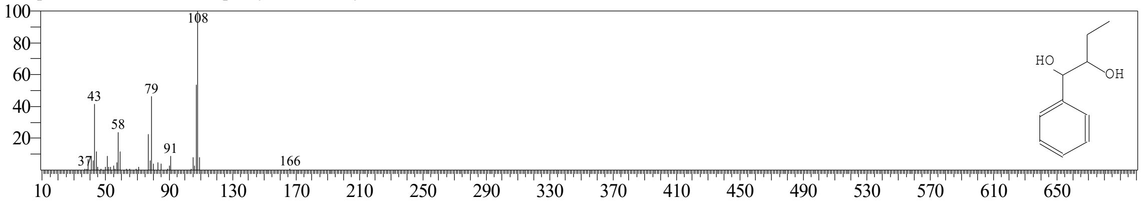

Hit#:2 Entry:90155 Library:NIST11.lib
SI:59 Formula:C15H20O4 CAS:0-00-0 MolWeight:264 RetIndex:1937
CompName:Oxalic acid, hexyl 2-methylphenyl ester

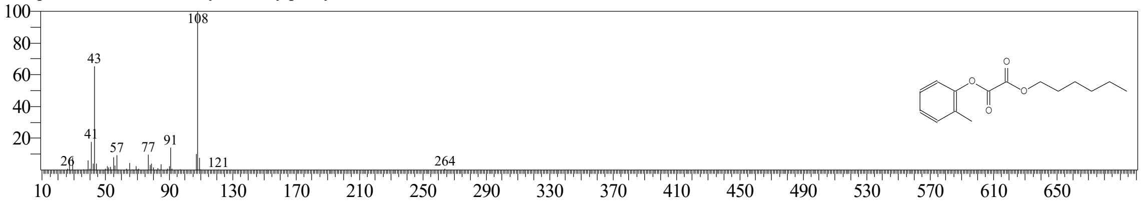

Hit#:3 Entry:67850 Library:NIST11.lib
SI:59 Formula:C15H22O2 CAS:5457-63-6 MolWeight:234 RetIndex:1770
CompName:Octanoic acid, 2-methylphenyl ester $$ 2-Methylphenyl octanoate # $$

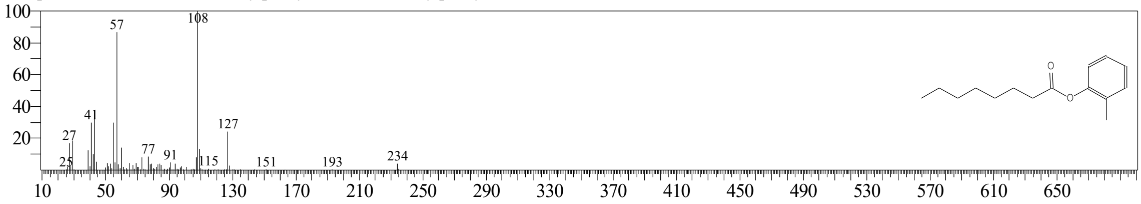

Hit#:4 Entry:63757 Library:NIST11.lib
SI:59 Formula:C14H16N2O CAS:0-00-0 MolWeight:228 RetIndex:1858
CompName:2-(2-Hydroxy-2-methyl-2-phenylethyl)-3-methyl $$ 1-(3-Methyl-2-pyrazinyl)-2-phenyl-2-propanol # $$

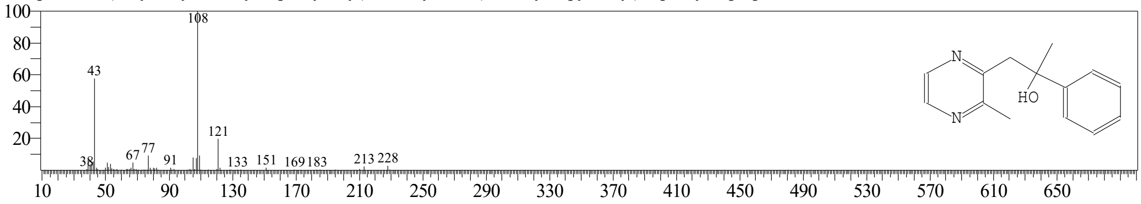

Hit#:5 Entry:30690 Library:NIST11.lib
SI:58 Formula:C9H13N3O CAS:0-00-0 MolWeight:179 RetIndex:1659
CompName:1-Acetyl-4-amino-5-ethyl-2,5-dihydro-1H-pyrrole-3-carbonitrile $$ Cl-4835 $$

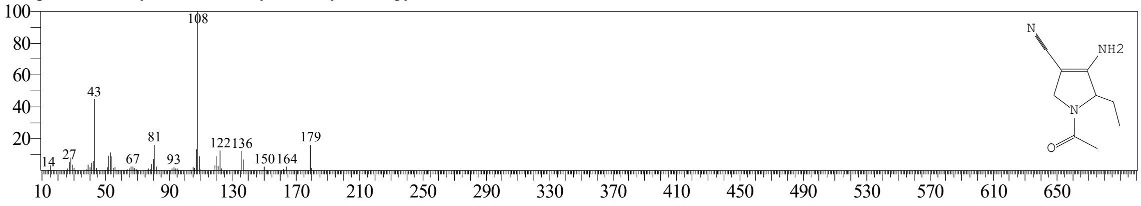

<< Target >>
Line#:140 R.Time:16.495(Scan#:2500) MassPeaks:357
RawMode:Averaged 16.490-16.500(2499-2501) BasePeak:122.95(13703)
BG Mode:Calc. from Peak Group 1 - Event 1 Scan

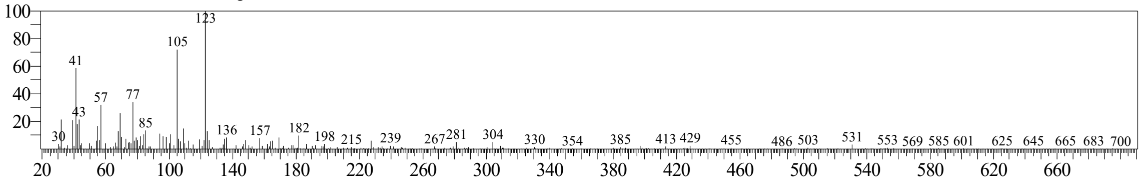

Hit#:1 Entry:121896 Library:NIST11.lib
SI:72 Formula:C20H32O2 CAS:0-00-0 MolWeight:304 RetIndex:2253
CompName:Benzoic acid, tridecyl ester

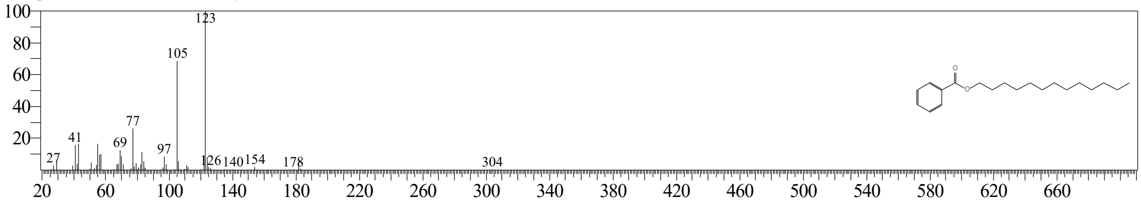

Hit#:2 Entry:143354 Library:NIST11.lib
SI:70 Formula:C22H36O2 CAS:0-00-0 MolWeight:332 RetIndex:2452
CompName:Benzoic acid, pentadecyl ester

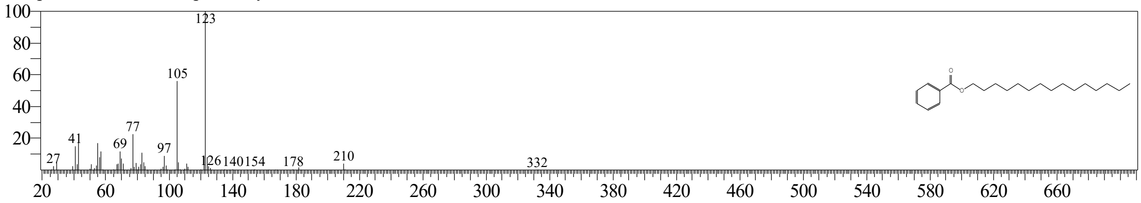

Hit#:3 Entry:67859 Library:NIST11.lib
SI:69 Formula:C15H22O2 CAS:94-50-8 MolWeight:234 RetIndex:1756
CompName:Benzoic acid, octyl ester $$ n-Octyl benzoate $$ Octyl benzoate $$

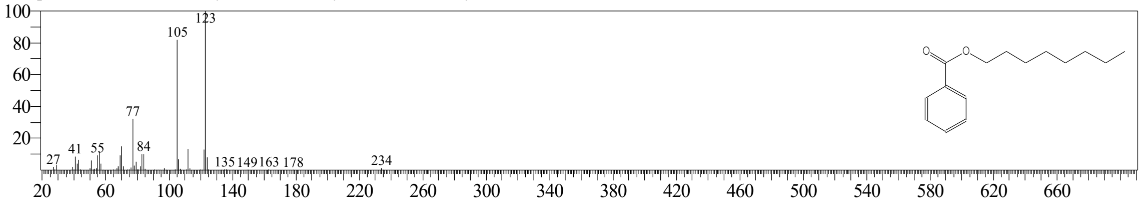

Hit#:4 Entry:132854 Library:NIST11.lib
SI:69 Formula:C21H34O2 CAS:0-00-0 MolWeight:318 RetIndex:2353
CompName:Benzoic acid, tetradecyl ester

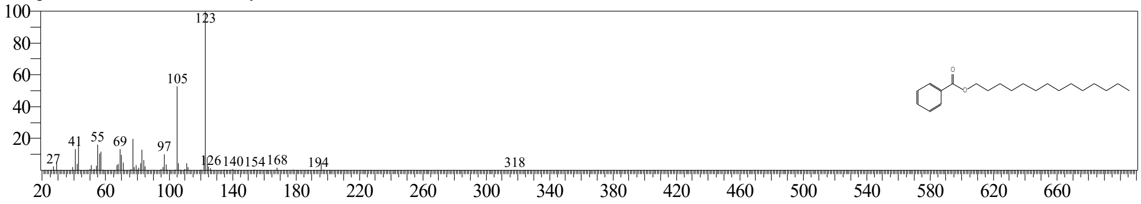

Hit#:5 Entry:47861 Library:NIST11.lib
SI:69 Formula:C13H18O2 CAS:6789-88-4 MolWeight:206 RetIndex:1557
CompName:Benzoic acid, hexyl ester $$ n-Hexyl benzoate $$ Hexyl benzoate $$ Benzoic acid n-hexyl ester $$ Hexylester kyseliny benzoove $$

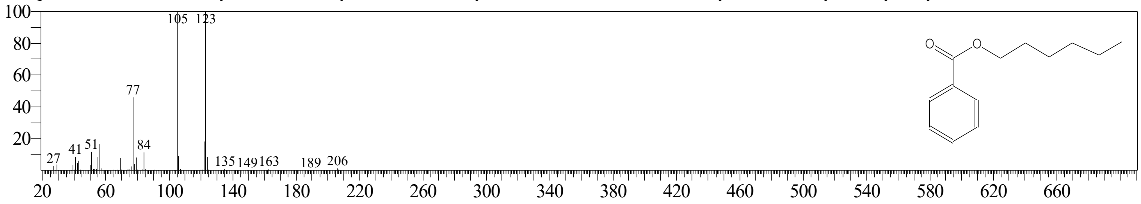

<< Target >>
Line#:141 R.Time:16.535(Scan#:2508) MassPeaks:360
RawMode:Averaged 16.530-16.540(2507-2509) BasePeak:74.15(6836)
BG Mode:Calc. from Peak Group 1 - Event 1 Scan

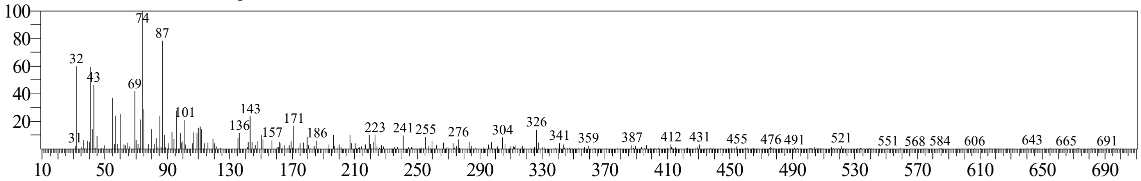

Hit#:1 Entry:35026 Library:NIST11.lib
SI:65 Formula:C10H18O3 CAS:1931-63-1 MolWeight:186 RetIndex:1371
CompName:Nonanoic acid, 9-oxo-, methyl ester $$ Azelaaldehydic acid, methyl ester $$ Methyl azelaaldehydate $$ Methyl 8-formyloctanoate $$ Methyl 9-oxo

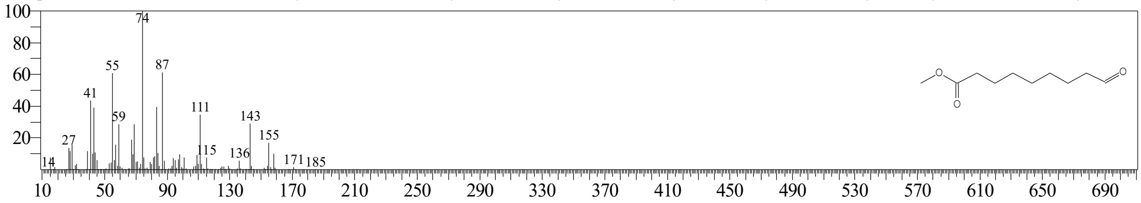

Hit#:2 Entry:138746 Library:NIST11.lib
SI:65 Formula:C21H42O2 CAS:1120-28-1 MolWeight:326 RetIndex:2276
CompName:Eicosanoic acid, methyl ester $$ Methyl arachisate $$ Methyl eicosanoate $$ Arachidic acid methyl ester $$ Kemester 2050 $$ Methyl aracidate $$

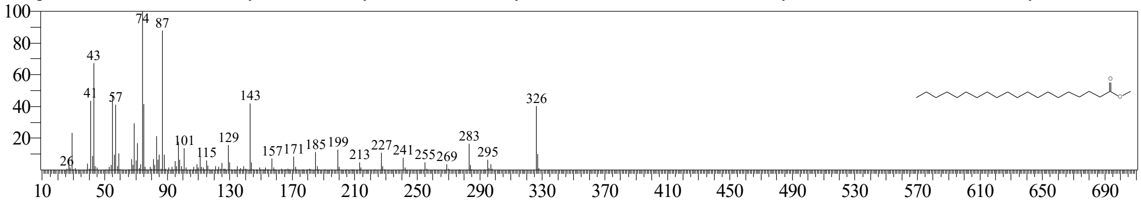

Hit#:3 Entry:138745 Library:NIST11.lib
SI:65 Formula:C21H42O2 CAS:0-00-0 MolWeight:326 RetIndex:2212
CompName:Methyl 18-methylnonadecanoate

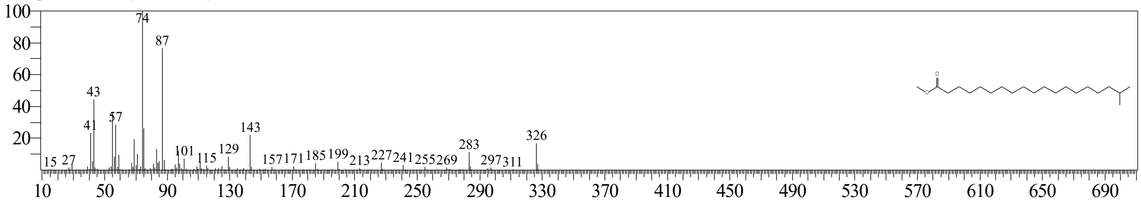

Hit#:4 Entry:115441 Library:NIST11.lib
SI:64 Formula:C19H36O2 CAS:24828-61-3 MolWeight:296 RetIndex:2120
CompName:Cyclopentanetridecanoic acid, methyl ester $$ Methyl dihydrochaulmoograte $$ Methyl 13-cyclopentyltridecanoate # $$

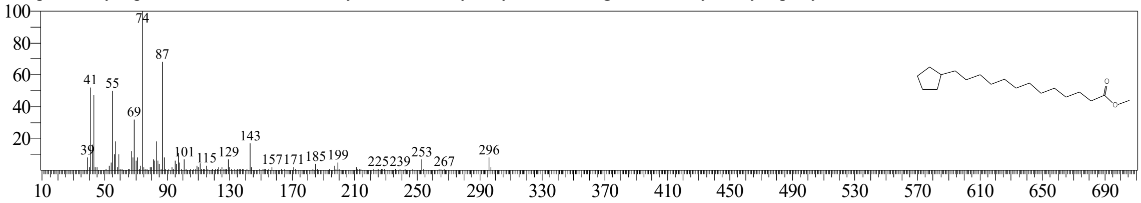

Hit#:5 Entry:190416 Library:NIST11.lib
SI:63 Formula:C28H56O2 CAS:55682-91-2 MolWeight:424 RetIndex:2972
CompName:Heptacosanoic acid, methyl ester $$ Methyl heptacosanoate # $$

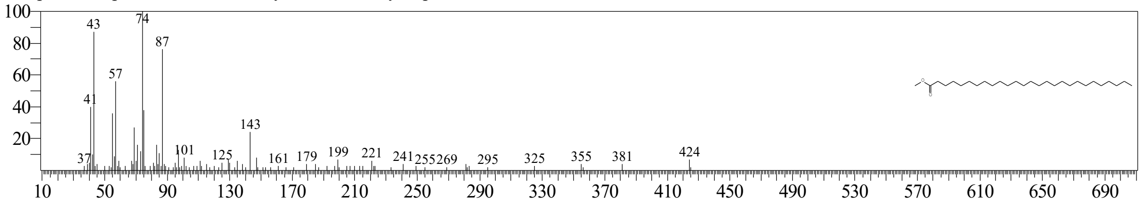

Qualitative Analysis Report

<< Target >>
Line#:142 R.Time:16.590(Scan#:2519) MassPeaks:402
RawMode:Averaged 16.585-16.595(2518-2520) BasePeak:82.15(7644)
BG Mode:Calc. from Peak Group 1 - Event 1 Scan

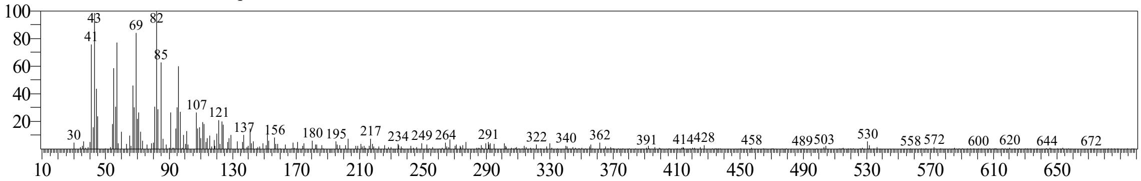

Hit#:1 Entry:62442 Library:NIST11.lib
SI:81 Formula:C15H30O CAS:2765-11-9 MolWeight:226 RetIndex:1701
CompName:Pentadecanal- $$ 1-Pentadecanal $$ n-Pentadecanal $$

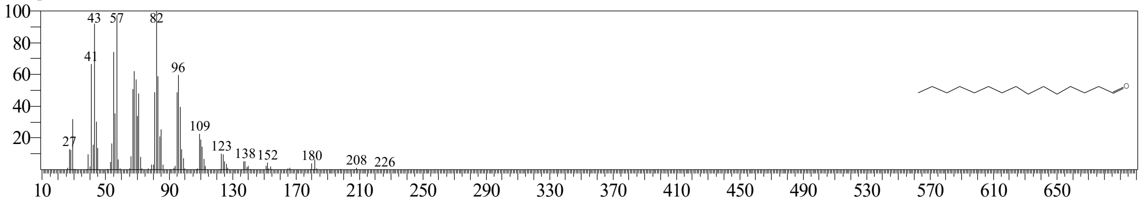

Hit#:2 Entry:72472 Library:NIST11.lib
SI:81 Formula:C16H32O CAS:629-80-1 MolWeight:240 RetIndex:1800
CompName:Hexadecanal $$ Palmitaldehyde $$ 1-Hexadecanal $$ n-Hexadecanal $$

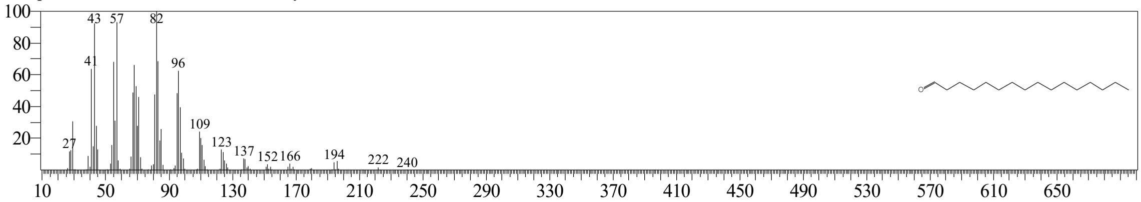

Hit#:3 Entry:82829 Library:NIST11.lib
SI:81 Formula:C17H34O CAS:0-00-0 MolWeight:254 RetIndex:1899
CompName:Heptadecanal

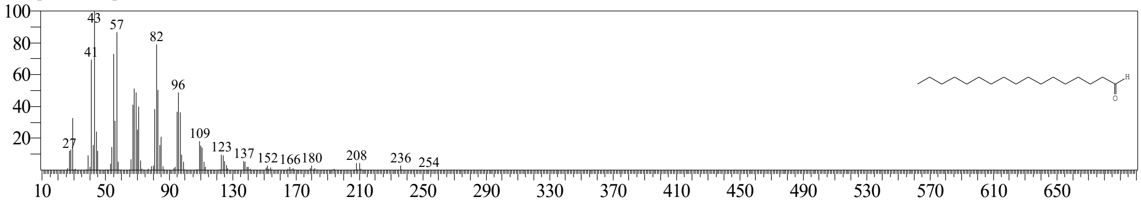

Hit#:4 Entry:93586 Library:NIST11.lib
SI:80 Formula:C18H36O CAS:638-66-4 MolWeight:268 RetIndex:1999
CompName:Octadecanal $$ Stearaldehyde $$ Octadecyl aldehyde $$ Stearyl aldehyde $$ n-Octadecanal $$

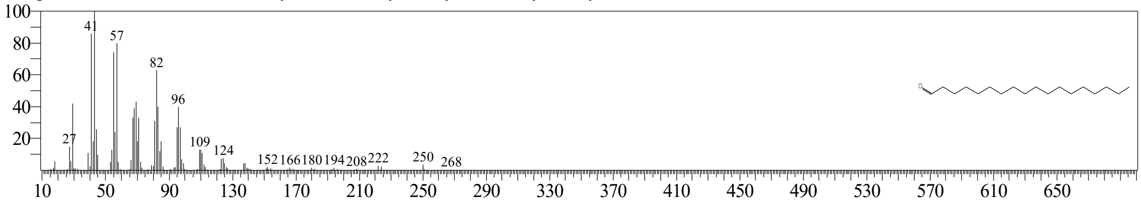

Hit#:5 Entry:52428 Library:NIST11.lib
SI:80 Formula:C14H28O CAS:124-25-4 MolWeight:212 RetIndex:1601
CompName:Tetradecanal $$ Myristaldehyde $$ Myristylaldehyde $$ Tetradecylaldehyde $$ n-Tetradecanal $$ Aldehyde C-14 $$ Aldehyde C-14, myristic $$ C-1

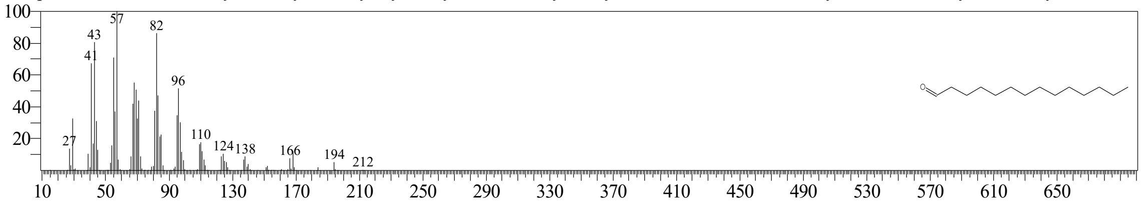

<< Target >>
Line#:143   R.Time:16.675(Scan#:2536) MassPeaks:373
RawMode:Averaged 16.670-16.680(2535-2537) BasePeak:55.10(6523)
BG Mode:Calc. from Peak Group 1 - Event 1 Scan

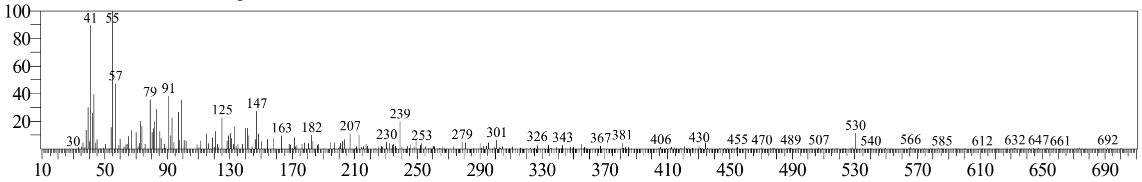

Hit#:1 Entry:140393 Library:NIST11.lib
SI:62 Formula:C22H32O2 CAS:60534-16-9 MolWeight:328 RetIndex:2413
CompName:Spiro[androst-5-ene-17,1'-cyclobutan]-2'-one, 3-hydroxy-, (3.beta.,17.beta.)-

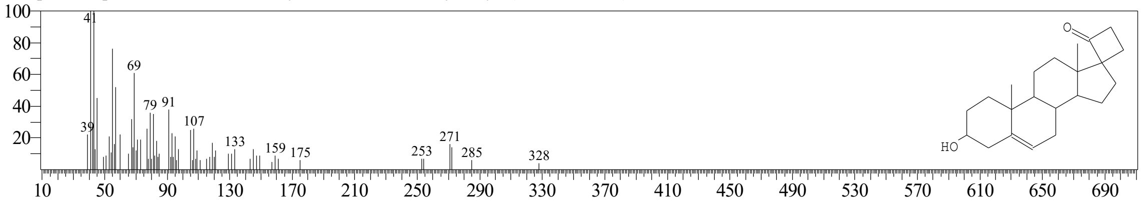

Hit#:2 Entry:144988 Library:NIST11.lib
SI:61 Formula:C23H42O CAS:0-00-0 MolWeight:334 RetIndex:2539
CompName:1-Heptadec-1-ynyl-cyclohexanol $$ 1-(1-Heptadecynyl)cyclohexanol # $$

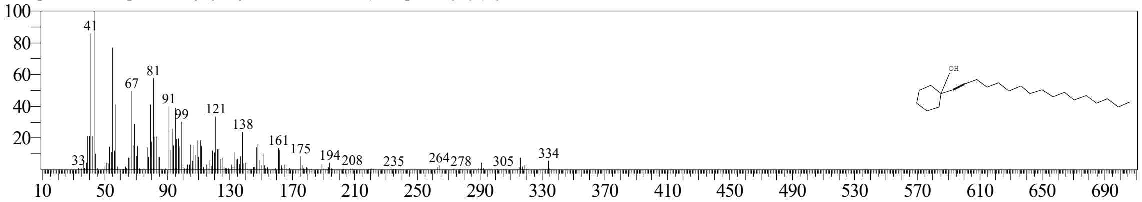

Hit#:3 Entry:143075 Library:NIST11.lib
SI:61 Formula:C19H37ClO2 CAS:53312-70-2 MolWeight:332 RetIndex:2302
CompName:3-Chloropropionic acid, hexadecyl ester $$ Hexadecyl 3-chloropropanoate # $$ Propanoic acid, 3-chloro, hexadecyl ester $$

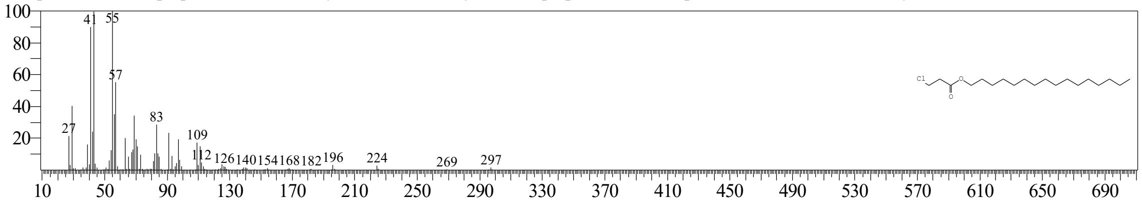

Hit#:4 Entry:6535 Library:NIST11.lib
SI:61 Formula:C8H14O CAS:4925-71-7 MolWeight:126 RetIndex:970
CompName:9-Oxabicyclo[6.1.0]nonane, cis- $$ 9-Oxabicyclo[6.1.0]nonane # $$

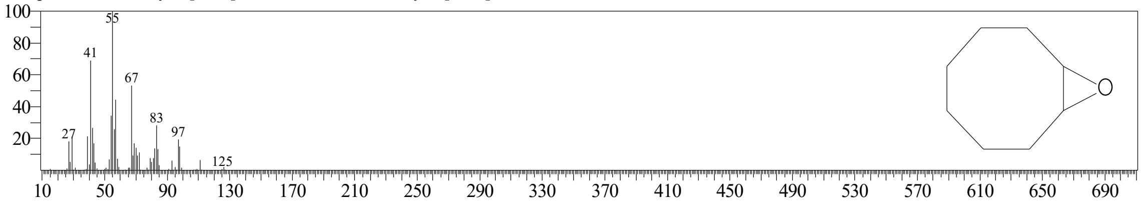

Hit#:5 Entry:156678 Library:NIST11.lib
SI:61 Formula:C18H37ClO2S CAS:0-00-0 MolWeight:352 RetIndex:2493
CompName:1-Octadecanesulphonyl chloride

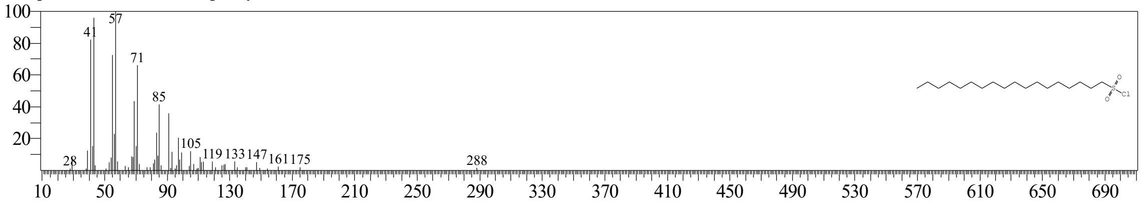

Qualitative Analysis Report

<< Target >>
Line#:144   R.Time:16.720(Scan#:2545) MassPeaks:364
RawMode:Averaged 16.715-16.725(2544-2546) BasePeak:83.05(5751)
BG Mode:Calc. from Peak Group 1 - Event 1 Scan

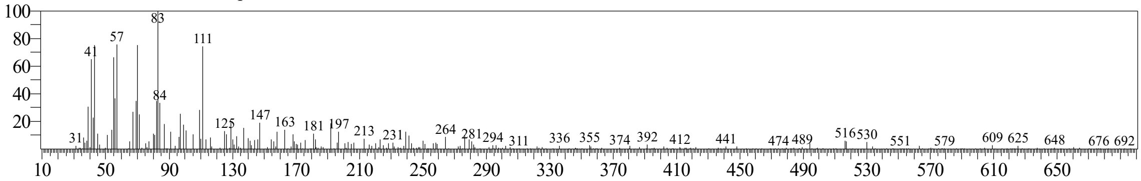

Hit#:1 Entry:32998 Library:NIST11.lib
SI:74 Formula:C13H26 CAS:2437-56-1 MolWeight:182 RetIndex:1304
CompName:1-Tridecene $$ n-Tridec-1-ene $$ 1-C13H26 $$ Tridecene-1 $$ .alpha.-Tridecene $$ Tridec-1-ene $$

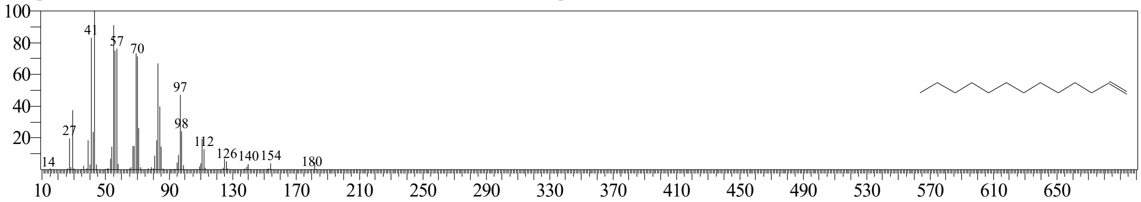

Hit#:2 Entry:121606 Library:NIST11.lib
SI:74 Formula:C17H33ClO2 CAS:64120-16-7 MolWeight:304 RetIndex:2104
CompName:3-Chloropropionic acid, tetradecyl ester $$ Tetradecyl 3-chloropropanoate # $$ Propanoic acid, 3-chloro, tetradecyl ester $$

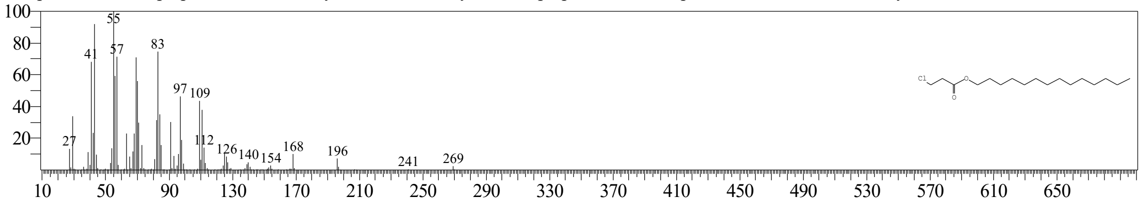

Hit#:3 Entry:84449 Library:NIST11.lib
SI:74 Formula:C17H36O CAS:1454-85-9 MolWeight:256 RetIndex:1954
CompName:n-Heptadecanol-1 $$ 1-Hydroxyheptadecane $$ 1-Heptadecanol $$ Heptadecan-1-ol $$ Heptadecyl alcohol $$

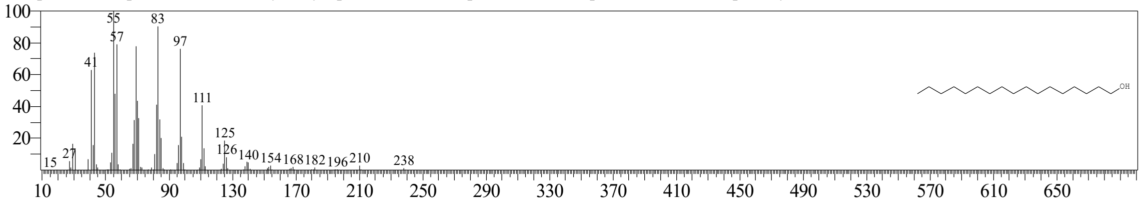

Hit#:4 Entry:53605 Library:NIST11.lib
SI:74 Formula:C13H26O2 CAS:28303-42-6 MolWeight:214 RetIndex:1578
CompName:Formic acid, dodecyl ester

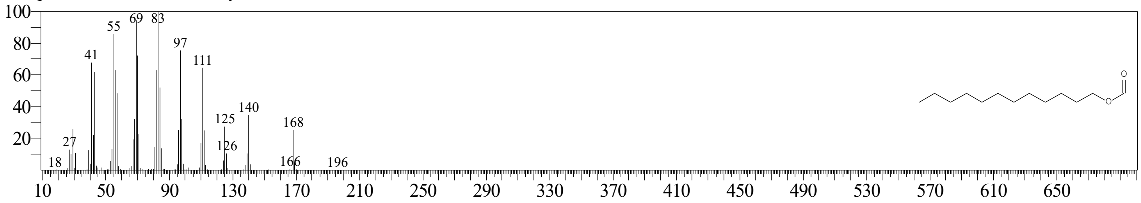

Hit#:5 Entry:102916 Library:NIST11.lib
SI:74 Formula:C20H40 CAS:74685-29-3 MolWeight:280 RetIndex:2017
CompName:9-Eicosene, (E)- $$ (9E)-9-Icosene # $$

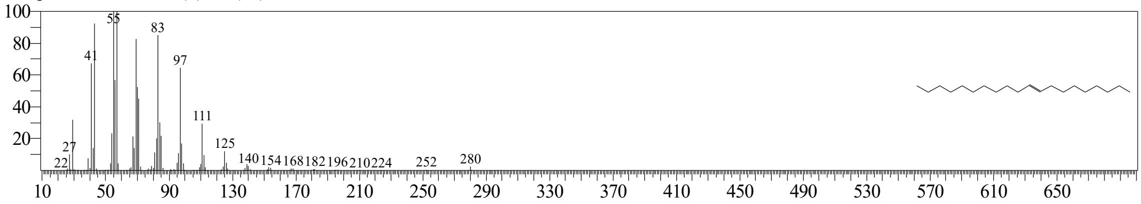

<< Target >>
Line#:145 R.Time:16.880(Scan#:2577) MassPeaks:420
RawMode:Averaged 16.875-16.885(2576-2578) BasePeak:41.05(143758)
BG Mode:Calc. from Peak Group 1 - Event 1 Scan

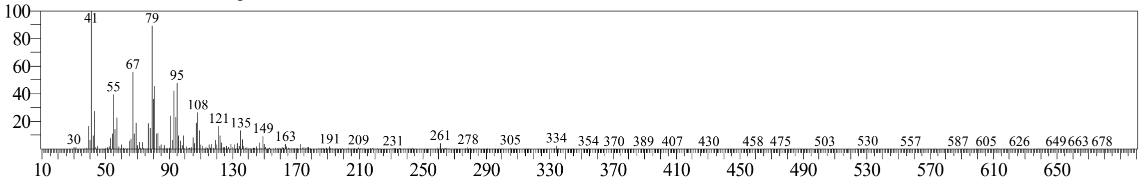

Hit#:1 Entry:101390 Library:NIST11.lib
SI:90 Formula:C18H30O2 CAS:463-40-1 MolWeight:278 RetIndex:2191
CompName:9,12,15-Octadecatrienoic acid, (Z,Z,Z)- $$ Linolenic acid $$ .alpha.-Linolenic acid $$ All-cis-9,12,15-Octadecatrienoic acid $$ cis,cis,cis-9,12,15-(

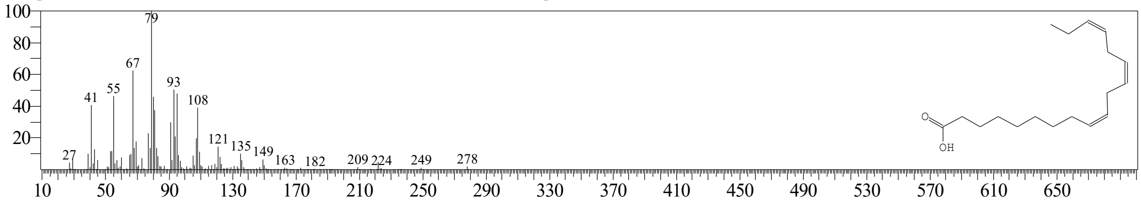

Hit#:2 Entry:90503 Library:NIST11.lib
SI:89 Formula:C18H32O CAS:506-44-5 MolWeight:264 RetIndex:2077
CompName:9,12,15-Octadecatrien-1-ol, (Z,Z,Z)- $$ (9E,12E,15E)-9,12,15-Octadecatrien-1-ol # $$ (Z,Z,Z)-9,12,15-Octadecatrien-1-ol $$

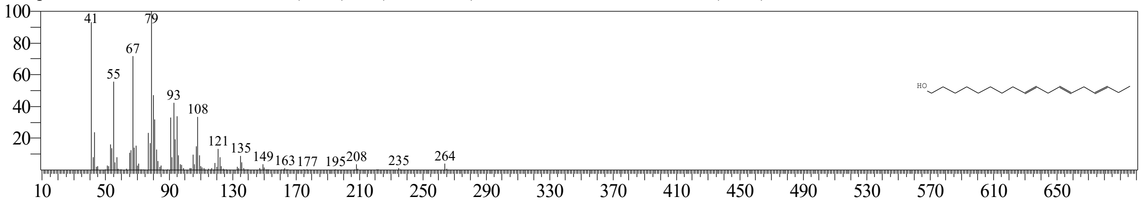

Hit#:3 Entry:67981 Library:NIST11.lib
SI:89 Formula:C17H30 CAS:74744-55-1 MolWeight:234 RetIndex:1736
CompName:3-Heptadecen-5-yne, (Z)- $$ (3Z)-3-Heptadecen-5-yne # $$

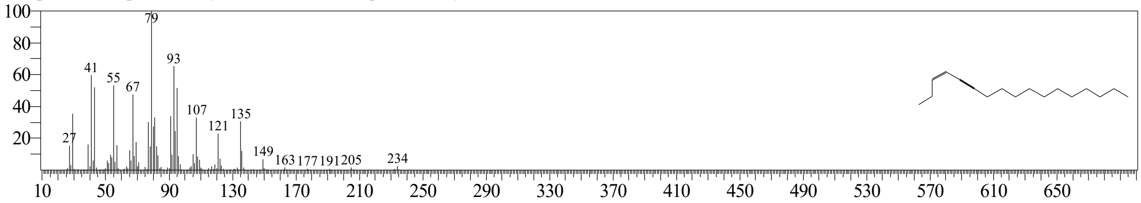

Hit#:4 Entry:132823 Library:NIST11.lib
SI:89 Formula:C21H34O2 CAS:59149-01-8 MolWeight:318 RetIndex:2308
CompName:Methyl (Z)-5,11,14,17-eicosatetraenoate $$ Methyl (5Z,11Z,14Z,17Z)-5,11,14,17-icosatetraenoate # $$

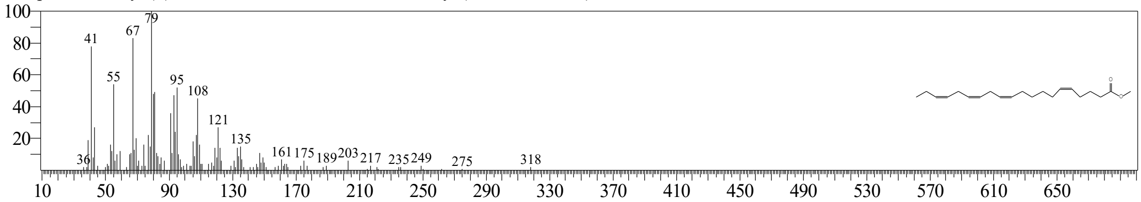

Hit#:5 Entry:67943 Library:NIST11.lib
SI:87 Formula:C16H26O CAS:56797-43-4 MolWeight:234 RetIndex:1824
CompName:cis,cis,cis-7,10,13-Hexadecatrienal $$ (7Z,10Z,13Z)-7,10,13-Hexadecatrienal # $$

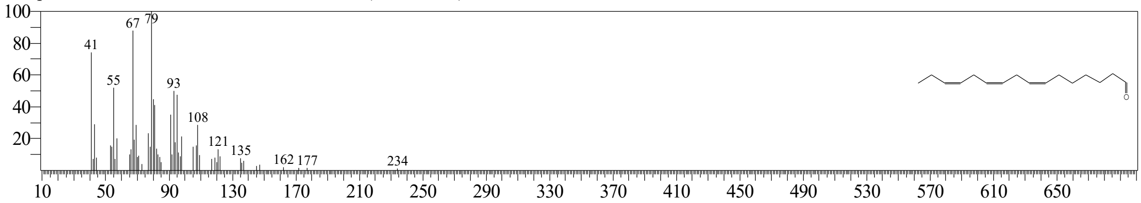

<< Target >>
Line#:146 R.Time:17.015(Scan#:2604) MassPeaks:361
RawMode:Averaged 17.010-17.020(2603-2605) BasePeak:43.05(20482)
BG Mode:Calc. from Peak Group 1 - Event 1 Scan

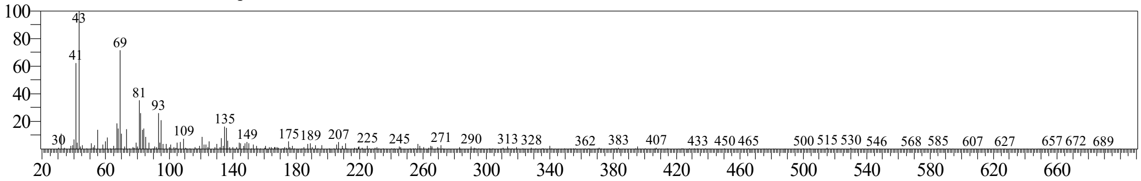

Hit#:1 Entry:89014 Library:NIST11.lib
SI:77 Formula:C18H30O CAS:1117-52-8 MolWeight:262 RetIndex:1902
CompName:5,9,13-Pentadecatrien-2-one, 6,10,14-trimethyl-, (E,E)- $$ Farnesyl acetone $$ (5E,9E)-6,10,14-Trimethyl-5,9,13-pentadecatrien-2-one # $$ 5,9,13-

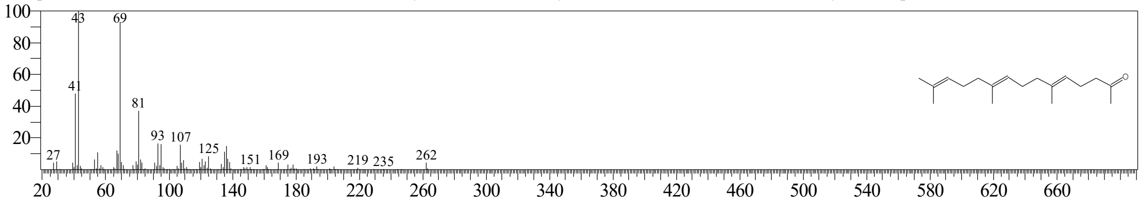

Hit#:2 Entry:41429 Library:NIST11.lib
SI:74 Formula:C12H20O2 CAS:0-00-0 MolWeight:196 RetIndex:1292
CompName:Myrcenylacetat $$ 6-Methyl-2-vinyl-5-heptenyl acetate # $$

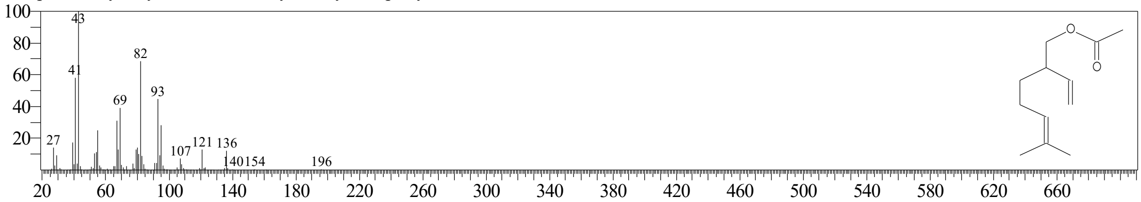

Hit#:3 Entry:102732 Library:NIST11.lib
SI:74 Formula:C17H28O3 CAS:125284-17-5 MolWeight:280 RetIndex:1900
CompName:4,8-Decadienoic acid, 2-acetyl-2,5,9-trimethyl-, ethyl ester, (E)- $$ Ethyl (4E)-2-acetyl-2,5,9-trimethyl-4,8-decadienoate # $$

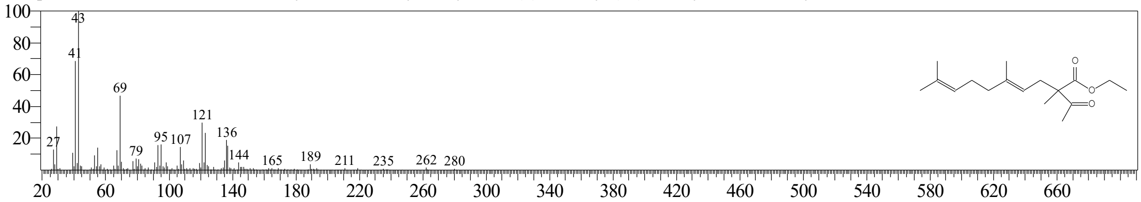

Hit#:4 Entry:102733 Library:NIST11.lib
SI:73 Formula:C17H28O3 CAS:0-00-0 MolWeight:280 RetIndex:2076
CompName:2,6,10-Dodecatriene, 12-acetoxy-6-hydroxymethyl-2,10-dimethyl-, (E,E)- $$ (2E,6Z)-7-(Hydroxymethyl)-3,11-dimethyl-2,6,10-dodecatrienyl acetat

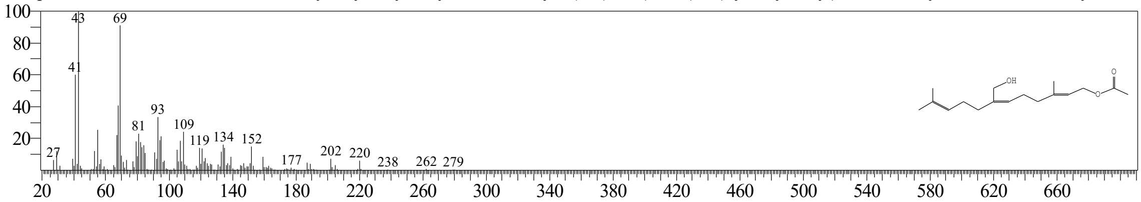

Hit#:5 Entry:143336 Library:NIST11.lib
SI:73 Formula:C22H36O2 CAS:61691-98-3 MolWeight:332 RetIndex:2316
CompName:2,6,10,14-Hexadecatetraen-1-ol, 3,7,11,15-tetramethyl-, acetate, (E,E,E)- $$ (2E,6E,10E)-3,7,11,15-Tetramethyl-2,6,10,14-hexadecatetraenyl acetat

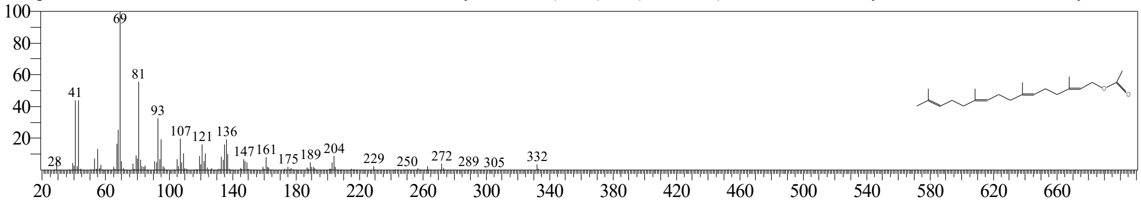

<< Target >>
Line#:147 R.Time:17.090(Scan#:2619) MassPeaks:372
RawMode:Averaged 17.085-17.095(2618-2620) BasePeak:56.10(10660)
BG Mode:Calc. from Peak Group 1 - Event 1 Scan

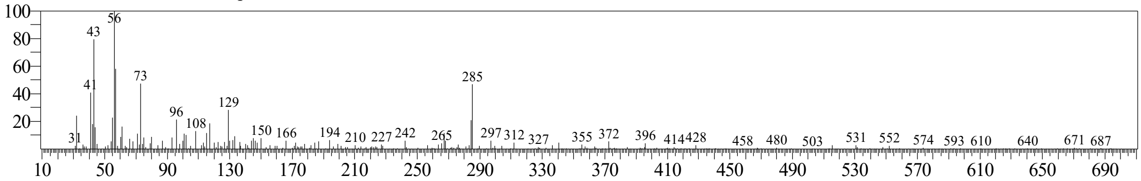

Hit#:1 Entry:149079 Library:NIST11.lib
SI:68 Formula:C22H44O2 CAS:123-95-5 MolWeight:340 RetIndex:2375
CompName:Octadecanoic acid, butyl ester $$ Stearic acid, butyl ester $$ n-Butyl octadecanoate $$ n-Butyl stearate $$ Apex 4 $$ Butyl octadecanoate $$ Butyl :

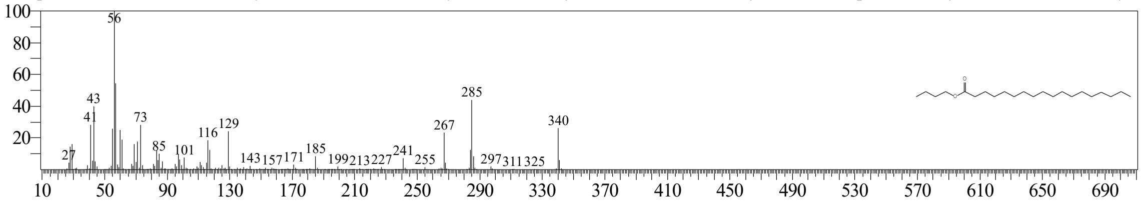

Hit#:2 Entry:149085 Library:NIST11.lib
SI:67 Formula:C22H44O2 CAS:646-13-9 MolWeight:340 RetIndex:2311
CompName:Octadecanoic acid, 2-methylpropyl ester $$ Stearic acid, isobutyl ester $$ Isobutyl stearate $$ Emerest 2324 $$ Estol 1476 $$ Kemester 5415 $$ Ke:

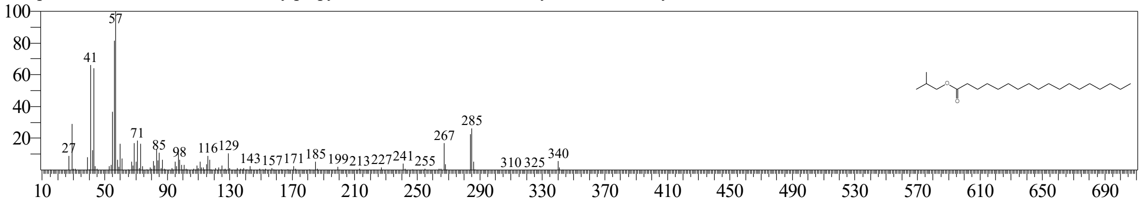

Hit#:3 Entry:106162 Library:NIST11.lib
SI:63 Formula:C18H36O2 CAS:110-36-1 MolWeight:284 RetIndex:1978
CompName:Butyl myristate $$ n-Butyl myristate $$ Myristic acid n-butyl ester $$ Tetradecanoic acid, butyl ester $$ Bumyr $$ Myristic acid, butyl ester $$ Buty

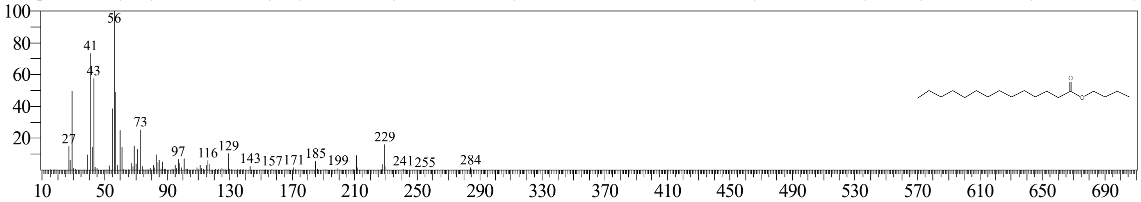

Hit#:4 Entry:43988 Library:NIST11.lib
SI:63 Formula:C12H24O2 CAS:589-75-3 MolWeight:200 RetIndex:1381
CompName:Butyl caprylate $$ Caprylic acid n-butyl ester $$ n-Caprylic acid n-butyl ester $$ Octanoic acid, butyl ester $$ n-Butylcaprylate $$ Butyl octanoate :

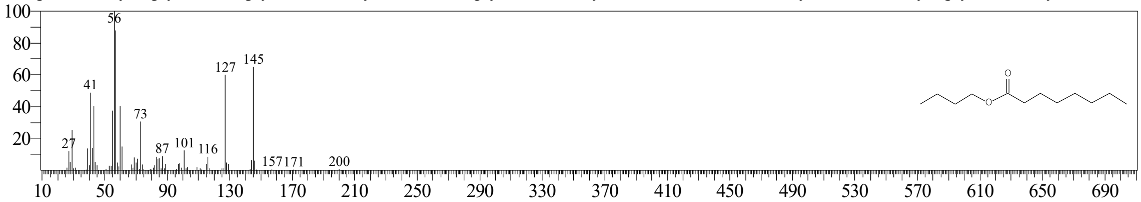

Hit#:5 Entry:128158 Library:NIST11.lib
SI:62 Formula:C20H40O2 CAS:31158-91-5 MolWeight:312 RetIndex:2092
CompName:Hexadecanoic acid, 1,1-dimethylethyl ester $$ tert-Butyl palmitate # $$

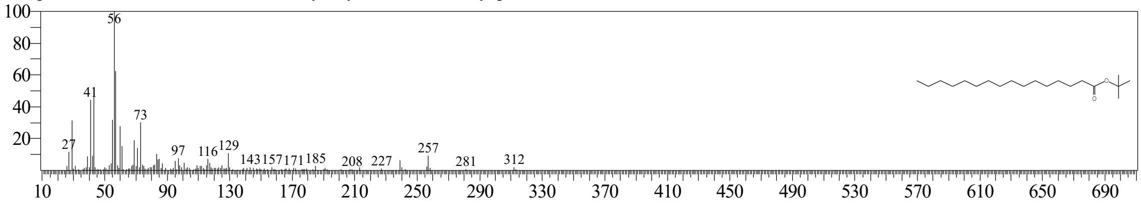

Qualitative Analysis Report

<< Target >>
Line#:148 R.Time:17.165(Scan#:2634) MassPeaks:393
RawMode:Averaged 17.160-17.170(2633-2635) BasePeak:43.10(359217)
BG Mode:Calc. from Peak Group 1 - Event 1 Scan

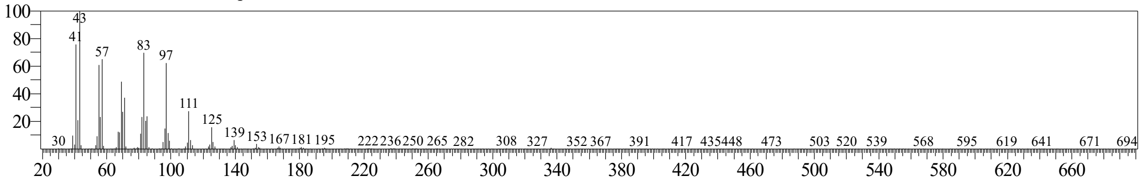

Hit#:1 Entry:81317 Library:NIST11.lib
SI:94 Formula:C17H32O CAS:0-00-0 MolWeight:252 RetIndex:1907
CompName:E-15-Heptadecenal $$ (15E)-15-Heptadecenal # $$

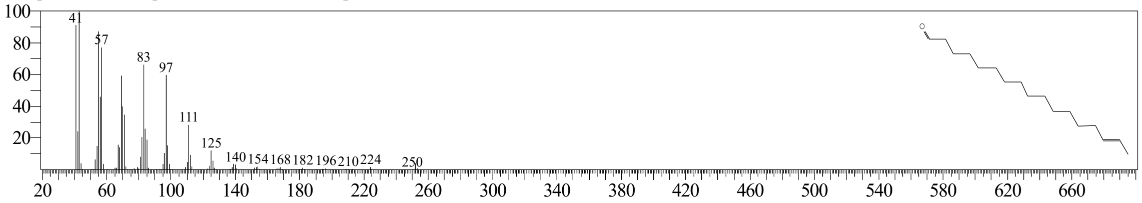

Hit#:2 Entry:146250 Library:NIST11.lib
SI:93 Formula:C24H48 CAS:297-03-0 MolWeight:336 RetIndex:2878
CompName:Cyclotetracosane

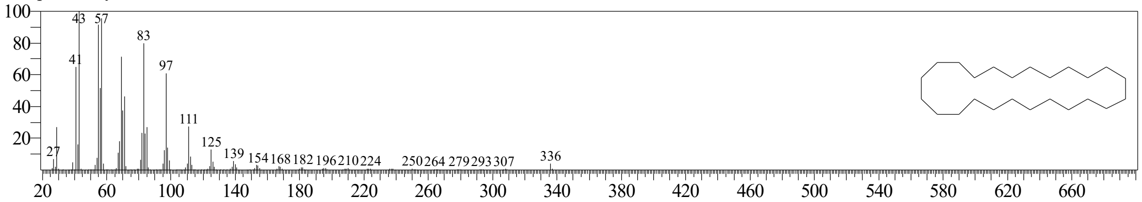

Hit#:3 Entry:82687 Library:NIST11.lib
SI:93 Formula:C16H30O2 CAS:56221-91-1 MolWeight:254 RetIndex:1769
CompName:13-Tetradecen-1-ol acetate $$ 13-Tetradecenyl acetate # $$

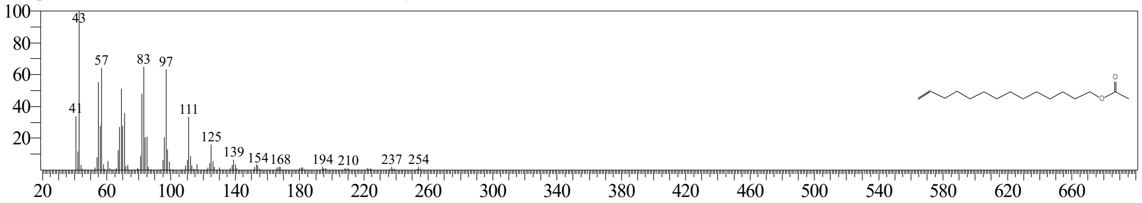

Hit#:4 Entry:197101 Library:NIST11.lib
SI:93 Formula:C21H35F7O2 CAS:959085-66-6 MolWeight:452 RetIndex:1833
CompName:Heptadecyl heptafluorobutyrate $$ Heptadecyl 2,2,3,3,4,4,4-heptafluorobutanoate $$ 1-Heptadecanol, heptafluorobutyrate $$ Heptadecyl perfluorob

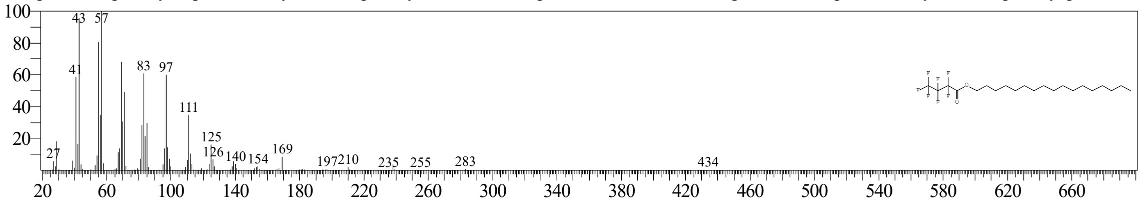

Hit#:5 Entry:102910 Library:NIST11.lib
SI:93 Formula:C20H40 CAS:296-56-0 MolWeight:280 RetIndex:2398
CompName:Cycloeicosane $$ Cycloicosane # $$

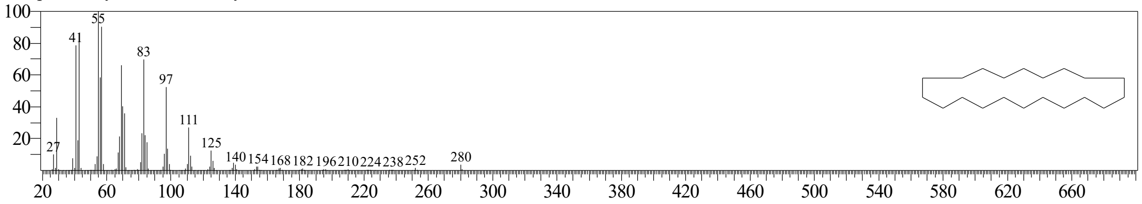

<< Target >>
Line#:149 R.Time:17.590(Scan#:2719) MassPeaks:360
RawMode:Averaged 17.585-17.595(2718-2720) BasePeak:43.00(10432)
BG Mode:Calc. from Peak Group 1 - Event 1 Scan

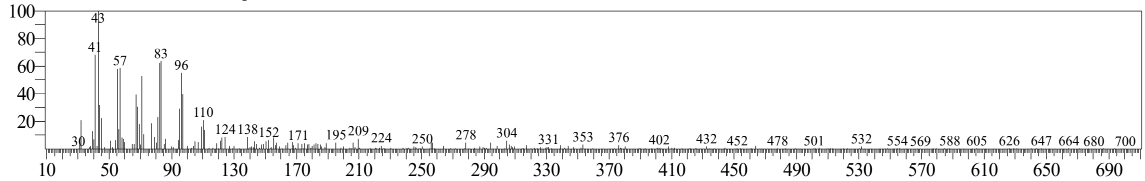

Hit#:1 Entry:93586 Library:NIST11.lib
SI:81 Formula:C18H36O CAS:638-66-4 MolWeight:268 RetIndex:1999
CompName:Octadecanal $$ Stearaldehyde $$ Octadecyl aldehyde $$ Stearyl aldehyde $$ n-Octadecanal $$

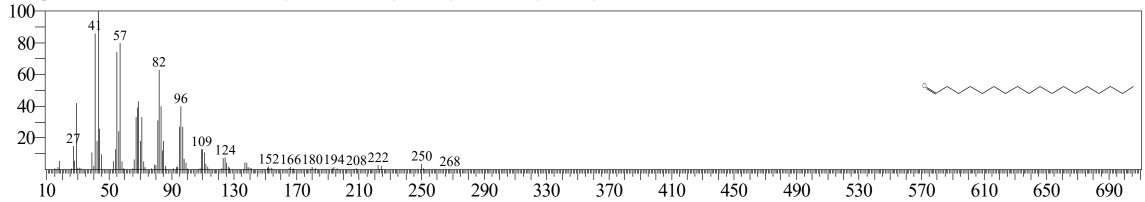

Hit#:2 Entry:82829 Library:NIST11.lib
SI:80 Formula:C17H34O CAS:0-00-0 MolWeight:254 RetIndex:1899
CompName:Heptadecanal

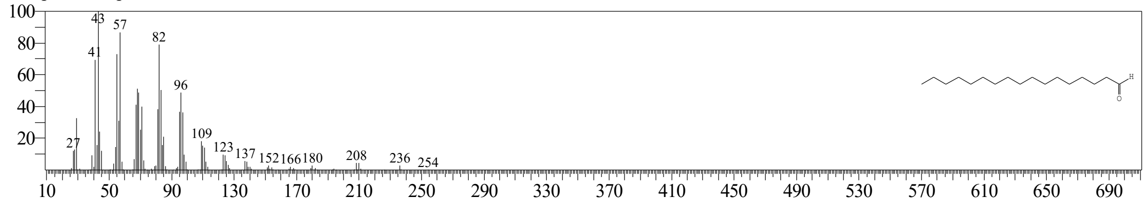

Hit#:3 Entry:104508 Library:NIST11.lib
SI:80 Formula:C19H38O CAS:67860-04-2 MolWeight:282 RetIndex:2000
CompName:Oxirane, heptadecyl- $$ 1,2-Epoxynonadecane $$ 2-Heptadecyloxirane # $$

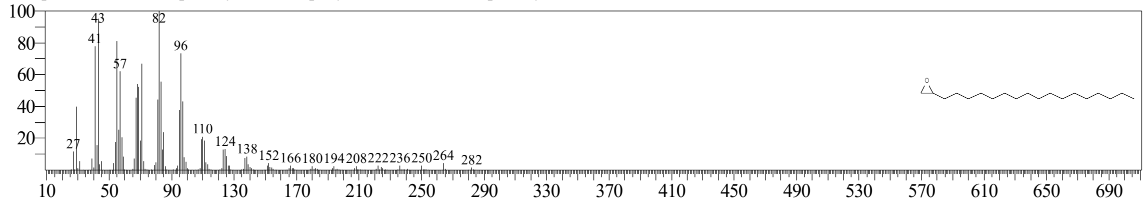

Hit#:4 Entry:72472 Library:NIST11.lib
SI:80 Formula:C16H32O CAS:629-80-1 MolWeight:240 RetIndex:1800
CompName:Hexadecanal $$ Palmitaldehyde $$ 1-Hexadecanal $$ n-Hexadecanal $$

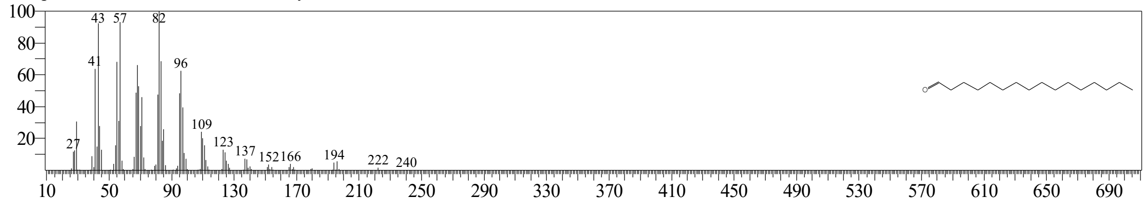

Hit#:5 Entry:62442 Library:NIST11.lib
SI:79 Formula:C15H30O CAS:2765-11-9 MolWeight:226 RetIndex:1701
CompName:Pentadecanal- $$ 1-Pentadecanal $$ n-Pentadecanal $$

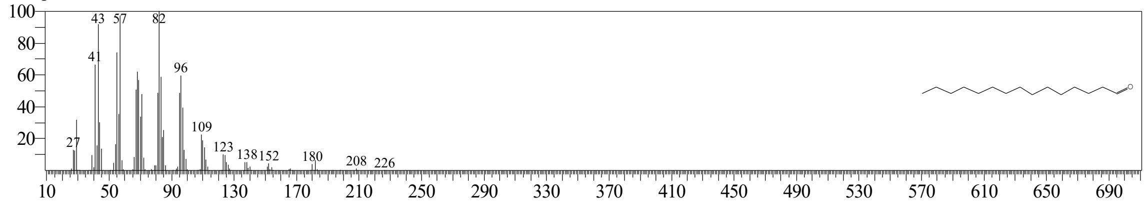

Qualitative Analysis Report

<< Target >>
Line#:150 R.Time:18.345(Scan#:2870) MassPeaks:403
RawMode:Averaged 18.340-18.350(2869-2871) BasePeak:43.05(349355)
BG Mode:Calc. from Peak Group 1 - Event 1 Scan

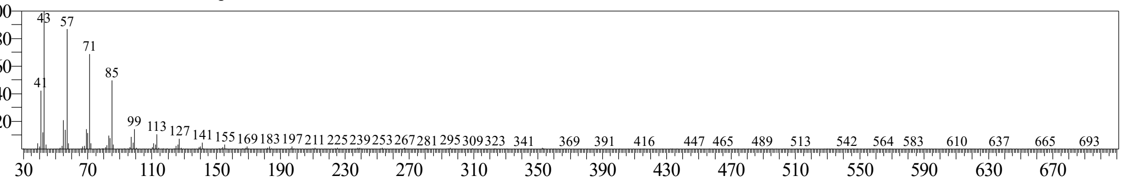

Hit#:1 Entry:157092 Library:NIST11.lib
SI:95 Formula:C25H52 CAS:7225-64-1 MolWeight:352 RetIndex:2442
CompName:Heptadecane, 9-octyl- $$ 9-Octylheptadecane # $$

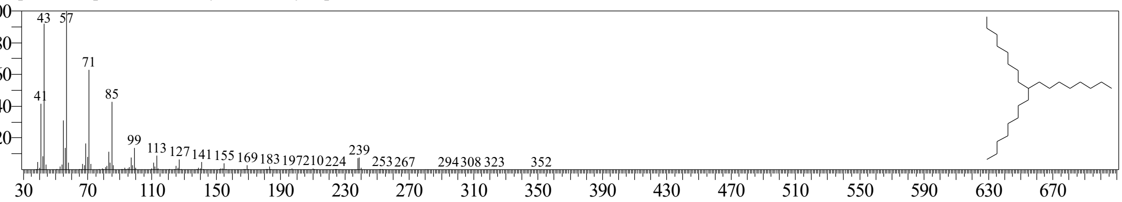

Hit#:2 Entry:93606 Library:NIST11.lib
SI:94 Formula:C19H40 CAS:61868-03-9 MolWeight:268 RetIndex:1782
CompName:Heptadecane, 2,3-dimethyl- $$ 2,3-Dimethylheptadecane # $$

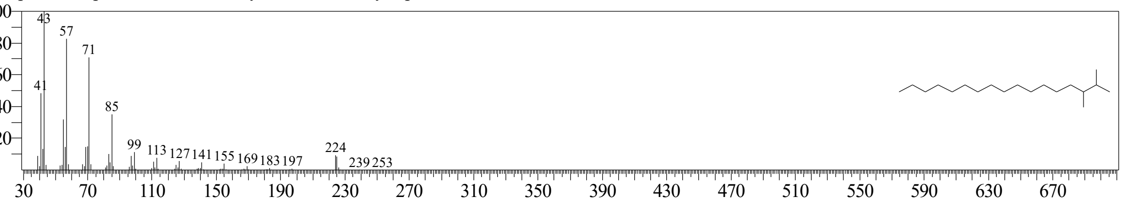

Hit#:3 Entry:165575 Library:NIST11.lib
SI:94 Formula:C26H54 CAS:55333-99-8 MolWeight:366 RetIndex:2542
CompName:Eicosane, 7-hexyl- $$ 7-n-Hexyleicosane $$ 7-Hexylicosane # $$

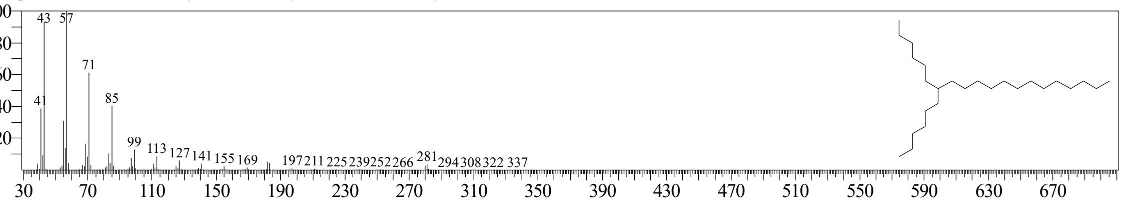

Hit#:4 Entry:165568 Library:NIST11.lib
SI:94 Formula:C26H54 CAS:55282-11-6 MolWeight:366 RetIndex:2477
CompName:Heneicosane, 11-(1-ethylpropyl)- $$ 11-(1-Ethylpropyl)heneicosane $$ 11-(3'-n-Pentyl)heneicosane $$ 11-(1-Ethylpropyl)henicosane # $$

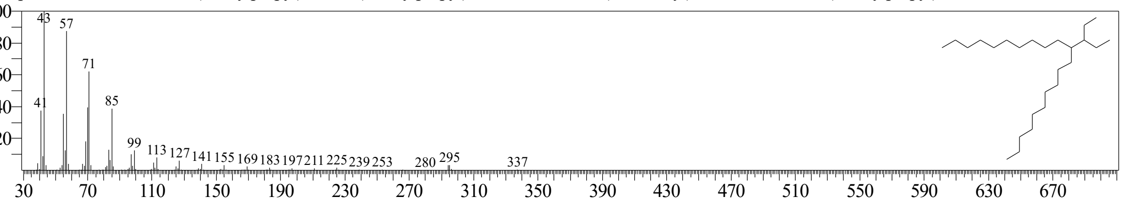

Hit#:5 Entry:165576 Library:NIST11.lib
SI:94 Formula:C26H54 CAS:13475-76-8 MolWeight:366 RetIndex:2542
CompName:Docosane, 11-butyl- $$ 11-Butyldocosane $$ Hexadecane, 5-decyl $$ 11-n-Butyldocosane $$

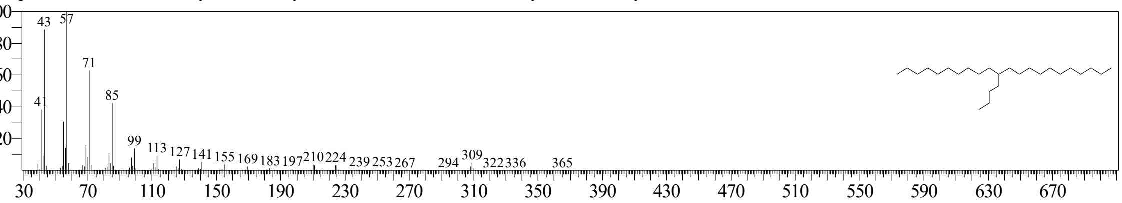

<< Target >>
Line#:151 R.Time:18.465(Scan#:2894) MassPeaks:380
RawMode:Averaged 18.460-18.470(2893-2895) BasePeak:108.05(138511)
BG Mode:Calc. from Peak Group 1 - Event 1 Scan

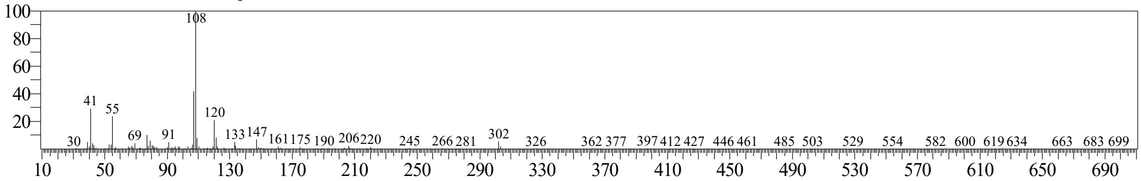

Hit#:1 Entry:29267 Library:NIST11.lib
SI:78 Formula:C12H16O CAS:156089-29-1 MolWeight:176 RetIndex:1388
CompName:Benzene, 1-methyl-4-(2-pentenyloxy)- $$ 1-Methyl-4-[(2E)-2-pentenyloxy]benzene # $$

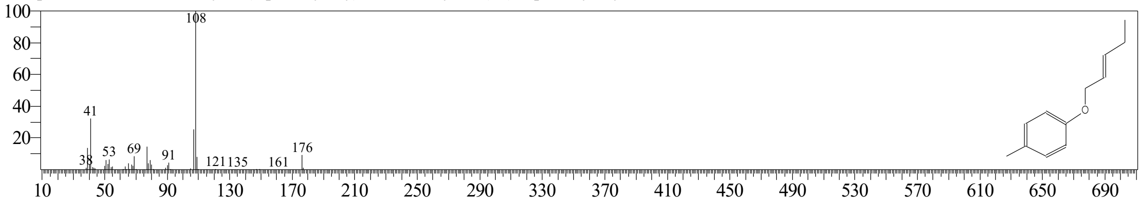

Hit#:2 Entry:46672 Library:NIST11.lib
SI:77 Formula:C15H24 CAS:0-00-0 MolWeight:204 RetIndex:1450
CompName:rotundene

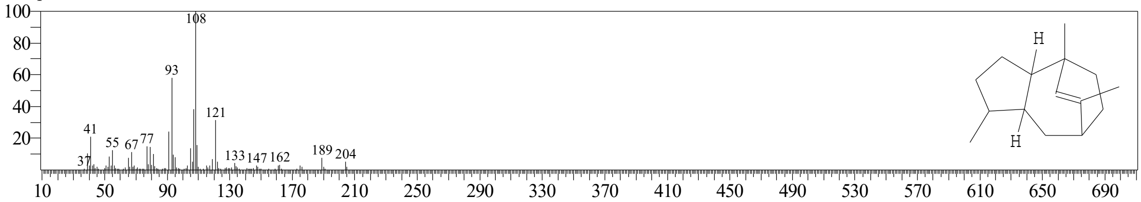

Hit#:3 Entry:29266 Library:NIST11.lib
SI:75 Formula:C12H16O CAS:23446-47-1 MolWeight:176 RetIndex:1365
CompName:Ether, 3-methyl-2-butenyl o-tolyl $$ Toluene, 2-(3-methyl-2-butenyl)oxy- $$ 1-Methyl-2-[(3-methyl-2-butenyl)oxy]benzene # $$

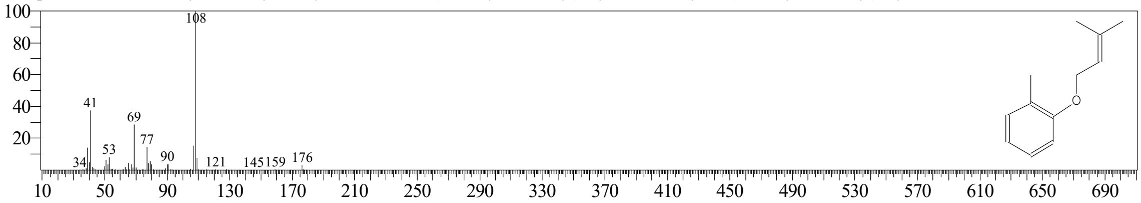

Hit#:4 Entry:15237 Library:NIST11.lib
SI:75 Formula:C9H10O2 CAS:140-39-6 MolWeight:150 RetIndex:1174
CompName:Acetic acid, 4-methylphenyl ester $$ Acetic acid, p-tolyl ester $$ p-Acetoxytoluene $$ p-Cresol acetate $$ p-Cresyl acetate $$ p-Methylphenyl aceta

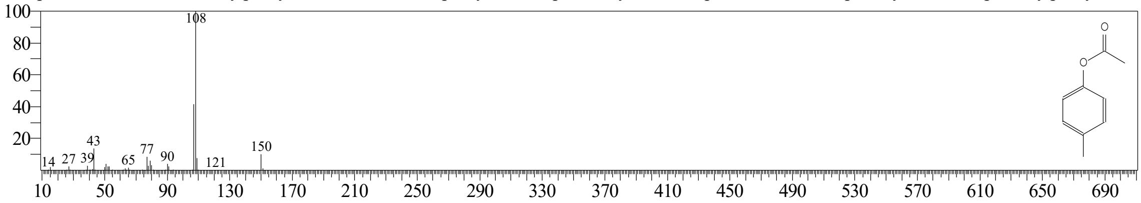

Hit#:5 Entry:66548 Library:NIST11.lib
SI:75 Formula:C15H20O2 CAS:0-00-0 MolWeight:232 RetIndex:1813
CompName:3-Cyclopentylpropionic acid, 3-methylphenyl ester

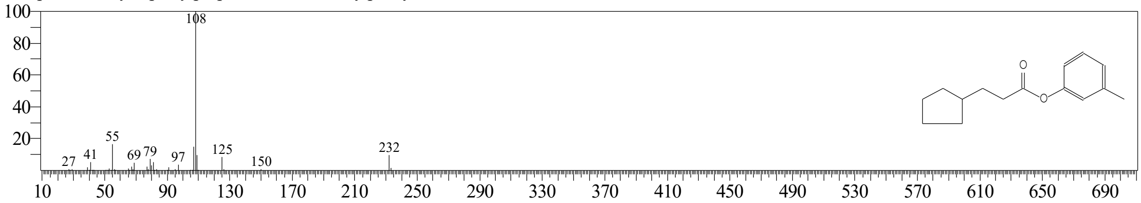

<< Target >>
Line#:152 R.Time:18.555(Scan#:2912) MassPeaks:385
RawMode:Averaged 18.550-18.560(2911-2913) BasePeak:108.05(367561)
BG Mode:Calc. from Peak Group 1 - Event 1 Scan

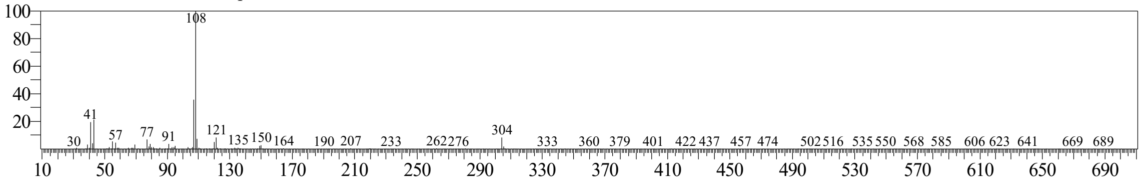

Hit#:1 Entry:121943 Library:NIST11.lib
SI:82 Formula:C21H36O CAS:501-24-6 MolWeight:304 RetIndex:2406
CompName:Phenol, 3-pentadecyl- $$ Phenol, m-pentadecyl- $$ m-Pentadecylphenol $$ Anacardol, tetrahydro- $$ Cyclogallipharaol $$ Hydrocardanol $$ Hydrc

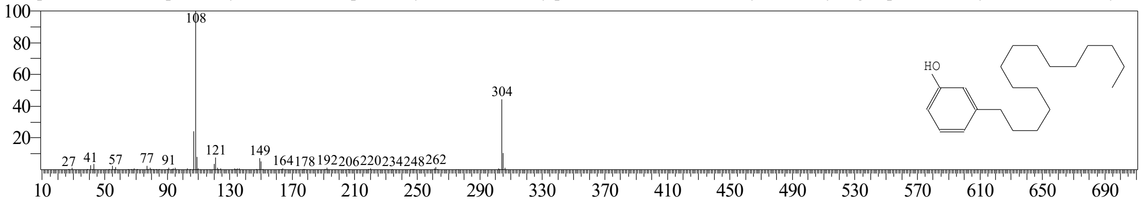

Hit#:2 Entry:79486 Library:NIST11.lib
SI:82 Formula:C14H18O4 CAS:0-00-0 MolWeight:250 RetIndex:1838
CompName:Oxalic acid, 2-methylphenyl pentyl ester

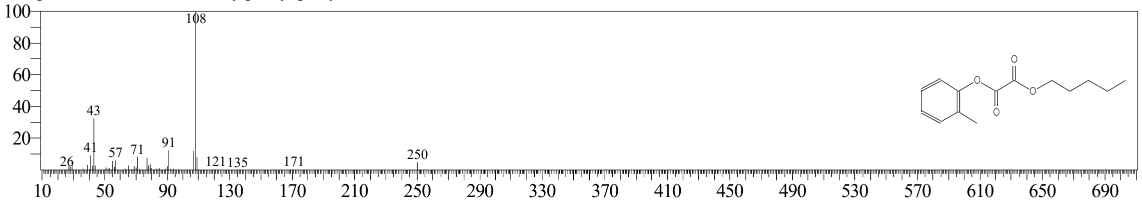

Hit#:3 Entry:15237 Library:NIST11.lib
SI:81 Formula:C9H10O2 CAS:140-39-6 MolWeight:150 RetIndex:1174
CompName:Acetic acid, 4-methylphenyl ester $$ Acetic acid, p-tolyl ester $$ p-Acetoxytoluene $$ p-Cresol acetate $$ p-Cresyl acetate $$ p-Methylphenyl aceta

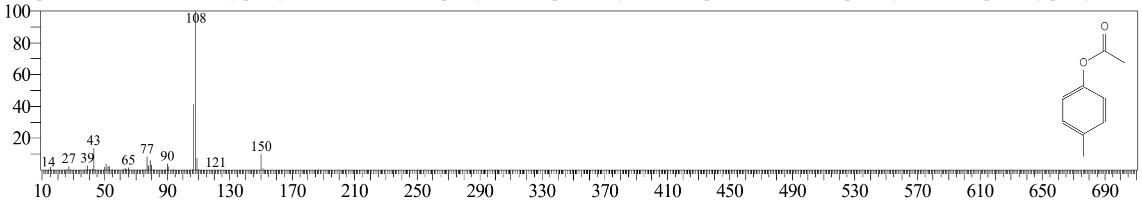

Hit#:4 Entry:123190 Library:NIST11.lib
SI:81 Formula:C18H26O4 CAS:0-00-0 MolWeight:306 RetIndex:2235
CompName:Oxalic acid, 2-methylphenyl nonyl ester

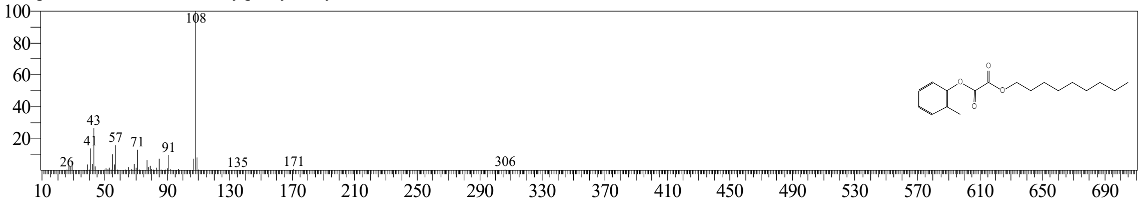

Hit#:5 Entry:30439 Library:NIST11.lib
SI:81 Formula:C12H18O CAS:85709-98-4 MolWeight:178 RetIndex:1296
CompName:Benzene, 1-(1,1-dimethylpropoxy)-4-methyl- $$ Toluene, 4-(1,1-dimethylpropoxy)- $$ 1-Methyl-4-(tert-pentyloxy)benzene # $$

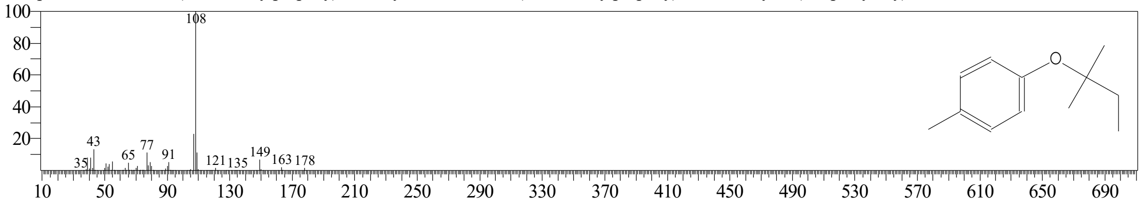

<< Target >>
Line#:153 R.Time:18.750(Scan#:2951) MassPeaks:360
RawMode:Averaged 18.745-18.755(2950-2952) BasePeak:74.00(12578)
BG Mode:Calc. from Peak Group 1 - Event 1 Scan

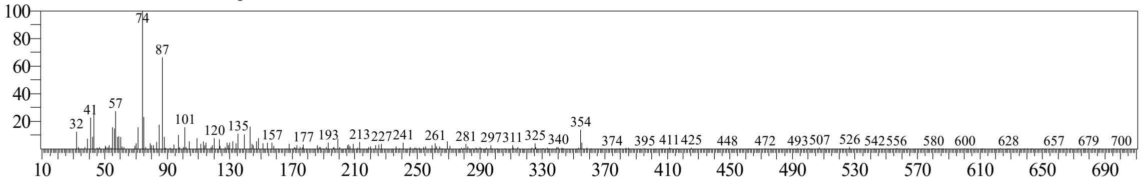

Hit#:1 Entry:158427 Library:NIST11.lib
SI:76 Formula:C23H46O2 CAS:929-77-1 MolWeight:354 RetIndex:2475
CompName:Docosanoic acid, methyl ester $$ Behenic acid, methyl ester $$ Methyl behenate $$ Methyl docosanoate $$ n-Docosanoic acid methyl ester $$ Kem

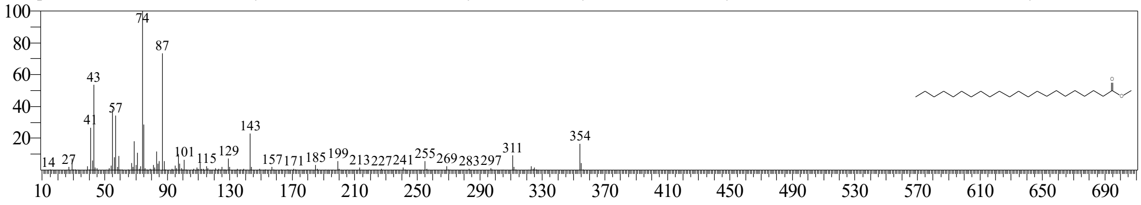

Hit#:2 Entry:166826 Library:NIST11.lib
SI:73 Formula:C24H48O2 CAS:2433-97-8 MolWeight:368 RetIndex:2574
CompName:Tricosanoic acid, methyl ester $$ Methyl tricosanoate $$

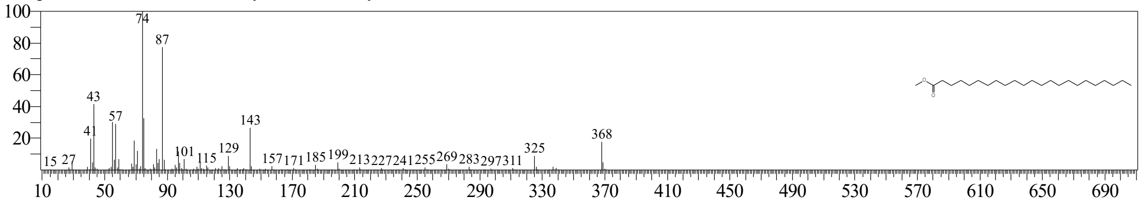

Hit#:3 Entry:73852 Library:NIST11.lib
SI:73 Formula:C15H30O2 CAS:5129-58-8 MolWeight:242 RetIndex:1615
CompName:Tridecanoic acid, 12-methyl-, methyl ester $$ Methyl isomyristate $$ Methyl 12-methyltridecanoate $$

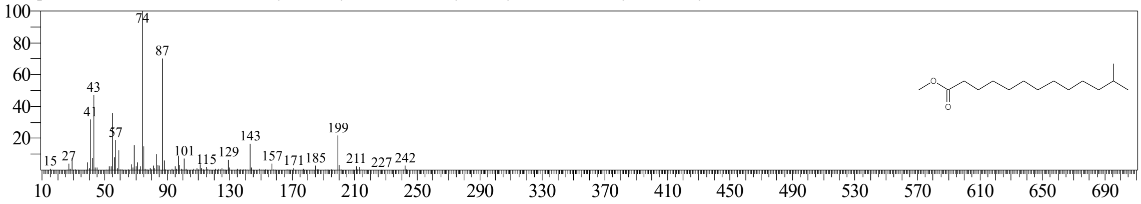

Hit#:4 Entry:117152 Library:NIST11.lib
SI:73 Formula:C19H38O2 CAS:112-61-8 MolWeight:298 RetIndex:2077
CompName:Methyl stearate $$ Octadecanoic acid, methyl ester $$ Stearic acid, methyl ester $$ n-Octadecanoic acid, methyl ester $$ Kemester 9718 $$ Methyl ı

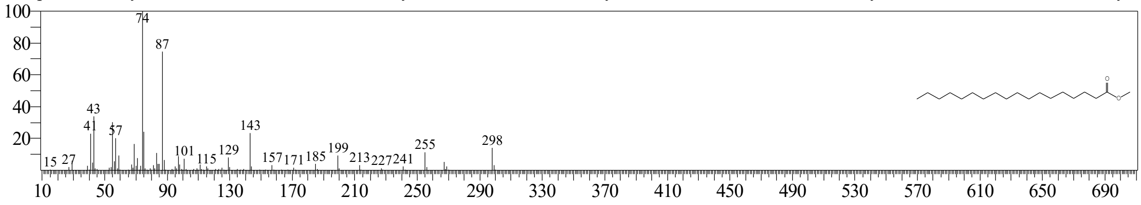

Hit#:5 Entry:106180 Library:NIST11.lib
SI:73 Formula:C18H36O2 CAS:6929-04-0 MolWeight:284 RetIndex:1914
CompName:Hexadecanoic acid, 15-methyl-, methyl ester $$ Methyl isoheptadecanoate $$ Methyl 15-methylhexadecanoate $$

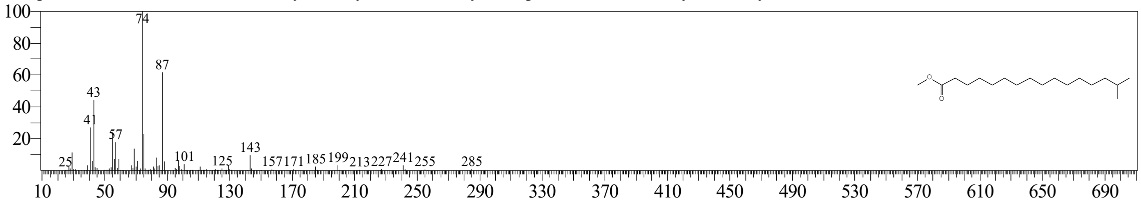

<< Target >>
Line#:154 R.Time:18.845(Scan#:2970) MassPeaks:425
RawMode:Averaged 18.840-18.850(2969-2971) BasePeak:149.00(80682)
BG Mode:Calc. from Peak Group 1 - Event 1 Scan

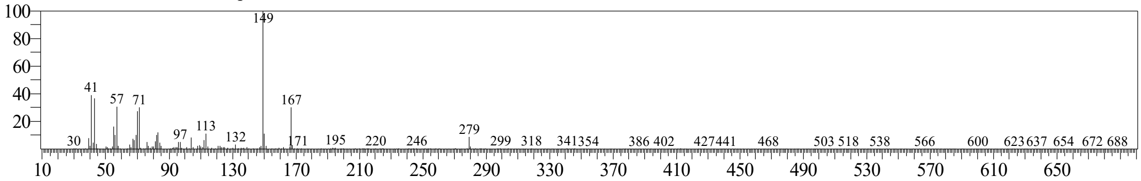

Hit#:1 Entry:178252 Library:NIST11.lib
SI:87 Formula:C24H38O4 CAS:131-20-4 MolWeight:390 RetIndex:2704
CompName:Diisooctyl phthalate $$ Bis(6-methylheptyl) phthalate $$ 1,2-Benzenedicarboxylic acid, bis(6-methylheptyl) ester $$ Phthalic acid, bis(6-methylhep

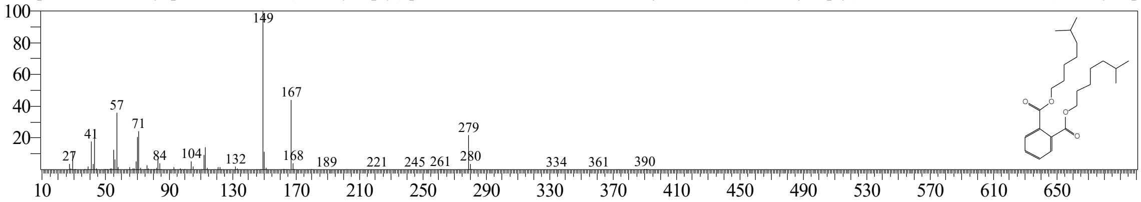

Hit#:2 Entry:178251 Library:NIST11.lib
SI:86 Formula:C24H38O4 CAS:117-81-7 MolWeight:390 RetIndex:2704
CompName:Bis(2-ethylhexyl) phthalate $$ Phthalic acid, bis(2-ethylhexyl) ester $$ Bis(2-ethylhexyl) 1,2-benzenedicarboxylate $$ Bisoflex 81 $$ Compound 8

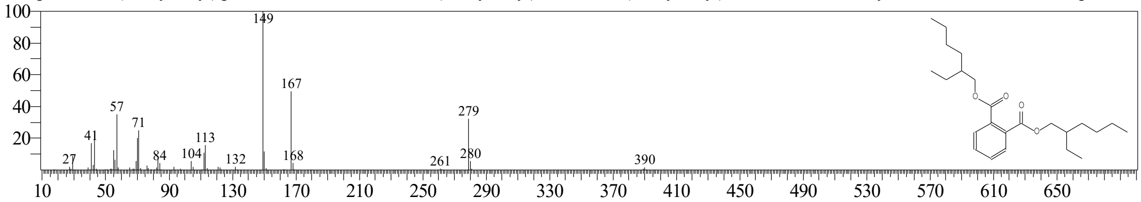

Hit#:3 Entry:126456 Library:NIST11.lib
SI:81 Formula:C20H26N2O CAS:2656-44-2 MolWeight:310 RetIndex:2510
CompName:Aspidofractinine-3-methanol, (2.alpha.,3.beta.,5.alpha.)- $$ Kopsinyl alcohol $$ Aspidofractinin-3-ylmethanol # $$

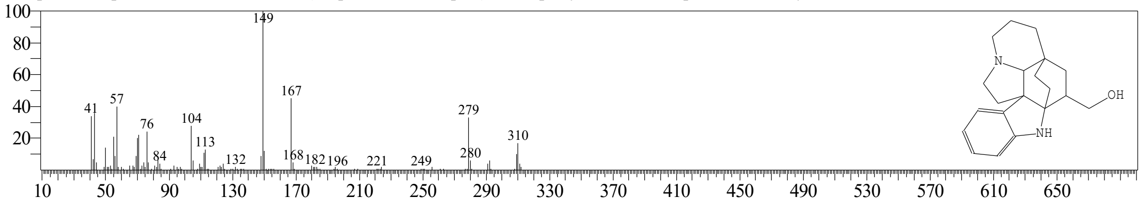

Hit#:4 Entry:178249 Library:NIST11.lib
SI:81 Formula:C24H38O4 CAS:0-00-0 MolWeight:390 RetIndex:2704
CompName:Phthalic acid, di(2-propylpentyl) ester

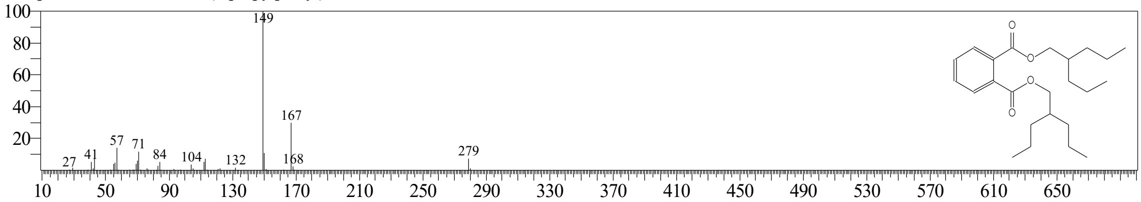

Hit#:5 Entry:203169 Library:NIST11.lib
SI:80 Formula:C28H25NO7 CAS:127611-84-1 MolWeight:487 RetIndex:4187
CompName:3',8,8'-Trimethoxy-3-piperidyl-2,2'-binaphthalene-1,1',4,4'-tetrone $$ 3',8,8'-Trimethoxy-3-piperidin-1-yl-2,2'-binaphthyl-1,1',4,4'-tetrone $$

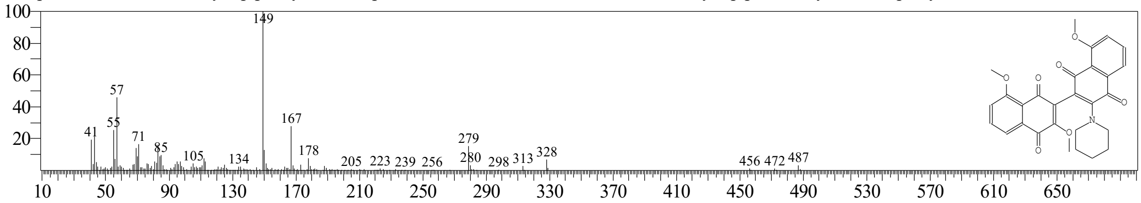

<< Target >>
Line#:155 R.Time:18.910(Scan#:2983) MassPeaks:380
RawMode:Averaged 18.905-18.915(2982-2984) BasePeak:85.10(4909)
BG Mode:Calc. from Peak Group 1 - Event 1 Scan

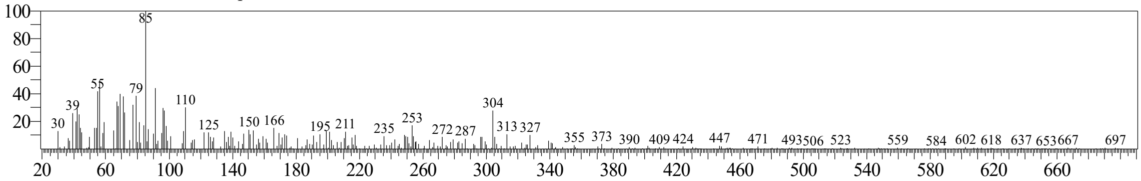

Hit#:1 Entry:34388 Library:NIST11.lib
SI:64 Formula:C9H15NO3 CAS:0-00-0 MolWeight:185 RetIndex:1429
CompName:1-(Cyclopropyl-nitro-methyl)-cyclopentanol

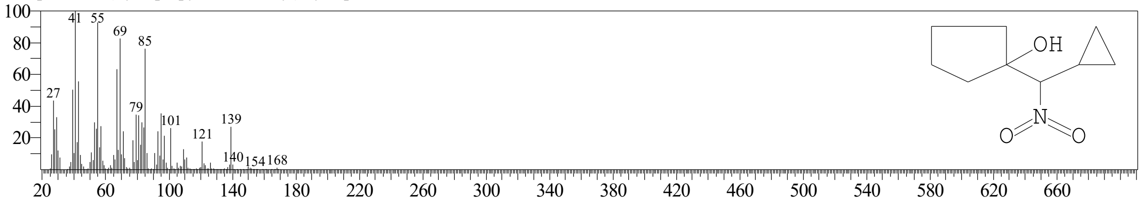

Hit#:2 Entry:17596 Library:NIST11.lib
SI:64 Formula:C10H18O CAS:7316-84-9 MolWeight:154 RetIndex:1125
CompName:NSC 408941 $$ 4,6,6-Trimethyl-bicyclo[3.1.1]heptan-2-ol $$

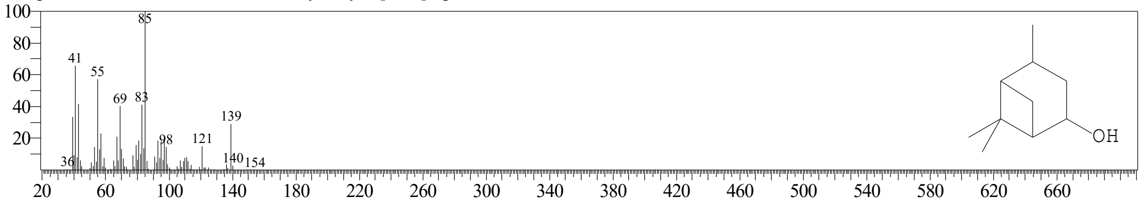

Hit#:3 Entry:12971 Library:NIST11.lib
SI:64 Formula:C8H16O2 CAS:59013-83-1 MolWeight:144 RetIndex:1106
CompName:3-cis-Methoxy-5-trans-methyl-1R-cyclohexanol $$ 3-cis-Methoxy-5-trans-methyl-1(R)-cyclohexanol $$ 3-(Z)-Methoxy-5-(E)-methylcyclohexanol

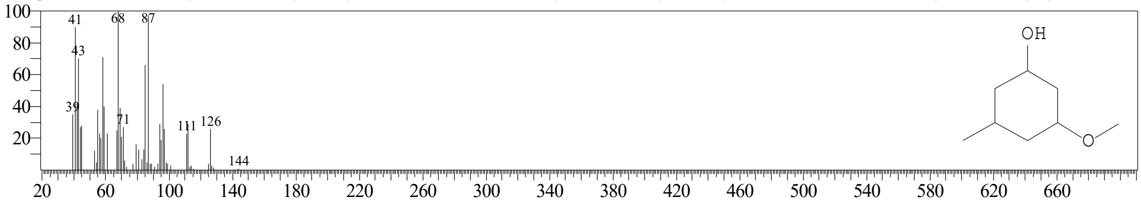

Hit#:4 Entry:32754 Library:NIST11.lib
SI:62 Formula:C11H18O2 CAS:141033-65-0 MolWeight:182 RetIndex:1337
CompName:Cyclohexanone, 2,2-dimethyl-5-(3-methyloxiranyl)-, [2.alpha.(R*),3.alpha.]-(.+-.)- $$ 2,2-Dimethyl-5-(3-methyl-2-oxiranyl)cyclohexanone # $$

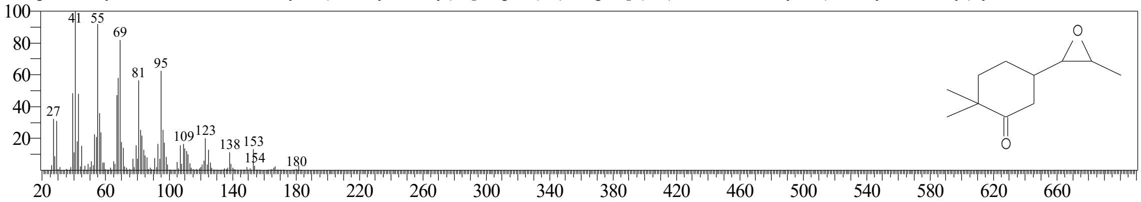

Hit#:5 Entry:33881 Library:NIST11.lib
SI:62 Formula:C11H20O2 CAS:35936-15-3 MolWeight:184 RetIndex:1435
CompName:Cyclopropaneacetic acid, 2-hexyl- $$ (2-Hexylcyclopropyl)acetic acid # $$

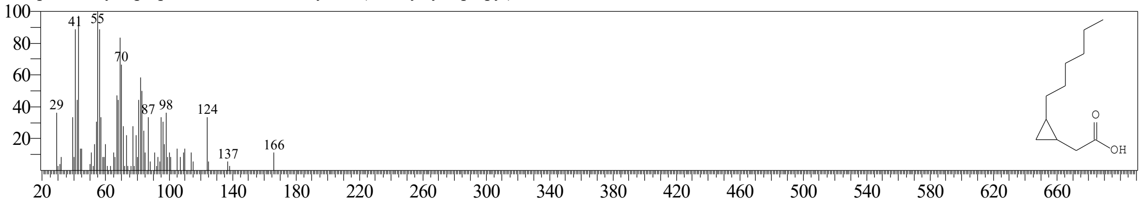

Qualitative Analysis Report

<< Target >>
Line#:156 R.Time:19.215(Scan#:3044) MassPeaks:373
RawMode:Averaged 19.210-19.220(3043-3045) BasePeak:43.00(11257)
BG Mode:Calc. from Peak Group 1 - Event 1 Scan

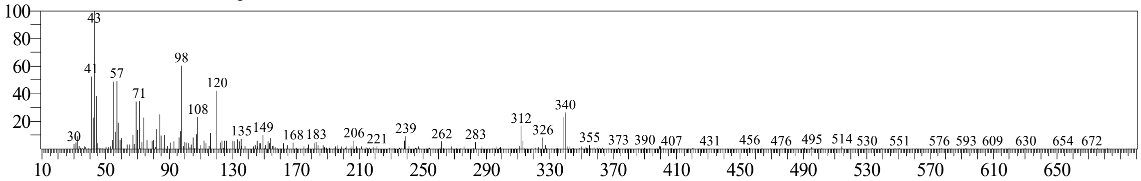

Hit#:1 Entry:211894 Library:NIST11.lib
SI:69 Formula:C37H74NO8P CAS:3026-45-7 MolWeight:691 RetIndex:0
CompName:Hexadecanoic acid, 1-[[[(2-aminoethoxy)hydroxyphosphinyl]oxy]methyl]-1,2-ethanediyl ester $$ Palmitin, 1,2-di-, 2-aminoethyl hydrogen phospha

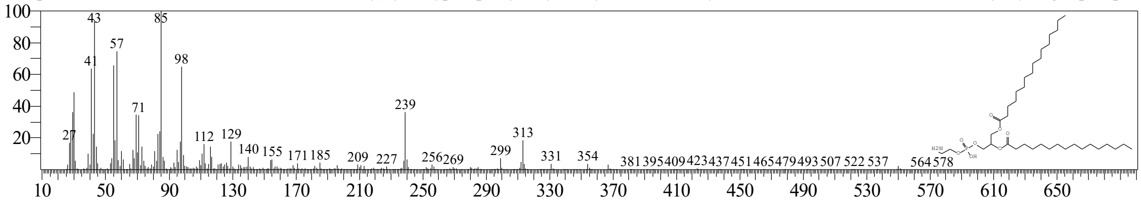

Hit#:2 Entry:25266 Library:NIST11.lib
SI:68 Formula:C11H23N CAS:14924-95-9 MolWeight:169 RetIndex:1268
CompName:n-Nonane, 1-[1-cycloazapropyl]- $$ 1-Nonylaziridine # $$

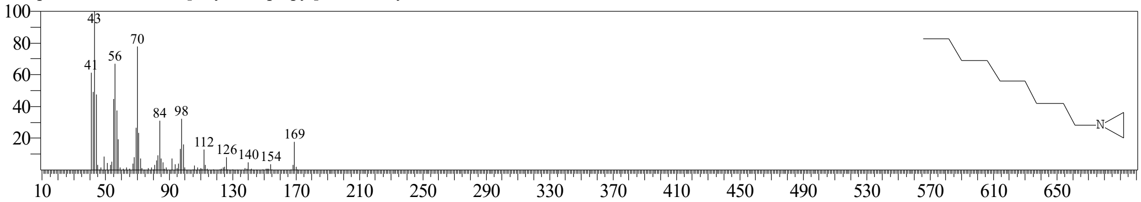

Hit#:3 Entry:124682 Library:NIST11.lib
SI:68 Formula:C17H31F3O CAS:141022-99-3 MolWeight:308 RetIndex:1681
CompName:1,1,1-Trifluoroheptadecen-2-one $$ 1,1,1-Trifluoro-2-heptadecanone # $$

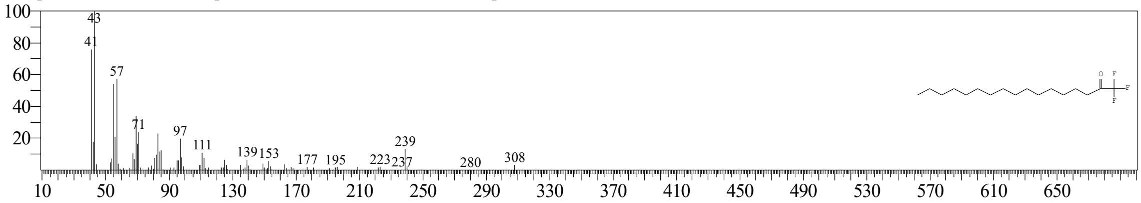

Hit#:4 Entry:13836 Library:NIST11.lib
SI:67 Formula:C9H19F CAS:463-18-3 MolWeight:146 RetIndex:889
CompName:1-Fluorononane $$ n-Nonyl fluoride $$ Nonane, 1-fluoro- $$

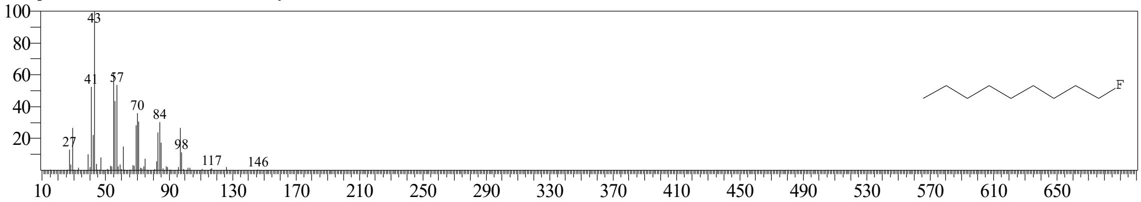

Hit#:5 Entry:141605 Library:NIST11.lib
SI:67 Formula:C19H38O4 CAS:23470-00-0 MolWeight:330 RetIndex:2498
CompName:Hexadecanoic acid, 2-hydroxy-1-(hydroxymethyl)ethyl ester $$ Palmitin, 2-mono- $$ Palmitic acid .beta.-monoglyceride $$ 2-Hexadecanoyl glycero

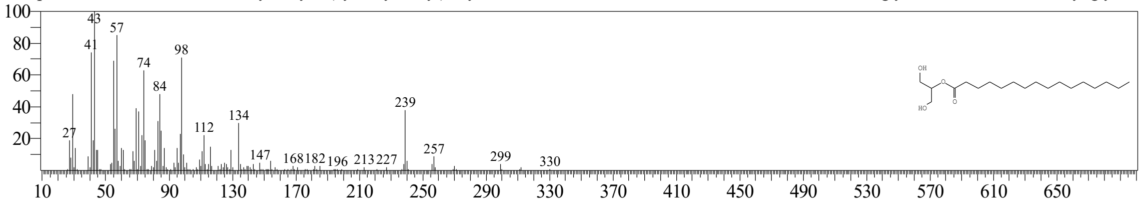

<< Target >>
Line#:157 R.Time:19.715(Scan#:3144) MassPeaks:411
RawMode:Averaged 19.710-19.720(3143-3145) BasePeak:43.05(340395)
BG Mode:Calc. from Peak Group 1 - Event 1 Scan

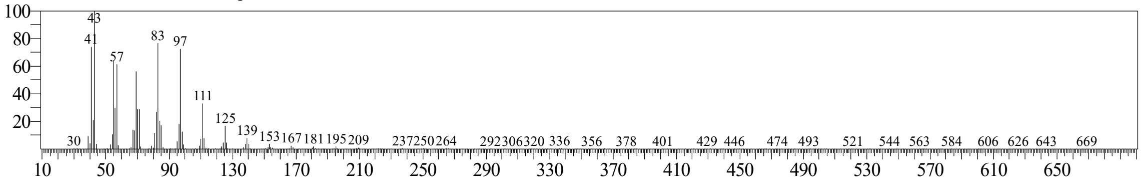

Hit#:1 Entry:81317 Library:NIST11.lib
SI:93 Formula:C17H32O CAS:0-00-0 MolWeight:252 RetIndex:1907
CompName:E-15-Heptadecenal $$ (15E)-15-Heptadecenal # $$

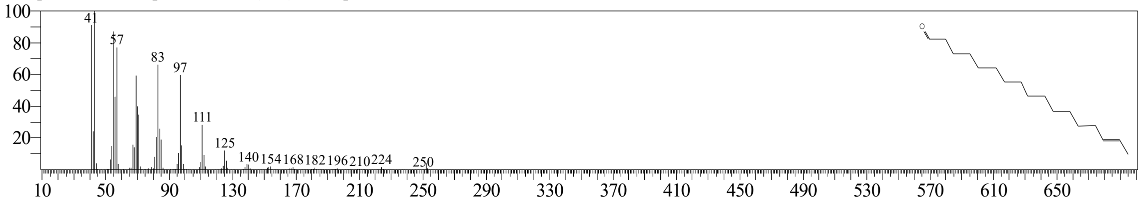

Hit#:2 Entry:147159 Library:NIST11.lib
SI:93 Formula:C18H33F3O2 CAS:6222-03-3 MolWeight:338 RetIndex:1812
CompName:Trifluoroacetoxy hexadecane $$ Hexadecyl trifluoroacetate $$ Hexadecyl 2,2,2-trifluoroacetate $$ 1-Hexadecanol, trifluoroacetate $$ Acetic acid, tr

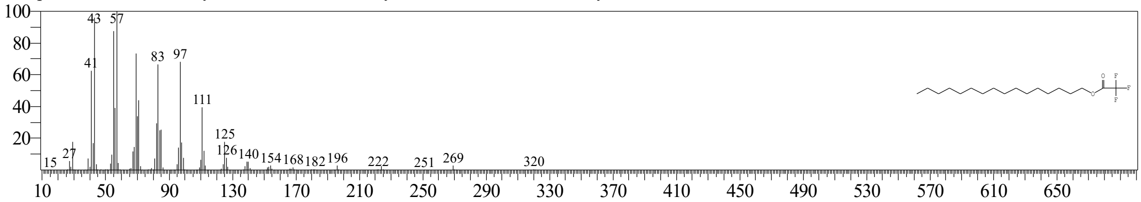

Hit#:3 Entry:155811 Library:NIST11.lib
SI:93 Formula:C25H50 CAS:0-00-0 MolWeight:350 RetIndex:2514
CompName:Z-12-Pentacosene $$ (12Z)-12-Pentacosene # $$

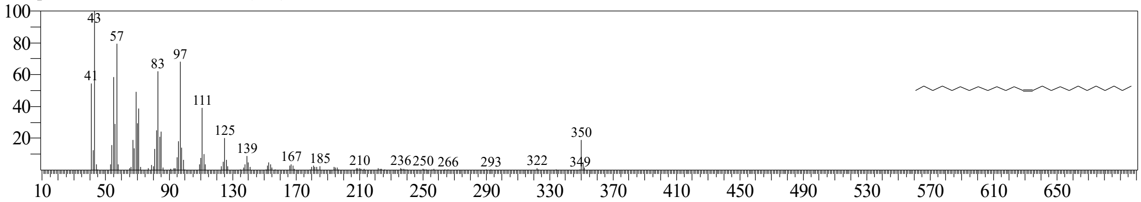

Hit#:4 Entry:82687 Library:NIST11.lib
SI:93 Formula:C16H30O2 CAS:56221-91-1 MolWeight:254 RetIndex:1769
CompName:13-Tetradecen-1-ol acetate $$ 13-Tetradecenyl acetate # $$

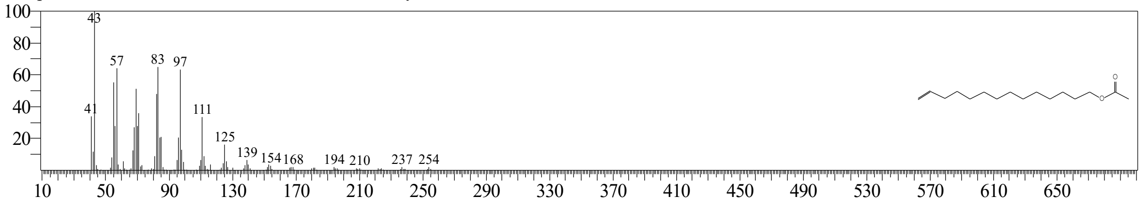

Hit#:5 Entry:114020 Library:NIST11.lib
SI:93 Formula:C21H42 CAS:95008-11-0 MolWeight:294 RetIndex:2117
CompName:10-Heneicosene (c,t) $$ (10E)-10-Henicosene # $$

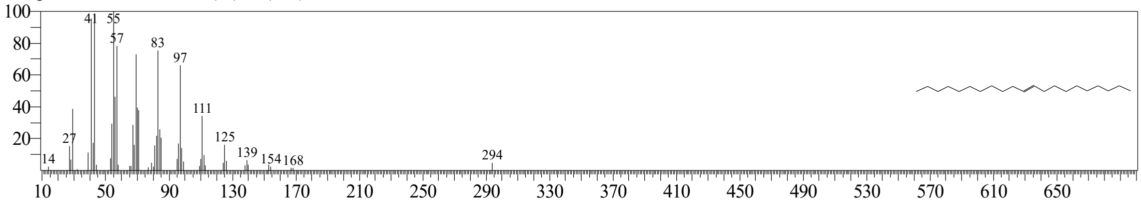

<< Target >>
Line#:158 R.Time:19.770(Scan#:3155) MassPeaks:362
RawMode:Averaged 19.765-19.775(3154-3156) BasePeak:43.10(60341)
BG Mode:Calc. from Peak Group 1 - Event 1 Scan

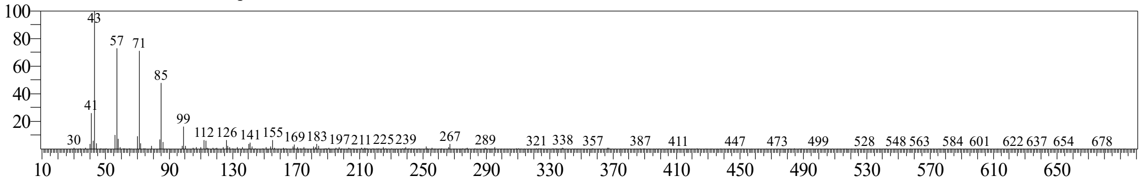

Hit#:1 Entry:34144 Library:NIST11.lib
SI:87 Formula:C13H28 CAS:6117-97-1 MolWeight:184 RetIndex:1249
CompName:Dodecane, 4-methyl- $$ 4-Methyldodecane $$

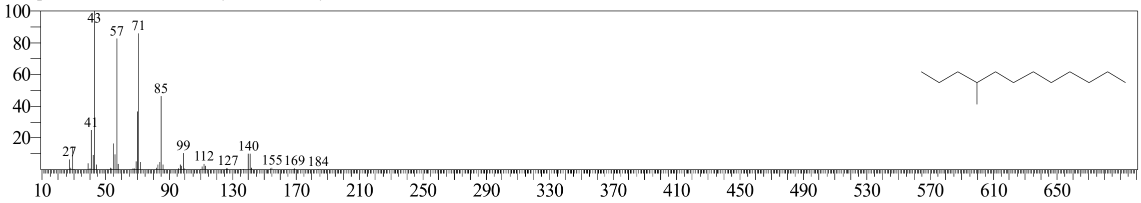

Hit#:2 Entry:34136 Library:NIST11.lib
SI:86 Formula:C13H28 CAS:17301-32-5 MolWeight:184 RetIndex:1185
CompName:Undecane, 4,7-dimethyl- $$ 4,7-Dimethylundecane # $$

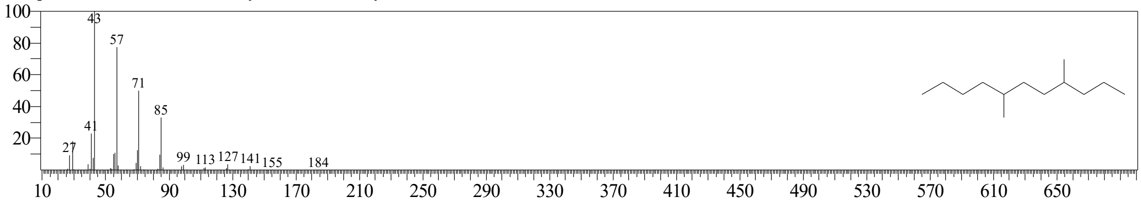

Hit#:3 Entry:34139 Library:NIST11.lib
SI:86 Formula:C13H28 CAS:62238-12-4 MolWeight:184 RetIndex:1121
CompName:Decane, 2,3,6-trimethyl- $$ 2,3,6-Trimethyldecane # $$

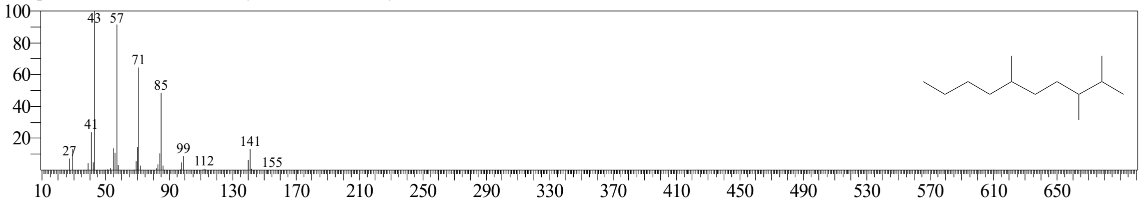

Hit#:4 Entry:34131 Library:NIST11.lib
SI:85 Formula:C13H28 CAS:17301-25-6 MolWeight:184 RetIndex:1185
CompName:Undecane, 2,8-dimethyl- $$ 2,8-Dimethylundecane # $$

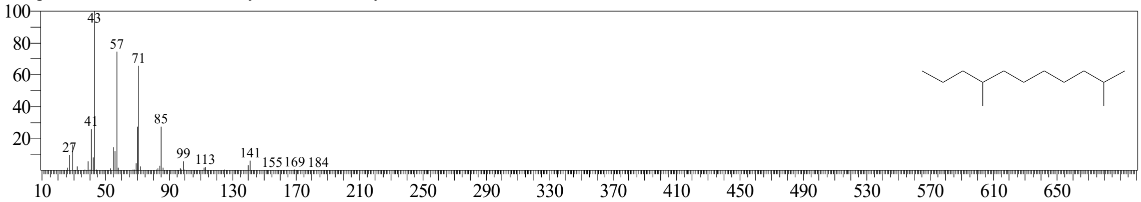

Hit#:5 Entry:34140 Library:NIST11.lib
SI:85 Formula:C13H28 CAS:62238-13-5 MolWeight:184 RetIndex:1121
CompName:Decane, 2,3,7-trimethyl- $$ 2,3,7-Trimethyldecane # $$

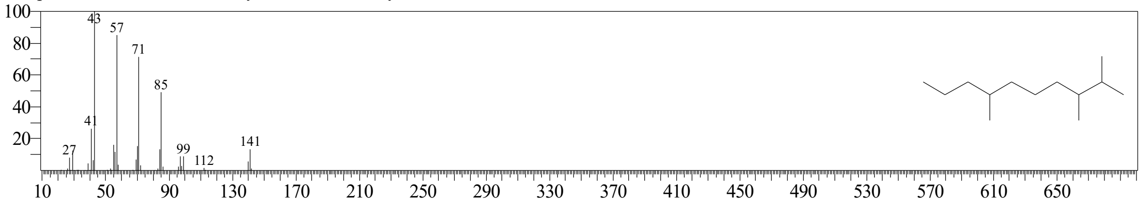

<< Target >>
Line#:159 R.Time:20.420(Scan#:3285) MassPeaks:380
RawMode:Averaged 20.415-20.425(3284-3286) BasePeak:43.05(58406)
BG Mode:Calc. from Peak Group 1 - Event 1 Scan

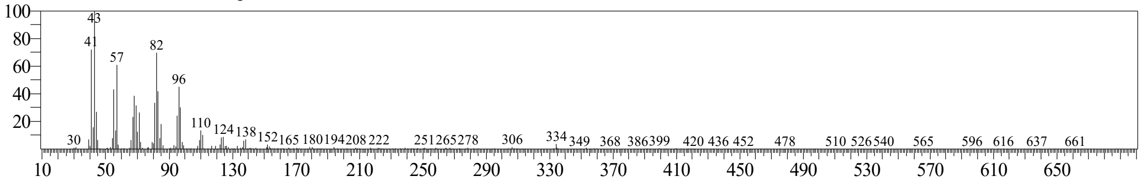

Hit#:1 Entry:93586 Library:NIST11.lib
SI:93 Formula:C18H36O CAS:638-66-4 MolWeight:268 RetIndex:1999
CompName:Octadecanal $$ Stearaldehyde $$ Octadecyl aldehyde $$ Stearyl aldehyde $$ n-Octadecanal $$

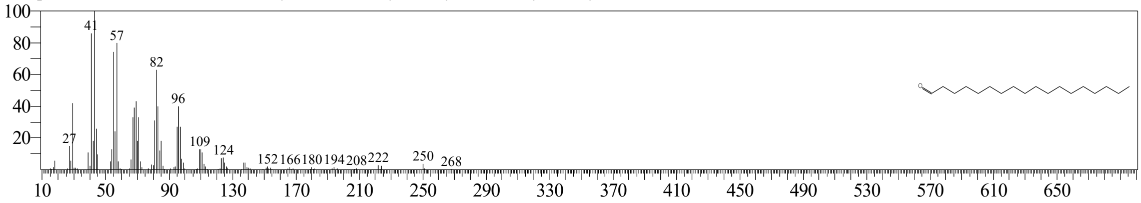

Hit#:2 Entry:82829 Library:NIST11.lib
SI:91 Formula:C17H34O CAS:0-00-0 MolWeight:254 RetIndex:1899
CompName:Heptadecanal

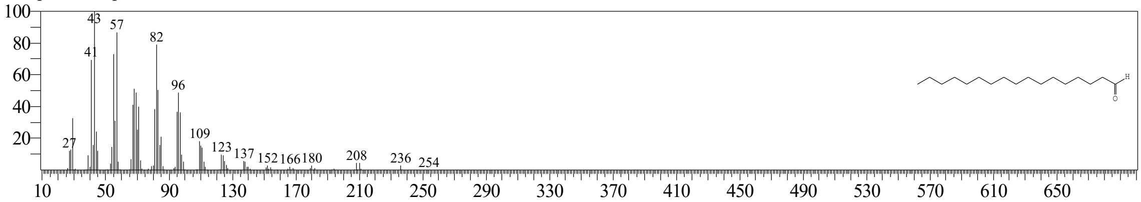

Hit#:3 Entry:42885 Library:NIST11.lib
SI:90 Formula:C13H26O CAS:10486-19-8 MolWeight:198 RetIndex:1502
CompName:Tridecanal $$ n-Tridecylaldehyde $$ Tridecanaldehyde $$ Tridecyl aldehyde $$ 1-Tridecanal $$ Tridecane aldehyde $$ n-Tridecanal $$

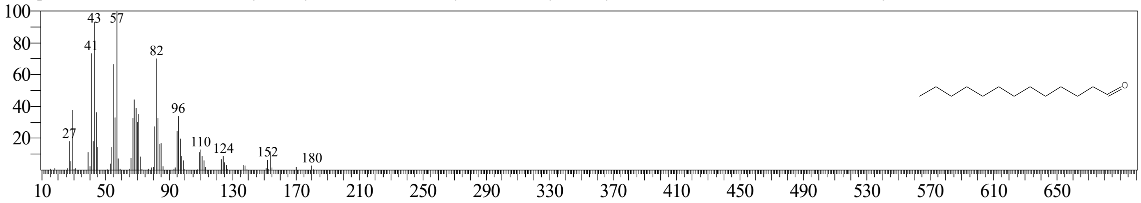

Hit#:4 Entry:93594 Library:NIST11.lib
SI:90 Formula:C18H36O CAS:0-00-0 MolWeight:268 RetIndex:2061
CompName:Z-2-Octadecen-1-ol $$ (2Z)-2-Octadecen-1-ol # $$

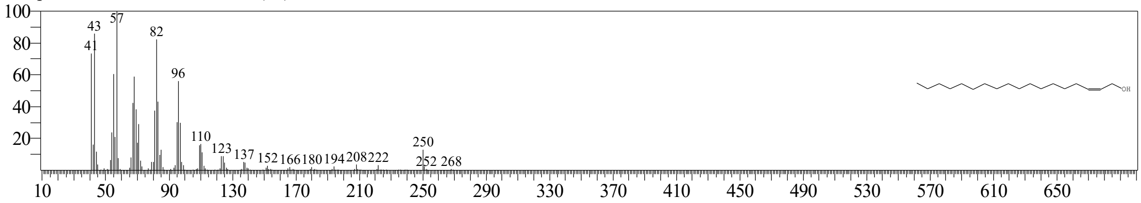

Hit#:5 Entry:52426 Library:NIST11.lib
SI:89 Formula:C14H28O CAS:0-00-0 MolWeight:212 RetIndex:1664
CompName:E-2-Tetradecen-1-ol $$ (2E)-2-Tetradecen-1-ol # $$

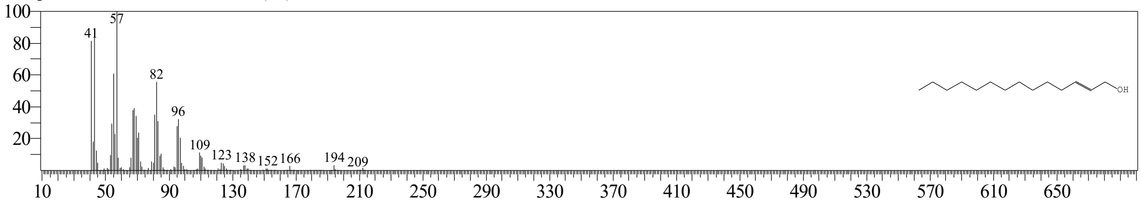

<< Target >>
Line#:160 R.Time:21.635(Scan#:3528) MassPeaks:446
RawMode:Averaged 21.630-21.640(3527-3529) BasePeak:43.10(702364)
BG Mode:Calc. from Peak Group 1 - Event 1 Scan

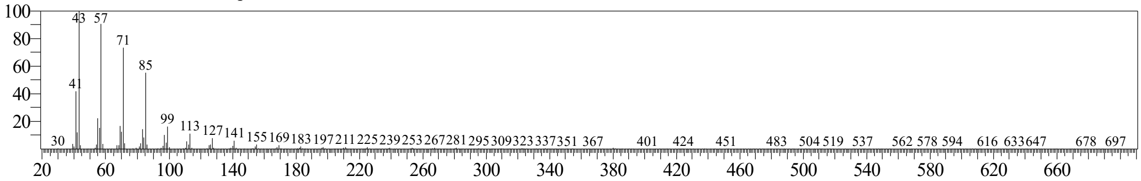

Hit#:1 Entry:104535 Library:NIST11.lib
SI:94 Formula:C20H42 CAS:112-95-8 MolWeight:282 RetIndex:2009
CompName:Eicosane $$ n-Eicosane $$ Icosane # $$ n-Icosane $$

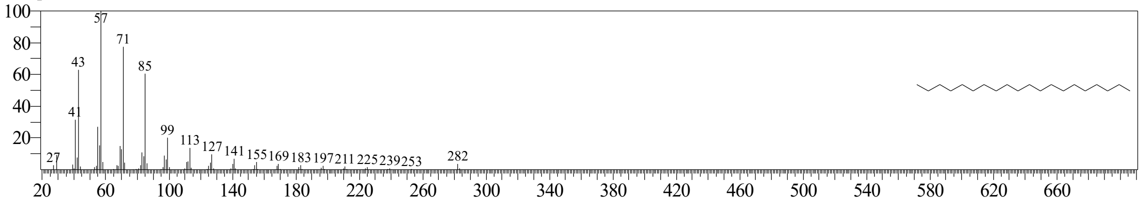

Hit#:2 Entry:165575 Library:NIST11.lib
SI:94 Formula:C26H54 CAS:55333-99-8 MolWeight:366 RetIndex:2542
CompName:Eicosane, 7-hexyl- $$ 7-n-Hexyleicosane $$ 7-Hexylicosane # $$

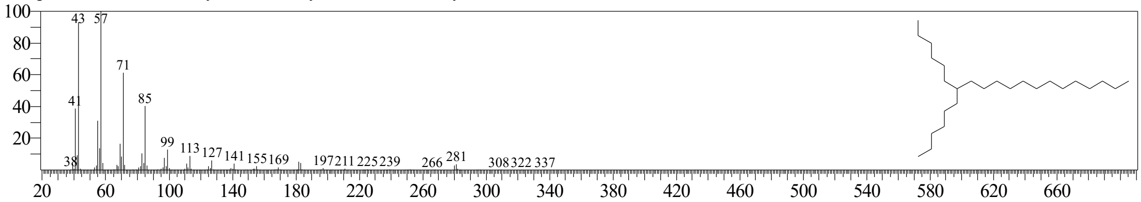

Hit#:3 Entry:157092 Library:NIST11.lib
SI:94 Formula:C25H52 CAS:7225-64-1 MolWeight:352 RetIndex:2442
CompName:Heptadecane, 9-octyl- $$ 9-Octylheptadecane # $$

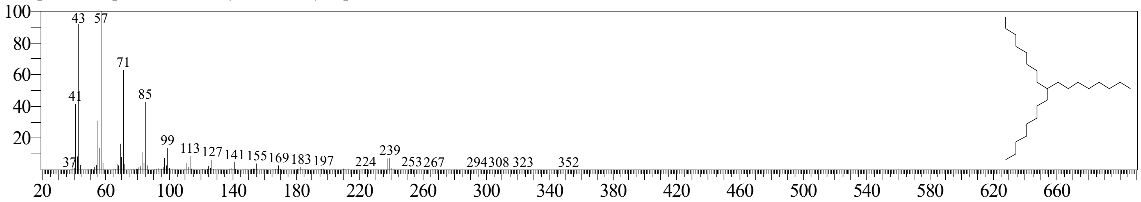

Hit#:4 Entry:165568 Library:NIST11.lib
SI:94 Formula:C26H54 CAS:55282-11-6 MolWeight:366 RetIndex:2477
CompName:Heneicosane, 11-(1-ethylpropyl)- $$ 11-(1-Ethylpropyl)heneicosane $$ 11-(3'-n-Pentyl)heneicosane $$ 11-(1-Ethylpropyl)henicosane # $$

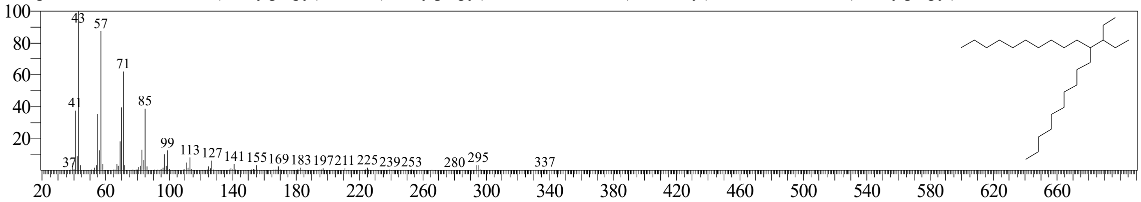

Hit#:5 Entry:93606 Library:NIST11.lib
SI:94 Formula:C19H40 CAS:61868-03-9 MolWeight:268 RetIndex:1782
CompName:Heptadecane, 2,3-dimethyl- $$ 2,3-Dimethylheptadecane # $$

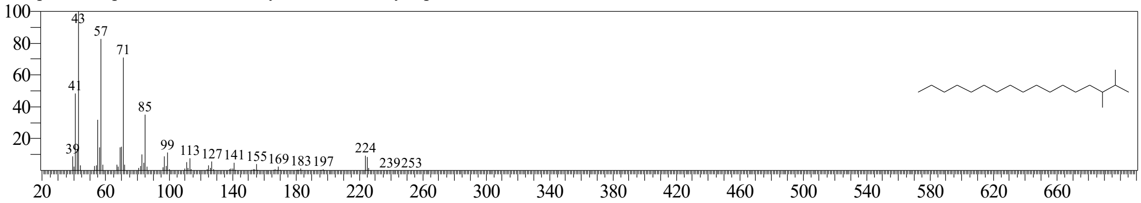

<< Target >>
Line#:161 R.Time:21.900(Scan#:3581) MassPeaks:368
RawMode:Averaged 21.895-21.905(3580-3582) BasePeak:108.05(59666)
BG Mode:Calc. from Peak Group 1 - Event 1 Scan

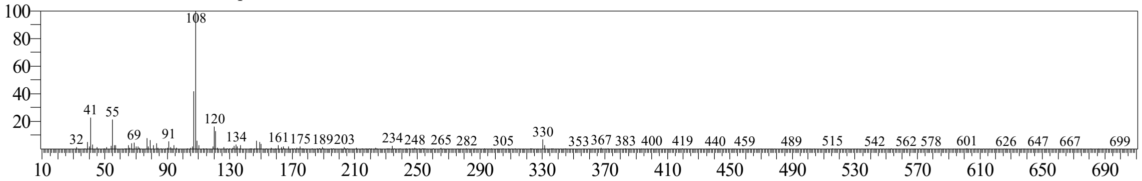

Hit#:1 Entry:22341 Library:NIST11.lib
SI:74 Formula:C10H16N2 CAS:91010-41-2 MolWeight:164 RetIndex:1228
CompName:2-Isoamyl-6-methylpyrazine $$ 2-Isopentyl-6-methylpyrazine # $$ 2-Methyl-6-isopentylpyrazine $$ 6-Methyl-2-(3-methylbutyl)-pyrazine $$ Pyrazi

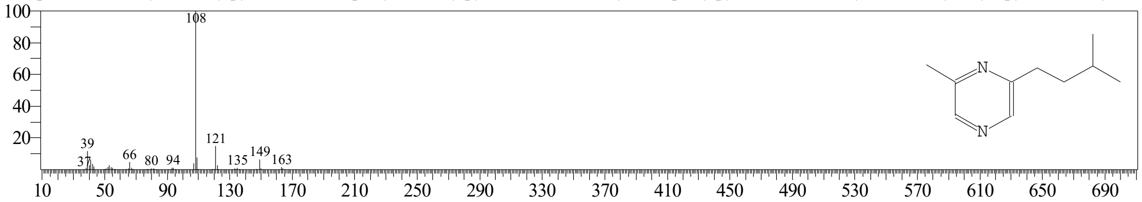

Hit#:2 Entry:29267 Library:NIST11.lib
SI:73 Formula:C12H16O CAS:156089-29-1 MolWeight:176 RetIndex:1388
CompName:Benzene, 1-methyl-4-(2-pentenyloxy)- $$ 1-Methyl-4-[(2E)-2-pentenyloxy]benzene # $$

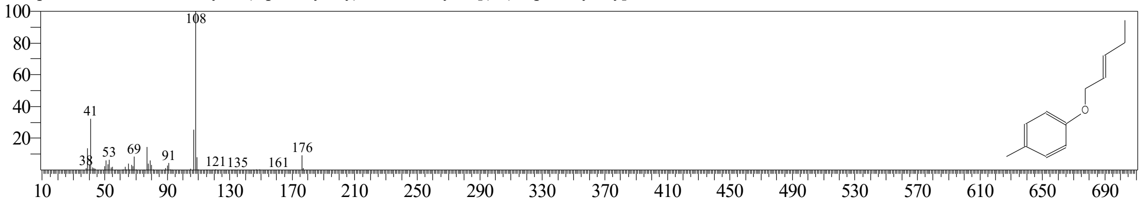

Hit#:3 Entry:30439 Library:NIST11.lib
SI:73 Formula:C12H18O CAS:85709-98-4 MolWeight:178 RetIndex:1296
CompName:Benzene, 1-(1,1-dimethylpropoxy)-4-methyl- $$ Toluene, 4-(1,1-dimethylpropoxy)- $$ 1-Methyl-4-(tert-pentyloxy)benzene # $$

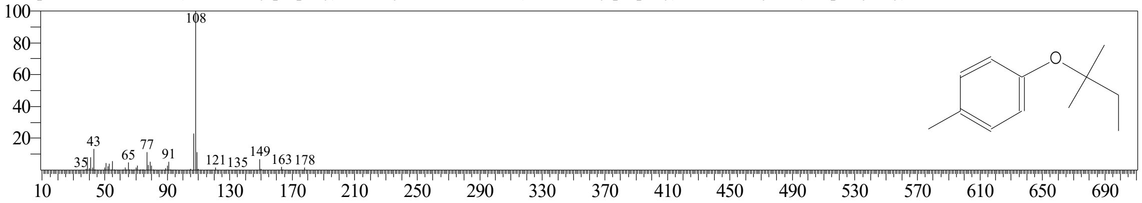

Hit#:4 Entry:22425 Library:NIST11.lib
SI:72 Formula:C11H16O CAS:10519-06-9 MolWeight:164 RetIndex:1281
CompName:p-Butoxytoluene $$ n-Butyl p-tolyl ether $$ 4-(n-Butoxy)toluene $$ Benzene, 1-butoxy-4-methyl- $$ 1-Butoxy-4-methylbenzene # $$

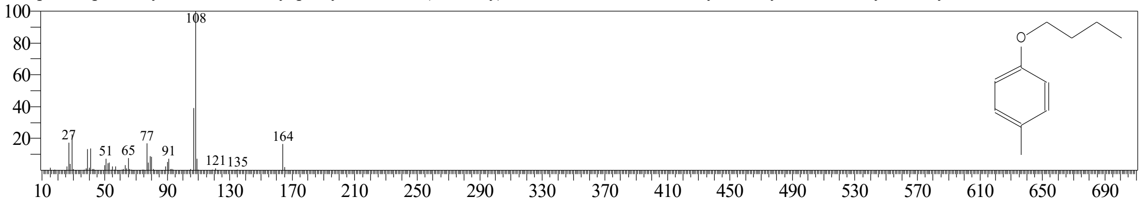

Hit#:5 Entry:61915 Library:NIST11.lib
SI:72 Formula:C12H15ClO2 CAS:0-00-0 MolWeight:226 RetIndex:1697
CompName:5-Chloropentanoic acid, 2-methylphenyl ester $$ 2-Methylphenyl 5-chloropentanoate # $$

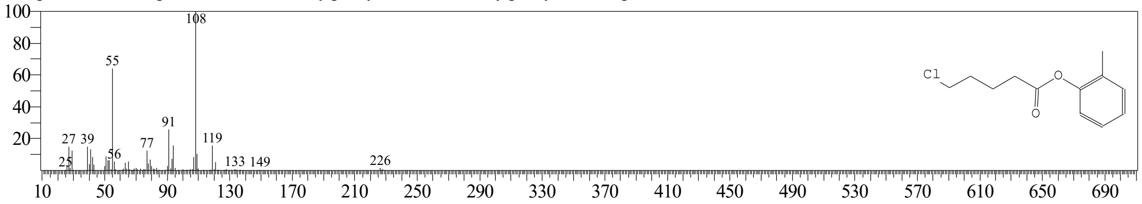

<< Target >>
Line#:162 R.Time:22.045(Scan#:3610) MassPeaks:351
RawMode:Averaged 22.040-22.050(3609-3611) BasePeak:108.05(41536)
BG Mode:Calc. from Peak Group 1 - Event 1 Scan

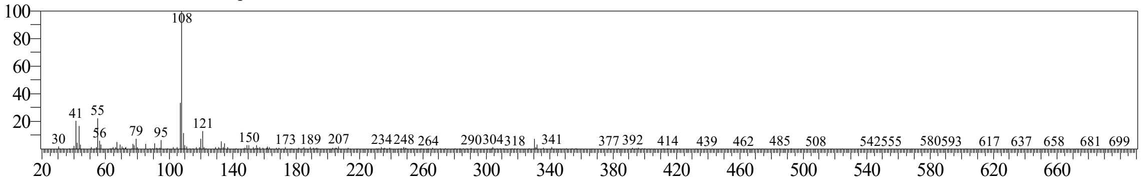

Hit#:1 Entry:101114 Library:NIST11.lib
SI:74 Formula:C16H22O4 CAS:0-00-0 MolWeight:278 RetIndex:2037
CompName:Oxalic acid, heptyl 2-methylphenyl ester

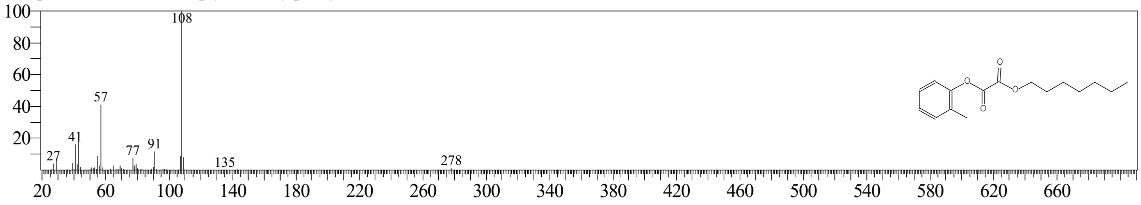

Hit#:2 Entry:90155 Library:NIST11.lib
SI:74 Formula:C15H20O4 CAS:0-00-0 MolWeight:264 RetIndex:1937
CompName:Oxalic acid, hexyl 2-methylphenyl ester

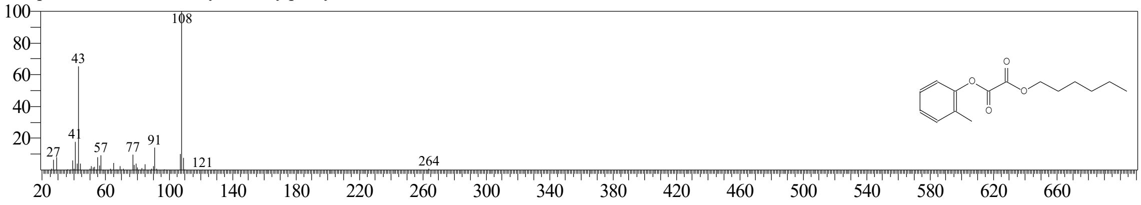

Hit#:3 Entry:154416 Library:NIST11.lib
SI:73 Formula:C21H32O4 CAS:0-00-0 MolWeight:348 RetIndex:2534
CompName:Oxalic acid, dodecyl 2-methylphenyl ester

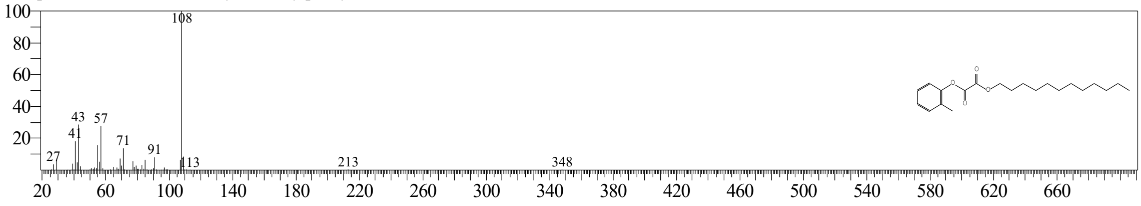

Hit#:4 Entry:144667 Library:NIST11.lib
SI:73 Formula:C20H30O4 CAS:0-00-0 MolWeight:334 RetIndex:2434
CompName:Oxalic acid, 2-methylphenyl undecyl ester

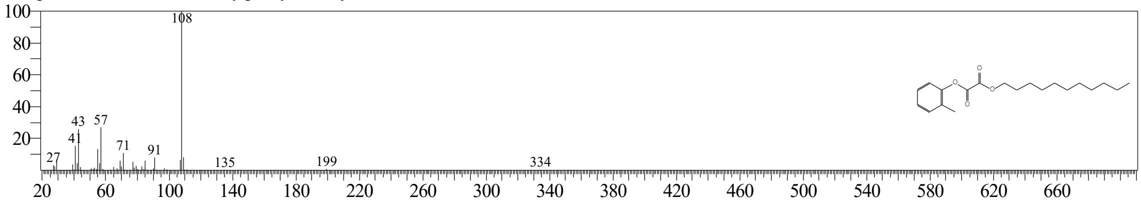

Hit#:5 Entry:30439 Library:NIST11.lib
SI:73 Formula:C12H18O CAS:85709-98-4 MolWeight:178 RetIndex:1296
CompName:Benzene, 1-(1,1-dimethylpropoxy)-4-methyl- $$ Toluene, 4-(1,1-dimethylpropoxy)- $$ 1-Methyl-4-(tert-pentyloxy)benzene # $$

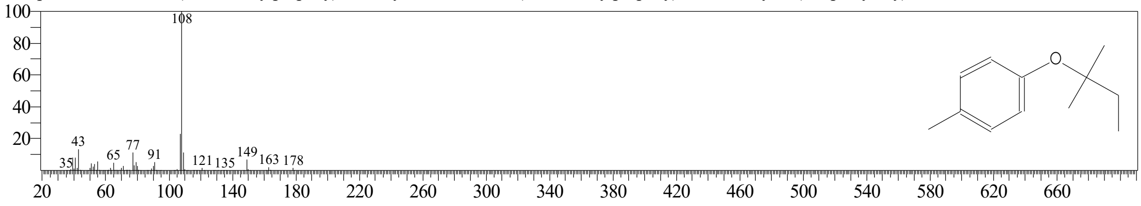

<< Target >>
Line#:163 R.Time:22.140(Scan#:3629) MassPeaks:350
RawMode:Averaged 22.135-22.145(3628-3630) BasePeak:43.10(4771)
BG Mode:Calc. from Peak Group 1 - Event 1 Scan

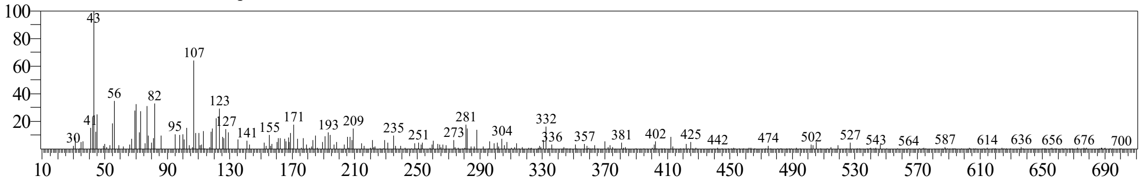

Hit#:1 Entry:17183 Library:NIST11.lib
SI:53 Formula:C8H14N2O CAS:0-00-0 MolWeight:154 RetIndex:1334
CompName:Butylamine, N-acetyl-1-cyano-2-methyl- $$ N-(1-Cyano-2-methylbutyl)acetamide # $$

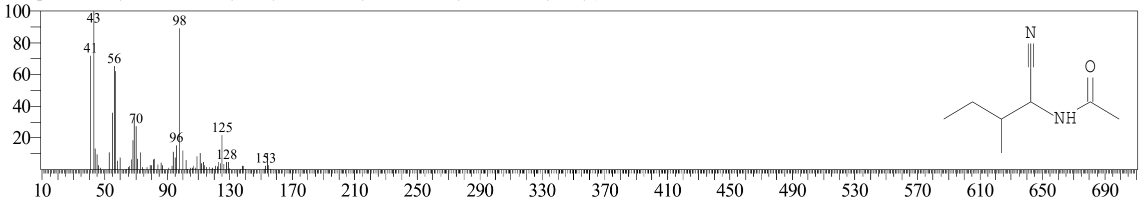

Hit#:2 Entry:79271 Library:NIST11.lib
SI:53 Formula:C12H14N2O4 CAS:0-00-0 MolWeight:250 RetIndex:2393
CompName:2-Acetylamino-4-(4-hydroxy-phenyl)-3-oxobutyramide $$ 2-(Acetylamino)-4-(4-hydroxyphenyl)-3-oxobutanamide # $$

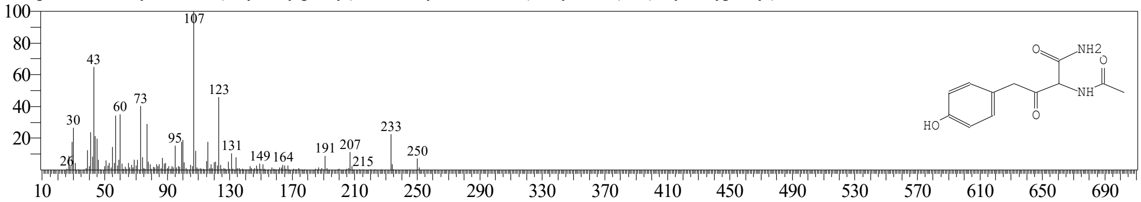

Hit#:3 Entry:187975 Library:NIST11.lib
SI:52 Formula:C25H20O6 CAS:55388-44-8 MolWeight:416 RetIndex:3886
CompName:2-Naphthalenecarboxylic acid, 4,4'-methylenebis[3-methoxy- $$ 2-Naphthoic acid, 3-methoxy-4-[(3-methoxy-2-naphthoic acid)methyl]- $$

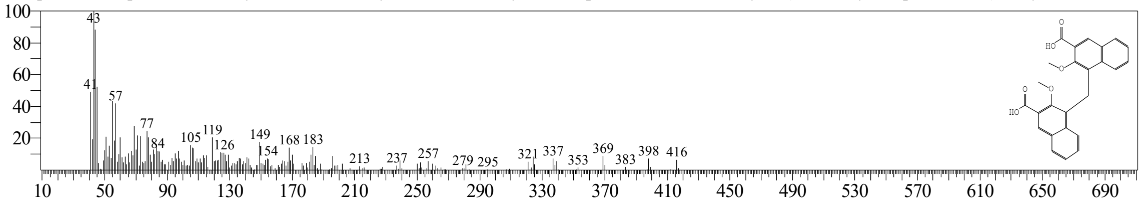

Hit#:4 Entry:9436 Library:NIST11.lib
SI:52 Formula:C6H13ClO CAS:33879-66-2 MolWeight:136 RetIndex:935
CompName:3-Pentanol, 1-chloro-3-methyl- $$ 1-Chloro-3-methyl-3-pentanol # $$

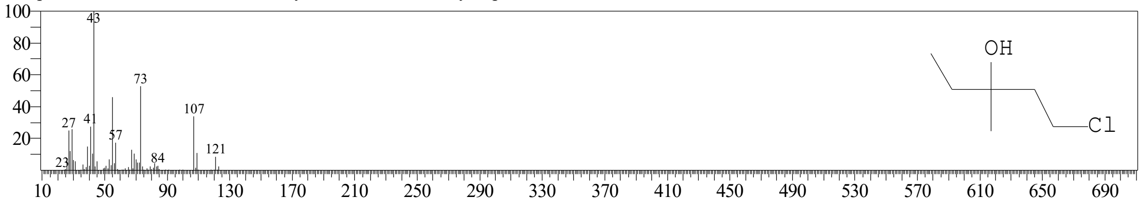

Hit#:5 Entry:25789 Library:NIST11.lib
SI:51 Formula:C10H18O2 CAS:3913-85-7 MolWeight:170 RetIndex:1380
CompName:2-Decenoic acid $$ (2E)-2-Decenoic acid # $$

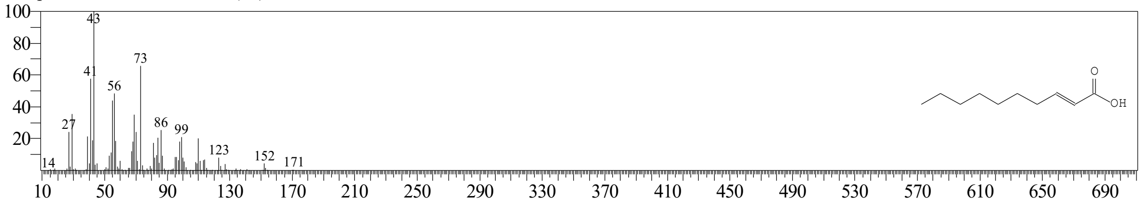

<< Target >>
Line#:164 R.Time:22.470(Scan#:3695) MassPeaks:361
RawMode:Averaged 22.465-22.475(3694-3696) BasePeak:43.05(17505)
BG Mode:Calc. from Peak Group 1 - Event 1 Scan

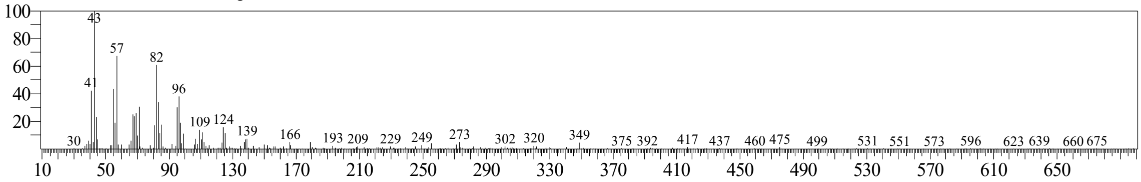

Hit#:1 Entry:93586 Library:NIST11.lib
SI:87 Formula:C18H36O CAS:638-66-4 MolWeight:268 RetIndex:1999
CompName:Octadecanal $$ Stearaldehyde $$ Octadecyl aldehyde $$ Stearyl aldehyde $$ n-Octadecanal $$

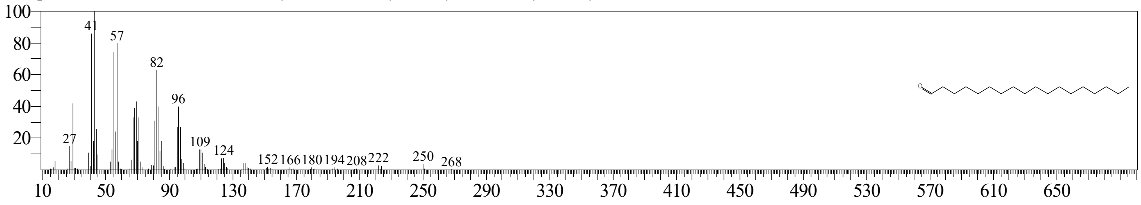

Hit#:2 Entry:42885 Library:NIST11.lib
SI:85 Formula:C13H26O CAS:10486-19-8 MolWeight:198 RetIndex:1502
CompName:Tridecanal $$ n-Tridecylaldehyde $$ Tridecanaldehyde $$ Tridecyl aldehyde $$ 1-Tridecanal $$ Tridecane aldehyde $$ n-Tridecanal $$

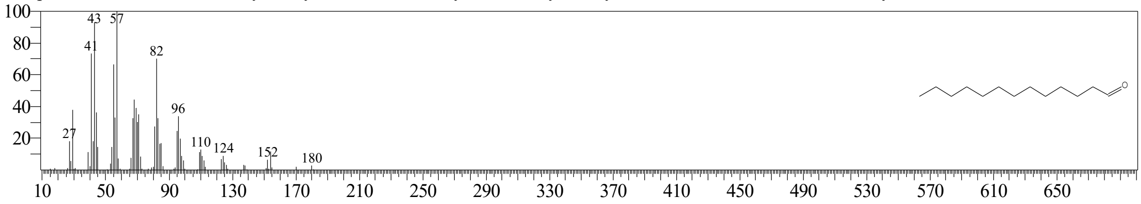

Hit#:3 Entry:82829 Library:NIST11.lib
SI:85 Formula:C17H34O CAS:0-00-0 MolWeight:254 RetIndex:1899
CompName:Heptadecanal

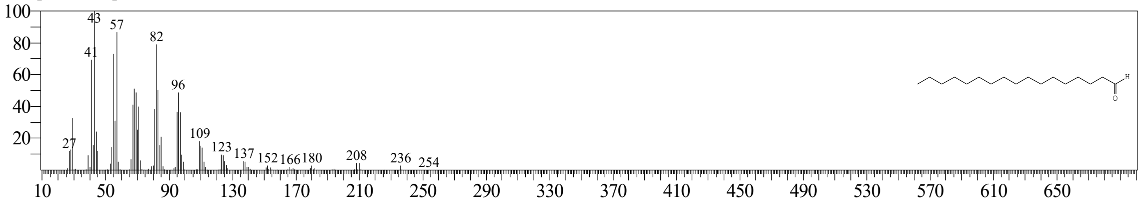

Hit#:4 Entry:140367 Library:NIST11.lib
SI:85 Formula:C21H44O2 CAS:5337-61-1 MolWeight:328 RetIndex:2530
CompName:2-Octadecyl-propane-1,3-diol $$ 2-Octadecyl-1,3-propanediol # $$

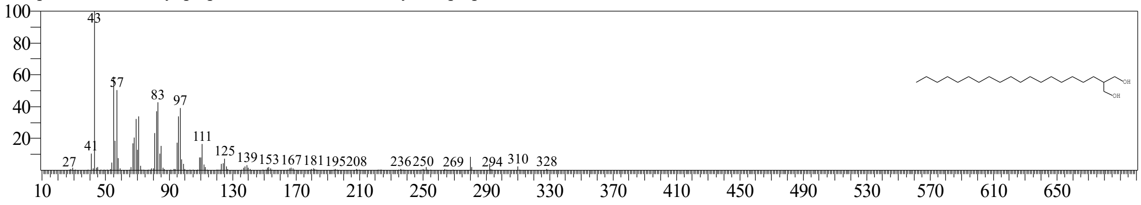

Hit#:5 Entry:107436 Library:NIST11.lib
SI:84 Formula:C16H30O4 CAS:56438-07-4 MolWeight:286 RetIndex:1882
CompName:1,1-Dodecanediol, diacetate $$ 1-(Acetyloxy)dodecyl acetate # $$

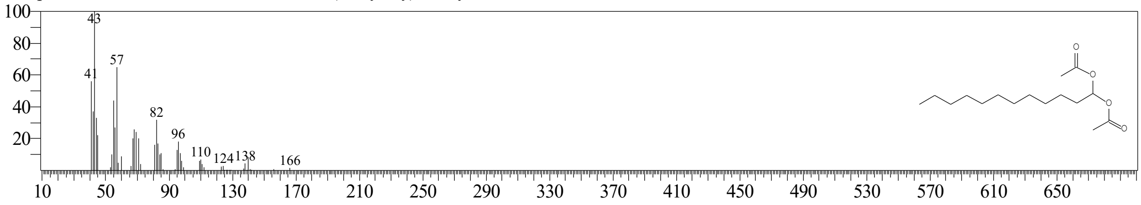

<< Target >>
Line#:165 R.Time:22.625(Scan#:3726) MassPeaks:374
RawMode:Averaged 22.620-22.630(3725-3727) BasePeak:69.10(2569)
BG Mode:Calc. from Peak Group 1 - Event 1 Scan

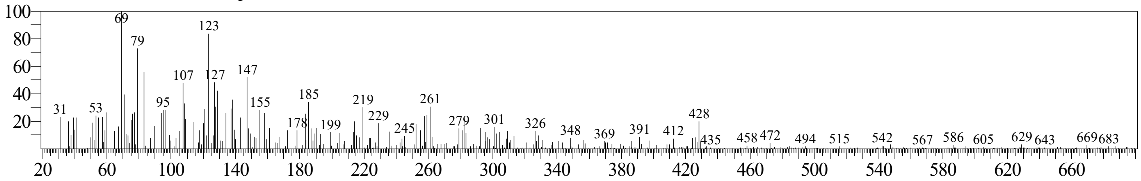

Hit#:1 Entry:34959 Library:NIST11.lib
SI:50 Formula:C10H18OS CAS:0-00-0 MolWeight:186 RetIndex:1458
CompName:Myrtanol, 2-mercapto- $$ (6,6-Dimethyl-2-sulfanylbicyclo[3.1.1]hept-2-yl)methanol # $$

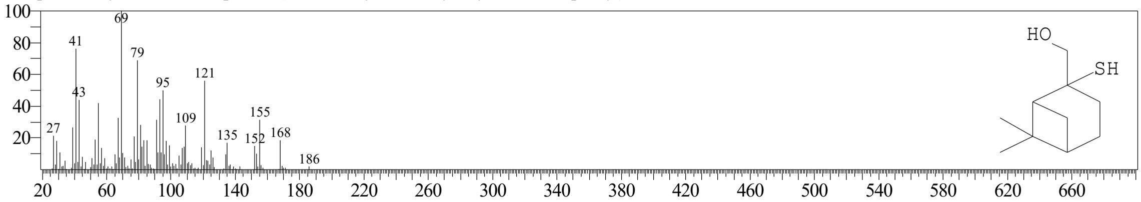

Hit#:2 Entry:189729 Library:NIST11.lib
SI:49 Formula:C26H46O4 CAS:0-00-0 MolWeight:422 RetIndex:2782
CompName:Adipic acid, di(.beta.-citronellyl) ester

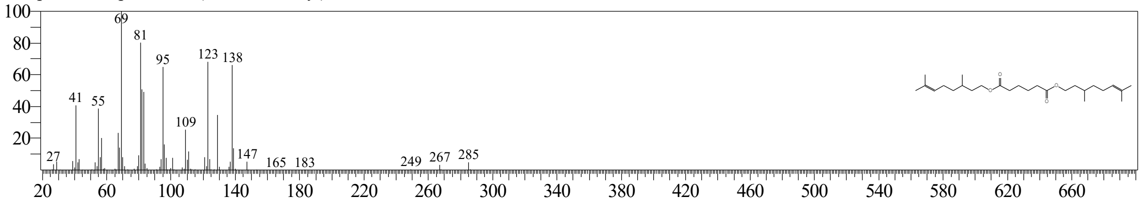

Hit#:3 Entry:32020 Library:NIST11.lib
SI:49 Formula:C9H15N3O CAS:7499-13-0 MolWeight:181 RetIndex:1791
CompName:Ketone, 1-cyclohexen-1-yl methyl, semicarbazone $$ 1-Acetylcyclohexene semicarbazone $$ (1E)-1-(1-Cyclohexen-1-yl)ethanone semicarbazone #

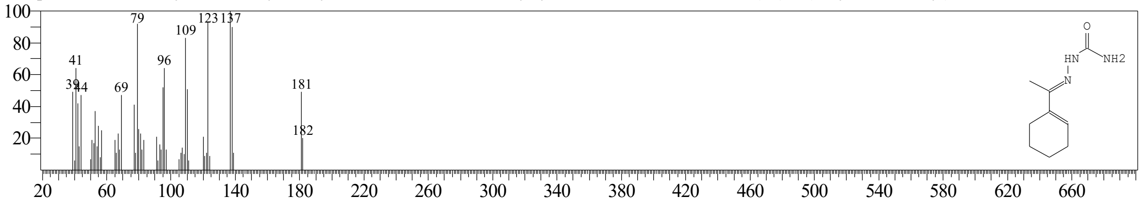

Hit#:4 Entry:79781 Library:NIST11.lib
SI:49 Formula:C16H26O2 CAS:81752-89-8 MolWeight:250 RetIndex:1704
CompName:Methyl (1.beta.,4a.alpha.,8a.beta.)-decahydro-5,5,8a-trimethyl-2-methylenenaphthalene-1-carboxylate $$ Methyl 5,5,8a-trimethyl-2-methylenedecal

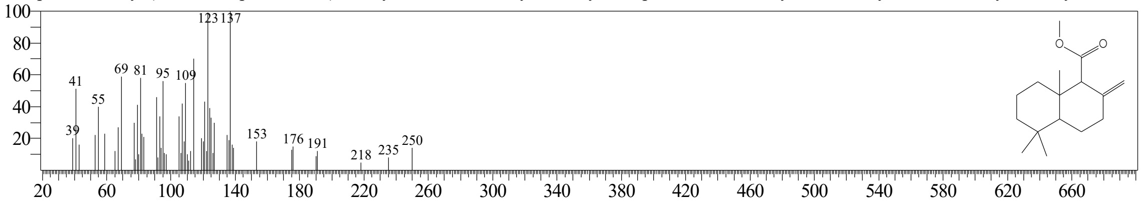

Hit#:5 Entry:16366 Library:NIST11.lib
SI:49 Formula:C10H16O CAS:6931-54-0 MolWeight:152 RetIndex:1020
CompName:Spiro[bicyclo[3.1.1]heptane-2,2'-oxirane], 6,6-dimethyl- $$ .beta.-Pinene oxide $$ Pinane, 2,10-epoxy- $$ .beta.-Pinene epoxide $$ 2,10-Epoxypina

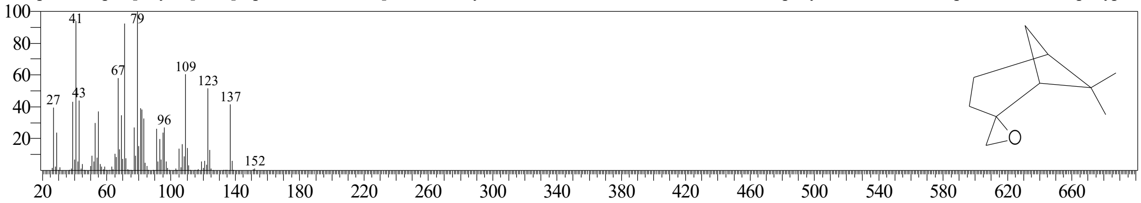

<< Target >>
Line#:166 R.Time:22.705(Scan#:3742) MassPeaks:375
RawMode:Averaged 22.700-22.710(3741-3743) BasePeak:41.10(4204)
BG Mode:Calc. from Peak Group 1 - Event 1 Scan

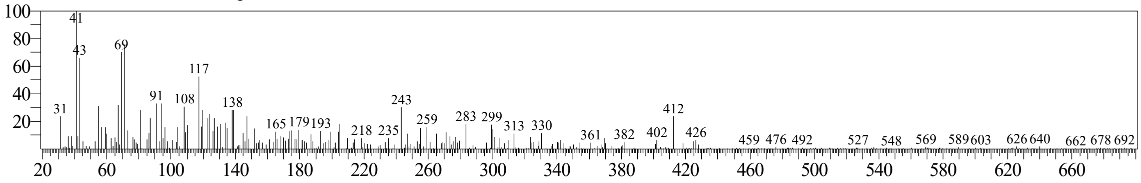

Hit#:1 Entry:182642 Library:NIST11.lib
SI:54 Formula:C27H44O2 CAS:56362-84-6 MolWeight:400 RetIndex:2801
CompName:Cholesta-5,24-diene-3,20-diol, (3.beta.,20R)- $$ Cholesta-5,24-diene-3,20-diol # $$

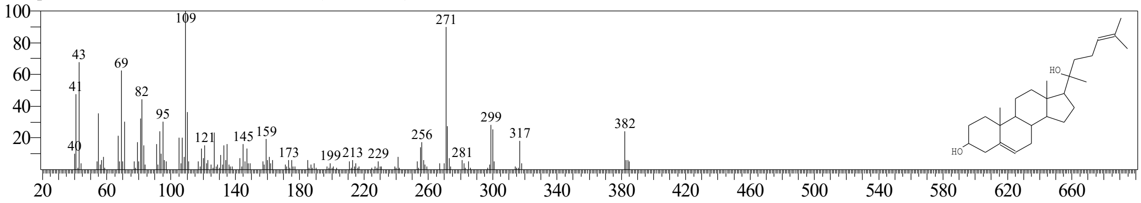

Hit#:2 Entry:186819 Library:NIST11.lib
SI:52 Formula:C29H48O CAS:2604-90-2 MolWeight:412 RetIndex:2714
CompName:Cholest-7-en-3-one, 4,4-dimethyl-, (5.alpha.)- $$ 5.alpha.-Cholest-7-en-3-one, 4,4-dimethyl- $$ 4,4-Dimethylcholest-7-en-3-one # $$

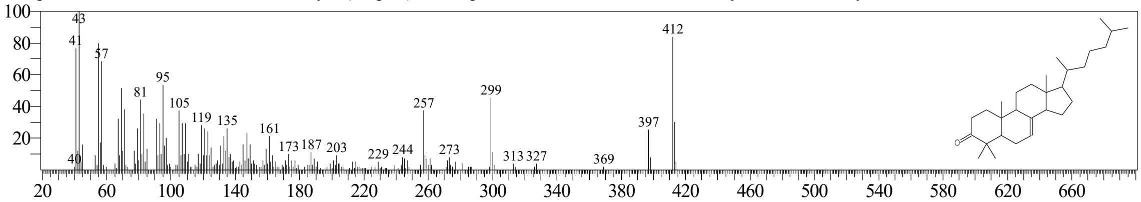

Hit#:3 Entry:62268 Library:NIST11.lib
SI:52 Formula:C14H26O2 CAS:141-16-2 MolWeight:226 RetIndex:1501
CompName:Citronellyl butyrate $$ Butanoic acid, 3,7-dimethyl-6-octenyl ester $$ Butyric acid, 3,7-dimethyl-6-octenyl ester $$ Citronellyl n-butyrate $$ Natura

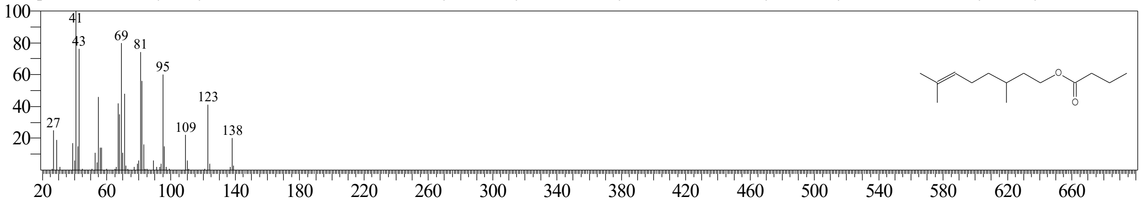

Hit#:4 Entry:18663 Library:NIST11.lib
SI:52 Formula:C10H20O CAS:2216-52-6 MolWeight:156 RetIndex:1164
CompName:Cyclohexanol, 5-methyl-2-(1-methylethyl)-, [1S-(1.alpha.,2.alpha.,5.beta.)]- $$ (1S, 2S, 5R)-(+)-Neomenthol $$ 2-Isopropyl-5-methylcyclohexanol

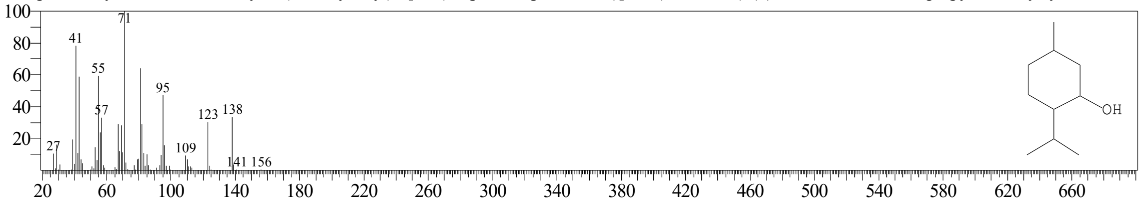

Hit#:5 Entry:205488 Library:NIST11.lib
SI:51 Formula:C30H52O6 CAS:56053-00-0 MolWeight:508 RetIndex:3493
CompName:Ergostane-3,5,6,12,25-pentol, 25-acetate, (3.beta.,5.alpha.,6.beta.,12.beta.)- $$ 3,5,6,12-Tetrahydroxyergostan-25-yl acetate # $$

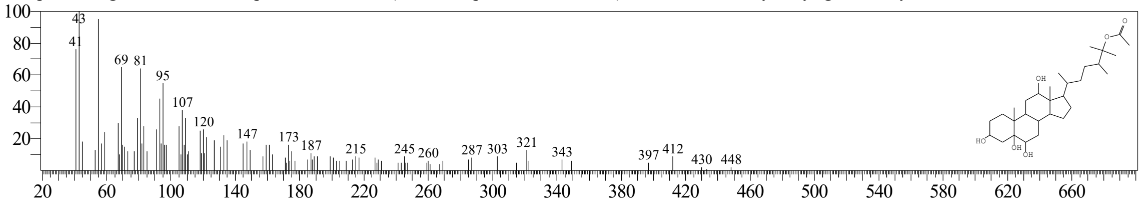

<< Target >>
Line#:167 R.Time:22.750(Scan#:3751) MassPeaks:376
RawMode:Averaged 22.745-22.755(3750-3752) BasePeak:69.10(4553)
BG Mode:Calc. from Peak Group 1 - Event 1 Scan

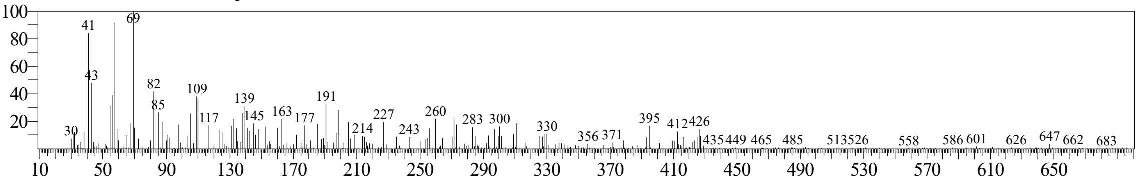

Hit#:1 Entry:82836 Library:NIST11.lib
SI:52 Formula:C17H34O CAS:0-00-0 MolWeight:254 RetIndex:1699
CompName:2-Methyl-Z-7,8-epoxyhexadecane $$ 2-(5-Methylhexyl)-3-octyloxirane # $$

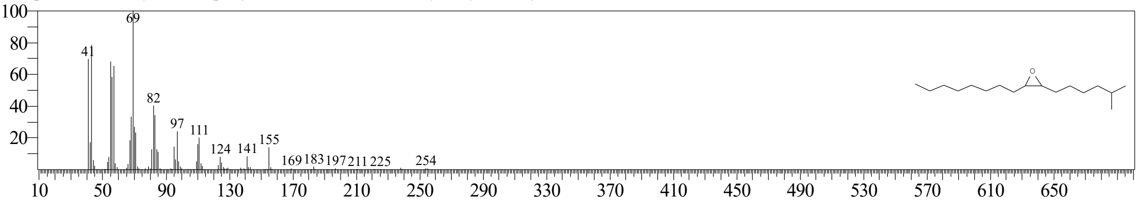

Hit#:2 Entry:44138 Library:NIST11.lib
SI:52 Formula:C13H28O CAS:0-00-0 MolWeight:200 RetIndex:1492
CompName:4-Methyl-dodecan-1-ol $$ 4-Methyl-1-dodecanol # $$

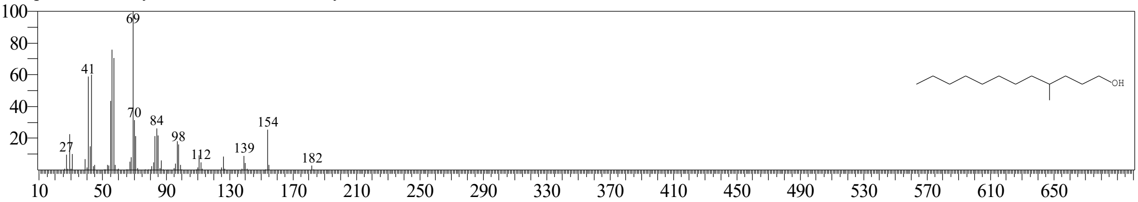

Hit#:3 Entry:18661 Library:NIST11.lib
SI:52 Formula:C10H20O CAS:0-00-0 MolWeight:156 RetIndex:1097
CompName:2,3,6-Trimethylhept-6-en-1-ol $$ 2,3,6-Trimethyl-6-hepten-1-ol # $$

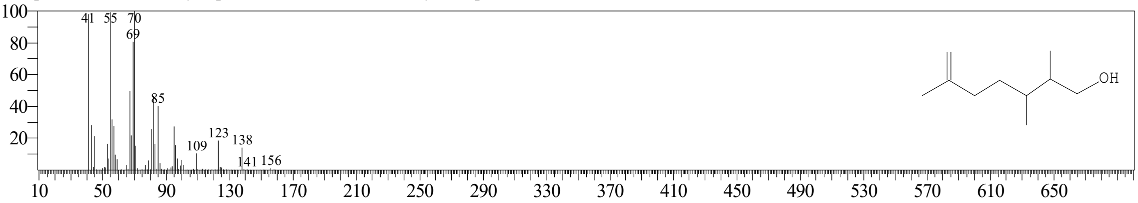

Hit#:4 Entry:104503 Library:NIST11.lib
SI:51 Formula:C19H38O CAS:29804-22-6 MolWeight:282 RetIndex:1898
CompName:Disparlure $$ Oxirane, 2-decyl-3-(5-methylhexyl)-, cis- $$ (.+/-cis-7,8-Epoxy-2-methyloctadecane $$ 2-Decyl-3-(5-methylhexyl)oxirane, (Z)- $$ cis

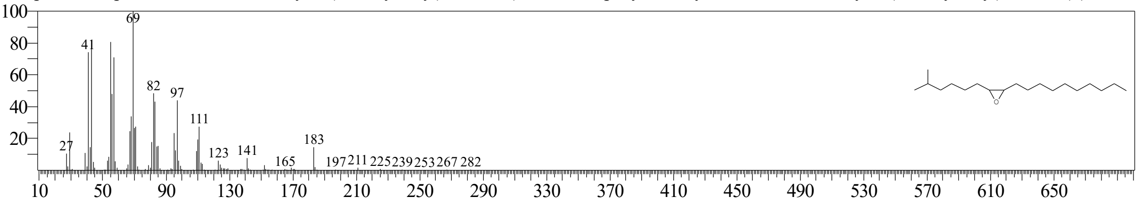

Hit#:5 Entry:50999 Library:NIST11.lib
SI:51 Formula:C14H26O CAS:141-13-9 MolWeight:210 RetIndex:1458
CompName:9-Undecenal, 2,6,10-trimethyl- $$ Adoxal $$ 2,6,10-Trimethyl-9-undecen-1-al $$ 2,6,10-Trimethyl-9-undecenal # $$

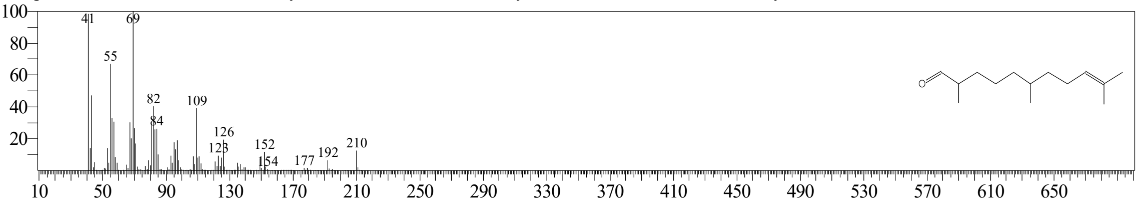

<< Target >>
Line#:168 R.Time:22.870(Scan#:3775) MassPeaks:354
RawMode:Averaged 22.865-22.875(3774-3776) BasePeak:55.10(4307)
BG Mode:Calc. from Peak Group 1 - Event 1 Scan

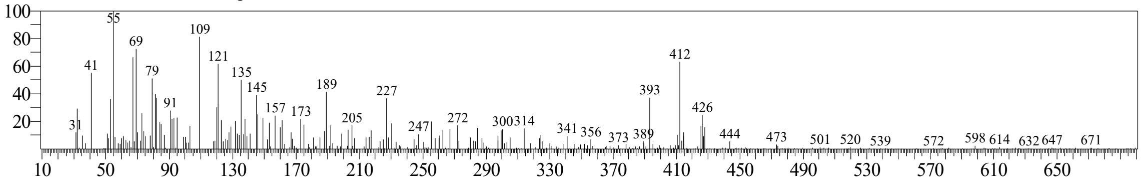

Hit#:1 Entry:196536 Library:NIST11.lib
SI:55 Formula:C28H48O4 CAS:56052-97-2 MolWeight:448 RetIndex:3179
CompName:Ergost-25-ene-3,5,6,12-tetrol, (3.beta.,5.alpha.,6.beta.,12.beta.)- $$ Ergost-25(27)-ene-3,5,6,12-tetrol # $$

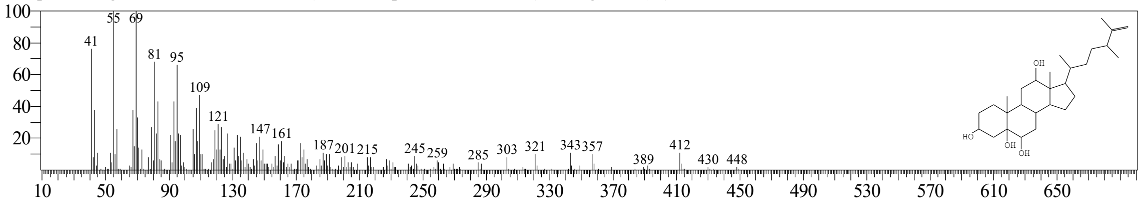

Hit#:2 Entry:183306 Library:NIST11.lib
SI:55 Formula:C25H38O4 CAS:0-00-0 MolWeight:402 RetIndex:3028
CompName:6-(1,5-Dimethyl-hex-4-enyl)-1,6-dihydroxy-1,8a-dimethyl-3-oxo-1,2,3,3a,5a,6,7,8,8a,9,10,10a-dodecahydrodicyclopenta[a,e]cyclooctene $$ 6-(1,5-

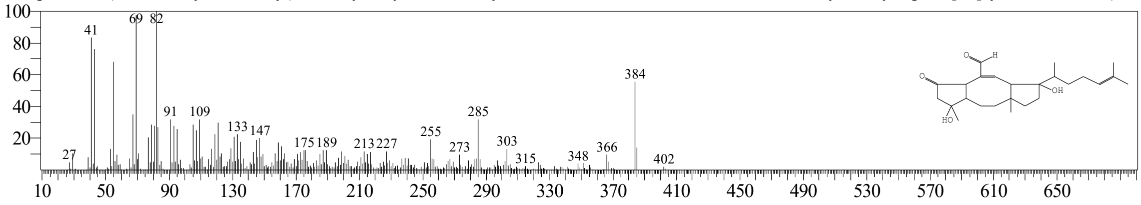

Hit#:3 Entry:191106 Library:NIST11.lib
SI:55 Formula:C30H50O CAS:469-38-5 MolWeight:426 RetIndex:2816
CompName:9,19-Cyclolanost-24-en-3-ol, (3.beta.)- $$ 9,19-Cyclo-9.beta.-lanost-24-en-3.beta.-ol $$ Cycloartenol $$ Handianol $$ 1-(1,5-Dimethyl-4-hexenyl)-

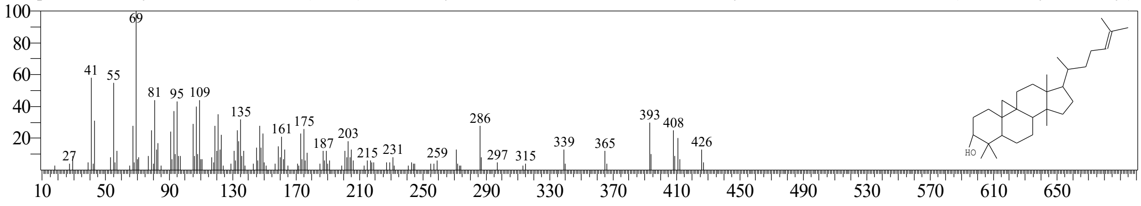

Hit#:4 Entry:115215 Library:NIST11.lib
SI:55 Formula:C17H28O4 CAS:0-00-0 MolWeight:296 RetIndex:1934
CompName:2-(2,7-Dimethylocta-2,7-dienyl)malonic acid, diethyl ester $$ Diethyl 2-[(2E)-2,7-dimethyl-2,7-octadienyl]malonate # $$

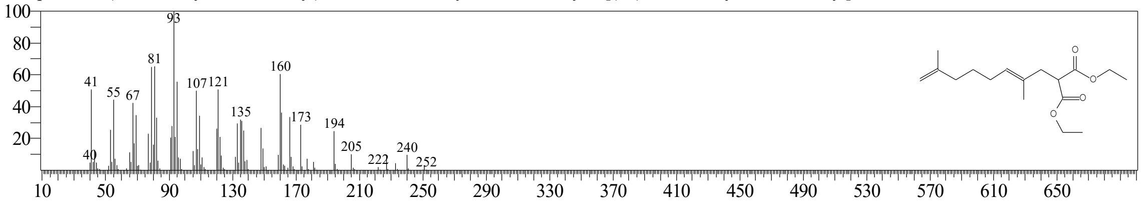

Hit#:5 Entry:168170 Library:NIST11.lib
SI:54 Formula:C26H42O CAS:35882-88-3 MolWeight:370 RetIndex:2546
CompName:26,27-Dinorergosta-5,23-dien-3-ol, (3.beta.)-

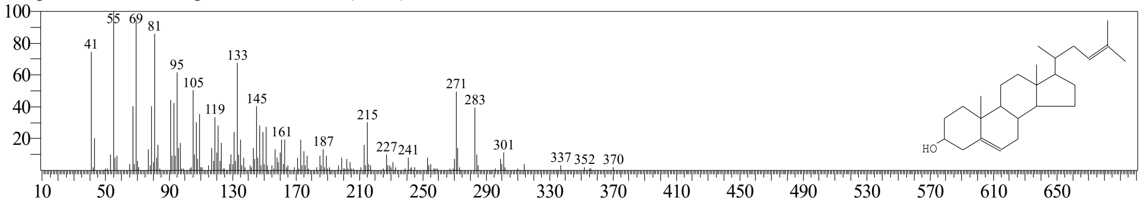

<< Target >>
Line#:169 R.Time:22.970(Scan#:3795) MassPeaks:344
RawMode:Averaged 22.965-22.975(3794-3796) BasePeak:69.10(3518)
BG Mode:Calc. from Peak Group 1 - Event 1 Scan

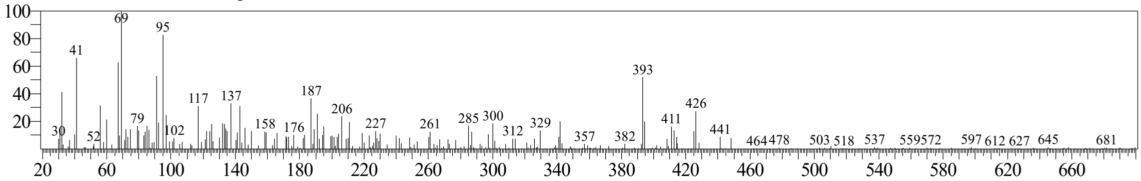

Hit#:1 Entry:57720 Library:NIST11.lib
SI:42 Formula:C15H24O CAS:0-00-0 MolWeight:220 RetIndex:1743
CompName:Cyclopentanone, 3-[3,5-decadienyl]-, (E,E)- $$ 3-[(3E,5E)-3,5-Decadienyl]cyclopentanone # $$

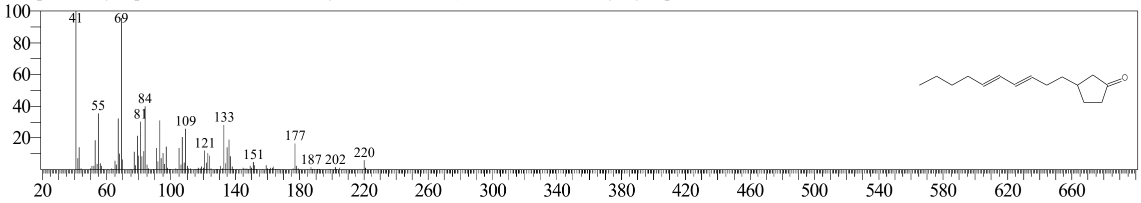

Hit#:2 Entry:24748 Library:NIST11.lib
SI:41 Formula:C11H20O CAS:78996-11-9 MolWeight:168 RetIndex:1266
CompName:(2,2,6-Trimethyl-bicyclo[4.1.0]hept-1-yl)-methanol

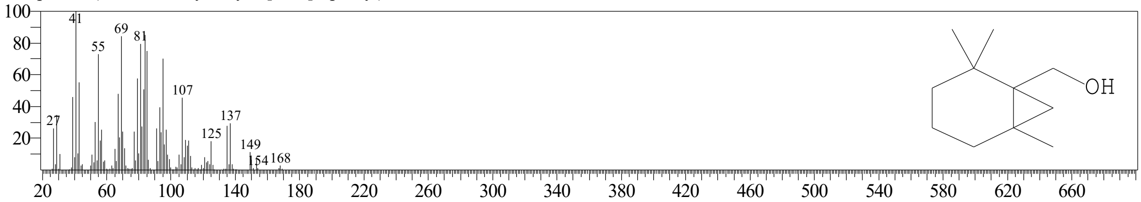

Hit#:3 Entry:47981 Library:NIST11.lib
SI:41 Formula:C14H22O CAS:0-00-0 MolWeight:206 RetIndex:1431
CompName:1-Oxaspiro[2.5]octane, 5,5-dimethyl-4-(3-methyl-1,3-butadienyl)- $$ 5,5-Dimethyl-4-[(1E)-3-methyl-1,3-butadienyl]-1-oxaspiro[2.5]octane # $$

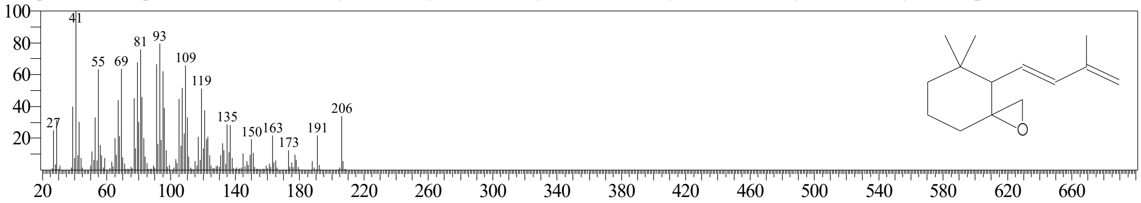

Hit#:4 Entry:96990 Library:NIST11.lib
SI:41 Formula:C20H32 CAS:70901-63-2 MolWeight:272 RetIndex:1922
CompName:(E,E)-7,11,15-Trimethyl-3-methylene-hexadeca-1,6,10,14-tetraene $$ (6E,10E)-7,11,15-Trimethyl-3-methylene-1,6,10,14-hexadecatetraene # $$

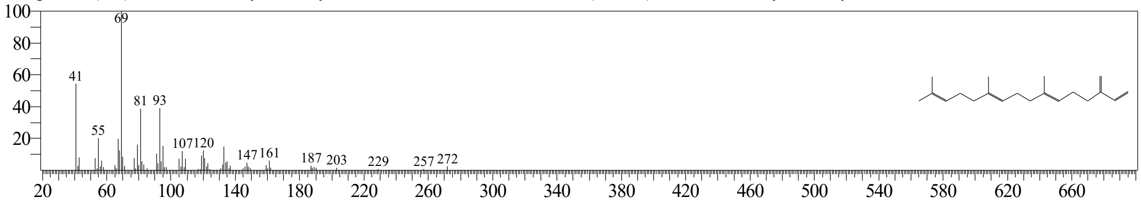

Hit#:5 Entry:96902 Library:NIST11.lib
SI:41 Formula:C18H24O2 CAS:0-00-0 MolWeight:272 RetIndex:2025
CompName:Neryl phenylacetate $$ Phenyl (4E)-5,9-dimethyl-4,8-decadienoate # $$

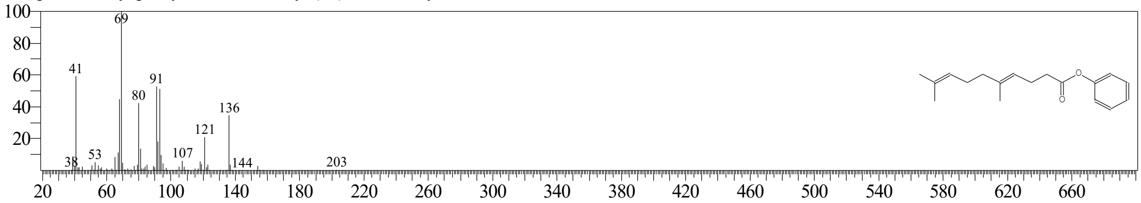

Qualitative Analysis Report

<< Target >>
Line#:170 R.Time:23.005(Scan#:3802) MassPeaks:381
RawMode:Averaged 23.000-23.010(3801-3803) BasePeak:98.10(3542)
BG Mode:Calc. from Peak Group 1 - Event 1 Scan

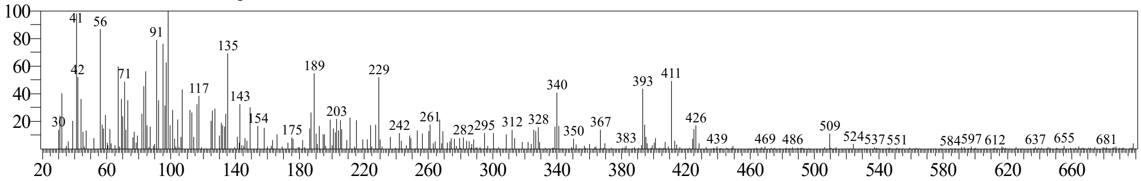

Hit#:1 Entry:92775 Library:NIST11.lib
SI:52 Formula:C10H16N6O3 CAS:329712-67-6 MolWeight:268 RetIndex:2465
CompName:Acetic acid, 2-(1-piperidyl)-, [4-aminofurazanyl-3-(amino)methylidenamino] ester $$ 4-Amino-N'-[(1-piperidinylacetyl)oxy]-1,2,5-oxadiazole-3-car

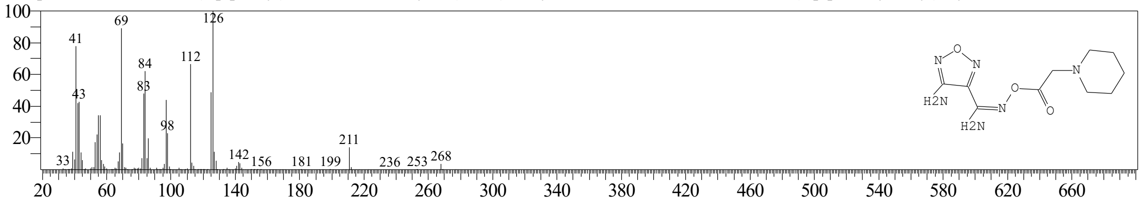

Hit#:2 Entry:132041 Library:NIST11.lib
SI:50 Formula:C12H21Cl3O3 CAS:0-00-0 MolWeight:318 RetIndex:1845
CompName:Carbonic acid, nonyl 2,2,2-trichloroethyl ester

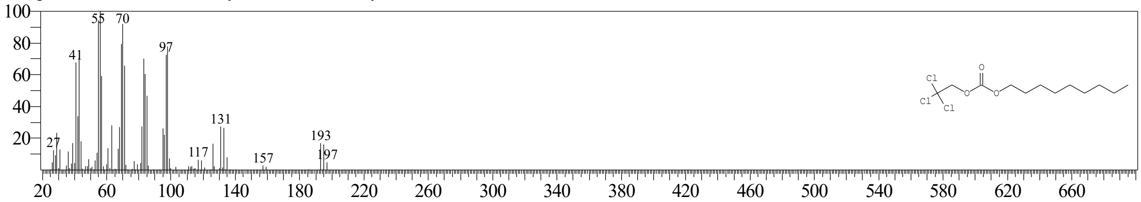

Hit#:3 Entry:27992 Library:NIST11.lib
SI:50 Formula:C9H18O3 CAS:78092-09-8 MolWeight:174 RetIndex:1530
CompName:1,2-Cyclopentanedimethanol, 3-hydroxy-4,4-dimethyl- $$ 4,5-Bis(hydroxymethyl)-2,2-dimethylcyclopentanol # $$

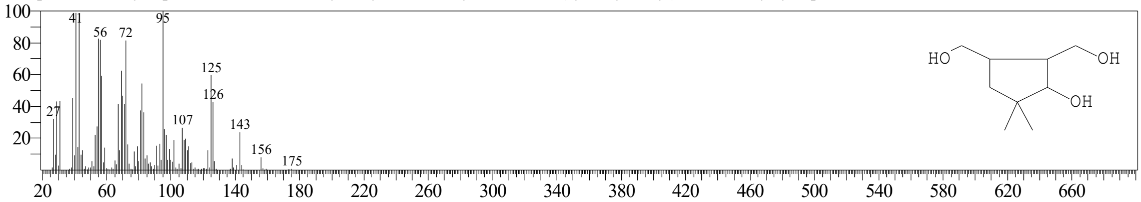

Hit#:4 Entry:161532 Library:NIST11.lib
SI:50 Formula:C15H27Cl3O3 CAS:0-00-0 MolWeight:360 RetIndex:2143
CompName:Carbonic acid, dodecyl 2,2,2-trichloroethyl ester

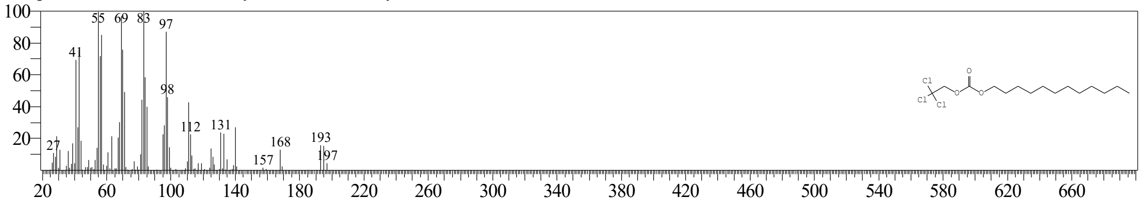

Hit#:5 Entry:169899 Library:NIST11.lib
SI:50 Formula:C16H29Cl3O3 CAS:0-00-0 MolWeight:374 RetIndex:2242
CompName:Carbonic acid, tridecyl 2,2,2-trichloroethyl ester

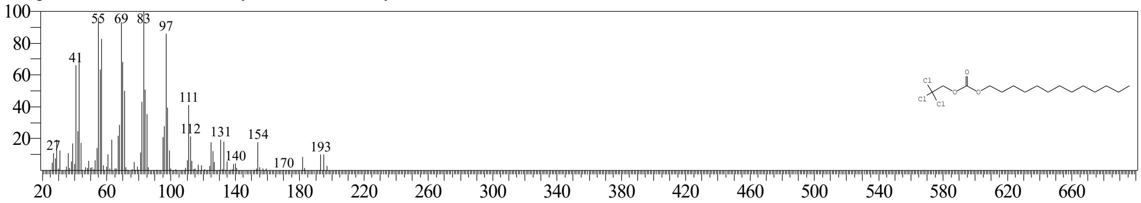

<< Target >>
Line#:171 R.Time:23.275(Scan#:3856) MassPeaks:360
RawMode:Averaged 23.270-23.280(3855-3857) BasePeak:71.10(2246)
BG Mode:Calc. from Peak Group 1 - Event 1 Scan

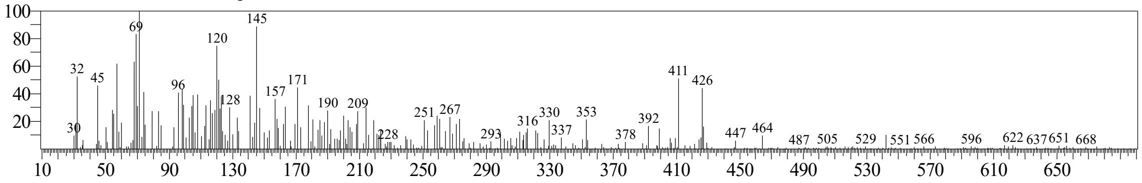

Hit#:1 Entry:135670 Library:NIST11.lib
SI:46 Formula:C20H28F2O CAS:90660-21-2 MolWeight:322 RetIndex:2120
CompName:4,4-Difluororetinol (all-trans) $$ 4,4-Difluororetinol # $$

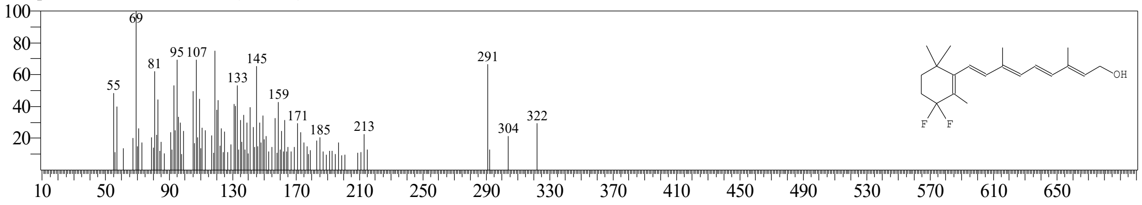

Hit#:2 Entry:191101 Library:NIST11.lib
SI:43 Formula:C30H50O CAS:469-39-6 MolWeight:426 RetIndex:2760
CompName:9,19-Cycloergost-24(28)-en-3-ol, 4,14-dimethyl-, (3.beta.,4.alpha.,5.alpha.)- $$ 9,19-Cyclo-5.alpha.,9.beta.-ergost-24(28)-en-3.beta.-ol, 4.alpha.,14

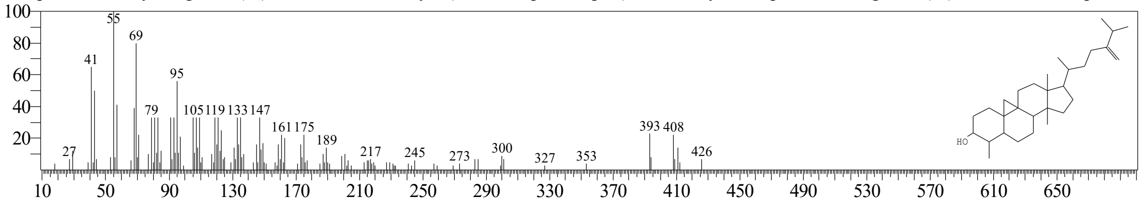

Hit#:3 Entry:210358 Library:NIST11.lib
SI:42 Formula:C33H46ClNO7 CAS:107024-98-6 MolWeight:603 RetIndex:4391
CompName:Milbemycin b, 13-chloro-5-demethoxy-28-deoxy-6,28-epoxy-5-(hydroxyimino)-25-(1-methylethyl)-, (6R,13R,25R)-

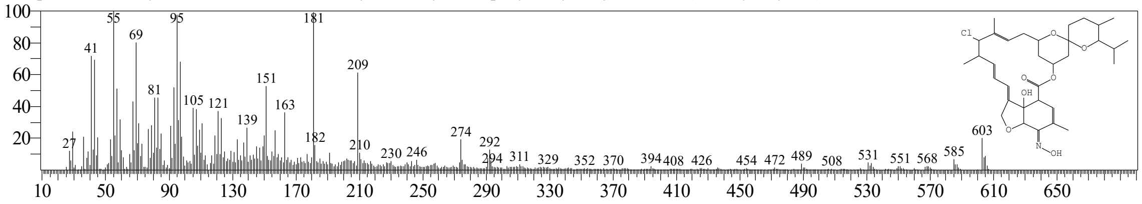

Hit#:4 Entry:50869 Library:NIST11.lib
SI:40 Formula:C13H22O2 CAS:0-00-0 MolWeight:210 RetIndex:1440
CompName:1-Ethynyl-3,trans(1,1-dimethylethyl)-4,cis-methoxycyclohexan-1-ol $$ 3-tert-Butyl-1-ethynyl-4-methoxycyclohexanol # $$

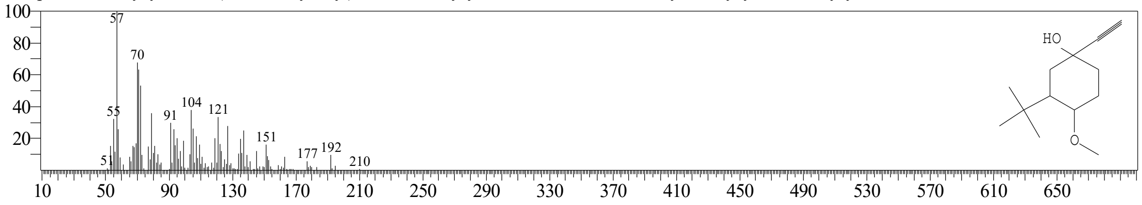

Hit#:5 Entry:191102 Library:NIST11.lib
SI:40 Formula:C30H50O CAS:132072-04-9 MolWeight:426 RetIndex:2800
CompName:24-Methyl-5,28-stigmastadien-3.beta.-ol $$ 24-Methylstigmasta-5,28-dien-3-ol # $$

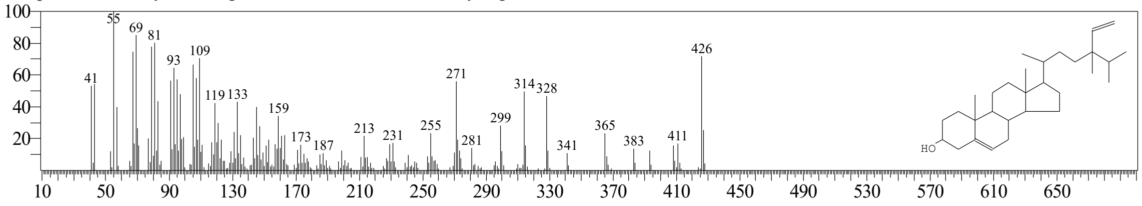

<< Target >>
Line#:172 R.Time:23.335(Scan#:3868) MassPeaks:352
RawMode:Averaged 23.330-23.340(3867-3869) BasePeak:69.10(3767)
BG Mode:Calc. from Peak Group 1 - Event 1 Scan

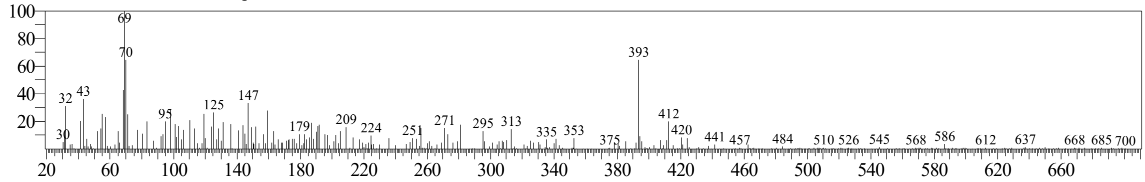

Hit#:1 Entry:190271 Library:NIST11.lib
SI:50 Formula:C26H48O4 CAS:0-00-0 MolWeight:424 RetIndex:2930
CompName:Succinic acid, heptadecyl pent-4-enyl ester

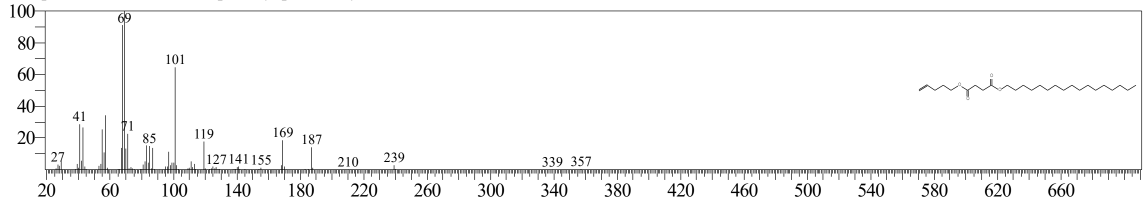

Hit#:2 Entry:199906 Library:NIST11.lib
SI:50 Formula:C29H54O4 CAS:0-00-0 MolWeight:466 RetIndex:3206
CompName:Succinic acid, eicosyl 3-methylbut-3-enyl ester

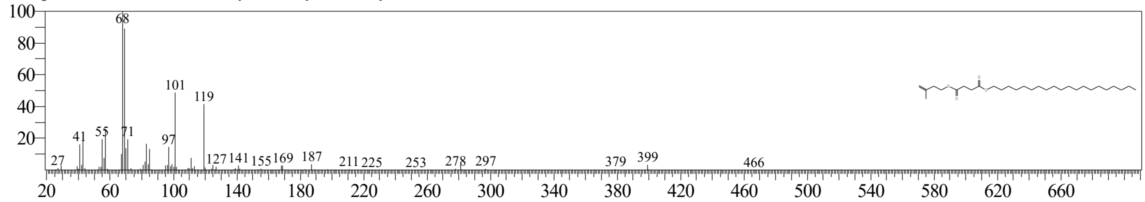

Hit#:3 Entry:166580 Library:NIST11.lib
SI:50 Formula:C22H40O4 CAS:0-00-0 MolWeight:368 RetIndex:2533
CompName:Succinic acid, pent-4-enyl tridecyl ester

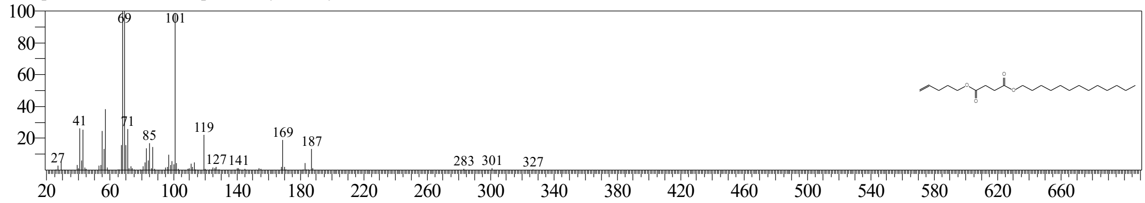

Hit#:4 Entry:180644 Library:NIST11.lib
SI:50 Formula:C24H44O4 CAS:0-00-0 MolWeight:396 RetIndex:2732
CompName:Succinic acid, pentadecyl pent-4-enyl ester

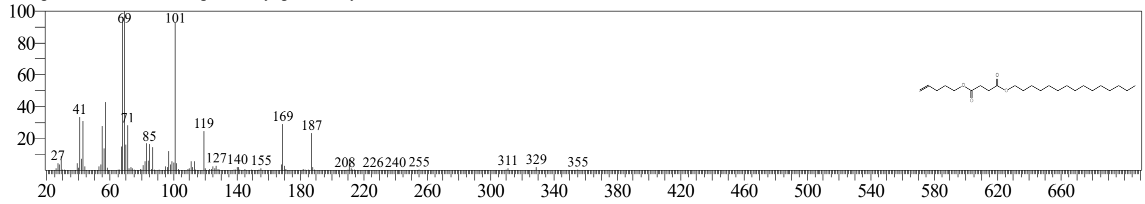

Hit#:5 Entry:194068 Library:NIST11.lib
SI:50 Formula:C27H50O4 CAS:0-00-0 MolWeight:438 RetIndex:3030
CompName:Succinic acid, octadecyl pent-4-enyl ester

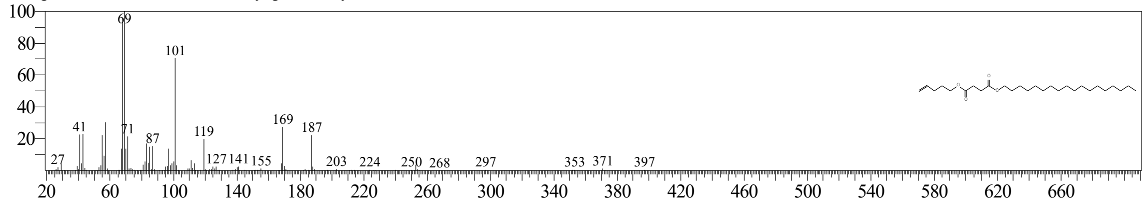

Qualitative Analysis Report

<< Target >>
Line#:173 R.Time:23.925(Scan#:3986) MassPeaks:475
RawMode:Averaged 23.920-23.930(3985-3987) BasePeak:43.10(200822)
BG Mode:Calc. from Peak Group 1 - Event 1 Scan

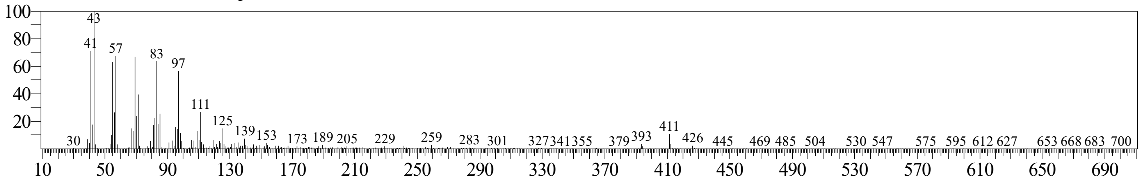

Hit#:1 Entry:164490 Library:NIST11.lib
SI:90 Formula:C26H52 CAS:18835-33-1 MolWeight:364 RetIndex:2596
CompName:1-Hexacosene

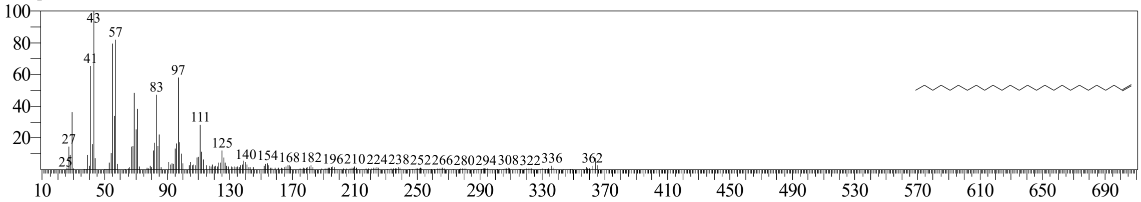

Hit#:2 Entry:177914 Library:NIST11.lib
SI:89 Formula:C20H39BrO2 CAS:18992-03-5 MolWeight:390 RetIndex:2473
CompName:Bromoacetic acid, octadecyl ester $$ Octadecyl bromoacetate # $$

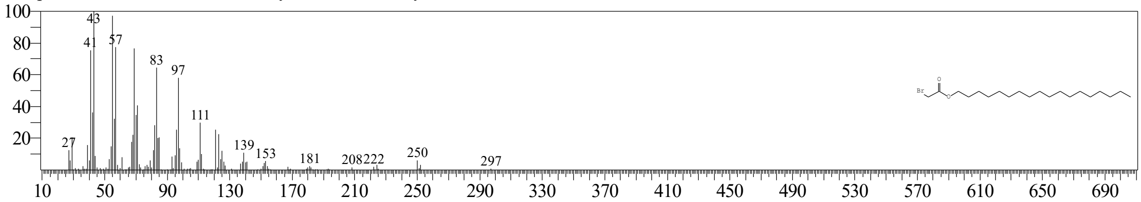

Hit#:3 Entry:197101 Library:NIST11.lib
SI:89 Formula:C21H35F7O2 CAS:959085-66-6 MolWeight:452 RetIndex:1833
CompName:Heptadecyl heptafluorobutyrate $$ Heptadecyl 2,2,3,3,4,4,4-heptafluorobutanoate $$ 1-Heptadecanol, heptafluorobutyrate $$ Heptadecyl perfluorob

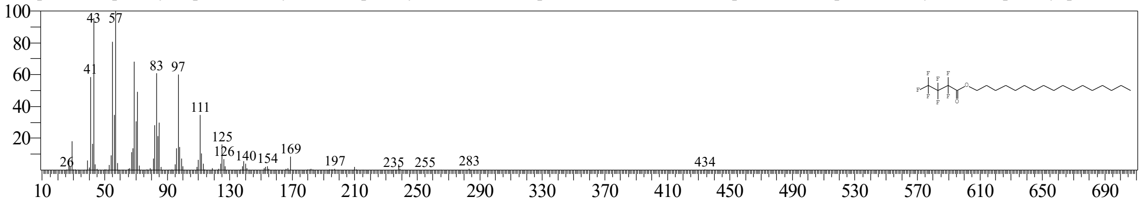

Hit#:4 Entry:146250 Library:NIST11.lib
SI:89 Formula:C24H48 CAS:297-03-0 MolWeight:336 RetIndex:2878
CompName:Cyclotetracosane

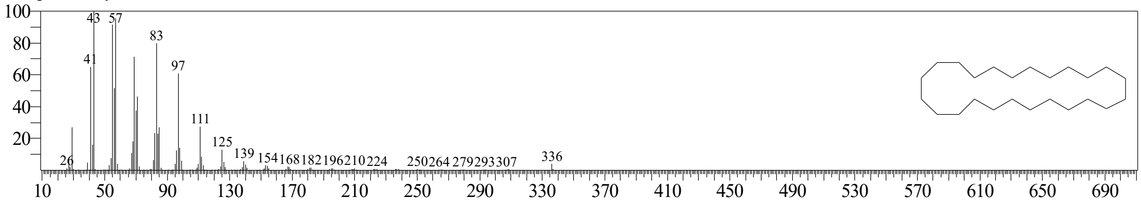

Hit#:5 Entry:114020 Library:NIST11.lib
SI:89 Formula:C21H42 CAS:95008-11-0 MolWeight:294 RetIndex:2117
CompName:10-Heneicosene (c,t) $$ (10E)-10-Henicosene # $$

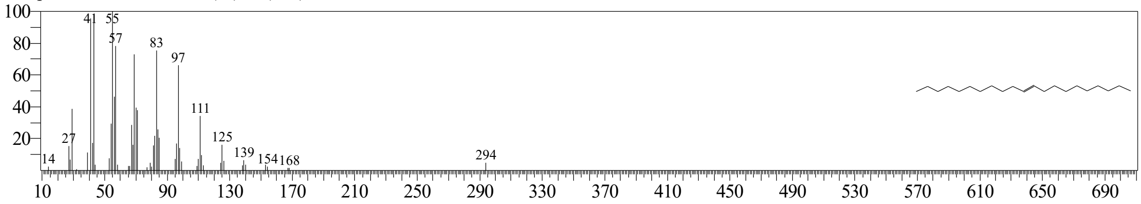

<< Target >>
Line#:174 R.Time:24.355(Scan#:4072) MassPeaks:444
RawMode:Averaged 24.350-24.360(4071-4073) BasePeak:69.10(2383727)
BG Mode:Calc. from Peak Group 1 - Event 1 Scan

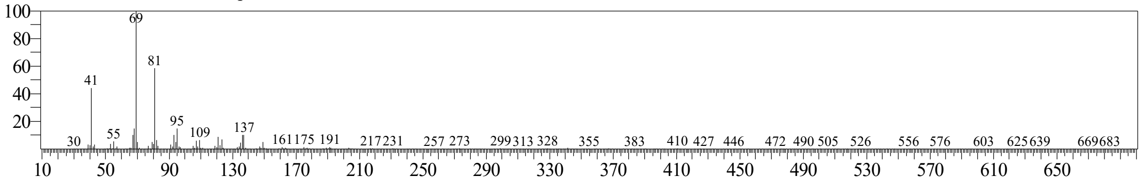

Hit#:1 Entry:186078 Library:NIST11.lib
SI:95 Formula:C30H50 CAS:111-02-4 MolWeight:410 RetIndex:2914
CompName:Squalene $$ 2,6,10,14,18,22-Tetracosahexaene, 2,6,10,15,19,23-hexamethyl-, (all-E)- $$ all-trans-Squalene $$ trans-Squalene $$ Spinacen $$ Spin

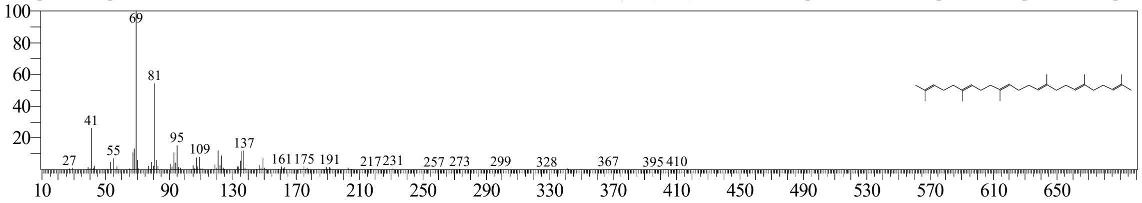

Hit#:2 Entry:110904 Library:NIST11.lib
SI:91 Formula:C20H34O CAS:7614-21-3 MolWeight:290 RetIndex:2192
CompName:Hexadeca-2,6,10,14-tetraen-1-ol, 3,7,11,16-tetramethyl- $$ 3,7,11,15-Tetramethyl-2,6,10,14-hexadecatetraen-1-ol $$ Tetraprenol $$

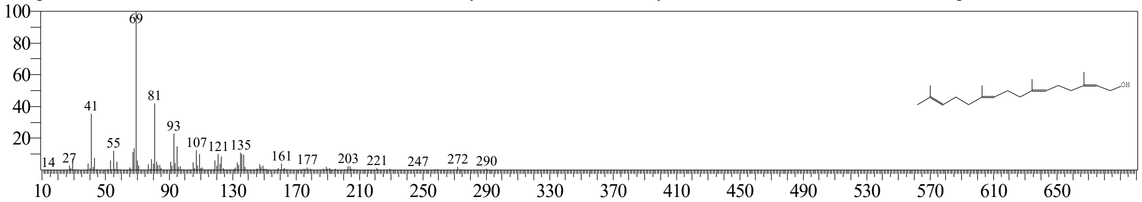

Hit#:3 Entry:150629 Library:NIST11.lib
SI:91 Formula:C25H42 CAS:75581-03-2 MolWeight:342 RetIndex:2432
CompName:2,6,10,14,18-Pentamethyl-2,6,10,14,18-eicosapentaene $$ (6E,10E,14E,18E)-2,6,10,14,18-Pentamethyl-2,6,10,14,18-icosapentaene # $$

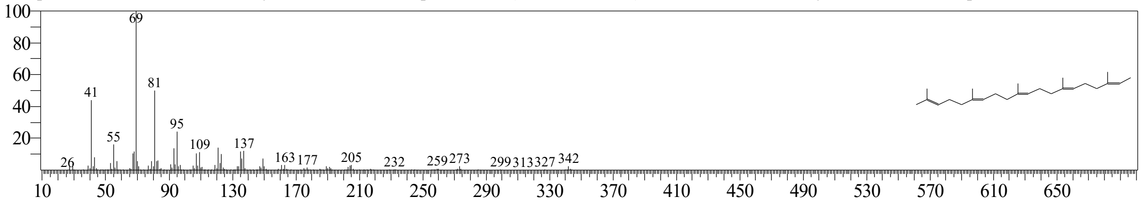

Hit#:4 Entry:59385 Library:NIST11.lib
SI:91 Formula:C15H26O CAS:0-00-0 MolWeight:222 RetIndex:1710
CompName:Farnesol isomer a $$ (2E,6E)-3,7,11-Trimethyl-2,6,10-dodecatrien-1-ol # $$

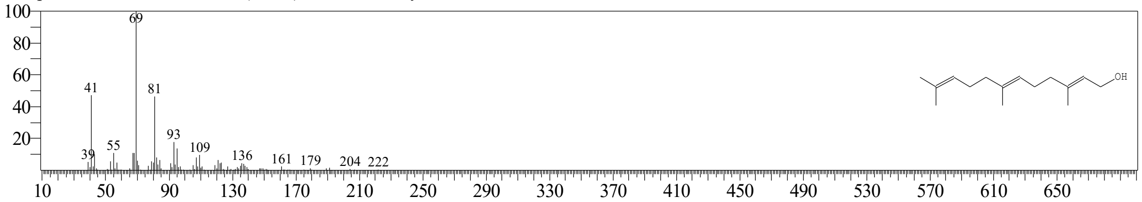

Hit#:5 Entry:175446 Library:NIST11.lib
SI:90 Formula:C27H44O CAS:0-00-0 MolWeight:384 RetIndex:2819
CompName:Docosa-2,6,10,14,18-pentaen-22-al, 2,6,10,15,18-pentamethyl-, all-trans $$ (4E,8E,12E,16E)-4,8,13,17,21-Pentamethyl-4,8,12,16,20-docosapenta

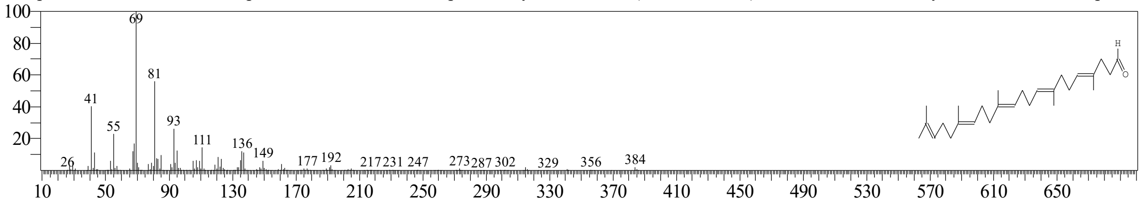

<< Target >>
Line#:175 R.Time:24.720(Scan#:4145) MassPeaks:344
RawMode:Averaged 24.715-24.725(4144-4146) BasePeak:81.05(7987)
BG Mode:Calc. from Peak Group 1 - Event 1 Scan

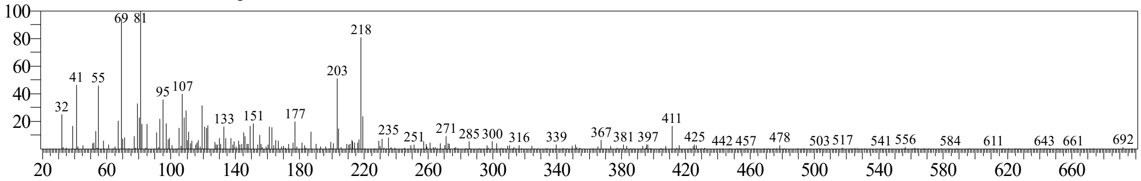

Hit#:1 Entry:186088 Library:NIST11.lib
SI:68 Formula:C30H50 CAS:464-97-1 MolWeight:410 RetIndex:2685
CompName:Urs-12-ene

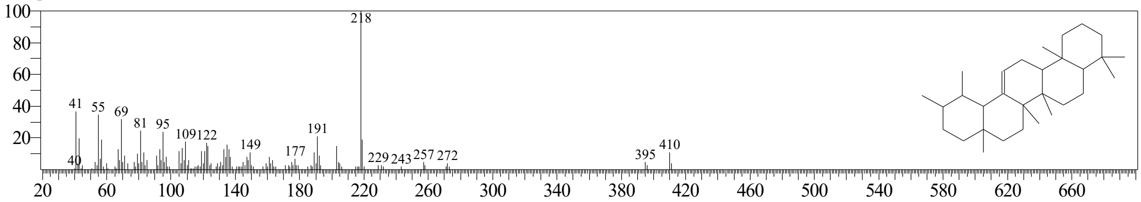

Hit#:2 Entry:190451 Library:NIST11.lib
SI:67 Formula:C30H48O CAS:0-00-0 MolWeight:424 RetIndex:2869
CompName:4,4,6a,6b,8a,11,11,14b-Octamethyl-1,4,4a,5,6,6a,6b,7,8,8a,9,10,11,12,12a,14,14a,14b-octadecahydro-2H-picen-3-one $$ Olean-12-en-3-one # $$

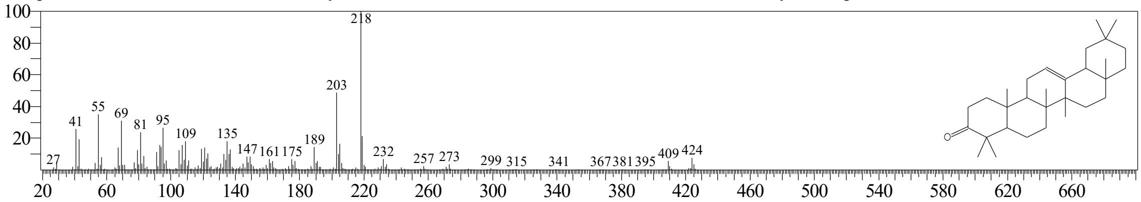

Hit#:3 Entry:186089 Library:NIST11.lib
SI:67 Formula:C30H50 CAS:56588-25-1 MolWeight:410 RetIndex:2669
CompName:D:A-Friedoolean-6-ene $$ 2,2,4a,6a,8a,9,12b,14a-Octamethyl-1,2,3,4,4a,5,6,6a,6b,8a,9,10,11,12,12a,12b,13,14,14a,14b-icosahydropicene # $$

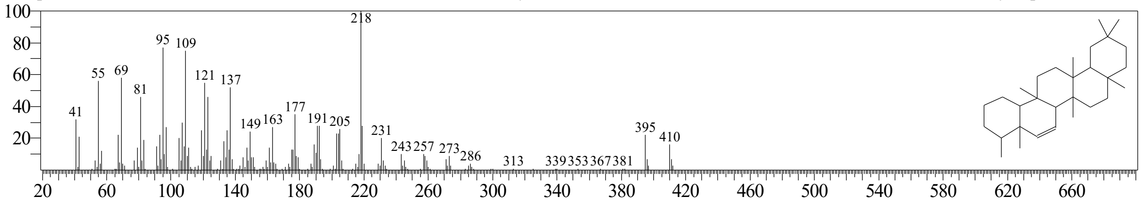

Hit#:4 Entry:197367 Library:NIST11.lib
SI:67 Formula:C33H56 CAS:0-00-0 MolWeight:452 RetIndex:2885
CompName:1,1,6-trimethyl-3-methylene-2-(3,6,9,13-tetramethyl-6-ethenye-10,14-dimethylene-pentadec-4-enyl)cyclohexane

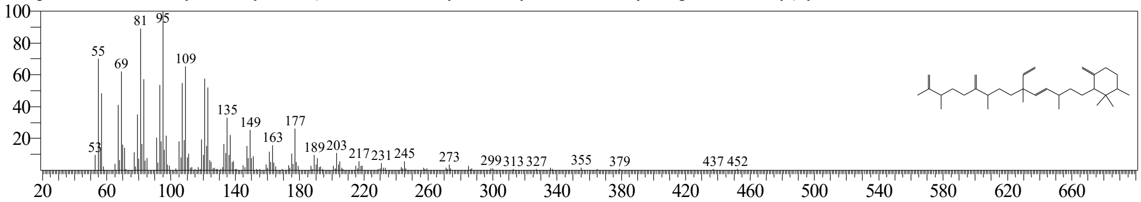

Hit#:5 Entry:132906 Library:NIST11.lib
SI:66 Formula:C22H38O CAS:0-00-0 MolWeight:318 RetIndex:2391
CompName:4,8,12,16-Octadecatetraen-1-ol, 4,9,13,17-tetramethyl- $$ (4E,8E,12E)-4,9,13,17-Tetramethyl-4,8,12,16-octadecatetraen-1-ol # $$

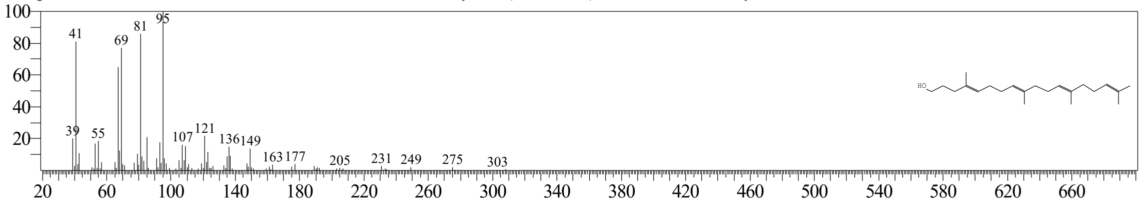

Qualitative Analysis Report

<< Target >>
Line#:176   R.Time:25.150(Scan#:4231) MassPeaks:409
RawMode:Averaged 25.145-25.155(4230-4232) BasePeak:43.05(73057)
BG Mode:Calc. from Peak Group 1 - Event 1 Scan

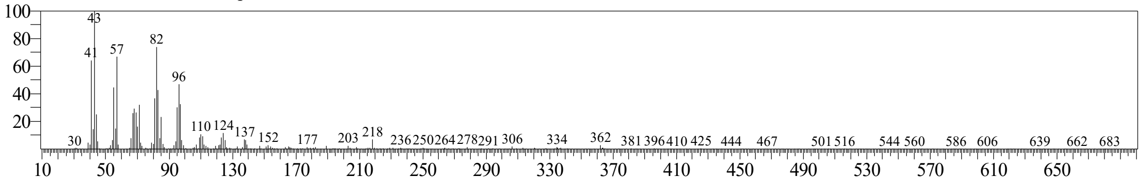

Hit#:1 Entry:93586 Library:NIST11.lib
SI:93 Formula:C18H36O CAS:638-66-4 MolWeight:268 RetIndex:1999
CompName:Octadecanal $$ Stearaldehyde $$ Octadecyl aldehyde $$ Stearyl aldehyde $$ n-Octadecanal $$

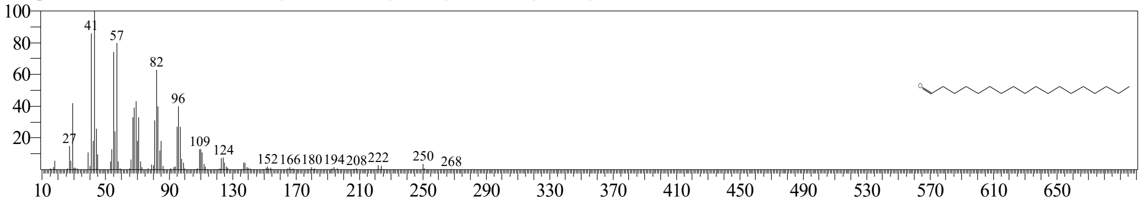

Hit#:2 Entry:82829 Library:NIST11.lib
SI:92 Formula:C17H34O CAS:0-00-0 MolWeight:254 RetIndex:1899
CompName:Heptadecanal

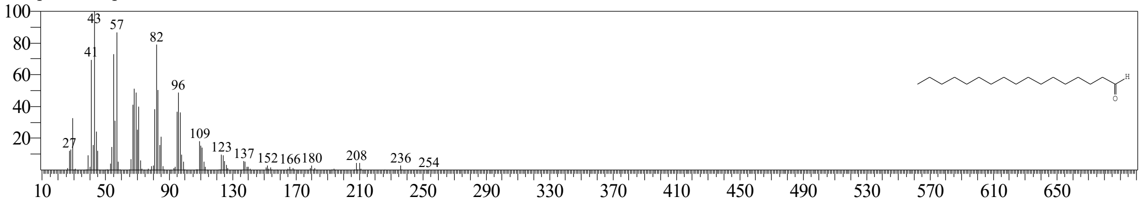

Hit#:3 Entry:93594 Library:NIST11.lib
SI:91 Formula:C18H36O CAS:0-00-0 MolWeight:268 RetIndex:2061
CompName:Z-2-Octadecen-1-ol $$ (2Z)-2-Octadecen-1-ol # $$

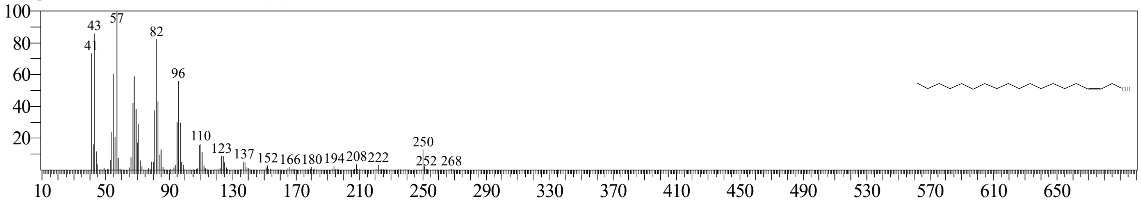

Hit#:4 Entry:42885 Library:NIST11.lib
SI:90 Formula:C13H26O CAS:10486-19-8 MolWeight:198 RetIndex:1502
CompName:Tridecanal $$ n-Tridecylaldehyde $$ Tridecanaldehyde $$ Tridecyl aldehyde $$ 1-Tridecanal $$ Tridecane aldehyde $$ n-Tridecanal $$

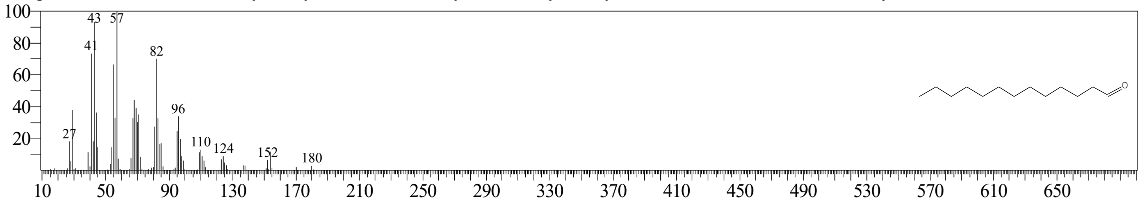

Hit#:5 Entry:52426 Library:NIST11.lib
SI:90 Formula:C14H28O CAS:0-00-0 MolWeight:212 RetIndex:1664
CompName:E-2-Tetradecen-1-ol $$ (2E)-2-Tetradecen-1-ol # $$

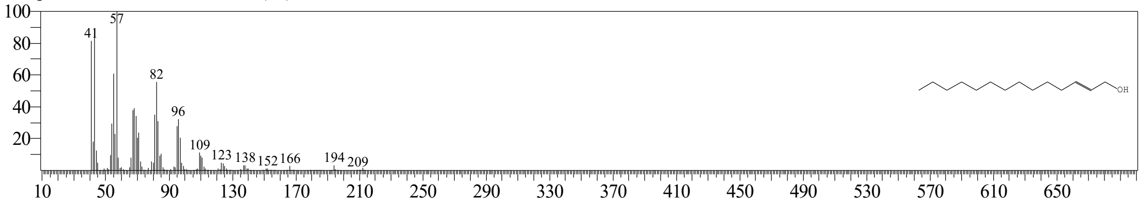

<< Target >>
Line#:177 R.Time:25.585(Scan#:4318) MassPeaks:393
RawMode:Averaged 25.580-25.590(4317-4319) BasePeak:95.10(13771)
BG Mode:Calc. from Peak Group 1 - Event 1 Scan

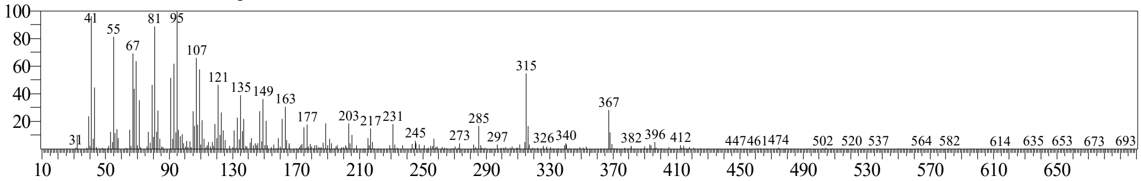

Hit#:1 Entry:123529 Library:NIST11.lib
SI:79 Formula:C20H34O2 CAS:7220-78-2 MolWeight:306 RetIndex:2400
CompName:4,8,13-Cyclotetradecatriene-1,3-diol, 1,5,9-trimethyl-12-(1-methylethyl)- $$ FW 306 $$ 4,8,13-Duvatriene-1,3-Diol $$ 12-Isopropyl-1,5,9-trimethy

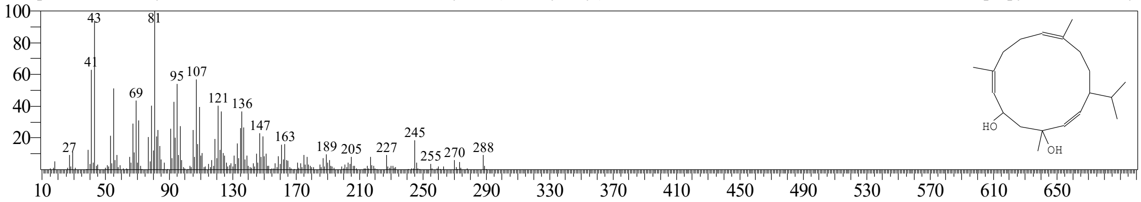

Hit#:2 Entry:143389 Library:NIST11.lib
SI:78 Formula:C23H40O CAS:106932-90-5 MolWeight:332 RetIndex:2385
CompName:Oct-5-en-2-ol, 8-(1,4,4a,5,6,7,8,8a-octahydro-2, 5, 5, 8a-tetramethylnaphth-1-yl)-6-methyl- $$ (5E)-8-(2,5,5,8a-Tetramethyl-1,4,4a,5,6,7,8,8a-octah

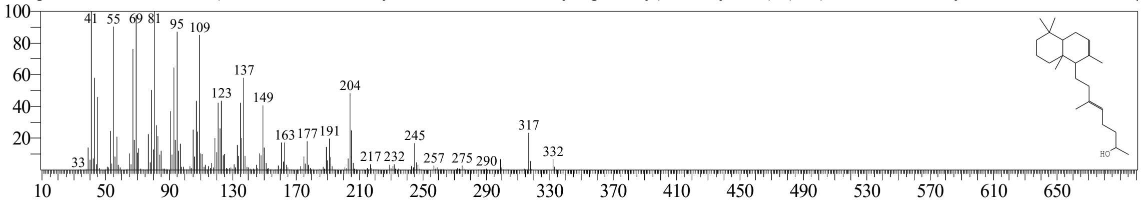

Hit#:3 Entry:59398 Library:NIST11.lib
SI:77 Formula:C15H26O CAS:0-00-0 MolWeight:222 RetIndex:1555
CompName:6-epi-shyobunol $$ elema-1,3-dien-6.alpha.-ol $$

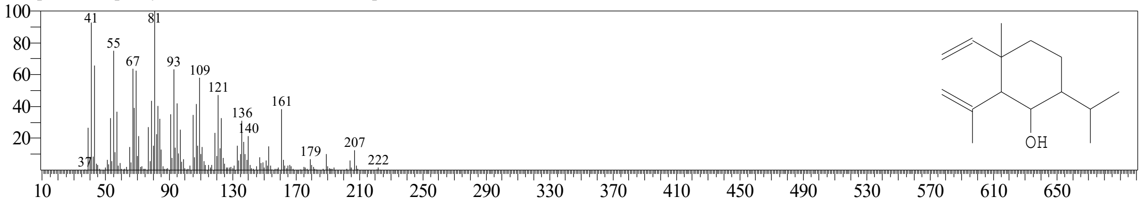

Hit#:4 Entry:76882 Library:NIST11.lib
SI:76 Formula:C18H30 CAS:0-00-0 MolWeight:246 RetIndex:1649
CompName:d-Norandrostane (5.alpha.,14.alpha.) $$ 8a,10a-Dimethylhexadecahydrocyclobuta[a]phenanthrene # $$

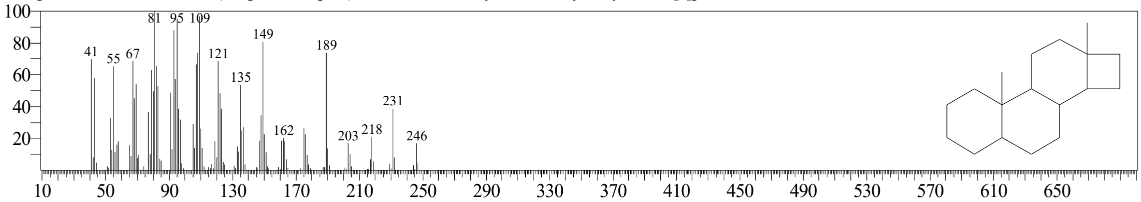

Hit#:5 Entry:187514 Library:NIST11.lib
SI:76 Formula:C29H50O CAS:54411-91-5 MolWeight:414 RetIndex:2584
CompName:Stigmastane, 23,24-epoxy-, (5.alpha.)- $$ Ergostane, 23,24-epoxy-, [5.alpha.,22(trans)]- $$ 20-[3-(1-Ethyl-2-methylpropyl)-2-oxiranyl]pregnane # $

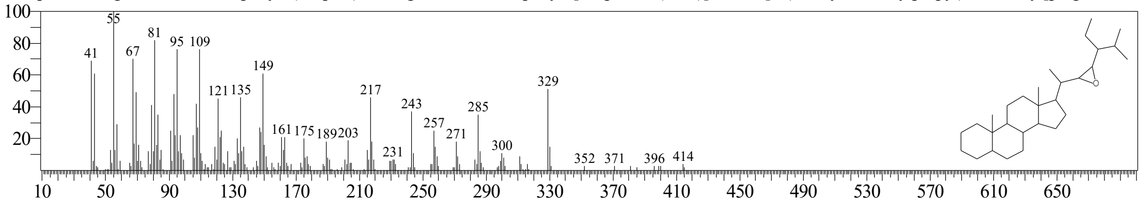

<< Target >>
Line#:178 R.Time:26.495(Scan#:4500) MassPeaks:407
RawMode:Averaged 26.490-26.500(4499-4501) BasePeak:177.15(6604)
BG Mode:Calc. from Peak Group 1 - Event 1 Scan

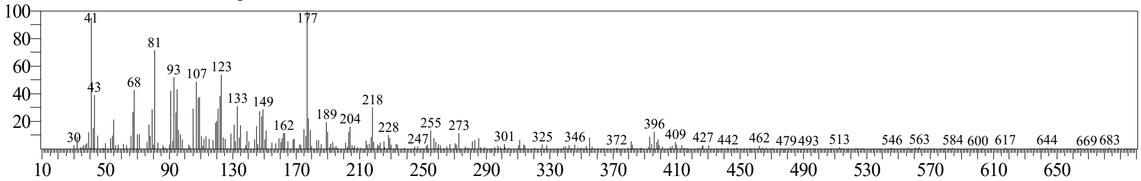

Hit#:1 Entry:46768 Library:NIST11.lib
SI:71 Formula:C15H24 CAS:473-13-2 MolWeight:204 RetIndex:1474
CompName:Naphthalene, 1,2,3,4,4a,5,6,8a-octahydro-4a,8-dimethyl-2-(1-methylethenyl)-, [2R-(2.alpha.,4a.alpha.,8a.beta.)]- $$ .alpha.-Selinene $$ 2-Isopropen

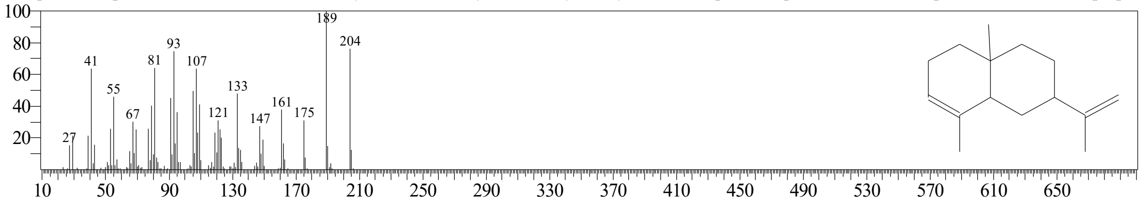

Hit#:2 Entry:56368 Library:NIST11.lib
SI:70 Formula:C15H22O CAS:102681-49-2 MolWeight:218 RetIndex:1438
CompName:2H-Cyclopropa[g]benzofuran, 4,5,5a,6,6a,6b-hexahydro-4,4,6b-trimethyl-2-(1-methylethenyl)- $$ 2-Isopropenyl-4,4,6b-trimethyl-4,5,5a,6,6a,6b-he

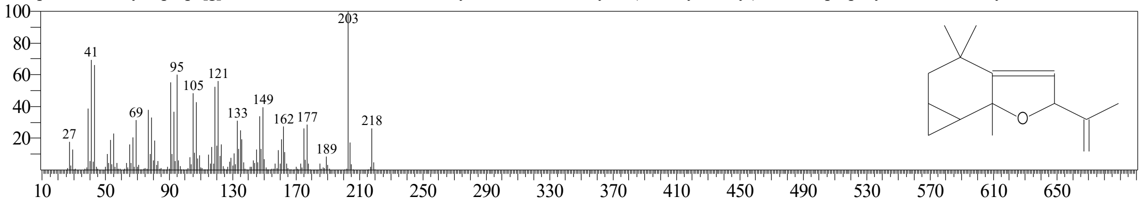

Hit#:3 Entry:59457 Library:NIST11.lib
SI:70 Formula:C15H26O CAS:25826-85-1 MolWeight:222 RetIndex:1651
CompName:5.beta.,7.beta.H,10.alpha.-Eudesm-11-en-1.alpha.-ol $$ 6-Isopropenyl-4,8a-dimethyldecahydro-1-naphthalenol # $$

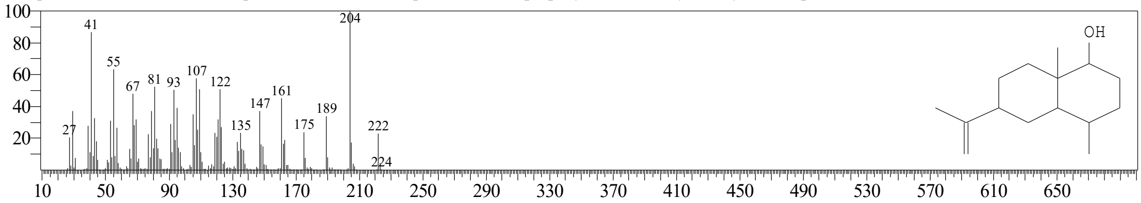

Hit#:4 Entry:46652 Library:NIST11.lib
SI:70 Formula:C15H24 CAS:3691-12-1 MolWeight:204 RetIndex:1490
CompName:.alpha.-Guaiene $$ Azulene, 1,2,3,4,5,6,7,8-octahydro-1,4-dimethyl-7-(1-methylethenyl)-, [1S-(1.alpha.,4.alpha.,7.alpha.)]- $$ Guaia-1(5),11-diene

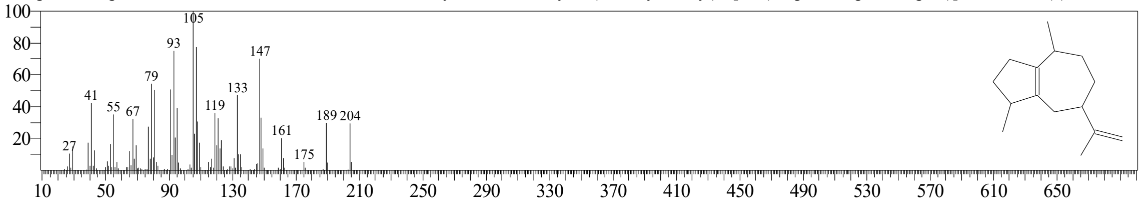

Hit#:5 Entry:46667 Library:NIST11.lib
SI:70 Formula:C15H24 CAS:3691-11-0 MolWeight:204 RetIndex:1490
CompName:Azulene, 1,2,3,5,6,7,8,8a-octahydro-1,4-dimethyl-7-(1-methylethenyl)-, [1S-(1.alpha.,7.alpha.,8a.beta.)]- $$ Guaia-1(10),11-diene $$ .alpha.-Bulnes

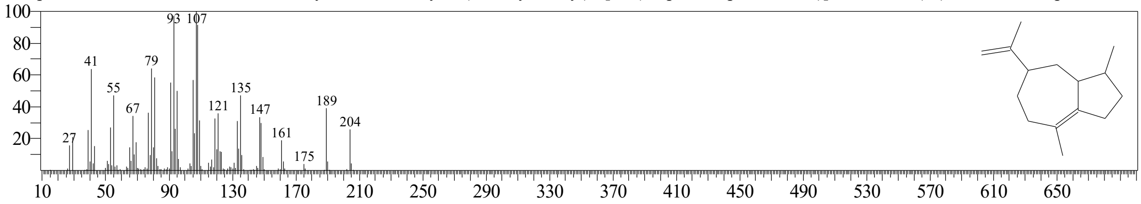

Qualitative Analysis Report

<< Target >>
Line#:179 R.Time:27.110(Scan#:4623) MassPeaks:401
RawMode:Averaged 27.105-27.115(4622-4624) BasePeak:43.05(134686)
BG Mode:Calc. from Peak Group 1 - Event 1 Scan

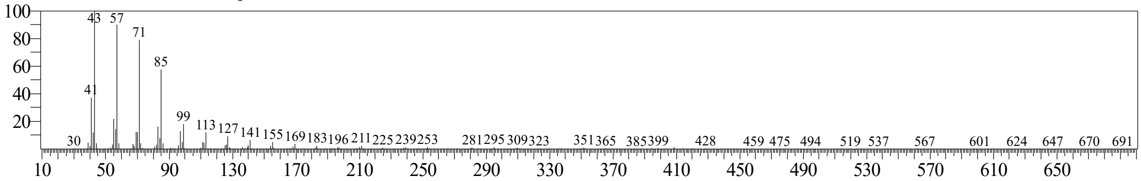

Hit#:1 Entry:104535 Library:NIST11.lib
SI:93 Formula:C20H42 CAS:112-95-8 MolWeight:282 RetIndex:2009
CompName:Eicosane $$ n-Eicosane $$ Icosane # $$ n-Icosane $$

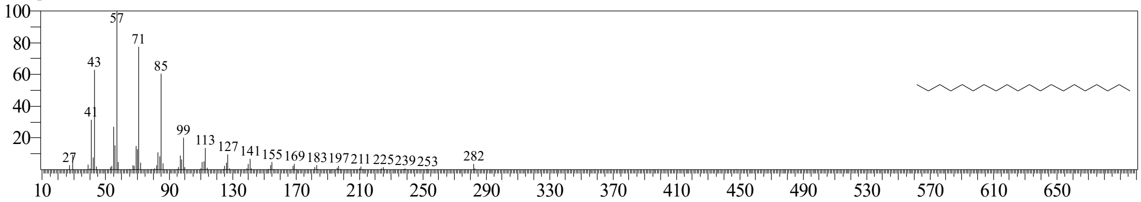

Hit#:2 Entry:104536 Library:NIST11.lib
SI:92 Formula:C20H42 CAS:13287-24-6 MolWeight:282 RetIndex:1945
CompName:Nonadecane, 9-methyl- $$ 9-Methylnonadecane # $$ 9-Methylnonadecane $$

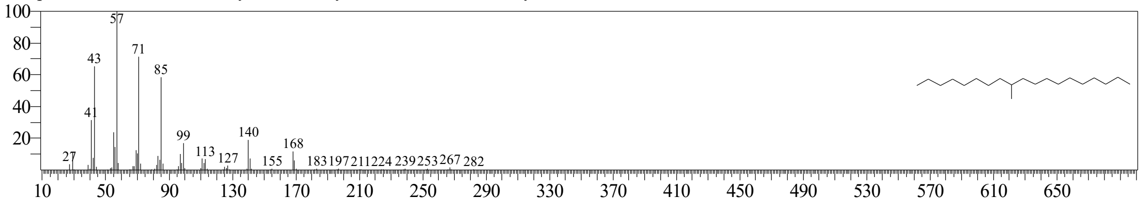

Hit#:3 Entry:115539 Library:NIST11.lib
SI:92 Formula:C21H44 CAS:54833-23-7 MolWeight:296 RetIndex:2045
CompName:Eicosane, 10-methyl- $$ 10-Methylicosane # $$ 10-Methyleicosane $$

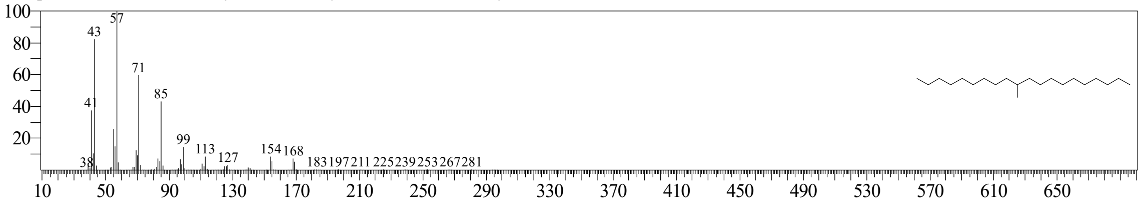

Hit#:4 Entry:173386 Library:NIST11.lib
SI:92 Formula:C27H56 CAS:593-49-7 MolWeight:380 RetIndex:2705
CompName:Heptacosane $$ n-Heptacosane $$

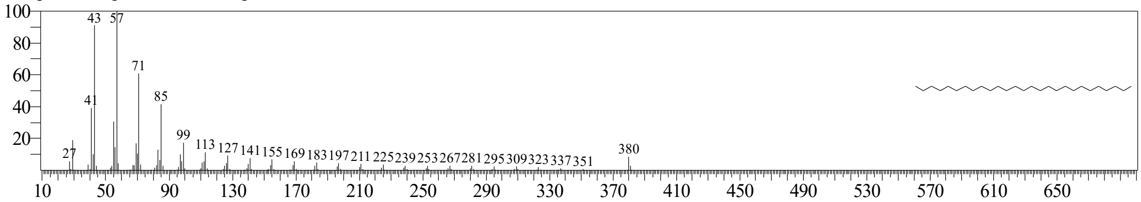

Hit#:5 Entry:165568 Library:NIST11.lib
SI:92 Formula:C26H54 CAS:55282-11-6 MolWeight:366 RetIndex:2477
CompName:Heneicosane, 11-(1-ethylpropyl)- $$ 11-(1-Ethylpropyl)heneicosane $$ 11-(3'-n-Pentyl)heneicosane $$ 11-(1-Ethylpropyl)henicosane # $$

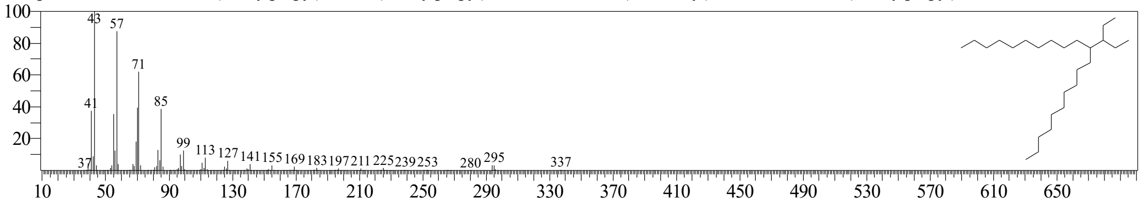

<< Target >>
Line#:180 R.Time:27.305(Scan#:4662) MassPeaks:365
RawMode:Averaged 27.300-27.310(4661-4663) BasePeak:83.10(4617)
BG Mode:Calc. from Peak Group 1 - Event 1 Scan

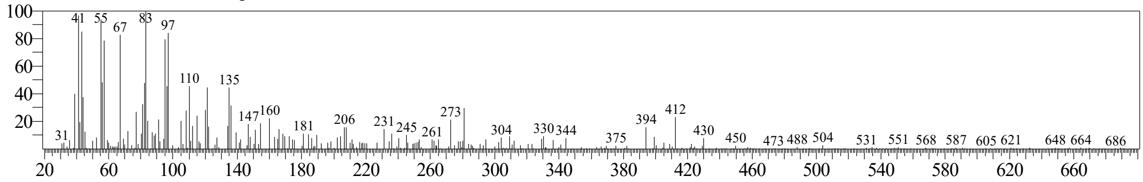

Hit#:1 Entry:17623 Library:NIST11.lib
SI:65 Formula:C10H18O CAS:54244-87-0 MolWeight:154 RetIndex:1170
CompName:Cyclohexanol, 2-methylene-3-(1-methylethyl)-, cis- $$ 3-Isopropyl-2-methylenecyclohexanol # $$

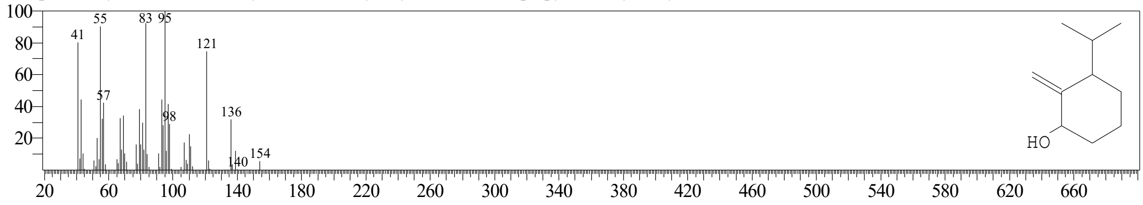

Hit#:2 Entry:41448 Library:NIST11.lib
SI:64 Formula:C12H20O2 CAS:0-00-0 MolWeight:196 RetIndex:1500
CompName:10-Methylundec-2-en-4-olide

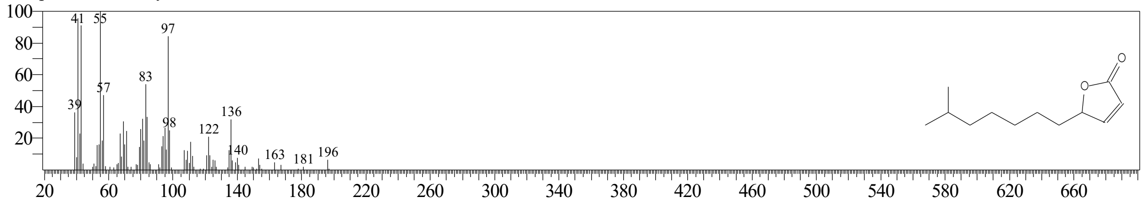

Hit#:3 Entry:52253 Library:NIST11.lib
SI:64 Formula:C13H24O2 CAS:63521-76-6 MolWeight:212 RetIndex:1690
CompName:Tridecanedial

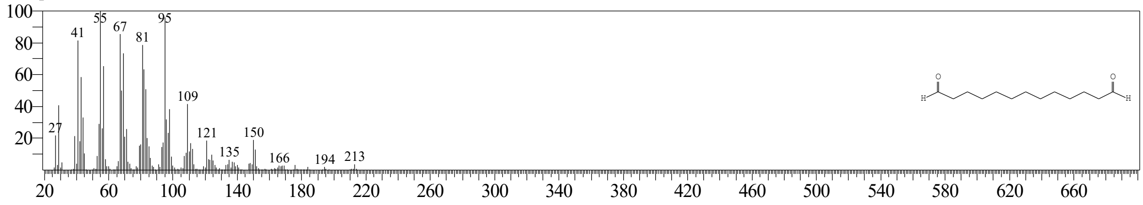

Hit#:4 Entry:24762 Library:NIST11.lib
SI:63 Formula:C11H20O CAS:54972-52-0 MolWeight:168 RetIndex:1363
CompName:1-Naphthalenol, decahydro-4a-methyl- $$ 4a-Methyldecahydro-1-naphthalenol # $$

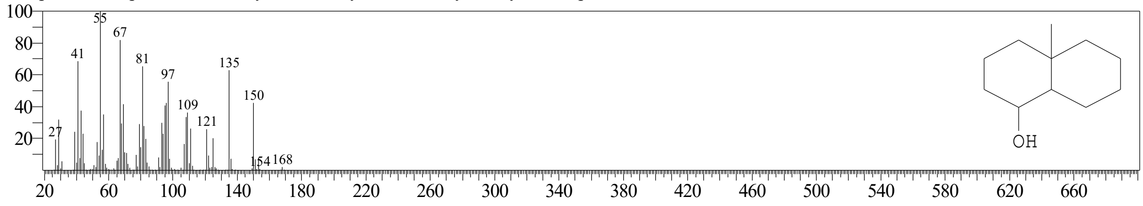

Hit#:5 Entry:17456 Library:NIST11.lib
SI:63 Formula:C10H18O CAS:90026-62-3 MolWeight:154 RetIndex:1125
CompName:Bicyclo[4.1.0]heptan-2-ol, 3,7,7-trimethyl-, (1.alpha.,2.alpha.,3.beta.,6.alpha.)- $$ 3,7,7-Trimethylbicyclo[4.1.0]heptan-2-ol # $$

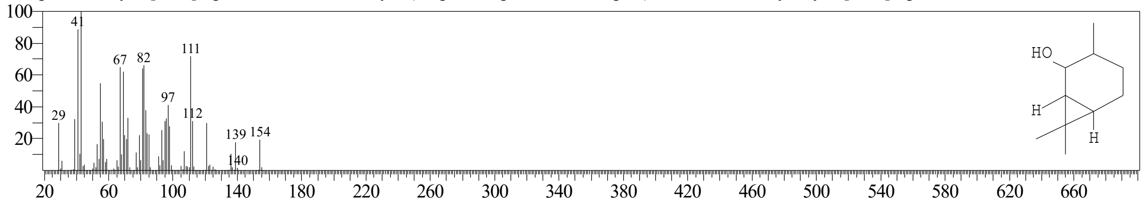

<< Target >>
Line#:181 R.Time:27.670(Scan#:4735) MassPeaks:365
RawMode:Averaged 27.665-27.675(4734-4736) BasePeak:71.05(3493)
BG Mode:Calc. from Peak Group 1 - Event 1 Scan

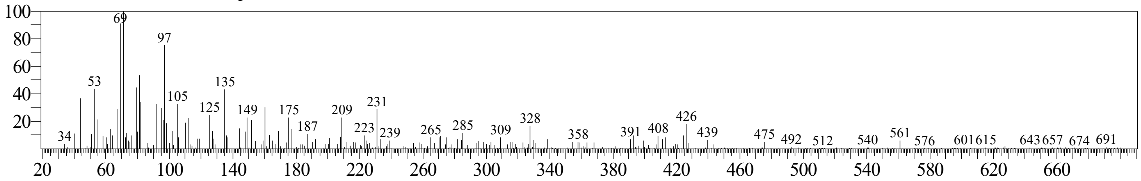

Hit#:1 Entry:207214 Library:NIST11.lib
SI:53 Formula:C37H70O CAS:133530-21-9 MolWeight:530 RetIndex:3851
CompName:Z,Z-6,28-Heptatriactontadien-2-one $$ Z,Z-6,28-Heptatriacontadien-2-one $$ (6Z,28Z)-6,28-Heptatriacontadien-2-one # $$

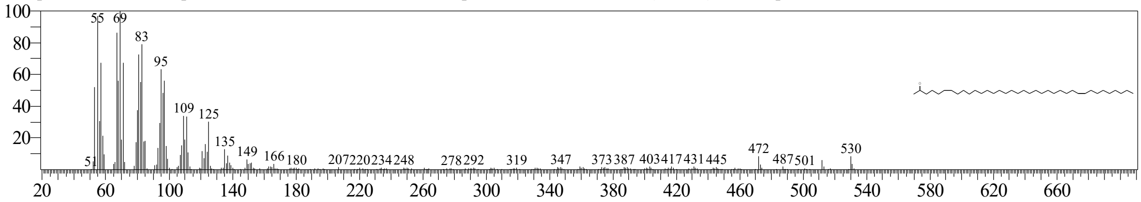

Hit#:2 Entry:18200 Library:NIST11.lib
SI:53 Formula:C7H8O4 CAS:5831-38-9 MolWeight:156 RetIndex:1404
CompName:7-Oxabicyclo[4.1.0]hept-3-en-2-one, 5-hydroxy-3-(hydroxymethyl)- $$ 5-Hydroxy-3-(hydroxymethyl)-7-oxabicyclo[4.1.0]hept-3-en-2-one # $$

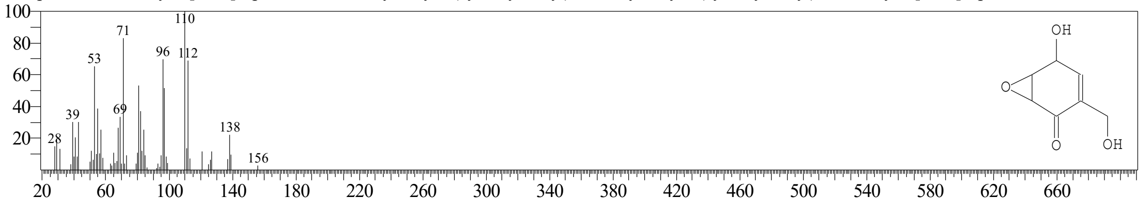

Hit#:3 Entry:206268 Library:NIST11.lib
SI:52 Formula:C36H68O CAS:133530-19-5 MolWeight:516 RetIndex:3752
CompName:Z,Z-6,27-Hexatriactontadien-2-one $$ (6Z,27Z)-6,27-Hexatriacontadien-2-one # $$

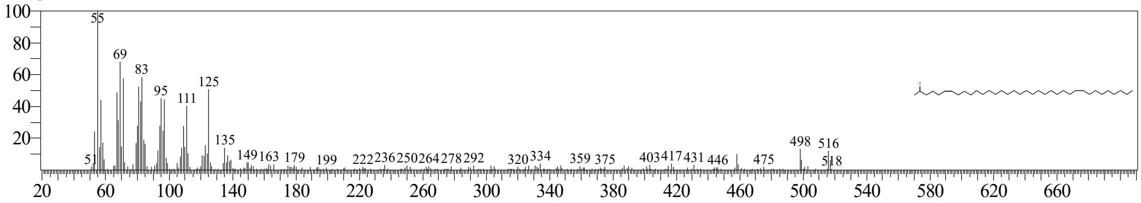

Hit#:4 Entry:126861 Library:NIST11.lib
SI:51 Formula:C16H25NO3S CAS:0-00-0 MolWeight:311 RetIndex:0
CompName:1-(10,10-Dimethyl-3,3-dioxo-3-thia-4-azatricyclo[5.2.1.0(1,5)]dec-4-yl)-3-methylpent-4-en-1-one

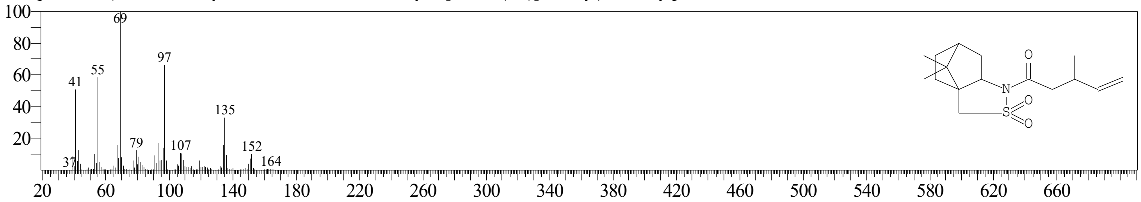

Hit#:5 Entry:204986 Library:NIST11.lib
SI:51 Formula:C35H66O CAS:133530-17-3 MolWeight:502 RetIndex:3652
CompName:Z,Z-6,26-Pentatriacontadien-2-one $$ (6E,26E)-6,26-Pentatriacontadien-2-one # $$

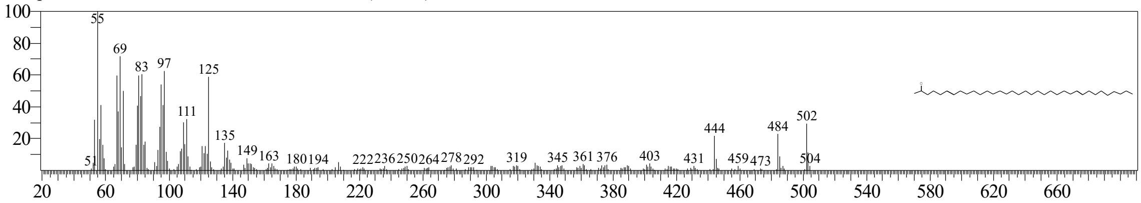

<< Target >>
Line#:182 R.Time:27.685(Scan#:4738) MassPeaks:380
RawMode:Averaged 27.680-27.690(4737-4739) BasePeak:69.10(9055)
BG Mode:Calc. from Peak Group 1 - Event 1 Scan

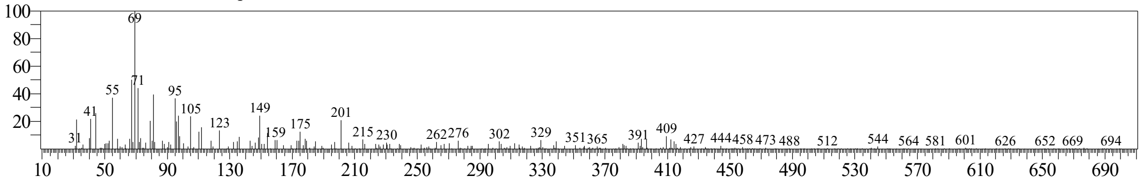

Hit#:1 Entry:61684 Library:NIST11.lib
SI:58 Formula:C9H13ClF2O2 CAS:0-00-0 MolWeight:226 RetIndex:1081
CompName:1-Cyclopentylethanol, chlorodifluoroacetate

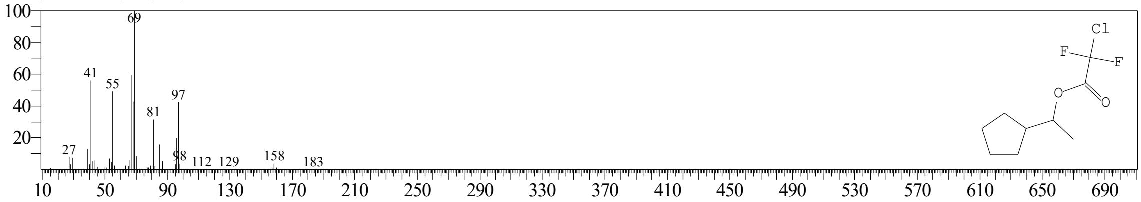

Hit#:2 Entry:18655 Library:NIST11.lib
SI:57 Formula:C10H20O CAS:1117-61-9 MolWeight:156 RetIndex:1179
CompName:6-Octen-1-ol, 3,7-dimethyl-, (R)- $$ (R)-(+)-.beta.-Citronellol $$ 3,7-Dimethyl-6-octen-1-ol # $$

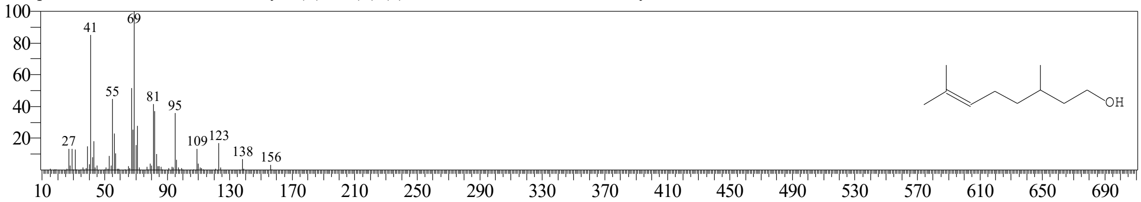

Hit#:3 Entry:17439 Library:NIST11.lib
SI:57 Formula:C10H18O CAS:141-26-4 MolWeight:154 RetIndex:1107
CompName:7-Octenal, 3,7-dimethyl- $$ Rhodinal $$ 3,7-Dimethyl-7-octenal $$

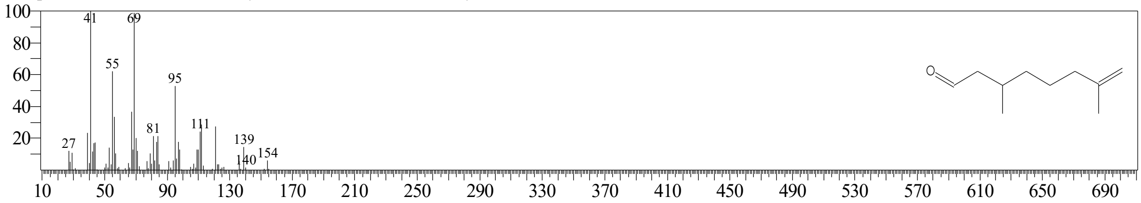

Hit#:4 Entry:18659 Library:NIST11.lib
SI:57 Formula:C10H20O CAS:21721-18-6 MolWeight:156 RetIndex:1160
CompName:Cyclopentaneethanol, .beta.,2,3-trimethyl- $$ 2-(2,3-Dimethylcyclopentyl)-1-propanol # $$

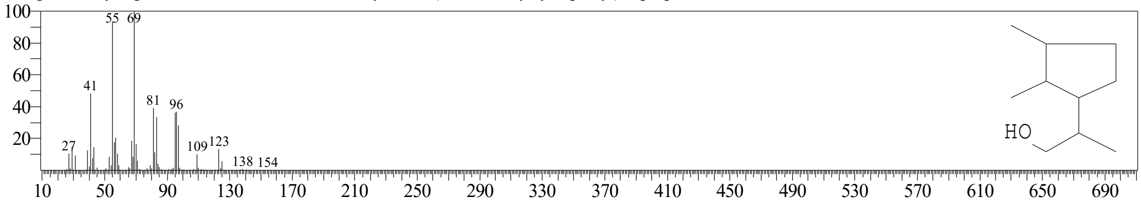

Hit#:5 Entry:38735 Library:NIST11.lib
SI:57 Formula:C13H20O CAS:0-00-0 MolWeight:192 RetIndex:1397
CompName:Bicyclo[3.1.1]heptan-3-one, 2-(but-3-enyl)-6,6-dimethyl- $$ 2-(3-Butenyl)-6,6-dimethylbicyclo[3.1.1]heptan-3-one # $$

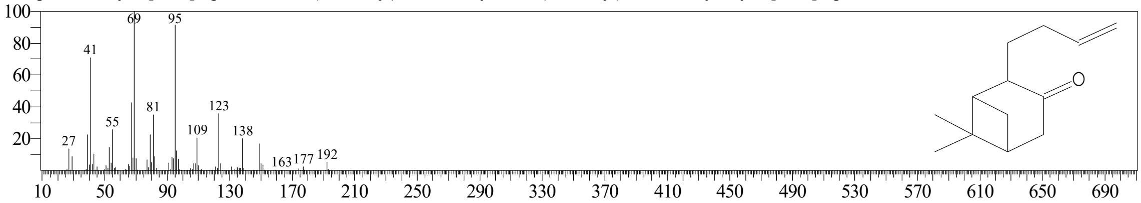

Qualitative Analysis Report

<< Target >>
Line#:183 R.Time:30.975(Scan#:5396) MassPeaks:370
RawMode:Averaged 30.970-30.980(5395-5397) BasePeak:43.05(78179)
BG Mode:Calc. from Peak Group 1 - Event 1 Scan

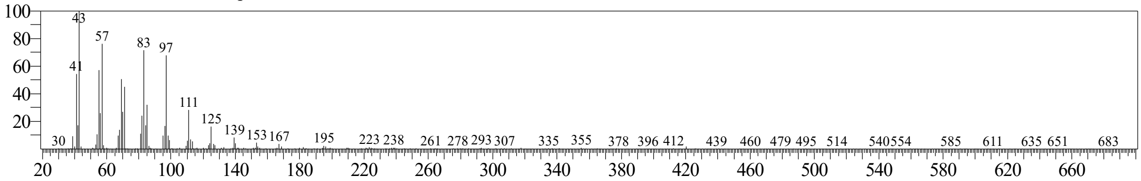

Hit#:1 Entry:197101 Library:NIST11.lib
SI:94 Formula:C21H35F7O2 CAS:959085-66-6 MolWeight:452 RetIndex:1833
CompName:Heptadecyl heptafluorobutyrate $$ Heptadecyl 2,2,3,3,4,4,4-heptafluorobutanoate $$ 1-Heptadecanol, heptafluorobutyrate $$ Heptadecyl perfluorob

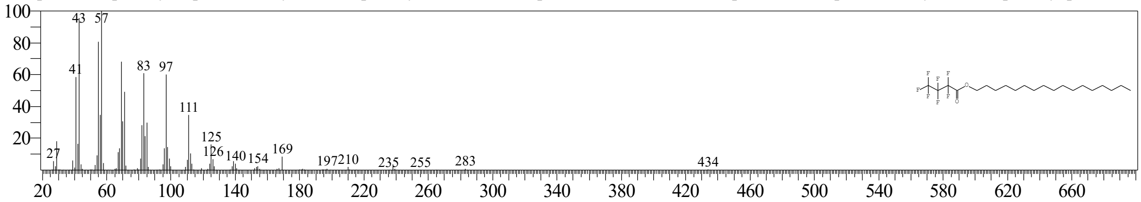

Hit#:2 Entry:135759 Library:NIST11.lib
SI:94 Formula:C23H46 CAS:18835-32-0 MolWeight:322 RetIndex:2298
CompName:1-Tricosene $$ Tricosene-1 $$

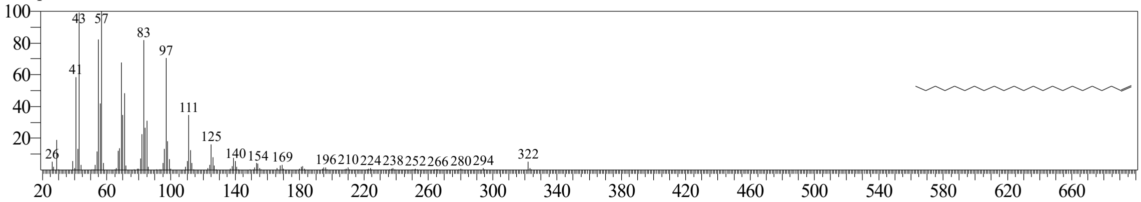

Hit#:3 Entry:155811 Library:NIST11.lib
SI:93 Formula:C25H50 CAS:0-00-0 MolWeight:350 RetIndex:2514
CompName:Z-12-Pentacosene $$ (12Z)-12-Pentacosene # $$

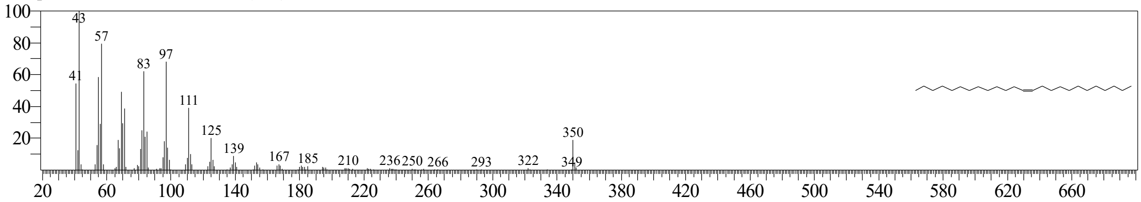

Hit#:4 Entry:165282 Library:NIST11.lib
SI:93 Formula:C20H37F3O2 CAS:79392-43-1 MolWeight:366 RetIndex:2011
CompName:Octadecyl trifluoroacetate $$ Octadecyl 2,2,2-trifluoroacetate $$ 1-Octadecanol, trifluoroacetate $$ Trifluoroacetic acid, n-octadecyl ester $$ Acetic

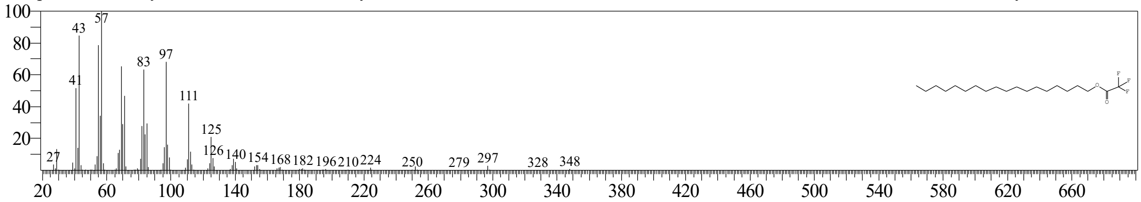

Hit#:5 Entry:205440 Library:NIST11.lib
SI:93 Formula:C25H43F7O2 CAS:0-00-0 MolWeight:508 RetIndex:2230
CompName:Heneicosyl heptafluorobutyrate $$ Heneicosyl 2,2,3,3,4,4,4-heptafluorobutanoate $$ 1-Heneicosanol, heptafluorobutyrate $$ Heneicosyl perfluorob

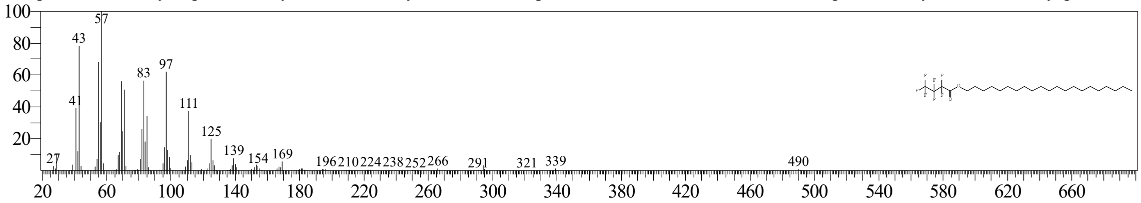

Qualitative Analysis Report

<< Target >>
Line#:184 R.Time:31.135(Scan#:5428) MassPeaks:360
RawMode:Averaged 31.130-31.140(5427-5429) BasePeak:41.00(2004)
BG Mode:Calc. from Peak Group 1 - Event 1 Scan

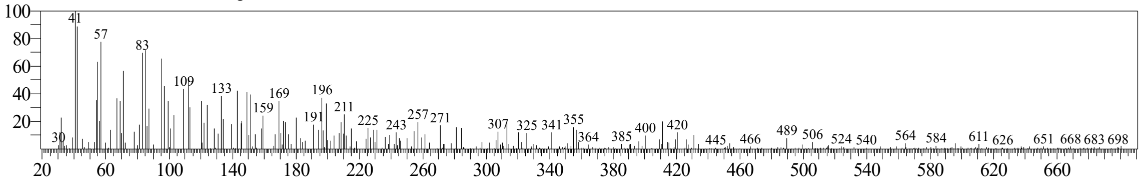

Hit#:1 Entry:138642 Library:NIST11.lib
SI:54 Formula:C20H38O3 CAS:0-00-0 MolWeight:326 RetIndex:2243
CompName:Carbonic acid, allyl hexadecyl ester

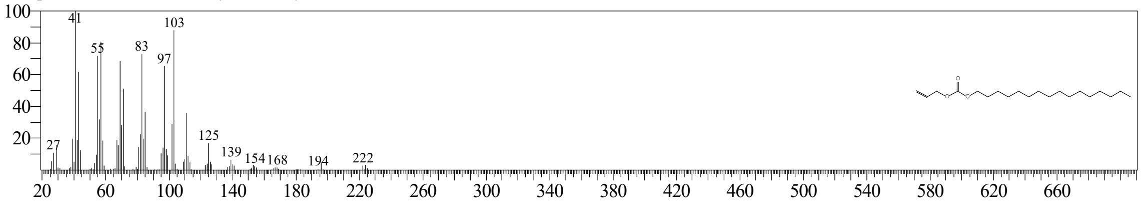

Hit#:2 Entry:177910 Library:NIST11.lib
SI:54 Formula:C20H39BrO2 CAS:0-00-0 MolWeight:390 RetIndex:2473
CompName:4-Bromobutanoic acid, hexadecyl ester $$ Hexadecyl 4-bromobutanoate # $$

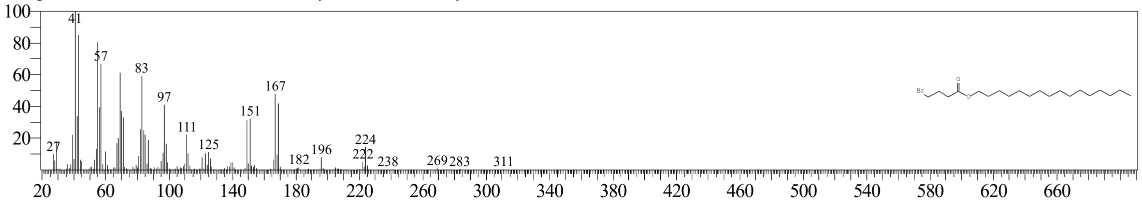

Hit#:3 Entry:158379 Library:NIST11.lib
SI:54 Formula:C22H42O3 CAS:0-00-0 MolWeight:354 RetIndex:2441
CompName:Carbonic acid, allyl octadecyl ester

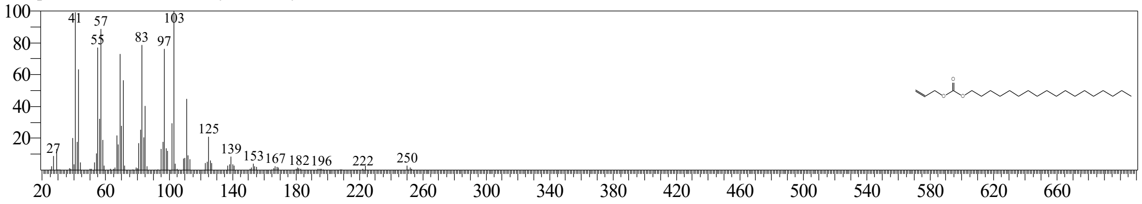

Hit#:4 Entry:104510 Library:NIST11.lib
SI:54 Formula:C19H38O CAS:101395-45-3 MolWeight:282 RetIndex:1789
CompName:2,6,10,14-Tetramethylpentadecan-3-one $$ 2,6,10,14-Tetramethyl-3-pentadecanone # $$

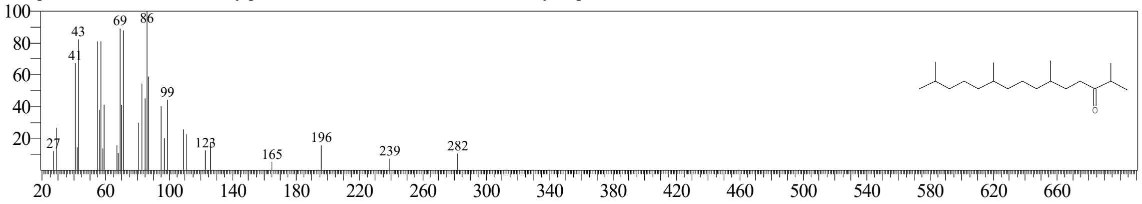

Hit#:5 Entry:128039 Library:NIST11.lib
SI:53 Formula:C19H36O3 CAS:0-00-0 MolWeight:312 RetIndex:2143
CompName:Carbonic acid, allyl pentadecyl ester

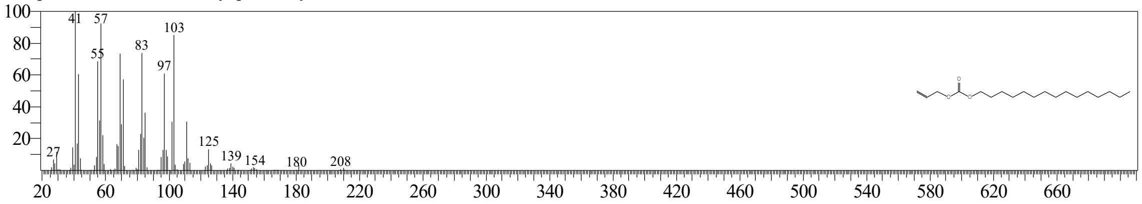

<< Target >>
Line#:185 R.Time:31.155(Scan#:5432) MassPeaks:354
RawMode:Averaged 31.150-31.160(5431-5433) BasePeak:41.05(5277)
BG Mode:Calc. from Peak Group 1 - Event 1 Scan

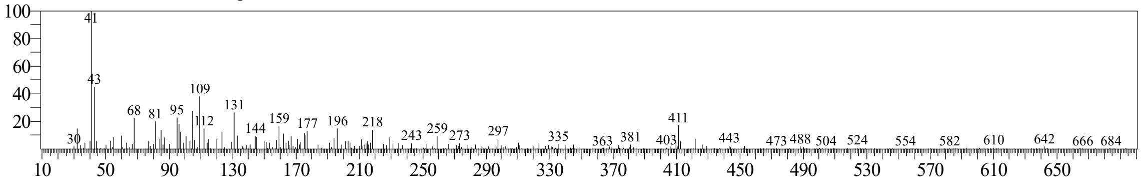

Hit#:1 Entry:57730 Library:NIST11.lib
SI:50 Formula:C15H24O CAS:0-00-0 MolWeight:220 RetIndex:1293
CompName:Ledene oxide-(II) $$ 1,4a,7,7-Tetramethyldecahydrocyclopropa[7,8]azuleno[3a,4-b]oxirene # $$

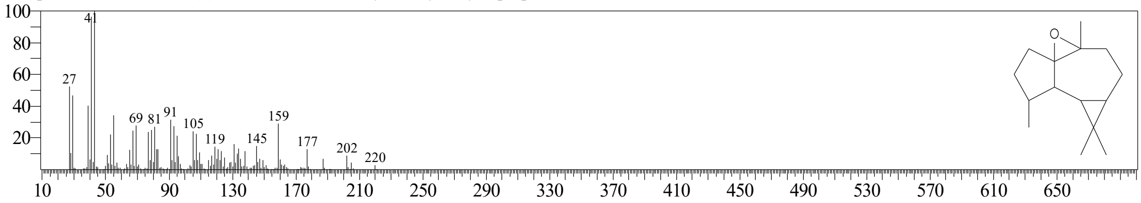

Hit#:2 Entry:10448 Library:NIST11.lib
SI:50 Formula:C9H14O CAS:85136-08-9 MolWeight:138 RetIndex:1015
CompName:2,6-Heptadienal, 2,4-dimethyl-

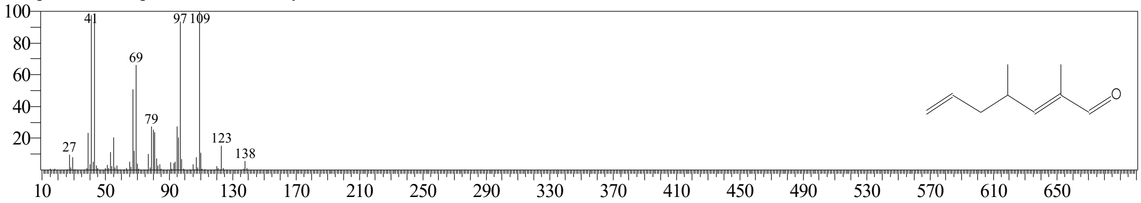

Hit#:3 Entry:208114 Library:NIST11.lib
SI:49 Formula:C37H52O3 CAS:0-00-0 MolWeight:544 RetIndex:3726
CompName:Carpesterol dehydrate $$ (20Z)-4-Methyl-6-oxostigmasta-7,20(22)-dien-3-yl benzoate # $$

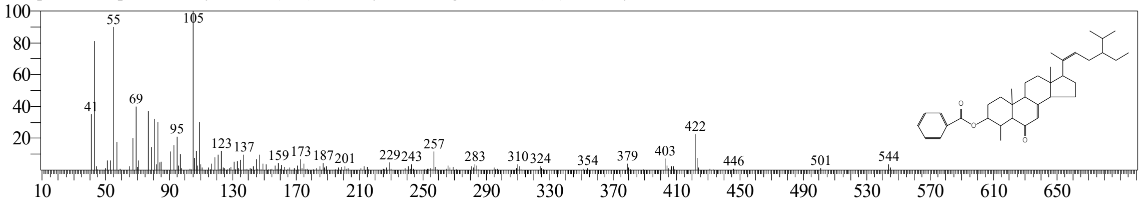

Hit#:4 Entry:93456 Library:NIST11.lib
SI:49 Formula:C17H32O2 CAS:0-00-0 MolWeight:268 RetIndex:1868
CompName:Heptanoic acid, 9-decen-1-yl ester $$ 9-Decenyl heptanoate # $$

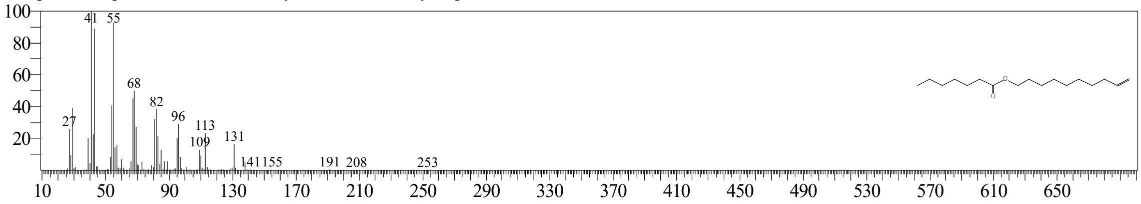

Hit#:5 Entry:78143 Library:NIST11.lib
SI:49 Formula:C15H20O3 CAS:0-00-0 MolWeight:248 RetIndex:1989
CompName:3H-Naphtho[2,3-b]furan-2-one, 4-hydroxy-4a,5-dimethyl-3-methylene-3a,4,4a,5,6,7,9,9a-octahydro-

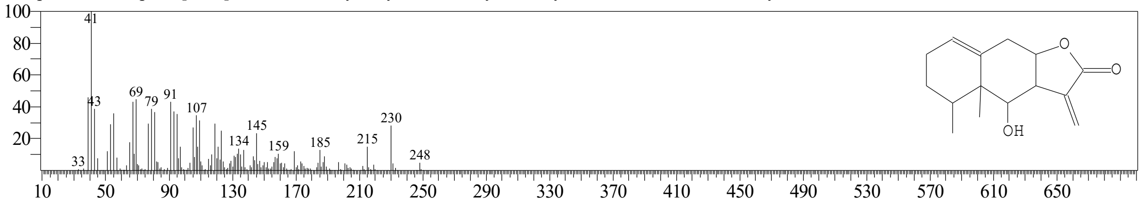

Qualitative Analysis Report

<< Target >>
Line#:186 R.Time:31.255(Scan#:5452) MassPeaks:370
RawMode:Averaged 31.250-31.260(5451-5453) BasePeak:69.10(3614)
BG Mode:Calc. from Peak Group 1 - Event 1 Scan

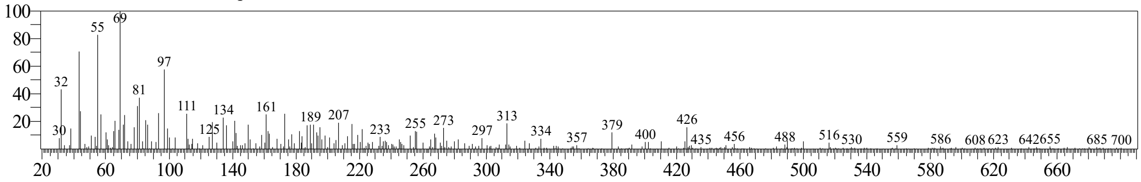

Hit#:1 Entry:17544 Library:NIST11.lib
SI:50 Formula:C10H18O CAS:17983-26-5 MolWeight:154 RetIndex:1151
CompName:Ketone, 2,2-dimethylcyclohexyl methyl $$ 1-(2,2-Dimethylcyclohexyl)ethanone # $$

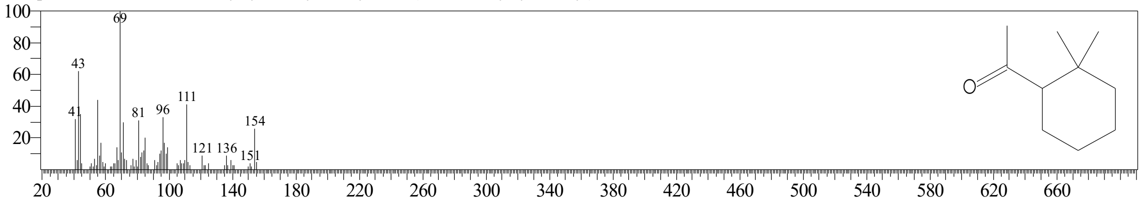

Hit#:2 Entry:189680 Library:NIST11.lib
SI:49 Formula:C24H35ClO4 CAS:0-00-0 MolWeight:422 RetIndex:3020
CompName:Fumaric acid, 2-chloro-5-methylphenyl tridecyl ester

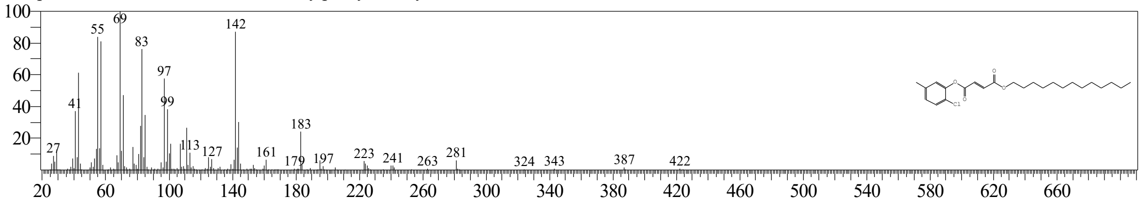

Hit#:3 Entry:185193 Library:NIST11.lib
SI:49 Formula:C23H33ClO4 CAS:0-00-0 MolWeight:408 RetIndex:2920
CompName:Fumaric acid, 2-chloro-5-methylphenyl dodecyl ester

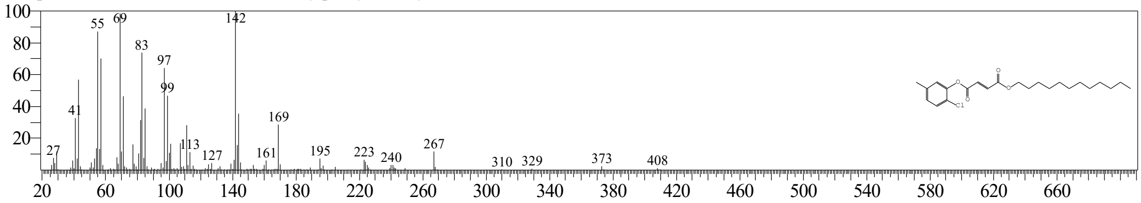

Hit#:4 Entry:27072 Library:NIST11.lib
SI:48 Formula:C10H20O2 CAS:0-00-0 MolWeight:172 RetIndex:1220
CompName:Citronellol epoxide (R or S) $$ 5-(3,3-Dimethyl-2-oxiranyl)-3-methyl-1-pentanol # $$

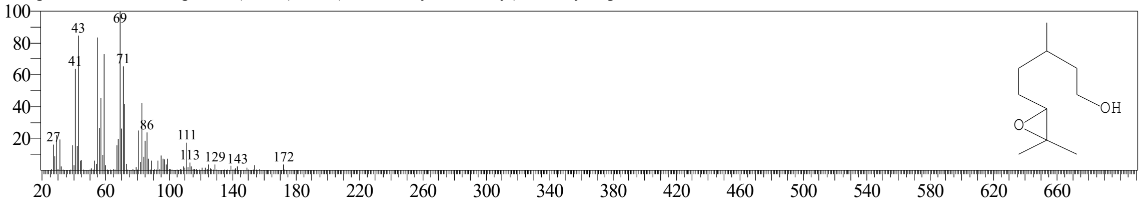

Hit#:5 Entry:59390 Library:NIST11.lib
SI:48 Formula:C15H26O CAS:0-00-0 MolWeight:222 RetIndex:1564
CompName:Nerolidol 2 $$ (6E)-3,7,11-Trimethyl-1,6,10-dodecatrien-3-ol # $$

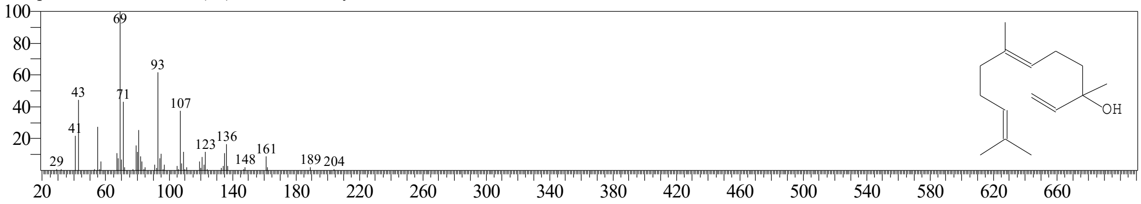

Qualitative Analysis Report

<< Target >>
Line#:187 R.Time:33.210(Scan#:5843) MassPeaks:434
RawMode:Averaged 33.205-33.215(5842-5844) BasePeak:43.10(61919)
BG Mode:Calc. from Peak Group 1 - Event 1 Scan

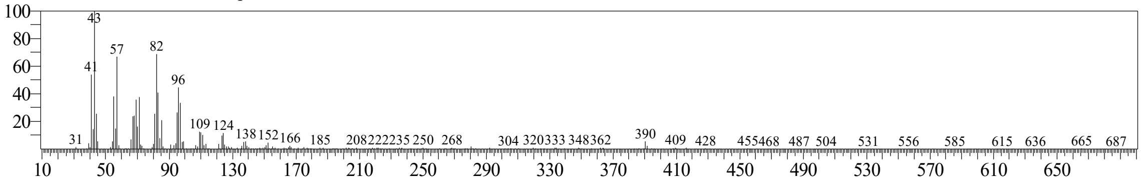

Hit#:1 Entry:93586 Library:NIST11.lib
SI:92 Formula:C18H36O CAS:638-66-4 MolWeight:268 RetIndex:1999
CompName:Octadecanal $$ Stearaldehyde $$ Octadecyl aldehyde $$ Stearyl aldehyde $$ n-Octadecanal $$

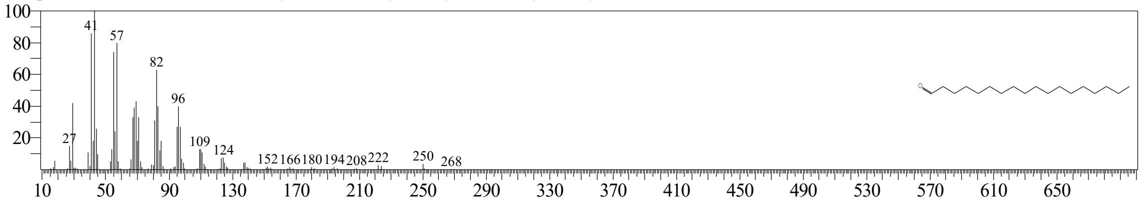

Hit#:2 Entry:82829 Library:NIST11.lib
SI:90 Formula:C17H34O CAS:0-00-0 MolWeight:254 RetIndex:1899
CompName:Heptadecanal

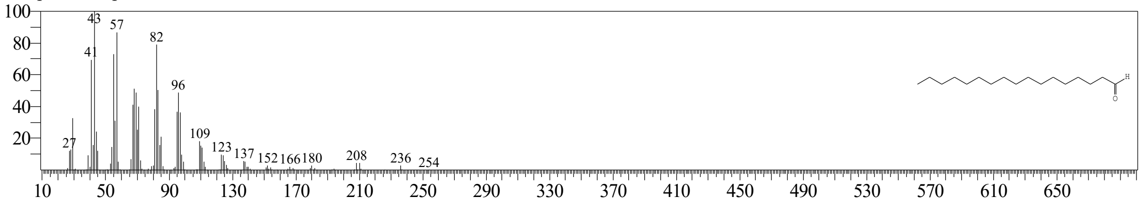

Hit#:3 Entry:42885 Library:NIST11.lib
SI:90 Formula:C13H26O CAS:10486-19-8 MolWeight:198 RetIndex:1502
CompName:Tridecanal $$ n-Tridecylaldehyde $$ Tridecanaldehyde $$ Tridecyl aldehyde $$ 1-Tridecanal $$ Tridecane aldehyde $$ n-Tridecanal $$

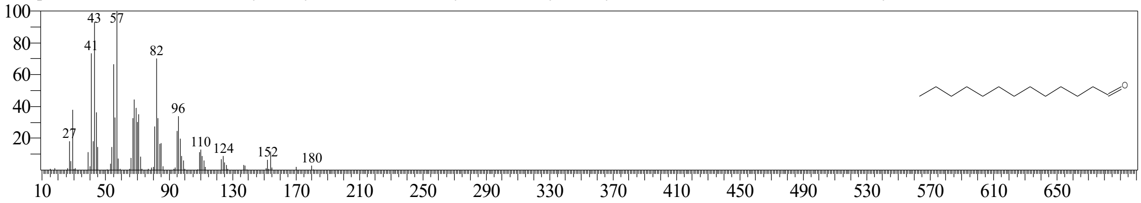

Hit#:4 Entry:93594 Library:NIST11.lib
SI:89 Formula:C18H36O CAS:0-00-0 MolWeight:268 RetIndex:2061
CompName:Z-2-Octadecen-1-ol $$ (2Z)-2-Octadecen-1-ol # $$

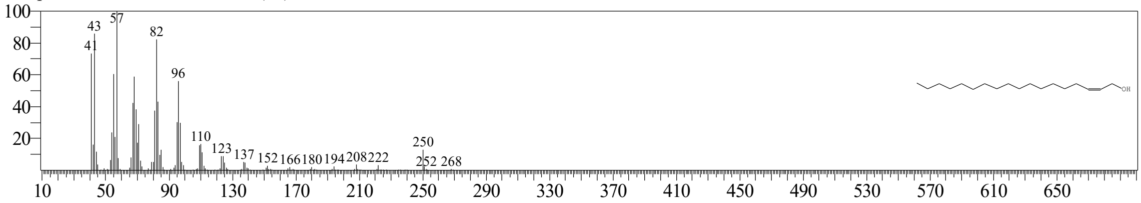

Hit#:5 Entry:52426 Library:NIST11.lib
SI:88 Formula:C14H28O CAS:0-00-0 MolWeight:212 RetIndex:1664
CompName:E-2-Tetradecen-1-ol $$ (2E)-2-Tetradecen-1-ol # $$

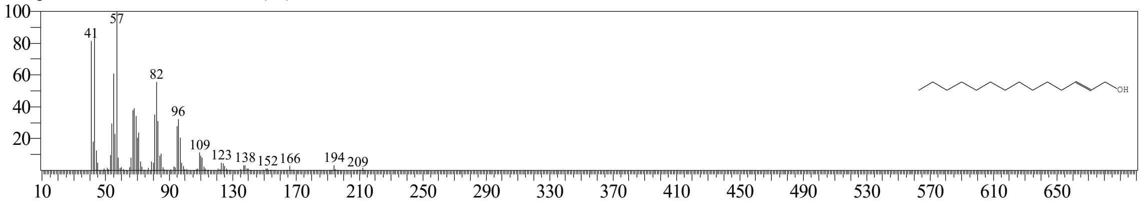

Supplement: Supplementary file 1 [file pharmaceuticals-19-01635-s001.zip › File S1—GC-MS analysis/Ozoroa paniculosa n-Hexane (Ramile).pdf]
